# Supplementary material for: Comparative genomics identifies thousands of candidate structured RNAs in human microbiomes
Source: Genome Biol. 2021 Apr 12;22:100. doi: 10.1186/s13059-021-02319-w (PMC8040213; doi:10.1186/s13059-021-02319-w)

HMP2_1_1

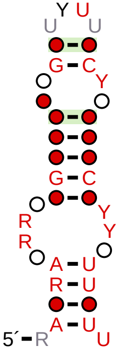

HMP2_10_1

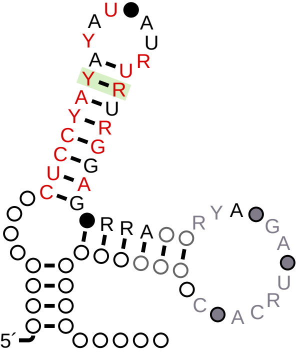

HMP2_1000_1

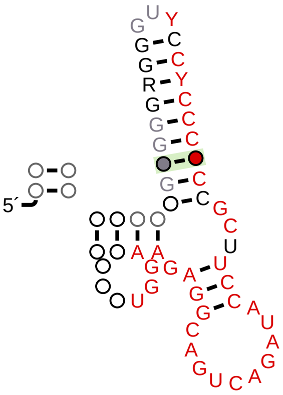

HMP2_10000_1

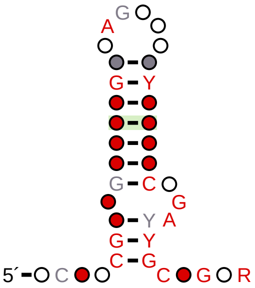

HMP2_10005_1

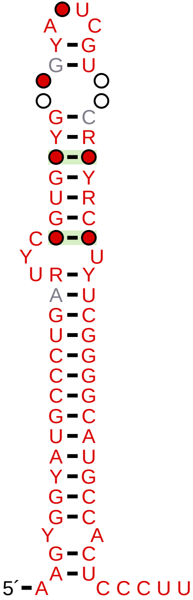

HMP2_10007_1

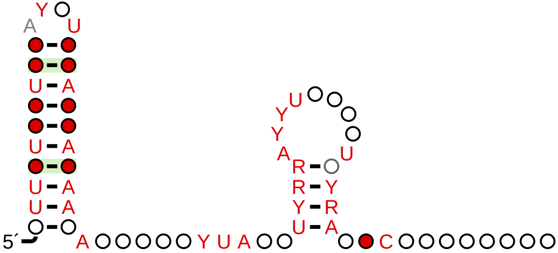

HMP2_10008_1

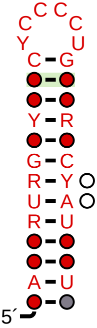

HMP2_1001_1

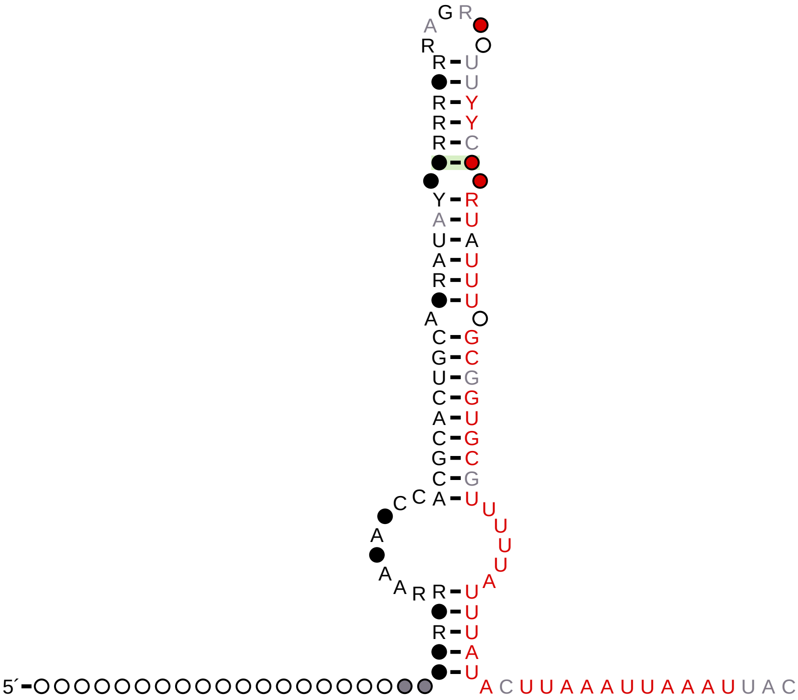

HMP2_10019_1

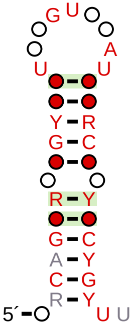

HMP2_10032_1

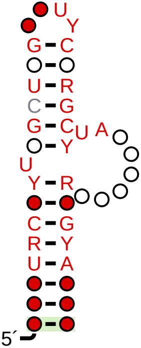

HMP2_10037_1

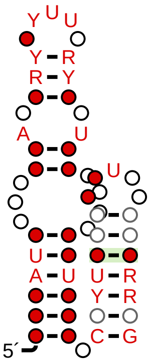

HMP2_1004_1

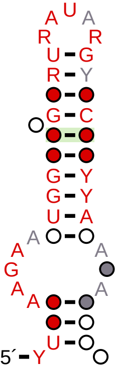

HMP2_10040_1

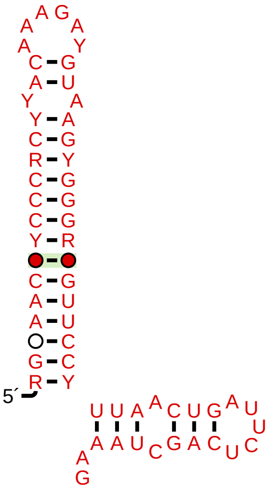

HMP2_10042_1

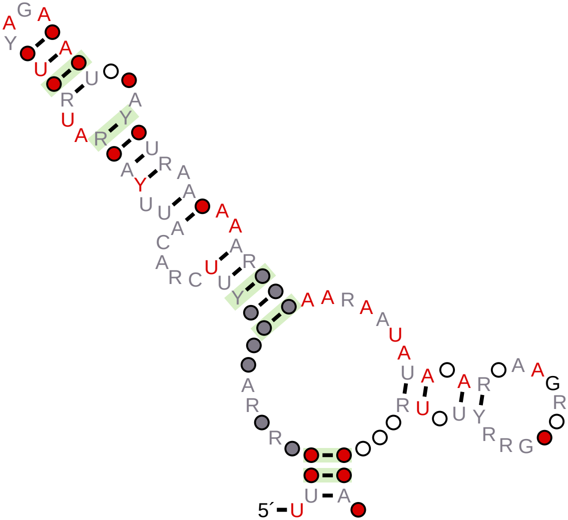

HMP2_10052_1

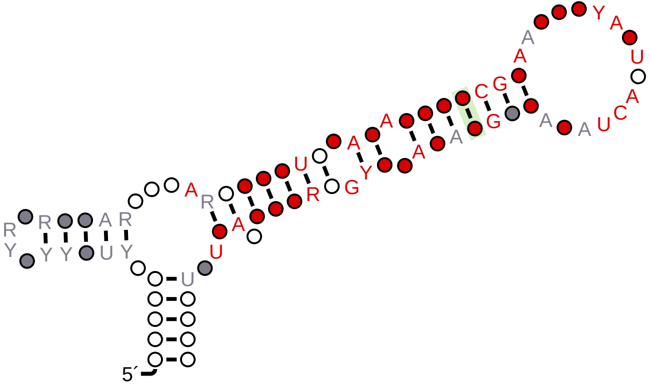

HMP2_10054_1

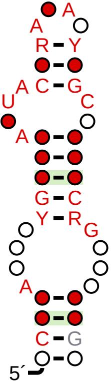

HMP2_10058_1

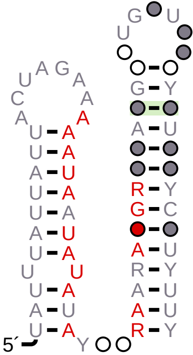

HMP2_10062_1

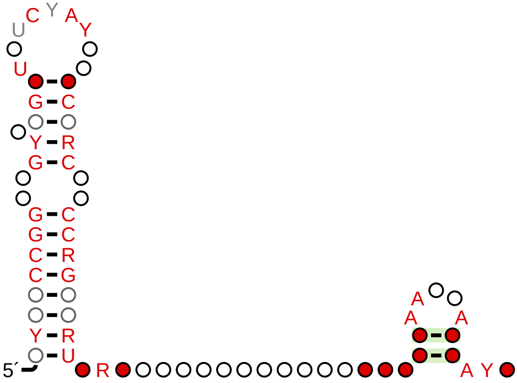

HMP2_10065_1

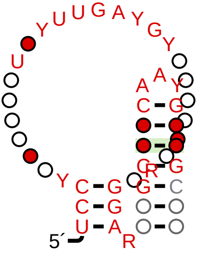

HMP2_1007_1

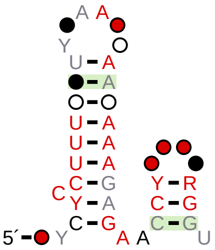

HMP2_10074_1

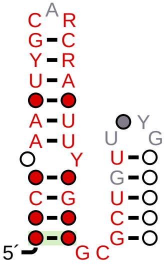

HMP2_10077_1

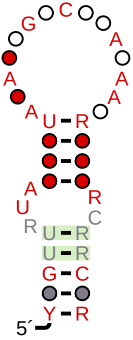

HMP2_10079_1

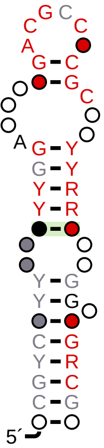

HMP2_10094_1

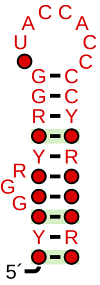

HMP2_10096_1

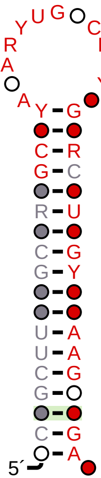

HMP2_101_1

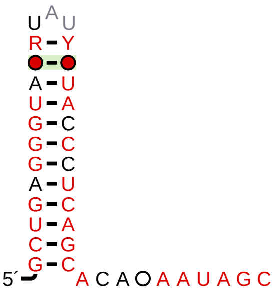

HMP2_10100_1

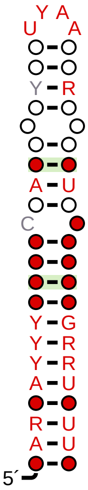

HMP2_10128_1

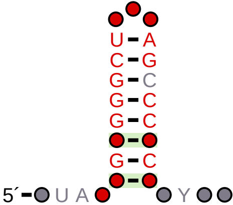

HMP2_10129_1

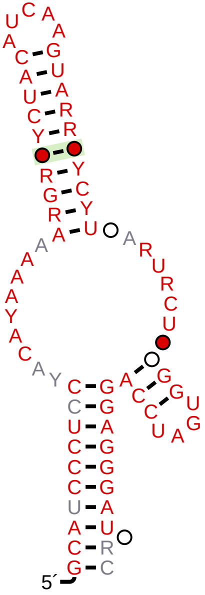

HMP2_10134_1

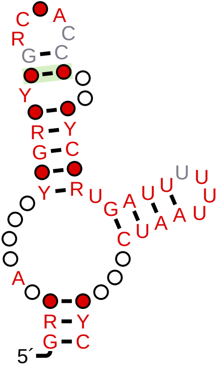

HMP2_10151_1

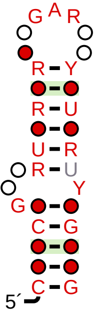

HMP2_10152_1

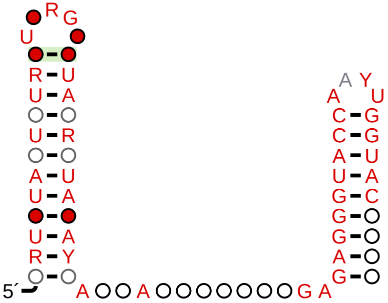

HMP2_10154_1

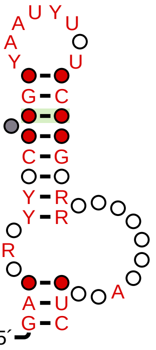

HMP2_1016_1

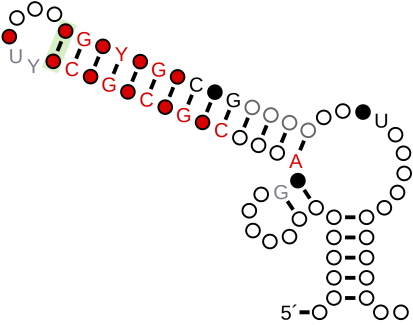

HMP2_10160_1

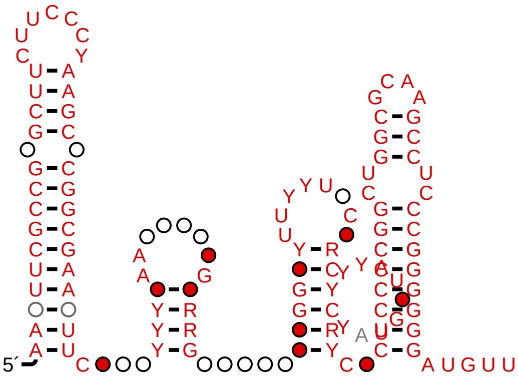

HMP2_10162_1

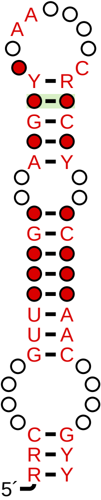

HMP2_10165_1

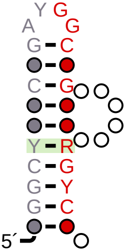

HMP2_1017_1

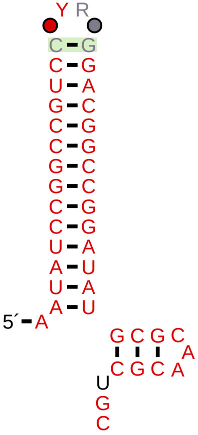

HMP2_10188_1

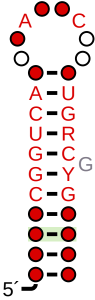

HMP2_10195_1

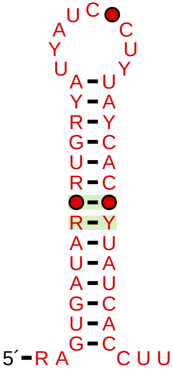

HMP2_10198_1

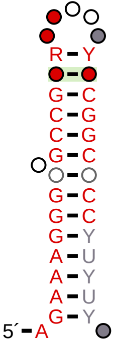

HMP2_1020_1

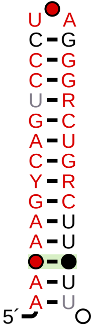

HMP2_10203_1

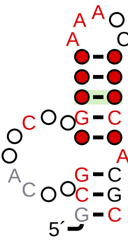

HMP2_10204_1

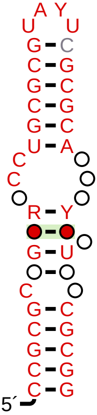

HMP2_10208_1

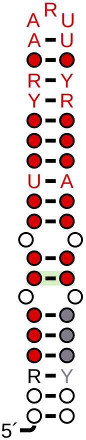

HMP2_10215_1

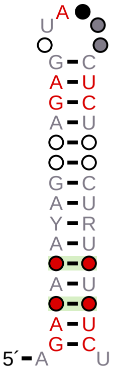

HMP2_10229_1

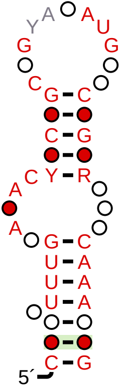

HMP2_10232_1

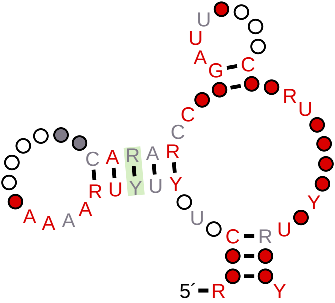

HMP2_10233_1

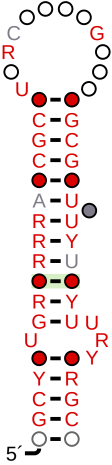

HMP2_10240_1

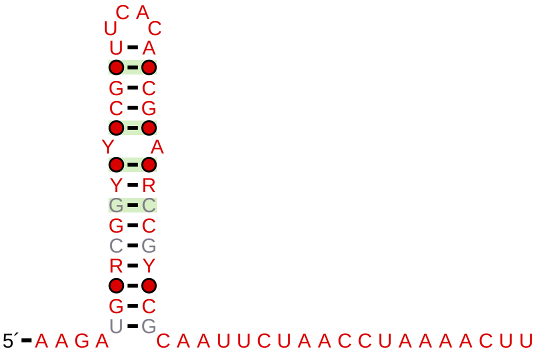

HMP2_10243_1

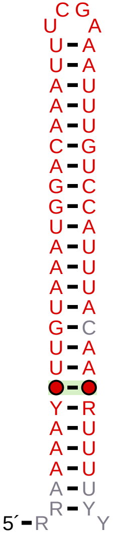

HMP2_10255_1

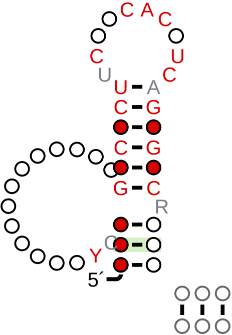

HMP2_10266_1

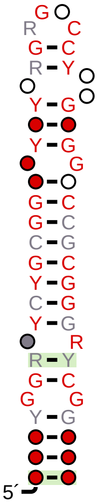

HMP2_10271_1

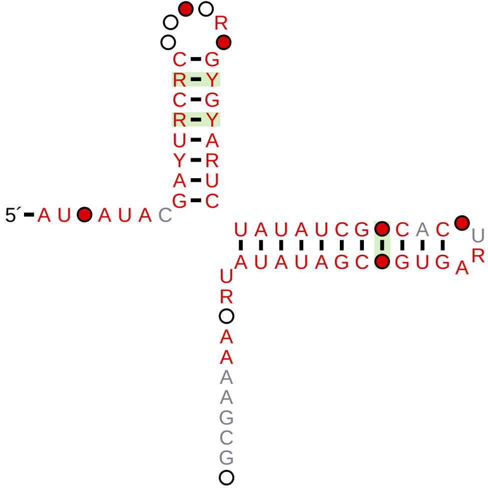

HMP2_10275_1

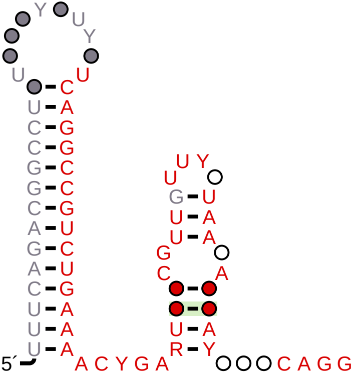

Y
U
Y
U
U
C
U
A
C
G
C
G
G
C
G
C
C
G
A
U
G
C
A
U
C
G
U
A
U
A
U
A
5´
A C Y G A
U Y
U
G
U
U
A
U
A
G
C
A
U
A
R
Y
C A G G

HMP2_10277_1

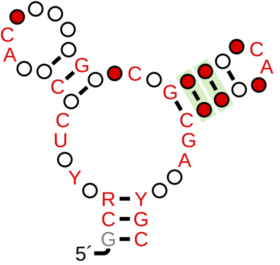

HMP2_10280_1

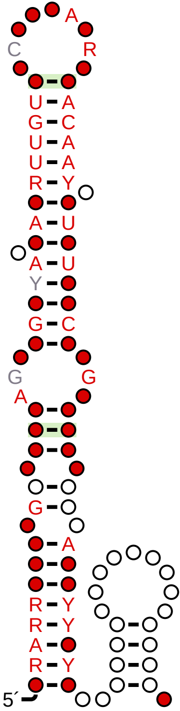

HMP2_10284_1

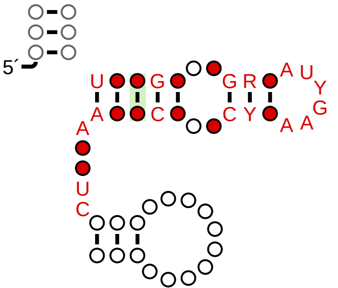

HMP2_10289_1

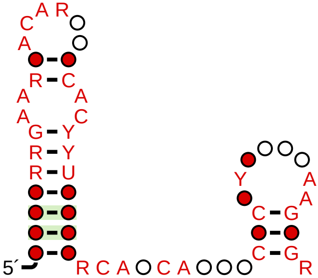

HMP2_10292_1

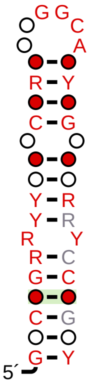

HMP2_10293_1

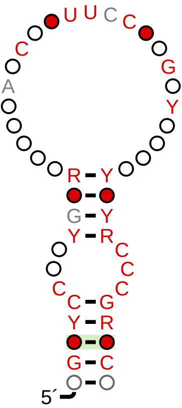

HMP2_10296_1

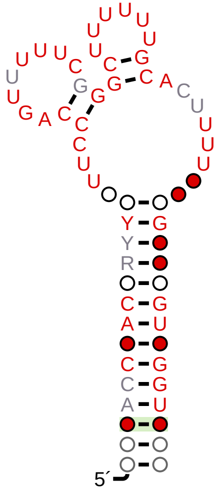

HMP2_10302_1

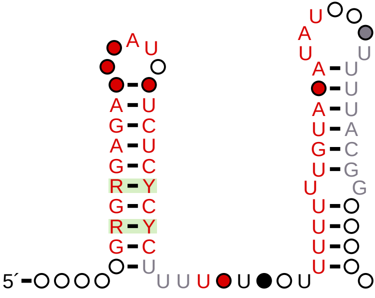

HMP2_10305_1

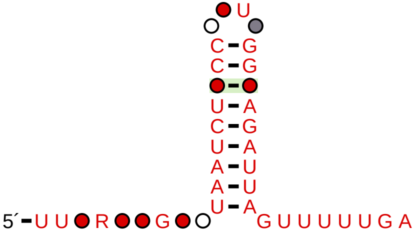

HMP2_10311_1

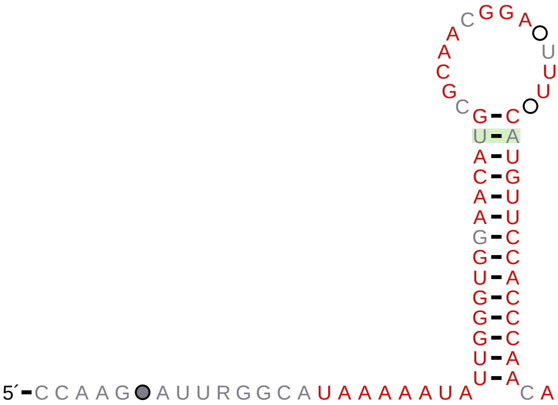
5´ C C A A G A U U R G G C A U A A A A A U A
U U G G G U G G A A C A U G C G C A A C G G A U U U C A U G U U C C A C C C A A C A

HMP2_10315_1

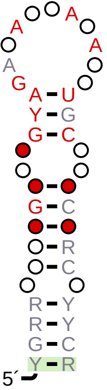

HMP2_10316_1

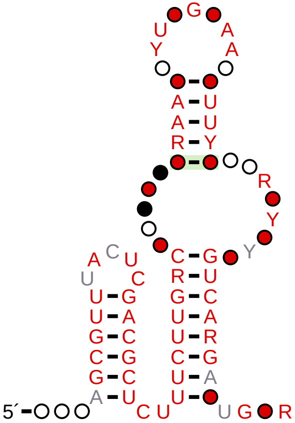

HMP2_10318_1

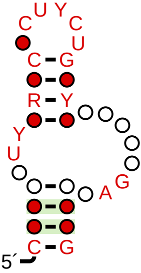

HMP2_1032_1

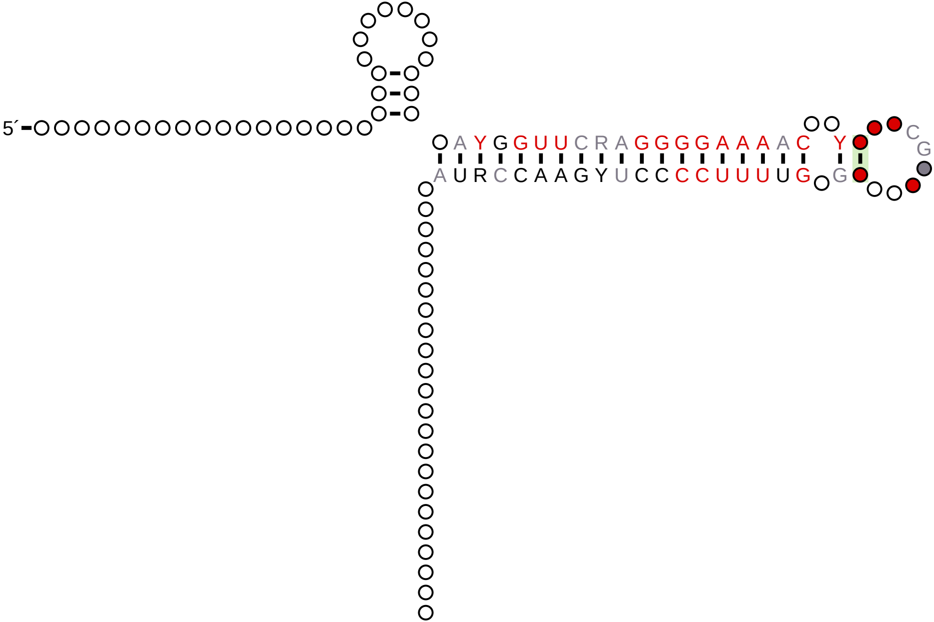

HMP2_10321_1

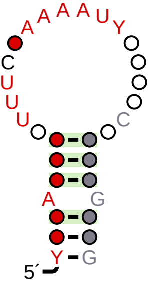

HMP2_10325_1

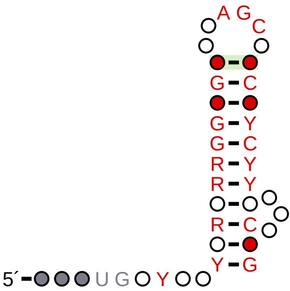

HMP2_10328_1

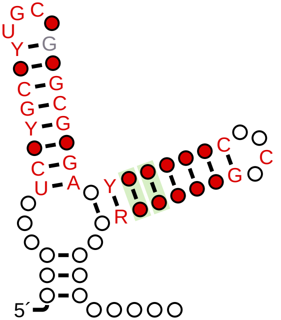

HMP2_10331_1

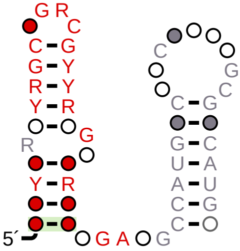

HMP2_10337_1

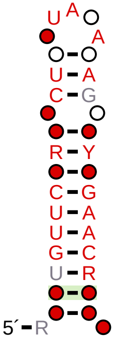

HMP2_1034_1

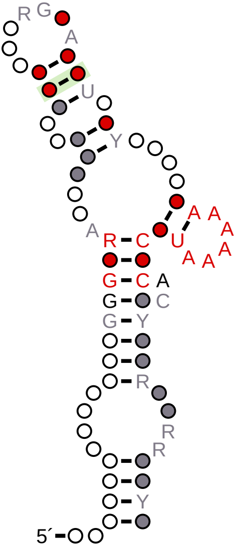

HMP2_10340_1

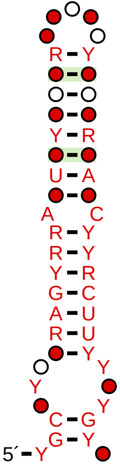

HMP2_10341_1

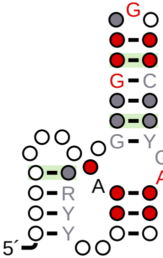

HMP2_1035_1

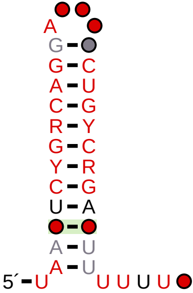

HMP2_10352_1

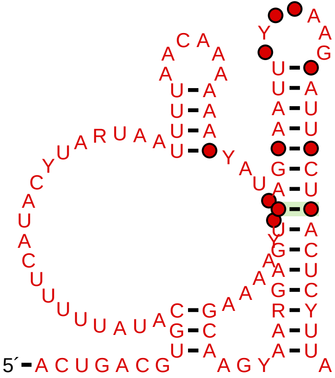

HMP2_10353_1

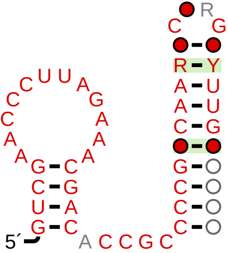

HMP2_10359_1

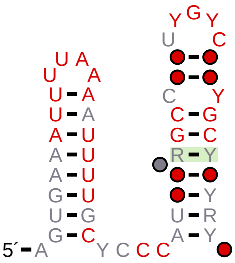

HMP2_10361_1

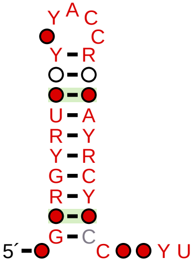

HMP2_10364_1

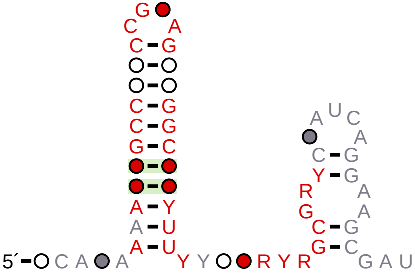

HMP2_10366_1

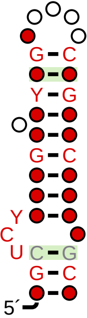

HMP2_10369_1

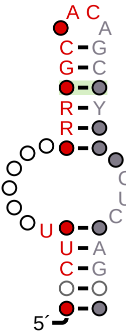

HMP2_1037_1

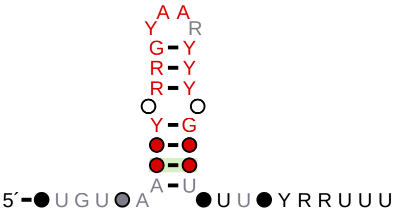

HMP2_10370_1

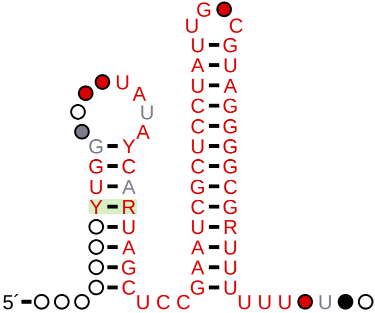

HMP2_10374_1

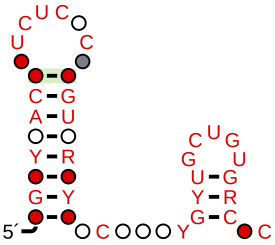

HMP2_10382_1

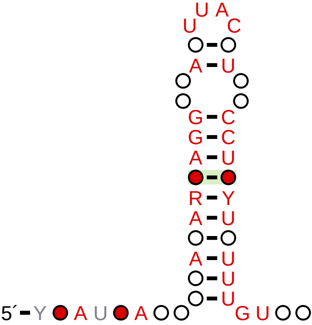

HMP2_10386_1

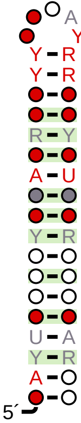

HMP2_10388_1

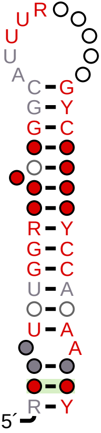

HMP2_10393_1

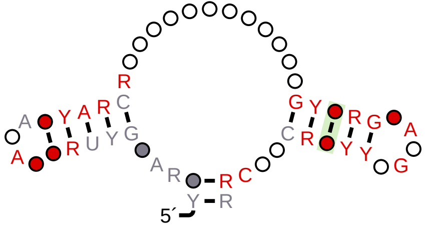

HMP2_10398_1

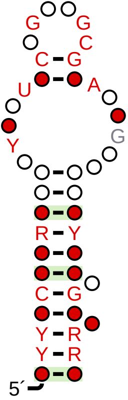

HMP2_104_1

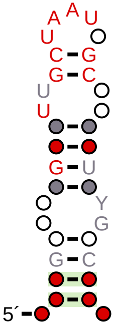

HMP2_1040_1

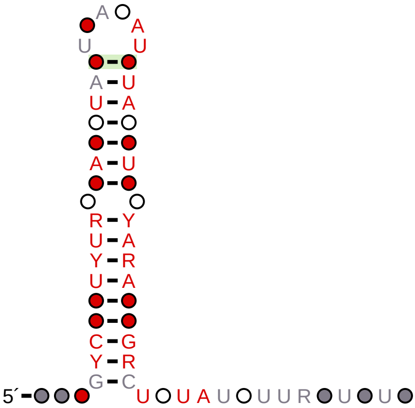

HMP2_10400_1

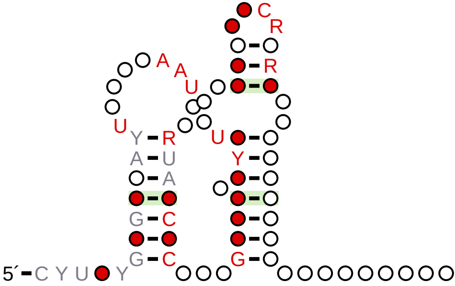

HMP2_10409_1

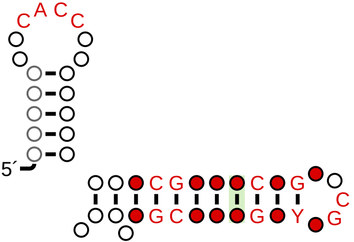

HMP2_1041_1

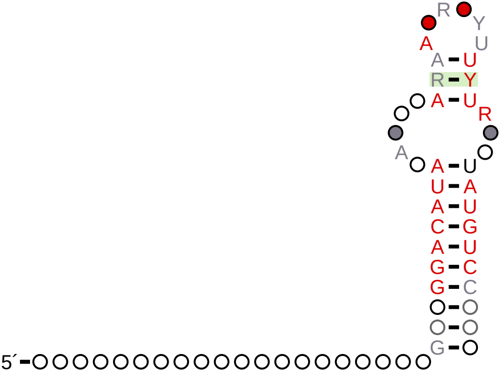

HMP2_10424_1

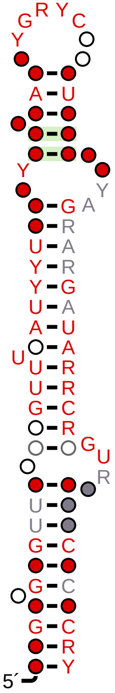

HMP2_10427_1

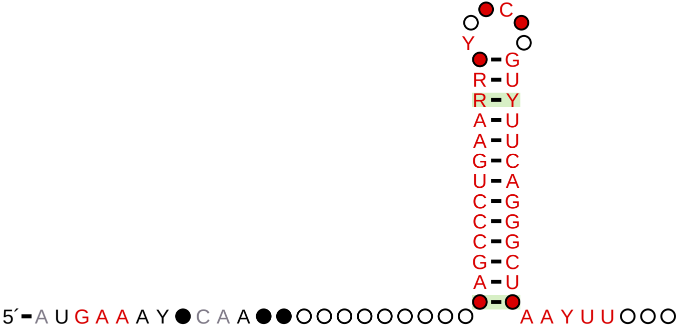

HMP2_10437_1

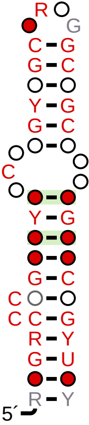

HMP2_10438_1

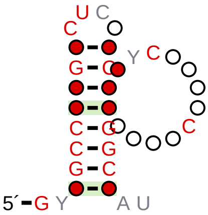

HMP2_10439_1

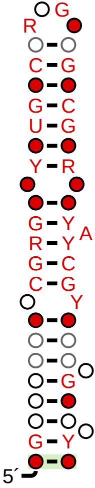

HMP2_10441_1

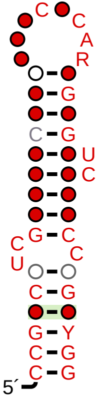

HMP2_10445_1

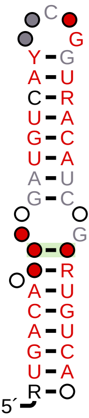

HMP2_10448_1

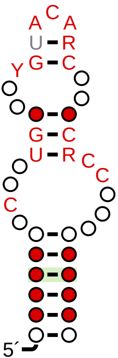

HMP2_10449_1

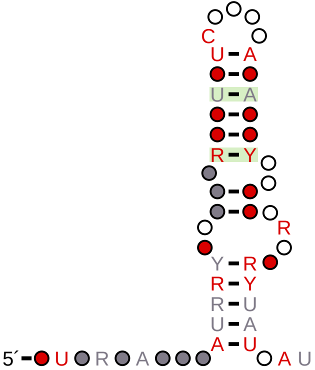

HMP2_10461_1

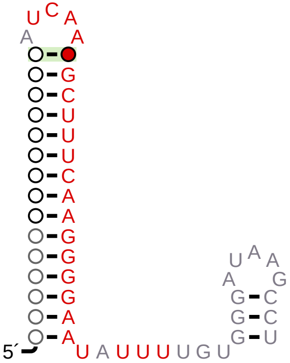

HMP2_1048_1

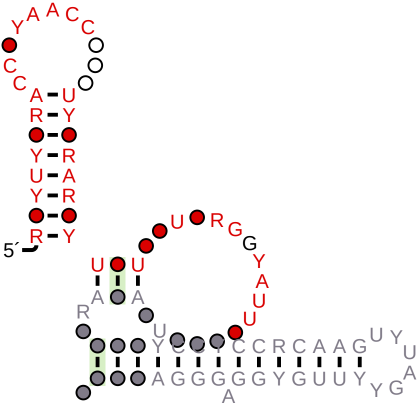

HMP2_10480_1

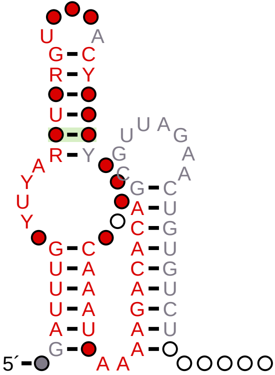

U A
G C
R Y
U
R Y
A Y U Y
U U A G
G A
C A
G C
A U
C G
A U
C G
A U
G C
A U
A
G C
U A
U A
U A
A U
G
5´
A A

HMP2_10481_1

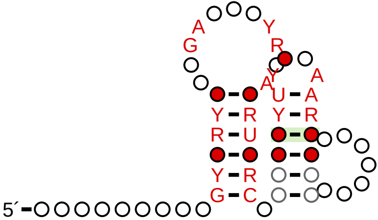

HMP2_10487_1

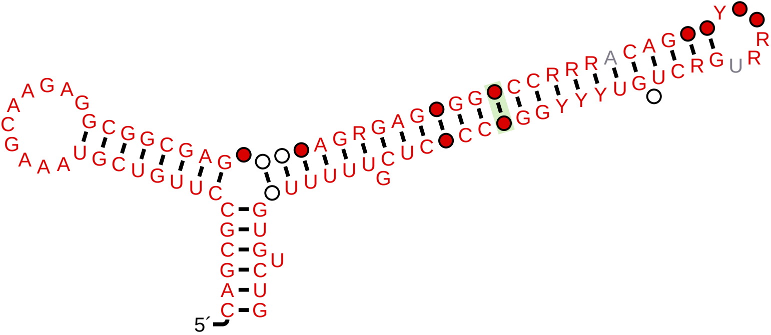

HMP2_1049_1

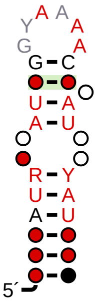

HMP2_10490_1

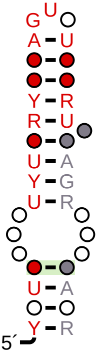

HMP2_105_1

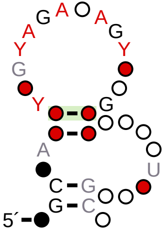

HMP2_10504_1

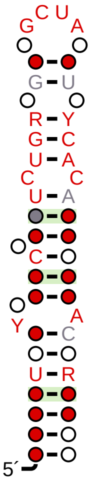

HMP2_10510_1

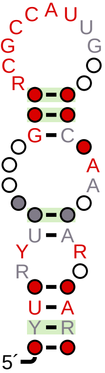

HMP2_10514_1

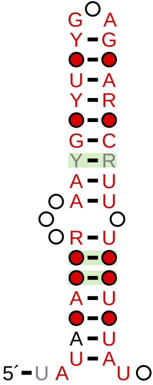

HMP2_10519_1

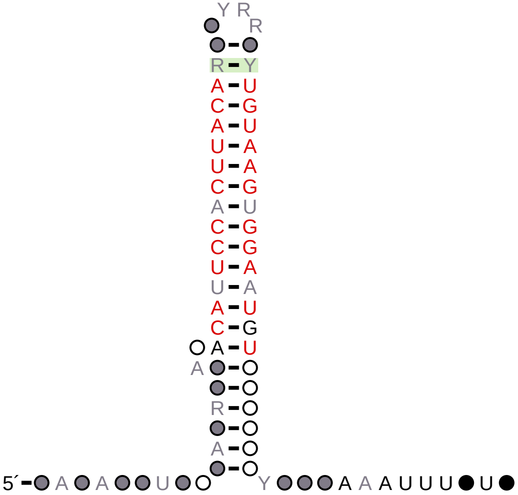

HMP2_10529_1

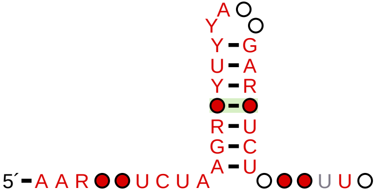

HMP2_1053_1

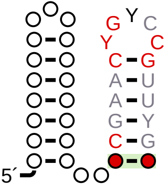

HMP2_10530_1

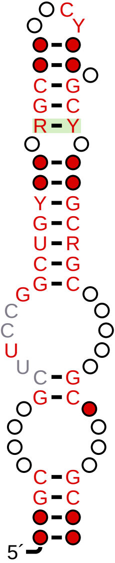

HMP2_10538_1

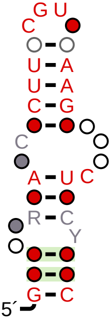

HMP2_10543_1

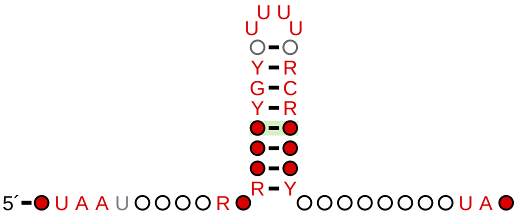

HMP2_1055_1

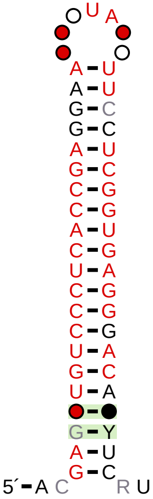

HMP2_10558_1

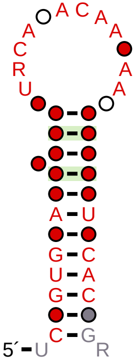

HMP2_10570_1

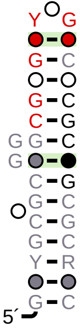

HMP2_10574_1

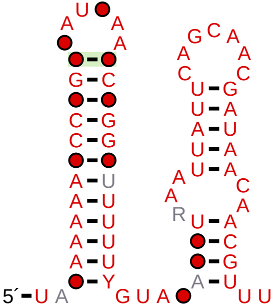

HMP2_10575_1

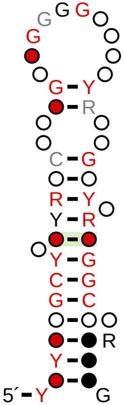

HMP2_1058_1

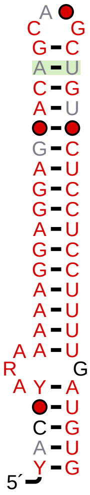

HMP2_10584_1

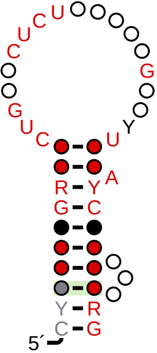

HMP2_10589_1

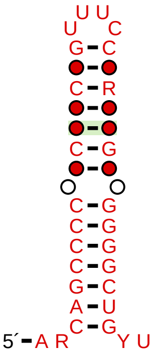

HMP2_10594_1

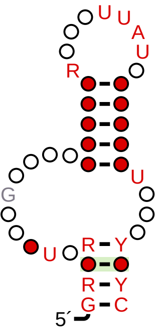

HMP2_10596_1

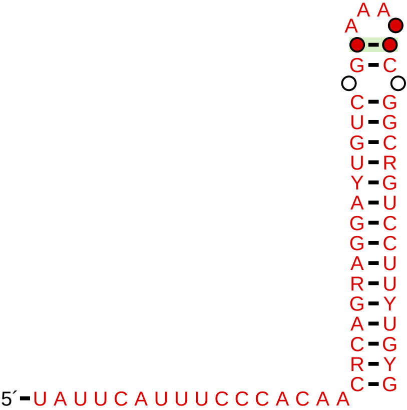

5´
U A U U C A U U U C C C A C A A
A A A
G C
C G
U G
G C
U R
Y G
A U
G C
G C
A U
R U
G Y
A U
C G
R Y
C G

HMP2_10600_1

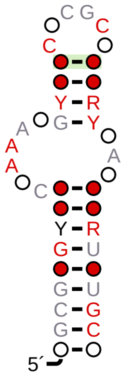

HMP2_10606_1

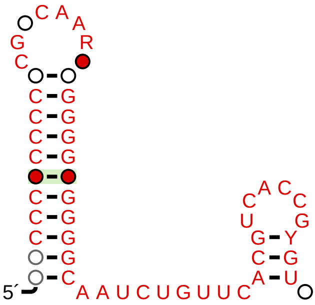

HMP2_10608_1

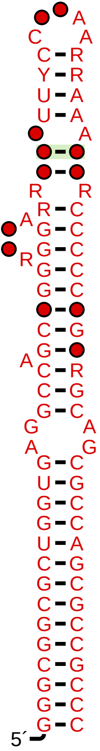

HMP2_1061_1

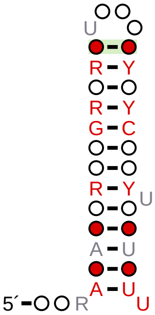

HMP2_10612_1

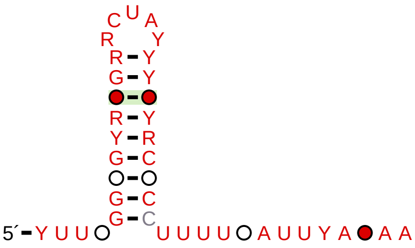

HMP2_10613_1

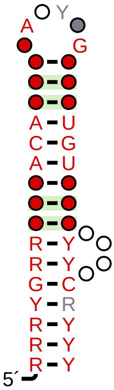

HMP2_10615_1

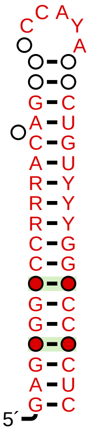

HMP2_10616_1

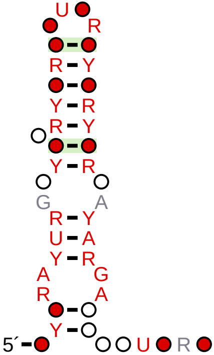

HMP2_1062_1

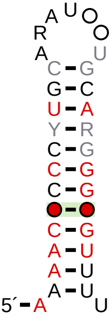

HMP2_10623_1

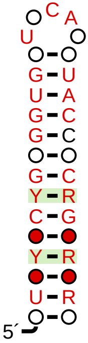

HMP2_10637_1

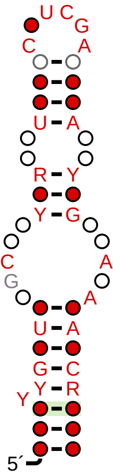

HMP2_10642_1

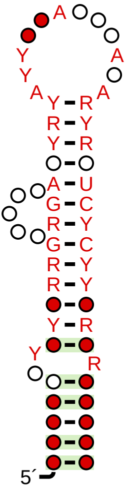

HMP2_1065_1

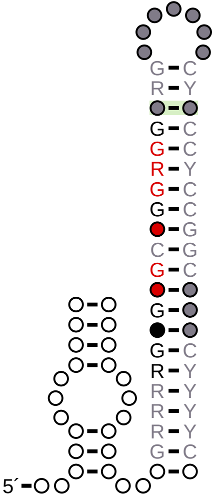

HMP2_10651_1

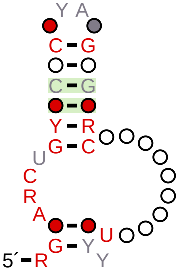

HMP2_10659_1

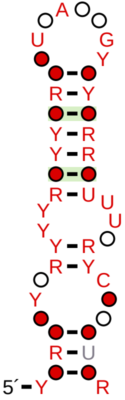

HMP2_10676_1

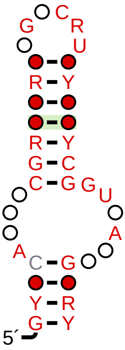

HMP2_10681_1

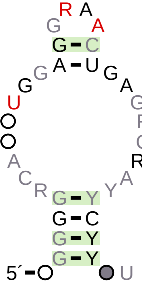

HMP2_10690_1

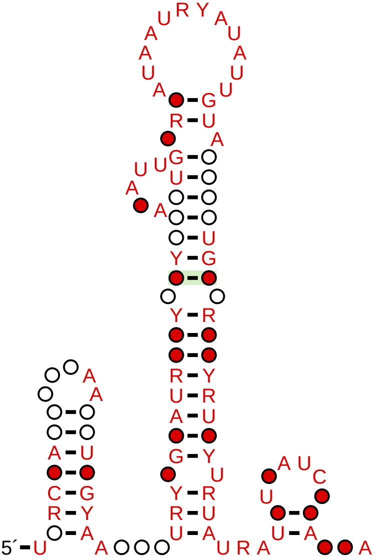

HMP2_10698_1

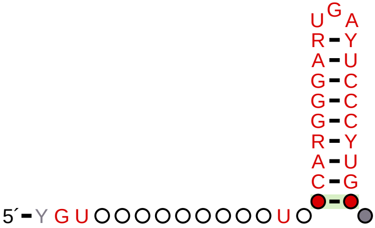

HMP2_1071_1

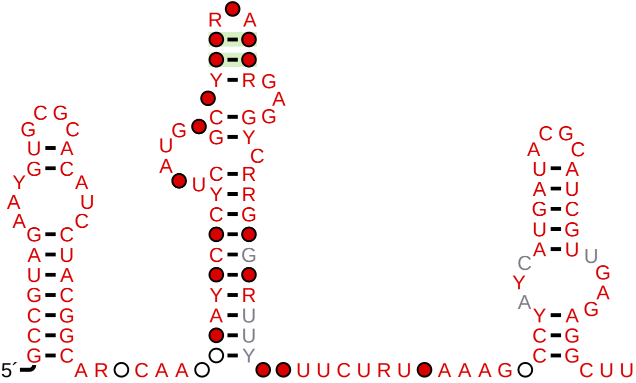

HMP2_10710_1

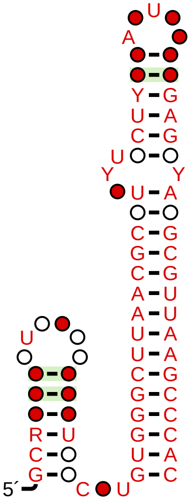

HMP2_10711_1

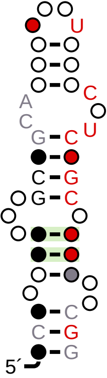

HMP2_10718_1

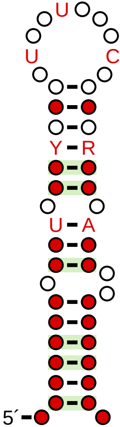

HMP2_10721_1

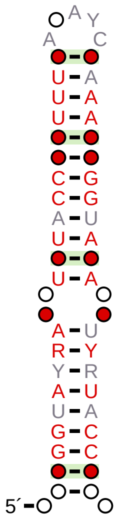

HMP2_10722_1

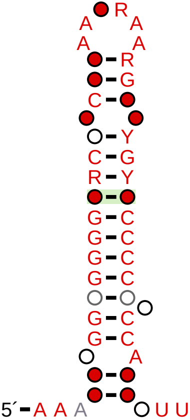

HMP2_10723_1

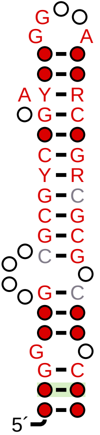

HMP2_10724_1

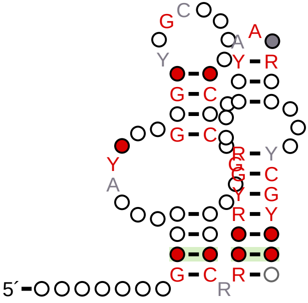

HMP2_1073_1

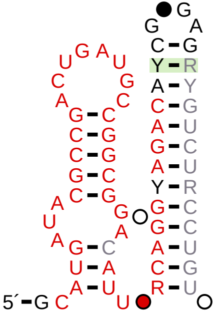

HMP2_10737_1

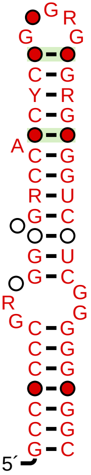

HMP2_10749_1

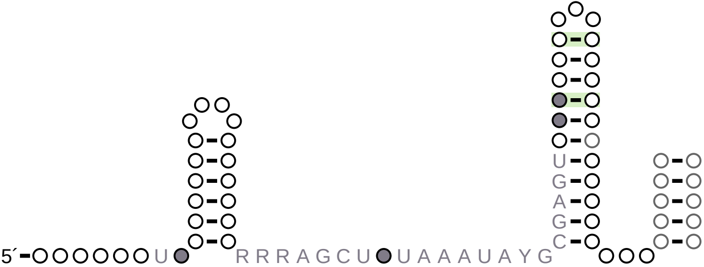

HMP2_10756_1

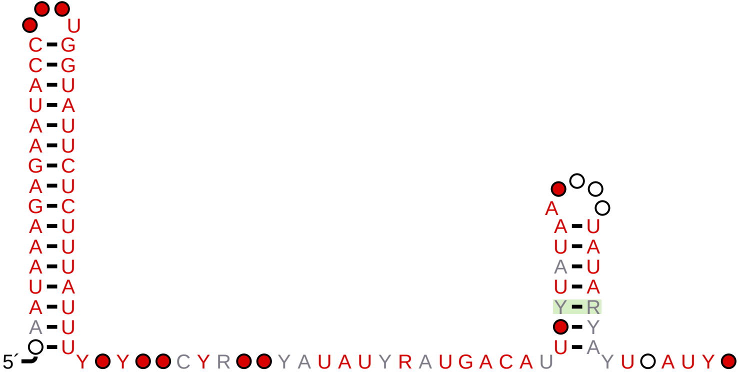

HMP2_10765_1

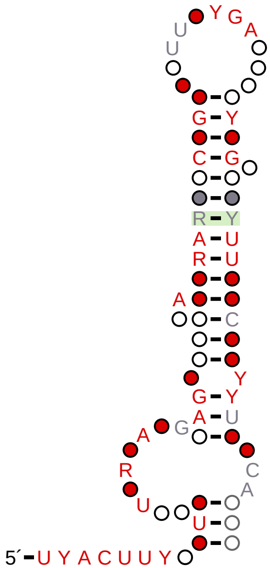

HMP2_10789_1

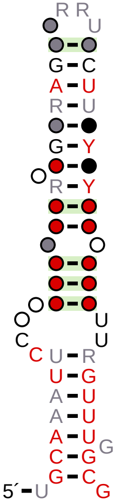

HMP2_1079_1

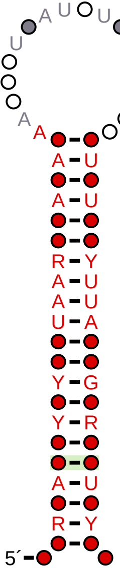

HMP2_1080_1

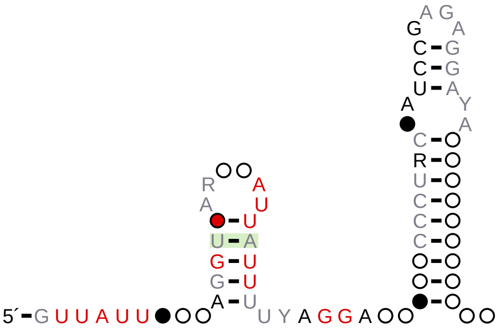

5´
G U U A U U
A G G A
G A A G A C C U A G G A Y A
C R U C C C
R A A U
U A G U G U G U A U
U Y A G G A

HMP2_10803_1

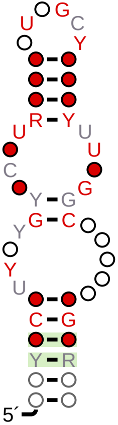

HMP2_10805_1

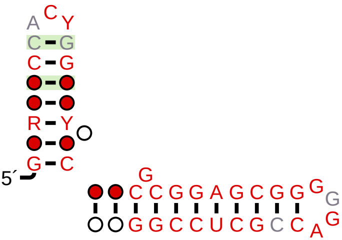

HMP2_10807_1

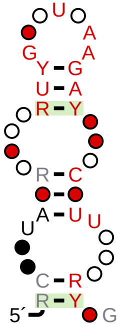

HMP2_10808_1

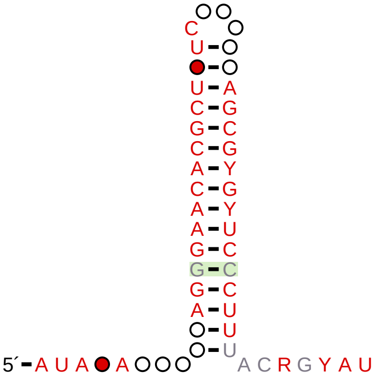

HMP2_10809_1

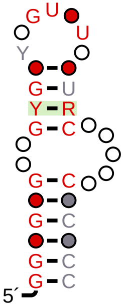

HMP2_10823_1

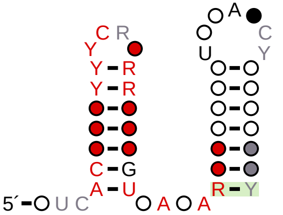

HMP2_10825_1

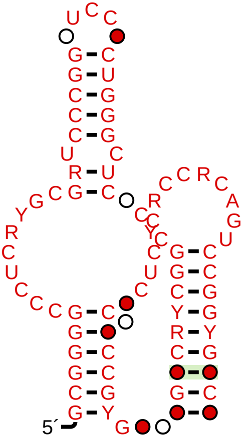

HMP2_10828_1

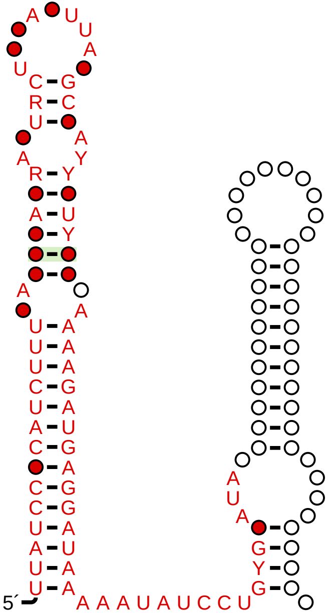

HMP2_1083_1

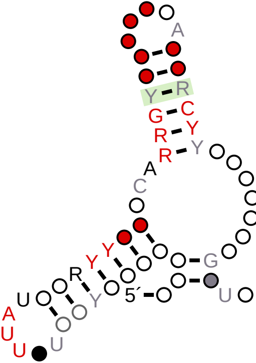

HMP2_10831_1

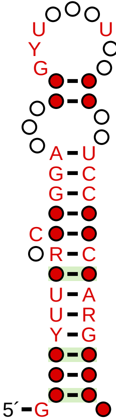

HMP2_10832_1

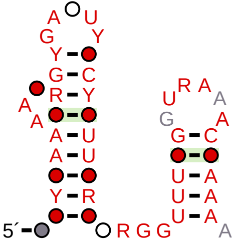

HMP2_1084_1

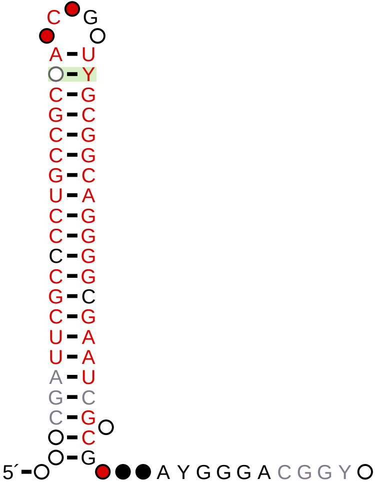

C G
A U
Y
C G
G C
C G
C G
G C
U A
C G
C G
C G
C G
G C
C G
U A
U A
A U
G C
C G
C
G
5´
A Y G G G A C G G Y

HMP2_10843_1

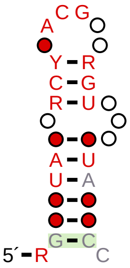

HMP2_10846_1

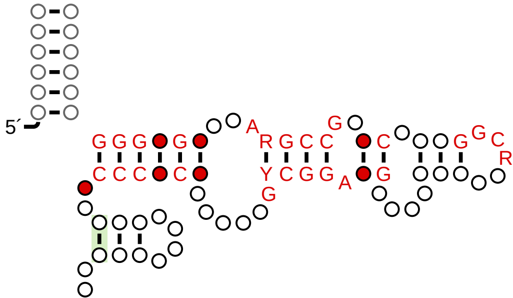

HMP2_10854_1

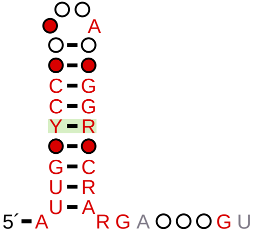

HMP2_10857_1

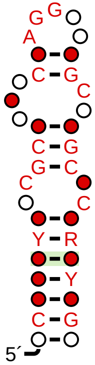

HMP2_10859_1

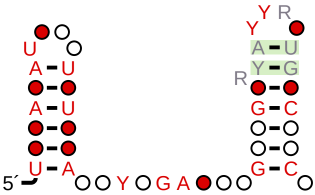

HMP2_1087_1

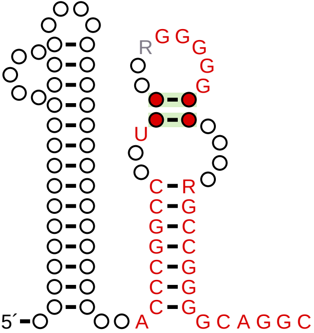

HMP2_10873_1

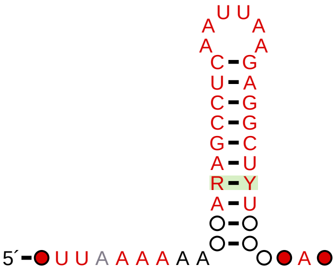

HMP2_10875_1

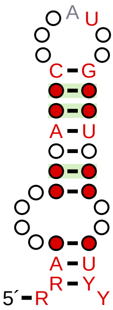

HMP2_10887_1

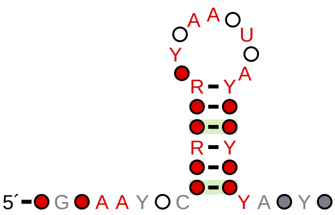

HMP2_1089_1

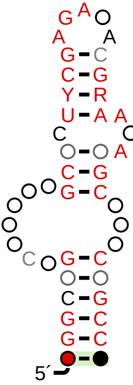

HMP2_10895_1

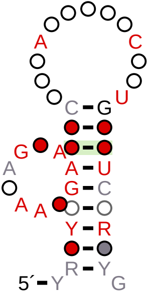

HMP2_10897_1

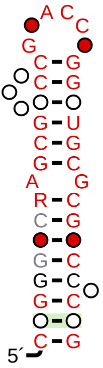

HMP2_10898_1

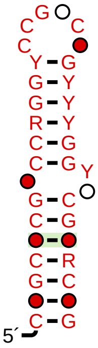

HMP2_10900_1

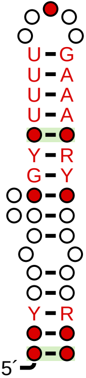

HMP2_10906_1

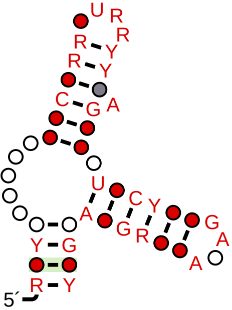

HMP2_1092_1

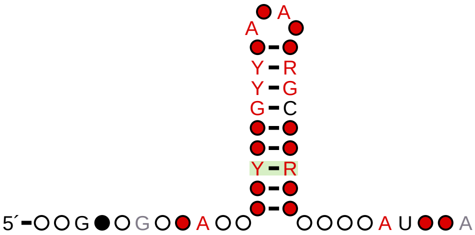

HMP2_1093_1

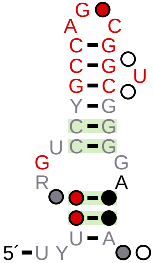

HMP2_10932_1

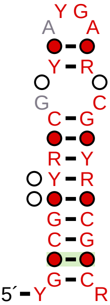

HMP2_10942_1

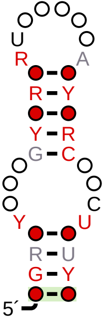

HMP2_10951_1

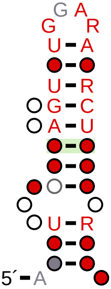

HMP2_10954_1

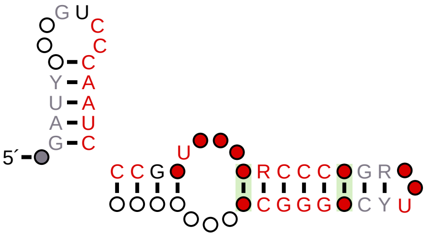

HMP2_10961_1

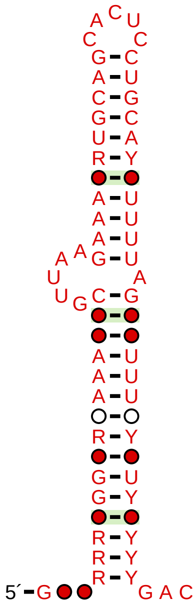

A C U
C C
G C
A U
C G
G C
U A
R Y
A U
A U
A U
A A G U
U A
U G C G
A U
A U
A U
R Y
G U
G Y
R Y
R Y
R Y
5´ G
G A C

HMP2_10964_1

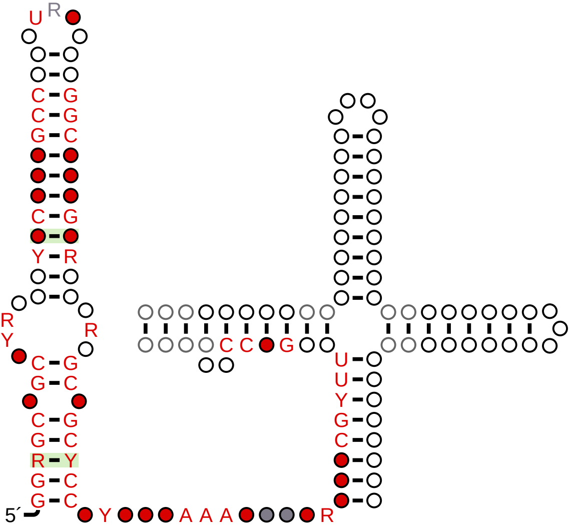

HMP2_10966_1

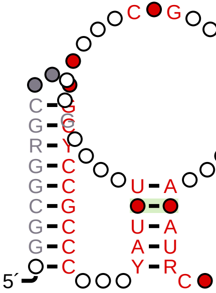

HMP2_10968_1

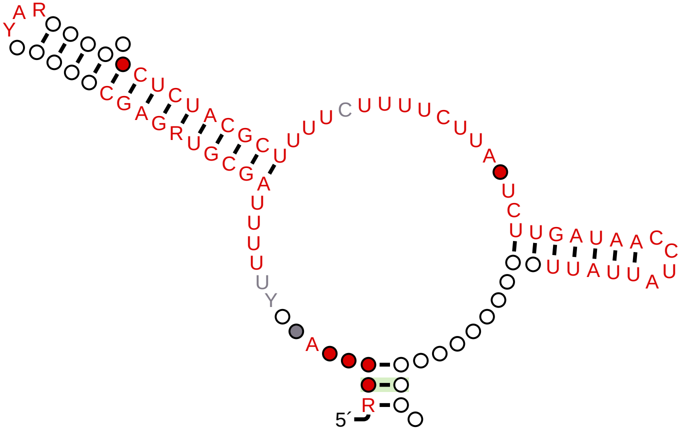

HMP2_10970_1

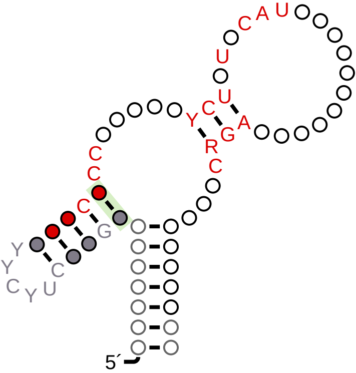

HMP2_10971_1

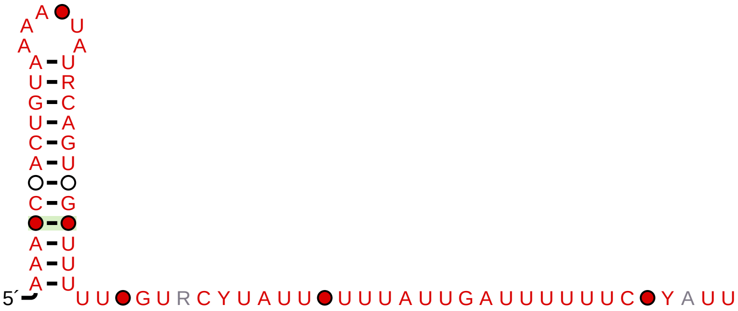

HMP2_10972_1

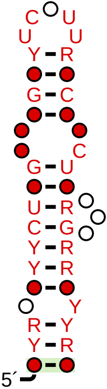

HMP2_10975_1

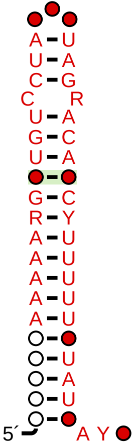

HMP2_10978_1

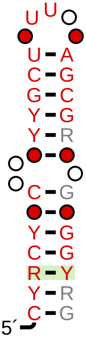

HMP2_10984_1

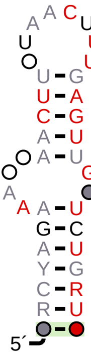

HMP2_1099_1

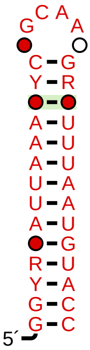

HMP2_10995_1

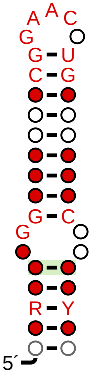

HMP2_10998_1

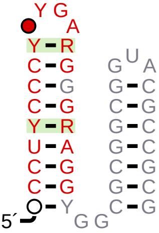

HMP2_11003_1

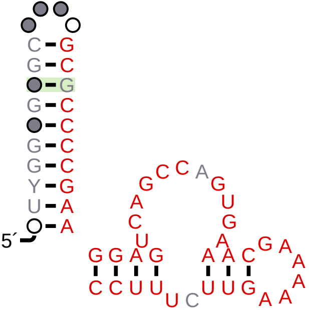

C G
G C
G
G C
C
G C
G C
Y G
U A
A
5´
G C C A G U G A A C G A A A A A G C U U U U C U U G C C G A U A

HMP2_1101_1

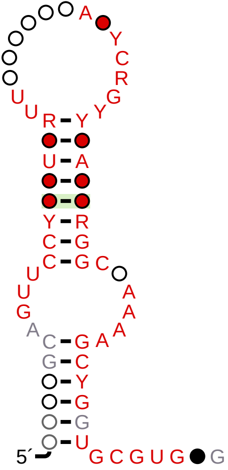

HMP2_11015_1

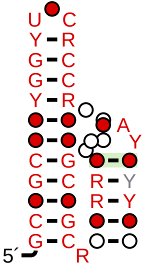

HMP2_11021_1

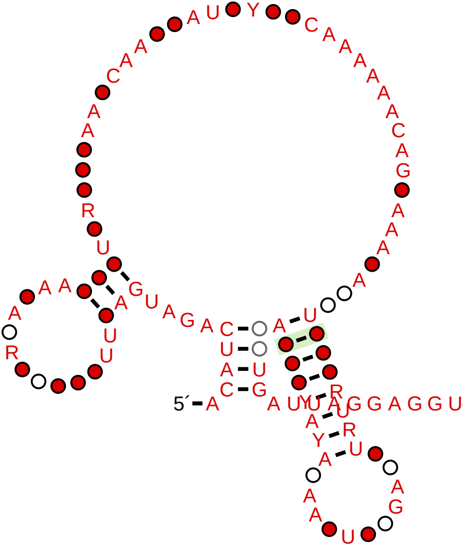

HMP2_11027_1

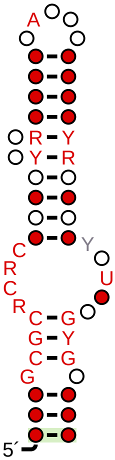

HMP2_11030_1

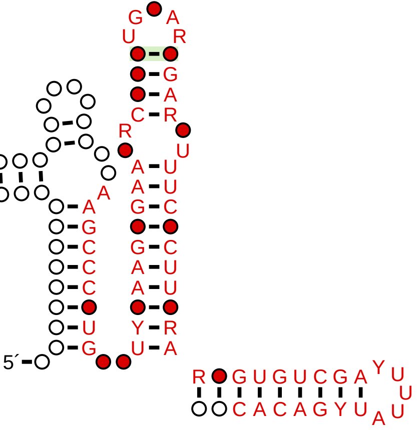

HMP2_11033_1

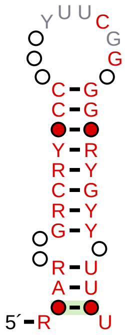

HMP2_11037_1

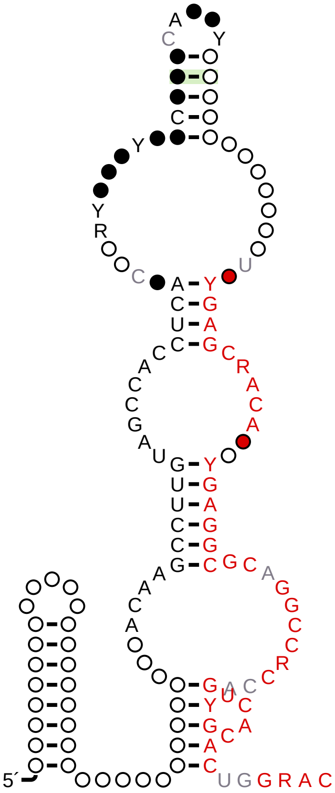

HMP2_11043_1

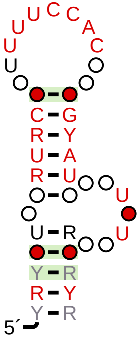

HMP2_11047_1

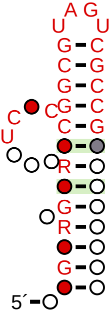

HMP2_11064_1

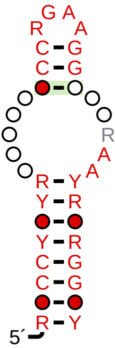

HMP2_11066_1

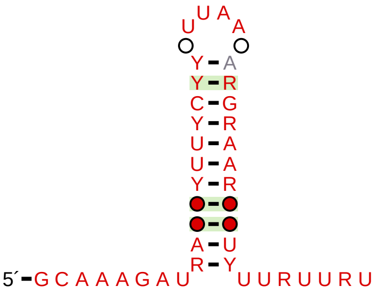

HMP2_11078_1

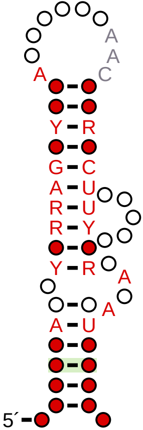

HMP2_11079_1

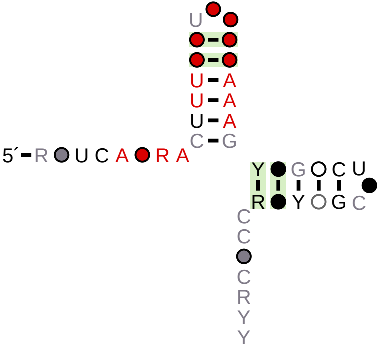

HMP2_11080_1

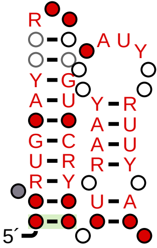

HMP2_11088_1

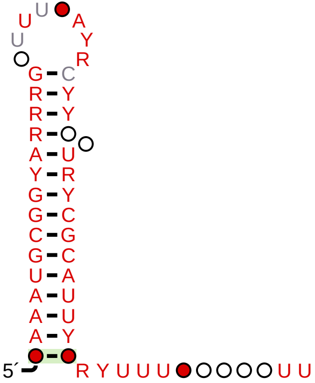

HMP2_11089_1

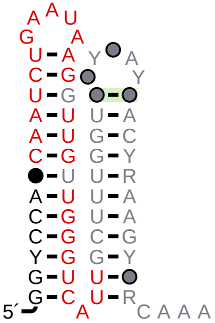

HMP2_11092_1

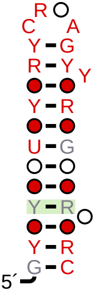

HMP2_11093_1

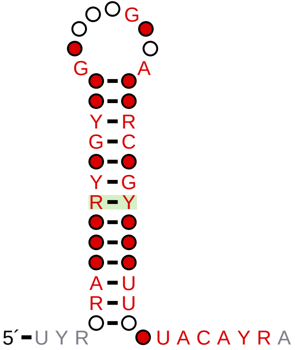

HMP2_111_1

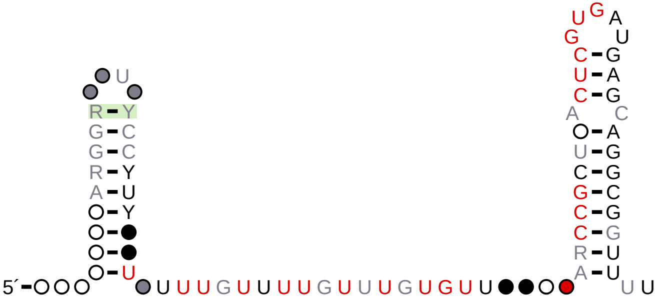

HMP2_1110_1

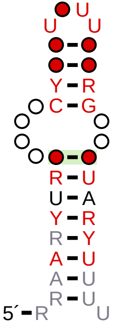

HMP2_11109_1

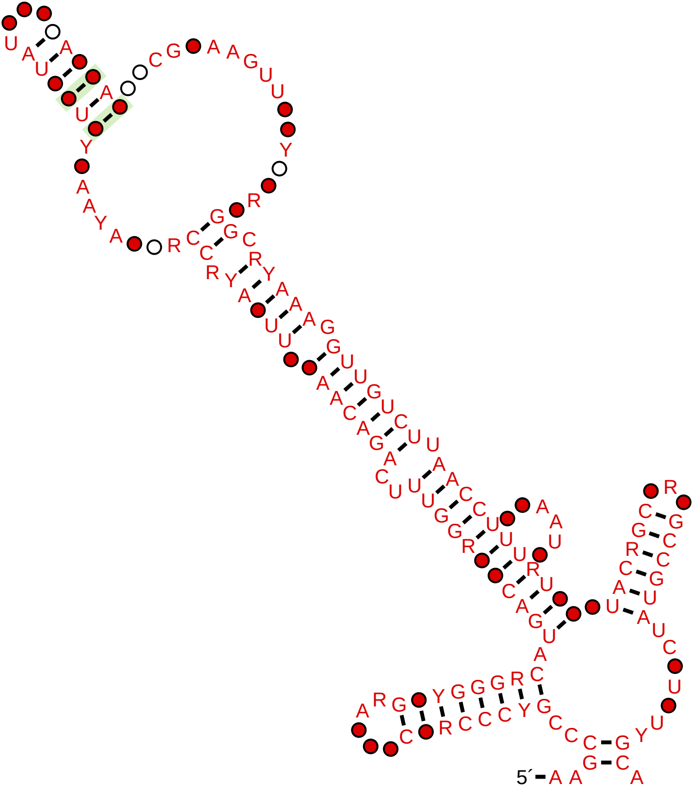

HMP2_11112_1

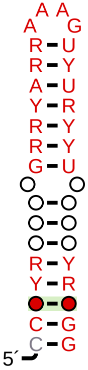

HMP2_11116_1

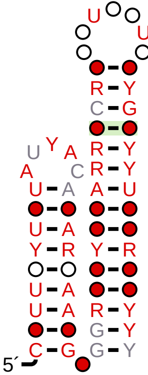

HMP2_11120_1

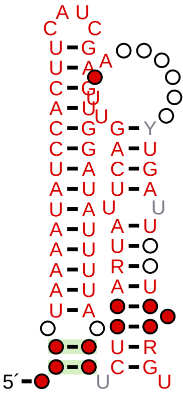

HMP2_11124_1

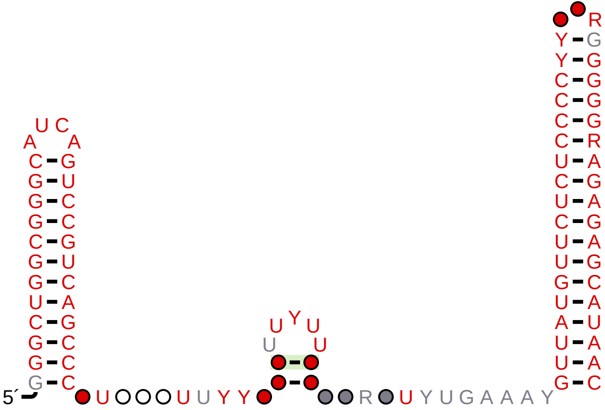

HMP2_11129_1

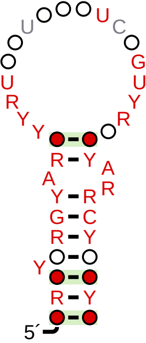

HMP2_11137_1

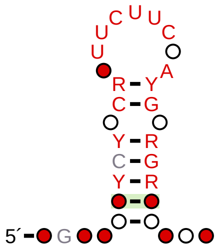

HMP2_11139_1

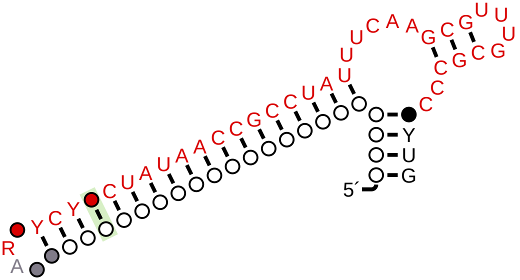

HMP2_11146_1

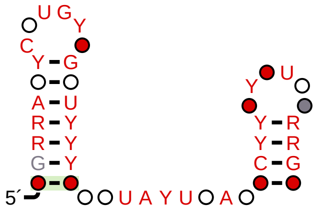

HMP2_11152_1

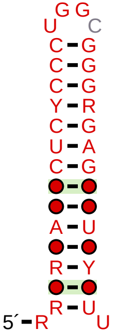

HMP2_11172_1

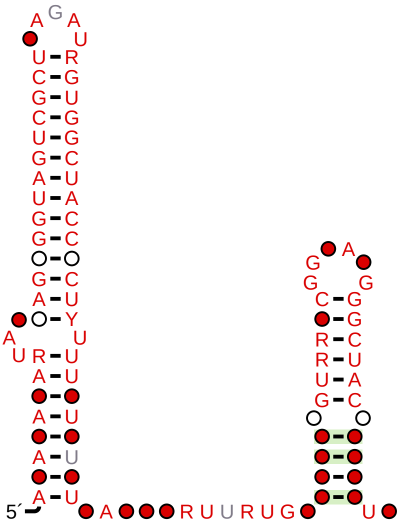

HMP2_11177_1

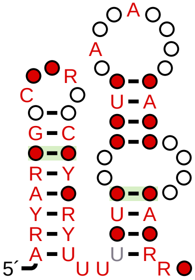

HMP2_11178_1

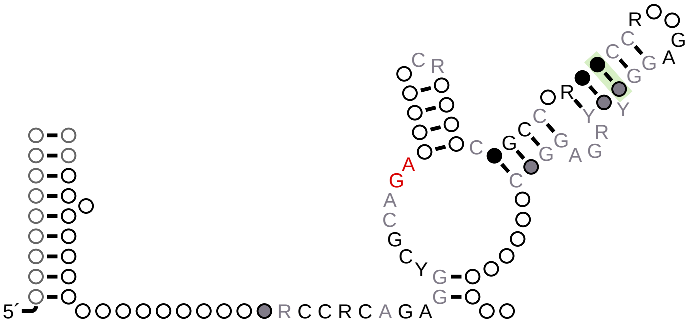

HMP2_11183_1

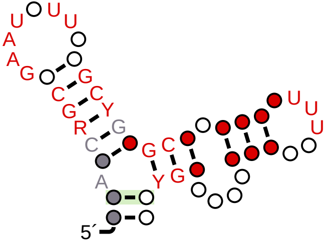

HMP2_11187_1

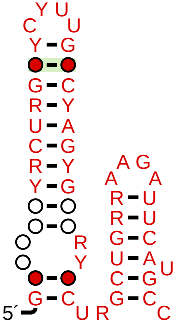

HMP2_11189_1

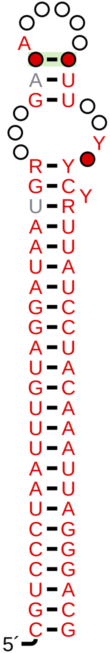
A
A-U
G-U
Y
R-Y
G-C
Y
U-R
A-U
A-U
U-A
A-U
G-C
G-C
A-U
U-A
G-C
U-A
U-A
U-A
A-U
A-U
U-A
C-G
C-G
C-G
U-A
G-C
C-G
5´

HMP2_11190_1

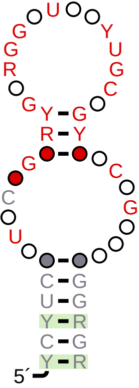

HMP2_11202_1

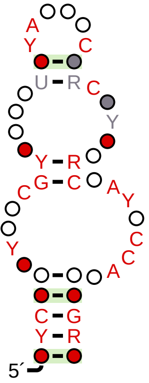

HMP2_11204_1

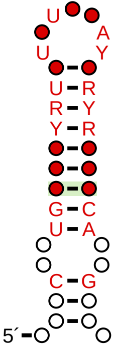

HMP2_11205_1

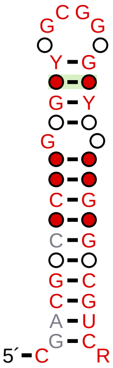

HMP2_11212_1

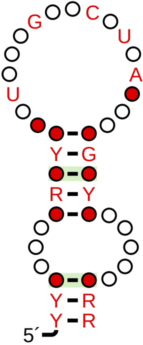

HMP2_11224_1

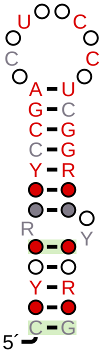

HMP2_1123_1

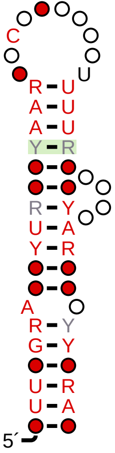

HMP2_11238_1

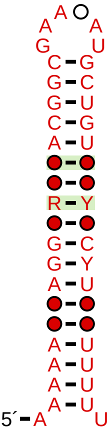

HMP2_11246_1

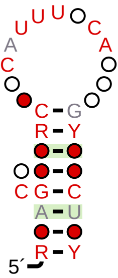

HMP2_11254_1

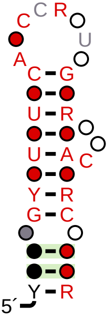

HMP2_11264_1

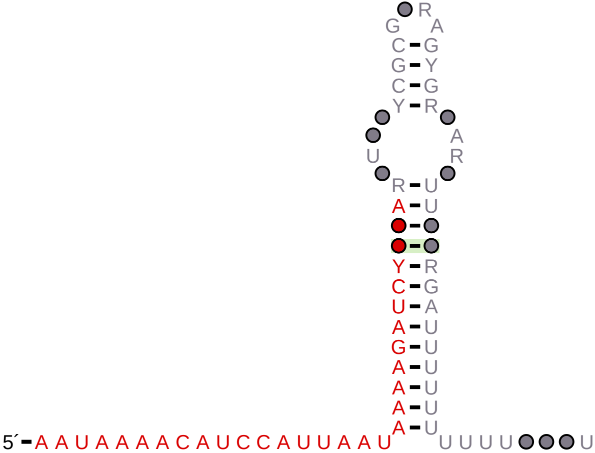
5´-A A U A A A A C A U C C A U U A A U
A A A A G A U C Y
U U U U U U U A G R
U U U U U
U
R A U
A R
R A G Y G C G C
G A

HMP2_11267_1

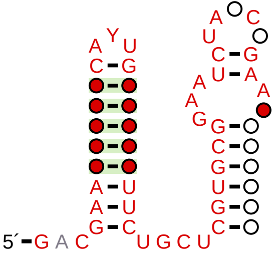

HMP2_1127_1

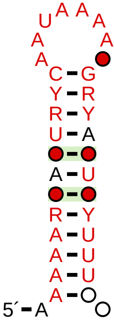

HMP2_11275_1

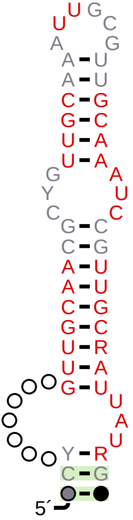

HMP2_11279_1

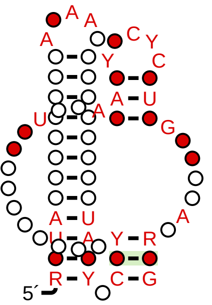

HMP2_11280_1

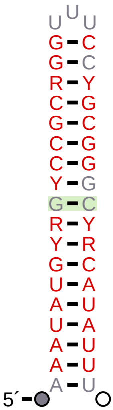

HMP2_11281_1

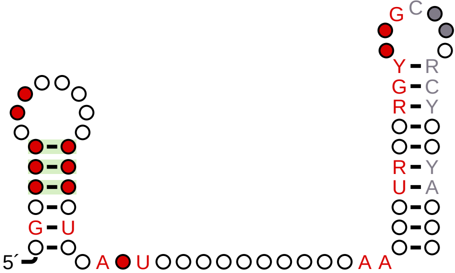

HMP2_11286_1

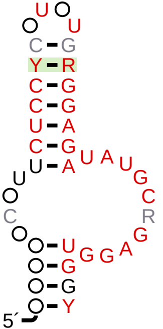

HMP2_11294_1

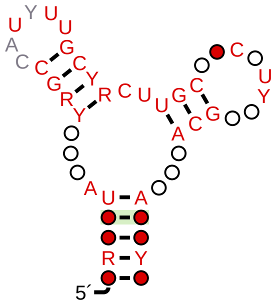

HMP2_1130_1

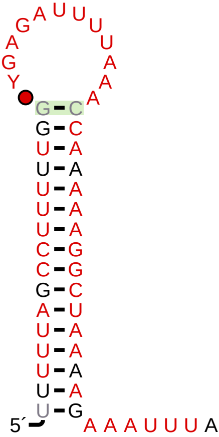

Y G A G A U U U U A A A
G-C
G-C
U-A
U-A
U-A
U-A
U-A
C-G
C-G
G-C
A-U
U-A
U-A
U-A
U-A
U-G
5´
A A A U U U A

HMP2_11306_1

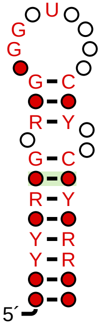

HMP2_11308_1

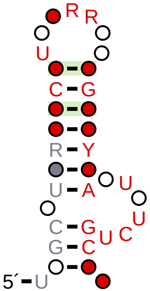

HMP2_11312_1

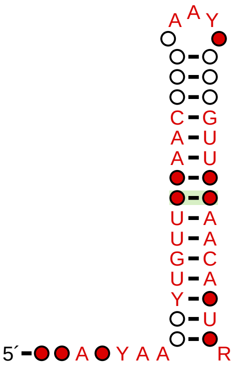

HMP2_11313_1

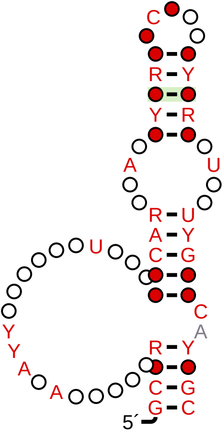

HMP2_11314_1

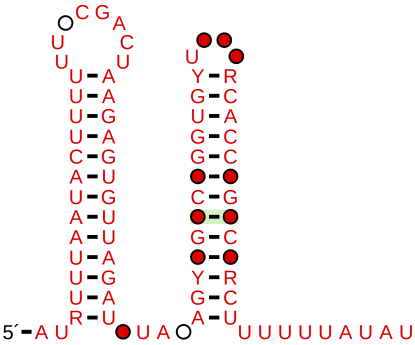

HMP2_11315_1

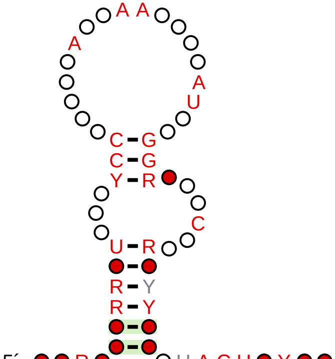

HMP2_11323_1

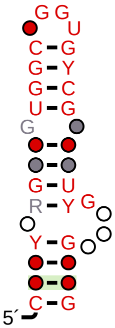

HMP2_11327_1

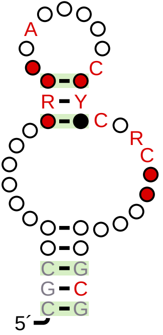

HMP2_11341_1

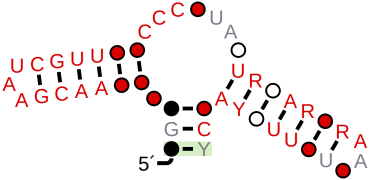

HMP2_11345_1

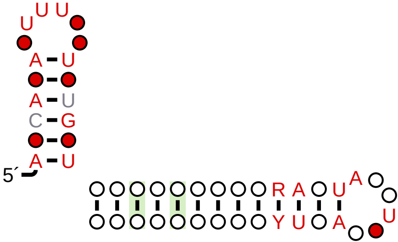

HMP2_11347_1

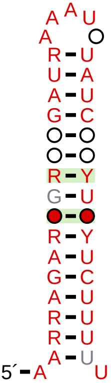

HMP2_11349_1

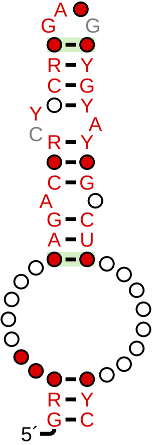

HMP2_11357_1

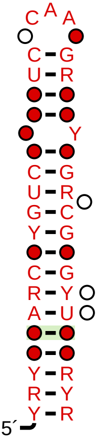

HMP2_11365_1

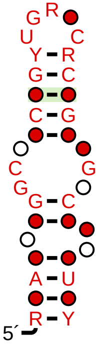

HMP2_11368_1

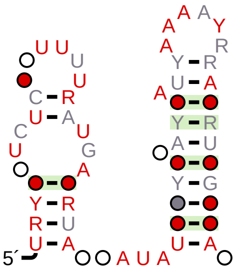

HMP2_11371_1

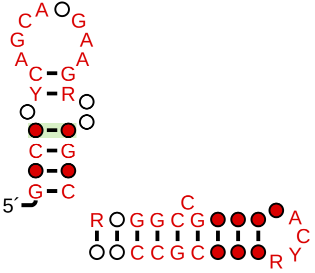

HMP2_11382_1

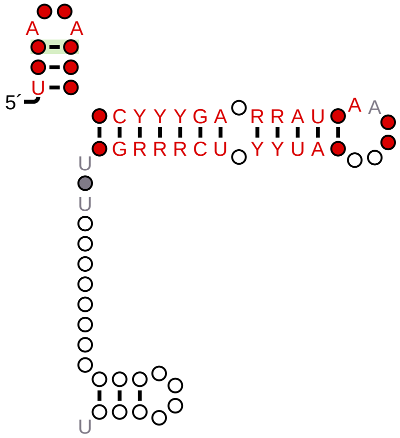

HMP2_1139_1

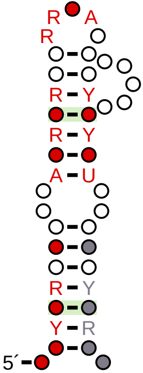

HMP2_11392_1

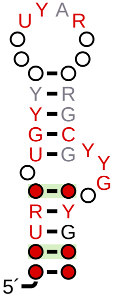

HMP2_11397_1

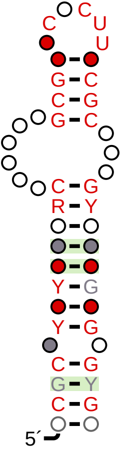

HMP2_11402_1

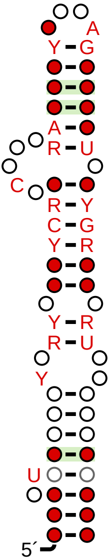

HMP2_11412_1

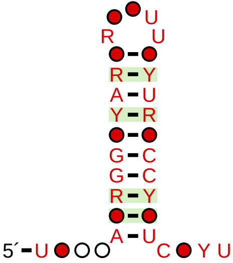

HMP2_11419_1

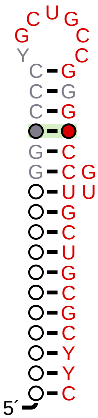

HMP2_11427_1

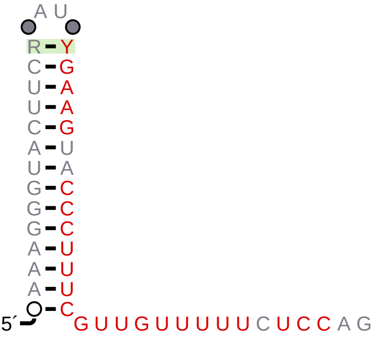

HMP2_11438_1

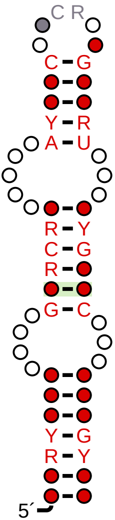

HMP2_1144_1

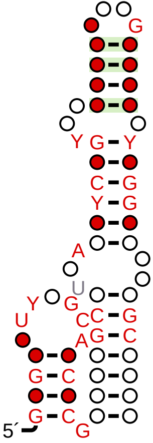

HMP2_11442_1

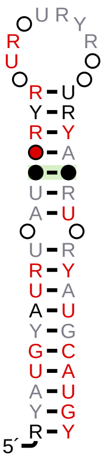

HMP2_11450_1

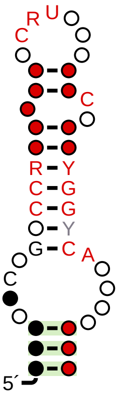

HMP2_11473_1

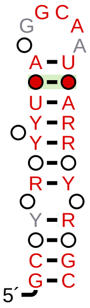

HMP2_1148_1

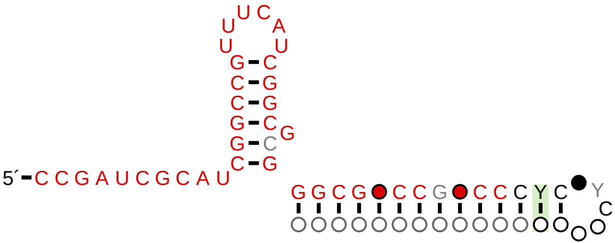

HMP2_11481_1

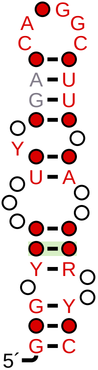

HMP2_11484_1

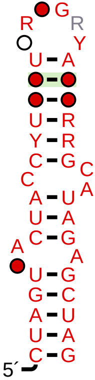

HMP2_11500_1

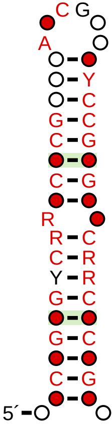

HMP2_11501_1

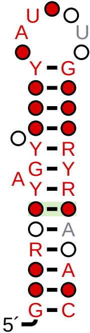

HMP2_11504_1

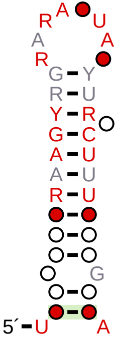

HMP2_11516_1

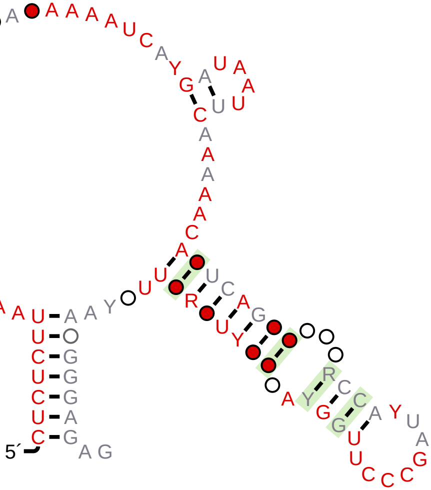

HMP2_1152_1

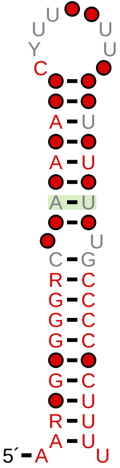

HMP2_11538_1

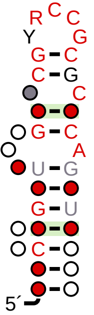

HMP2_1154_1

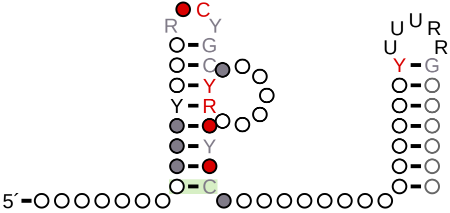

HMP2_11543_1

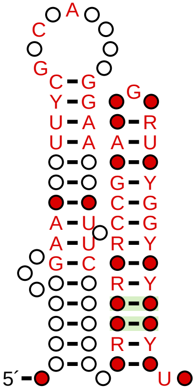

HMP2_11556_1

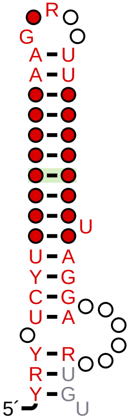

HMP2_11561_1

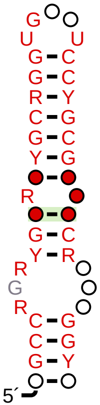

HMP2_11563_1

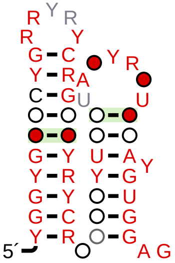

HMP2_11566_1

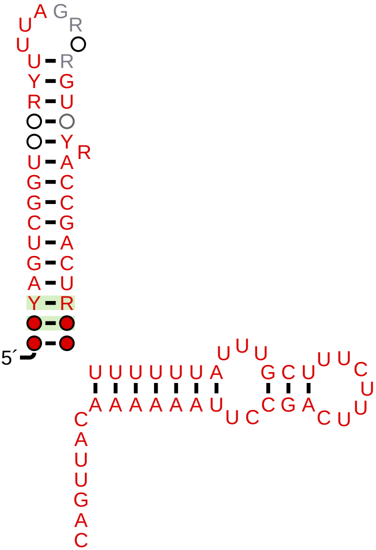

HMP2_11569_1

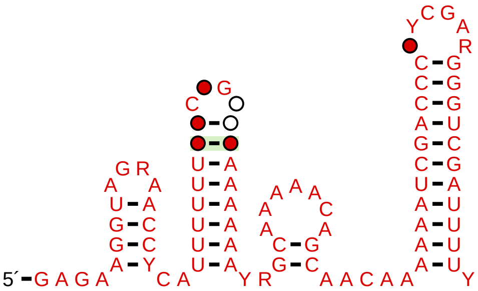

5´
G A G A
A G R A
U G G A
A C C Y
C A
C G
U U U U U U
A A A A A A
Y R
A A A A A
C G
C A
G C
A A C A A
Y C G A R
C C C A G C U A A A A
G G G U C G A U U U U
Y

HMP2_1157_1

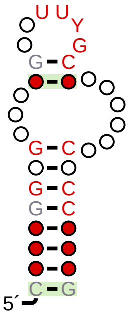

HMP2_11576_1

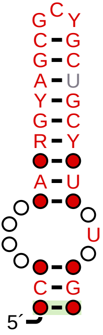

HMP2_11577_1

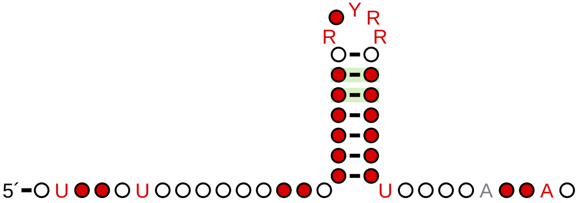

HMP2_11585_1

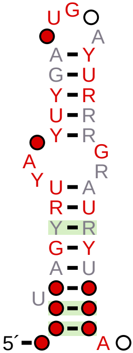

HMP2_11598_1

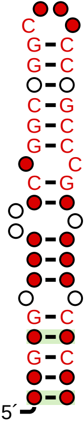

HMP2_116_1

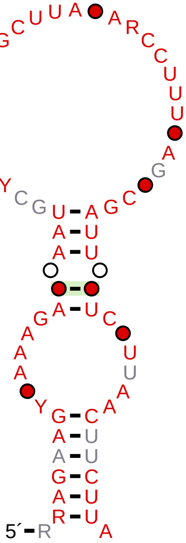

HMP2_11602_1

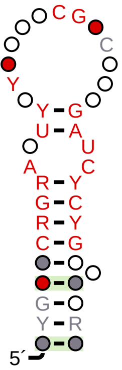

HMP2_11606_1

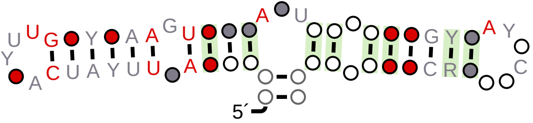

HMP2_11607_1

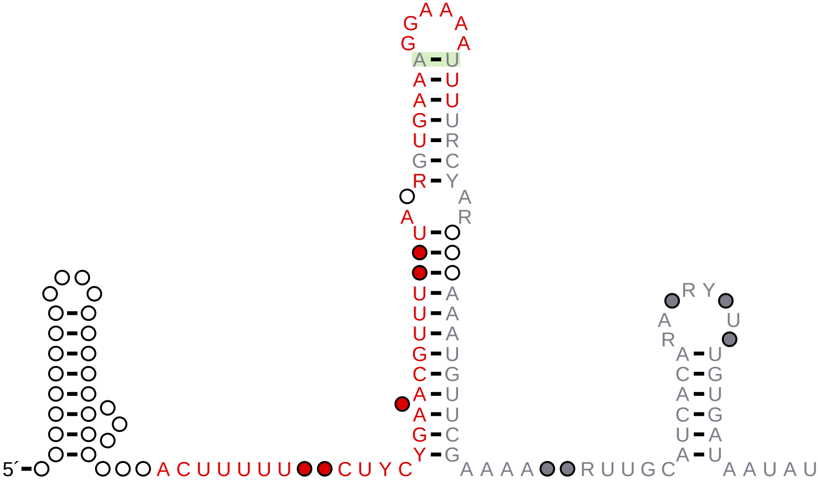

HMP2_11613_1

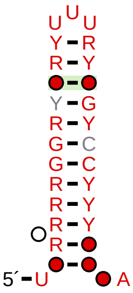

HMP2_11634_1

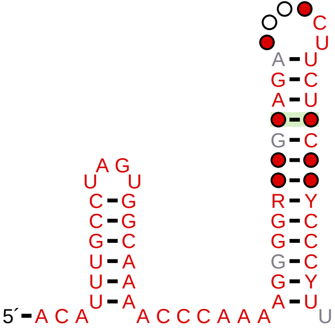

HMP2_11643_1

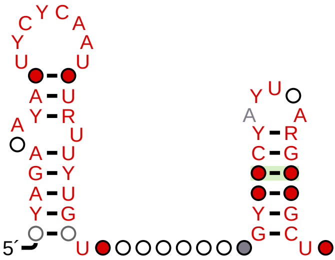

HMP2_11656_1

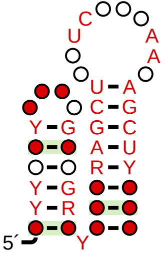

HMP2_1166_1

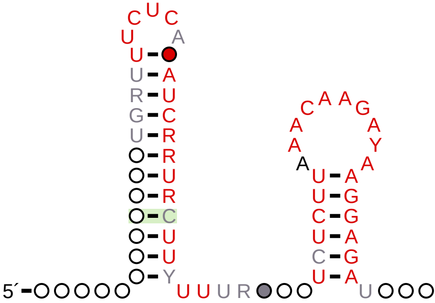

C U C
U A
U
U A
R U
G C
U R
R
U
R
C
U
U
Y
A C A A G
A A
A Y
A A
U A
U G
C G
U A
C G
U A
5´
U U U R
U

HMP2_11662_1

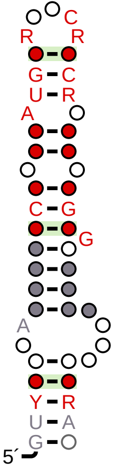

HMP2_11665_1

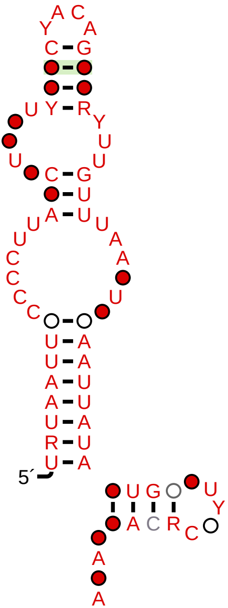

A C
Y A
C G
U Y R Y
U U
U C G U
U
A U
U U
U A
C A
C
C U
C
U A
U A
A U
A U
U A
R U
U A
5´
U G U
Y
A C R C
A
A

HMP2_11672_1

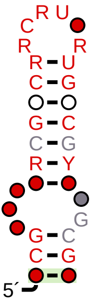

HMP2_11683_1

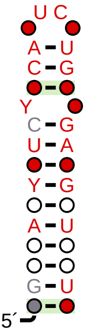

HMP2_11701_1

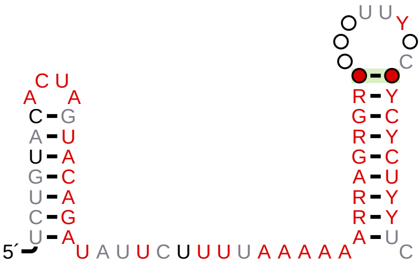

HMP2_11709_1

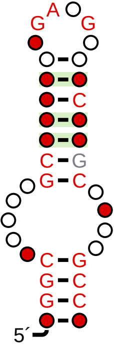

HMP2_11714_1

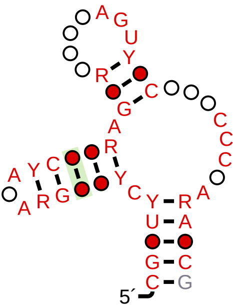

HMP2_11715_1

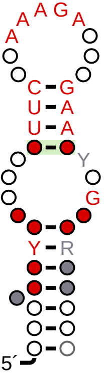

HMP2_1172_1

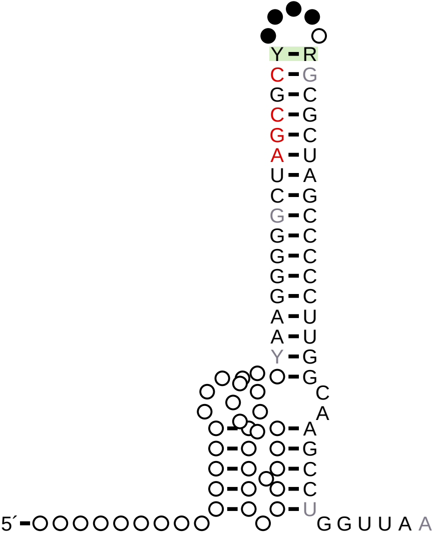

HMP2_11720_1

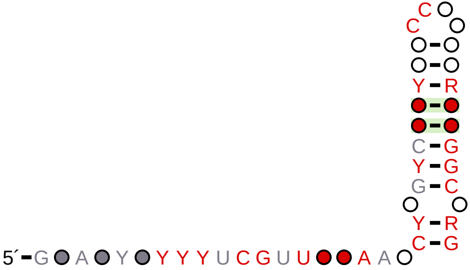

HMP2_11724_1

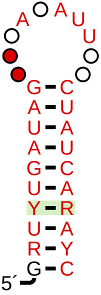

HMP2_11732_1

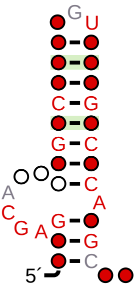

HMP2_11735_1

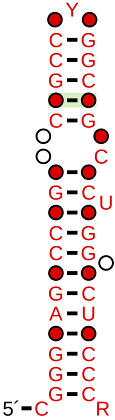

HMP2_11741_1

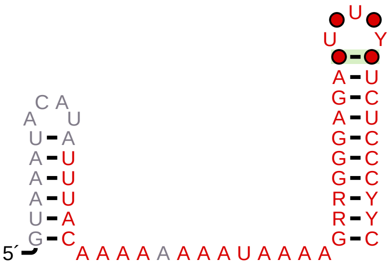
5´
CA
A U
U-A
A-U
A-U
A-U
U-A
G-C
AAAAAAAAUAAAA
U
U Y
A-U
G-C
A-U
G-C
G-C
G-C
R-Y
R-Y
G-C

HMP2_1175_1

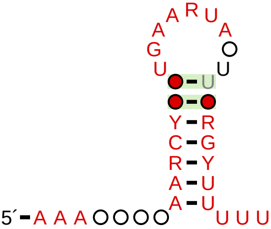

HMP2_11759_1

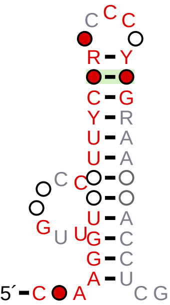

HMP2_1176_1

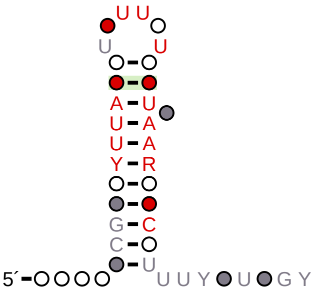

HMP2_11760_1

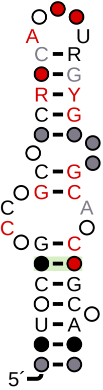

HMP2_11763_1

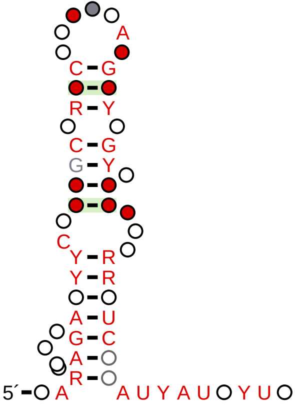

HMP2_11770_1

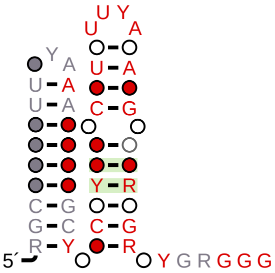

HMP2_11771_1

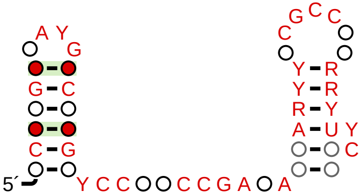

HMP2_11780_1

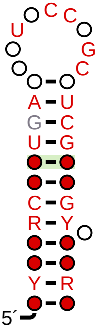

HMP2_11784_1

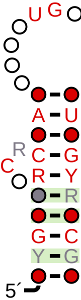

HMP2_11794_1

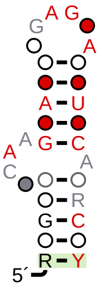

HMP2_1180_1

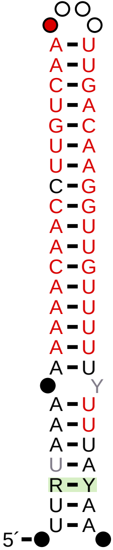
A-U
A-U
C-G
U-A
G-C
U-A
U-A
C-G
C-G
A-U
A-U
C-G
A-U
A-U
A-U
A-U
A-U
Y
A-U
A-U
A-U
U-A
R-Y
U-A
U-A
5´

HMP2_11803_1

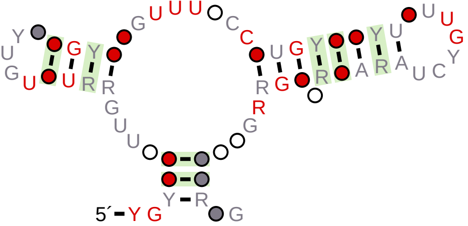

HMP2_11826_1

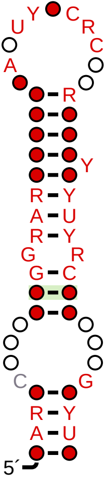

HMP2_11830_1

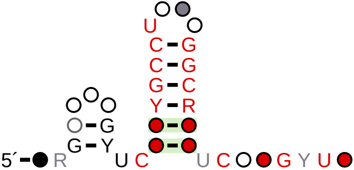

HMP2_11832_1

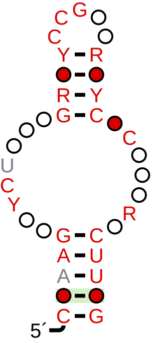

HMP2_11836_1

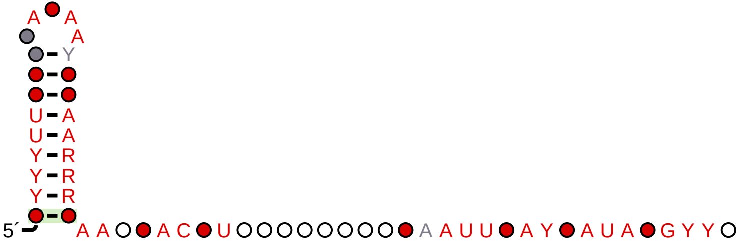

HMP2_11838_1

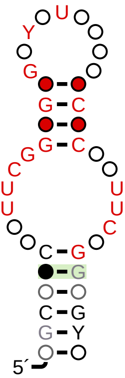

HMP2_1184_1

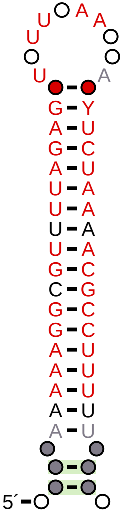

HMP2_11845_1

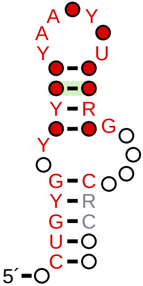

HMP2_11850_1

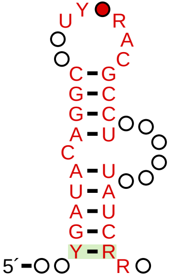

HMP2_11855_1

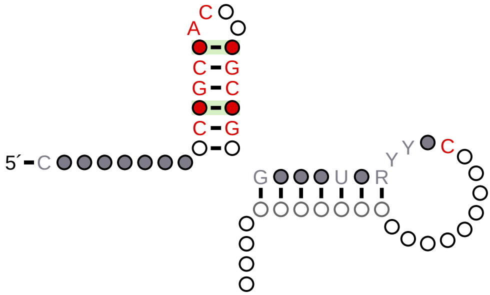

HMP2_11857_1

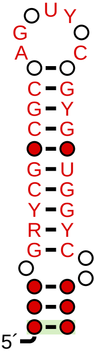

HMP2_11858_1

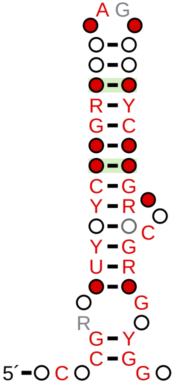

HMP2_1186_1

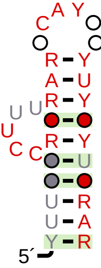

HMP2_11872_1

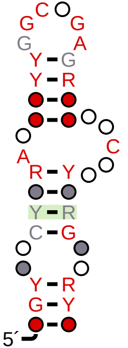

HMP2_11874_1

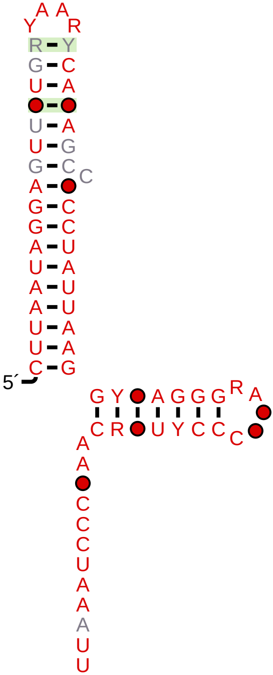

HMP2_11879_1

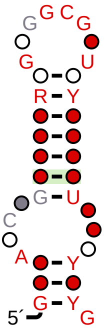

HMP2_11891_1

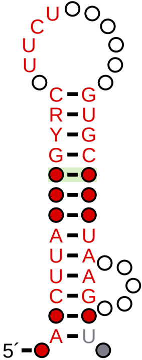

HMP2_11897_1

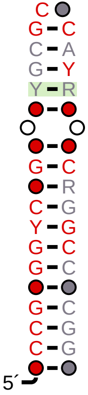

HMP2_11899_1

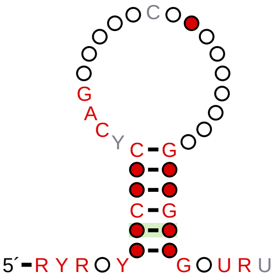

HMP2_11900_1

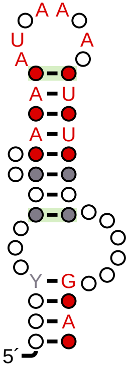

HMP2_11908_1

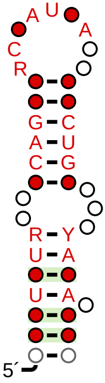

HMP2_11909_1

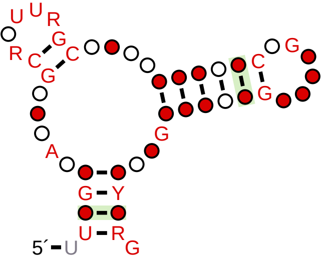

HMP2_11917_1

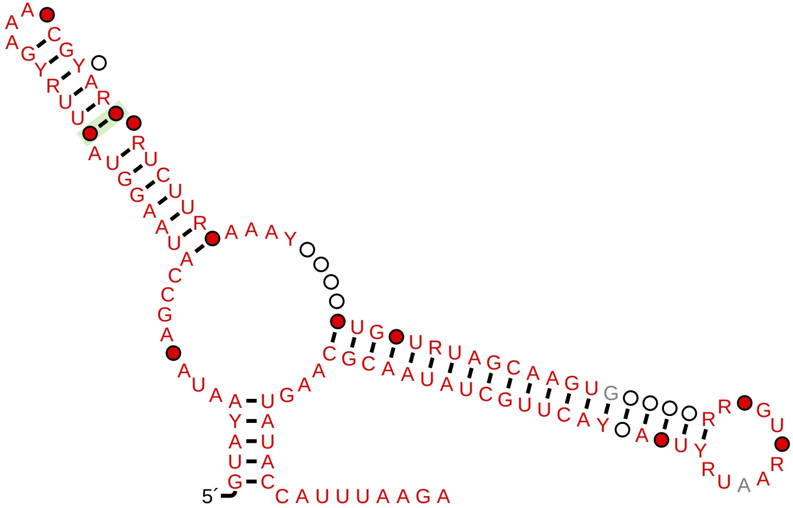

HMP2_11928_1

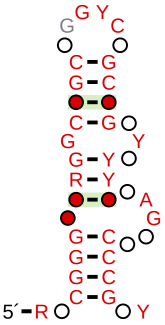

HMP2_11931_1

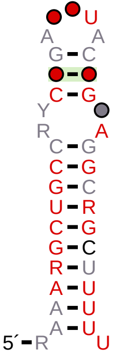

HMP2_11935_1

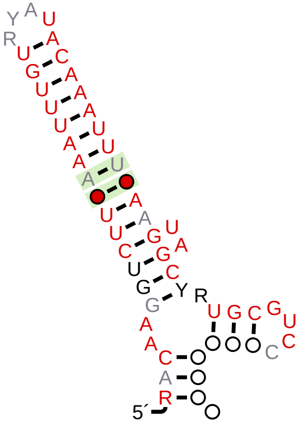

HMP2_11936_1

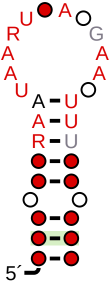

HMP2_11938_1

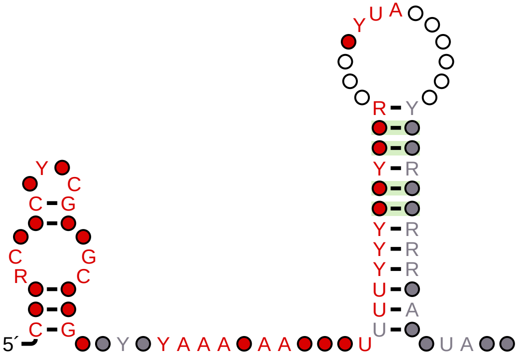

HMP2_1194_1

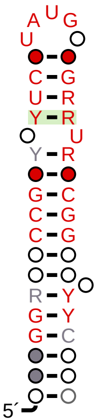

HMP2_1195_1

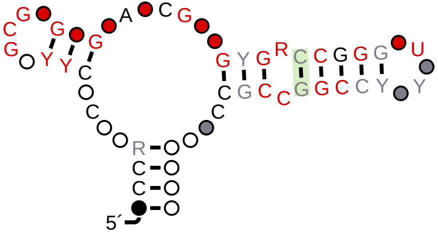

HMP2_11953_1

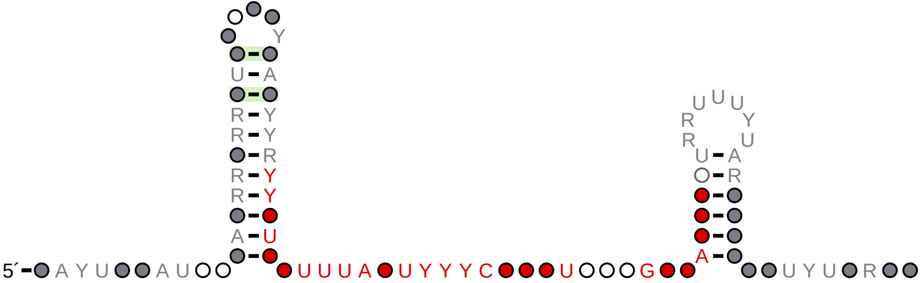

HMP2_1196_1

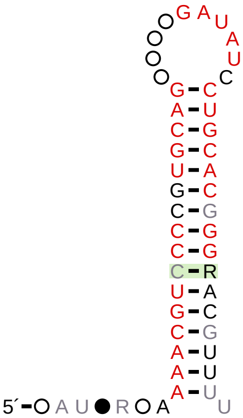

HMP2_11961_1

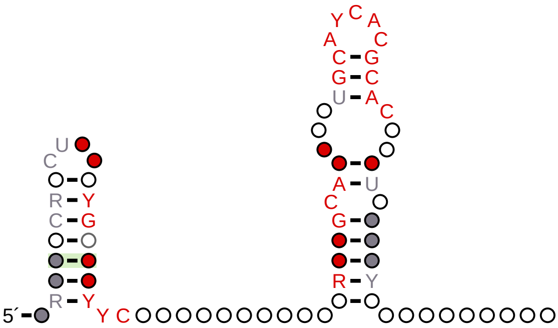

HMP2_11969_1

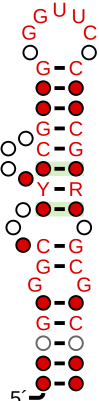

HMP2_11973_1

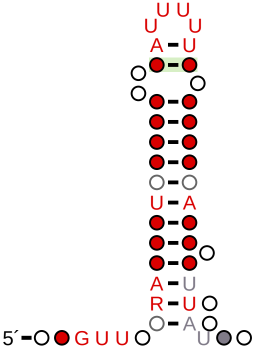

HMP2_11979_1

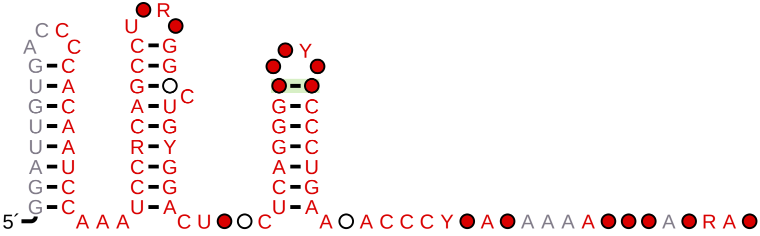

HMP2_11983_1

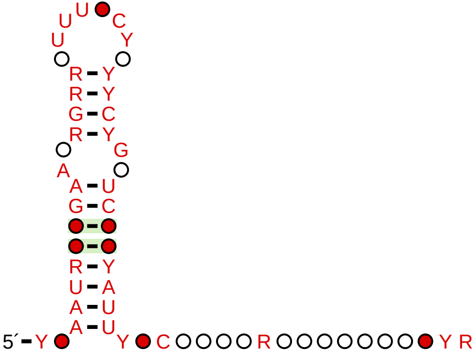

HMP2_11986_1

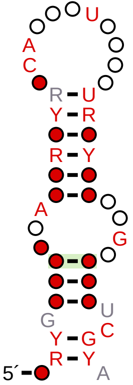

HMP2_120_1

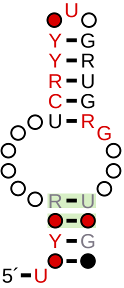

HMP2_12004_1

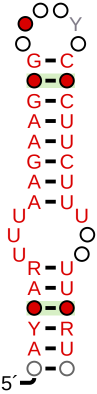

HMP2_12006_1

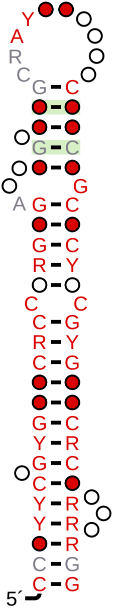

HMP2_12007_1

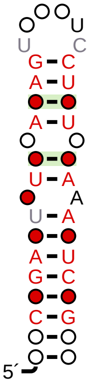

HMP2_12012_1

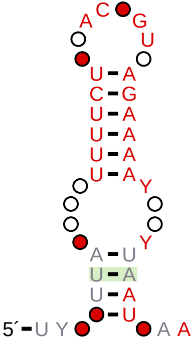

HMP2_12022_1

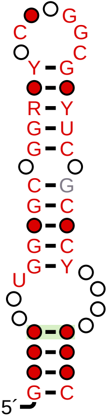

HMP2_12029_1

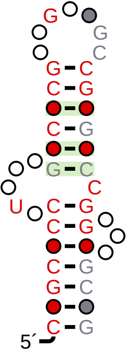

HMP2_1203_1

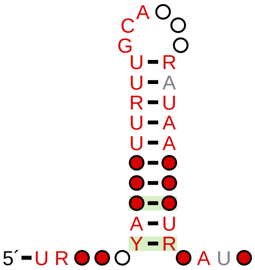

HMP2_12043_1

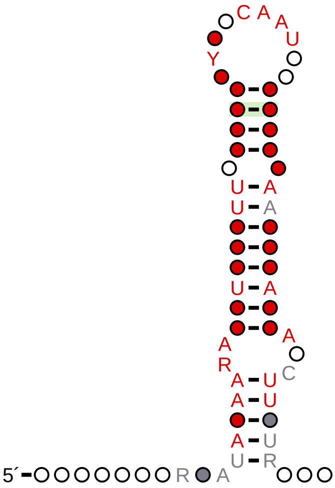

HMP2_12048_1

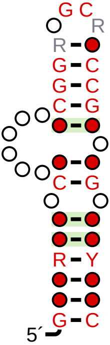

HMP2_12049_1

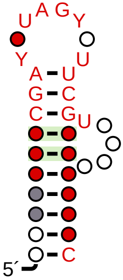

HMP2_1206_1

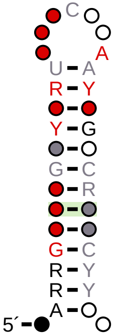

HMP2_12062_1

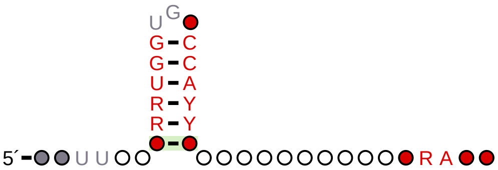

HMP2_1207_1

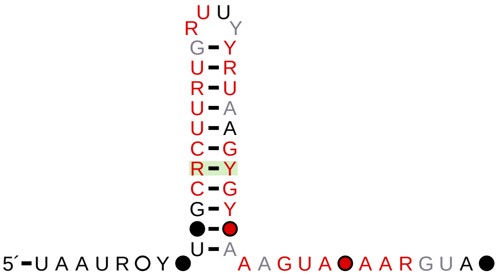

HMP2_12074_1

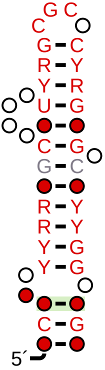

HMP2_12080_1

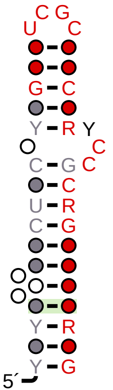

HMP2_12090_1

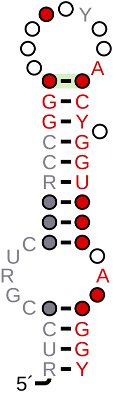

HMP2_12100_1

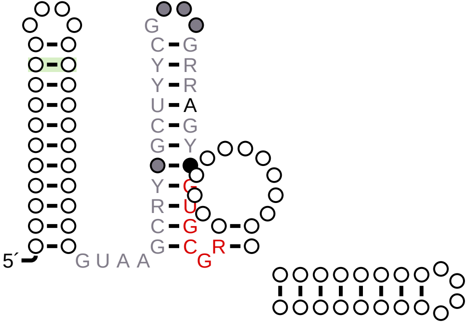

HMP2_12103_1

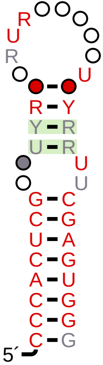

HMP2_12108_1

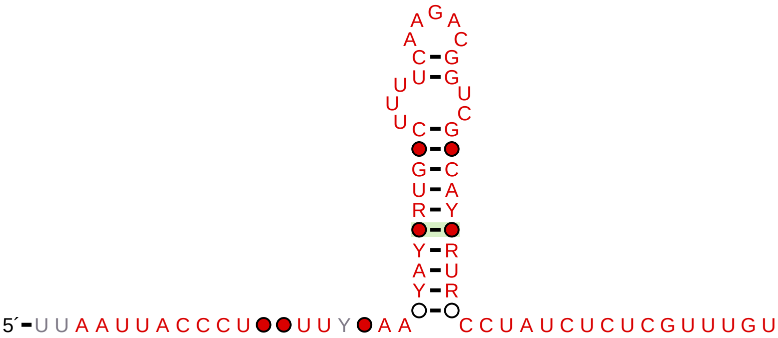
5´
U U A A U U A C C C U U U Y A A
C C U A U C U C U C G U U U G U
Y A Y R U G
R U R Y A C
A G A
A C
C G
U G
U U U
U C
C G

HMP2_1211_1

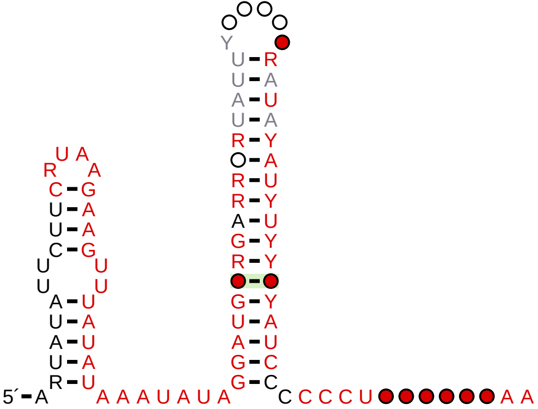

HMP2_12114_1

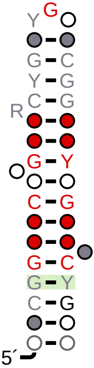

HMP2_12122_1

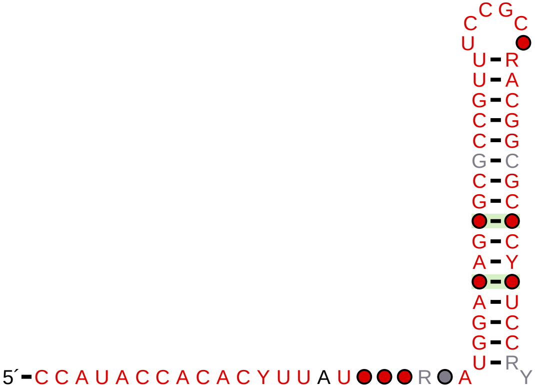

C C G
C C
U
U R
U A
G C
C G
C G
G C
C G
G C
G C
A Y
A U
G C
G C
U R
5´ C C A U A C C A C A C Y U U A U R A
Y

HMP2_12123_1

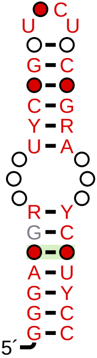

HMP2_12137_1

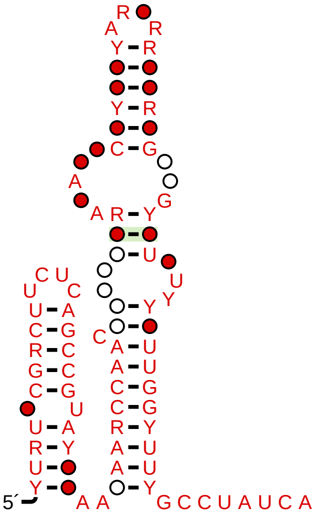

R
A R
Y R
Y R
C G
A
G
A R Y
U
U
C U
U C
U A
Y
Y
C G
C
R C
A U
G C
A U
C G
C G
C G
U
R Y
U A
A U
R Y
A U
U
Y
Y
5´
A A
G C C U A U C A

HMP2_12152_1

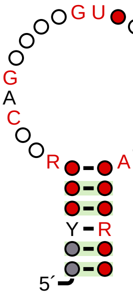

HMP2_12153_1

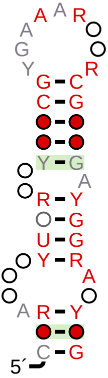

HMP2_12161_1

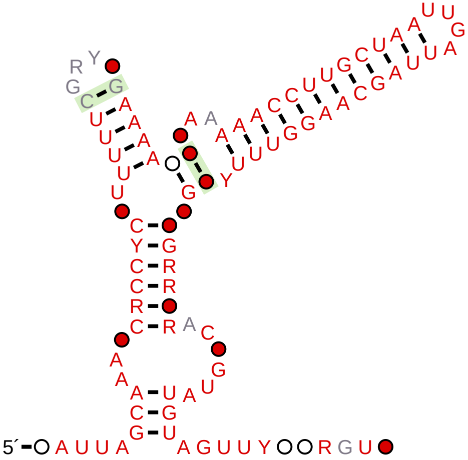

HMP2_12164_1

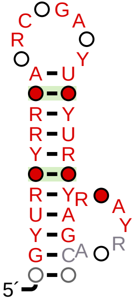

HMP2_1217_1

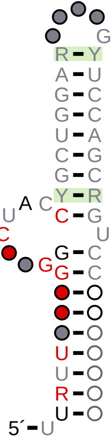

HMP2_12177_1

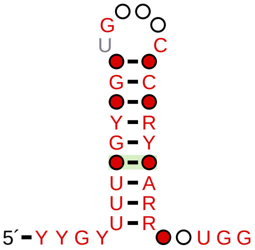

HMP2_12190_1

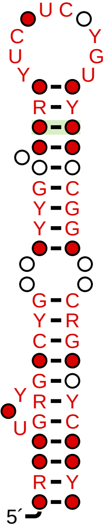

HMP2_12193_1

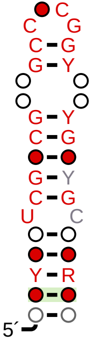

HMP2_122_1

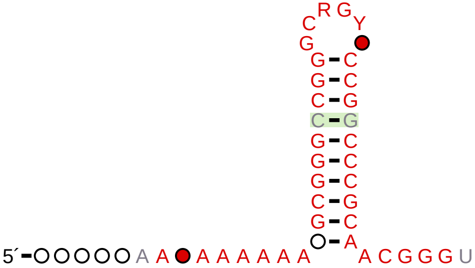

HMP2_12203_1

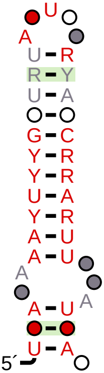

HMP2_12235_1

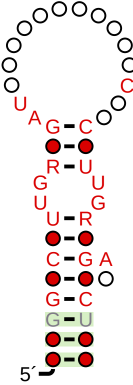

HMP2_12236_1

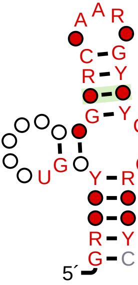

HMP2_12247_1

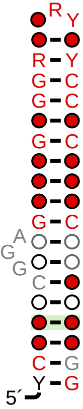

HMP2_12255_1

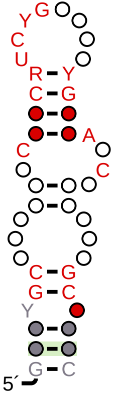

HMP2_12263_1

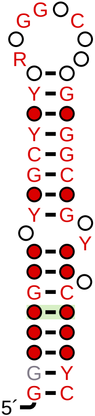

HMP2_12270_1

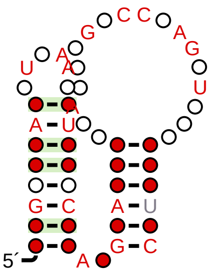

HMP2_12274_1

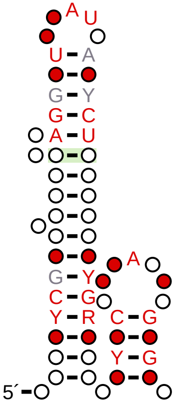

HMP2_12277_1

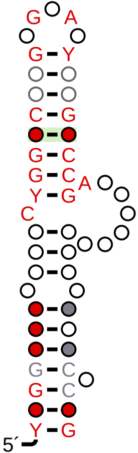

HMP2_12288_1

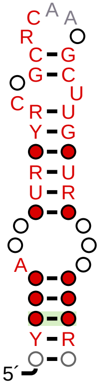

HMP2_12294_1

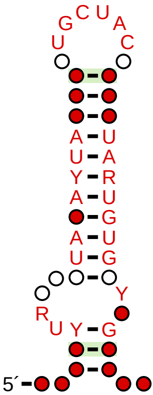

HMP2_12297_1

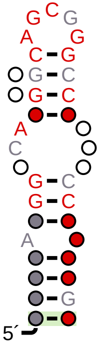

HMP2_12309_1

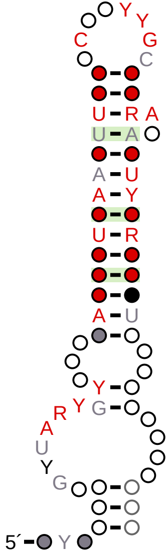

HMP2_1231_1

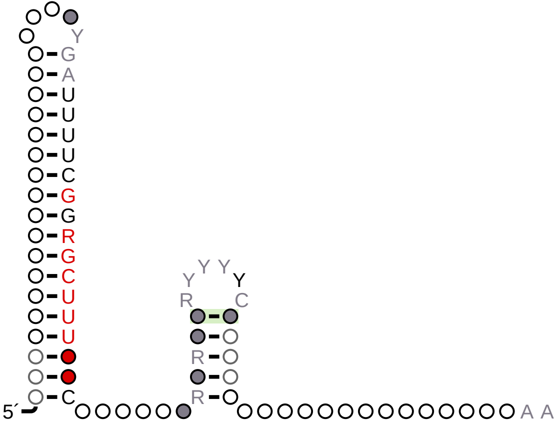

HMP2_12320_1

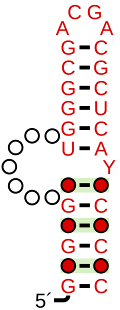

HMP2_12321_1

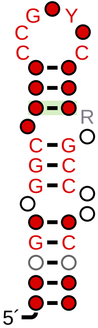

HMP2_12338_1

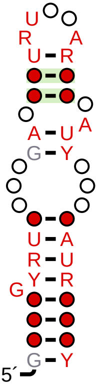

HMP2_1236_1

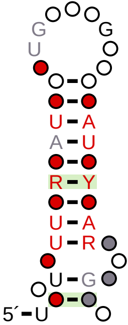

HMP2_12362_1

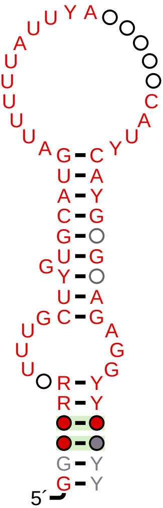

A
Y
U
U
A
U
U
U
U
U
A
G
C
U
A
C
G
C
Y
U
A
C
A
G
U
A
Y
C
G
G
U
G
Y
U
A
C
G
G
U
A
U
G
U
G
R
Y
R
Y
G
Y
G
Y
5´

HMP2_12375_1

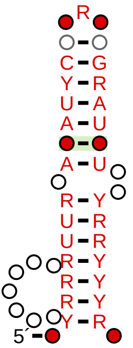

HMP2_12381_1

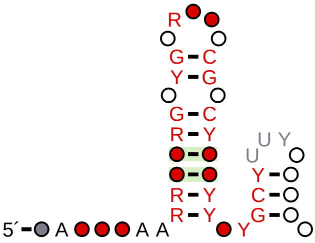

HMP2_12385_1

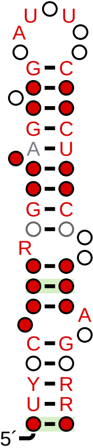

HMP2_12389_1

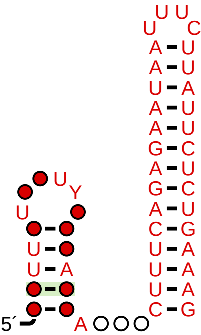

HMP2_1241_1

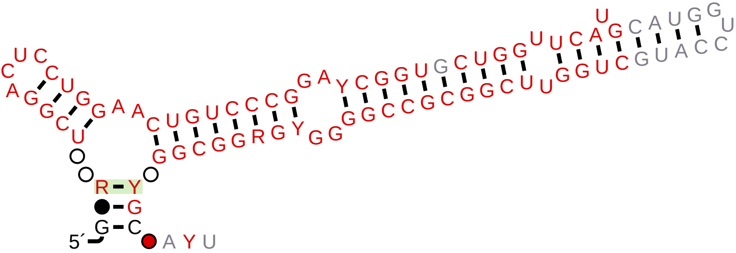

HMP2_12429_1

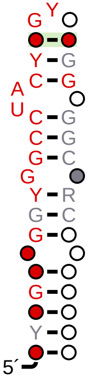

HMP2_12443_1

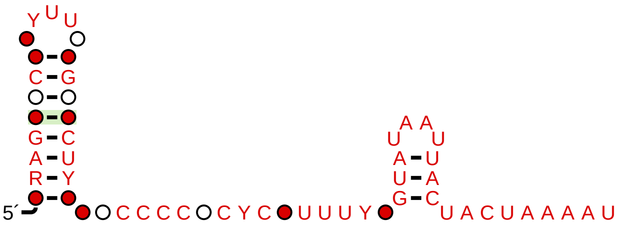

HMP2_12449_1

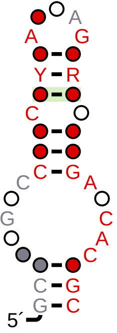

HMP2_1245_1

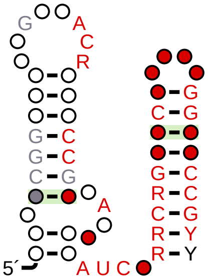

HMP2_12451_1

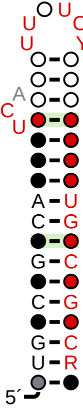

HMP2_12483_1

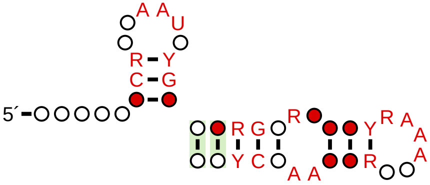

HMP2_12512_1

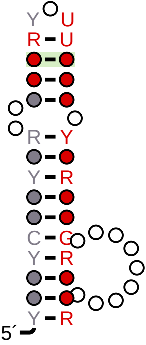

HMP2_12528_1

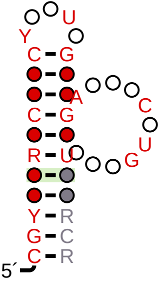

HMP2_12542_1

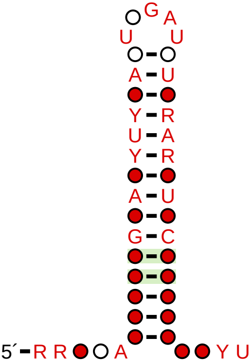

HMP2_12544_1

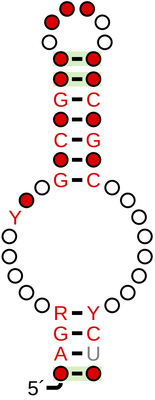

HMP2_12556_1

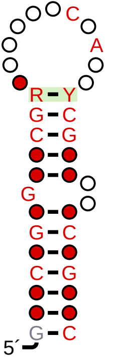

HMP2_12562_1

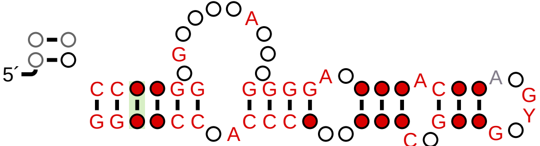

HMP2_12575_1

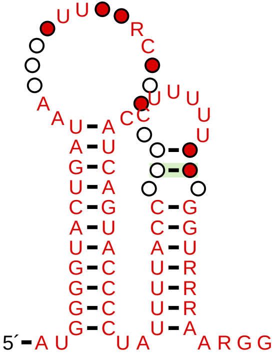

HMP2_1258_1

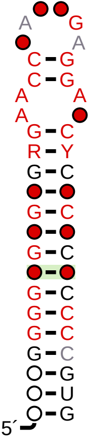

HMP2_12581_1

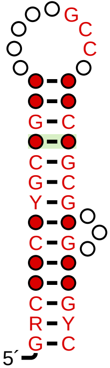

HMP2_12584_1

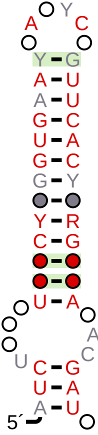

HMP2_126_1

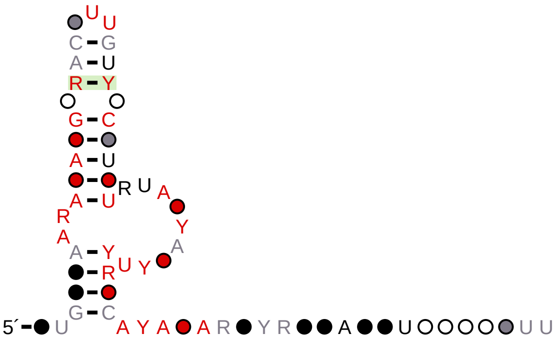

HMP2_12612_1

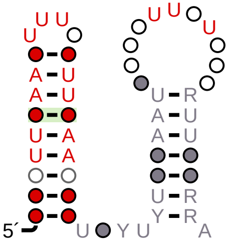

HMP2_12620_1

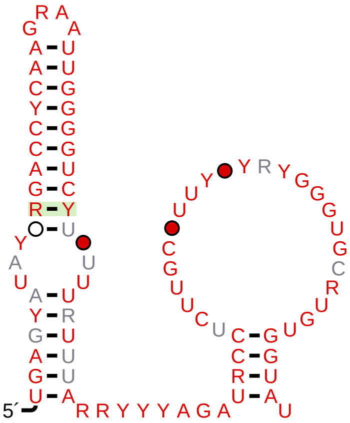

HMP2_12622_1

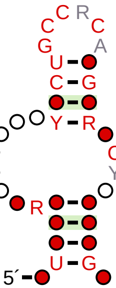

HMP2_12626_1

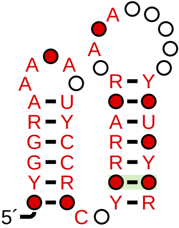

HMP2_12628_1

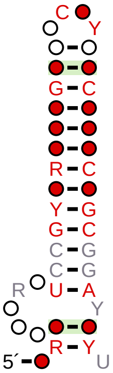

HMP2_12642_1

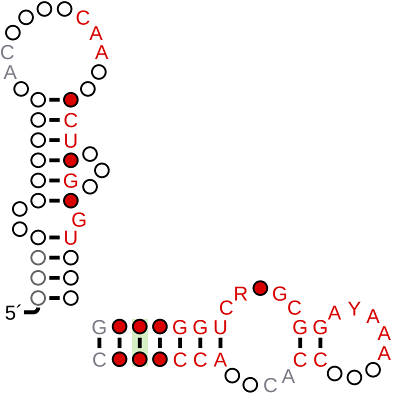

HMP2_12646_1

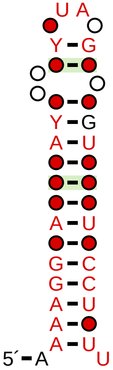

HMP2_1266_1

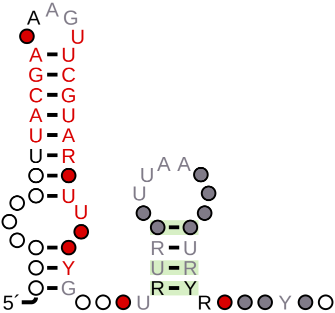

HMP2_12668_1

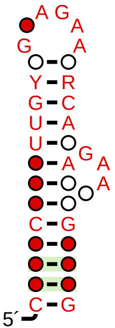

HMP2_12670_1

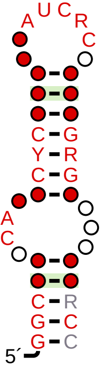

HMP2_12699_1

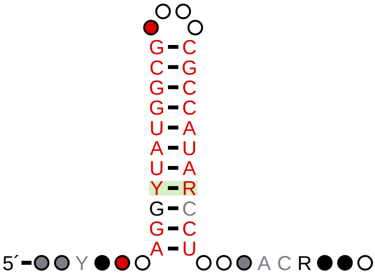

HMP2_12701_1

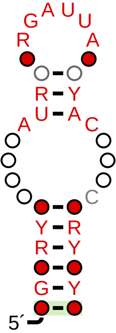

HMP2_12731_1

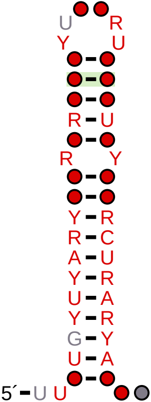

HMP2_12733_1

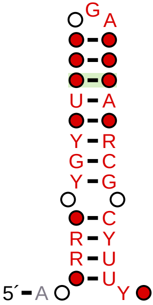

HMP2_12747_1

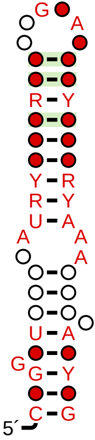

HMP2_12765_1

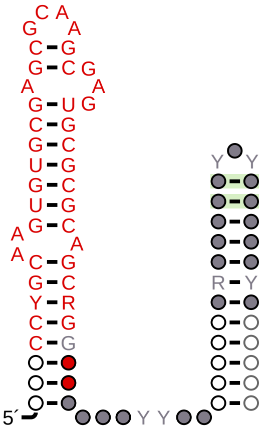

HMP2_1277_1

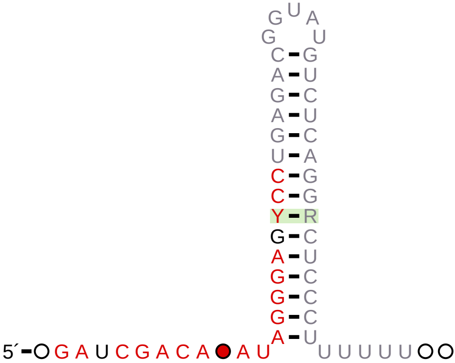

5´
G A U C G A C A
A U
A G G G A G Y C C U G A G A C
G G U A U
G A C U C A G G R C U C C C U
U U U U U

HMP2_12771_1

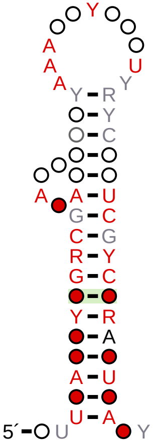

HMP2_12773_1

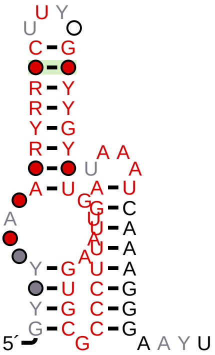

HMP2_12798_1

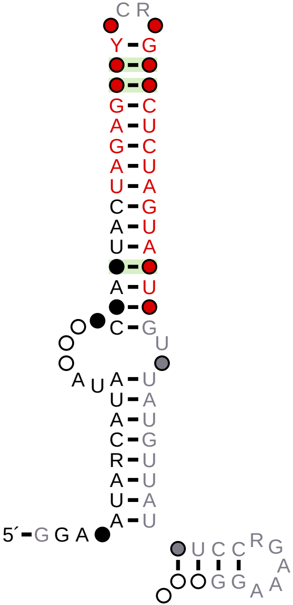

C R
Y G
G C
A U
G C
A U
U A
C G
A U
U A
A U
C G
U
A U A U
U A
A U
C G
R U
A U
U A
A U
5′ G G A
U C C R G
A
G G A A

HMP2_12808_1

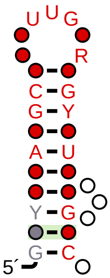

HMP2_12832_1

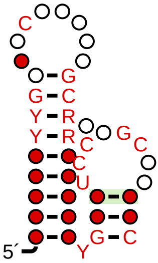

HMP2_1285_1

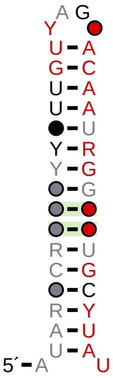

HMP2_12856_1

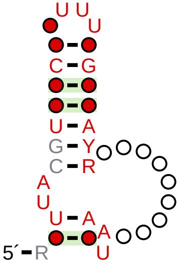

HMP2_12859_1

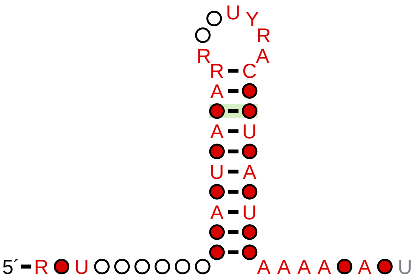

HMP2_12877_1

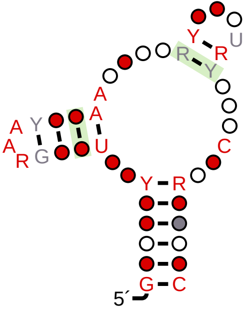

HMP2_12891_1

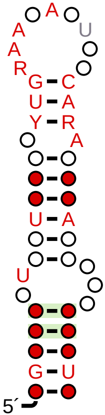

HMP2_12895_1

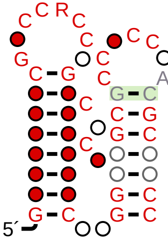

HMP2_12899_1

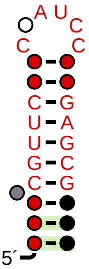

HMP2_12905_1

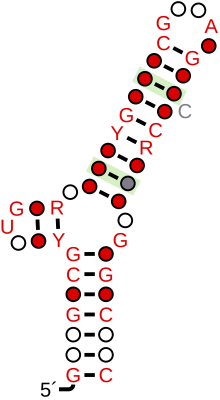

HMP2_12907_1

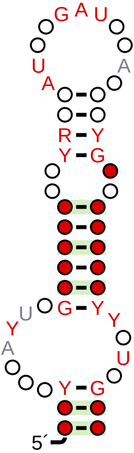

HMP2_1291_1

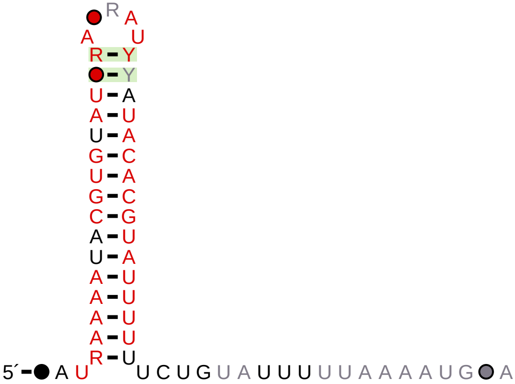
R
A
A
U
R Y
Y
U A
A U
U A
G C
U A
G C
C G
A U
U A
A U
A U
A U
A U
R U
5´ A U
U C U G U A U U U U U A A A A U G A

HMP2_12911_1

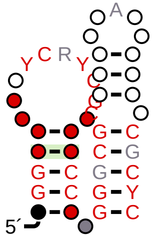

HMP2_12920_1

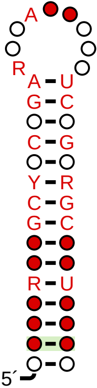

HMP2_12921_1

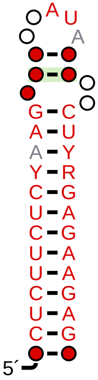

HMP2_12929_1

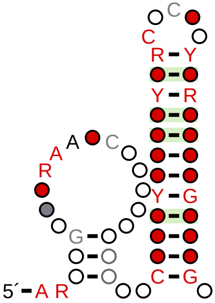

HMP2_12932_1

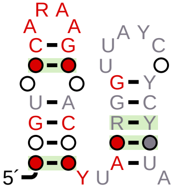

HMP2_12933_1

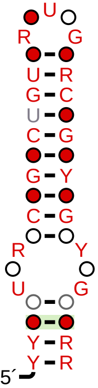

HMP2_12936_1

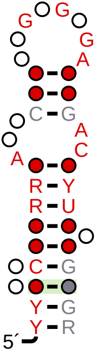

HMP2_12937_1

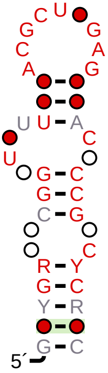

HMP2_12942_1

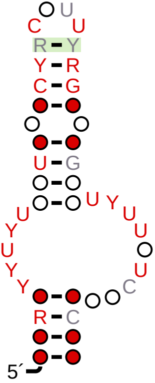

HMP2_12949_1

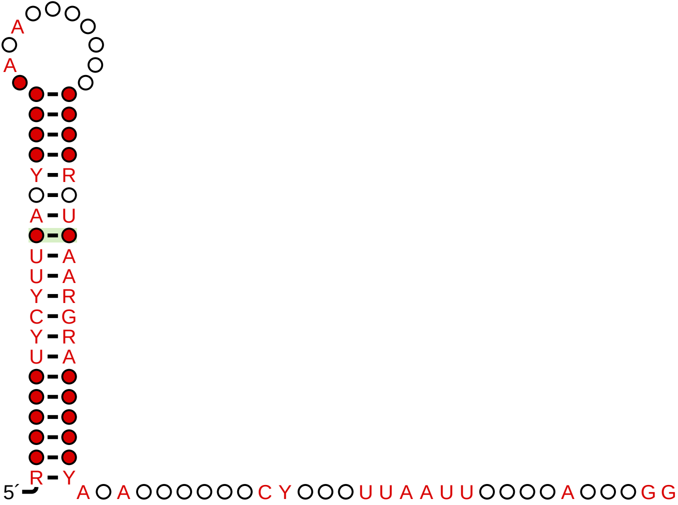

HMP2_12955_1

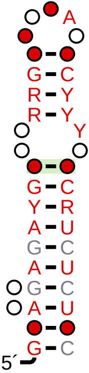

HMP2_12958_1

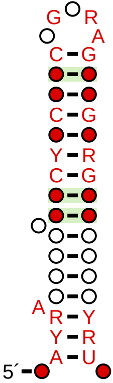

HMP2_12963_1

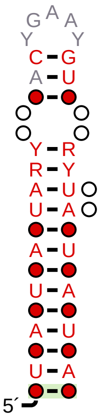

HMP2_12969_1

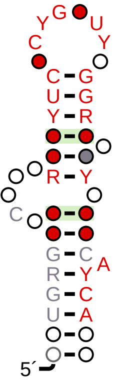

HMP2_1297_1

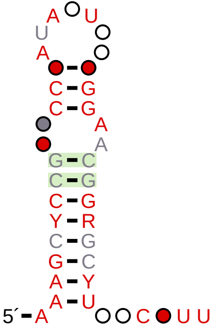

HMP2_1298_1

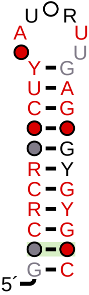

HMP2_1299_1

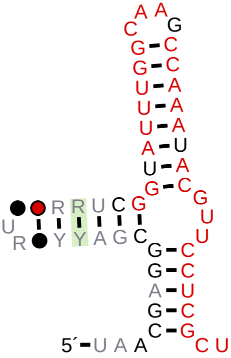

HMP2_12997_1

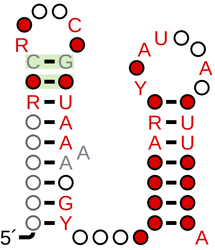

HMP2_13_1

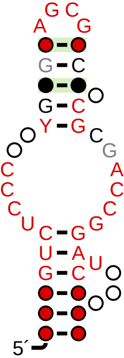

HMP2_13009_1

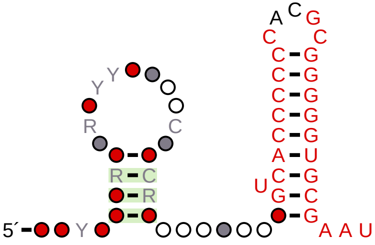

HMP2_1301_1

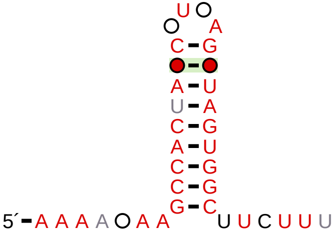

HMP2_1302_1

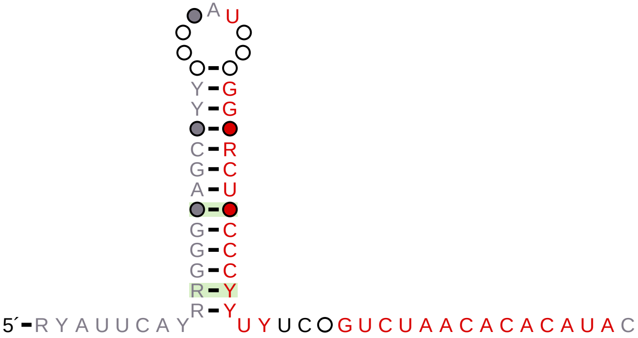
A U
Y G
Y G
C R
G C
A U
G C
G C
G C
R Y
R Y
5´ R Y A U U C A Y
U Y U C
G U C U A A C A C A C A U A C

HMP2_13026_1

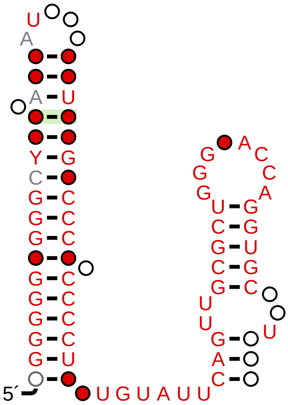

HMP2_13028_1

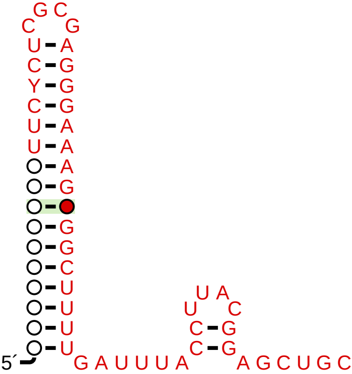

HMP2_13039_1

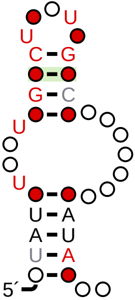

HMP2_13046_1

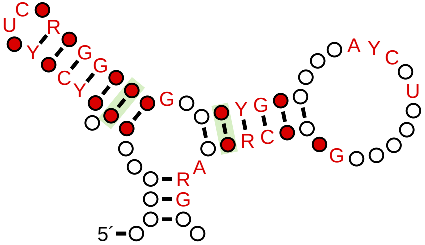

HMP2_13049_1

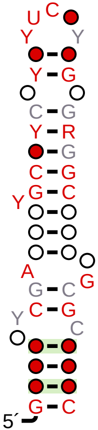

HMP2_1305_1

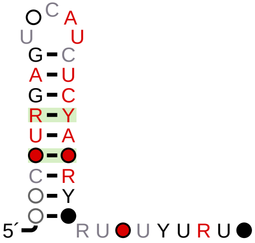

HMP2_13060_1

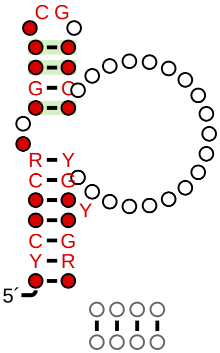

HMP2_13066_1

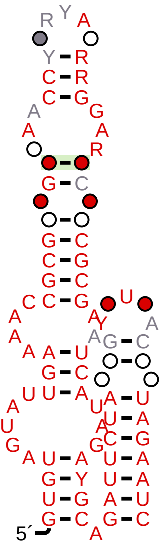

HMP2_13074_1

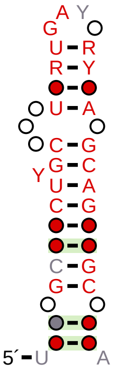

HMP2_13080_1

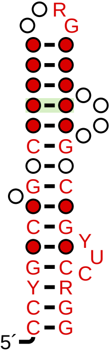

HMP2_13082_1

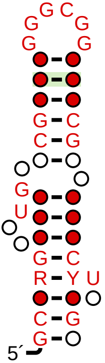

HMP2_1309_1

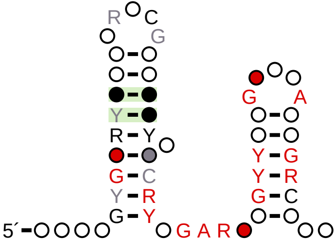

HMP2_13090_1

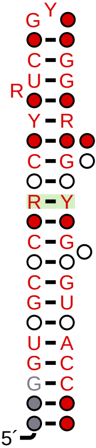

HMP2_13091_1

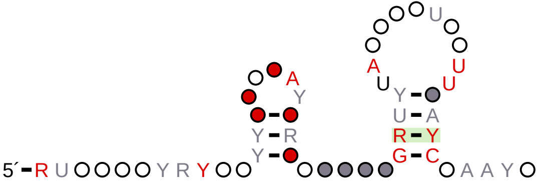

HMP2_13117_1

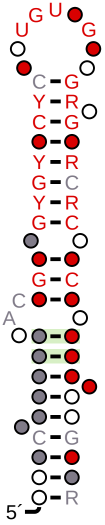

HMP2_1312_1

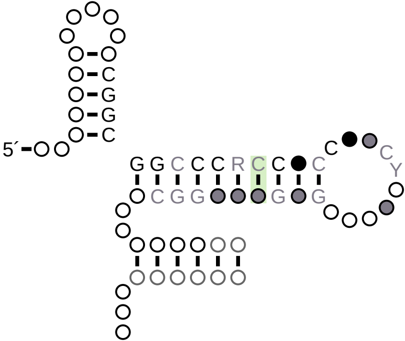

HMP2_13121_1

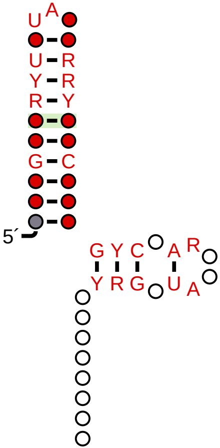

HMP2_13122_1

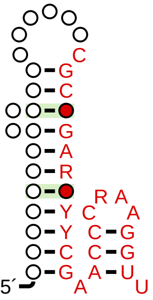

HMP2_13125_1

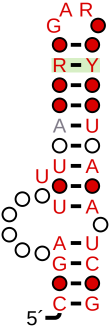

HMP2_13128_1

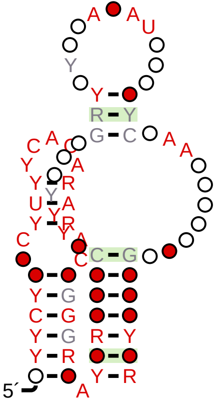

HMP2_1313_1

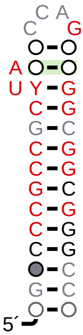

HMP2_13131_1

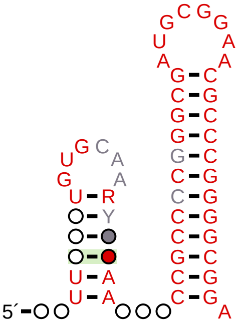

HMP2_13140_1

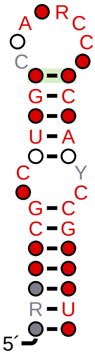

HMP2_13141_1

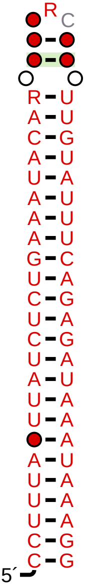
R
C
R U
A U
C G
A U
U A
A U
A U
A U
G C
U A
C G
U A
C G
U A
A U
U A
U A
A
A U
U A
U A
U A
C G
C G
5´

HMP2_13149_1

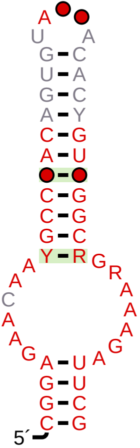

HMP2_13153_1

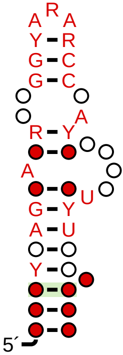

HMP2_13171_1

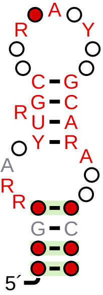

HMP2_13173_1

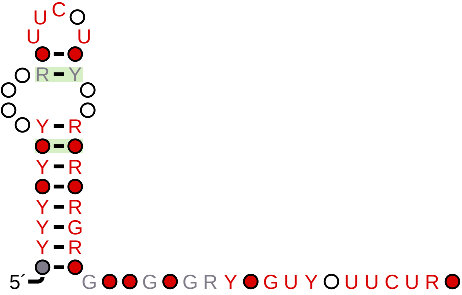

HMP2_13174_1

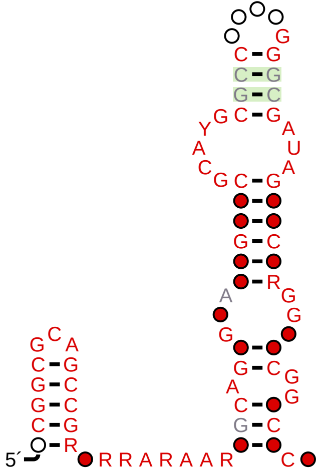

HMP2_13176_1

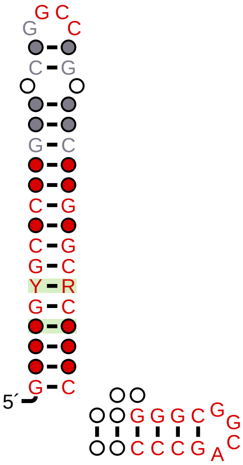

HMP2_13181_1

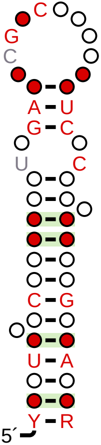

HMP2_13186_1

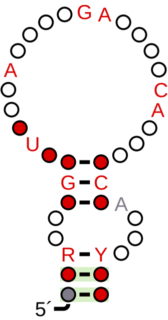

HMP2_13189_1

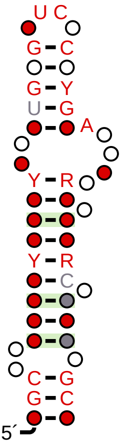

HMP2_13192_1

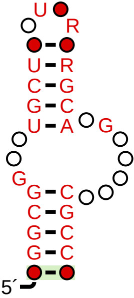

HMP2_132_1

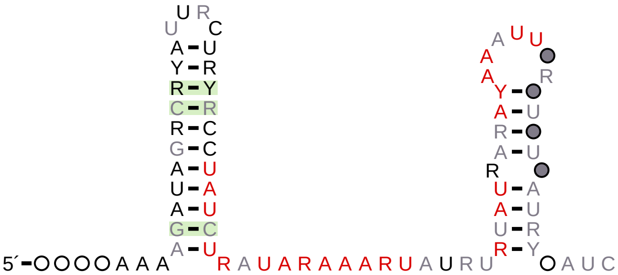

HMP2_1320_1

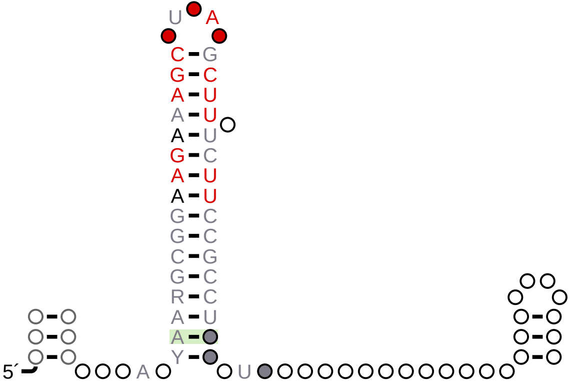

HMP2_13202_1

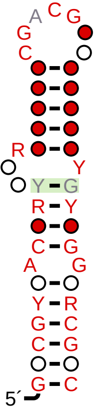

HMP2_13211_1

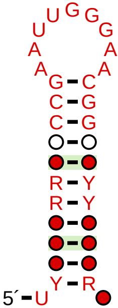

HMP2_1322_1

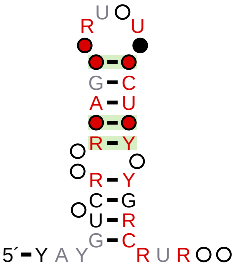

HMP2_13225_1

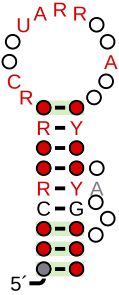

HMP2_13228_1

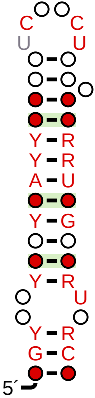

HMP2_13235_1

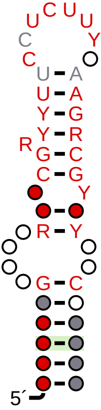

HMP2_1325_1

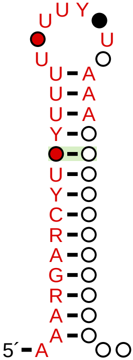

HMP2_13251_1

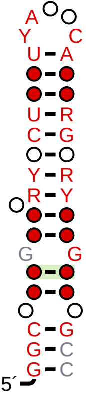

HMP2_13252_1

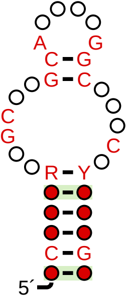

HMP2_1327_1

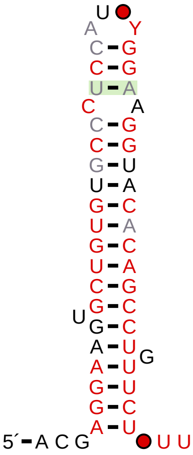

U
A Y
C G
C G
U A
C A
C G
C G
G U
U A
G C
U A
G C
U A
C G
G C
U
G C
A U
G
A U
G U
G C
A U
5´ A C G
U U

HMP2_13271_1

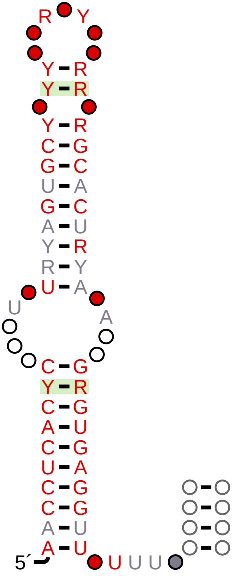

HMP2_13272_1

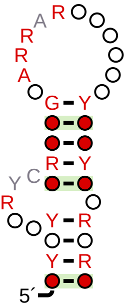

HMP2_13273_1

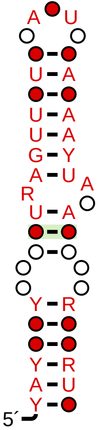

HMP2_13276_1

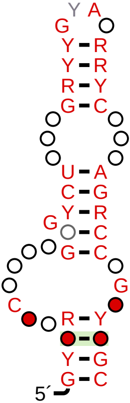

HMP2_13277_1

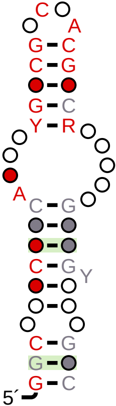

HMP2_13285_1

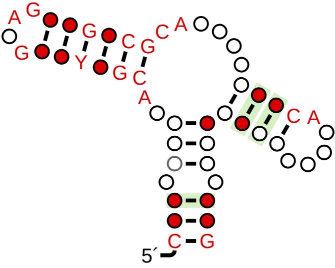

HMP2_13289_1

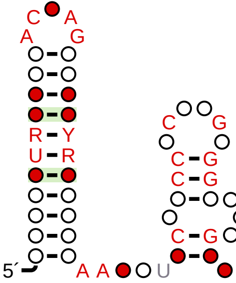

HMP2_13293_1

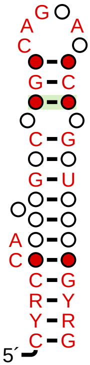

HMP2_13294_1

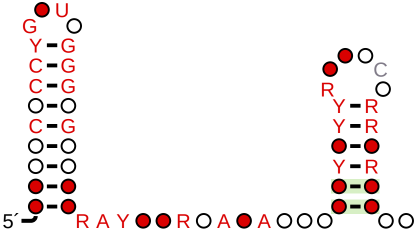

HMP2_13298_1

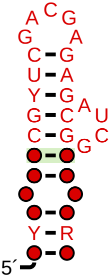

HMP2_1330_1

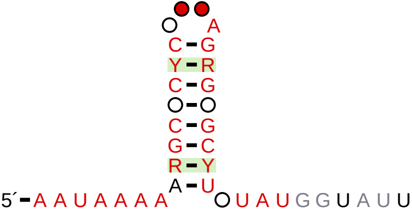

HMP2_13302_1

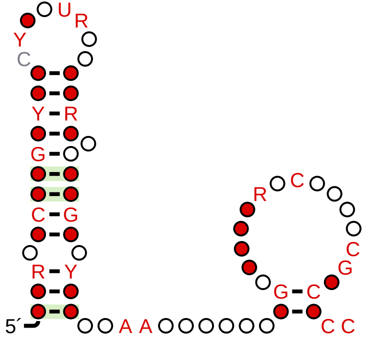

HMP2_13303_1

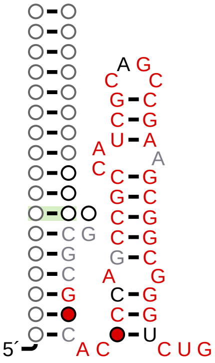

HMP2_13305_1

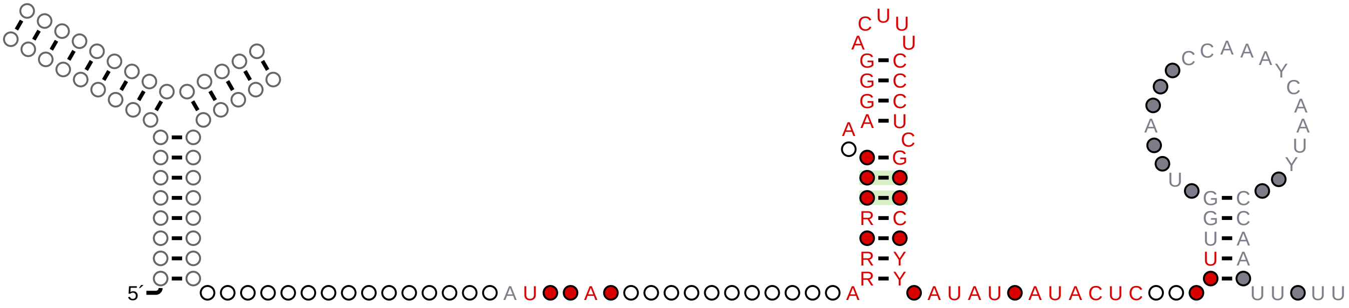

HMP2_13306_1

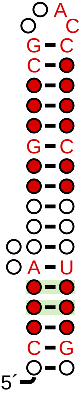

HMP2_13308_1

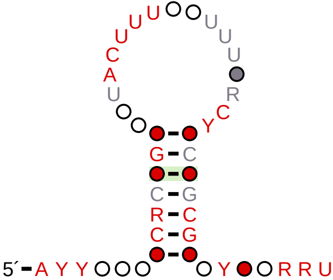

HMP2_13317_1

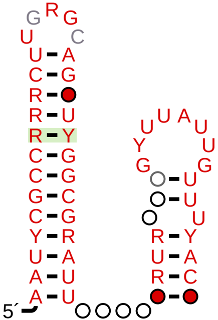

G R G
U C
U A
C G
R
R U
R Y
C G
C G
G C
C G
Y R
U A
A U
A U
5´
U U A U
U U
Y U
G G
U
U
U
R Y
U A
R C

HMP2_13319_1

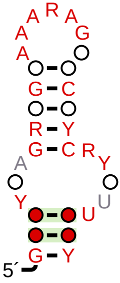

HMP2_1332_1

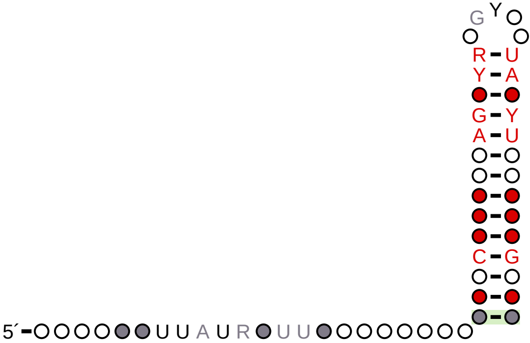

HMP2_13321_1

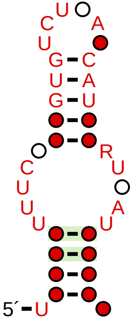

HMP2_13329_1

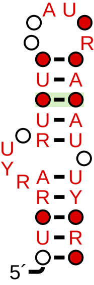

HMP2_13337_1

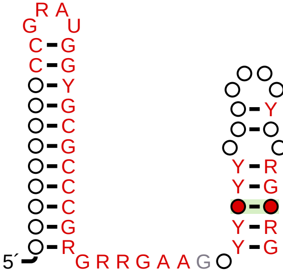

HMP2_13341_1

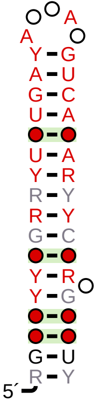

HMP2_13342_1

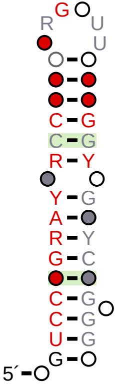

HMP2_13350_1

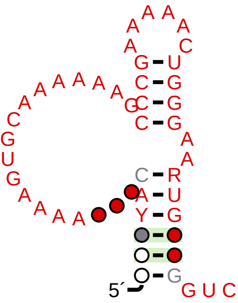

HMP2_1336_1

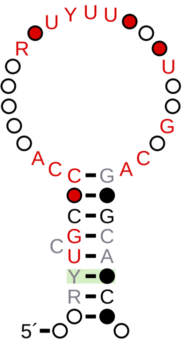

HMP2_13373_1

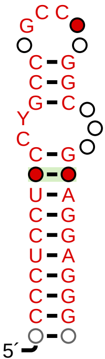

HMP2_13378_1

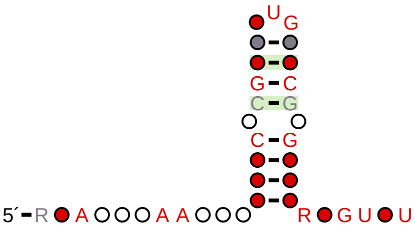

HMP2_13382_1

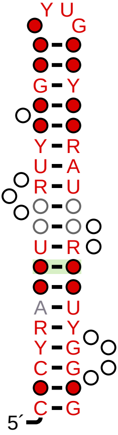

HMP2_13384_1

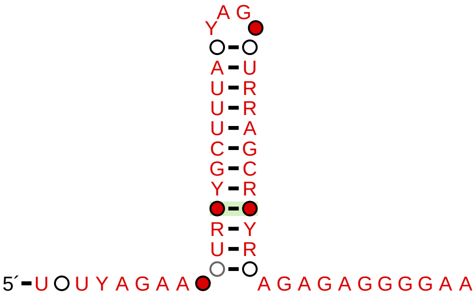

HMP2_13388_1

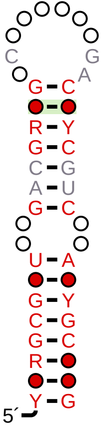

HMP2_13392_1

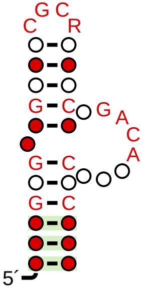

HMP2_13394_1

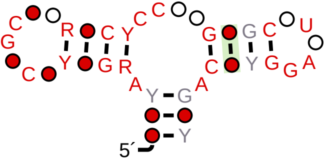

HMP2_134_1

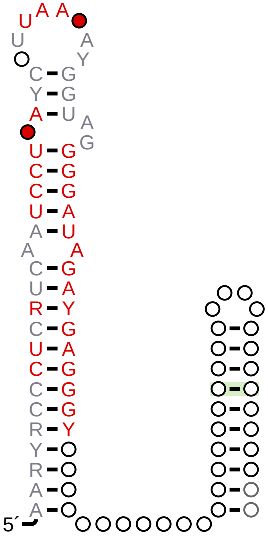

HMP2_1340_1

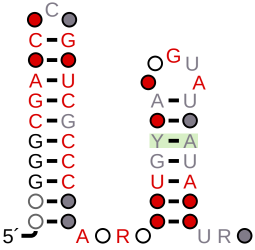

HMP2_1341_1

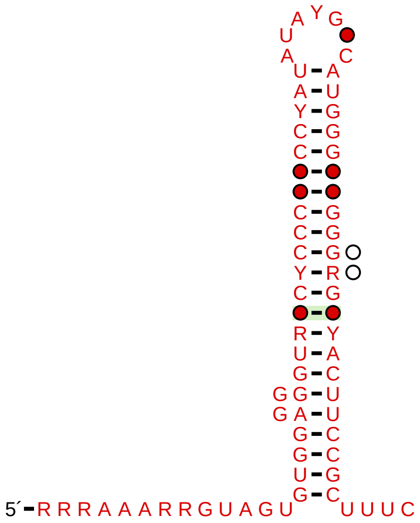

A Y G
U
A C
U A
A U
Y G
C G
C G
C G
C G
C G
Y R
C G
R Y
U A
G C
G G U
G A U
G C
G C
U G
G C
5´ R R R A A A R R G U A G U
U U U C

HMP2_13410_1

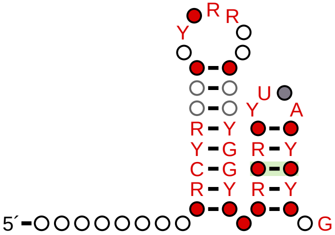

HMP2_13432_1

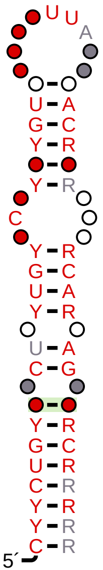

HMP2_13440_1

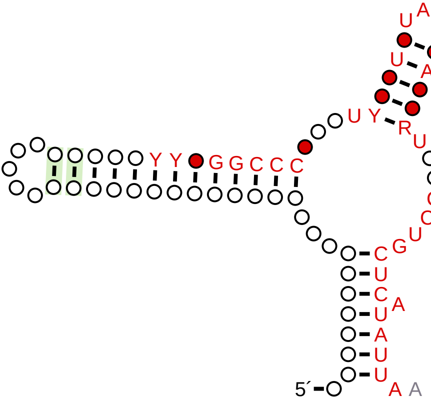

HMP2_13450_1

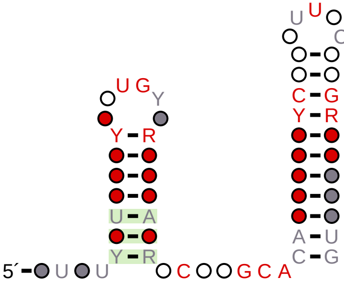

HMP2_13458_1

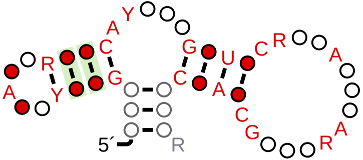

HMP2_1347_1

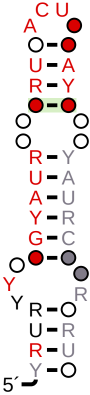

HMP2_13485_1

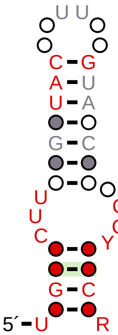

HMP2_13487_1

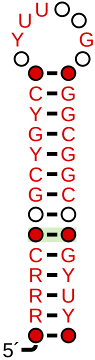

HMP2_1352_1

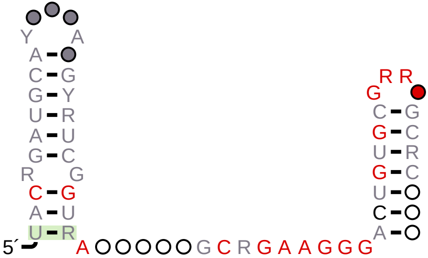

HMP2_13522_1

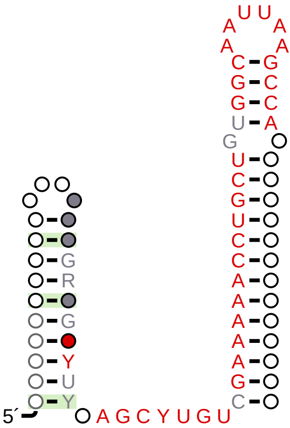

HMP2_13530_1

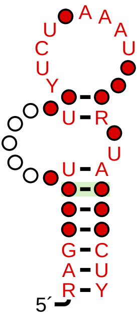

HMP2_13548_1

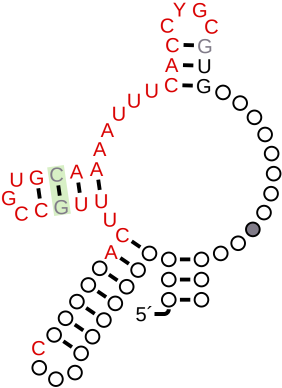

HMP2_13552_1

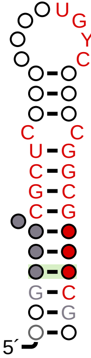

HMP2_13557_1

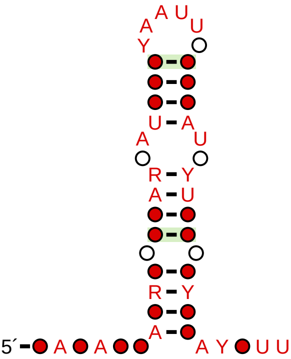

HMP2_13559_1

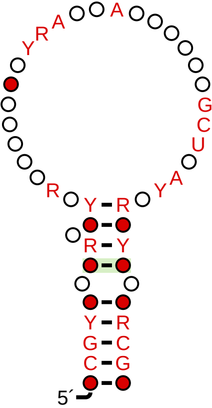

HMP2_13560_1

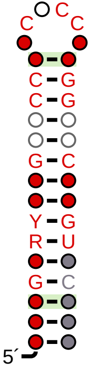

HMP2_13573_1

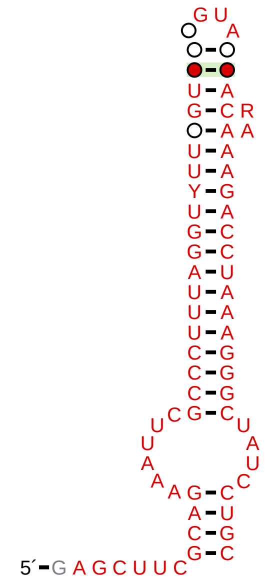

HMP2_13577_1

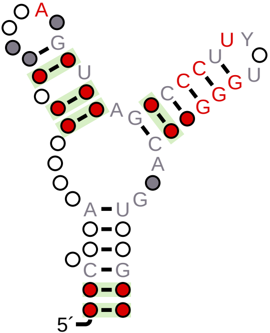

HMP2_13584_1

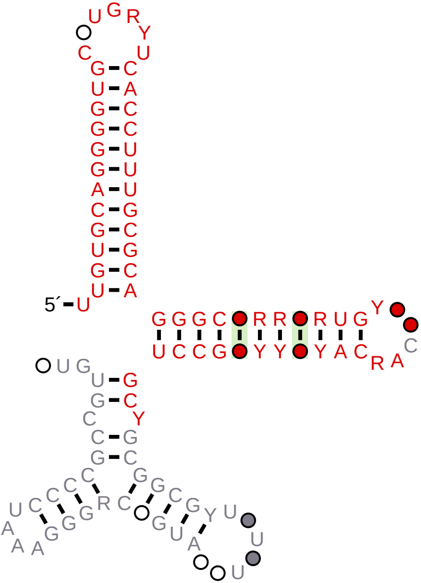

HMP2_13588_1

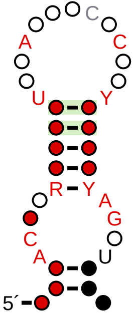

HMP2_13596_1

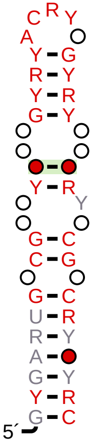

HMP2_13599_1

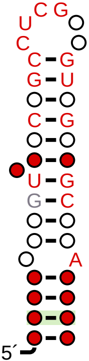

HMP2_1360_1

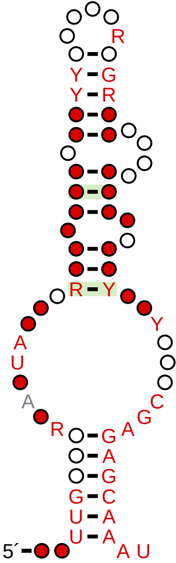

HMP2_13601_1

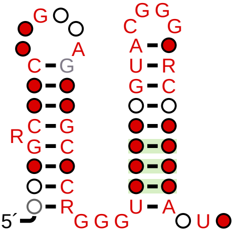

HMP2_13603_1

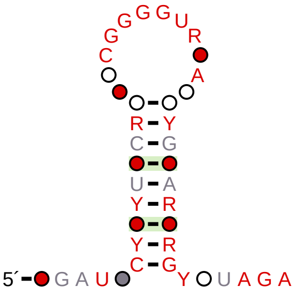

HMP2_13606_1

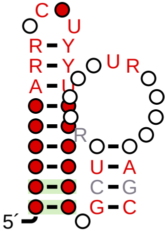

HMP2_13607_1

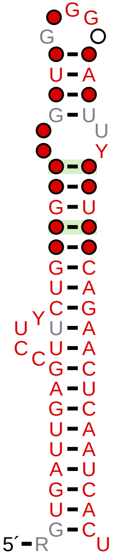

HMP2_13612_1

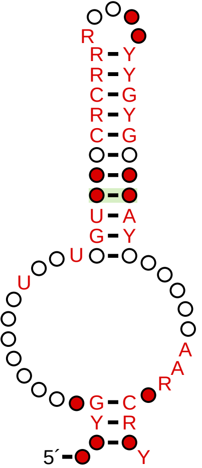

HMP2_13619_1

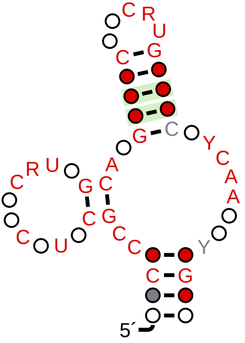

HMP2_13621_1

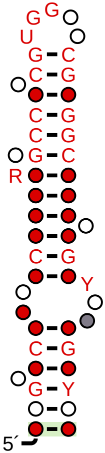

HMP2_13627_1

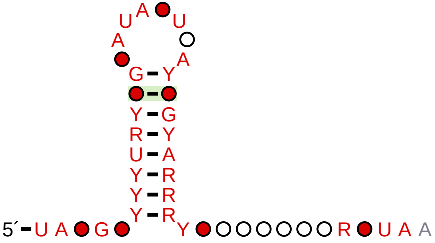

HMP2_13635_1

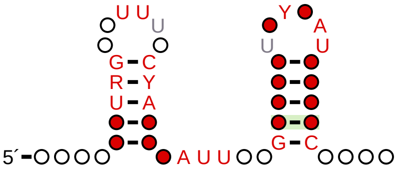

HMP2_13643_1

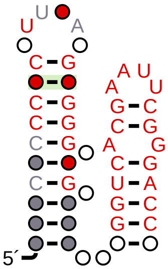

HMP2_13644_1

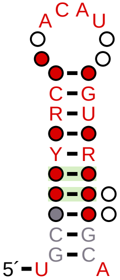

HMP2_13645_1

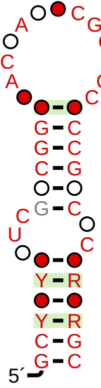

HMP2_13651_1

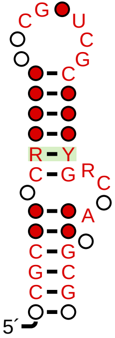

HMP2_13655_1

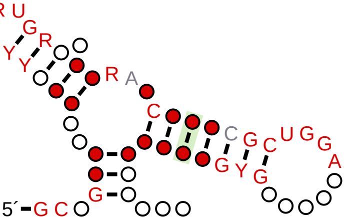

HMP2_13659_1

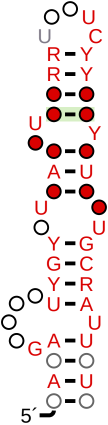

HMP2_13660_1

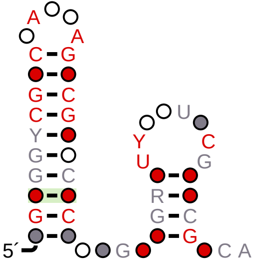

HMP2_13663_1

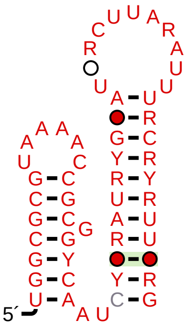

HMP2_13668_1

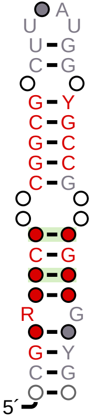

HMP2_13676_1

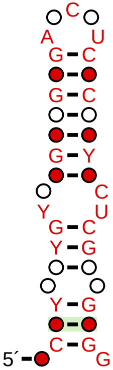

HMP2_13678_1

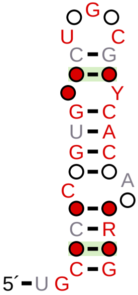

HMP2_13683_1

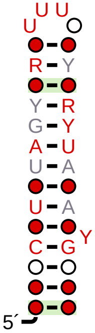

HMP2_13684_1

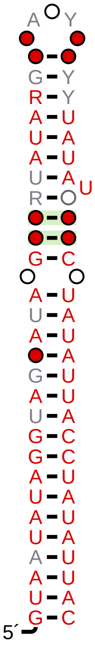

HMP2_13687_1

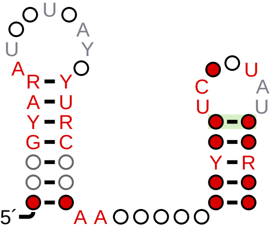

HMP2_13691_1

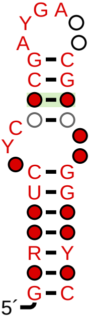

HMP2_13700_1

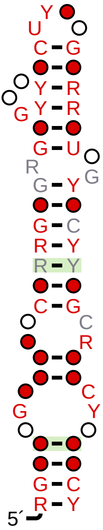

HMP2_13704_1

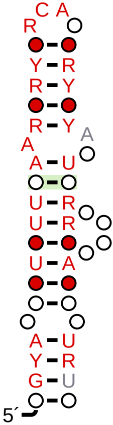

HMP2_13706_1

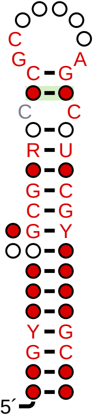

HMP2_13707_1

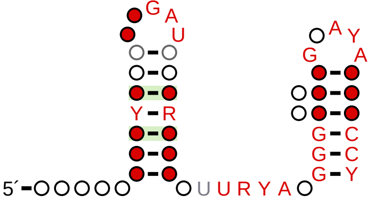

HMP2_13711_1

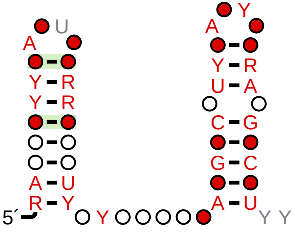

HMP2_13712_1

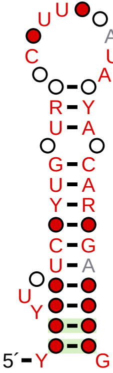

HMP2_13714_1

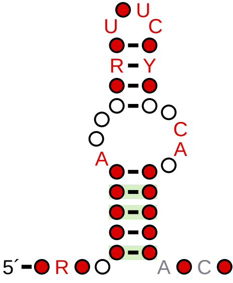

HMP2_1372_1

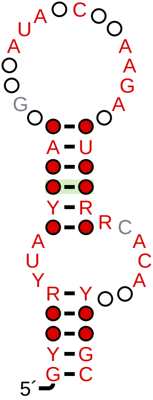

HMP2_13724_1

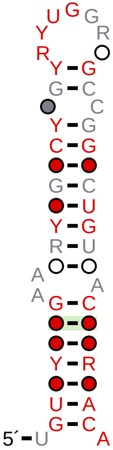

HMP2_13727_1

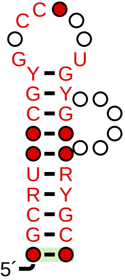

HMP2_13728_1

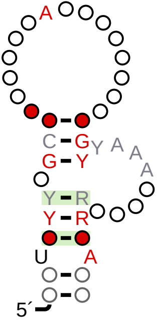

HMP2_13729_1

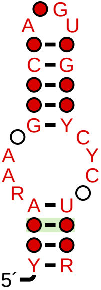

HMP2_13755_1

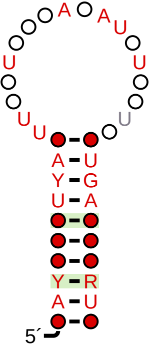

HMP2_13762_1

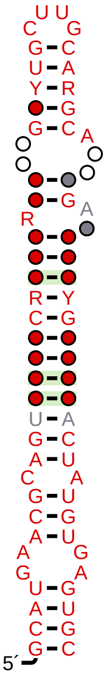

HMP2_13766_1

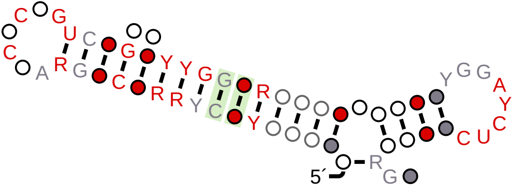

HMP2_13770_1

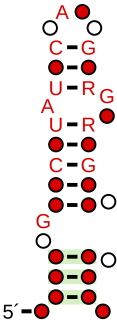

HMP2_13775_1

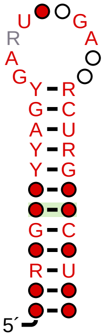

HMP2_1378_1

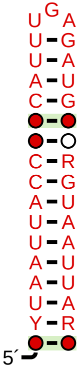

HMP2_13780_1

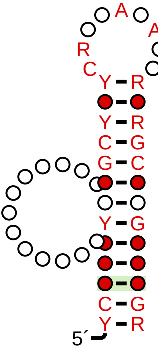

HMP2_1379_1

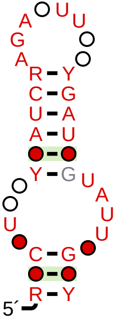

HMP2_13798_1

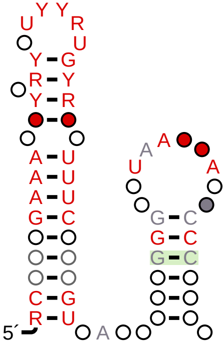

HMP2_138_1

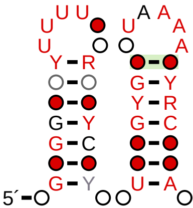

HMP2_13800_1

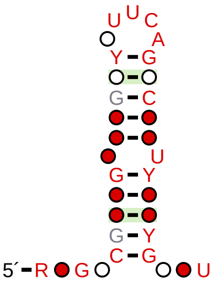

HMP2_1382_1

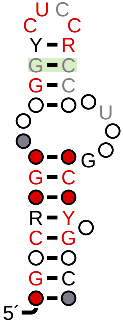

HMP2_13822_1

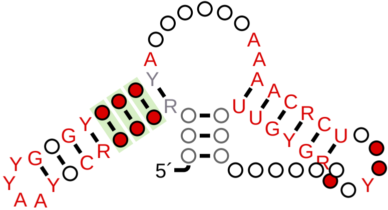

HMP2_13828_1

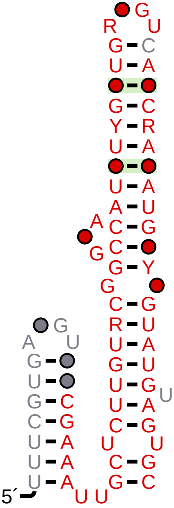

HMP2_13831_1

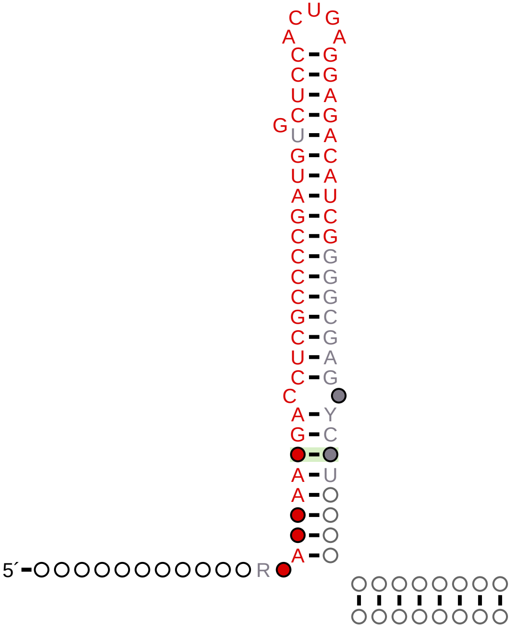

C U G
A A
C G
C G
U A
G C G
U A
G C
U A
A U
G C
C G
C G
C G
C G
G C
C G
U A
C G
C
A Y
G C
A U
A
A
5´
R

HMP2_13834_1

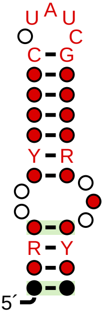

HMP2_13841_1

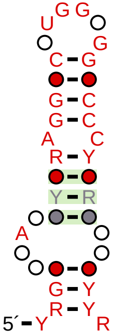

HMP2_13845_1

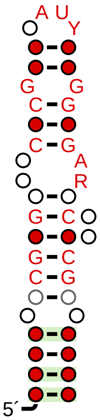

HMP2_13847_1

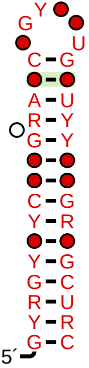

HMP2_13850_1

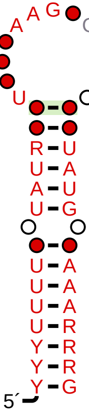

HMP2_13856_1

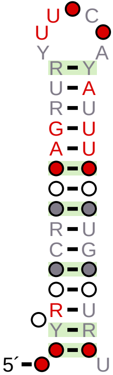

HMP2_13866_1

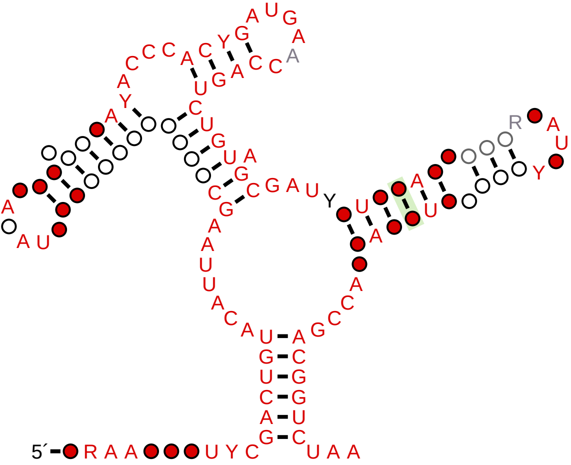

HMP2_13867_1

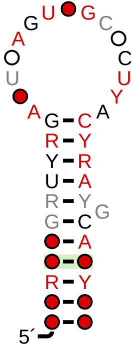

HMP2_13874_1

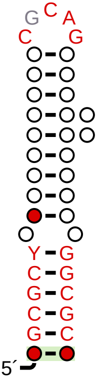

HMP2_13876_1

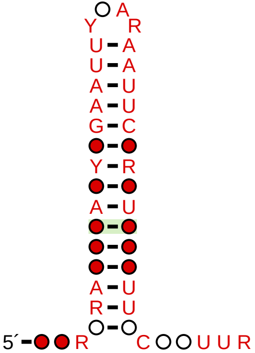

HMP2_13880_1

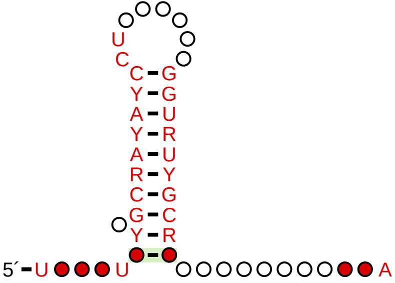

HMP2_13885_1

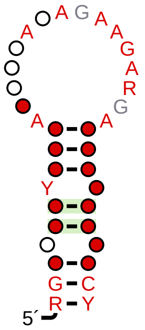

HMP2_13886_1

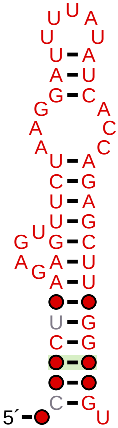

HMP2_13888_1

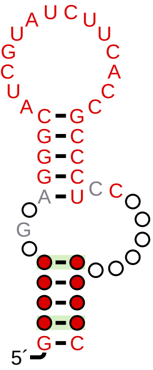

HMP2_1389_1

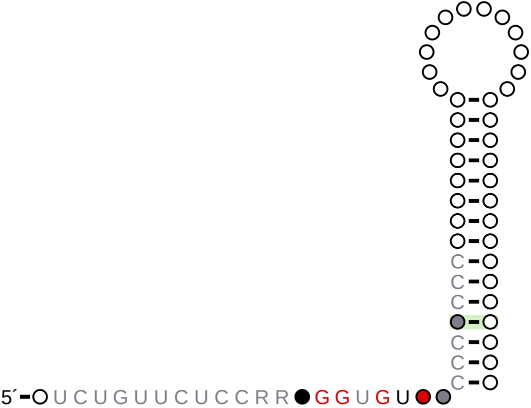

HMP2_13890_1

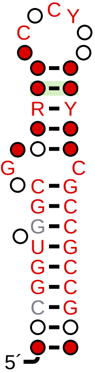

HMP2_13896_1

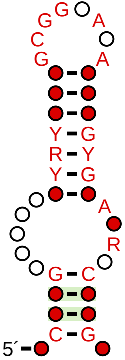

HMP2_13899_1

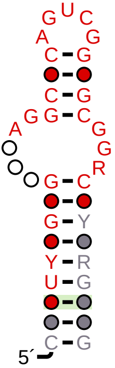

HMP2_139_1

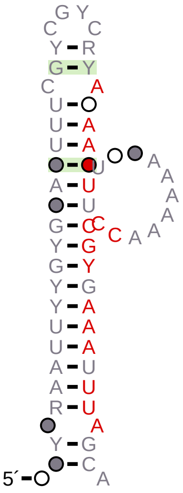

HMP2_13907_1

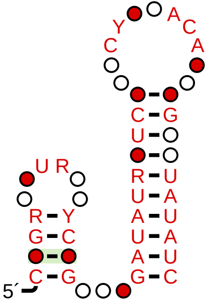

HMP2_13915_1

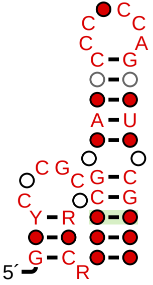

HMP2_13916_1

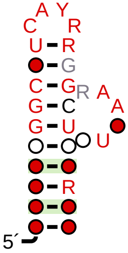

HMP2_13918_1

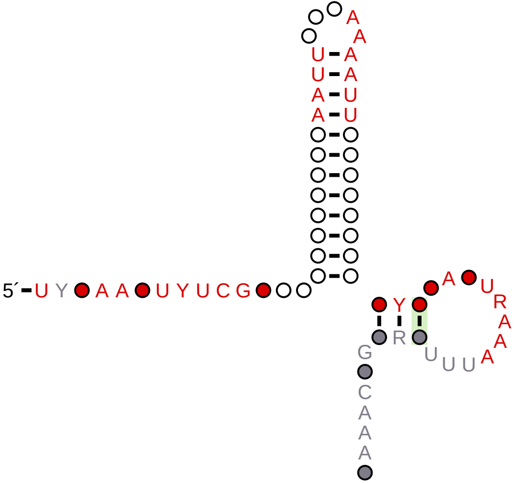

HMP2_1392_1

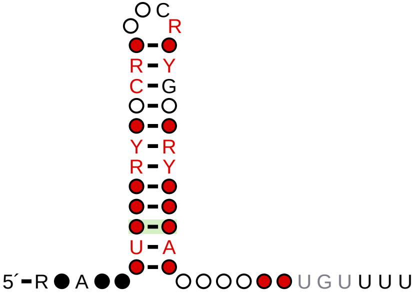

HMP2_13923_1

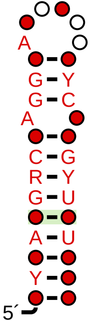

HMP2_13924_1

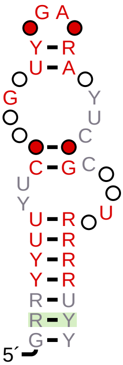

HMP2_13927_1

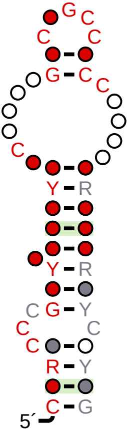

HMP2_13929_1

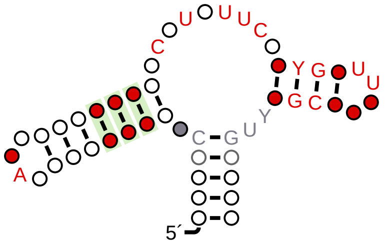

HMP2_1393_1

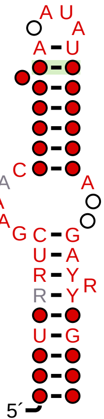

HMP2_13932_1

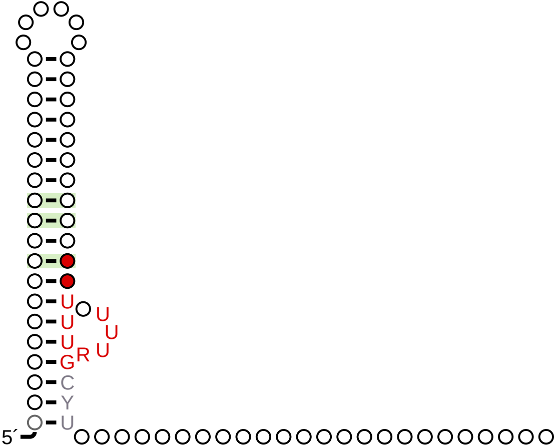

HMP2_13937_1

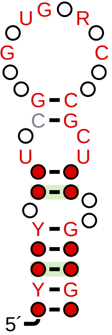

HMP2_13944_1

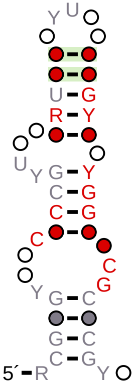

HMP2_1396_1

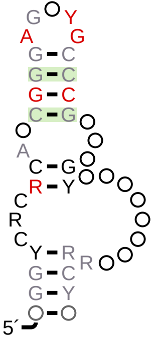

HMP2_13965_1

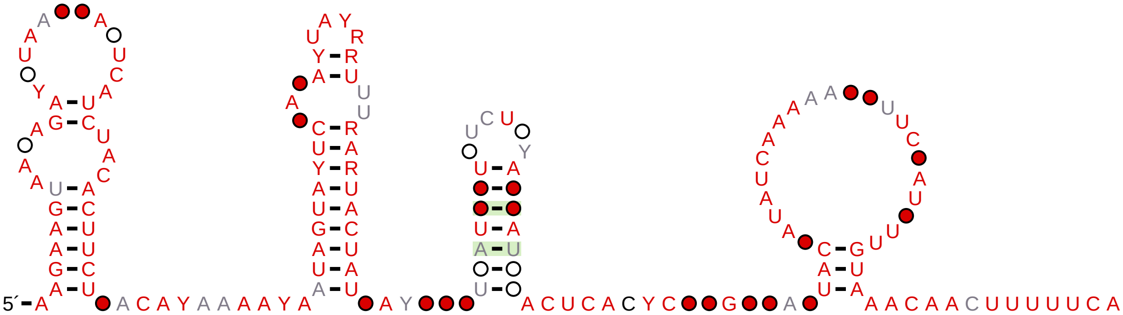

HMP2_13967_1

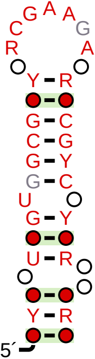

HMP2_13992_1

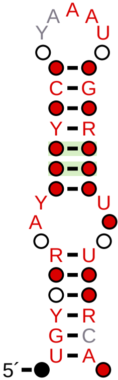

HMP2_1400_1

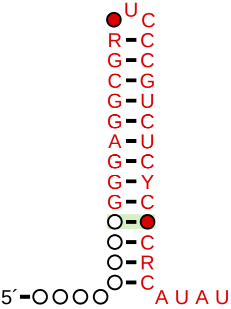

HMP2_14004_1

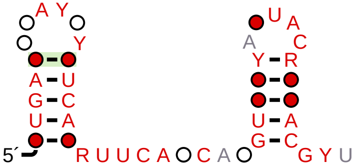

HMP2_14011_1

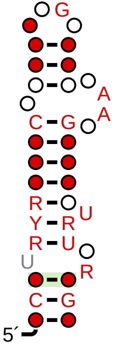

HMP2_14012_1

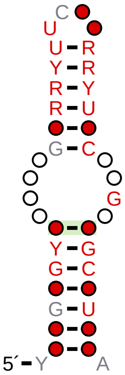

HMP2_14013_1

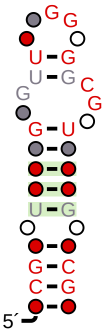

HMP2_14021_1

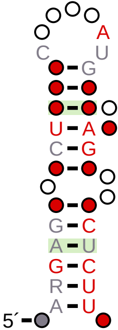

HMP2_14024_1

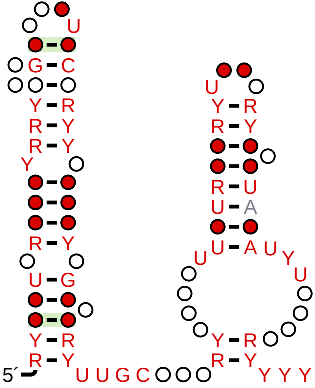

HMP2_1403_1

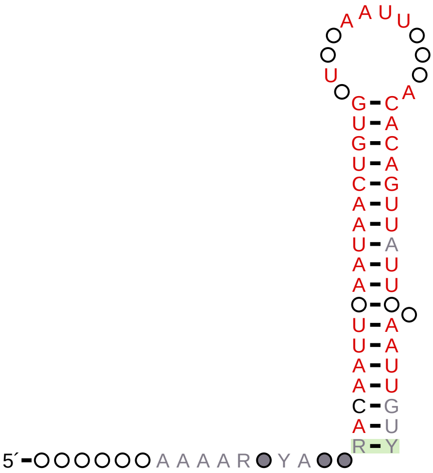

HMP2_14031_1

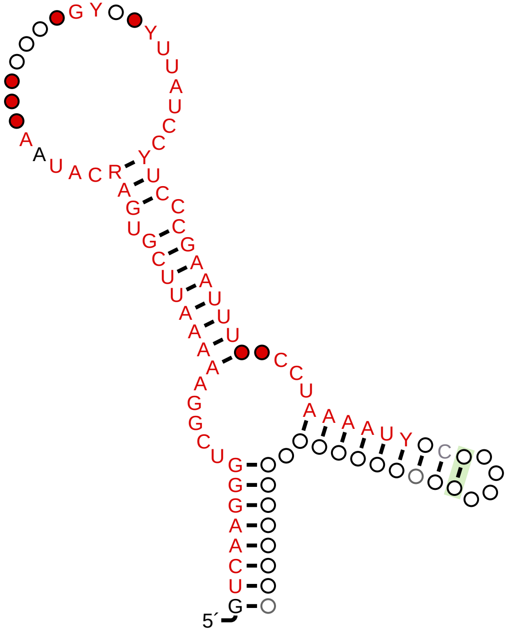

HMP2_14037_1

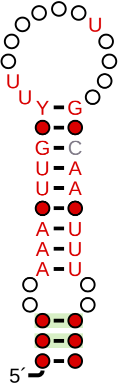

HMP2_14038_1

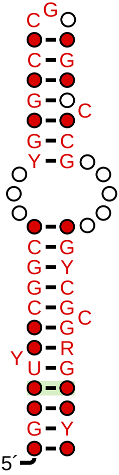

HMP2_14040_1

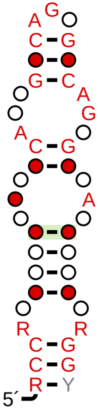

HMP2_14051_1

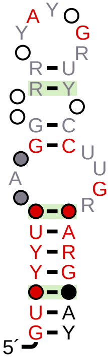

HMP2_14056_1

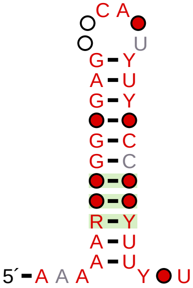

HMP2_14070_1

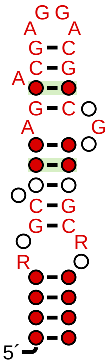

HMP2_14078_1

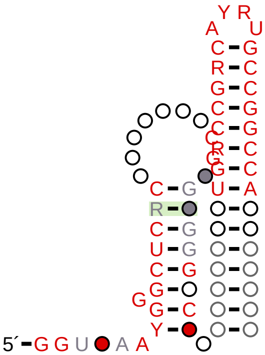

HMP2_14080_1

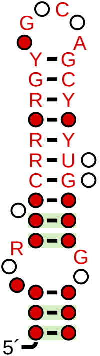

HMP2_1409_1

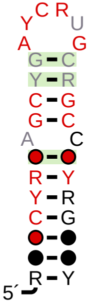

HMP2_14090_1

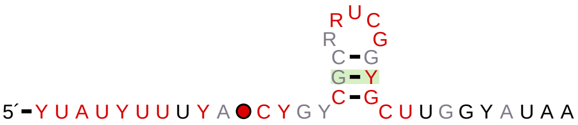

HMP2_14091_1

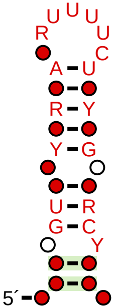

HMP2_14096_1

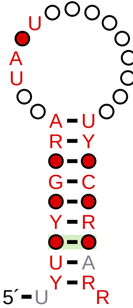

HMP2_14103_1

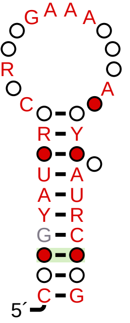

HMP2_14109_1

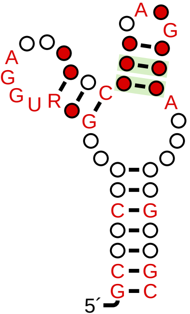

HMP2_14114_1

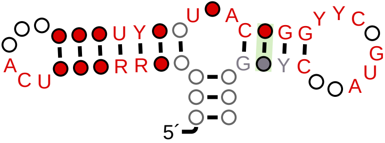

HMP2_14124_1

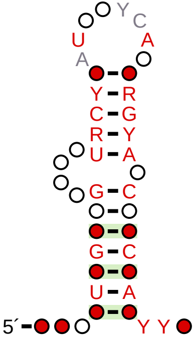

HMP2_14127_1

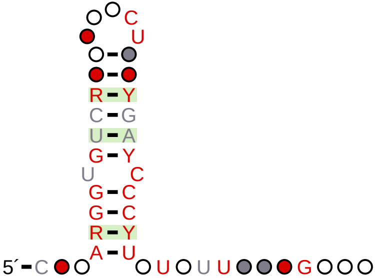

HMP2_14140_1

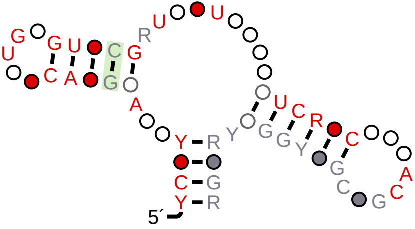

HMP2_14146_1

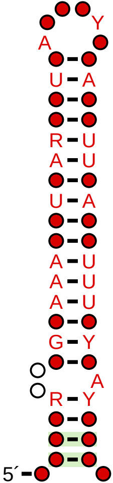

HMP2_14158_1

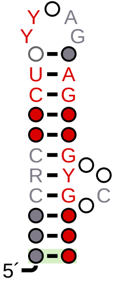

HMP2_14161_1

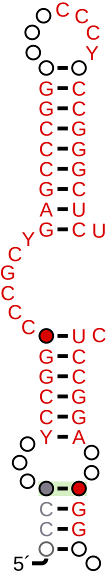

HMP2_14165_1

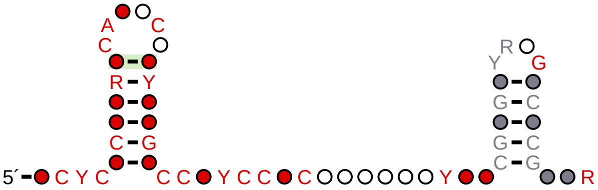

HMP2_14170_1

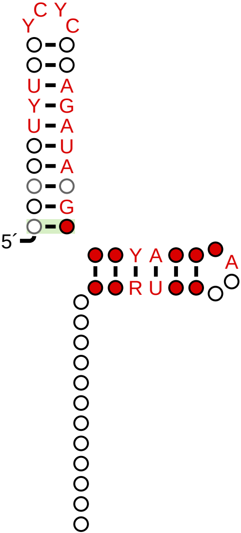

HMP2_14171_1

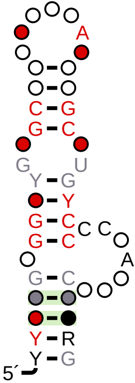

HMP2_14176_1

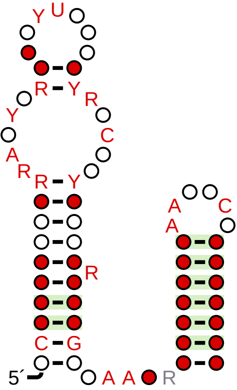

HMP2_14177_1

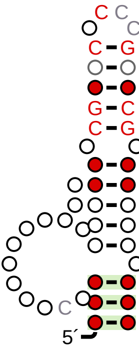

HMP2_1418_1

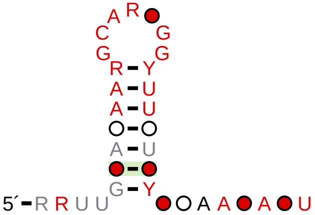

HMP2_14181_1

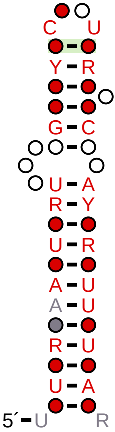

HMP2_14183_1

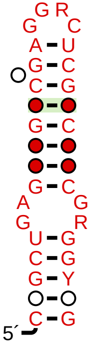

HMP2_14198_1

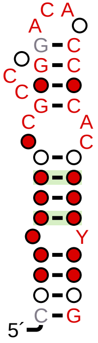

HMP2_14201_1

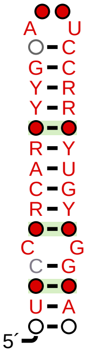

HMP2_14202_1

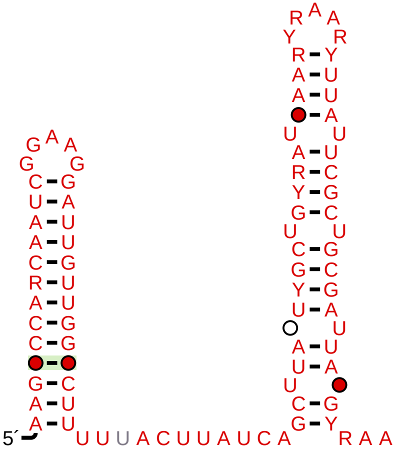

HMP2_14204_1

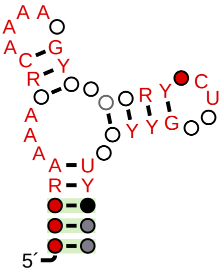

HMP2_14205_1

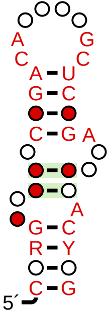

HMP2_14208_1

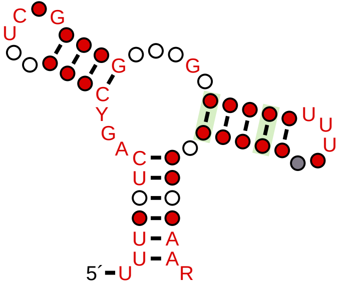

HMP2_14215_1

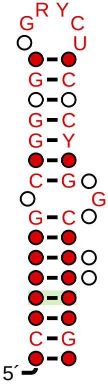

HMP2_14217_1

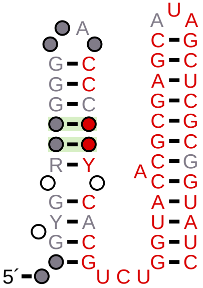

HMP2_14218_1

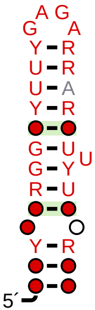

HMP2_1422_1

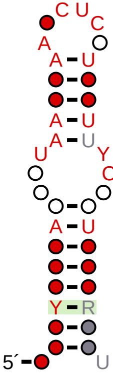

HMP2_14225_1

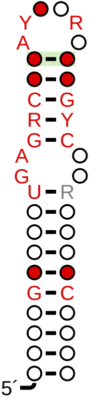

HMP2_1423_1

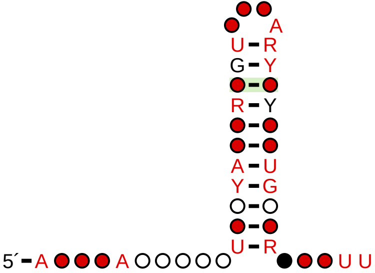

HMP2_14232_1

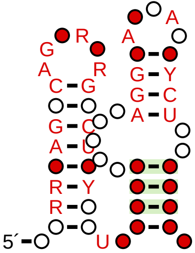

HMP2_1424_1

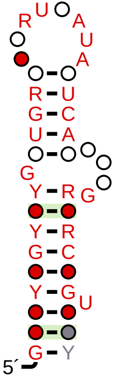

HMP2_14249_1

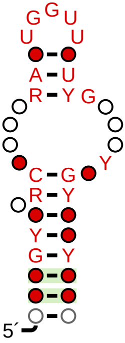

HMP2_14256_1

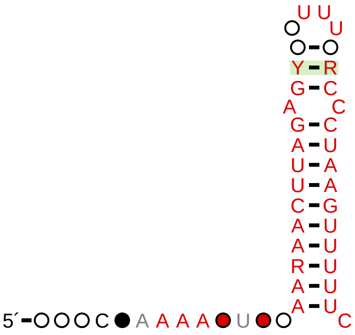

HMP2_14259_1

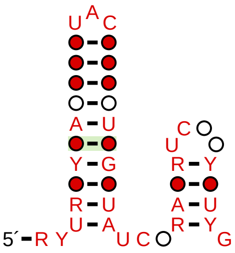

HMP2_1426_1

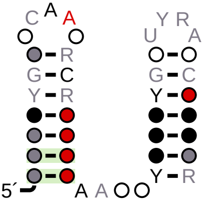

HMP2_14263_1

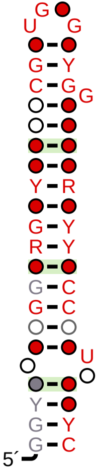

HMP2_14266_1

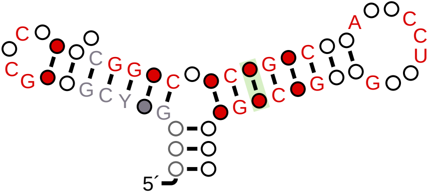

HMP2_14273_1

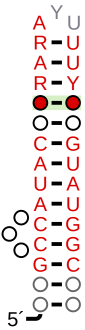

HMP2_14274_1

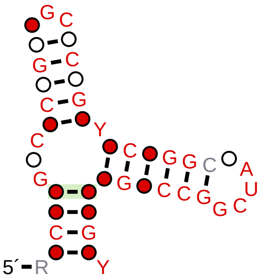

HMP2_14278_1

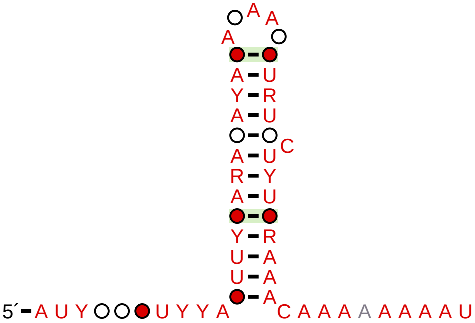

HMP2_14281_1

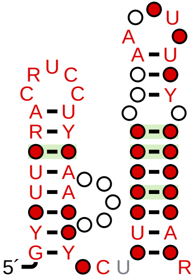

HMP2_14283_1

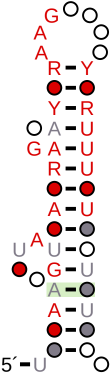

HMP2_1429_1

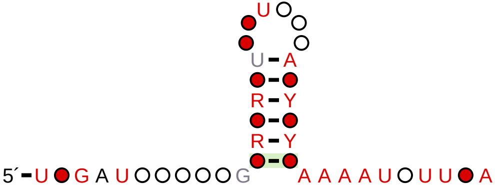

HMP2_14290_1

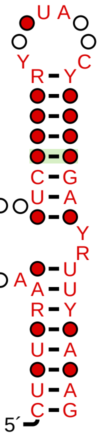

HMP2_14295_1

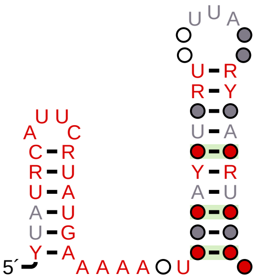

HMP2_14303_1

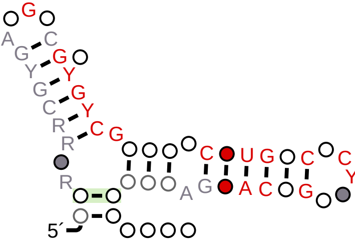

HMP2_14304_1

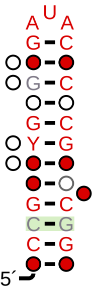

HMP2_14306_1

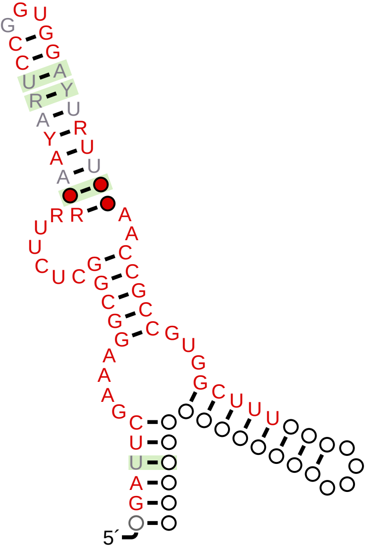

HMP2_14311_1

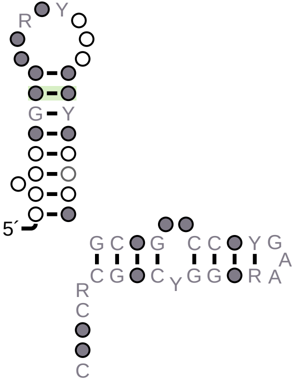

HMP2_14317_1

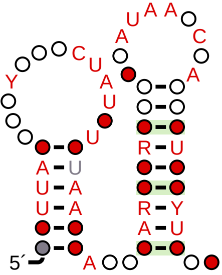

HMP2_14322_1

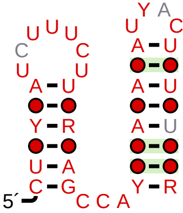

HMP2_14325_1

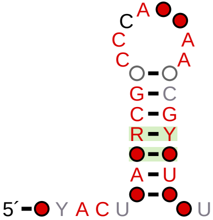

HMP2_14333_1

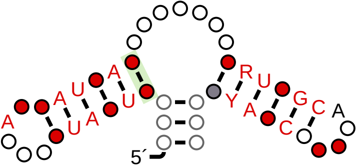

HMP2_14339_1

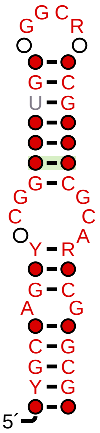

HMP2_1434_1

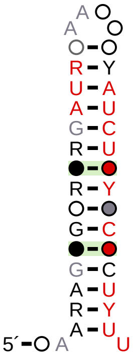

HMP2_1435_1

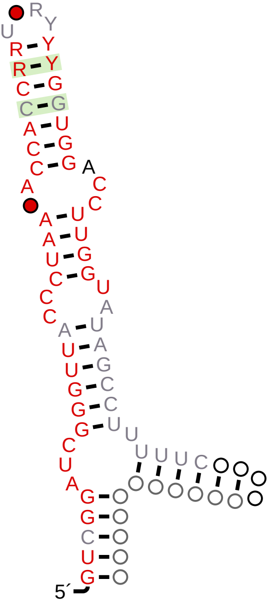

HMP2_14350_1

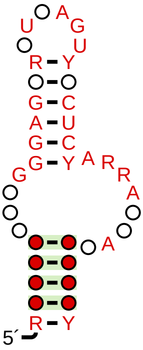

HMP2_14352_1

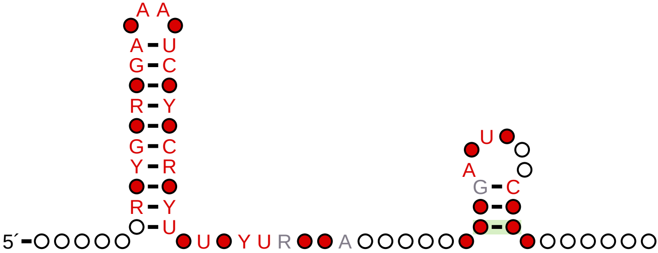

HMP2_14355_1

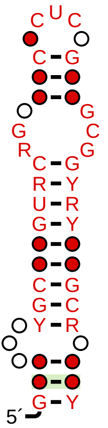

HMP2_14389_1

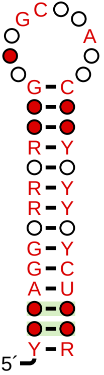

HMP2_14396_1

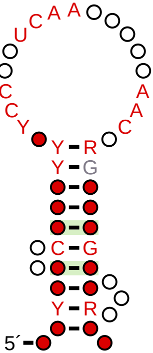

HMP2_14398_1

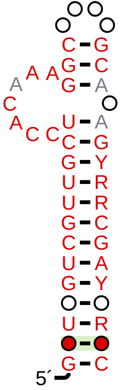

HMP2_1440_1

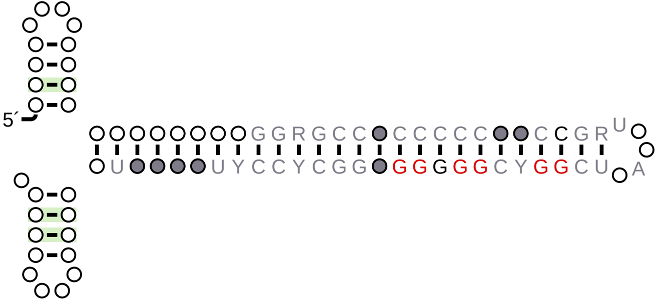

HMP2_14408_1

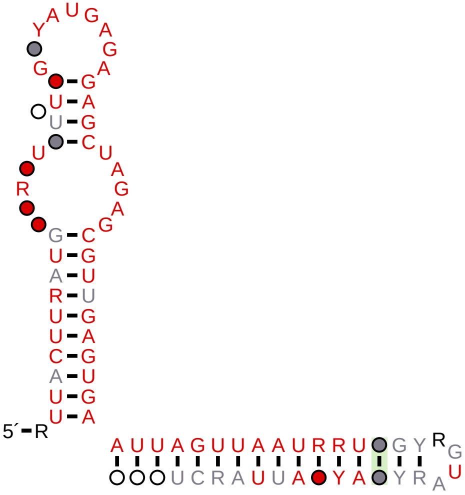

HMP2_1441_1

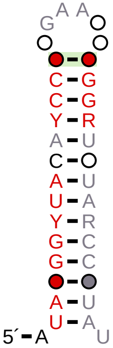

HMP2_14416_1

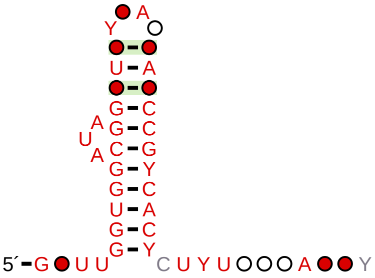

HMP2_1442_1

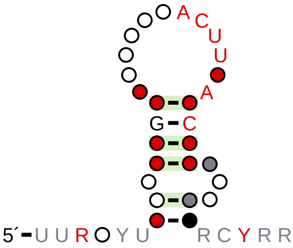

HMP2_14428_1

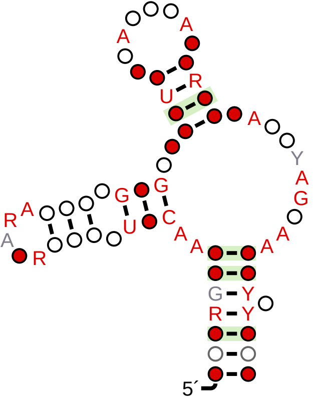

HMP2_14439_1

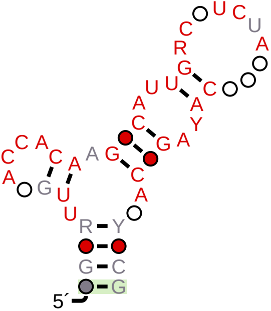

HMP2_14453_1

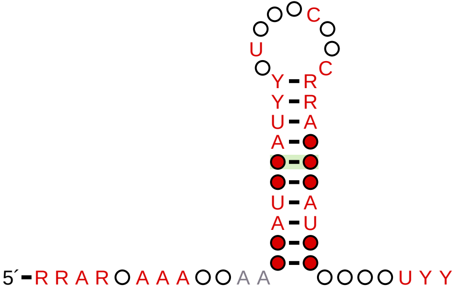

HMP2_14460_1

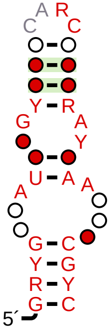

HMP2_1447_1

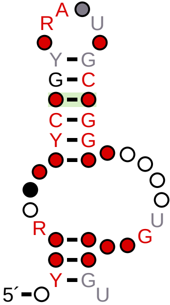

HMP2_14470_1

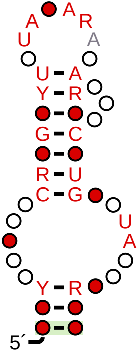

HMP2_14479_1

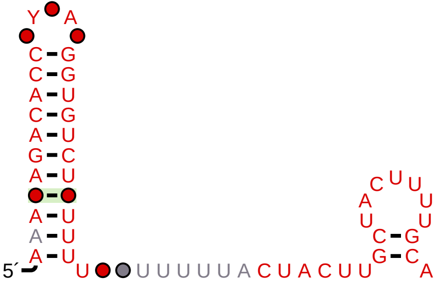

HMP2_14484_1

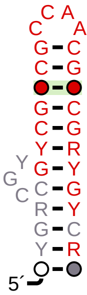

HMP2_14488_1

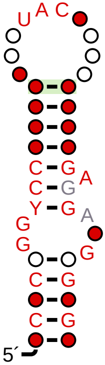

HMP2_14496_1

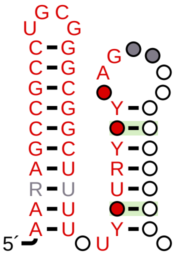

HMP2_14497_1

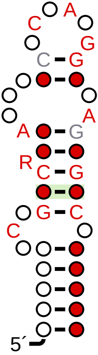

HMP2_14502_1

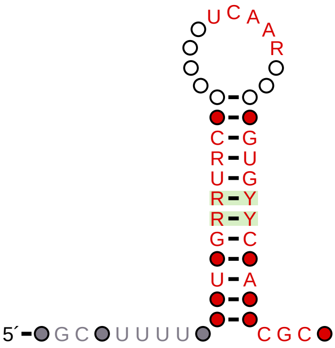

HMP2_14503_1

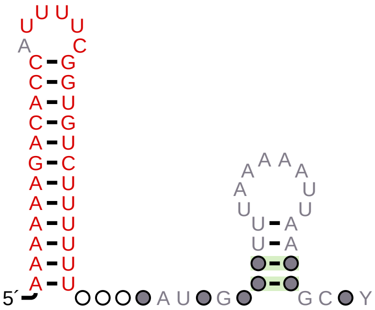

5´
U U U U A C
C G C G A U C G A U G C A U A U A U A U A U A U
A U G
A A A A A U U U U U U A U A
G C Y

HMP2_14506_1

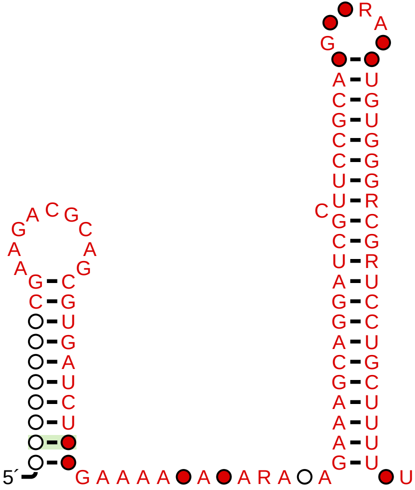

HMP2_14512_1

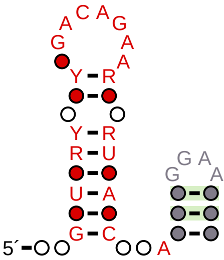

HMP2_14513_1

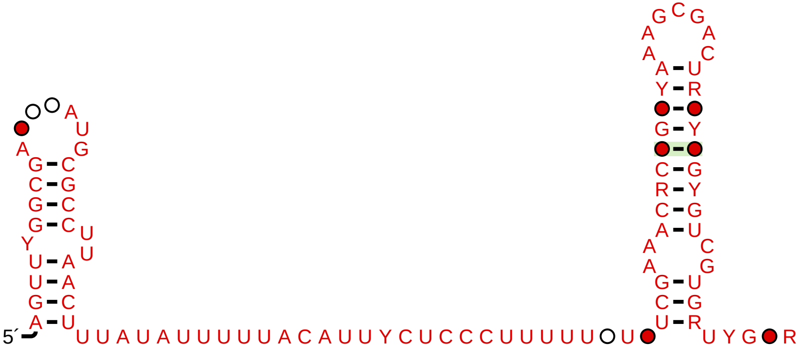

HMP2_14522_1

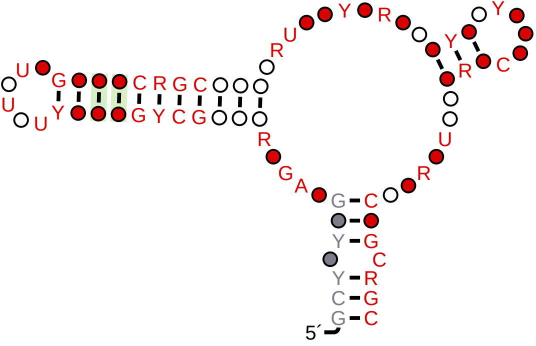

HMP2_14523_1

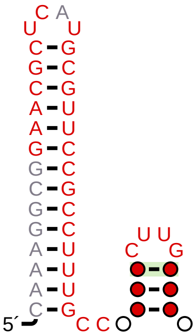

HMP2_14530_1

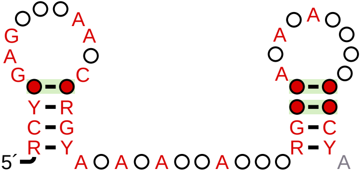

HMP2_14534_1

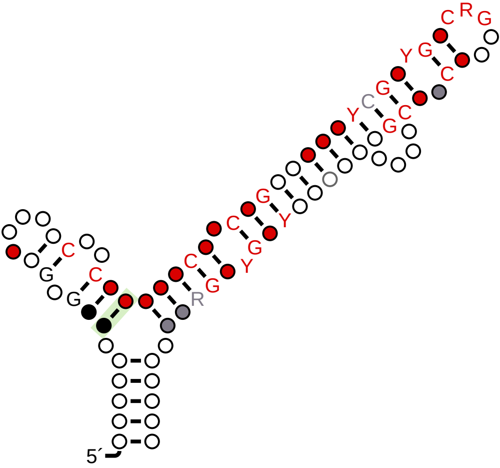

HMP2_14535_1

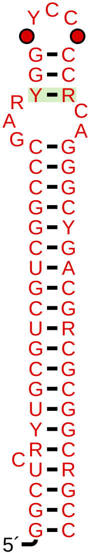

HMP2_14539_1

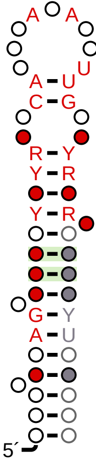

HMP2_14545_1

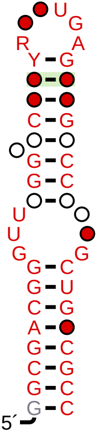

HMP2_14546_1

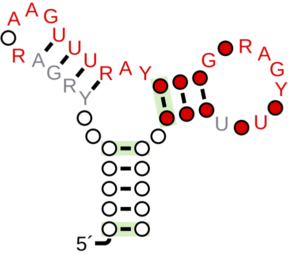

HMP2_14549_1

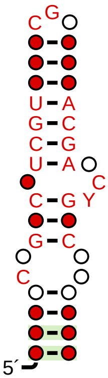

HMP2_14554_1

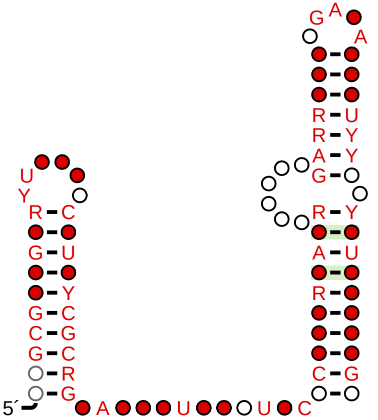

HMP2_1456_1

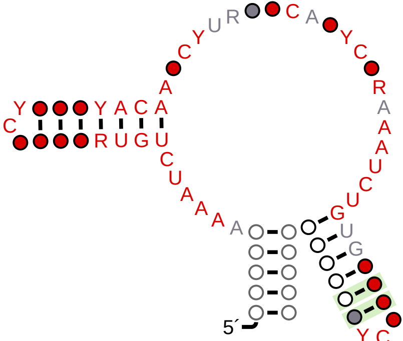

HMP2_14568_1

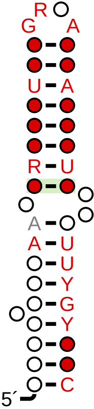

HMP2_1457_1

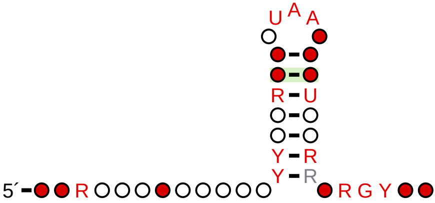

HMP2_14571_1

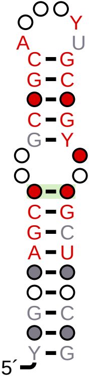

HMP2_14579_1

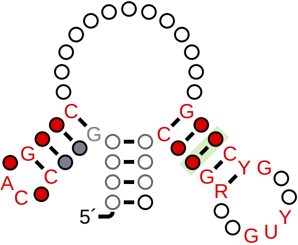

HMP2_14581_1

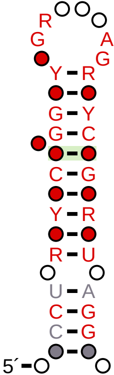

HMP2_14595_1

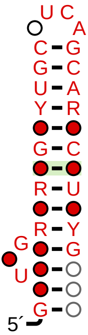

HMP2_14599_1

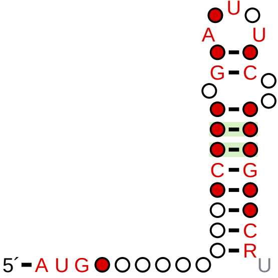

HMP2_14601_1

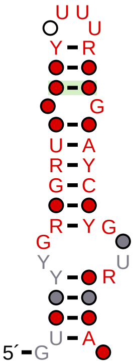

HMP2_14603_1

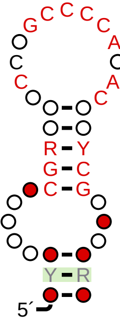

HMP2_14606_1

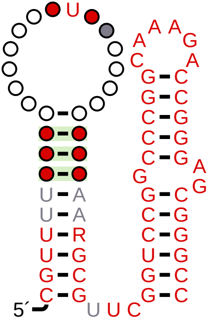

HMP2_1461_1

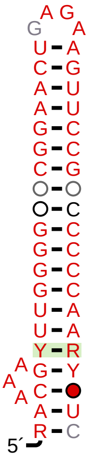

HMP2_14619_1

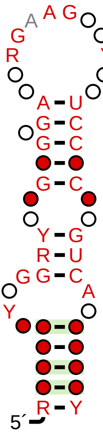

HMP2_1462_1

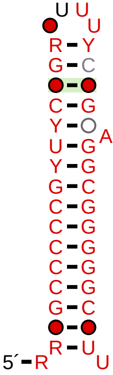

HMP2_14624_1

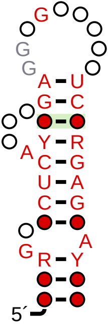

HMP2_14625_1

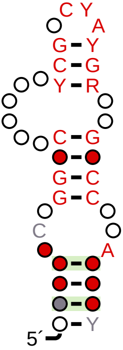

HMP2_14631_1

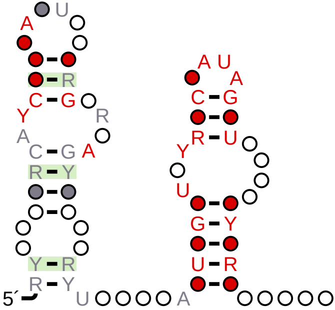

HMP2_14636_1

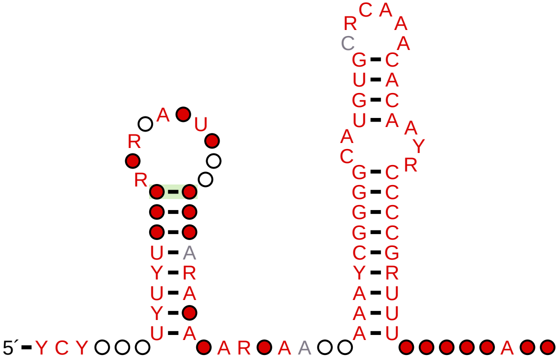

HMP2_14642_1

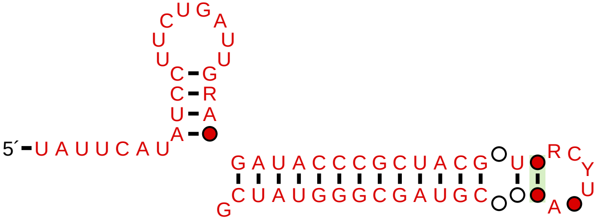

5´
UAUUCAU
ACCUUCUGAUUUGRA
GAUACCCGCUACGU
RCYUA
CUAUGGGCGAUGC
G

HMP2_14643_1

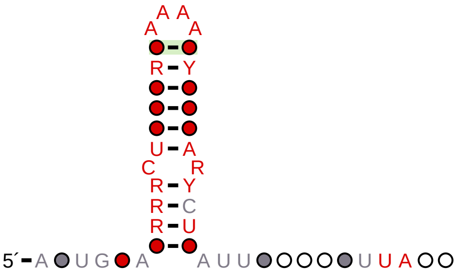

HMP2_14656_1

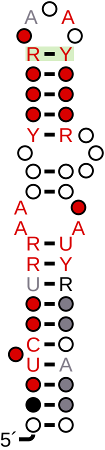

HMP2_14660_1

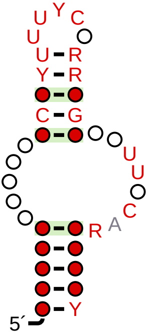

HMP2_14664_1

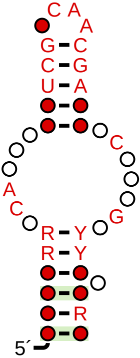

HMP2_14665_1

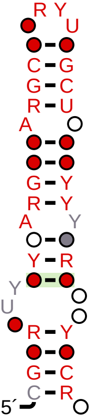

HMP2_14668_1

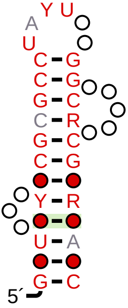

HMP2_14670_1

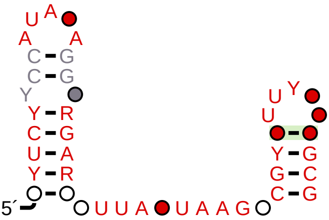

HMP2_14681_1

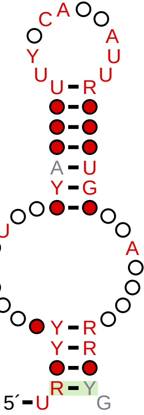

HMP2_14689_1

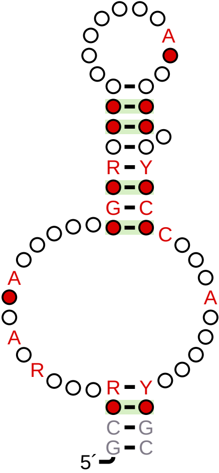

HMP2_14694_1

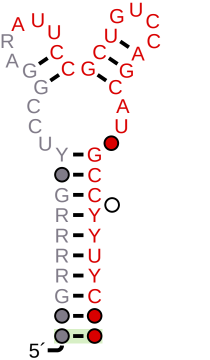

HMP2_14697_1

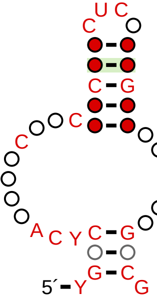

HMP2_147_1

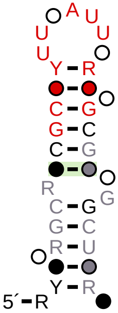

HMP2_1470_1

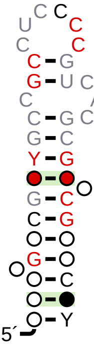

HMP2_14705_1

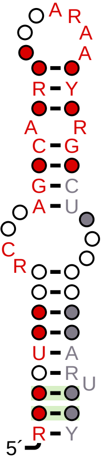

HMP2_14709_1

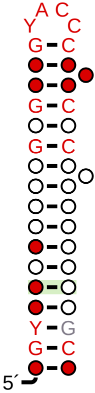

HMP2_14722_1

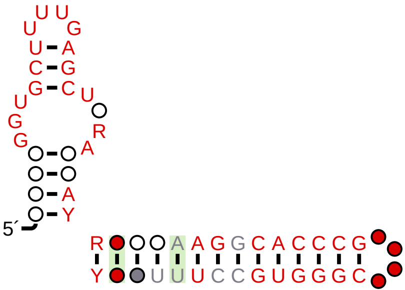

HMP2_14724_1

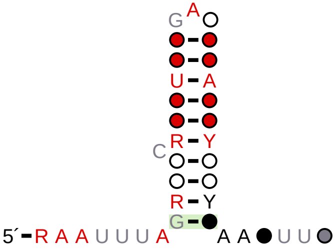

HMP2_14728_1

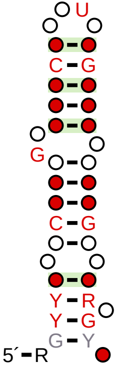

HMP2_14731_1

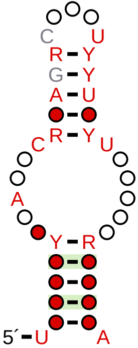

HMP2_14736_1

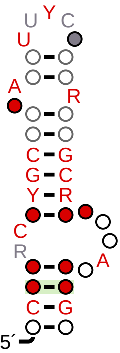

HMP2_14744_1

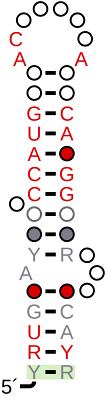

HMP2_14747_1

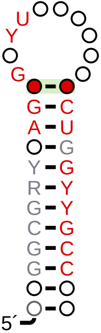

HMP2_14763_1

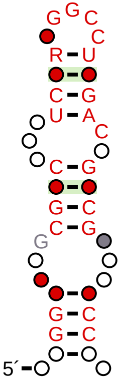

HMP2_1477_1

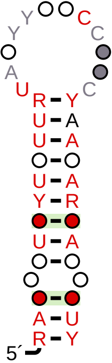

HMP2_14780_1

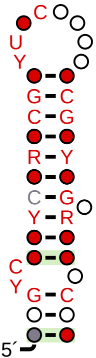

HMP2_1479_1

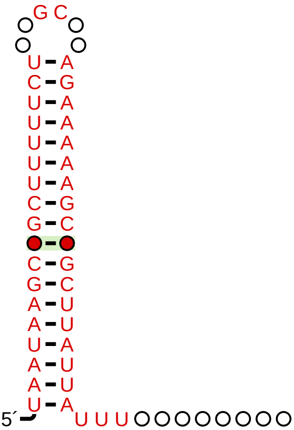

HMP2_14791_1

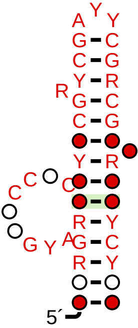

HMP2_14793_1

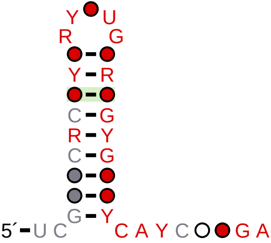

HMP2_148_1

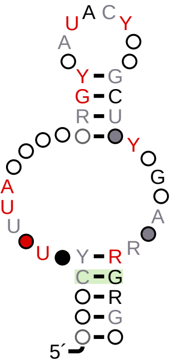

HMP2_14803_1

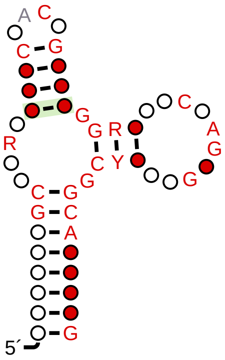

HMP2_14806_1

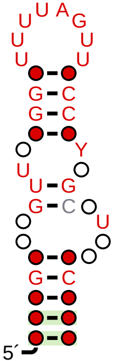

HMP2_1481_1

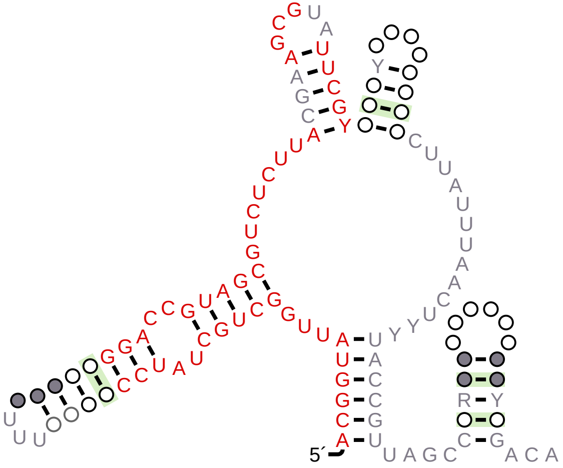

HMP2_14812_1

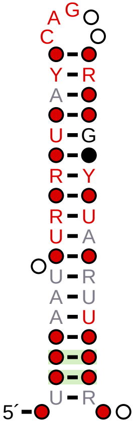

HMP2_14817_1

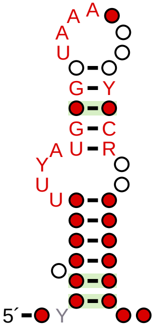

HMP2_1483_1

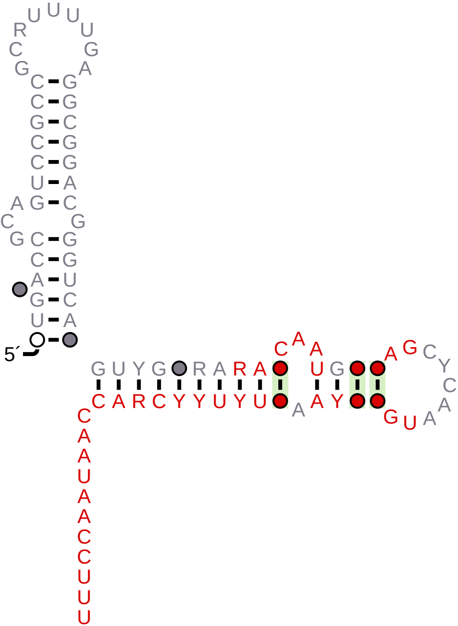

HMP2_14831_1

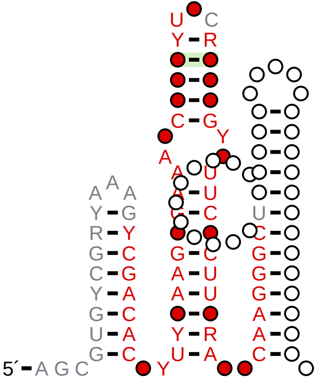

HMP2_14834_1

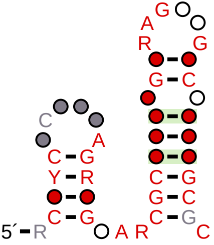

HMP2_14835_1

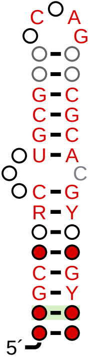

HMP2_14840_1

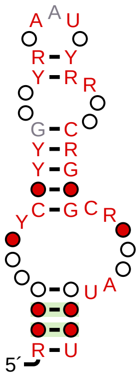

HMP2_14841_1

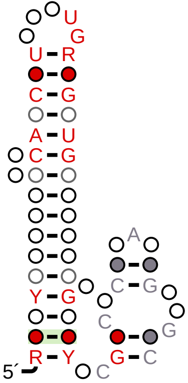

HMP2_14852_1

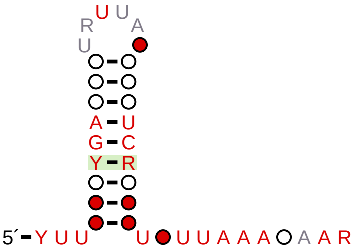

HMP2_14853_1

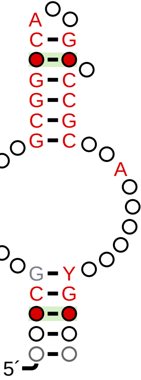

HMP2_14855_1

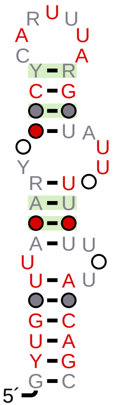

HMP2_14858_1

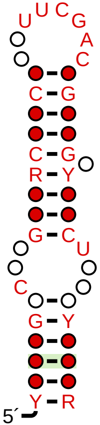

HMP2_14859_1

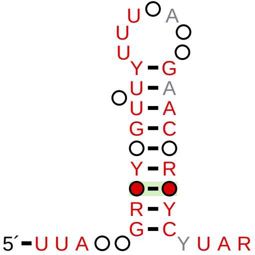

HMP2_14872_1

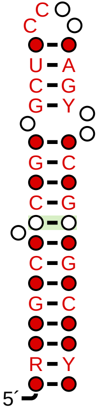

HMP2_14873_1

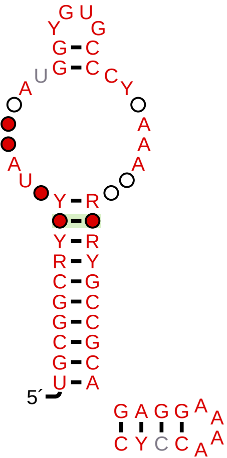

HMP2_14880_1

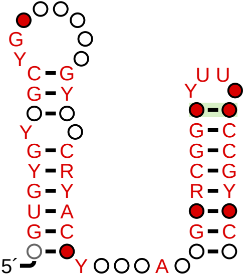

HMP2_14883_1

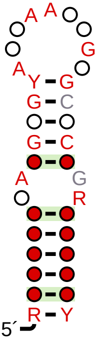

HMP2_14910_1

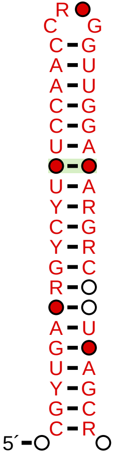

HMP2_14921_1

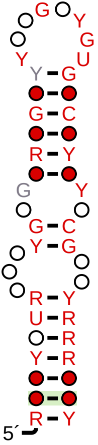

HMP2_14924_1

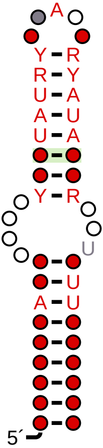

HMP2_14928_1

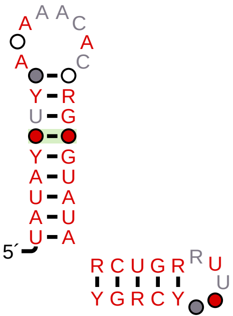

HMP2_14933_1

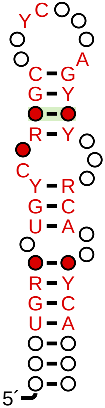

HMP2_14935_1

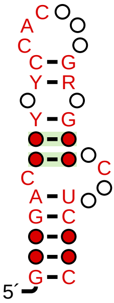

HMP2_14936_1

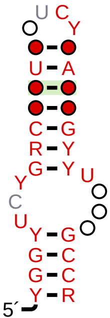

HMP2_14939_1

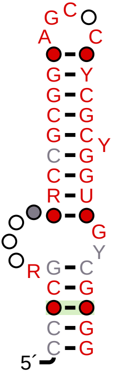

HMP2_14940_1

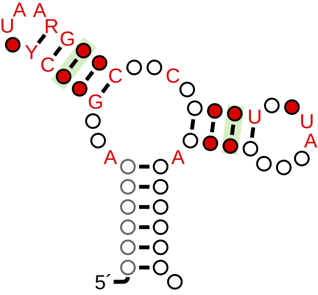

HMP2_14943_1

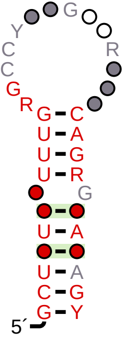

HMP2_1495_1

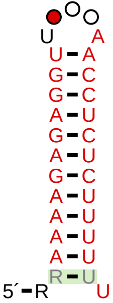

HMP2_14953_1

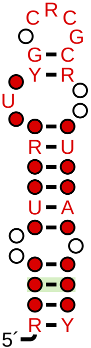

HMP2_14969_1

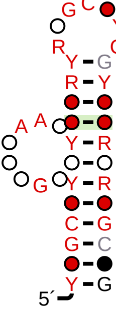

HMP2_14971_1

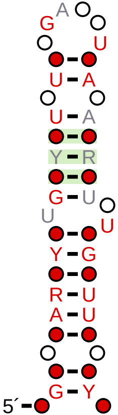

HMP2_14975_1

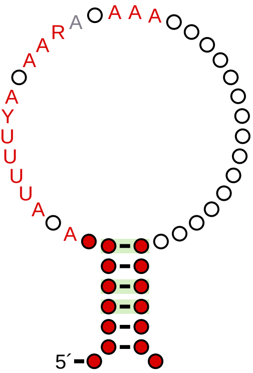

HMP2_14977_1

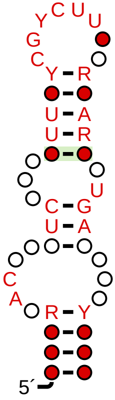

HMP2_14982_1

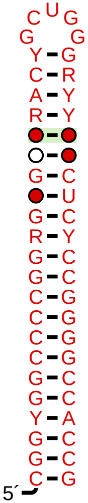

HMP2_14985_1

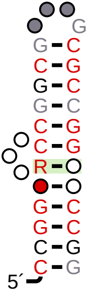

HMP2_14986_1

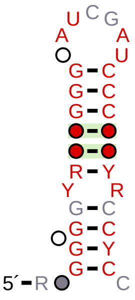

HMP2_14988_1

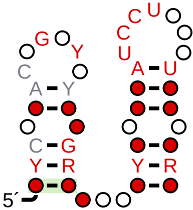

HMP2_1499_1

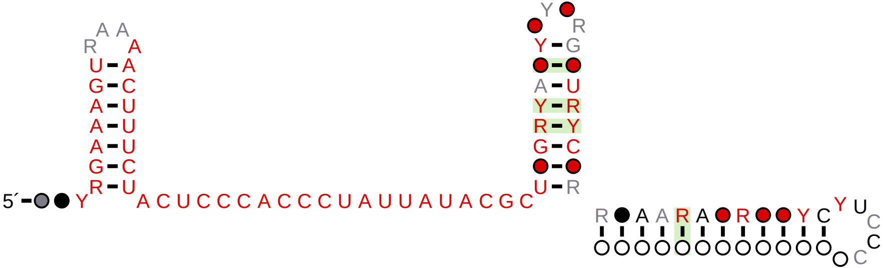

HMP2_14991_1

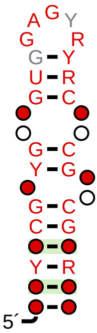

HMP2_14992_1

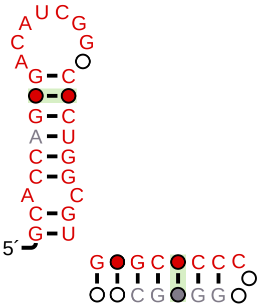

HMP2_14994_1

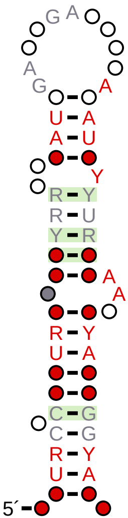

HMP2_150_1

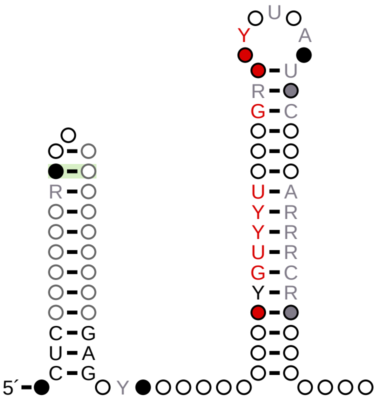

HMP2_15000_1

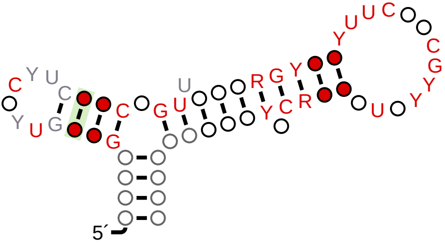

HMP2_15006_1

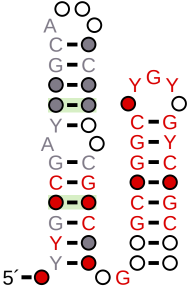

HMP2_15008_1

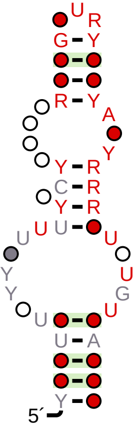

HMP2_15016_1

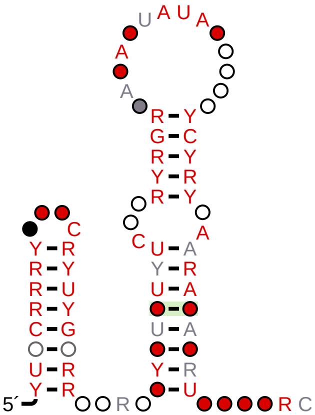

HMP2_15019_1

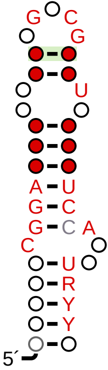

HMP2_1502_1

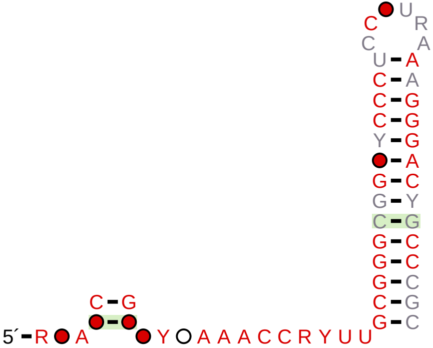

HMP2_15021_1

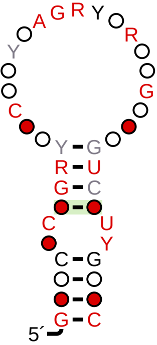

HMP2_15027_1

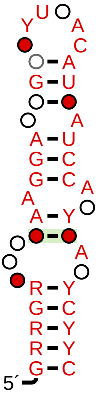

HMP2_15029_1

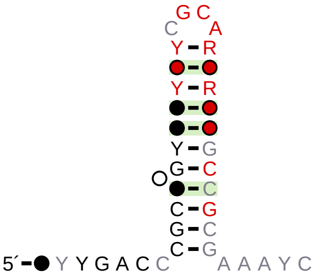

HMP2_15039_1

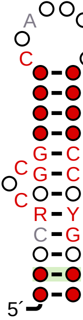

HMP2_15043_1

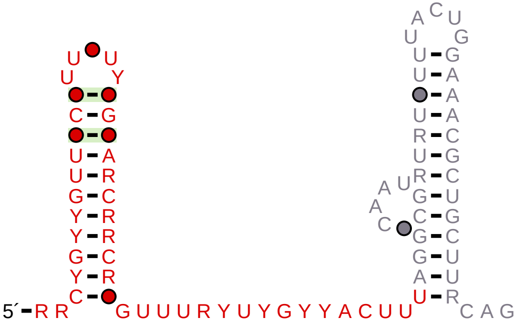

HMP2_15044_1

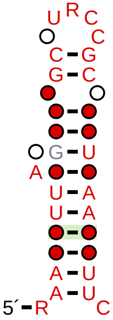

HMP2_15047_1

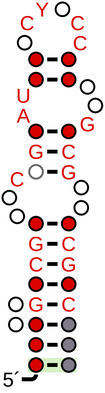

HMP2_15048_1

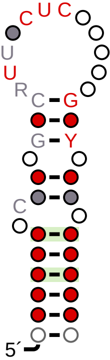

HMP2_15053_1

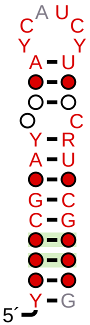

HMP2_15060_1

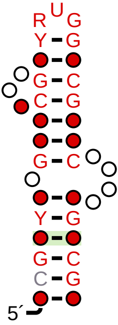

HMP2_15061_1

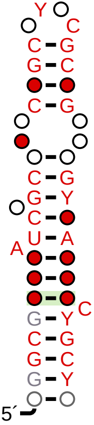

HMP2_15062_1

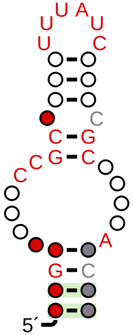

HMP2_15064_1

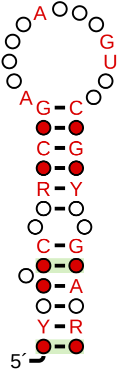

HMP2_15069_1

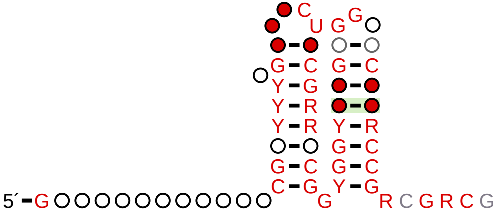

HMP2_15070_1

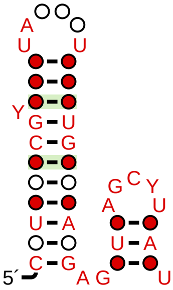

HMP2_15074_1

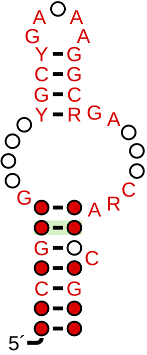

HMP2_15082_1

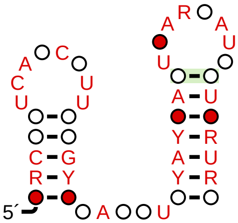

HMP2_15083_1

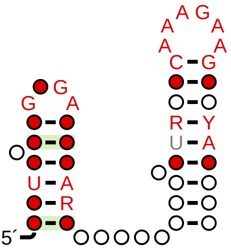

HMP2_15087_1

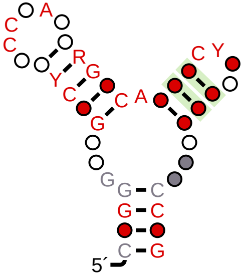

HMP2_1509_1

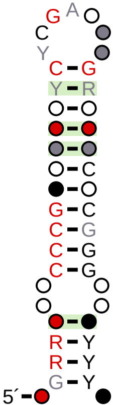

HMP2_15090_1

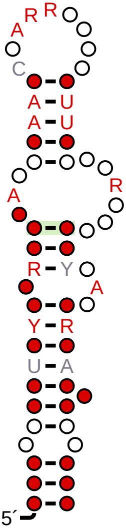

HMP2_15095_1

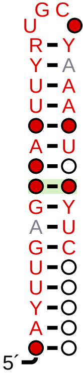

HMP2_1510_1

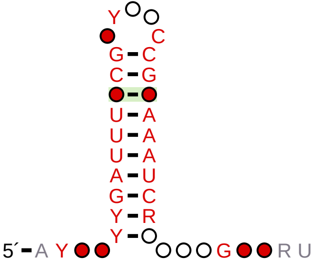

HMP2_15101_1

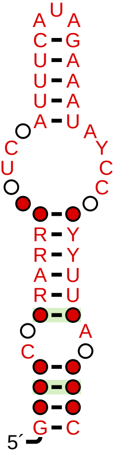

HMP2_15103_1

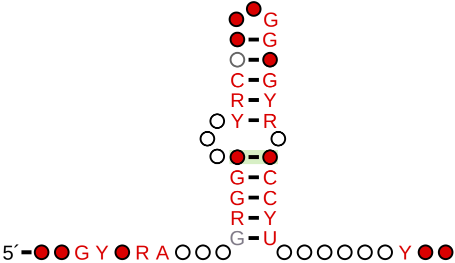

HMP2_15112_1

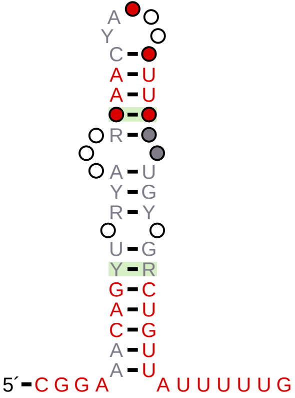

HMP2_15125_1

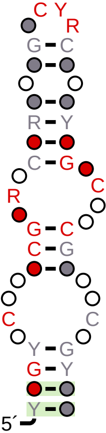

HMP2_15139_1

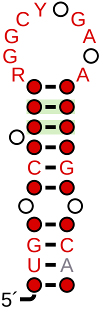

HMP2_15144_1

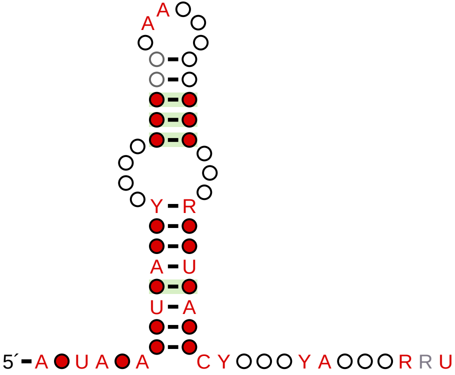

HMP2_15146_1

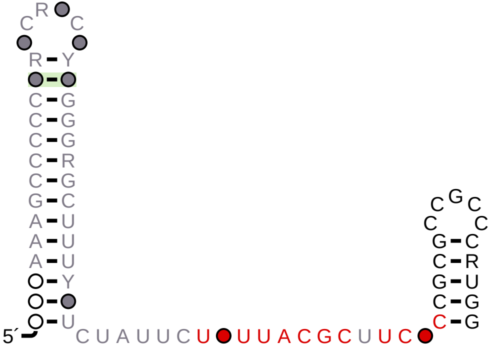
5´
C U A U U C U U U A C G C U U C
CRC C
R-Y
C-G
C-G
C-G
C-R
C-G
G-C
A-U
A-U
A-U
Y
U
CGC
C C
G-C
C-R
G-U
C-G
C-G

HMP2_1515_1

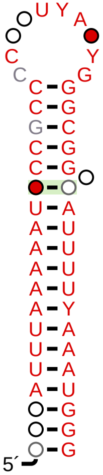

HMP2_15164_1

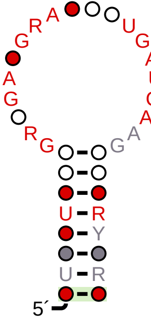

HMP2_15165_1

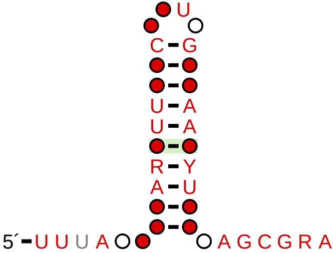

HMP2_15170_1

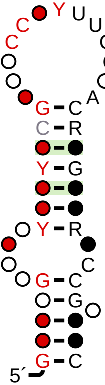

HMP2_15172_1

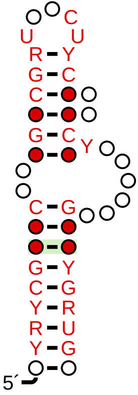

HMP2_15178_1

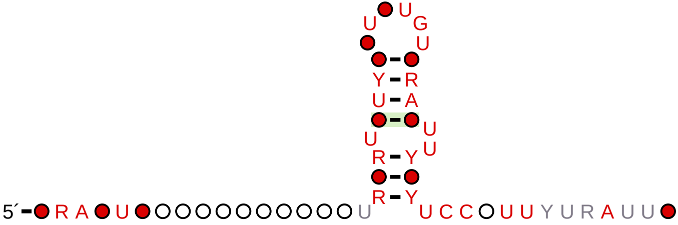

HMP2_15179_1

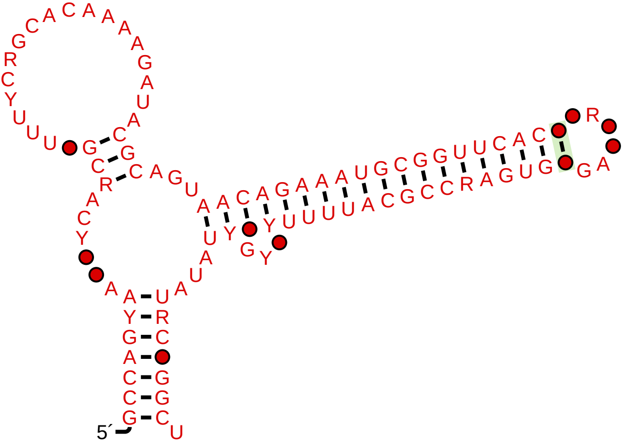

HMP2_1518_1

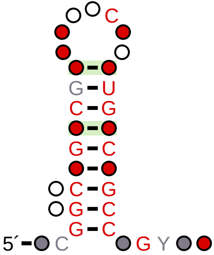

HMP2_15184_1

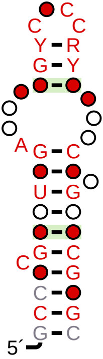

HMP2_15187_1

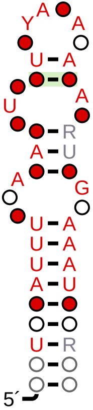

HMP2_1519_1

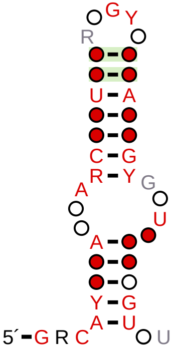

HMP2_15190_1

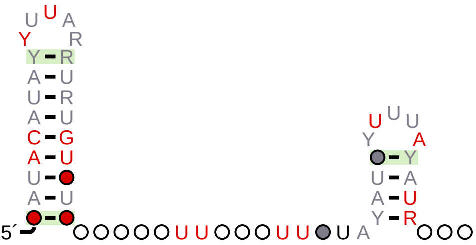

HMP2_15191_1

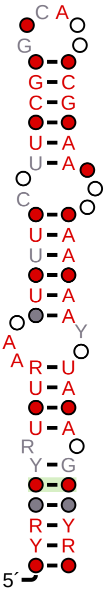

HMP2_15206_1

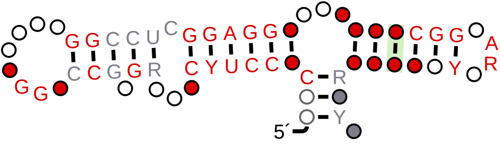

HMP2_15219_1

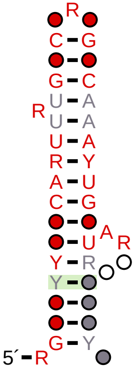

HMP2_15221_1

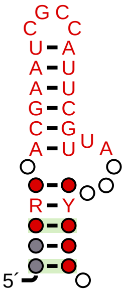

HMP2_15226_1

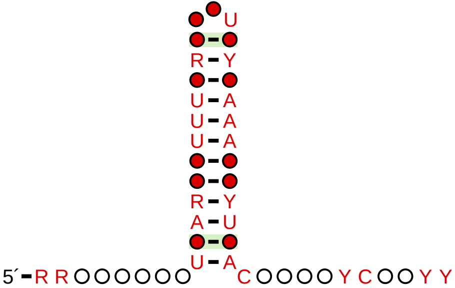

HMP2_15229_1

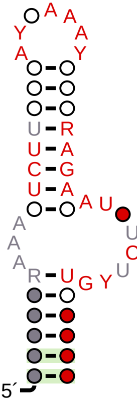

HMP2_1524_1

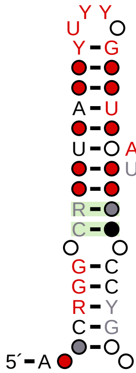

HMP2_15241_1

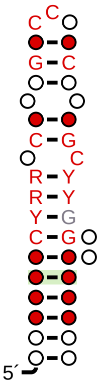

HMP2_15242_1

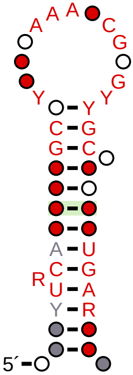

HMP2_15243_1

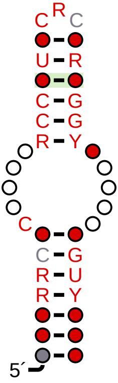

HMP2_15246_1

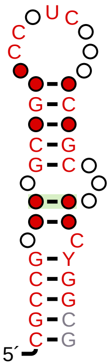

HMP2_1525_1

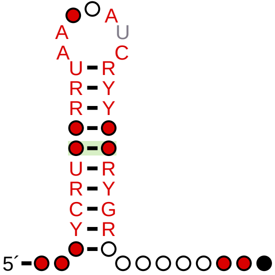

HMP2_15253_1

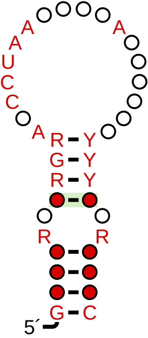

HMP2_15272_1

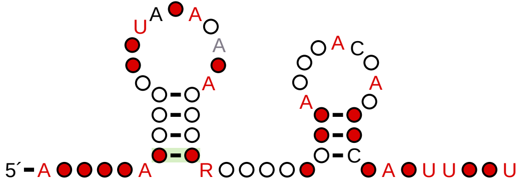

HMP2_15278_1

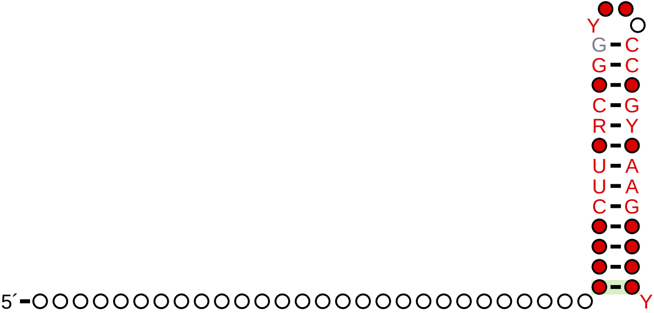

HMP2_15281_1

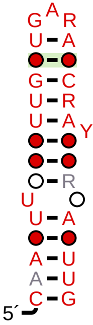

HMP2_15290_1

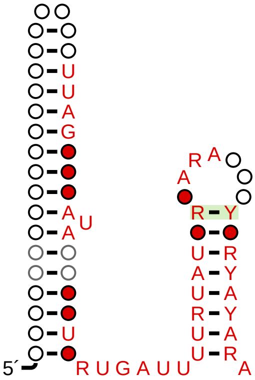

HMP2_15294_1

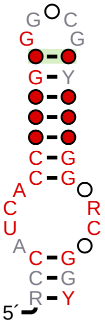

HMP2_15296_1

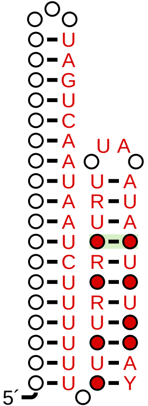

HMP2_15299_1

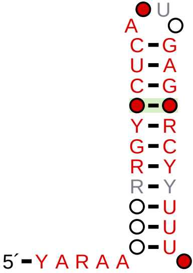

HMP2_1530_1

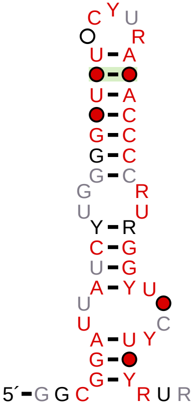

HMP2_15301_1

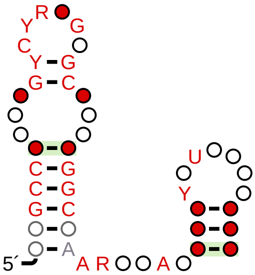

HMP2_15302_1

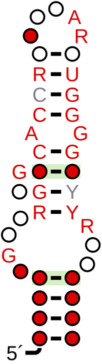

HMP2_15303_1

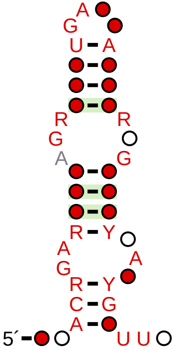

HMP2_15311_1

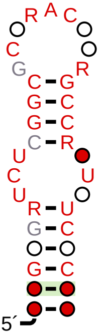

HMP2_1532_1

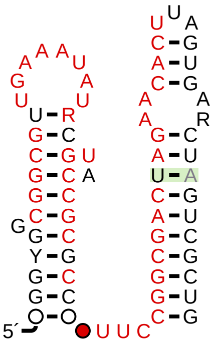

HMP2_15321_1

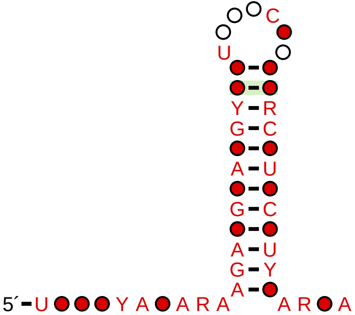

HMP2_15322_1

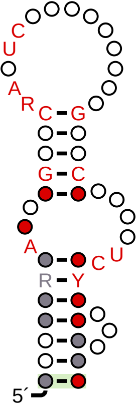

HMP2_15323_1

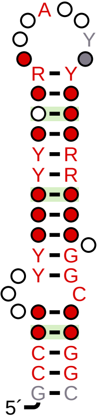

HMP2_15343_1

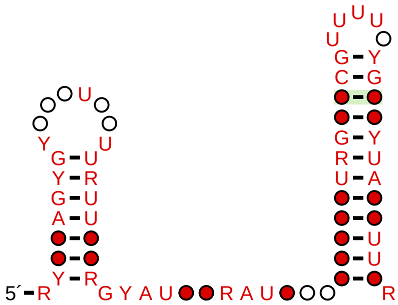

HMP2_1535_1

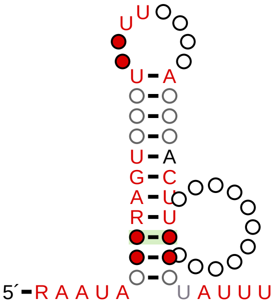

HMP2_15350_1

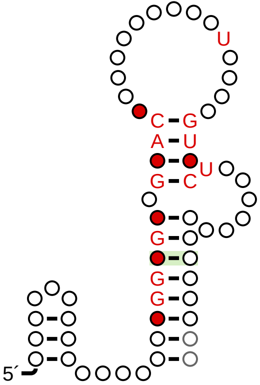

HMP2_15352_1

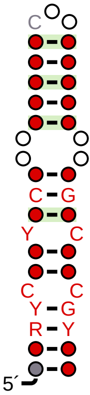

HMP2_15354_1

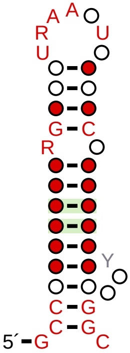

HMP2_15360_1

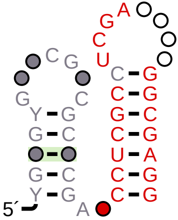

HMP2_15368_1

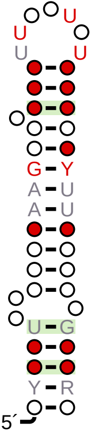

HMP2_15387_1

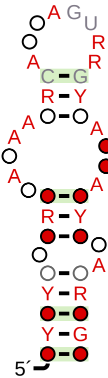

HMP2_15389_1

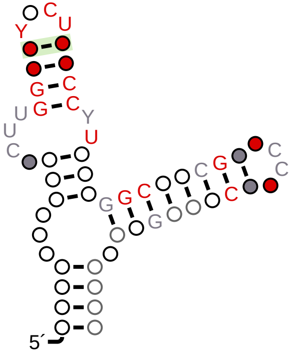

HMP2_15392_1

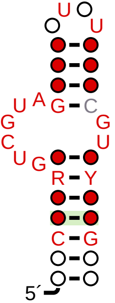

HMP2_15393_1

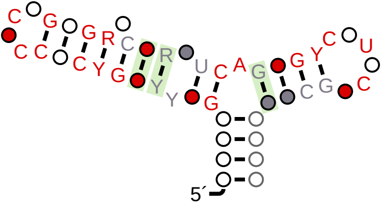

HMP2_15395_1

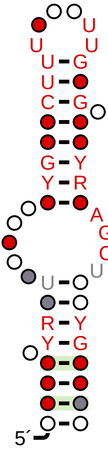

HMP2_15402_1

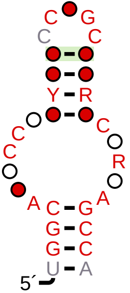

HMP2_15407_1

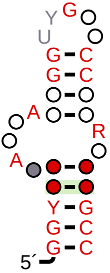

HMP2_15414_1

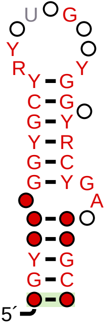

HMP2_15416_1

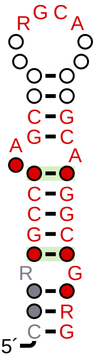

HMP2_15417_1

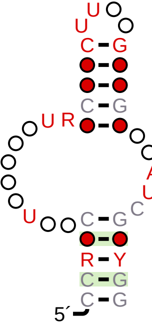

HMP2_15424_1

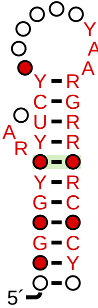

HMP2_15431_1

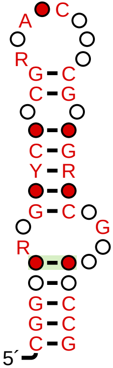

HMP2_15432_1

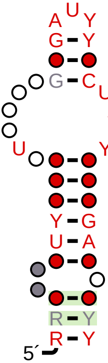

HMP2_15433_1

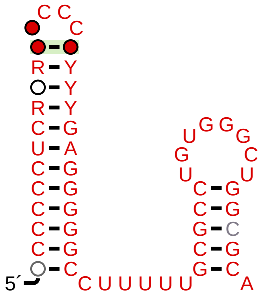

C C
C
R Y
Y
R Y
C G
U A
C G
C G
C G
C G
C G
C
5´
C U U U U U
U G G G
G G
G C
U U
C G
C G
G C
C G
G C
A

HMP2_15437_1

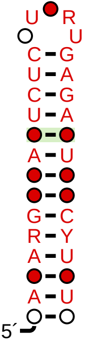

HMP2_15440_1

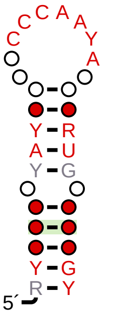

HMP2_15441_1

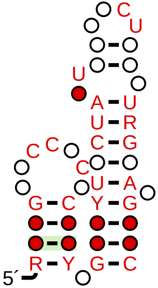

HMP2_15443_1

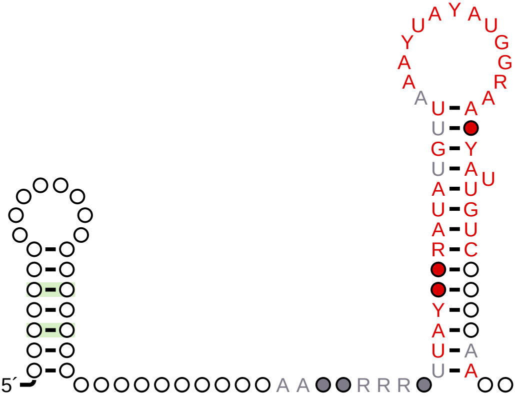

HMP2_15458_1

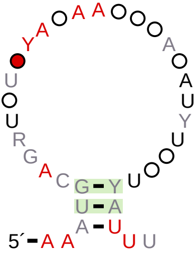

HMP2_15459_1

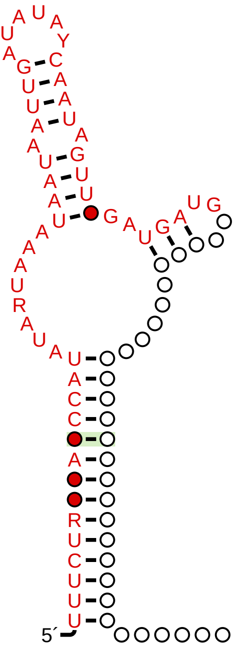

HMP2_15460_1

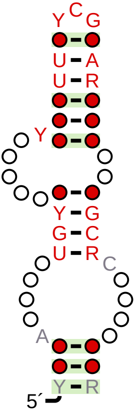

HMP2_15463_1

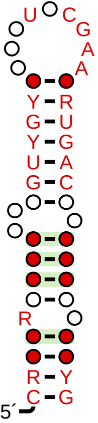

HMP2_15465_1

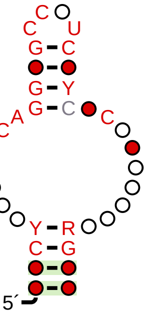

HMP2_15469_1

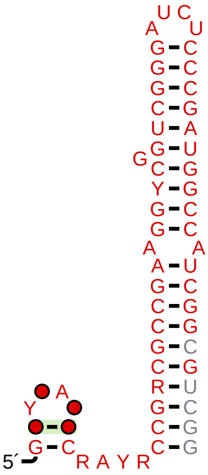

U C
A U
G C
G C
G C
C G
U A
G U
G
C G
Y G
G C
G C
A A
A U
G C
C G
C G
G C
C G
R U
G C
C G
C G
A
Y
G C
5´
R A Y R

HMP2_15472_1

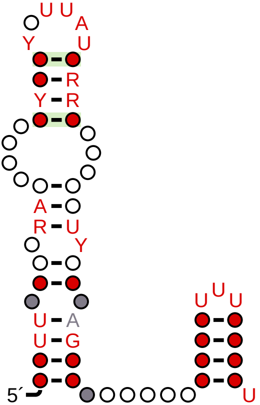

HMP2_15473_1

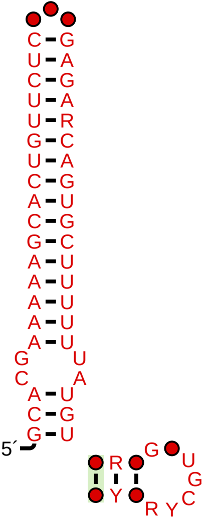

C G
U A
C G
U A
U R
G C
U A
C G
A U
C G
G C
A U
A U
A U
A U
A U
G U
C A
A U
C G
G U
5´
R G U
Y G
R Y C

HMP2_15478_1

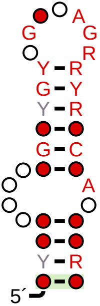

HMP2_1548_1

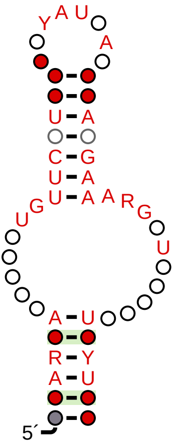

HMP2_15489_1

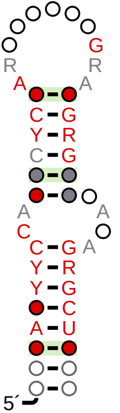

HMP2_15491_1

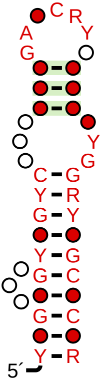

HMP2_15497_1

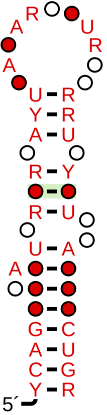

HMP2_1550_1

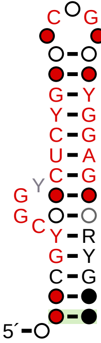

HMP2_15501_1

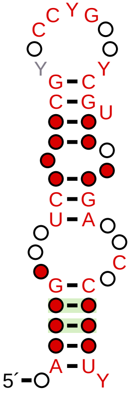

HMP2_15506_1

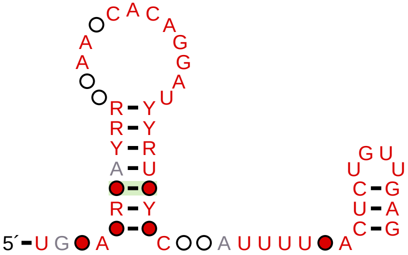

HMP2_15507_1

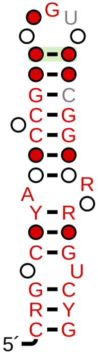

HMP2_15514_1

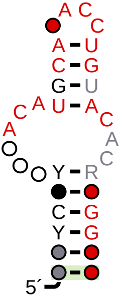

HMP2_1552_1

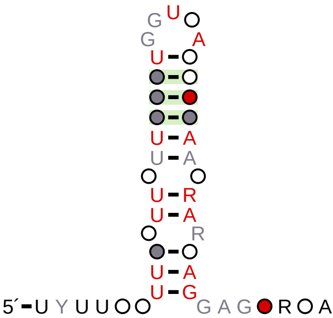

HMP2_15524_1

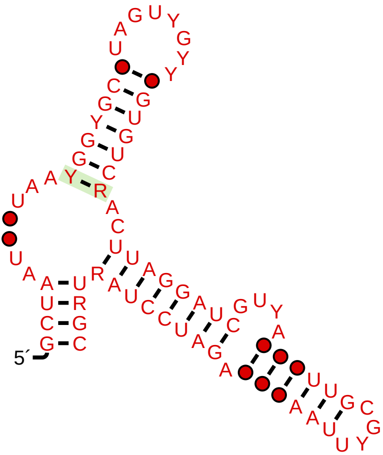

HMP2_15525_1

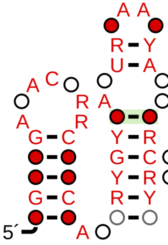

HMP2_15528_1

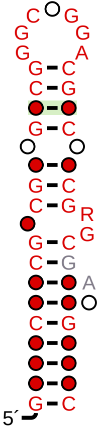

HMP2_15551_1

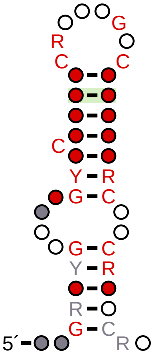

HMP2_15562_1

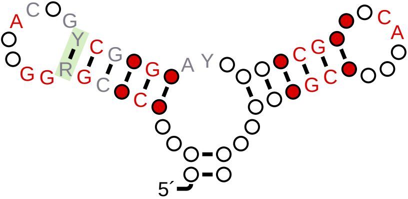

HMP2_15563_1

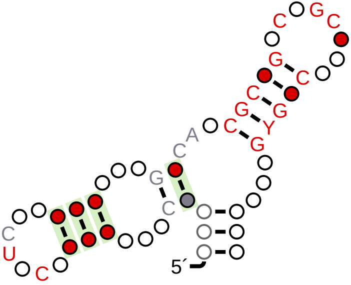

HMP2_1557_1

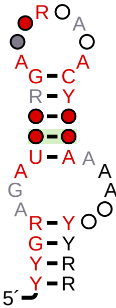

HMP2_15581_1

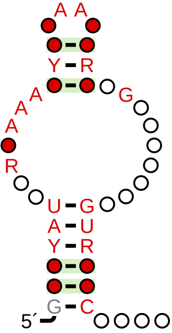

HMP2_15586_1

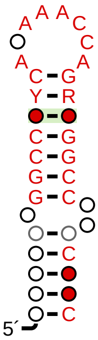

HMP2_15587_1

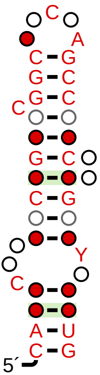

HMP2_15588_1

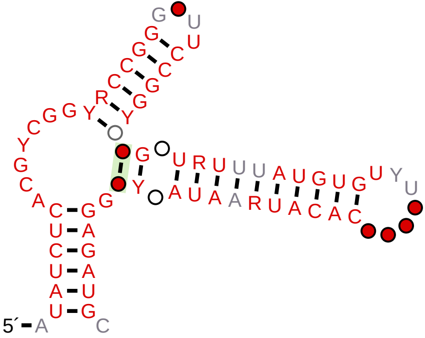

HMP2_15591_1

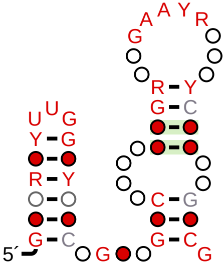

HMP2_15594_1

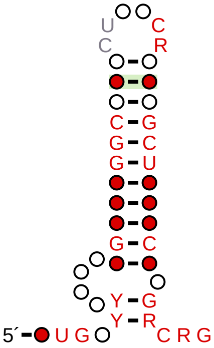

HMP2_15595_1

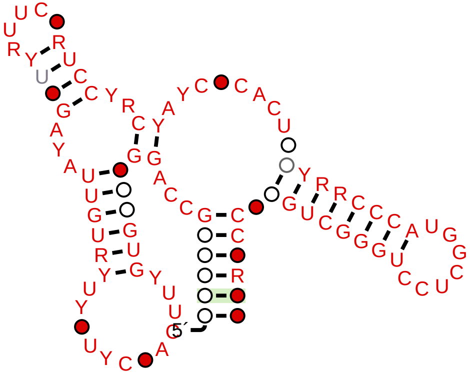

HMP2_15599_1

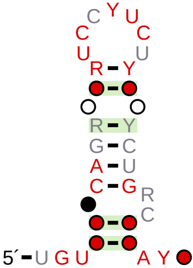

HMP2_1560_1

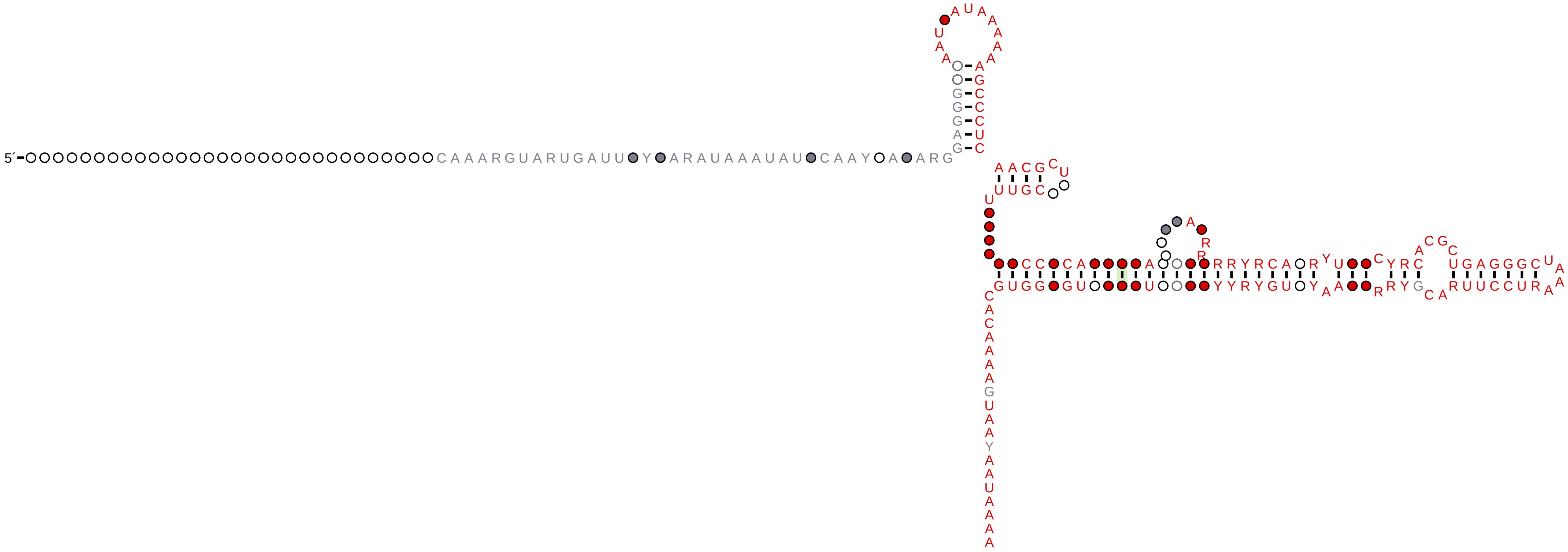

HMP2_15607_1

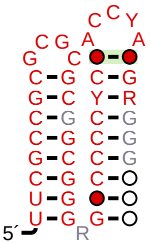

HMP2_15610_1

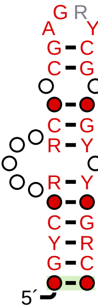

HMP2_15617_1

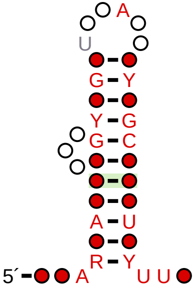

HMP2_1562_1

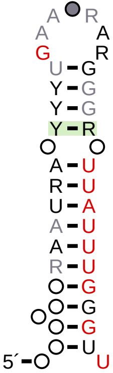

HMP2_15627_1

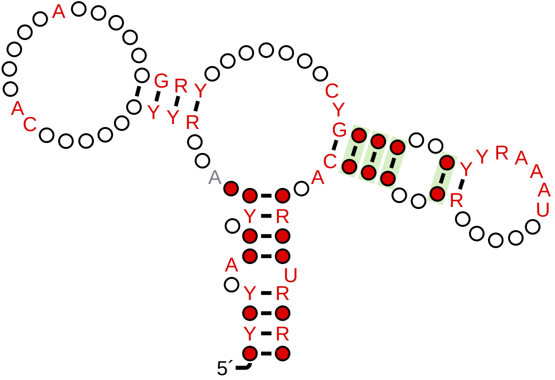

HMP2_15628_1

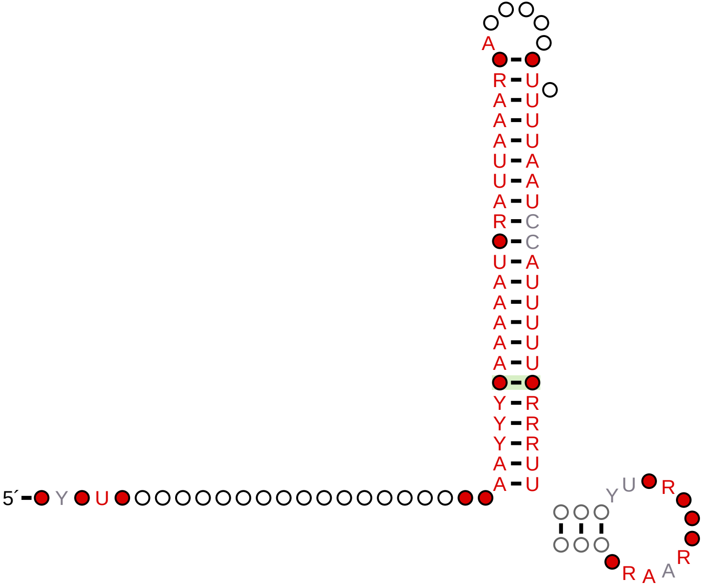

HMP2_15635_1

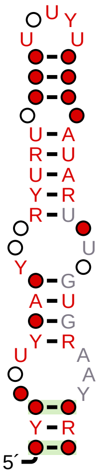

HMP2_15641_1

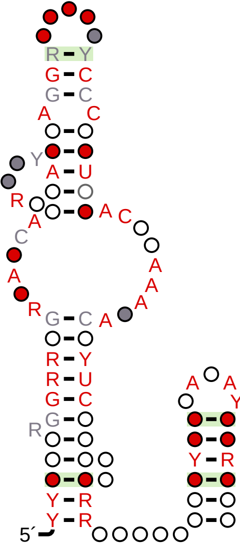

HMP2_15642_1

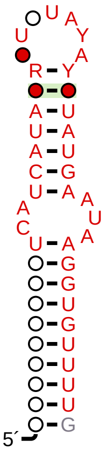

HMP2_15644_1

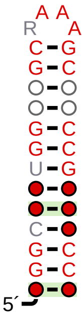

HMP2_15654_1

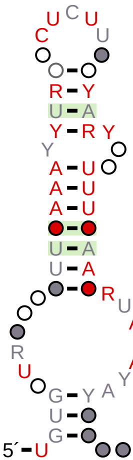

HMP2_15661_1

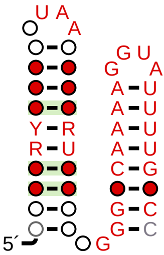

HMP2_15666_1

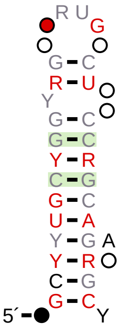

HMP2_15667_1

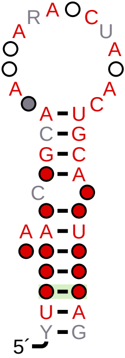

HMP2_15674_1

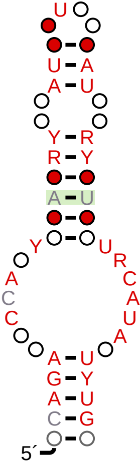

HMP2_15677_1

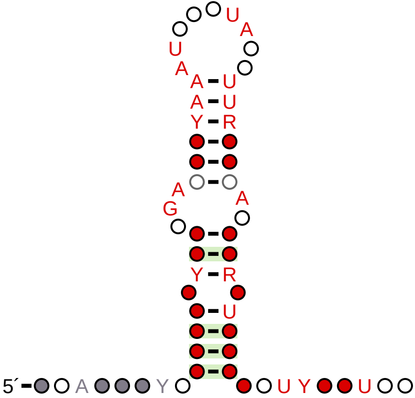

HMP2_15683_1

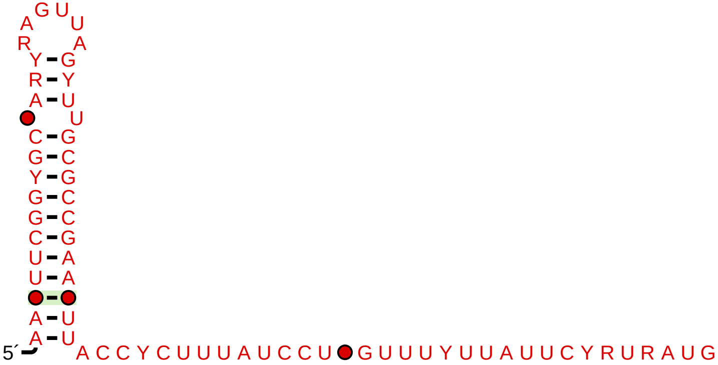

A G U U
R A
Y G
R Y
A U
U
C G
G C
Y G
G C
G C
C G
U A
U A
A U
A U
5´
A C C Y C U U U A U C C U G U U U Y U U A U U C Y R U R A U G

HMP2_15685_1

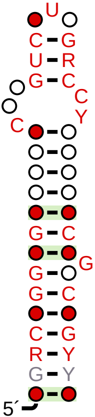

HMP2_15686_1

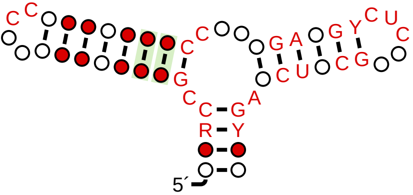

HMP2_1569_1

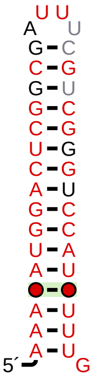

HMP2_15695_1

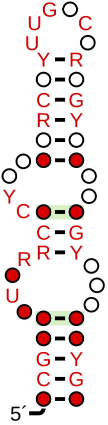

HMP2_15696_1

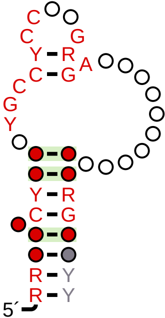

HMP2_15697_1

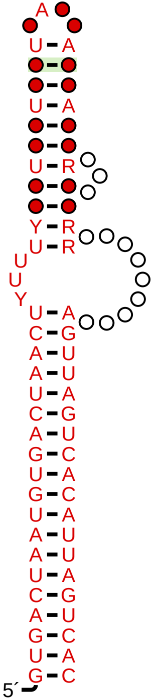

HMP2_15702_1

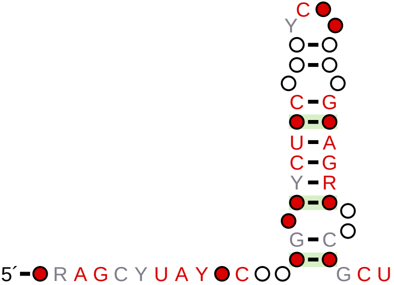

HMP2_15712_1

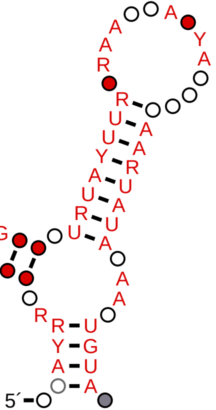

HMP2_15713_1

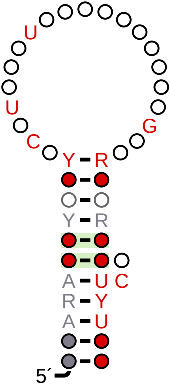

HMP2_15721_1

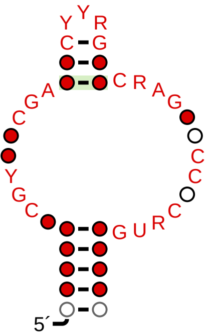

HMP2_15729_1

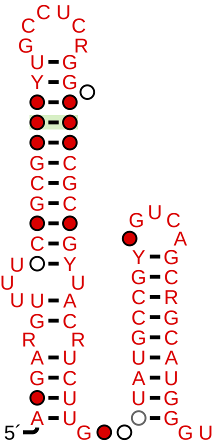

HMP2_15730_1

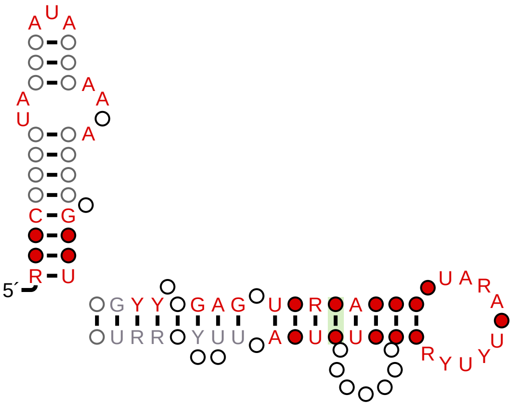

HMP2_15739_1

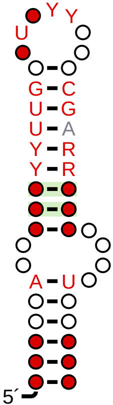

HMP2_15745_1

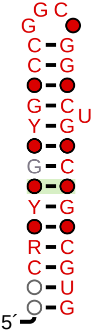

HMP2_15747_1

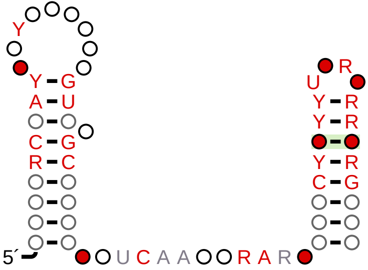

HMP2_15756_1

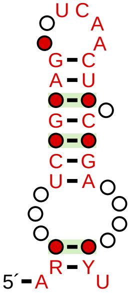

HMP2_15758_1

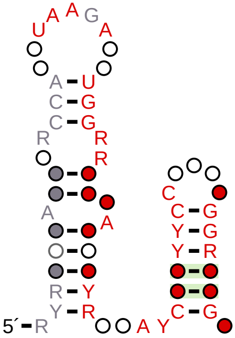

HMP2_15759_1

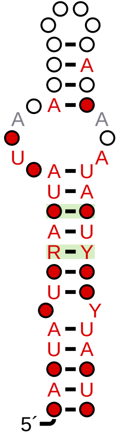

HMP2_15770_1

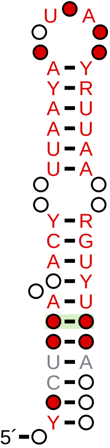

HMP2_15771_1

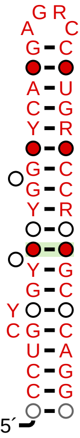

HMP2_15776_1

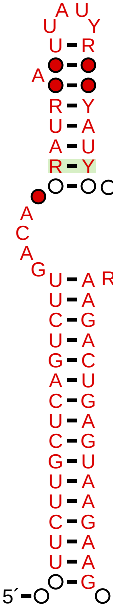

HMP2_15786_1

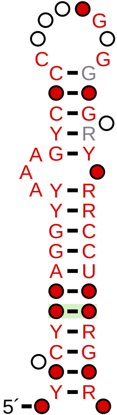

HMP2_15789_1

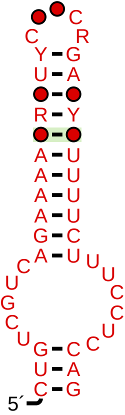

HMP2_1579_1

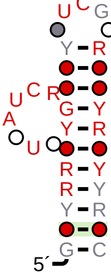

HMP2_15790_1

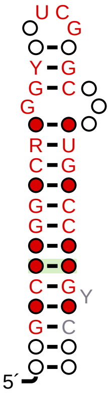

HMP2_15792_1

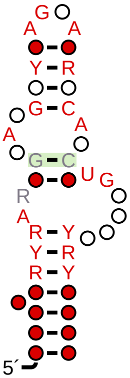

HMP2_15797_1

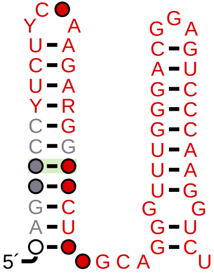

HMP2_15798_1

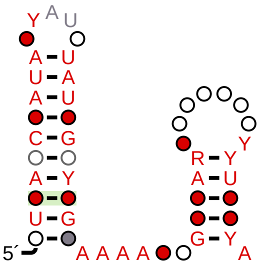

HMP2_15799_1

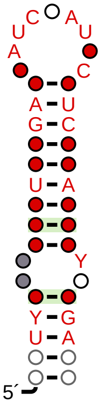

HMP2_15800_1

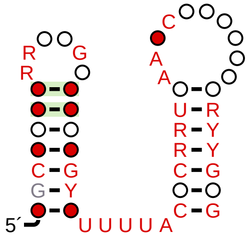

HMP2_15804_1

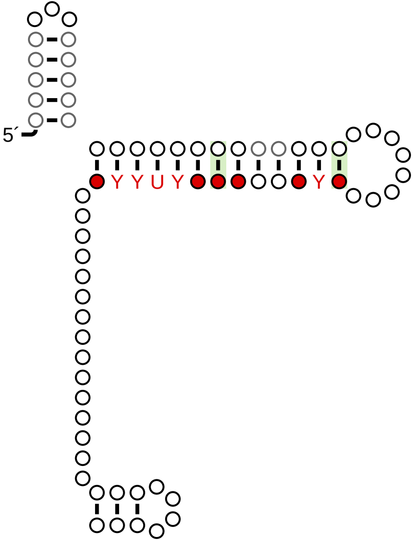

HMP2_15805_1

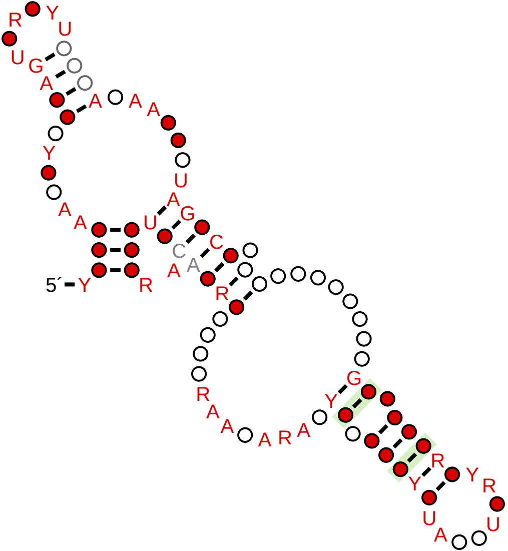

HMP2_15812_1

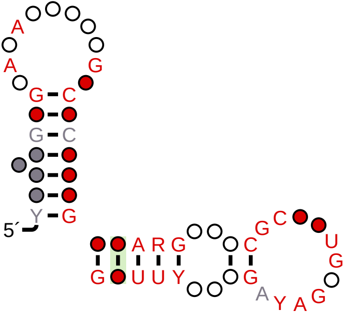

HMP2_15814_1

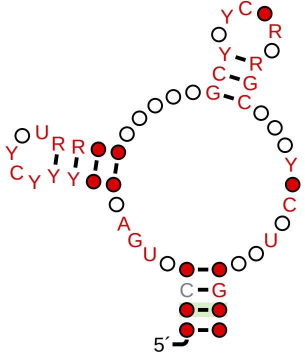

HMP2_15817_1

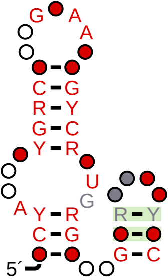

HMP2_15820_1

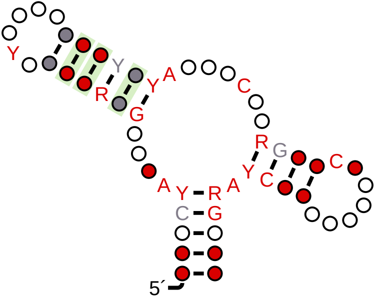

HMP2_15821_1

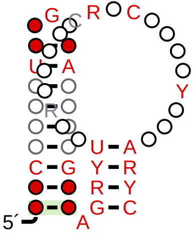

HMP2_15823_1

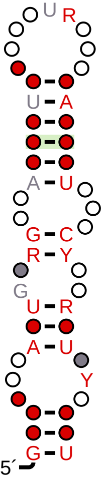

HMP2_15824_1

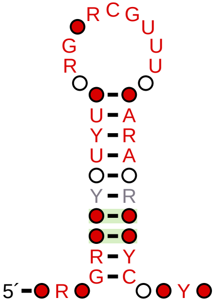

HMP2_1583_1

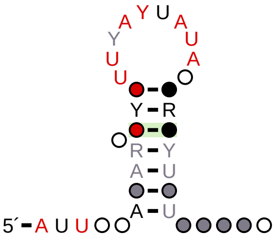

HMP2_15830_1

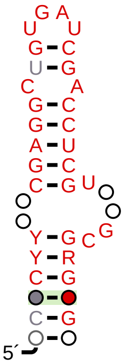

HMP2_15831_1

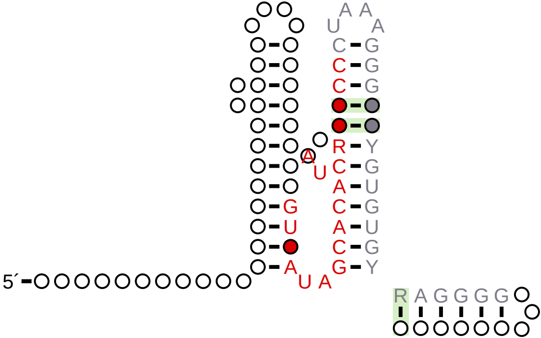

HMP2_15835_1

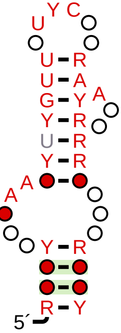

HMP2_15838_1

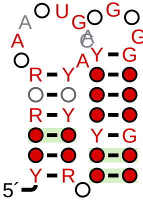

HMP2_1584_1

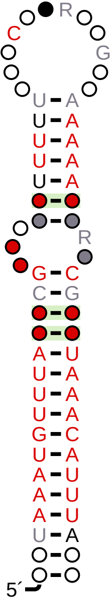

HMP2_15847_1

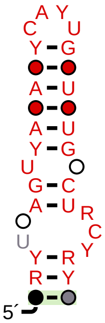

HMP2_15848_1

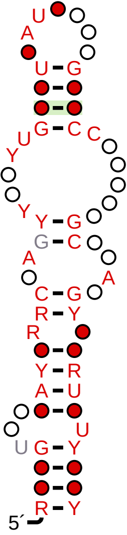

HMP2_15849_1

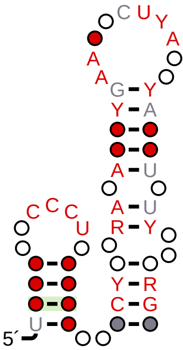

HMP2_15850_1

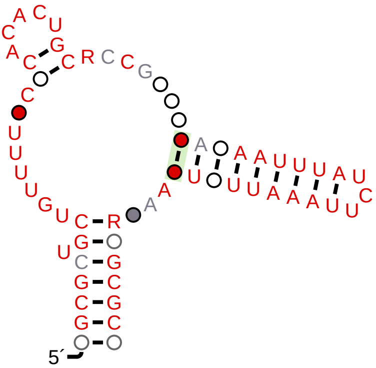

HMP2_15851_1

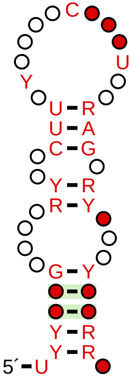

HMP2_15852_1

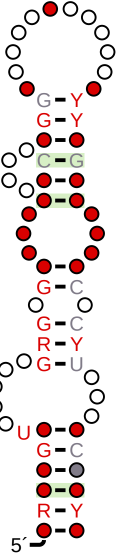

HMP2_15858_1

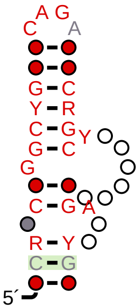

HMP2_15861_1

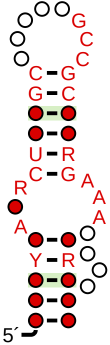

HMP2_15869_1

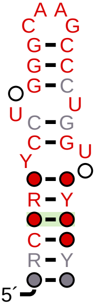

HMP2_1587_1

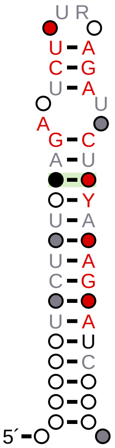

HMP2_1588_1

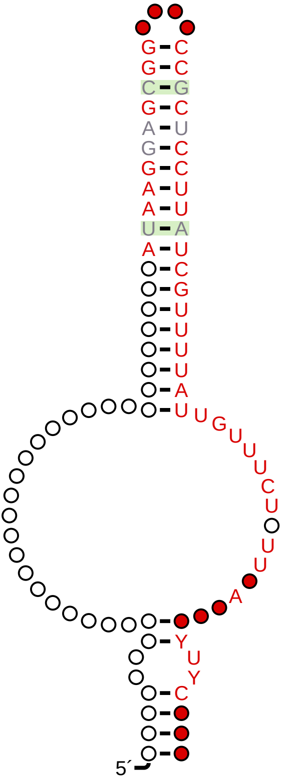

HMP2_15886_1

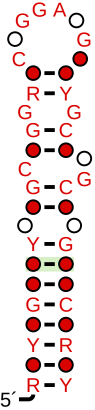

HMP2_15887_1

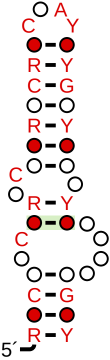

HMP2_15893_1

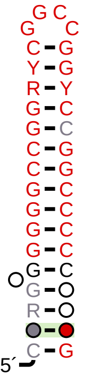

HMP2_15898_1

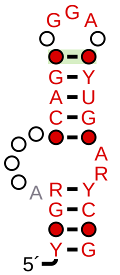

HMP2_15901_1

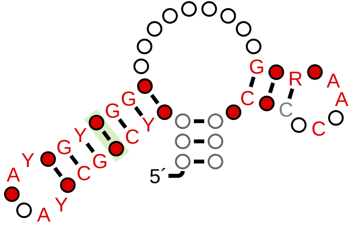

HMP2_15903_1

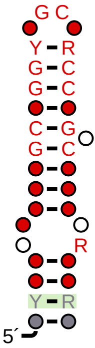

HMP2_15908_1

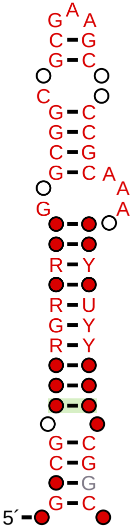

HMP2_15909_1

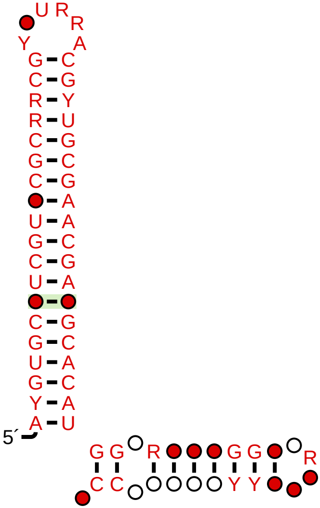

HMP2_15912_1

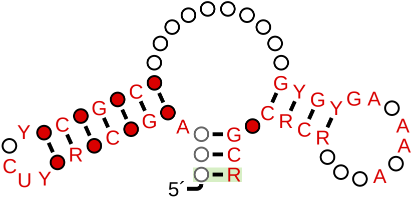

HMP2_15918_1

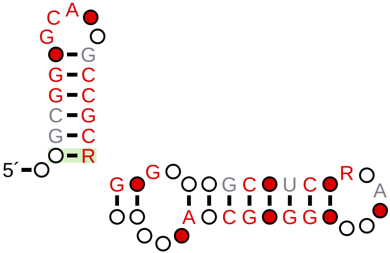

HMP2_15920_1

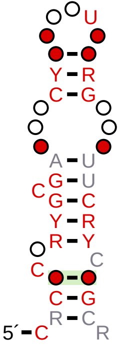

HMP2_15927_1

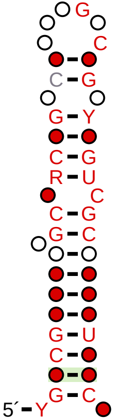

HMP2_15929_1

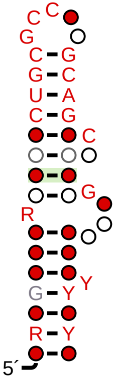

HMP2_15930_1

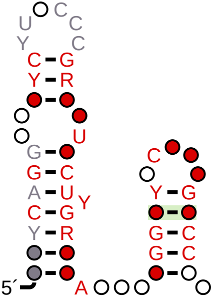

HMP2_15932_1

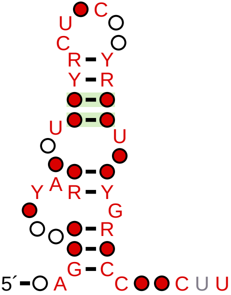

HMP2_15938_1

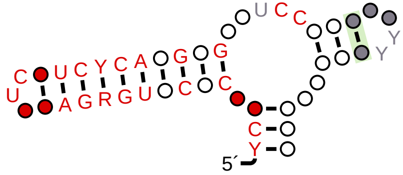

HMP2_15943_1

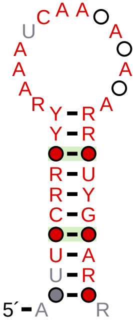

HMP2_15944_1

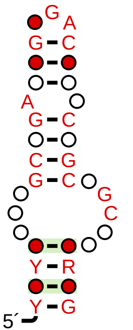

HMP2_15947_1

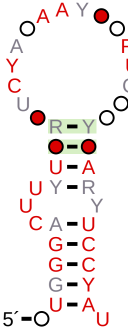

HMP2_15948_1

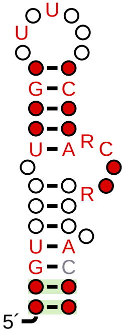

HMP2_15956_1

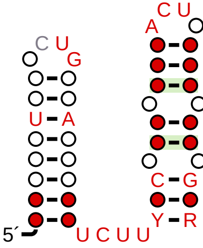

HMP2_15960_1

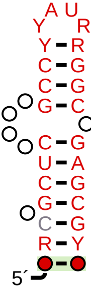

HMP2_15965_1

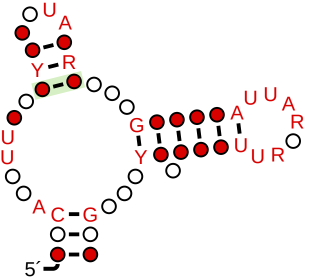

HMP2_15972_1

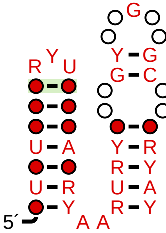

HMP2_15974_1

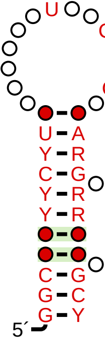

HMP2_15976_1

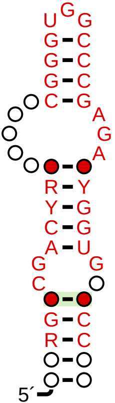

HMP2_1598_1

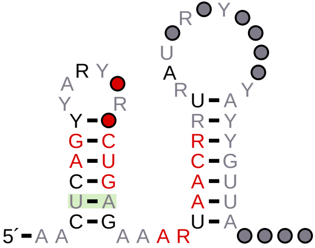

HMP2_15986_1

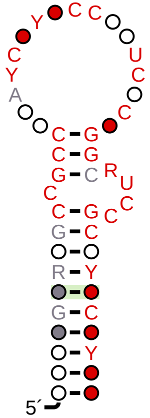

HMP2_15987_1

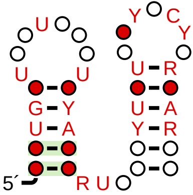

HMP2_15991_1

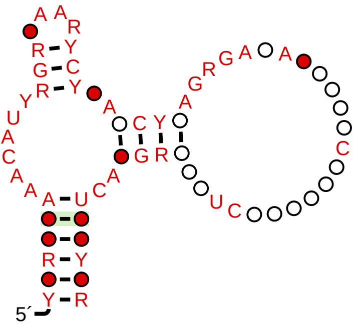

HMP2_16000_1

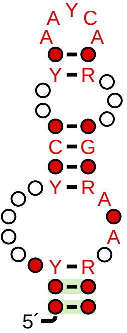

HMP2_16004_1

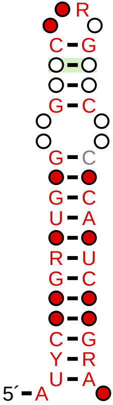

HMP2_16009_1

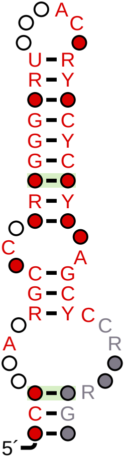

HMP2_16012_1

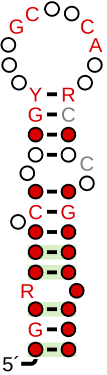

HMP2_16013_1

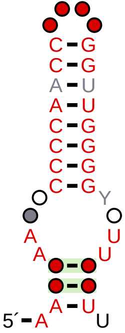

HMP2_1602_1

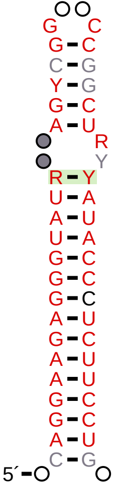

HMP2_1603_1

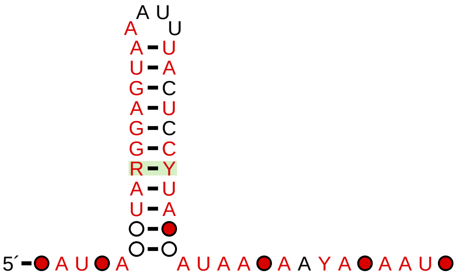

HMP2_16034_1

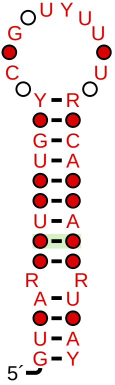

HMP2_16038_1

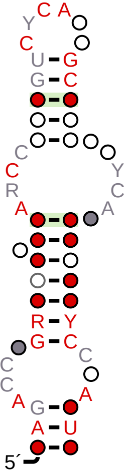

HMP2_16041_1

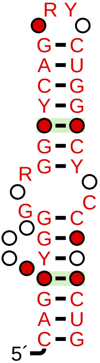

HMP2_1605_1

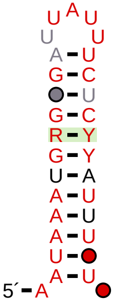

HMP2_16050_1

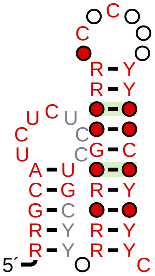

HMP2_16064_1

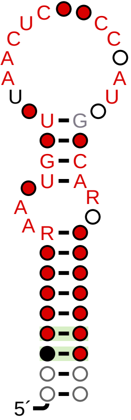

HMP2_16069_1

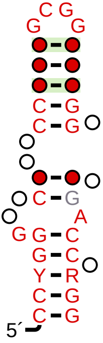

HMP2_1607_1

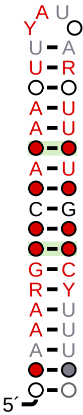

HMP2_16071_1

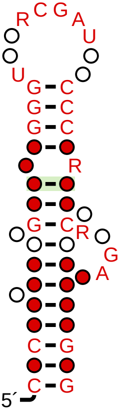

HMP2_16075_1

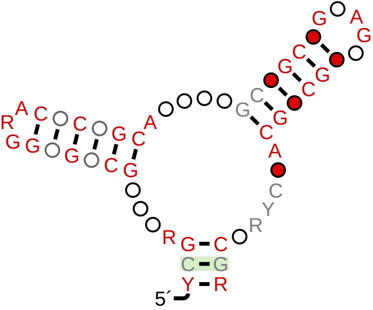

HMP2_16079_1

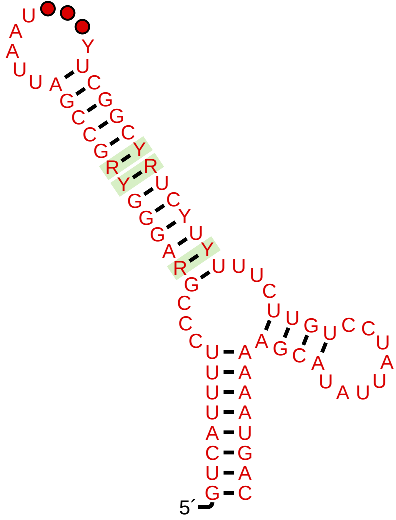

HMP2_1608_1

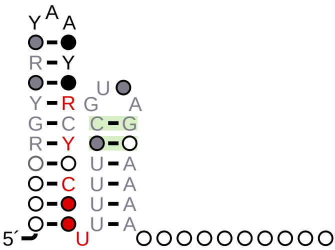

HMP2_16080_1

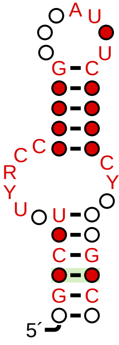

HMP2_16088_1

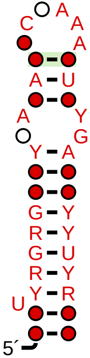

HMP2_1609_1

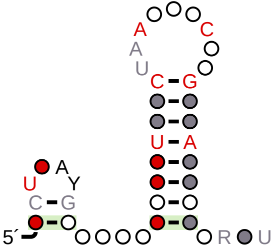

HMP2_16094_1

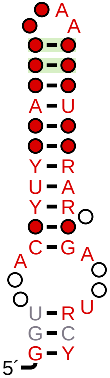

HMP2_16099_1

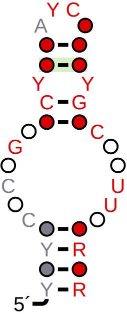

HMP2_161_1

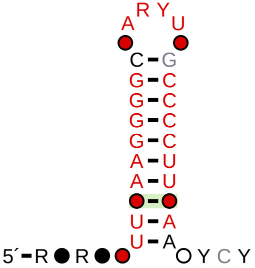

HMP2_16101_1

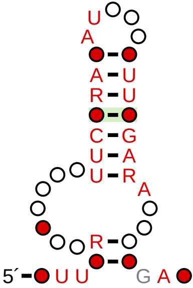

HMP2_16103_1

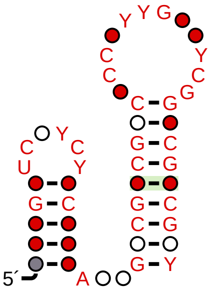

HMP2_16104_1

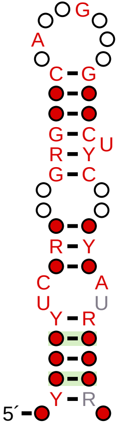

HMP2_16109_1

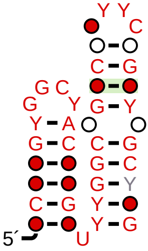

HMP2_16115_1

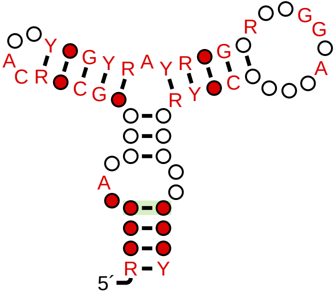

HMP2_16116_1

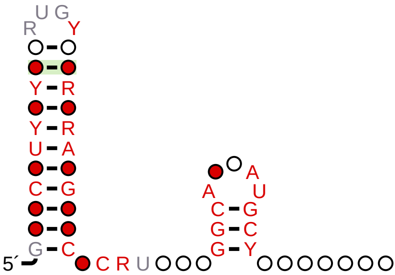

HMP2_16128_1

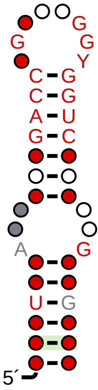

HMP2_16129_1

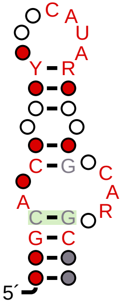

HMP2_16131_1

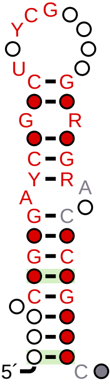

HMP2_16140_1

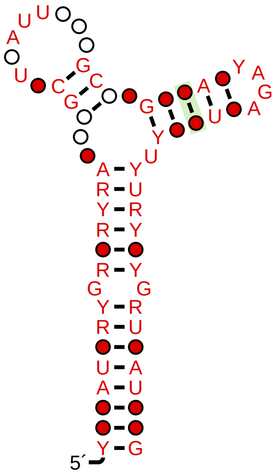

HMP2_16141_1

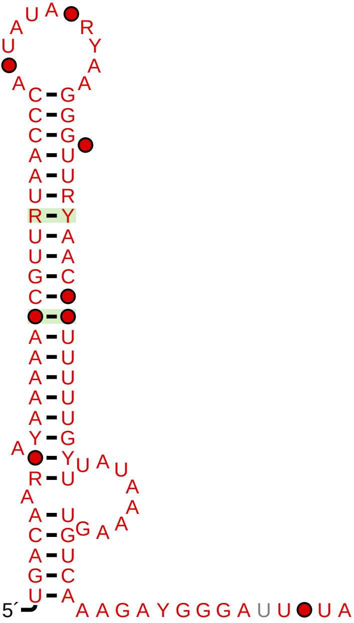

HMP2_16147_1

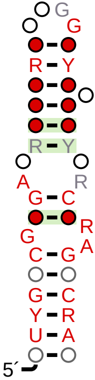

HMP2_1615_1

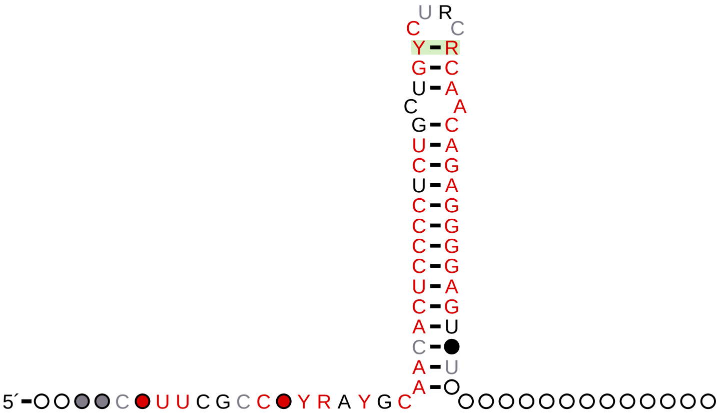

HMP2_16150_1

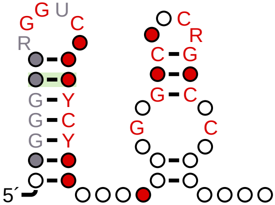

HMP2_16152_1

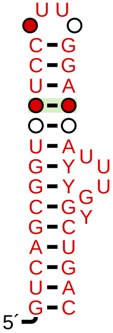

HMP2_1616_1

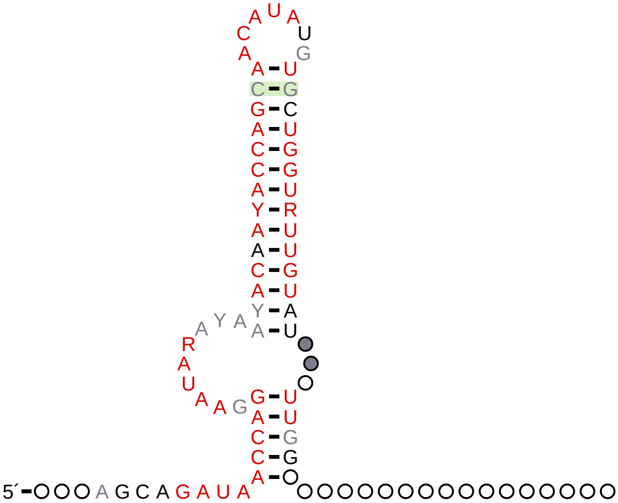

HMP2_16160_1

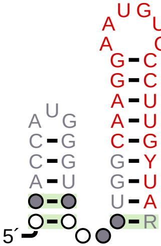

HMP2_16161_1

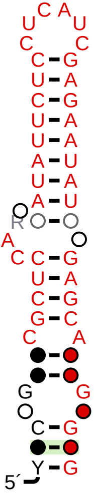

HMP2_16168_1

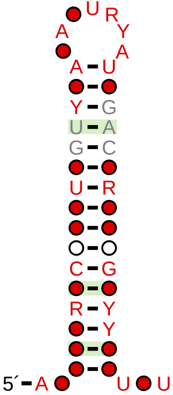

HMP2_1617_1

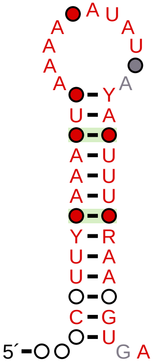

HMP2_16171_1

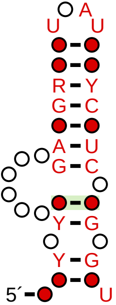

HMP2_16174_1

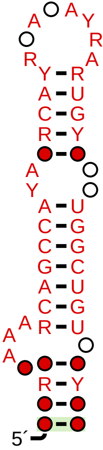

HMP2_16176_1

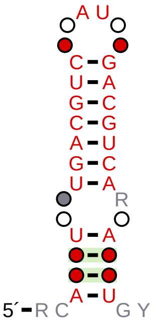

HMP2_16177_1

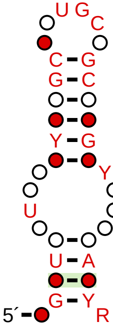

HMP2_16178_1

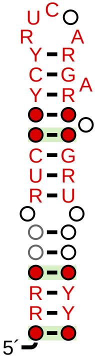

HMP2_1618_1

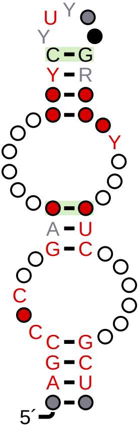

HMP2_162_1

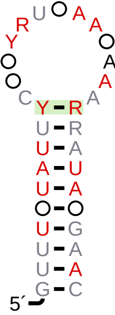

HMP2_16202_1

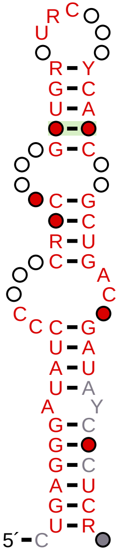

HMP2_16204_1

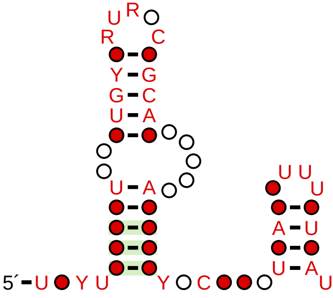

HMP2_16213_1

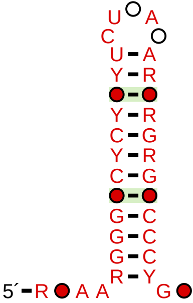

HMP2_16214_1

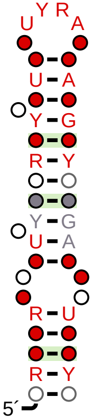

HMP2_16222_1

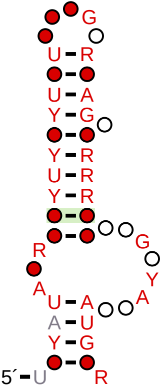

HMP2_16227_1

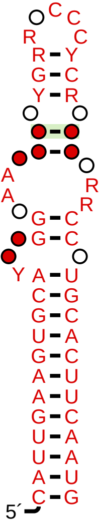

HMP2_1623_1

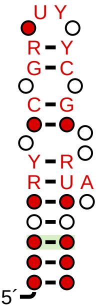

HMP2_16230_1

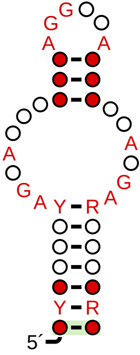

HMP2_16236_1

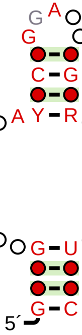

HMP2_16246_1

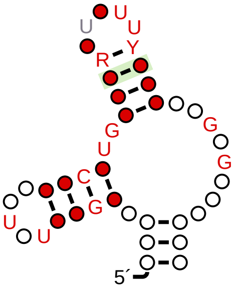

HMP2_16249_1

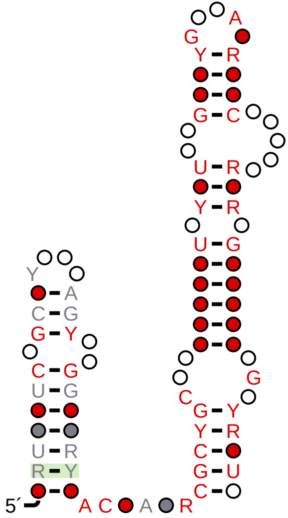

HMP2_16256_1

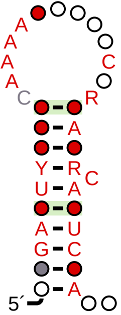

HMP2_16257_1

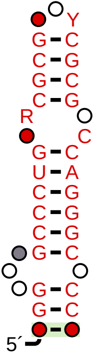

HMP2_16258_1

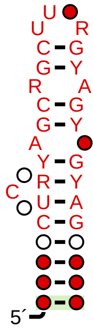

HMP2_16261_1

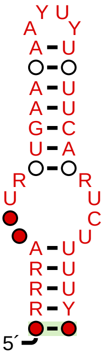

HMP2_16264_1

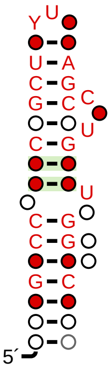

HMP2_16269_1

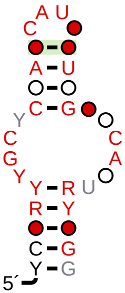

HMP2_1628_1

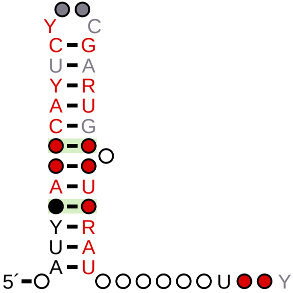

HMP2_16281_1

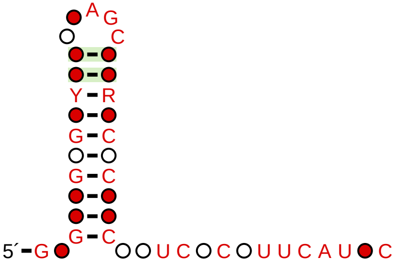

HMP2_16282_1

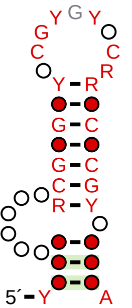

HMP2_16289_1

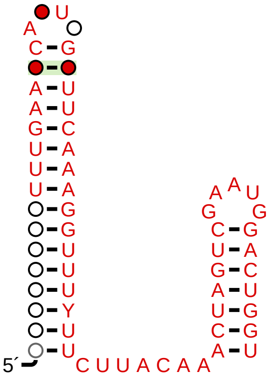

HMP2_16296_1

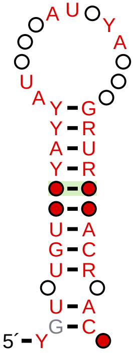

HMP2_16302_1

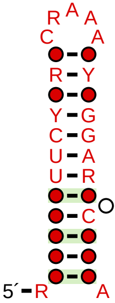

HMP2_16305_1

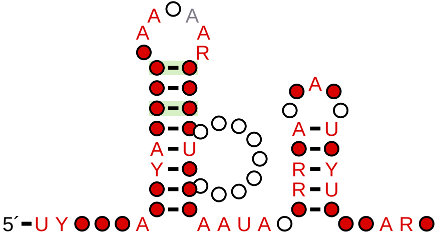

HMP2_16306_1

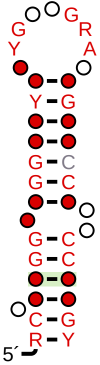

HMP2_16317_1

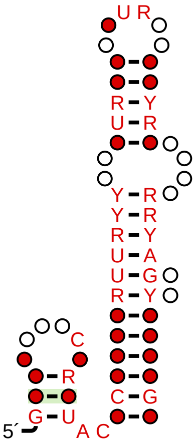

HMP2_16318_1

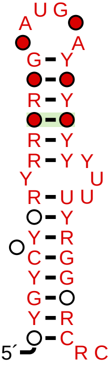

HMP2_16320_1

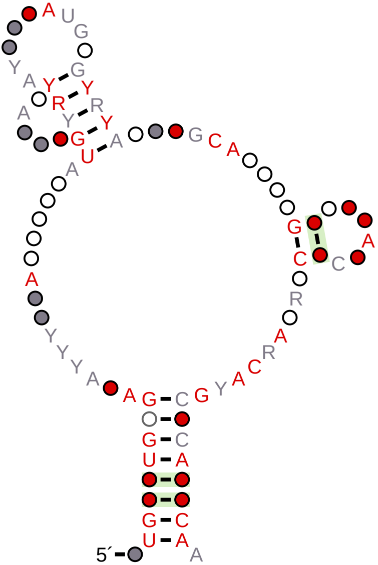

HMP2_16332_1

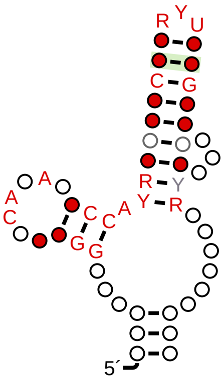

HMP2_16335_1

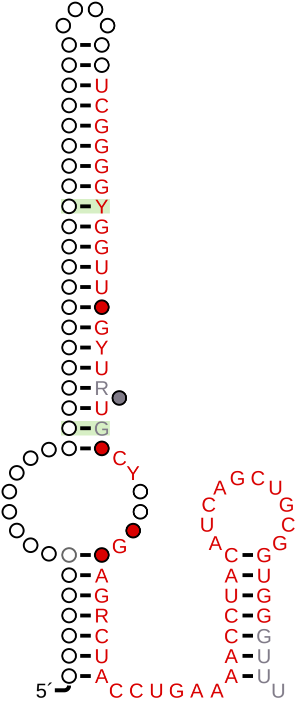

U
C
G
G
G
G
Y
G
G
U
U
G
Y
U
R
U
G
C
Y
G
A
G
R
C
U
A
5´
C C U G A A
A G C U
C G
U C
A G
C G
A U
U G
C G
C G
A U
A U
U

HMP2_16350_1

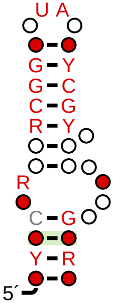

HMP2_16353_1

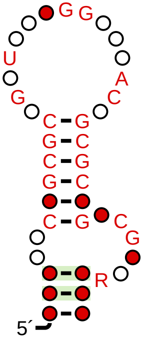

HMP2_16356_1

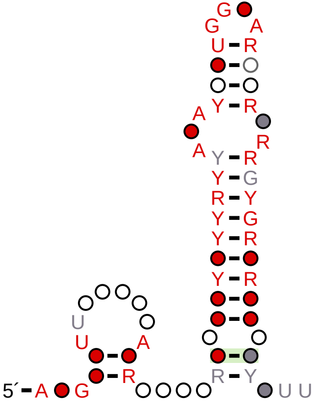

HMP2_16360_1

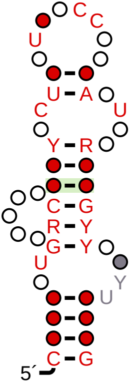

HMP2_16362_1

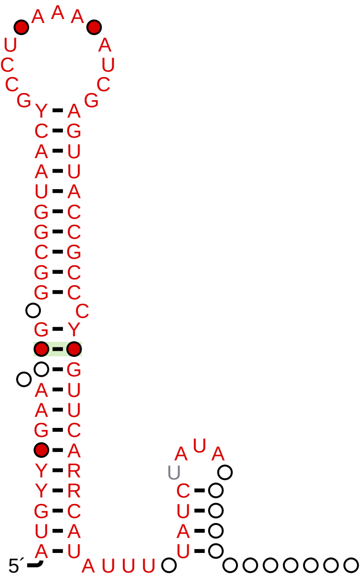

HMP2_16367_1

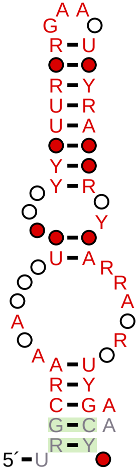

HMP2_16369_1

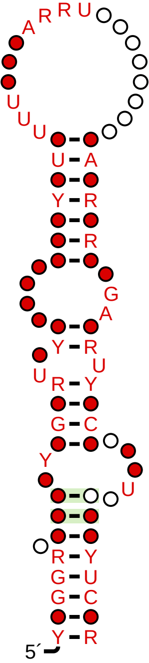

HMP2_1637_1

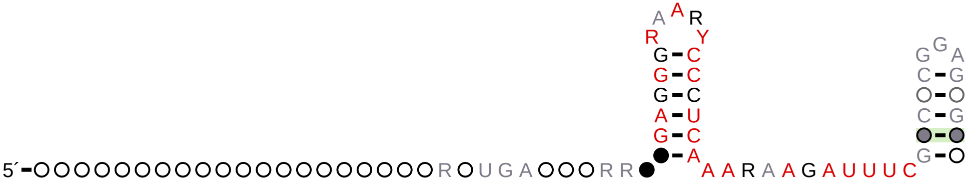

HMP2_16378_1

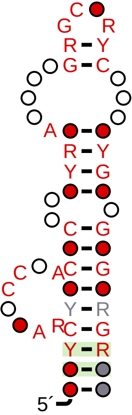

HMP2_16380_1

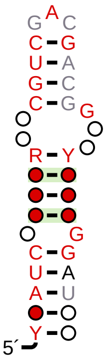

HMP2_16383_1

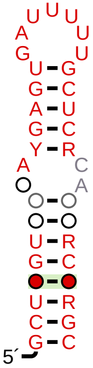

HMP2_16386_1

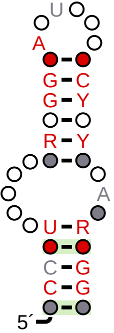

HMP2_16387_1

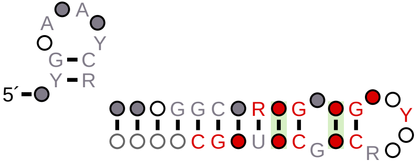

HMP2_16391_1

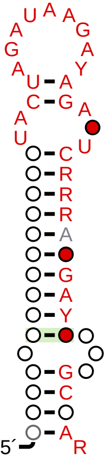

HMP2_16392_1

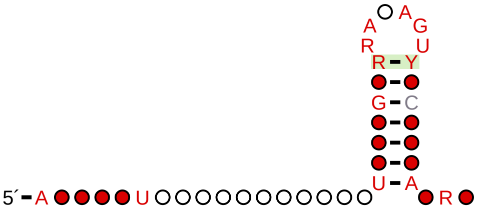

HMP2_16400_1

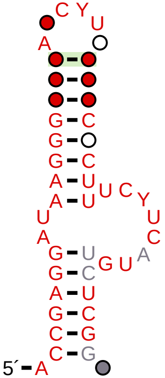

HMP2_16401_1

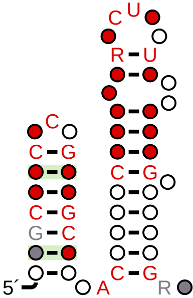

HMP2_16403_1

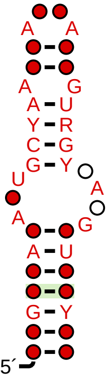

HMP2_16406_1

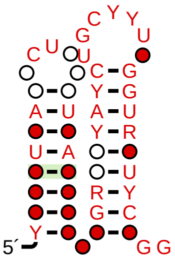

HMP2_16409_1

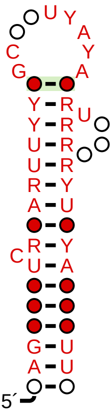

HMP2_16410_1

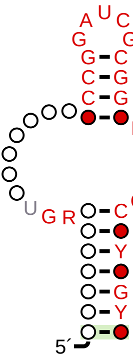

HMP2_16411_1

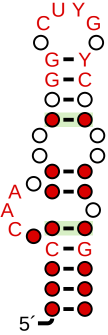

HMP2_16414_1

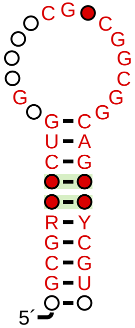

HMP2_16421_1

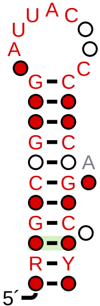

HMP2_16424_1

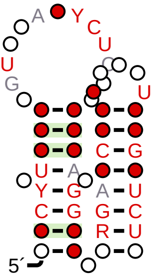

HMP2_16425_1

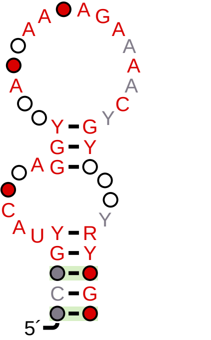

HMP2_16429_1

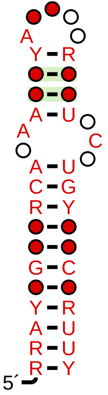

HMP2_16434_1

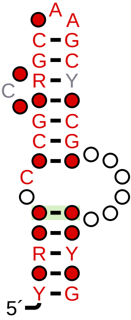

HMP2_16440_1

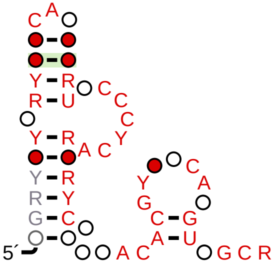

HMP2_16443_1

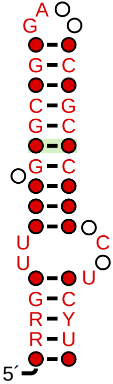

HMP2_16447_1

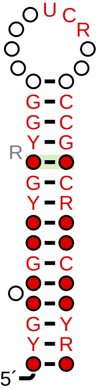

HMP2_16451_1

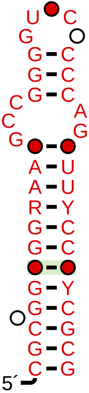

HMP2_16455_1

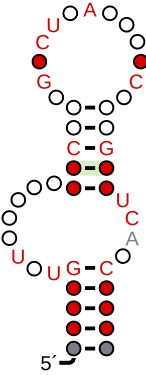

HMP2_16460_1

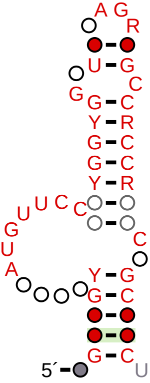

HMP2_16462_1

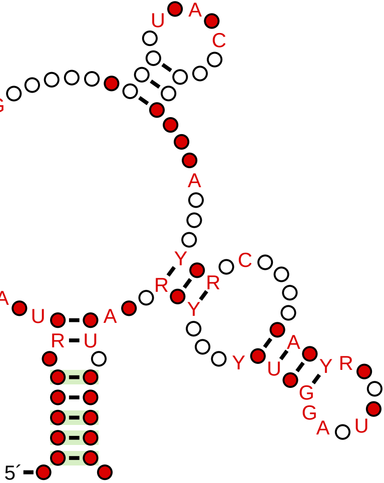

HMP2_16465_1

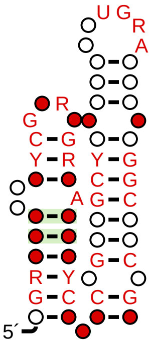

HMP2_16468_1

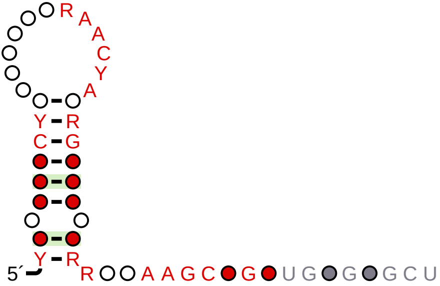

HMP2_16470_1

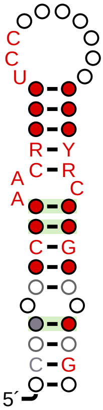

HMP2_16472_1

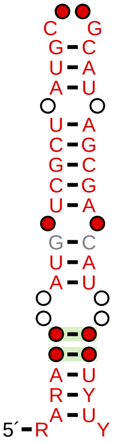

HMP2_16476_1

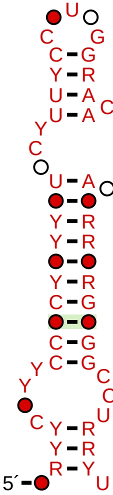

HMP2_16478_1

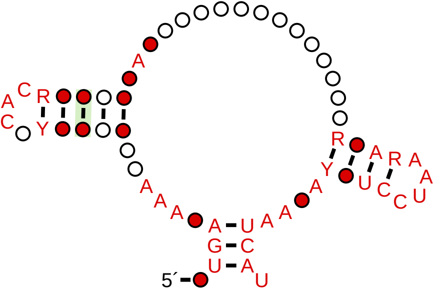

HMP2_1648_1

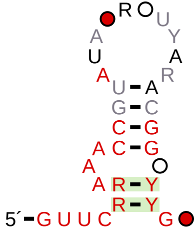

HMP2_16482_1

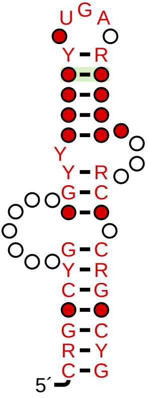

HMP2_16484_1

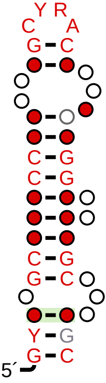

HMP2_16485_1

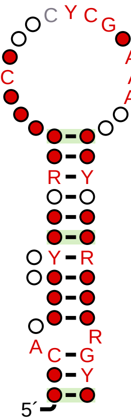

HMP2_16487_1

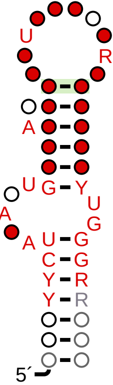

HMP2_16492_1

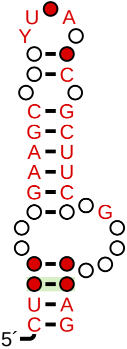

HMP2_16495_1

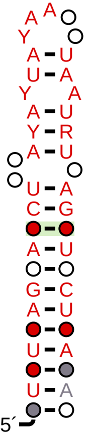

HMP2_16501_1

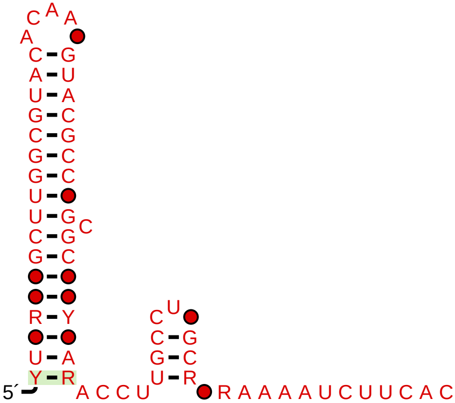

5´
C A A
A
C G
A U
U A
G C
C G
G C
G C
U
U G
C
C G
G C
R Y
U A
Y R
A C C U
C U
C G
G C
U R
R A A A A U C U U C A C

HMP2_16503_1

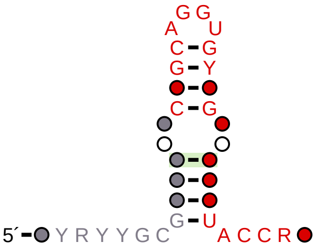

HMP2_16507_1

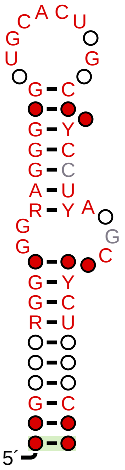

HMP2_16509_1

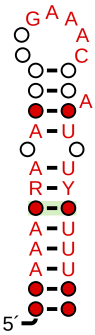

HMP2_16514_1

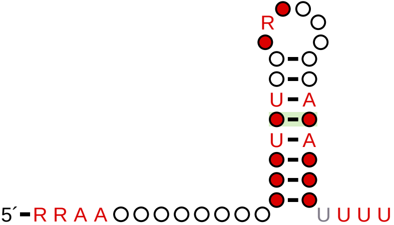

HMP2_16516_1

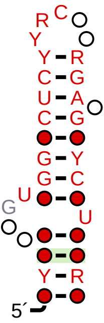

HMP2_16517_1

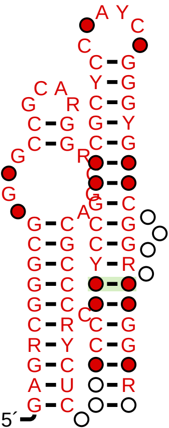

HMP2_16522_1

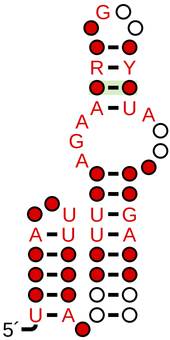

HMP2_16523_1

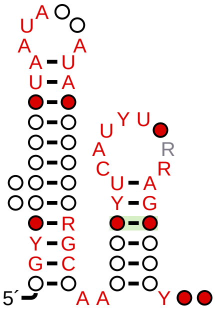

HMP2_1655_1

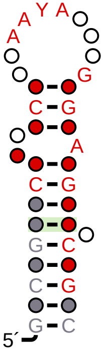

HMP2_1656_1

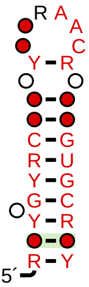

HMP2_1658_1

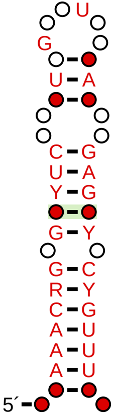

HMP2_1661_1

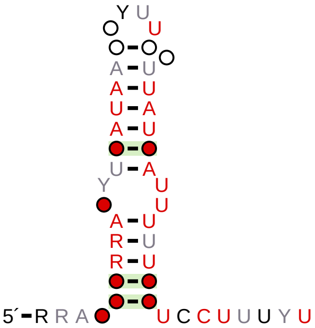

HMP2_1664_1

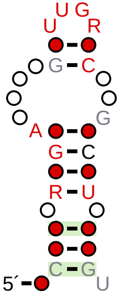

HMP2_167_1

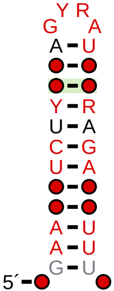

HMP2_1671_1

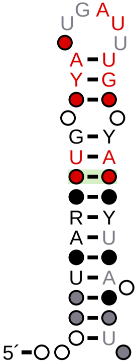

HMP2_1676_1

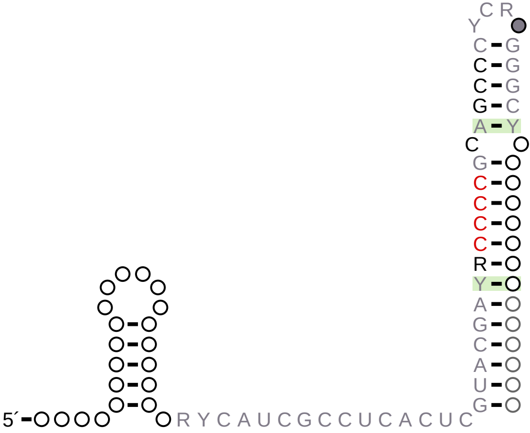

HMP2_1678_1

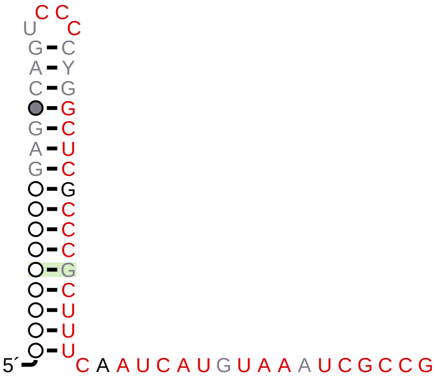

HMP2_1685_1

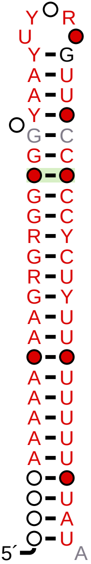

HMP2_1693_1

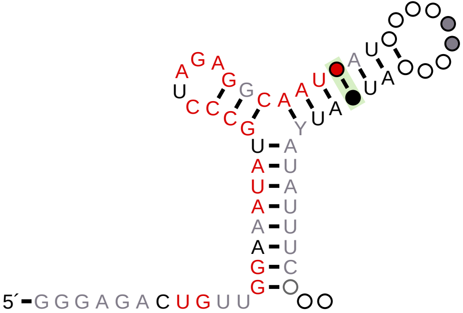

5´
GGGAGACUGUU
GGAAUAUU
CCGUCCUAGAGG
CAUUA
UAUA
ACUUAUAUUAC
Y

HMP2_1695_1

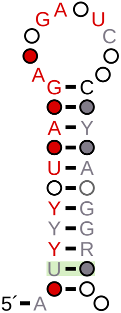

HMP2_1696_1

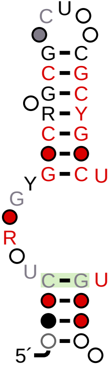

HMP2_1701_1

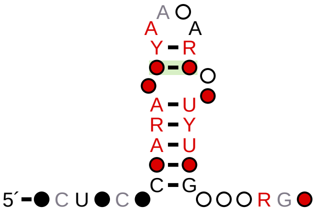

HMP2_171_1

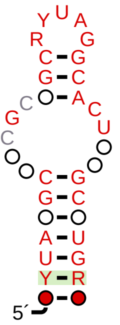

HMP2_1715_1

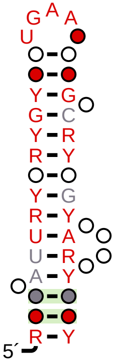

HMP2_1717_1

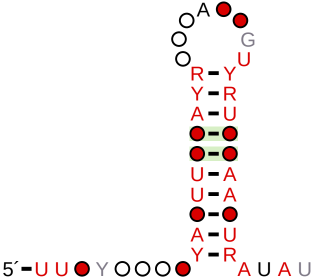

HMP2_1725_1

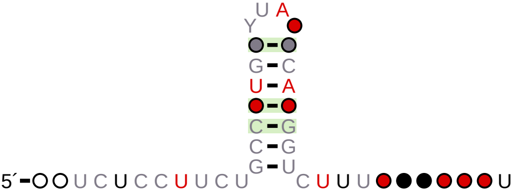

HMP2_1728_1

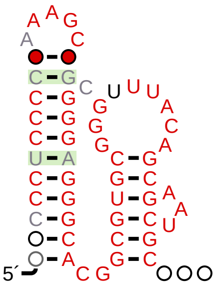

HMP2_1729_1

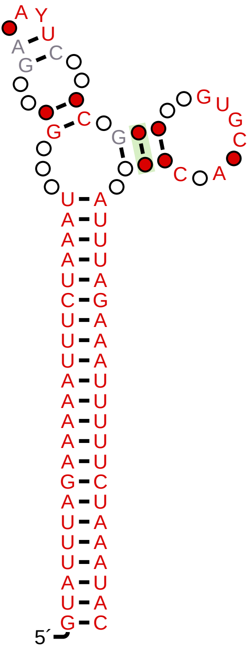

HMP2_1732_1

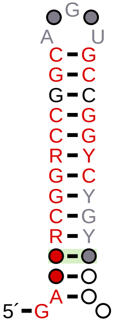

HMP2_1734_1

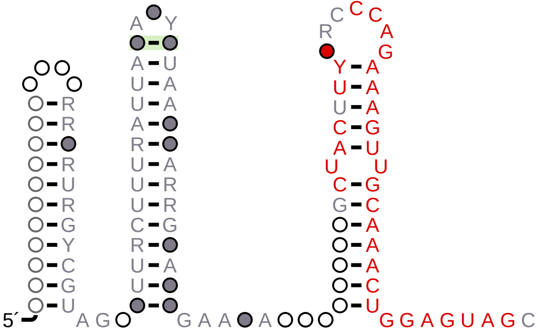

HMP2_1736_1

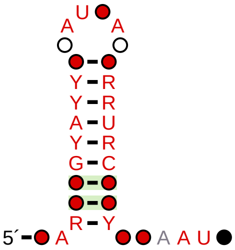

HMP2_1737_1

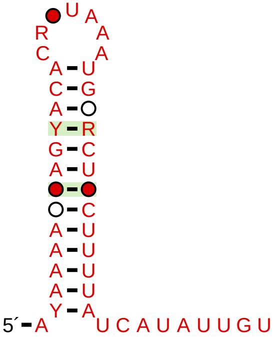

HMP2_1742_1

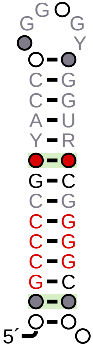

HMP2_1743_1

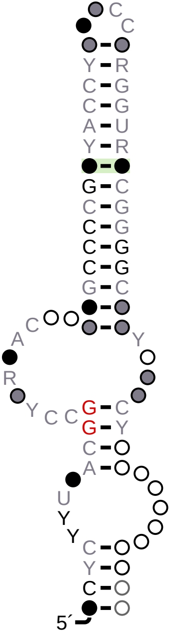

HMP2_175_1

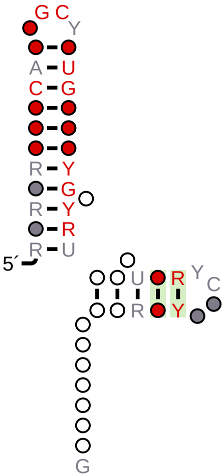

HMP2_1751_1

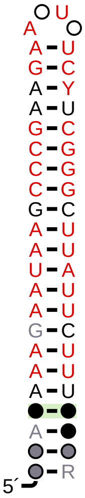

HMP2_176_1

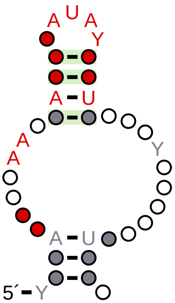

HMP2_1760_1

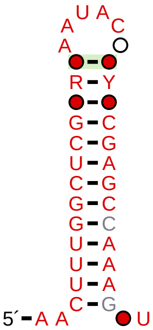

HMP2_1764_1

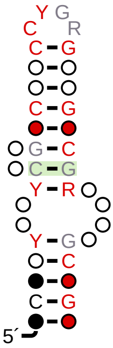

HMP2_1771_1

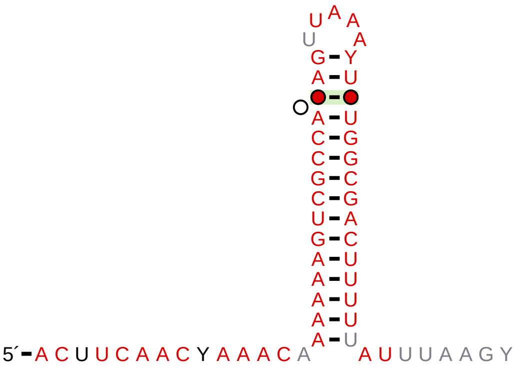
5´-ACUUCAACYAAACA
AUUUAAGY
UAA
U A
G-Y
A-U
A-U
C-G
C-G
G-C
C-G
U-A
G-C
A-U
A-U
A-U
A-U
A-U

HMP2_1784_1

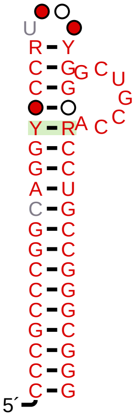

HMP2_1794_1

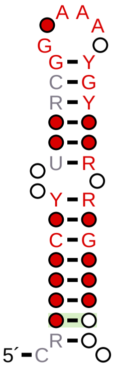

HMP2_1795_1

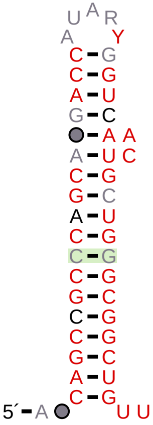

HMP2_1799_1

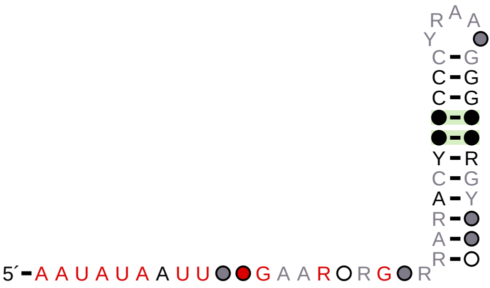

HMP2_18_1

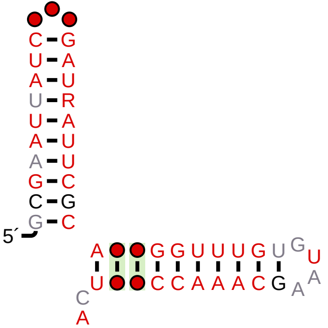

HMP2_1808_1

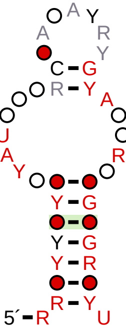

HMP2_1809_1

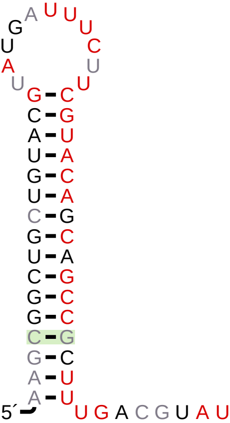

HMP2_1812_1

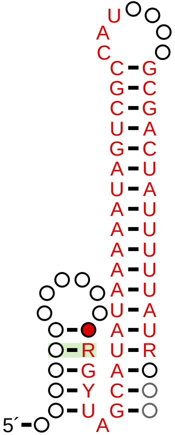

HMP2_1813_1

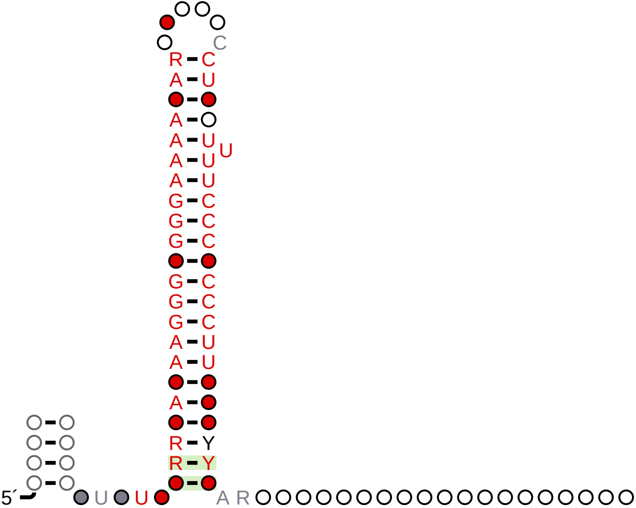

HMP2_1816_1

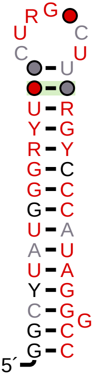

HMP2_1819_1

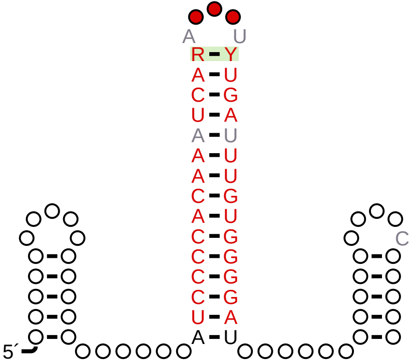

HMP2_1823_1

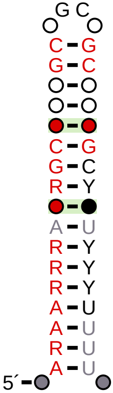

HMP2_1825_1

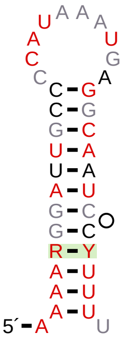

HMP2_1830_1

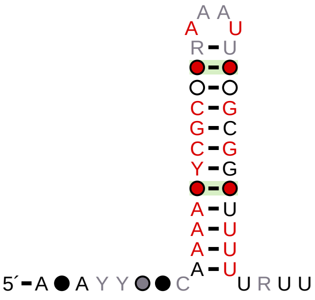

HMP2_1831_1

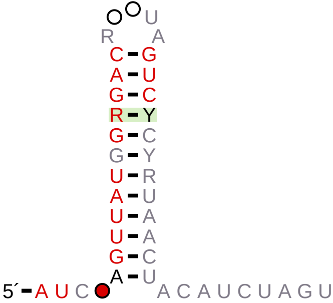

HMP2_1832_1

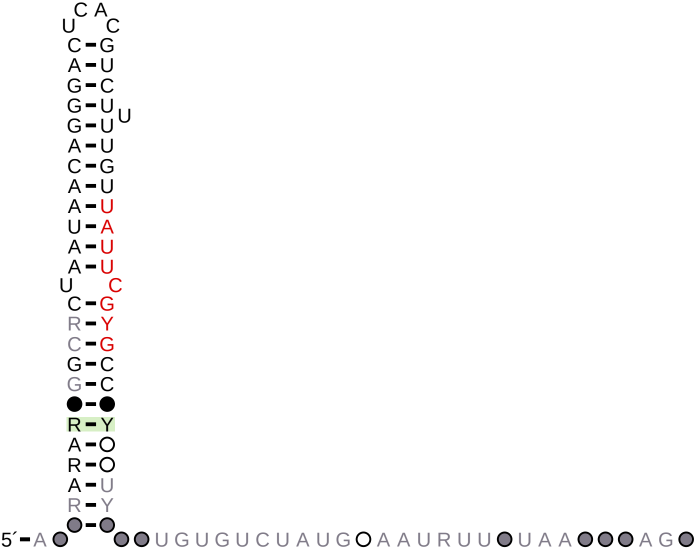

HMP2_1833_1

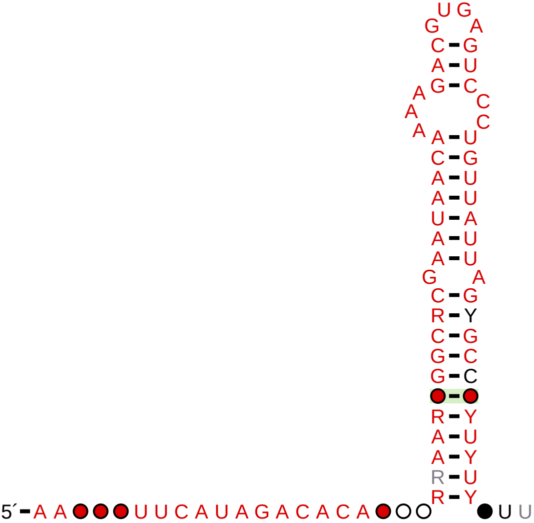

HMP2_1841_1

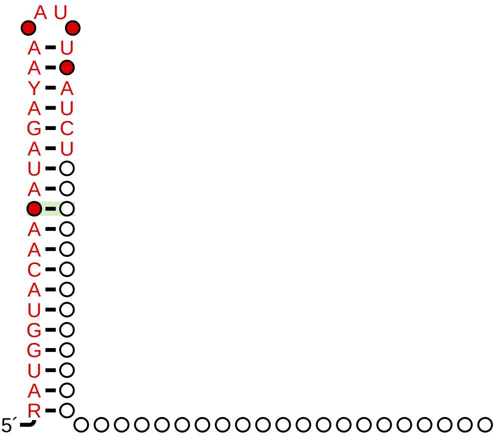

HMP2_1843_1

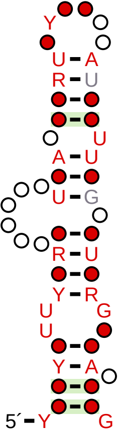

HMP2_1849_1

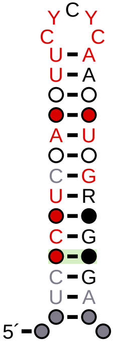

HMP2_1850_1

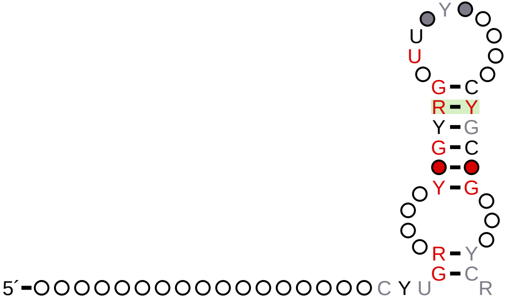

HMP2_1859_1

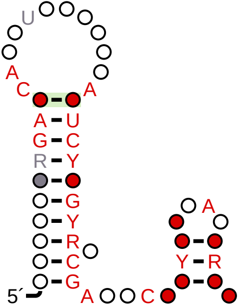

HMP2_1870_1

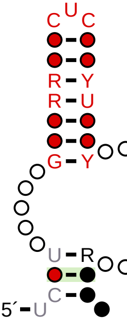

HMP2_1871_1

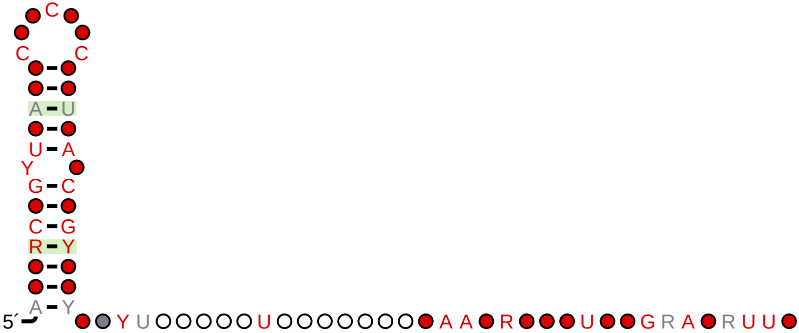

HMP2_1873_1

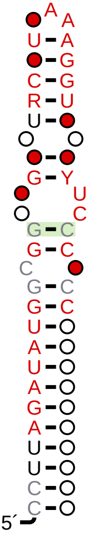

HMP2_1881_1

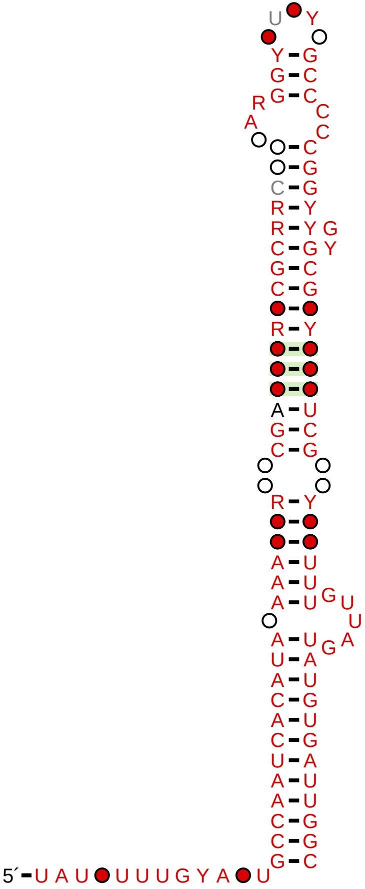

HMP2_1885_1

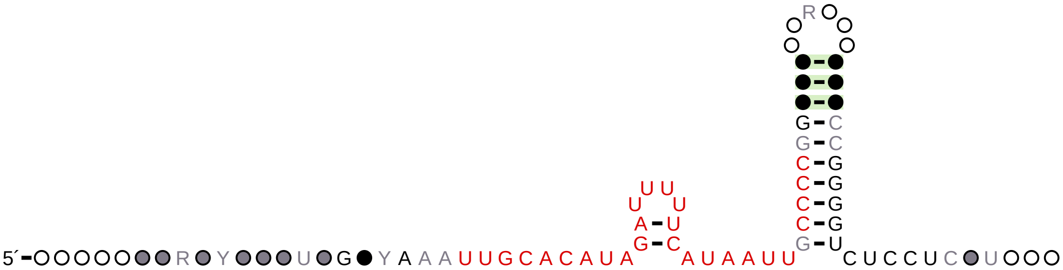
5´
R Y U G Y A A A U U G C A C A U A
G A U U U U U C
A U A A U U
G C C C C G G
R
C C G G G G U
C U C C U C U

HMP2_1889_1

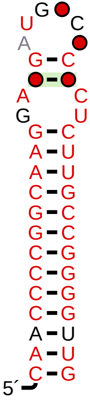

HMP2_1894_1

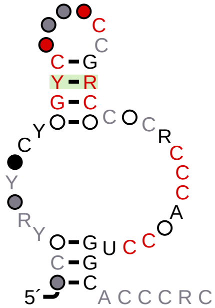

HMP2_1897_1

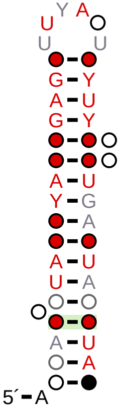

HMP2_19_1

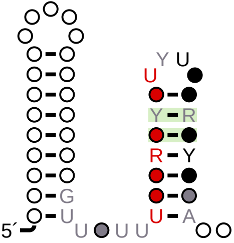

HMP2_1901_1

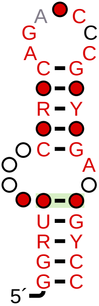

HMP2_1905_1

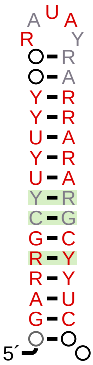

HMP2_1907_1

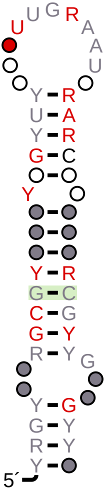

HMP2_1909_1

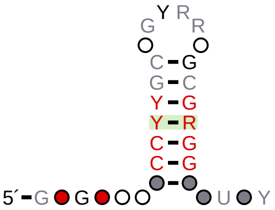

HMP2_1910_1

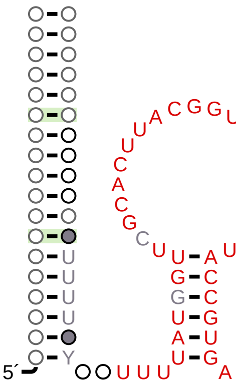

HMP2_1917_1

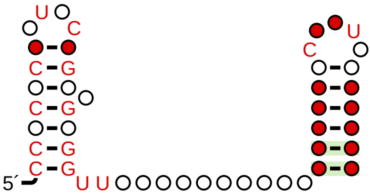

HMP2_1929_1

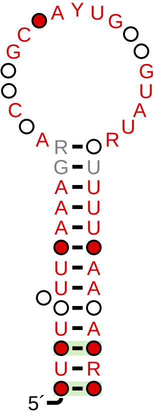

HMP2_1933_1

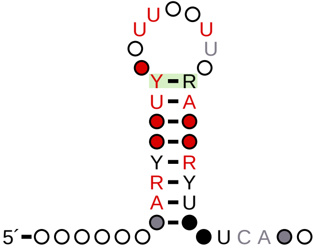

HMP2_1934_1

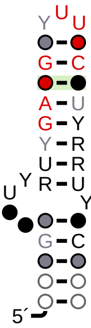

HMP2_1935_1

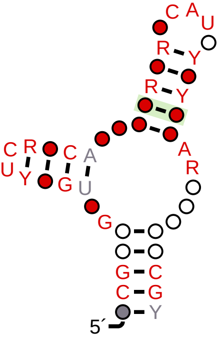

HMP2_1936_1

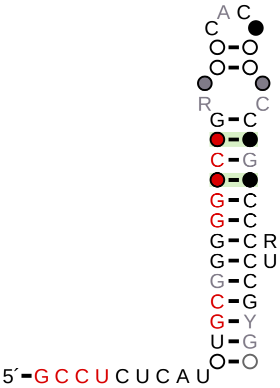

HMP2_1939_1

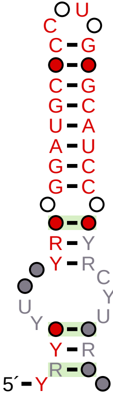

HMP2_194_1

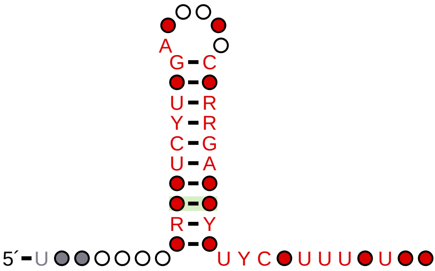

HMP2_1942_1

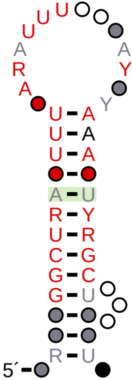

HMP2_1943_1

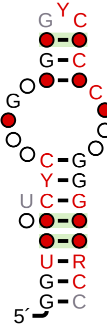

HMP2_1945_1

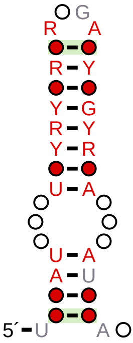

HMP2_1948_1

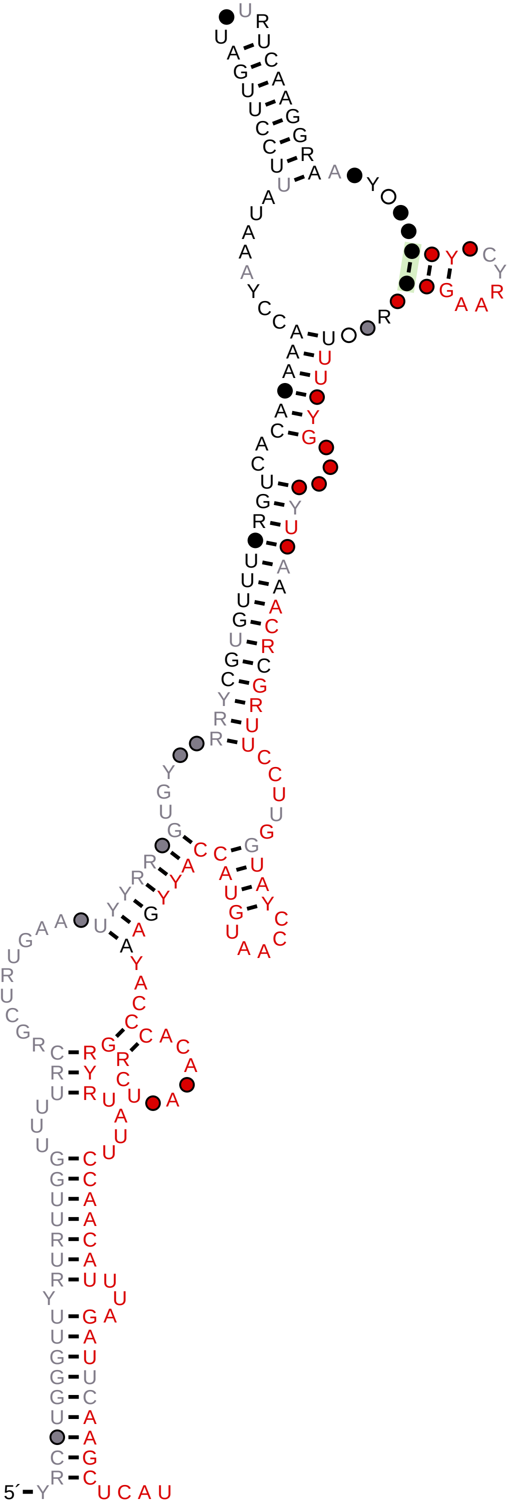

HMP2_1949_1

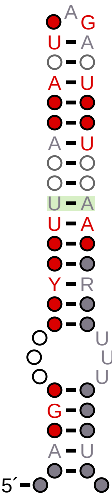

HMP2_1951_1

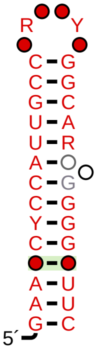

HMP2_1952_1

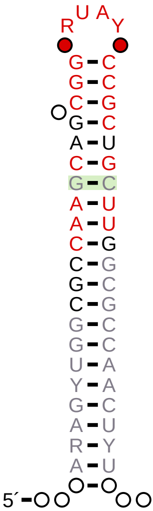

HMP2_1954_1

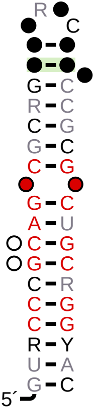

HMP2_1955_1

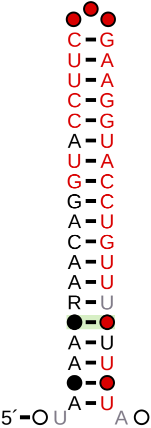

HMP2_1956_1

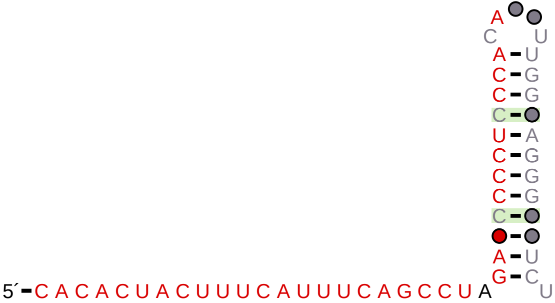

HMP2_1957_1

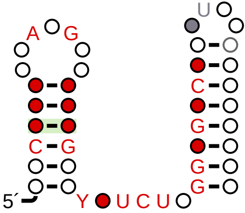

HMP2_1962_1

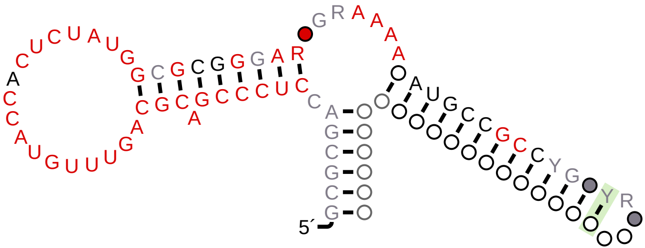

HMP2_1970_1

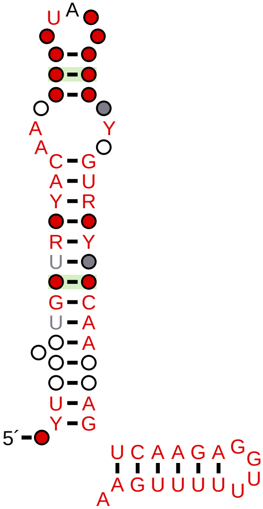

HMP2_1977_1

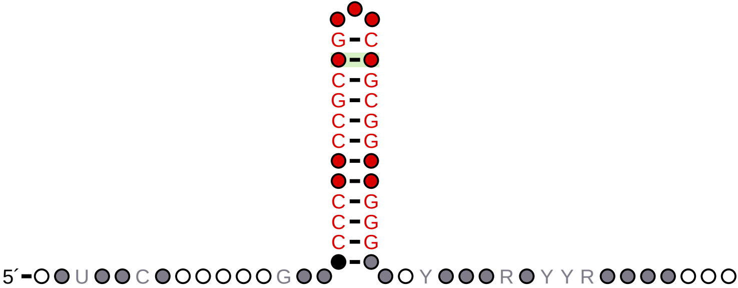

HMP2_1982_1

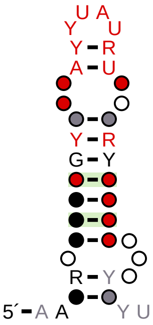

HMP2_1986_1

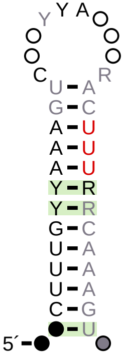

HMP2_1987_1

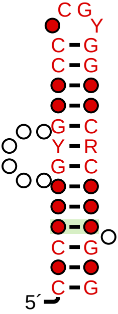

HMP2_1988_1

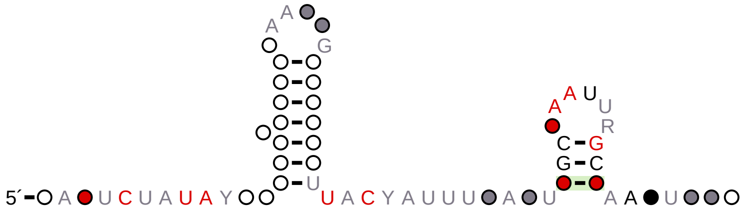

HMP2_1991_1

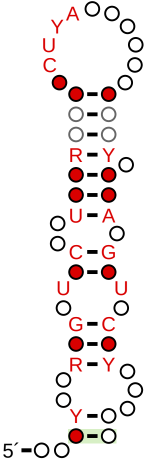

HMP2_1992_1

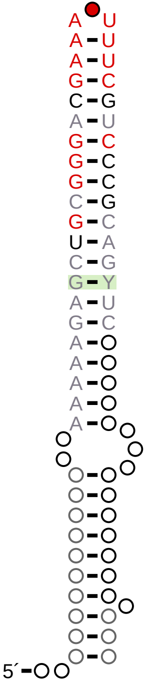

HMP2_1994_1

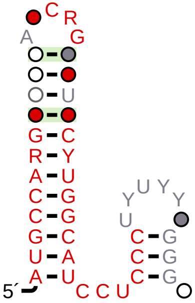

HMP2_1995_1

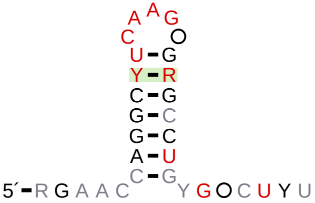

HMP2_2002_1

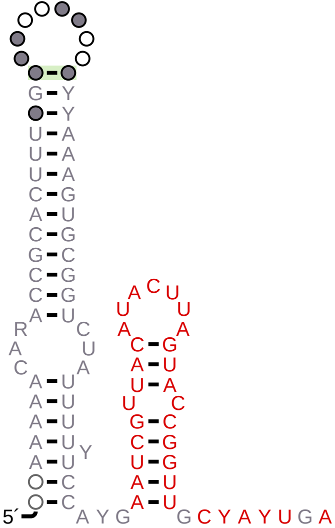

HMP2_2008_1

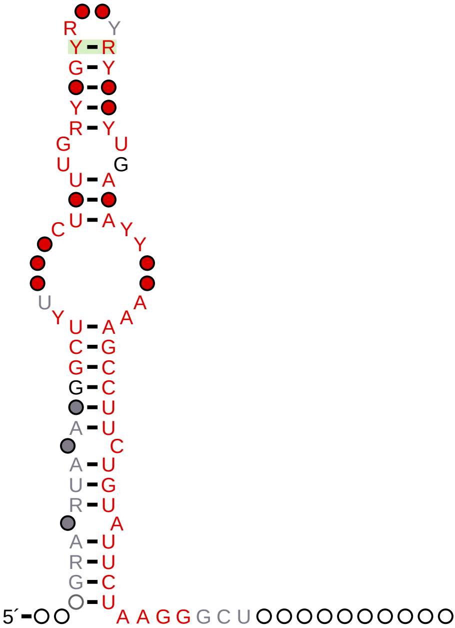

HMP2_2015_1

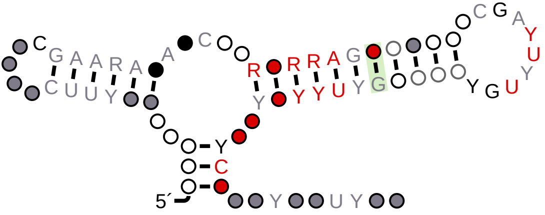

HMP2_2016_1

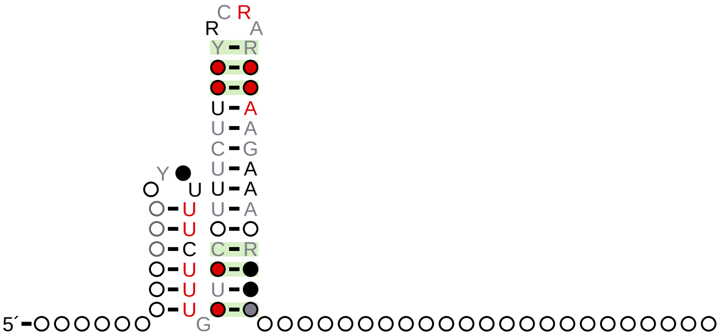

HMP2_2019_1

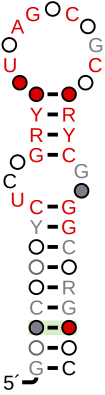

HMP2_2020_1

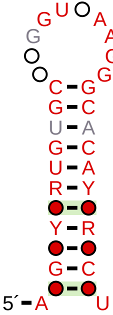

HMP2_2024_1

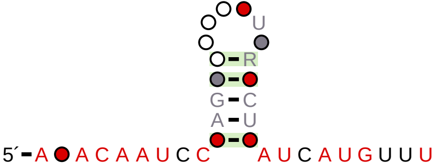

HMP2_2026_1

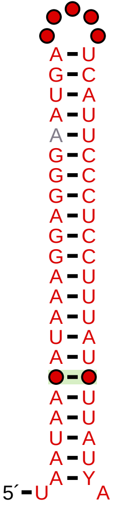

HMP2_2027_1

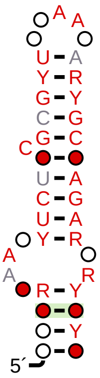

HMP2_2028_1

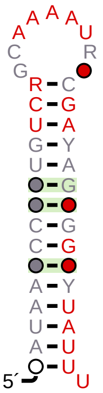

HMP2_2029_1

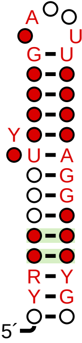

HMP2_2037_1

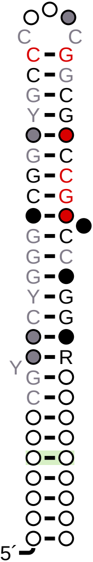

HMP2_2041_1

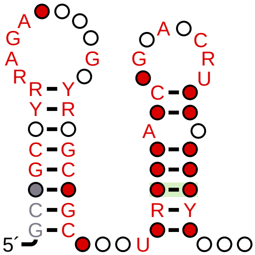

HMP2_2042_1

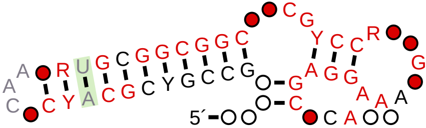

HMP2_2043_1

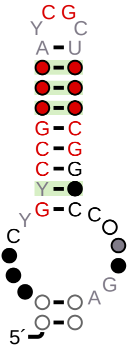

HMP2_205_1

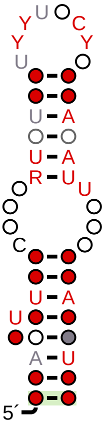

HMP2_2055_1

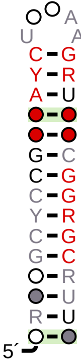

HMP2_2058_1

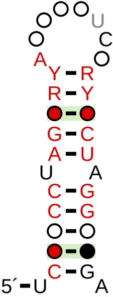

HMP2_2059_1

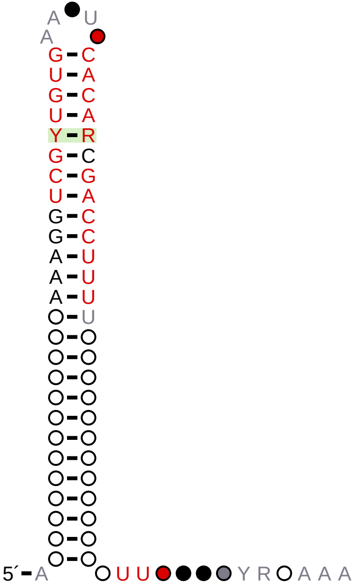

HMP2_2060_1

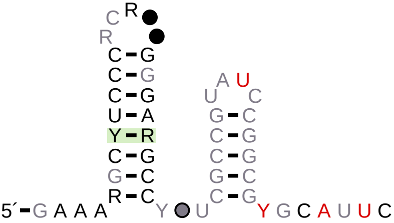

HMP2_207_1

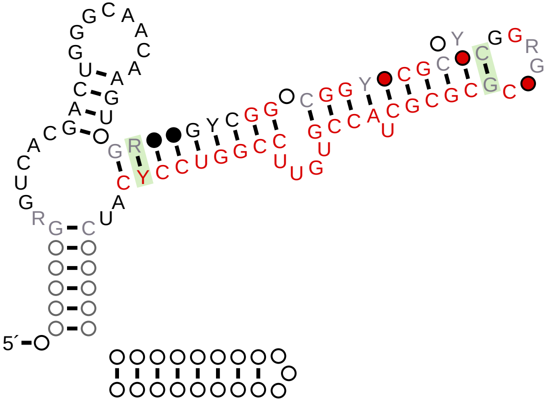

HMP2_2076_1

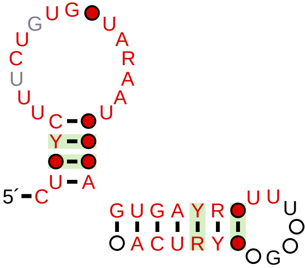

HMP2_2077_1

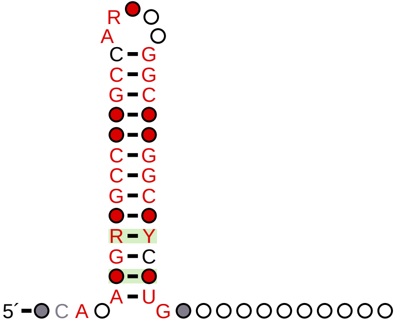

HMP2_2078_1

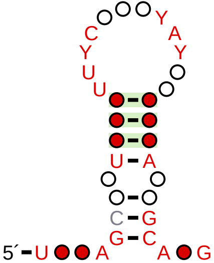

HMP2_2080_1

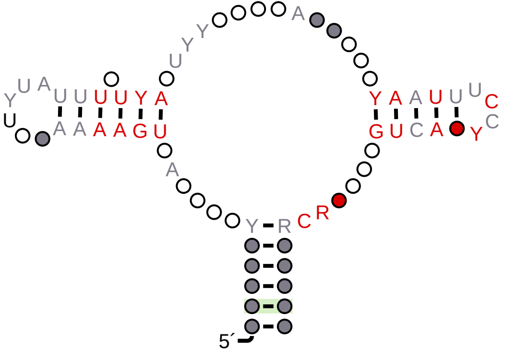

HMP2_2089_1

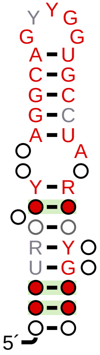

HMP2_2094_1

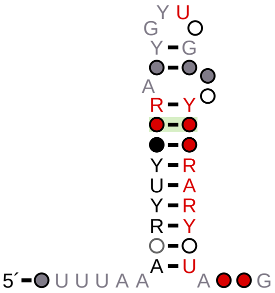

HMP2_2100_1

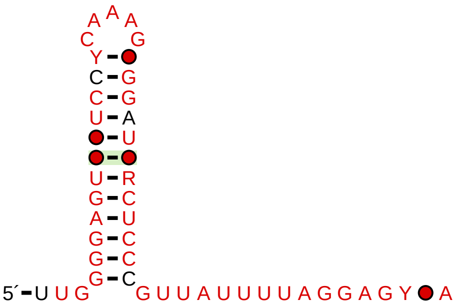

HMP2_2103_1

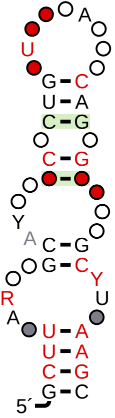

HMP2_2104_1

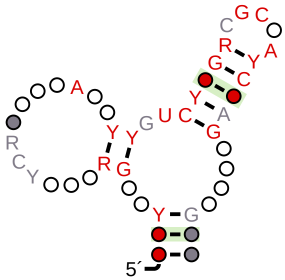

HMP2_2105_1

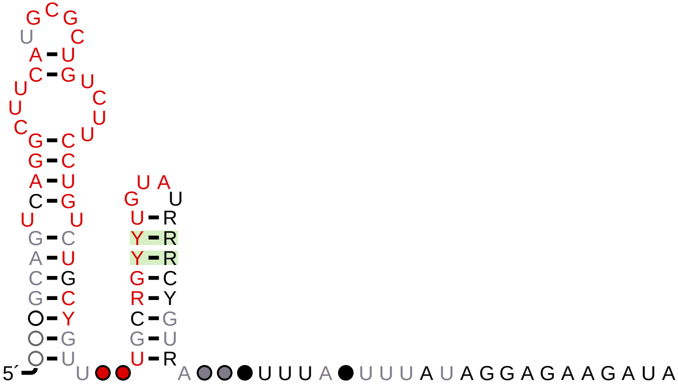

HMP2_2108_1

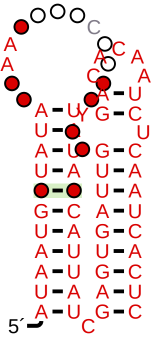
C
A
A
A
C
A
A
C
A
U
A
U
Y
G
C
U
U
A
U
G
C
U
A
U
A
U
A
G
C
A
U
U
A
G
C
U
A
U
A
G
C
A
U
U
A
G
C
U
A
A
U
A
U
A
U
G
C
5´
C

HMP2_2112_1

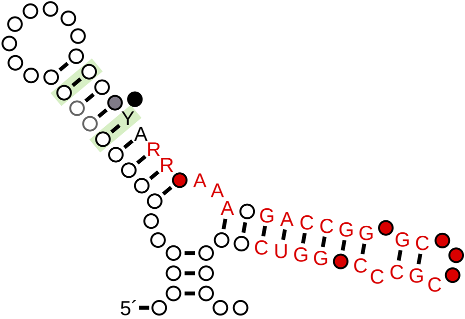

HMP2_2119_1

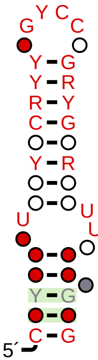

HMP2_2131_1

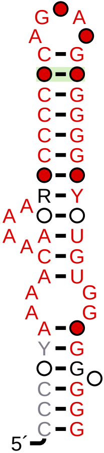

HMP2_2136_1

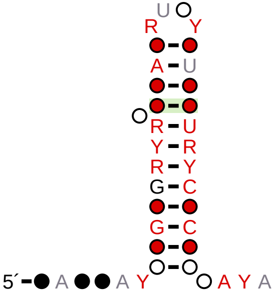

HMP2_2163_1

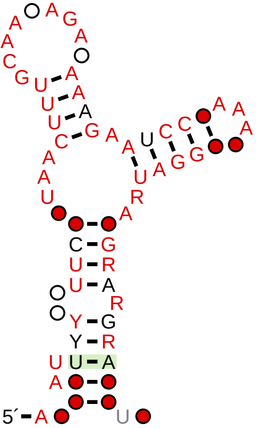

HMP2_2169_1

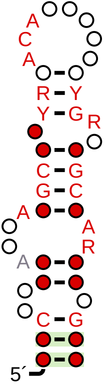

HMP2_2176_1

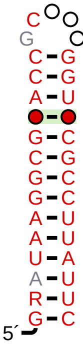

HMP2_2179_1

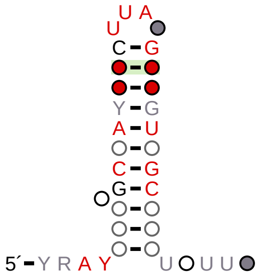

HMP2_2180_1

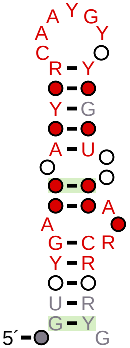

HMP2_2187_1

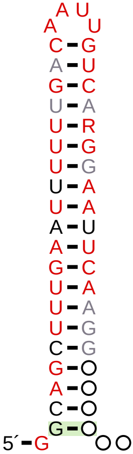

HMP2_2190_1

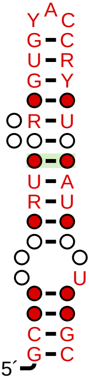

HMP2_2202_1

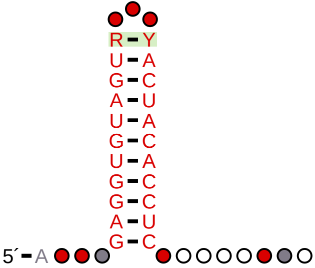

HMP2_2203_1

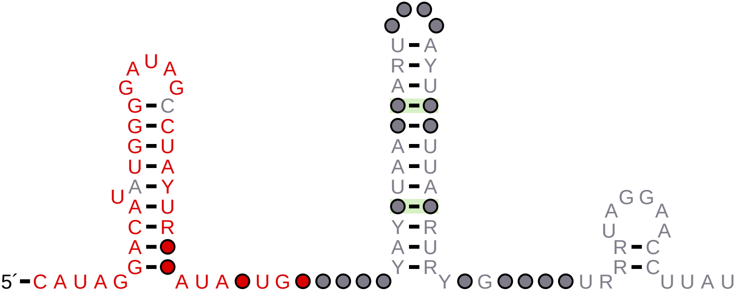

HMP2_2204_1

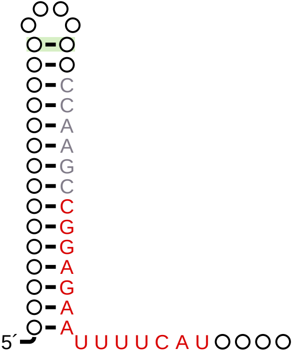

HMP2_2210_1

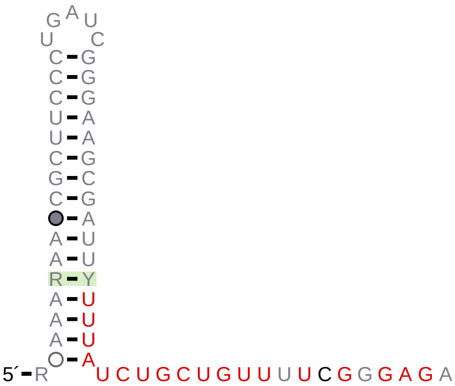

G A U
U C
C G
C G
C G
U A
U A
C G
G C
C G
A
A U
A U
R Y
A U
A U
A U
A
5´ R
U C U G C U G U U U U C G G G A G A

HMP2_2217_1

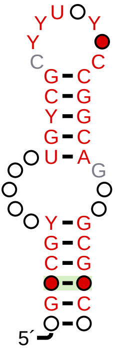

HMP2_2233_1

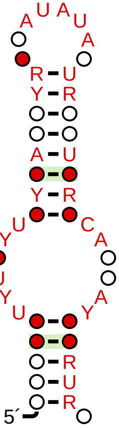

HMP2_2235_1

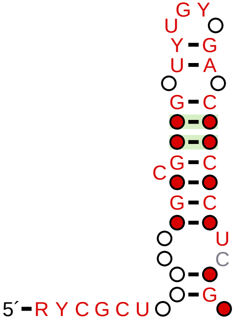

HMP2_2236_1

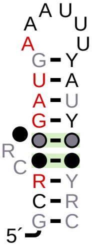

HMP2_2237_1

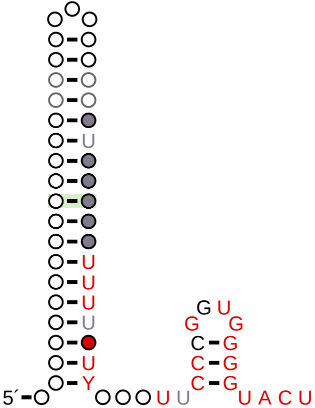

HMP2_2239_1

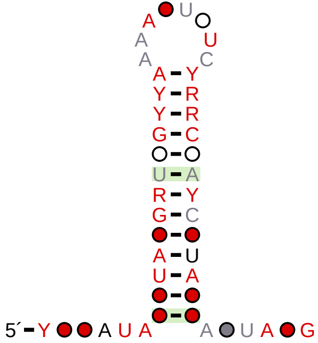

HMP2_224_1

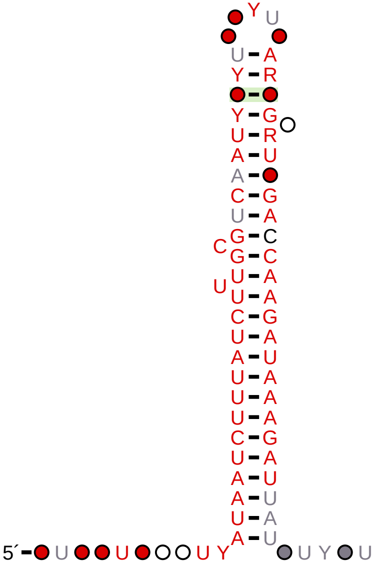

Y
U
U A
Y R
Y G
U R
A U
A
C G
U A
G C
C
G C
U
U A
U A
C G
U A
A U
U A
U A
U A
C G
U A
A U
A U
U A
A U
5´ U U U Y
U Y U

HMP2_2240_1

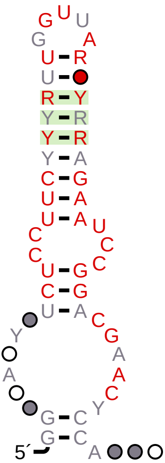

HMP2_2249_1

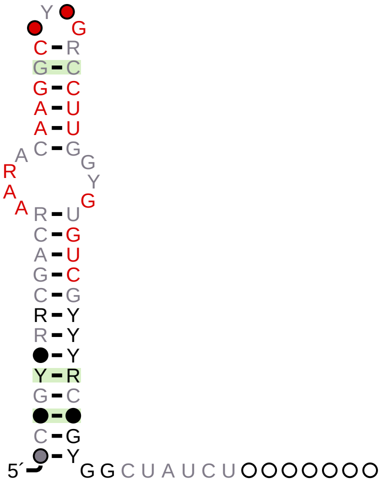

HMP2_2265_1

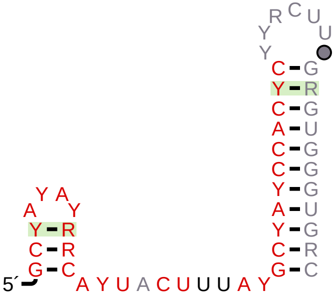

HMP2_2277_1

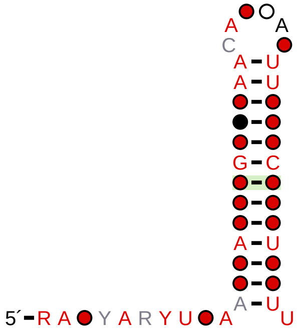

HMP2_2278_1

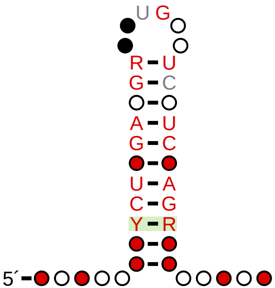

HMP2_228_1

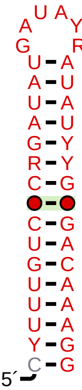

HMP2_2282_1

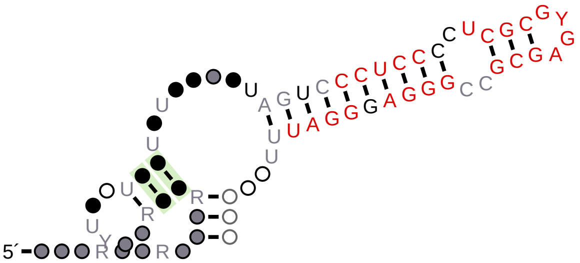

HMP2_2286_1

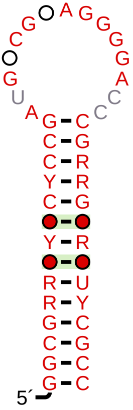

HMP2_229_1

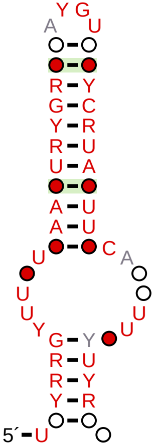

HMP2_2290_1

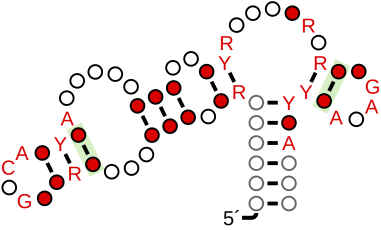

HMP2_2293_1

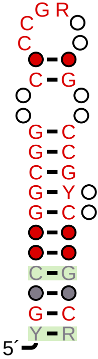

HMP2_2297_1

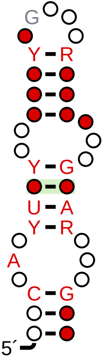

HMP2_2315_1

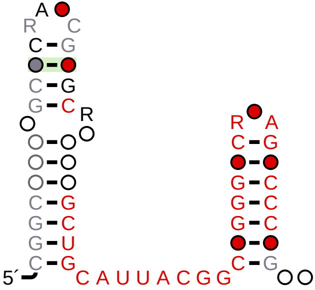

HMP2_2317_1

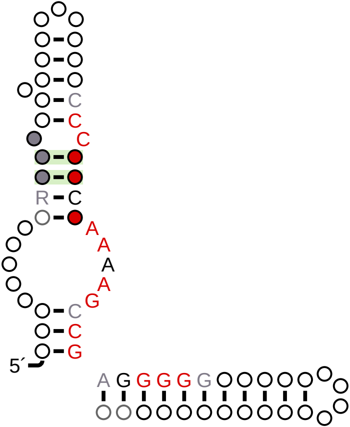

HMP2_2318_1

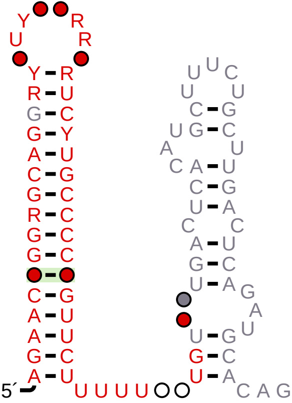

HMP2_232_1

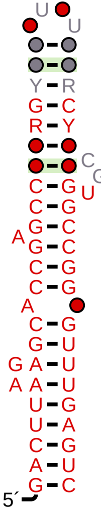

HMP2_2321_1

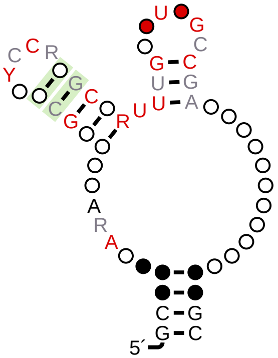

HMP2_2332_1

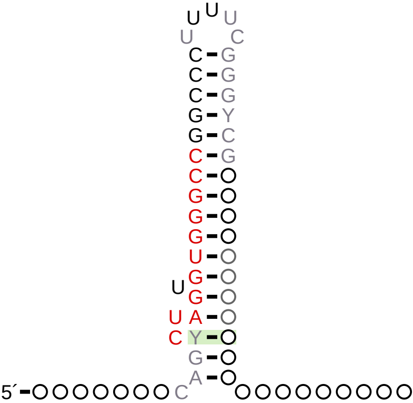

HMP2_2336_1

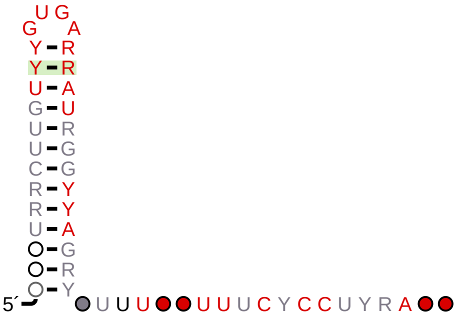

HMP2_2337_1

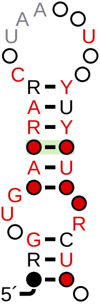

HMP2_2339_1

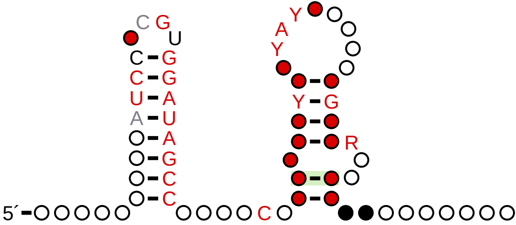

HMP2_2342_1

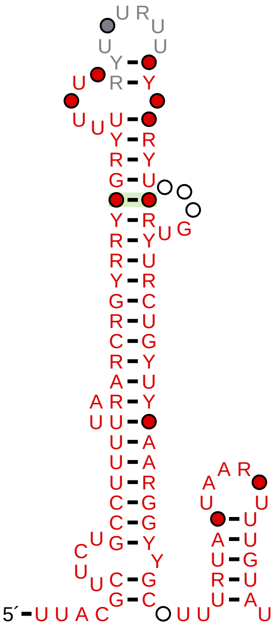

HMP2_2343_1

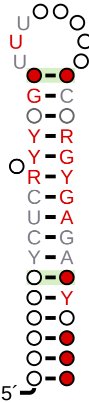

HMP2_2345_1

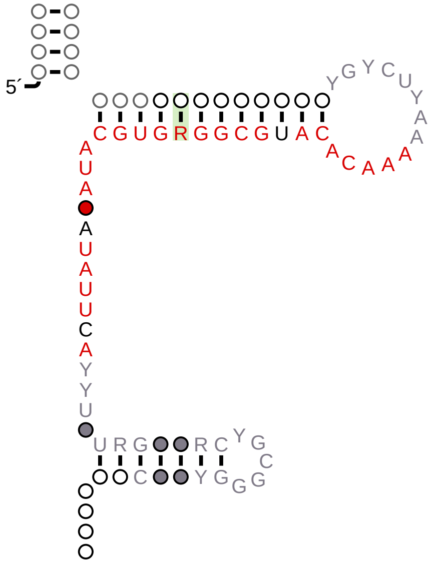

5´
CGUGRGGCGUAC
YGYCUYAA
ACAAAA
AUA
AUAUUCAYYU
URGRCYGC
CYGGG

HMP2_2347_1

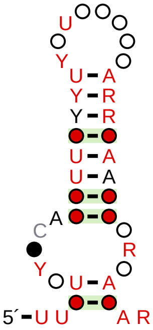

HMP2_2350_1

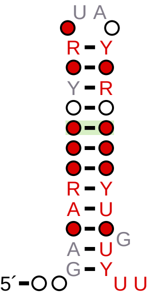

HMP2_2352_1

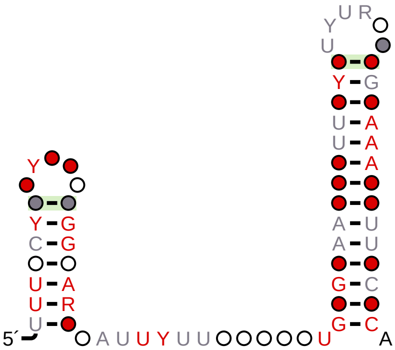

HMP2_2353_1

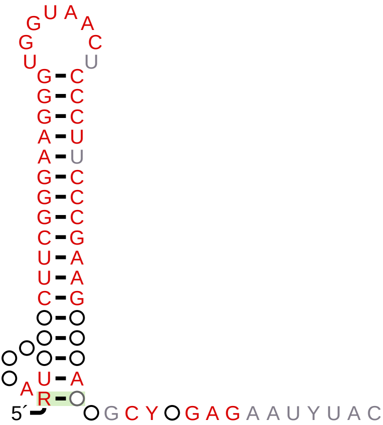

G U A A C G U U
G C
G C
G C
A U
A U
G C
G C
G C
C G
U A
U A
C G
A U
A R
5´
G C Y G A G A A U Y U A C

HMP2_2354_1

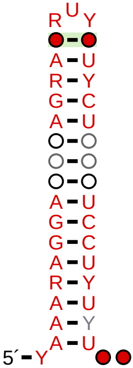

HMP2_2359_1

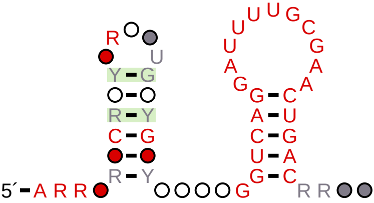

HMP2_2361_1

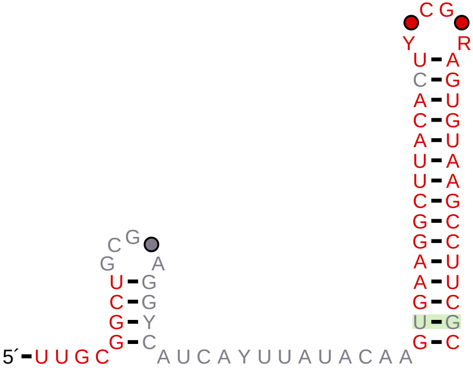

C G
Y R
U A
C G
A U
C G
A U
U A
U A
C G
G C
G C
A U
A U
G C
U G
G C
C G
G A
U G
C G
G Y
G C
5´ U U G C
A U C A Y U U A U A C A A

HMP2_2363_1

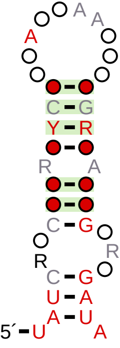

HMP2_2364_1

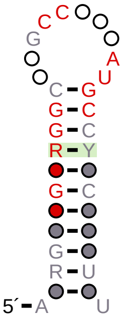

HMP2_2367_1

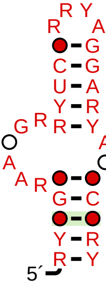

HMP2_2370_1

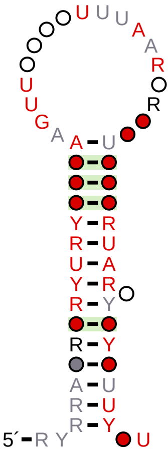

HMP2_2373_1

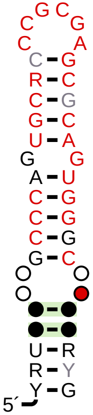

HMP2_238_1

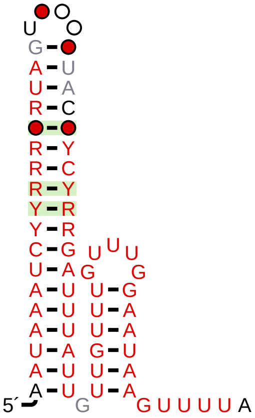

HMP2_2381_1

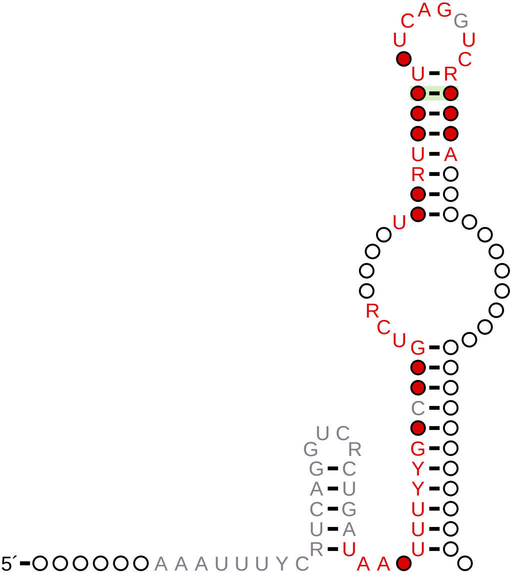

HMP2_2384_1

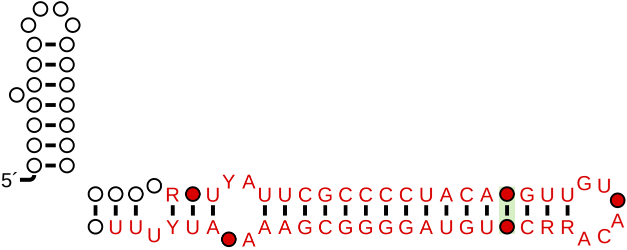

HMP2_2385_1

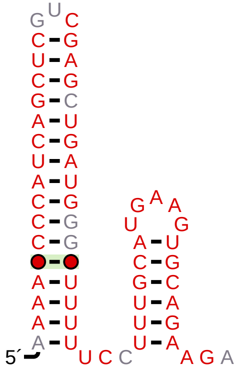

HMP2_239_1

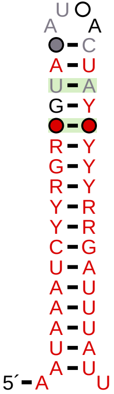

HMP2_2391_1

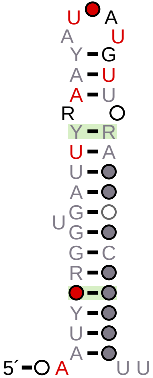

HMP2_2403_1

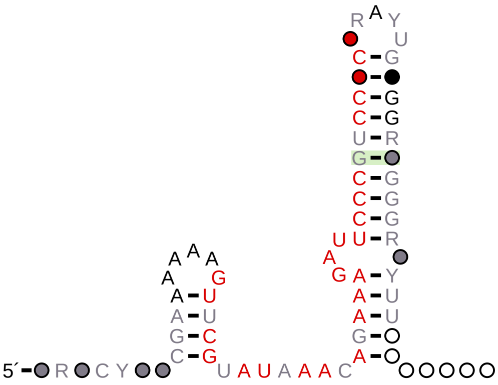

HMP2_2404_1

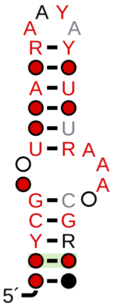

HMP2_2406_1

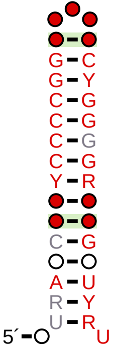

HMP2_2409_1

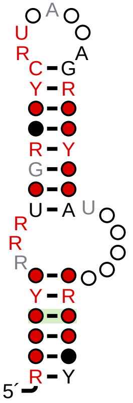

HMP2_2412_1

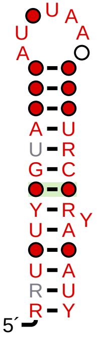

HMP2_2413_1

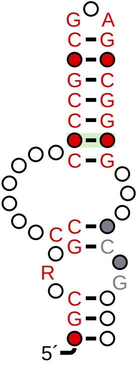

HMP2_2416_1

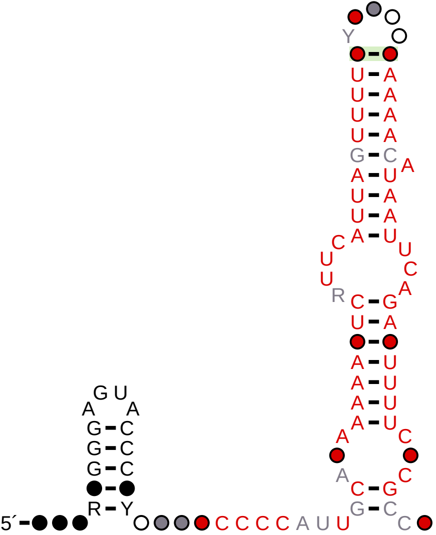

HMP2_2419_1

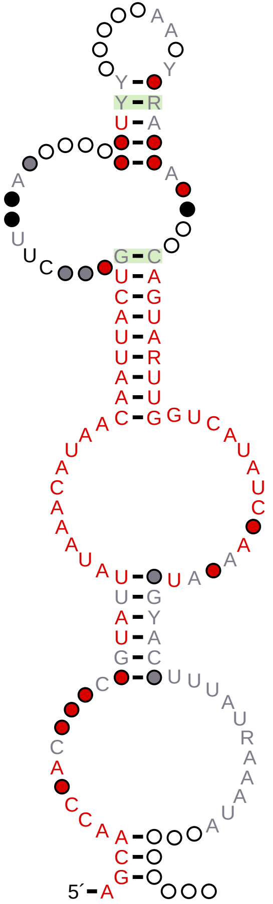

HMP2_242_1

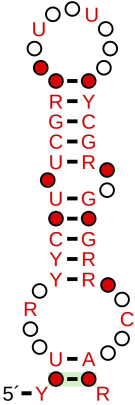

HMP2_2421_1

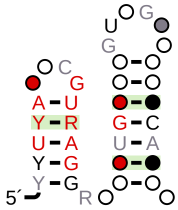

HMP2_2426_1

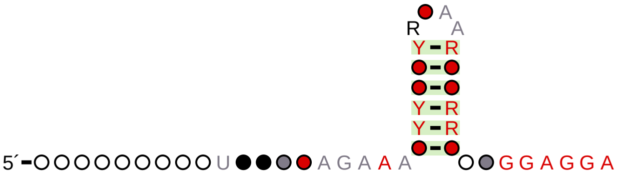

HMP2_2429_1

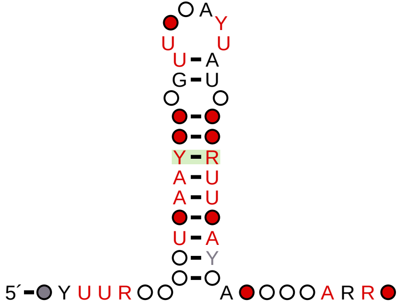

HMP2_2439_1

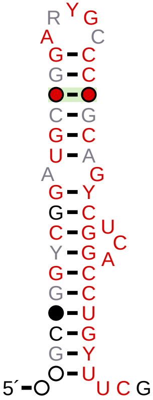

HMP2_2451_1

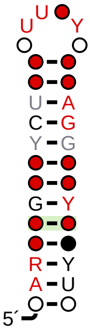

HMP2_2452_1

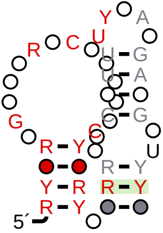

HMP2_2457_1

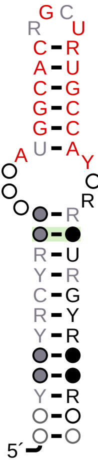

HMP2_2458_1

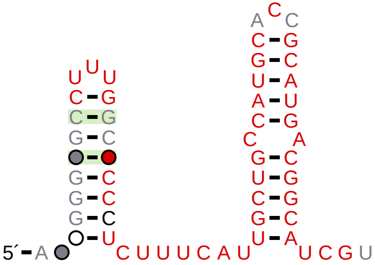

HMP2_246_1

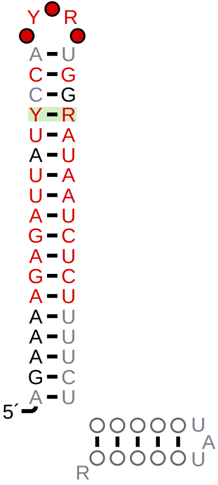

HMP2_2464_1

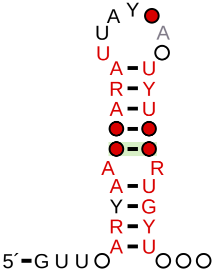

HMP2_2465_1

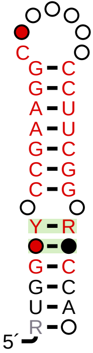

HMP2_2474_1

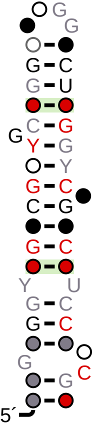

HMP2_2483_1

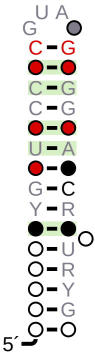

HMP2_2485_1

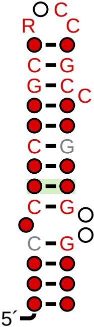

HMP2_2486_1

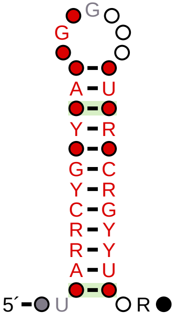

HMP2_2489_1

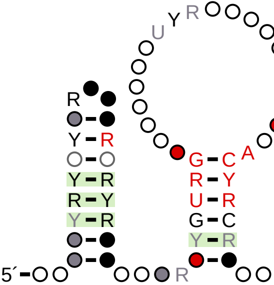

HMP2_2490_1

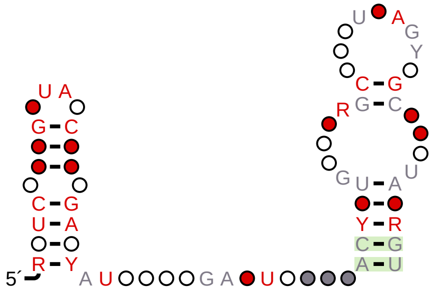

HMP2_2495_1

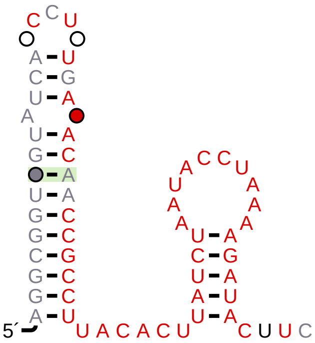

HMP2_2498_1

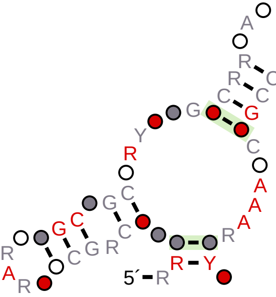

HMP2_25_1

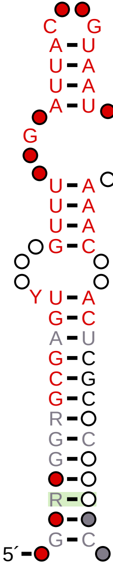

HMP2_2501_1

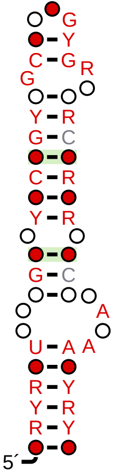

HMP2_2504_1

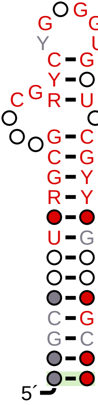

HMP2_2506_1

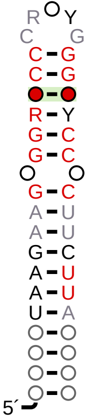

HMP2_2507_1

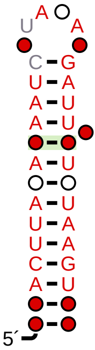

HMP2_252_1

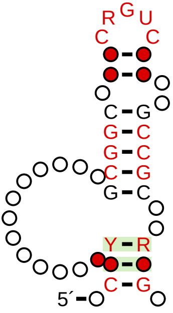

HMP2_2522_1

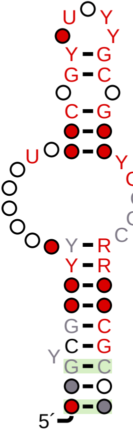

HMP2_2531_1

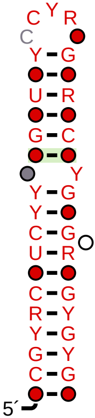

HMP2_2537_1

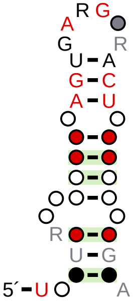

HMP2_2539_1

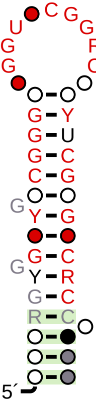

HMP2_254_1

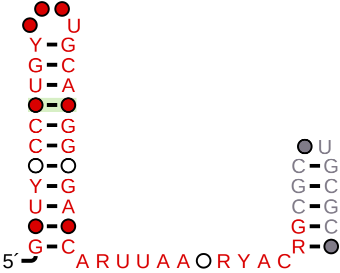

HMP2_2541_1

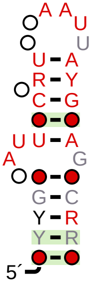

HMP2_2545_1

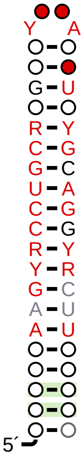

HMP2_255_1

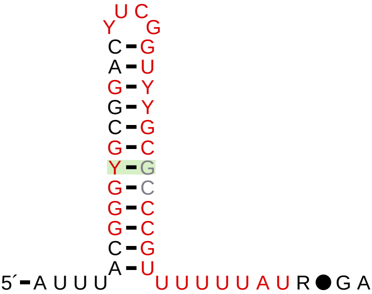

HMP2_2559_1

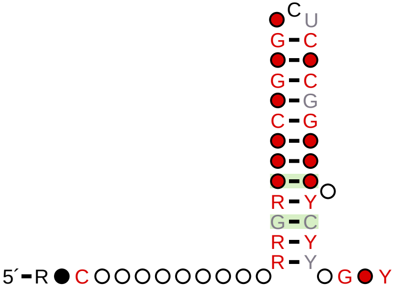

HMP2_256_1

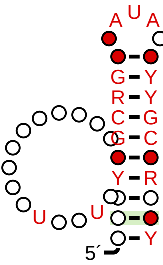

HMP2_2565_1

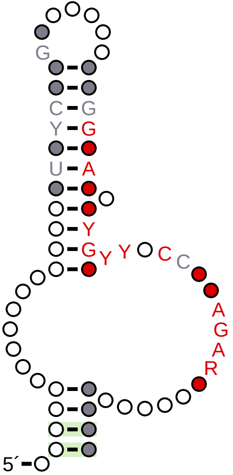

HMP2_2568_1

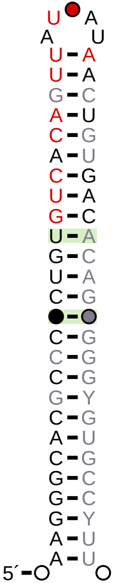

HMP2_2569_1

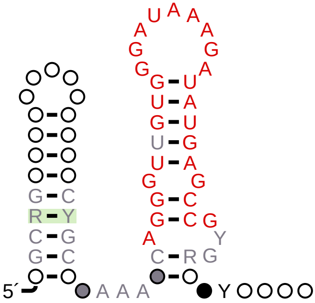

HMP2_2570_1

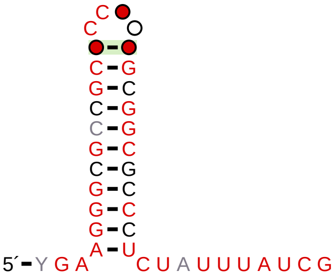

HMP2_2577_1

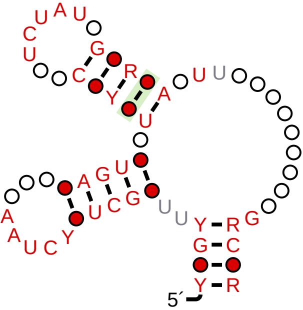

HMP2_2579_1

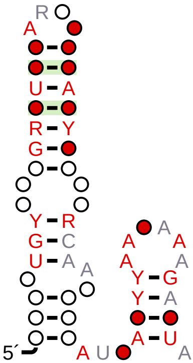

HMP2_2583_1

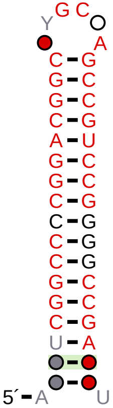

HMP2_2585_1

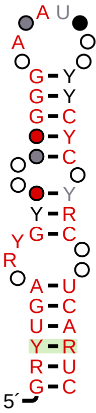

HMP2_2586_1

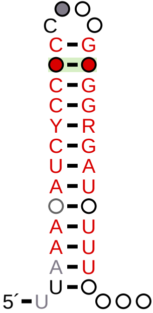

HMP2_2587_1

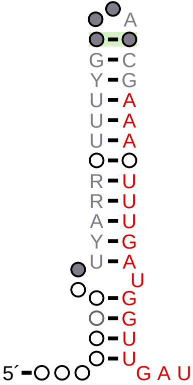

HMP2_2589_1

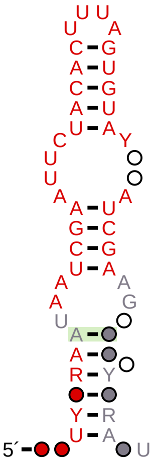

HMP2_2590_1

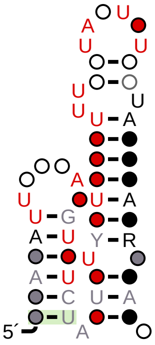

HMP2_2591_1

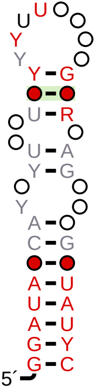

HMP2_2593_1

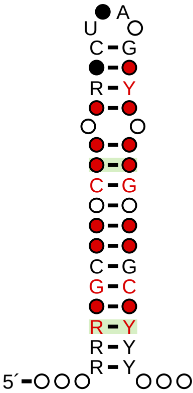

HMP2_2594_1

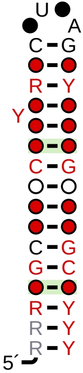

HMP2_2598_1

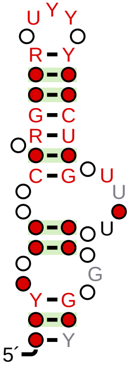

HMP2_2599_1

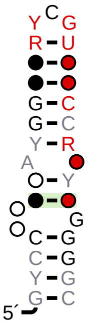

HMP2_26_1

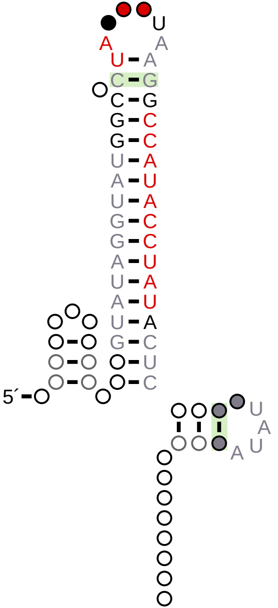

HMP2_2602_1

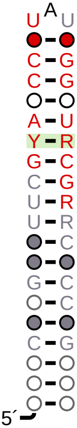

HMP2_2608_1

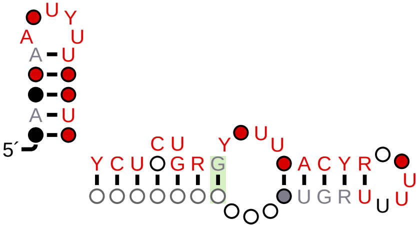

HMP2_2615_1

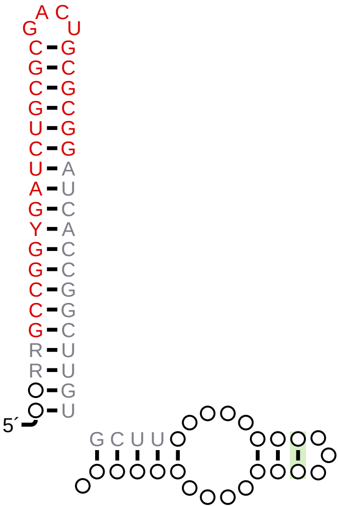

HMP2_2620_1

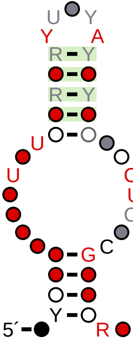

HMP2_263_1

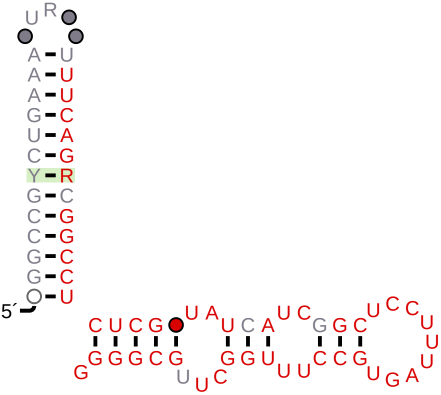

U R
A U
A U
A U
G C
U A
C G
Y R
G C
C G
C G
G C
G C
U
5′
C U C G U A U C A U C G G C U C C U
G G G C G G G U U U C C G U G A U U U
G U U C

HMP2_2631_1

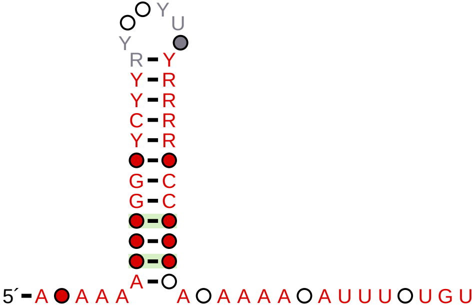

HMP2_2633_1

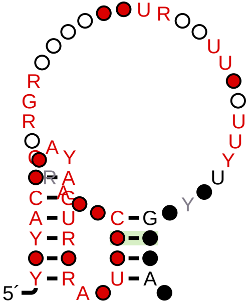

HMP2_2634_1

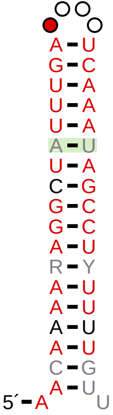

HMP2_264_1

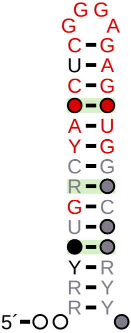

HMP2_2645_1

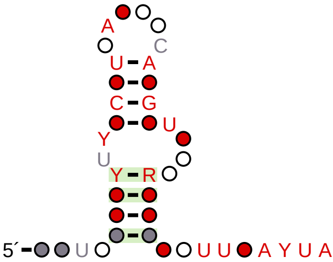

HMP2_2646_1

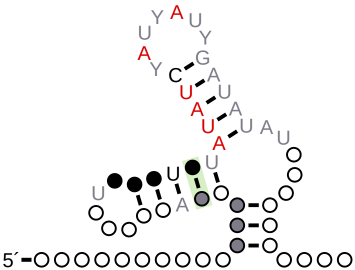

HMP2_2648_1

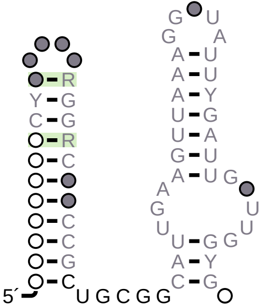

HMP2_2658_1

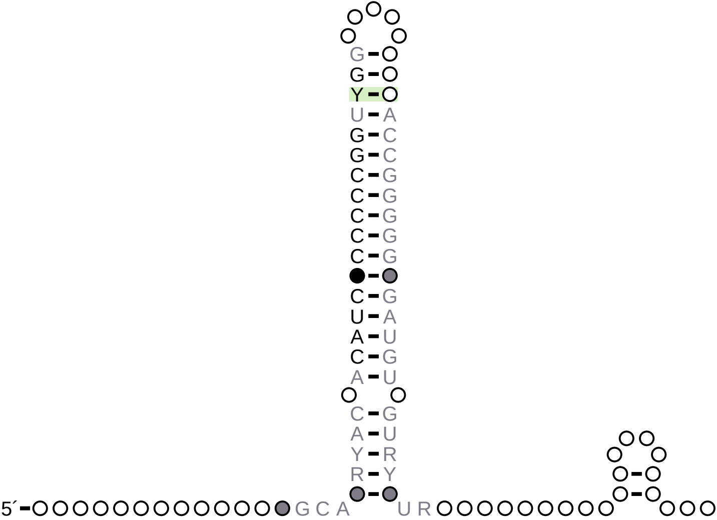

HMP2_2660_1

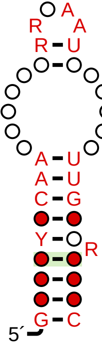

HMP2_2665_1

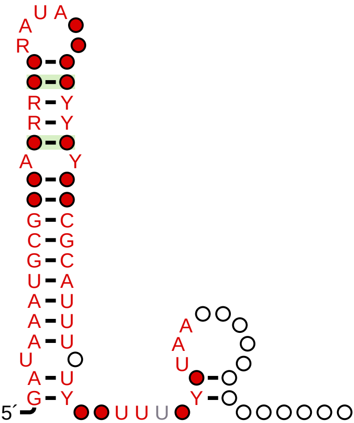

HMP2_2670_1

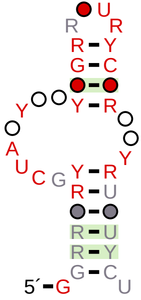

HMP2_2687_1

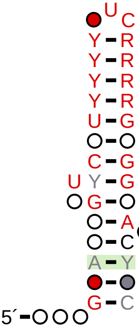

HMP2_269_1

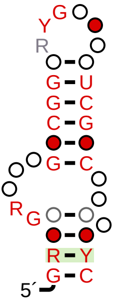

HMP2_270_1

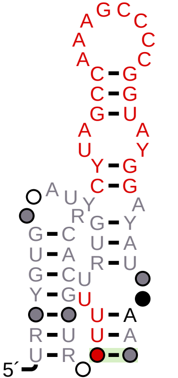

HMP2_2702_1

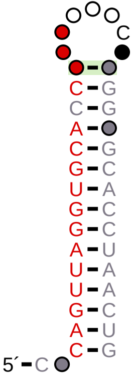

HMP2_2705_1

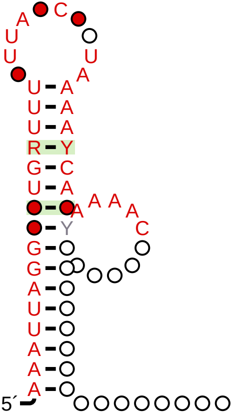

HMP2_2713_1

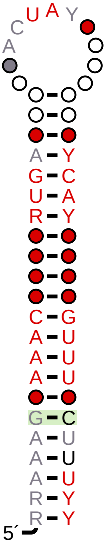

HMP2_2717_1

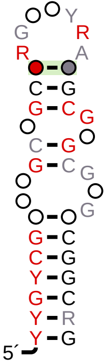

HMP2_2718_1

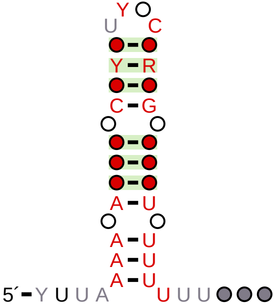

HMP2_2725_1

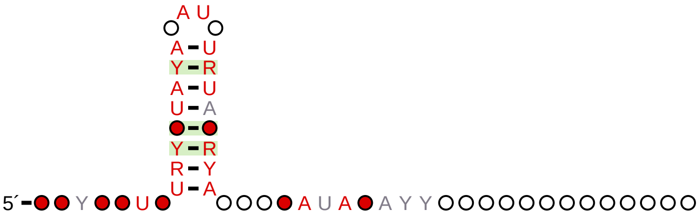

HMP2_2727_1

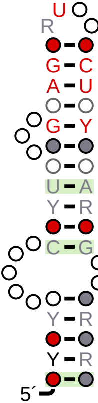

HMP2_2738_1

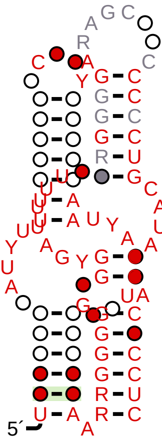

HMP2_2742_1

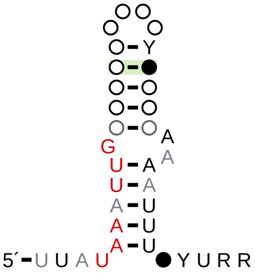

HMP2_2744_1

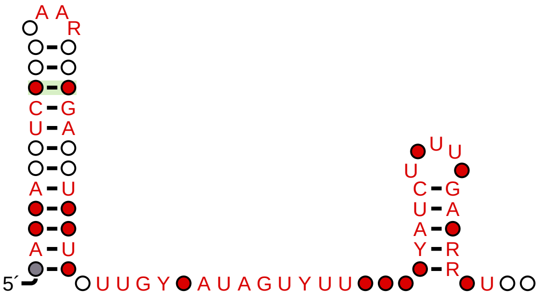

HMP2_2746_1

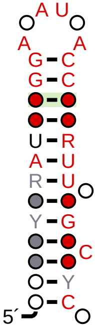

HMP2_2749_1

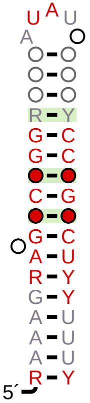

HMP2_275_1

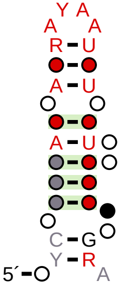

HMP2_2754_1

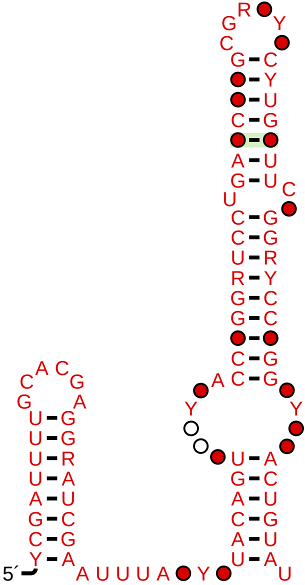

HMP2_2763_1

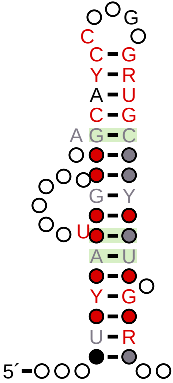

HMP2_2766_1

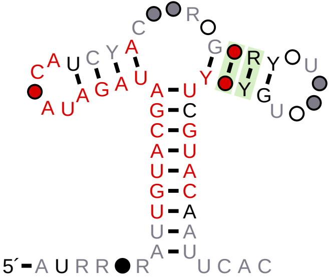

HMP2_2769_1

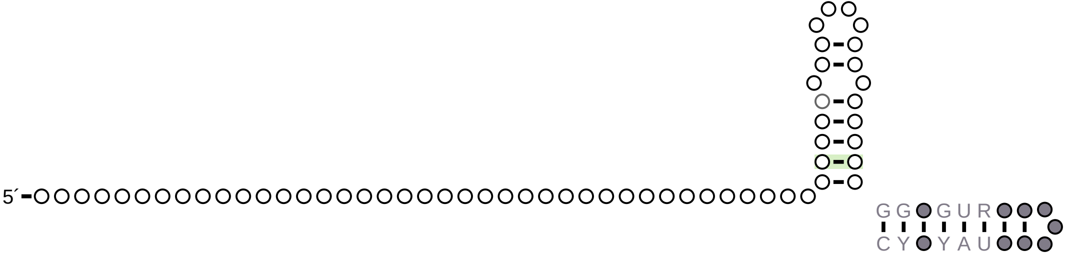

HMP2_2770_1

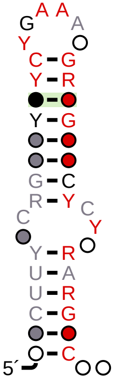

HMP2_2771_1

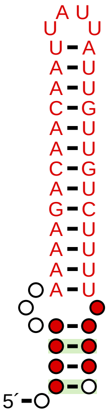

HMP2_2774_1

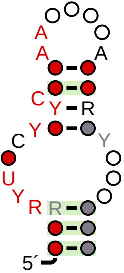

HMP2_2775_1

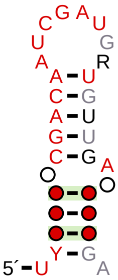

HMP2_2798_1

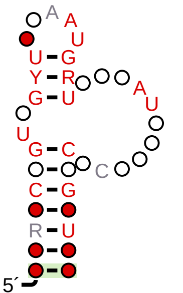

HMP2_2800_1

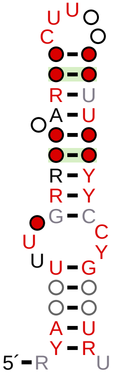

HMP2_2807_1

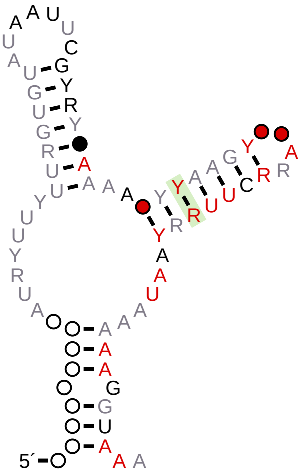

HMP2_2808_1

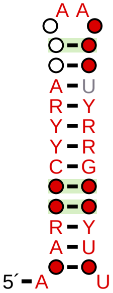

HMP2_2809_1

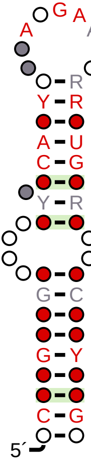

HMP2_281_1

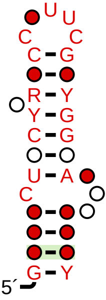

HMP2_2815_1

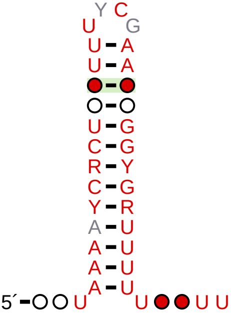

HMP2_2819_1

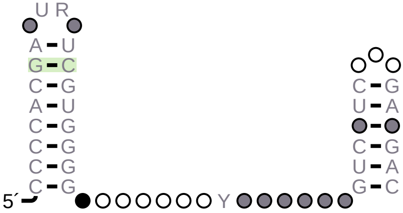

HMP2_2821_1

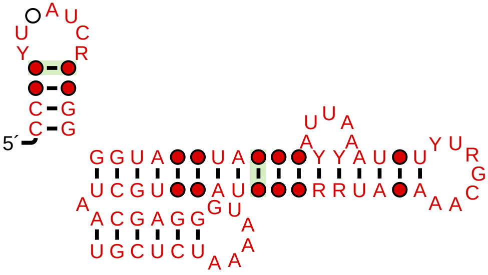

HMP2_2826_1

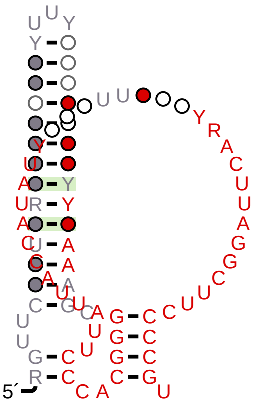

HMP2_2828_1

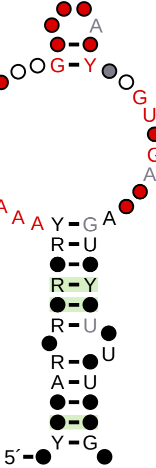

HMP2_2834_1

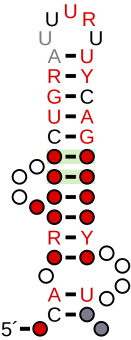

HMP2_2835_1

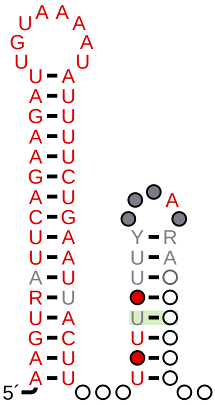

UAAAAU
GU
U-A
A-U
G-U
A-U
A-U
G-C
A-U
C-G
U-A
U-A
A-U
R-U
U-A
G-C
A-U
A-U
5´
A
Y-R
U-A
U
U
U
U

HMP2_2837_1

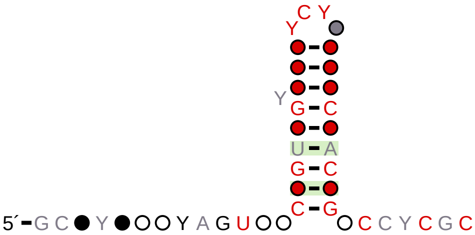

HMP2_2844_1

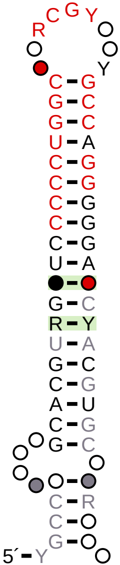

HMP2_2850_1

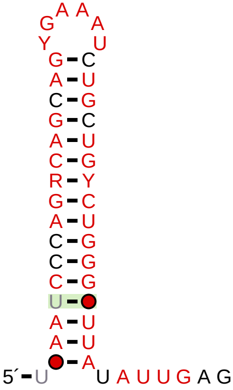

HMP2_2854_1

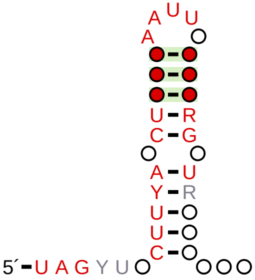

HMP2_2857_1

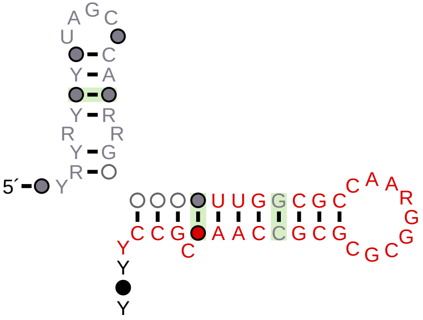

HMP2_286_1

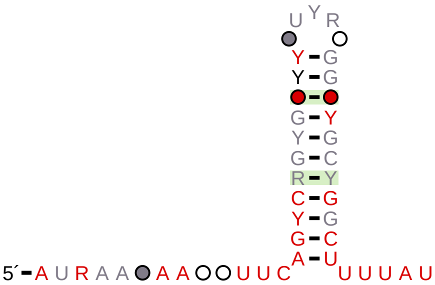

HMP2_2874_1

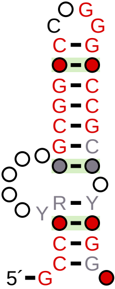

HMP2_2875_1

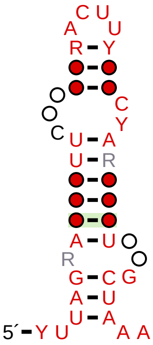

HMP2_288_1

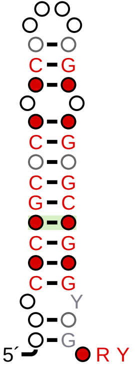

HMP2_2881_1

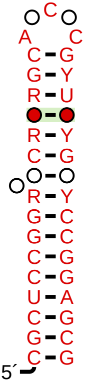

HMP2_2884_1

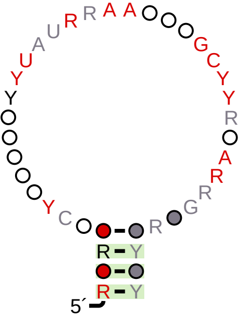

HMP2_2885_1

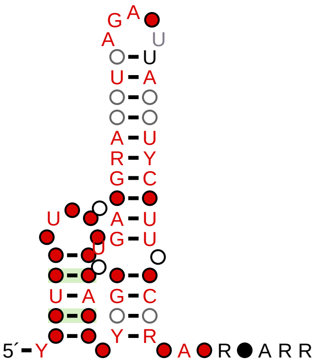

HMP2_289_1

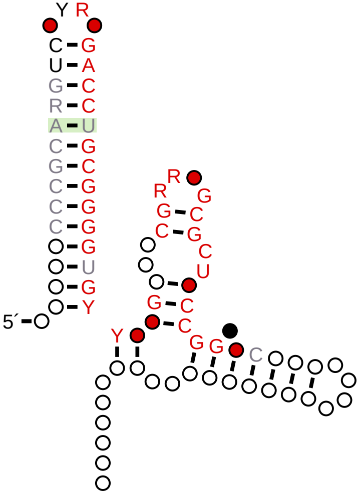

HMP2_2899_1

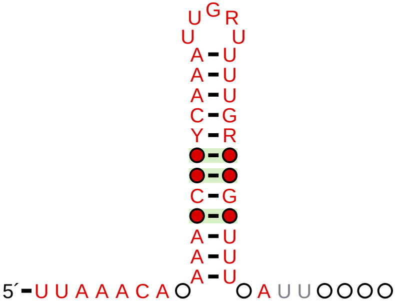

HMP2_29_1

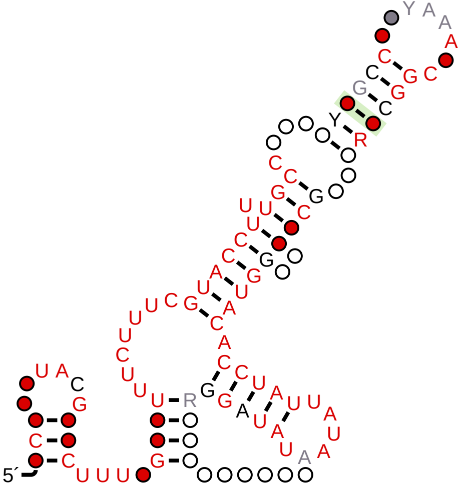

HMP2_2906_1

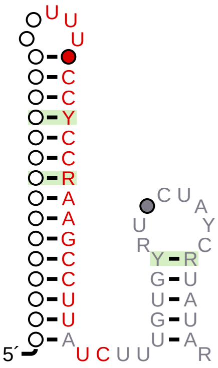

HMP2_2911_1

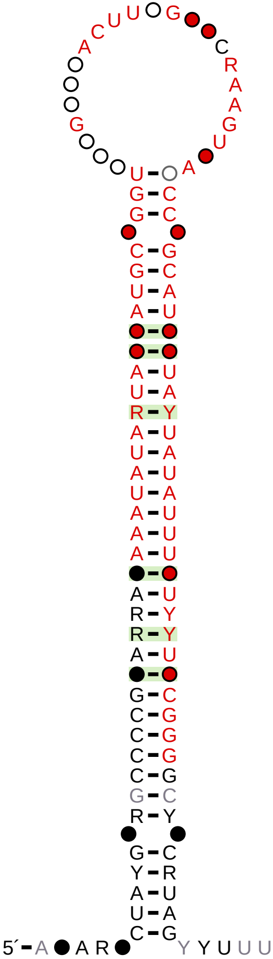
A C U U G
C R A A G U A
G
U - G - G - C - G - U - A
C - C - G - C - A - U
A - U
U - A
R - Y
A - U
U - A
A - U
U - A
A - U
A - U
A - U
A - U
R - Y
R - Y
A - U
G - C
C - G
C - G
C - G
C - G
G - C
R - Y
G - C
Y - R
A - U
U - A
C - G
5´ - A A R
Y Y U U U

HMP2_2914_1

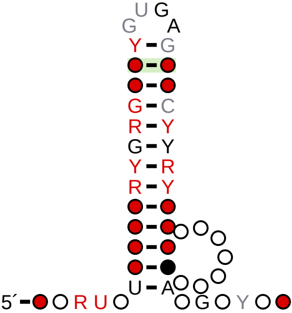

HMP2_2922_1

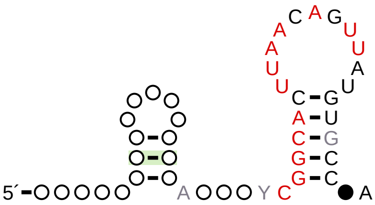

HMP2_2931_1

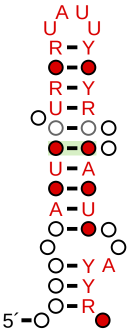

HMP2_2937_1

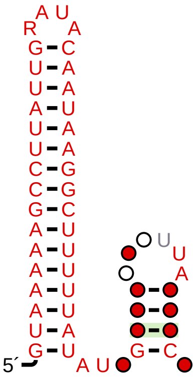

HMP2_2940_1

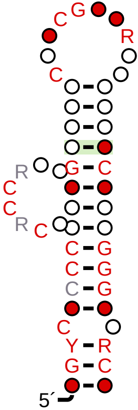

HMP2_2943_1

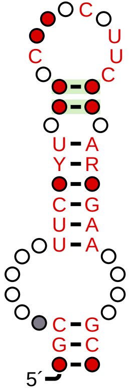

HMP2_2949_1

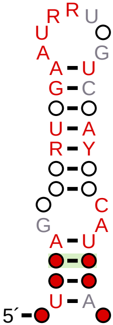

HMP2_2953_1

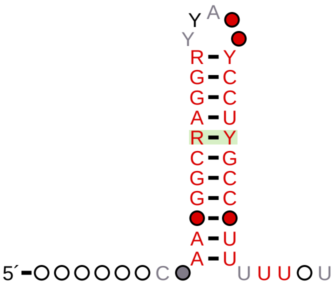

HMP2_2954_1

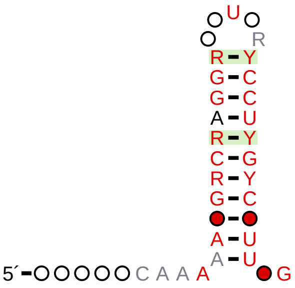

HMP2_2957_1

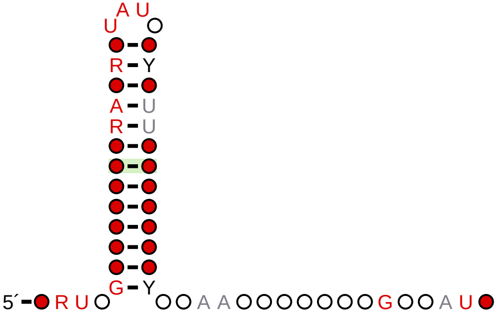

HMP2_2958_1

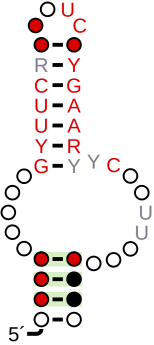

HMP2_2973_1

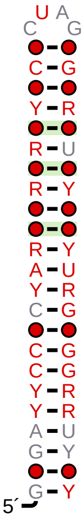

HMP2_2978_1

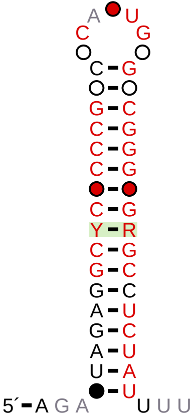

HMP2_2980_1

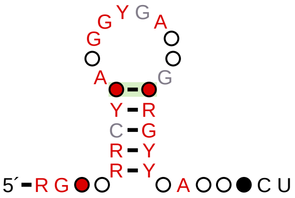

HMP2_2987_1

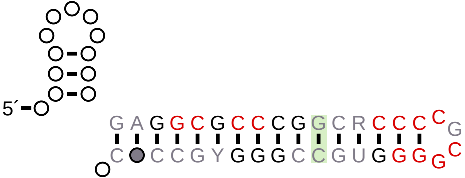

HMP2_2990_1

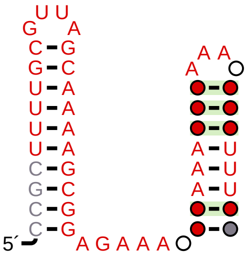

HMP2_2991_1

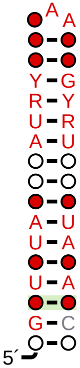

HMP2_2995_1

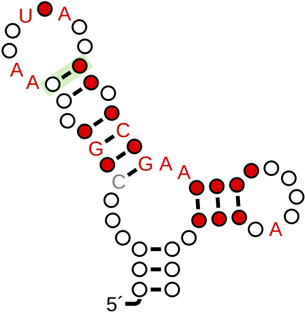

HMP2_3_1

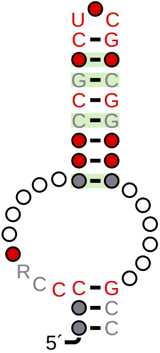

HMP2_300_1

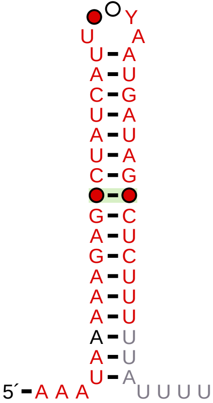

HMP2_3000_1

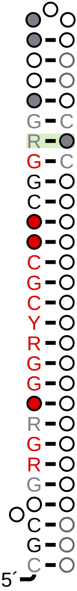

HMP2_3006_1

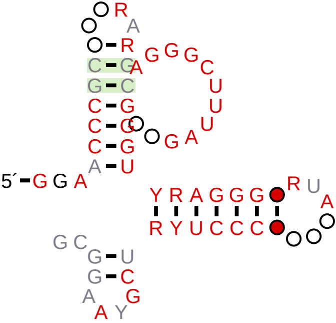

HMP2_301_1

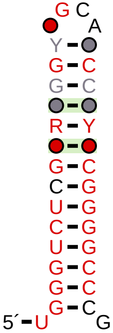

HMP2_3010_1

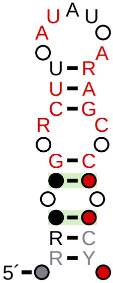

HMP2_3023_1

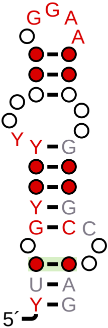

HMP2_3024_1

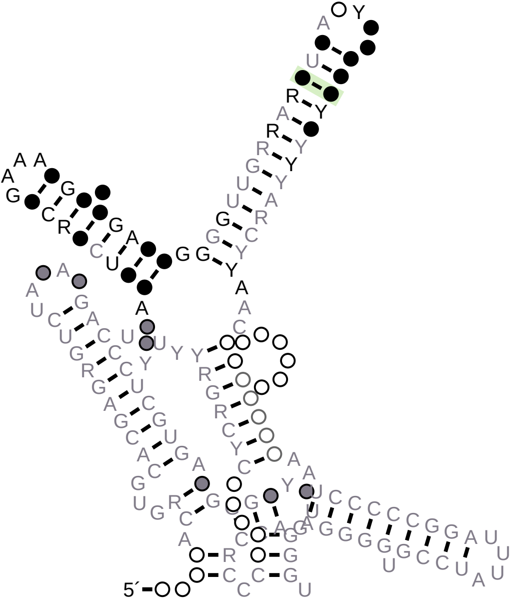

HMP2_3039_1

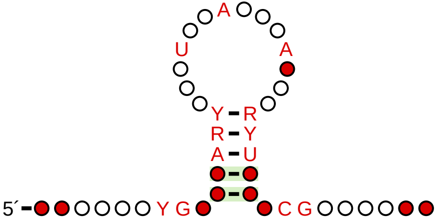

HMP2_304_1

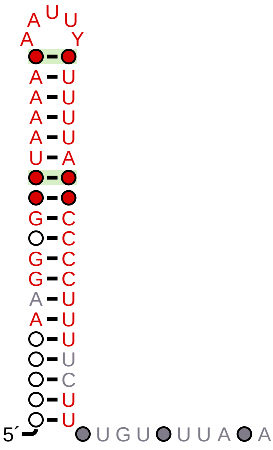

HMP2_3043_1

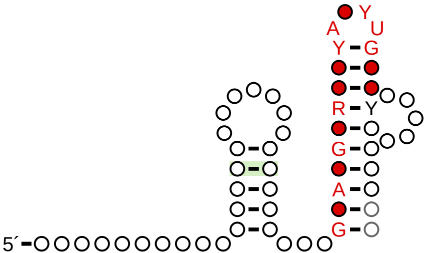

HMP2_3053_1

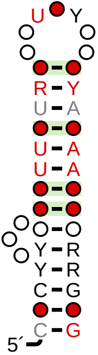

HMP2_3055_1

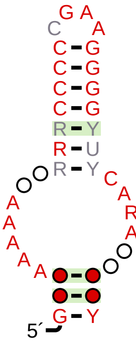

HMP2_3056_1

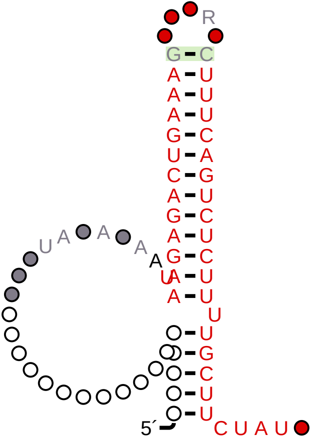

HMP2_3060_1

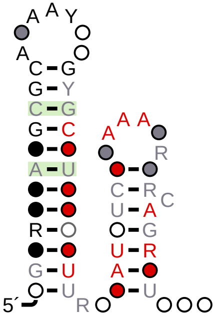

HMP2_3061_1

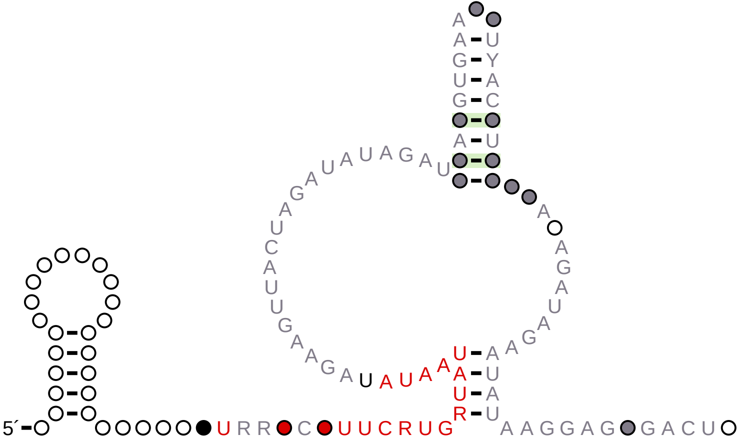

HMP2_3069_1

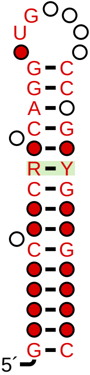

HMP2_3070_1

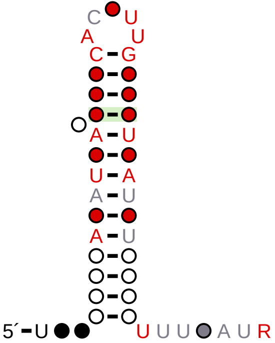

HMP2_3072_1

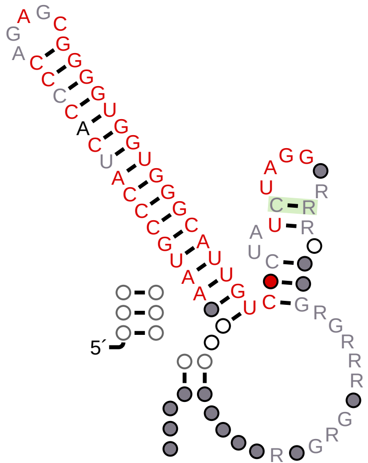

HMP2_3074_1

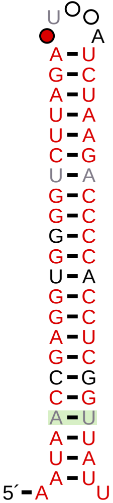

HMP2_3080_1

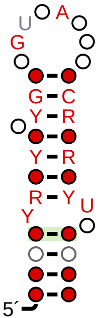

HMP2_3081_1

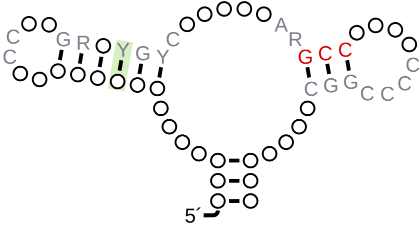

HMP2_3092_1

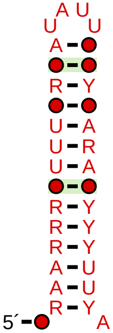

HMP2_3093_1

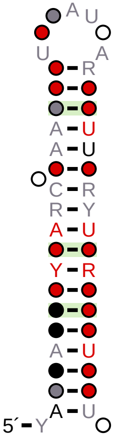

HMP2_3098_1

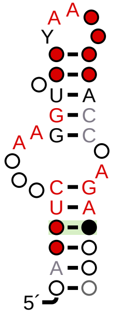

HMP2_310_1

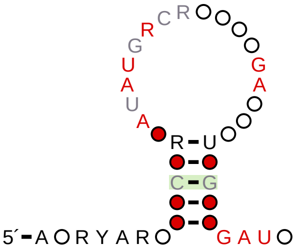

HMP2_3103_1

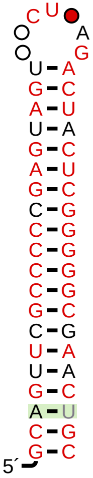

HMP2_3106_1

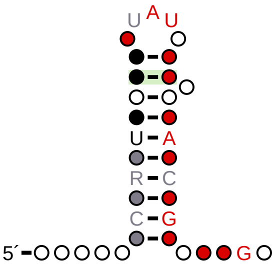

HMP2_3112_1

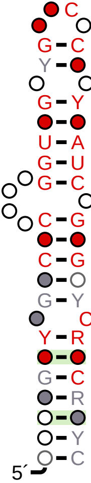

HMP2_3115_1

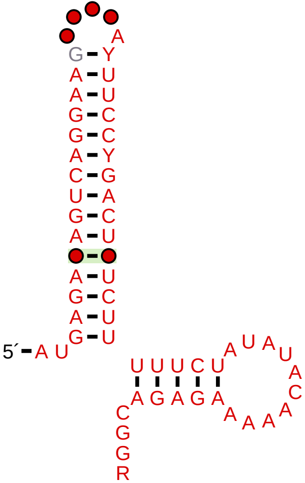

A
G Y
A U
A U
G C
G C
A Y
C G
U A
G C
A U
A U
G C
A U
G U
5´ A U
A U A
U
A
C
A
U U U C U
A G A G A
A A A
C
G
G
R

HMP2_3118_1

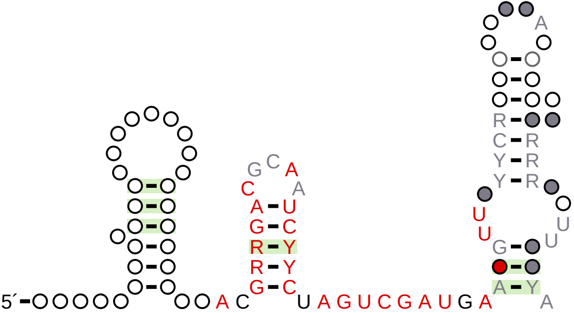

HMP2_3121_1

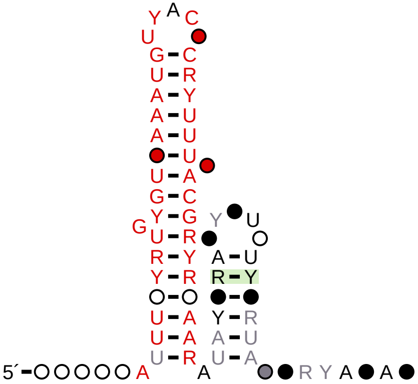

Y A C
U
G C
U R
A Y
A U
A U
U
U A
G C
Y G
G
U R
R Y
Y R
U A
U A
U R
Y U
A U
R Y
Y R
A U
U A
5´
A
A
R Y A A

HMP2_3125_1

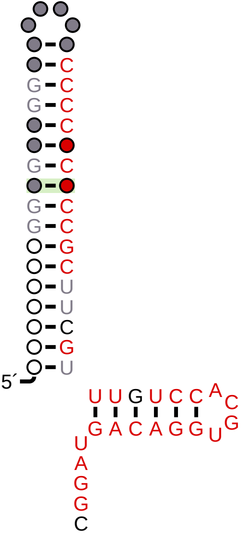

HMP2_3129_1

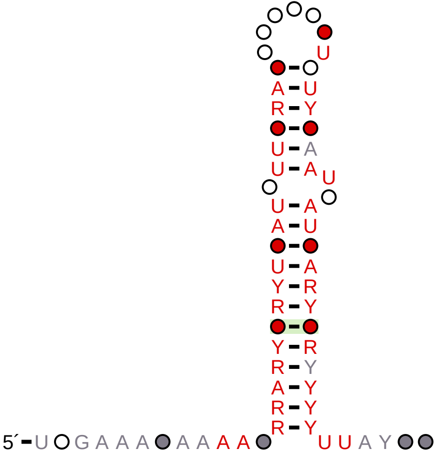

HMP2_3132_1

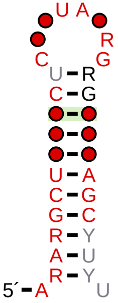

HMP2_3136_1

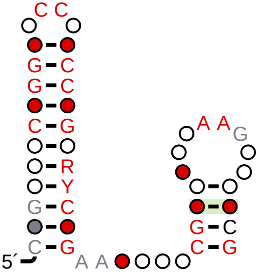

HMP2_3140_1

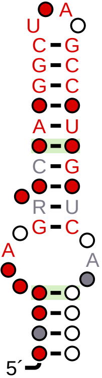

HMP2_3146_1

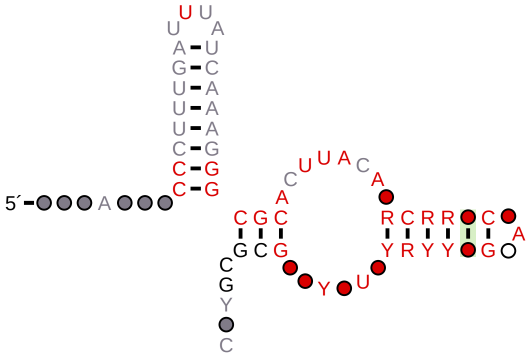

HMP2_315_1

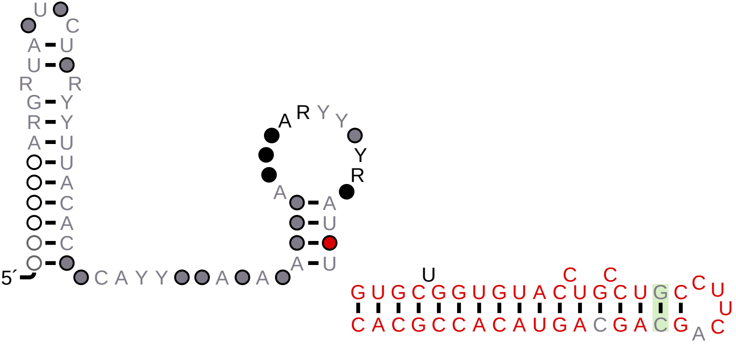

HMP2_3156_1

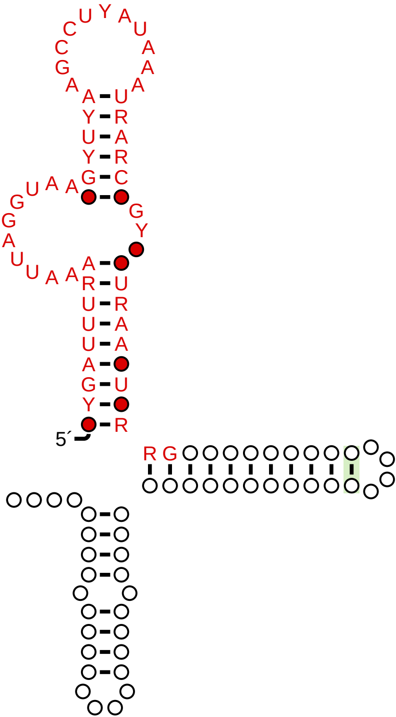
U Y A
C U
C A
G A
A A
A U
Y R
U A
Y R
G C
G U A A
G
G
Y
A
U
U A A A
R U
U R
U A
U A
A
G U
Y
R
5´
R G

HMP2_3162_1

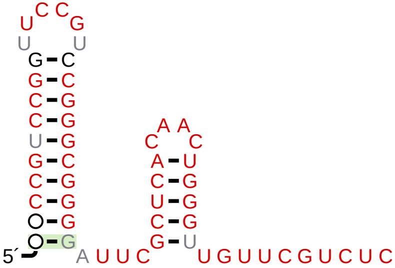
5´
U C C G
U U
G C
G C
C G
C G
U G
G C
C G
C G
G
G
A U U C
C A A C
A U
C G
U G
C G
G U
U G U U C G U C U C

HMP2_3175_1

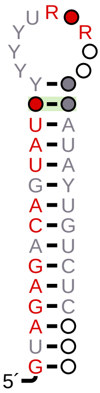

HMP2_3177_1

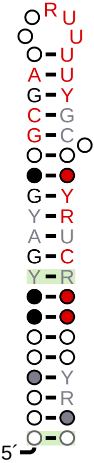

HMP2_3178_1

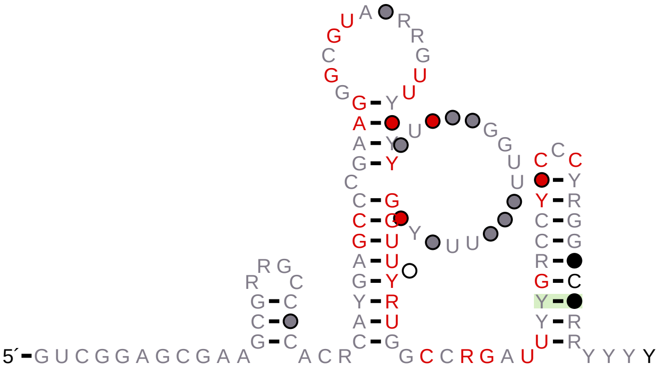

HMP2_3181_1

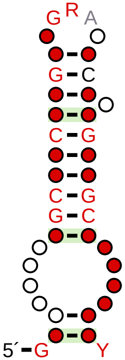

HMP2_3183_1

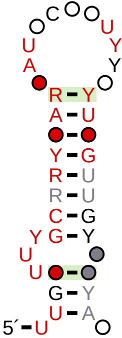

HMP2_3184_1

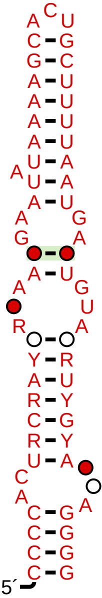

HMP2_3186_1

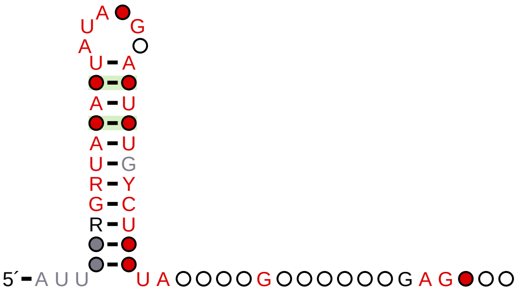

HMP2_3187_1

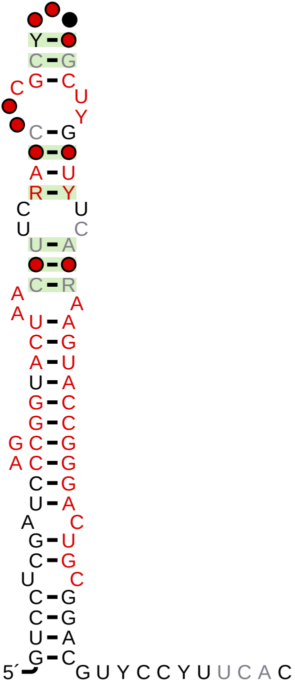

HMP2_319_1

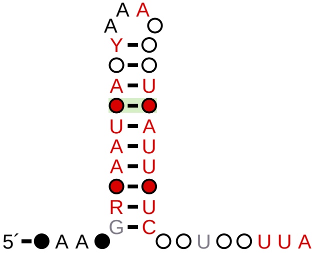

HMP2_3190_1

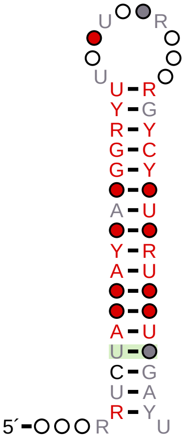

HMP2_3191_1

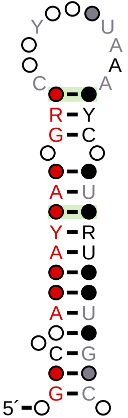

HMP2_3203_1

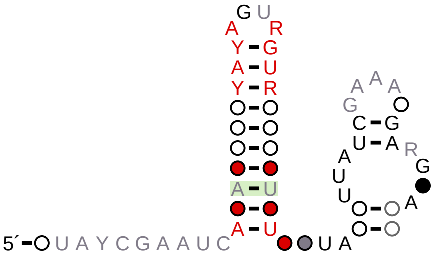

HMP2_321_1

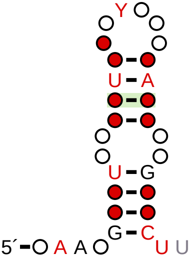

HMP2_3218_1

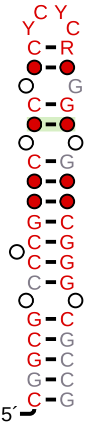

HMP2_3219_1

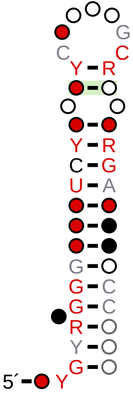

HMP2_322_1

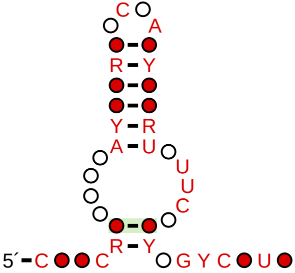

HMP2_3221_1

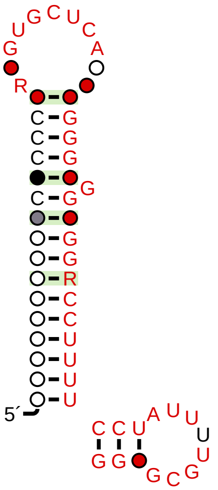

HMP2_3227_1

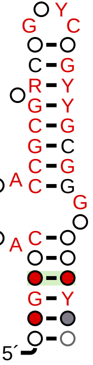

HMP2_3233_1

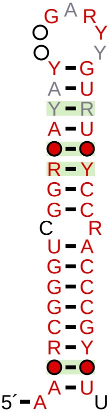

HMP2_3240_1

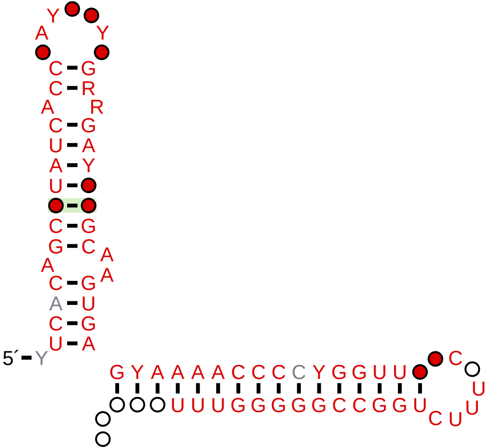

HMP2_3241_1

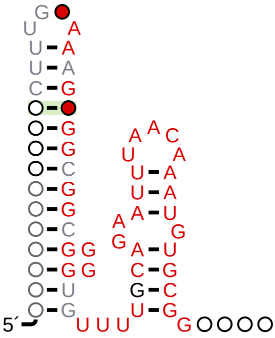

HMP2_3242_1

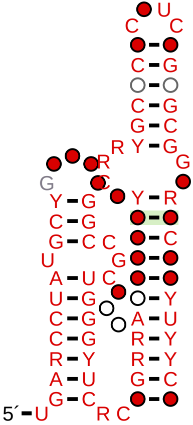

HMP2_3249_1

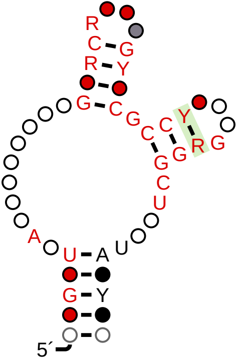

HMP2_3256_1

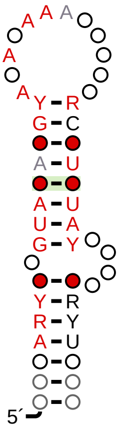

HMP2_3261_1

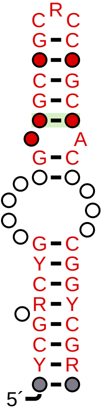

HMP2_3262_1

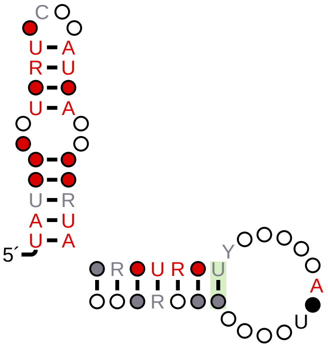

HMP2_3267_1

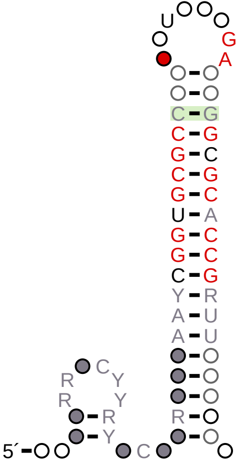

HMP2_3277_1

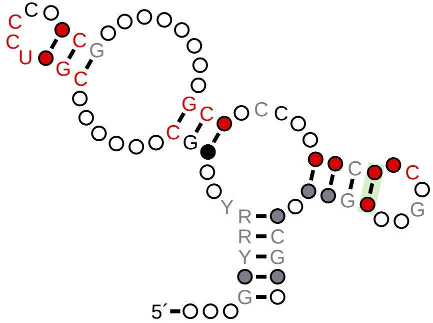

HMP2_328_1

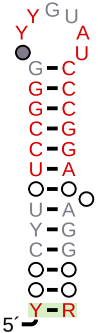

HMP2_3280_1

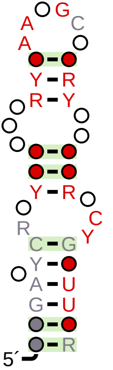

HMP2_3282_1

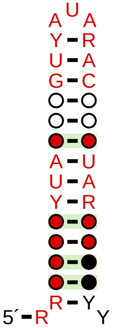

HMP2_3289_1

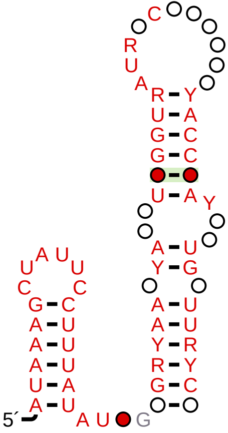

HMP2_3290_1

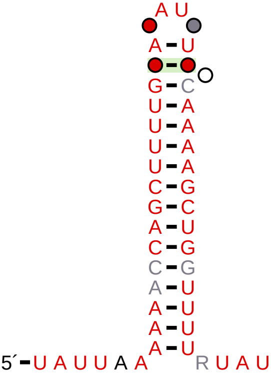

HMP2_3294_1

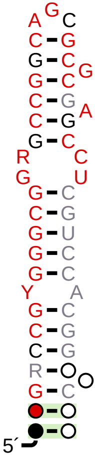

HMP2_3295_1

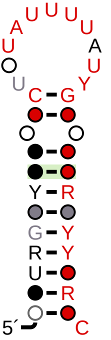

HMP2_3302_1

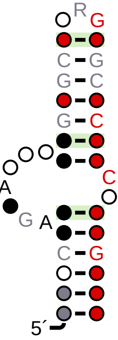

HMP2_3303_1

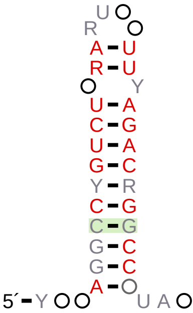

HMP2_3306_1

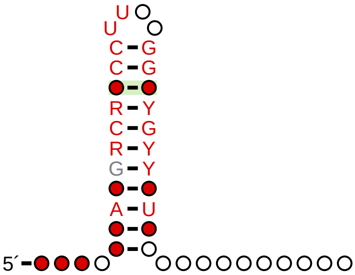

HMP2_331_1

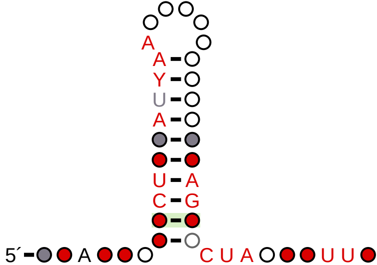

HMP2_3310_1

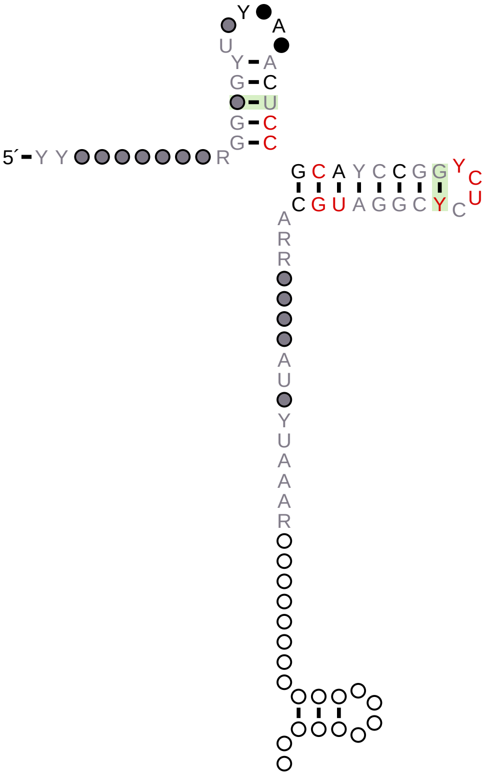

HMP2_3311_1

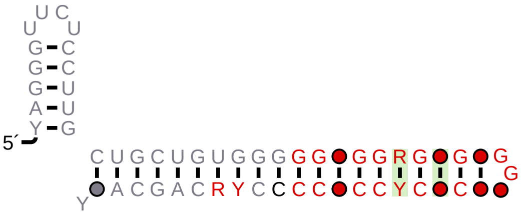

HMP2_3314_1

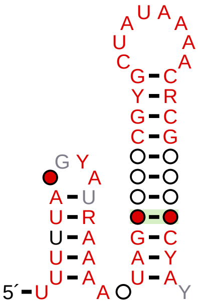

HMP2_3317_1

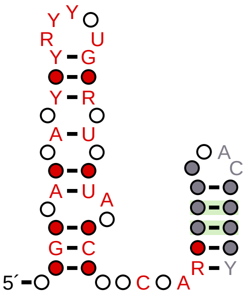

HMP2_332_1

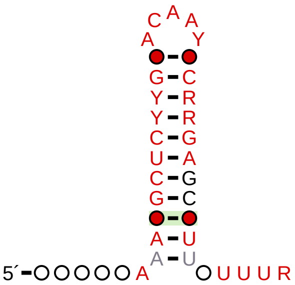

HMP2_3322_1

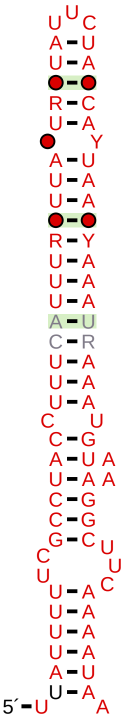

HMP2_3327_1

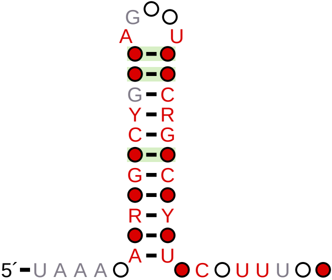

HMP2_3332_1

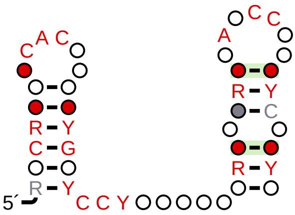

HMP2_3334_1

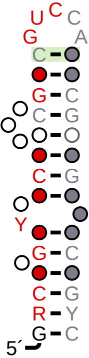

HMP2_3337_1

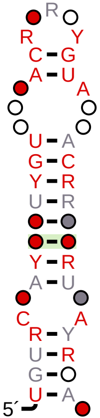

HMP2_3344_1

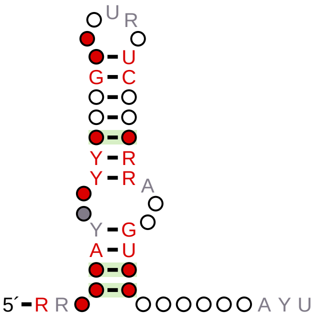

HMP2_3347_1

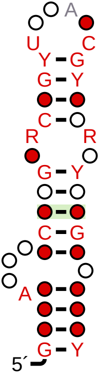

HMP2_3352_1

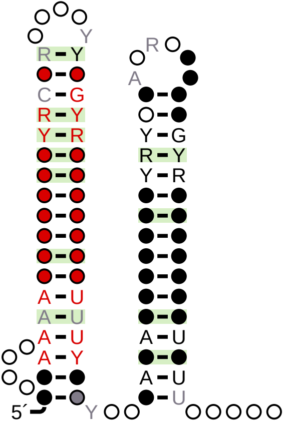

HMP2_3353_1

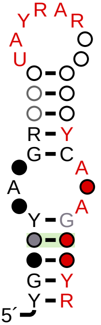

HMP2_3365_1

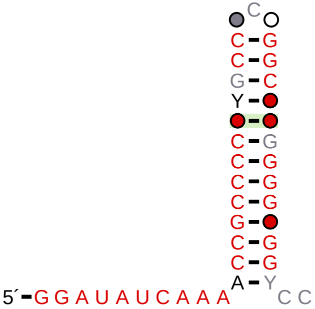

HMP2_3371_1

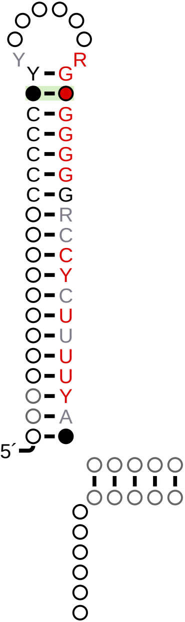

HMP2_3379_1

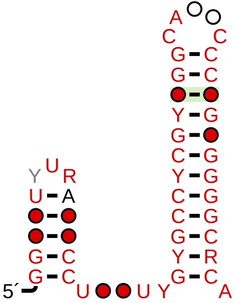

HMP2_3387_1

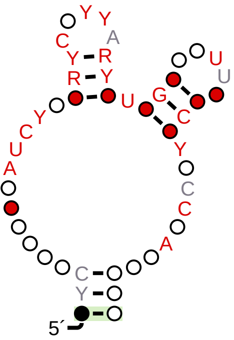

HMP2_3392_1

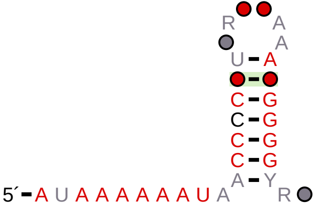

HMP2_3393_1

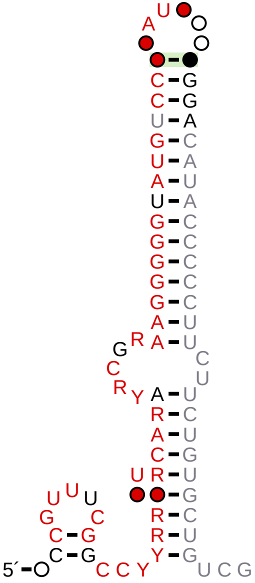

HMP2_3403_1

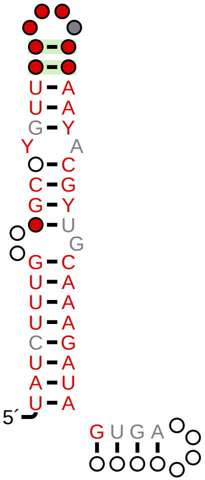

HMP2_3404_1

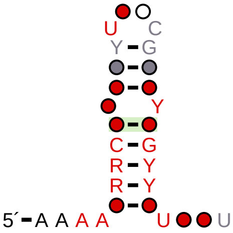

HMP2_3406_1

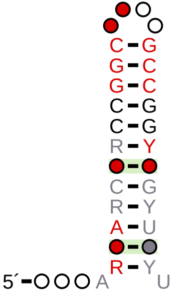

HMP2_3411_1

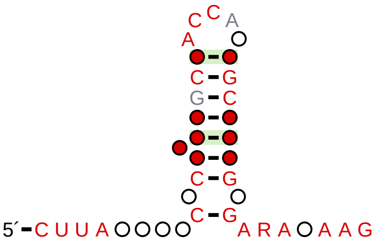

HMP2_3413_1

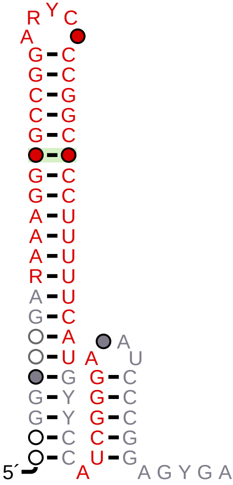

HMP2_3414_1

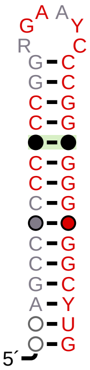

HMP2_3424_1

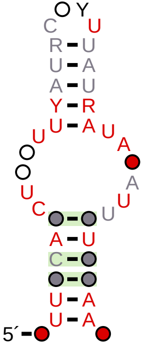

HMP2_3427_1

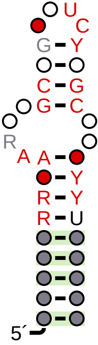

HMP2_3429_1

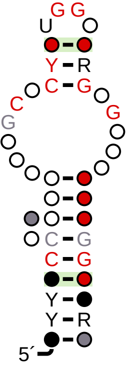

HMP2_344_1

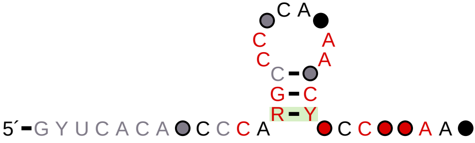

HMP2_3443_1

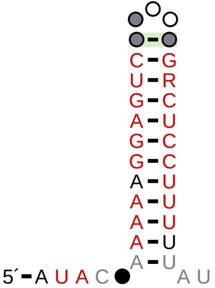

HMP2_3444_1

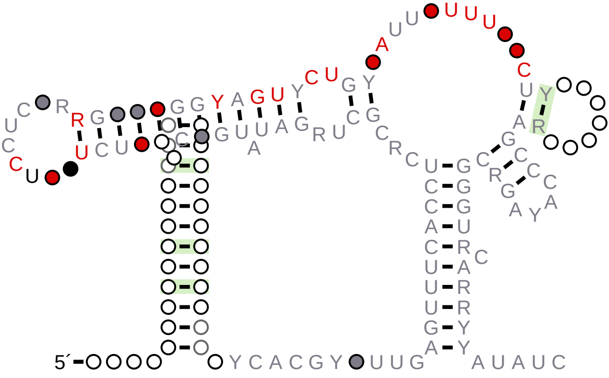

HMP2_3452_1

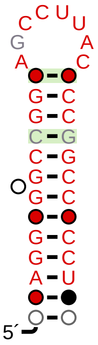

HMP2_3459_1

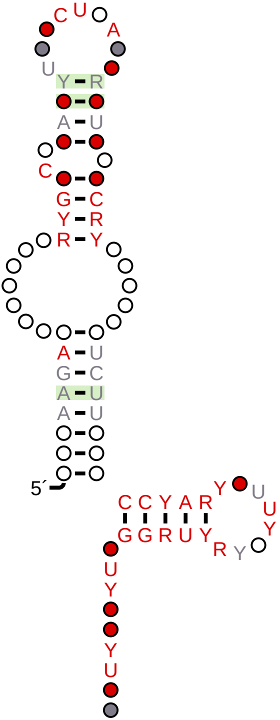

HMP2_3469_1

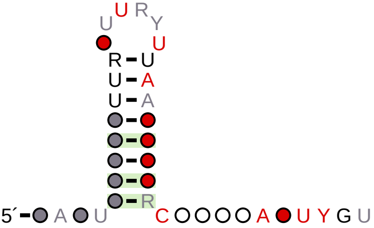

HMP2_3474_1

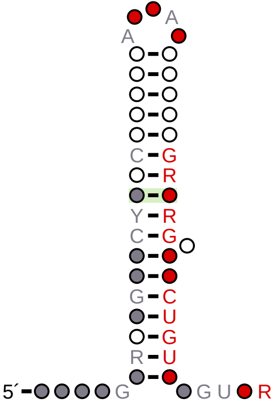

HMP2_3475_1

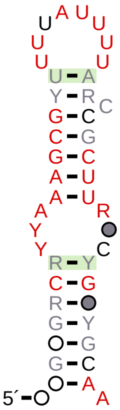

HMP2_3493_1

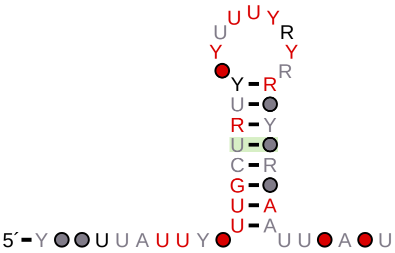

HMP2_3495_1

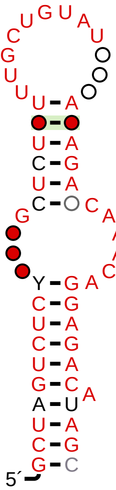

HMP2_3496_1

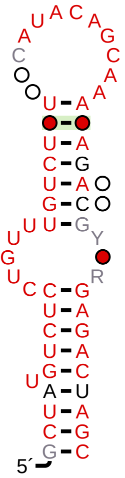

HMP2_350_1

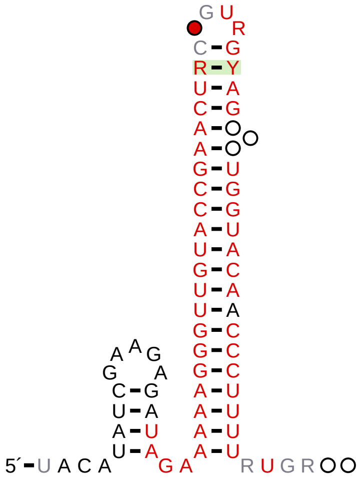

G U
R
C G
R Y
U A
C G
A
A
G U
C G
C G
A U
U A
G C
U A
U A
G C
G C
G C
A U
A U
A U
A U
A A G
G A
C G
U A
A U
U A
5´ U A C A
G A
R U G R

HMP2_3500_1

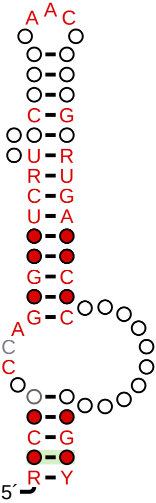

HMP2_3502_1

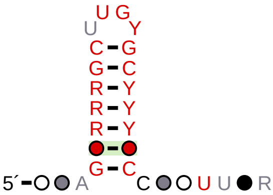

HMP2_3504_1

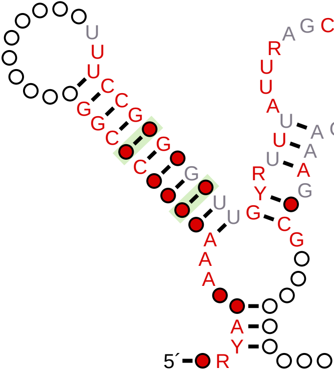

HMP2_351_1

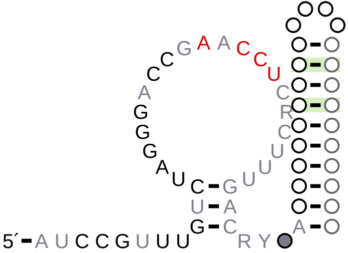

HMP2_3510_1

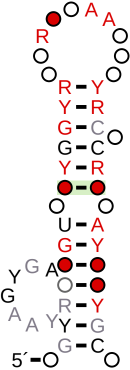

HMP2_3514_1

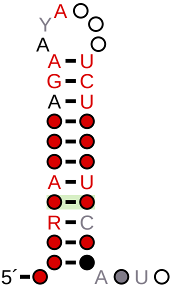

HMP2_3516_1

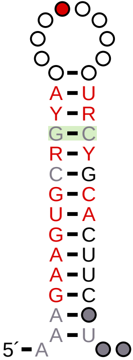

HMP2_3521_1

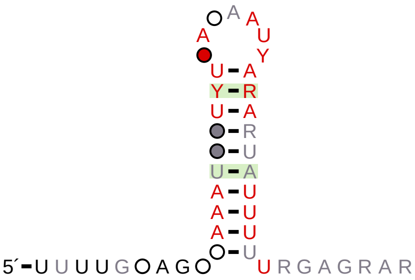

HMP2_3524_1

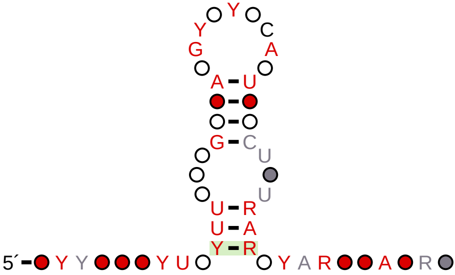

HMP2_3526_1

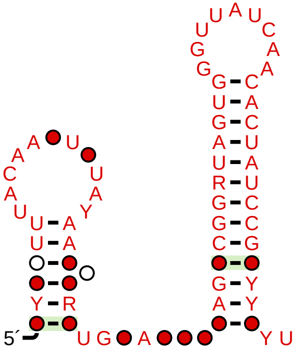

HMP2_3533_1

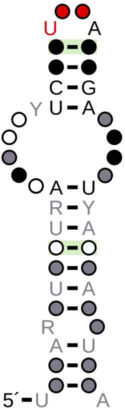

HMP2_3540_1

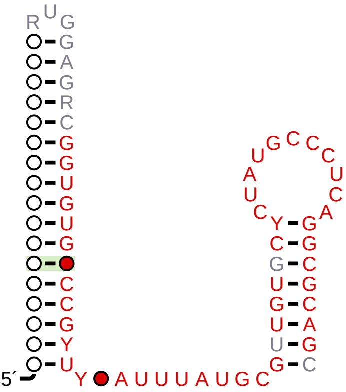

HMP2_3551_1

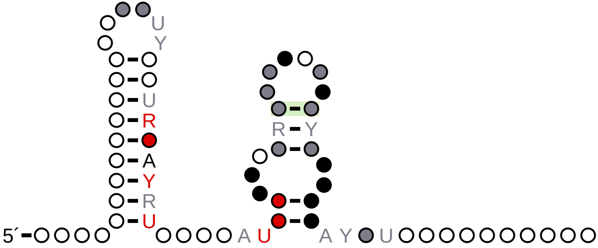

HMP2_3556_1

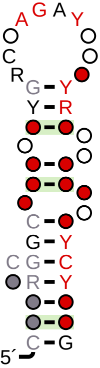

HMP2_3559_1

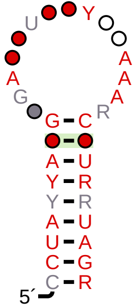

HMP2_3563_1

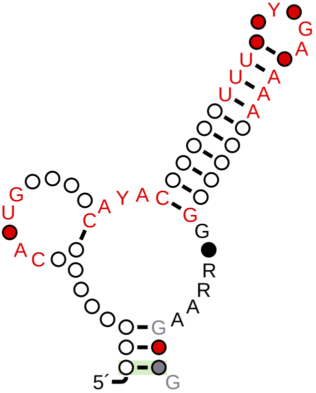

HMP2_3564_1

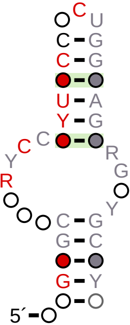

HMP2_3565_1

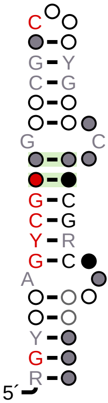

HMP2_3570_1

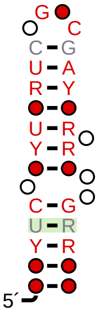

HMP2_3571_1

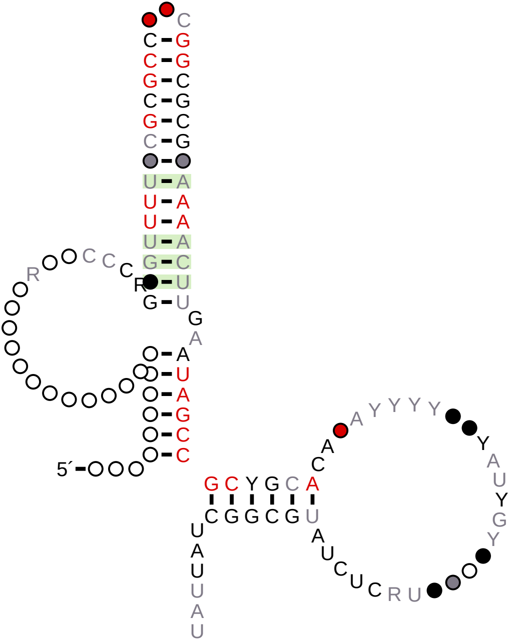

HMP2_3576_1

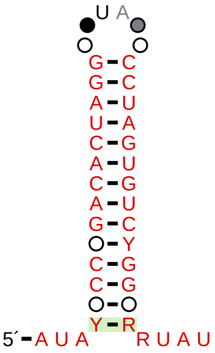

HMP2_359_1

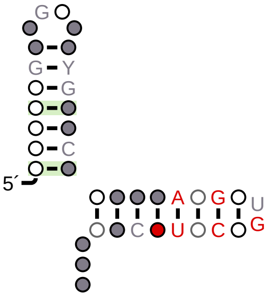

HMP2_3594_1

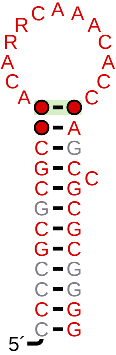

HMP2_3597_1

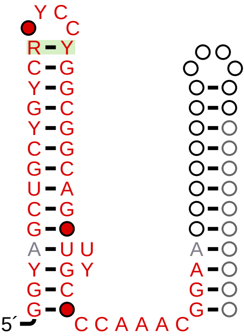

HMP2_3598_1

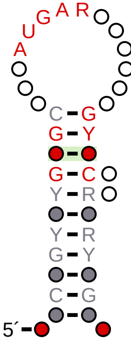

HMP2_3599_1

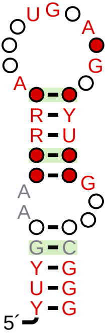

HMP2_3614_1

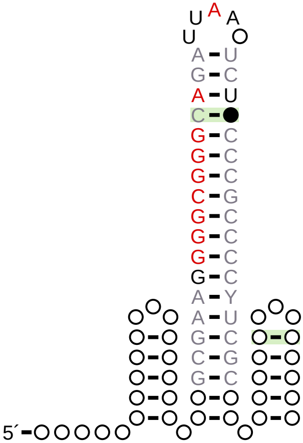

HMP2_362_1

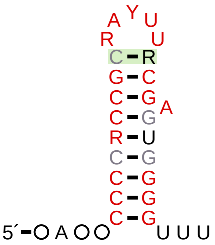

HMP2_3621_1

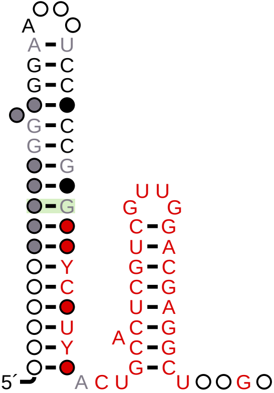

HMP2_3628_1

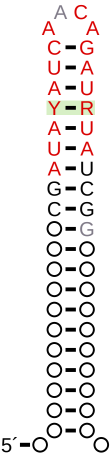

HMP2_3629_1

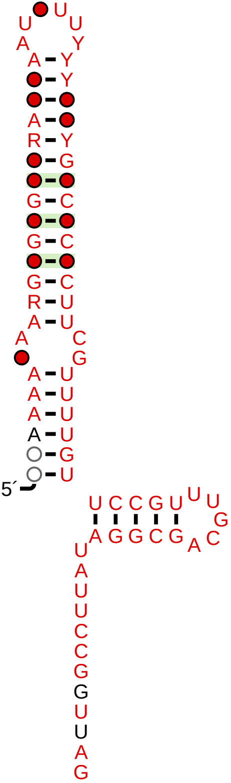

HMP2_3634_1

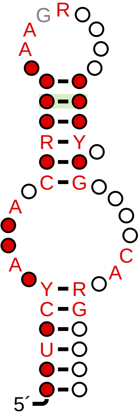

HMP2_3638_1

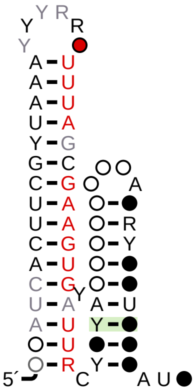

HMP2_3640_1

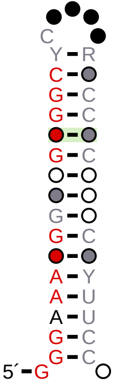

HMP2_3645_1

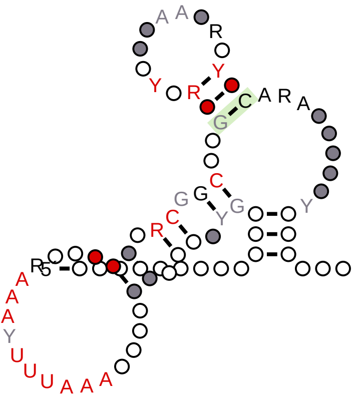

HMP2_3651_1

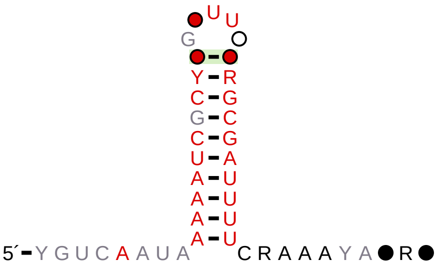

HMP2_3655_1

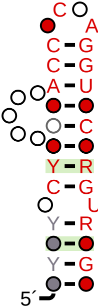

HMP2_3678_1

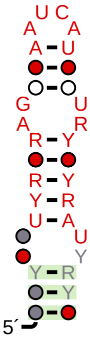

HMP2_368_1

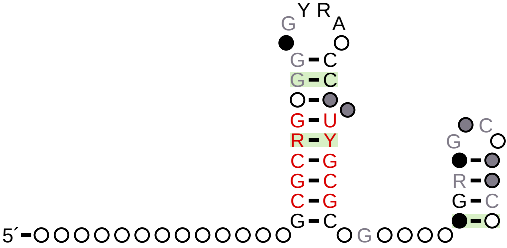

HMP2_3680_1

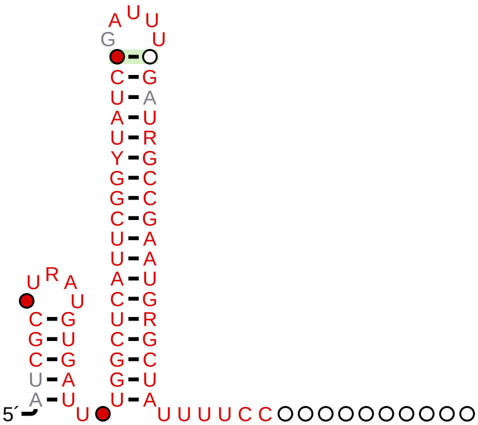

HMP2_3686_1

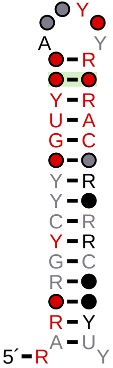

HMP2_3687_1

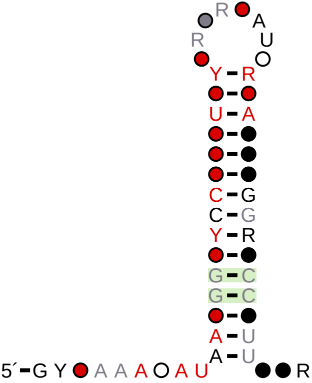

HMP2_3689_1

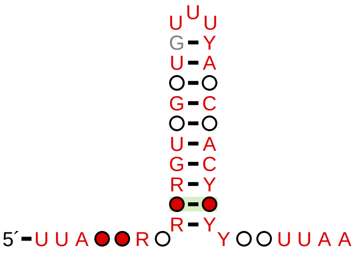

HMP2_3698_1

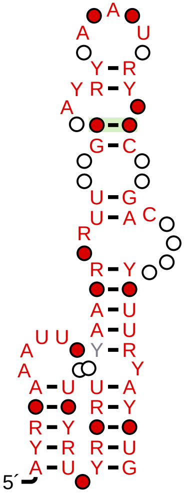

HMP2_3701_1

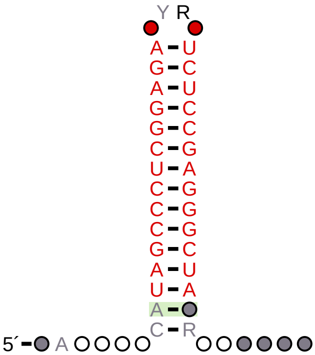

HMP2_3715_1

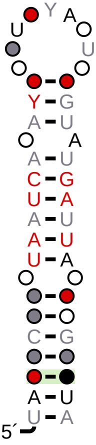

HMP2_3716_1

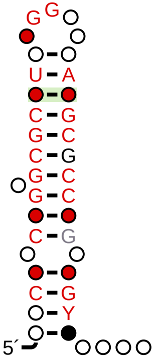

HMP2_3718_1

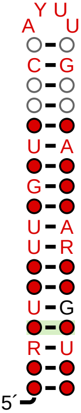

HMP2_372_1

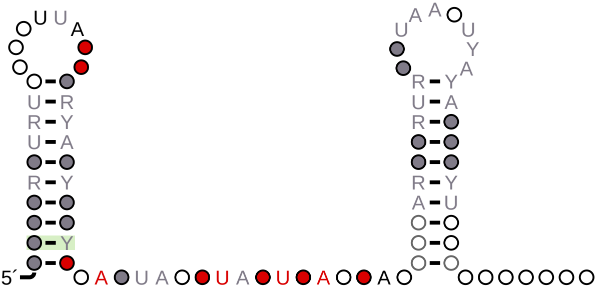

HMP2_3720_1

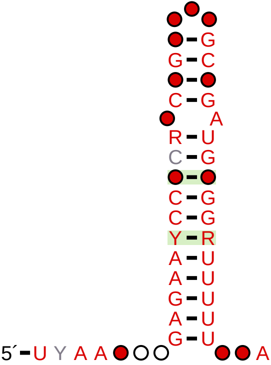

HMP2_3723_1

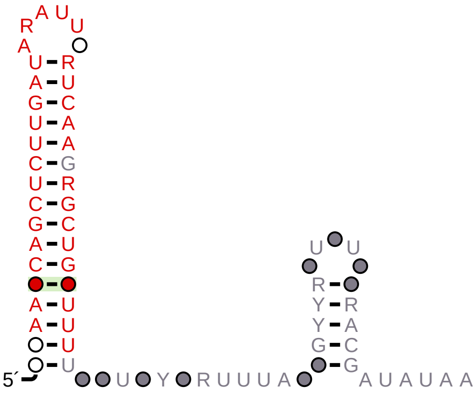
A U
R U
A
U R
A U
G C
U A
U A
C G
U R
C G
G C
A U
C G
A U
A U
U
U
5´
U Y R U U U A
U U
R
Y R
Y A
G C
G
A U A U A A

HMP2_3724_1

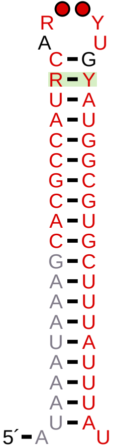

HMP2_373_1

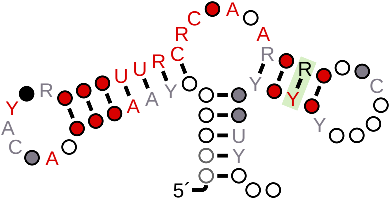

HMP2_3732_1

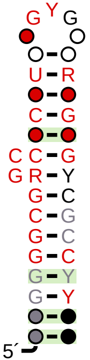

HMP2_3745_1

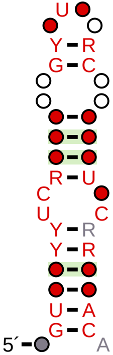

HMP2_3746_1

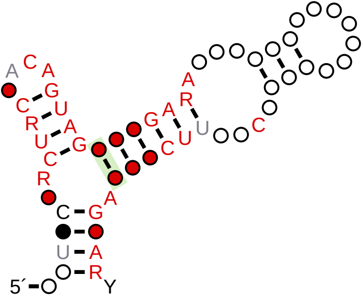

HMP2_3747_1

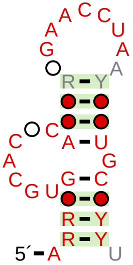

HMP2_3748_1

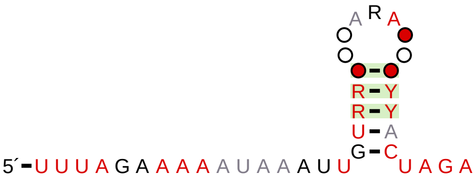

HMP2_3750_1

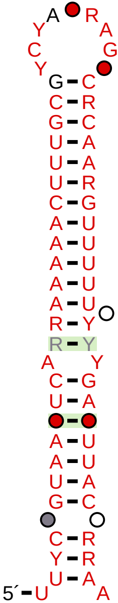

HMP2_3756_1

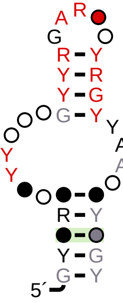

HMP2_376_1

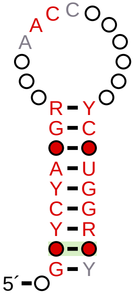

HMP2_3766_1

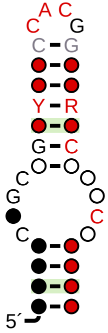

HMP2_3772_1

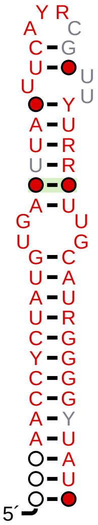

HMP2_3783_1

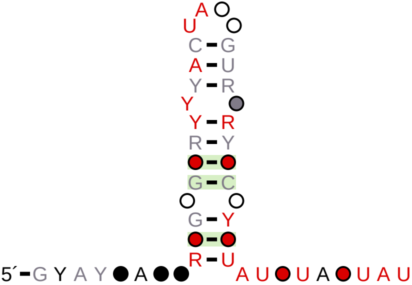

HMP2_3794_1

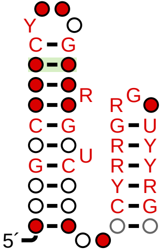

HMP2_3795_1

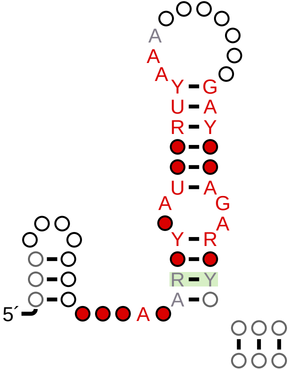

HMP2_38_1

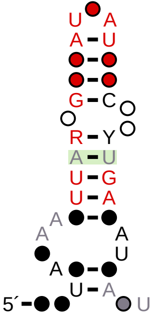

HMP2_3804_1

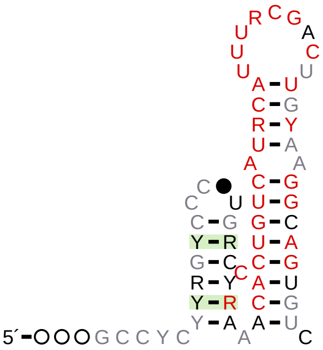

HMP2_3806_1

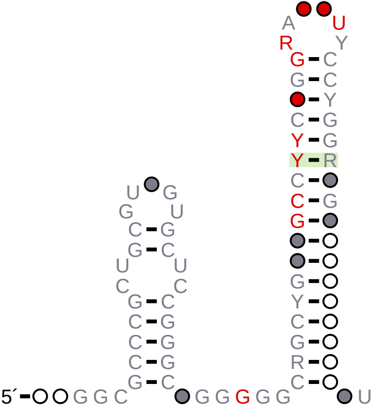

HMP2_3807_1

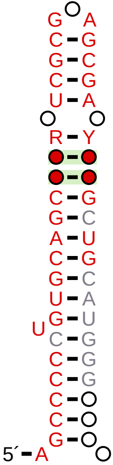

HMP2_381_1

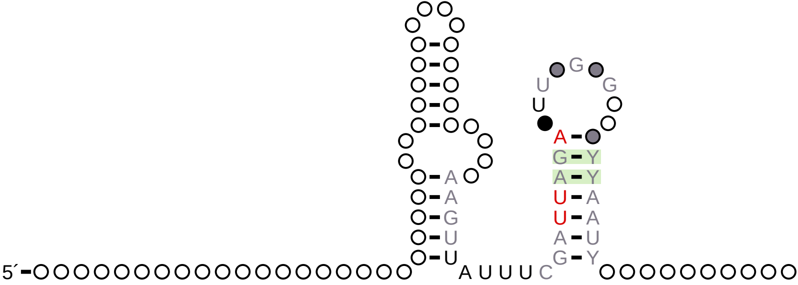

HMP2_3816_1

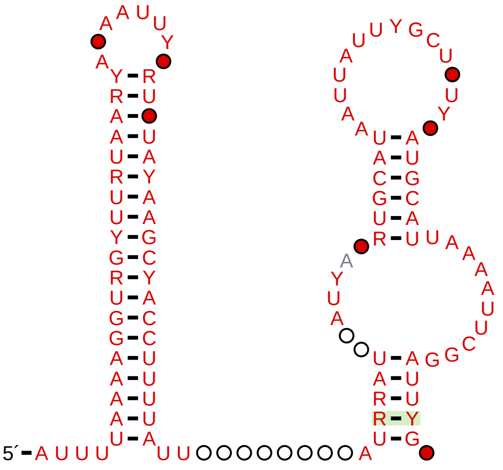

HMP2_382_1

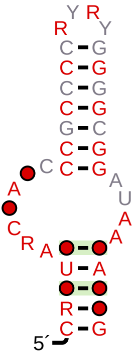

HMP2_3822_1

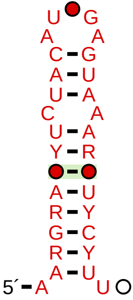

HMP2_3826_1

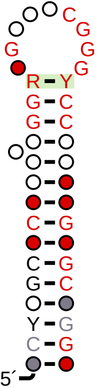

HMP2_3837_1

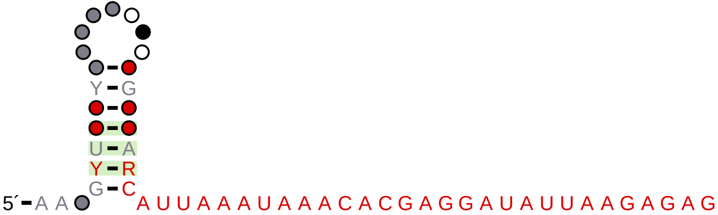

HMP2_384_1

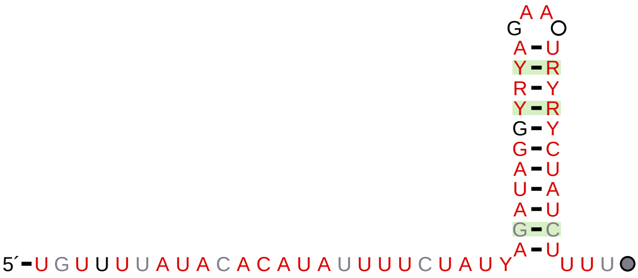
5´-UGUUUUAUACACAUAUUUUCUAUY
AGAUAGGYRYAGAA
UURYRUCUAUAU
UUU

HMP2_3841_1

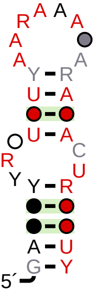

HMP2_3848_1

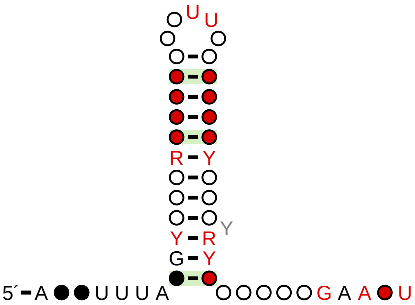

HMP2_3849_1

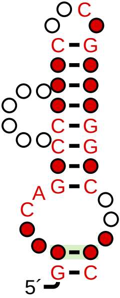

HMP2_3854_1

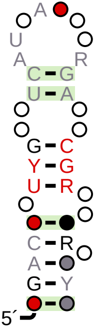

HMP2_3859_1

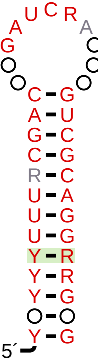

HMP2_3865_1

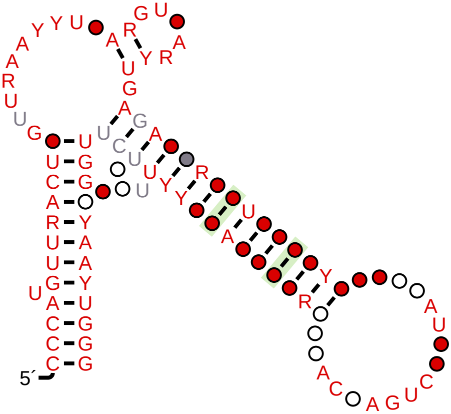

HMP2_3867_1

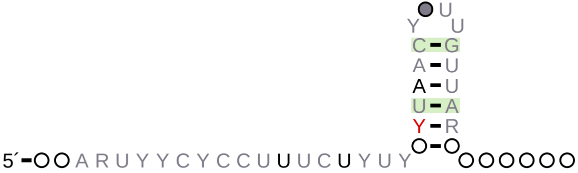

HMP2_3870_1

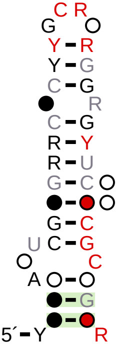

HMP2_3871_1

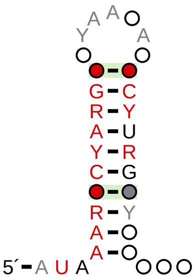

HMP2_3874_1

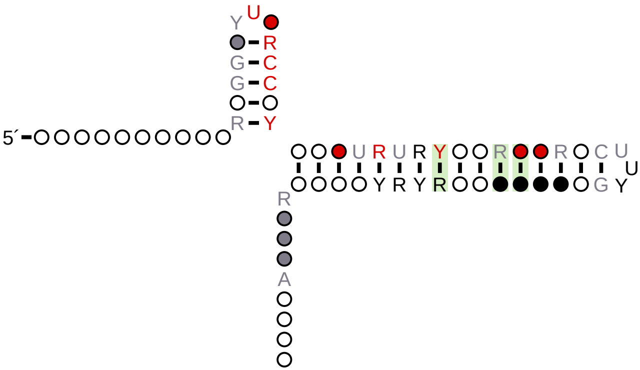

HMP2_3877_1

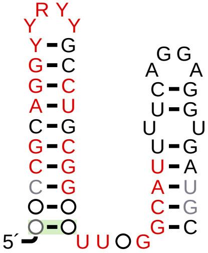

HMP2_3893_1

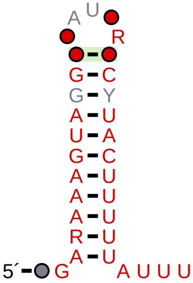

HMP2_3895_1

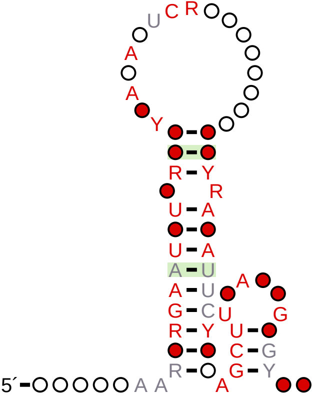

HMP2_3897_1

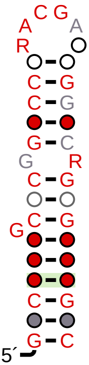

HMP2_3899_1

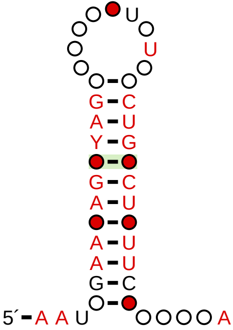

HMP2_39_1

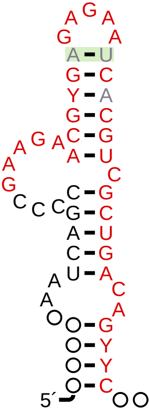

HMP2_3901_1

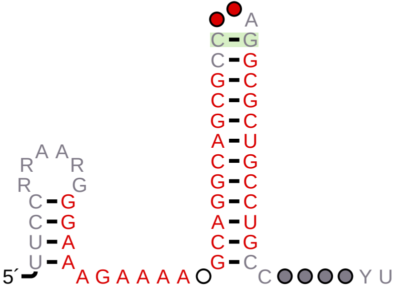

HMP2_3911_1

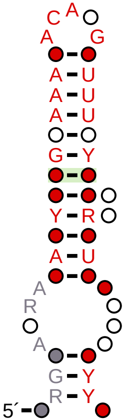

HMP2_3917_1

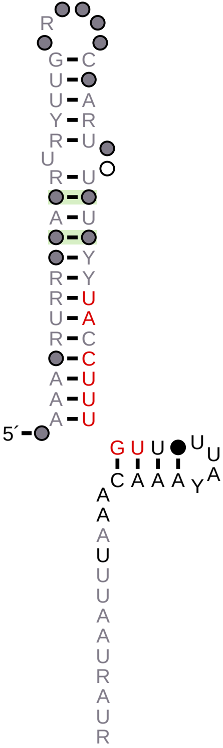

HMP2_3922_1

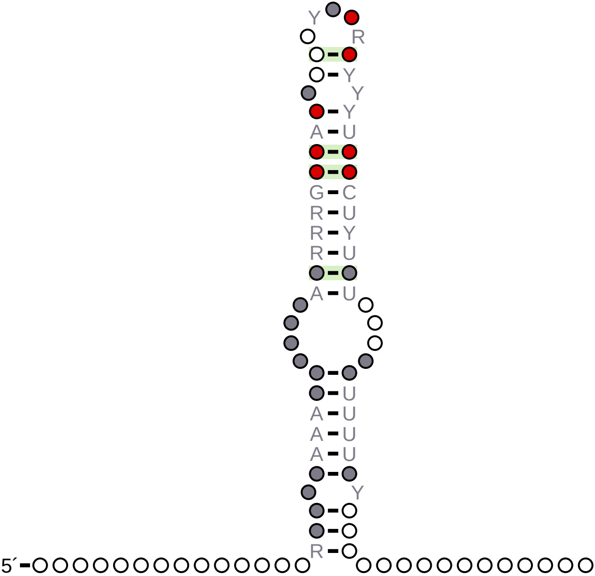

HMP2_3926_1

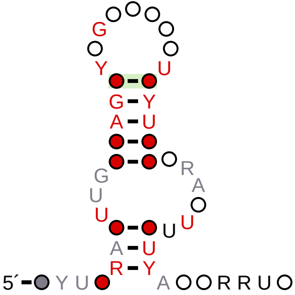

HMP2_3933_1

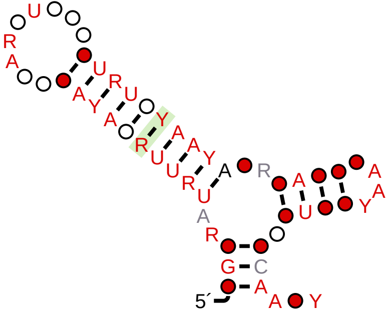

HMP2_3945_1

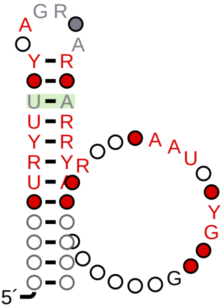

HMP2_3963_1

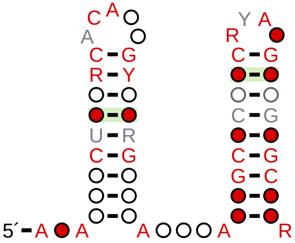

HMP2_3977_1

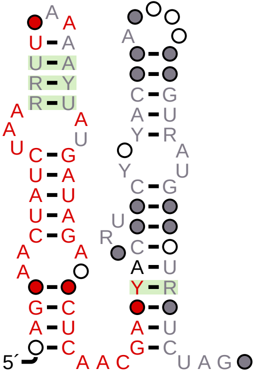

HMP2_3979_1

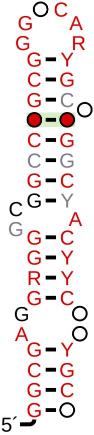

HMP2_3987_1

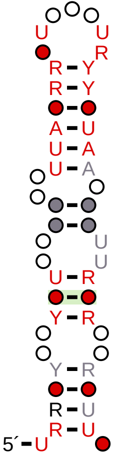

HMP2_3992_1

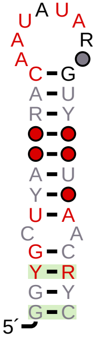

HMP2_3993_1

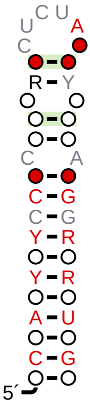

HMP2_3994_1

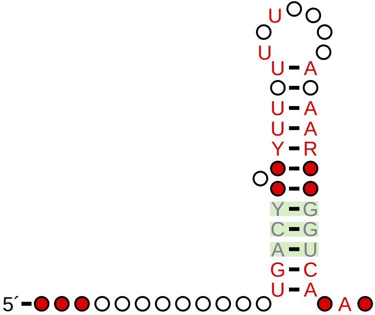

HMP2_40_1

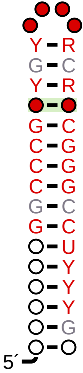

HMP2_4000_1

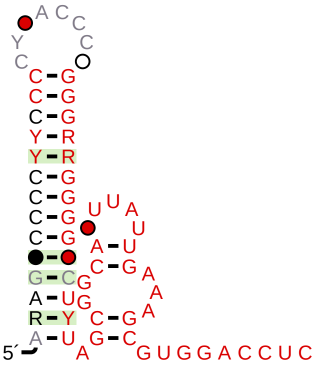

HMP2_4009_1

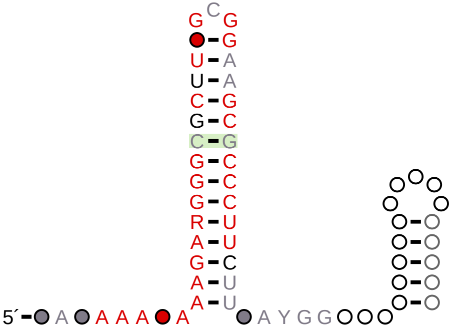

HMP2_4010_1

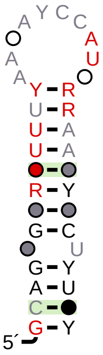

HMP2_4013_1

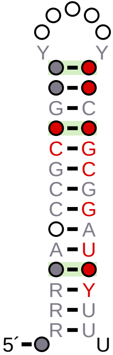

HMP2_4014_1

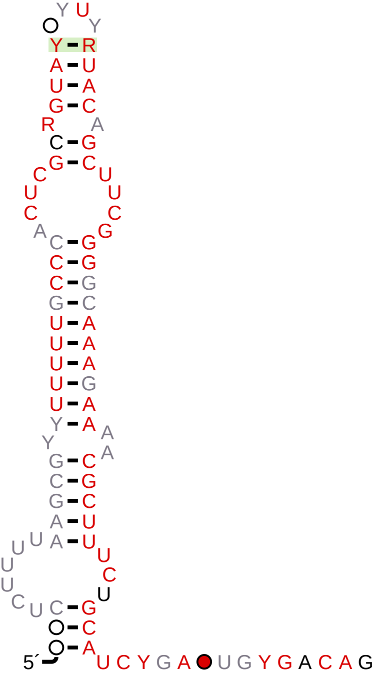

HMP2_4019_1

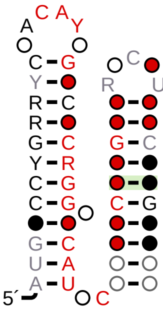

HMP2_4024_1

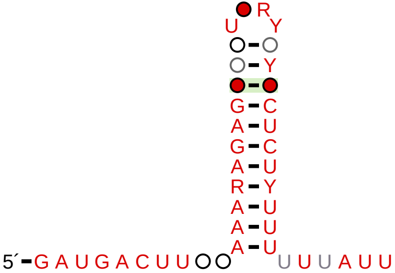

HMP2_4031_1

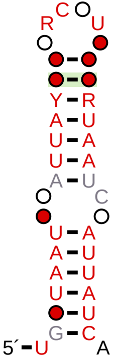

HMP2_4034_1

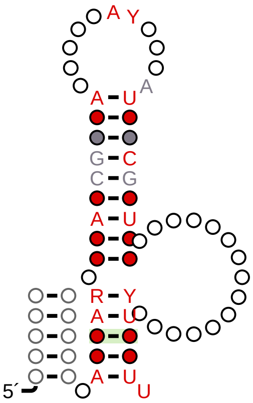

HMP2_4036_1

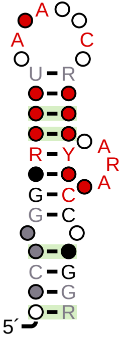

HMP2_4041_1

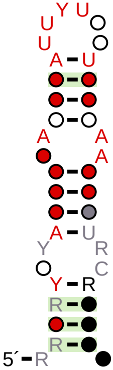

HMP2_4043_1

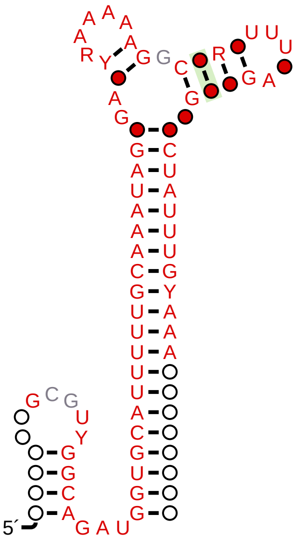

HMP2_4046_1

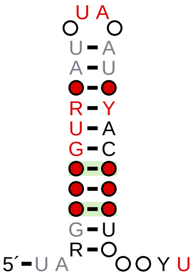

HMP2_4048_1

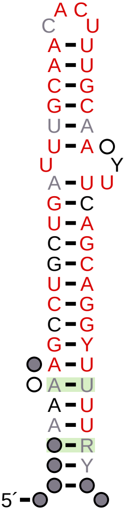

HMP2_405_1

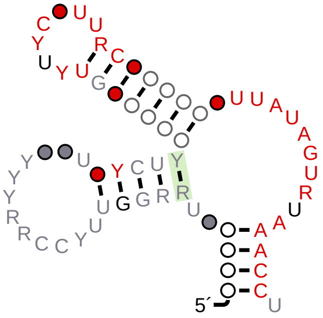

HMP2_4057_1

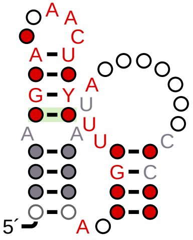

HMP2_4060_1

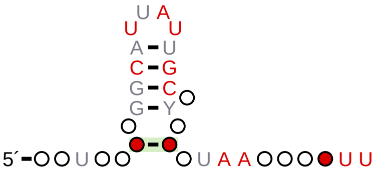

HMP2_4061_1

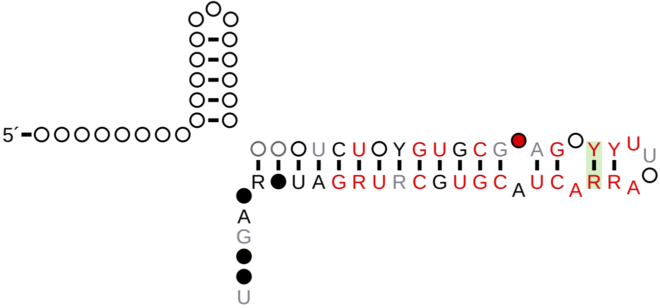

HMP2_4069_1

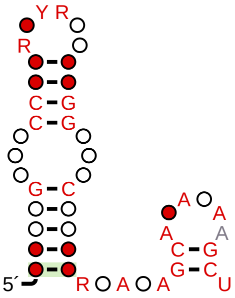

HMP2_4070_1

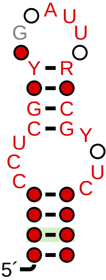

HMP2_4072_1

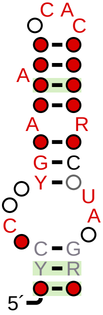

HMP2_4078_1

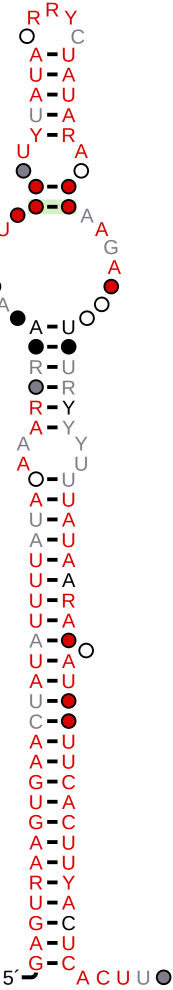

HMP2_4079_1

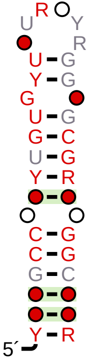

HMP2_408_1

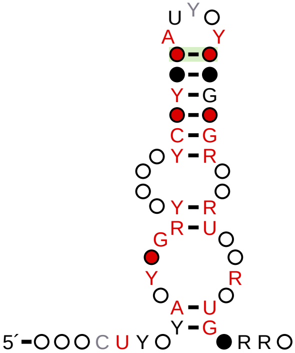

HMP2_4080_1

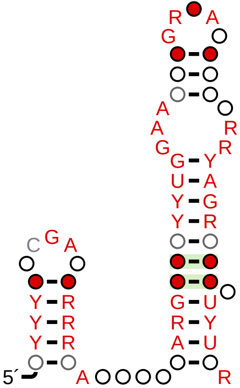

HMP2_4089_1

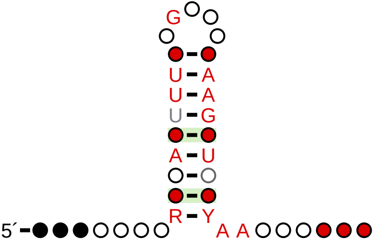

HMP2_4094_1

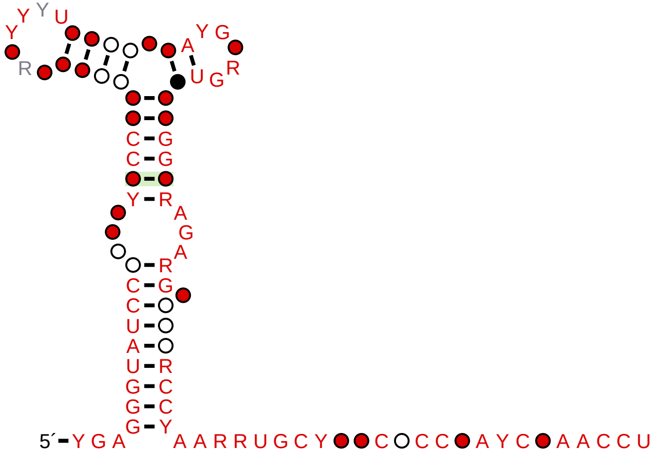

HMP2_4105_1

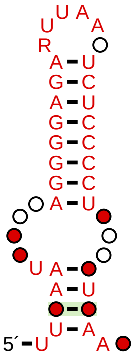

HMP2_4107_1

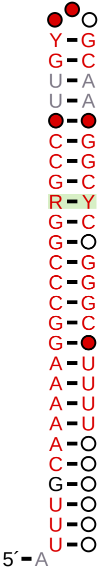

HMP2_4114_1

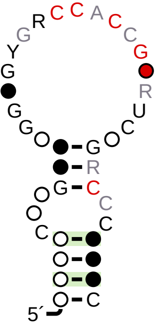

HMP2_4118_1

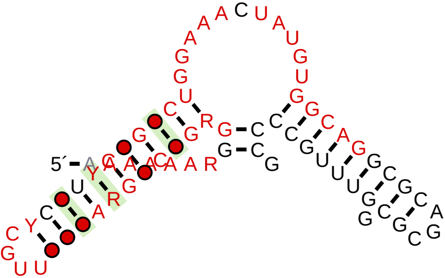

HMP2_4127_1

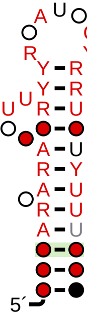

HMP2_4129_1

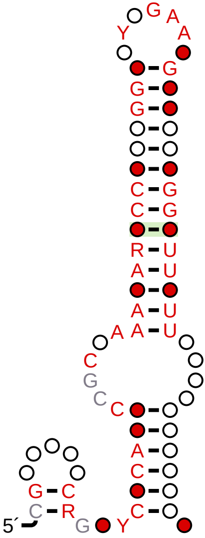

HMP2_4130_1

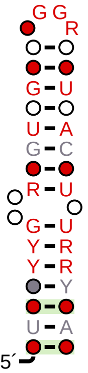

HMP2_4141_1

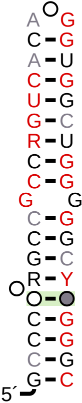

HMP2_4143_1

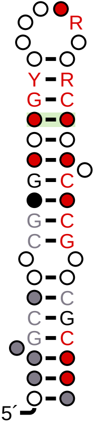

HMP2_4146_1

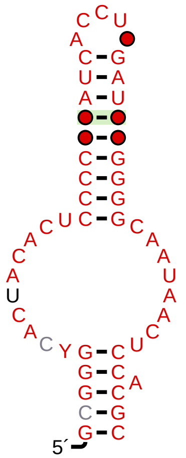

HMP2_4154_1

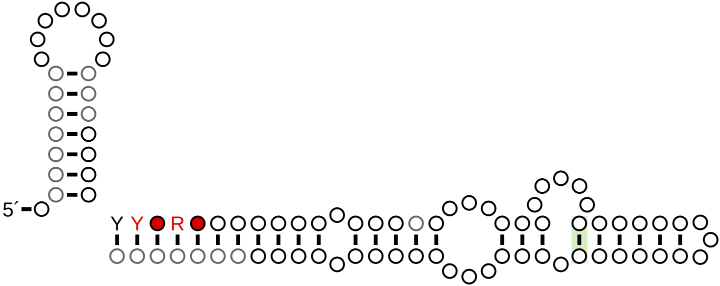

HMP2_4156_1

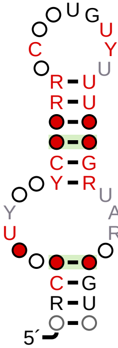

HMP2_4160_1

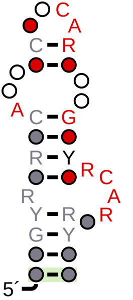

HMP2_4168_1

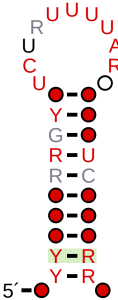

HMP2_4169_1

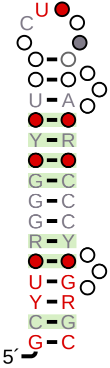

HMP2_417_1

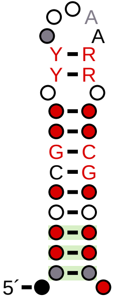

HMP2_4170_1

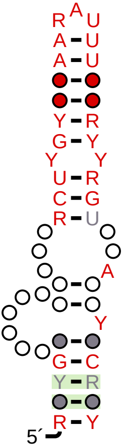

HMP2_4171_1

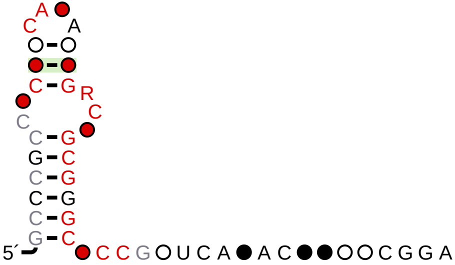

HMP2_4184_1

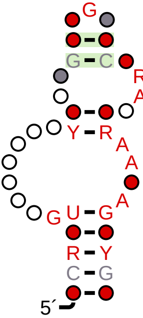

HMP2_4186_1

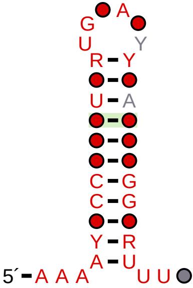

HMP2_4198_1

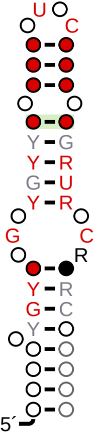

HMP2_42_1

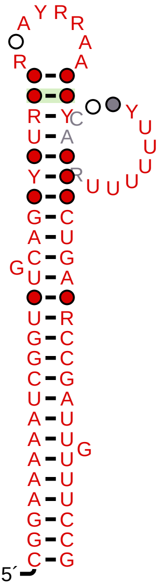

HMP2_420_1

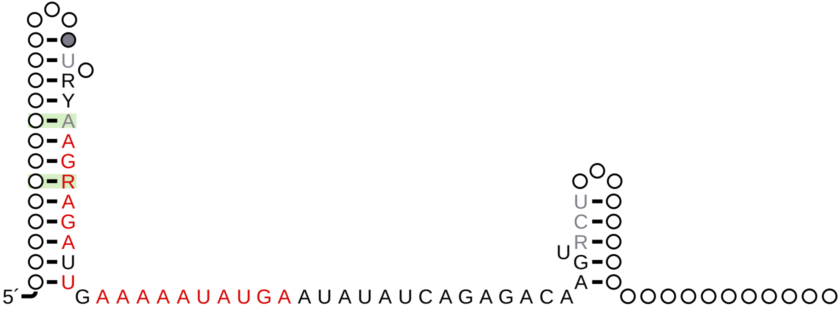

HMP2_4201_1

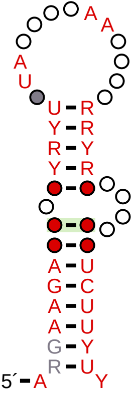

HMP2_4204_1

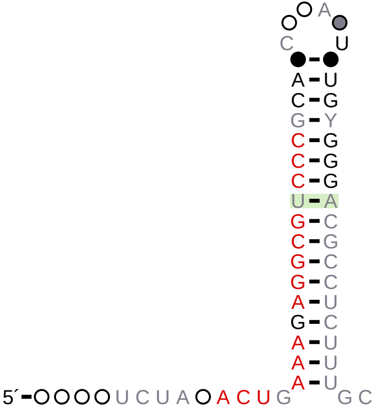

HMP2_4205_1

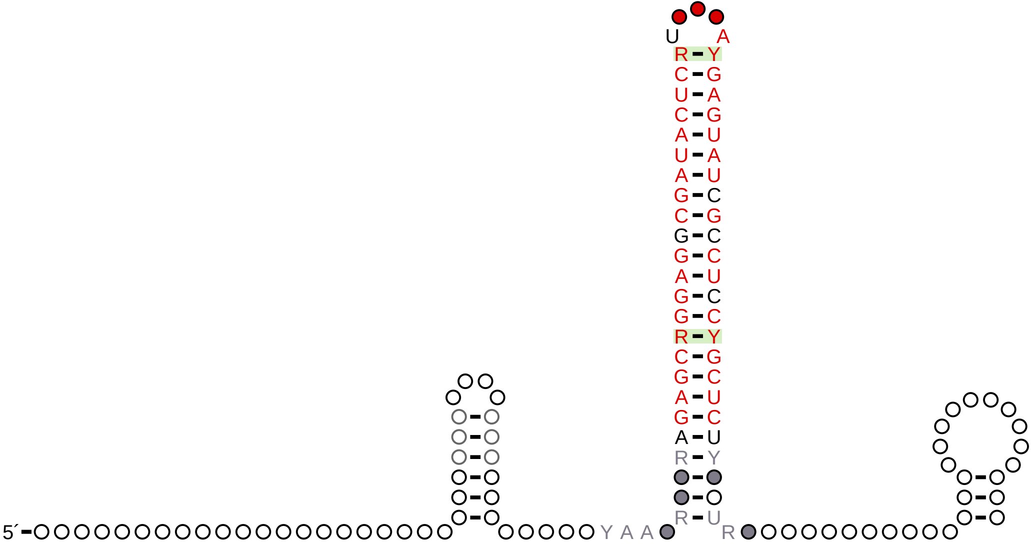

5´
U
A
R Y
C G
U A
C G
A U
U A
A U
G C
C G
G C
G C
A U
G C
G C
R Y
C G
G C
A U
G C
A U
R Y
R U
Y A A
R

HMP2_4209_1

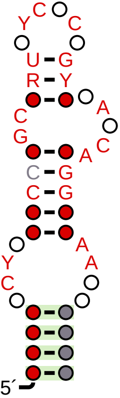

HMP2_421_1

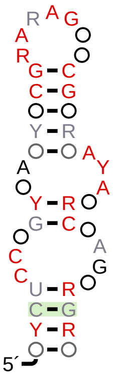

HMP2_4219_1

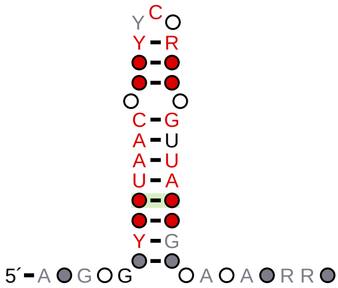

HMP2_4226_1

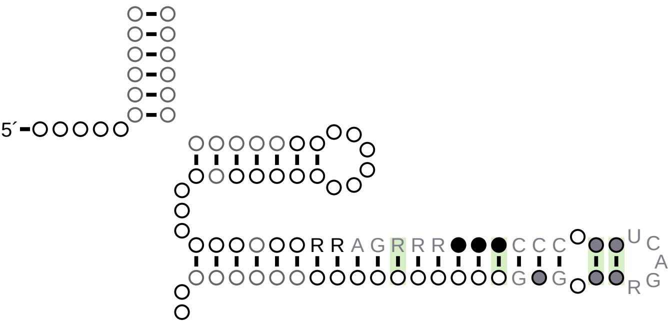

HMP2_4235_1

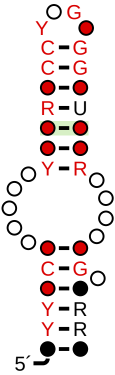

HMP2_4237_1

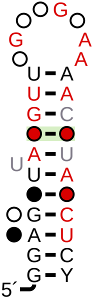

HMP2_4241_1

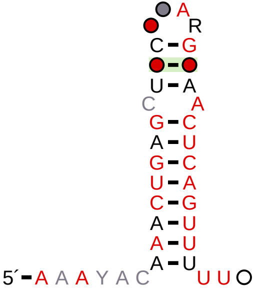

HMP2_4244_1

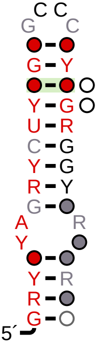

HMP2_4246_1

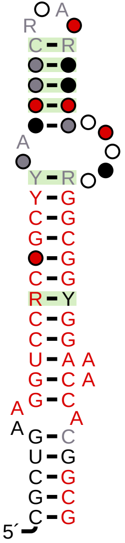

HMP2_4247_1

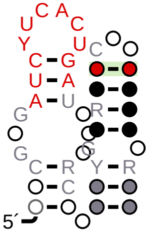

HMP2_4248_1

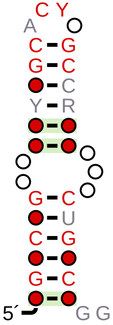

HMP2_4251_1

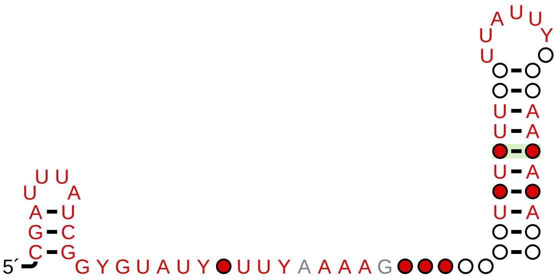

HMP2_426_1

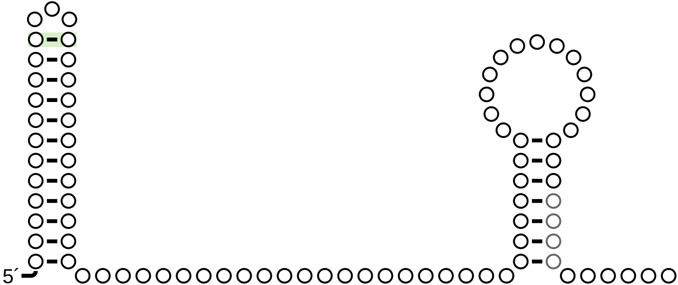

HMP2_4262_1

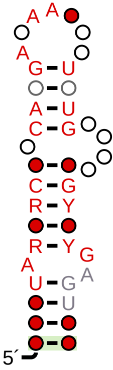

HMP2_4266_1

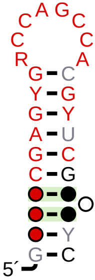

HMP2_4267_1

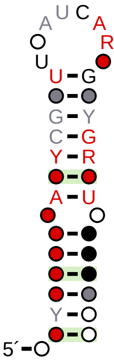

HMP2_4272_1

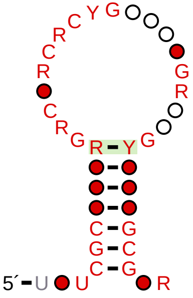

HMP2_4277_1

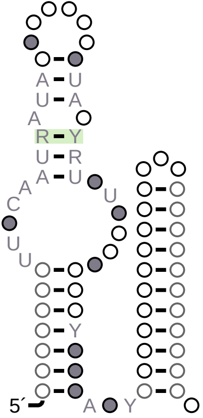

HMP2_4278_1

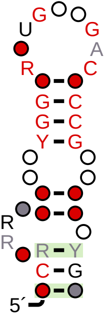

HMP2_4280_1

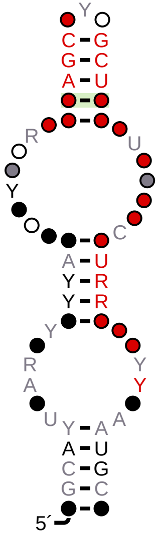

HMP2_4282_1

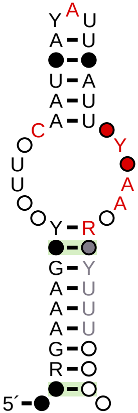

HMP2_4288_1

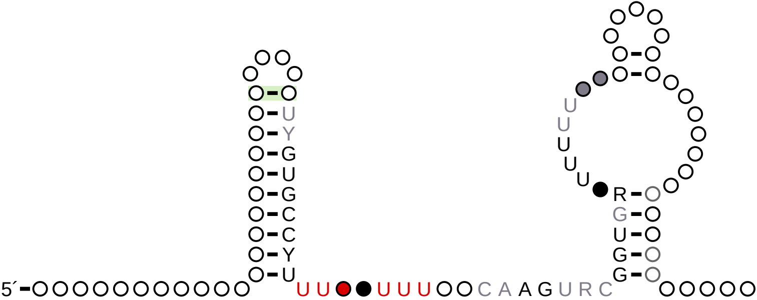

HMP2_429_1

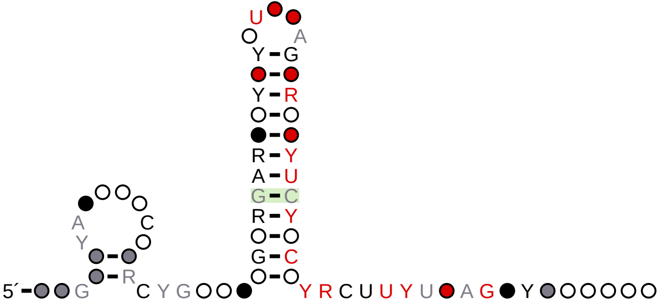

HMP2_4299_1

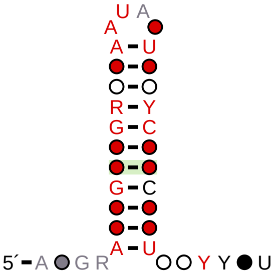

HMP2_4301_1

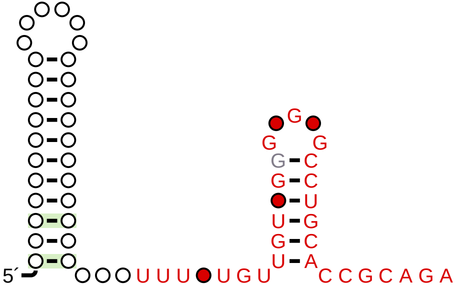

HMP2_4308_1

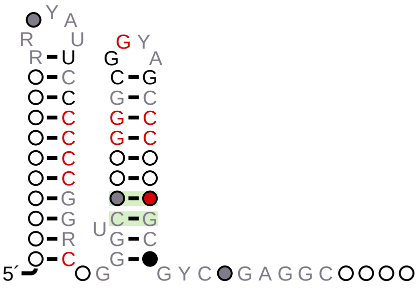

HMP2_4309_1

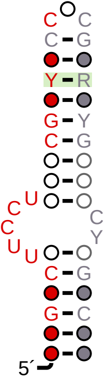

HMP2_4312_1

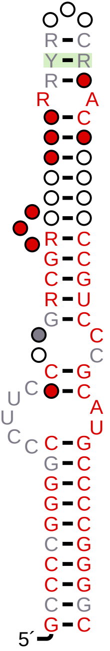

HMP2_4313_1

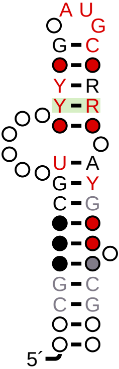

HMP2_4321_1

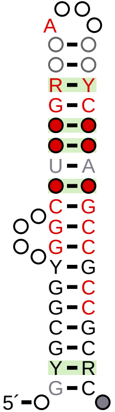

HMP2_4322_1

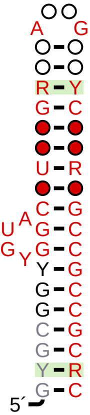

HMP2_4324_1

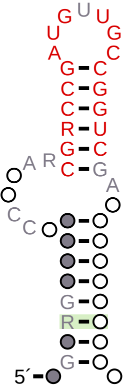

HMP2_4325_1

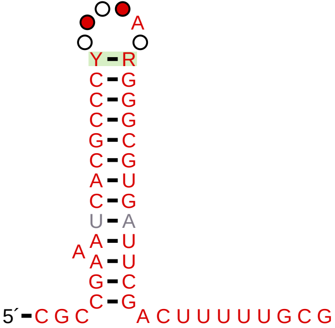

HMP2_4327_1

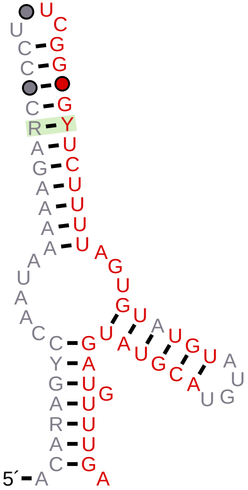

HMP2_4329_1

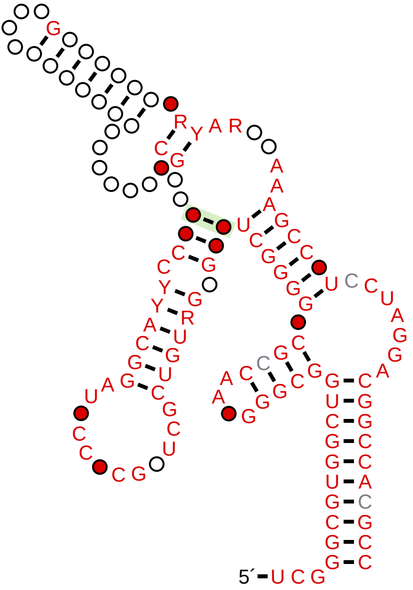

HMP2_4330_1

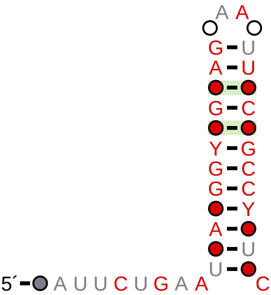

HMP2_4331_1

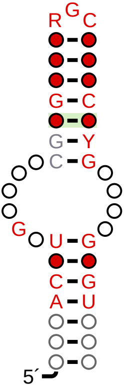

HMP2_4333_1

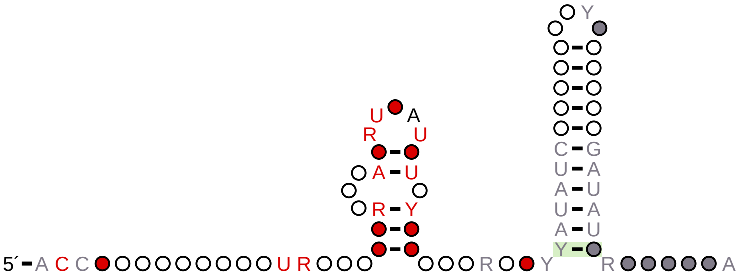

HMP2_4342_1

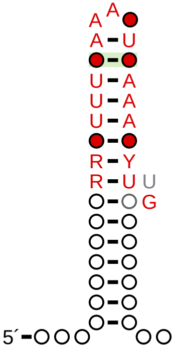

HMP2_4345_1

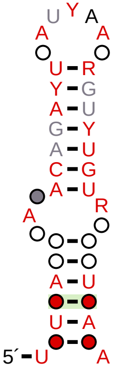

HMP2_4346_1

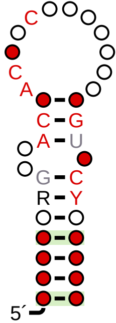

HMP2_4348_1

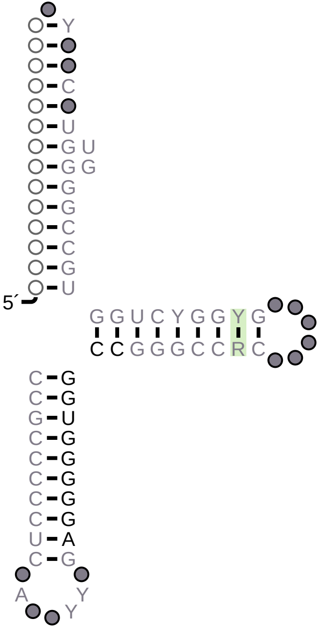

Y
C
U
G U
G G
G
G
C
C
G
U
5´
G G U C Y G G Y G
C C G G G C C R C
C G
C G
G U
C G
C G
C G
C G
C G
U A
C G
A Y
Y

HMP2_435_1

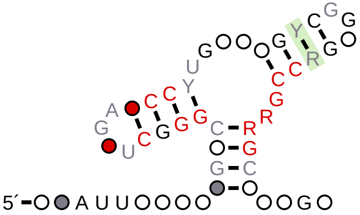

HMP2_4350_1

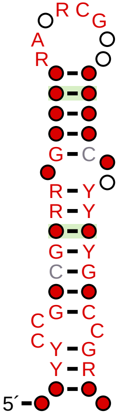

HMP2_4356_1

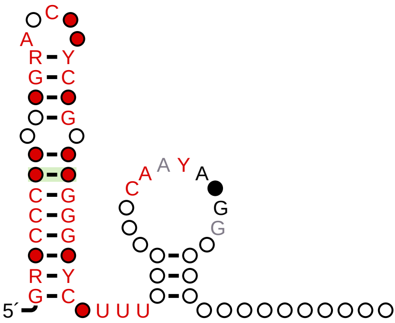

HMP2_4369_1

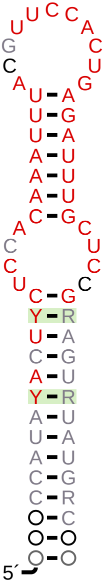

HMP2_4374_1

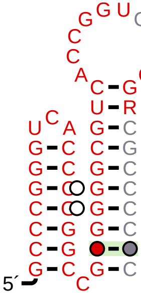

HMP2_438_1

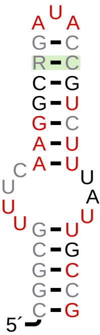

HMP2_4392_1

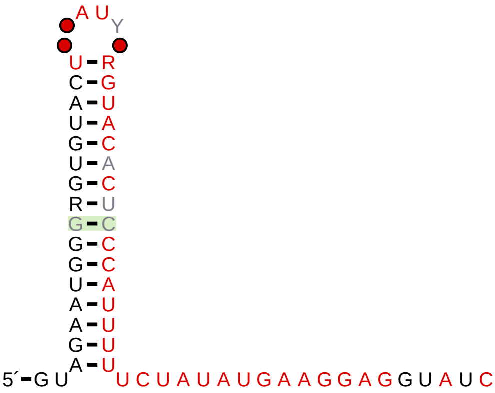

A U
Y
U R
C G
A U
U A
G C
U A
G C
R U
G C
G C
G C
U A
A U
A U
G U
A U
5´ G U
U C U A U A U G A A G G A G G U A U C

HMP2_4397_1

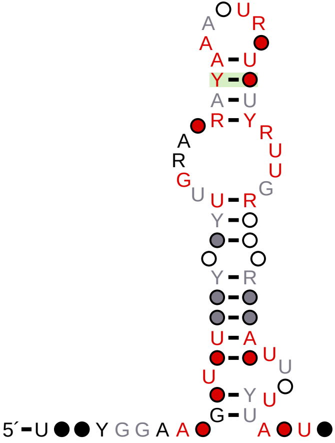

HMP2_4405_1

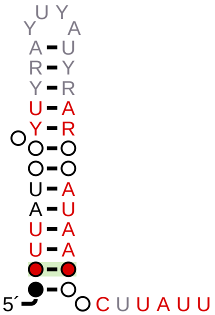

HMP2_4411_1

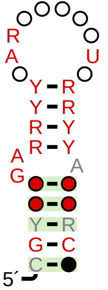

HMP2_4416_1

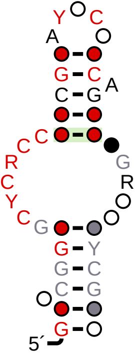

HMP2_4419_1

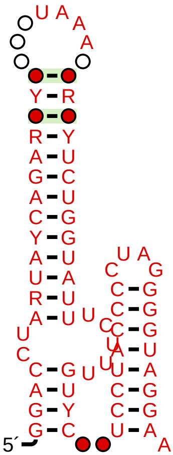

HMP2_4425_1

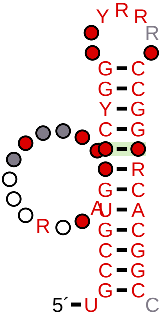

HMP2_4429_1

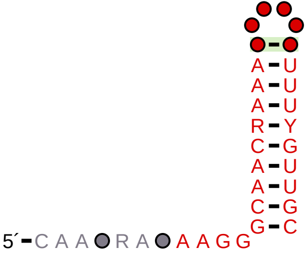

HMP2_443_1

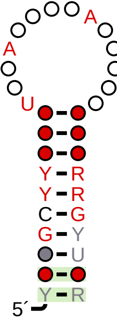

HMP2_4431_1

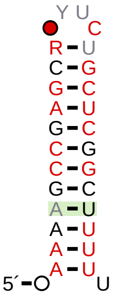

HMP2_4436_1

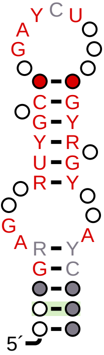

HMP2_4438_1

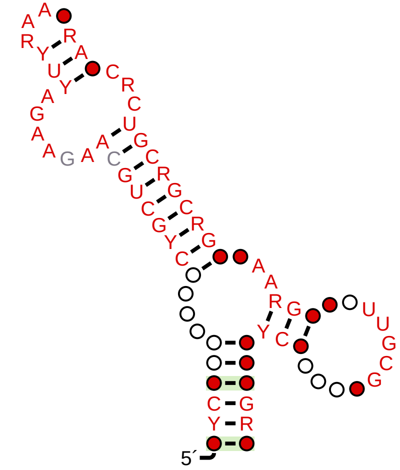

HMP2_4442_1

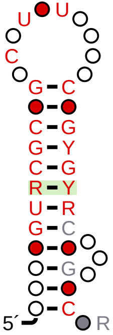

HMP2_4446_1

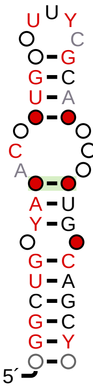

HMP2_445_1

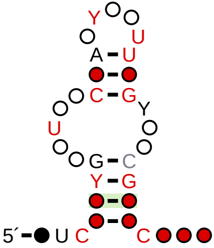

HMP2_4451_1

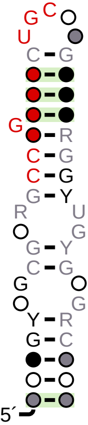

HMP2_4461_1

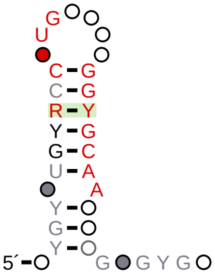

HMP2_4468_1

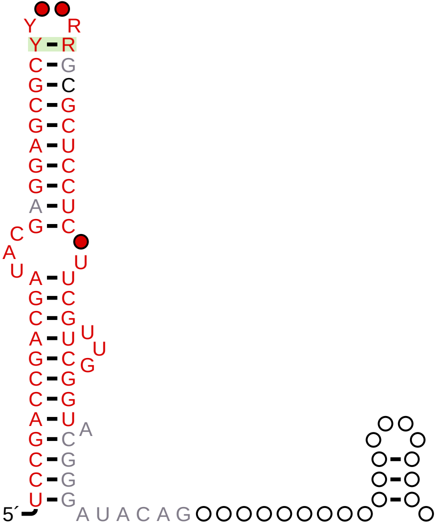

Y R
Y R
C G
G C
C G
G C
A U
G C
G C
A U
G C
C
A
U
U
A U
G C
C G
A U
G C
C G
C G
A U
G C
C G
C G
U G
U U G
A
5´
AUACAG

HMP2_4472_1

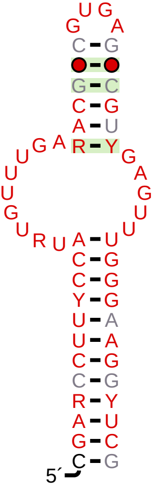

HMP2_4473_1

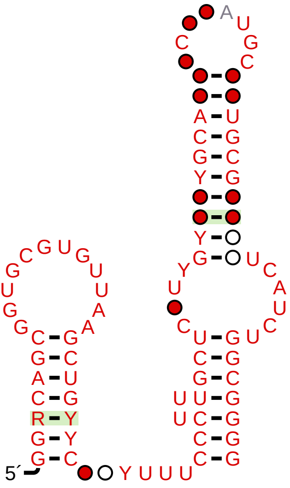

HMP2_4476_1

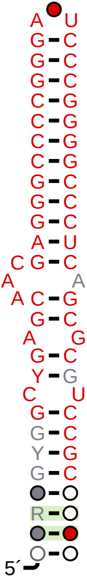

HMP2_4481_1

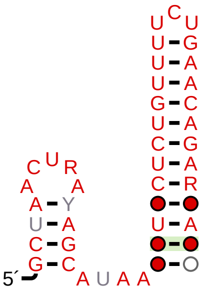

HMP2_4482_1

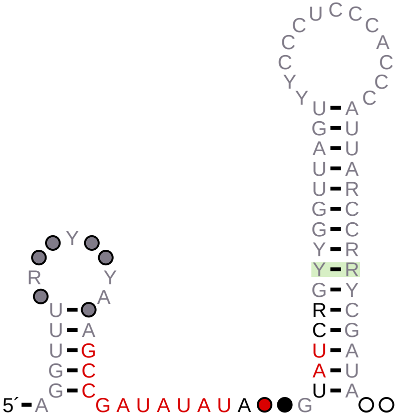

HMP2_4486_1

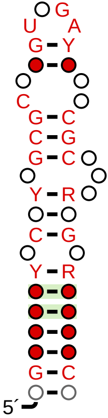

HMP2_4493_1

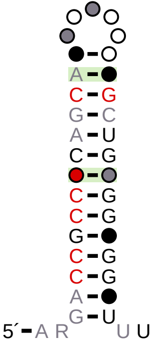

HMP2_4495_1

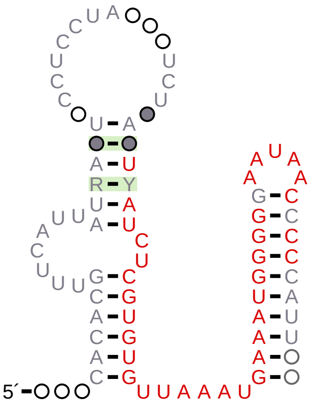

HMP2_4496_1

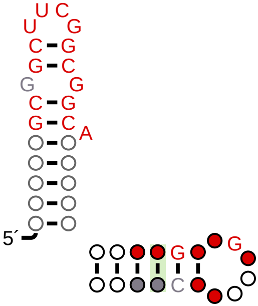

HMP2_450_1

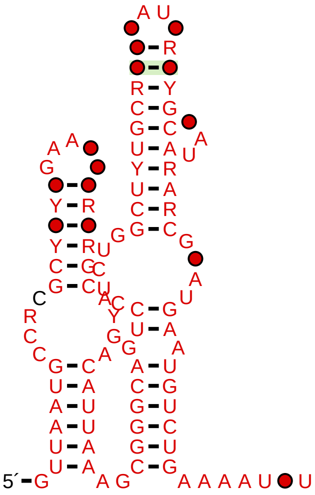

HMP2_4505_1

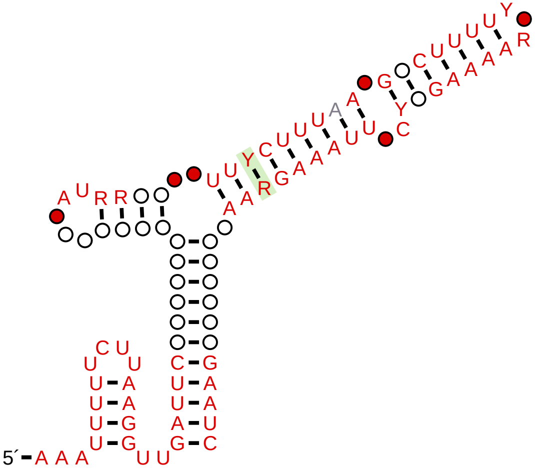

HMP2_4507_1

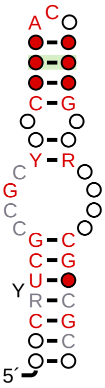

HMP2_451_1

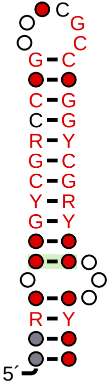

HMP2_4512_1

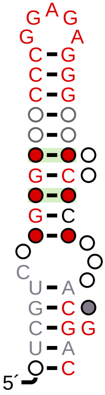

HMP2_4521_1

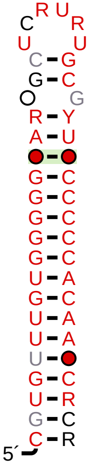

HMP2_4523_1

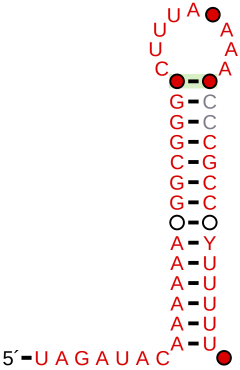

HMP2_4524_1

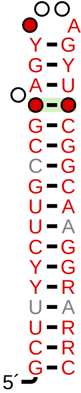

HMP2_4527_1

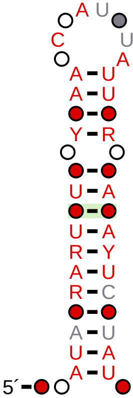

HMP2_4534_1

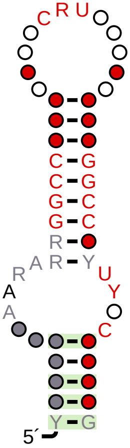

HMP2_454_1

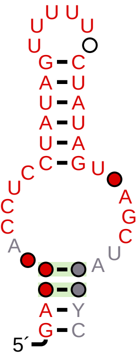

HMP2_4543_1

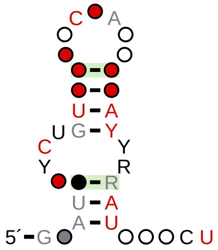

HMP2_4547_1

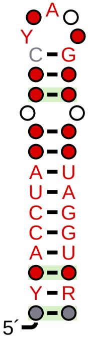

HMP2_4552_1

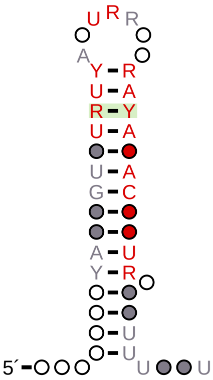

HMP2_4555_1

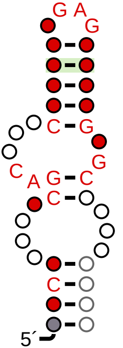

HMP2_4560_1

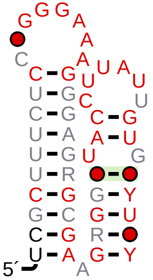

HMP2_4561_1

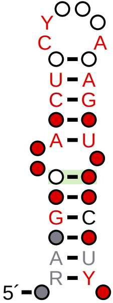

HMP2_4572_1

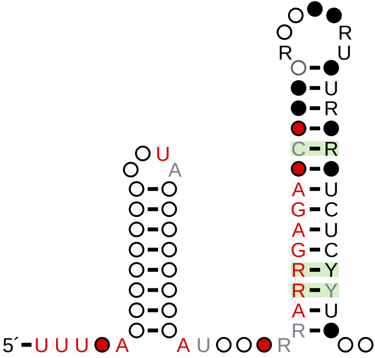

HMP2_458_1

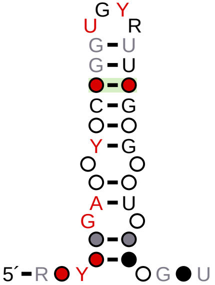

HMP2_4583_1

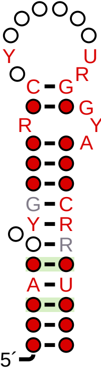

HMP2_4585_1

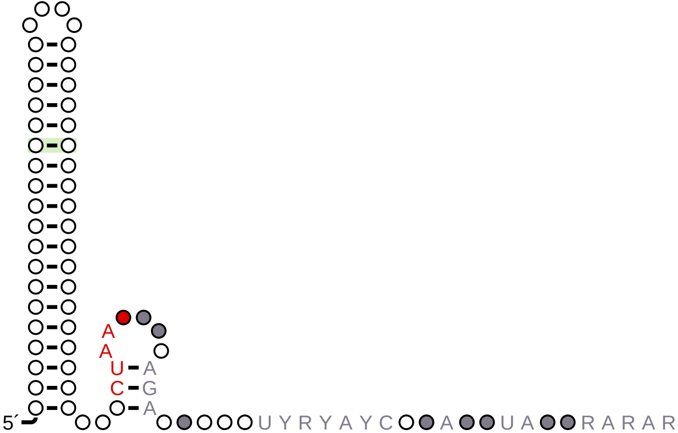

HMP2_4594_1

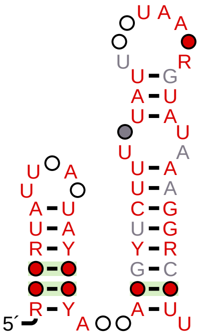

HMP2_460_1

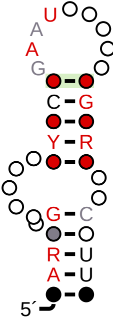

HMP2_4601_1

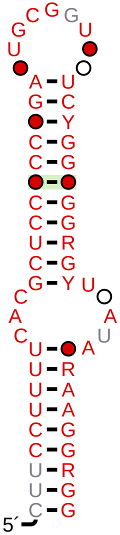

HMP2_4602_1

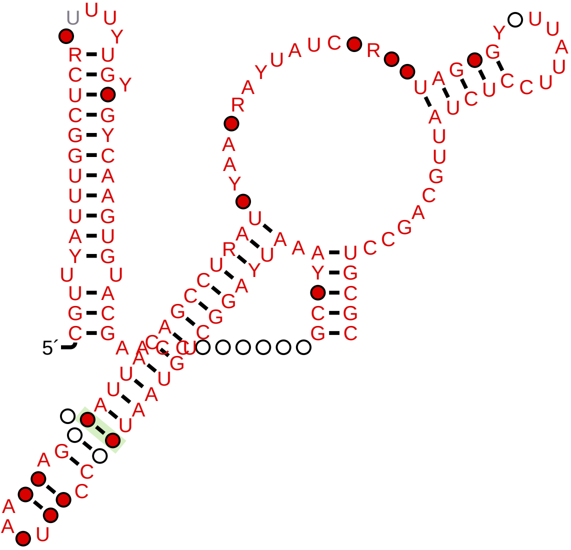

HMP2_4604_1

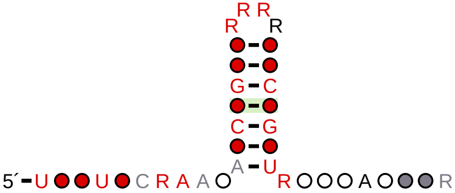

HMP2_4606_1

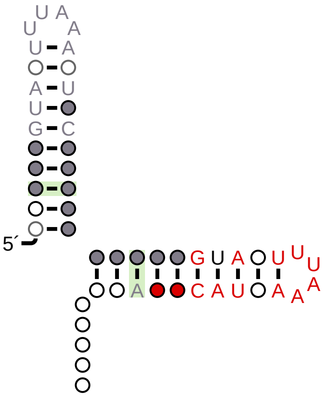

HMP2_461_1

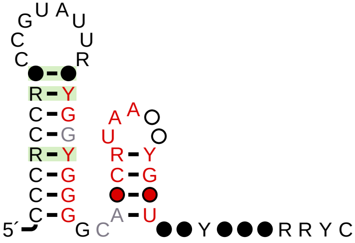

HMP2_4610_1

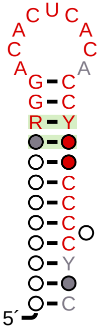

HMP2_4612_1

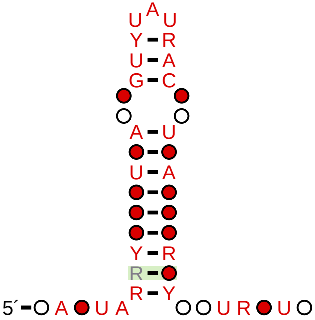

HMP2_4613_1

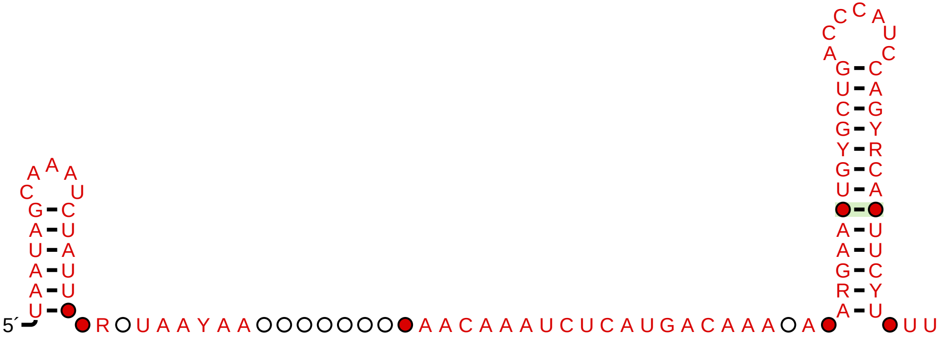

HMP2_4616_1

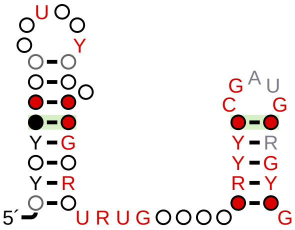

HMP2_4619_1

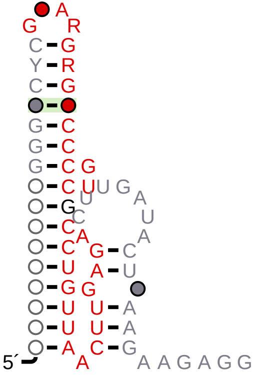

HMP2_4624_1

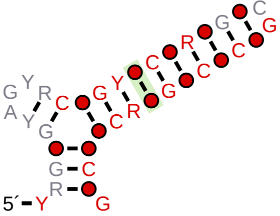

HMP2_4627_1

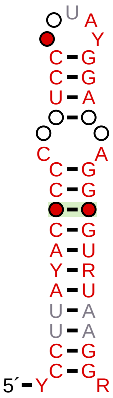

HMP2_4642_1

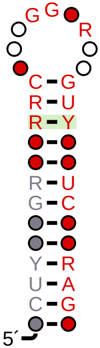

HMP2_4646_1

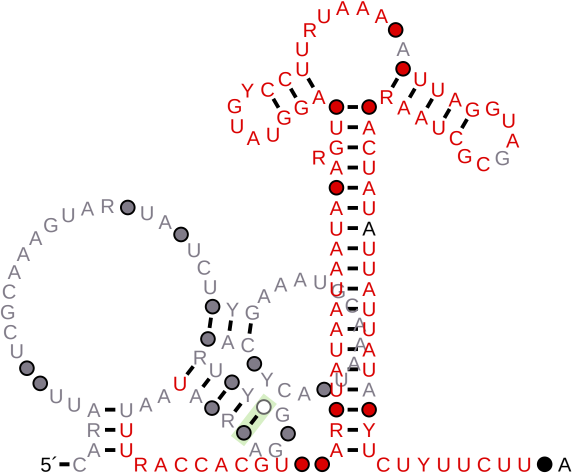

HMP2_4648_1

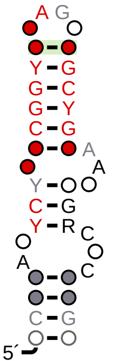

HMP2_465_1

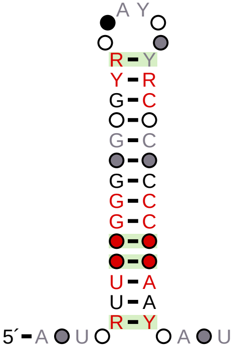

HMP2_4658_1

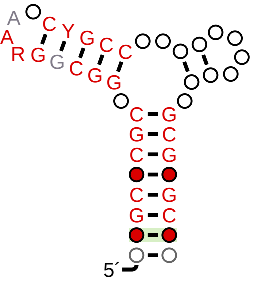

HMP2_4677_1

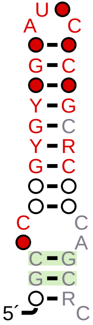

HMP2_4679_1

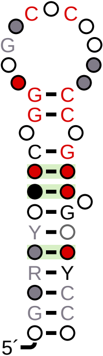

HMP2_4681_1

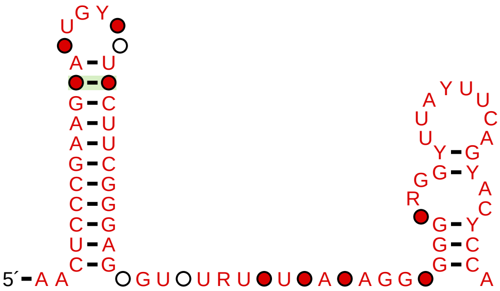

HMP2_4683_1

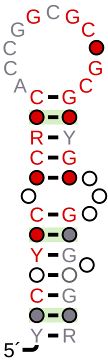

HMP2_4690_1

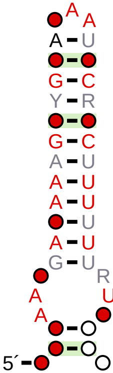

HMP2_4692_1

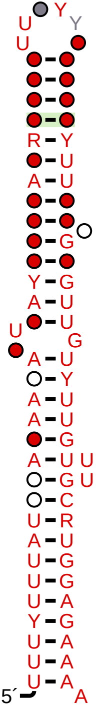

HMP2_4694_1

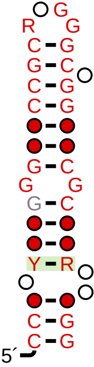

HMP2_47_1

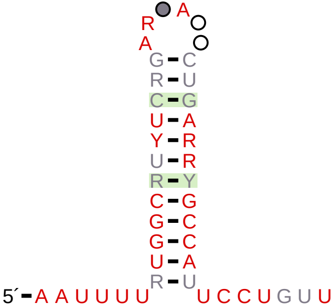

HMP2_4700_1

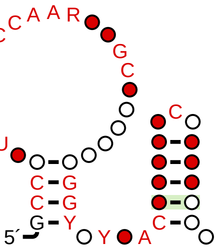

HMP2_4708_1

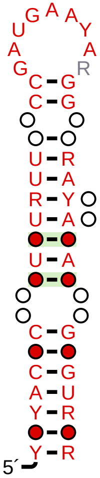

HMP2_4709_1

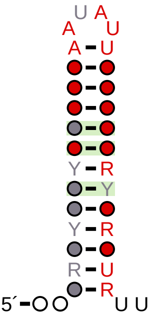

HMP2_471_1

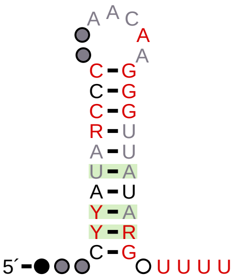

HMP2_4712_1

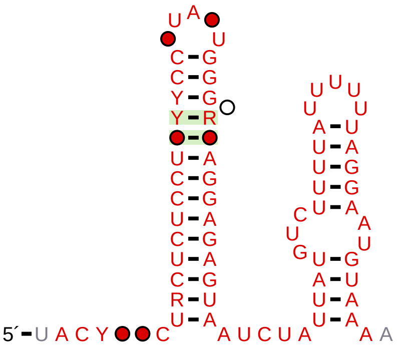

HMP2_4714_1

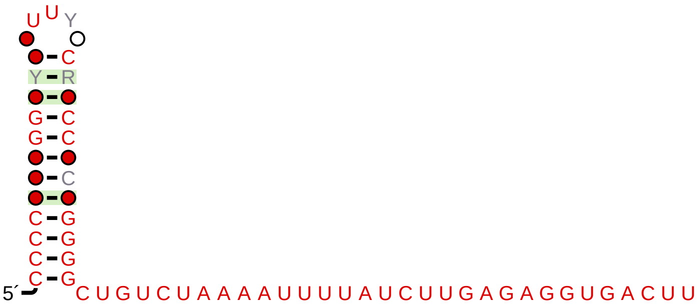
U U Y
C
Y R
G C
G C
C
C G
C G
C G
C G
5´
C U G U C U A A A A U U U U A U C U U G A G A G G U G A C U U

HMP2_4715_1

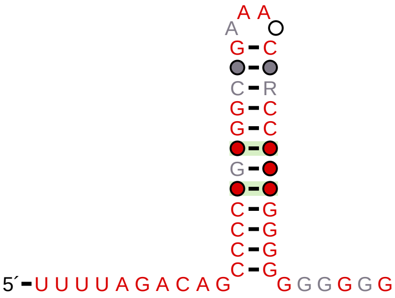

HMP2_4720_1

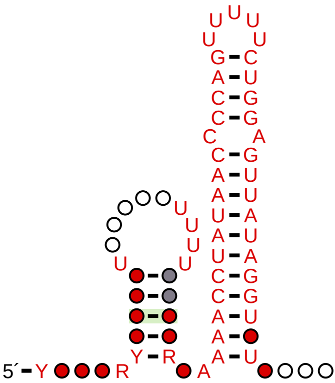

HMP2_4733_1

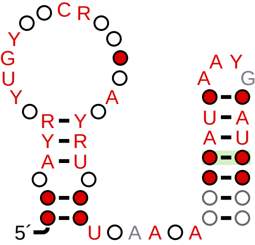

HMP2_474_1

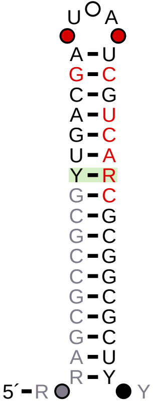

HMP2_4750_1

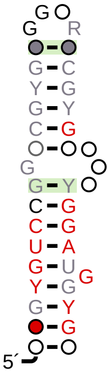

HMP2_4756_1

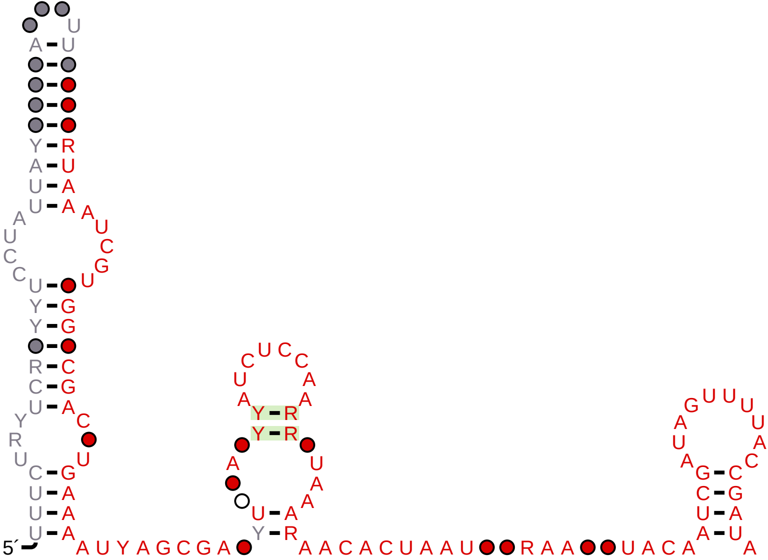

HMP2_4765_1

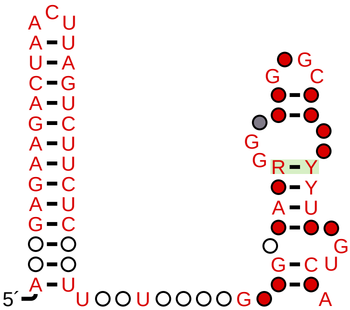

HMP2_4770_1

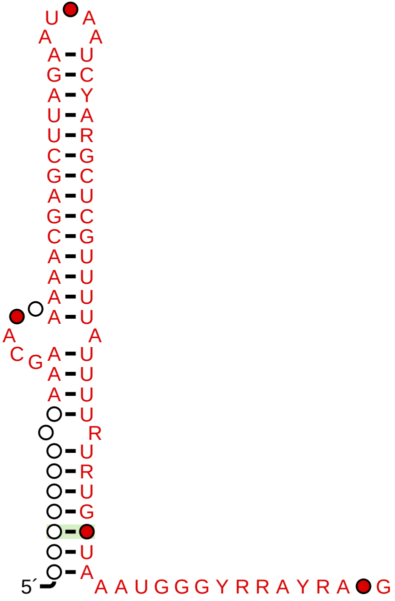

HMP2_4781_1

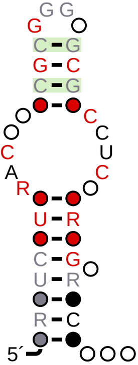

HMP2_4791_1

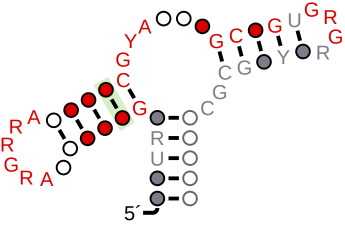

HMP2_4795_1

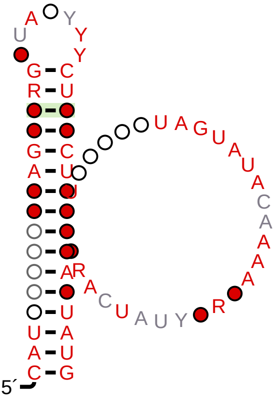

HMP2_4798_1

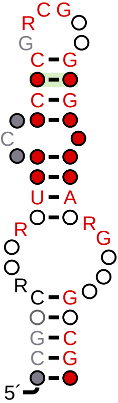

HMP2_4799_1

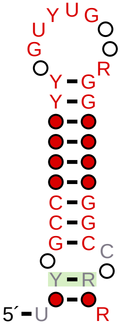

HMP2_4803_1

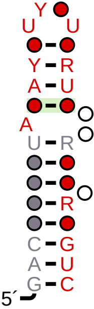

HMP2_4805_1

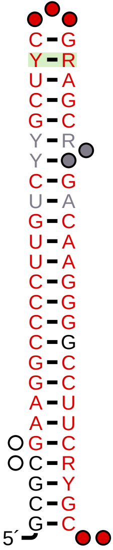

HMP2_4809_1

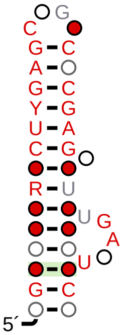

HMP2_4825_1

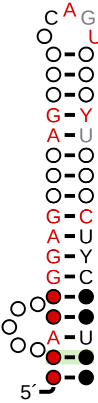

HMP2_4834_1

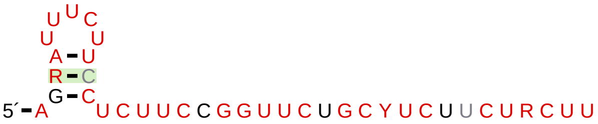

HMP2_4837_1

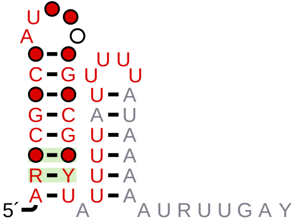

HMP2_4838_1

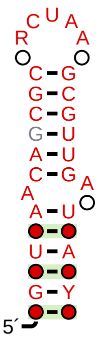

HMP2_4840_1

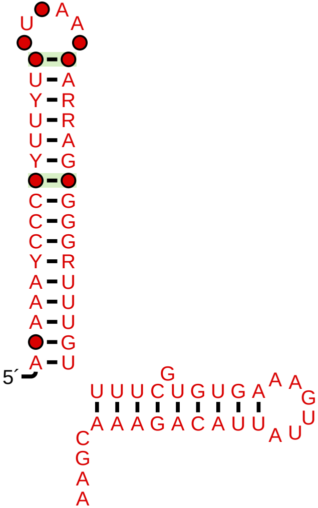

HMP2_4842_1

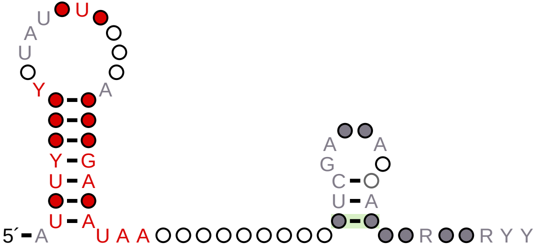

HMP2_4843_1

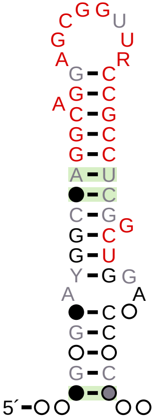

HMP2_4846_1

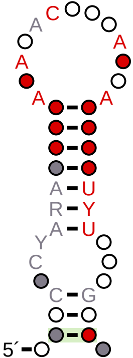

HMP2_4847_1

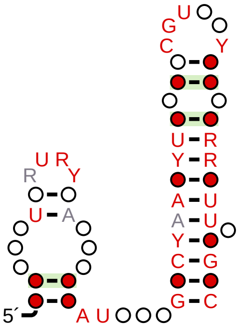

HMP2_4850_1

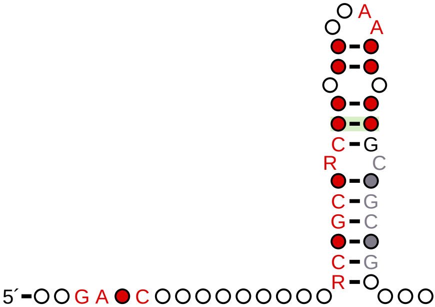

HMP2_4865_1

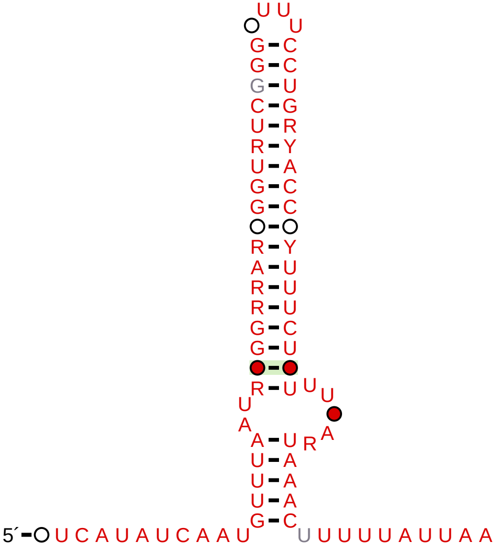

HMP2_4875_1

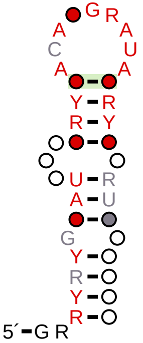

HMP2_4886_1

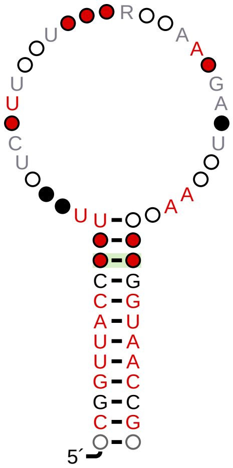

HMP2_4890_1

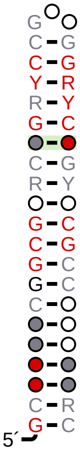

HMP2_4895_1

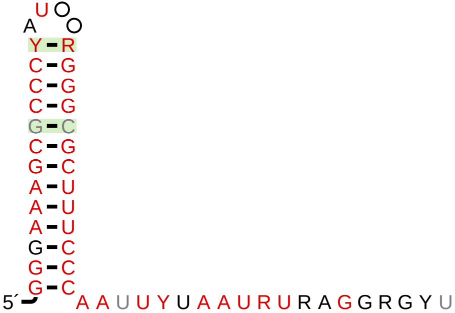

U O
A O
Y-R
C-G
C-G
C-G
G-C
C-G
G-C
A-U
A-U
A-U
G-C
G-C
G-C
5´ A A U U Y U A A U R U R A G G R G Y U

HMP2_4898_1

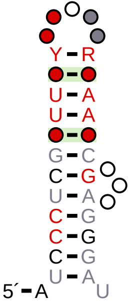

HMP2_4899_1

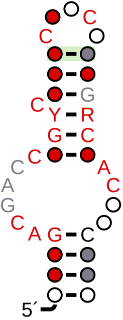

HMP2_49_1

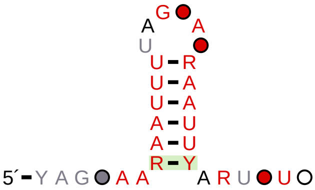

HMP2_491_1

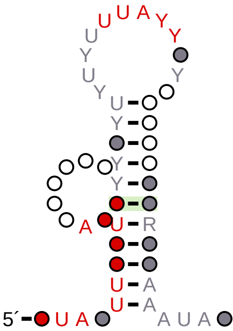

HMP2_4917_1

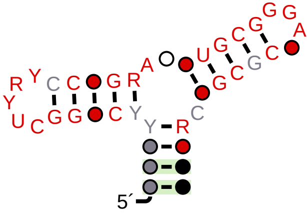

HMP2_492_1

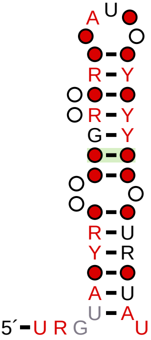

HMP2_4921_1

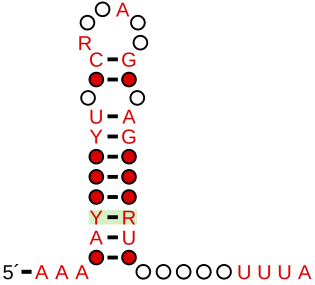

HMP2_4928_1

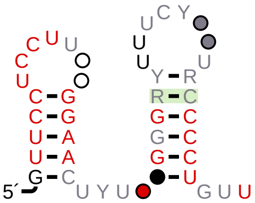

HMP2_493_1

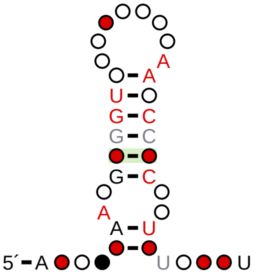

HMP2_4930_1

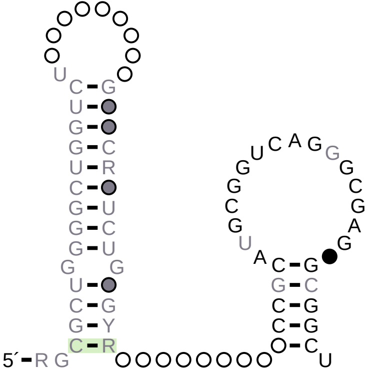

HMP2_4945_1

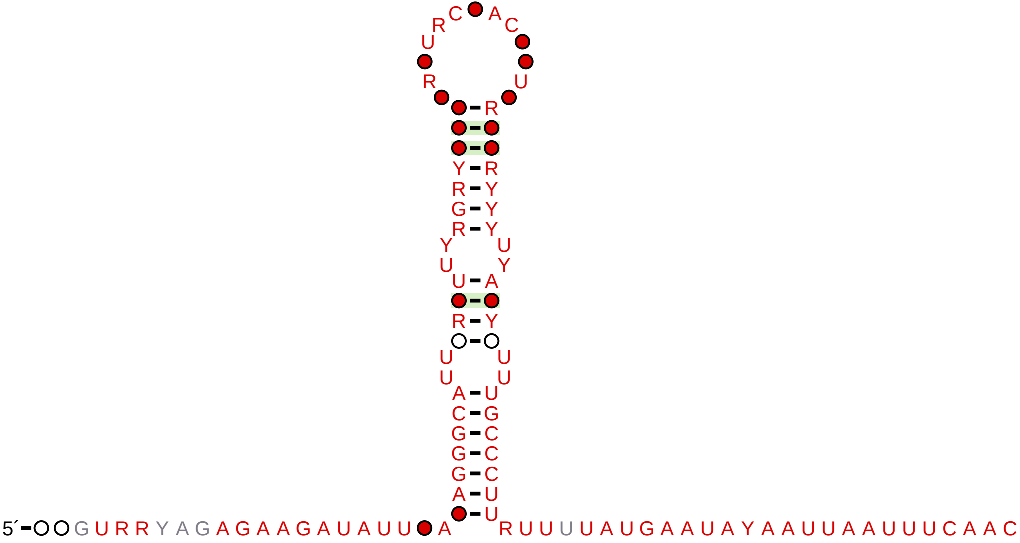

HMP2_4946_1

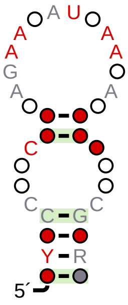

HMP2_495_1

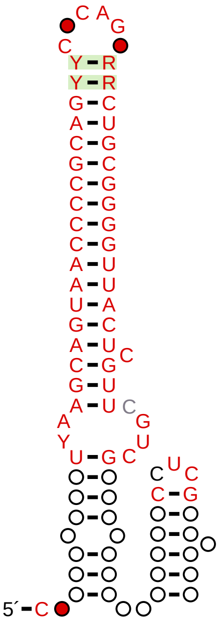

HMP2_4962_1

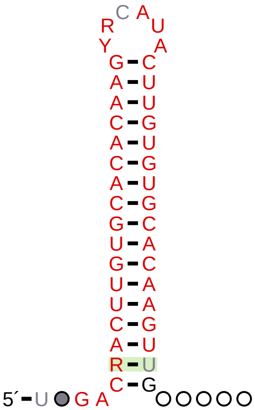

HMP2_4972_1

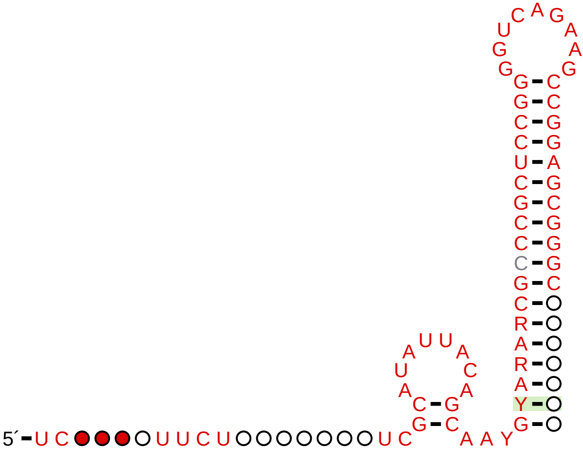

HMP2_4974_1

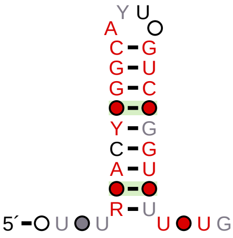

HMP2_4975_1

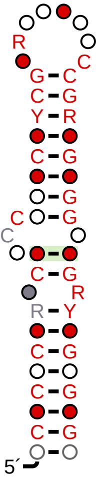

HMP2_4977_1

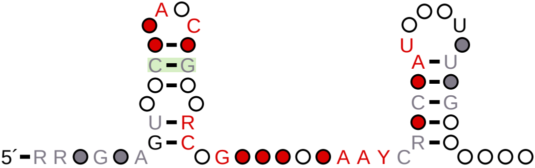

HMP2_4979_1

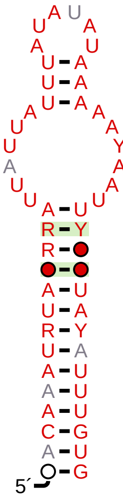

HMP2_4980_1

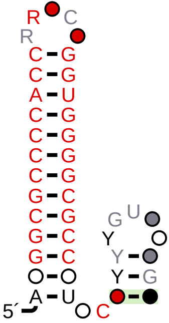

HMP2_4981_1

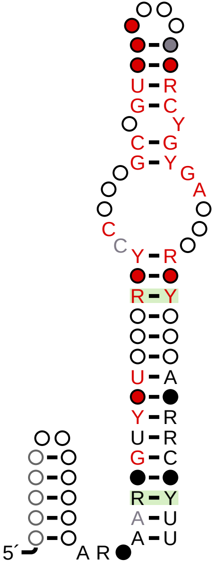

HMP2_4984_1

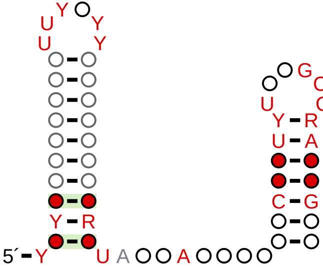

HMP2_499_1

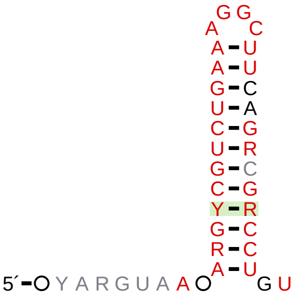

HMP2_4991_1

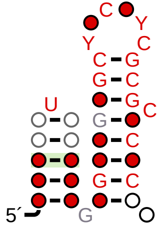

HMP2_4998_1

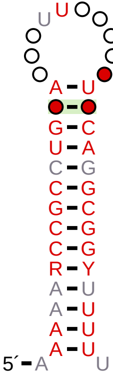

HMP2_50_1

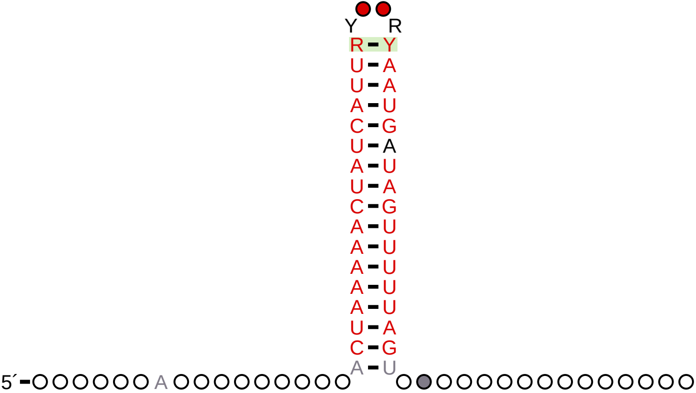

HMP2_500_1

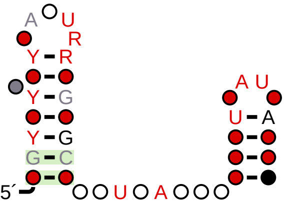

HMP2_501_1

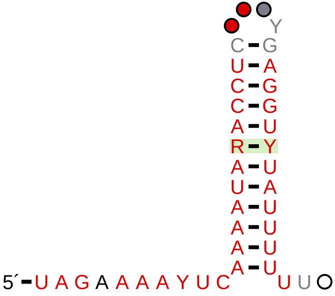

HMP2_5018_1

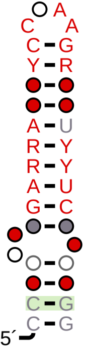

HMP2_5019_1

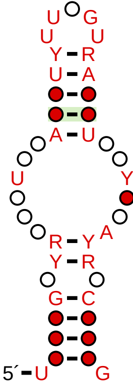

HMP2_5028_1

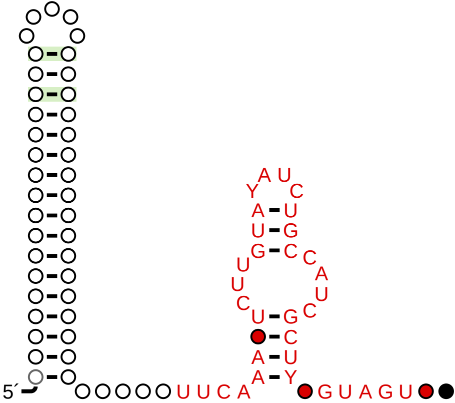

HMP2_5035_1

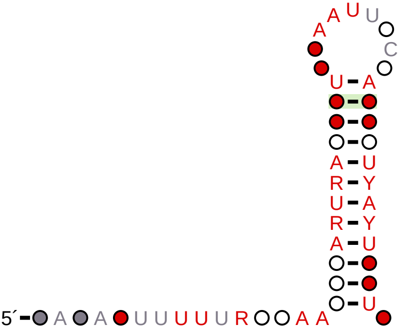

HMP2_5042_1

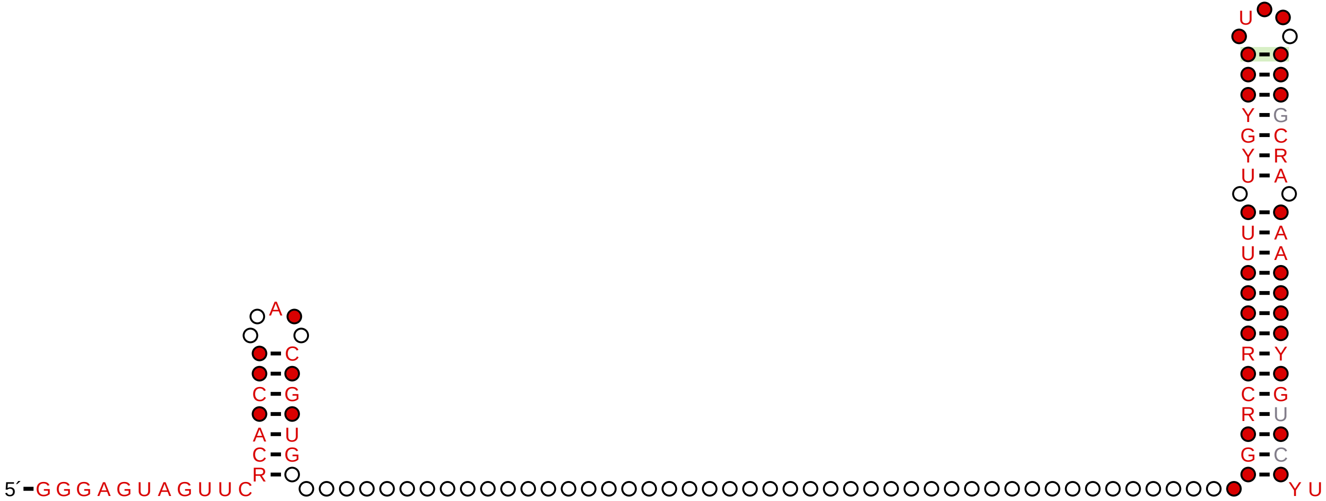

HMP2_5043_1

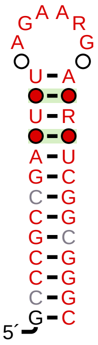

HMP2_505_1

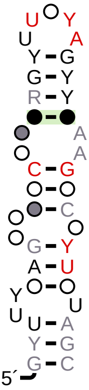

HMP2_5054_1

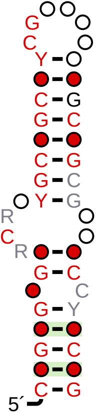

HMP2_5057_1

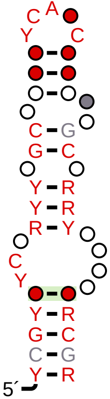

HMP2_506_1

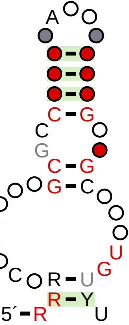

HMP2_5062_1

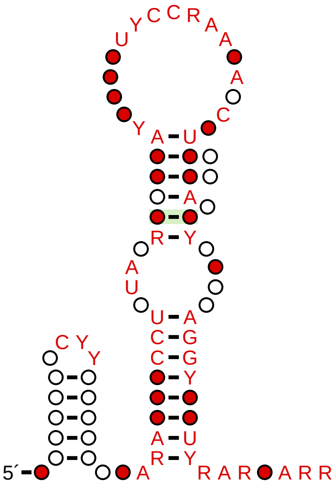

HMP2_5066_1

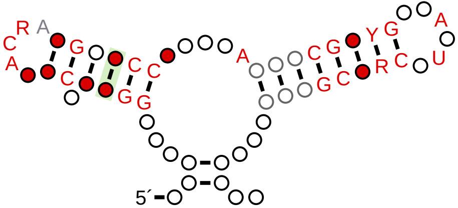

HMP2_5067_1

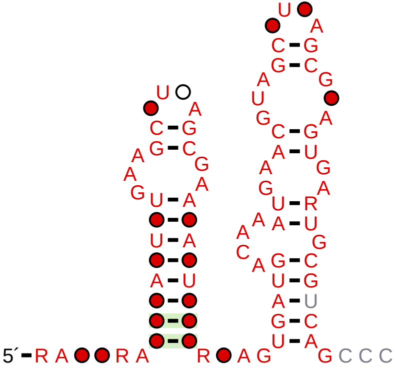
5´
R A R A R A G G C C C
U A C G G C A A G U A C G A
U A C G G C A U G C G A A U G A G U R A A U G A C A G C U G A U G C U A

HMP2_5069_1

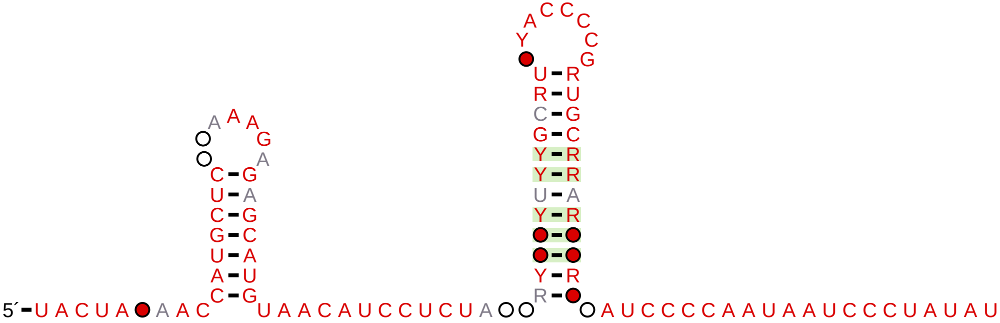

5´
U A C U A
A A C
U A A C A U C C U C U A
A U C C C C A A U A A U C C C U A U A U
A A A
G
A
C G
U A
C G
G C
U A
A U
C G
A C C C
Y C
C
G
U R
R U
C G
G C
Y R
Y R
U A
Y R
Y R
R

HMP2_5070_1

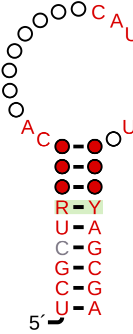

HMP2_5074_1

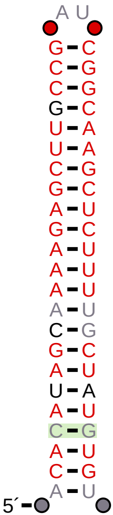

HMP2_5084_1

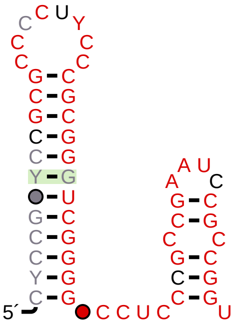

HMP2_5090_1

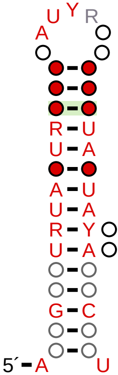

HMP2_5093_1

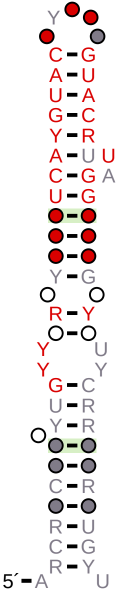

HMP2_5097_1

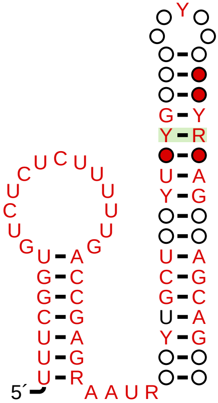

Y
G-Y
Y-R
U-A
Y-G
U-A
C-G
G-C
U-A
Y-G
CUCUU
UU
CU
UU
UG
GUAG
U-A
G-C
G-C
C-G
U-A
U-G
U-R
5´
AAUR

HMP2_5111_1

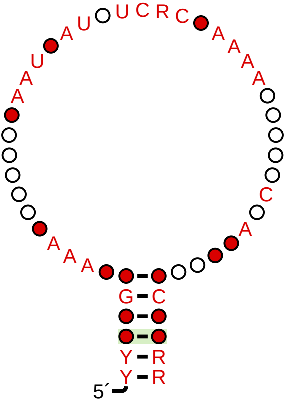

HMP2_5116_1

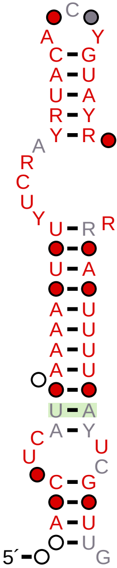

HMP2_5120_1

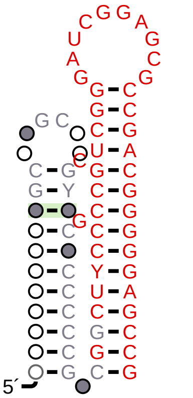

HMP2_5127_1

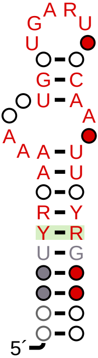

HMP2_5129_1

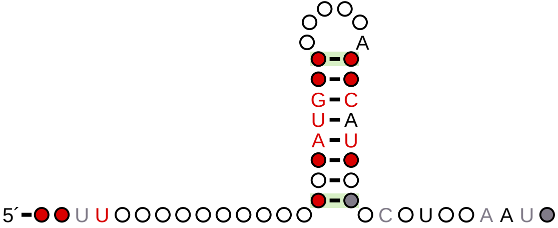

HMP2_5132_1

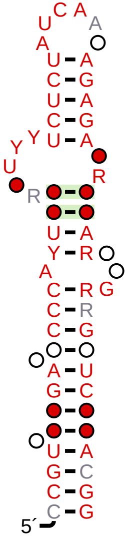

HMP2_5150_1

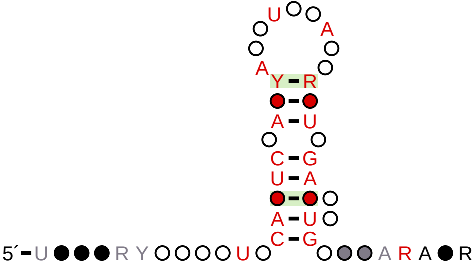

HMP2_5151_1

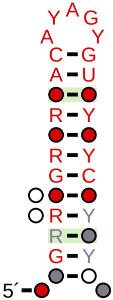

HMP2_5153_1

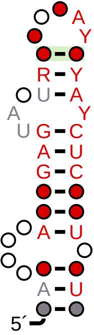

HMP2_5154_1

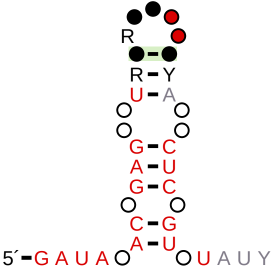

HMP2_5158_1

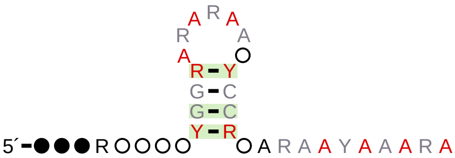

HMP2_5165_1

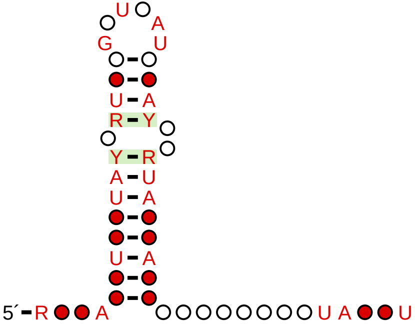

HMP2_5167_1

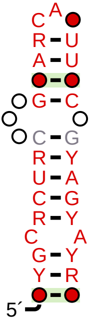

HMP2_5168_1

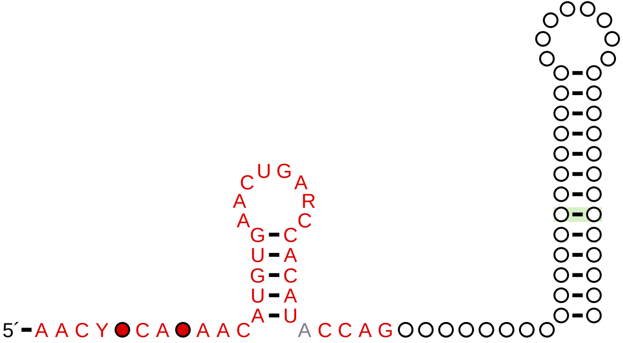

HMP2_5176_1

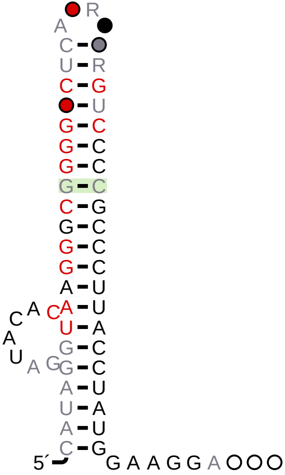

R
A
C
U R
C G
U
G C
G C
G C
G C
C G
G C
G C
G C
A U
C A C A U
A U A
C
A
U
A G G C
G C
A U
U A
A U
C G
5´
G A A G G A

HMP2_5177_1

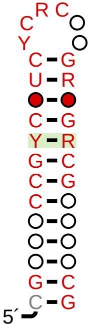

HMP2_5180_1

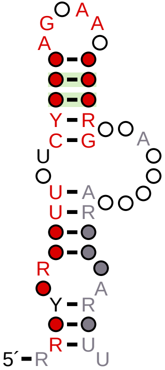

HMP2_5187_1

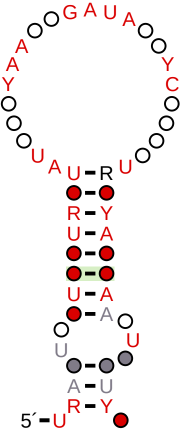

HMP2_5189_1

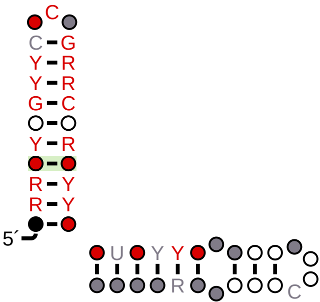

HMP2_5198_1

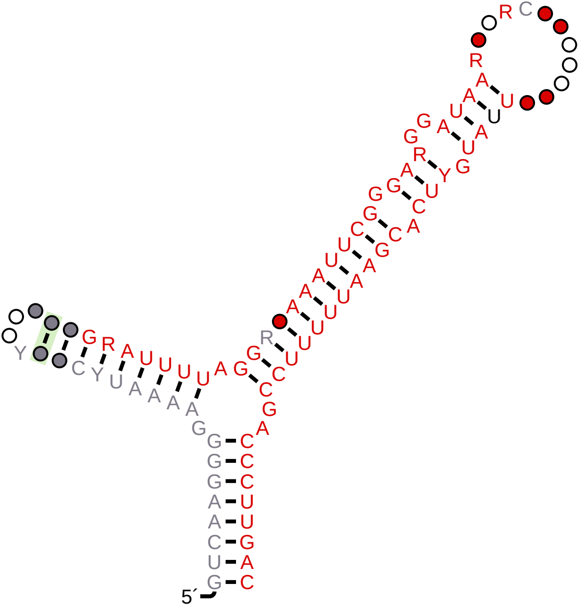

HMP2_5204_1

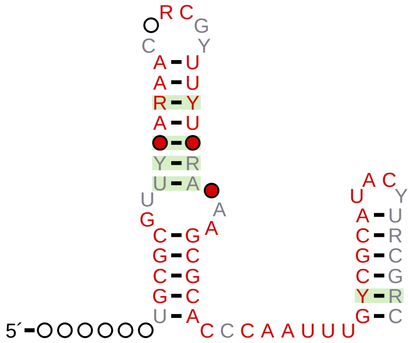

HMP2_5208_1

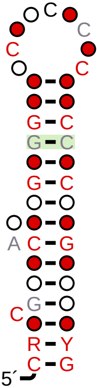

HMP2_5221_1

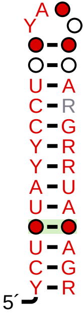

HMP2_5222_1

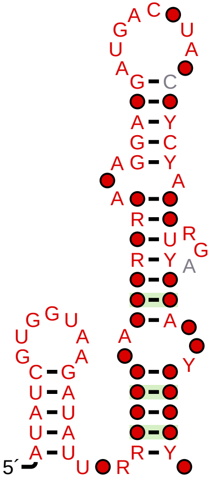

HMP2_5223_1

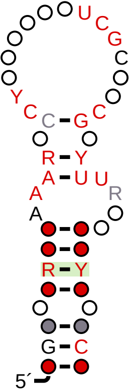

HMP2_5226_1

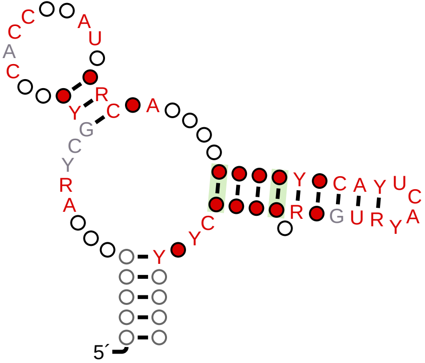

HMP2_5233_1

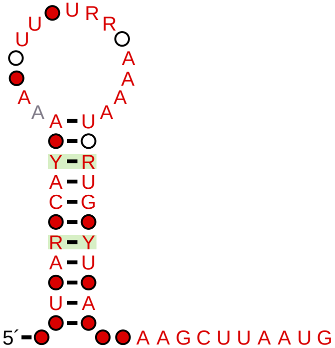

HMP2_5238_1

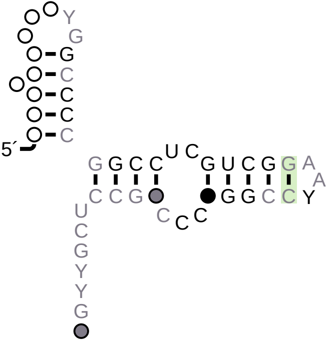

HMP2_524_1

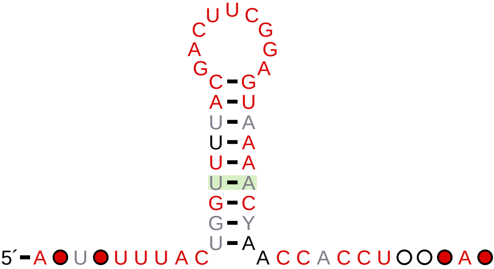

HMP2_5245_1

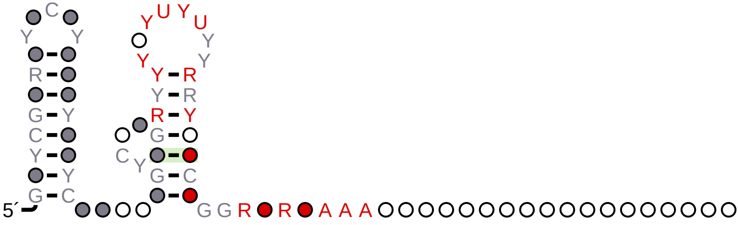

HMP2_5253_1

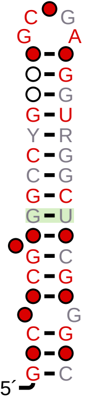

HMP2_5265_1

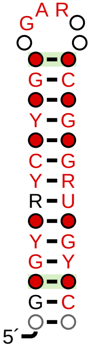

HMP2_5268_1

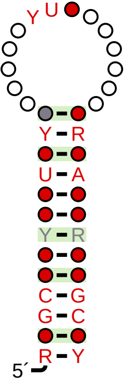

HMP2_5270_1

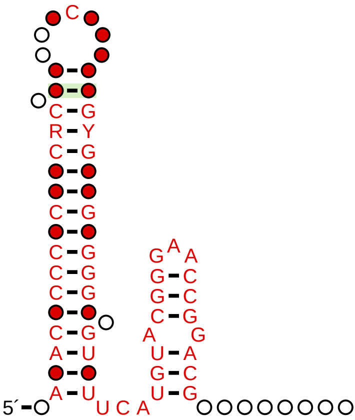

HMP2_5272_1

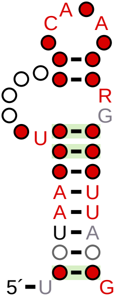

HMP2_5274_1

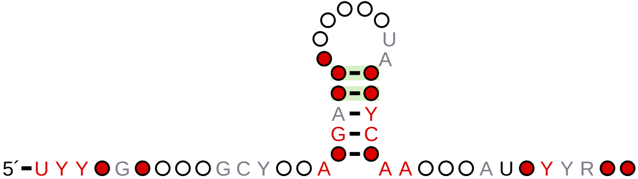

HMP2_5275_1

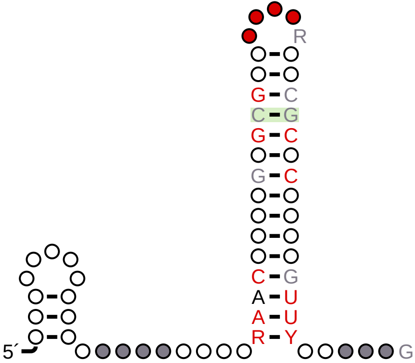

HMP2_5284_1

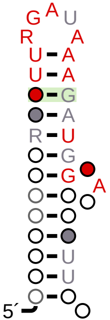

HMP2_529_1

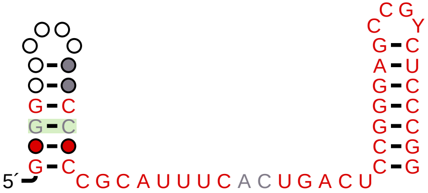

HMP2_5291_1

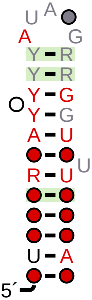

HMP2_5299_1

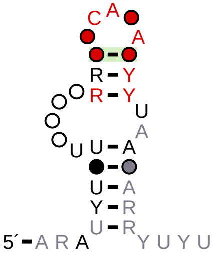

HMP2_53_1

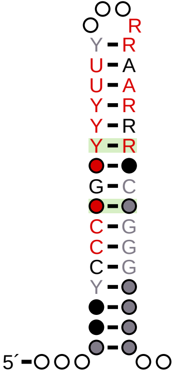

HMP2_530_1

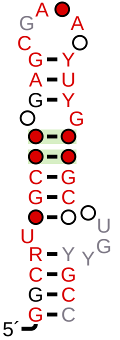

HMP2_5301_1

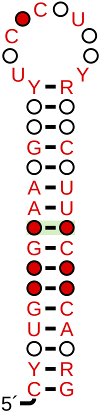

HMP2_5304_1

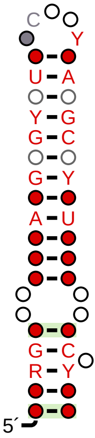

HMP2_5309_1

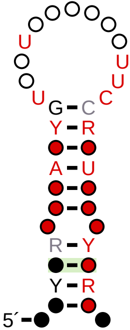

HMP2_5332_1

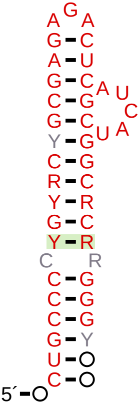

HMP2_5333_1

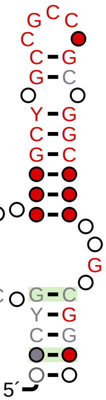

HMP2_5336_1

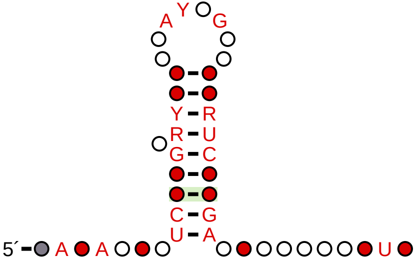

HMP2_5340_1

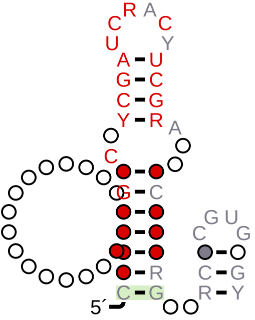

HMP2_5343_1

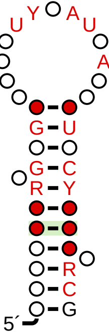

HMP2_5346_1

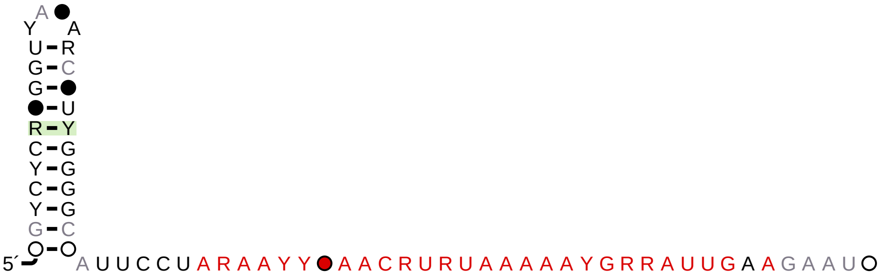

HMP2_5355_1

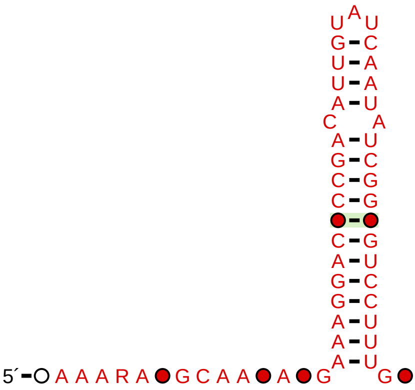

5´
A A A R A
G C A A
A
G
G
A A A G G A C C C G A C A U U G U
A U U C
U A U U A C U
G G C C G C
A C U G U U A A

HMP2_5357_1

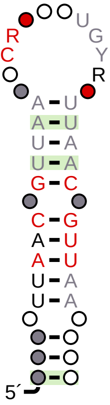

HMP2_5358_1

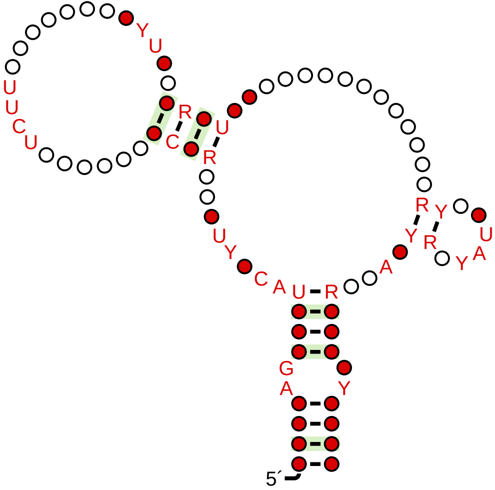

HMP2_5363_1

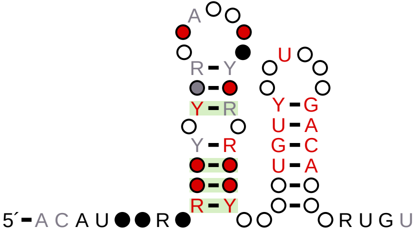

HMP2_5367_1

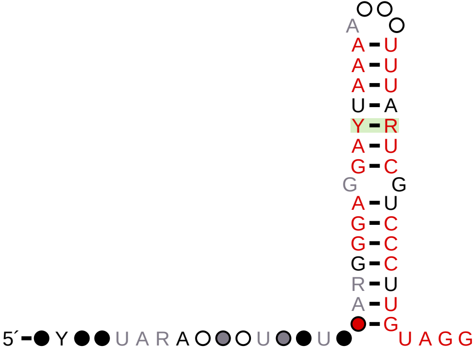

HMP2_5368_1

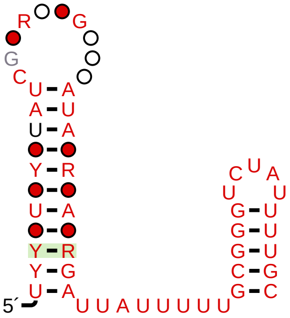

HMP2_5369_1

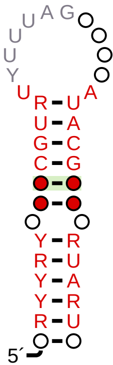

HMP2_5381_1

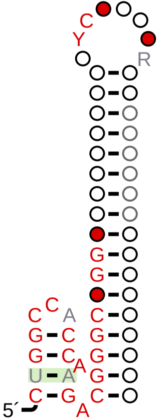

HMP2_5384_1

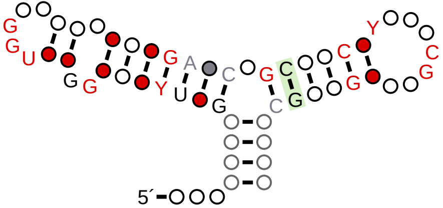

HMP2_5386_1

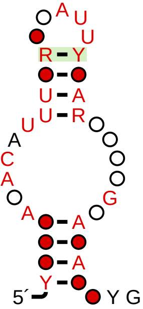

HMP2_5390_1

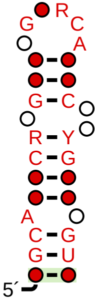

HMP2_5392_1

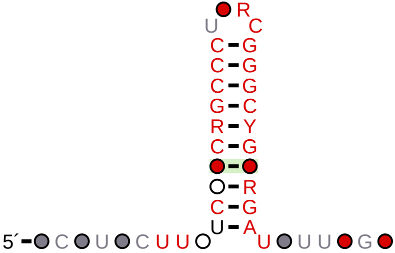

HMP2_5397_1

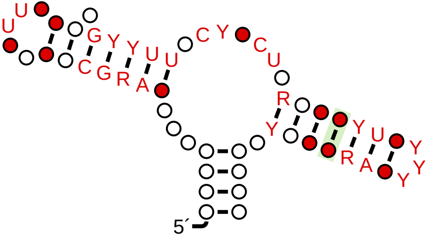

HMP2_540_1

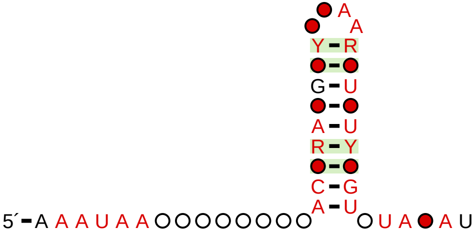

HMP2_5405_1

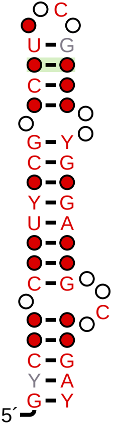

HMP2_5409_1

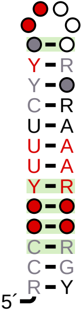

HMP2_5411_1

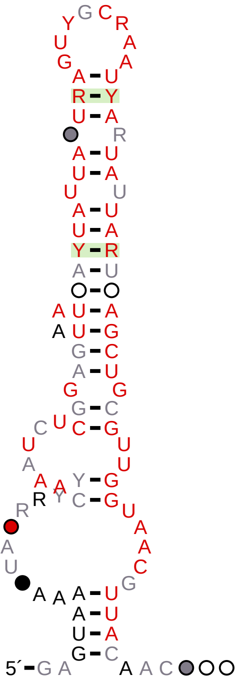

HMP2_5421_1

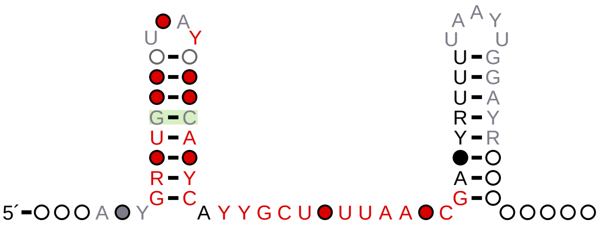

HMP2_5423_1

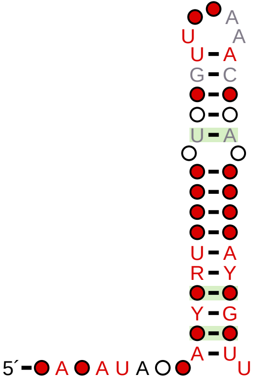

HMP2_5429_1

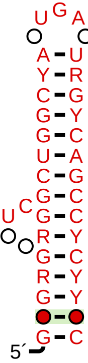

HMP2_5434_1

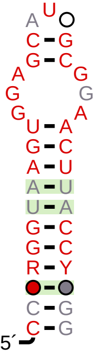

HMP2_5452_1

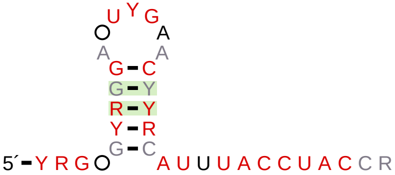

HMP2_5454_1

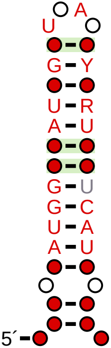

HMP2_5461_1

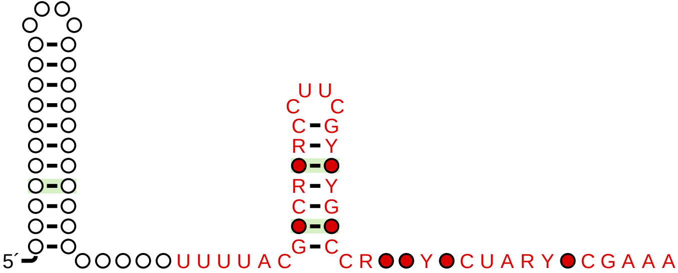

HMP2_5465_1

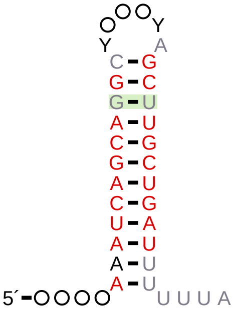

HMP2_5466_1

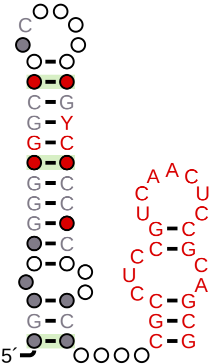

HMP2_5472_1

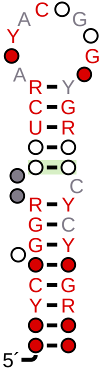

HMP2_5478_1

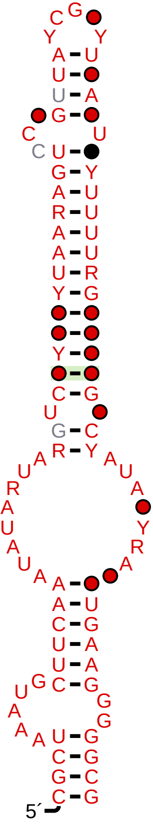

HMP2_5481_1

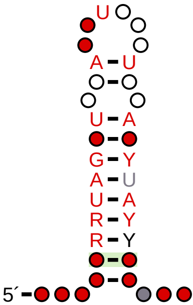

HMP2_5483_1

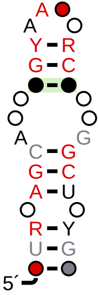

HMP2_5497_1

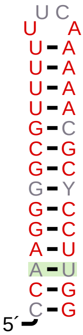

HMP2_5498_1

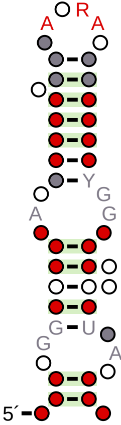

HMP2_5502_1

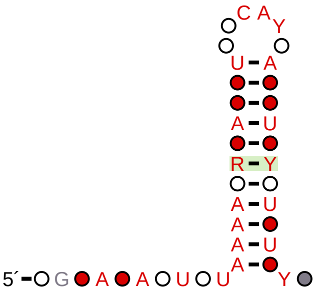

HMP2_5508_1

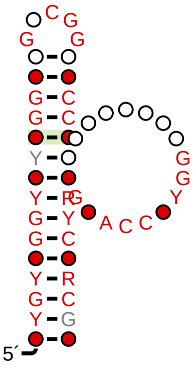

HMP2_5517_1

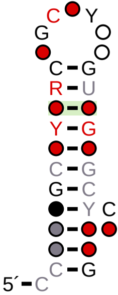

HMP2_5521_1

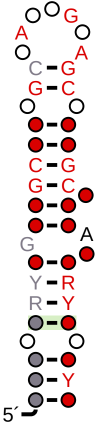

HMP2_5538_1

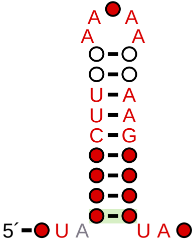

HMP2_5547_1

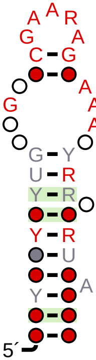

HMP2_555_1

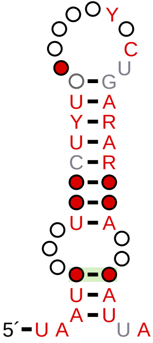

HMP2_5550_1

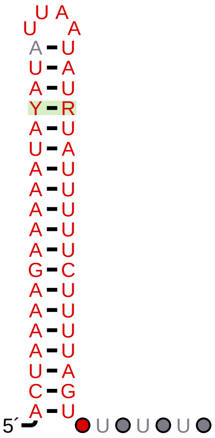

HMP2_5551_1

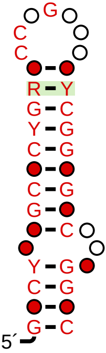

HMP2_5552_1

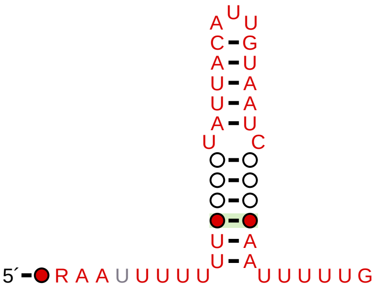

HMP2_5558_1

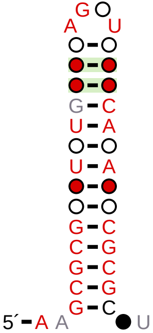

HMP2_556_1

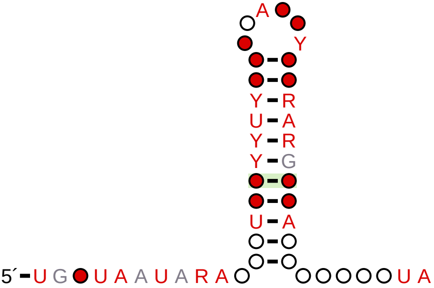

HMP2_5563_1

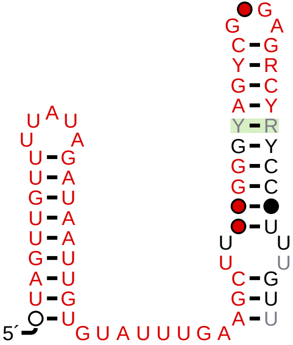

HMP2_5566_1

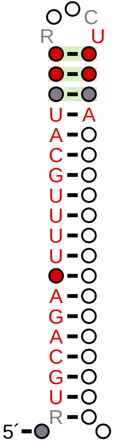

HMP2_5572_1

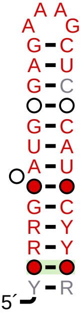

HMP2_5589_1

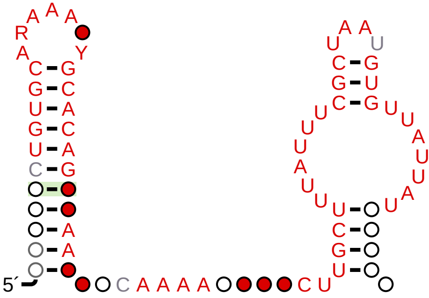

HMP2_560_1

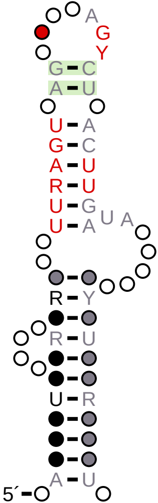

HMP2_5606_1

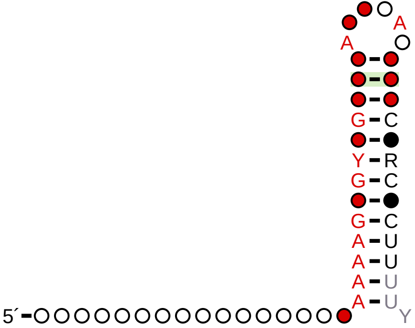

HMP2_5608_1

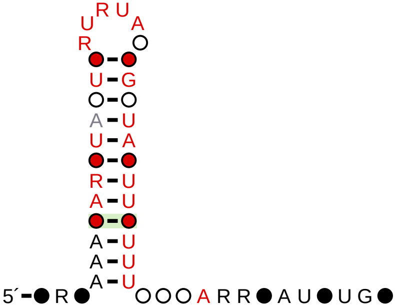

HMP2_5619_1

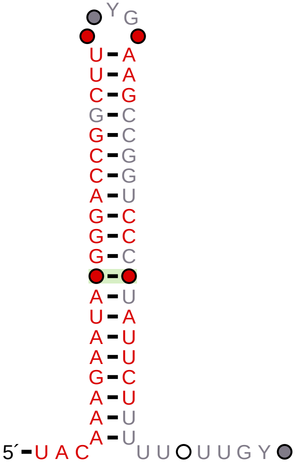

HMP2_562_1

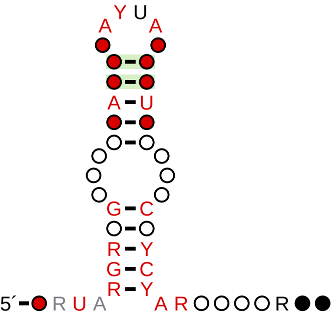

HMP2_5637_1

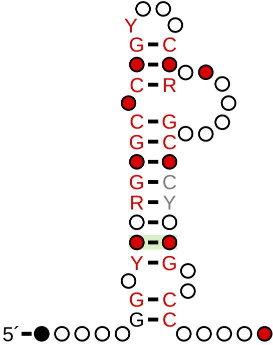

HMP2_5640_1

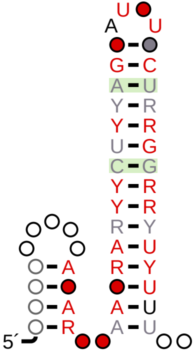

HMP2_5642_1

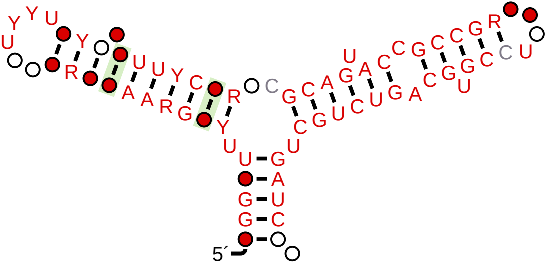

HMP2_5645_1

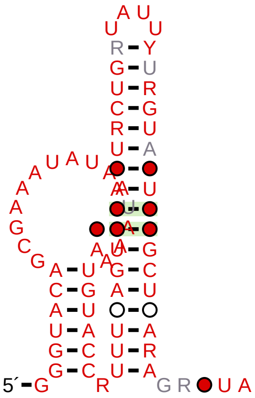

HMP2_5647_1

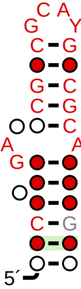

HMP2_5654_1

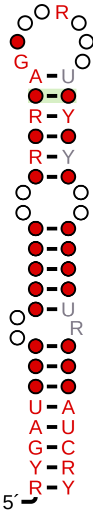

HMP2_5658_1

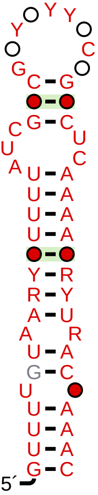

HMP2_5662_1

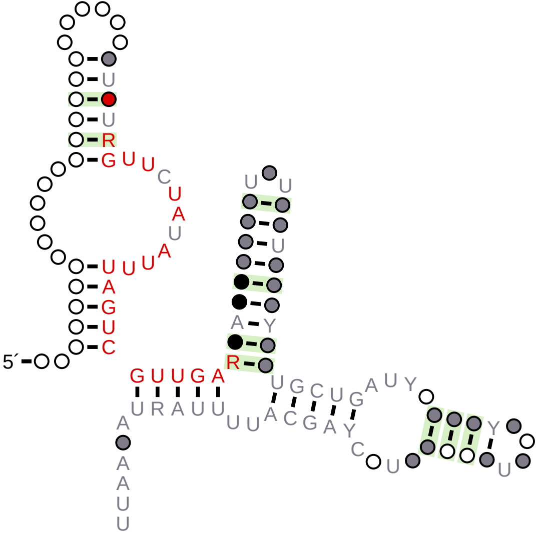

HMP2_5665_1

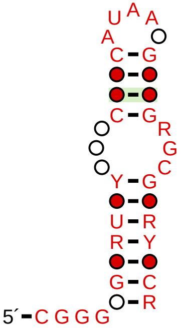

HMP2_5685_1

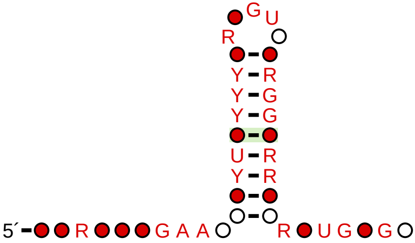

HMP2_569_1

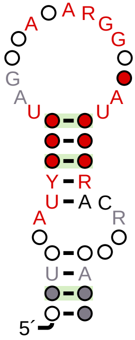

HMP2_5691_1

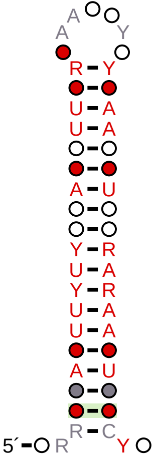

HMP2_5699_1

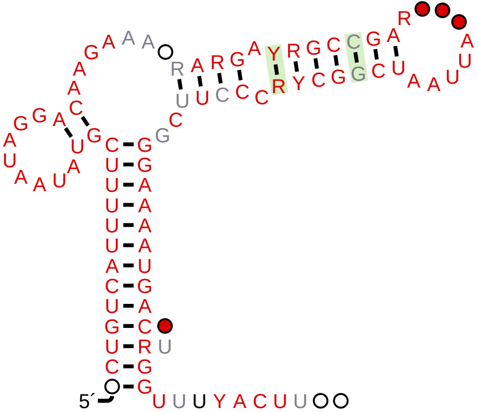

HMP2_5702_1

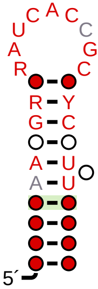

HMP2_5703_1

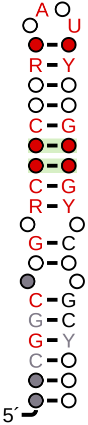

HMP2_5706_1

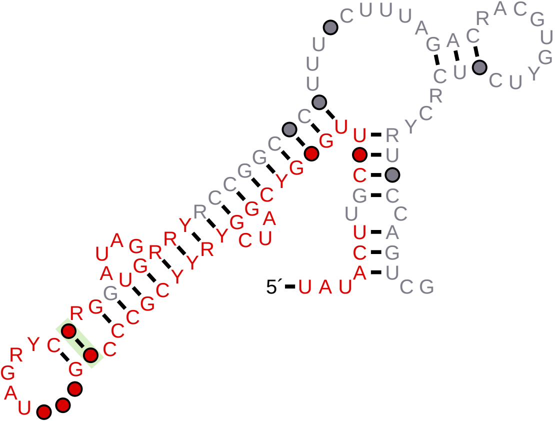

HMP2_5709_1

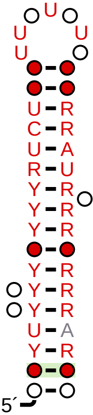

HMP2_5711_1

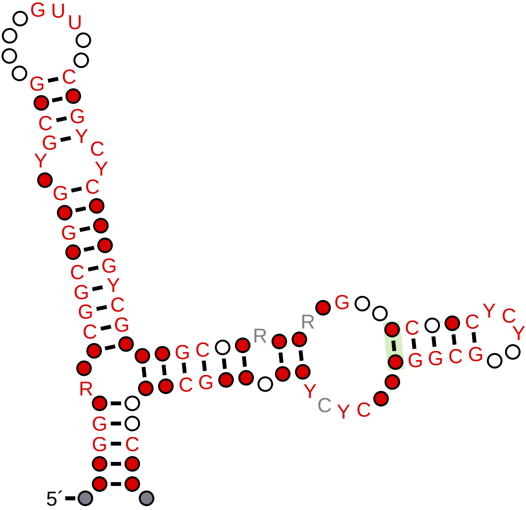

HMP2_5712_1

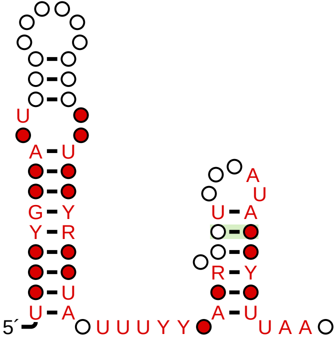

HMP2_5736_1

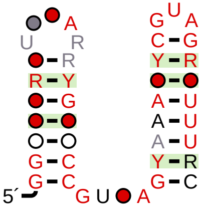

HMP2_574_1

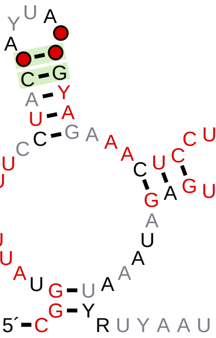

HMP2_5742_1

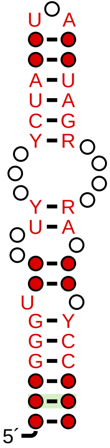

HMP2_5750_1

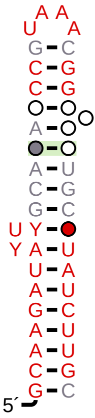

HMP2_5759_1

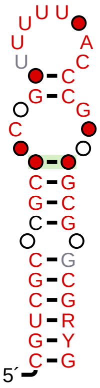

HMP2_5763_1

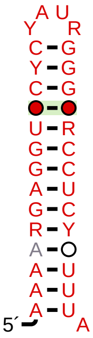

HMP2_5766_1

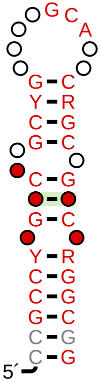

HMP2_577_1

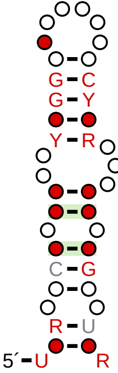

HMP2_5773_1

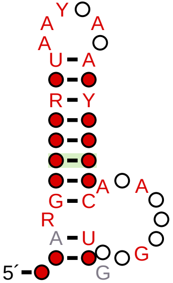

HMP2_5776_1

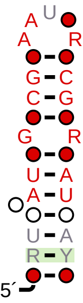

HMP2_5785_1

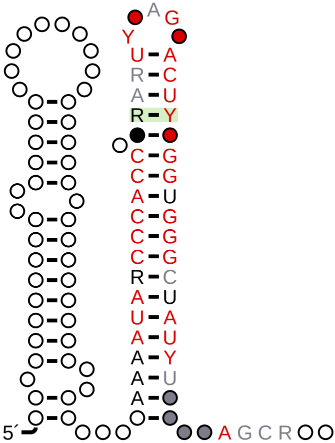

HMP2_5790_1

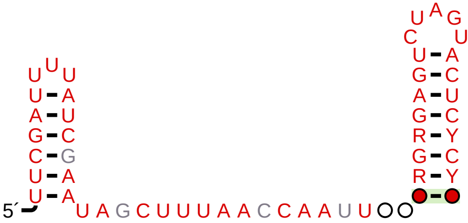

5´
U U U
U A
A U
G C
C G
U A
U A
U A G C U U U A A C C A A U U
U A G
C U
U A
G C
A U
G C
R Y
G C
R Y

HMP2_5794_1

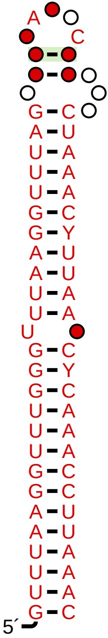

A
C
G C
A U
U A
U A
U A
G C
G Y
A U
A U
U A
U A
U
G C
G Y
G C
U A
U A
U A
G C
G C
A U
A U
U A
U A
U A
G C
5´

HMP2_5801_1

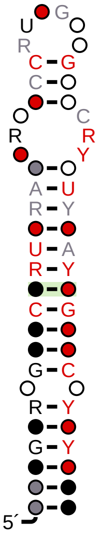

HMP2_5807_1

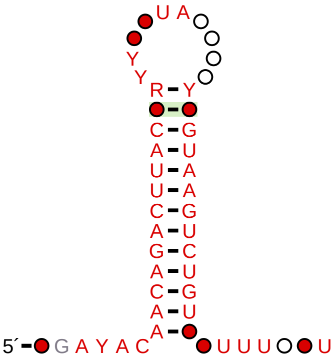

HMP2_5817_1

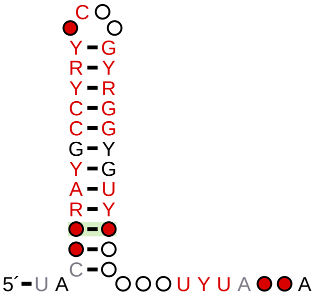

HMP2_5818_1

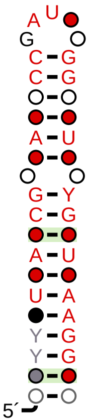

HMP2_5822_1

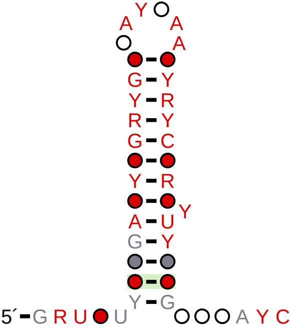

HMP2_5823_1

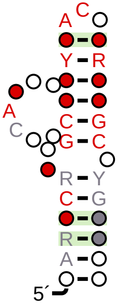

HMP2_5826_1

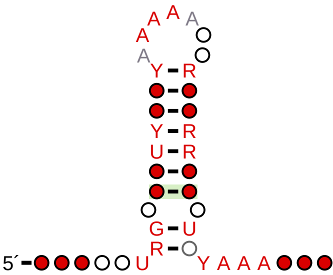

HMP2_583_1

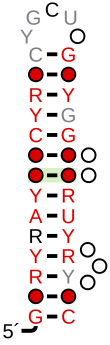

HMP2_5831_1

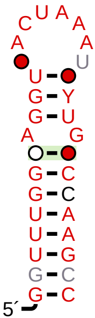

HMP2_5840_1

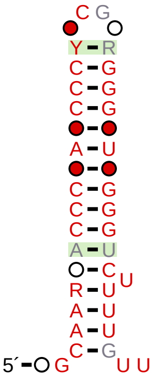

HMP2_585_1

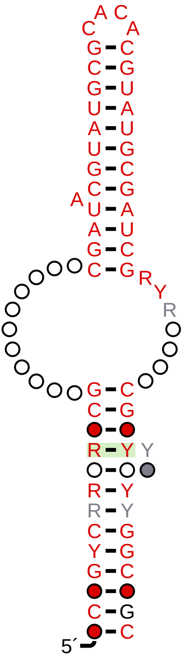

HMP2_5863_1

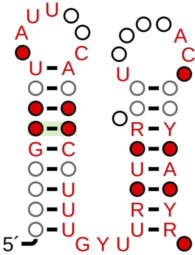

HMP2_5864_1

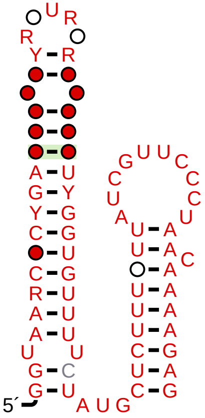

HMP2_587_1

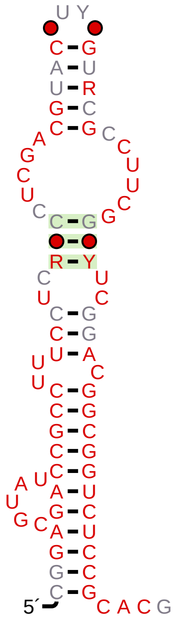

HMP2_5870_1

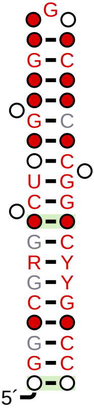

HMP2_5875_1

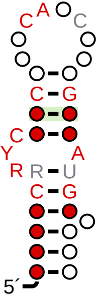

HMP2_5881_1

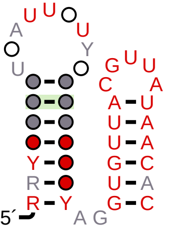

HMP2_5883_1

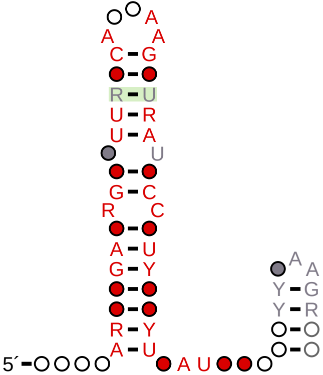

HMP2_590_1

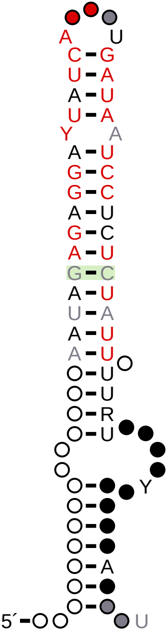

HMP2_5903_1

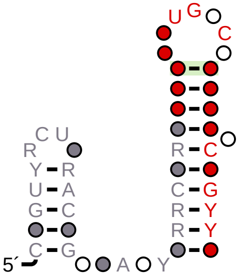

HMP2_5910_1

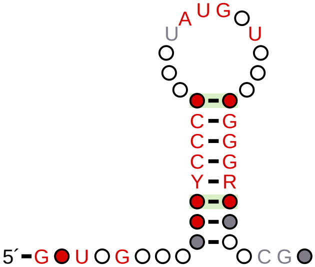

HMP2_5911_1

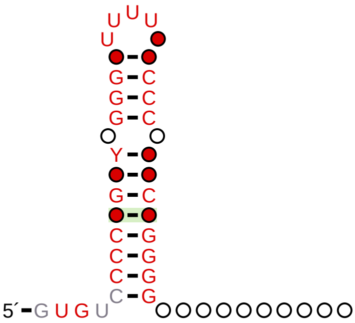

HMP2_5914_1

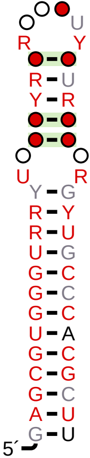

HMP2_5920_1

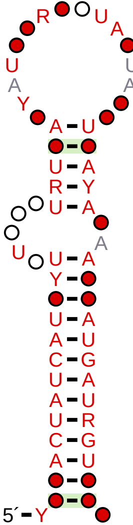

HMP2_5926_1

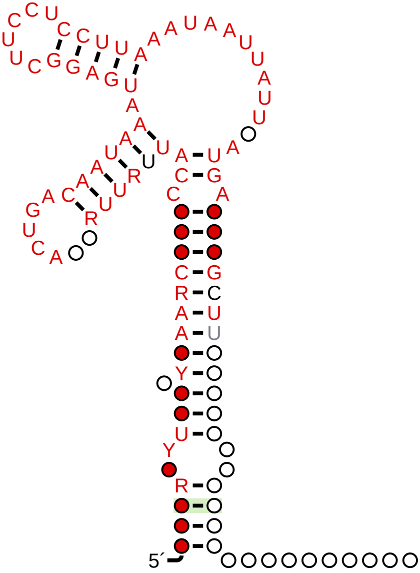

HMP2_5933_1

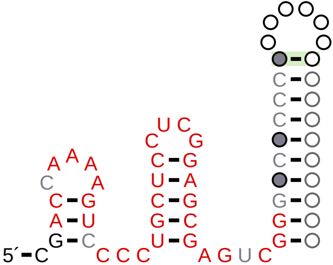

HMP2_5935_1

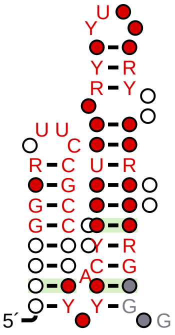

HMP2_5937_1

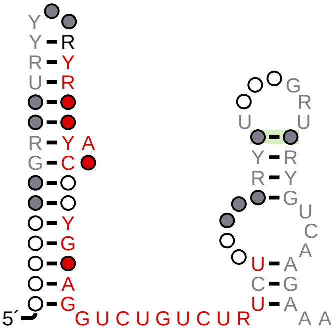

HMP2_5938_1

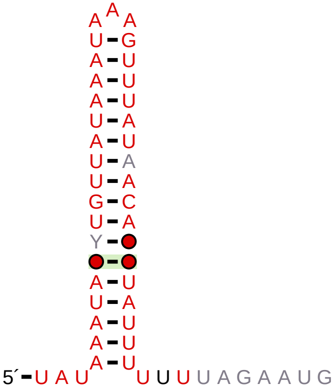

HMP2_5941_1

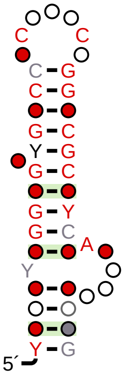

HMP2_5948_1

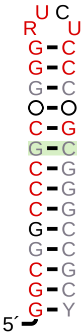

HMP2_5952_1

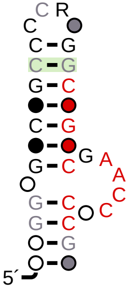

HMP2_5954_1

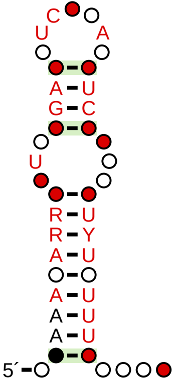

HMP2_5956_1

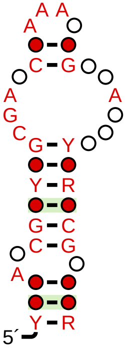

HMP2_596_1

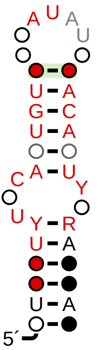

HMP2_5961_1

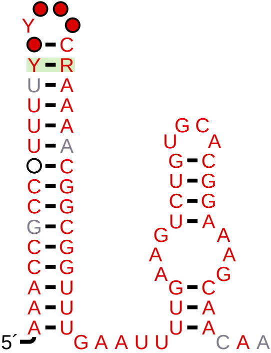

HMP2_5971_1

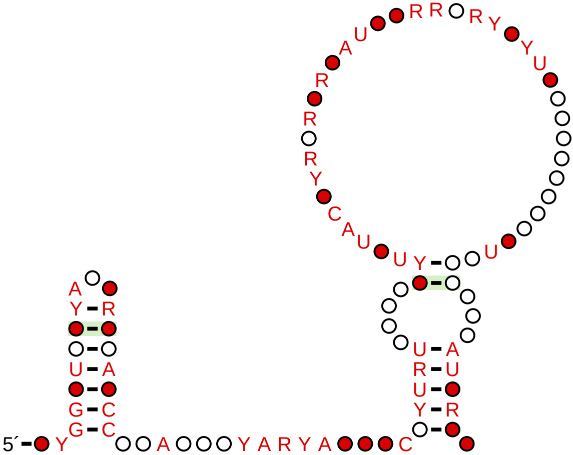

HMP2_5977_1

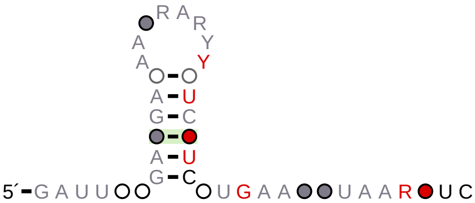

HMP2_598_1

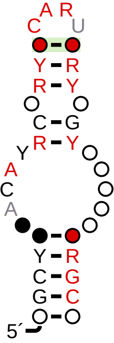

HMP2_5983_1

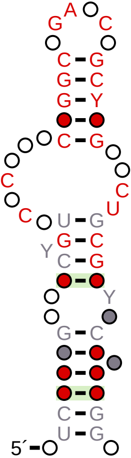

HMP2_5992_1

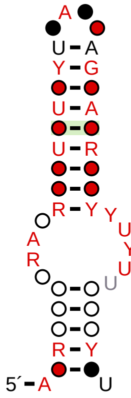

HMP2_5993_1

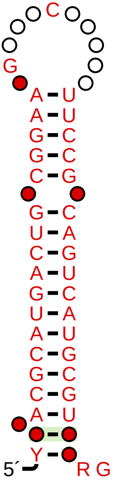

HMP2_5997_1

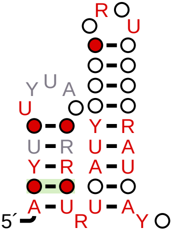

HMP2_5999_1

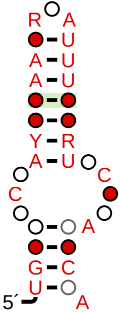

HMP2_6001_1

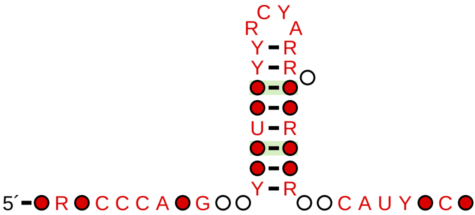

HMP2_6003_1

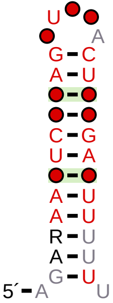

HMP2_6005_1

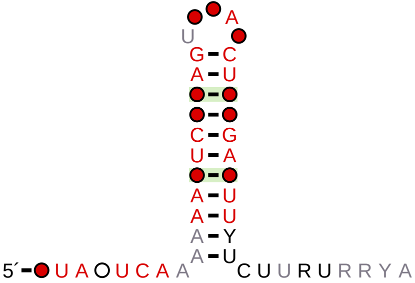

HMP2_6009_1

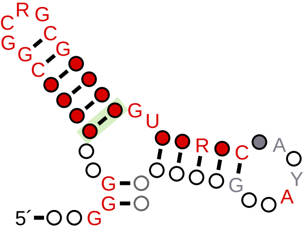

HMP2_6014_1

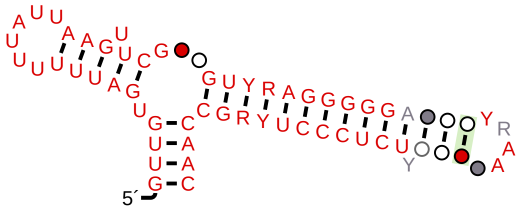

HMP2_6018_1

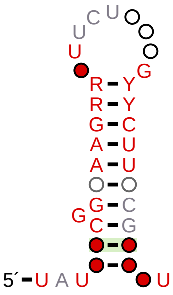

HMP2_6021_1

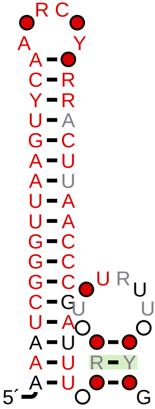

HMP2_6022_1

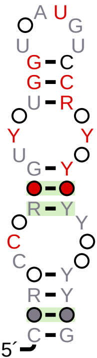

HMP2_6031_1

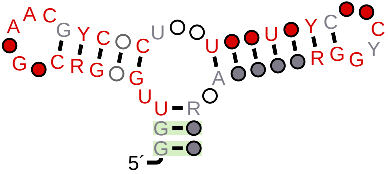

HMP2_6035_1

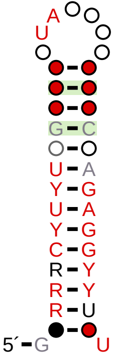

HMP2_6037_1

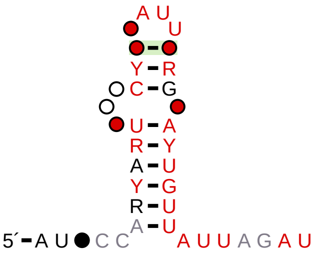

HMP2_6045_1

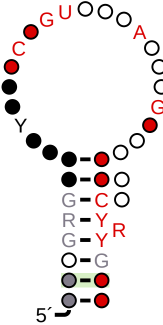

HMP2_6046_1

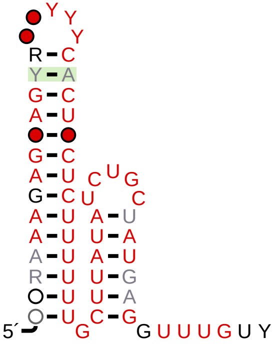

HMP2_6048_1

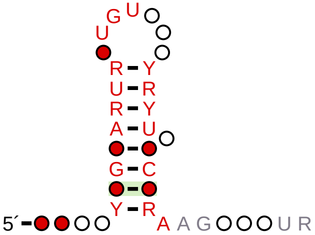

HMP2_6055_1

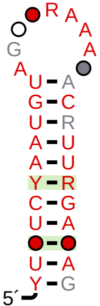

HMP2_6066_1

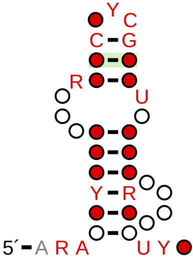

HMP2_6080_1

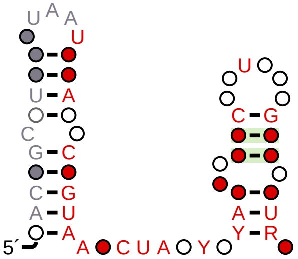

HMP2_6082_1

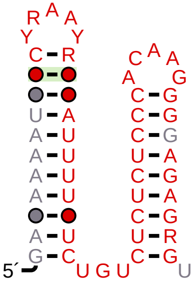

HMP2_6085_1

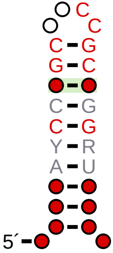

HMP2_6086_1

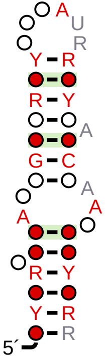

HMP2_6091_1

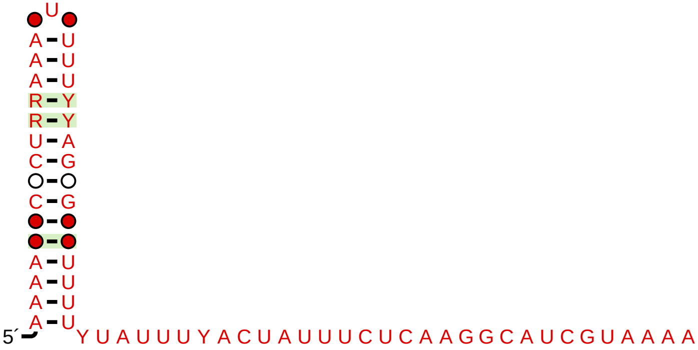
U
A U
A U
A U
R Y
R Y
U A
C G
C G
A U
A U
A U
A U
5´
YUAUUUYACUAUUUCUCAAGGCAUCGUAAAA

HMP2_6097_1

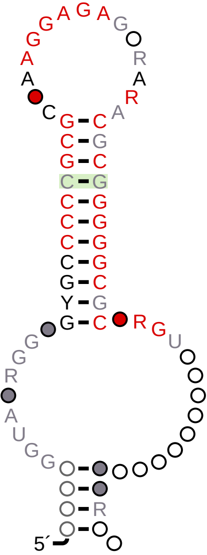

HMP2_6104_1

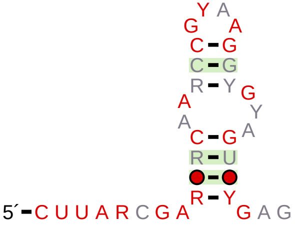

HMP2_6107_1

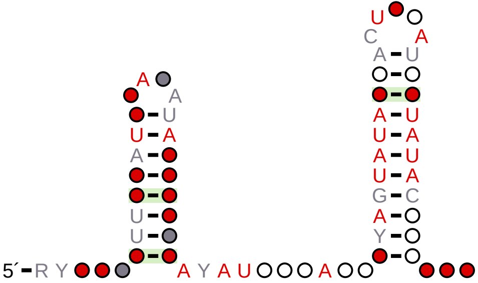

HMP2_6108_1

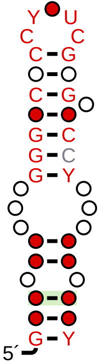

HMP2_6109_1

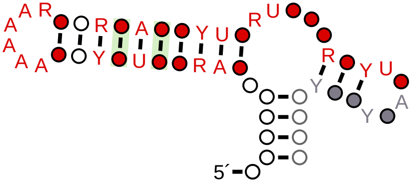

HMP2_6114_1

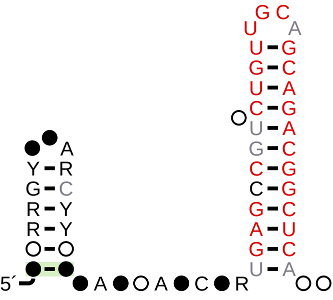

HMP2_6123_1

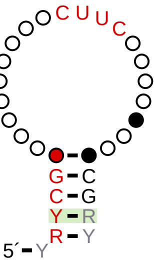

HMP2_6125_1

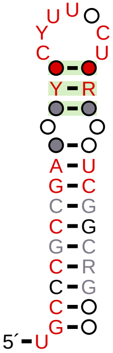

HMP2_6134_1

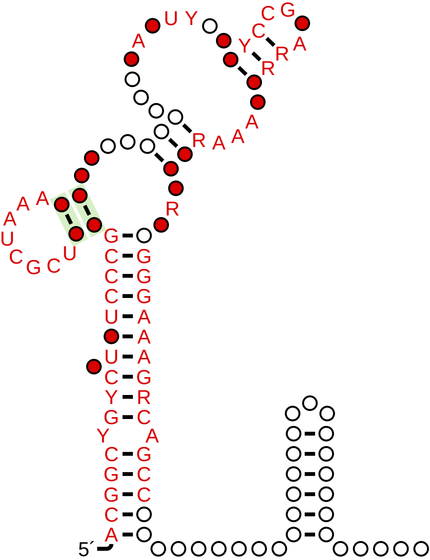

HMP2_6143_1

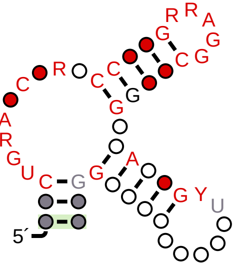

HMP2_6158_1

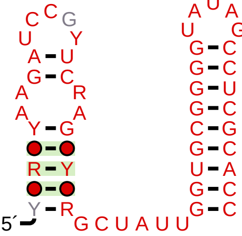

HMP2_6159_1

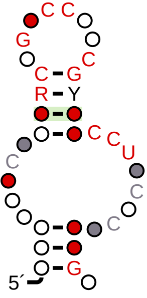

HMP2_6166_1

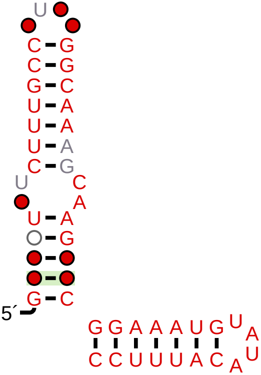

HMP2_6169_1

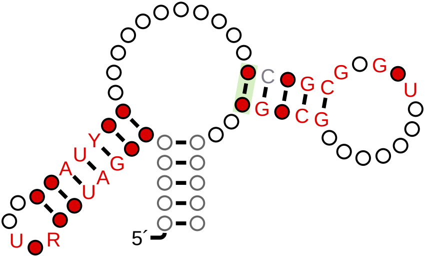

HMP2_6170_1

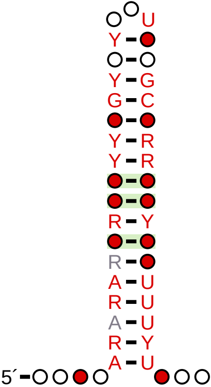

HMP2_6178_1

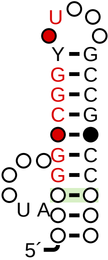

HMP2_6179_1

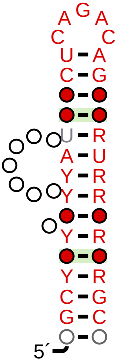

HMP2_618_1

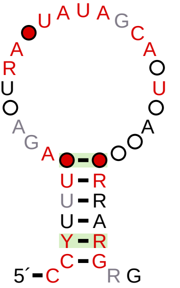

HMP2_6184_1

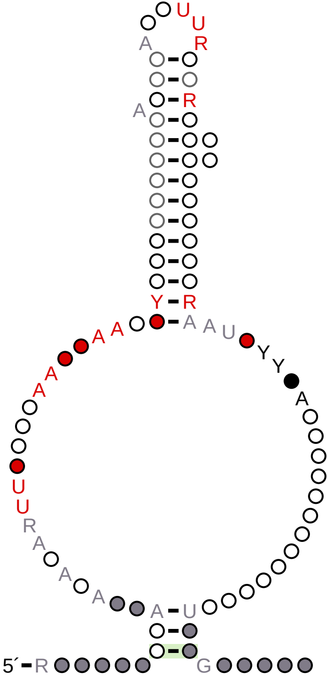

HMP2_6185_1

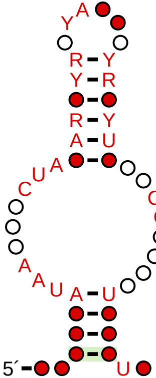

HMP2_6187_1

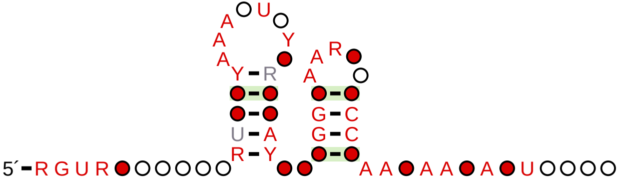

HMP2_6198_1

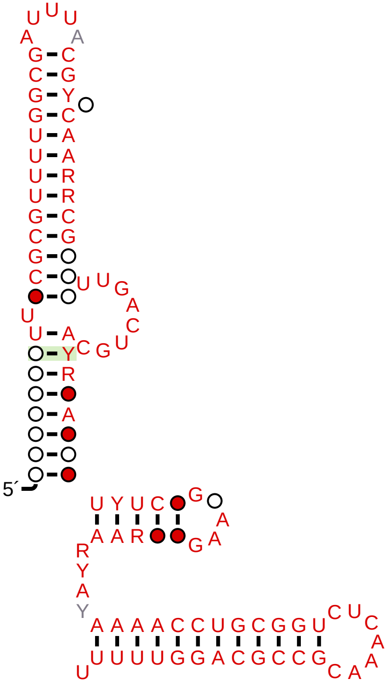

HMP2_62_1

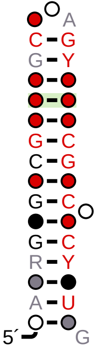

HMP2_6204_1

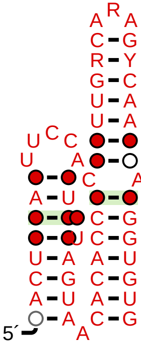

HMP2_6205_1

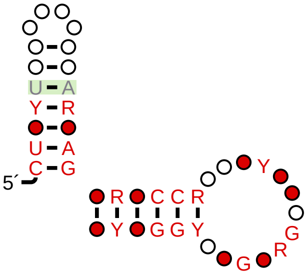

HMP2_6208_1

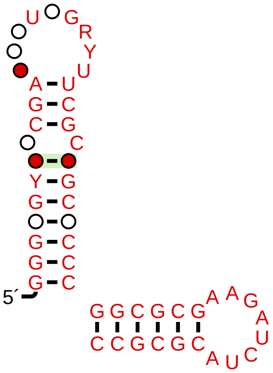

HMP2_6219_1

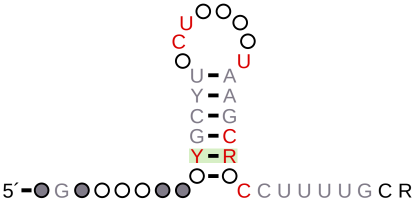

HMP2_6221_1

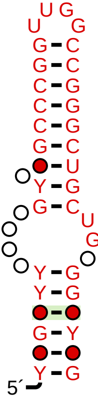

HMP2_6226_1

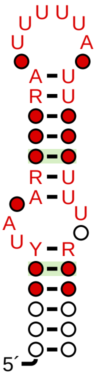

HMP2_6231_1

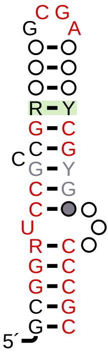

HMP2_624_1

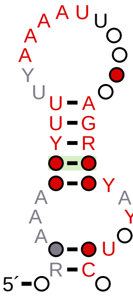

HMP2_6240_1

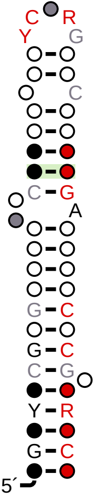

HMP2_6242_1

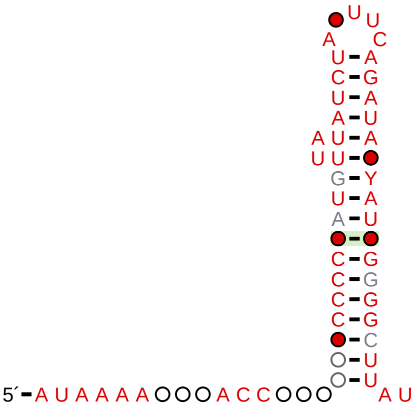

HMP2_6243_1

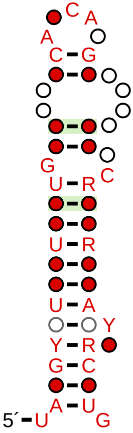

HMP2_6253_1

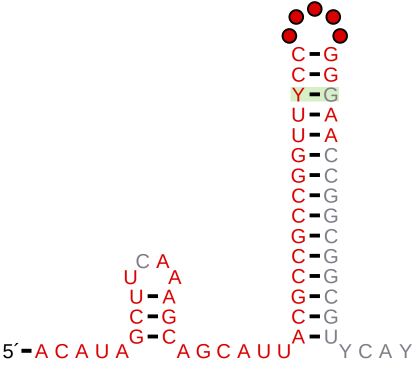

C G
C G
Y G
U A
U A
G C
G C
C G
C G
G C
C G
C G
G C
C G
A U
C A
U A
U A
C G
G C
5´ A C A U A
A G C A U U
Y C A Y

HMP2_6258_1

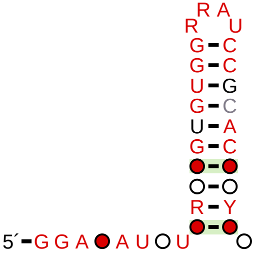

HMP2_6265_1

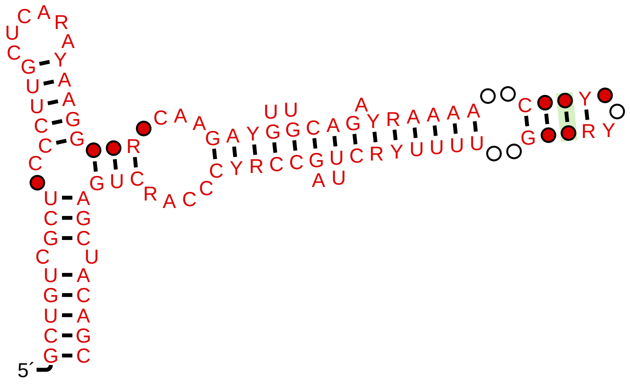

HMP2_6268_1

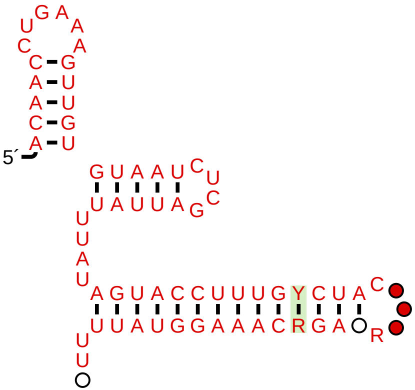

HMP2_6273_1

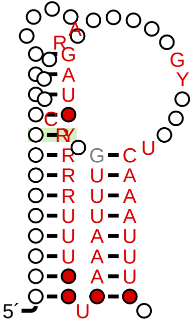

HMP2_6288_1

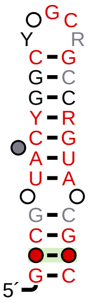

HMP2_6290_1

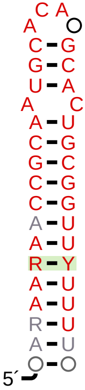

HMP2_6295_1

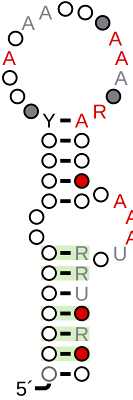

HMP2_6299_1

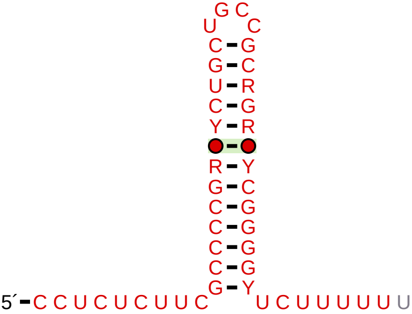

HMP2_6301_1

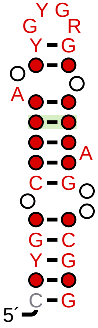

HMP2_6309_1

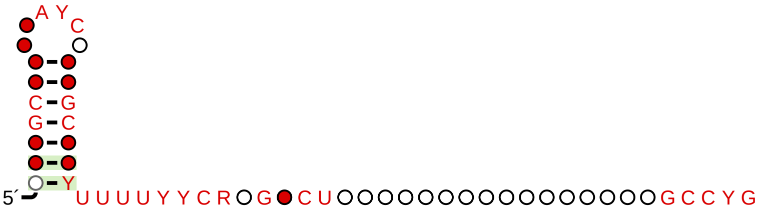

HMP2_6311_1

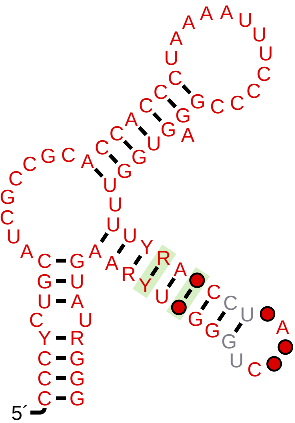

HMP2_6313_1

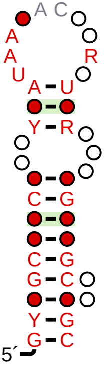

HMP2_6315_1

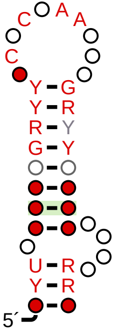

HMP2_6317_1

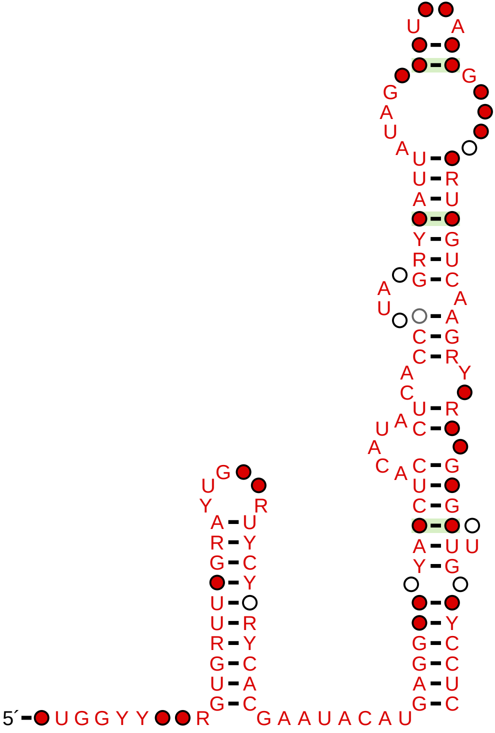

HMP2_6320_1

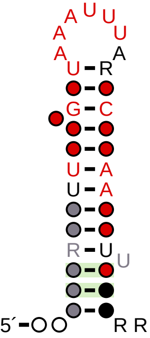

HMP2_6323_1

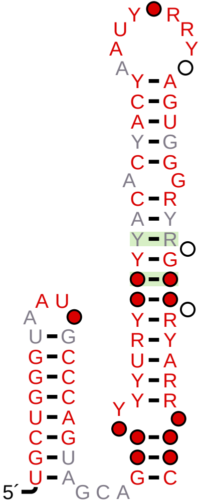

HMP2_6326_1

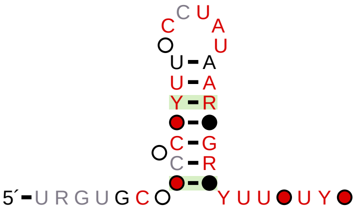

HMP2_6327_1

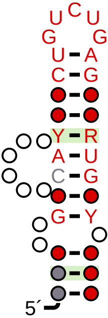

HMP2_6328_1

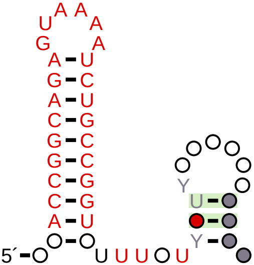

HMP2_6330_1

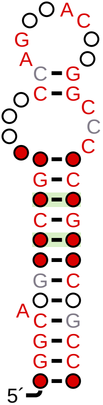

HMP2_6365_1

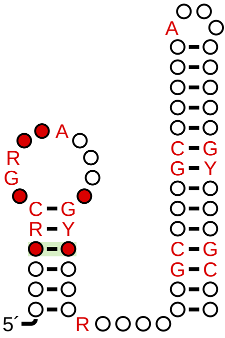

HMP2_6367_1

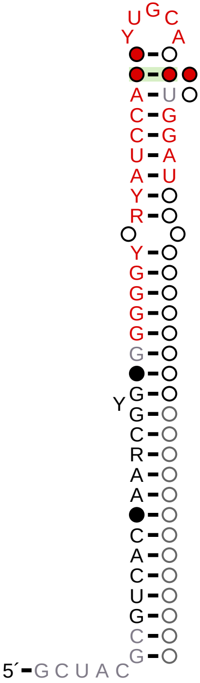

HMP2_637_1

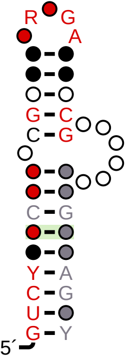

HMP2_6376_1

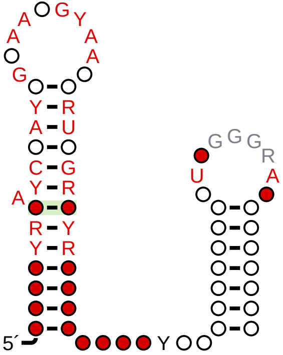

HMP2_6384_1

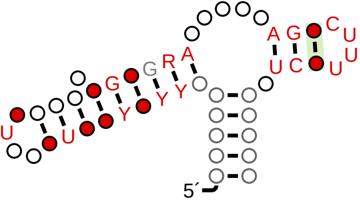

HMP2_6386_1

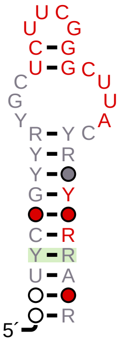

HMP2_6387_1

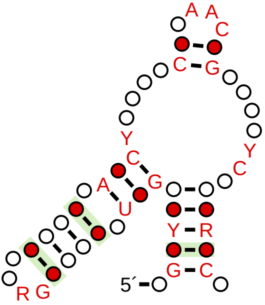

HMP2_6391_1

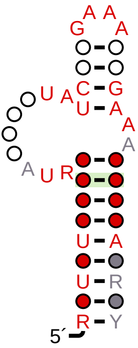

HMP2_6405_1

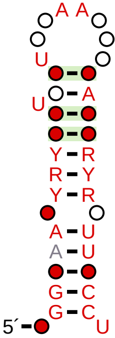

HMP2_6408_1

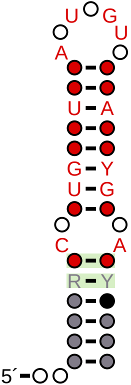

HMP2_6416_1

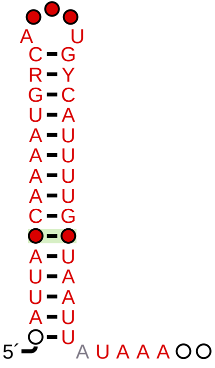

HMP2_6422_1

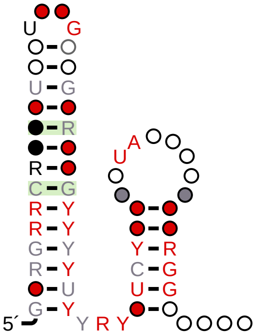

HMP2_6423_1

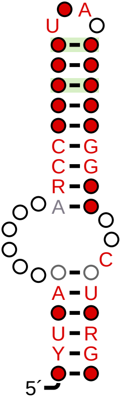

HMP2_6426_1

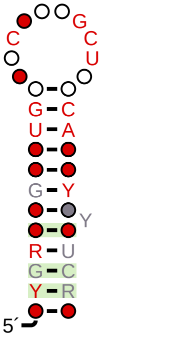

HMP2_6428_1

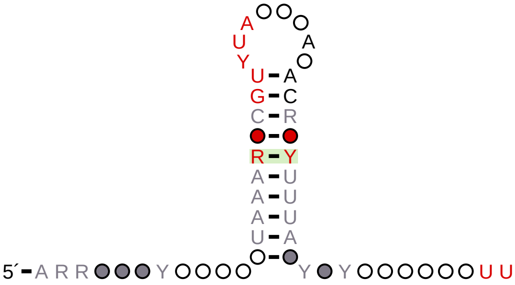

HMP2_6433_1

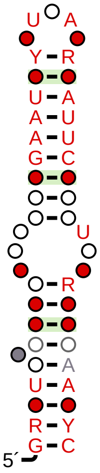

HMP2_644_1

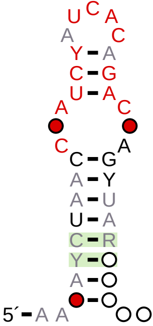

HMP2_6447_1

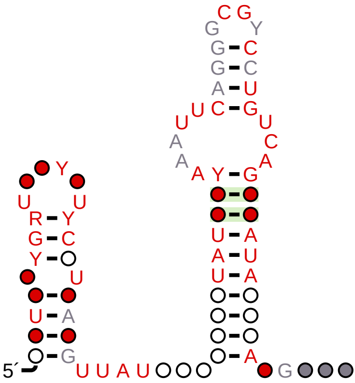

HMP2_6449_1

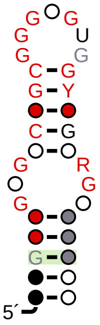

HMP2_6465_1

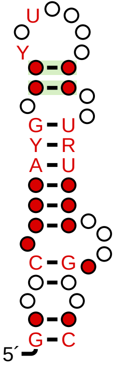

HMP2_6466_1

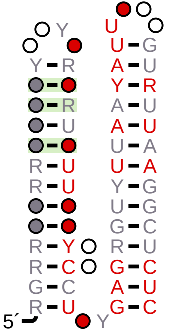

HMP2_6467_1

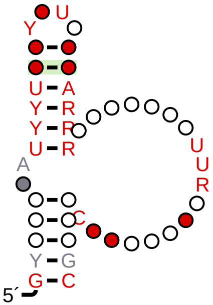

HMP2_6473_1

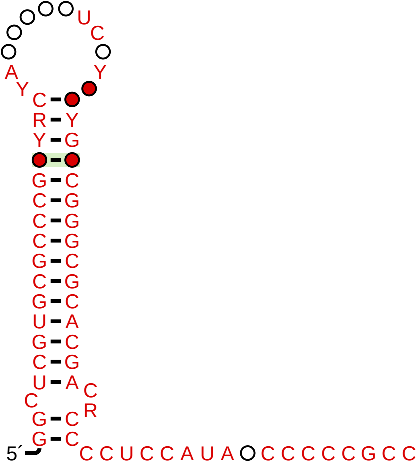

HMP2_648_1

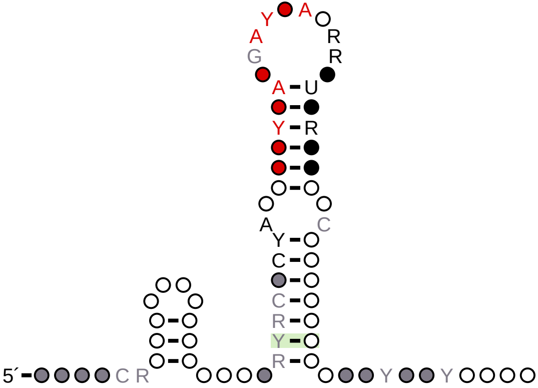

HMP2_6481_1

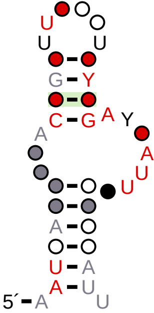

HMP2_6484_1

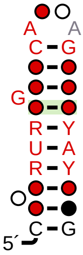

HMP2_6491_1

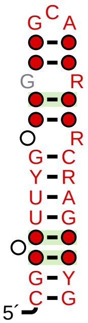

HMP2_6497_1

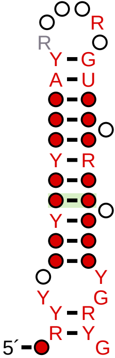

HMP2_6504_1

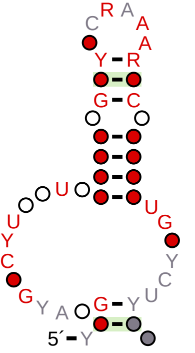

HMP2_6509_1

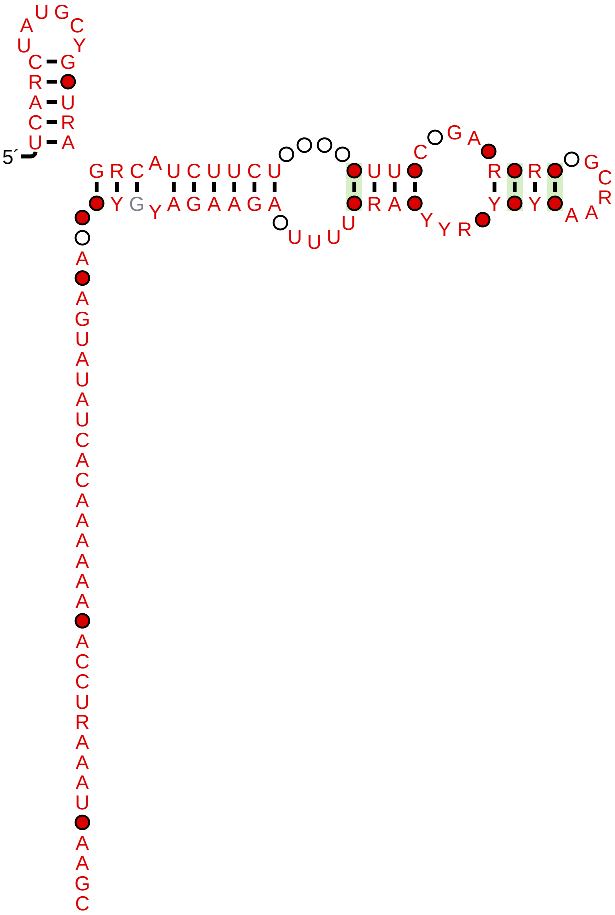

HMP2_6518_1

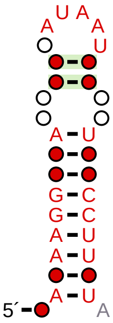

HMP2_6523_1

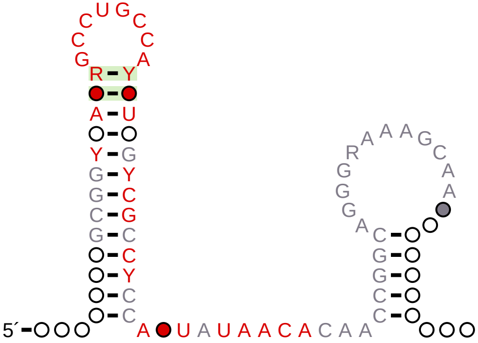

HMP2_6529_1

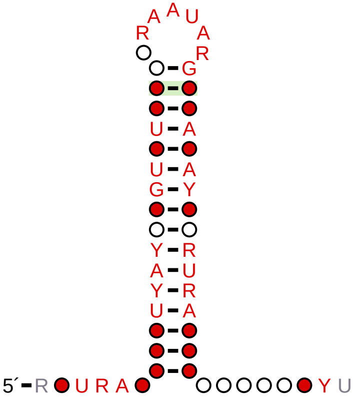

HMP2_6533_1

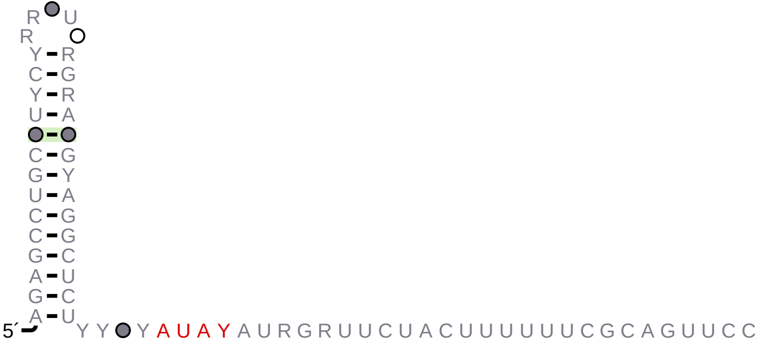

5´
R U
R
Y R
C G
Y R
U A
C G
G Y
U A
C G
C G
G C
A U
G C
A U
Y Y Y A U A Y A U R G R U U C U A C U U U U U U C G C A G U U C C

HMP2_6540_1

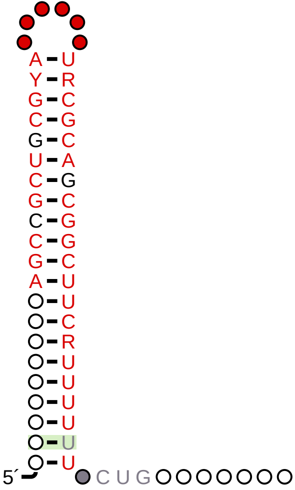

HMP2_6541_1

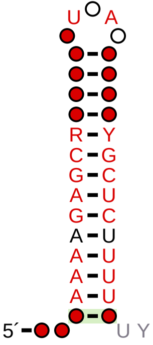

HMP2_6547_1

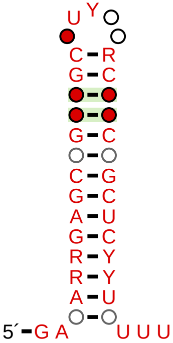

HMP2_6562_1

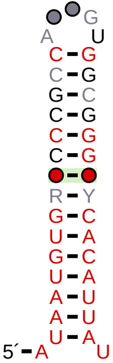

HMP2_6564_1

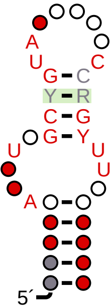

HMP2_6565_1

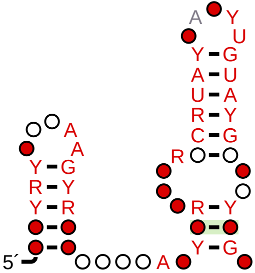

HMP2_6566_1

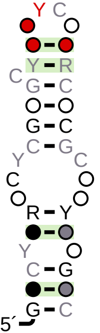

HMP2_6568_1

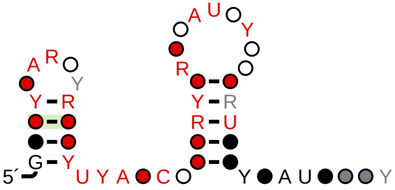

HMP2_6572_1

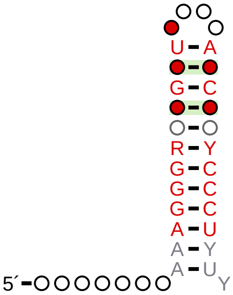

HMP2_6573_1

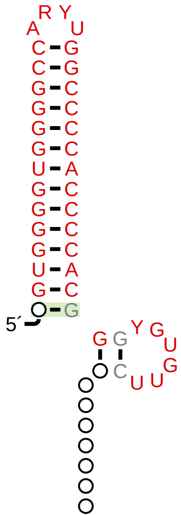

HMP2_6577_1

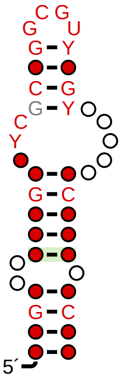

HMP2_6579_1

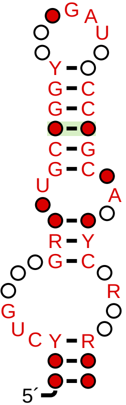

HMP2_6581_1

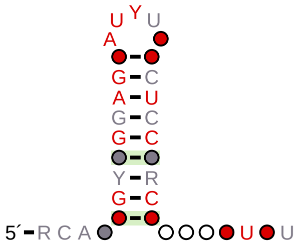

HMP2_6590_1

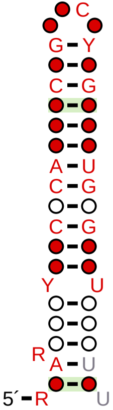

HMP2_6596_1

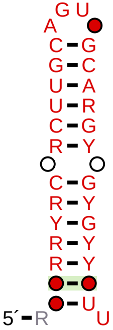

HMP2_66_1

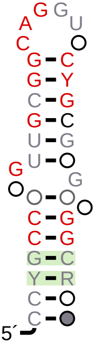

HMP2_6600_1

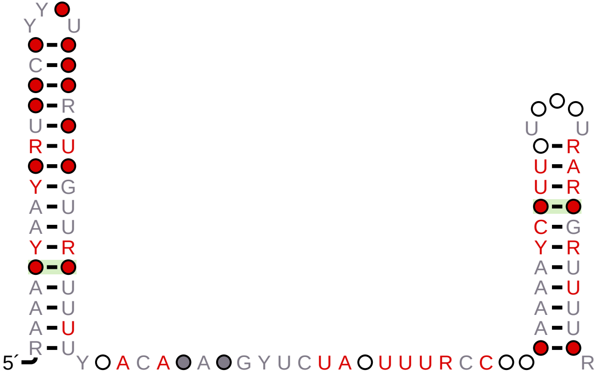

HMP2_6602_1

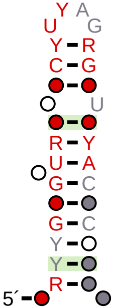

HMP2_6612_1

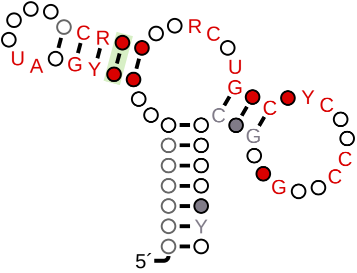

HMP2_6617_1

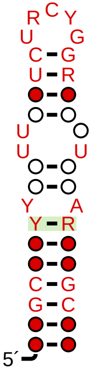

HMP2_6624_1

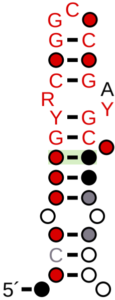

HMP2_6632_1

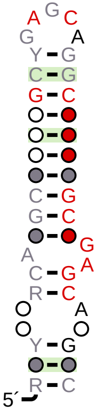

HMP2_6636_1

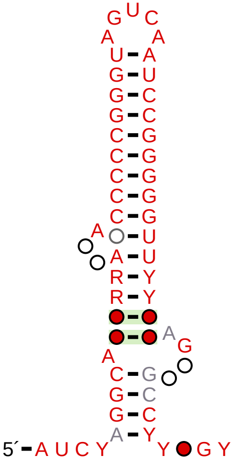

HMP2_6637_1

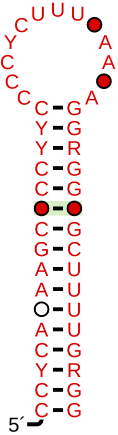

HMP2_6639_1

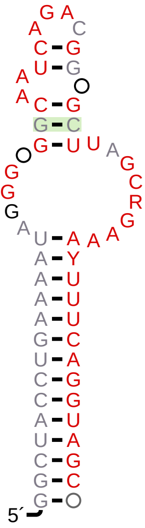

HMP2_6643_1

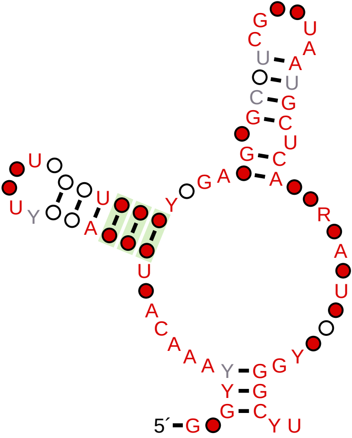

HMP2_6659_1

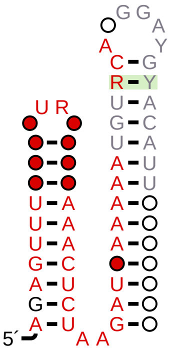

HMP2_6663_1

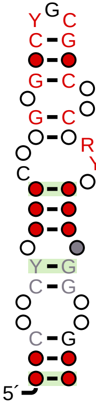

HMP2_6669_1

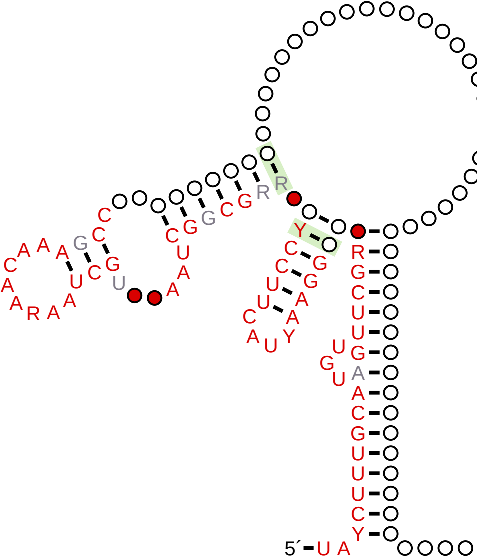

HMP2_6673_1

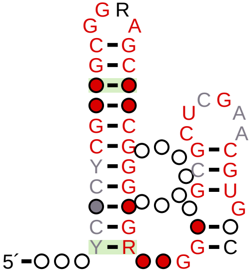

HMP2_6682_1

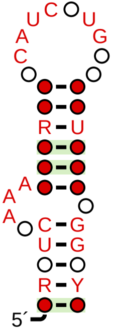

HMP2_6688_1

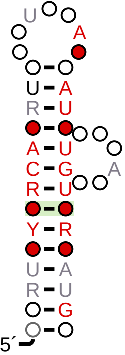

HMP2_669_1

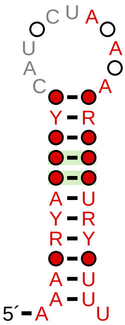

HMP2_6695_1

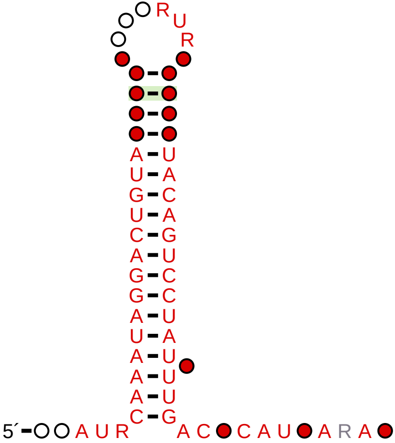

HMP2_6696_1

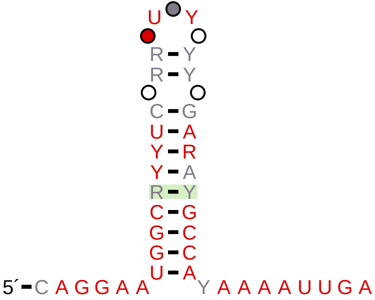

HMP2_6703_1

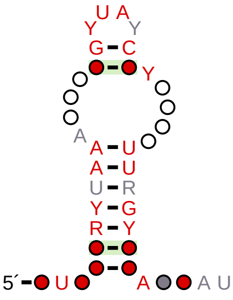

HMP2_6707_1

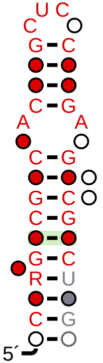

HMP2_6712_1

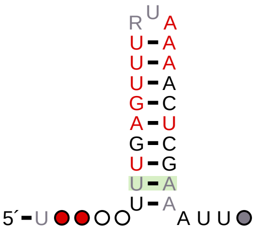

HMP2_6713_1

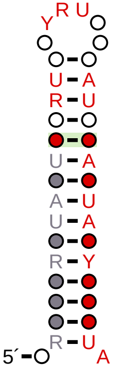

HMP2_6719_1

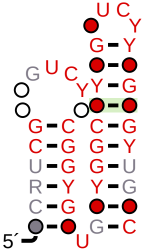

HMP2_6737_1

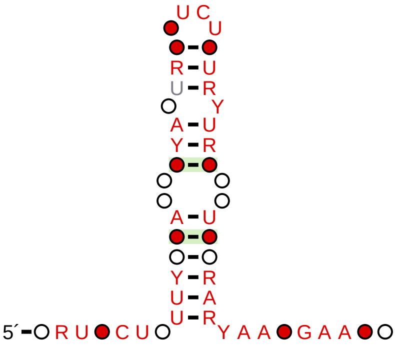

HMP2_6739_1

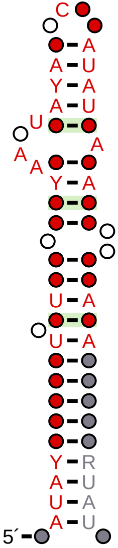

HMP2_6740_1

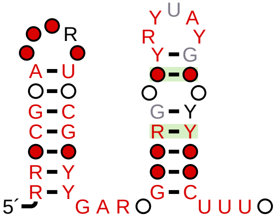

HMP2_6744_1

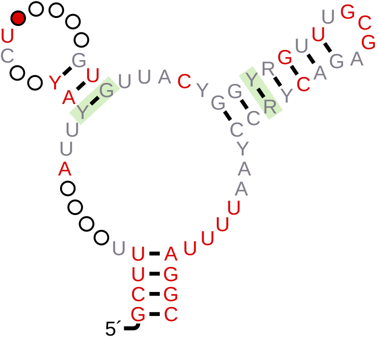

HMP2_6746_1

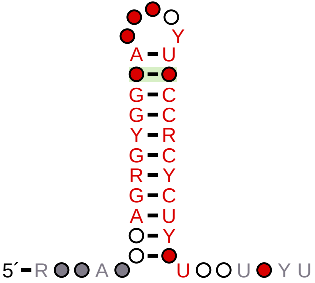

HMP2_6760_1

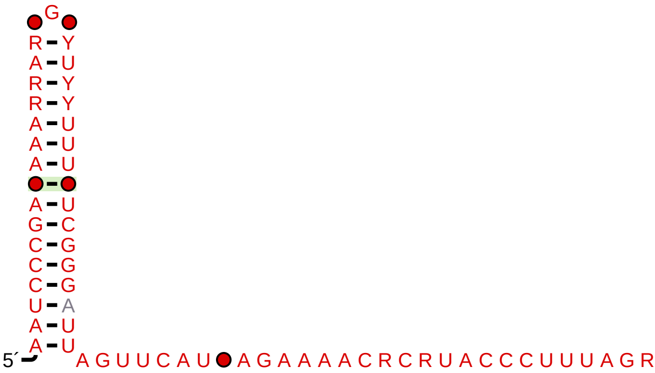

G
R Y
A U
R Y
R Y
A U
A U
A U
A U
G C
C G
C G
C G
U A
A U
A U
5´
A G U U C A U
A G A A A A C R C R U A C C C U U U A G R

HMP2_6763_1

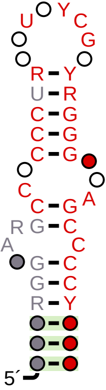

HMP2_6764_1

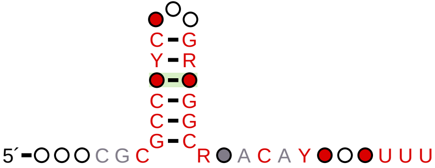

HMP2_6767_1

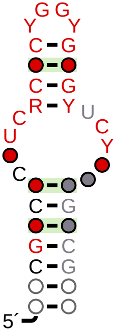

HMP2_6768_1

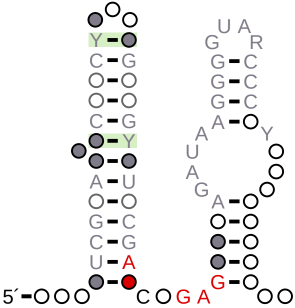

HMP2_6776_1

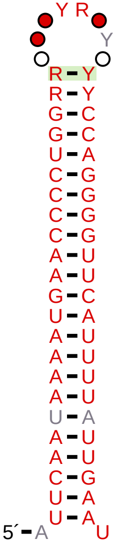

Y R
Y
R Y
R Y
G C
G C
U A
C G
C G
C G
C G
A U
A U
G C
U A
A U
A U
A U
A U
U A
A U
A U
C G
U A
U A
5´ A
U

HMP2_6780_1

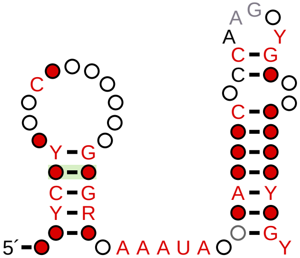

HMP2_6788_1

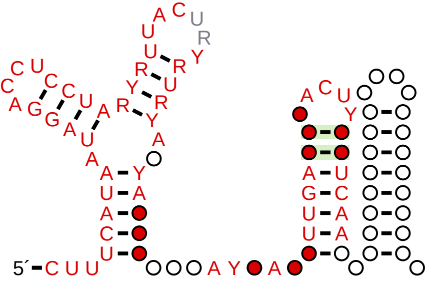

HMP2_679_1

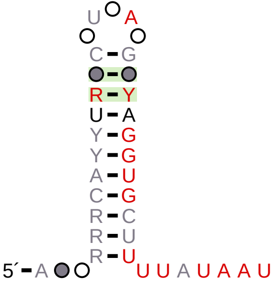

HMP2_6791_1

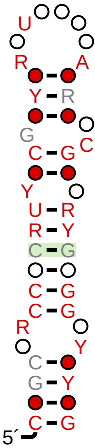

HMP2_6794_1

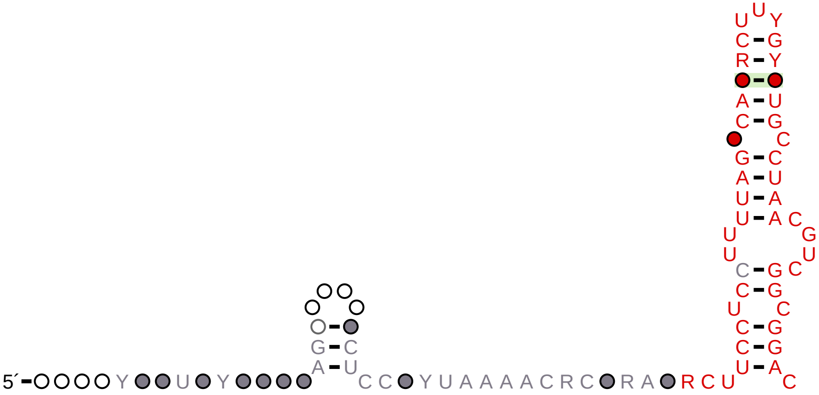

HMP2_68_1

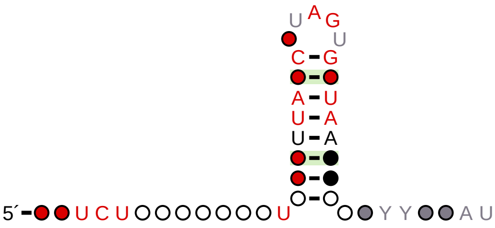

HMP2_6800_1

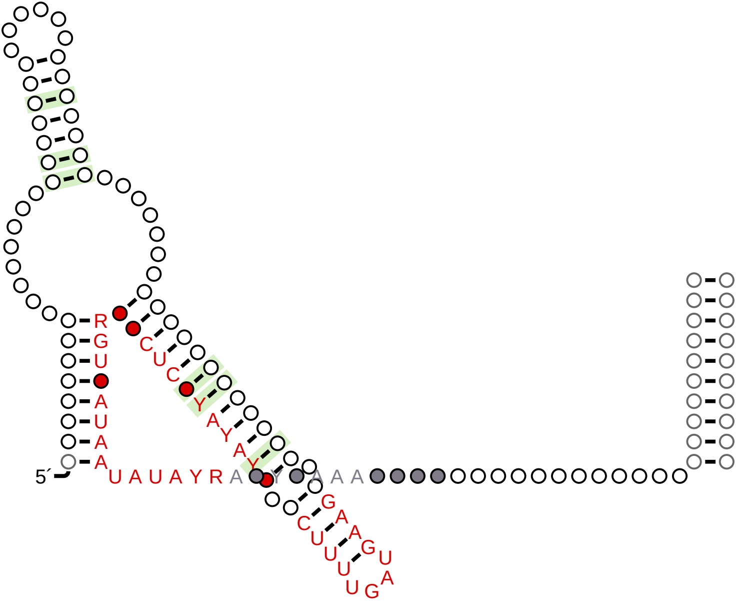

HMP2_6804_1

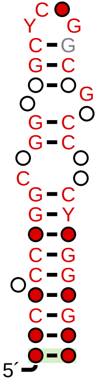

HMP2_6805_1

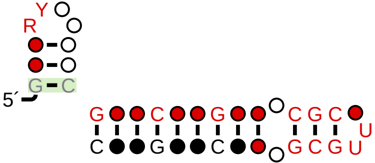

HMP2_681_1

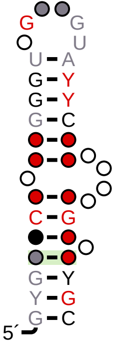

HMP2_6818_1

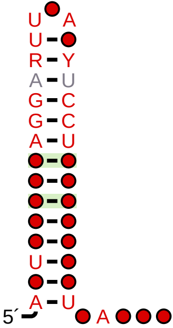

HMP2_6821_1

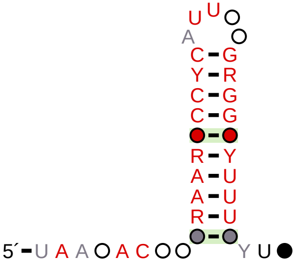

HMP2_6825_1

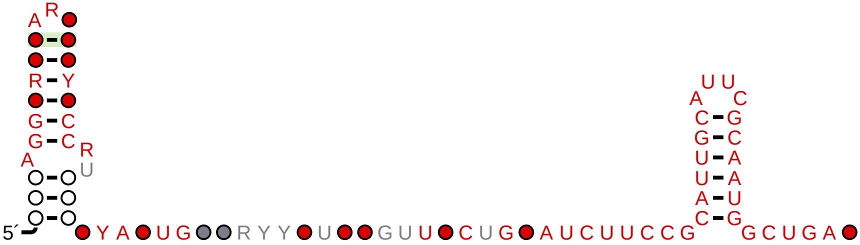

5´
Y A U G R Y Y U G U U C U G A U C U U C C G G C U G A
A R
R Y
G C
G C
A
R
U
U U
A C
C G
G C
U A
U A
A U
C G

HMP2_6826_1

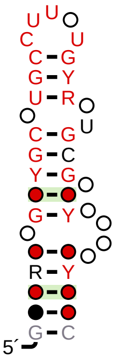

HMP2_683_1

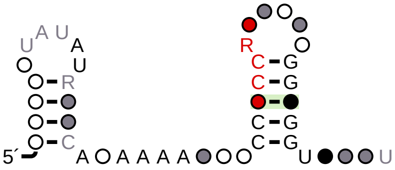

HMP2_6834_1

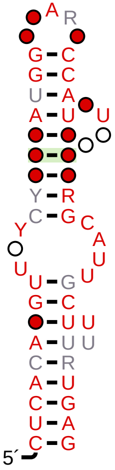

HMP2_6835_1

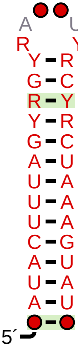

HMP2_6846_1

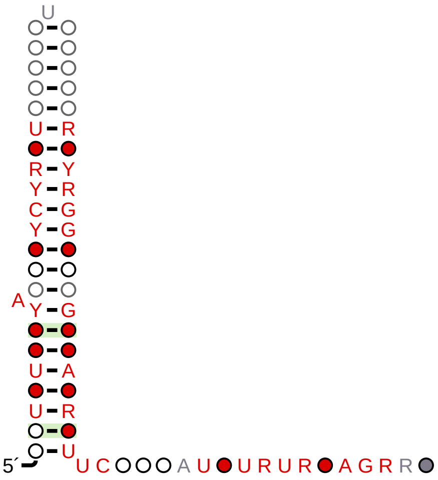

HMP2_6858_1

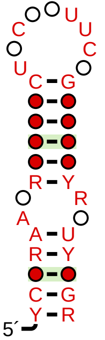

HMP2_686_1

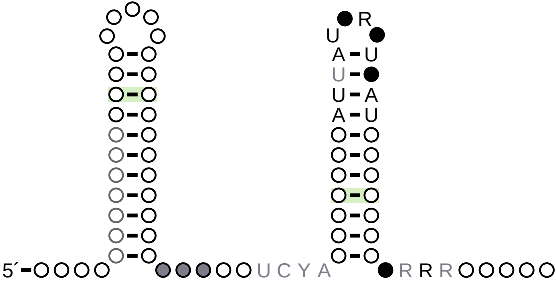

HMP2_6867_1

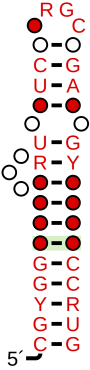

HMP2_6868_1

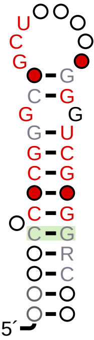

HMP2_687_1

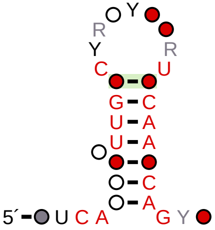

HMP2_6873_1

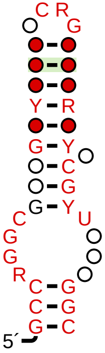

HMP2_6879_1

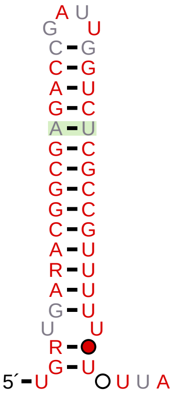

HMP2_6885_1

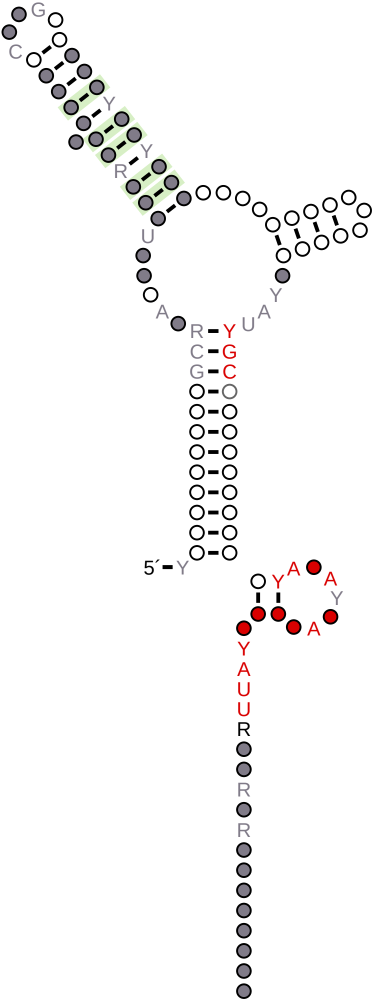

HMP2_6893_1

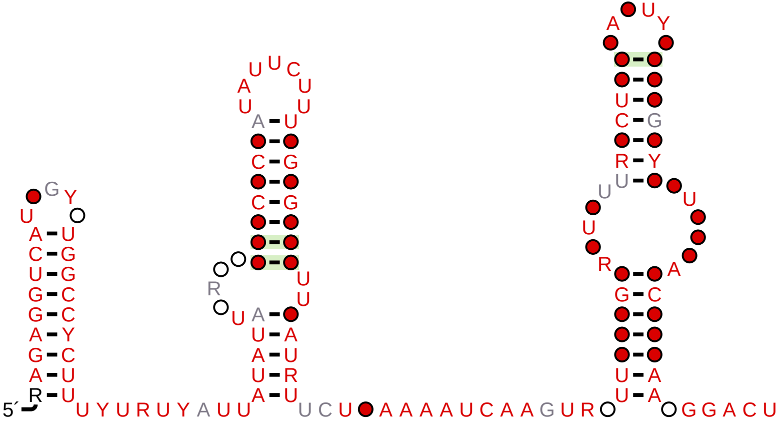

HMP2_690_1

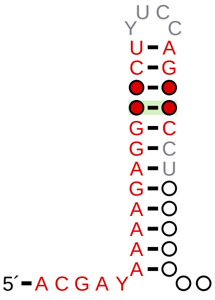

HMP2_6902_1

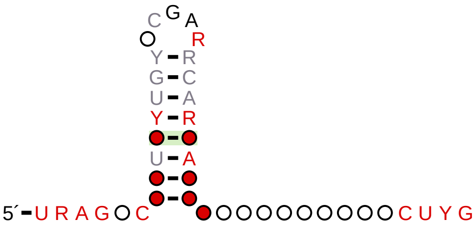

HMP2_6906_1

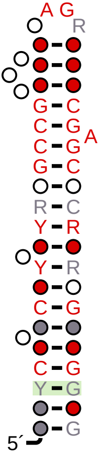

HMP2_6920_1

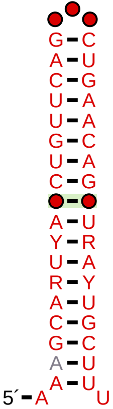

HMP2_6921_1

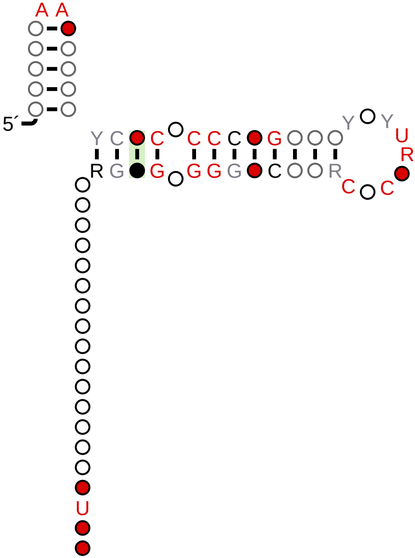

HMP2_6922_1

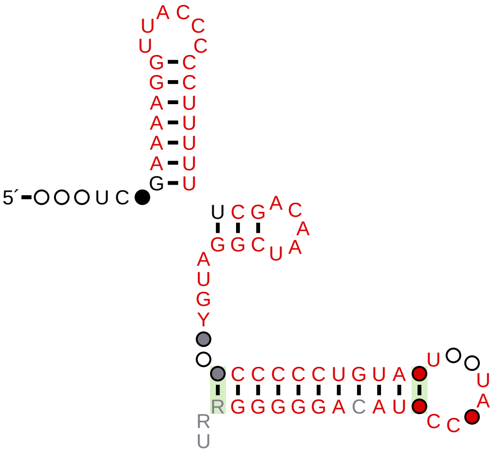

HMP2_6923_1

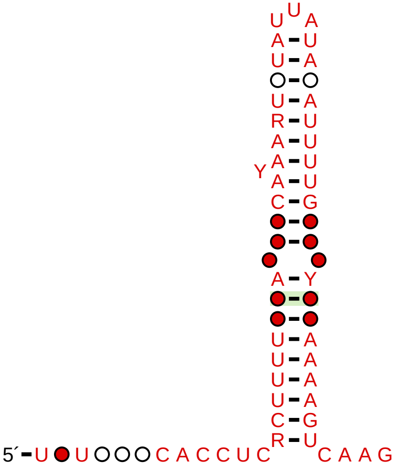

HMP2_6927_1

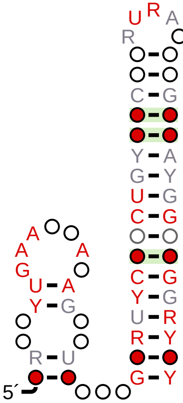

HMP2_6930_1

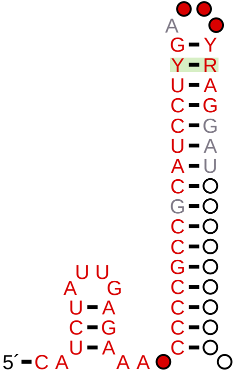

HMP2_6936_1

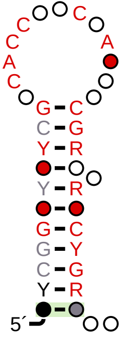

HMP2_694_1

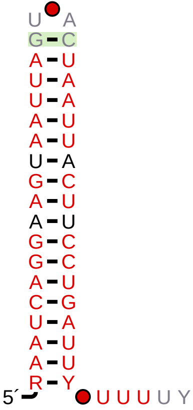

HMP2_6953_1

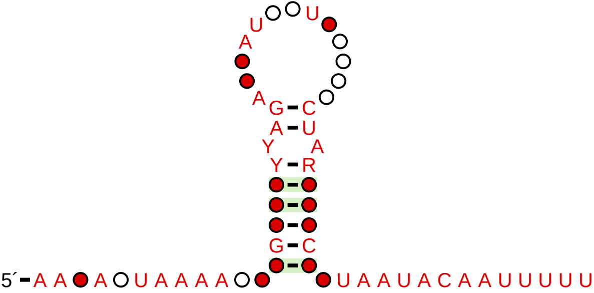

HMP2_6955_1

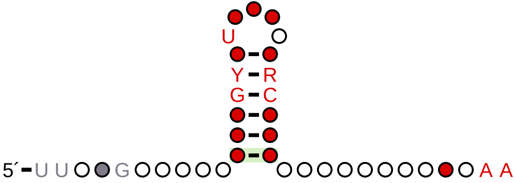

HMP2_6957_1

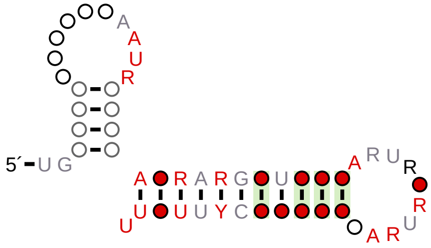

HMP2_6966_1

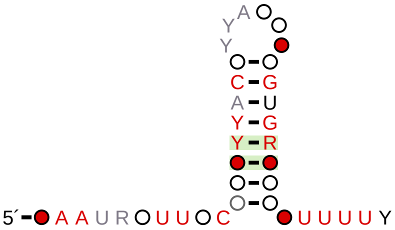

HMP2_6967_1

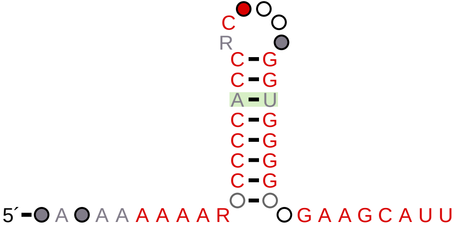

HMP2_6985_1

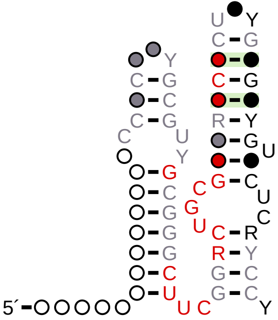

HMP2_6988_1

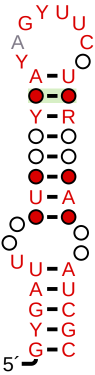

HMP2_699_1

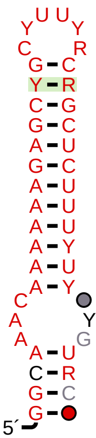

HMP2_6992_1

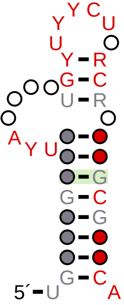

HMP2_6999_1

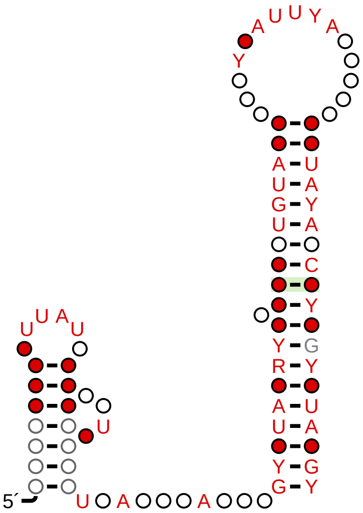

HMP2_7003_1

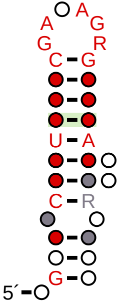

HMP2_7016_1

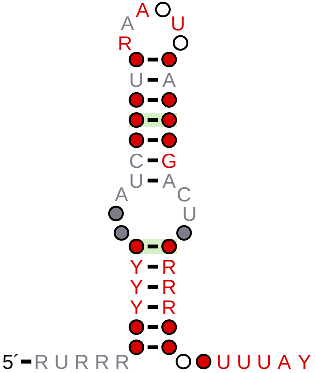

HMP2_7017_1

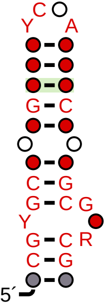

HMP2_7020_1

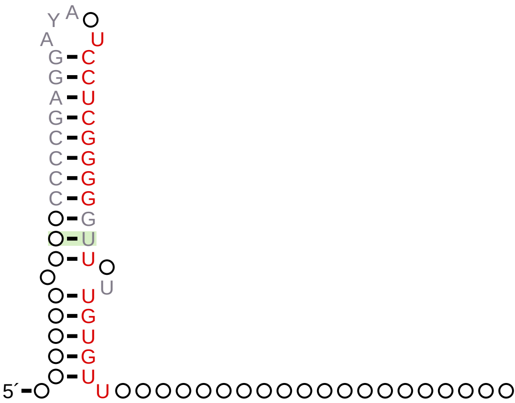

HMP2_7038_1

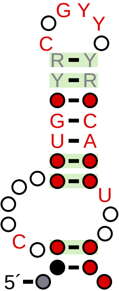

HMP2_7042_1

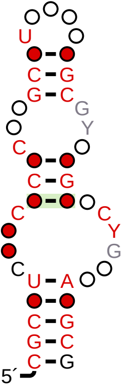

HMP2_7064_1

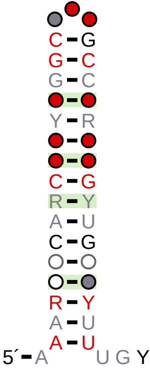

HMP2_7067_1

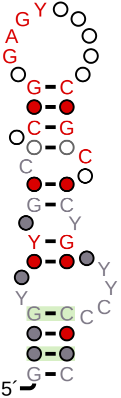

HMP2_7072_1

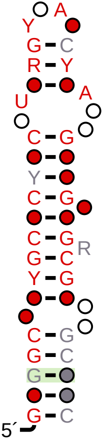

HMP2_7082_1

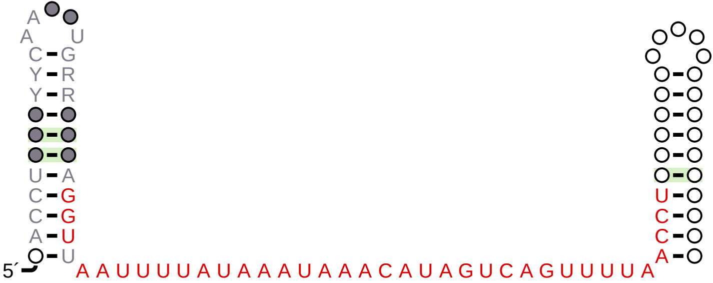

A
A
U
C G
Y R
Y R
U A
C G
C G
A U
U
5´
A A U U U U A U A A A U A A A C A U A G U C A G U U U U A
U
C
C
A

HMP2_7092_1

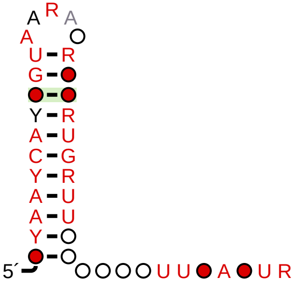

HMP2_7093_1

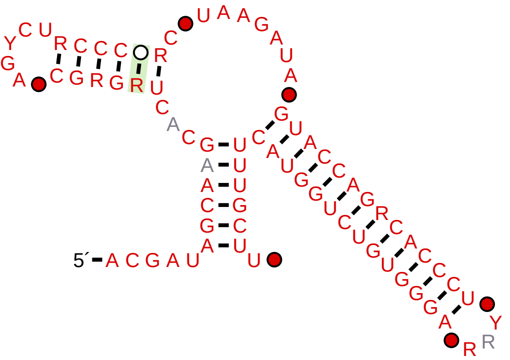

HMP2_71_1

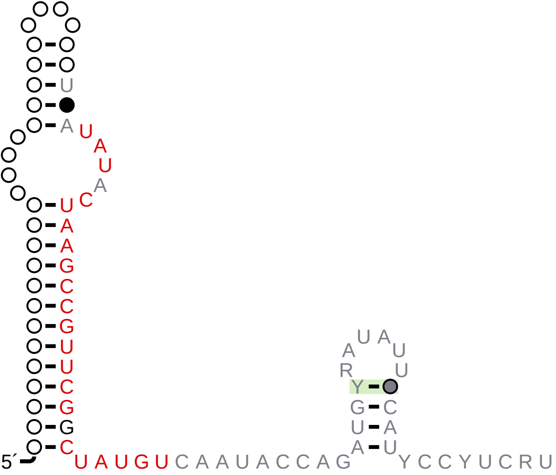

HMP2_710_1

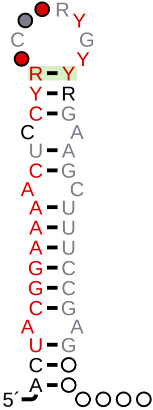

HMP2_7101_1

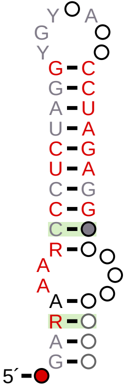

HMP2_7114_1

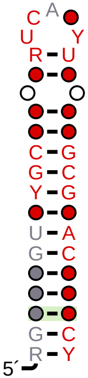

HMP2_712_1

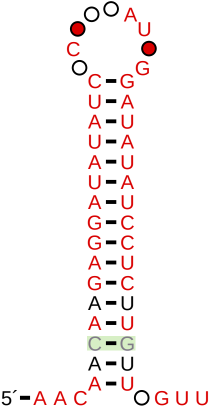

HMP2_7121_1

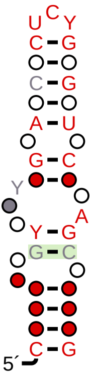

HMP2_7127_1

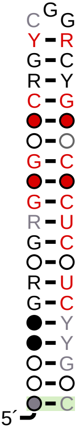

HMP2_7133_1

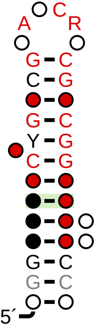

HMP2_7157_1

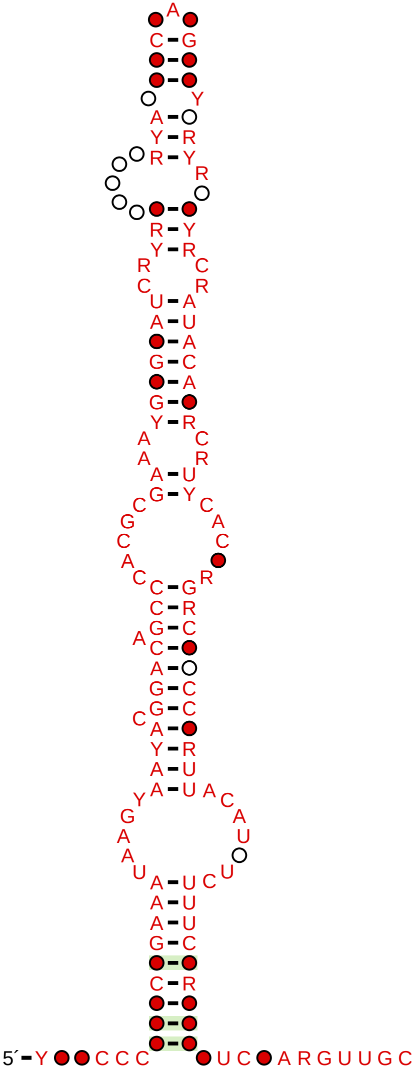

HMP2_7163_1

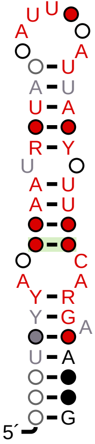

HMP2_7170_1

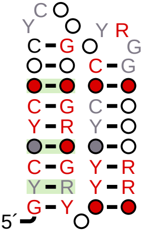

HMP2_7174_1

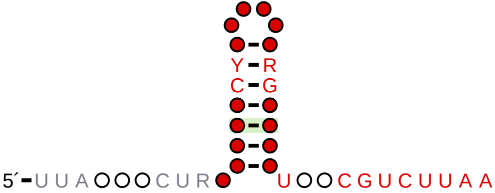

HMP2_7176_1

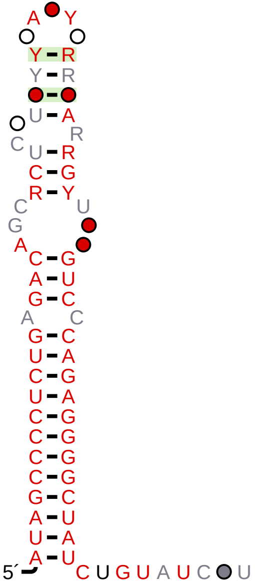

HMP2_719_1

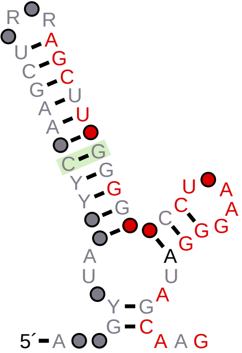

HMP2_7202_1

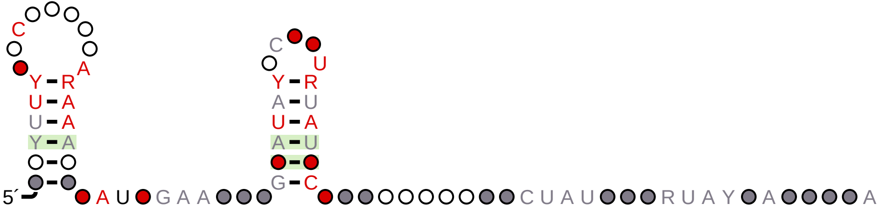

HMP2_7207_1

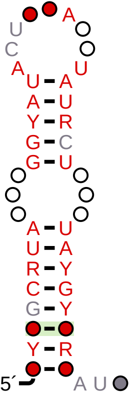

HMP2_721_1

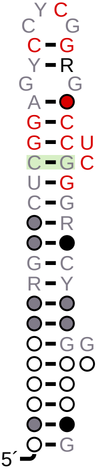

HMP2_722_1

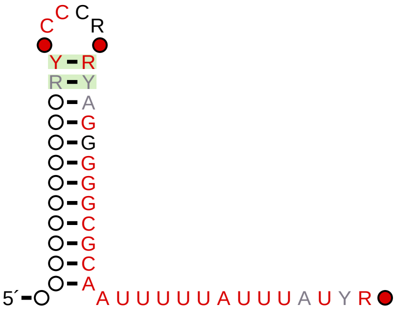

HMP2_7223_1

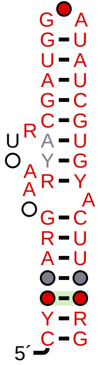

HMP2_7227_1

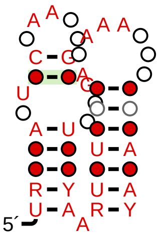

HMP2_7228_1

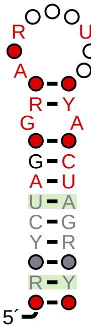

HMP2_7229_1

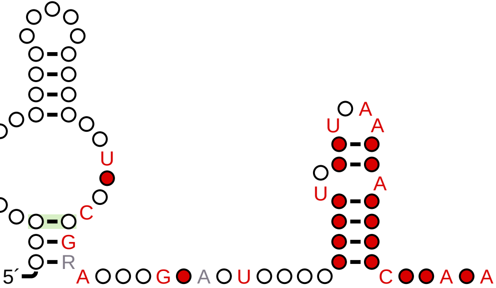

HMP2_723_1

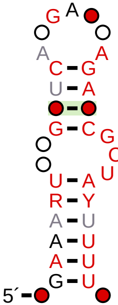

HMP2_7231_1

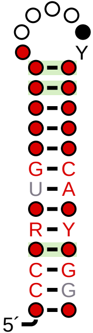

HMP2_7237_1

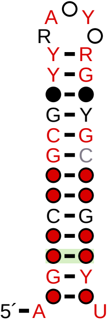

HMP2_7246_1

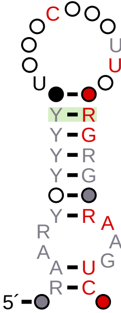

HMP2_7249_1

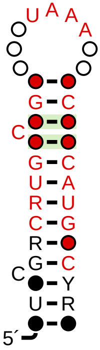

HMP2_7250_1

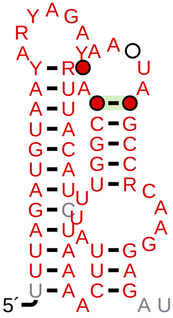

HMP2_7263_1

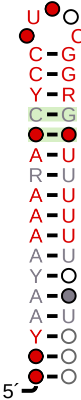

HMP2_7265_1

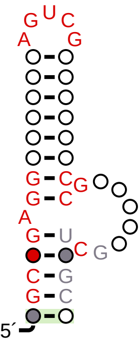

HMP2_7269_1

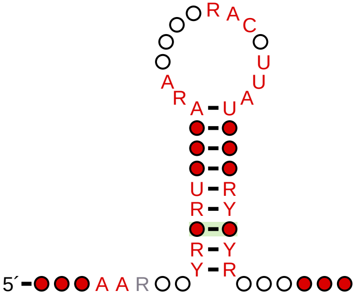

HMP2_7275_1

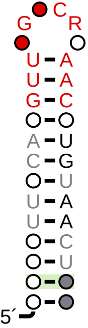

HMP2_7277_1

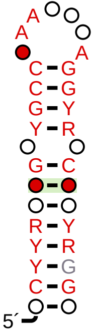

HMP2_728_1

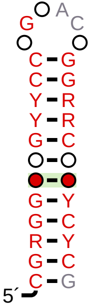

HMP2_7289_1

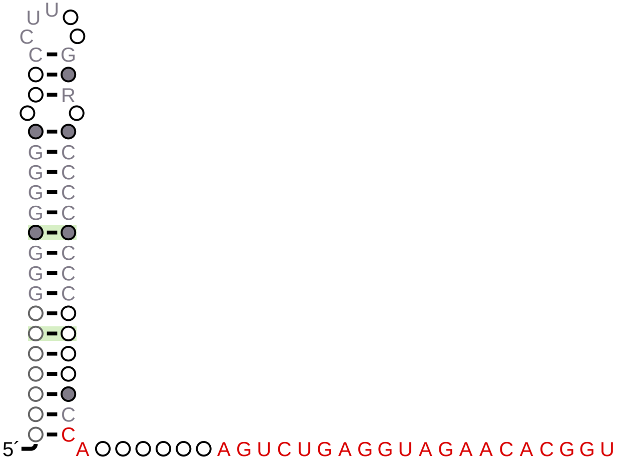

HMP2_7292_1

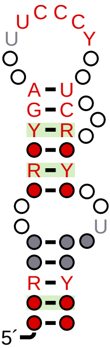

HMP2_7295_1

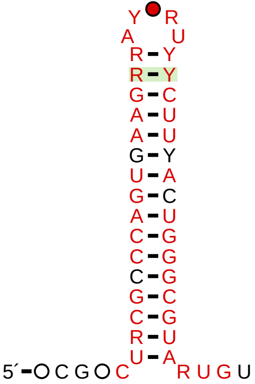

HMP2_7302_1

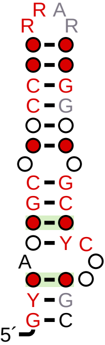

HMP2_7306_1

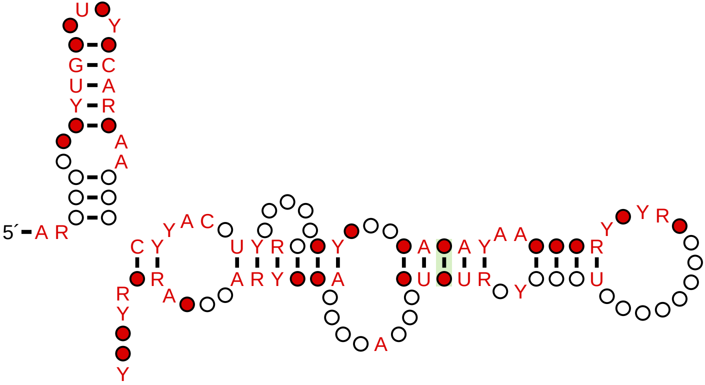

HMP2_731_1

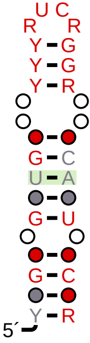

HMP2_7312_1

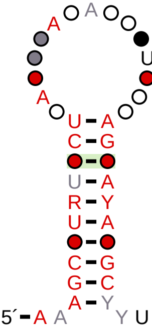

HMP2_7313_1

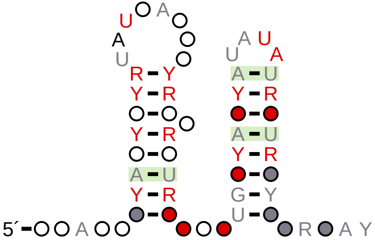

HMP2_7316_1

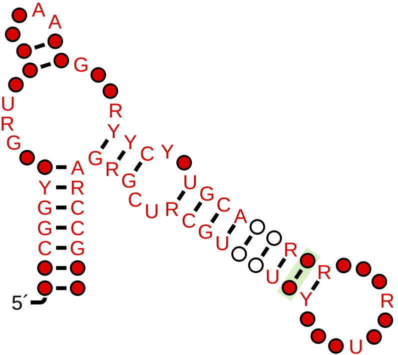

HMP2_7320_1

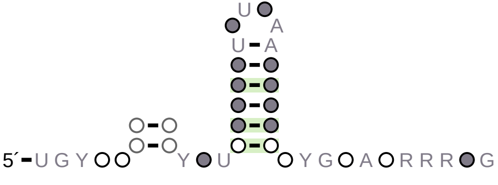

HMP2_7323_1

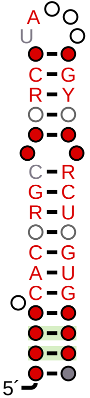

HMP2_7330_1

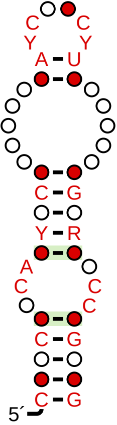

HMP2_7331_1

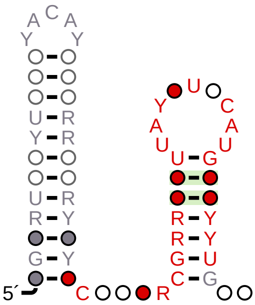

HMP2_7336_1

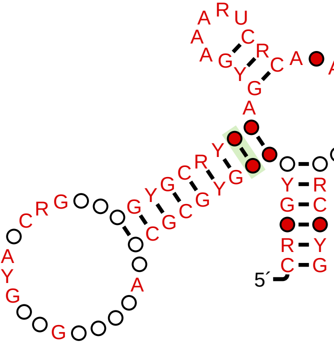

HMP2_7338_1

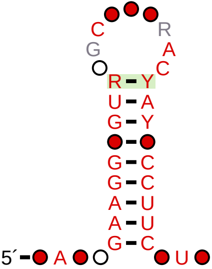

HMP2_734_1

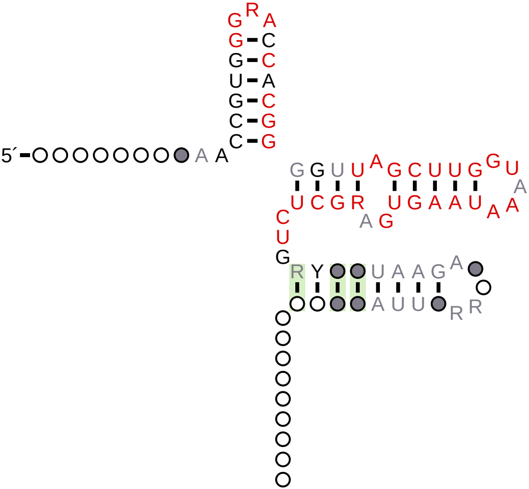

HMP2_7341_1

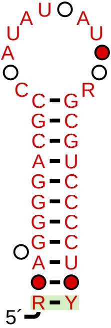

HMP2_7342_1

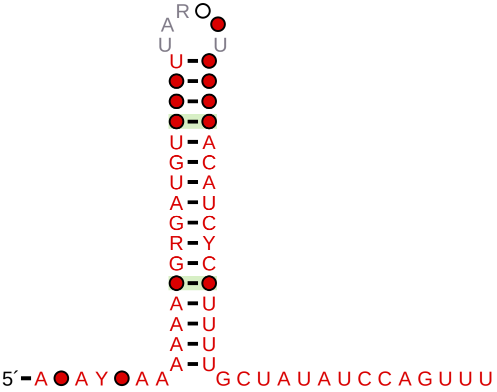

HMP2_7349_1

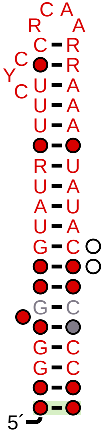

HMP2_7351_1

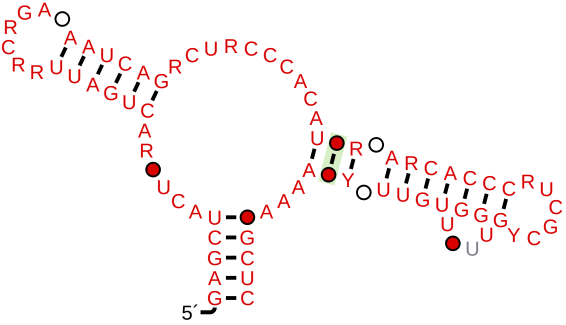

HMP2_7355_1

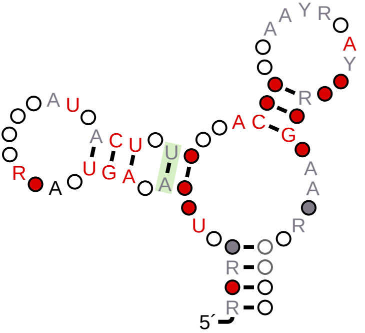

HMP2_7357_1

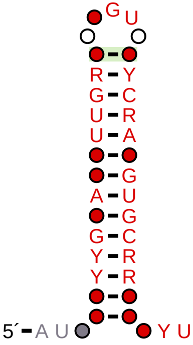

HMP2_7364_1

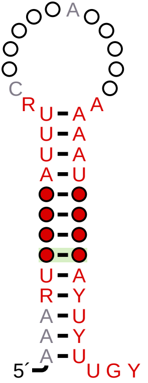

HMP2_7373_1

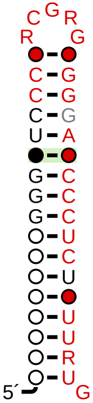

HMP2_7374_1

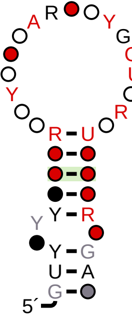

HMP2_7380_1

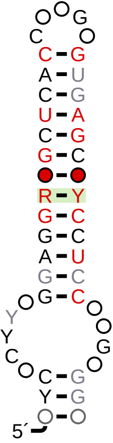

HMP2_739_1

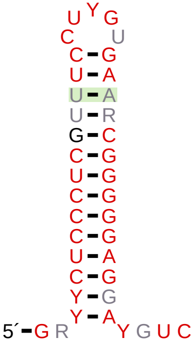

HMP2_7390_1

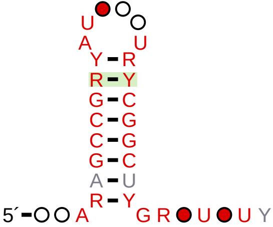

HMP2_7397_1

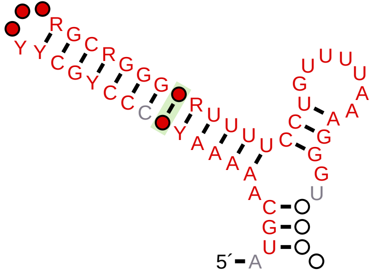

HMP2_74_1

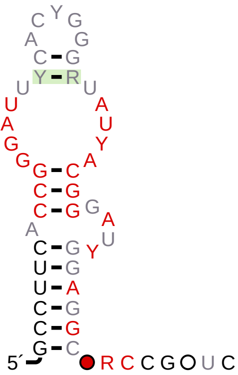

HMP2_7408_1

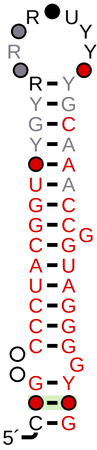

HMP2_7415_1

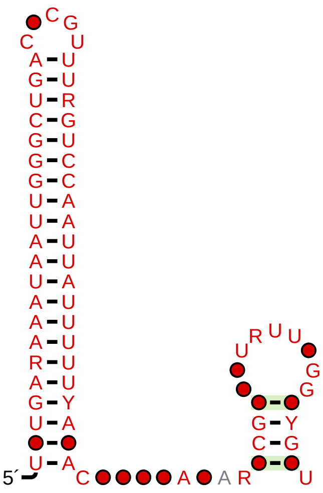

HMP2_7417_1

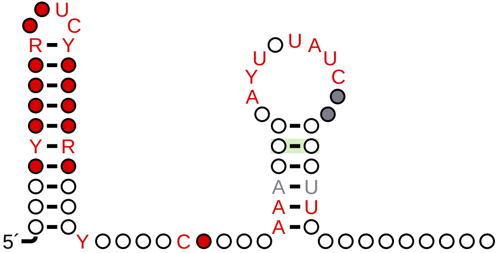

HMP2_742_1

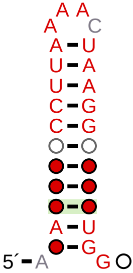

HMP2_7420_1

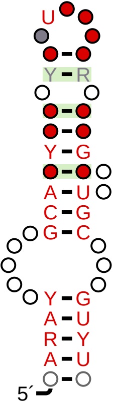

HMP2_7422_1

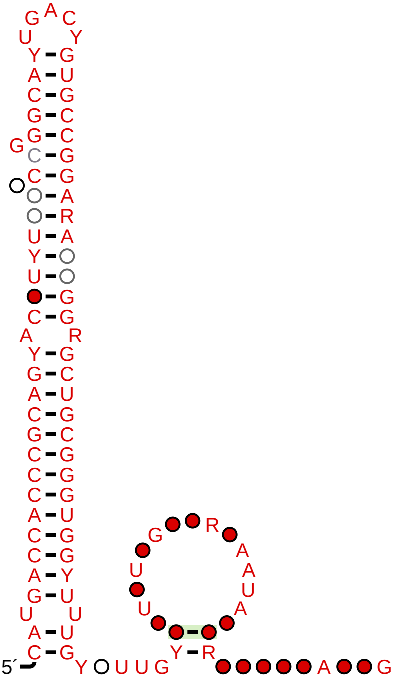

HMP2_7425_1

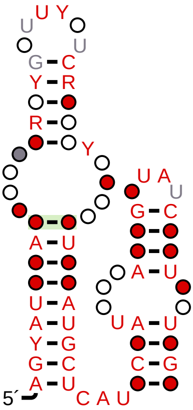

HMP2_7435_1

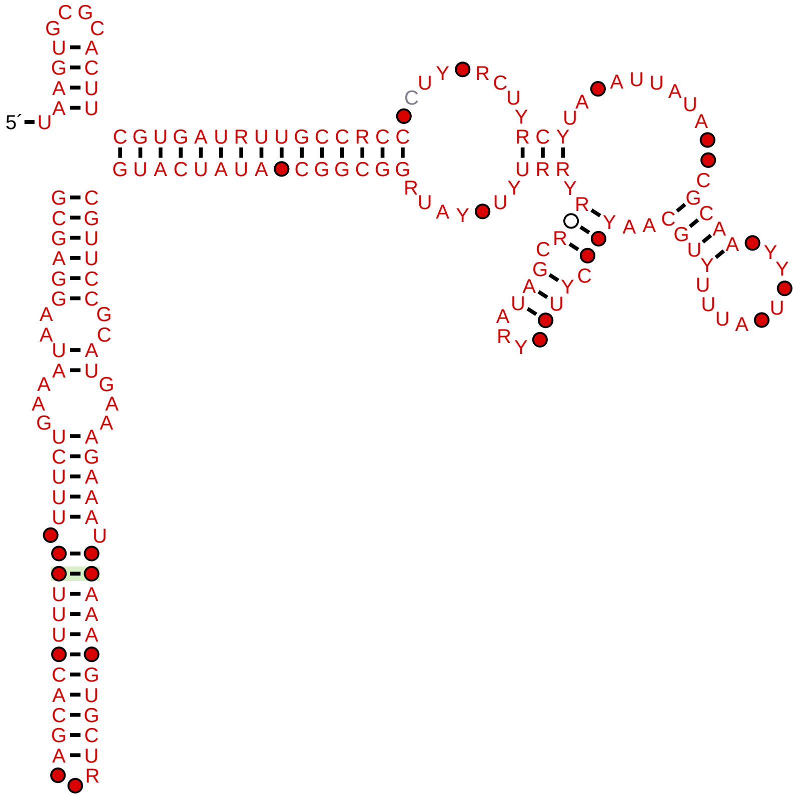

HMP2_7443_1

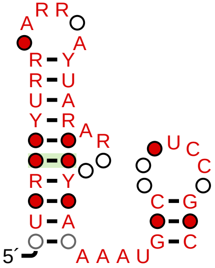

HMP2_7457_1

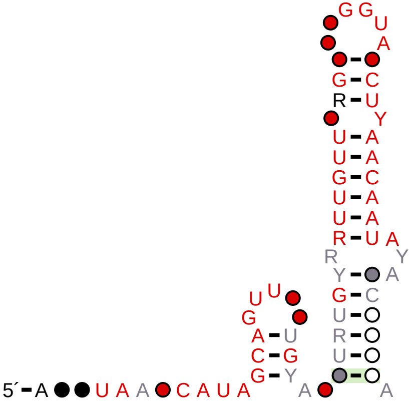

HMP2_7458_1

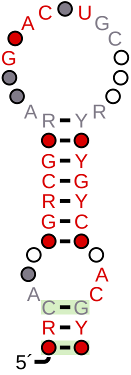

HMP2_7461_1

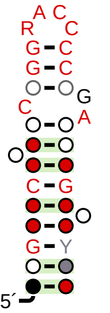

HMP2_7464_1

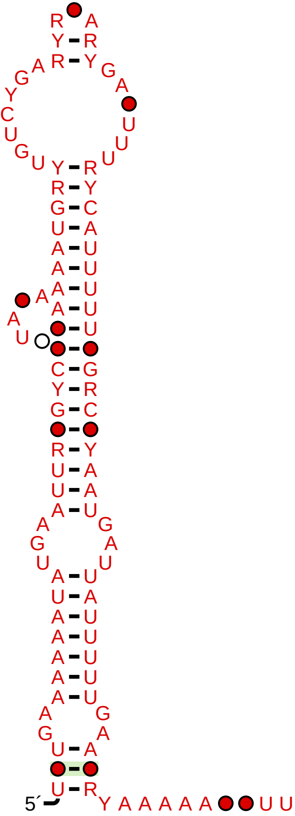

HMP2_7466_1

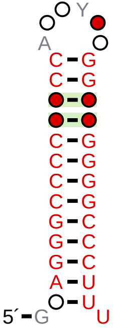

HMP2_7474_1

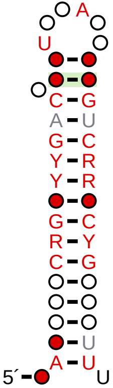

HMP2_7477_1

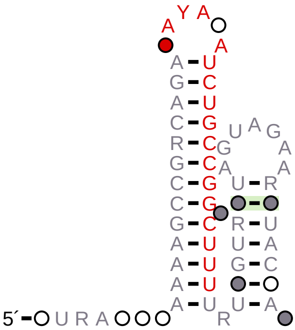

HMP2_7478_1

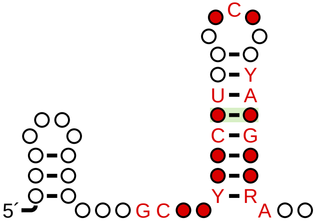

HMP2_7484_1

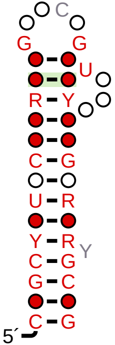

HMP2_749_1

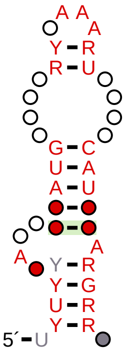

HMP2_7495_1

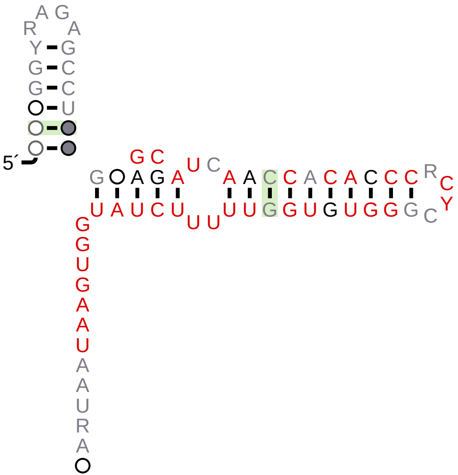

HMP2_7497_1

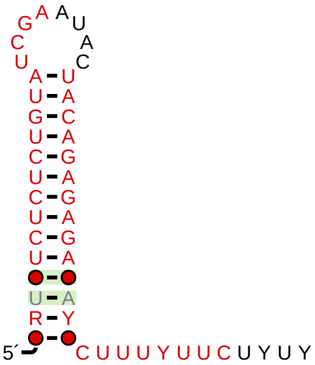

HMP2_7501_1

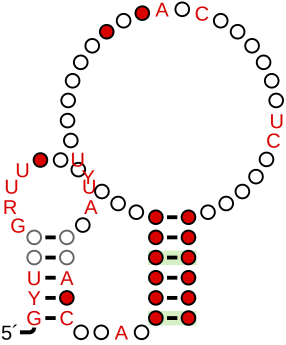

HMP2_751_1

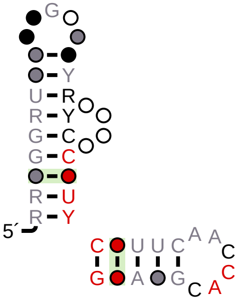

HMP2_7510_1

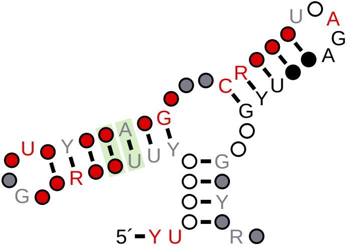

HMP2_7512_1

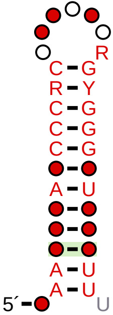

HMP2_7519_1

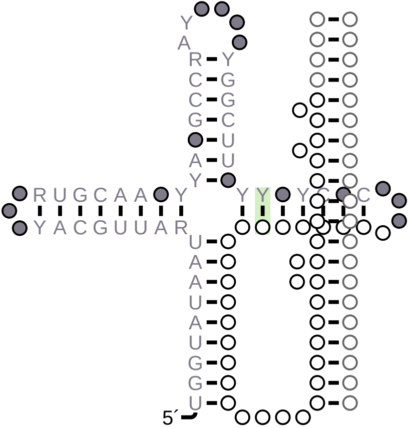

HMP2_7522_1

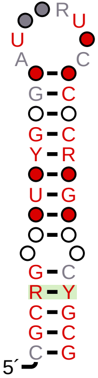

HMP2_7528_1

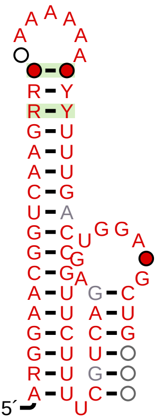

HMP2_7529_1

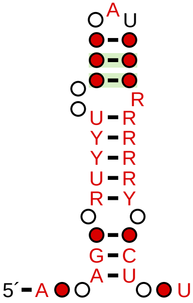

HMP2_753_1

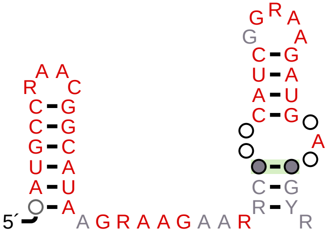

HMP2_7530_1

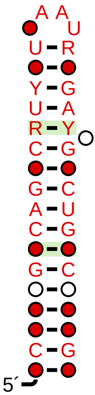

HMP2_7535_1

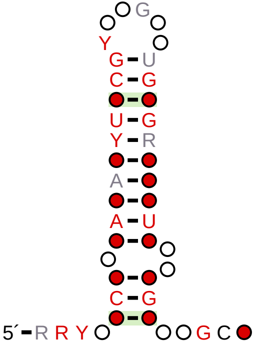

HMP2_7542_1

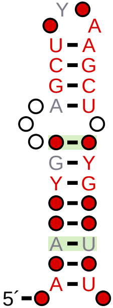

HMP2_7547_1

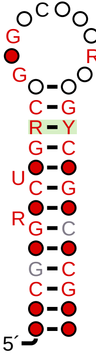

HMP2_7549_1

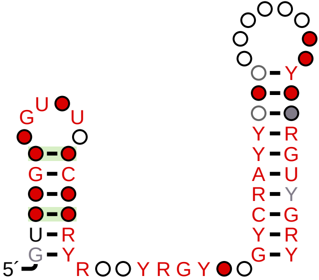

HMP2_7555_1

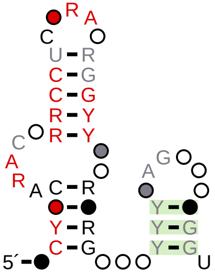

HMP2_7569_1

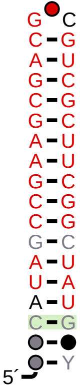

HMP2_7573_1

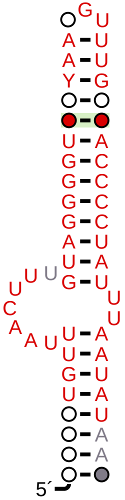

HMP2_7577_1

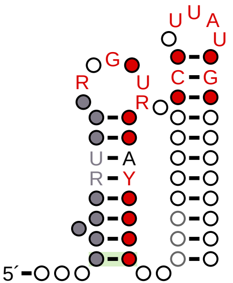

HMP2_758_1

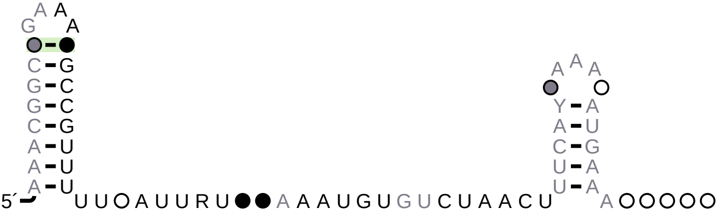

HMP2_7584_1

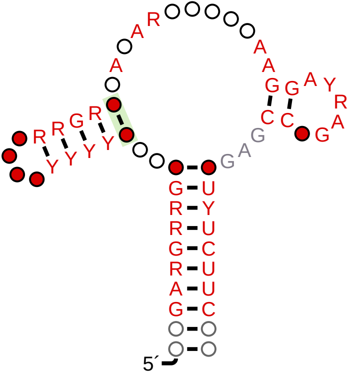

HMP2_7595_1

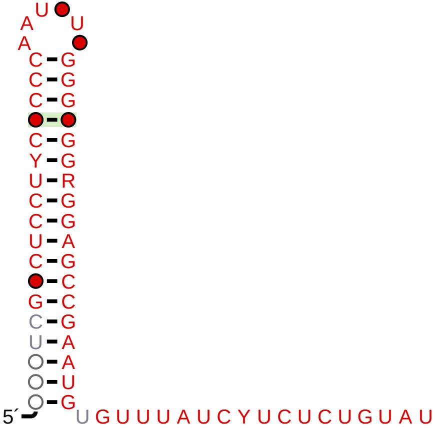

U
A U
A
C G
C G
C G
C G
Y G
U R
C G
C G
U A
C G
C
G C
C G
U A
A
U
G
5´
U G U U U A U C Y U C U C U G U A U

HMP2_7605_1

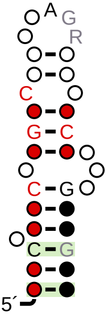

HMP2_7627_1

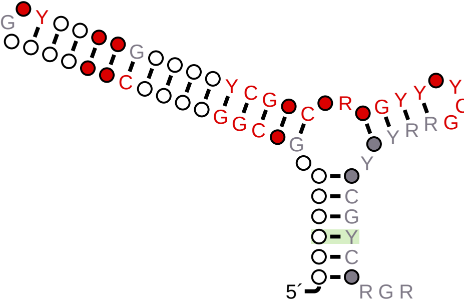

HMP2_7628_1

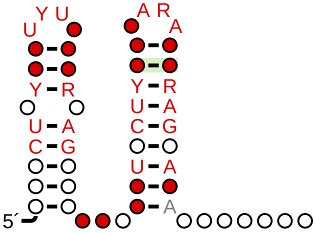

HMP2_7632_1

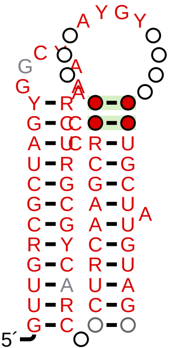

HMP2_7640_1

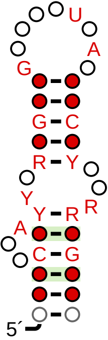

HMP2_7642_1

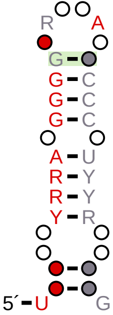

HMP2_7643_1

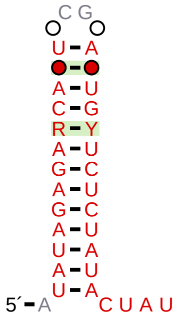

HMP2_7653_1

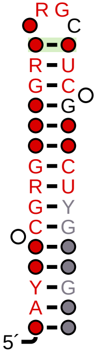

HMP2_7659_1

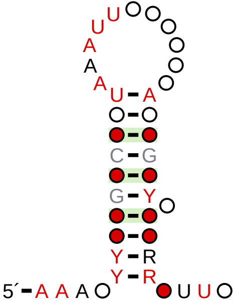

HMP2_7660_1

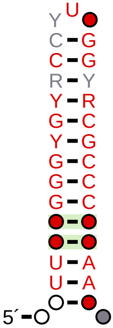

HMP2_7663_1

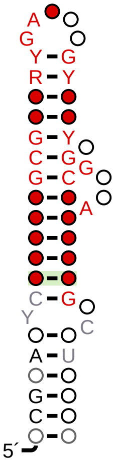

HMP2_7667_1

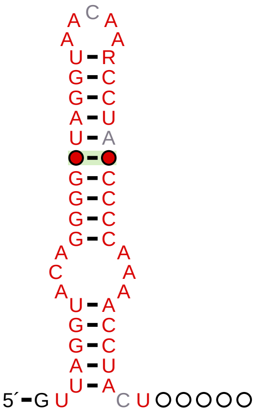

HMP2_7669_1

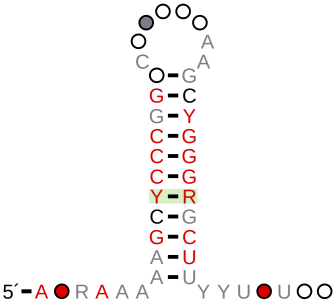

HMP2_767_1

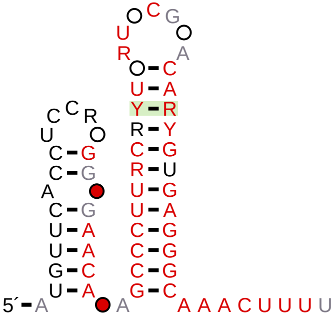

HMP2_7674_1

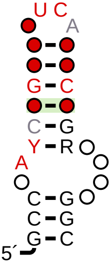

HMP2_7676_1

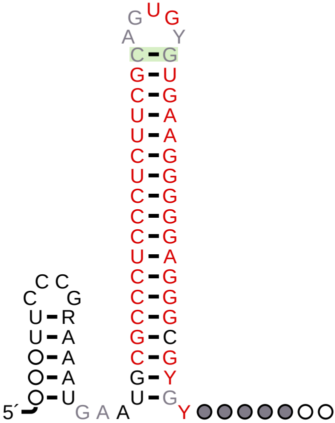

HMP2_7687_1

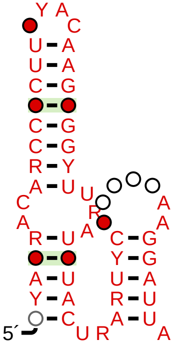

HMP2_7697_1

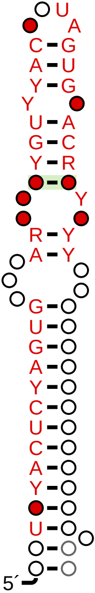

HMP2_771_1

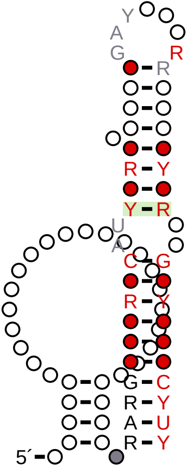

HMP2_7710_1

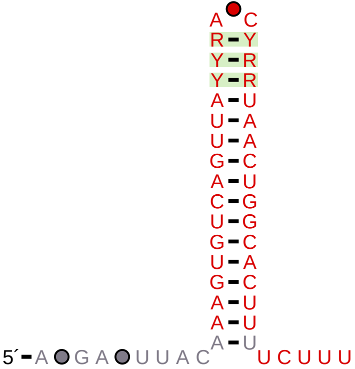

HMP2_7712_1

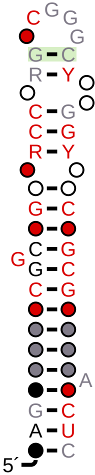

HMP2_7714_1

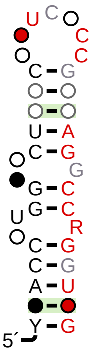

HMP2_7718_1

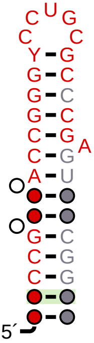

HMP2_7725_1

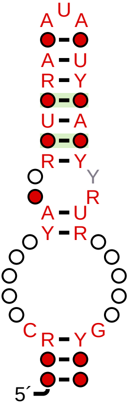

HMP2_7730_1

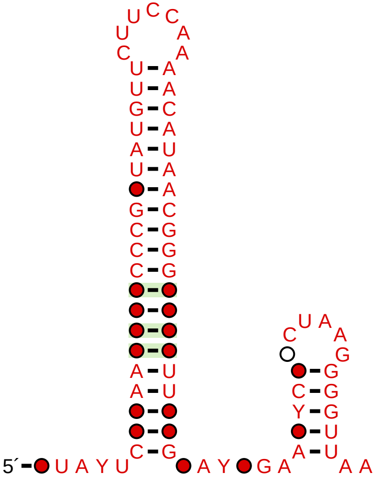

HMP2_7733_1

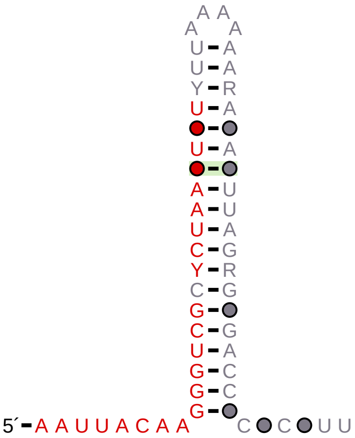

HMP2_7743_1

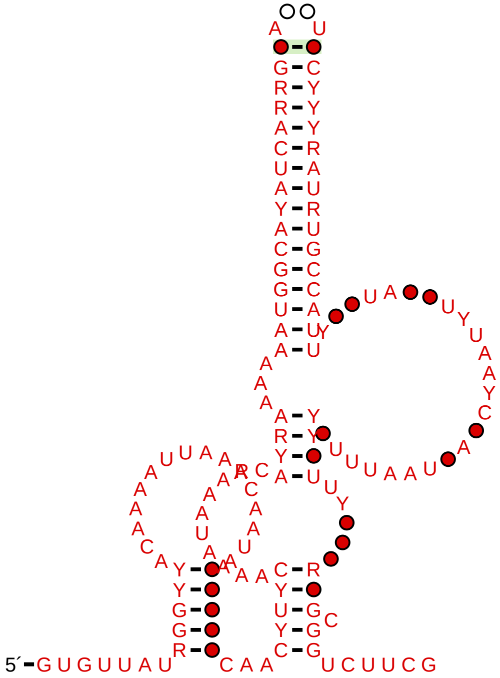

HMP2_7747_1

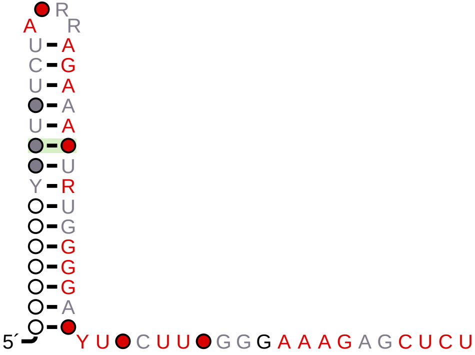

HMP2_7748_1

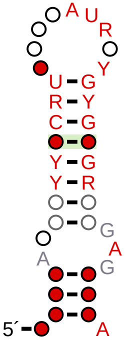

HMP2_7753_1

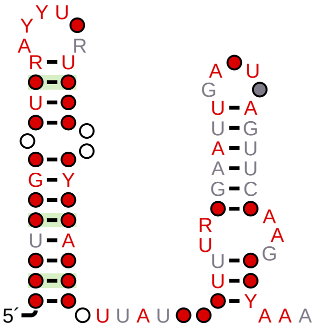

HMP2_7756_1

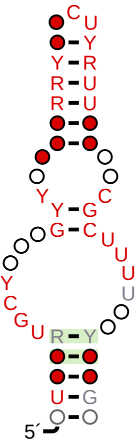

HMP2_7757_1

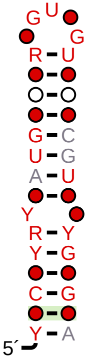

HMP2_7766_1

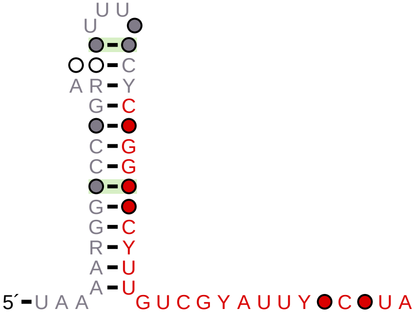

HMP2_777_1

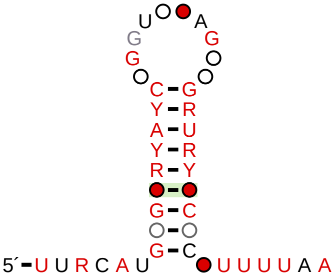

HMP2_7771_1

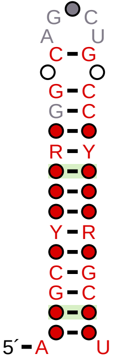

HMP2_7775_1

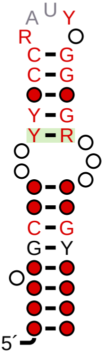

HMP2_7776_1

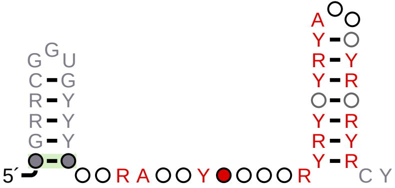

HMP2_7777_1

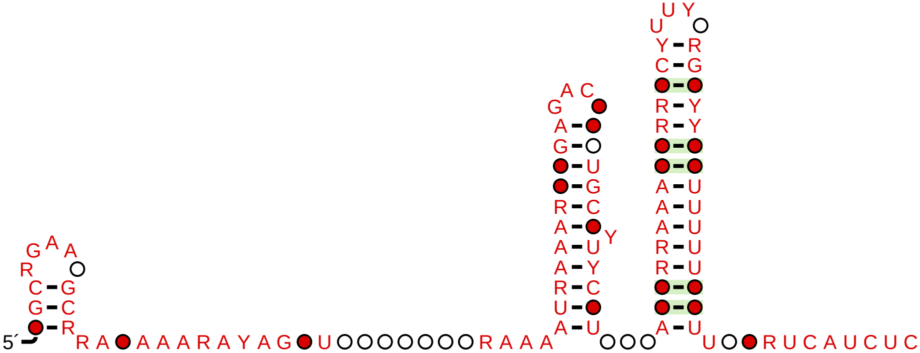

HMP2_778_1

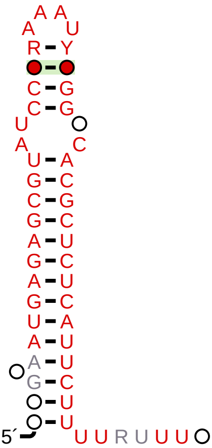

HMP2_7790_1

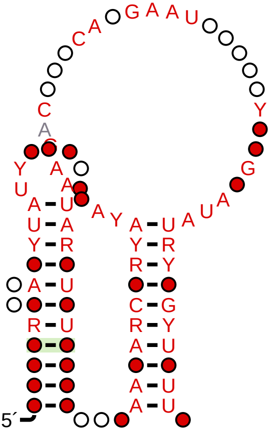

HMP2_7794_1

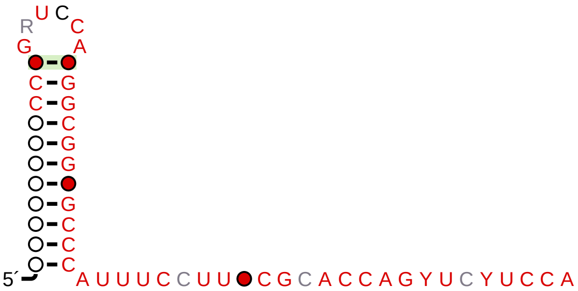

HMP2_7797_1

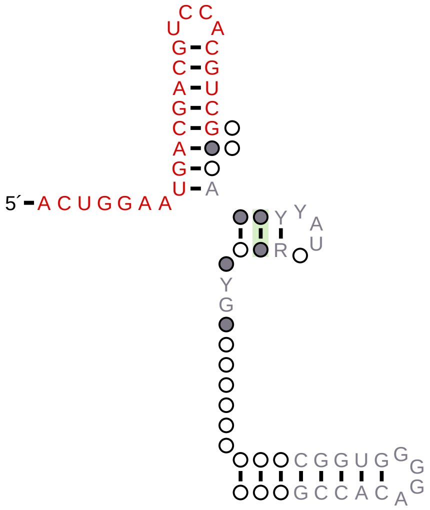

HMP2_7798_1

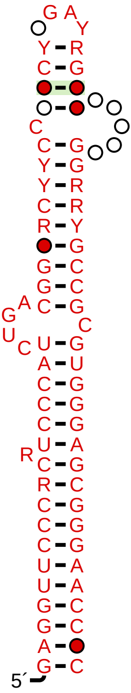

G A
Y
Y R
C G
C
C G
Y G
Y R
C R
R Y
G
G C
G C
A
G C G
C
U
C U G
A U
C G
C G
C G
U A
R
C G
R C
C G
C G
C G
U A
U A
G C
G C
A
G C
5´

HMP2_7799_1

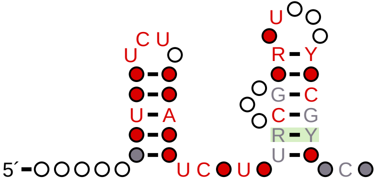

HMP2_7802_1

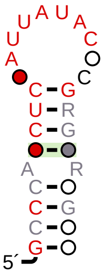

HMP2_7805_1

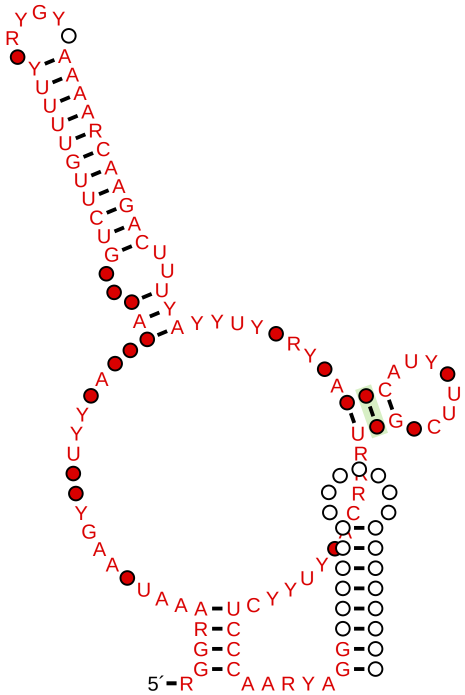

HMP2_781_1

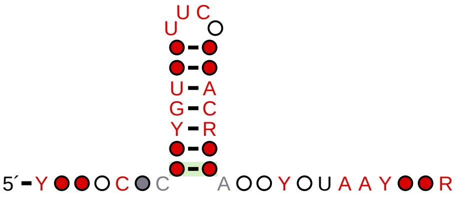

HMP2_7813_1

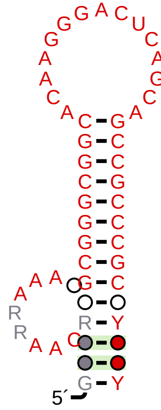

HMP2_7816_1

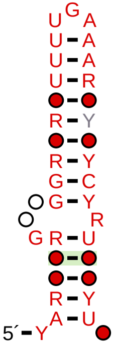

HMP2_7819_1

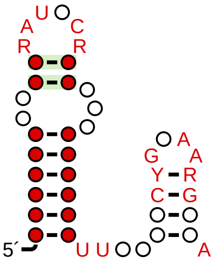

HMP2_7825_1

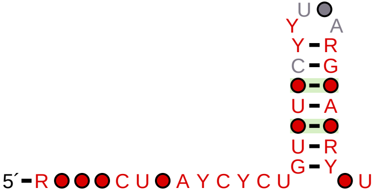

HMP2_7831_1

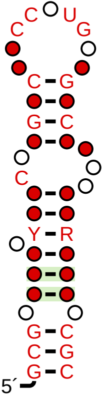

HMP2_7835_1

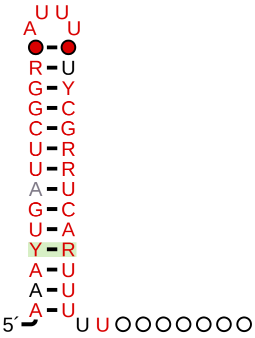

HMP2_7838_1

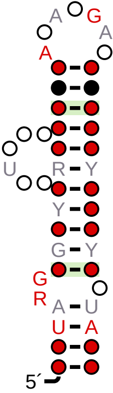

HMP2_7842_1

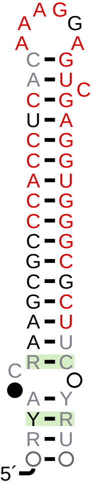

HMP2_7845_1

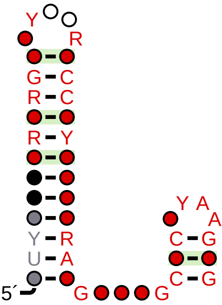

HMP2_7848_1

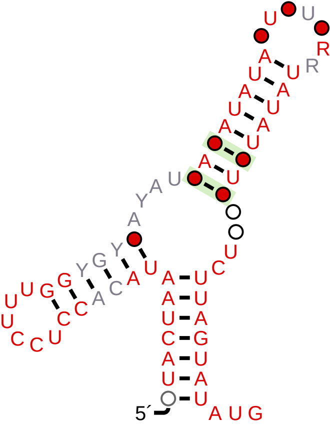

HMP2_7849_1

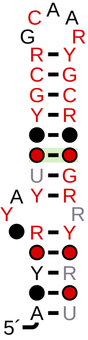

HMP2_7859_1

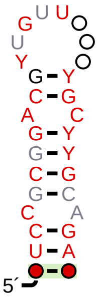

HMP2_786_1

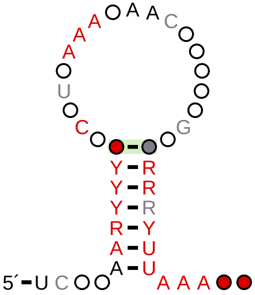

HMP2_7866_1

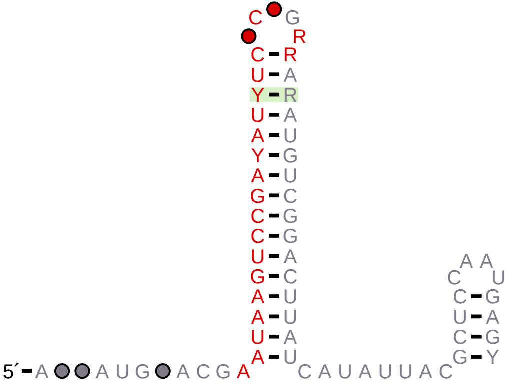
5´
A A U G A C G A
C U Y U A Y A G C C U G A A U A
C G R R A R A U G U C G G A C U U A U
C A U A U U A C
G C U C C A A U G A G Y

HMP2_787_1

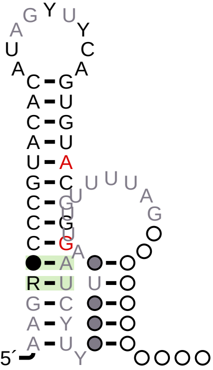

HMP2_7871_1

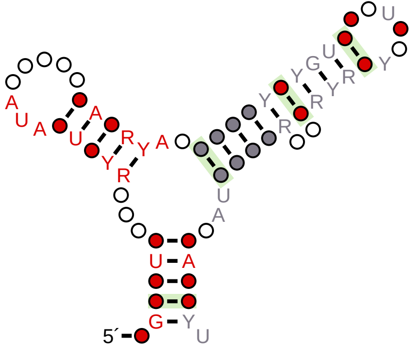

HMP2_7874_1

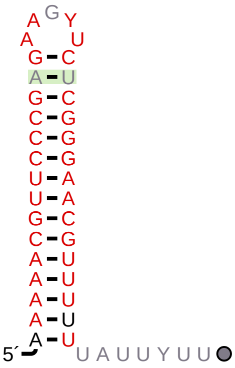

HMP2_7883_1

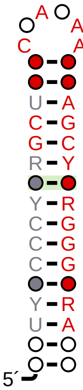

HMP2_7884_1

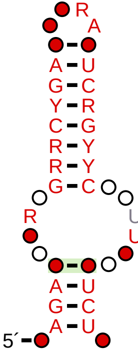

HMP2_7886_1

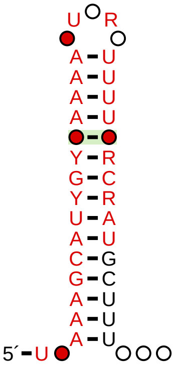

HMP2_7888_1

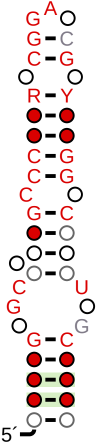

HMP2_7893_1

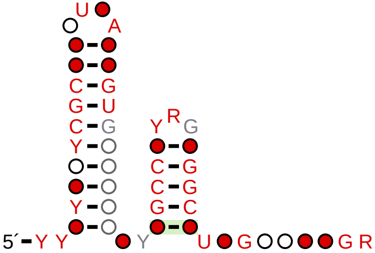

HMP2_7894_1

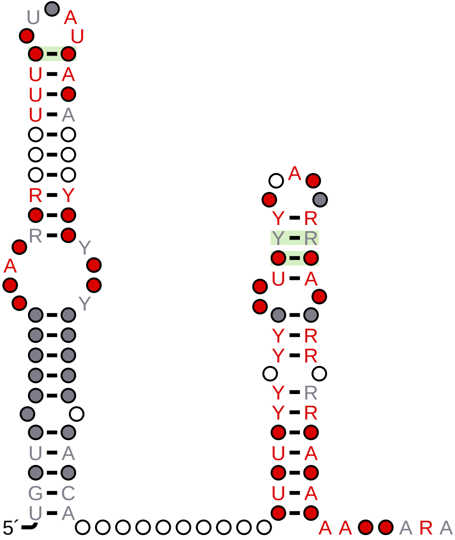

HMP2_7917_1

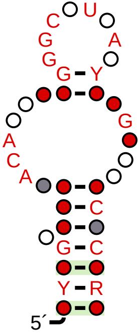

HMP2_7918_1

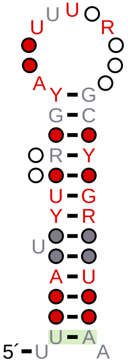

HMP2_7919_1

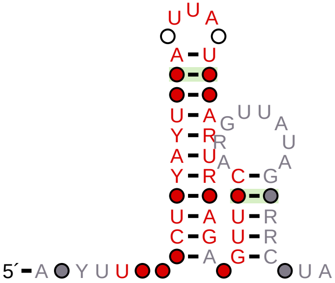

HMP2_792_1

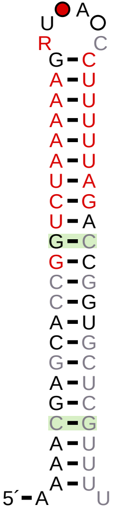

HMP2_7926_1

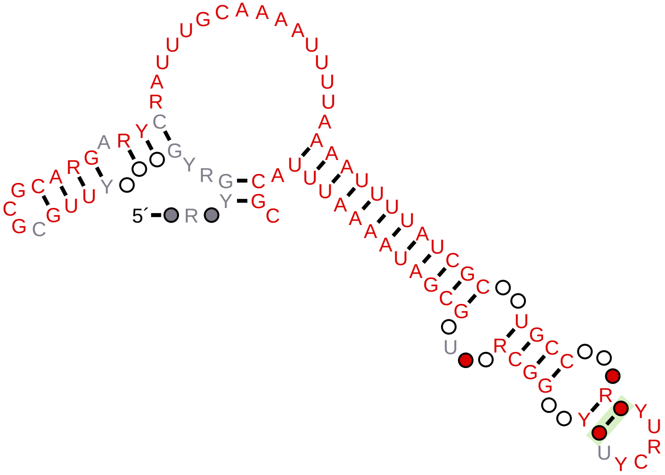

HMP2_7929_1

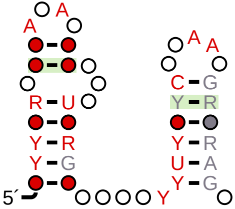

HMP2_7937_1

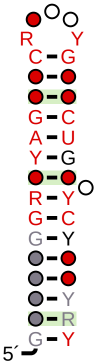

HMP2_7943_1

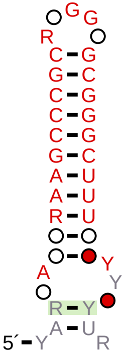

HMP2_7950_1

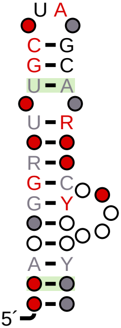

HMP2_7952_1

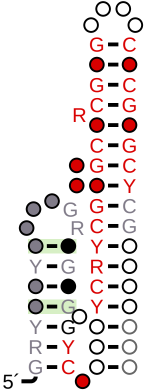

HMP2_7957_1

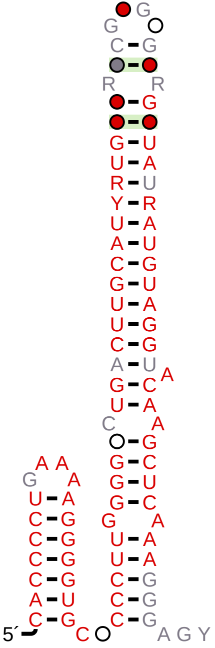

HMP2_7962_1

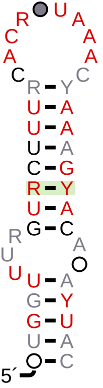

HMP2_7964_1

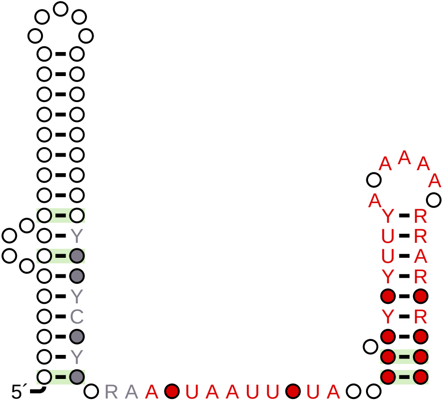

HMP2_7975_1

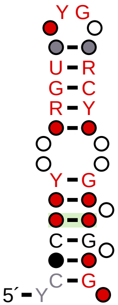

HMP2_7976_1

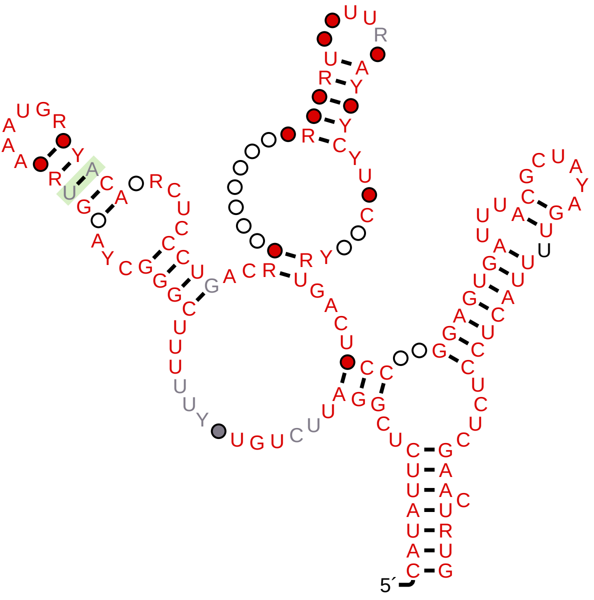

HMP2_7979_1

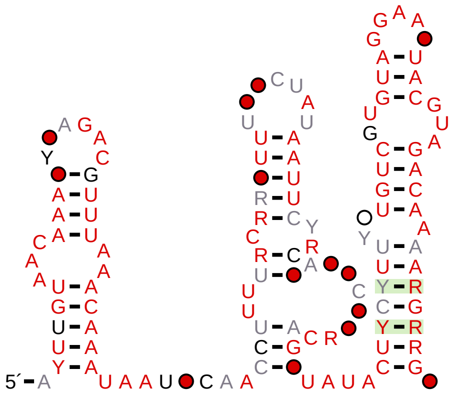

HMP2_7980_1

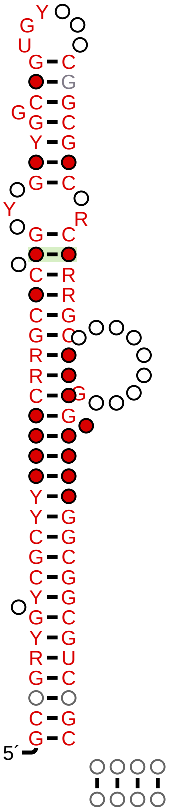

HMP2_7982_1

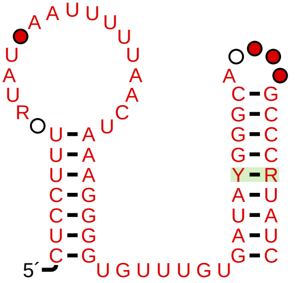

HMP2_8006_1

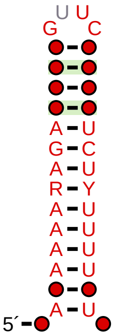

HMP2_8009_1

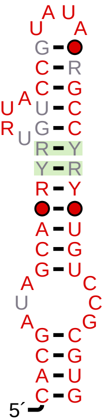

HMP2_8015_1

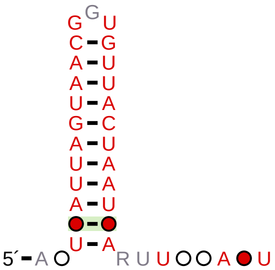

HMP2_8026_1

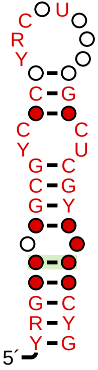

HMP2_8028_1

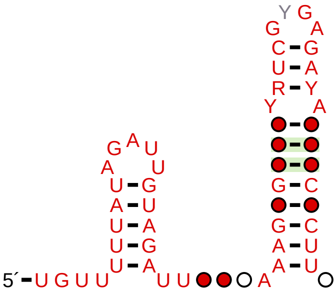

HMP2_8036_1

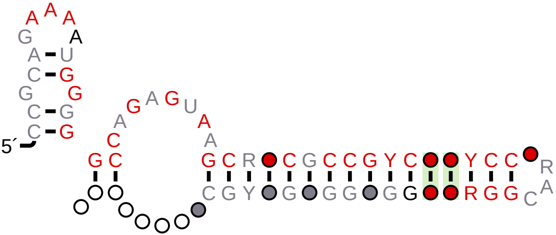

HMP2_8041_1

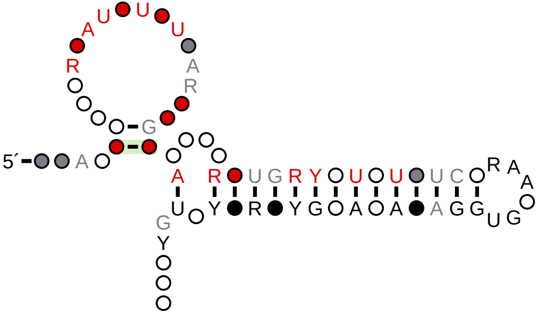

HMP2_8042_1

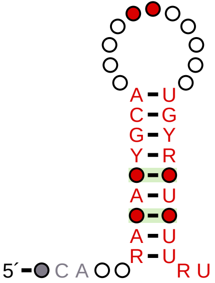

HMP2_8046_1

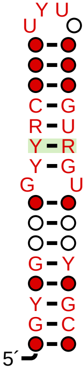

HMP2_8048_1

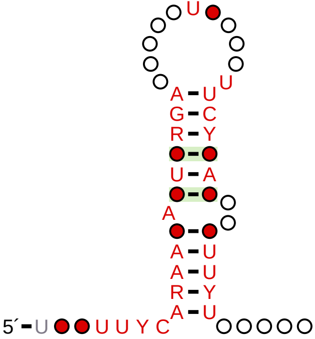

HMP2_8054_1

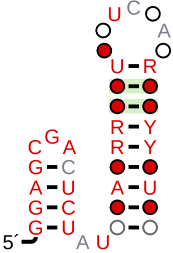

HMP2_8058_1

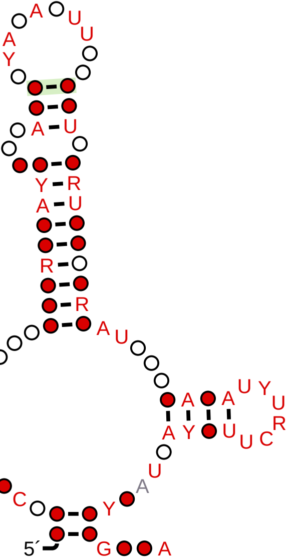

HMP2_8060_1

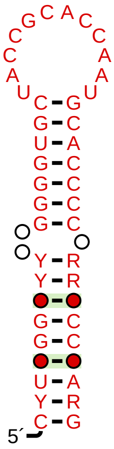

HMP2_8067_1

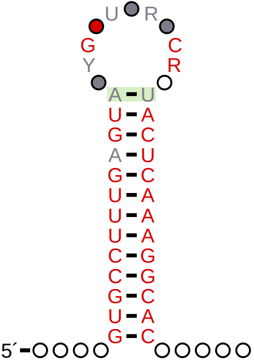

HMP2_8069_1

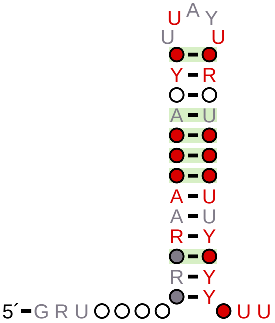

HMP2_8073_1

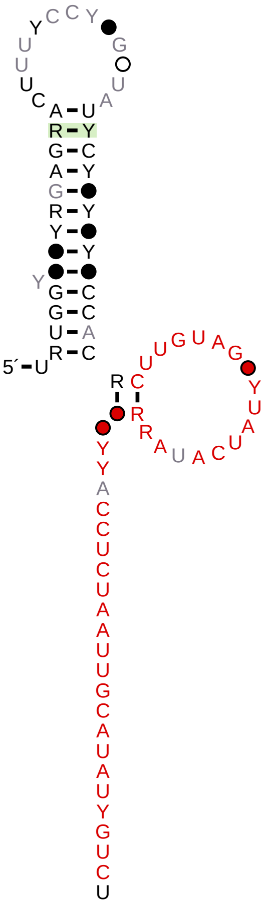

HMP2_8074_1

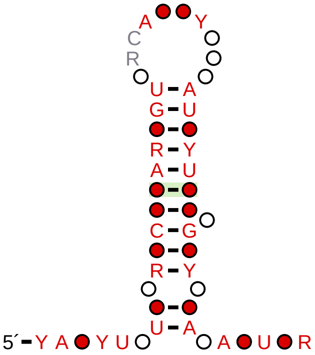

HMP2_8079_1

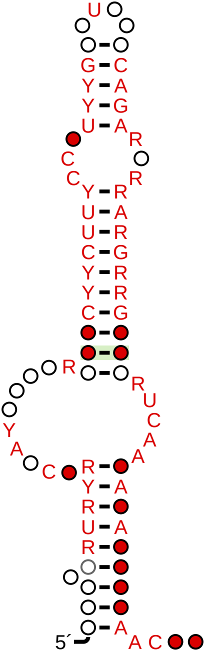

HMP2_808_1

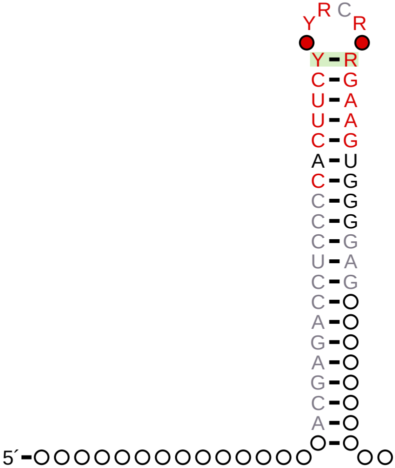

HMP2_8087_1

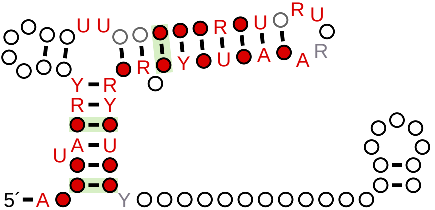

HMP2_8094_1

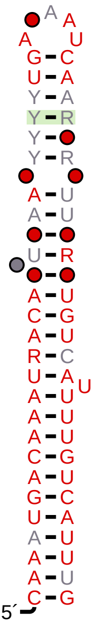

HMP2_8095_1

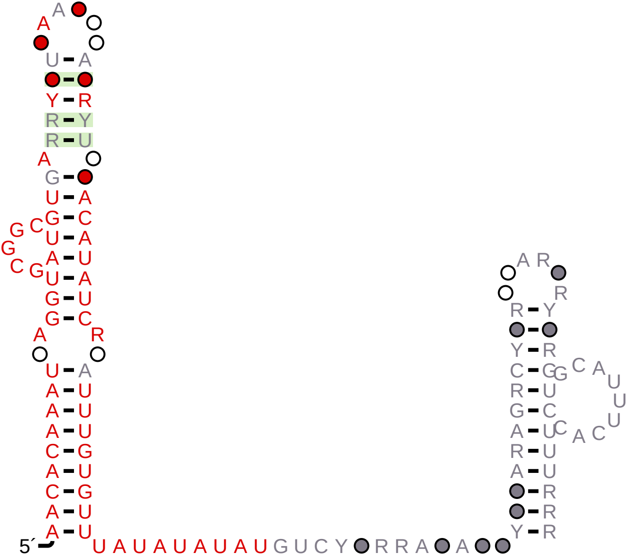
5´
A
U
A
R
U
A
G
Y
R
R
Y
R
U
A
G
A
U
G
C
G
G
C
G
U
A
C
A
U
A
U
G
U
G
C
A
R
U
A
A
U
A
U
A
U
C
G
A
U
C
G
A
U
A
U
U A U A U A U A U G U C Y R R A A
A R
R
R Y
Y R
C G G C A
U
U
U
R U
G C
A U C A C
R U
A U
R
R
Y R

HMP2_8096_1

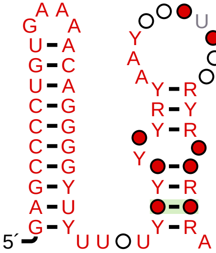

HMP2_8097_1

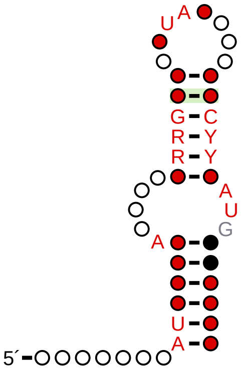

HMP2_8109_1

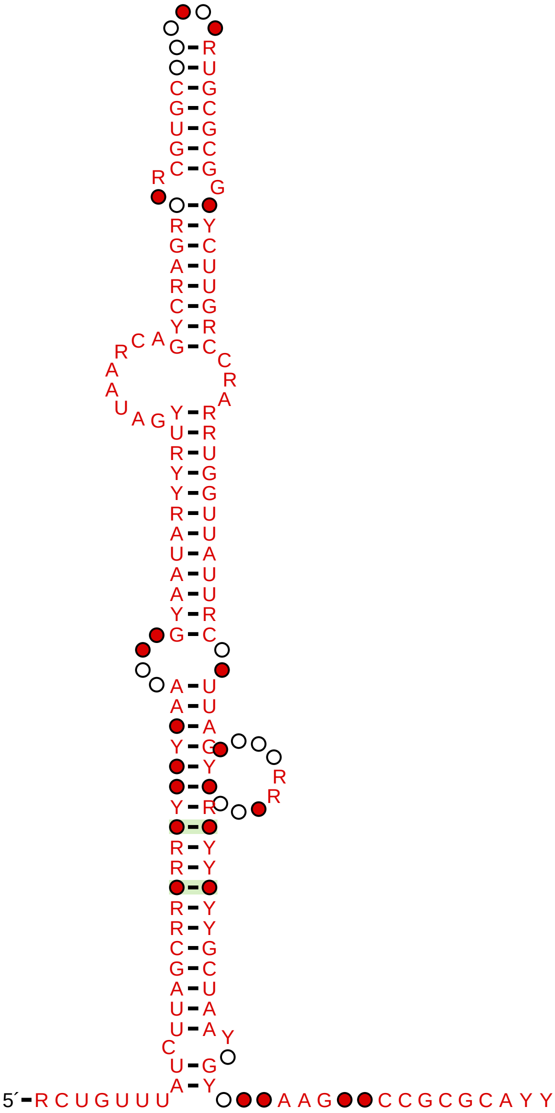

HMP2_8110_1

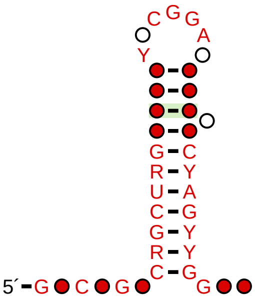

HMP2_8111_1

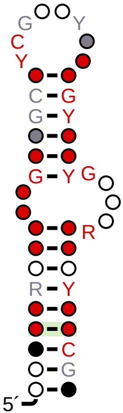

HMP2_8114_1

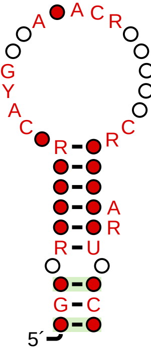

HMP2_8115_1

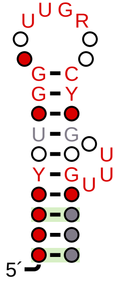

HMP2_8116_1

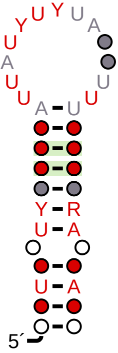

HMP2_8121_1

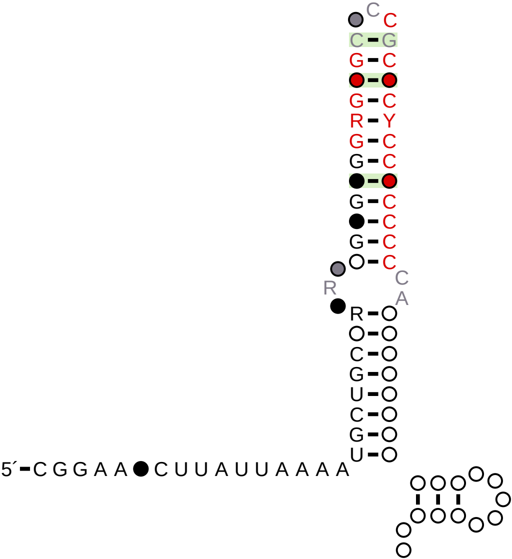

HMP2_8122_1

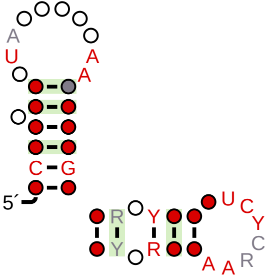

HMP2_8123_1

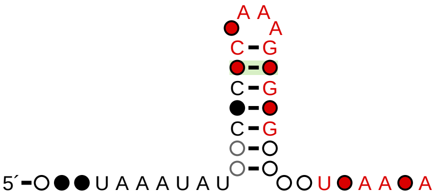

HMP2_8134_1

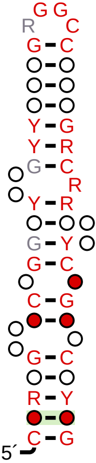

HMP2_8135_1

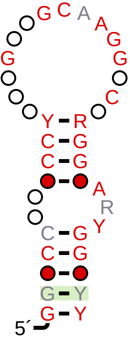

HMP2_8139_1

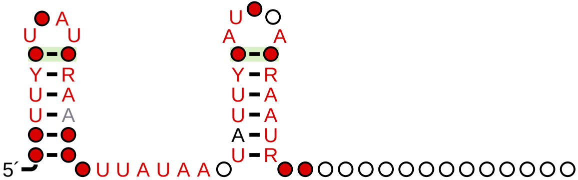

HMP2_8140_1

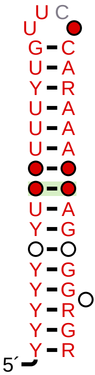

HMP2_8141_1

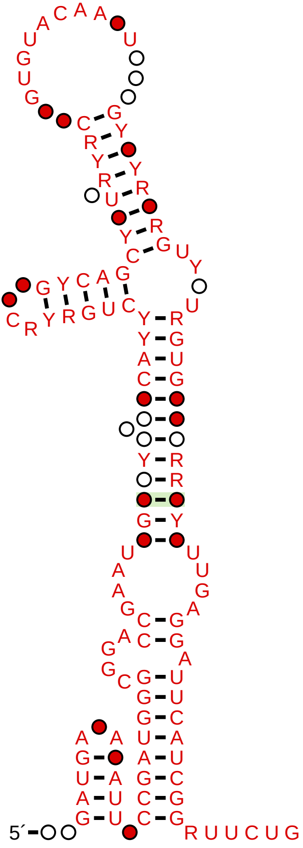

HMP2_815_1

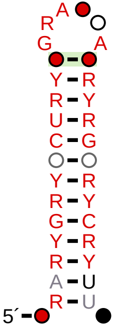

HMP2_8154_1

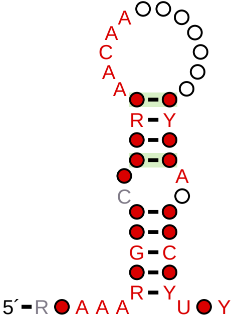

HMP2_8155_1

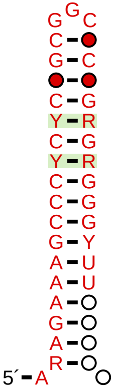

HMP2_8157_1

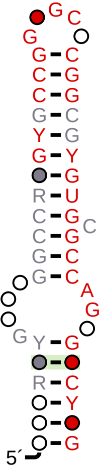

HMP2_8165_1

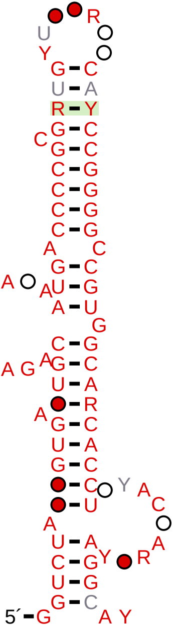

HMP2_82_1

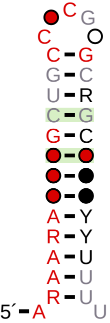

HMP2_821_1

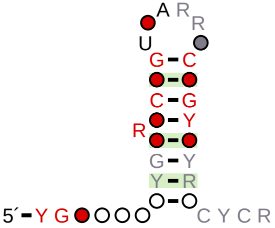

HMP2_8215_1

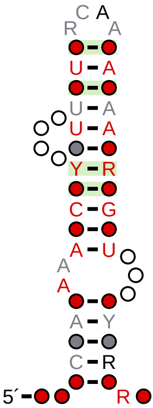

HMP2_8218_1

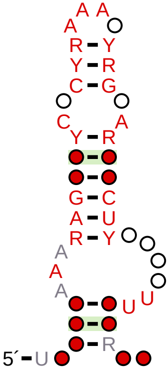

HMP2_822_1

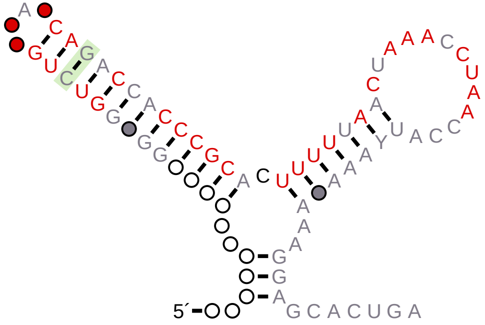

HMP2_8224_1

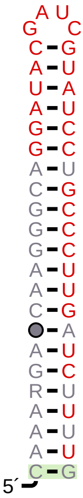

HMP2_8227_1

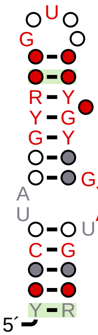

HMP2_8234_1

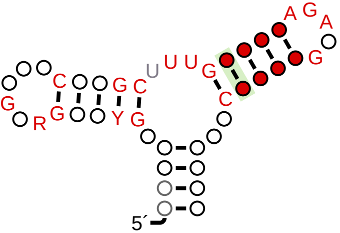

HMP2_8235_1

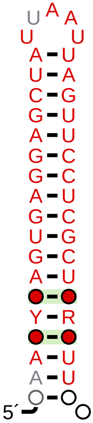

HMP2_8237_1

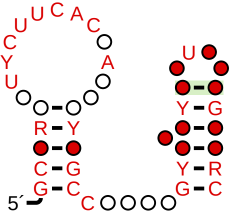

HMP2_8243_1

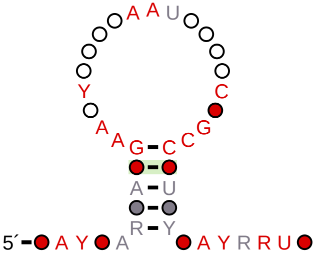

HMP2_8247_1

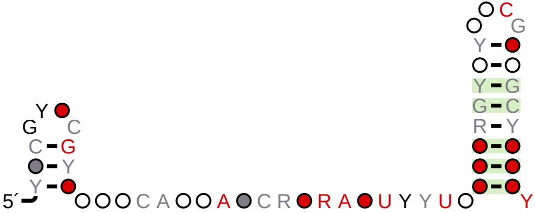

HMP2_8256_1

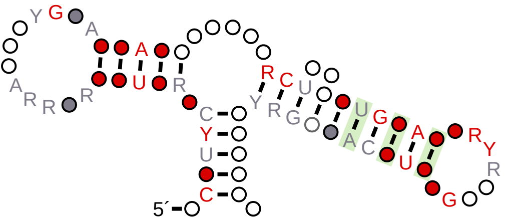

HMP2_8264_1

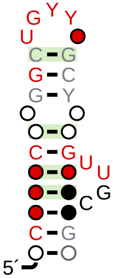

HMP2_8265_1

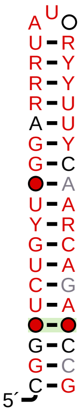

HMP2_8274_1

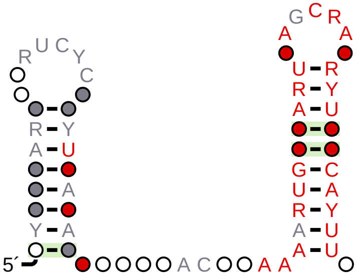

HMP2_8279_1

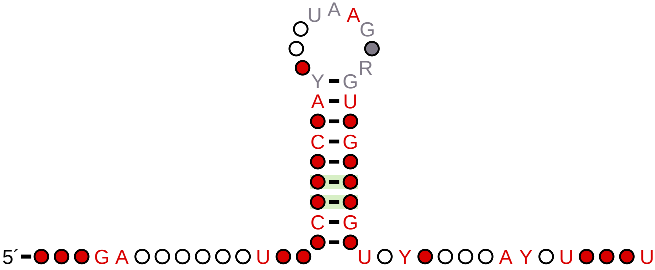

HMP2_8280_1

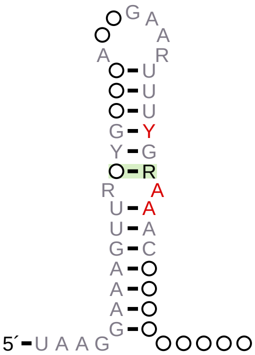

HMP2_8288_1

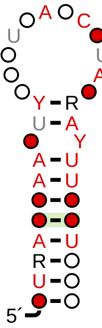

HMP2_8289_1

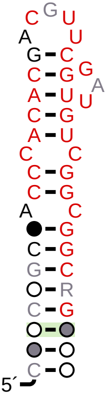

HMP2_829_1

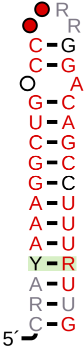

HMP2_8293_1

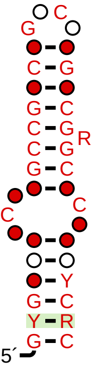

HMP2_8294_1

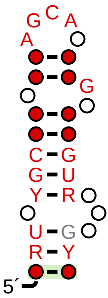

HMP2_83_1

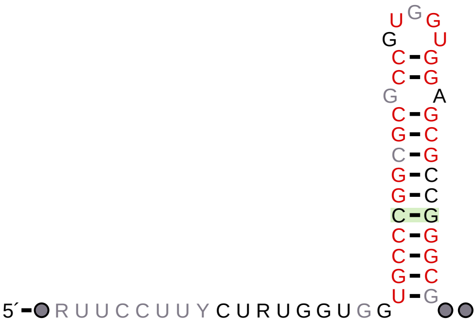

HMP2_8304_1

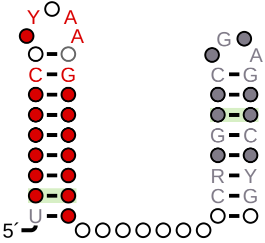

HMP2_8306_1

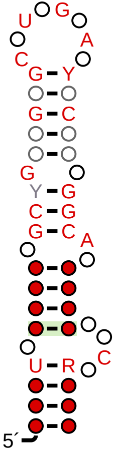

HMP2_8307_1

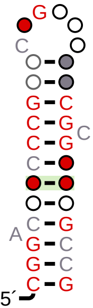

HMP2_8316_1

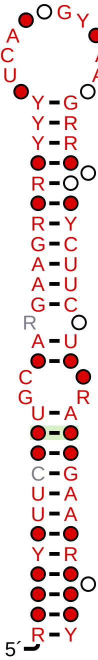

HMP2_8321_1

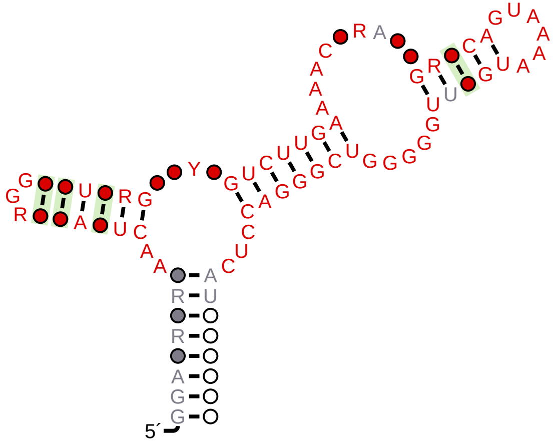

HMP2_8327_1

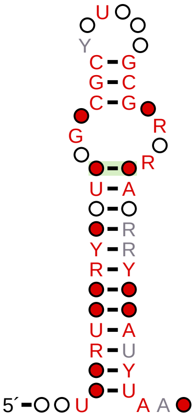

HMP2_833_1

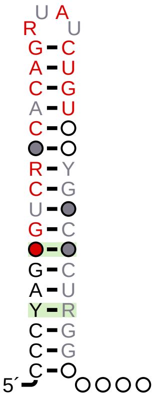

HMP2_8338_1

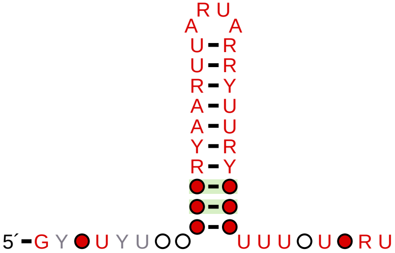

HMP2_834_1

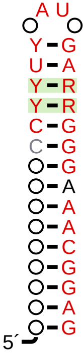

HMP2_8341_1

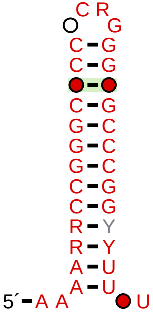

HMP2_8343_1

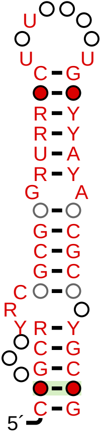

HMP2_8348_1

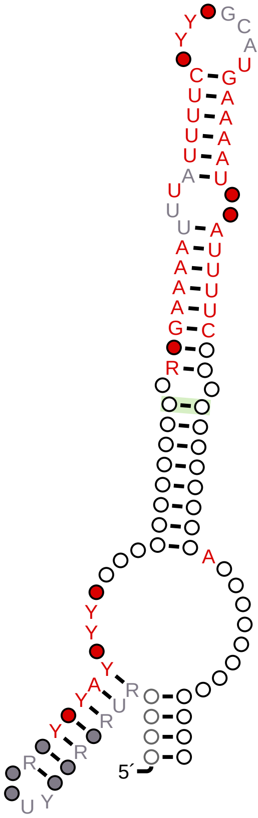

HMP2_8359_1

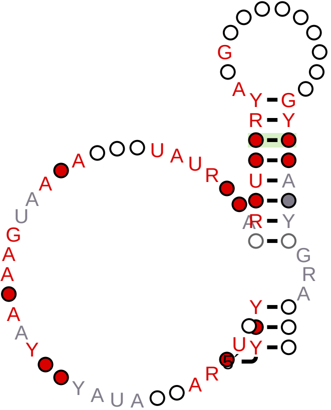

HMP2_8363_1

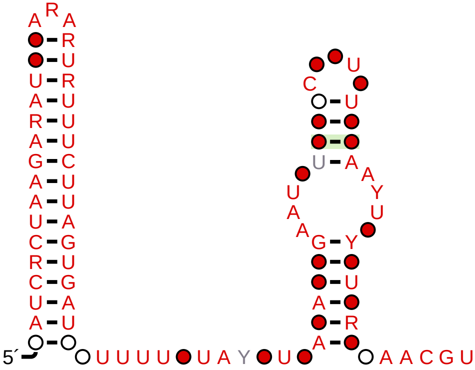

HMP2_8368_1

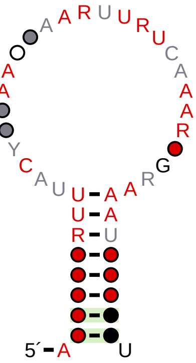

HMP2_8371_1

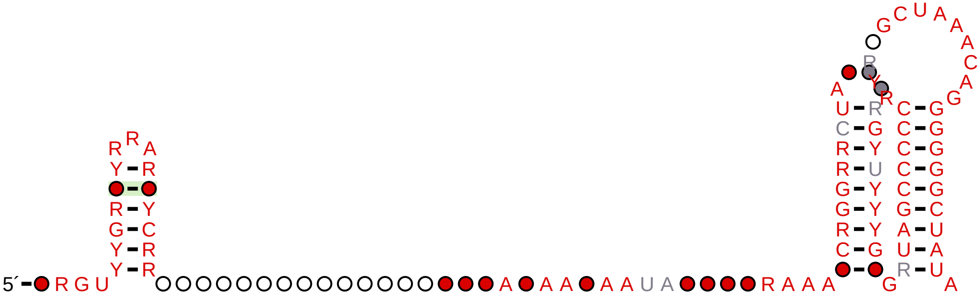

HMP2_8378_1

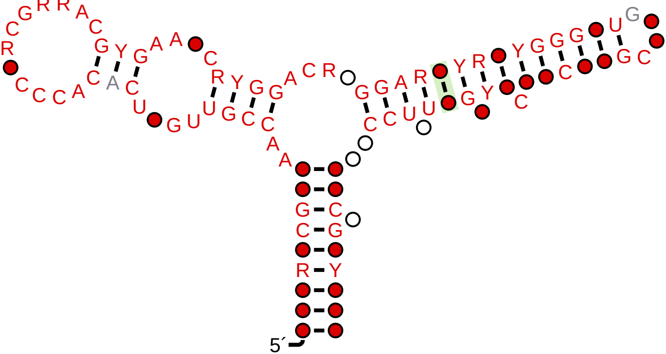

HMP2_8379_1

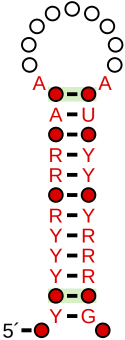

HMP2_838_1

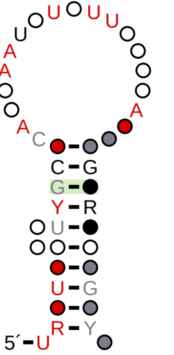

HMP2_8382_1

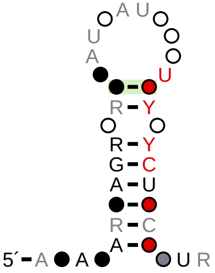

HMP2_8392_1

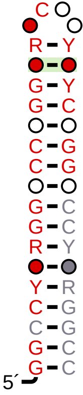

HMP2_8398_1

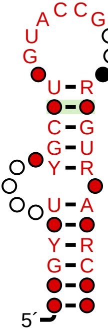

HMP2_840_1

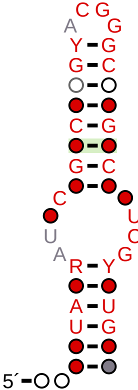

HMP2_8408_1

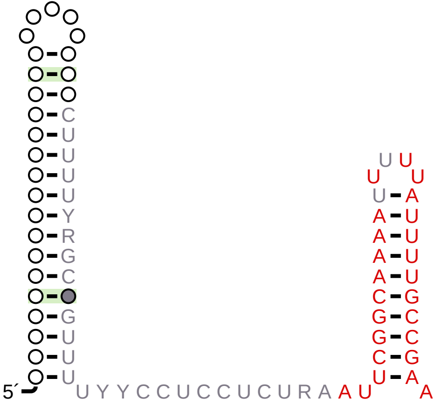

5´
C
U
U
U
U
Y
R
G
C
G
U
U
U
U Y Y C C U C C U C U R A A U
U U
U U
U A
A U
A U
A U
A U
C G
G C
G C
C G
U A
A

HMP2_841_1

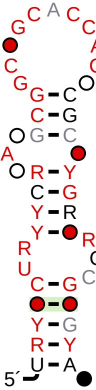

HMP2_8419_1

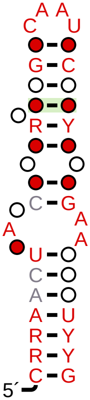

HMP2_843_1

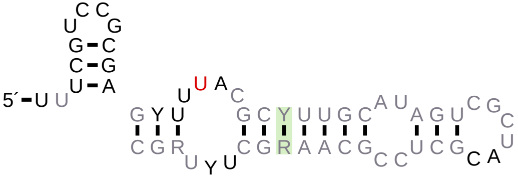

HMP2_8438_1

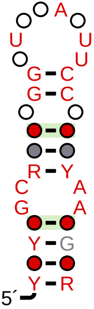

HMP2_8440_1

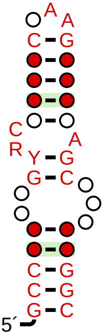

HMP2_846_1

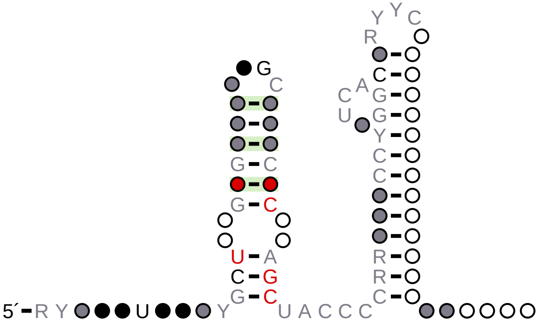

HMP2_8461_1

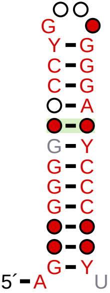

HMP2_847_1

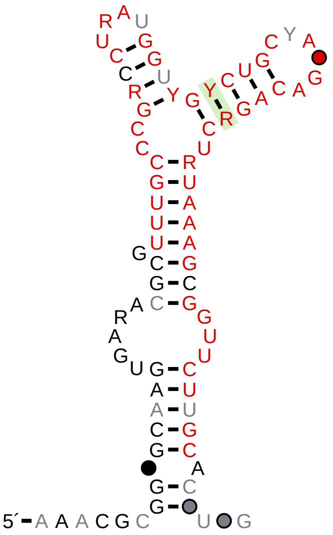

HMP2_8487_1

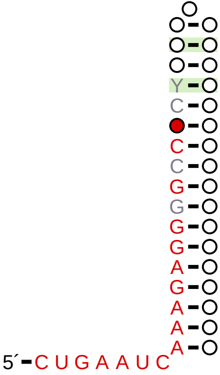

HMP2_8490_1

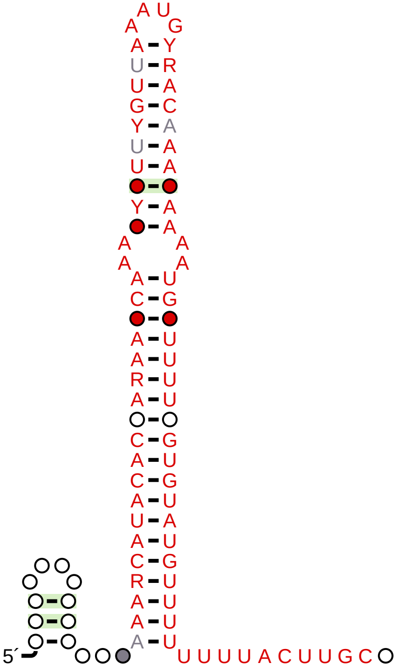

HMP2_8491_1

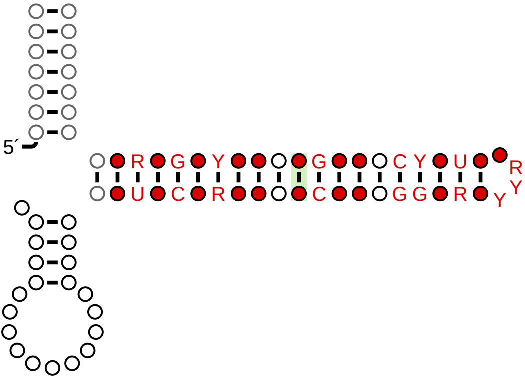

HMP2_8492_1

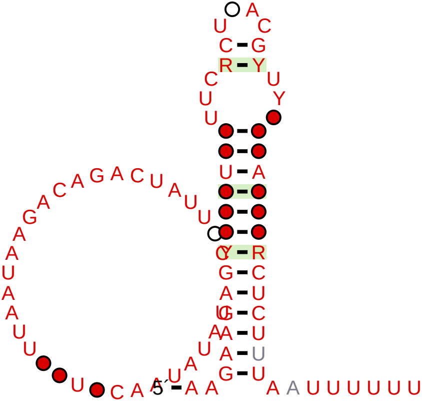

HMP2_8500_1

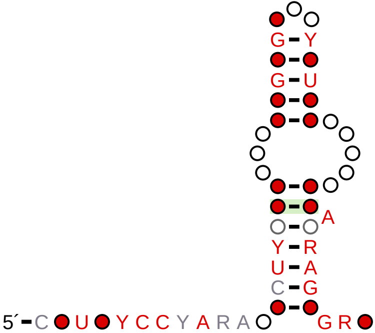

HMP2_8504_1

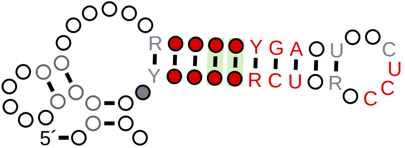

HMP2_8513_1

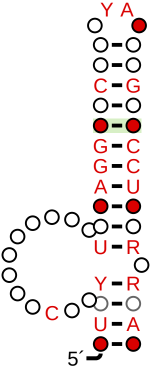

HMP2_8517_1

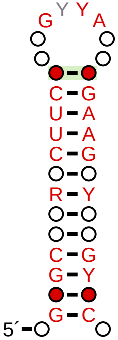

HMP2_8520_1

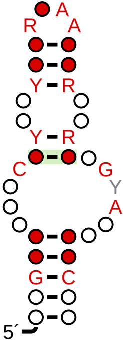

HMP2_8526_1

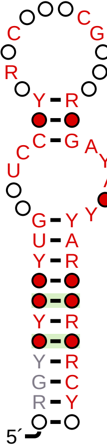

HMP2_8535_1

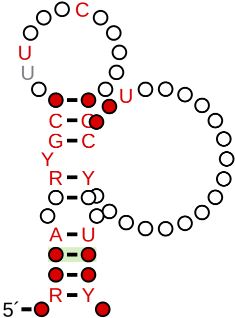

HMP2_8536_1

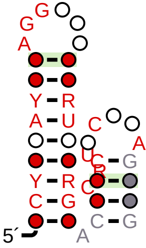

HMP2_8537_1

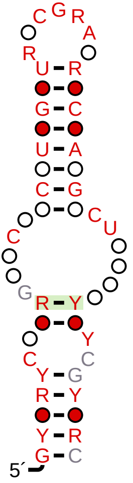

HMP2_8541_1

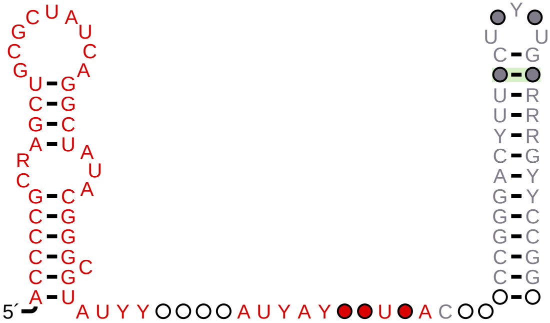

HMP2_8543_1

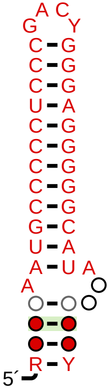

HMP2_8544_1

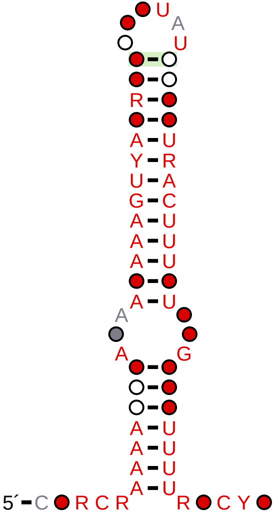

HMP2_8545_1

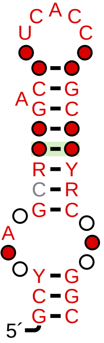

HMP2_8549_1

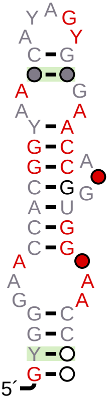

HMP2_856_1

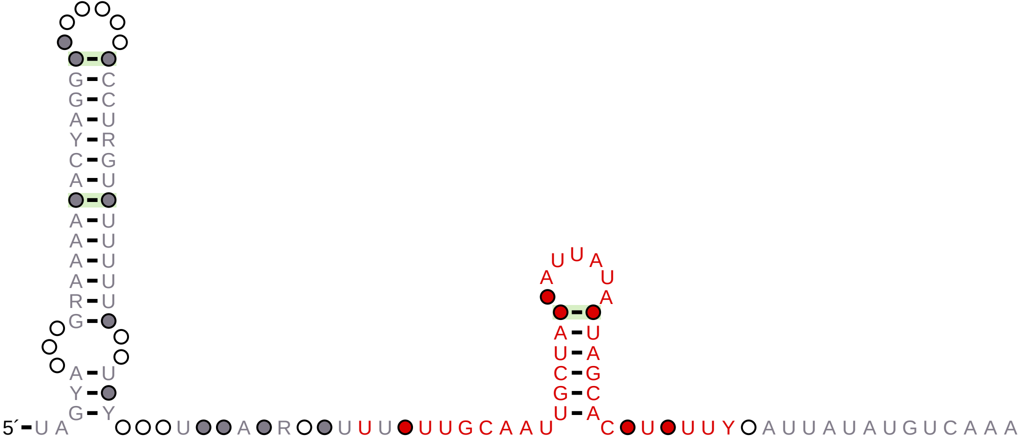

HMP2_8574_1

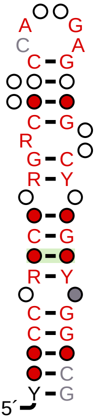

HMP2_8575_1

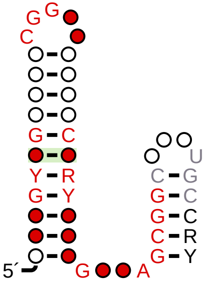

HMP2_858_1

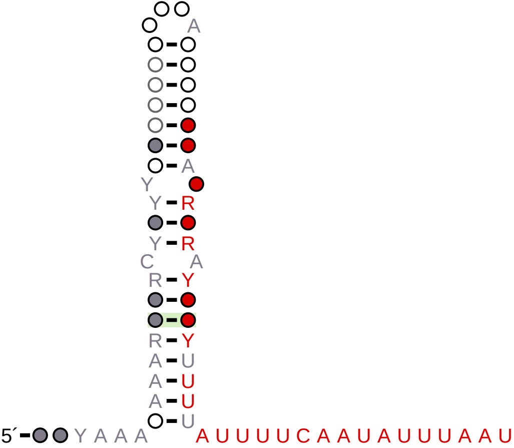

HMP2_859_1

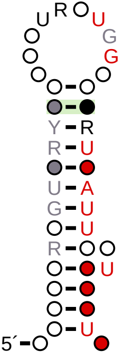

HMP2_8591_1

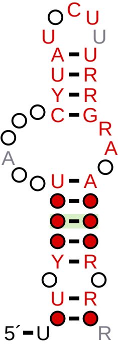

HMP2_8596_1

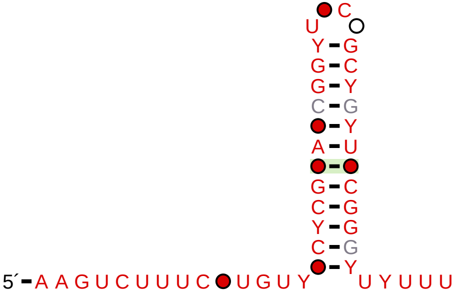

HMP2_8597_1

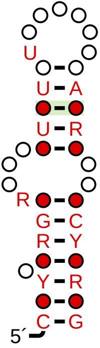

HMP2_86_1

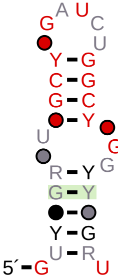

HMP2_8609_1

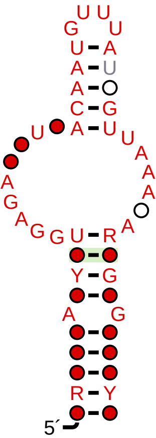

HMP2_8613_1

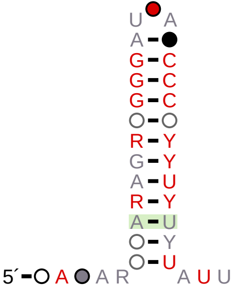

HMP2_8616_1

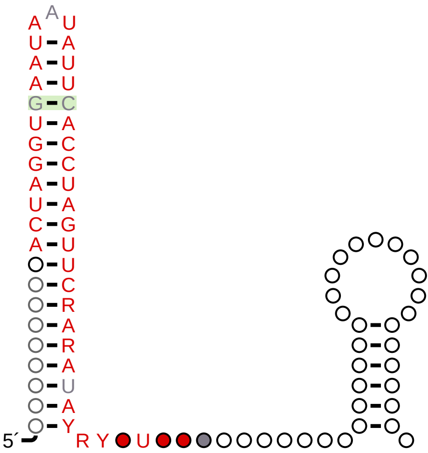

HMP2_8617_1

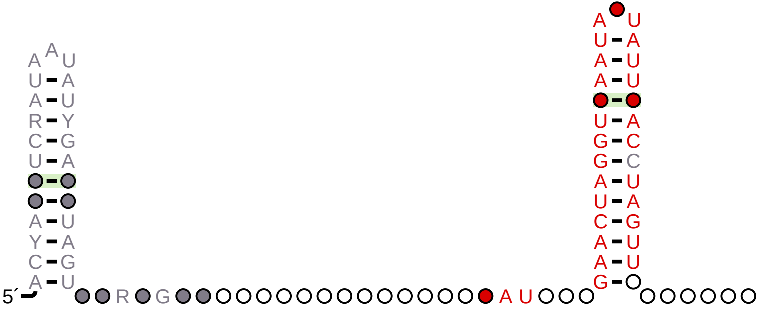

HMP2_8619_1

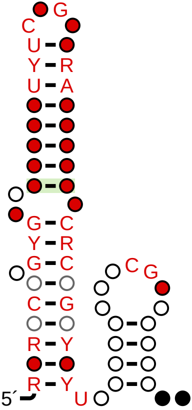

HMP2_8622_1

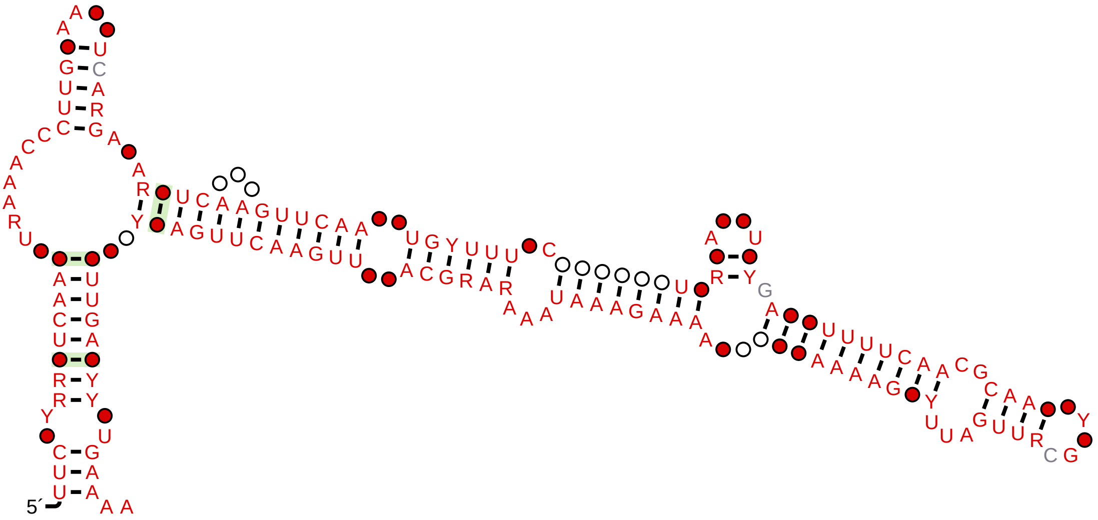

HMP2_8623_1

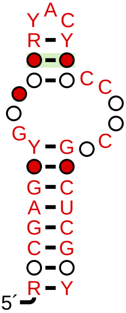

HMP2_8624_1

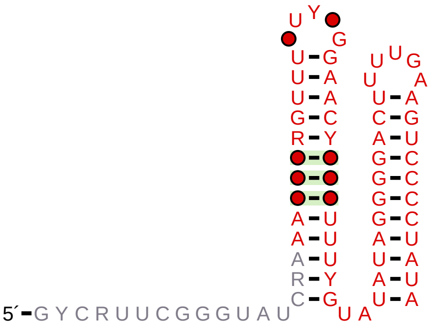
5´-GYCRUUCGGGUAU
U Y G
UUUGR AAR C
GAACY UUUY G
UA
UCAGGGGAUAU UUG A
UGUCCCCUAUA

HMP2_863_1

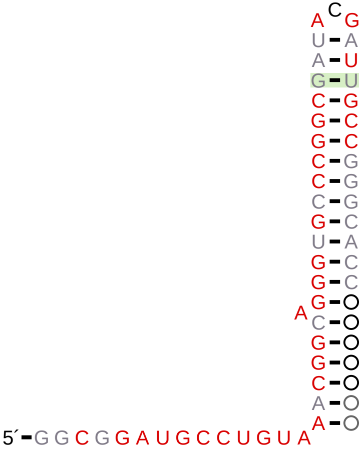

HMP2_8635_1

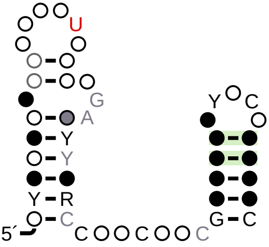

HMP2_8637_1

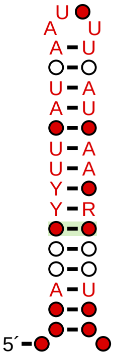

HMP2_8638_1

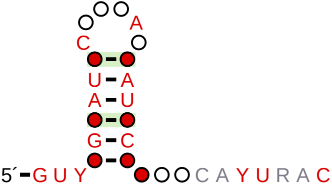

HMP2_8644_1

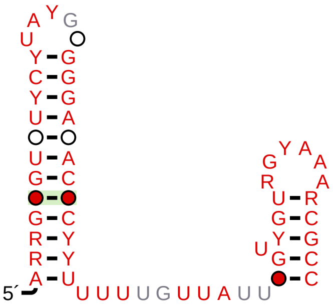

HMP2_8647_1

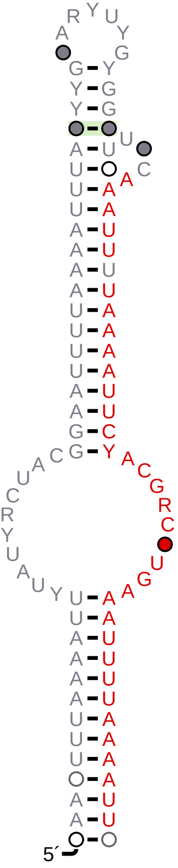

HMP2_8663_1

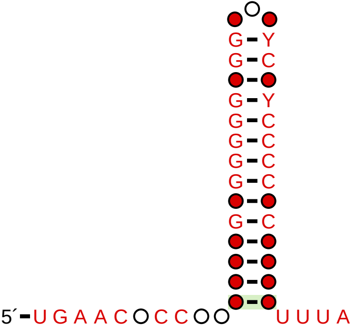

HMP2_8670_1

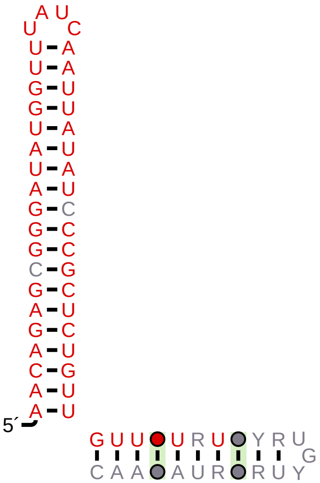

HMP2_8678_1

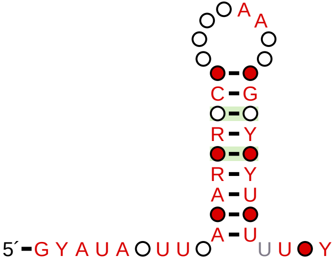

HMP2_8679_1

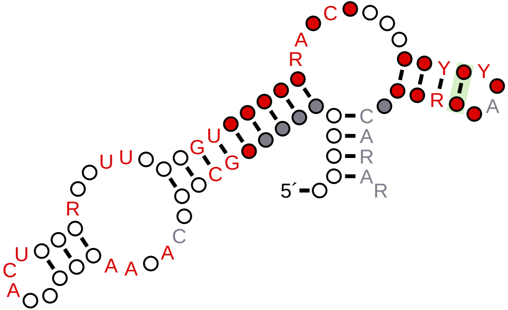

HMP2_8681_1

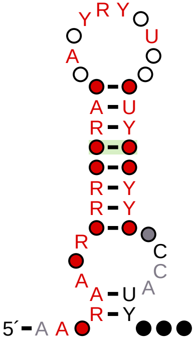

HMP2_8683_1

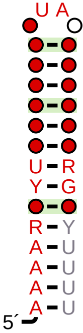

HMP2_8686_1

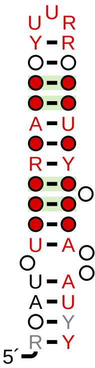

HMP2_8687_1

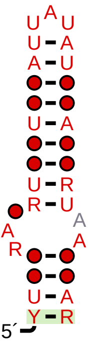

HMP2_8694_1

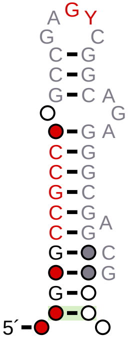

HMP2_8705_1

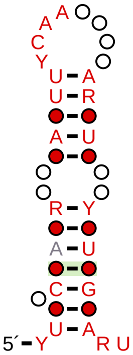

HMP2_8706_1

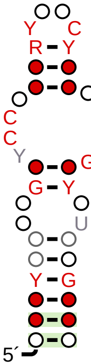

HMP2_8708_1

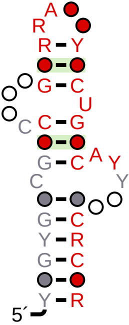

HMP2_8718_1

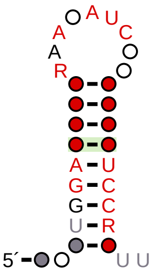

HMP2_872_1

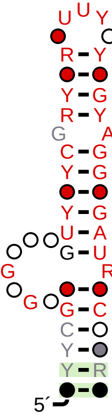

HMP2_8728_1

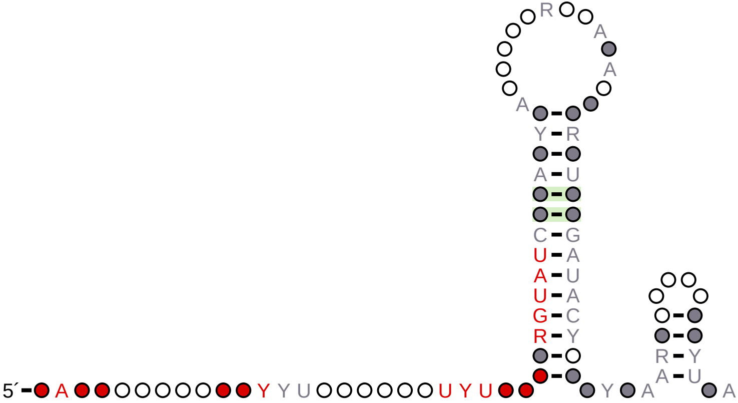

HMP2_873_1

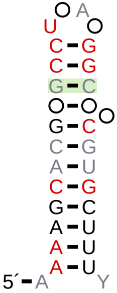

HMP2_8730_1

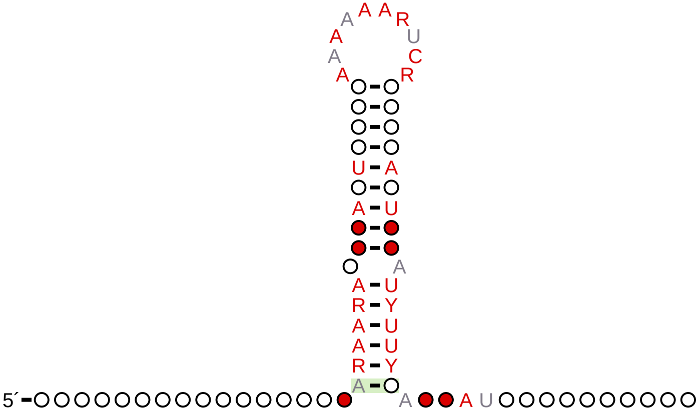

HMP2_8733_1

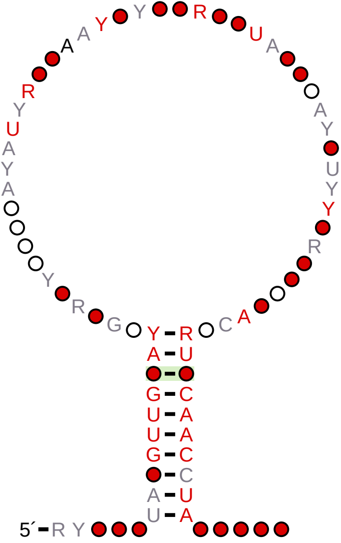

HMP2_8734_1

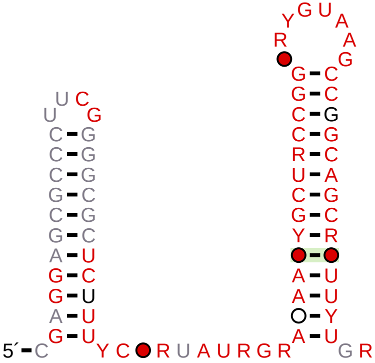

HMP2_8744_1

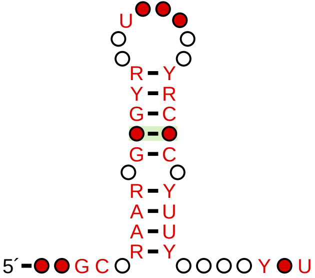

HMP2_8753_1

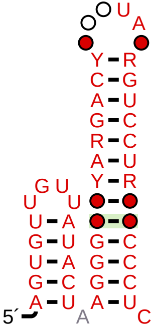

HMP2_8759_1

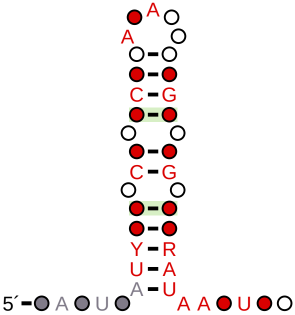

HMP2_8765_1

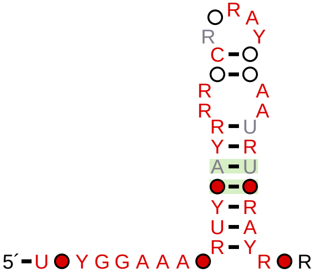

HMP2_8772_1

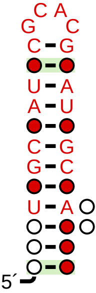

HMP2_8782_1

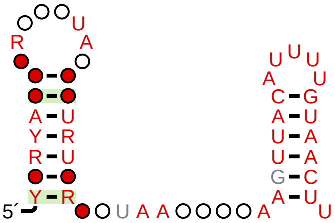

HMP2_8783_1

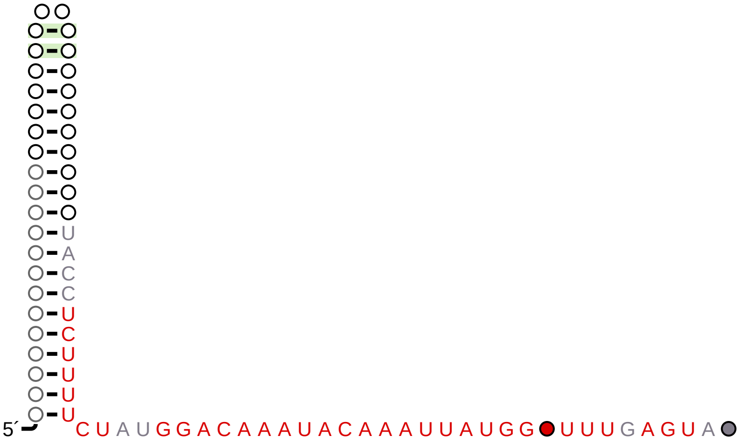

HMP2_8800_1

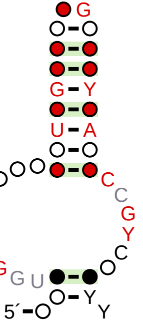

HMP2_8808_1

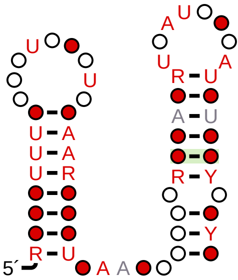

HMP2_8809_1

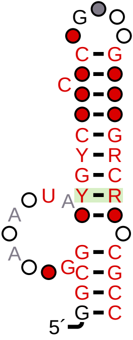

HMP2_881_1

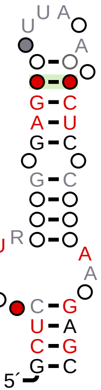

HMP2_8812_1

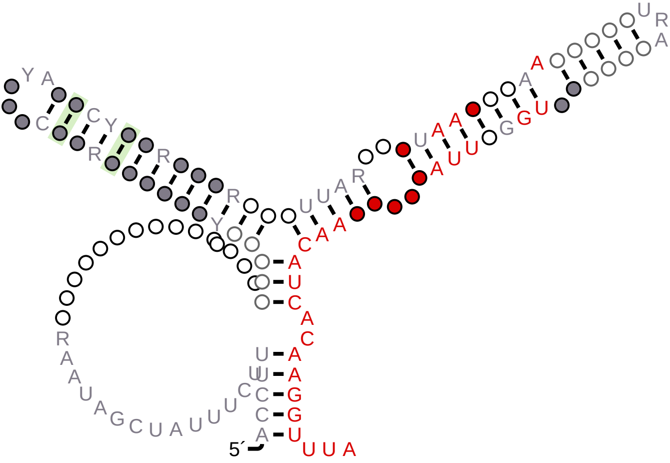

HMP2_8819_1

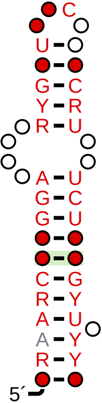

HMP2_8823_1

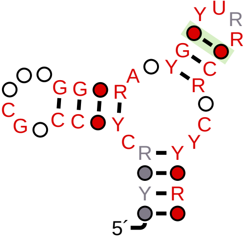

HMP2_8828_1

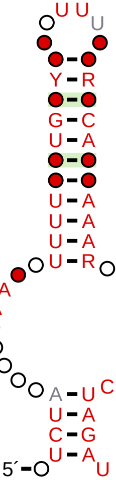

HMP2_883_1

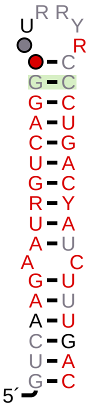

HMP2_8830_1

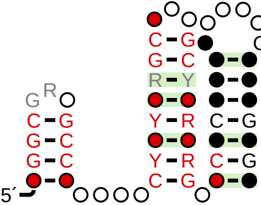

HMP2_8838_1

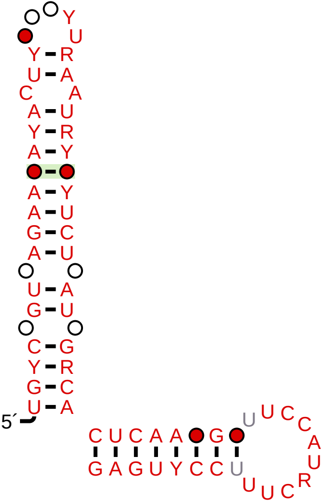

Y
U
Y R
U A
C A
A U
Y R
A Y
A Y
A U
G C
A U
U A
G U
C G
Y R
G C
U A
5´
C U C A A G
G A G U Y C C U
U U C C A U R C U U

HMP2_884_1

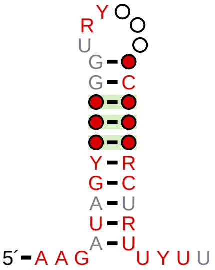

HMP2_8844_1

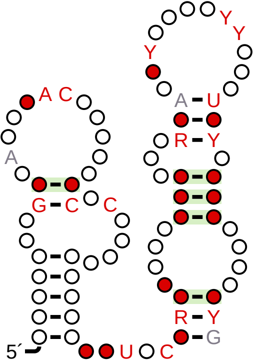

HMP2_8852_1

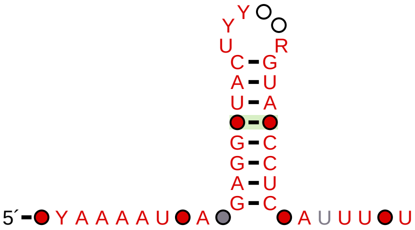

HMP2_8855_1

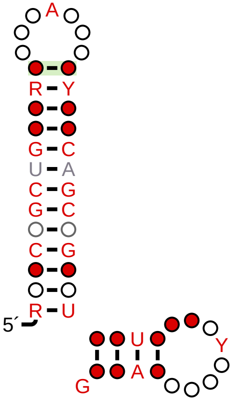

HMP2_8856_1

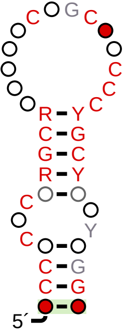

HMP2_8858_1

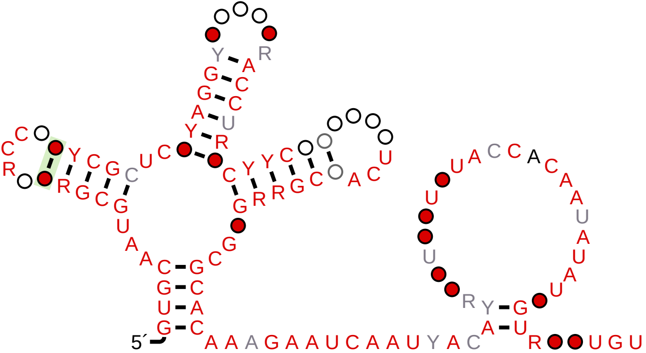

HMP2_8861_1

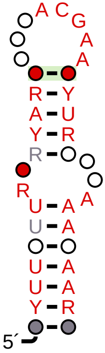

HMP2_8862_1

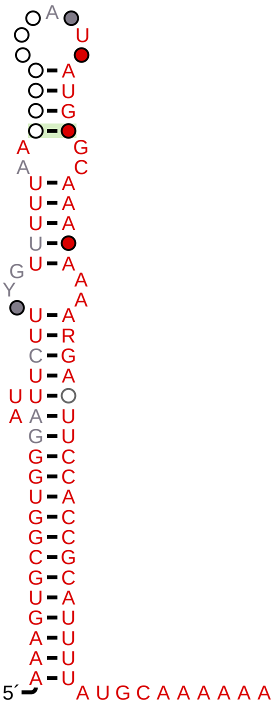

HMP2_8869_1

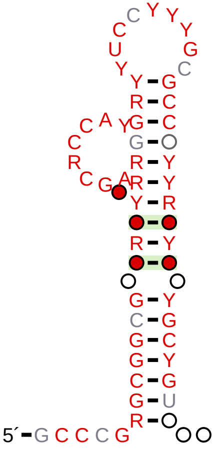

HMP2_8872_1

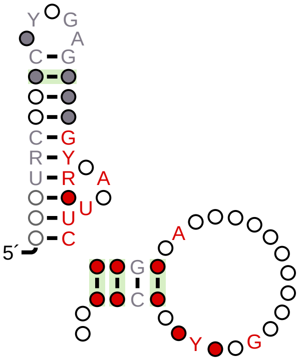

HMP2_8882_1

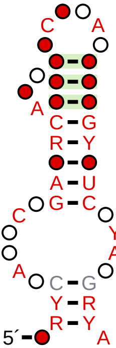

HMP2_8883_1

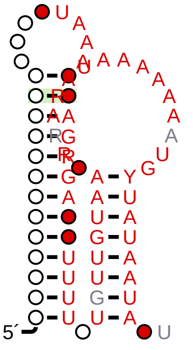

HMP2_889_1

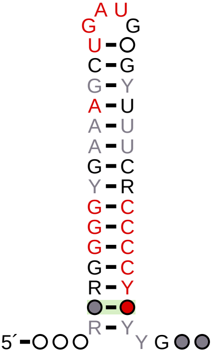

HMP2_8913_1

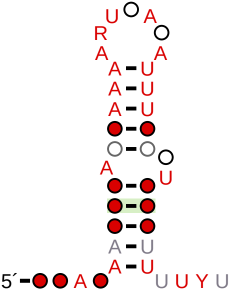

HMP2_892_1

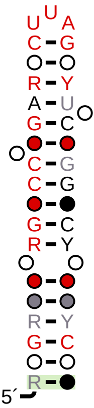

HMP2_8921_1

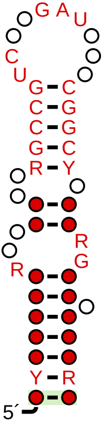

HMP2_8931_1

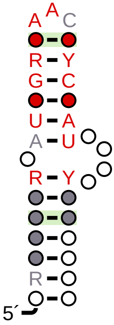

HMP2_8935_1

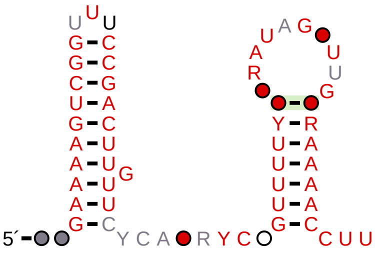

5´
UUU
G C
G C
C G
U A
G C
A U
A U
G
A U
A U
G C
Y C A R Y C
U A G
A U
R U
G
Y R
U A
U A
U A
U A
G C
C U U

HMP2_8936_1

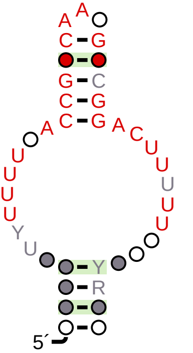

HMP2_8937_1

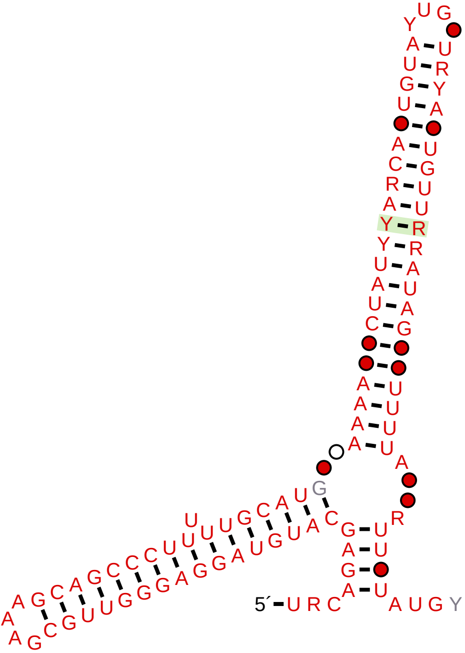

HMP2_8946_1

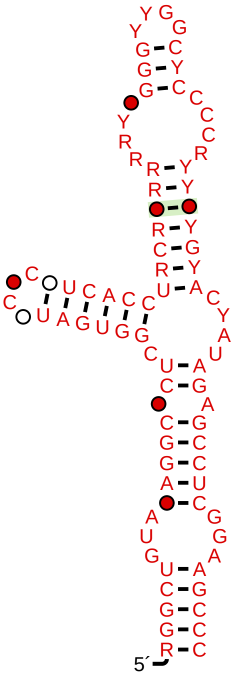

HMP2_8951_1

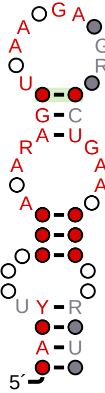

HMP2_8954_1

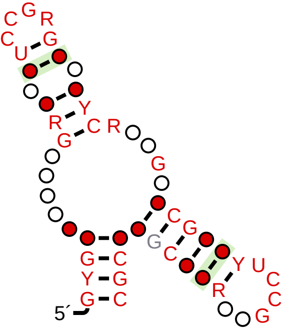

HMP2_8957_1

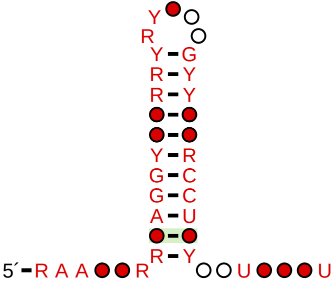

HMP2_8959_1

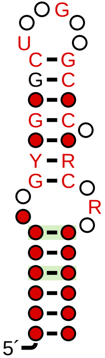

HMP2_8962_1

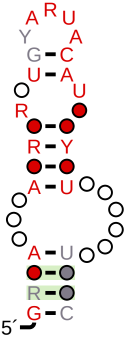

HMP2_8966_1

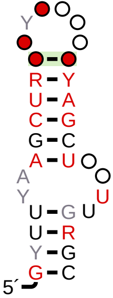

HMP2_897_1

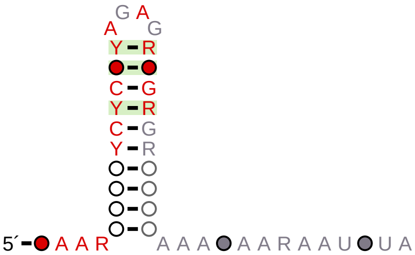

HMP2_8972_1

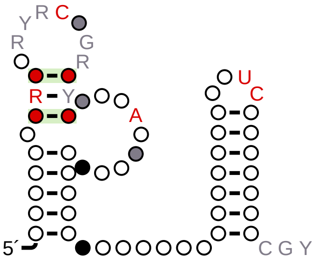

HMP2_8981_1

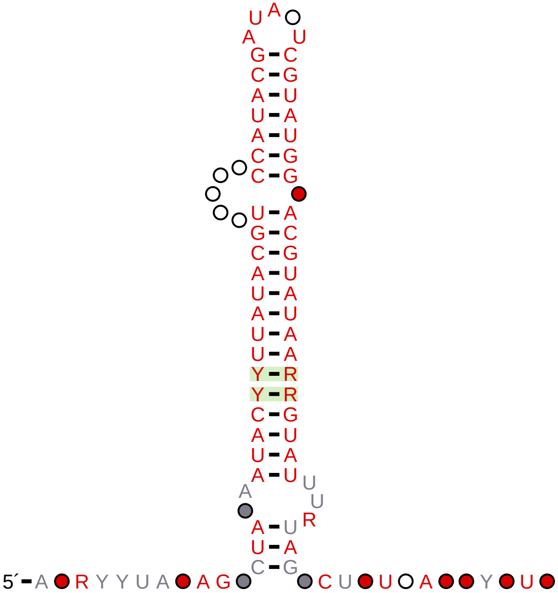

HMP2_8985_1

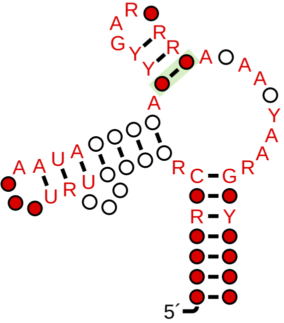

HMP2_8988_1

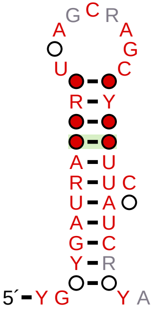

HMP2_8989_1

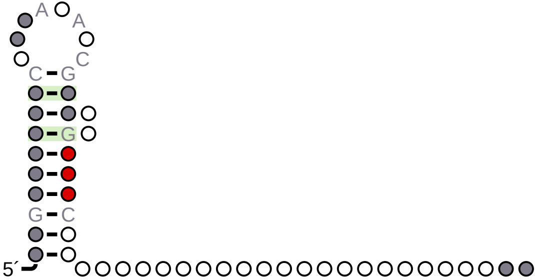

HMP2_9_1

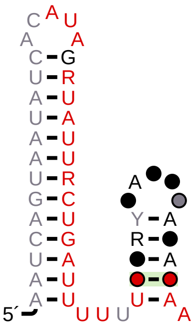

HMP2_9013_1

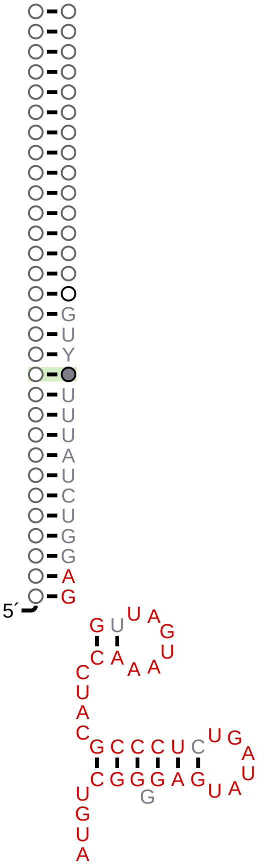

HMP2_9017_1

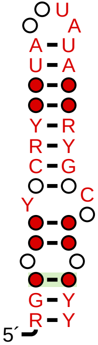

HMP2_9027_1

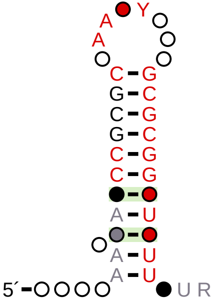

HMP2_9048_1

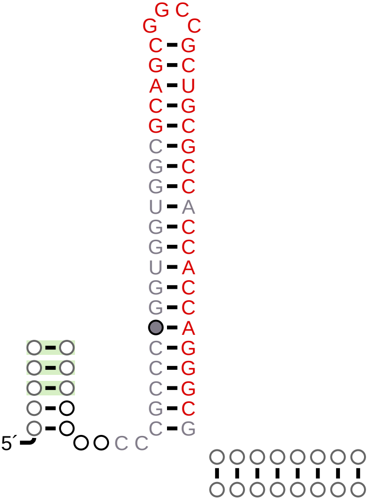

HMP2_905_1

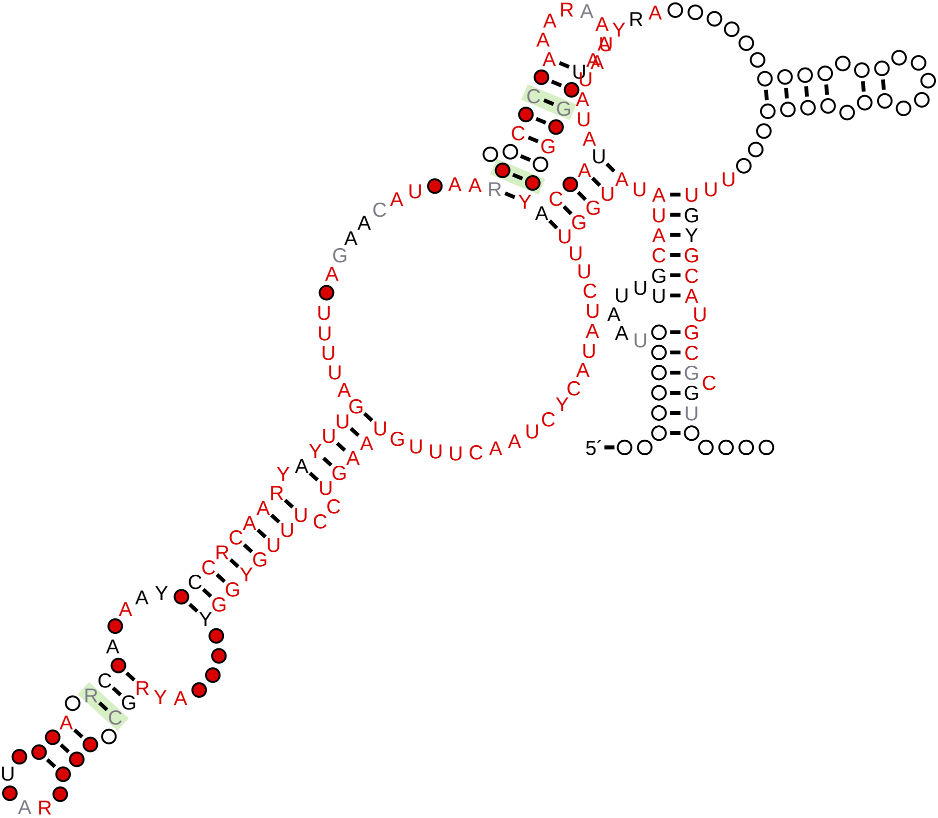

HMP2_9059_1

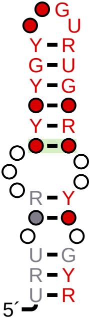

HMP2_9062_1

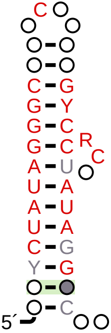

HMP2_9064_1

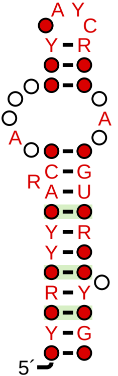

HMP2_9066_1

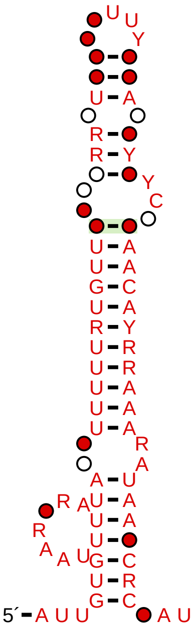

5´
A U U G U G U U A U U G U R U U U U U R G U U A U A U U U
C R C U A A U A A R A A A R R Y A C A A Y C Y R A Y U U
A U
R A R A A U

HMP2_9075_1

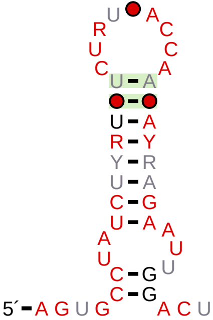

HMP2_9080_1

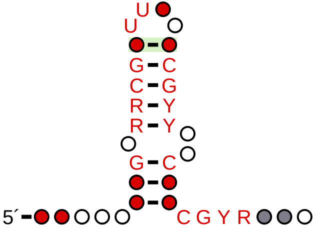

HMP2_9100_1

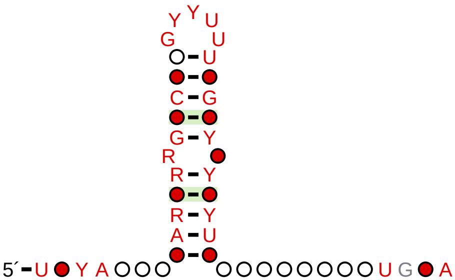

HMP2_9101_1

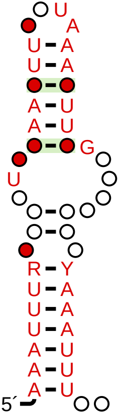

HMP2_9112_1

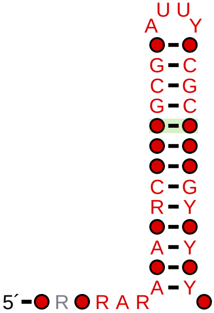

HMP2_9118_1

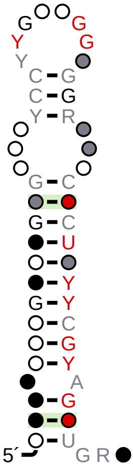

HMP2_9119_1

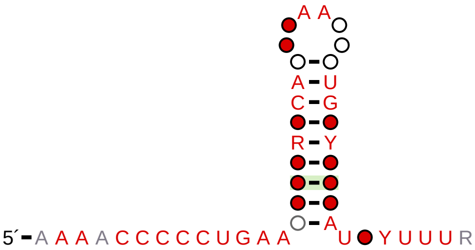

HMP2_9123_1

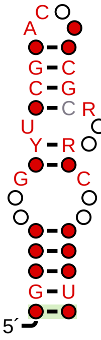

HMP2_9132_1

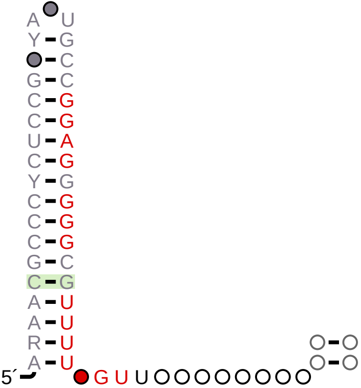

HMP2_9134_1

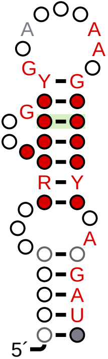

HMP2_9138_1

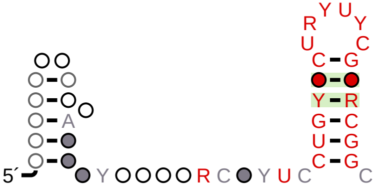

HMP2_9145_1

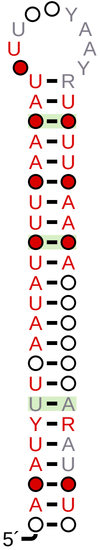

HMP2_9153_1

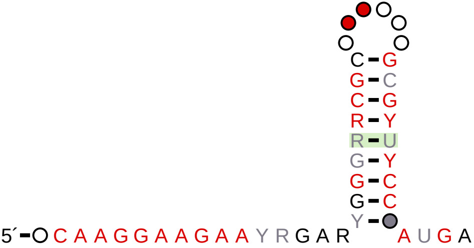

HMP2_9161_1

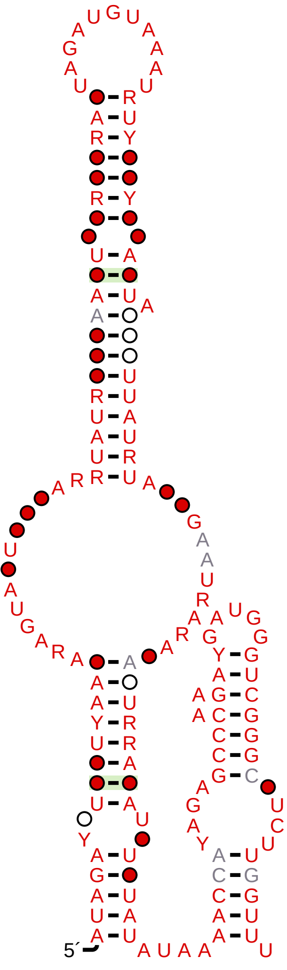

HMP2_917_1

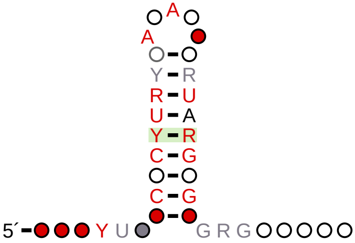

HMP2_9170_1

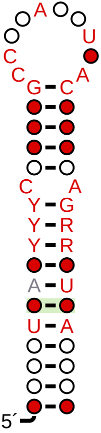

HMP2_9171_1

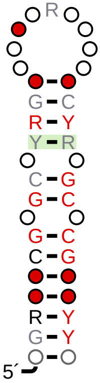

HMP2_9173_1

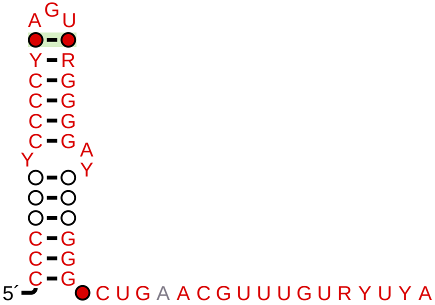

HMP2_9176_1

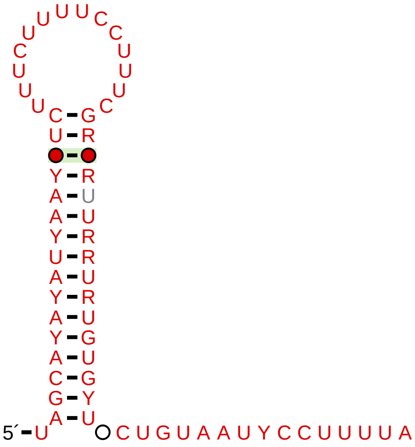

HMP2_9180_1

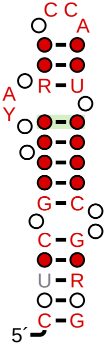

HMP2_9184_1

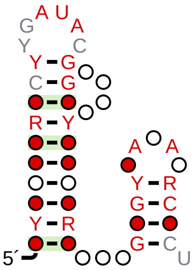

HMP2_9191_1

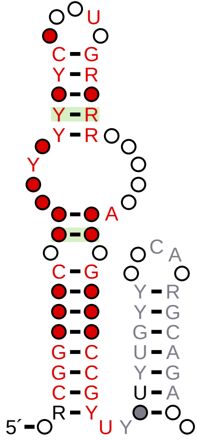

HMP2_9193_1

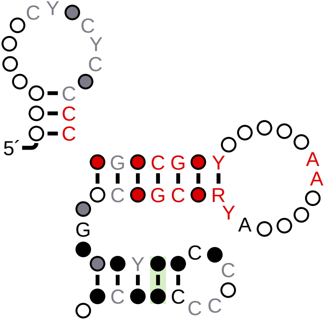

HMP2_9198_1

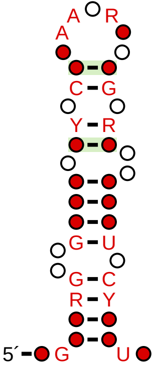

HMP2_9203_1

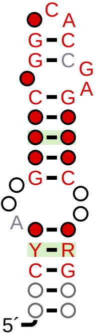

HMP2_9226_1

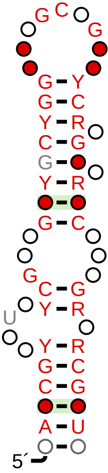

HMP2_9228_1

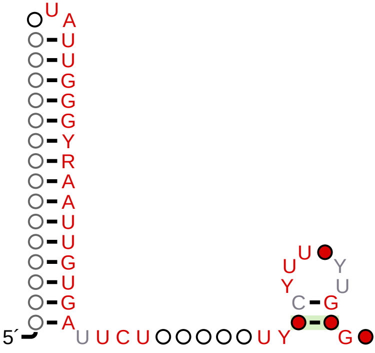

HMP2_9235_1

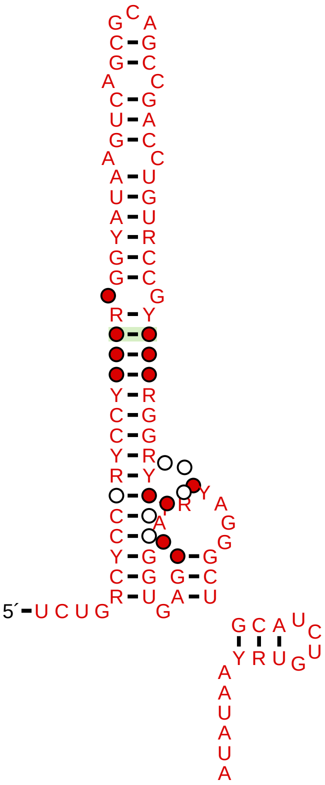

HMP2_924_1

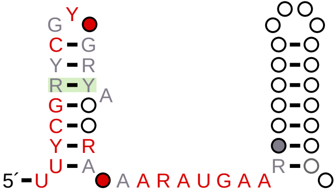

HMP2_9242_1

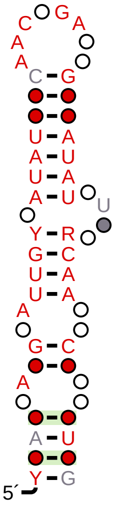

HMP2_9246_1

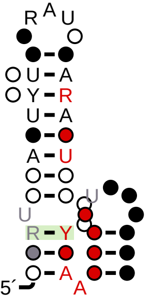

HMP2_9251_1

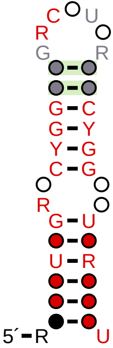

HMP2_9253_1

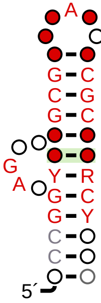

HMP2_9256_1

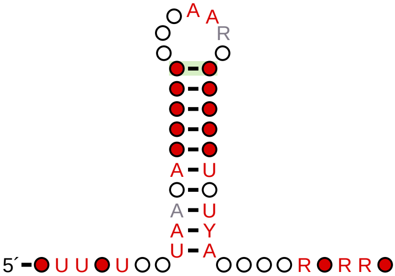

HMP2_9258_1

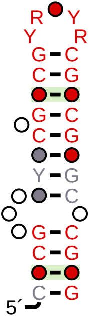

HMP2_9260_1

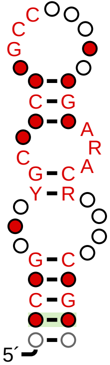

HMP2_9262_1

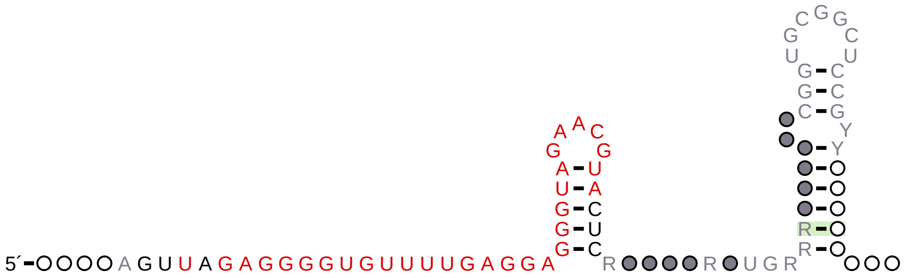

HMP2_9269_1

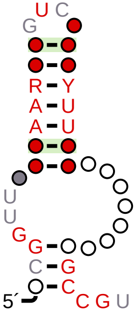

HMP2_9274_1

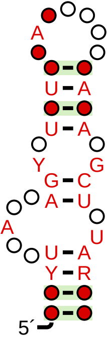

HMP2_9275_1

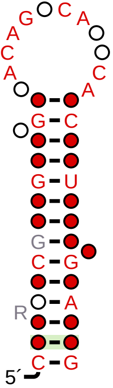

HMP2_928_1

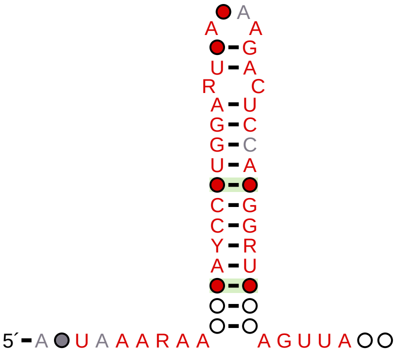

HMP2_9282_1

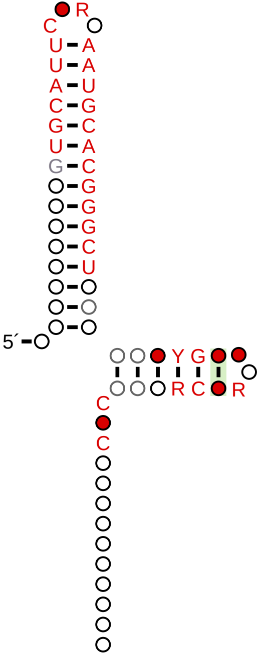

HMP2_9284_1

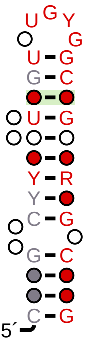

HMP2_929_1

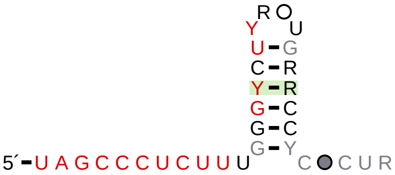

HMP2_9294_1

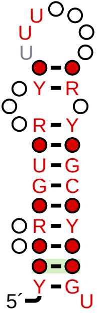

HMP2_9297_1

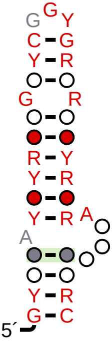

HMP2_9306_1

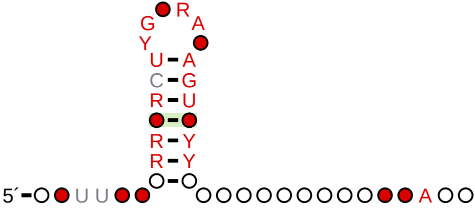

HMP2_9308_1

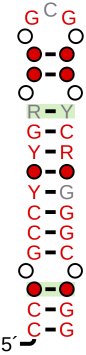

HMP2_931_1

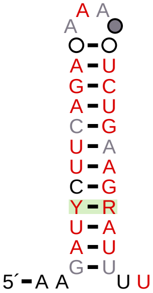

HMP2_9310_1

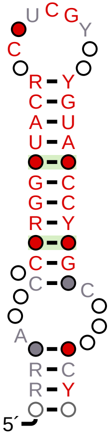

HMP2_9314_1

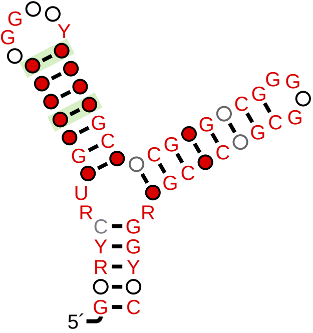

HMP2_9315_1

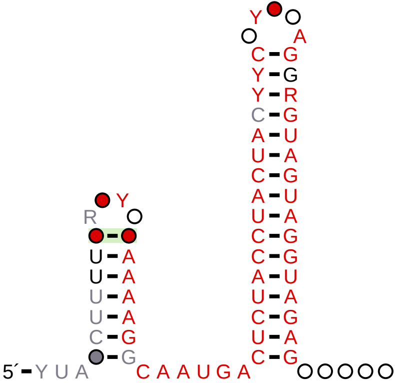

HMP2_9316_1

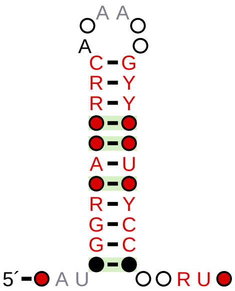

HMP2_932_1

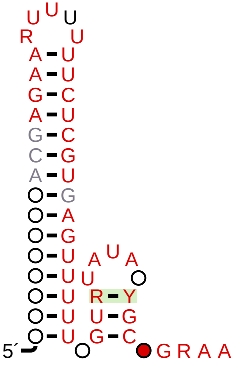

HMP2_9320_1

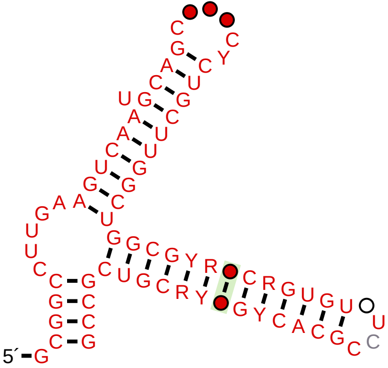

HMP2_9324_1

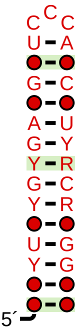

HMP2_9328_1

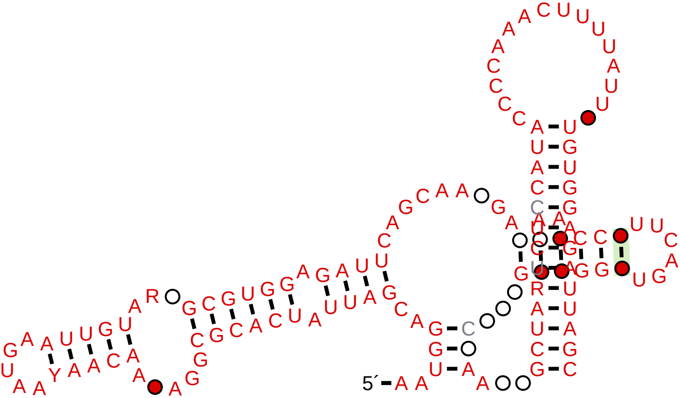

HMP2_9330_1

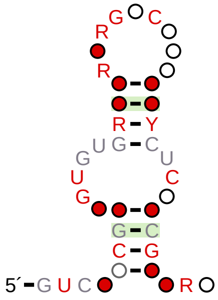

HMP2_9335_1

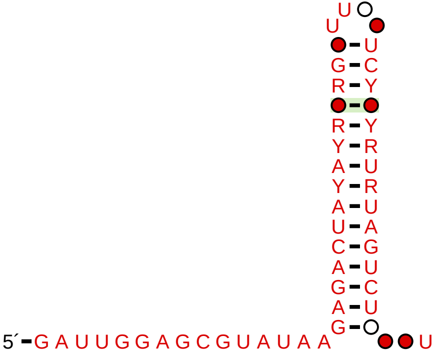

HMP2_934_1

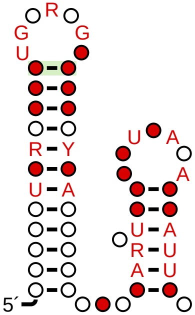

HMP2_9342_1

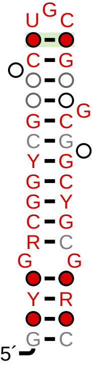

HMP2_9343_1

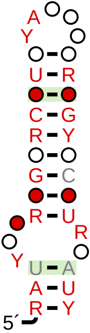

HMP2_9345_1

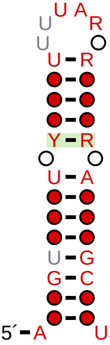

HMP2_9348_1

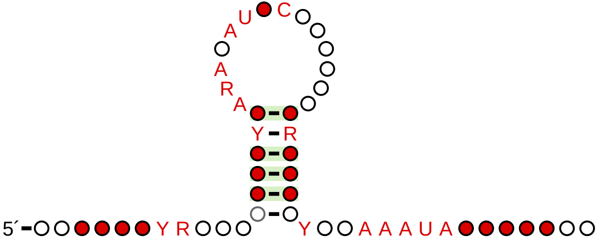

HMP2_9349_1

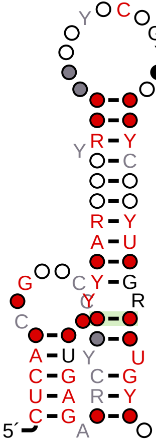

HMP2_9356_1

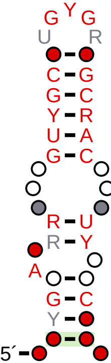

HMP2_9363_1

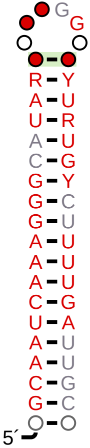

HMP2_9364_1

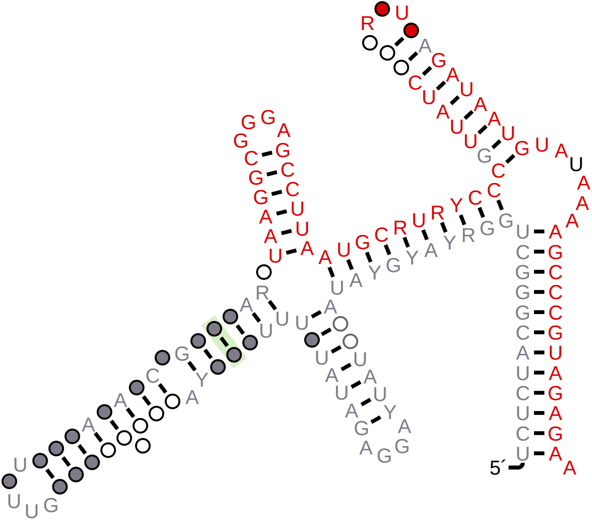

HMP2_9367_1

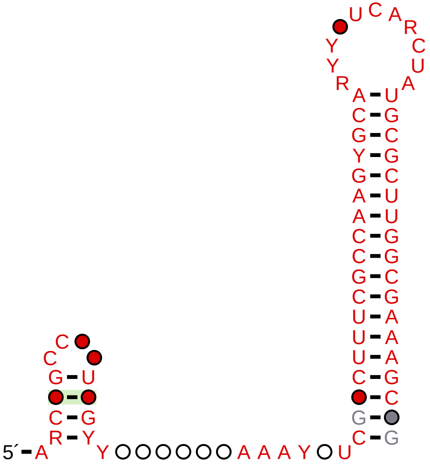

HMP2_9368_1

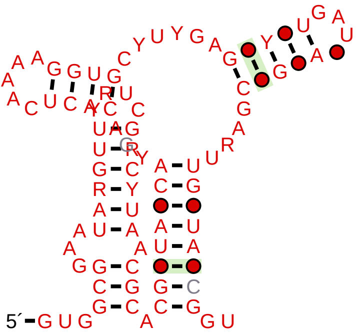

HMP2_938_1

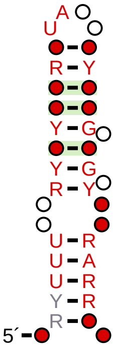

HMP2_9380_1

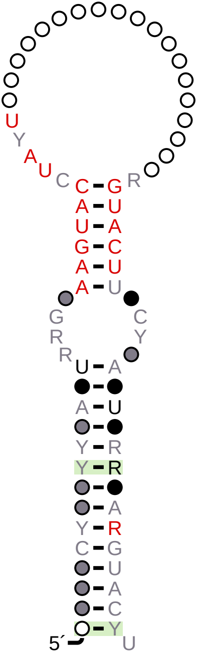

HMP2_9386_1

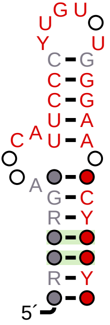

HMP2_9393_1

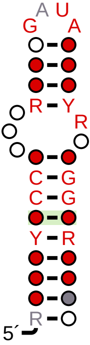

HMP2_9402_1

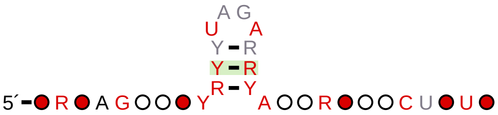

HMP2_9407_1

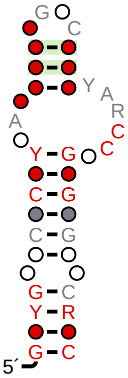

HMP2_9415_1

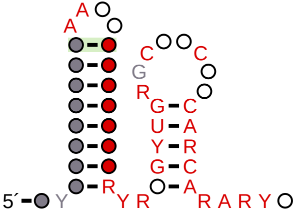

HMP2_9419_1

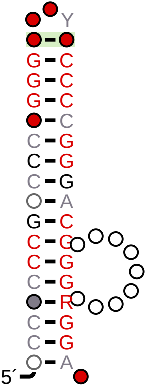

HMP2_942_1

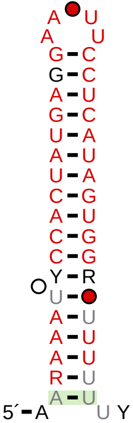

HMP2_9436_1

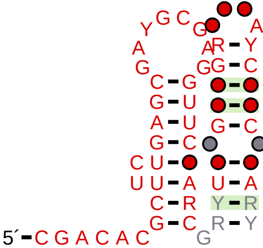

HMP2_9438_1

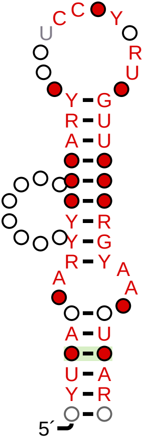

HMP2_9439_1

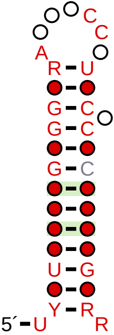

HMP2_9448_1

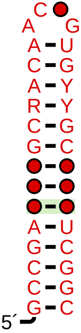

HMP2_9454_1

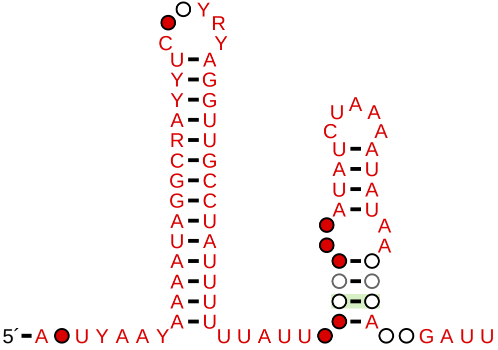

HMP2_9455_1

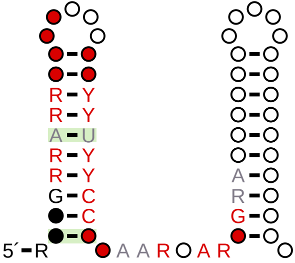

HMP2_9456_1

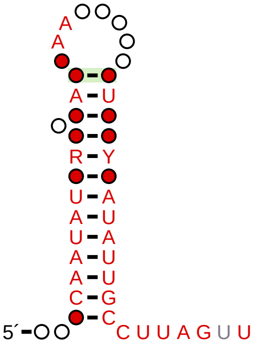

HMP2_9461_1

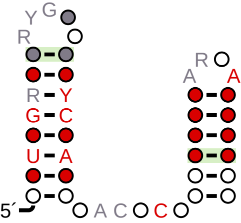

HMP2_9462_1

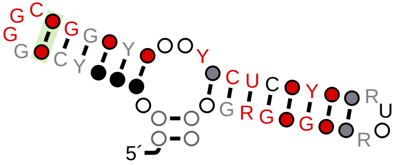

HMP2_9466_1

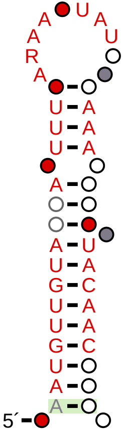

HMP2_9473_1

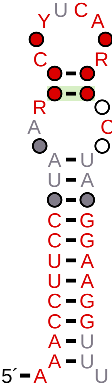

HMP2_9477_1

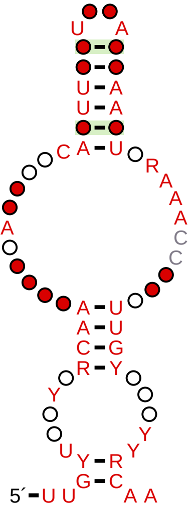

HMP2_9480_1

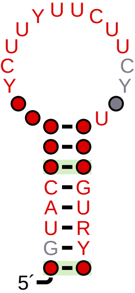

HMP2_9481_1

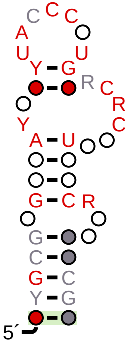

HMP2_9487_1

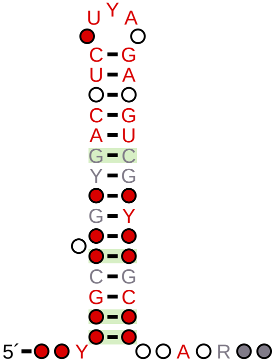

HMP2_9488_1

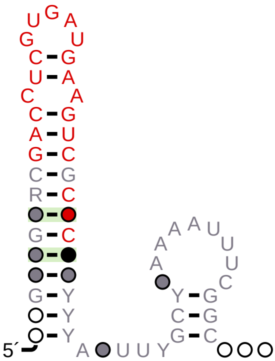

HMP2_9489_1

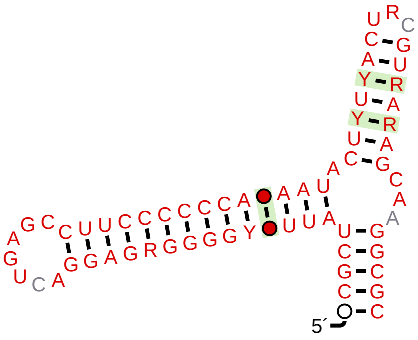

HMP2_949_1

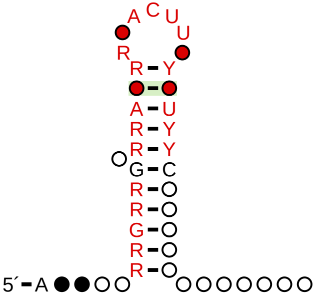

HMP2_9492_1

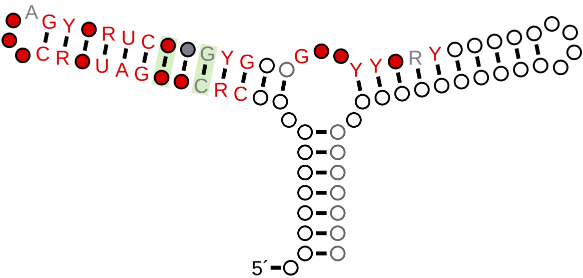

HMP2_95_1

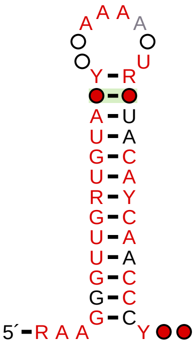

HMP2_9500_1

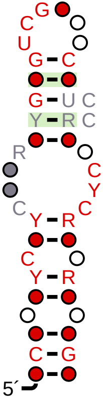

HMP2_9507_1

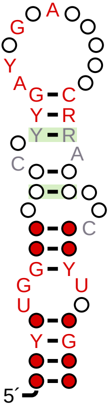

HMP2_9512_1

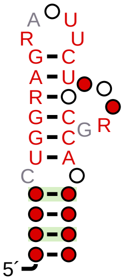

HMP2_9519_1

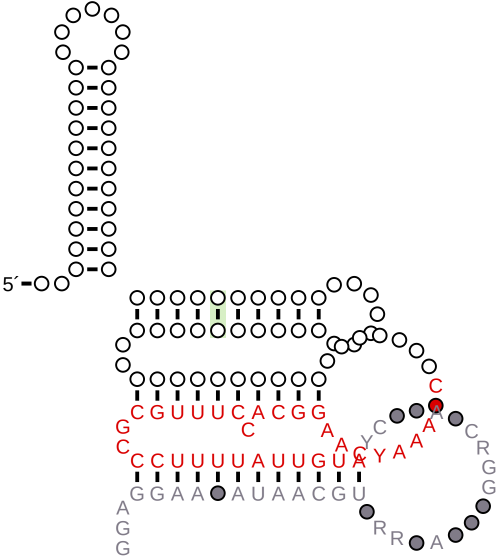

5´
C G U U U C A C G G
C
G
C
A
A
C
C
Y
A
A
A
C
C C U U U U A U U G U A
G G A A A U A A C G U
A
G
G
R
R
A
C
R
G
G

HMP2_9520_1

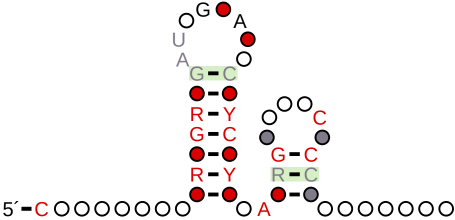

HMP2_9531_1

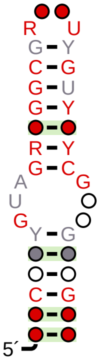

HMP2_9535_1

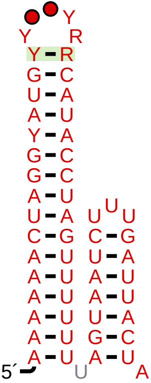

HMP2_9536_1

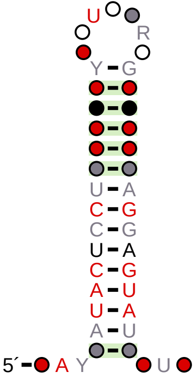

HMP2_9540_1

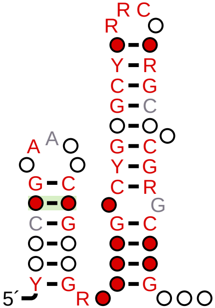

HMP2_9549_1

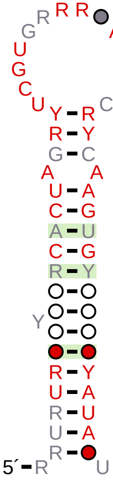

HMP2_9555_1

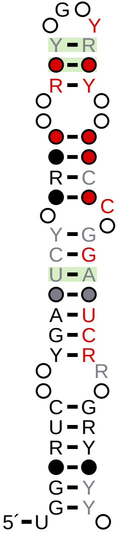

HMP2_9559_1

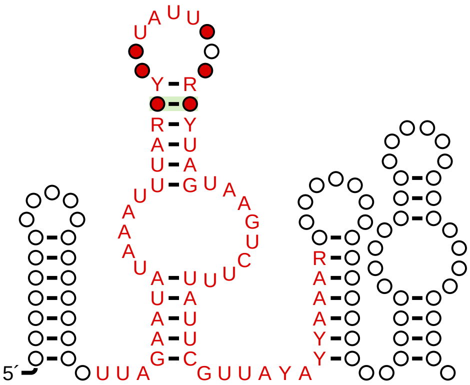

HMP2_9561_1

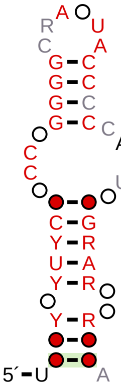

HMP2_957_1

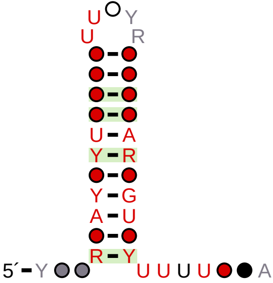

HMP2_9573_1

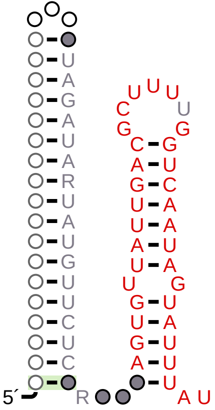

HMP2_9579_1

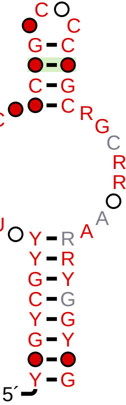

HMP2_9582_1

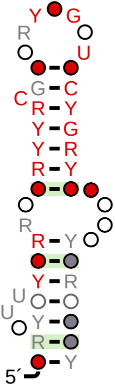

HMP2_9593_1

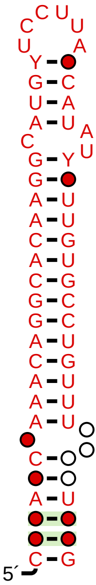

HMP2_9594_1

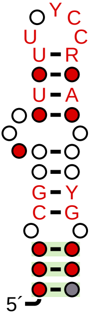

HMP2_9605_1

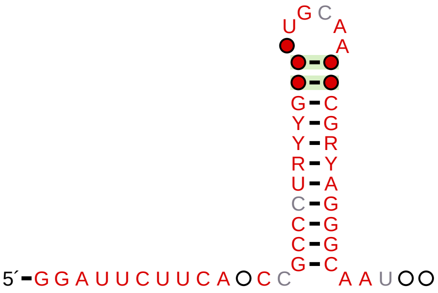

HMP2_9606_1

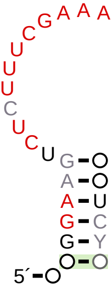

HMP2_9607_1

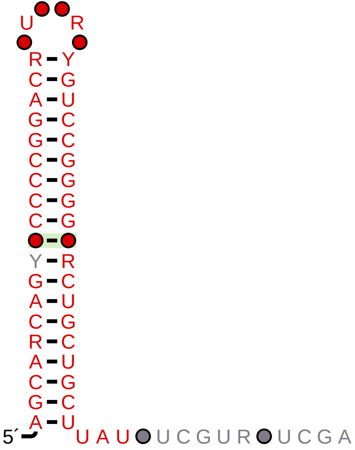

5´
U
R
R Y
C G
A U
G C
G C
C G
C G
C G
C G
Y R
G C
A U
C G
R C
A U
C G
G C
A U
U A U
U C G U R
U C G A

HMP2_9609_1

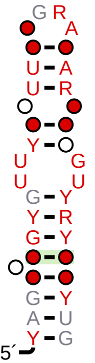

HMP2_9610_1

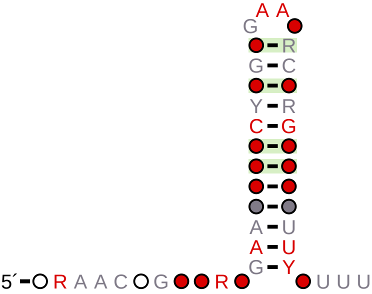

HMP2_9614_1

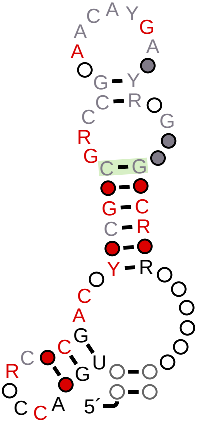

HMP2_9618_1

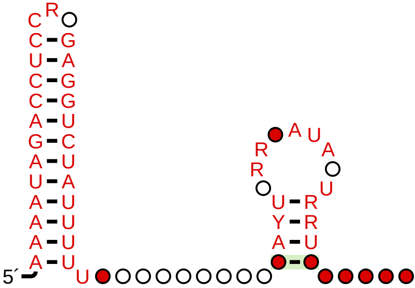

HMP2_962_1

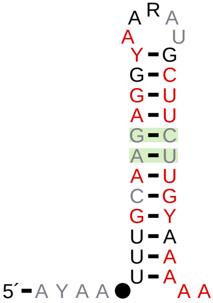

HMP2_9620_1

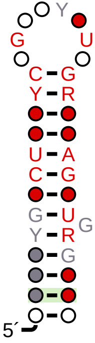

HMP2_9622_1

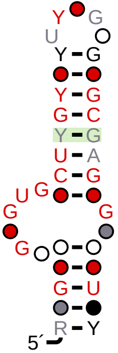

HMP2_9630_1

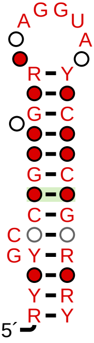

HMP2_9638_1

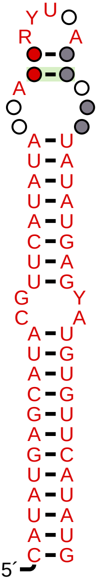

HMP2_9643_1

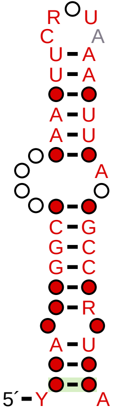

HMP2_965_1

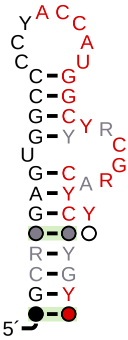

HMP2_9652_1

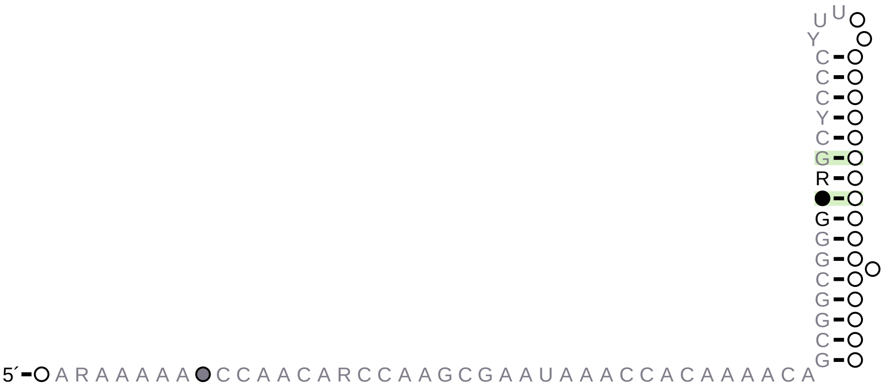
5´
A R A A A A A
C C A A C A R C C A A G C G A A U A A A C C A C A A A A C A
G C G G C G G G R G C Y C C C Y U U

HMP2_9654_1

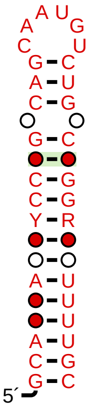

HMP2_9657_1

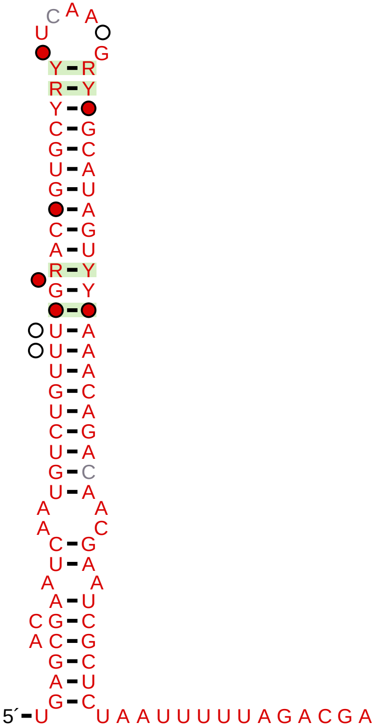

HMP2_9662_1

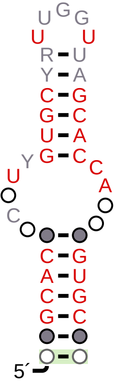

HMP2_9664_1

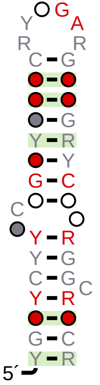

HMP2_9666_1

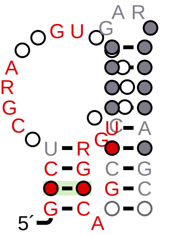

HMP2_9668_1

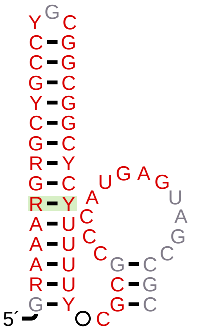

G
Y C
C G
C G
G C
Y G
C G
G C
R Y
G C
R Y
A U
A U
A U
R U
G Y
5´
U G A G
A U
C A
C G
C C
G C
C G
G C
C

HMP2_9679_1

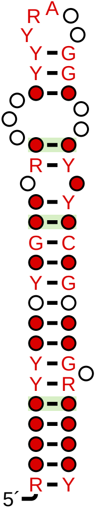

HMP2_9688_1

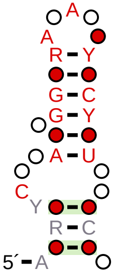

HMP2_9690_1

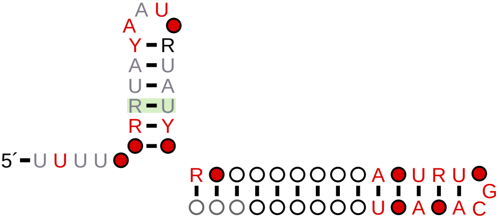

HMP2_9695_1

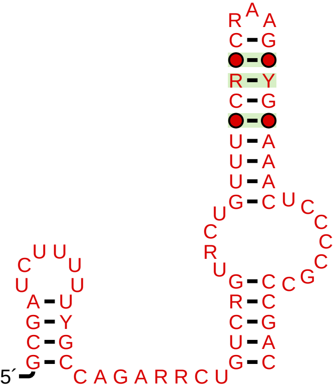

R A A
C G
R Y
C G
U A
U A
U A
G C
U C C C C G C C
U C R U
G C
R C
C G
U A
G C
C U U U U U
A U
G Y
C G
G C
5´
C A G A R R C U

HMP2_9696_1

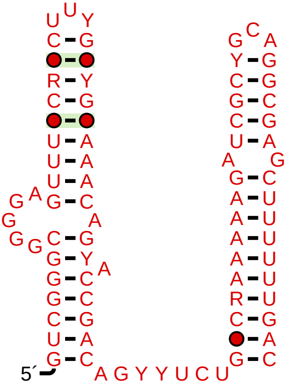

HMP2_9698_1

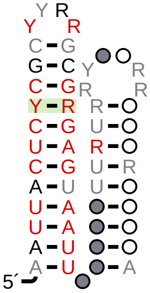

HMP2_970_1

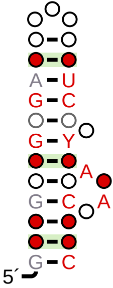

HMP2_9700_1

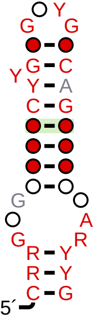

HMP2_971_1

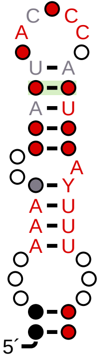

HMP2_9711_1

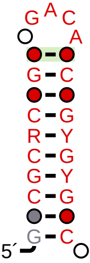

HMP2_9712_1

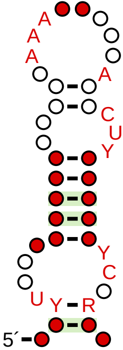

HMP2_9725_1

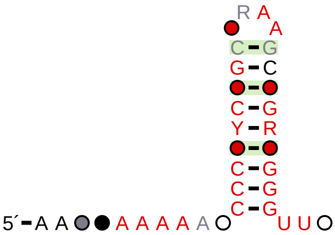

HMP2_9726_1

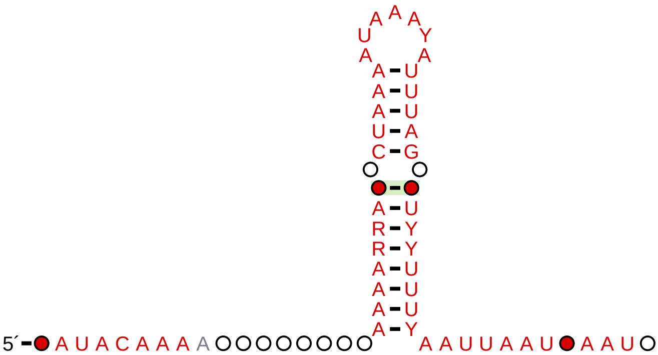

5´
A U A C A A A A
A A U U A A U A A U
A U A A A Y A
A A A U C
U U U A G
A R R A A A A
U Y Y U U U Y

HMP2_9733_1

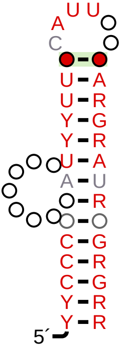

HMP2_9736_1

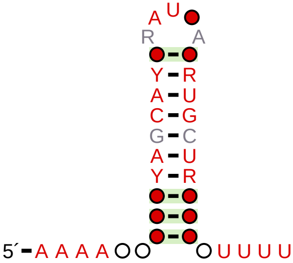

HMP2_9738_1

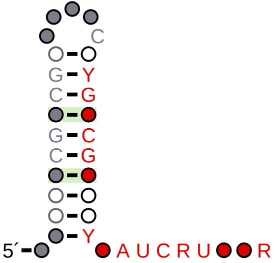

HMP2_9740_1

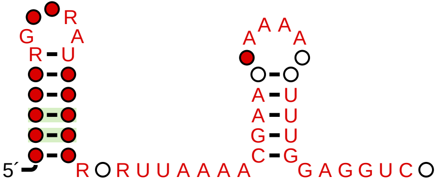

HMP2_9751_1

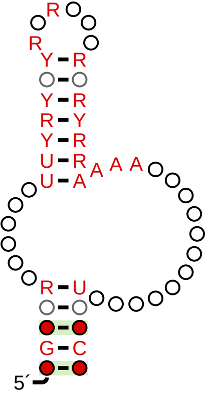

HMP2_9758_1

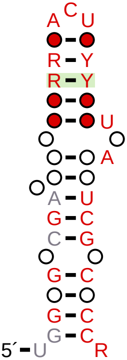

HMP2_9767_1

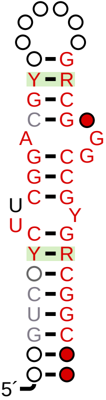

HMP2_9769_1

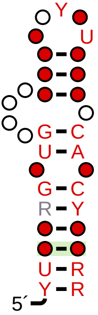

HMP2_977_1

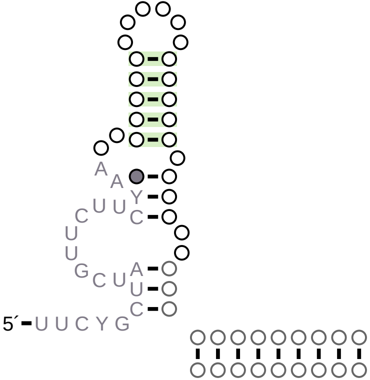

HMP2_9779_1

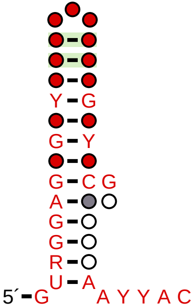

HMP2_9784_1

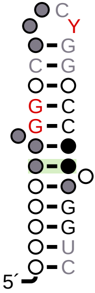

HMP2_9785_1

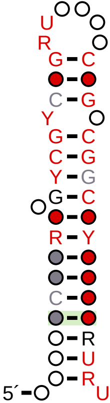

HMP2_9791_1

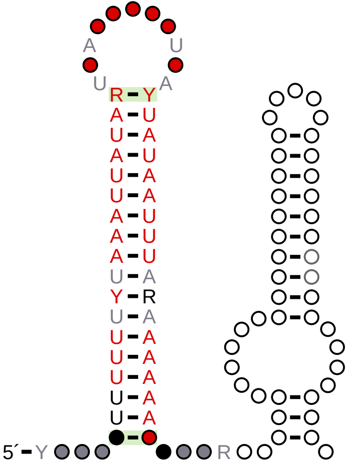

HMP2_9792_1

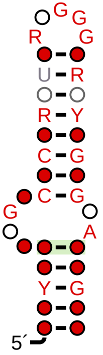

HMP2_9805_1

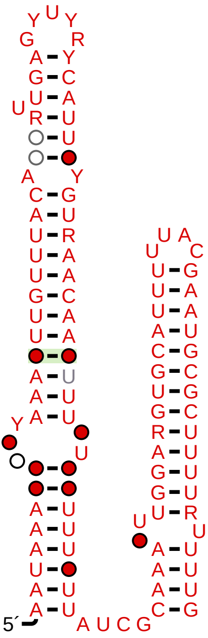

HMP2_9806_1

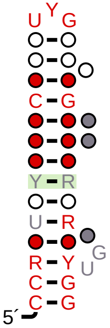

HMP2_981_1

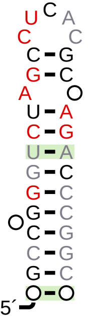

HMP2_9817_1

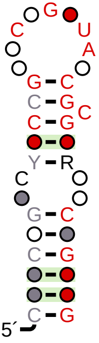

HMP2_9818_1

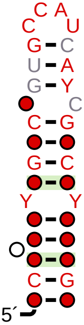

HMP2_9820_1

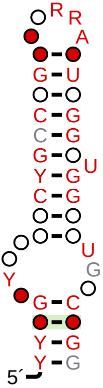

HMP2_9822_1

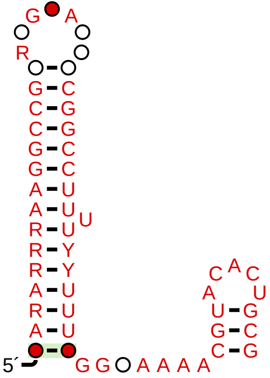

HMP2_9823_1

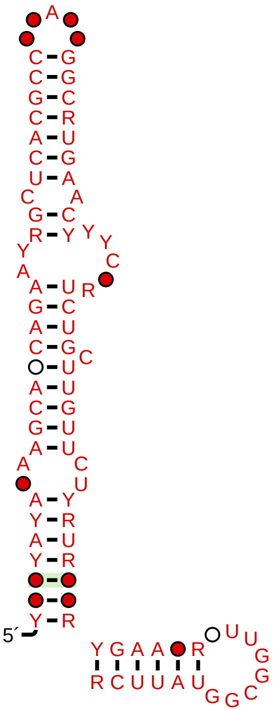

HMP2_9848_1

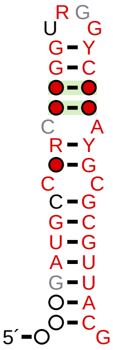

HMP2_985_1

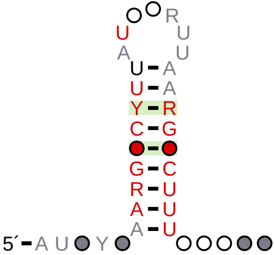

HMP2_9850_1

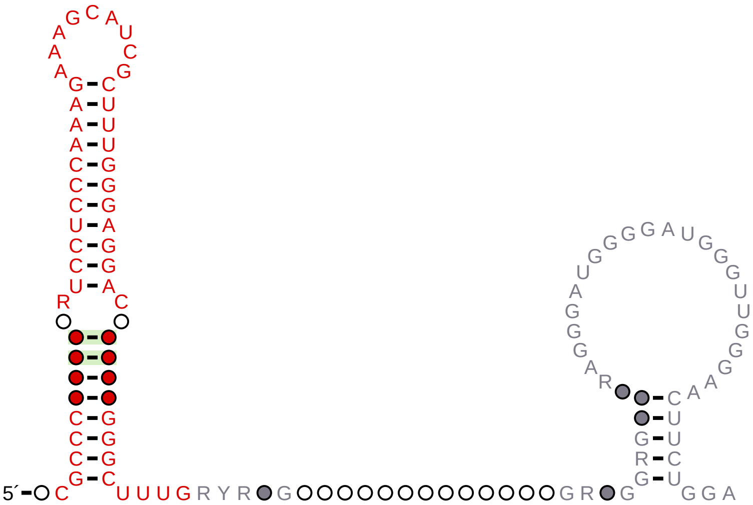

HMP2_9855_1

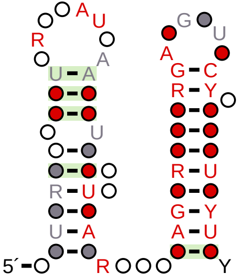

HMP2_9856_1

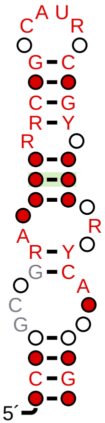

HMP2_9857_1

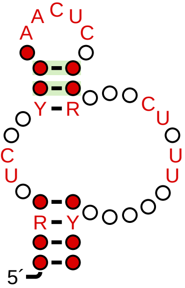

HMP2_9861_1

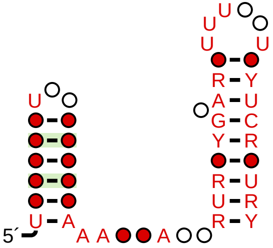

HMP2_9882_1

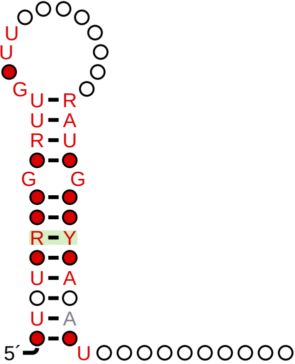

HMP2_9883_1

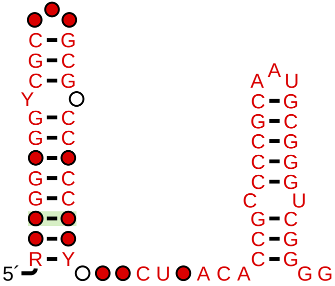

HMP2_9889_1

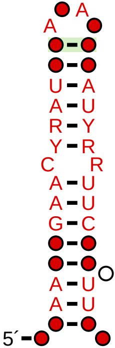

HMP2_9892_1

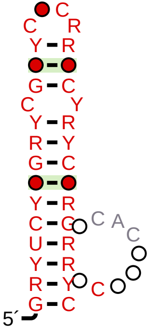

HMP2_9893_1

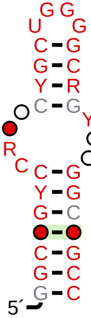

HMP2_9895_1

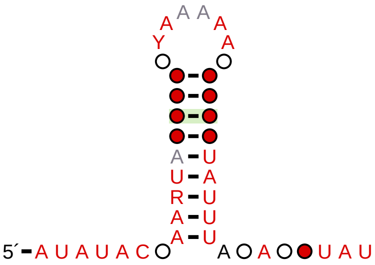

HMP2_9898_1

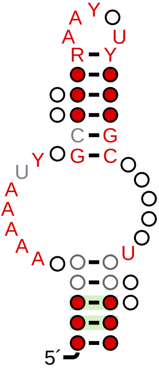

HMP2_99_1

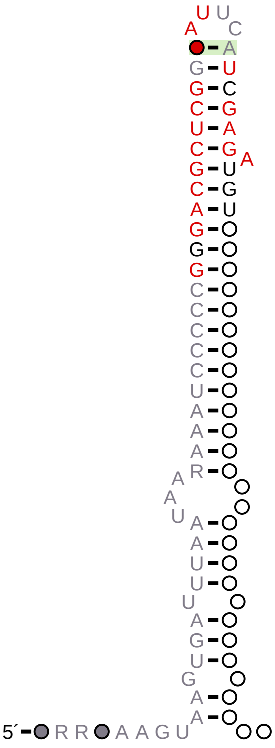

HMP2_9905_1

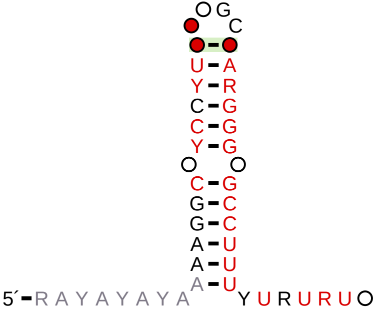

HMP2_9910_1

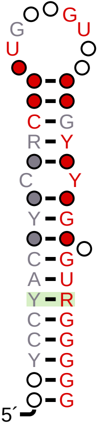

HMP2_9911_1

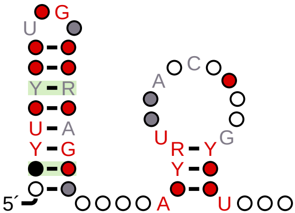

HMP2_9918_1

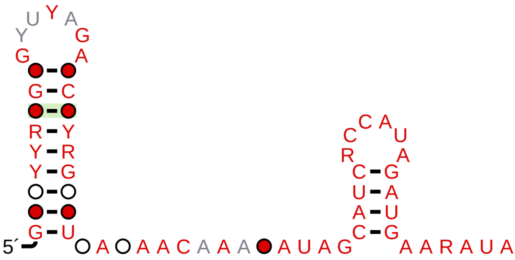

HMP2_992_1

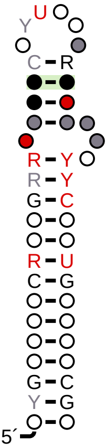

HMP2_9923_1

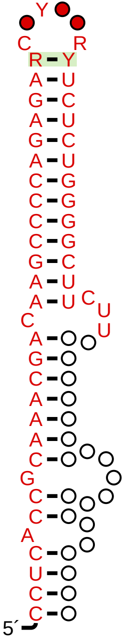

HMP2_9930_1

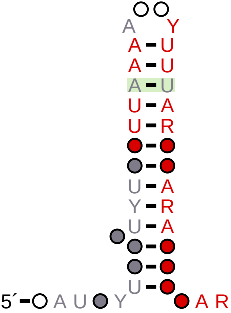

HMP2_9931_1

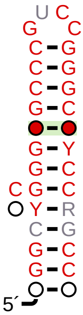

HMP2_9956_1

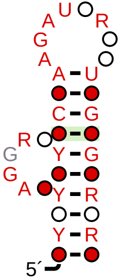

HMP2_9957_1

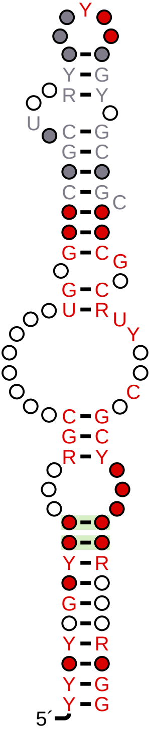

HMP2_9960_1

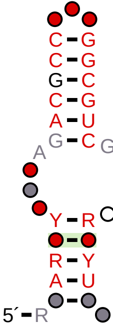

HMP2_9965_1

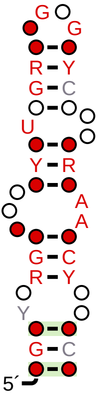

HMP2_997_1

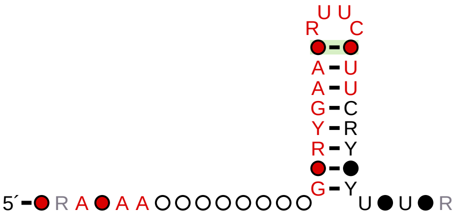

HMP2_9974_1

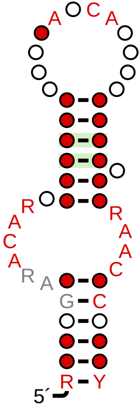

HMP2_9978_1

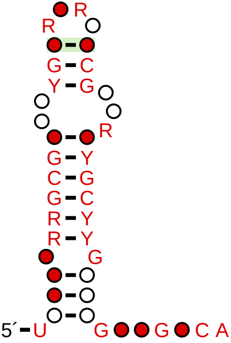

HMP2_9981_1

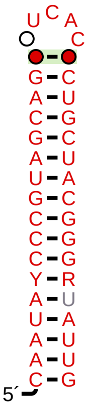

HMP2_9988_1

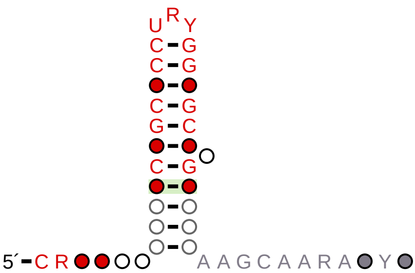

HMP2_9990_1

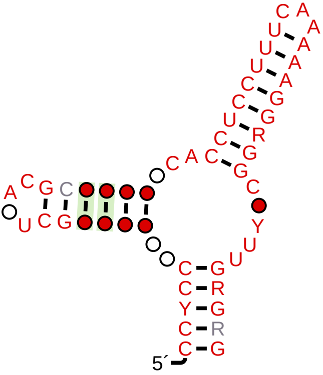

HMP2_9993_1

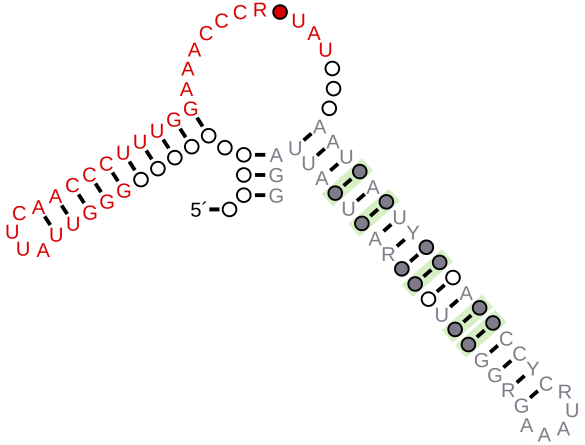

HMP2_9997_1

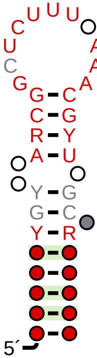

HMP2_1_1

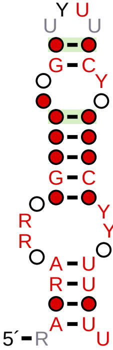

HMP2_10_1

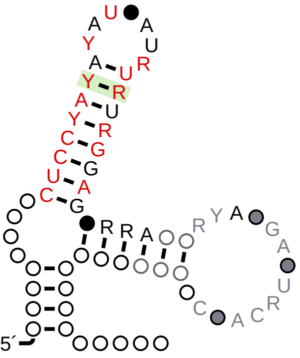

HMP2_1000_1

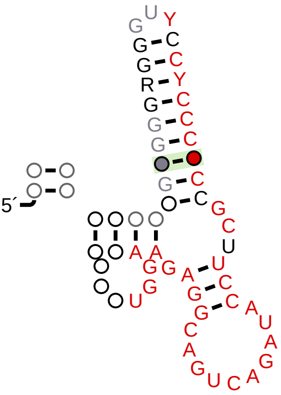

HMP2_10000_1

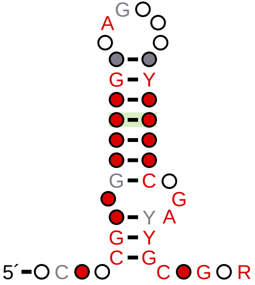

HMP2_10005_1

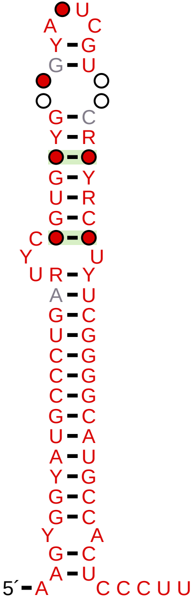

HMP2_10007_1

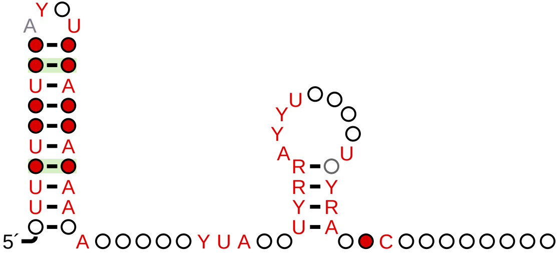

HMP2_10008_1

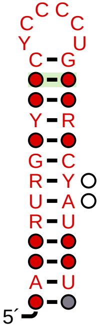

HMP2_1001_1

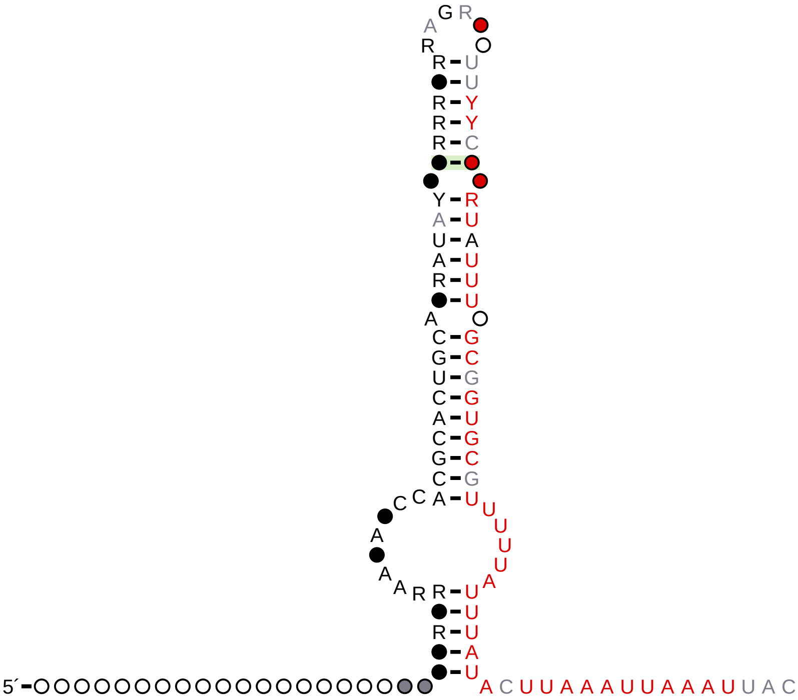

HMP2_10019_1

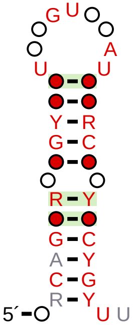

HMP2_10032_1

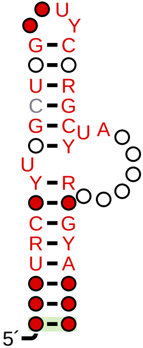

HMP2_10037_1

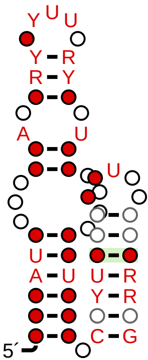

HMP2_1004_1

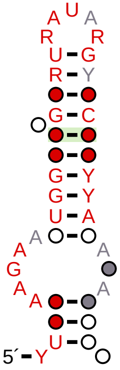

HMP2_10040_1

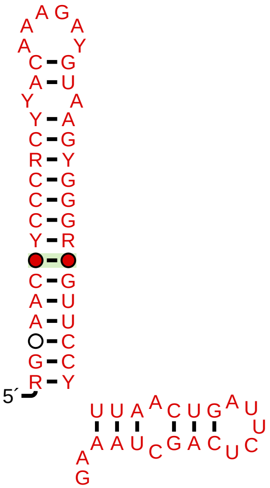

HMP2_10042_1

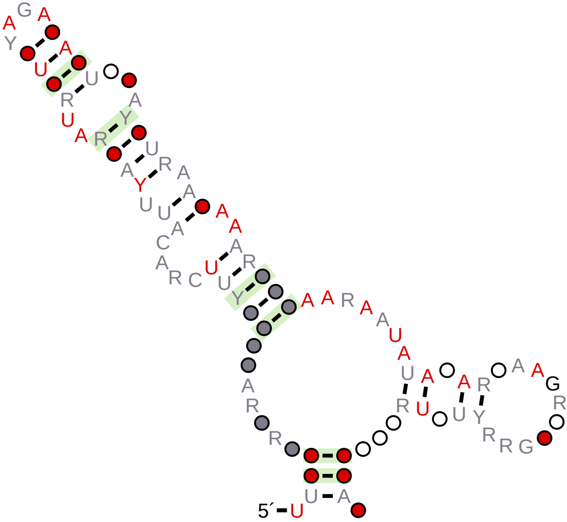

HMP2_10052_1

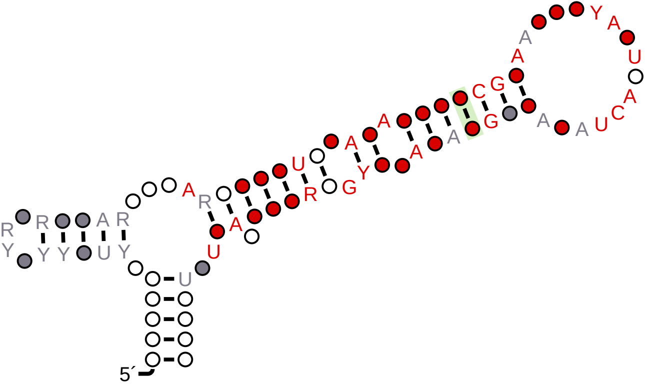

HMP2_10054_1

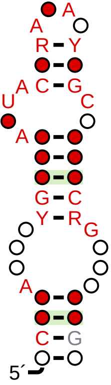

HMP2_10058_1

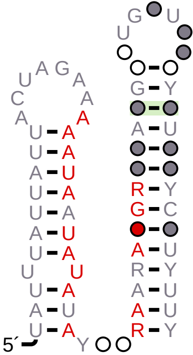

HMP2_10062_1

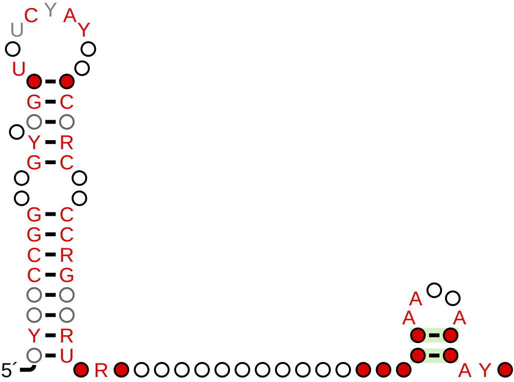

HMP2_10065_1

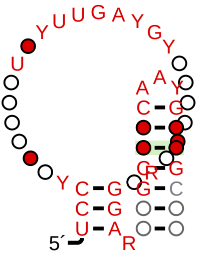

HMP2_1007_1

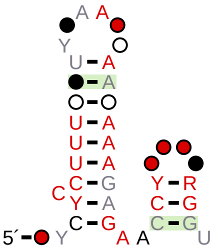

HMP2_10074_1

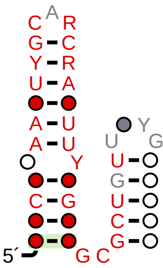

HMP2_10077_1

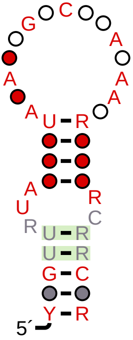

HMP2_10079_1

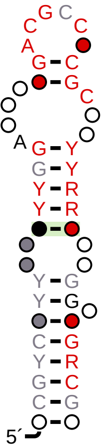

HMP2_10094_1

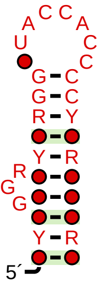

HMP2_10096_1

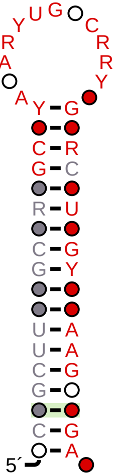

HMP2_101_1

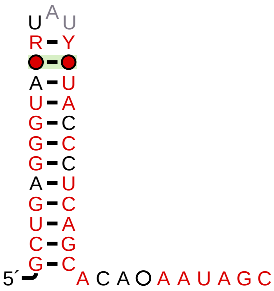

HMP2_10100_1

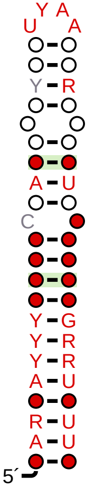

HMP2_10128_1

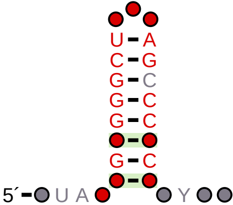

HMP2_10129_1

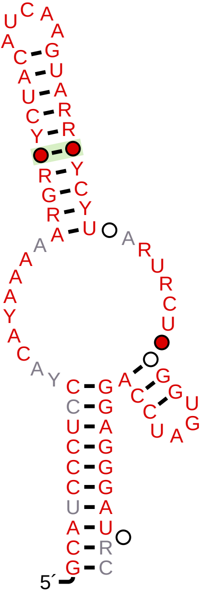

HMP2_10134_1

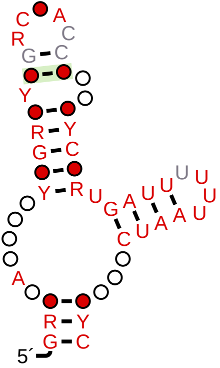

HMP2_10151_1

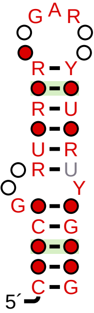

HMP2_10152_1

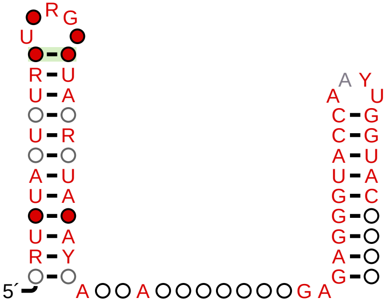

HMP2_10154_1

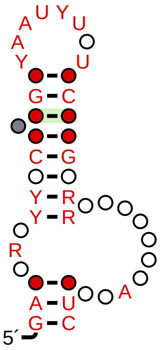

HMP2_1016_1

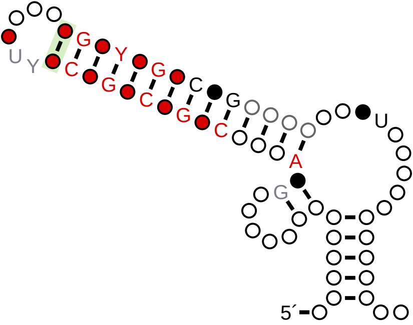

HMP2_10160_1

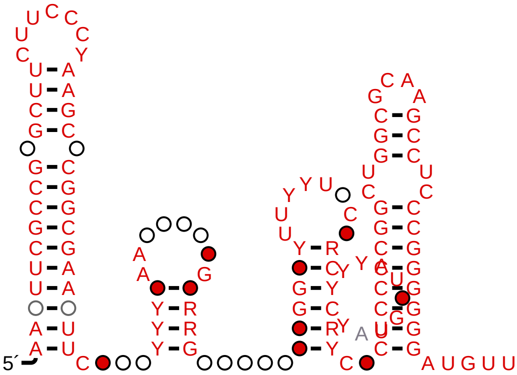

HMP2_10162_1

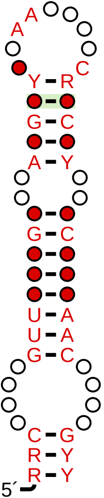

HMP2_10165_1

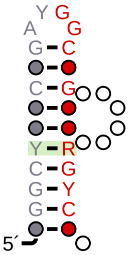

HMP2_1017_1

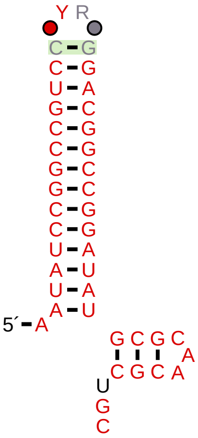

HMP2_10188_1

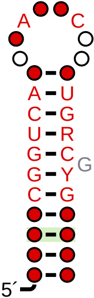

HMP2_10195_1

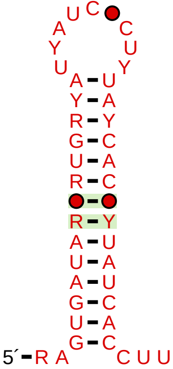

HMP2_10198_1

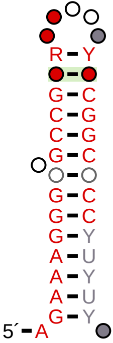

HMP2_1020_1

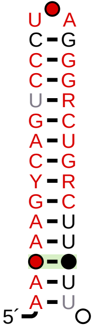

HMP2_10203_1

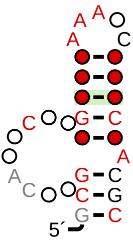

HMP2_10204_1

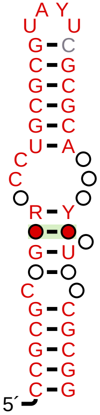

HMP2_10208_1

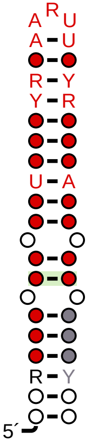

HMP2_10215_1

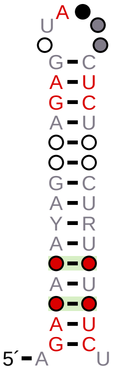

HMP2_10229_1

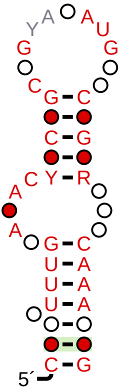

HMP2_10232_1

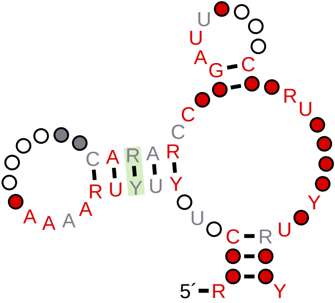

HMP2_10233_1

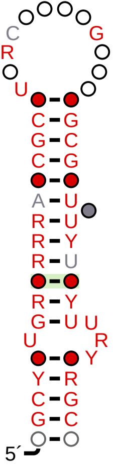

HMP2_10240_1

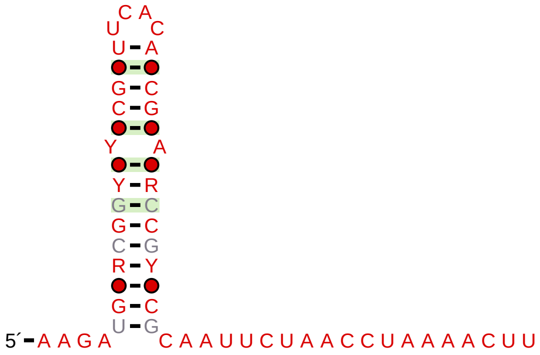

C A
U C
U-A
G-C
C-G
Y A
Y-R
G-C
G-C
C-G
R-Y
G-C
U-G
5´-A A G A C A A U U C U A A C C U A A A A C U U

HMP2_10243_1

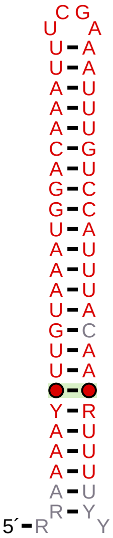

HMP2_10255_1

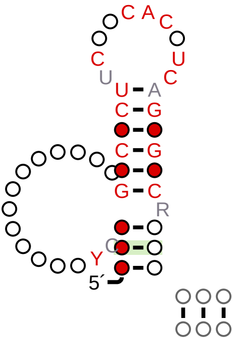

HMP2_10266_1

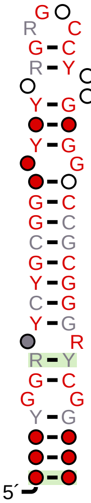

HMP2_10271_1

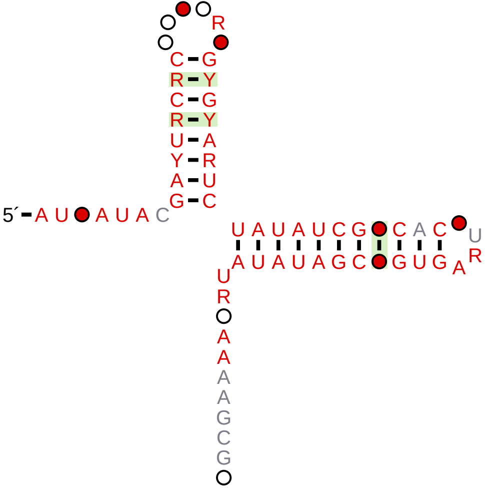

R
C G
R Y
C G
R Y
U A
Y R
A U
G C
5´ A U A U A C
U A U A U C G C A C U
A U A U A G C G U G A R
U
R
A
A
A
A
G
C
G

HMP2_10275_1

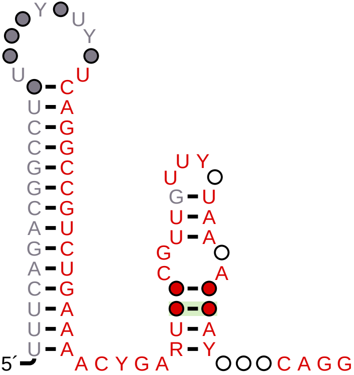

5´
Y
U
Y
U
U
C
U
A
C
G
C
G
G
C
G
C
C
G
A
U
G
C
A
U
C
G
U
A
U
A
U
A
A C Y G A
U Y
U
G
U
U
A
U
A
G
C
A
U
A
R
Y
C A G G

HMP2_10277_1

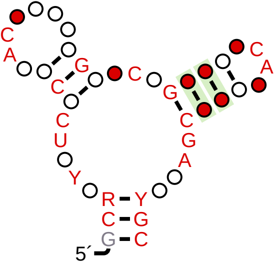

HMP2_10280_1

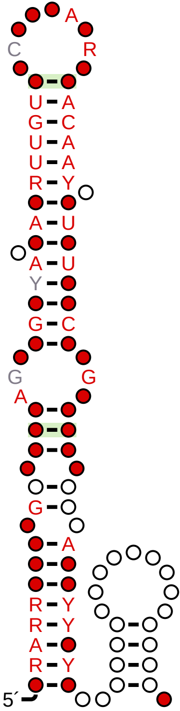

HMP2_10284_1

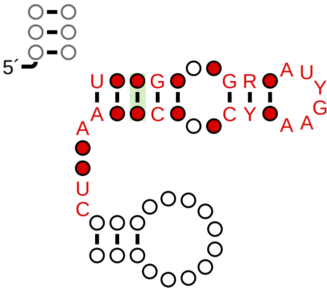

HMP2_10289_1

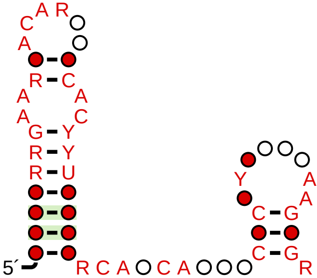

HMP2_10292_1

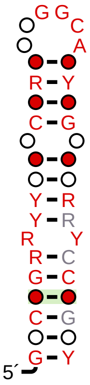

HMP2_10293_1

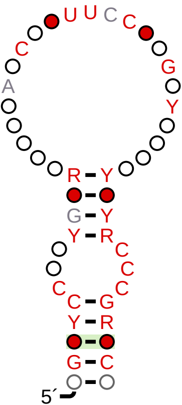

HMP2_10296_1

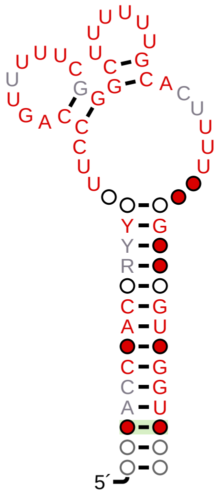

HMP2_10302_1

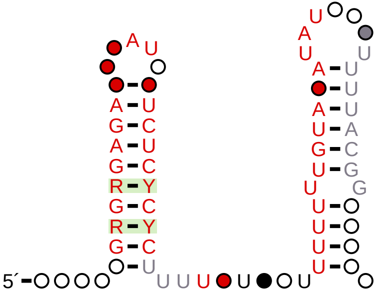

HMP2_10305_1

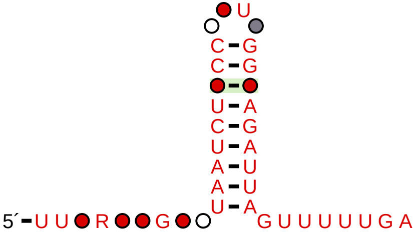

HMP2_10311_1

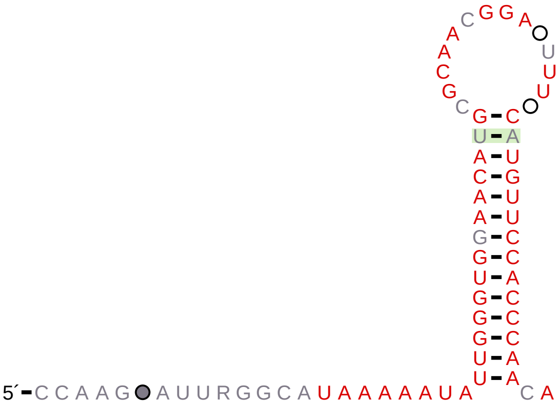
5´
C C A A G A U U R G G C A U A A A A A U A
U U G G G U G G A A C A U G
C G C A A C G G A
U U U
C G U G A A U C C A C C C U U A A
C A

HMP2_10315_1

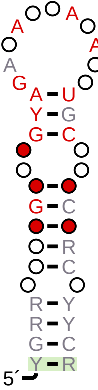

HMP2_10316_1

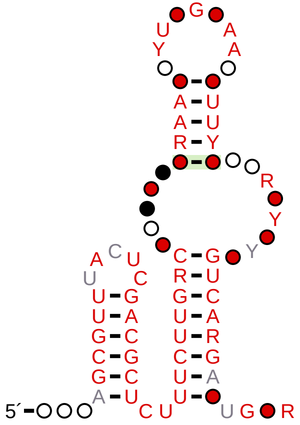

G
U A
Y A
A U
A U
R Y
R
Y
Y
C G
R U
G C
U A
U R
C G
U A
U
A C U
U C
U G
U A
G C
C G
G C
A U
5´
C U
U G R

HMP2_10318_1

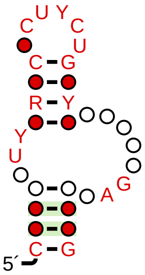

HMP2_1032_1

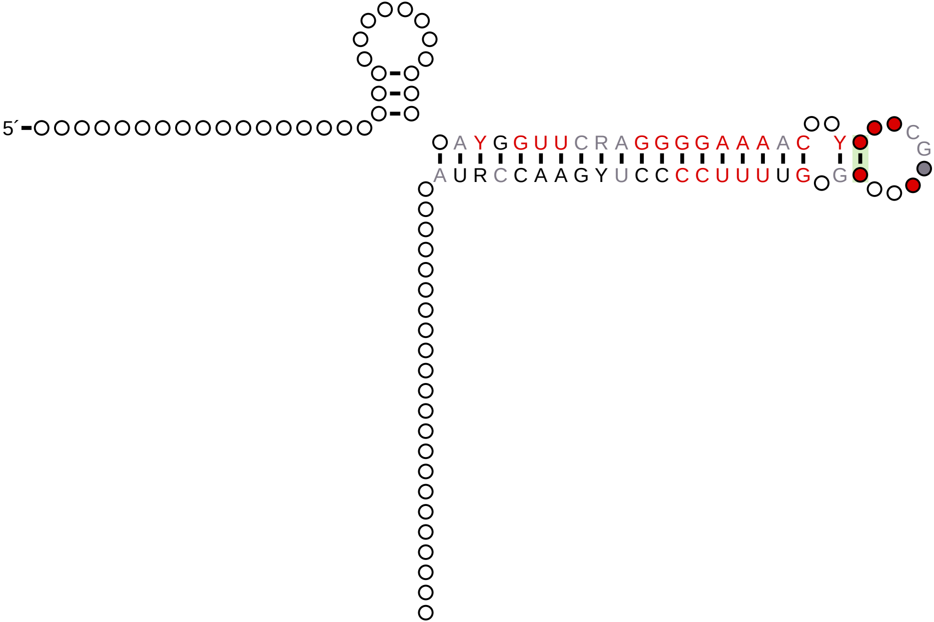

HMP2_10321_1

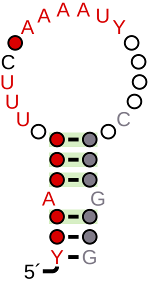

HMP2_10325_1

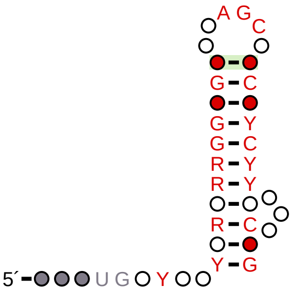

HMP2_10328_1

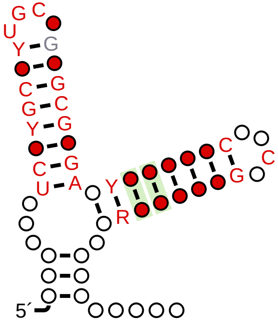

HMP2_10331_1

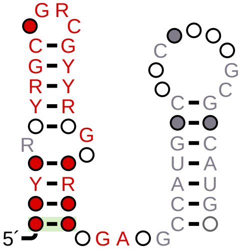

HMP2_10337_1

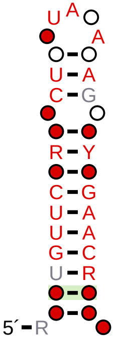

HMP2_1034_1

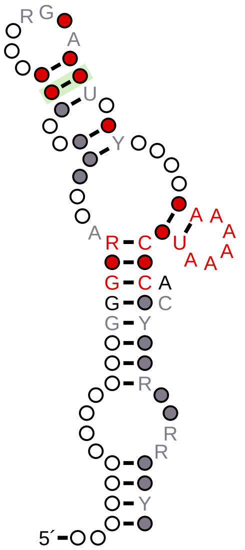

HMP2_10340_1

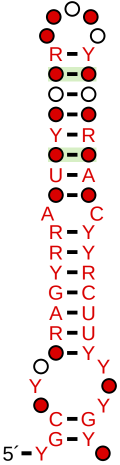

HMP2_10341_1

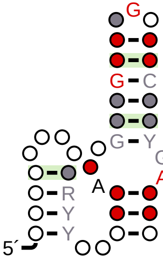

HMP2_1035_1

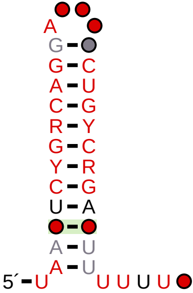

HMP2_10352_1

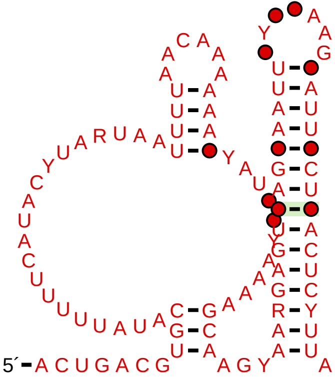

HMP2_10353_1

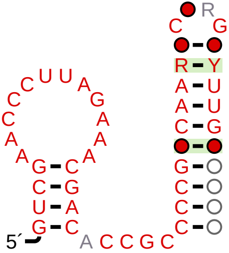

HMP2_10359_1

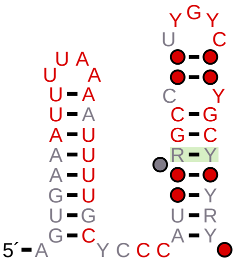

HMP2_10361_1

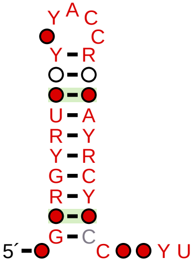

HMP2_10364_1

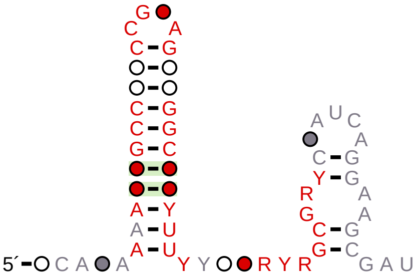

HMP2_10366_1

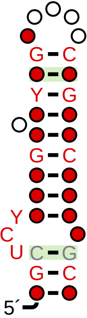

HMP2_10369_1

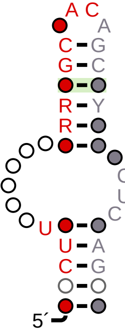

HMP2_1037_1

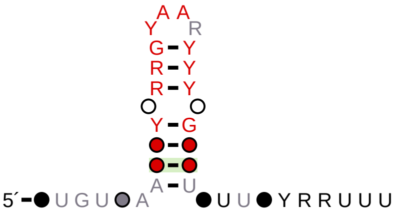

HMP2_10370_1

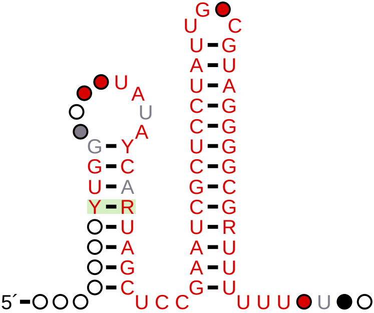

G
U C
U G
A U
U A
C G
C G
U G
C G
G C
C G
U R
A U
A U
G U
U
A
U
A
G Y
G C
U A
Y R
U
A
G
C
5´
U C C
U U U
U

HMP2_10374_1

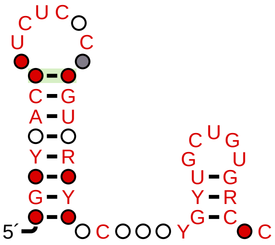

HMP2_10382_1

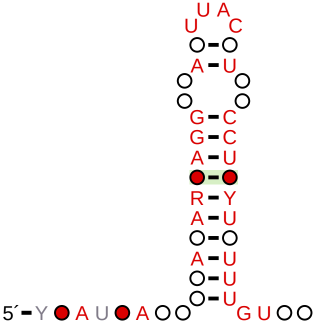

HMP2_10386_1

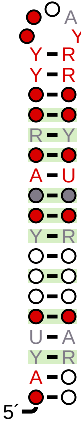

HMP2_10388_1

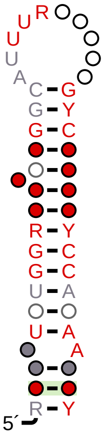

HMP2_10393_1

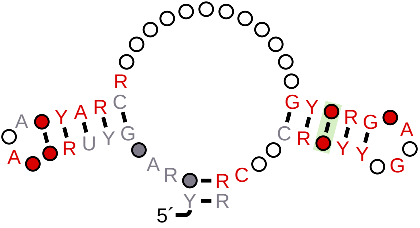

HMP2_10398_1

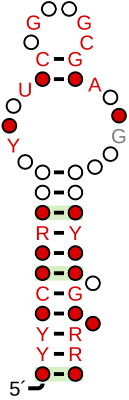

HMP2_104_1

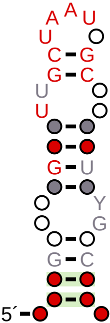

HMP2_1040_1

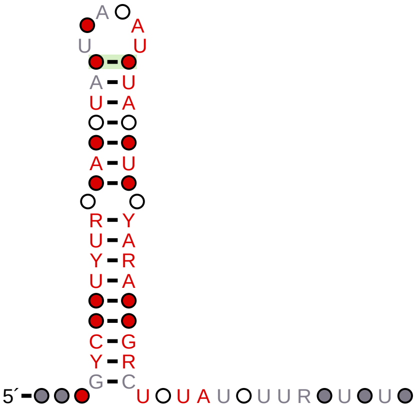

HMP2_10400_1

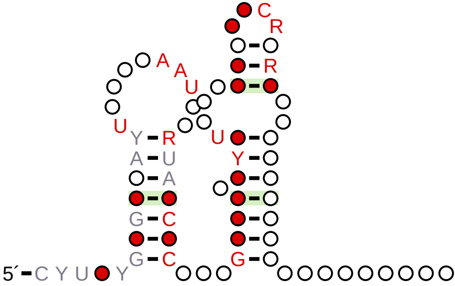

HMP2_10409_1

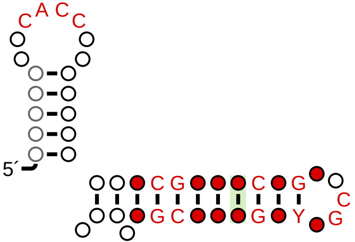

HMP2_1041_1

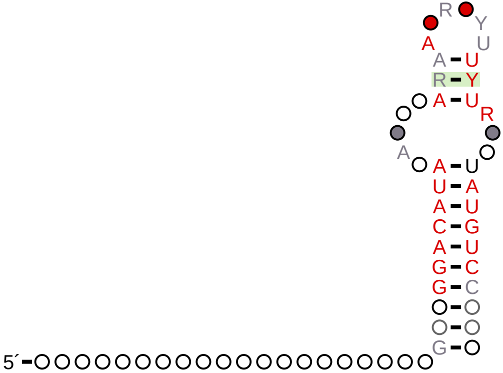

HMP2_10424_1

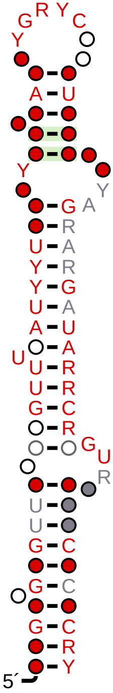

HMP2_10427_1

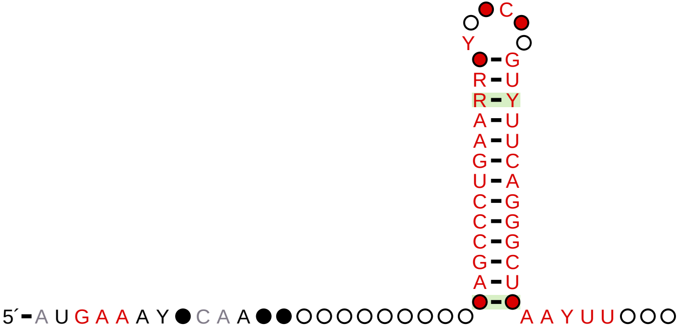

HMP2_10437_1

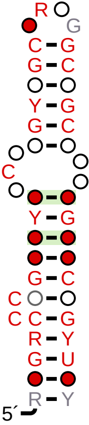

HMP2_10438_1

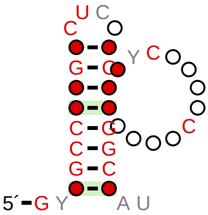

HMP2_10439_1

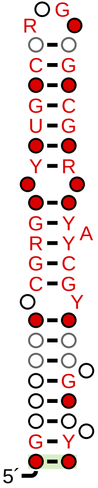

HMP2_10441_1

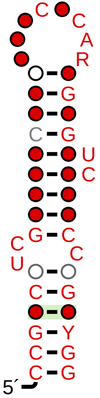

HMP2_10445_1

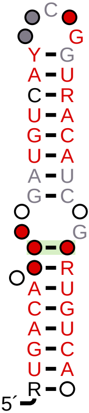

HMP2_10448_1

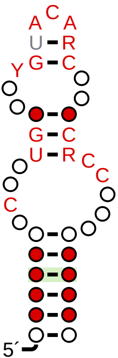

HMP2_10449_1

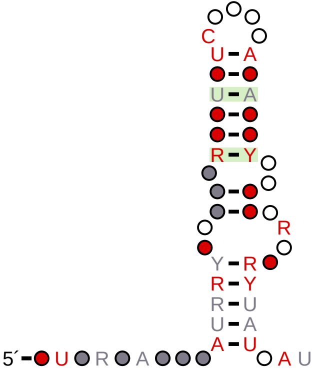

HMP2_10461_1

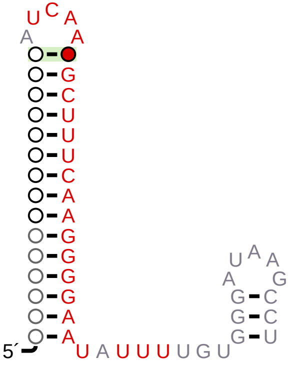

HMP2_1048_1

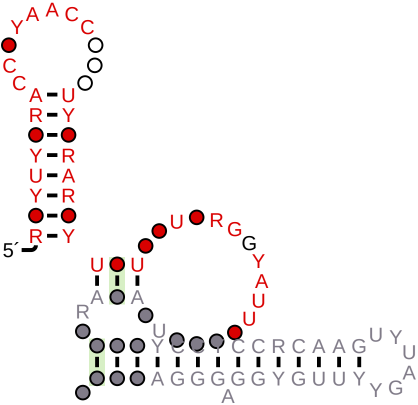

HMP2_10480_1

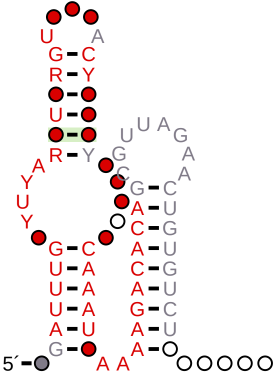

HMP2_10481_1

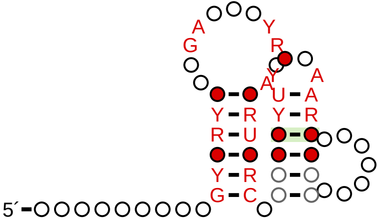

HMP2_10487_1

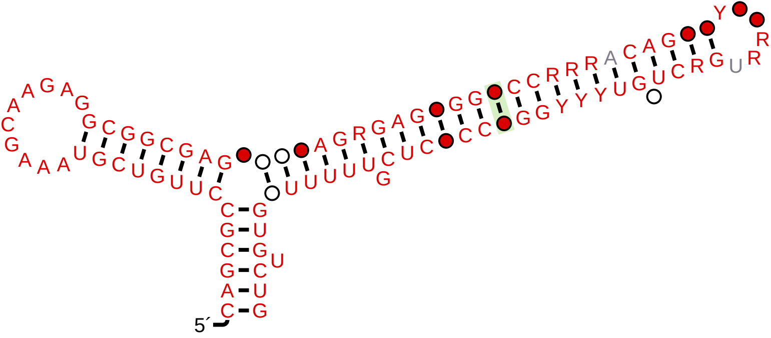

HMP2_1049_1

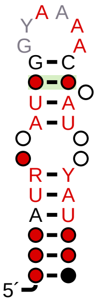

HMP2_10490_1

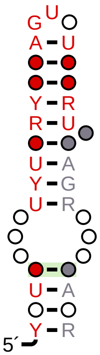

HMP2_105_1

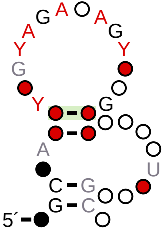

HMP2_10504_1

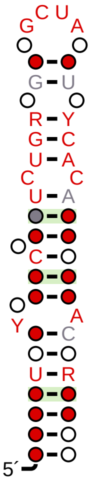

HMP2_10510_1

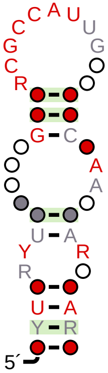

HMP2_10514_1

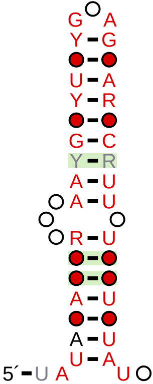

HMP2_10519_1

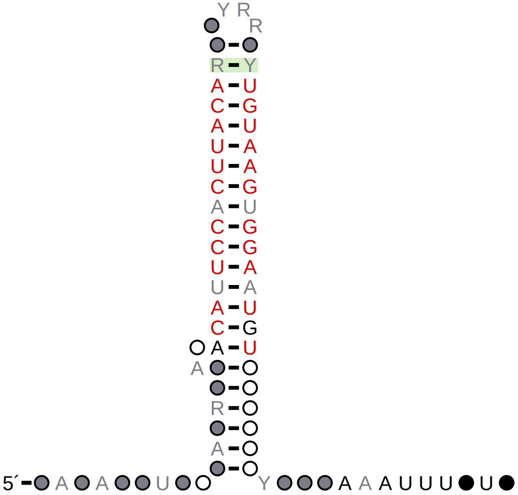

HMP2_10529_1

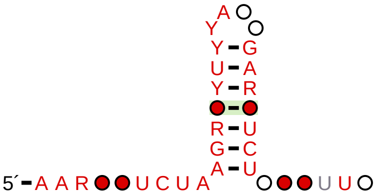

HMP2_1053_1

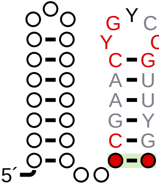

HMP2_10530_1

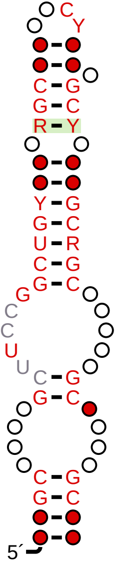

HMP2_10538_1

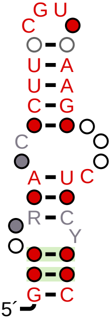

HMP2_10543_1

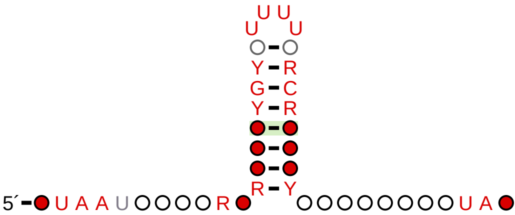

HMP2_1055_1

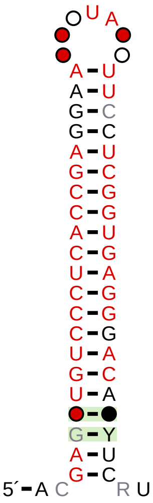

HMP2_10558_1

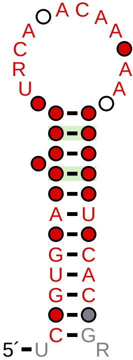

HMP2_10570_1

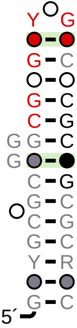

HMP2_10574_1

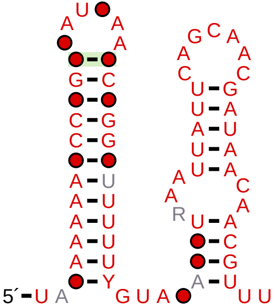

HMP2_10575_1

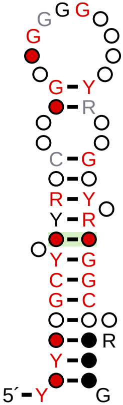

HMP2_1058_1

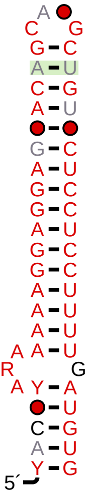

HMP2_10584_1

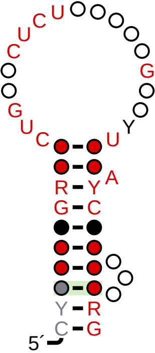

HMP2_10589_1

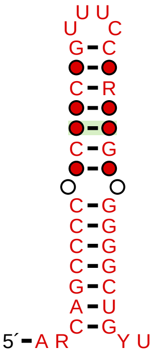

HMP2_10594_1

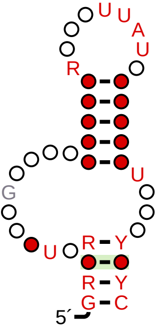

HMP2_10596_1

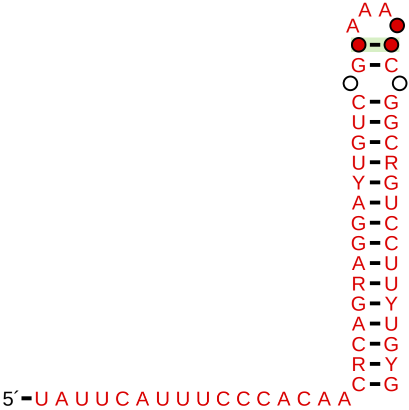
5´
U A U U C A U U U C C C A C A A
A A A
G C
C G
U G
G C
U R
Y G
A U
G C
G C
A U
R U
G Y
A U
C G
R Y
C G

HMP2_10600_1

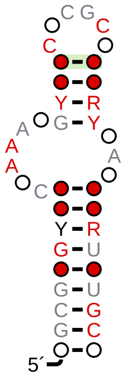

HMP2_10606_1

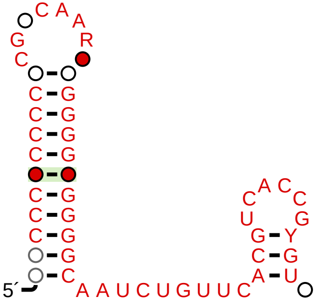

HMP2_10608_1

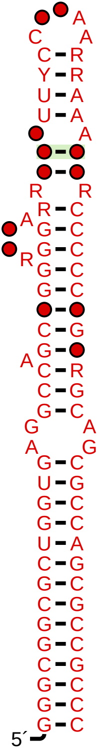

HMP2_1061_1

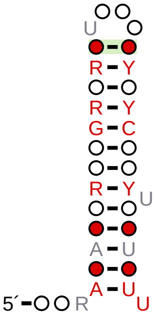

HMP2_10612_1

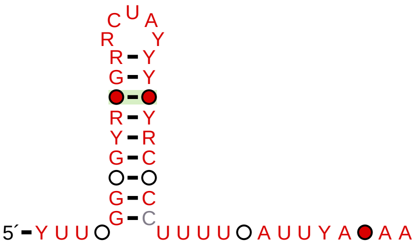

HMP2_10613_1

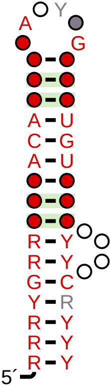

HMP2_10615_1

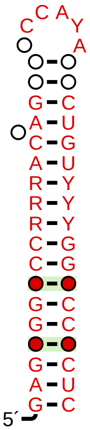

HMP2_10616_1

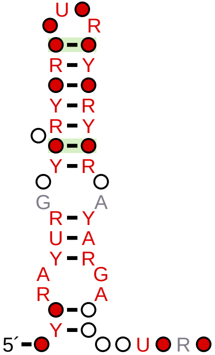

HMP2_1062_1

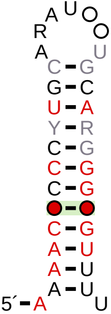

HMP2_10623_1

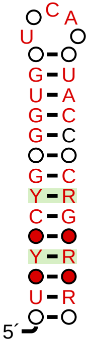

HMP2_10637_1

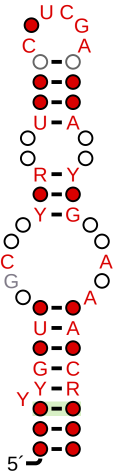

HMP2_10642_1

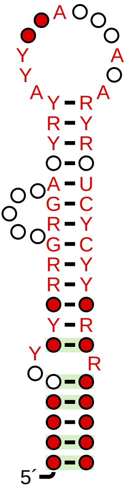

HMP2_1065_1

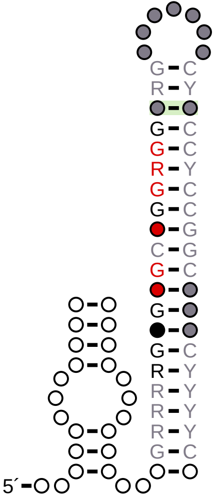

HMP2_10651_1

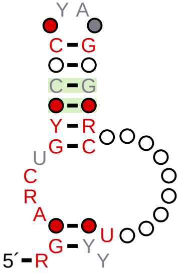

HMP2_10659_1

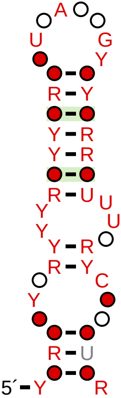

HMP2_10676_1

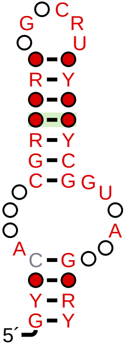

HMP2_10681_1

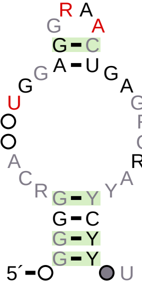

HMP2_10690_1

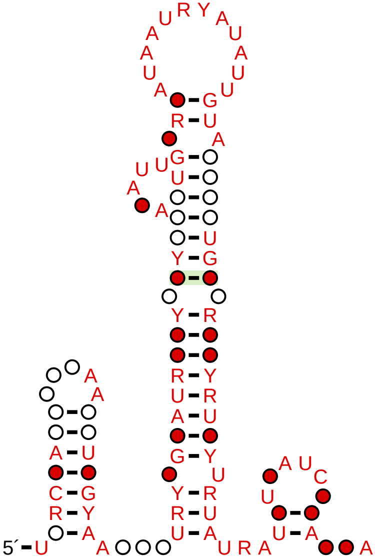

HMP2_10698_1

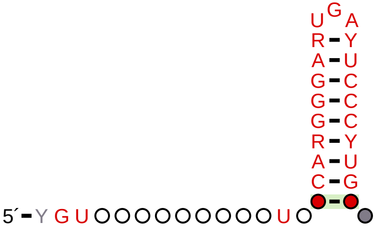

HMP2_1071_1

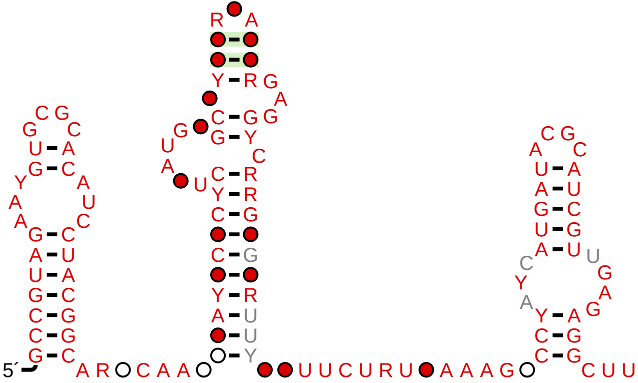

HMP2_10710_1

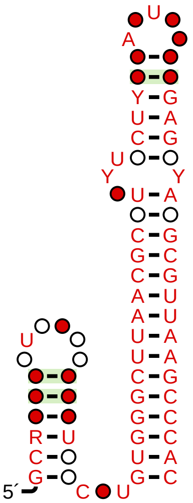

HMP2_10711_1

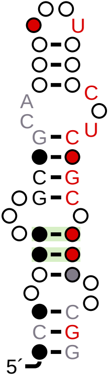

HMP2_10718_1

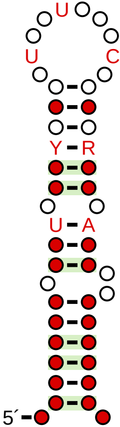

HMP2_10721_1

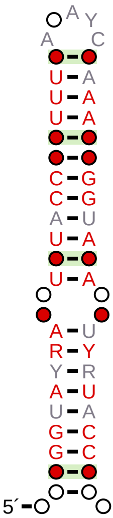

HMP2_10722_1

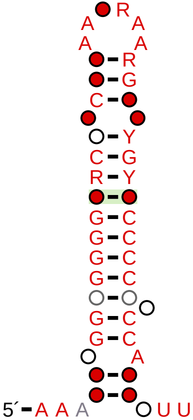

HMP2_10723_1

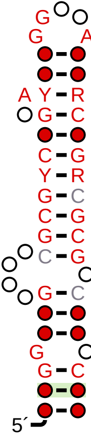

HMP2_10724_1

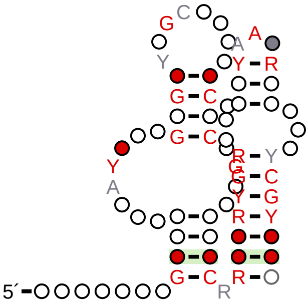

HMP2_1073_1

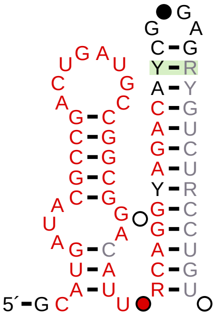

HMP2_10737_1

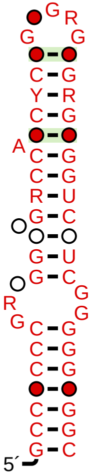

HMP2_10749_1

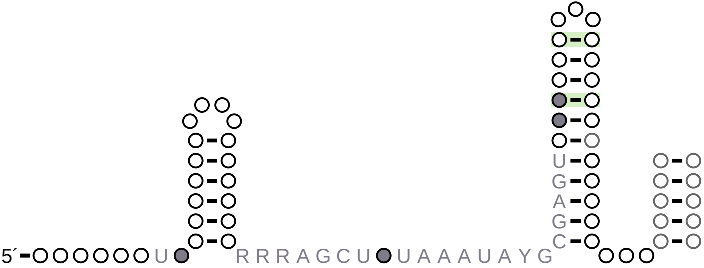

HMP2_10756_1

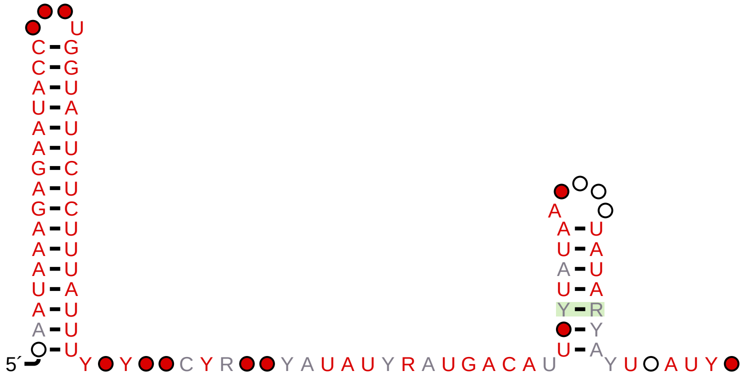

HMP2_10765_1

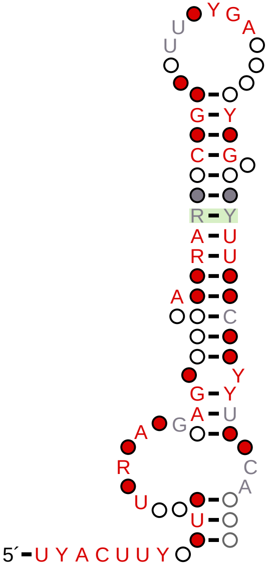

HMP2_10789_1

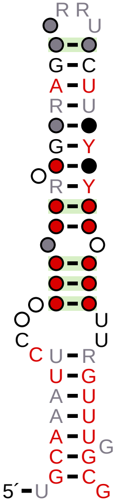

HMP2_1079_1

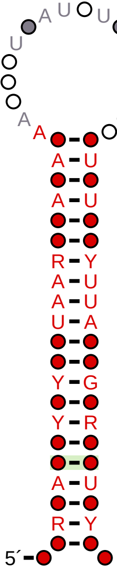

HMP2_1080_1

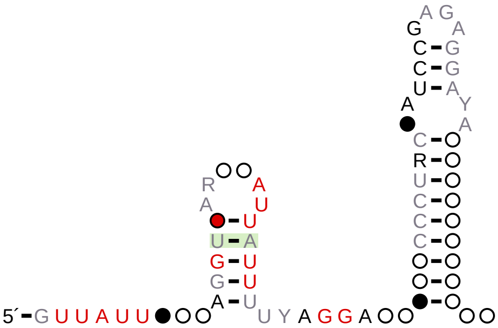

HMP2_10803_1

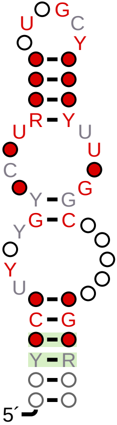

HMP2_10805_1

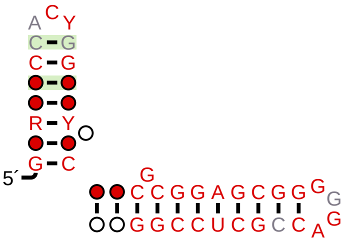

HMP2_10807_1

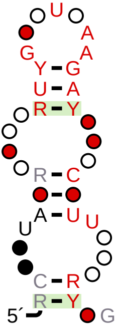

HMP2_10808_1

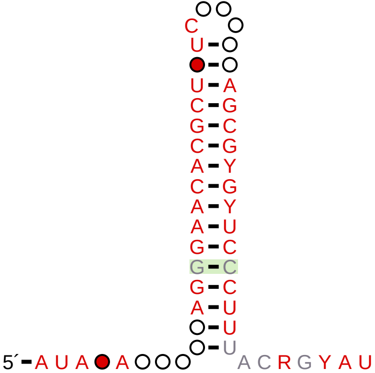

HMP2_10809_1

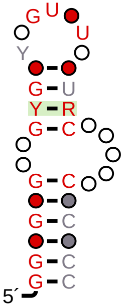

HMP2_10823_1

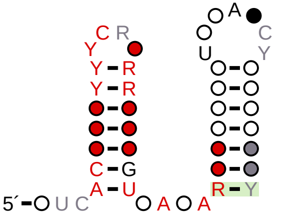

HMP2_10825_1

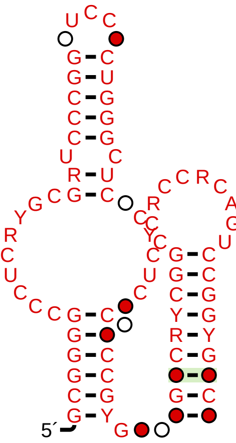

HMP2_10828_1

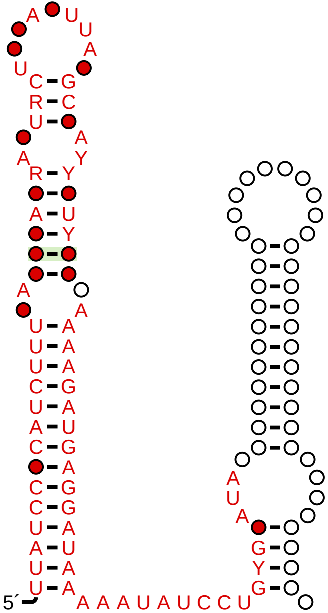

HMP2_1083_1

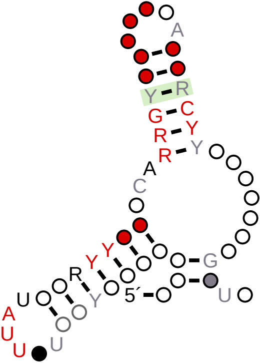

HMP2_10831_1

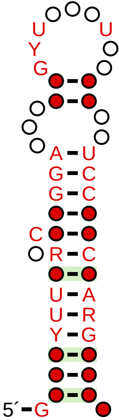

HMP2_10832_1

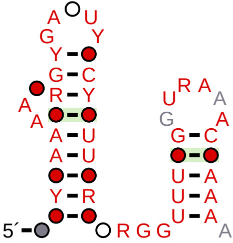

HMP2_1084_1

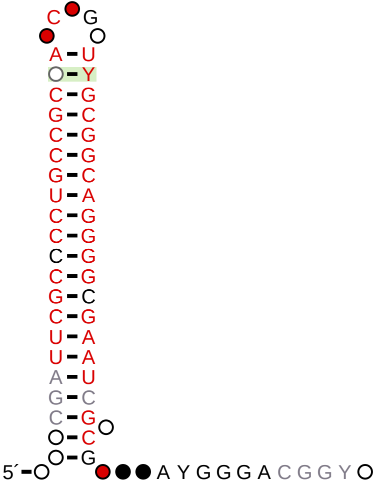
C G
A U
Y
C G
G C
C G
C G
G C
U A
C G
C G
C G
C G
G C
C G
U A
U A
A U
G C
C G
C
G
5´
A Y G G G A C G G Y

HMP2_10843_1

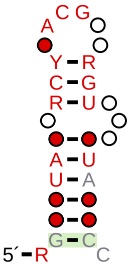

HMP2_10846_1

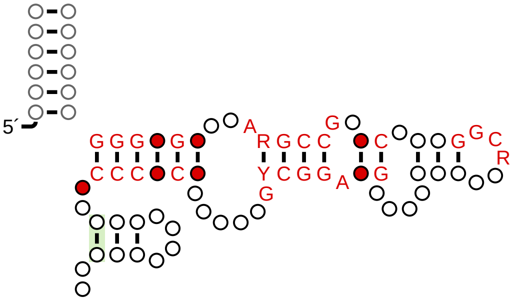

HMP2_10854_1

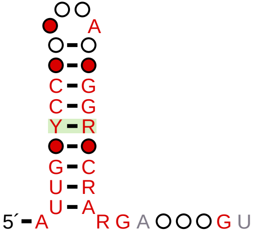

HMP2_10857_1

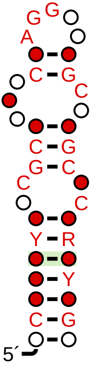

HMP2_10859_1

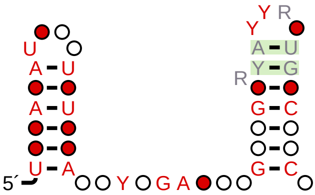

HMP2_1087_1

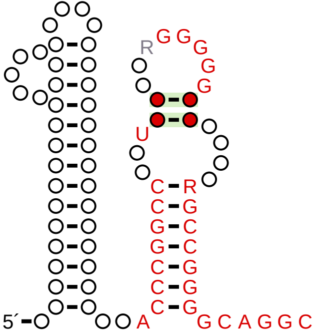

HMP2_10873_1

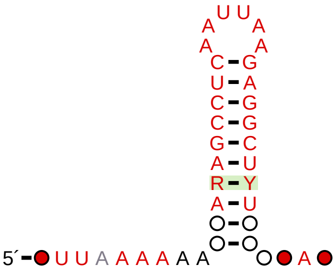

HMP2_10875_1

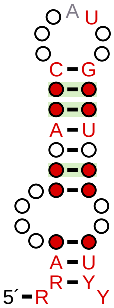

HMP2_10887_1

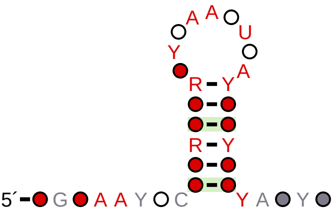

HMP2_1089_1

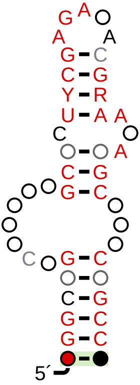

HMP2_10895_1

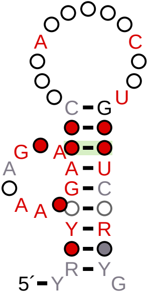

HMP2_10897_1

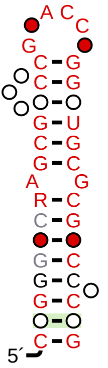

HMP2_10898_1

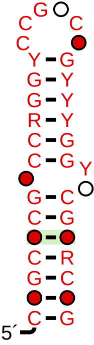

HMP2_10900_1

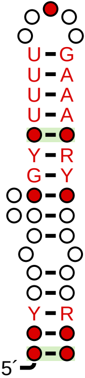

HMP2_10906_1

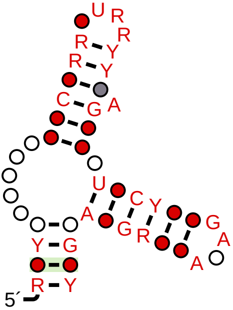

HMP2_1092_1

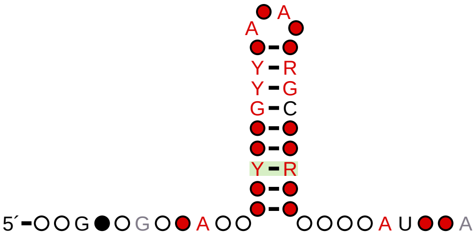

HMP2_1093_1

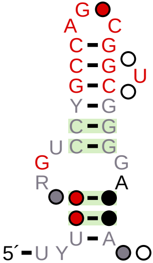

HMP2_10932_1

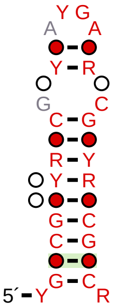

HMP2_10942_1

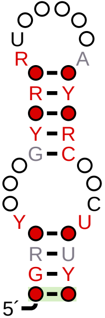

HMP2_10951_1

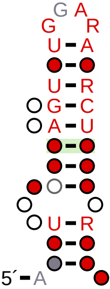

HMP2_10954_1

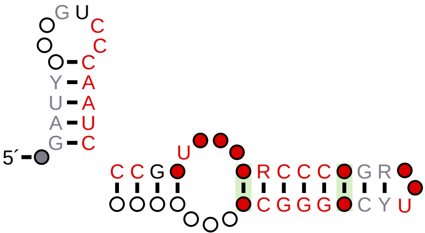

HMP2_10961_1

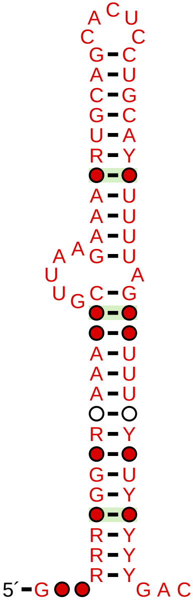
5´
G
A
C
U
C
C
G
A
C
G
U
R
C
U
G
C
A
Y
A
A
A
G
A
A
U
U
G
C
U
U
U
U
A
G
A
A
A
U
U
U
R
Y
G
U
G
Y
R
R
R
Y
Y
Y
G A C

HMP2_10964_1

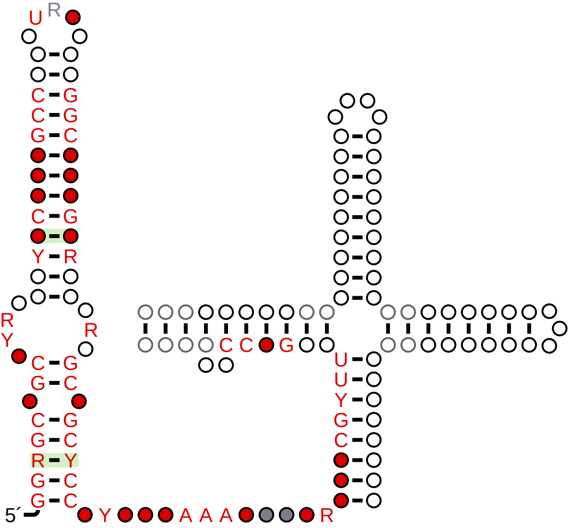

HMP2_10966_1

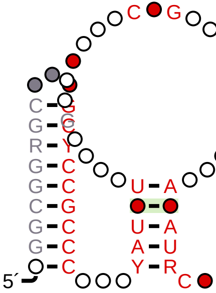

HMP2_10968_1

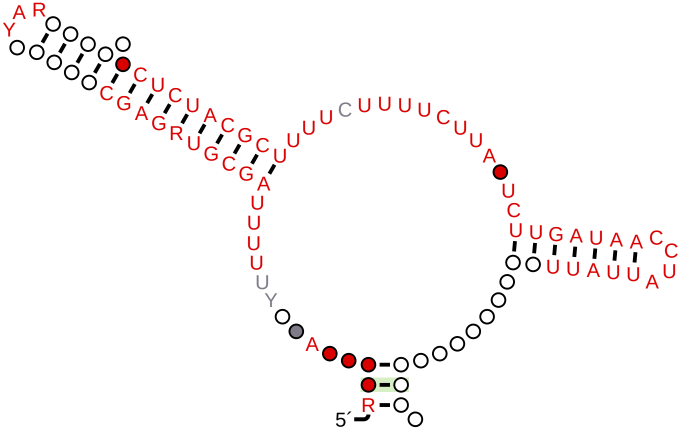

HMP2_10970_1

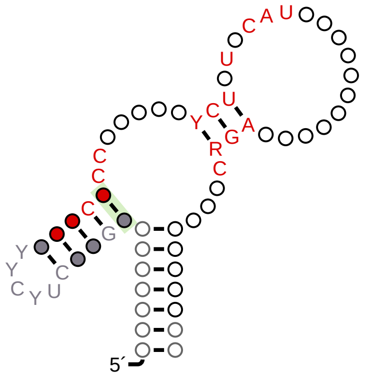

HMP2_10971_1

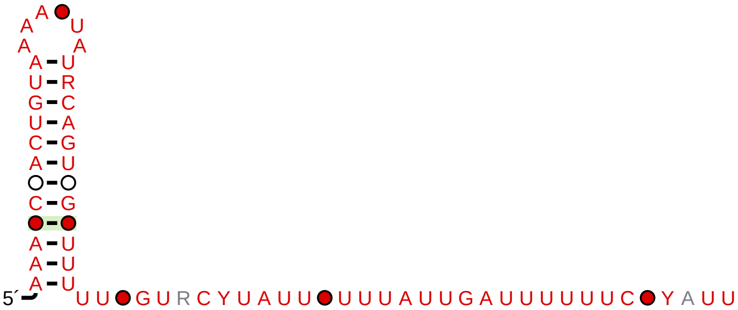

HMP2_10972_1

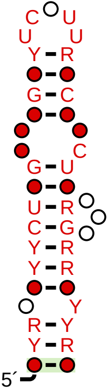

HMP2_10975_1

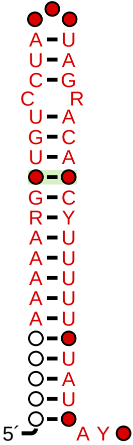

HMP2_10978_1

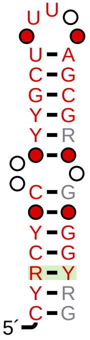

HMP2_10984_1

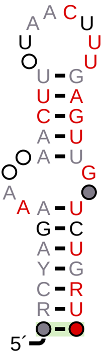

HMP2_1099_1

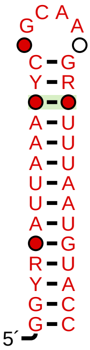

HMP2_10995_1

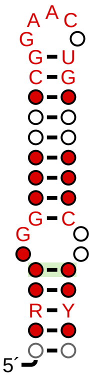

HMP2_10998_1

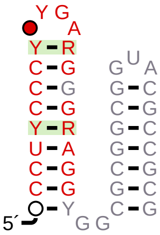

HMP2_11003_1

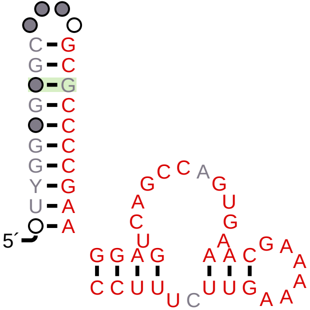

HMP2_1101_1

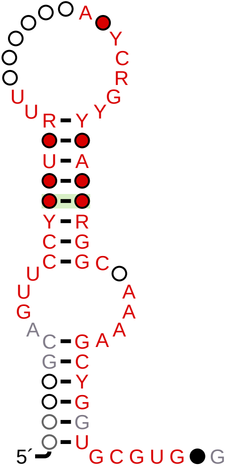

HMP2_11015_1

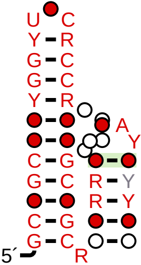

HMP2_11021_1

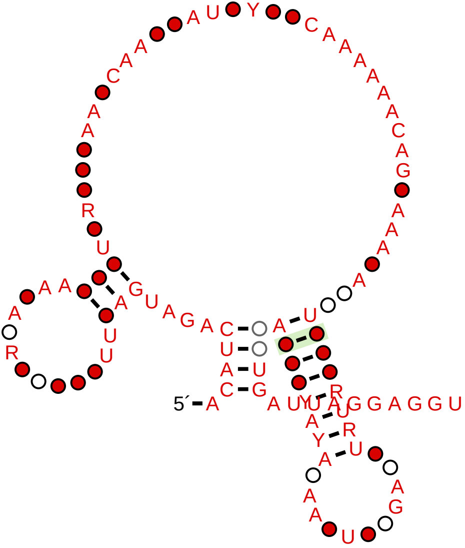

HMP2_11027_1

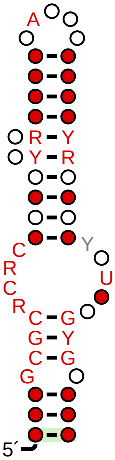

HMP2_11030_1

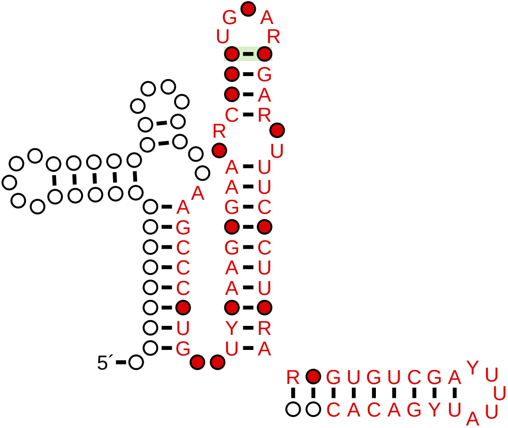

HMP2_11033_1

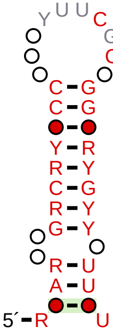

HMP2_11037_1

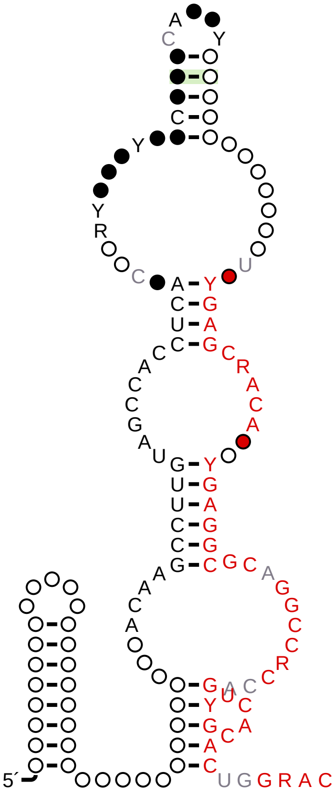

HMP2_11043_1

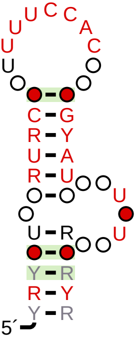

HMP2_11047_1

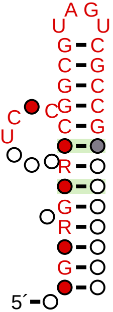

HMP2_11064_1

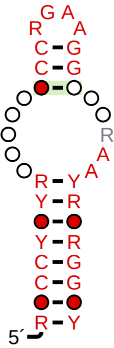

HMP2_11066_1

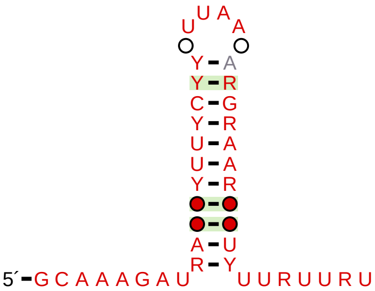

HMP2_11078_1

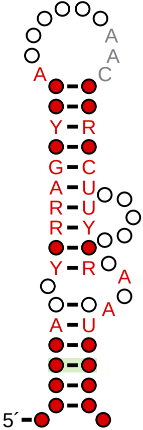

HMP2_11079_1

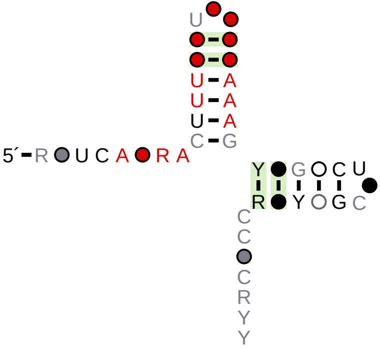

HMP2_11080_1

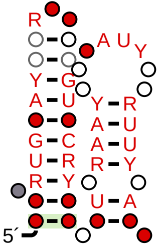

HMP2_11088_1

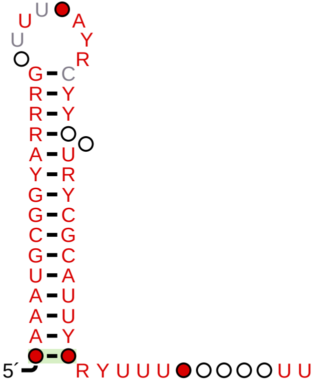

HMP2_11089_1

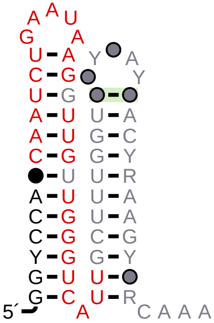

HMP2_11092_1

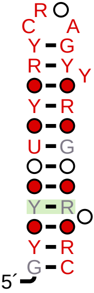

HMP2_11093_1

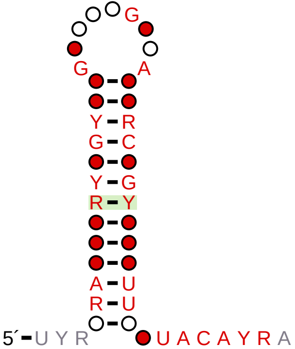

HMP2_111_1

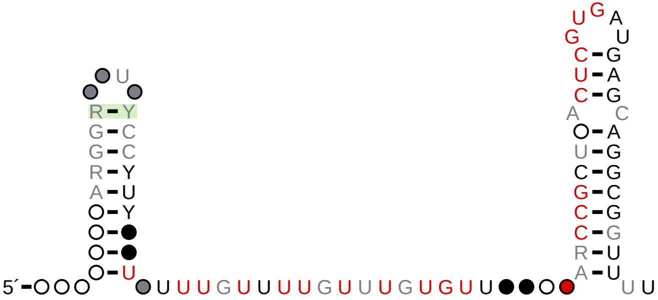

HMP2_1110_1

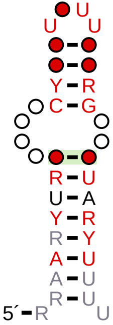

HMP2_11109_1

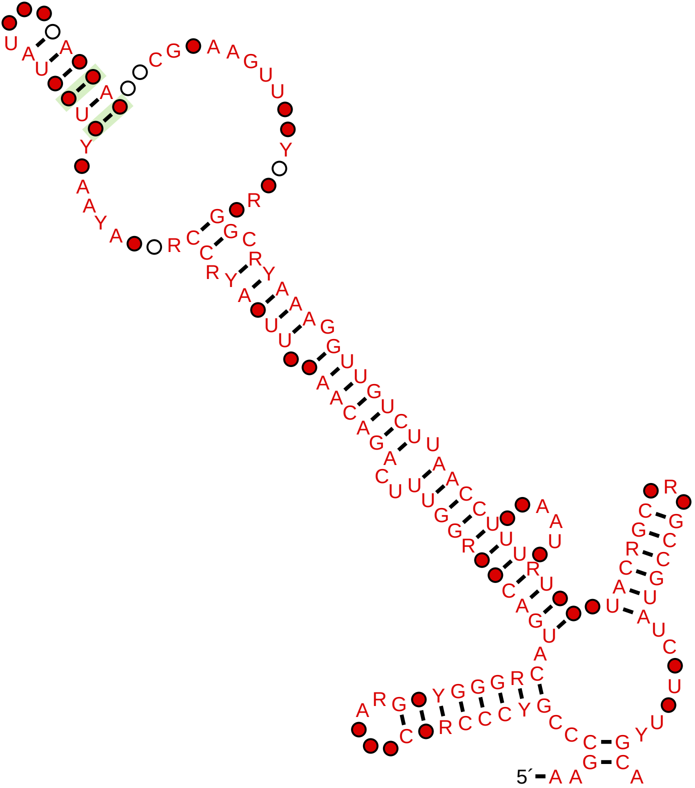

HMP2_11112_1

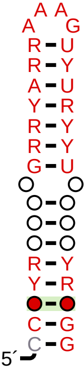

HMP2_11116_1

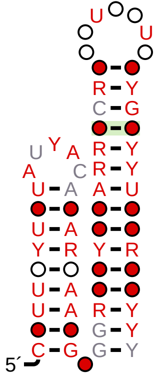

HMP2_11120_1

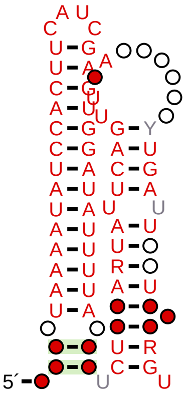

HMP2_11124_1

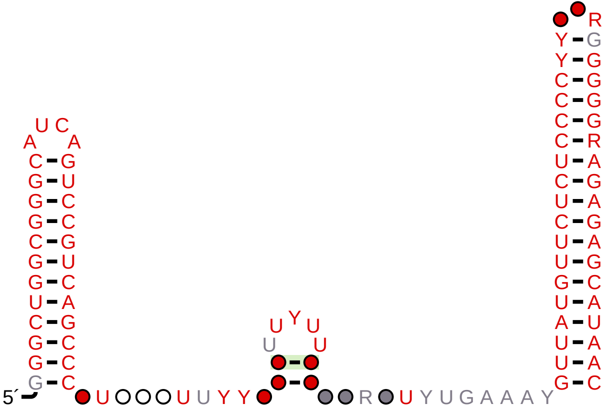

HMP2_11129_1

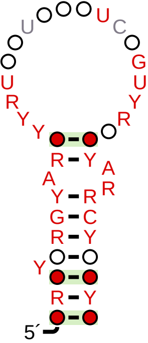

HMP2_11137_1

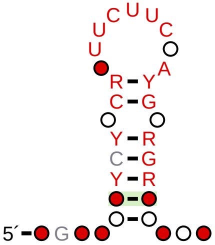

HMP2_11139_1

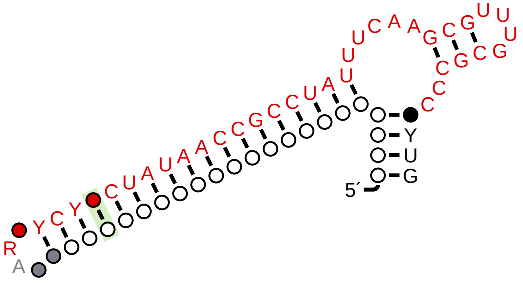

HMP2_11146_1

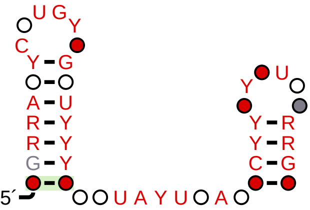

HMP2_11152_1

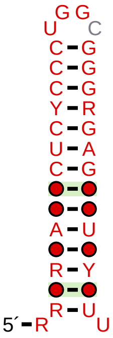

HMP2_11172_1

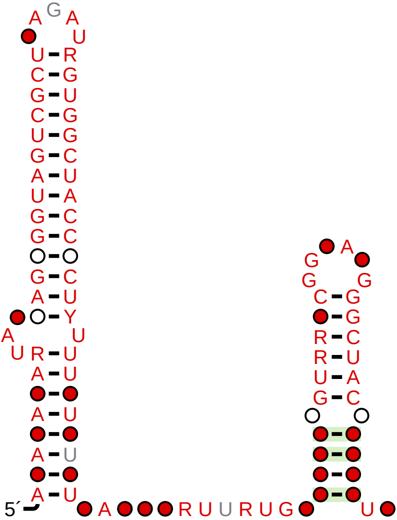

HMP2_11177_1

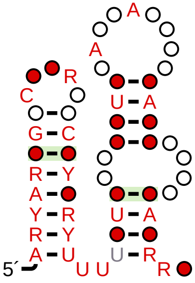

HMP2_11178_1

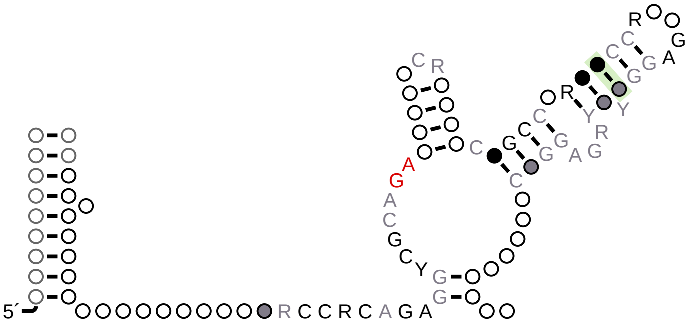

HMP2_11183_1

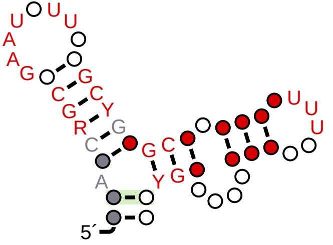

HMP2_11187_1

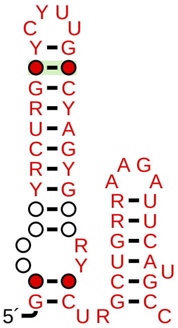

HMP2_11189_1

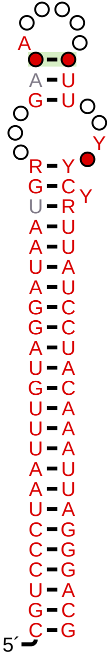

HMP2_11190_1

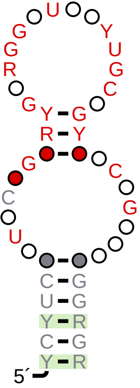

HMP2_11202_1

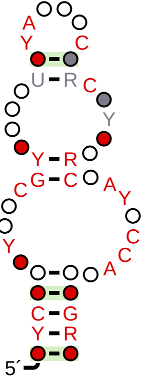

HMP2_11204_1

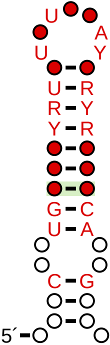

HMP2_11205_1

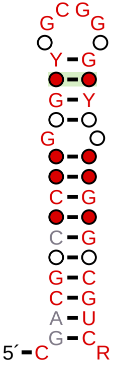

HMP2_11212_1

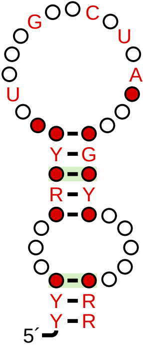

HMP2_11224_1

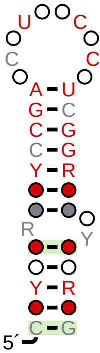

HMP2_1123_1

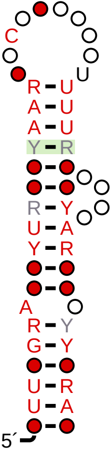

HMP2_11238_1

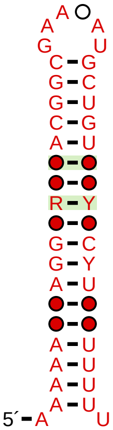

HMP2_11246_1

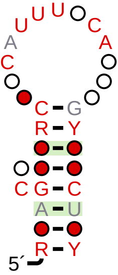

HMP2_11254_1

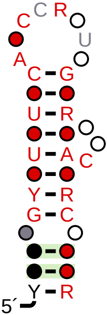

HMP2_11264_1

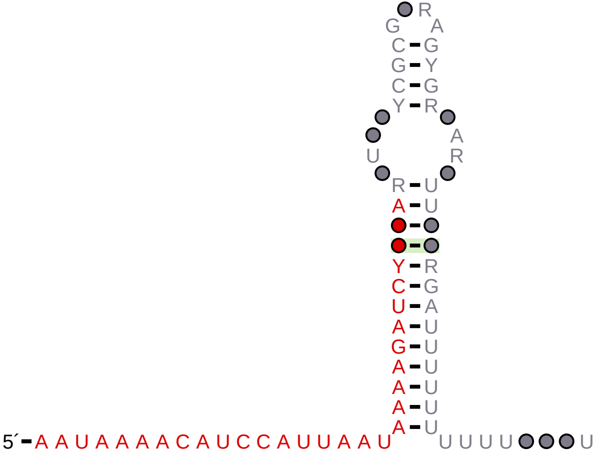

HMP2_11267_1

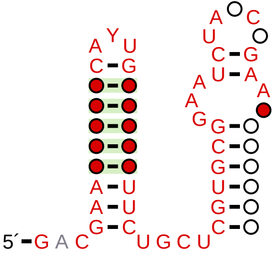

HMP2_1127_1

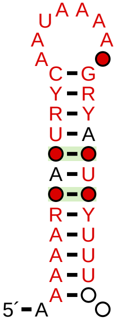

HMP2_11275_1

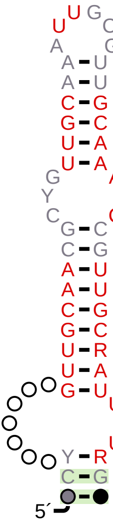

HMP2_11279_1

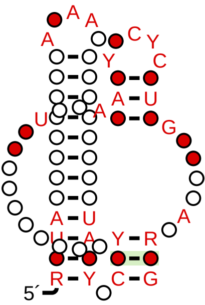

HMP2_11280_1

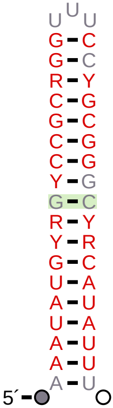

HMP2_11281_1

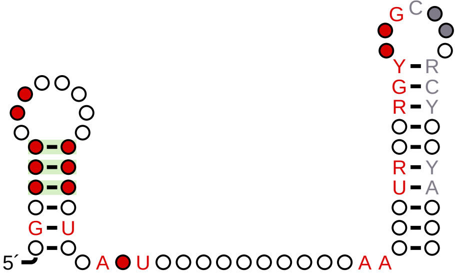

HMP2_11286_1

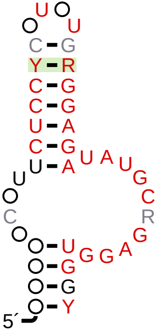

HMP2_11294_1

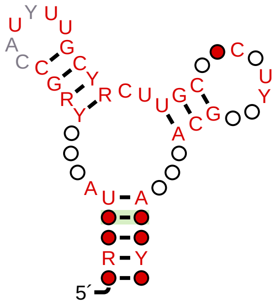

HMP2_1130_1

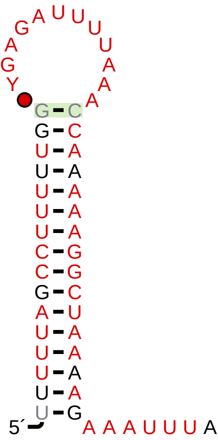

5´
G A U U U U A A A Y G A
G C C A A A A G G C U A A A A G
U U U U C C G A U U U U U G G
A A A U U U A

HMP2_11306_1

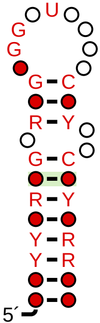

HMP2_11308_1

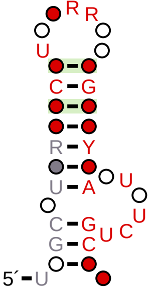

HMP2_11312_1

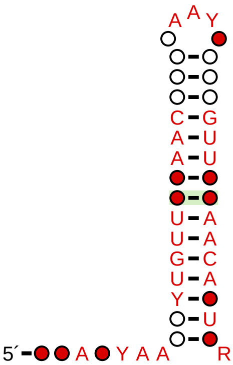

HMP2_11313_1

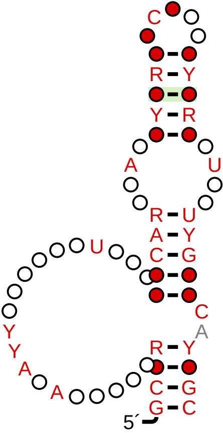

HMP2_11314_1

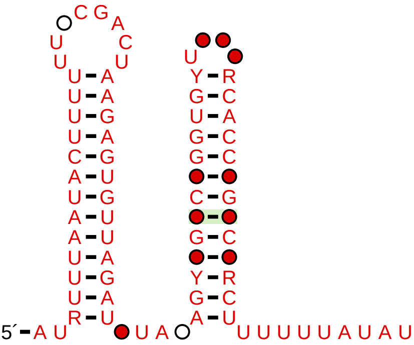

HMP2_11315_1

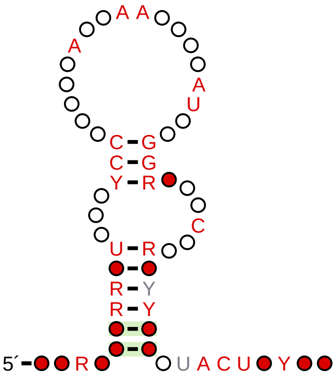

HMP2_11323_1

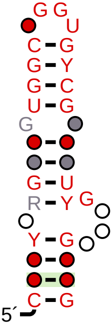

HMP2_11327_1

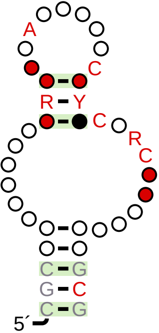

HMP2_11341_1

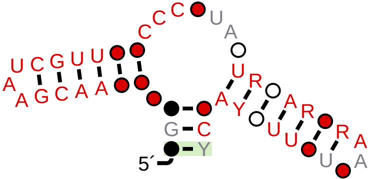

HMP2_11345_1

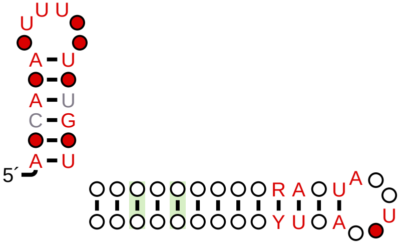

HMP2_11347_1

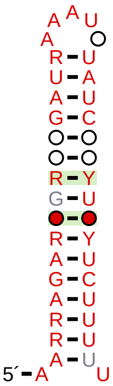

HMP2_11349_1

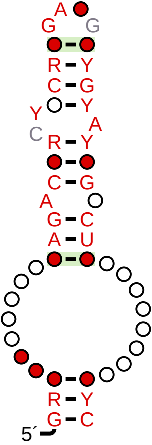

HMP2_11357_1

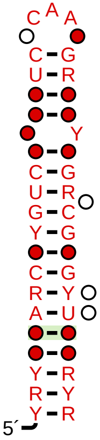

HMP2_11365_1

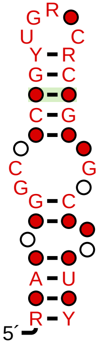

HMP2_11368_1

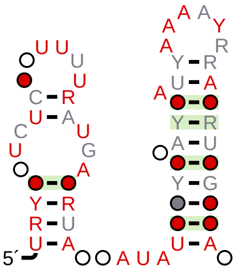

HMP2_11371_1

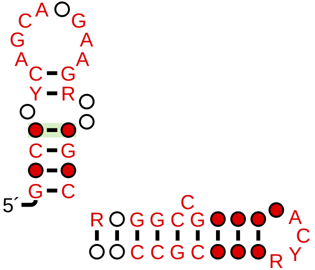

HMP2_11382_1

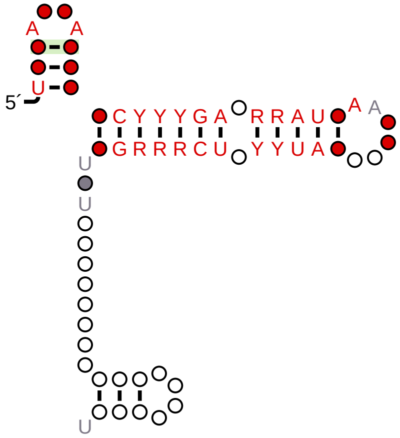

HMP2_1139_1

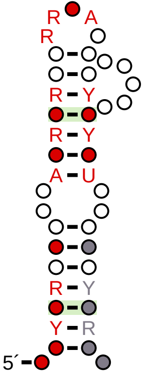

HMP2_11392_1

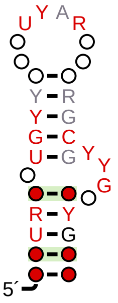

HMP2_11397_1

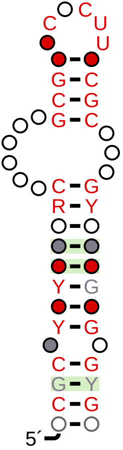

HMP2_11402_1

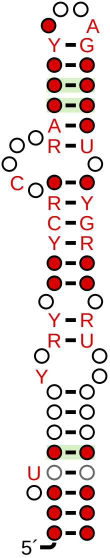

HMP2_11412_1

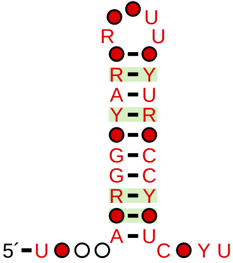

HMP2_11419_1

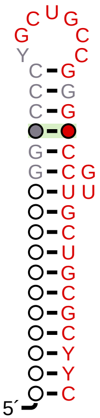

HMP2_11427_1

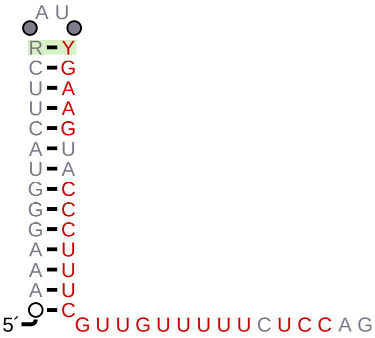

HMP2_11438_1

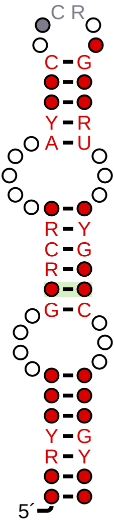

HMP2_1144_1

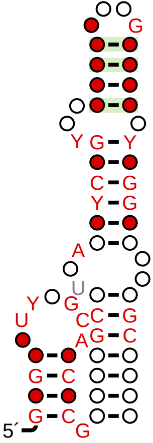

HMP2_11442_1

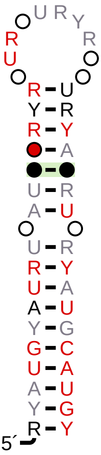

HMP2_11450_1

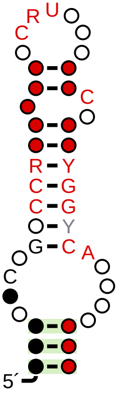

HMP2_11473_1

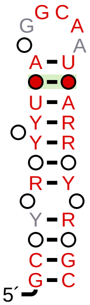

HMP2_1148_1

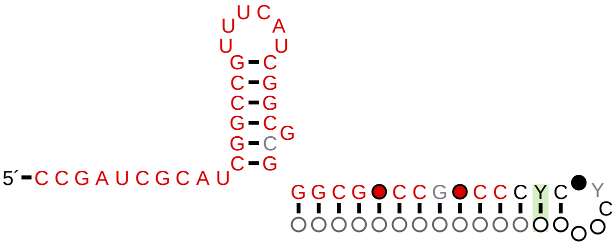

HMP2_11481_1

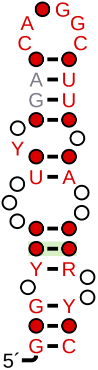

HMP2_11484_1

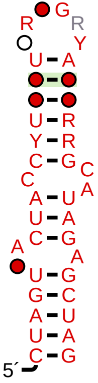

HMP2_11500_1

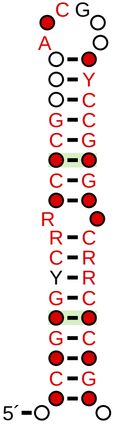

HMP2_11501_1

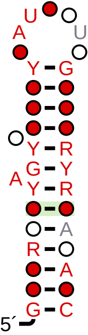

HMP2_11504_1

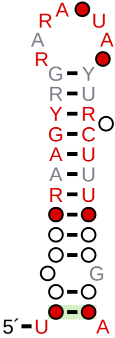

HMP2_11516_1

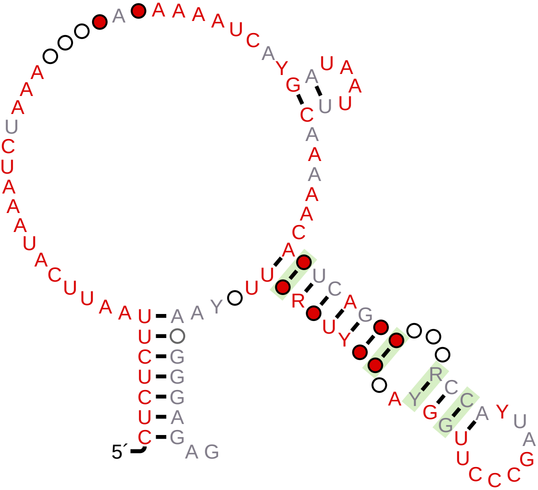

HMP2_1152_1

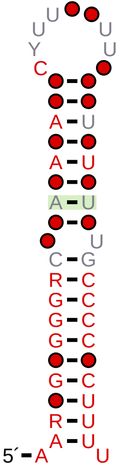

HMP2_11538_1

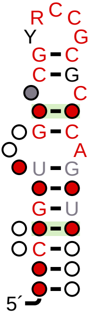

HMP2_1154_1

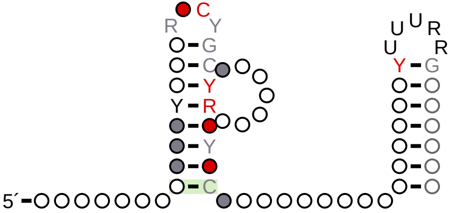

HMP2_11543_1

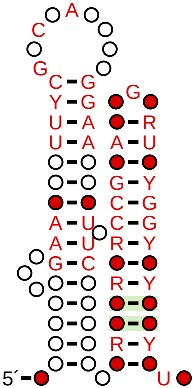

HMP2_11556_1

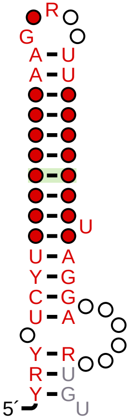

HMP2_11561_1

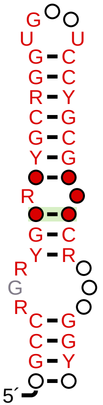

HMP2_11563_1

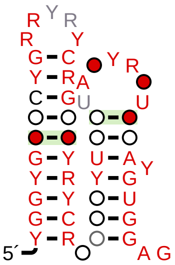

HMP2_11566_1

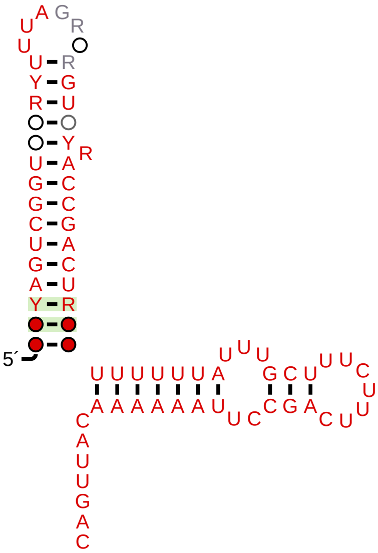

HMP2_11569_1

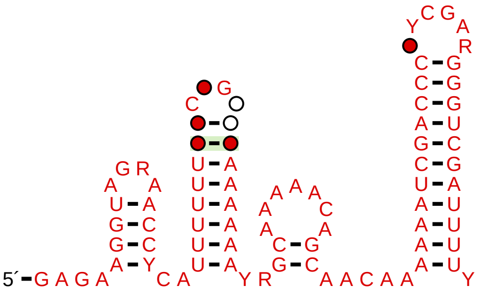

HMP2_1157_1

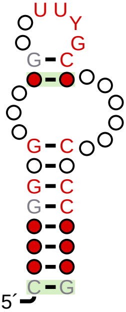

HMP2_11576_1

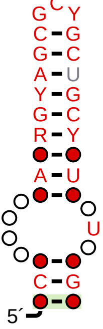

HMP2_11577_1

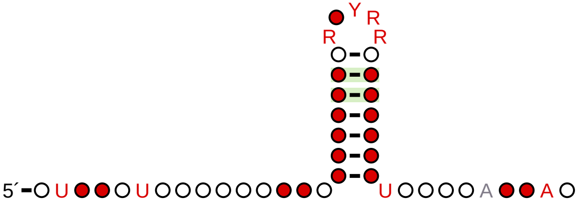

HMP2_11585_1

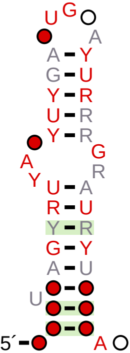

HMP2_11598_1

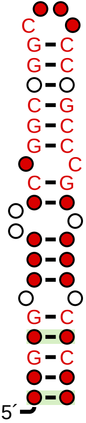

HMP2_116_1

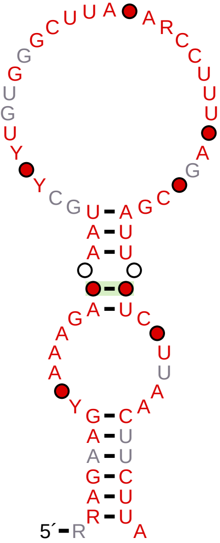

HMP2_11602_1

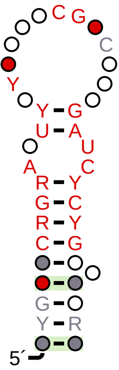

HMP2_11606_1

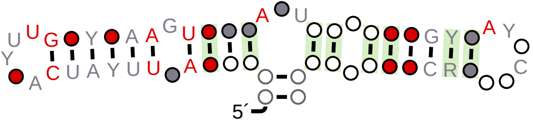

HMP2_11607_1

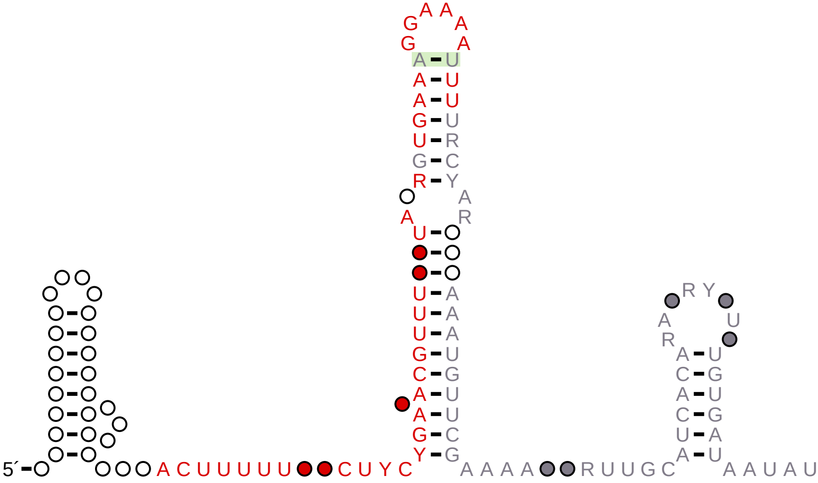

HMP2_11613_1

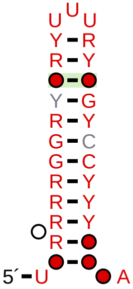

HMP2_11634_1

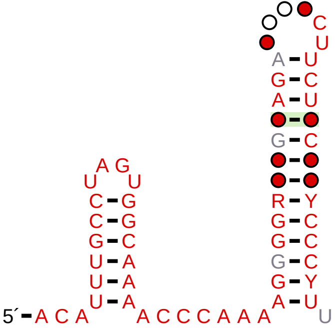

HMP2_11643_1

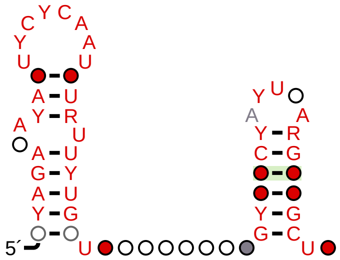

HMP2_11656_1

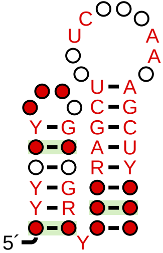

HMP2_1166_1

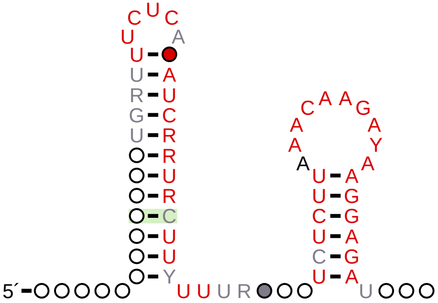

HMP2_11662_1

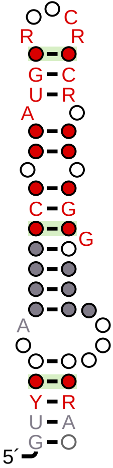

HMP2_11665_1

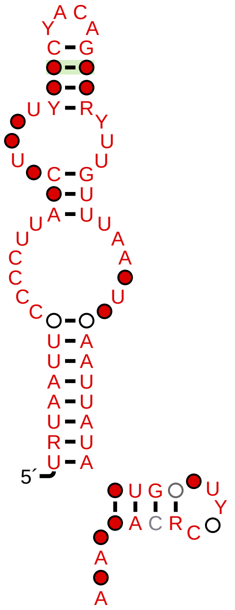

HMP2_11672_1

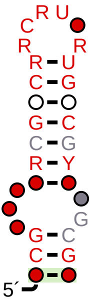

HMP2_11683_1

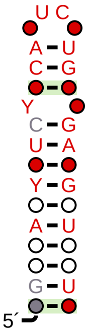

HMP2_11701_1

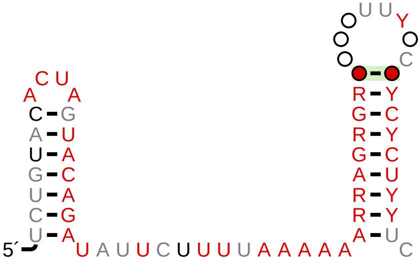

HMP2_11709_1

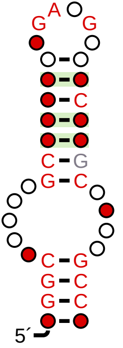

HMP2_11714_1

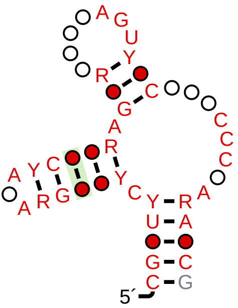

HMP2_11715_1

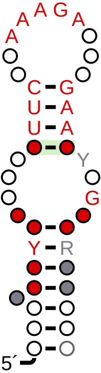

HMP2_1172_1

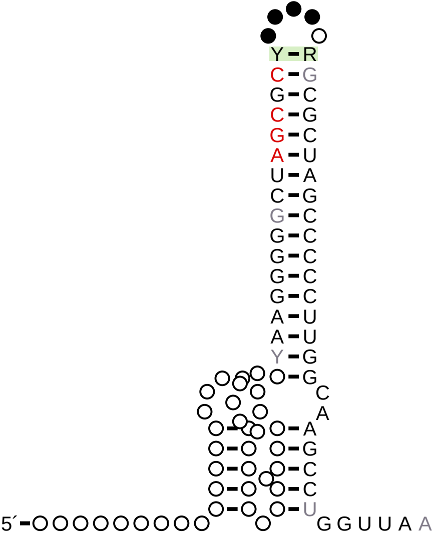

HMP2_11720_1

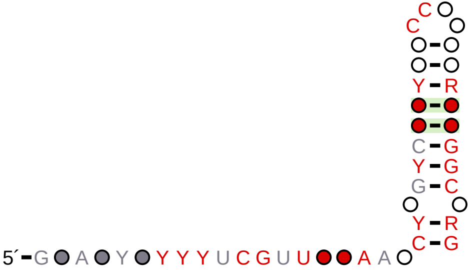

HMP2_11724_1

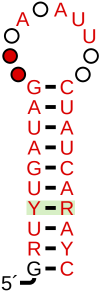

HMP2_11732_1

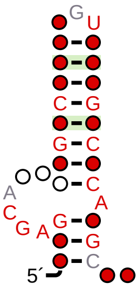

HMP2_11735_1

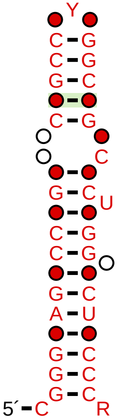

HMP2_11741_1

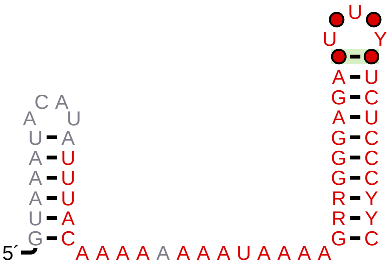

5´
U
U Y
A U
G C
A U
G C
G C
G C
R Y
R Y
G C
C A
A U
U A
A U
A U
A U
U A
G C
A A A A A A A A U A A A A

HMP2_1175_1

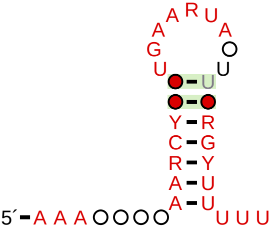

HMP2_11759_1

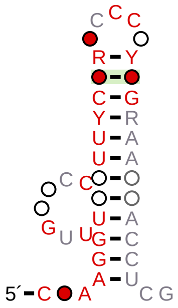

HMP2_1176_1

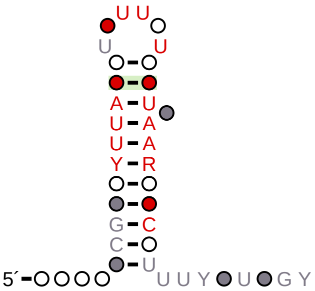

HMP2_11760_1

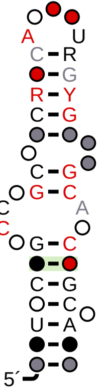

HMP2_11763_1

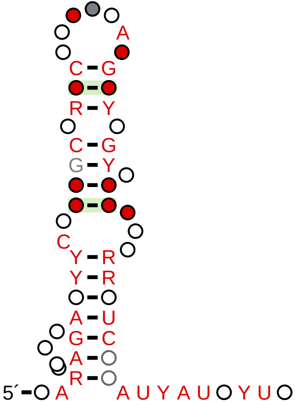

HMP2_11770_1

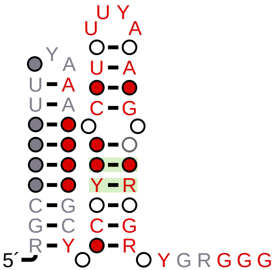

HMP2_11771_1

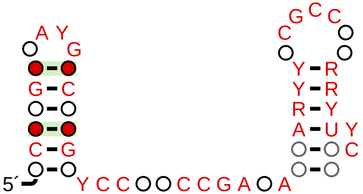

HMP2_11780_1

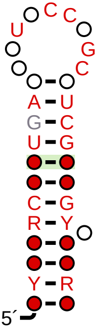

HMP2_11784_1

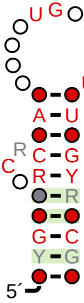

HMP2_11794_1

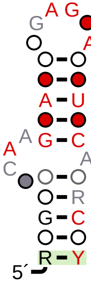

HMP2_1180_1

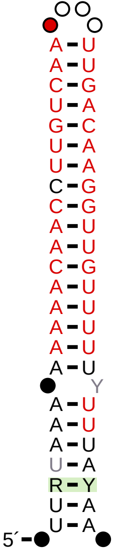

HMP2_11803_1

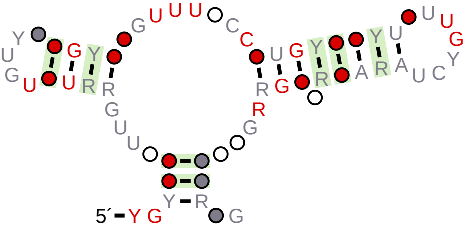

HMP2_11826_1

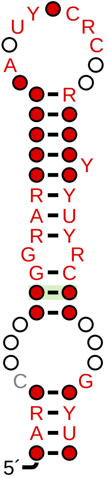

HMP2_11830_1

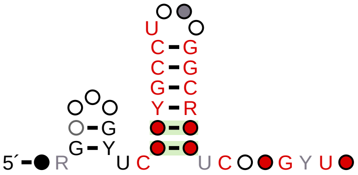

HMP2_11832_1

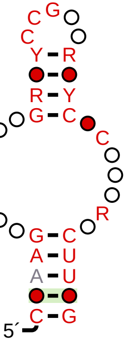

HMP2_11836_1

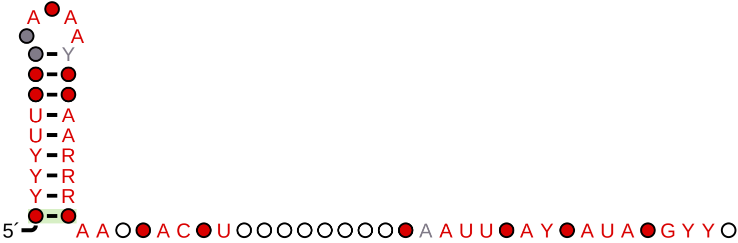

HMP2_11838_1

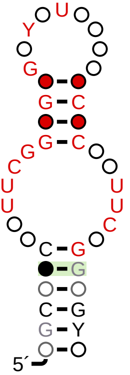

HMP2_1184_1

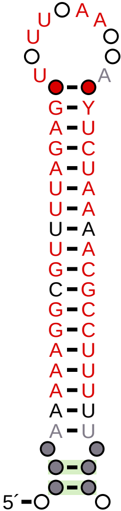

HMP2_11845_1

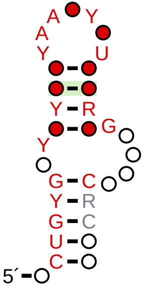

HMP2_11850_1

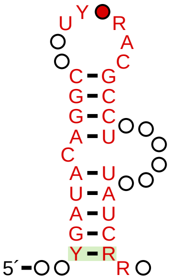

HMP2_11855_1

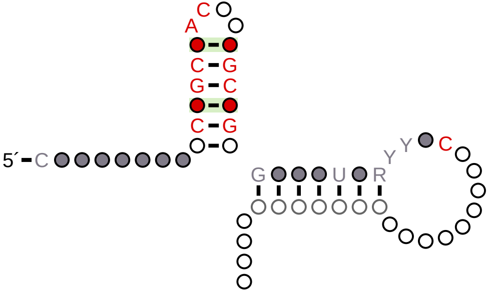

HMP2_11857_1

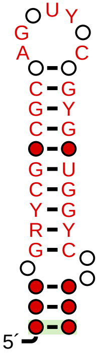

HMP2_11858_1

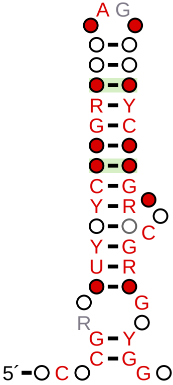

HMP2_1186_1

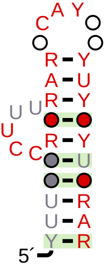

HMP2_11872_1

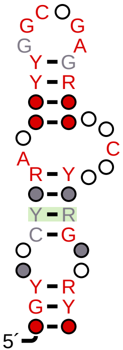

HMP2_11874_1

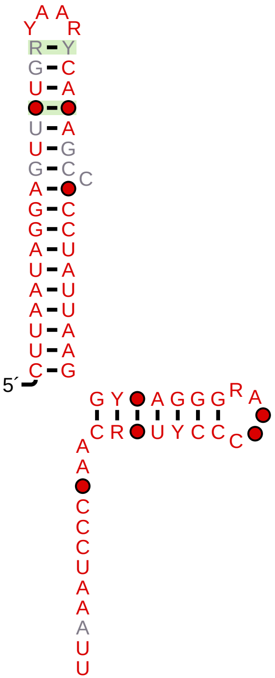

HMP2_11879_1

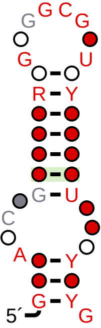

HMP2_11891_1

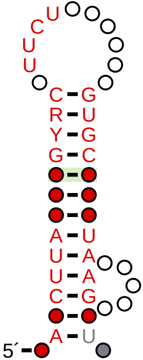

HMP2_11897_1

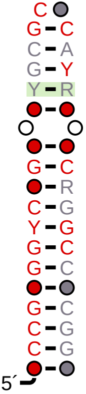

HMP2_11899_1

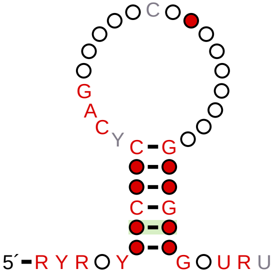

HMP2_11900_1

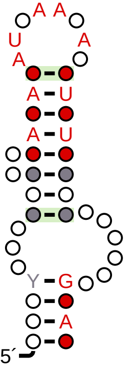

HMP2_11908_1

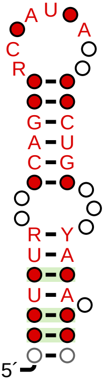

HMP2_11909_1

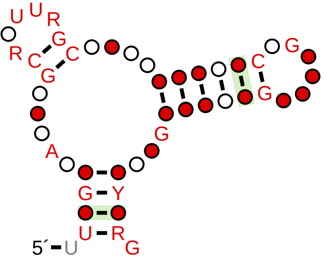

HMP2_11917_1

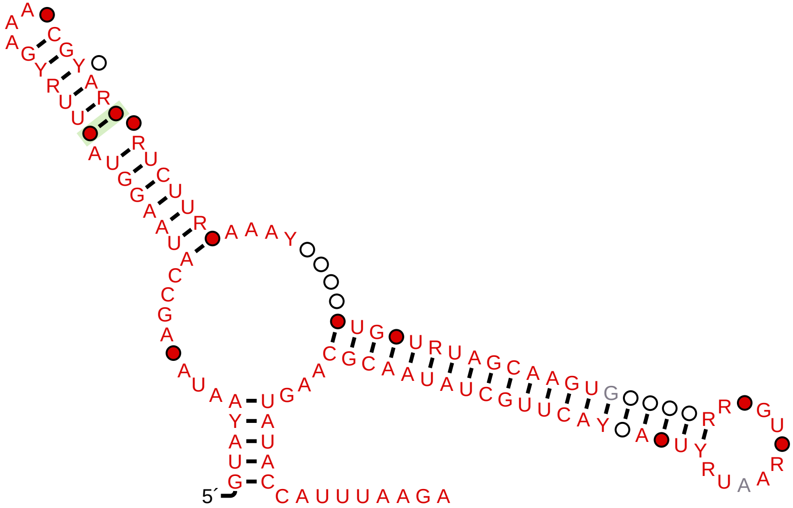

HMP2_11928_1

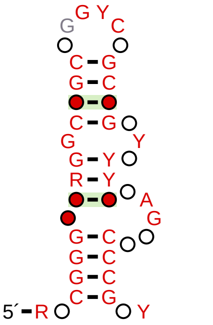

HMP2_11931_1

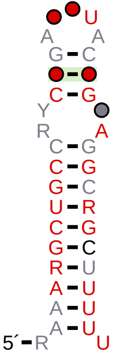

HMP2_11935_1

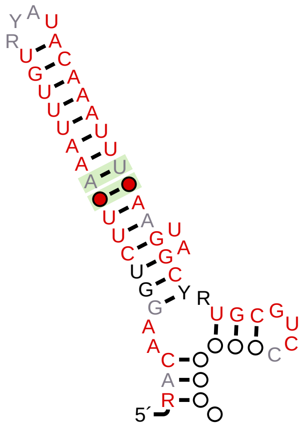

HMP2_11936_1

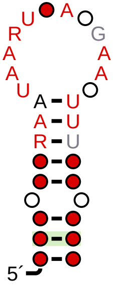

HMP2_11938_1

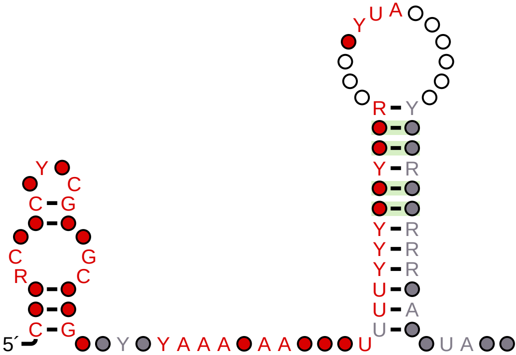

HMP2_1194_1

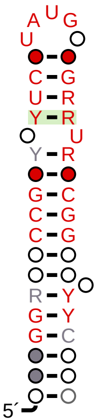

HMP2_1195_1

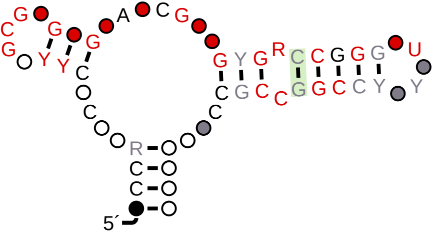

HMP2_11953_1

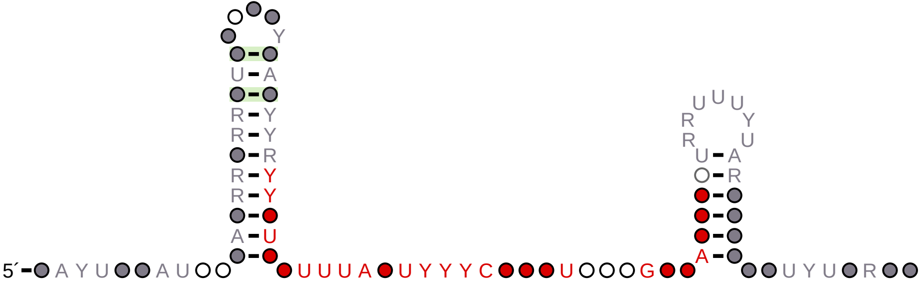

HMP2_1196_1

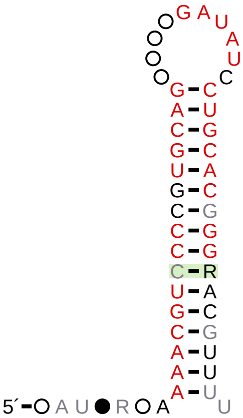

HMP2_11961_1

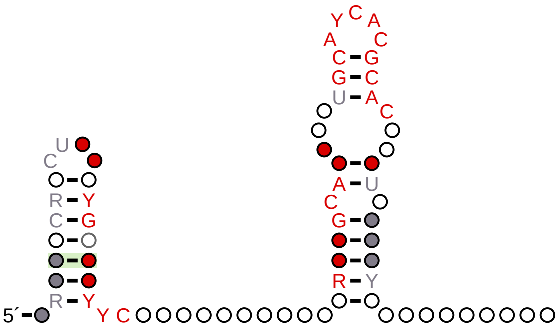

HMP2_11969_1

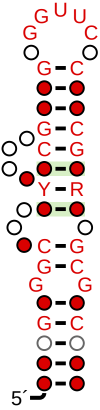

HMP2_11973_1

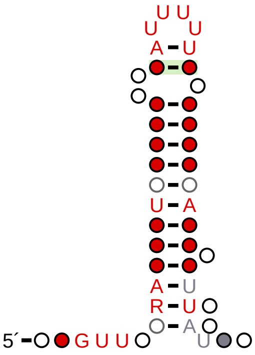

HMP2_11979_1

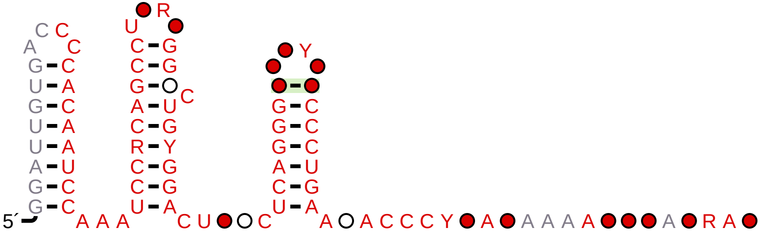

HMP2_11983_1

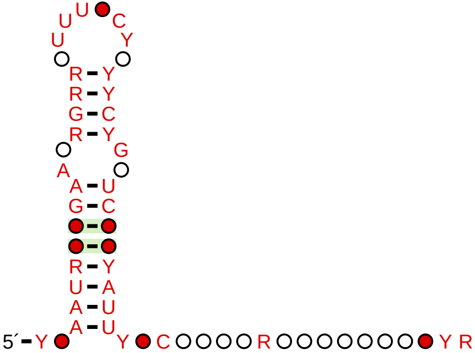

HMP2_11986_1

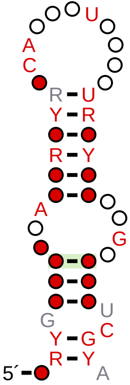

HMP2_120_1

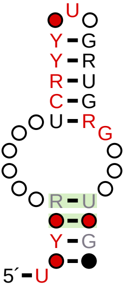

HMP2_12004_1

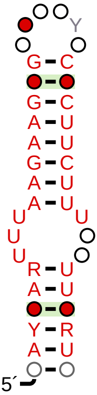

HMP2_12006_1

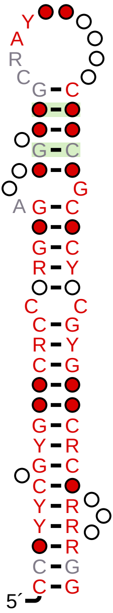

HMP2_12007_1

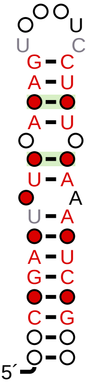

HMP2_12012_1

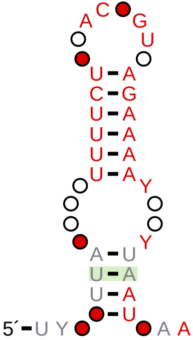

HMP2_12022_1

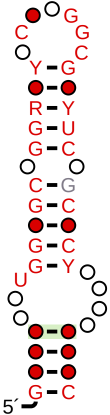

HMP2_12029_1

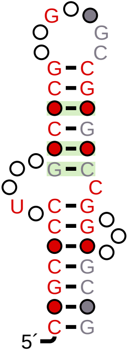

HMP2_1203_1

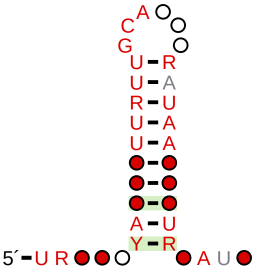

HMP2_12043_1

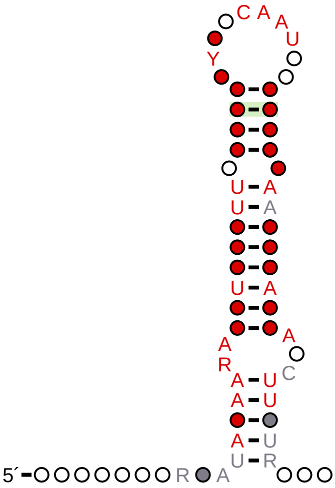

HMP2_12048_1

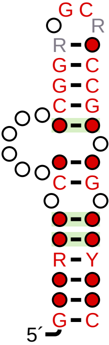

HMP2_12049_1

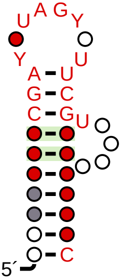

HMP2_1206_1

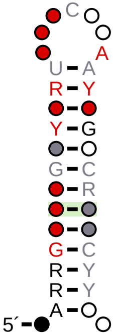

HMP2_12062_1

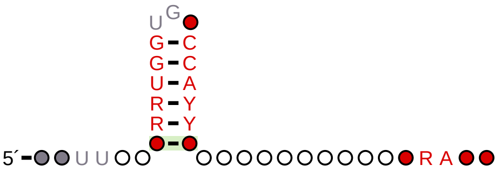

HMP2_1207_1

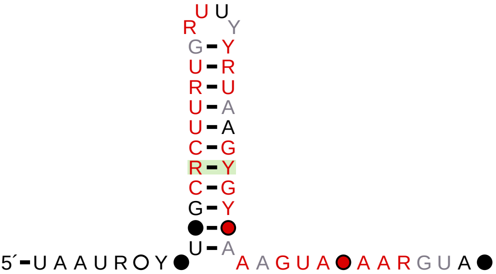

HMP2_12074_1

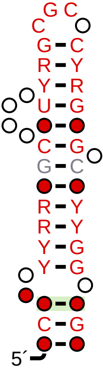

HMP2_12080_1

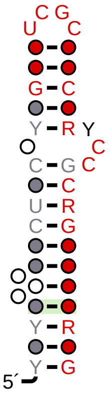

HMP2_12090_1

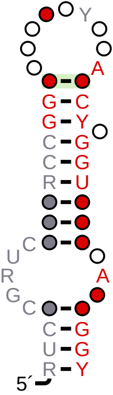

HMP2_12100_1

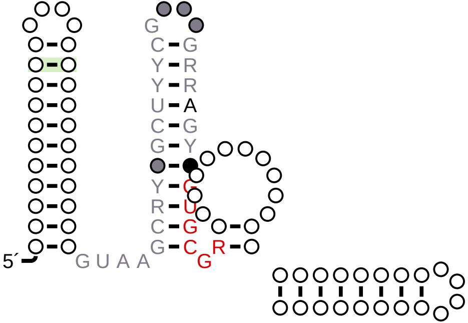

HMP2_12103_1

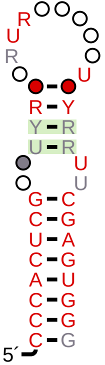

HMP2_12108_1

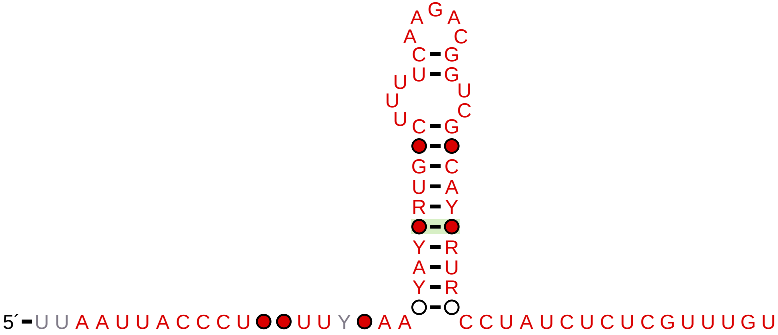
5´
U U A A U U A C C C U
U U Y
A A
C C U A U C U C U C G U U U G U
A G A
A C
C G
U G
U U U
U C
C G
G C
U A
R Y
Y R
A U
Y R

HMP2_1211_1

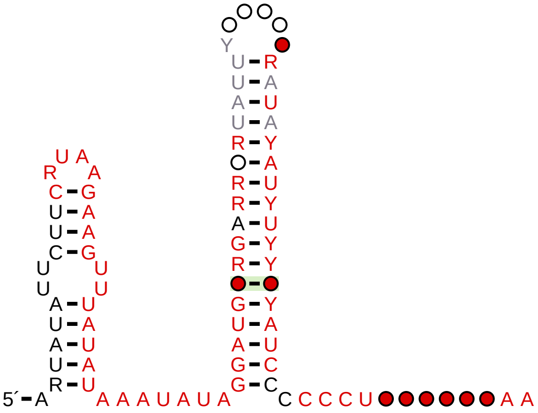

HMP2_12114_1

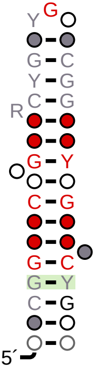

HMP2_12122_1

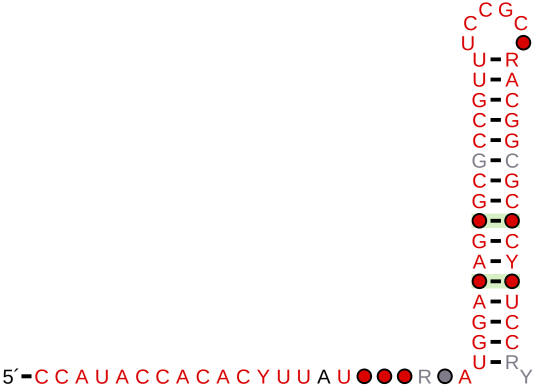

HMP2_12123_1

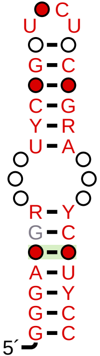

HMP2_12137_1

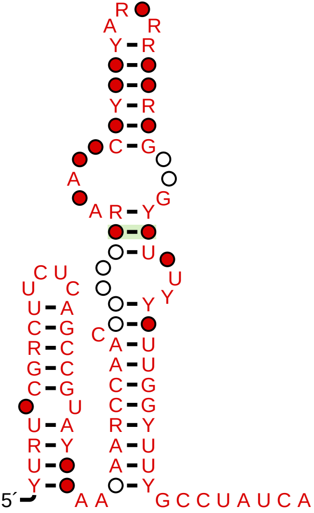

HMP2_12152_1

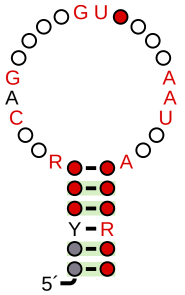

HMP2_12153_1

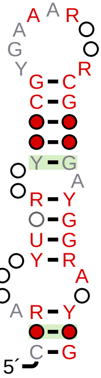

HMP2_12161_1

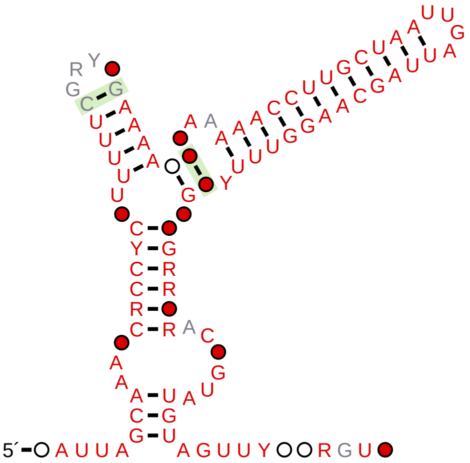

HMP2_12164_1

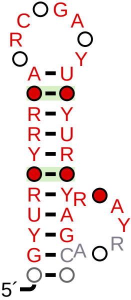

HMP2_1217_1

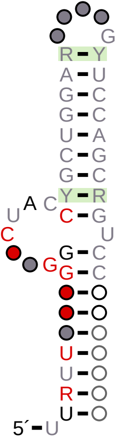

HMP2_12177_1

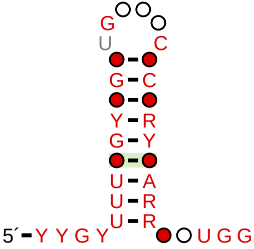

HMP2_12190_1

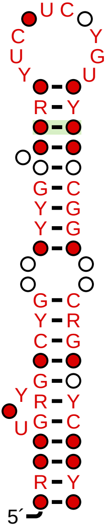

HMP2_12193_1

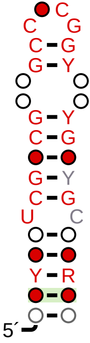

HMP2_122_1

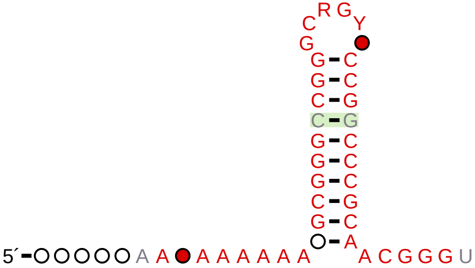

HMP2_12203_1

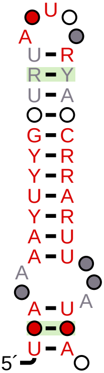

HMP2_12235_1

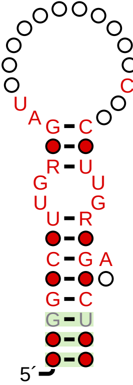

HMP2_12236_1

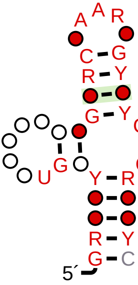

HMP2_12247_1

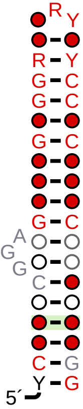

HMP2_12255_1

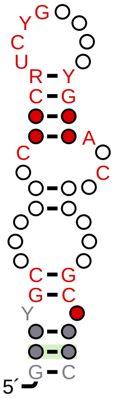

HMP2_12263_1

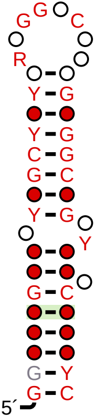

HMP2_12270_1

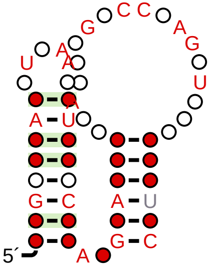

HMP2_12274_1

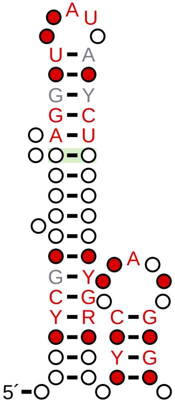

HMP2_12277_1

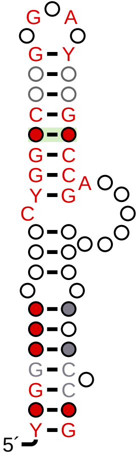

HMP2_12288_1

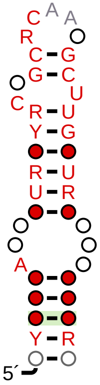

HMP2_12294_1

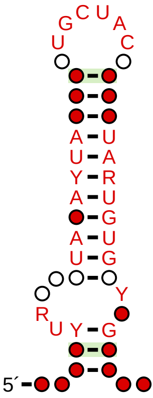

HMP2_12297_1

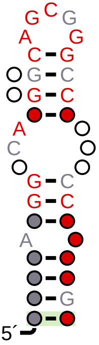

HMP2_12309_1

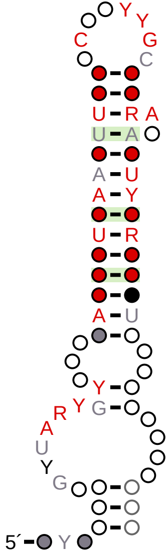

HMP2_1231_1

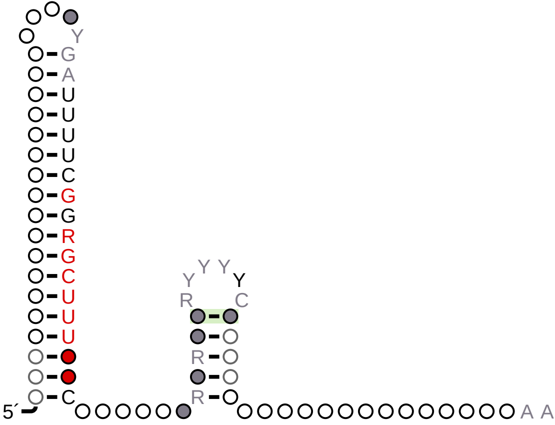

HMP2_12320_1

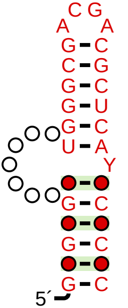

HMP2_12321_1

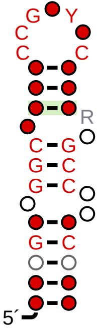

HMP2_12338_1

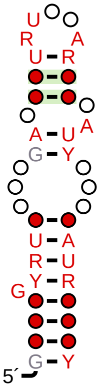

HMP2_1236_1

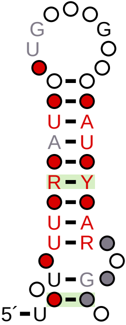

HMP2_12362_1

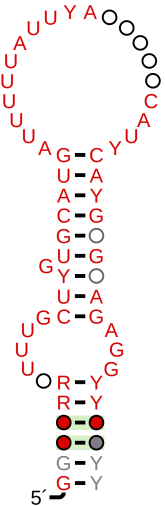

HMP2_12375_1

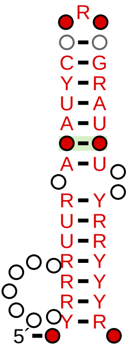

HMP2_12381_1

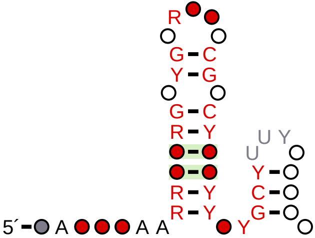

HMP2_12385_1

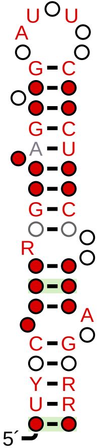

HMP2_12389_1

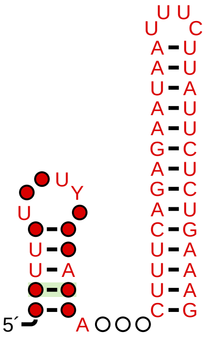

HMP2_1241_1

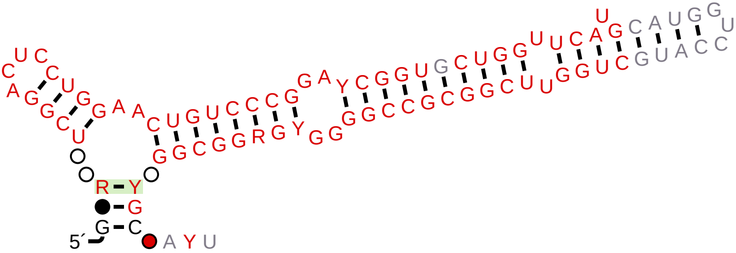

HMP2_12429_1

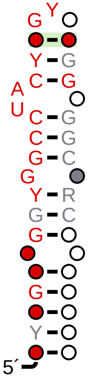

HMP2_12443_1

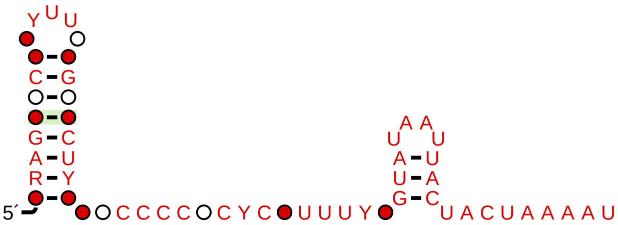

HMP2_12449_1

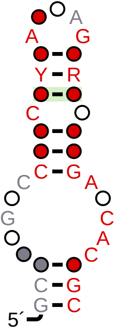

HMP2_1245_1

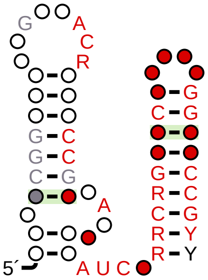

HMP2_12451_1

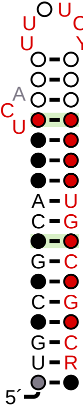

HMP2_12483_1

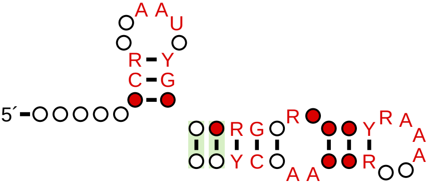

HMP2_12512_1

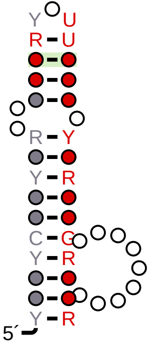

HMP2_12528_1

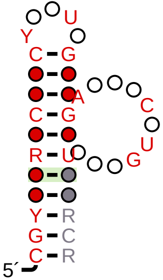

HMP2_12542_1

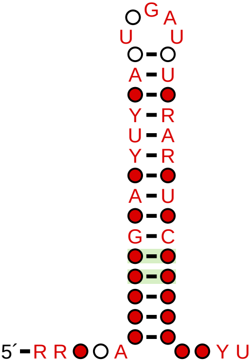

HMP2_12544_1

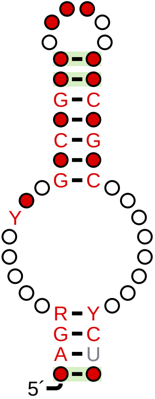

HMP2_12556_1

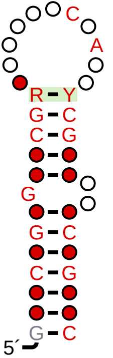

HMP2_12562_1

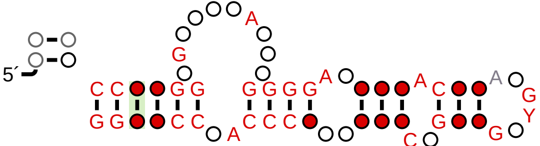

HMP2_12575_1

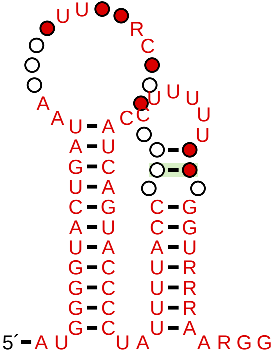

HMP2_1258_1

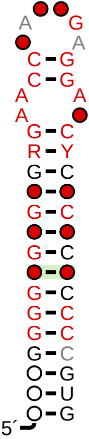

HMP2_12581_1

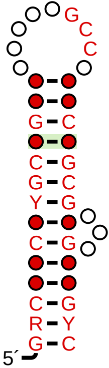

HMP2_12584_1

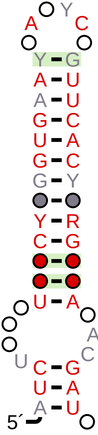

HMP2_126_1

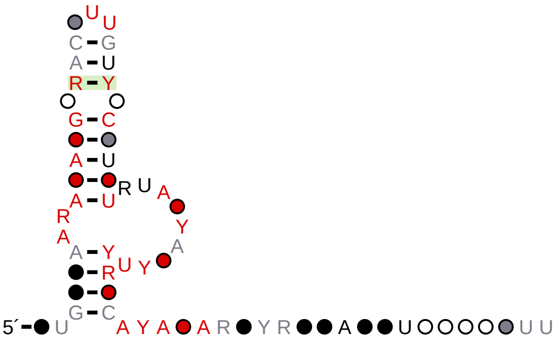

HMP2_12612_1

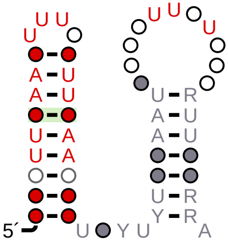

HMP2_12620_1

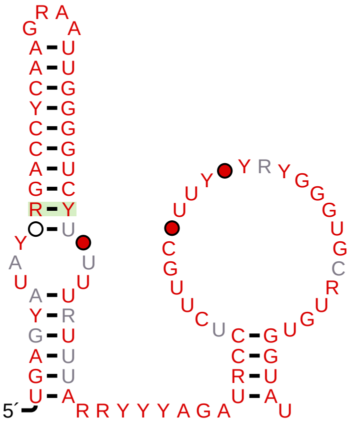

HMP2_12622_1

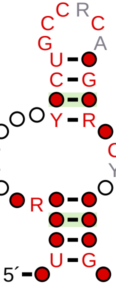

HMP2_12626_1

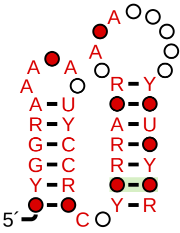

HMP2_12628_1

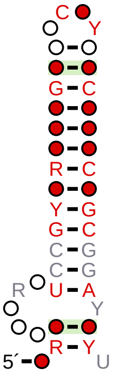

HMP2_12642_1

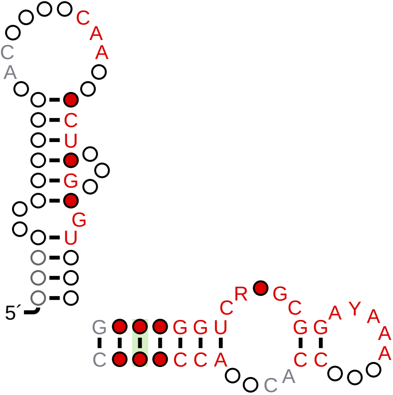

HMP2_12646_1

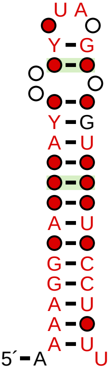

HMP2_1266_1

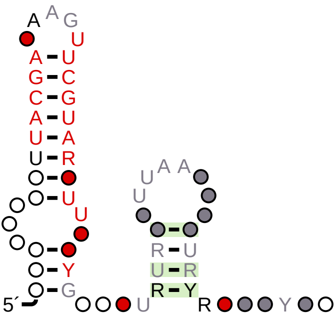

HMP2_12668_1

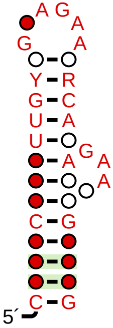

HMP2_12670_1

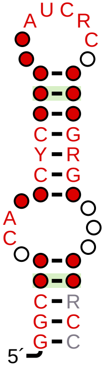

HMP2_12699_1

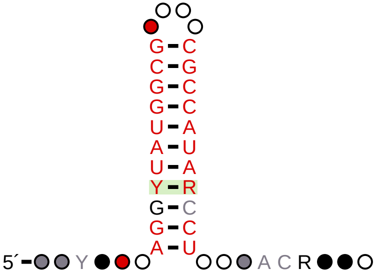

HMP2_12701_1

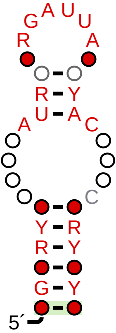

HMP2_12731_1

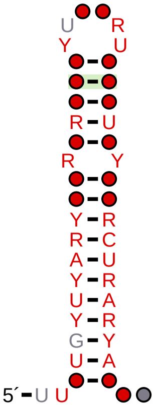

HMP2_12733_1

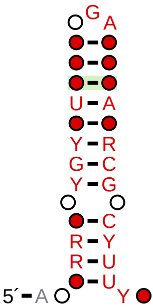

HMP2_12747_1

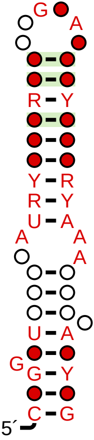

HMP2_12765_1

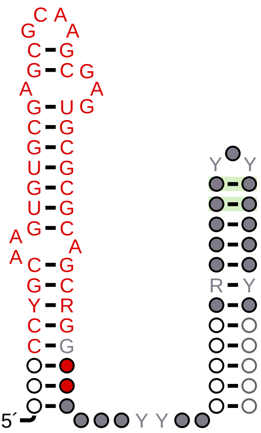

HMP2_1277_1

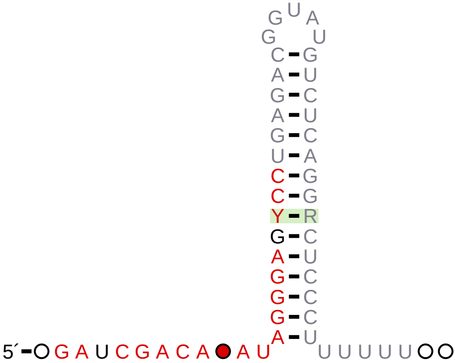
5´
G A U C G A C A A U
U U U U U
G U A
G U
C G
A U
G C
A U
G C
U A
C G
C G
Y R
G C
A U
G C
G C
G C
A U

HMP2_12771_1

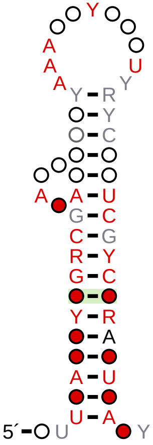

HMP2_12773_1

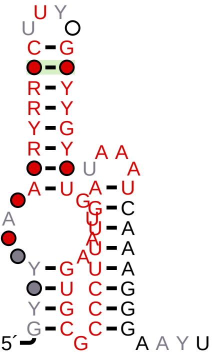

HMP2_12798_1

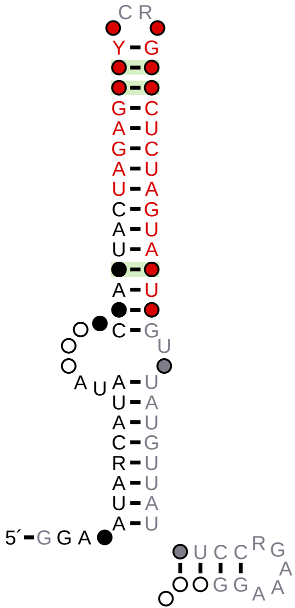

C R
Y G
G C
A U
G C
A U
U A
C G
A U
U A
A U
C G
U
A U A U
U A
A U
C G
R U
A U
U A
A U
5´ G G A
U C C R G
A
G G A A

HMP2_12808_1

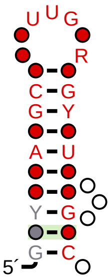

HMP2_12832_1

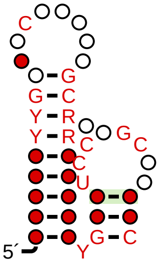

HMP2_1285_1

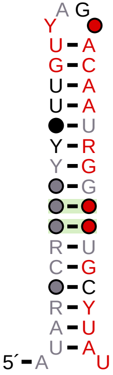

HMP2_12856_1

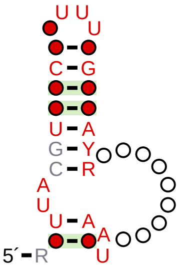

HMP2_12859_1

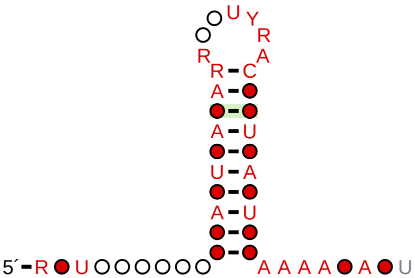

HMP2_12877_1

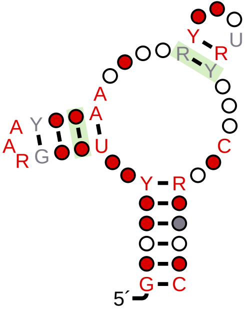

HMP2_12891_1

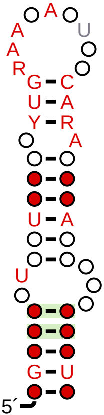

HMP2_12895_1

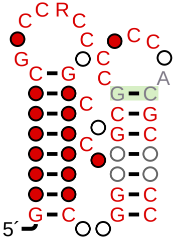

HMP2_12899_1

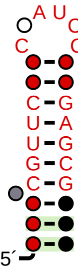

HMP2_12905_1

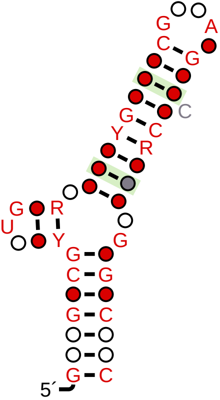

HMP2_12907_1

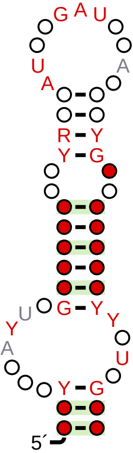

HMP2_1291_1

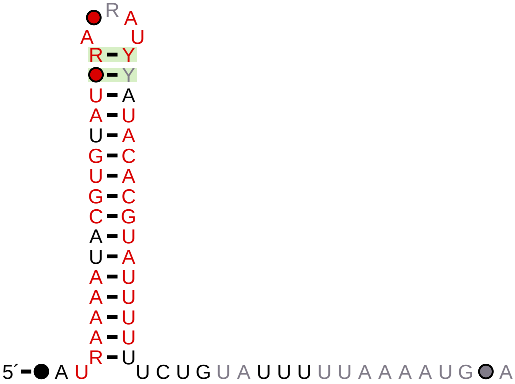
R
A A
A U
R Y
Y
U A
A U
U A
G C
U A
G C
C G
A U
U A
A U
A U
A U
A U
R U
5´ A U U C U G U A U U U U U A A A A U G A

HMP2_12911_1

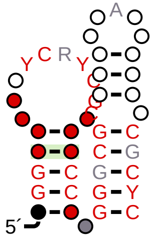

HMP2_12920_1

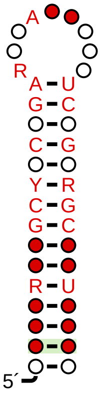

HMP2_12921_1

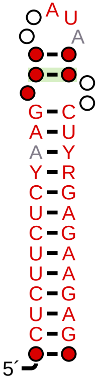

HMP2_12929_1

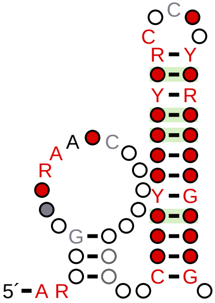

HMP2_12932_1

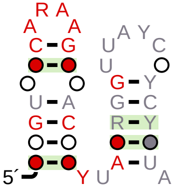

HMP2_12933_1

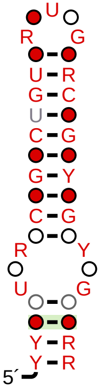

HMP2_12936_1

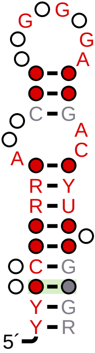

HMP2_12937_1

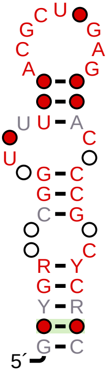

HMP2_12942_1

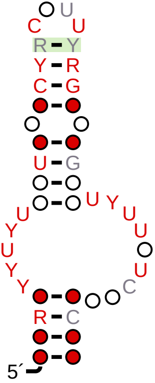

HMP2_12949_1

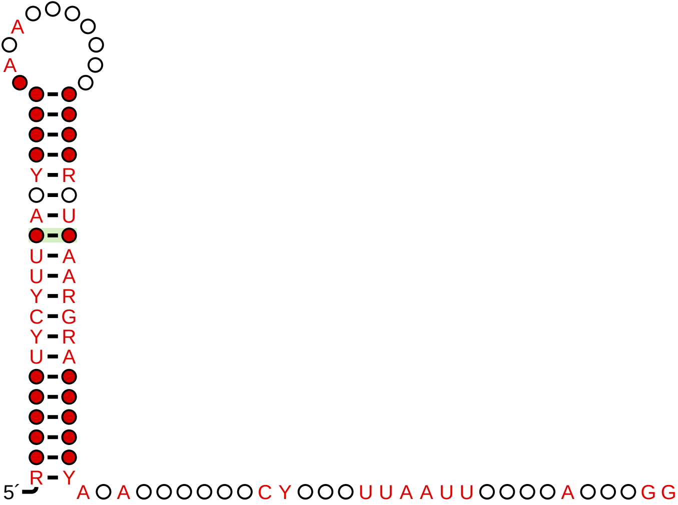

HMP2_12955_1

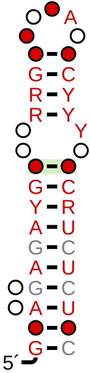

HMP2_12958_1

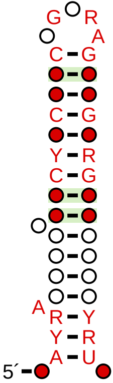

HMP2_12963_1

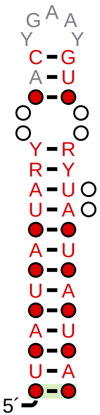

HMP2_12969_1

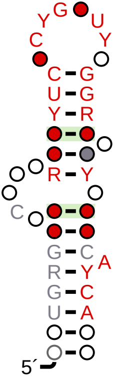

HMP2_1297_1

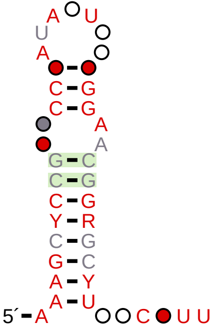

HMP2_1298_1

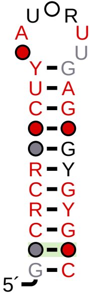

HMP2_1299_1

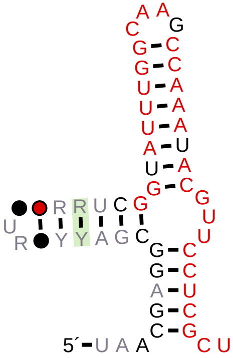

HMP2_12997_1

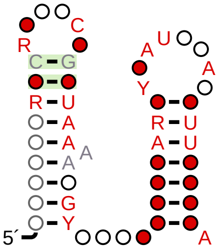

HMP2_13_1

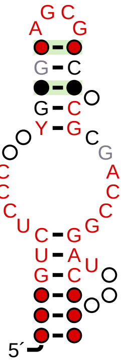

HMP2_13009_1

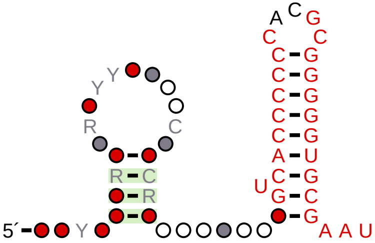

HMP2_1301_1

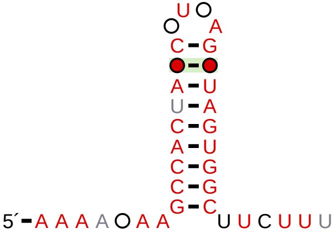

HMP2_1302_1

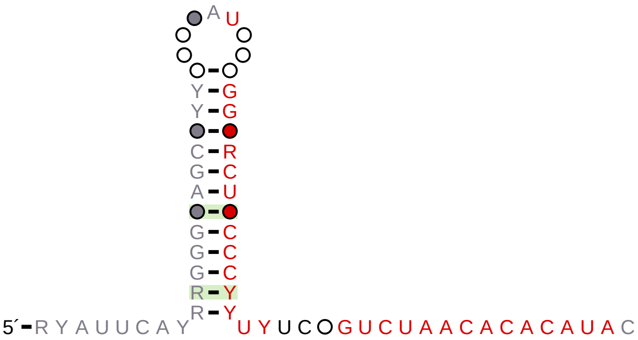
A
U
Y
G
Y
G
C
R
G
C
A
U
G
C
G
C
G
C
R
Y
R
Y
5´ R Y A U U C A Y
U Y U C
G U C U A A C A C A C A C A U A C

HMP2_13026_1

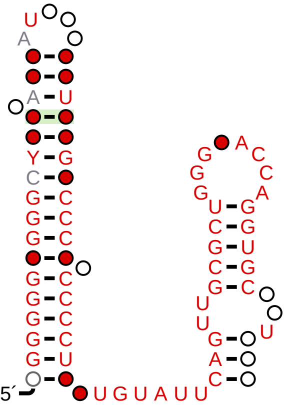

HMP2_13028_1

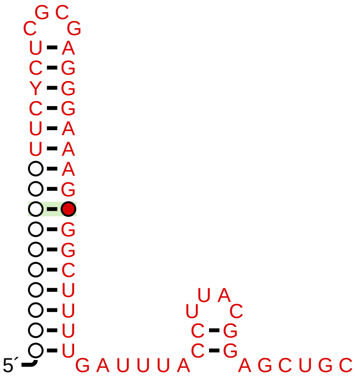

HMP2_13039_1

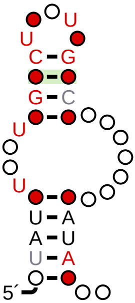

HMP2_13046_1

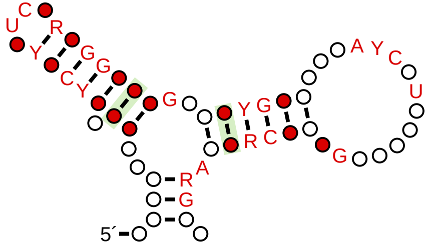

HMP2_13049_1

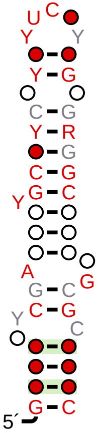

HMP2_1305_1

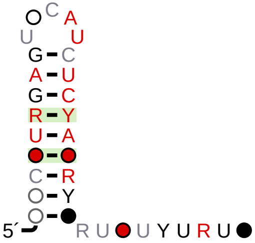

HMP2_13060_1

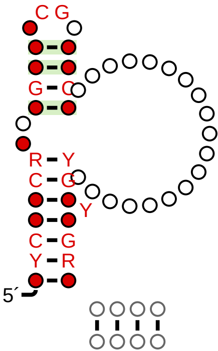

HMP2_13066_1

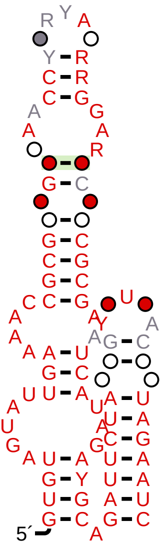

HMP2_13074_1

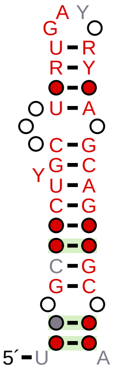

HMP2_13080_1

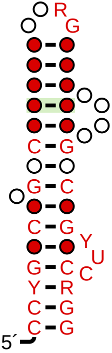

HMP2_13082_1

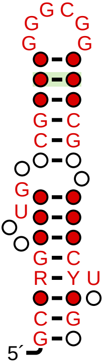

HMP2_1309_1

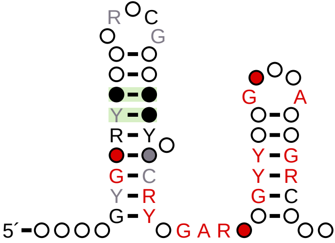

HMP2_13090_1

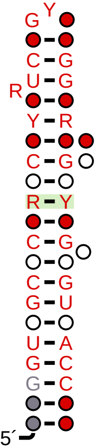

HMP2_13091_1

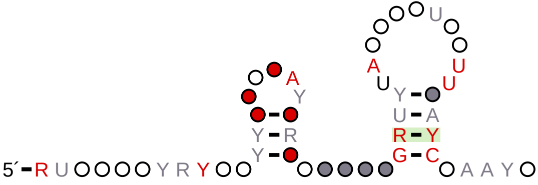

HMP2_13117_1

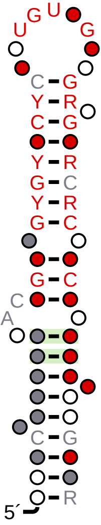

HMP2_1312_1

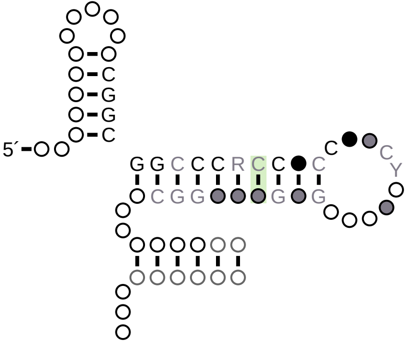

HMP2_13121_1

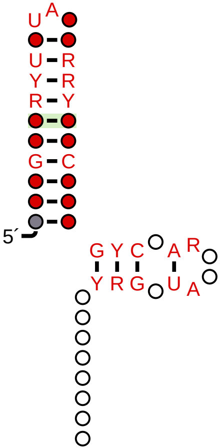

HMP2_13122_1

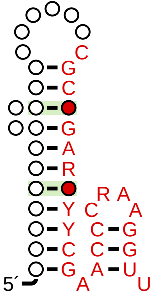

HMP2_13125_1

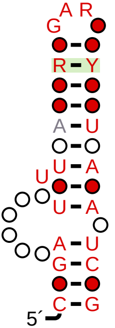

HMP2_13128_1

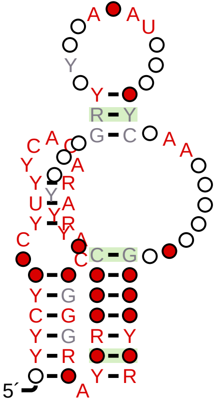

HMP2_1313_1

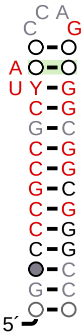

HMP2_13131_1

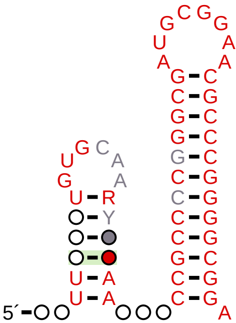

HMP2_13140_1

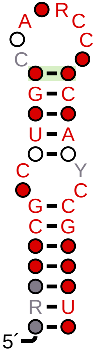

HMP2_13141_1

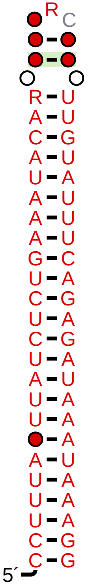

R
C
R U
A U
C G
A U
U A
A U
A U
A U
G C
U A
C G
U A
C G
U A
A U
U A
U A
A
A U
U A
U A
U A
C G
C G
5´

HMP2_13149_1

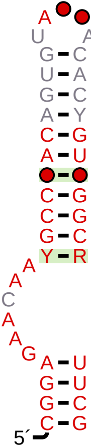

HMP2_13153_1

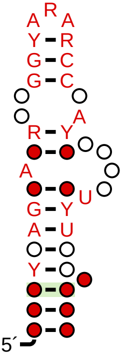

HMP2_13171_1

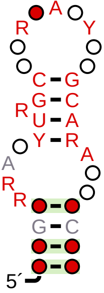

HMP2_13173_1

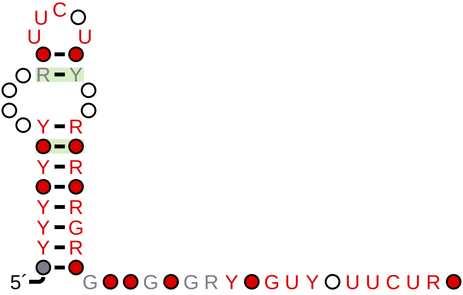

HMP2_13174_1

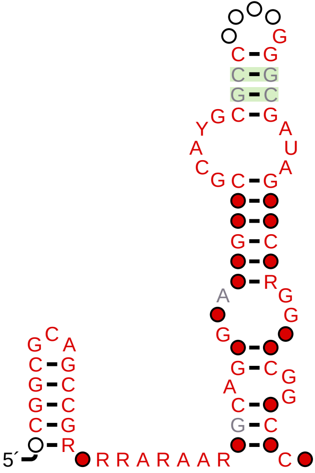

HMP2_13176_1

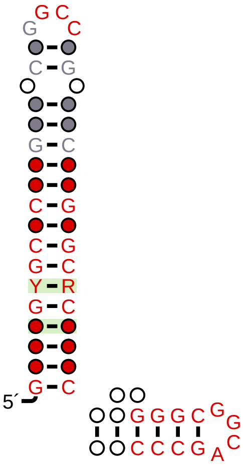

HMP2_13181_1

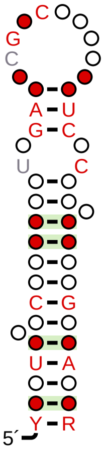

HMP2_13186_1

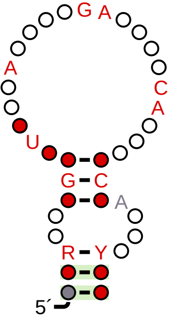

HMP2_13189_1

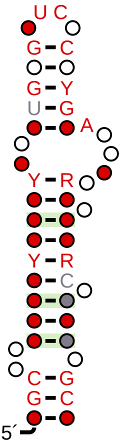

HMP2_13192_1

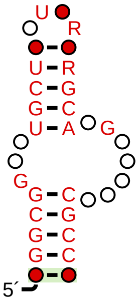

HMP2_132_1

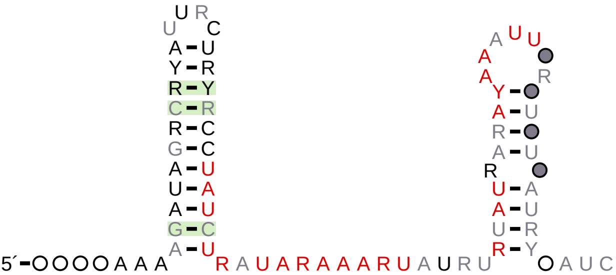

HMP2_1320_1

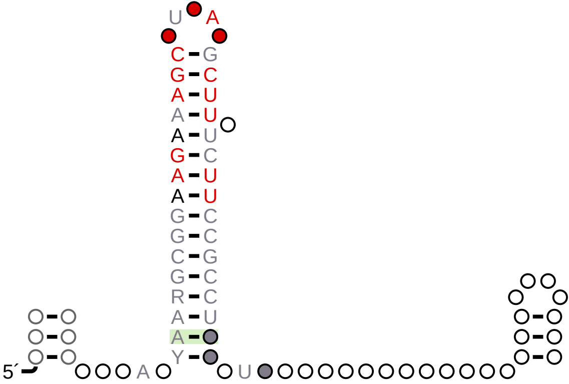

HMP2_13202_1

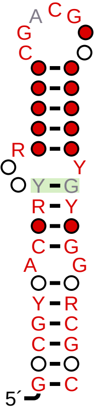

HMP2_13211_1

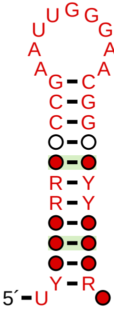

HMP2_1322_1

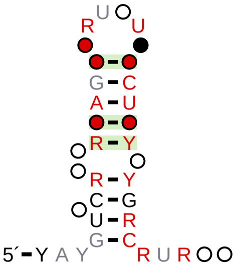

HMP2_13225_1

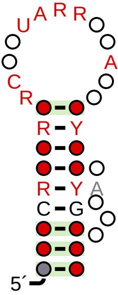

HMP2_13228_1

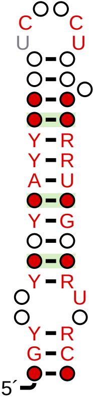

HMP2_13235_1

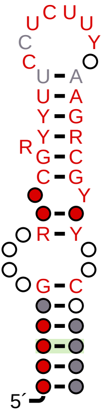

HMP2_1325_1

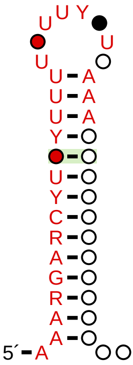

HMP2_13251_1

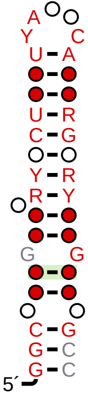

HMP2_13252_1

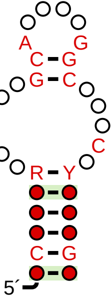

HMP2_1327_1

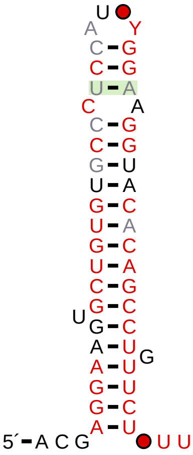
U
A Y
C G
C G
U A
C A
C G
C G
G U
U A
G C
U A
G C
U A
C G
U G C
G C
A U G
A U
G U
G C
A U
5´ A C G U U

HMP2_13271_1

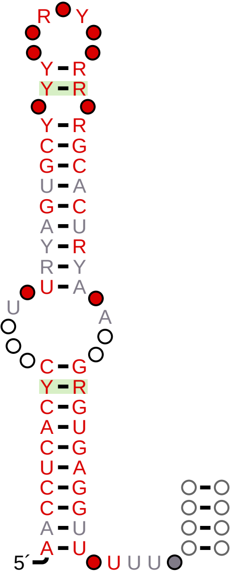

R Y
Y R
Y R
Y R
C G
G C
U A
G C
A U
Y R
R Y
U A
U
A
C G
Y R
C G
A U
C G
U A
C G
C G
A U
A U
5´
U U U

HMP2_13272_1

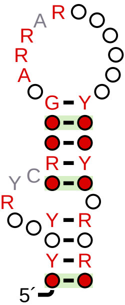

HMP2_13273_1

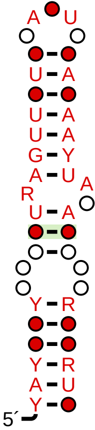

HMP2_13276_1

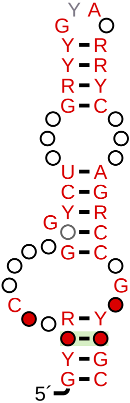

HMP2_13277_1

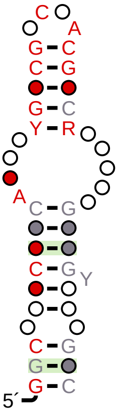

HMP2_13285_1

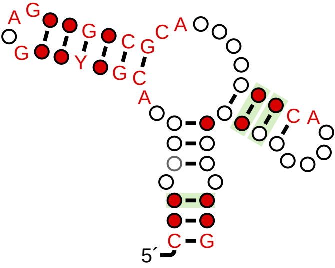

HMP2_13289_1

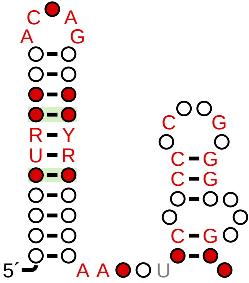

HMP2_13293_1

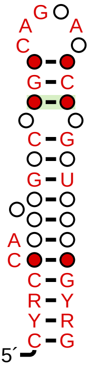

HMP2_13294_1

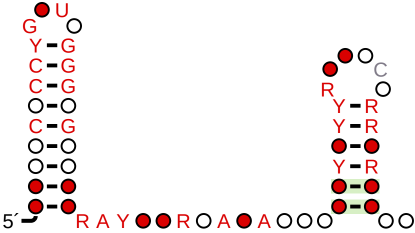

HMP2_13298_1

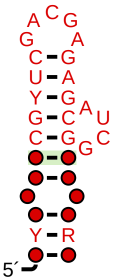

HMP2_1330_1

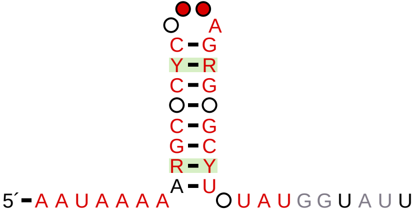

HMP2_13302_1

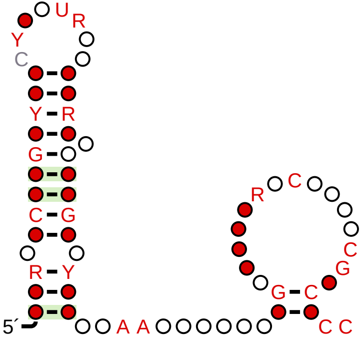

HMP2_13303_1

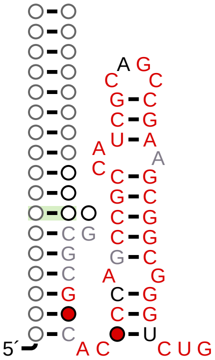

HMP2_13305_1

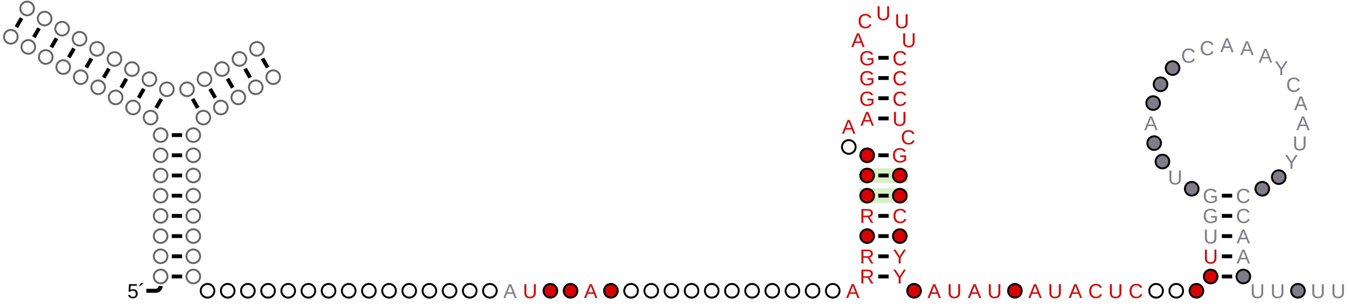

HMP2_13306_1

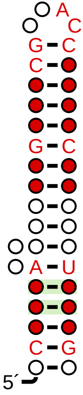

HMP2_13308_1

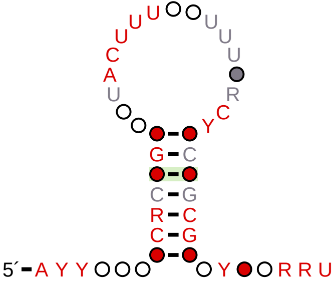

HMP2_13317_1

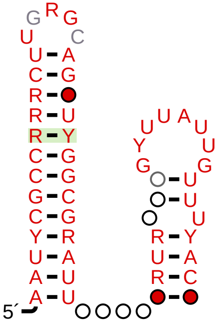

HMP2_13319_1

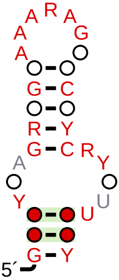

HMP2_1332_1

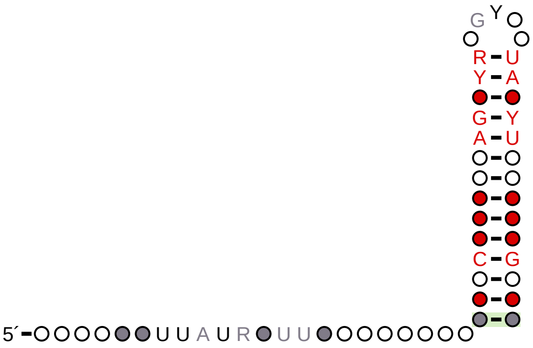

HMP2_13321_1

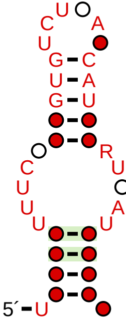

HMP2_13329_1

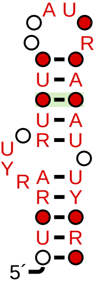

HMP2_13337_1

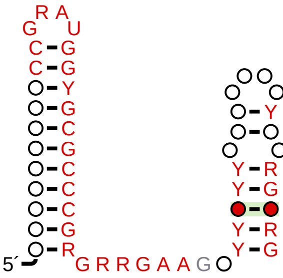

HMP2_13341_1

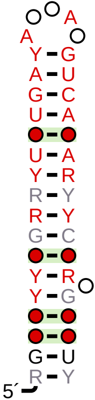

HMP2_13342_1

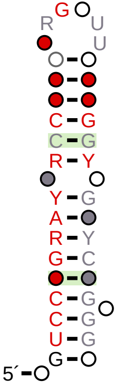

HMP2_13350_1

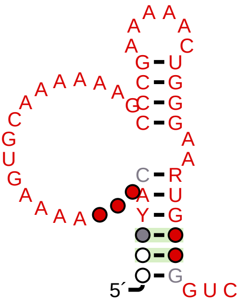

HMP2_1336_1

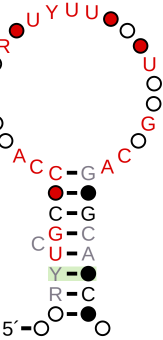

HMP2_13373_1

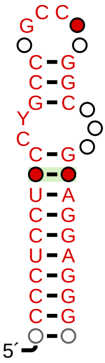

HMP2_13378_1

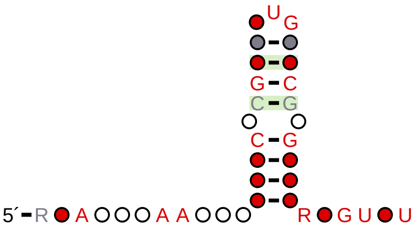

HMP2_13382_1

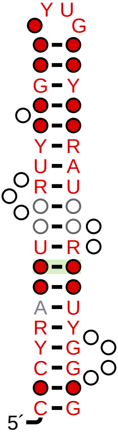

HMP2_13384_1

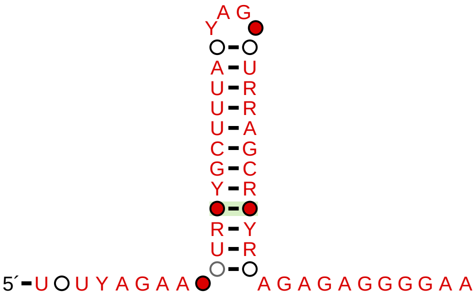

HMP2_13388_1

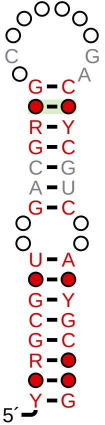

HMP2_13392_1

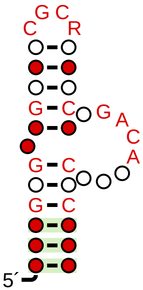

HMP2_13394_1

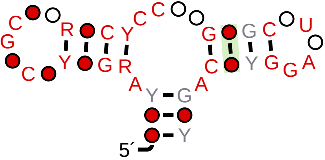

HMP2_134_1

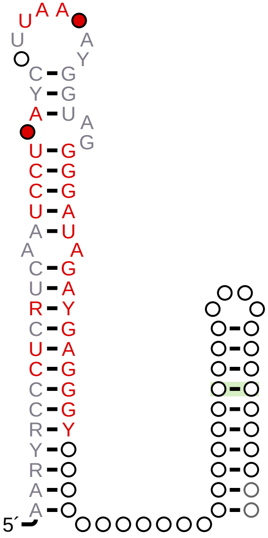

HMP2_1340_1

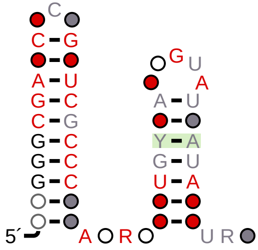

HMP2_1341_1

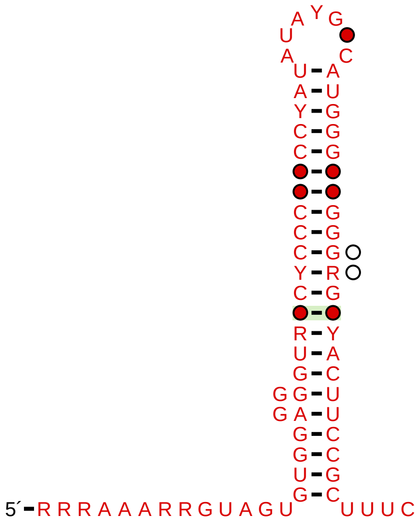

5´
RRRAAARRGUAGU
GUGGGAGGUGRCYCCCCC
UAYCCCCAUAY
GCACUAGGRGGGGGAUGG
UUUC

HMP2_13410_1

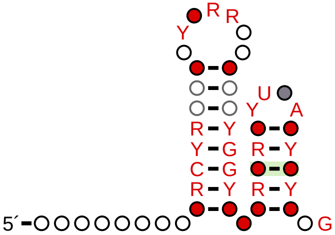

HMP2_13432_1

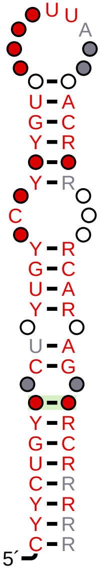

HMP2_13440_1

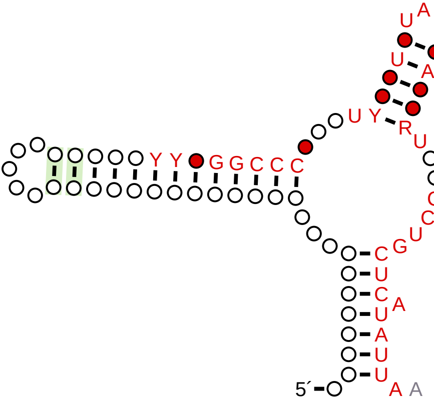

HMP2_13450_1

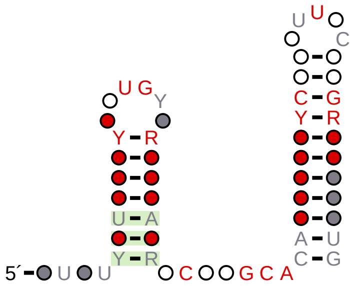

HMP2_13458_1

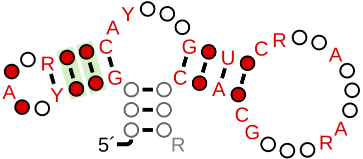

HMP2_1347_1

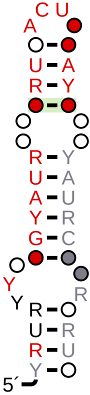

HMP2_13485_1

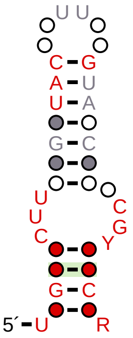

HMP2_13487_1

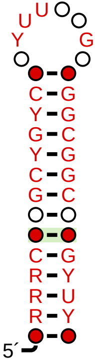

HMP2_1352_1

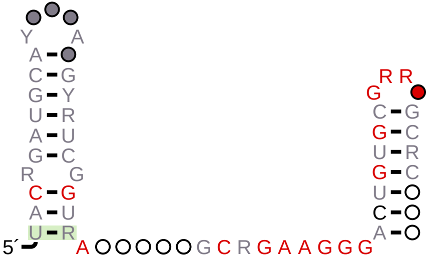

HMP2_13522_1

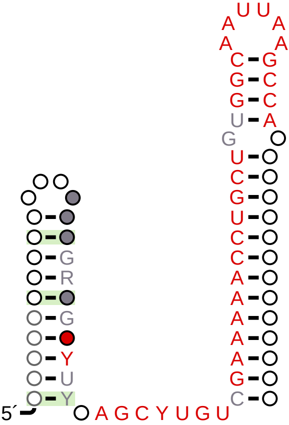

HMP2_13530_1

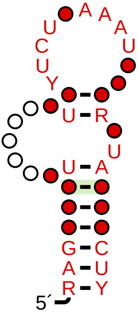

HMP2_13548_1

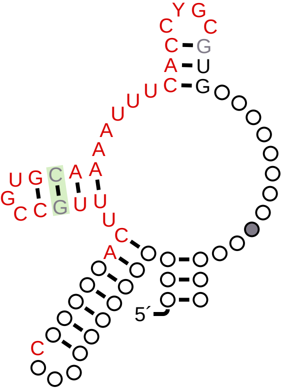

HMP2_13552_1

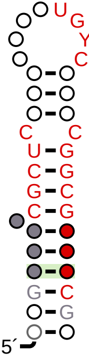

HMP2_13557_1

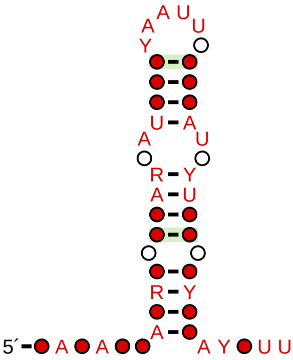

HMP2_13559_1

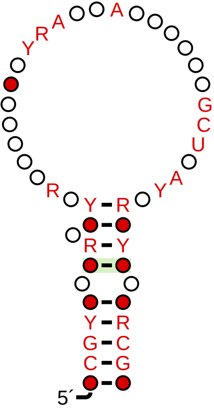

HMP2_13560_1

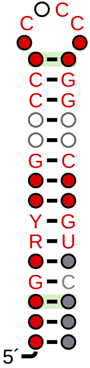

HMP2_13573_1

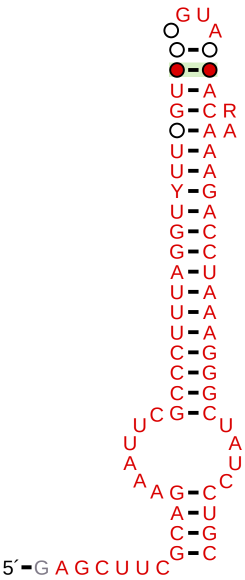

HMP2_13577_1

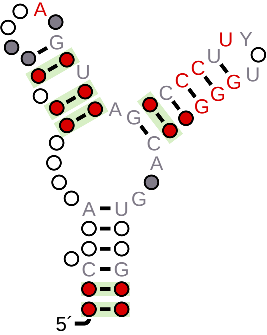

HMP2_13584_1

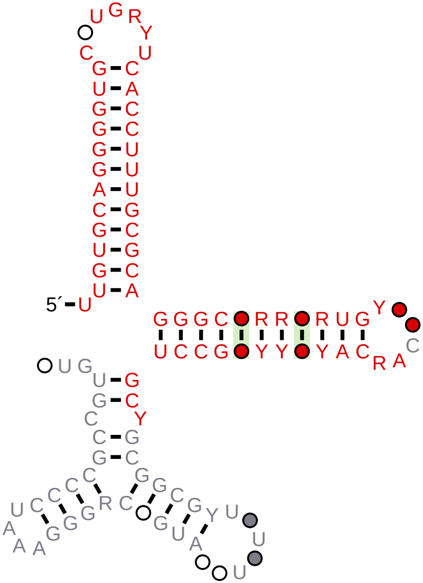

HMP2_13588_1

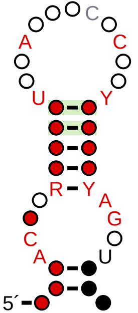

HMP2_13596_1

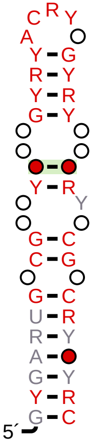

HMP2_13599_1

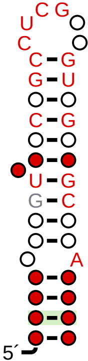

HMP2_1360_1

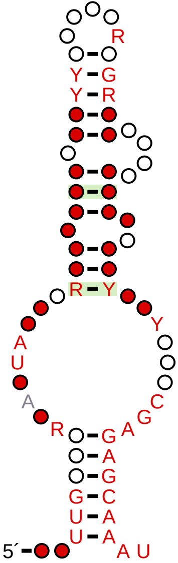

HMP2_13601_1

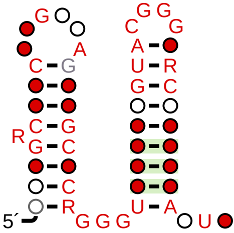

HMP2_13603_1

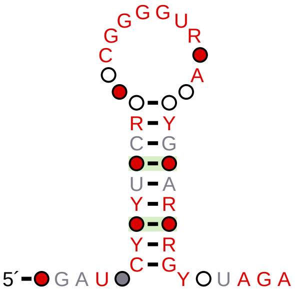

HMP2_13606_1

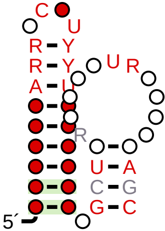

HMP2_13607_1

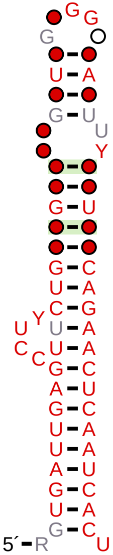

HMP2_13612_1

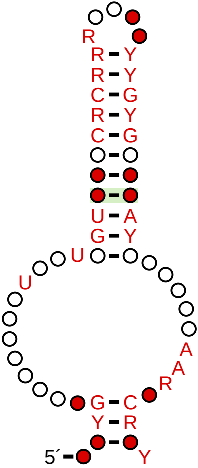

HMP2_13619_1

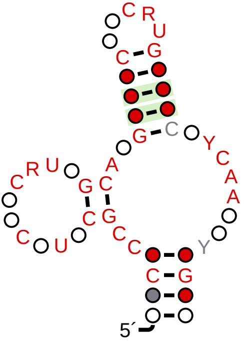

HMP2_13621_1

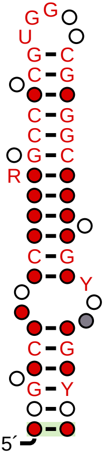

HMP2_13627_1

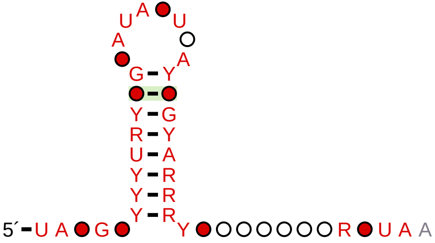

HMP2_13635_1

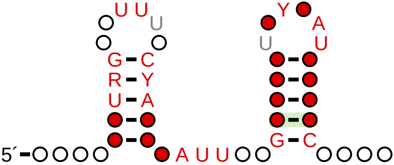

HMP2_13643_1

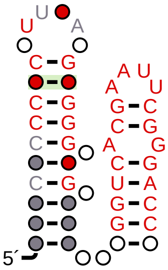

HMP2_13644_1

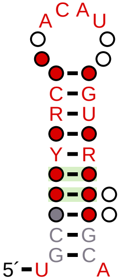

HMP2_13645_1

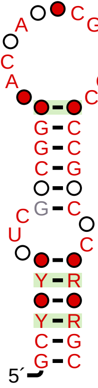

HMP2_13651_1

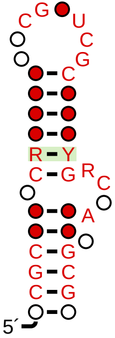

HMP2_13655_1

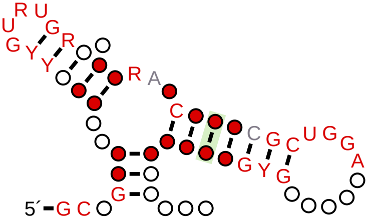

HMP2_13659_1

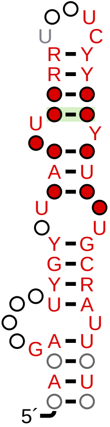

HMP2_13660_1

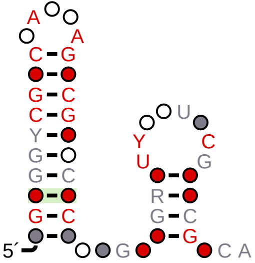

HMP2_13663_1

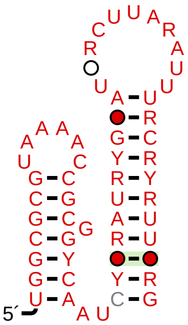

HMP2_13668_1

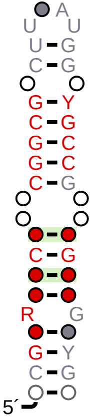

HMP2_13676_1

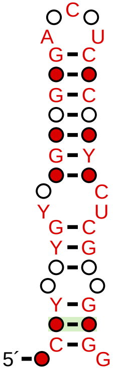

HMP2_13678_1

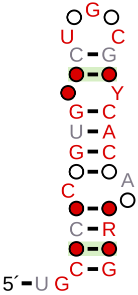

HMP2_13683_1

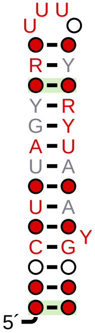

HMP2_13684_1

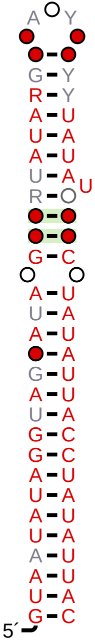

HMP2_13687_1

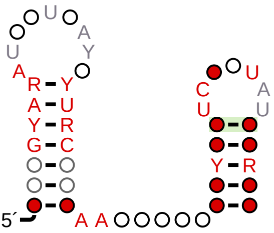

HMP2_13691_1

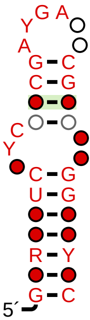

HMP2_13700_1

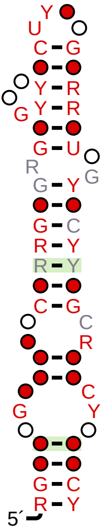

HMP2_13704_1

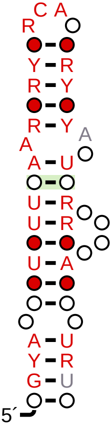

HMP2_13706_1

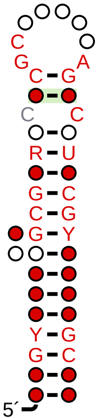

HMP2_13707_1

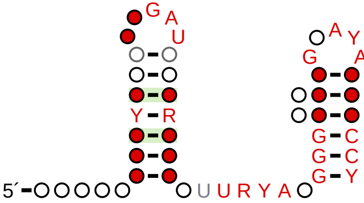

HMP2_13711_1

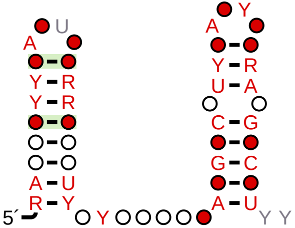

HMP2_13712_1

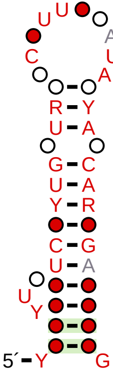

HMP2_13714_1

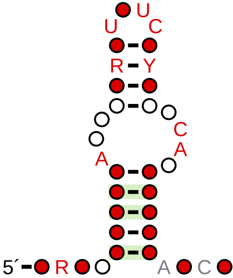

HMP2_1372_1

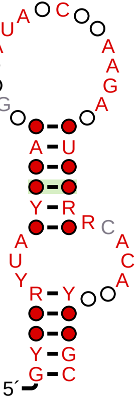

HMP2_13724_1

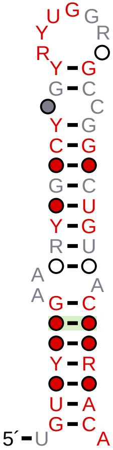

HMP2_13727_1

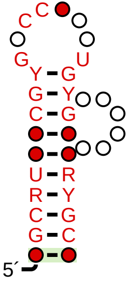

HMP2_13728_1

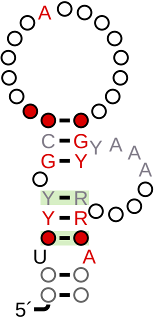

HMP2_13729_1

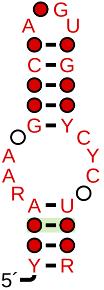

HMP2_13755_1

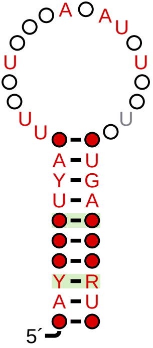

HMP2_13762_1

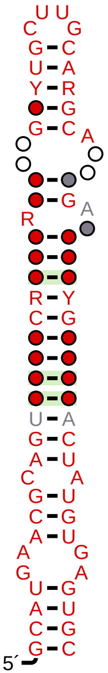

HMP2_13766_1

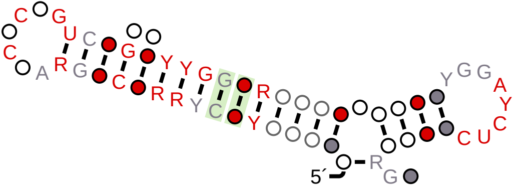

HMP2_13770_1

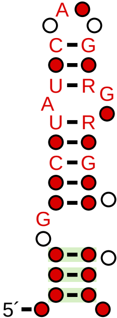

HMP2_13775_1

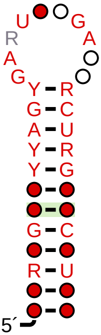

HMP2_1378_1

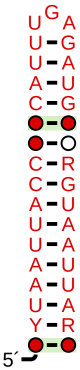

HMP2_13780_1

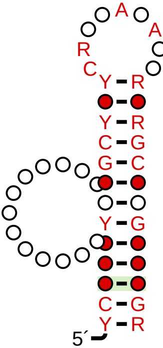

HMP2_1379_1

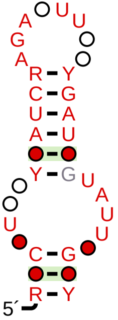

HMP2_13798_1

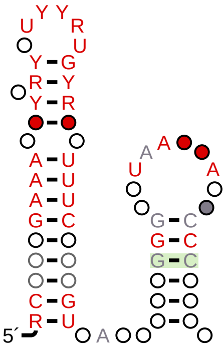

HMP2_138_1

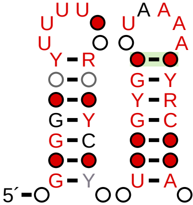

HMP2_13800_1

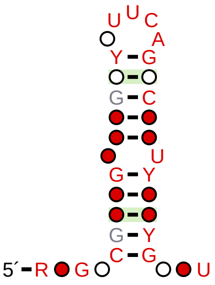

HMP2_1382_1

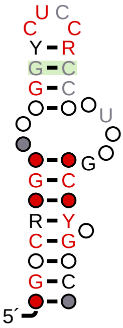

HMP2_13822_1

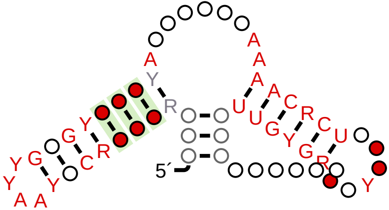

HMP2_13828_1

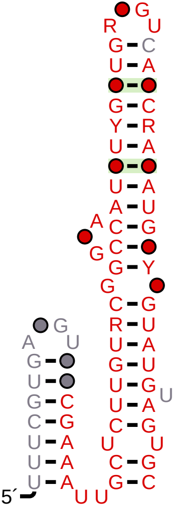

HMP2_13831_1

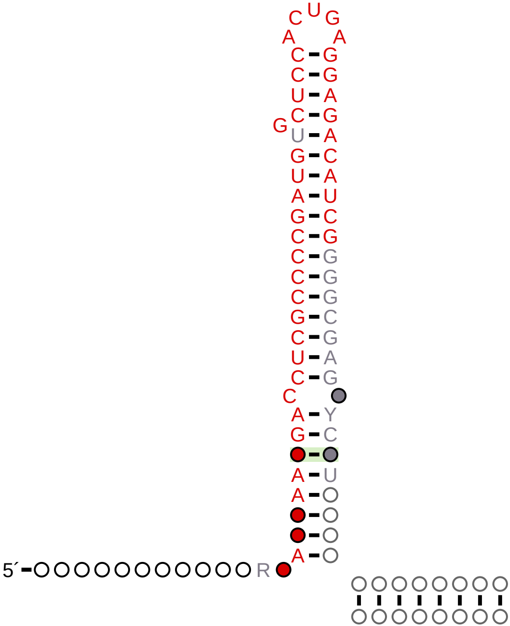

HMP2_13834_1

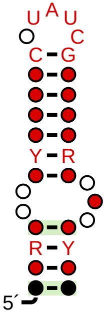

HMP2_13841_1

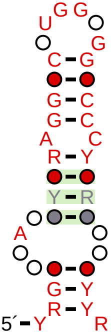

HMP2_13845_1

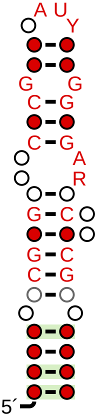

HMP2_13847_1

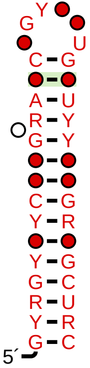

HMP2_13850_1

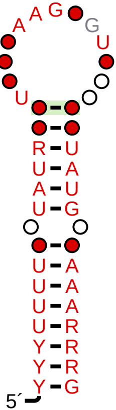

HMP2_13856_1

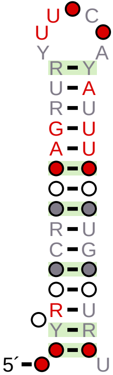

HMP2_13866_1

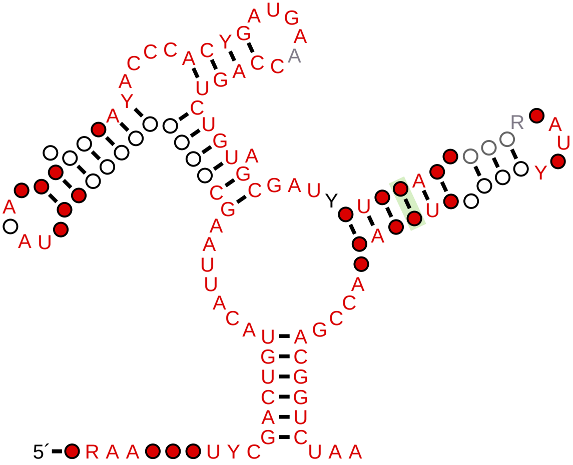

HMP2_13867_1

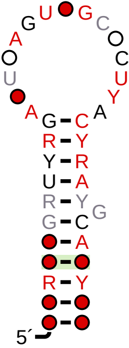

HMP2_13874_1

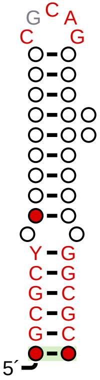

HMP2_13876_1

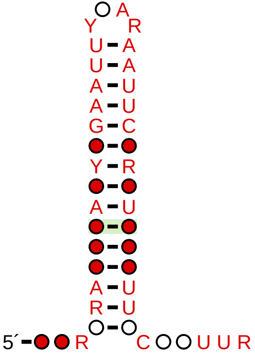

HMP2_13880_1

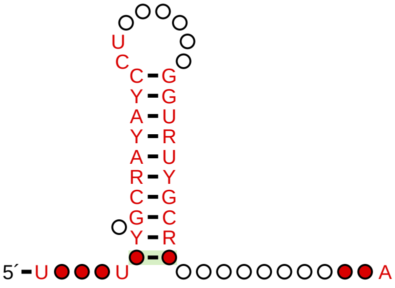

HMP2_13885_1

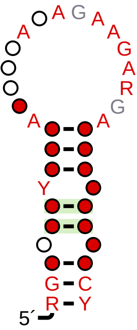

HMP2_13886_1

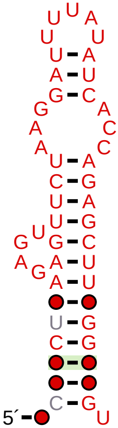

HMP2_13888_1

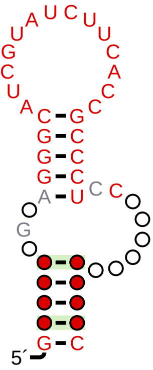

HMP2_1389_1

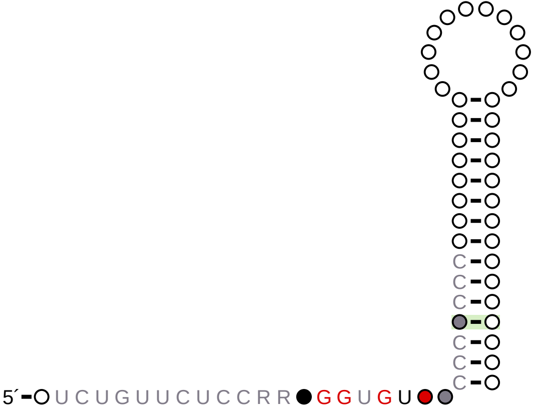

HMP2_13890_1

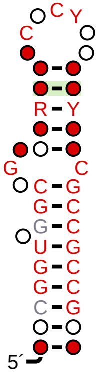

HMP2_13896_1

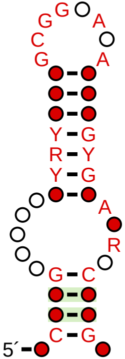

HMP2_13899_1

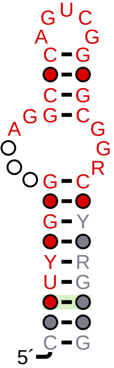

HMP2_139_1

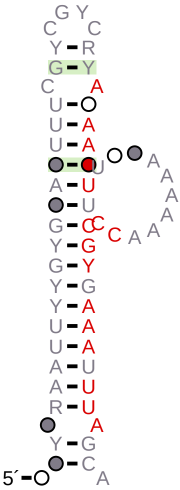

HMP2_13907_1

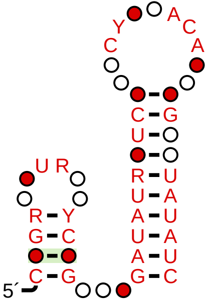

HMP2_13915_1

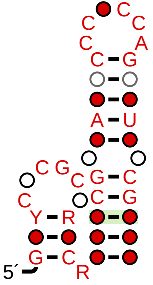

HMP2_13916_1

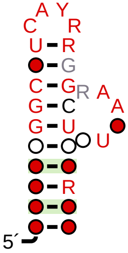

HMP2_13918_1

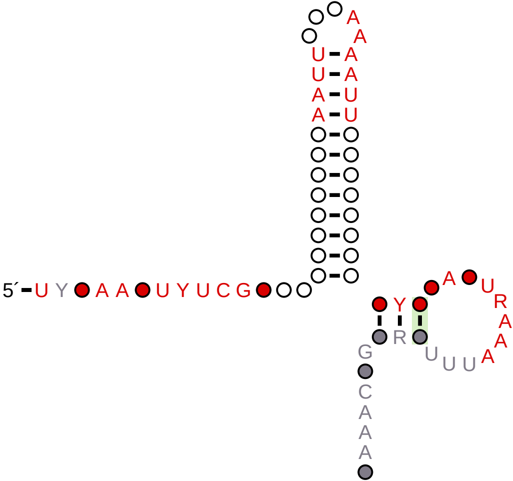

HMP2_1392_1

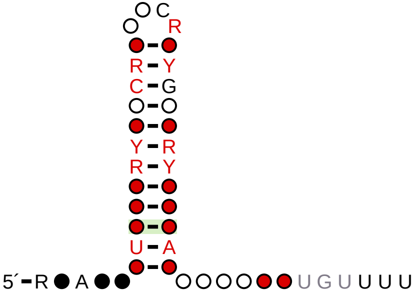

HMP2_13923_1

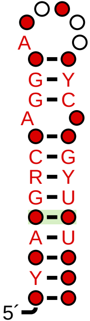

HMP2_13924_1

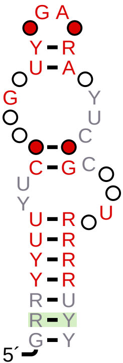

HMP2_13927_1

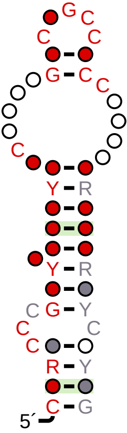

HMP2_13929_1

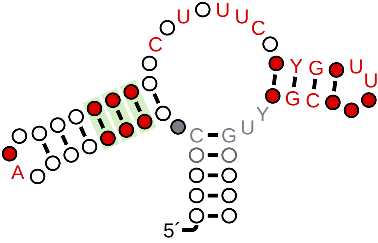

HMP2_1393_1

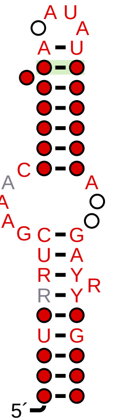

HMP2_13932_1

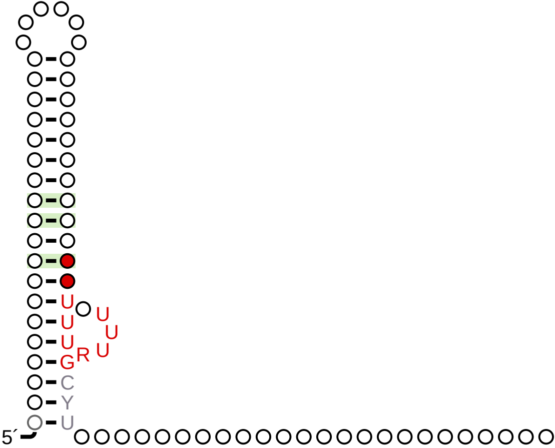

HMP2_13937_1

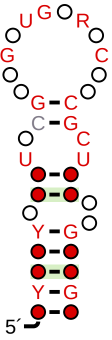

HMP2_13944_1

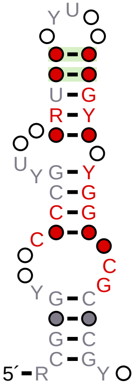

HMP2_1396_1

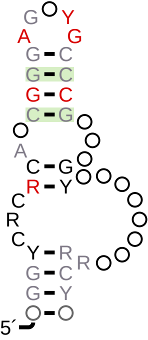

HMP2_13965_1

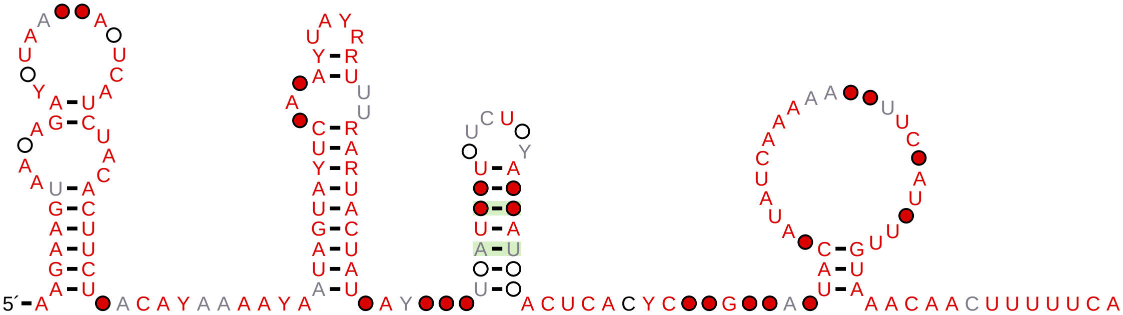

HMP2_13967_1

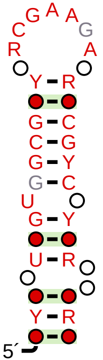

HMP2_13992_1

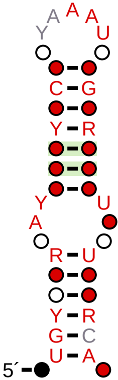

HMP2_1400_1

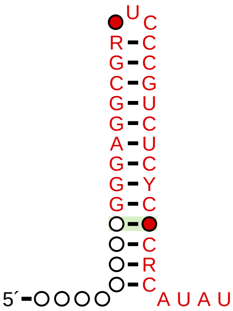

HMP2_14004_1

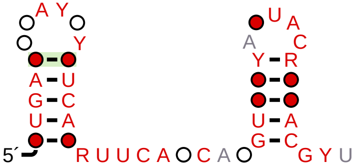

HMP2_14011_1

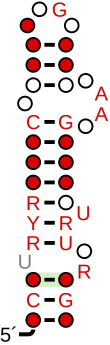

HMP2_14012_1

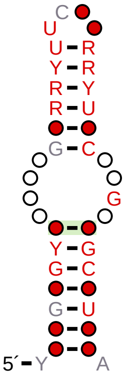

HMP2_14013_1

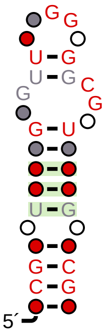

HMP2_14021_1

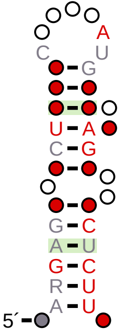

HMP2_14024_1

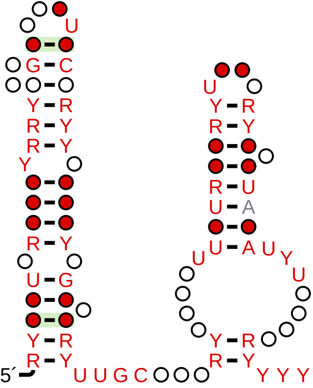

HMP2_1403_1

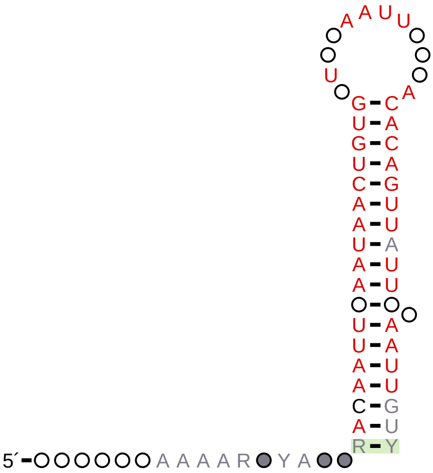

HMP2_14031_1

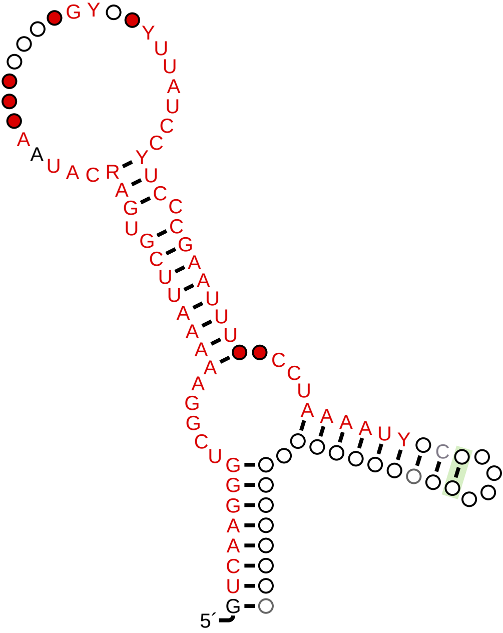

HMP2_14037_1

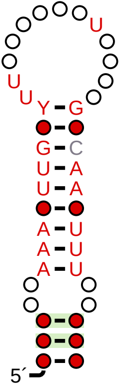

HMP2_14038_1

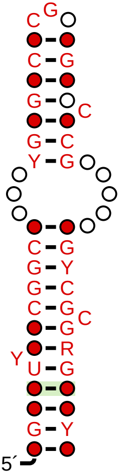

HMP2_14040_1

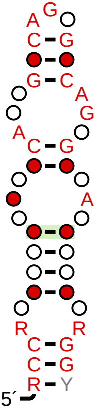

HMP2_14051_1

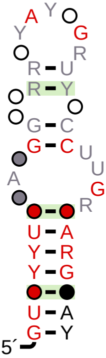

HMP2_14056_1

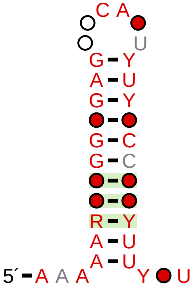

HMP2_14070_1

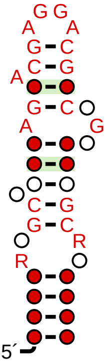

HMP2_14078_1

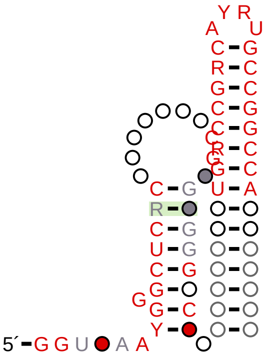

HMP2_14080_1

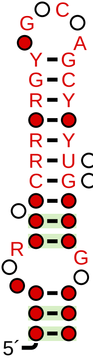

HMP2_1409_1

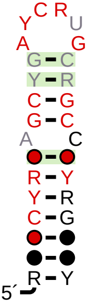

HMP2_14090_1

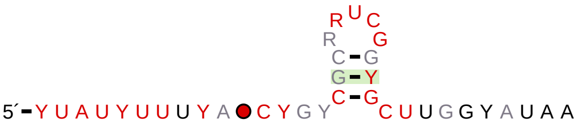

HMP2_14091_1

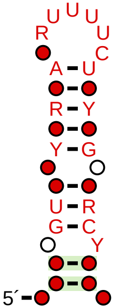

HMP2_14096_1

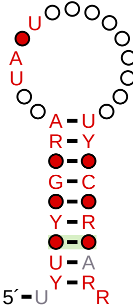

HMP2_14103_1

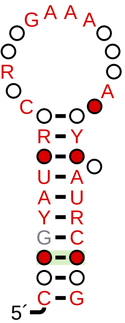

HMP2_14109_1

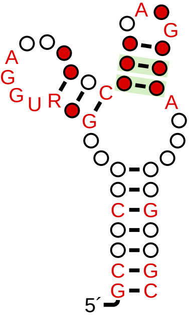

HMP2_14114_1

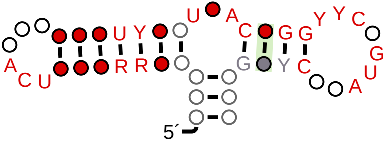

HMP2_14124_1

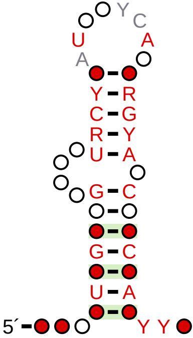

HMP2_14127_1

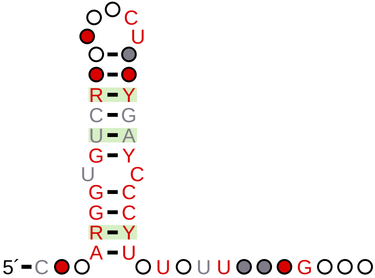

HMP2_14140_1

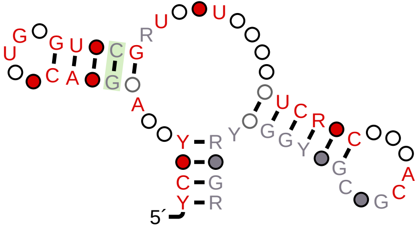

HMP2_14146_1

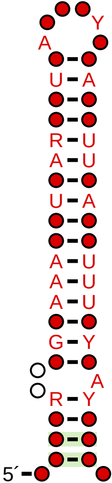

HMP2_14158_1

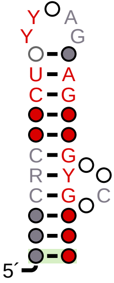

HMP2_14161_1

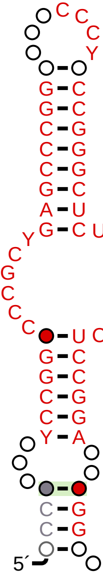

HMP2_14165_1

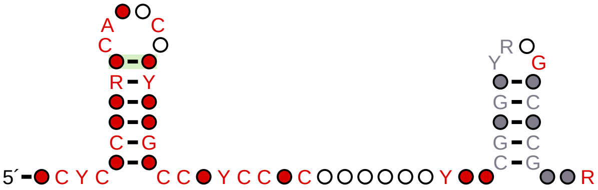

HMP2_14170_1

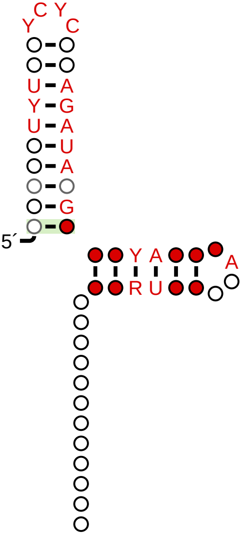

HMP2_14171_1

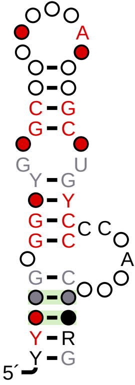

HMP2_14176_1

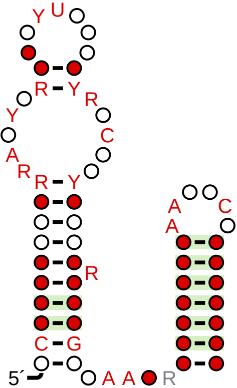

HMP2_14177_1

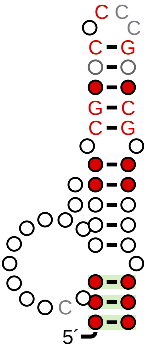

HMP2_1418_1

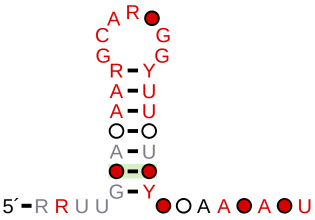

HMP2_14181_1

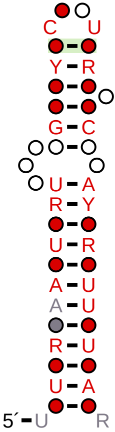

HMP2_14183_1

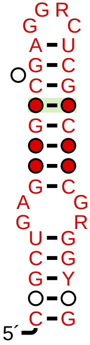

HMP2_14198_1

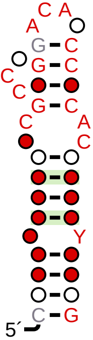

HMP2_14201_1

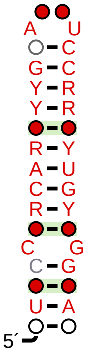

HMP2_14202_1

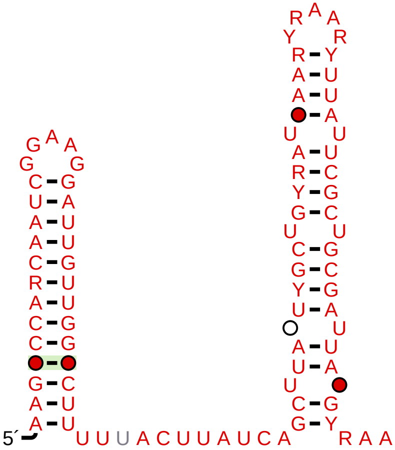

HMP2_14204_1

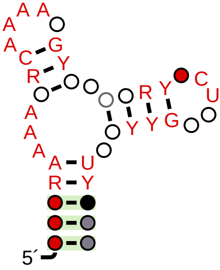

HMP2_14205_1

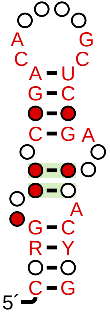

HMP2_14208_1

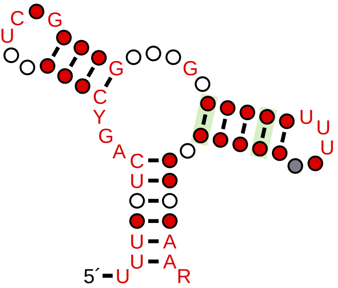

HMP2_14215_1

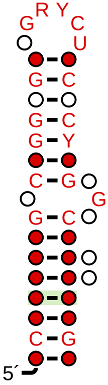

HMP2_14217_1

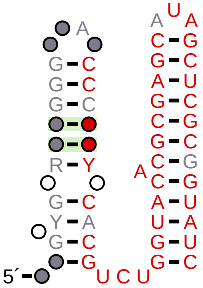

HMP2_14218_1

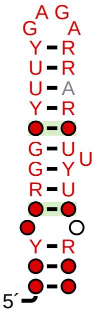

HMP2_1422_1

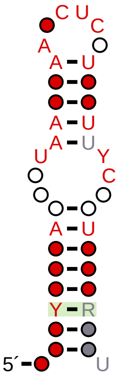

HMP2_14225_1

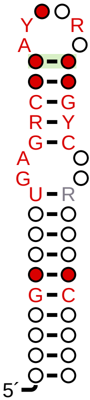

HMP2_1423_1

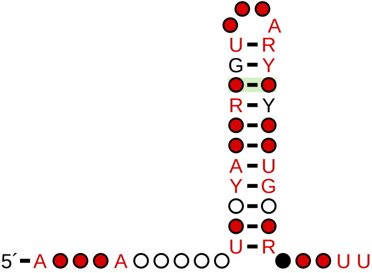

HMP2_14232_1

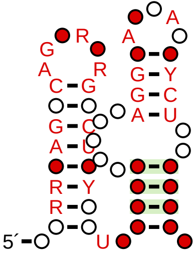

HMP2_1424_1

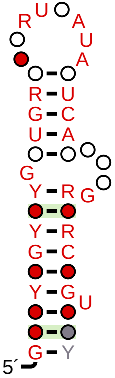

HMP2_14249_1

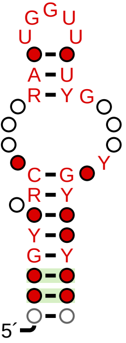

HMP2_14256_1

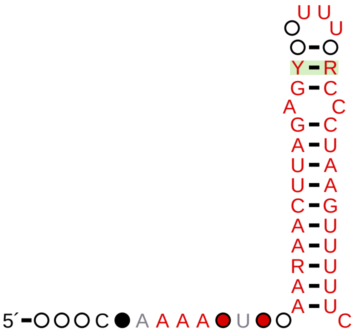

HMP2_14259_1

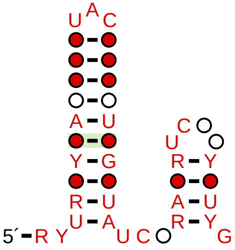

HMP2_1426_1

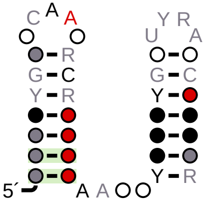

HMP2_14263_1

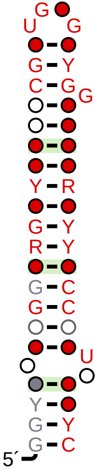

HMP2_14266_1

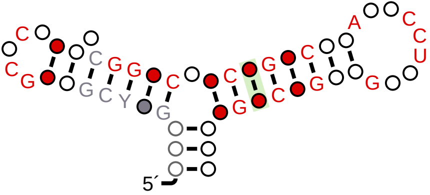

HMP2_14273_1

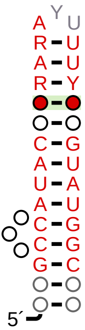

HMP2_14274_1

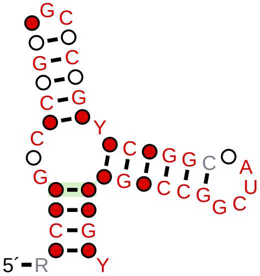

HMP2_14278_1

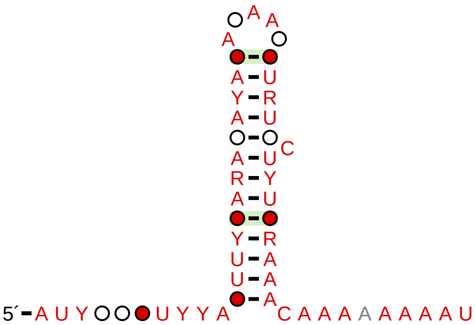

HMP2_14281_1

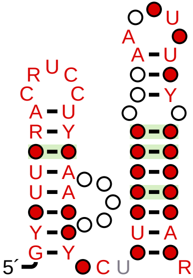

HMP2_14283_1

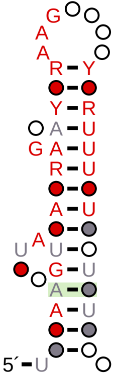

HMP2_1429_1

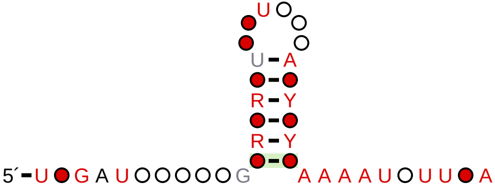

HMP2_14290_1

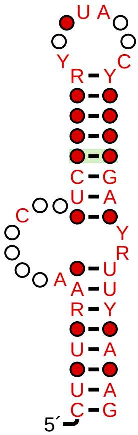

HMP2_14295_1

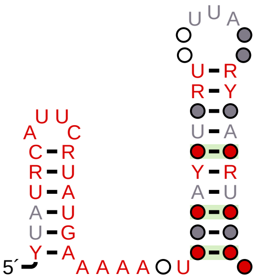

HMP2_14303_1

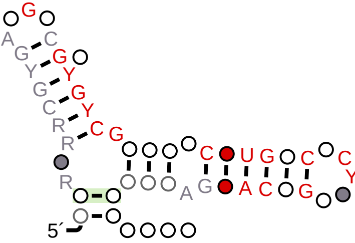

HMP2_14304_1

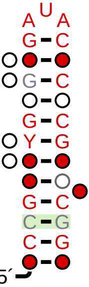

HMP2_14306_1

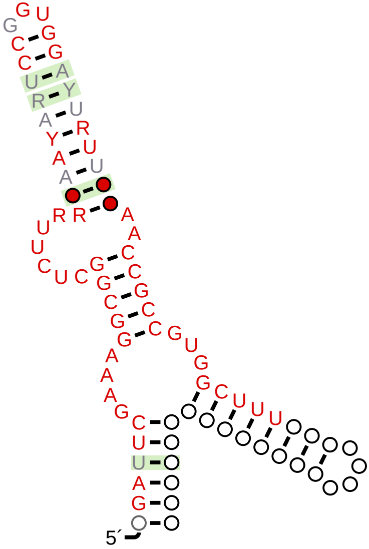

HMP2_14311_1

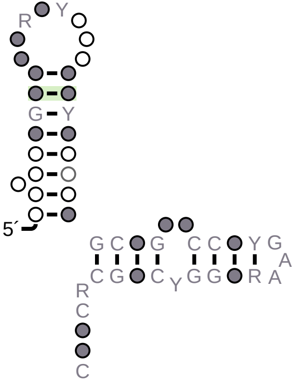

HMP2_14317_1

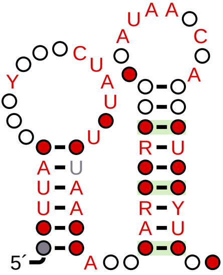

HMP2_14322_1

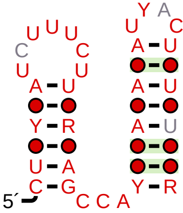

HMP2_14325_1

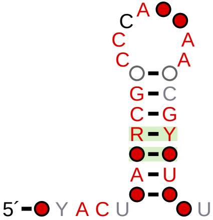

HMP2_14333_1

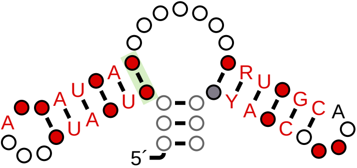

HMP2_14339_1

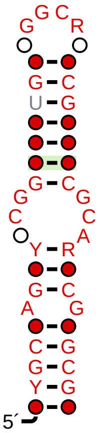

HMP2_1434_1

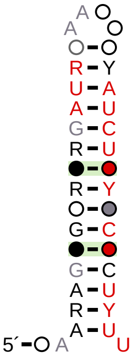

HMP2_1435_1

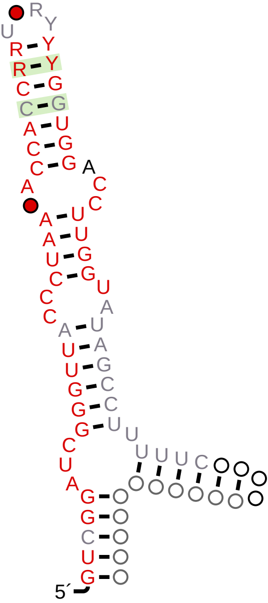

HMP2_14350_1

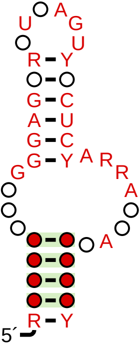

HMP2_14352_1

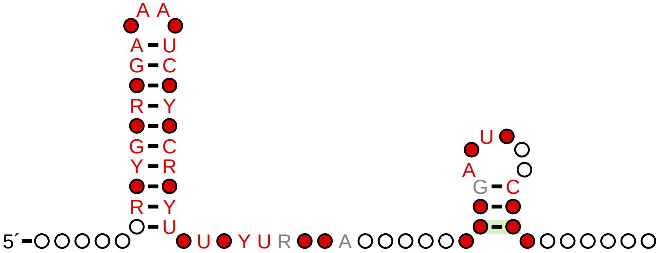

HMP2_14355_1

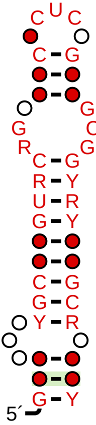

HMP2_14389_1

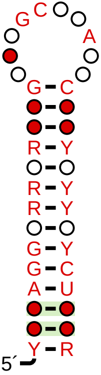

HMP2_14396_1

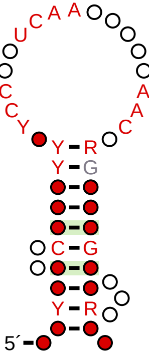

HMP2_14398_1

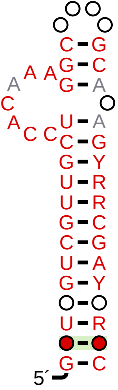

HMP2_1440_1

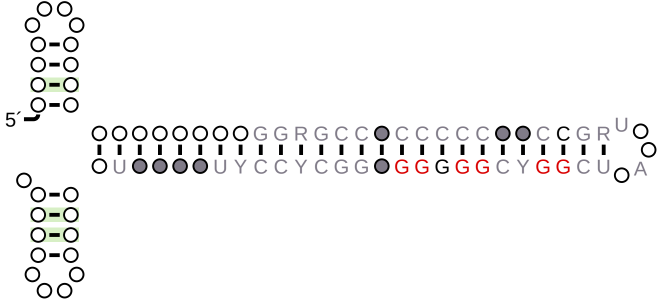

HMP2_14408_1

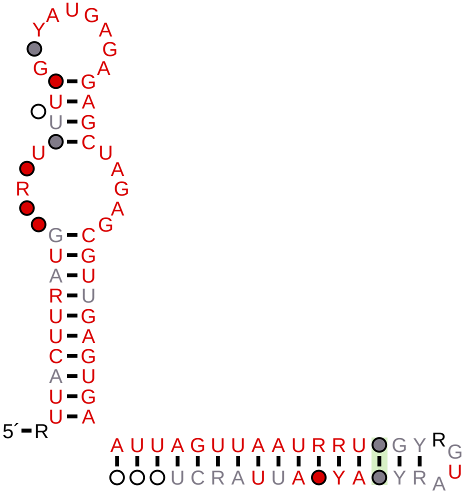

HMP2_1441_1

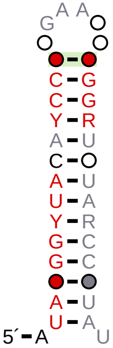

HMP2_14416_1

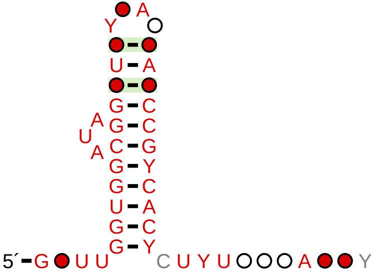

HMP2_1442_1

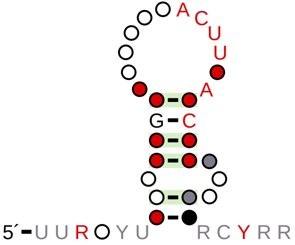

HMP2_14428_1

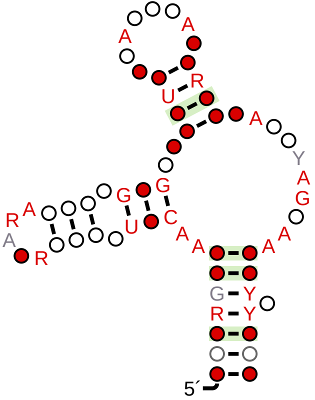

HMP2_14439_1

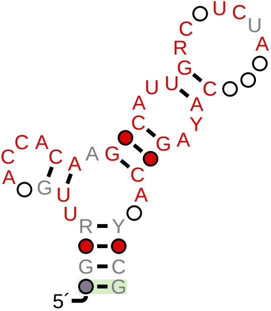

HMP2_14453_1

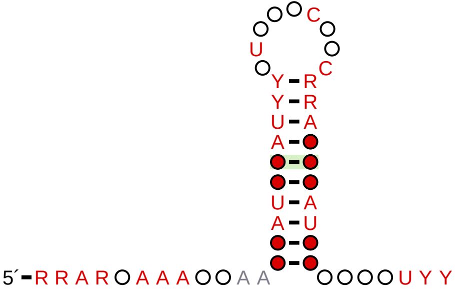

HMP2_14460_1

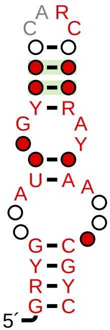

HMP2_1447_1

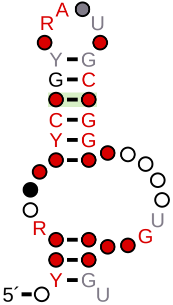

HMP2_14470_1

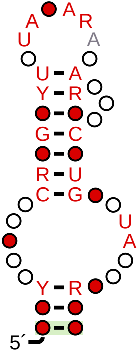

HMP2_14479_1

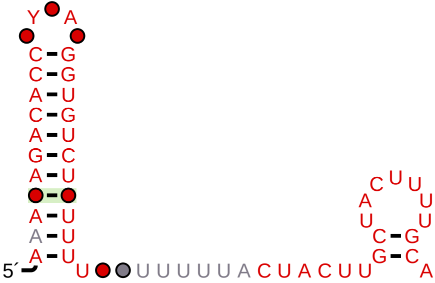

HMP2_14484_1

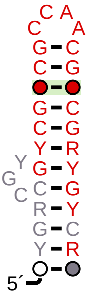

HMP2_14488_1

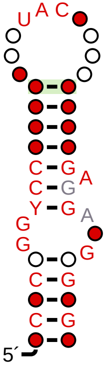

HMP2_14496_1

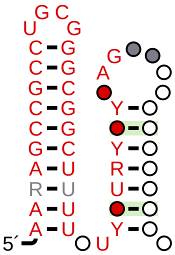

HMP2_14497_1

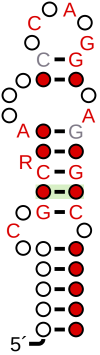

HMP2_14502_1

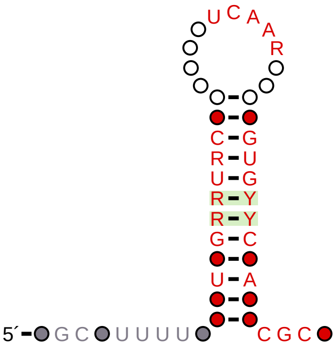

HMP2_14503_1

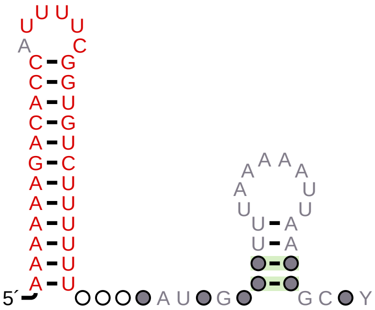

UUUU
A C
C-G
C-G
A-U
C-G
A-U
G-C
A-U
A-U
A-U
A-U
A-U
A-U
5´
AUGG
AAAA
A U
U U
U-A
U-A
GCY

HMP2_14506_1

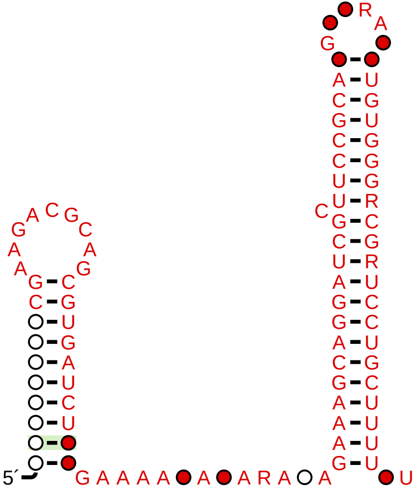

HMP2_14512_1

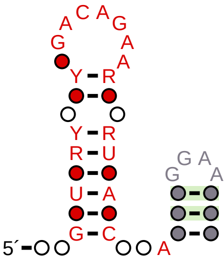

HMP2_14513_1

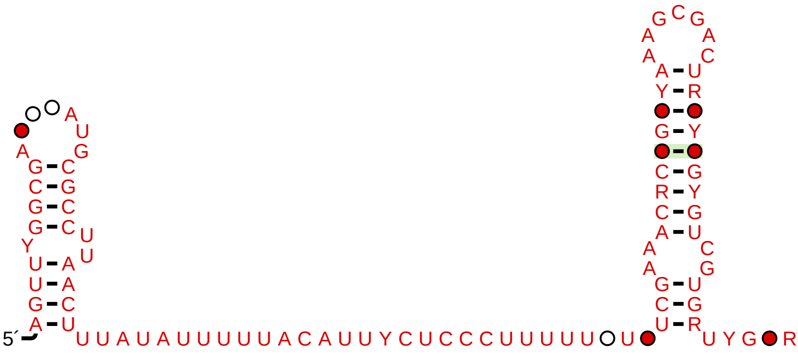

HMP2_14522_1

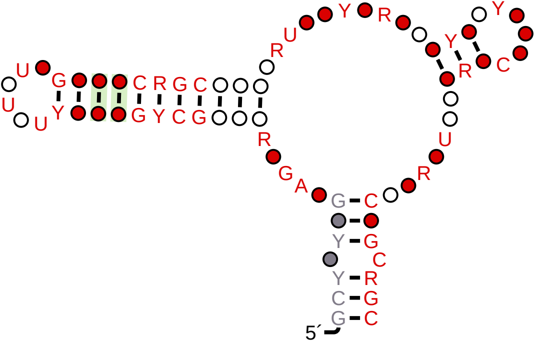

HMP2_14523_1

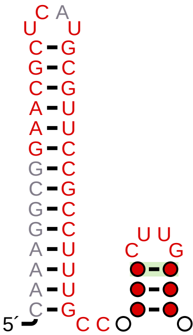

HMP2_14530_1

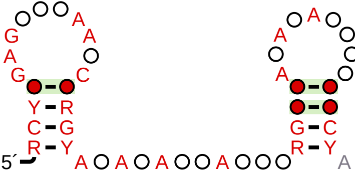

HMP2_14534_1

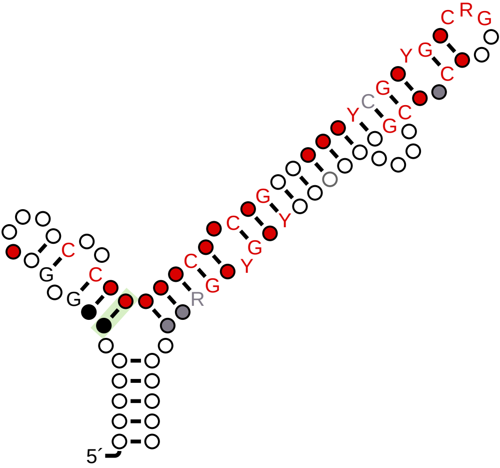

HMP2_14535_1

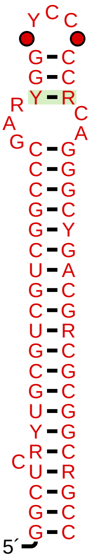

HMP2_14539_1

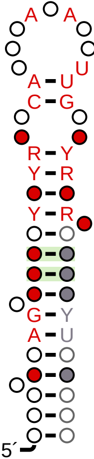

HMP2_14545_1

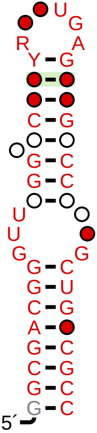

HMP2_14546_1

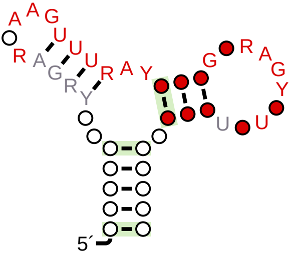

HMP2_14549_1

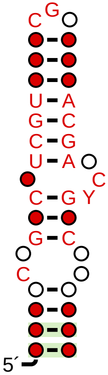

HMP2_14554_1

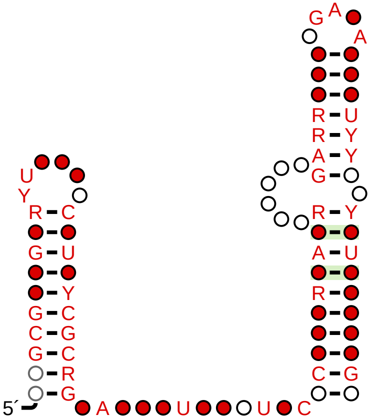

HMP2_1456_1

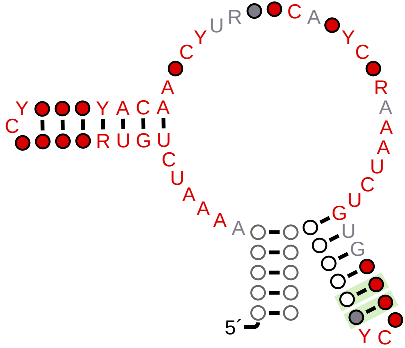

HMP2_14568_1

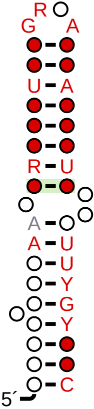

HMP2_1457_1

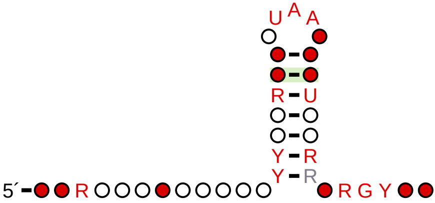

HMP2_14571_1

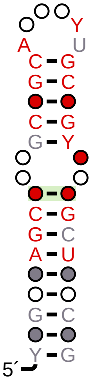

HMP2_14579_1

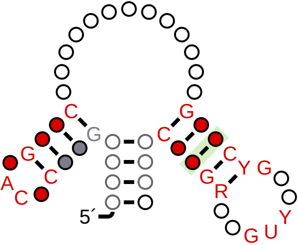

HMP2_14581_1

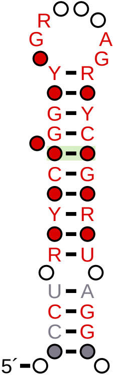

HMP2_14595_1

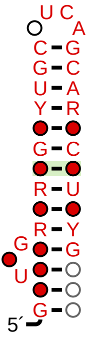

HMP2_14599_1

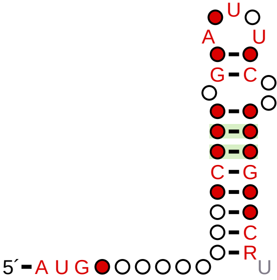

HMP2_14601_1

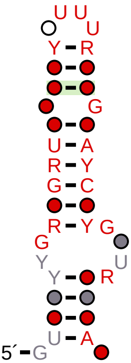

HMP2_14603_1

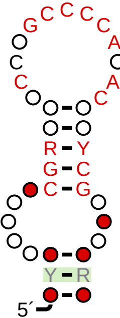

HMP2_14606_1

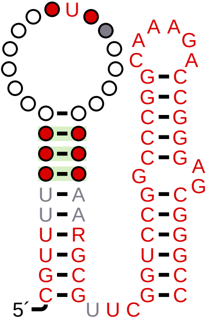

HMP2_1461_1

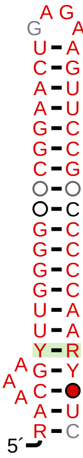

HMP2_14619_1

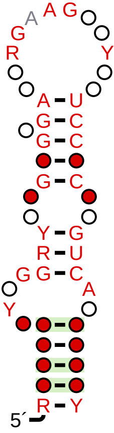

HMP2_1462_1

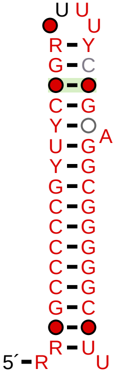

HMP2_14624_1

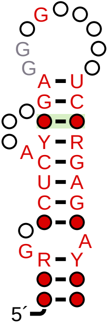

HMP2_14625_1

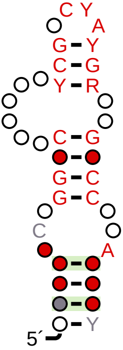

HMP2_14631_1

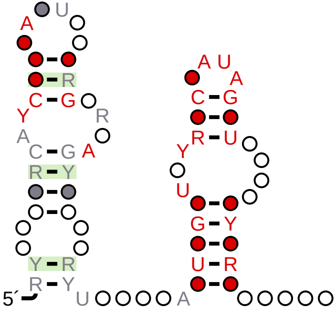

HMP2_14636_1

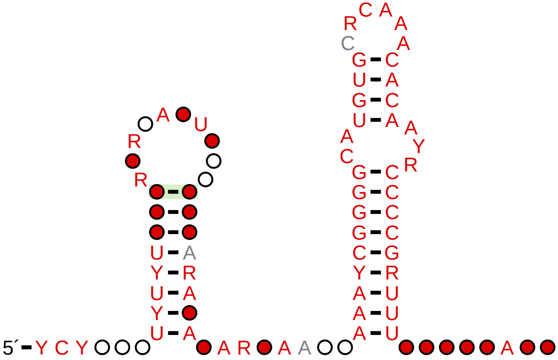

HMP2_14642_1

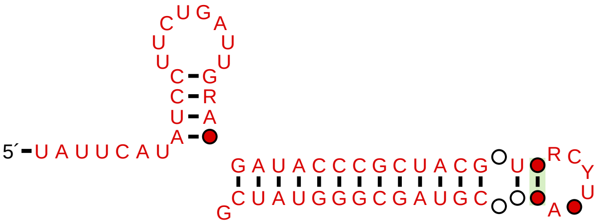

HMP2_14643_1

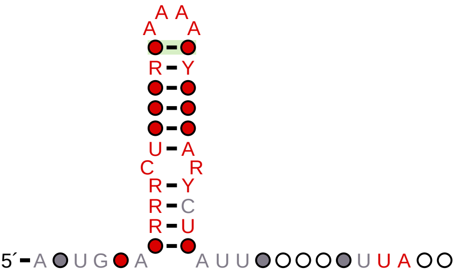

HMP2_14656_1

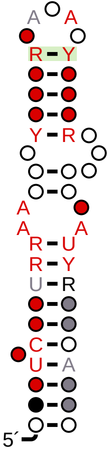

HMP2_14660_1

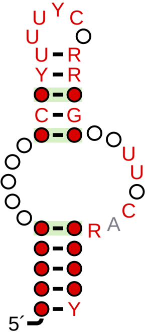

HMP2_14664_1

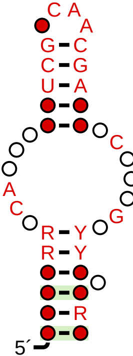

HMP2_14665_1

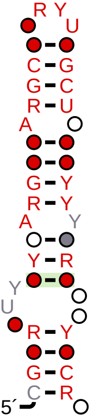

HMP2_14668_1

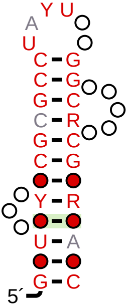

HMP2_14670_1

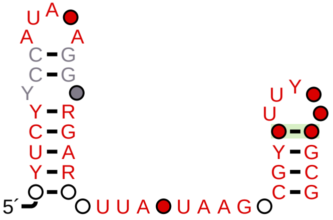

HMP2_14681_1

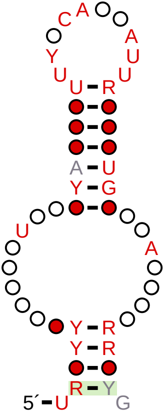

HMP2_14689_1

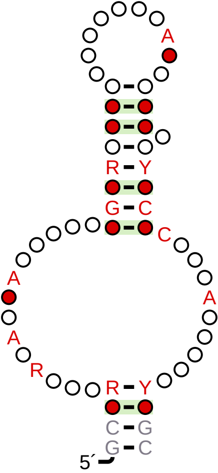

HMP2_14694_1

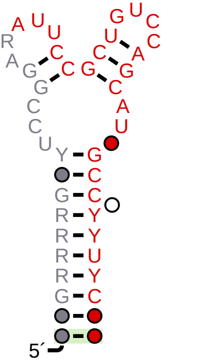

HMP2_14697_1

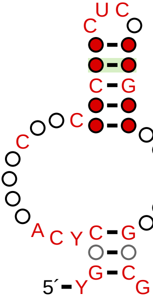

HMP2_147_1

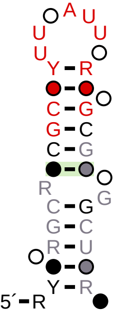

HMP2_1470_1

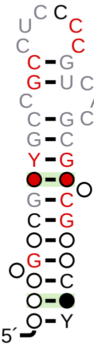

HMP2_14705_1

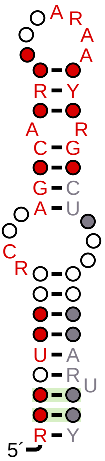

HMP2_14709_1

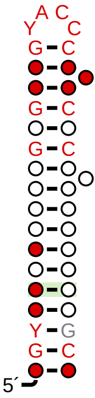

HMP2_14722_1

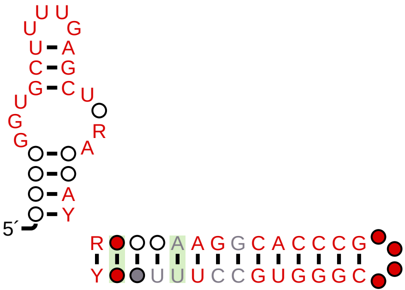

HMP2_14724_1

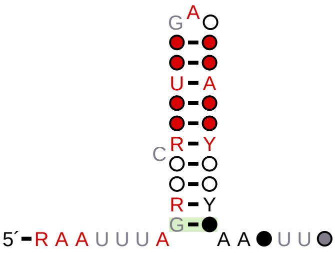

HMP2_14728_1

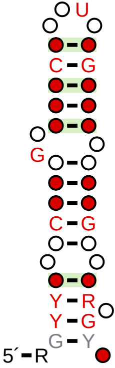

HMP2_14731_1

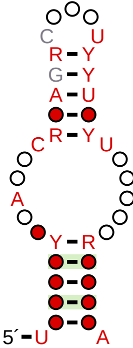

HMP2_14736_1

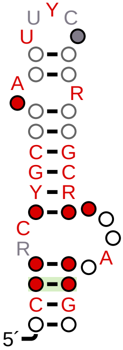

HMP2_14744_1

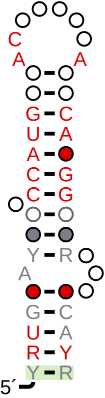

HMP2_14747_1

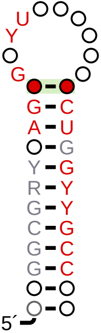

HMP2_14763_1

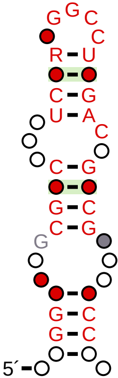

HMP2_1477_1

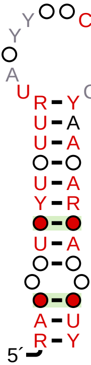

HMP2_14780_1

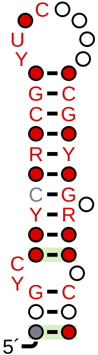

HMP2_1479_1

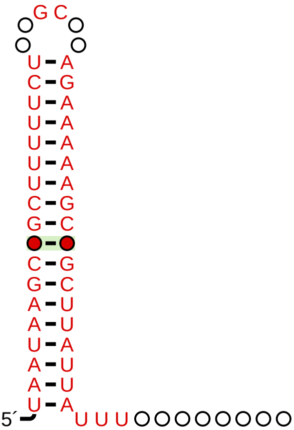

HMP2_14791_1

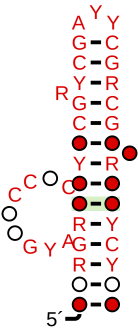

HMP2_14793_1

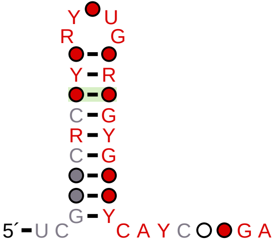

HMP2_148_1

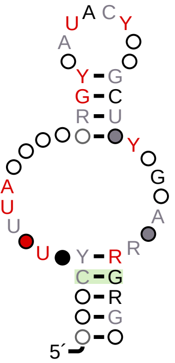

HMP2_14803_1

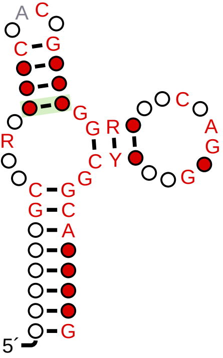

HMP2_14806_1

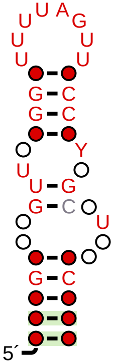

HMP2_1481_1

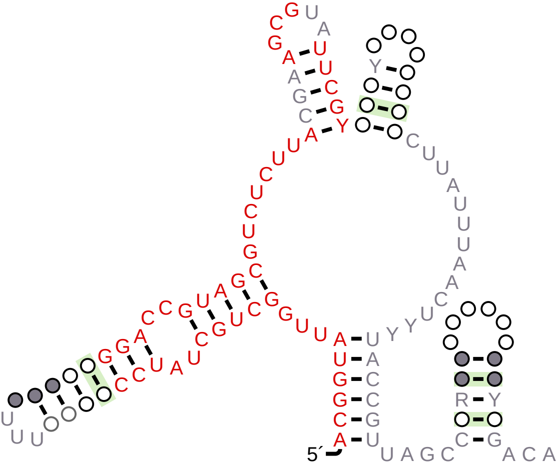

HMP2_14812_1

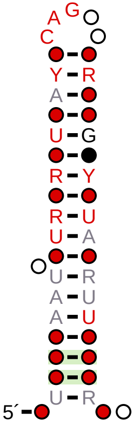

HMP2_14817_1

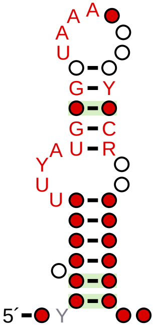

HMP2_1483_1

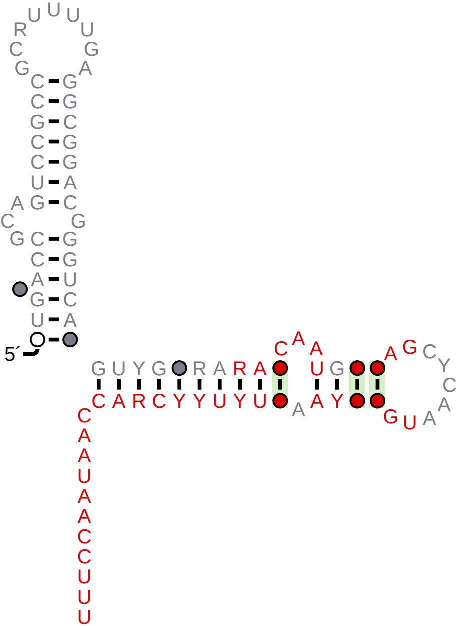

HMP2_14831_1

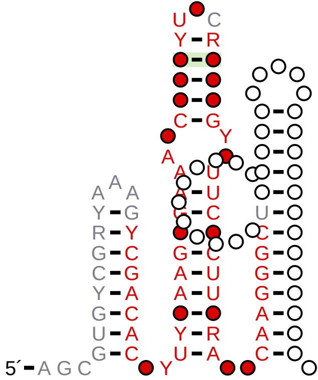

HMP2_14834_1

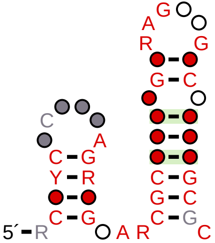

HMP2_14835_1

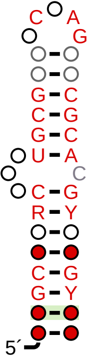

HMP2_14840_1

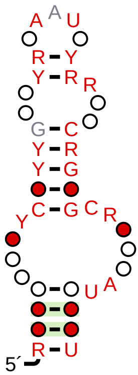

HMP2_14841_1

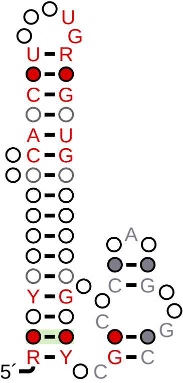

HMP2_14852_1

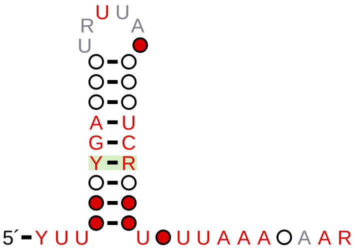

HMP2_14853_1

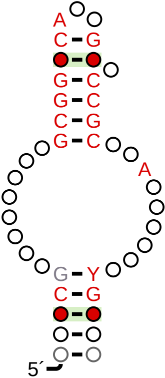

HMP2_14855_1

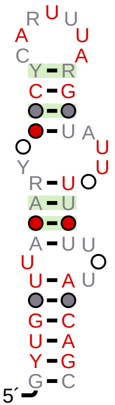

HMP2_14858_1

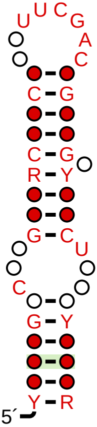

HMP2_14859_1

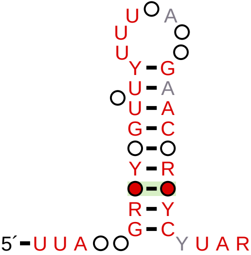

HMP2_14872_1

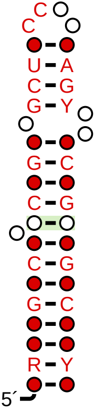

HMP2_14873_1

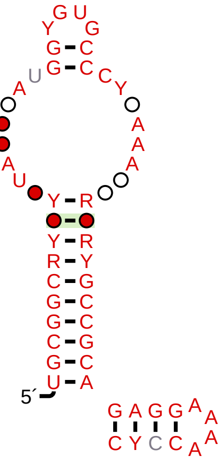

HMP2_14880_1

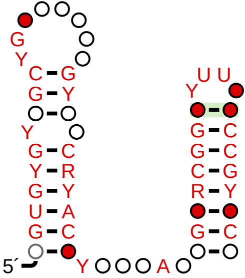

HMP2_14883_1

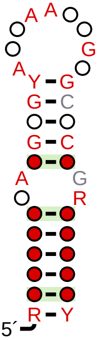

HMP2_14910_1

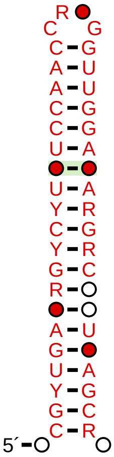

HMP2_14921_1

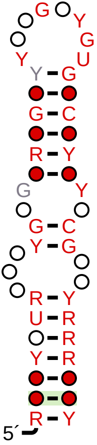

HMP2_14924_1

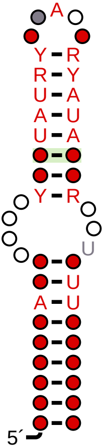

HMP2_14928_1

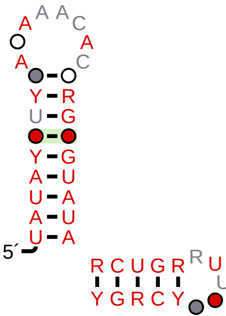

HMP2_14933_1

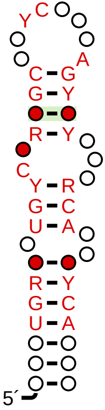

HMP2_14935_1

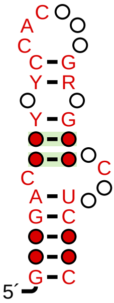

HMP2_14936_1

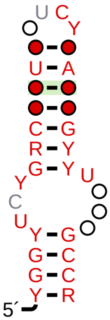

HMP2_14939_1

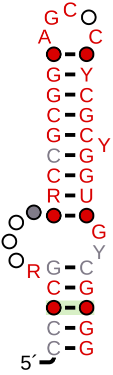

HMP2_14940_1

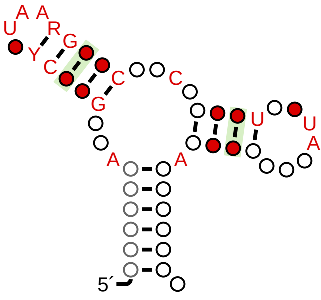

HMP2_14943_1

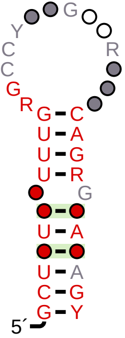

HMP2_1495_1

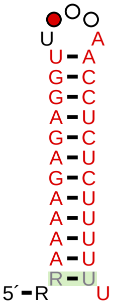

HMP2_14953_1

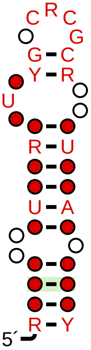

HMP2_14969_1

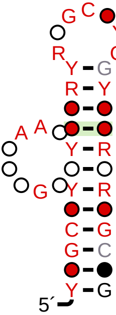

HMP2_14971_1

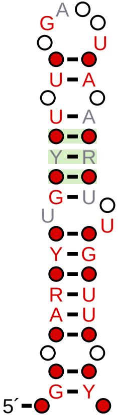

HMP2_14975_1

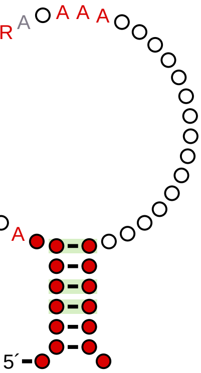

HMP2_14977_1

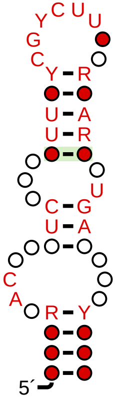

HMP2_14982_1

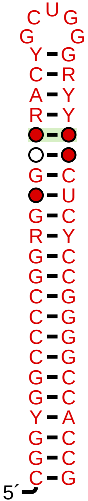

HMP2_14985_1

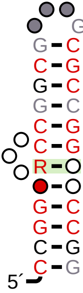

HMP2_14986_1

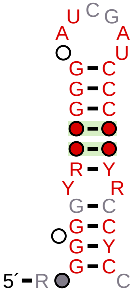

HMP2_14988_1

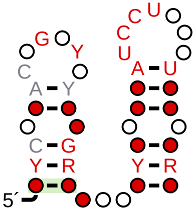

HMP2_1499_1

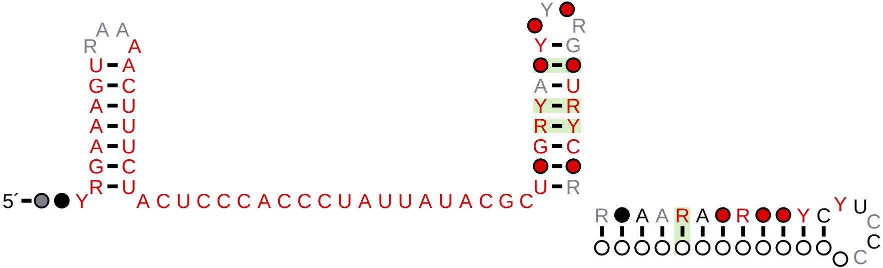

HMP2_14991_1

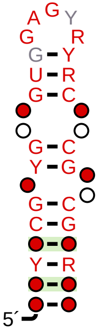

HMP2_14992_1

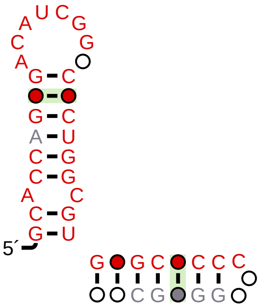

HMP2_14994_1

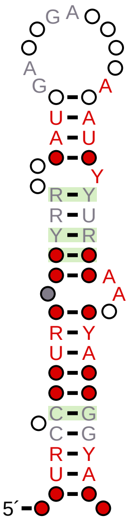

HMP2_150_1

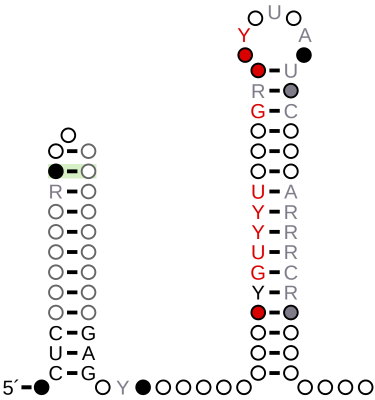

HMP2_15000_1

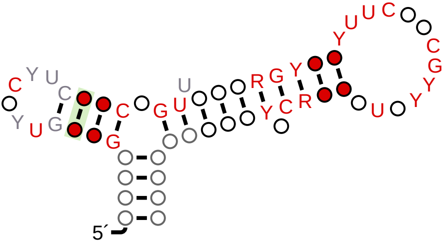

HMP2_15006_1

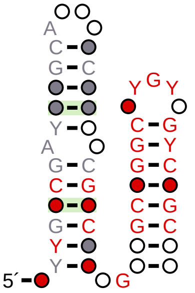

HMP2_15008_1

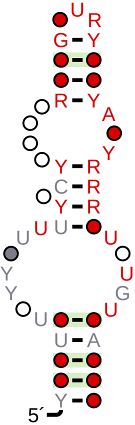

HMP2_15016_1

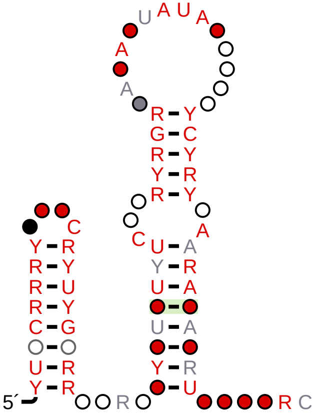

HMP2_15019_1

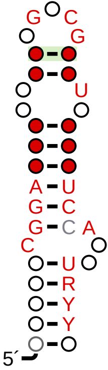

HMP2_1502_1

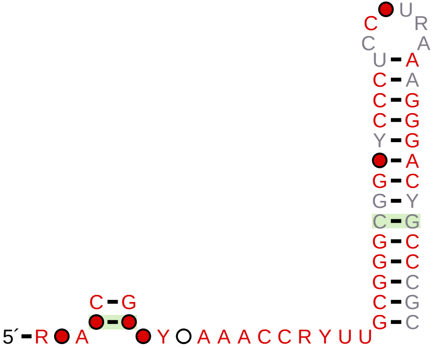

HMP2_15021_1

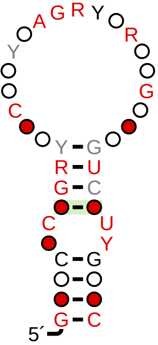

HMP2_15027_1

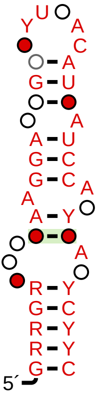

HMP2_15029_1

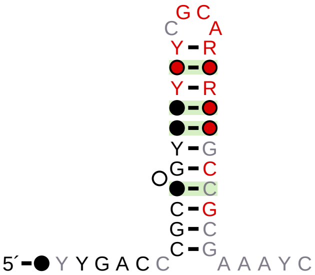

HMP2_15039_1

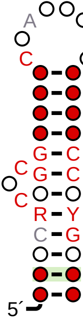

HMP2_15043_1

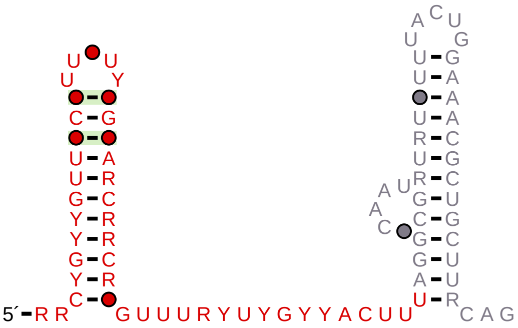

HMP2_15044_1

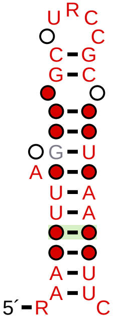

HMP2_15047_1

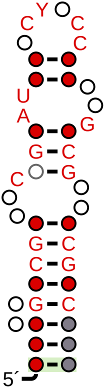

HMP2_15048_1

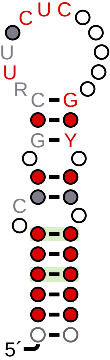

HMP2_15053_1

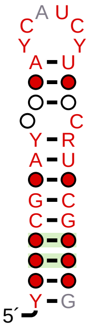

HMP2_15060_1

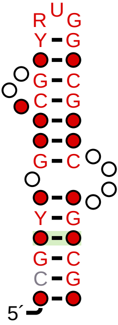

HMP2_15061_1

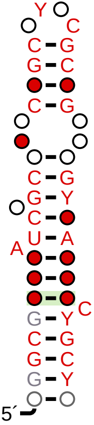

HMP2_15062_1

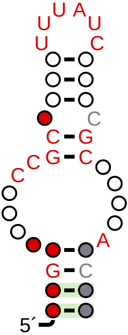

HMP2_15064_1

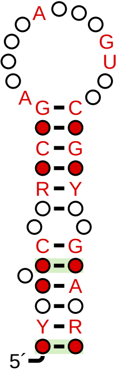

HMP2_15069_1

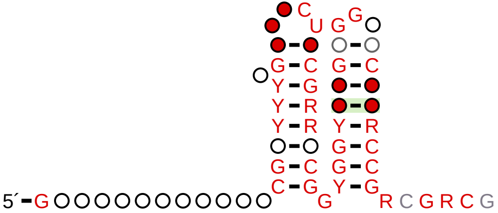

HMP2_15070_1

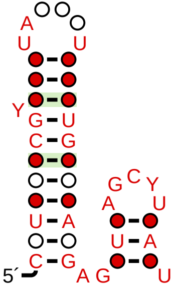

HMP2_15074_1

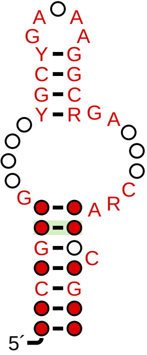

HMP2_15082_1

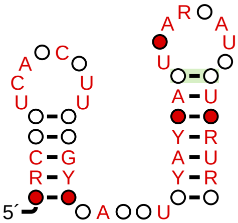

HMP2_15083_1

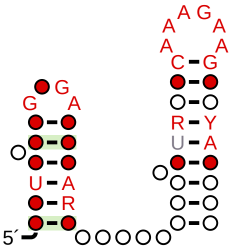

HMP2_15087_1

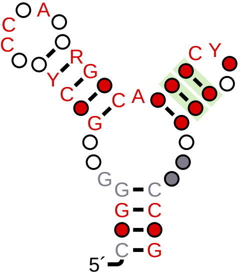

HMP2_1509_1

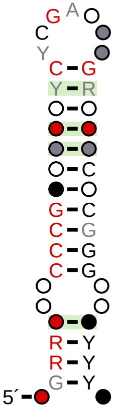

HMP2_15090_1

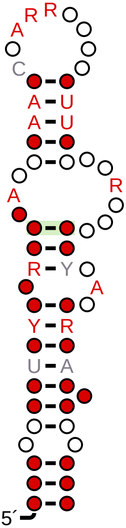

HMP2_15095_1

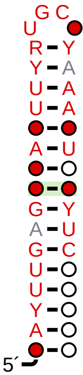

HMP2_1510_1

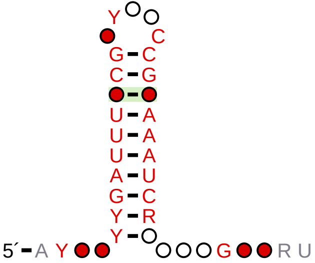

HMP2_15101_1

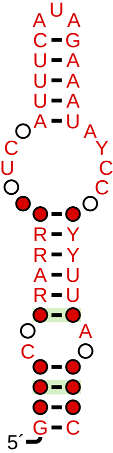

HMP2_15103_1

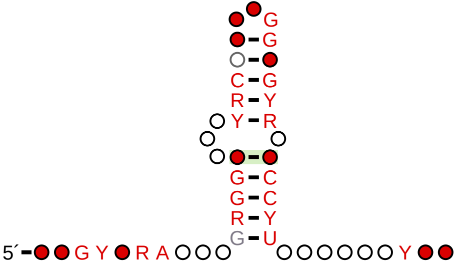

HMP2_15112_1

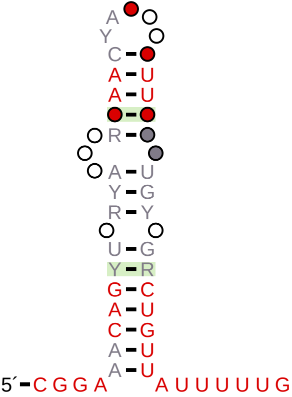

HMP2_15125_1

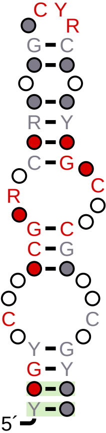

HMP2_15139_1

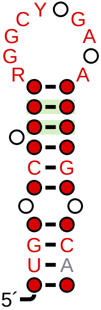

HMP2_15144_1

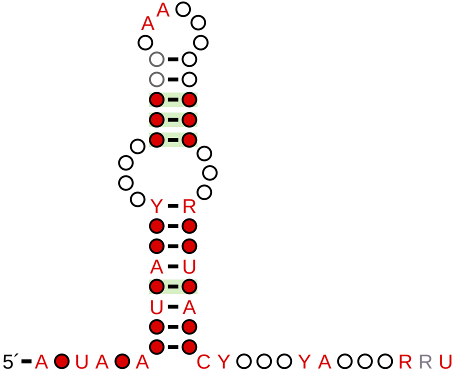

HMP2_15146_1

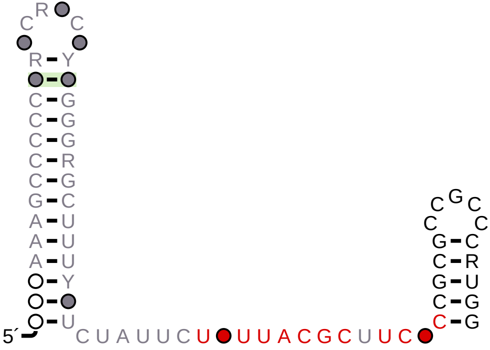

HMP2_1515_1

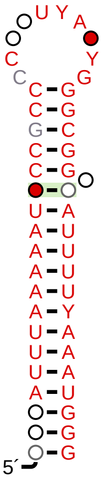

HMP2_15164_1

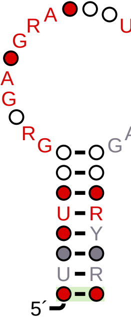

HMP2_15165_1

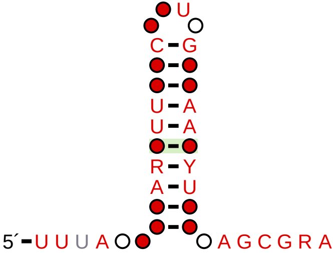

HMP2_15170_1

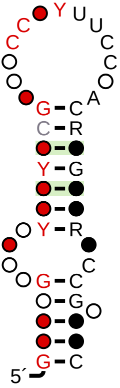

HMP2_15172_1

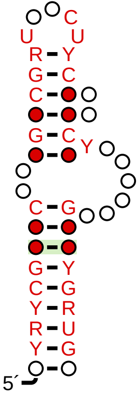

HMP2_15178_1

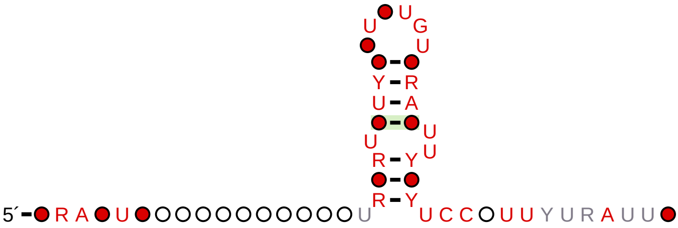

HMP2_15179_1

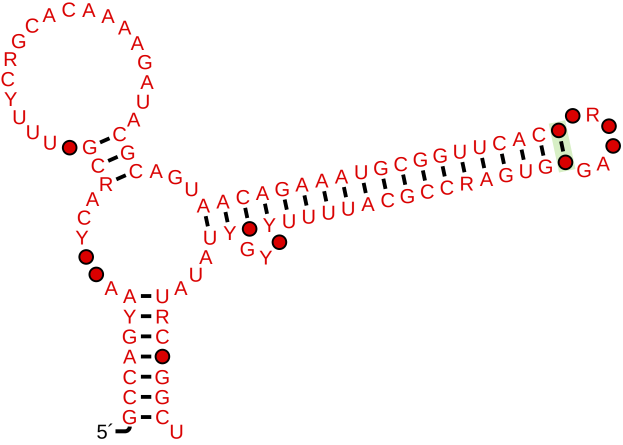

HMP2_1518_1

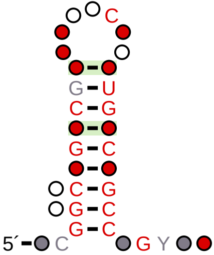

HMP2_15184_1

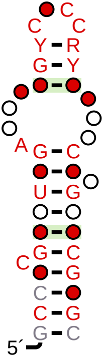

HMP2_15187_1

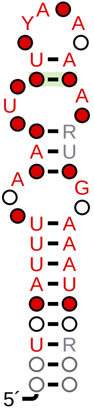

HMP2_1519_1

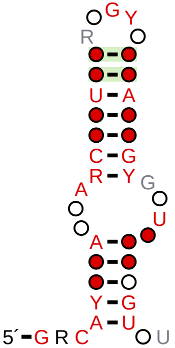

HMP2_15190_1

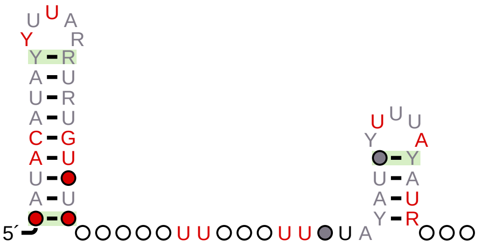

HMP2_15191_1

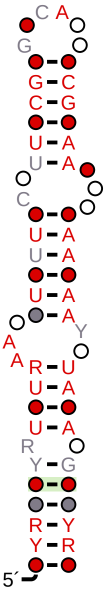

HMP2_15206_1

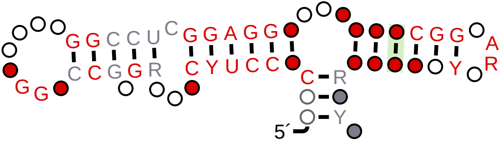

HMP2_15219_1

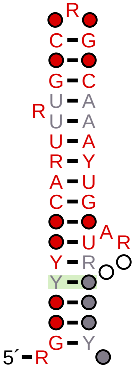

HMP2_15221_1

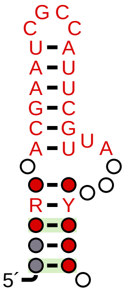

HMP2_15226_1

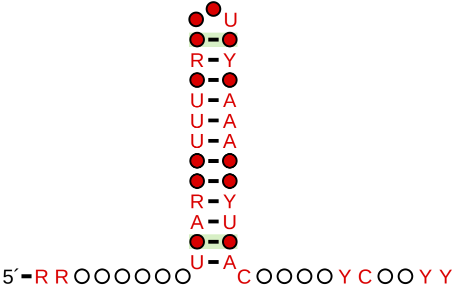

HMP2_15229_1

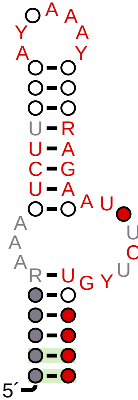

HMP2_1524_1

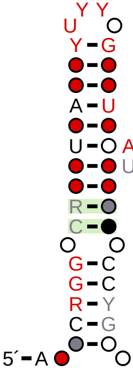

HMP2_15241_1

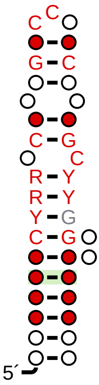

HMP2_15242_1

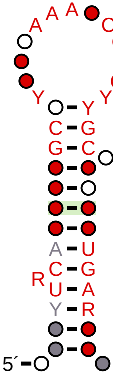

HMP2_15243_1

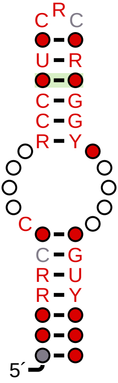

HMP2_15246_1

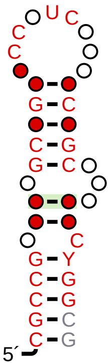

HMP2_1525_1

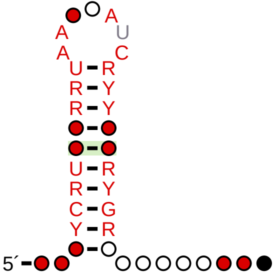

HMP2_15253_1

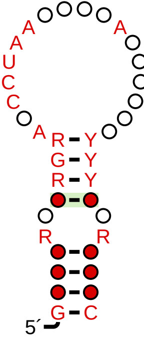

HMP2_15272_1

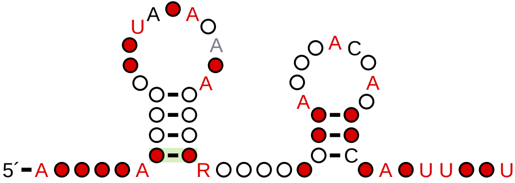

HMP2_15278_1

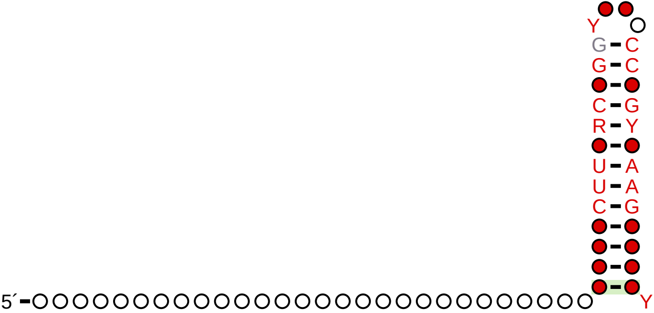

HMP2_15281_1

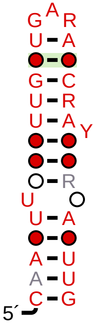

HMP2_15290_1

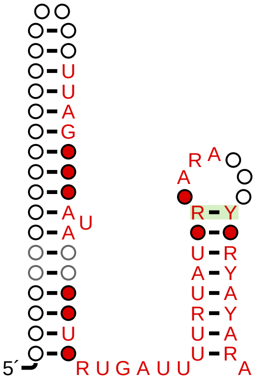

HMP2_15294_1

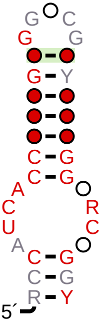

HMP2_15296_1

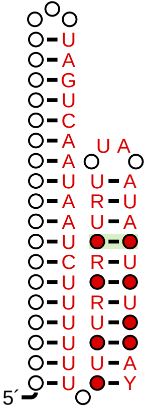

HMP2_15299_1

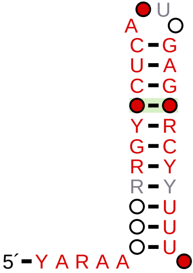

HMP2_1530_1

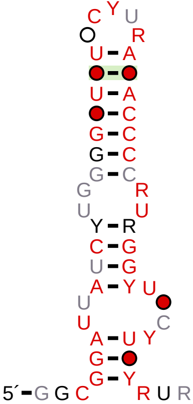

HMP2_15301_1

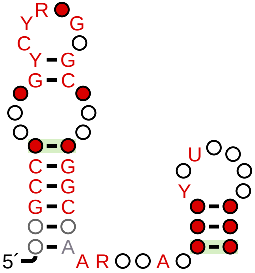

HMP2_15302_1

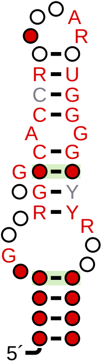

HMP2_15303_1

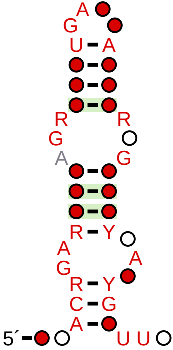

HMP2_15311_1

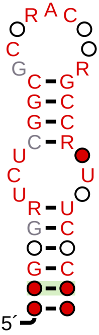

HMP2_1532_1

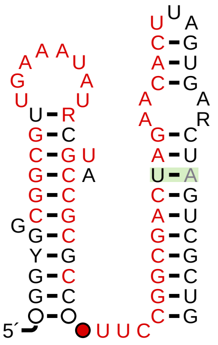

HMP2_15321_1

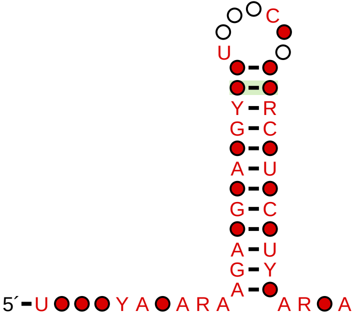

HMP2_15322_1

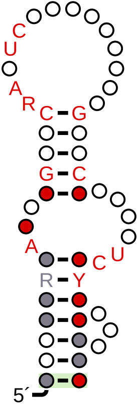

HMP2_15323_1

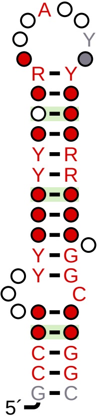

HMP2_15343_1

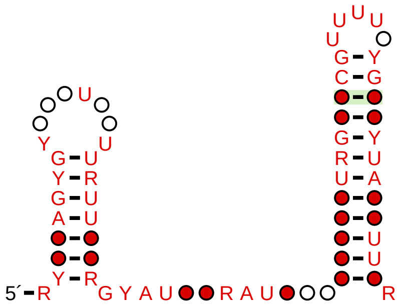

HMP2_1535_1

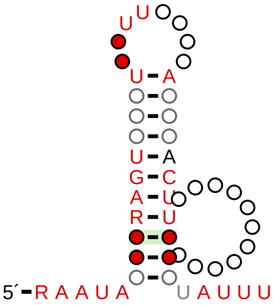

HMP2_15350_1

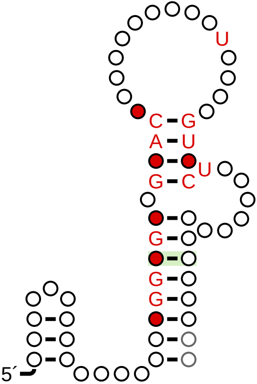

HMP2_15352_1

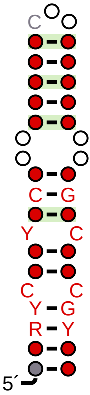

HMP2_15354_1

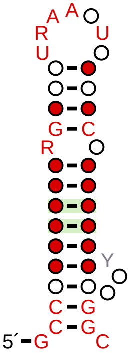

HMP2_15360_1

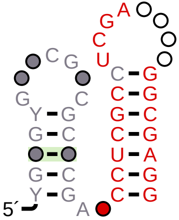

HMP2_15368_1

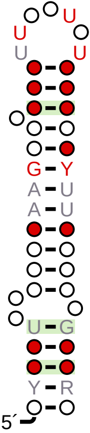

HMP2_15387_1

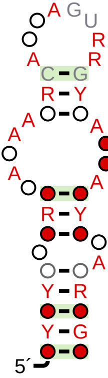

HMP2_15389_1

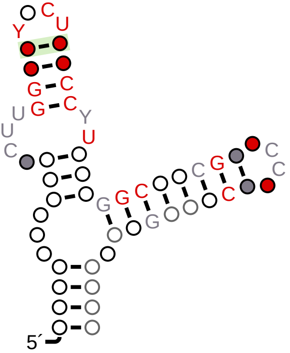

HMP2_15392_1

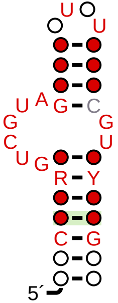

HMP2_15393_1

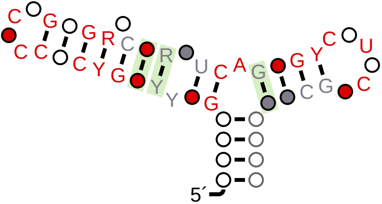

HMP2_15395_1

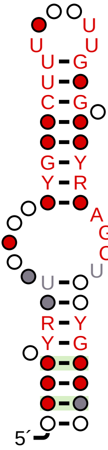

HMP2_15402_1

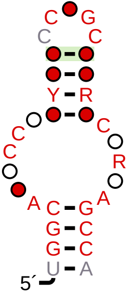

HMP2_15407_1

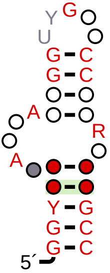

HMP2_15414_1

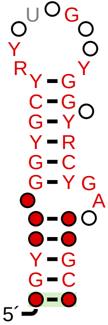

HMP2_15416_1

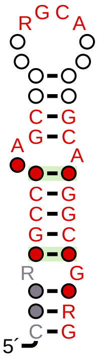

HMP2_15417_1

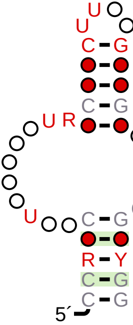

HMP2_15424_1

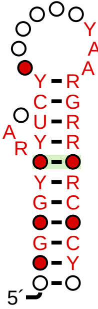

HMP2_15431_1

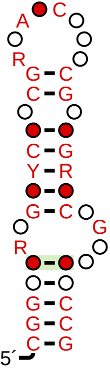

HMP2_15432_1

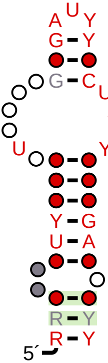

HMP2_15433_1

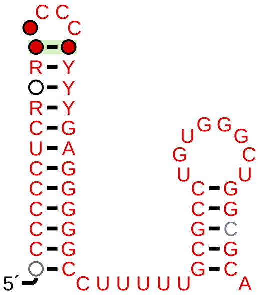

HMP2_15437_1

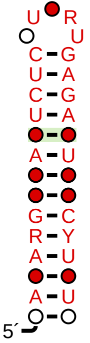

HMP2_15440_1

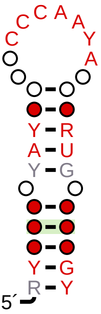

HMP2_15441_1

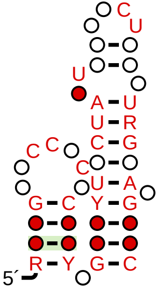

HMP2_15443_1

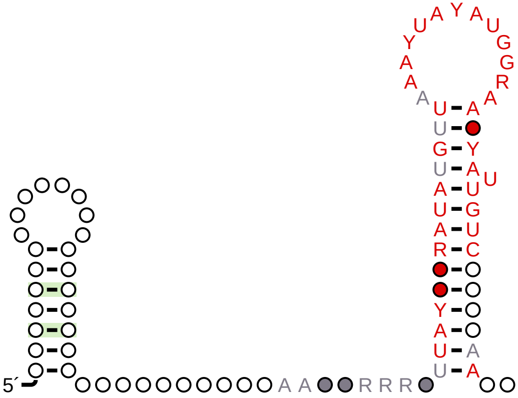

HMP2_15458_1

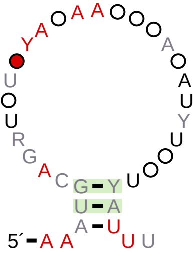

HMP2_15459_1

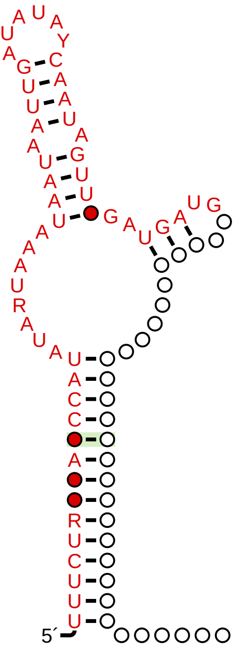

HMP2_15460_1

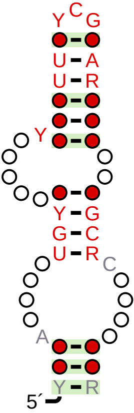

HMP2_15463_1

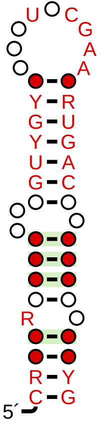

HMP2_15465_1

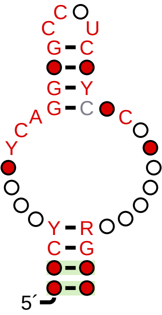

HMP2_15469_1

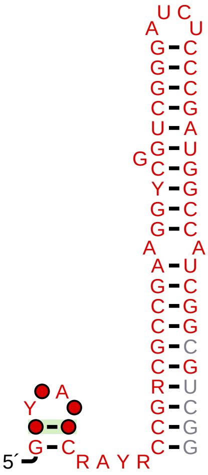

HMP2_15472_1

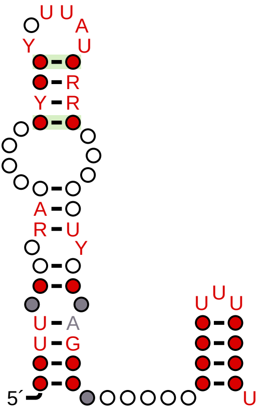

HMP2_15473_1

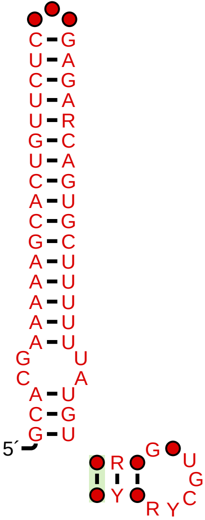

C G
U A
C G
U A
U R
G C
U A
C G
A U
C G
G C
A U
A U
A U
A U
A U
G U
C A
A U
C G
G U
5´
R G U G C Y R Y

HMP2_15478_1

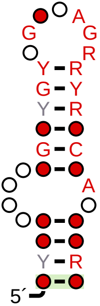

HMP2_1548_1

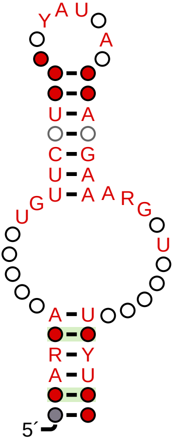

HMP2_15489_1

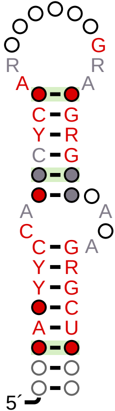

HMP2_15491_1

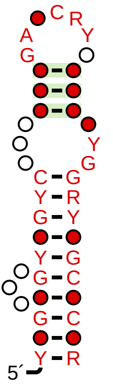

HMP2_15497_1

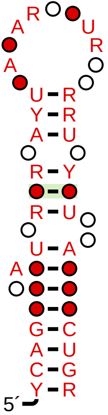

HMP2_1550_1

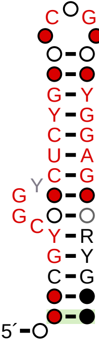

HMP2_15501_1

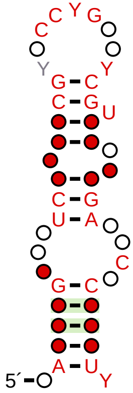

HMP2_15506_1

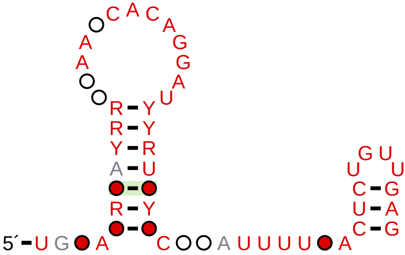

HMP2_15507_1

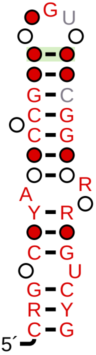

HMP2_15514_1

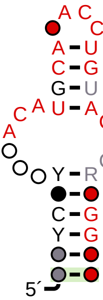

HMP2_1552_1

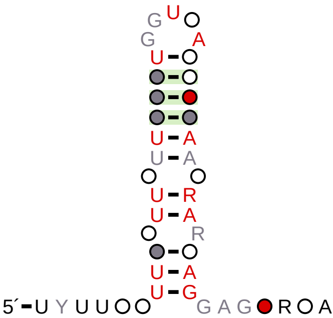

HMP2_15524_1

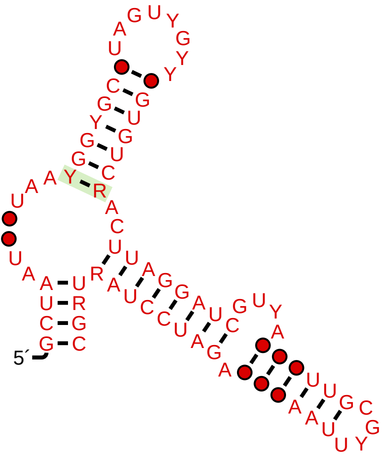

HMP2_15525_1

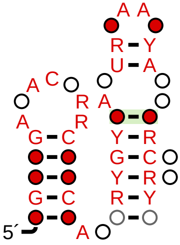

HMP2_15528_1

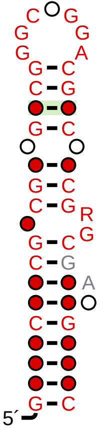

HMP2_15551_1

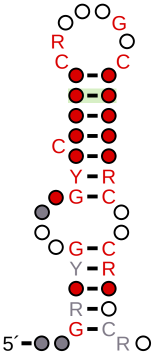

HMP2_15562_1

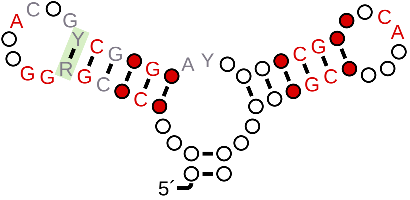

HMP2_15563_1

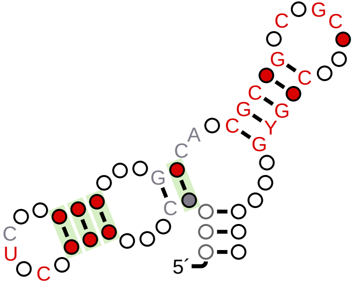

HMP2_1557_1

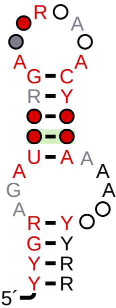

HMP2_15581_1

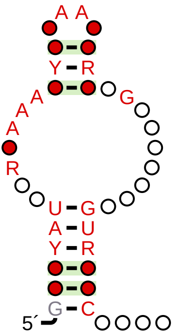

HMP2_15586_1

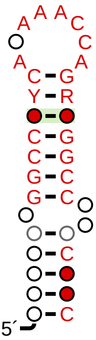

HMP2_15587_1

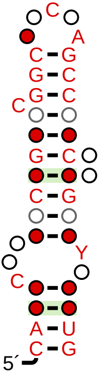

HMP2_15588_1

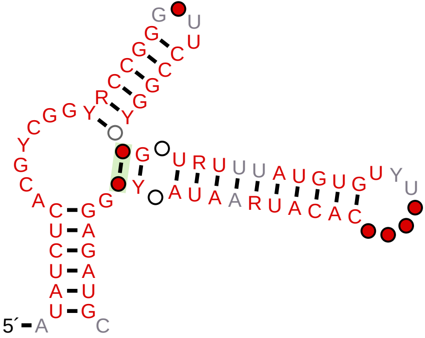

HMP2_15591_1

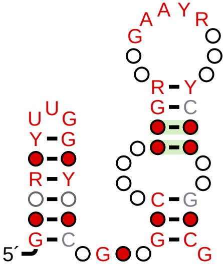

HMP2_15594_1

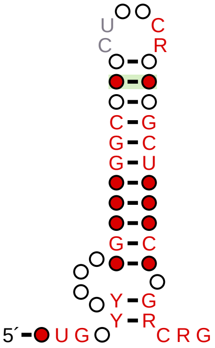

HMP2_15595_1

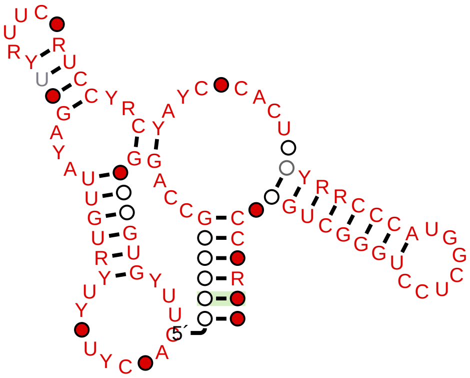

HMP2_15599_1

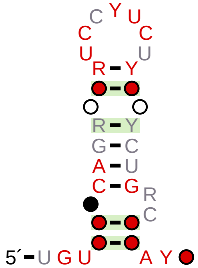

HMP2_1560_1

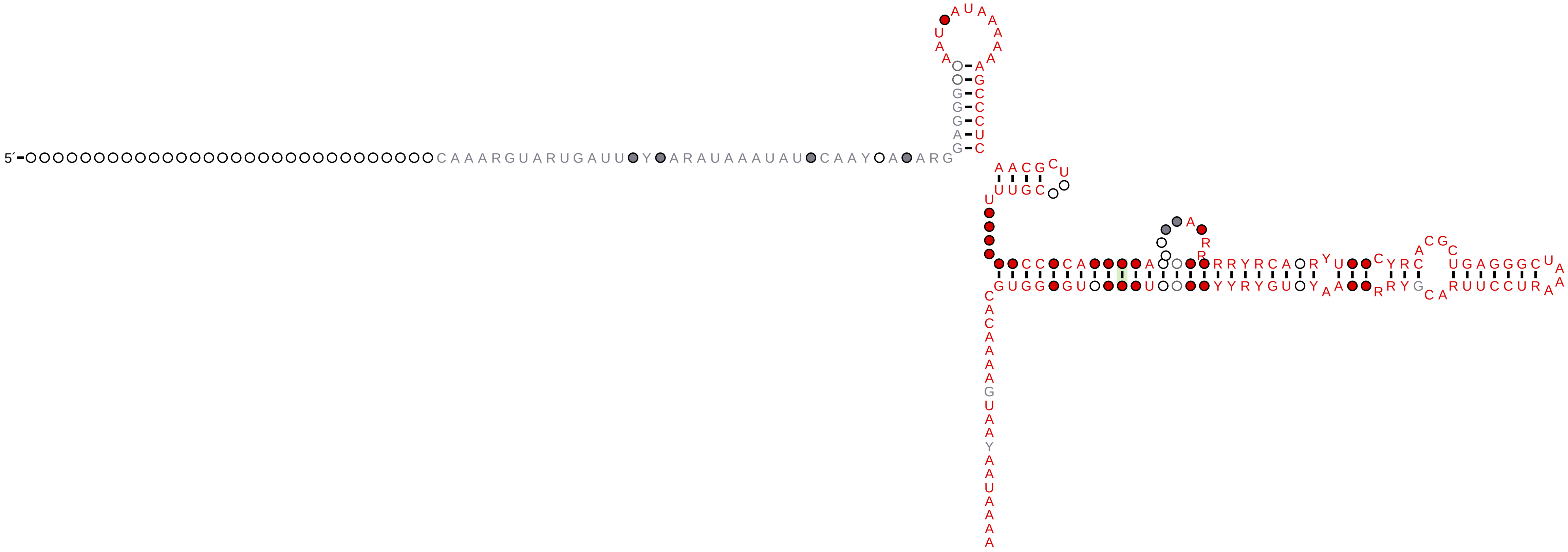

HMP2_15607_1

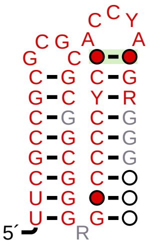

HMP2_15610_1

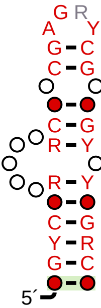

HMP2_15617_1

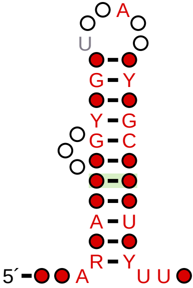

HMP2_1562_1

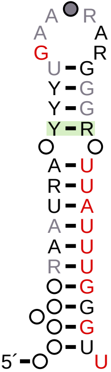

HMP2_15627_1

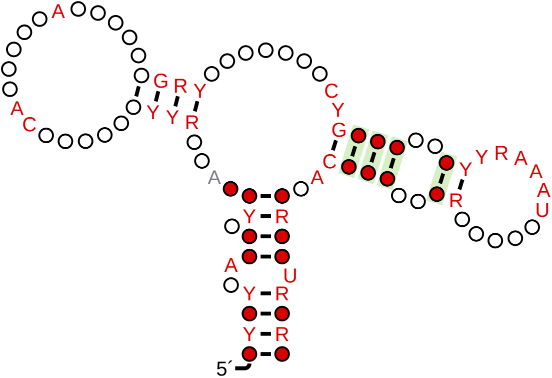

HMP2_15628_1

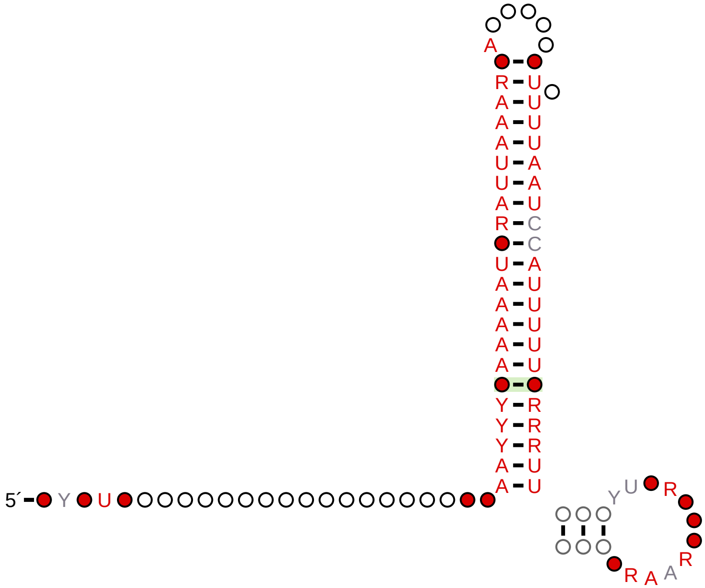

HMP2_15635_1

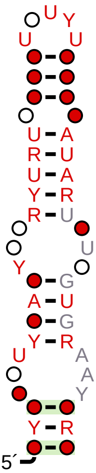

HMP2_15641_1

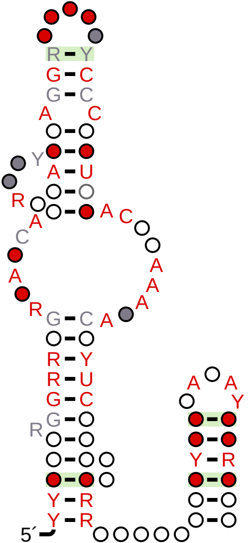

HMP2_15642_1

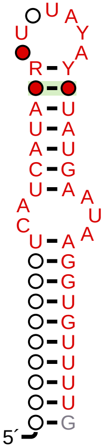

HMP2_15644_1

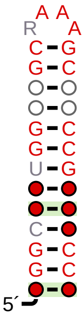

HMP2_15654_1

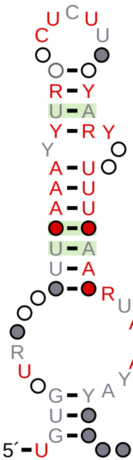

HMP2_15661_1

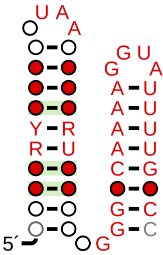

HMP2_15666_1

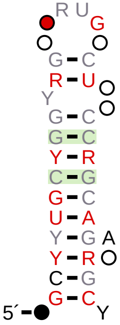

HMP2_15667_1

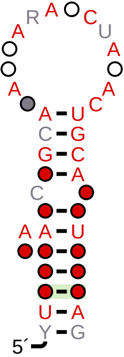

HMP2_15674_1

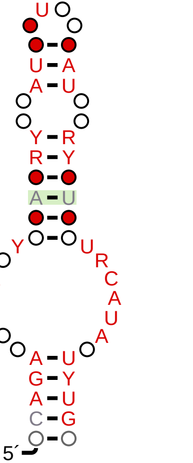

HMP2_15677_1

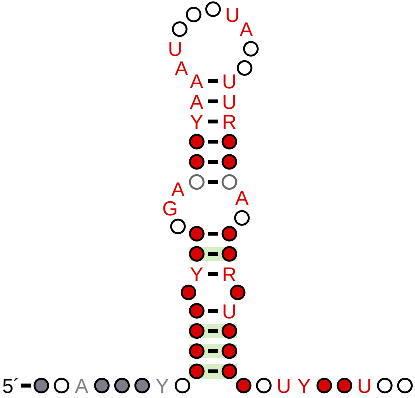

HMP2_15683_1

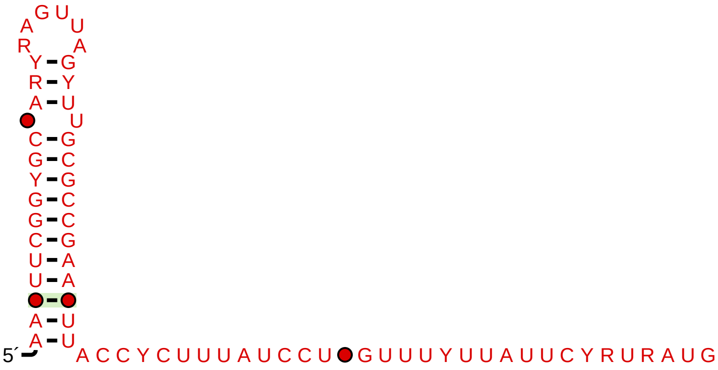

A G U U
R A
Y G
R Y
A U
U
C G
G C
Y G
G C
G C
C G
U A
U A
A U
A U
5´
A C C Y C U U U A U C C U G U U U Y U U A U U C Y R U R A U G

HMP2_15685_1

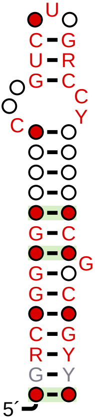

HMP2_15686_1

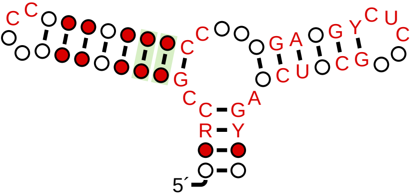

HMP2_1569_1

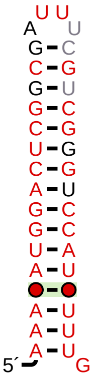

HMP2_15695_1

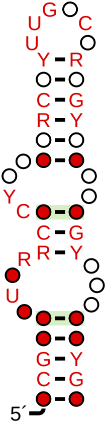

HMP2_15696_1

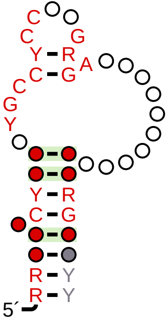

HMP2_15697_1

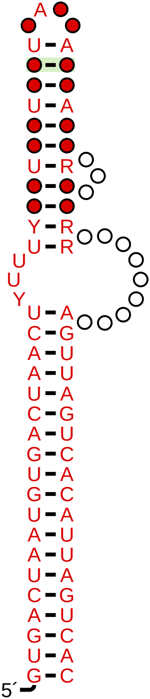

HMP2_15702_1

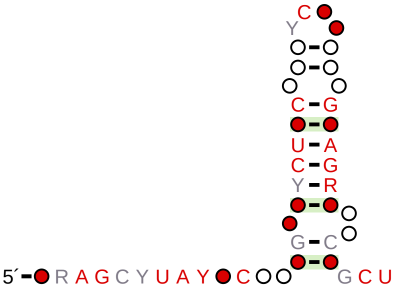

HMP2_15712_1

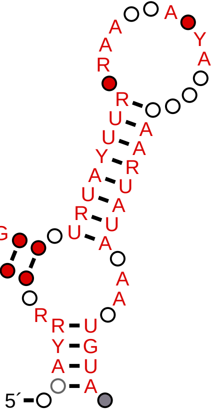

HMP2_15713_1

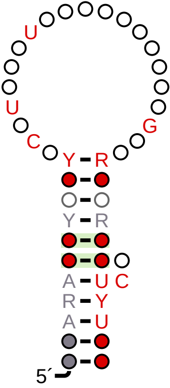

HMP2_15721_1

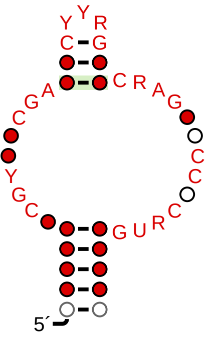

HMP2_15729_1

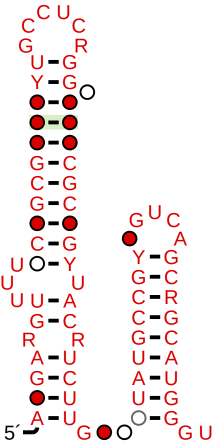

HMP2_15730_1

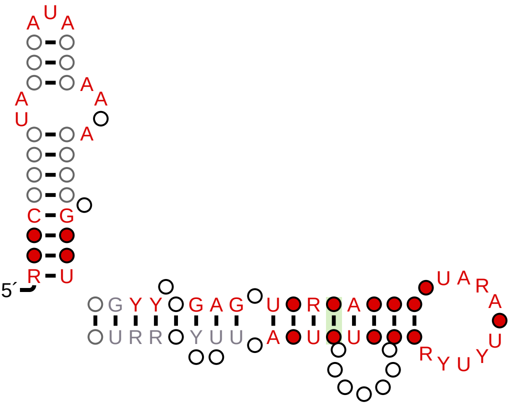

HMP2_15739_1

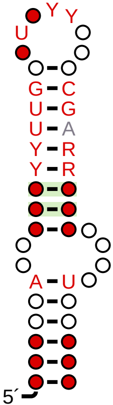

HMP2_15745_1

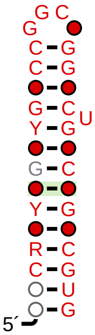

HMP2_15747_1

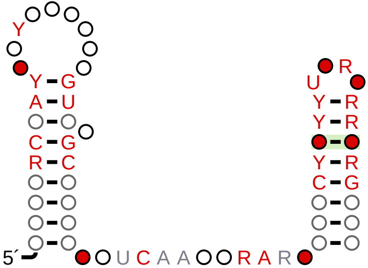

HMP2_15756_1

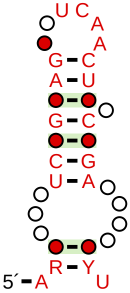

HMP2_15758_1

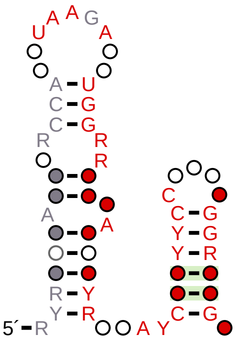

HMP2_15759_1

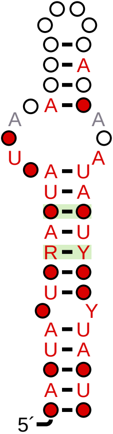

HMP2_15770_1

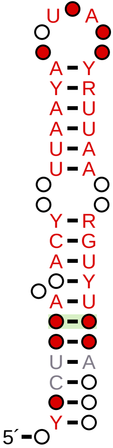

HMP2_15771_1

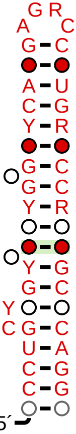

HMP2_15776_1

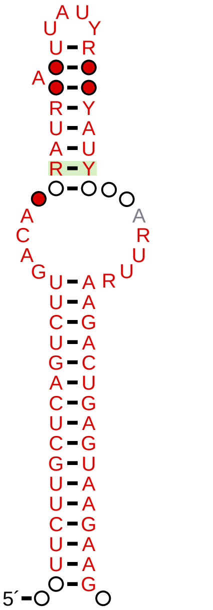

HMP2_15786_1

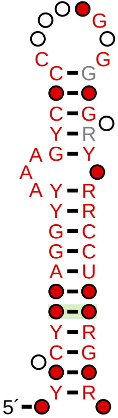

HMP2_15789_1

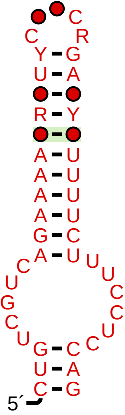

HMP2_1579_1

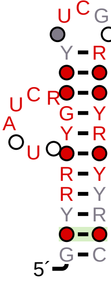

HMP2_15790_1

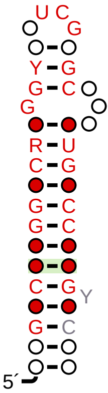

HMP2_15792_1

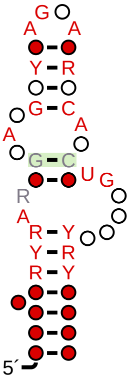

HMP2_15797_1

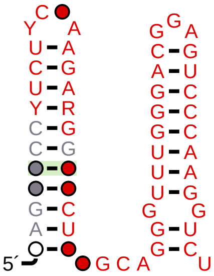

HMP2_15798_1

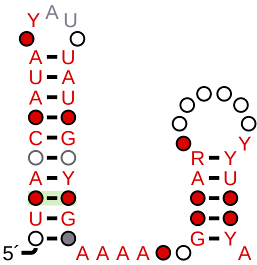

HMP2_15799_1

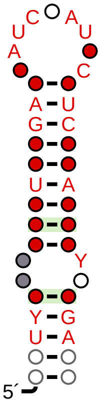

HMP2_15800_1

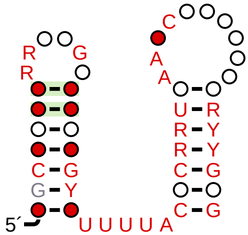

HMP2_15804_1

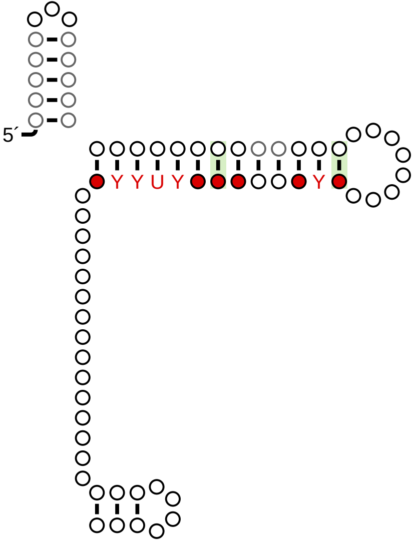

HMP2_15805_1

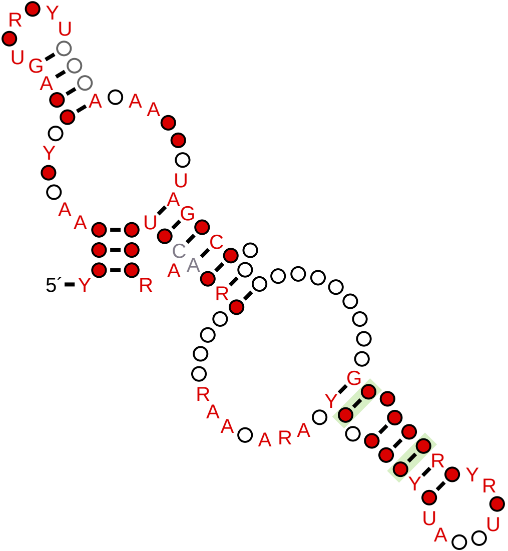

HMP2_15812_1

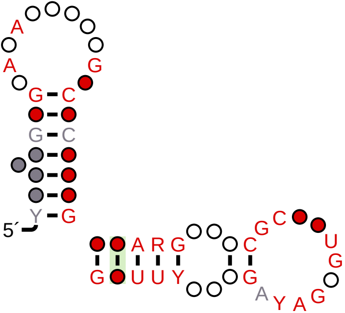

HMP2_15814_1

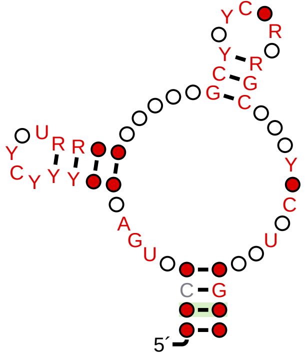

HMP2_15817_1

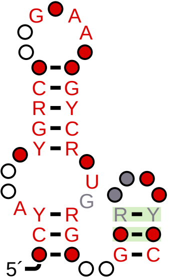

HMP2_15820_1

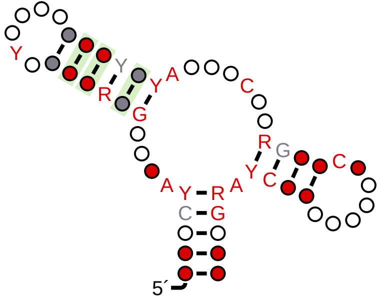

HMP2_15821_1

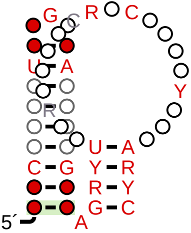

HMP2_15823_1

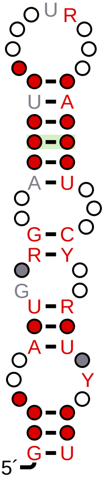

HMP2_15824_1

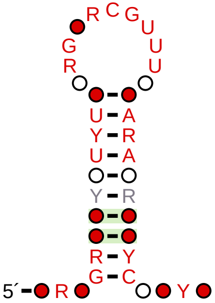

HMP2_1583_1

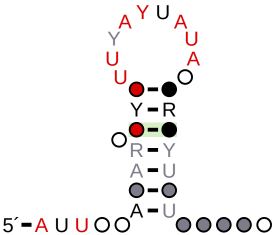

HMP2_15830_1

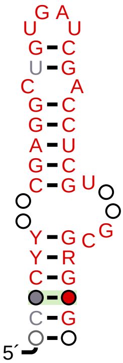

HMP2_15831_1

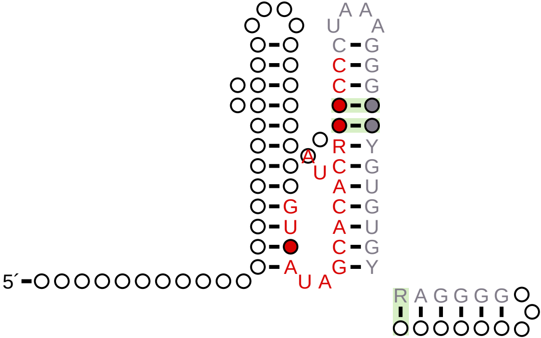

HMP2_15835_1

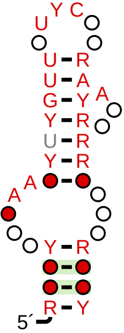

HMP2_15838_1

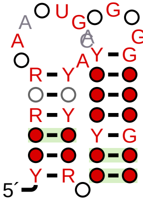

HMP2_1584_1

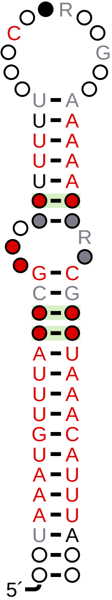

HMP2_15847_1

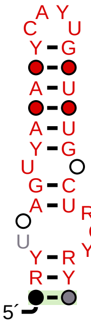

HMP2_15848_1

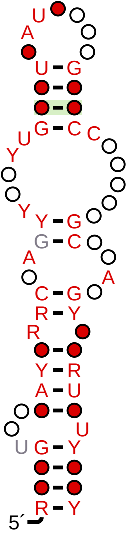

HMP2_15849_1

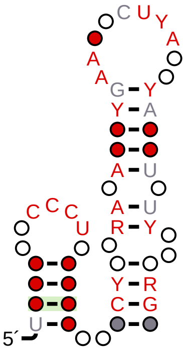

HMP2_15850_1

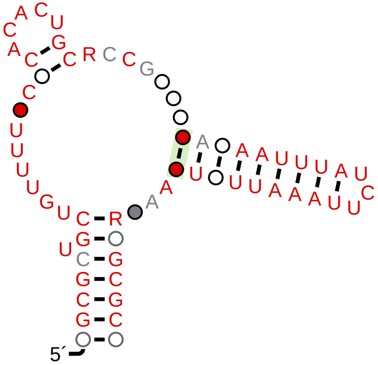

HMP2_15851_1

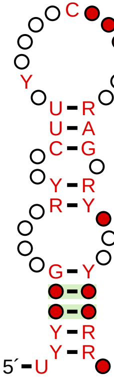

HMP2_15852_1

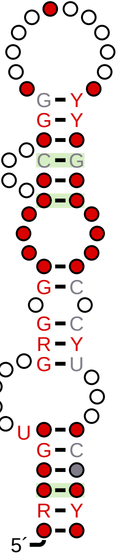

HMP2_15858_1

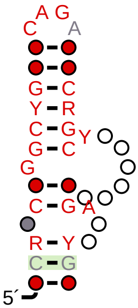

HMP2_15861_1

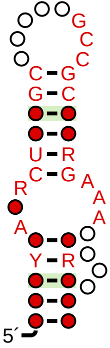

HMP2_15869_1

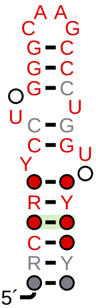

HMP2_1587_1

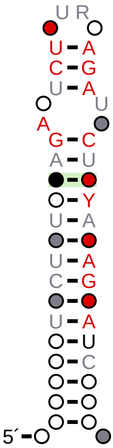

HMP2_1588_1

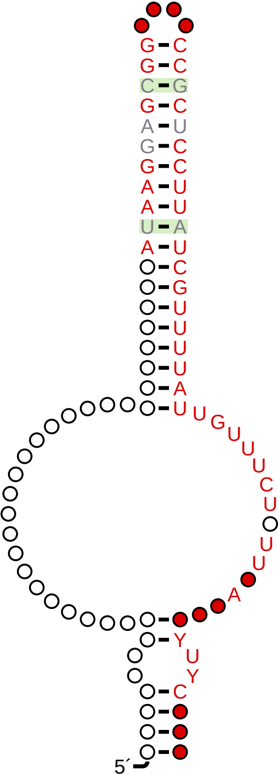

HMP2_15886_1

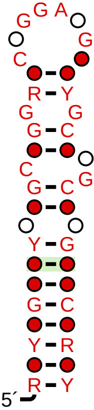

HMP2_15887_1

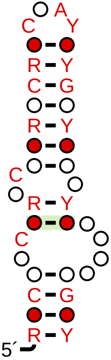

HMP2_15893_1

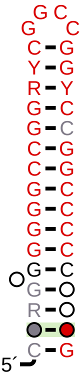

HMP2_15898_1

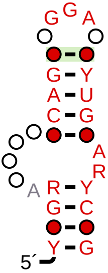

HMP2_15901_1

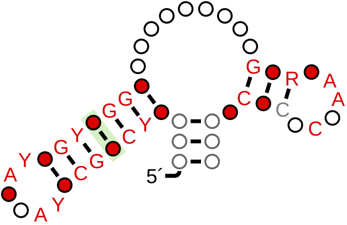

HMP2_15903_1

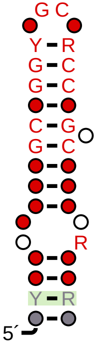

HMP2_15908_1

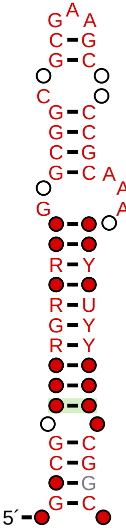

HMP2_15909_1

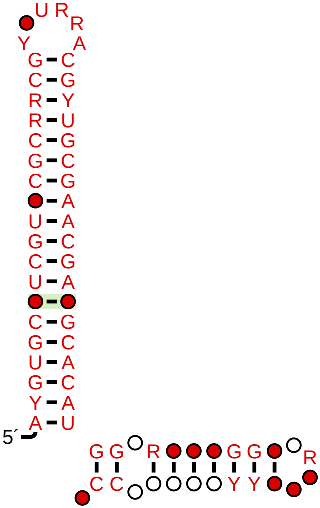

U R R
Y A
G C
C G
R Y
R U
C G
G C
C G
A
U A
G C
C G
U A
C G
G C
U A
G C
Y A
A U
5′
G G R G G R
C C Y Y

HMP2_15912_1

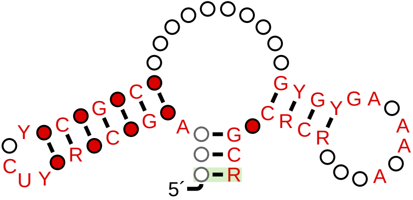

HMP2_15918_1

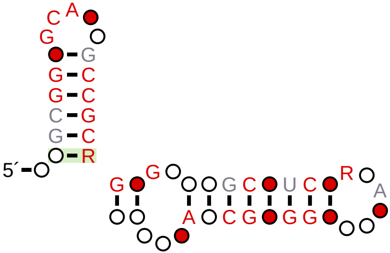

HMP2_15920_1

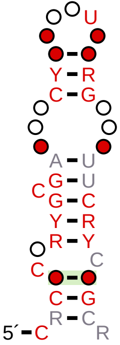

HMP2_15927_1

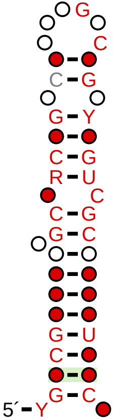

HMP2_15929_1

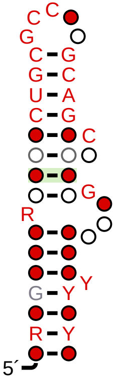

HMP2_15930_1

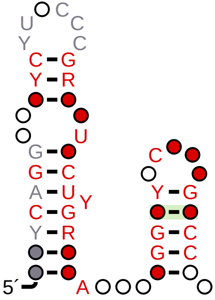

HMP2_15932_1

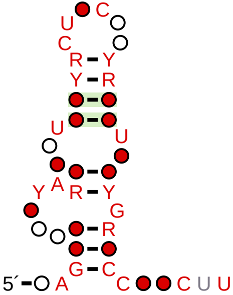

HMP2_15938_1

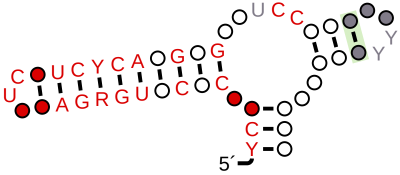

HMP2_15943_1

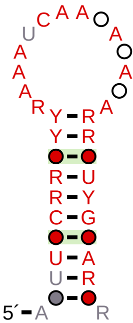

HMP2_15944_1

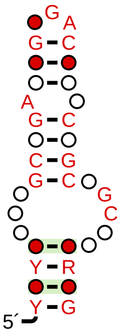

HMP2_15947_1

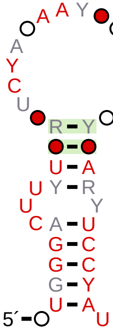

HMP2_15948_1

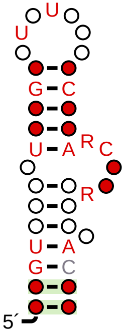

HMP2_15956_1

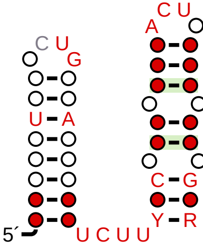

HMP2_15960_1

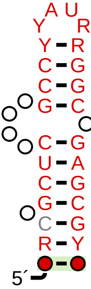

HMP2_15965_1

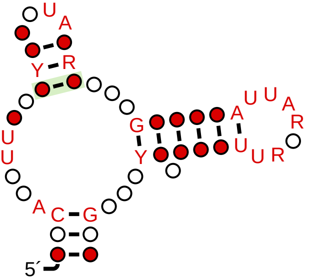

HMP2_15972_1

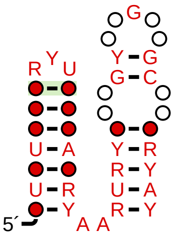

HMP2_15974_1

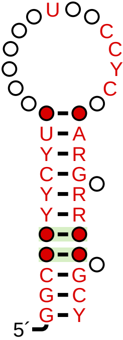

HMP2_15976_1

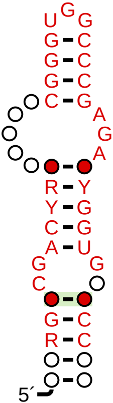

HMP2_1598_1

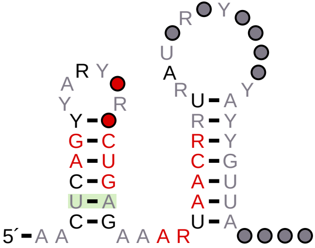

HMP2_15986_1

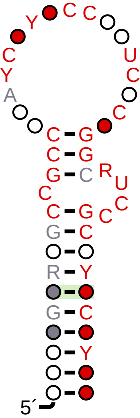

HMP2_15987_1

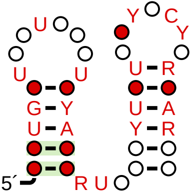

HMP2_15991_1

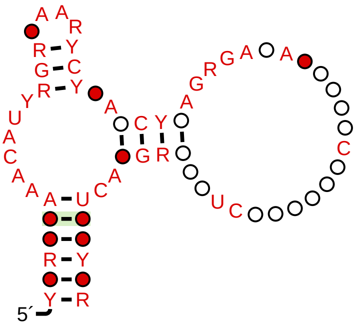

HMP2_16000_1

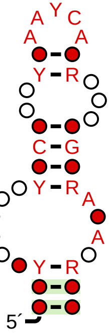

HMP2_16004_1

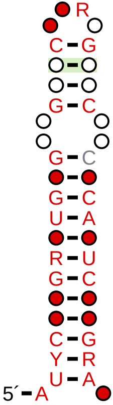

HMP2_16009_1

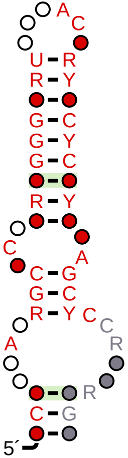

HMP2_16012_1

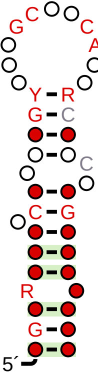

HMP2_16013_1

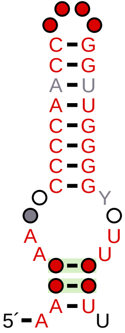

HMP2_1602_1

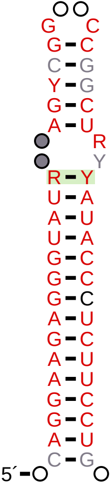

HMP2_1603_1

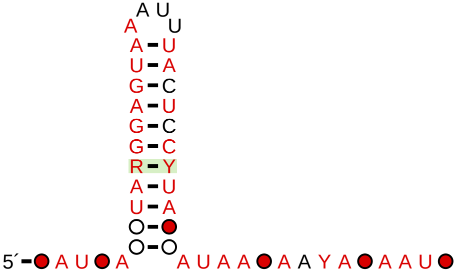

HMP2_16034_1

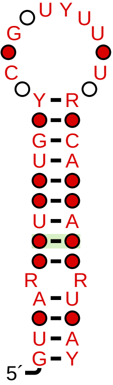

HMP2_16038_1

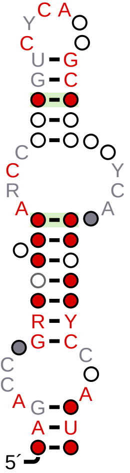

HMP2_16041_1

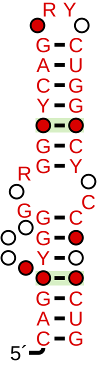

HMP2_1605_1

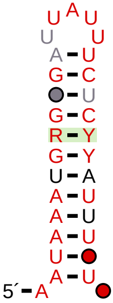

HMP2_16050_1

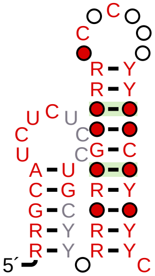

HMP2_16064_1

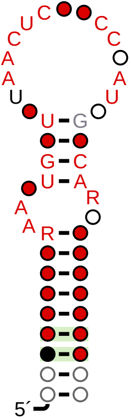

HMP2_16069_1

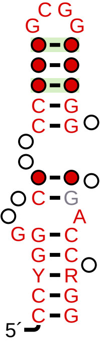

HMP2_1607_1

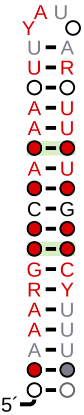

HMP2_16071_1

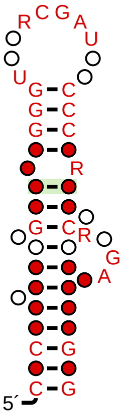

HMP2_16075_1

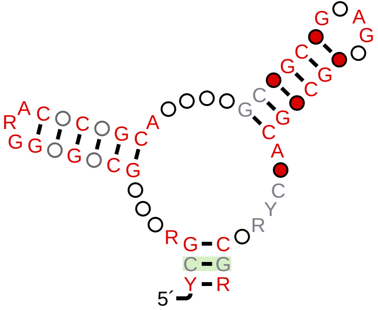

HMP2_16079_1

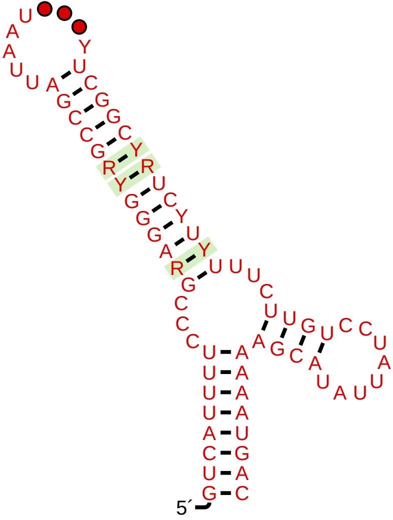

HMP2_1608_1

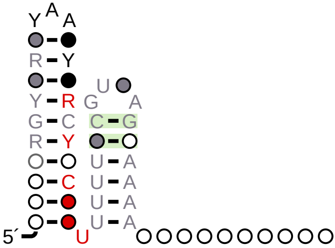

HMP2_16080_1

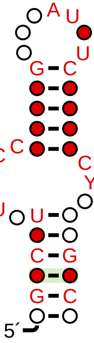

HMP2_16088_1

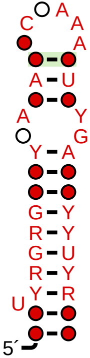

HMP2_1609_1

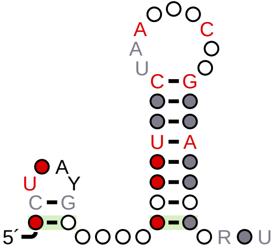

HMP2_16094_1

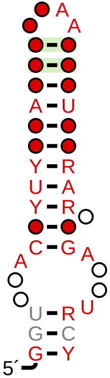

HMP2_16099_1

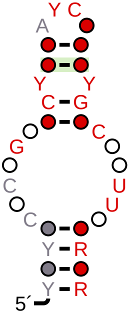

HMP2_161_1

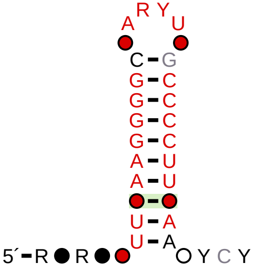

HMP2_16101_1

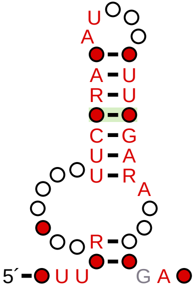

HMP2_16103_1

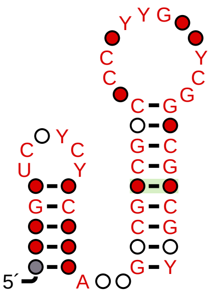

HMP2_16104_1

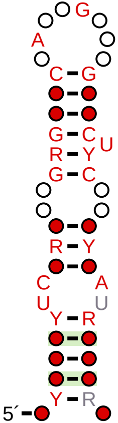

HMP2_16109_1

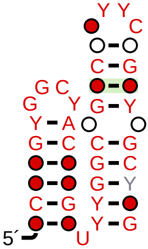

HMP2_16115_1

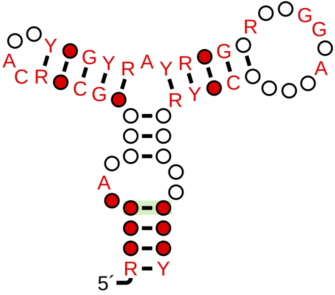

HMP2_16116_1

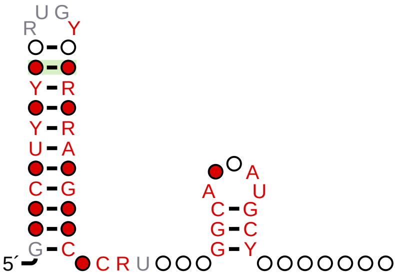

HMP2_16128_1

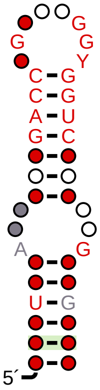

HMP2_16129_1

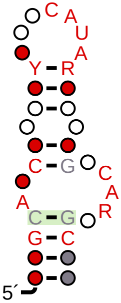

HMP2_16131_1

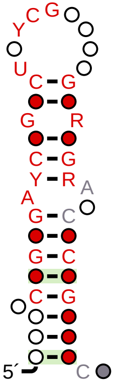

HMP2_16140_1

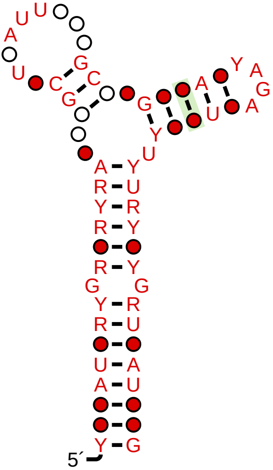

HMP2_16141_1

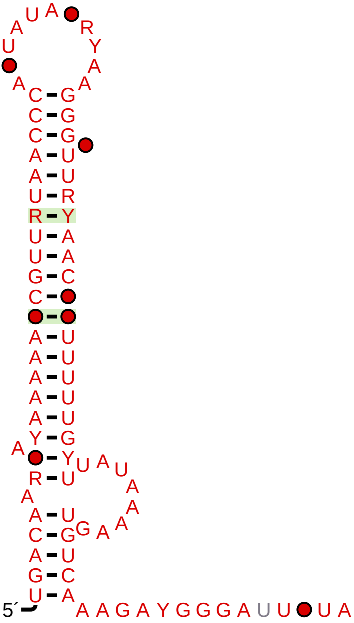

HMP2_16147_1

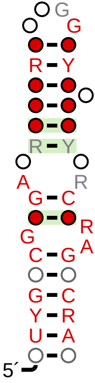

HMP2_1615_1

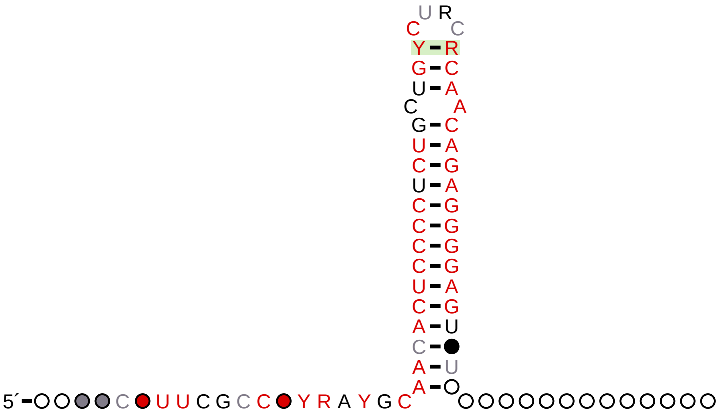

HMP2_16150_1

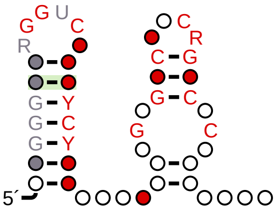

HMP2_16152_1

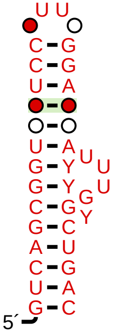

HMP2_1616_1

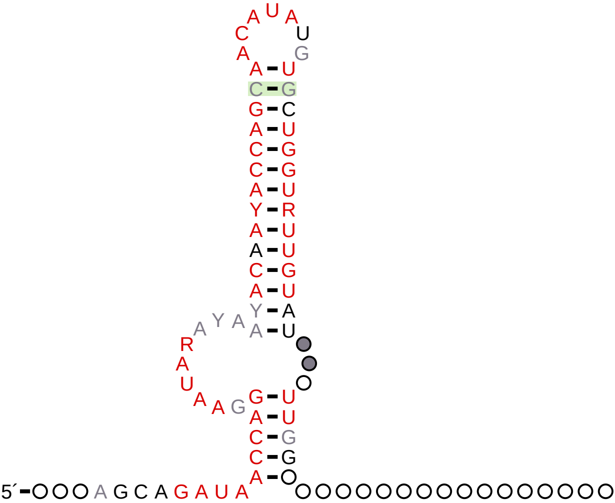

A U A
C U
A G
A U
C G
G C
A U
C G
C G
A U
Y R
A U
A U
C G
A U
Y A
A Y A
A U
R
A
U
A A G
G U
A U
C G
C G
A
5´ A G C A G A U A

HMP2_16160_1

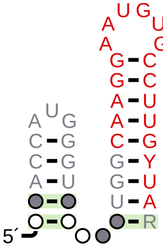

HMP2_16161_1

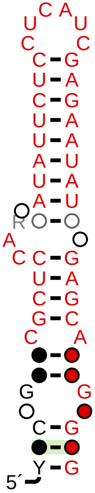

HMP2_16168_1

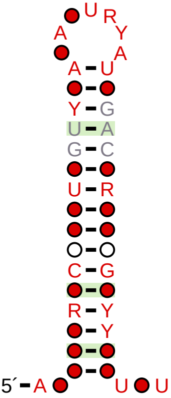

HMP2_1617_1

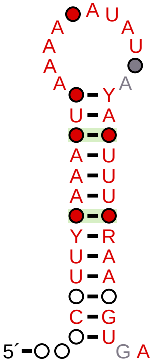

HMP2_16171_1

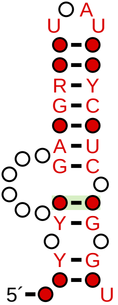

HMP2_16174_1

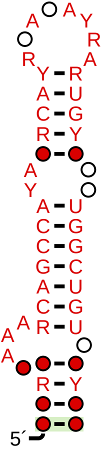

HMP2_16176_1

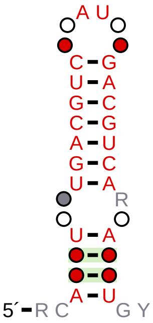

HMP2_16177_1

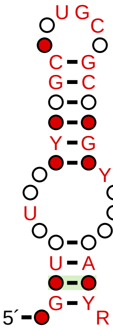

HMP2_16178_1

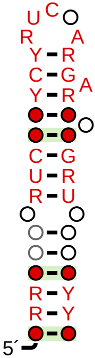

HMP2_1618_1

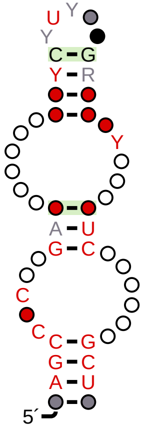

HMP2_162_1

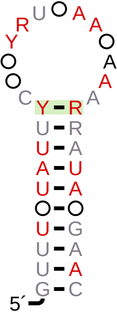

HMP2_16202_1

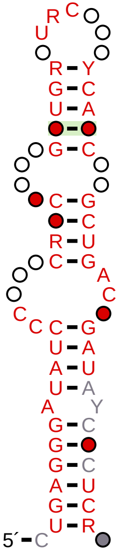

HMP2_16204_1

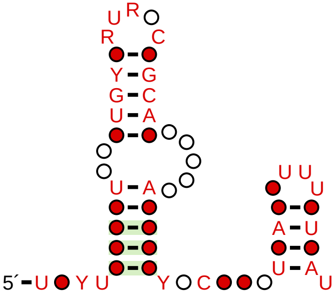

HMP2_16213_1

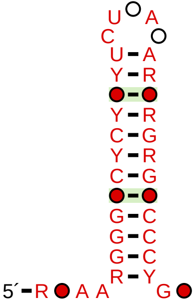

HMP2_16214_1

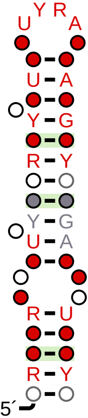

HMP2_16222_1

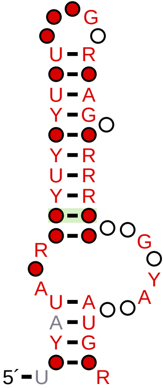

HMP2_16227_1

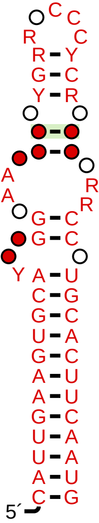

HMP2_1623_1

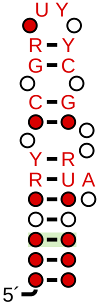

HMP2_16230_1

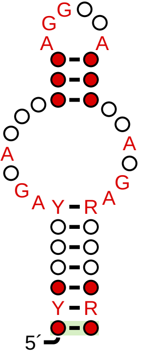

HMP2_16236_1

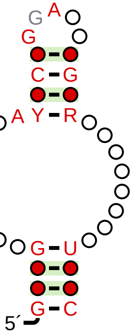

HMP2_16246_1

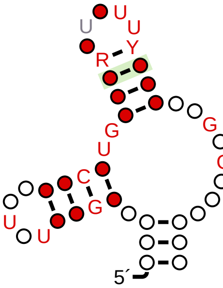

HMP2_16249_1

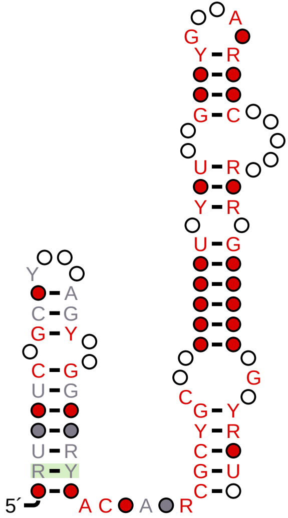

HMP2_16256_1

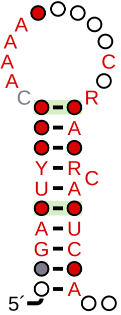

HMP2_16257_1

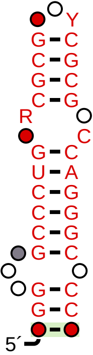

HMP2_16258_1

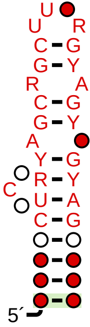

HMP2_16261_1

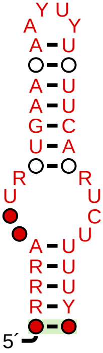

HMP2_16264_1

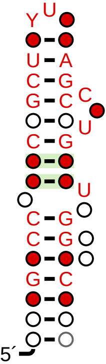

HMP2_16269_1

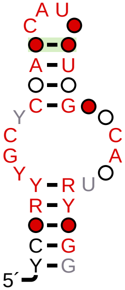

HMP2_1628_1

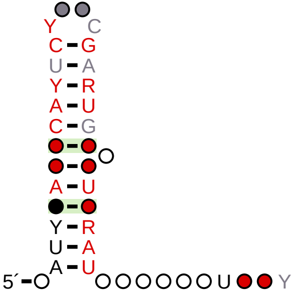

HMP2_16281_1

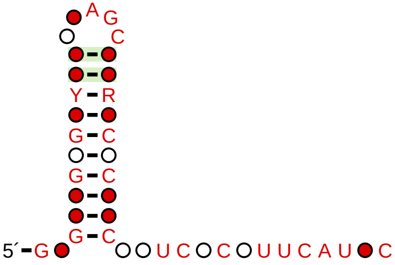

HMP2_16282_1

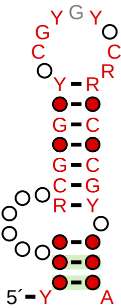

HMP2_16289_1

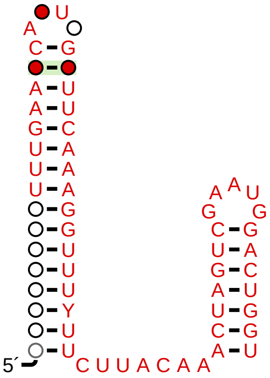
U
A
C G
A U
A U
G C
U A
U A
U A
G
G
U
U
U
Y
U
U
5´
C U U A C A A
A A U
G G
C G
U A
G C
A U
U G
C G
A U

HMP2_16296_1

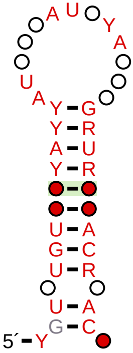

HMP2_16302_1

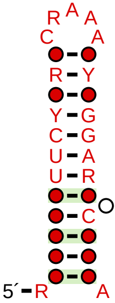

HMP2_16305_1

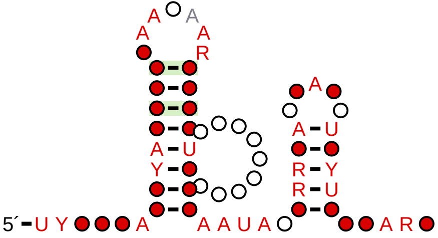

HMP2_16306_1

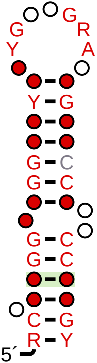

HMP2_16317_1

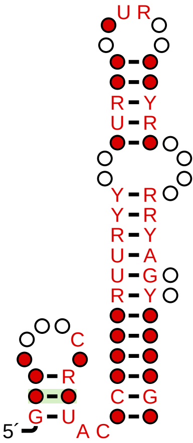

HMP2_16318_1

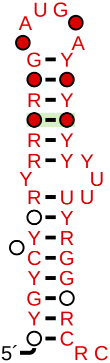

HMP2_16320_1

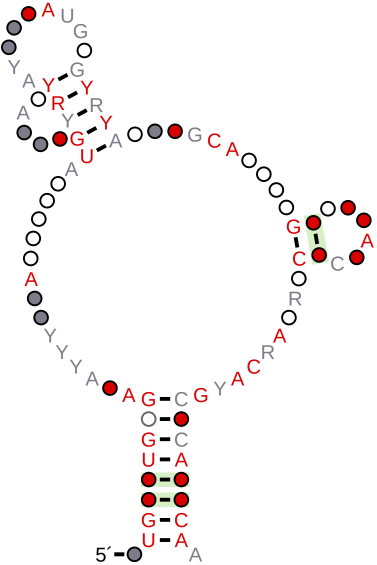

HMP2_16332_1

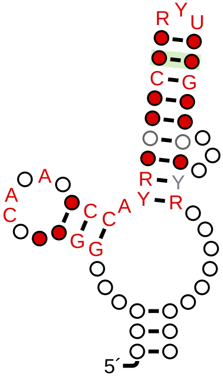

HMP2_16335_1

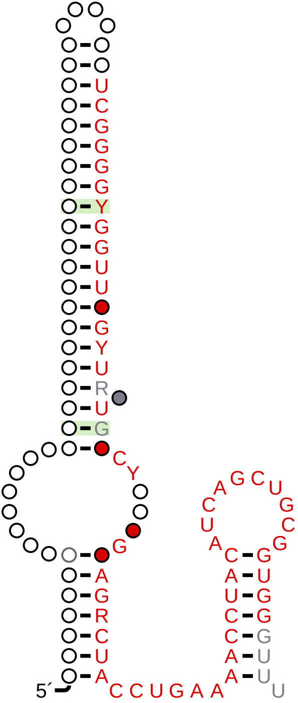

HMP2_16350_1

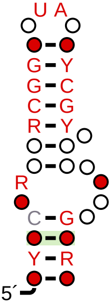

HMP2_16353_1

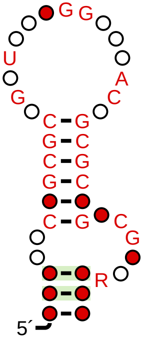

HMP2_16356_1

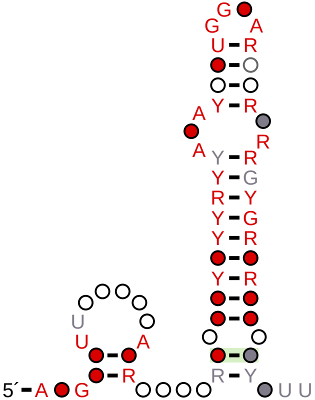

HMP2_16360_1

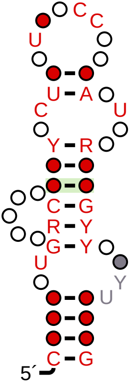

HMP2_16362_1

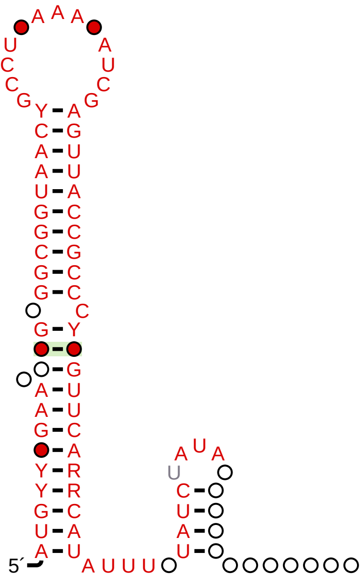

HMP2_16367_1

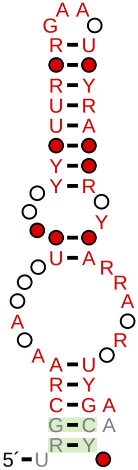

HMP2_16369_1

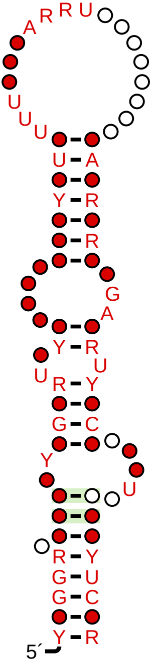

HMP2_1637_1

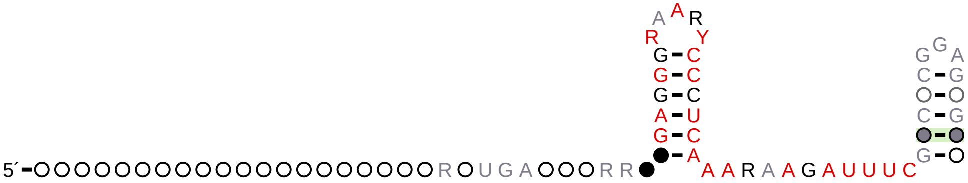

HMP2_16378_1

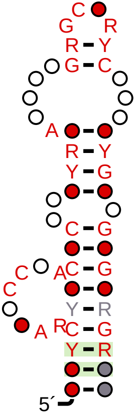

HMP2_16380_1

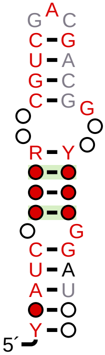

HMP2_16383_1

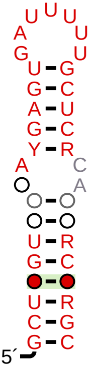

HMP2_16386_1

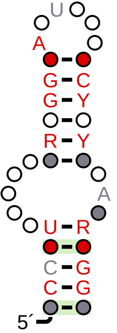

HMP2_16387_1

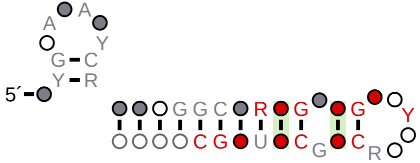

HMP2_16391_1

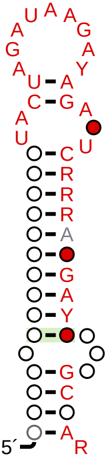

HMP2_16392_1

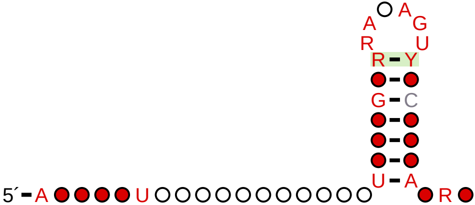

HMP2_16400_1

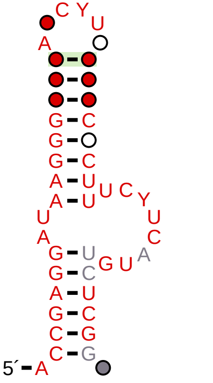

HMP2_16401_1

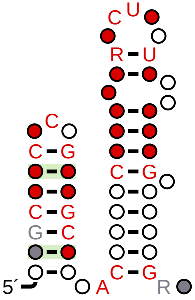

HMP2_16403_1

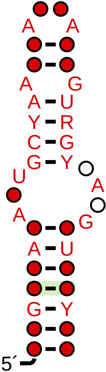

HMP2_16406_1

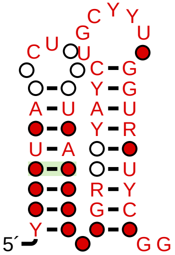

HMP2_16409_1

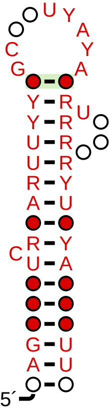

HMP2_16410_1

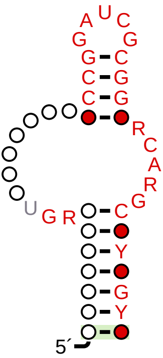

HMP2_16411_1

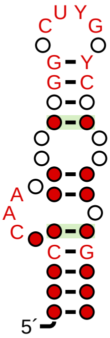

HMP2_16414_1

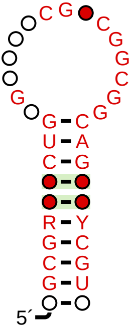

HMP2_16421_1

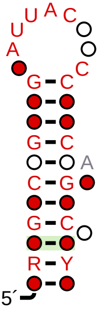

HMP2_16424_1

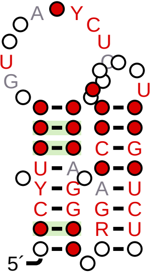

HMP2_16425_1

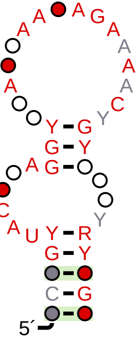

HMP2_16429_1

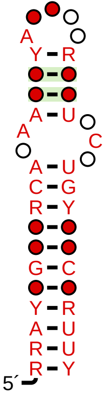

HMP2_16434_1

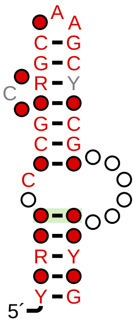

HMP2_16440_1

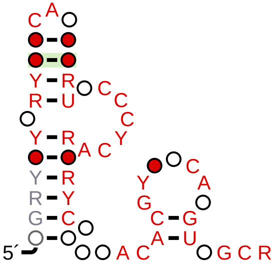

HMP2_16443_1

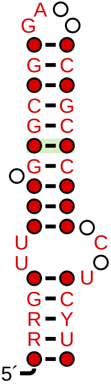

HMP2_16447_1

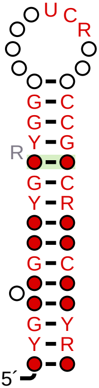

HMP2_16451_1

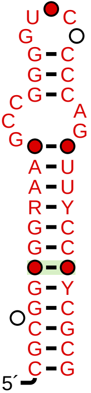

HMP2_16455_1

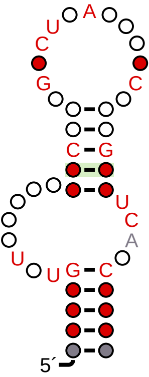

HMP2_16460_1

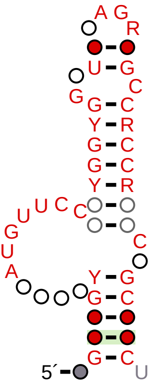

HMP2_16462_1

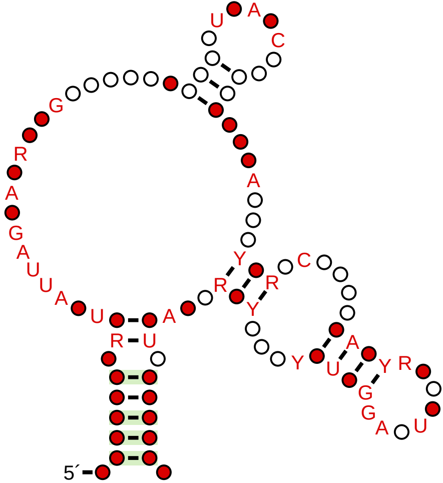

HMP2_16465_1

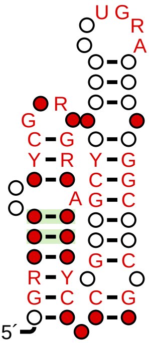

HMP2_16468_1

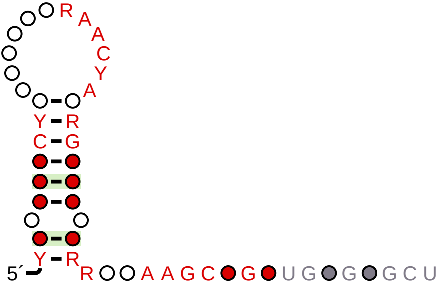

HMP2_16470_1

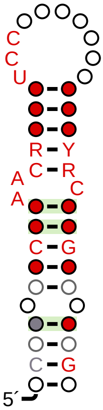

HMP2_16472_1

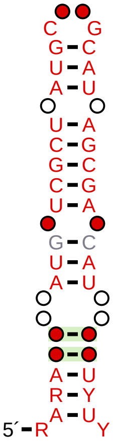

HMP2_16476_1

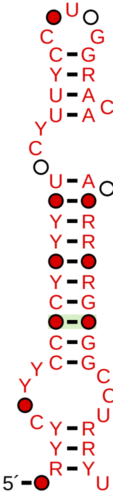

HMP2_16478_1

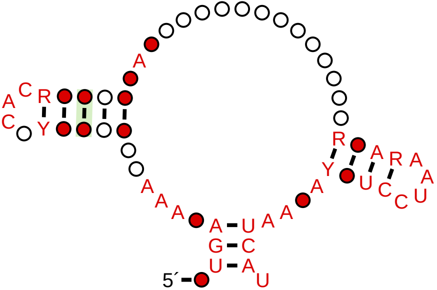

HMP2_1648_1

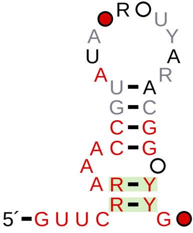

HMP2_16482_1

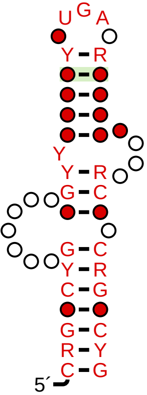

HMP2_16484_1

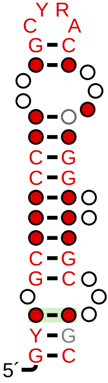

HMP2_16485_1

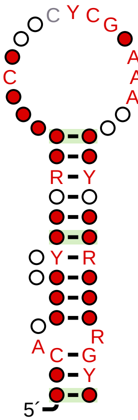

HMP2_16487_1

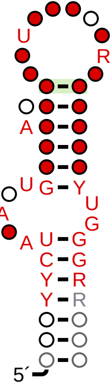

HMP2_16492_1

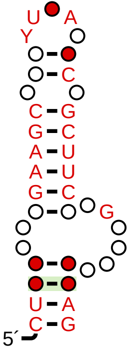

HMP2_16495_1

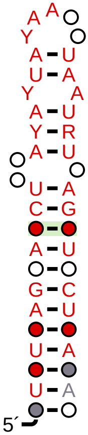

HMP2_16501_1

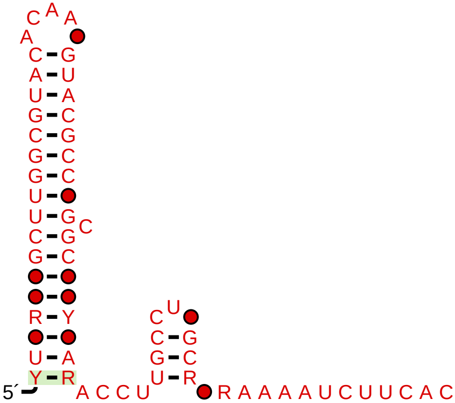

HMP2_16503_1

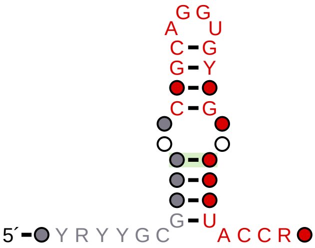

HMP2_16507_1

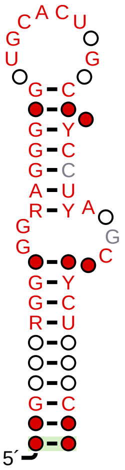

HMP2_16509_1

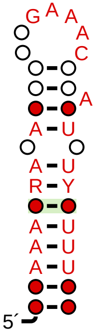

HMP2_16514_1

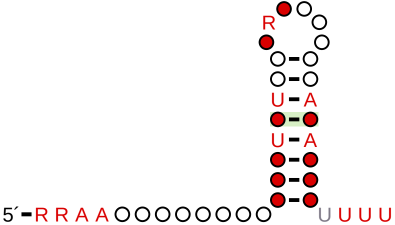

HMP2_16516_1

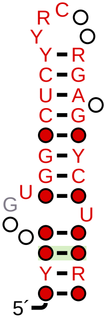

HMP2_16517_1

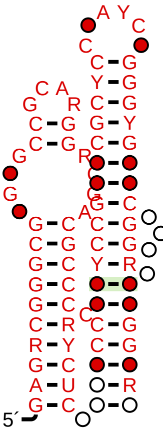

HMP2_16522_1

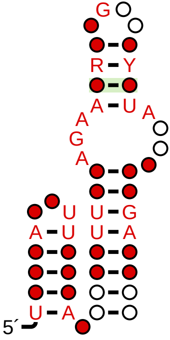

HMP2_16523_1

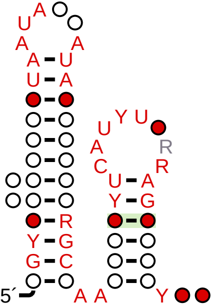

HMP2_1655_1

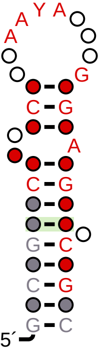

HMP2_1656_1

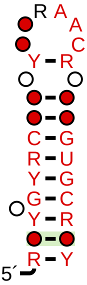

HMP2_1658_1

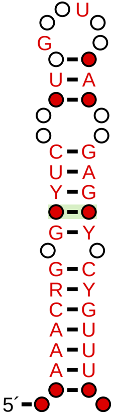

HMP2_1661_1

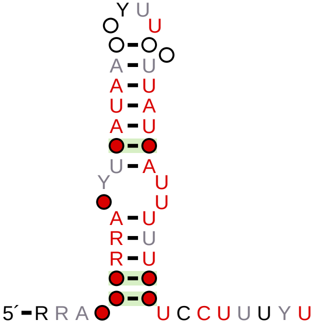

HMP2_1664_1

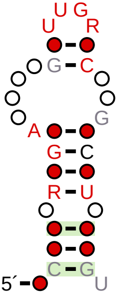

HMP2_167_1

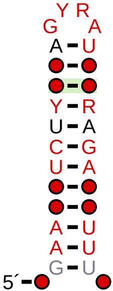

HMP2_1671_1

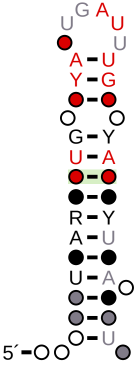

HMP2_1676_1

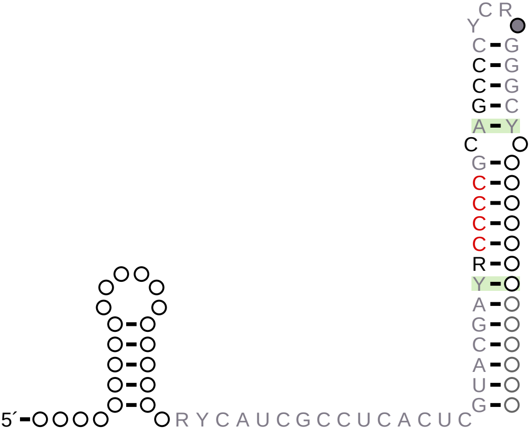

HMP2_1678_1

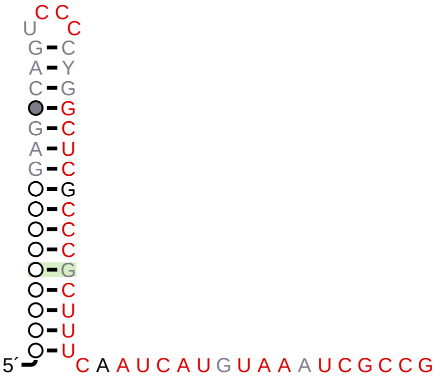

HMP2_1685_1

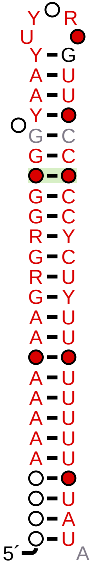

HMP2_1693_1

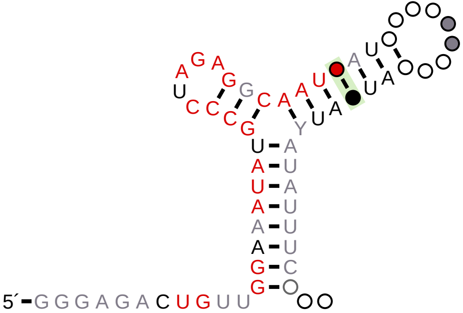

HMP2_1695_1

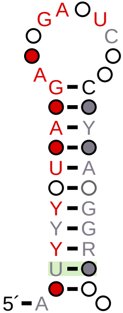

HMP2_1696_1

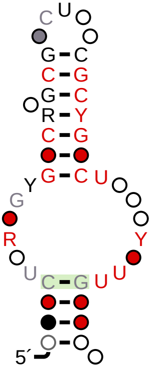

HMP2_1701_1

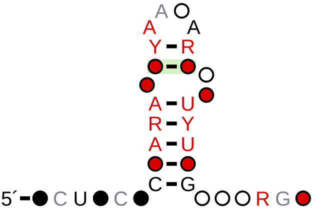

HMP2_171_1

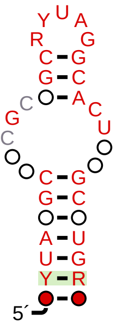

HMP2_1715_1

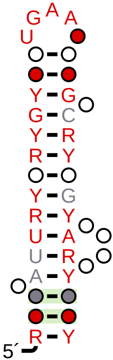

HMP2_1717_1

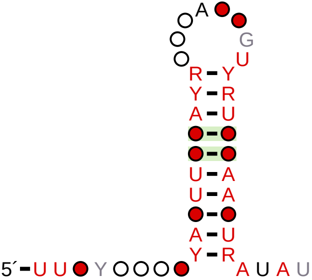

HMP2_1725_1

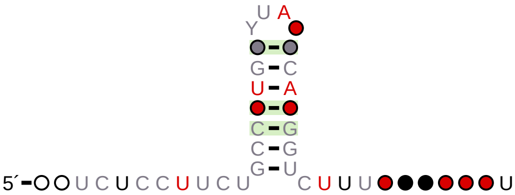

HMP2_1728_1

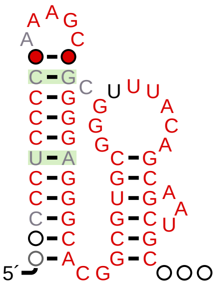

HMP2_1729_1

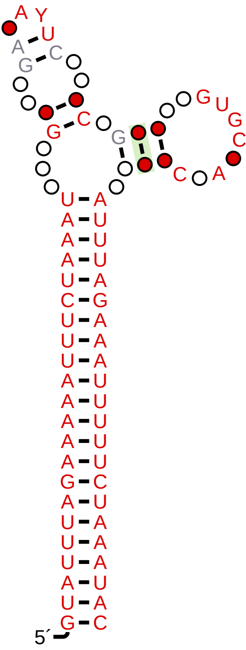

A
Y
U
A
C
G
C
C
G
G
G
U
G
C
C
A
U A
A U
A U
A U
U A
C G
U A
U A
U A
A U
A U
A U
A U
A U
G C
A U
U A
U A
U A
A U
U A
G C
5´

HMP2_1732_1

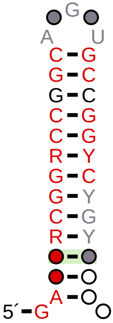

HMP2_1734_1

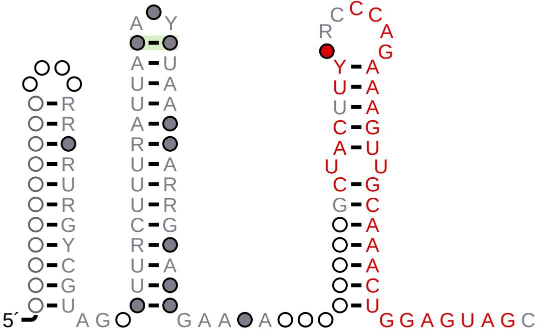

HMP2_1736_1

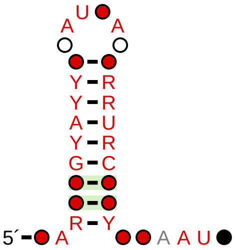

HMP2_1737_1

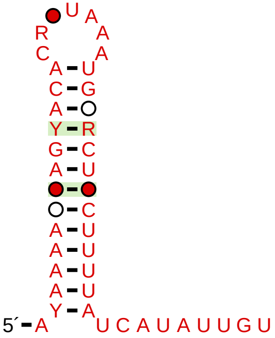

HMP2_1742_1

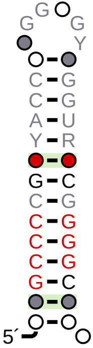

HMP2_1743_1

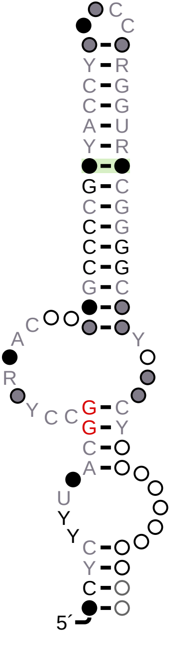

HMP2_175_1

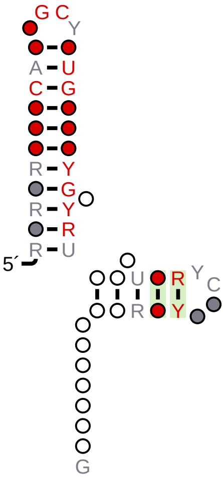

HMP2_1751_1

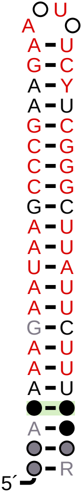

HMP2_176_1

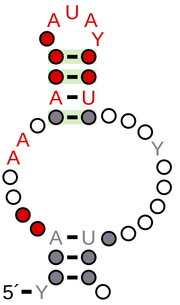

HMP2_1760_1

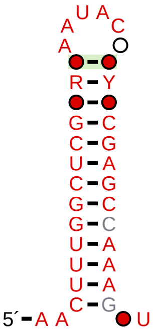

HMP2_1764_1

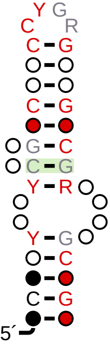

HMP2_1771_1

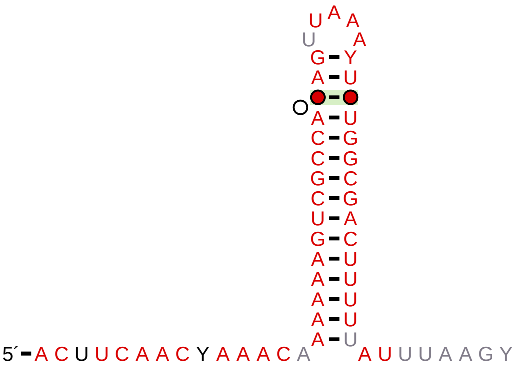

U A A
U A
G Y
A U
A U
C G
C G
G C
C G
U A
G C
A U
A U
A U
A U
A U
5´ A C U U C A A C Y A A A C A
A U U U A A G Y

HMP2_1784_1

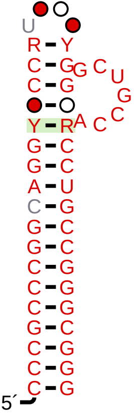

HMP2_1794_1

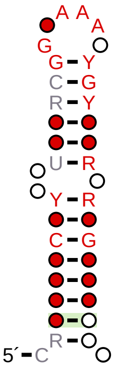

HMP2_1795_1

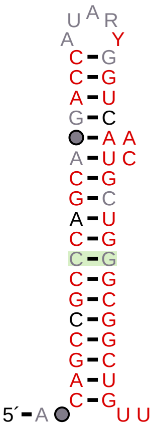

HMP2_1799_1

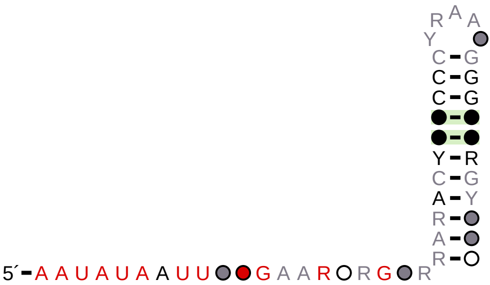

HMP2_18_1

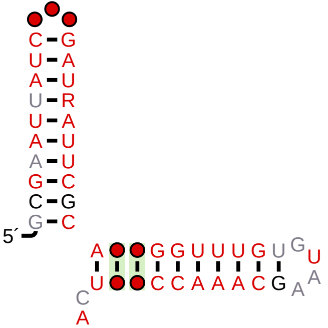

HMP2_1808_1

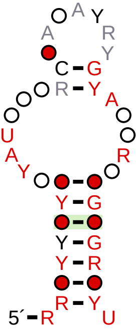

HMP2_1809_1

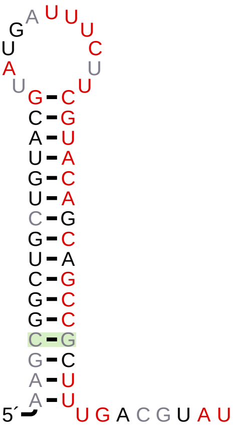

HMP2_1812_1

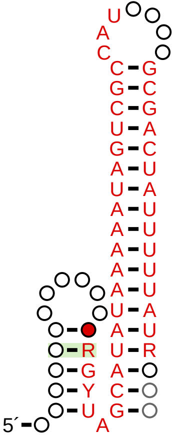

HMP2_1813_1

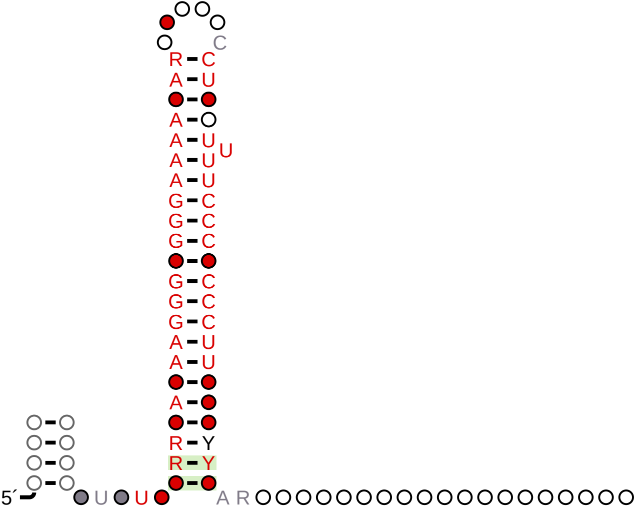

HMP2_1816_1

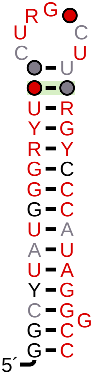

HMP2_1819_1

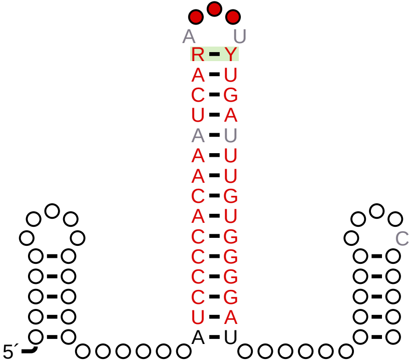

HMP2_1823_1

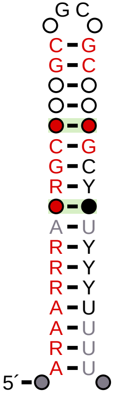

HMP2_1825_1

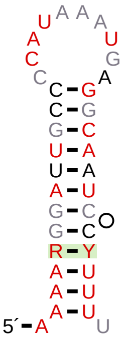

HMP2_1830_1

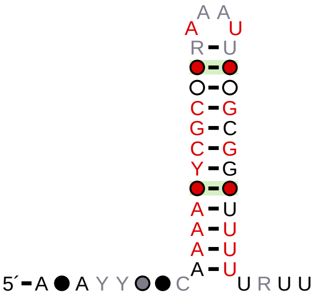

HMP2_1831_1

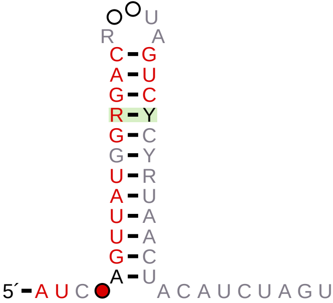

HMP2_1832_1

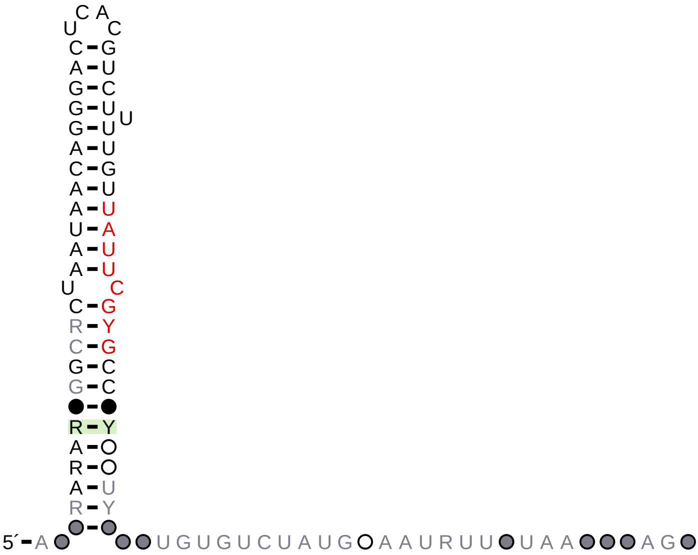

HMP2_1833_1

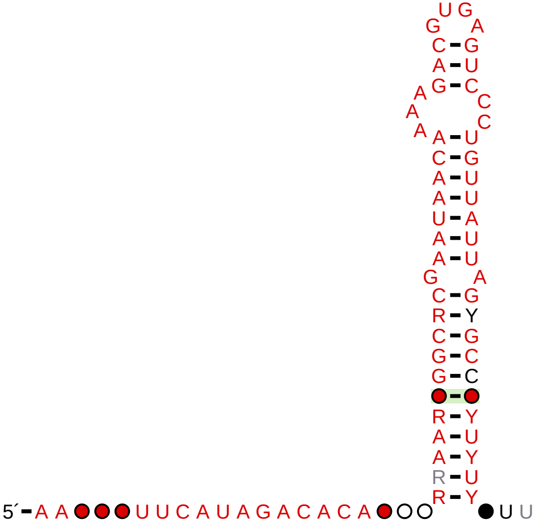

U G
G A
C G
A U
G C
A C
A C
A A U
C G
A U
A U
U A
A U
A U
G A
C G
R Y
C G
G C
G C
R Y
A U
A Y
R U
R Y
5´ A A UUCAUAGACACA UU

HMP2_1841_1

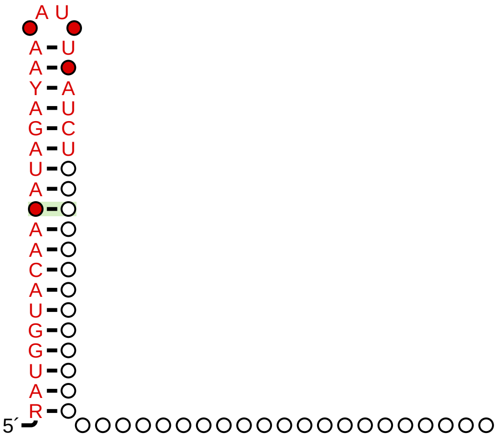

HMP2_1843_1

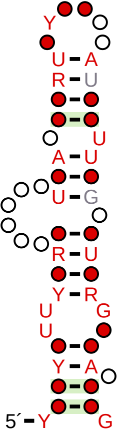

HMP2_1849_1

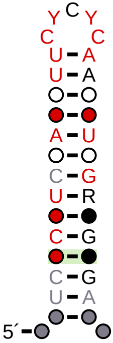

HMP2_1850_1

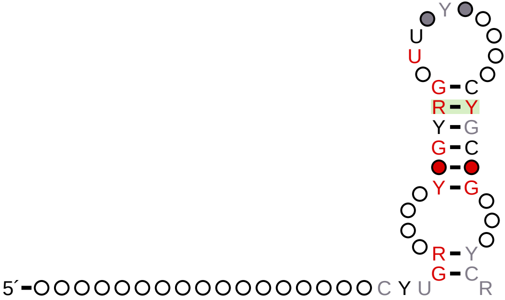

HMP2_1859_1

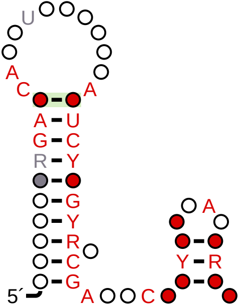

HMP2_1870_1

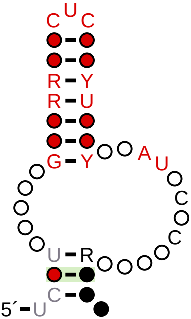

HMP2_1871_1

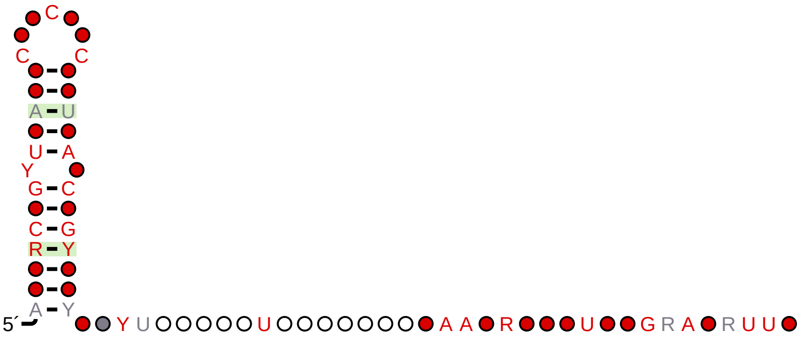

HMP2_1873_1

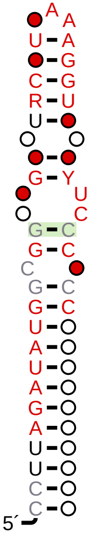

HMP2_1881_1

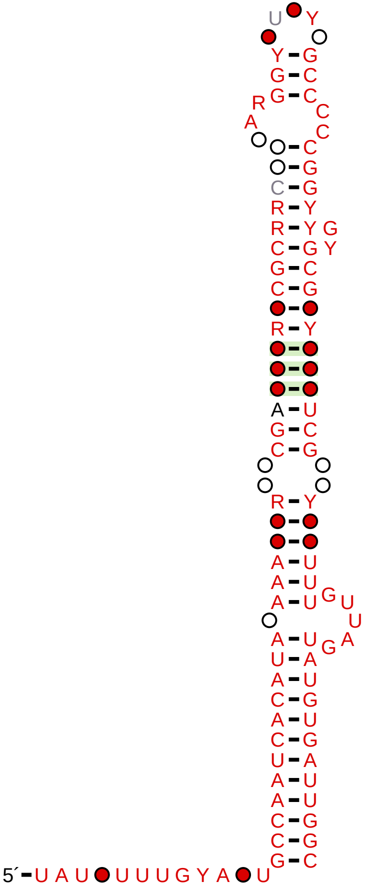

HMP2_1885_1

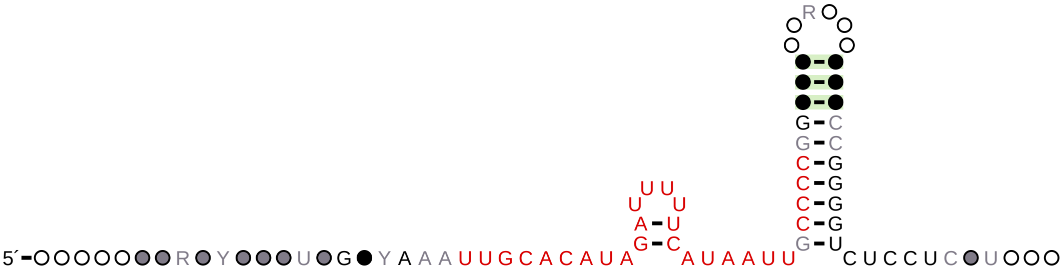

HMP2_1889_1

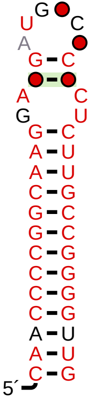

HMP2_1894_1

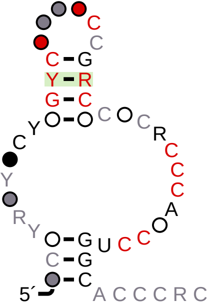

HMP2_1897_1

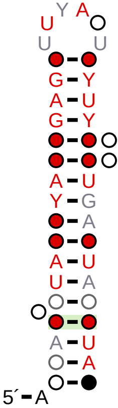

HMP2_19_1

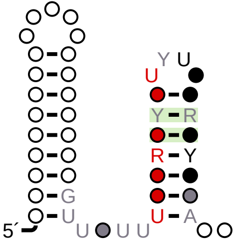

HMP2_1901_1

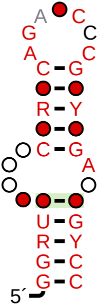

HMP2_1905_1

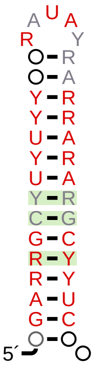

HMP2_1907_1

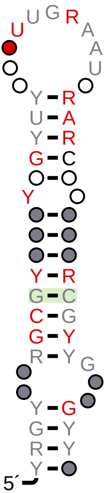

HMP2_1909_1

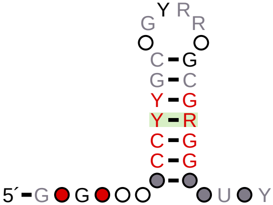

HMP2_1910_1

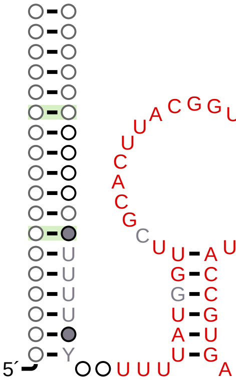

HMP2_1917_1

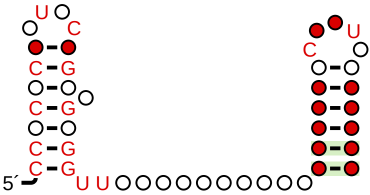

HMP2_1929_1

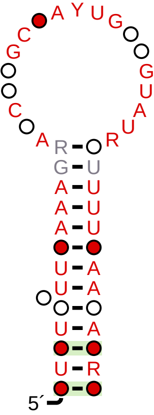

HMP2_1933_1

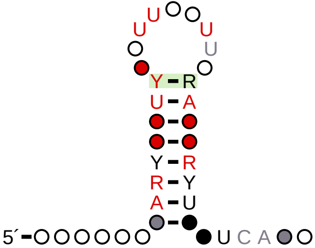

HMP2_1934_1

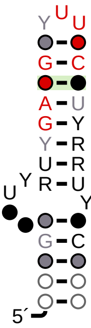

HMP2_1935_1

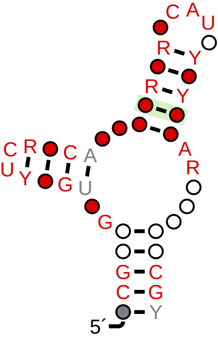

HMP2_1936_1

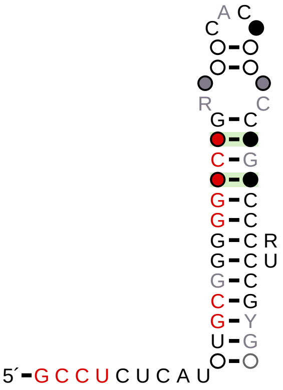

HMP2_1939_1

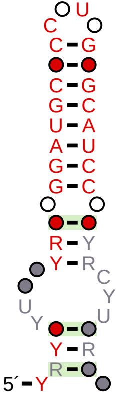

HMP2_194_1

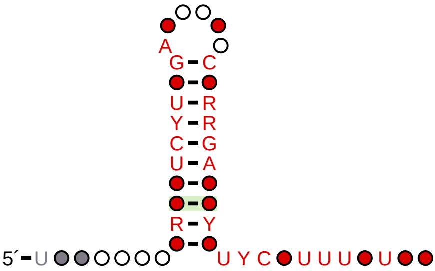

HMP2_1942_1

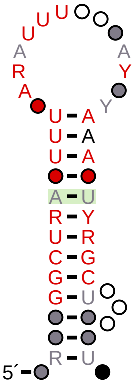

HMP2_1943_1

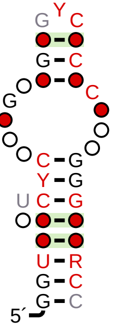

HMP2_1945_1

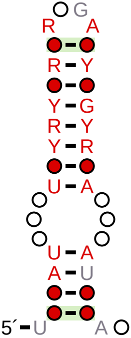

HMP2_1948_1

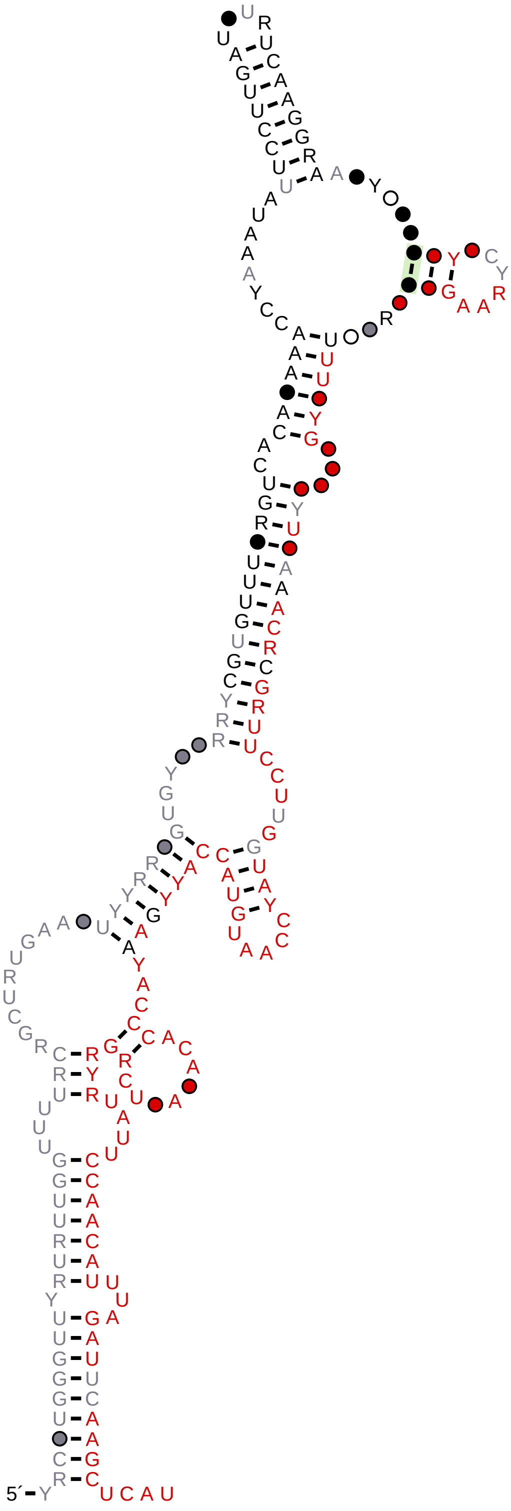

HMP2_1949_1

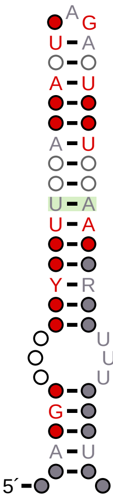

HMP2_1951_1

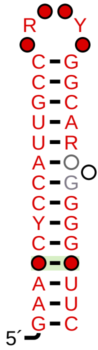

HMP2_1952_1

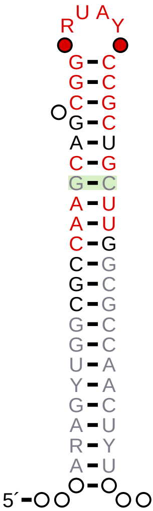

HMP2_1954_1

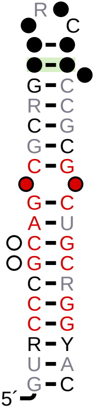

HMP2_1955_1

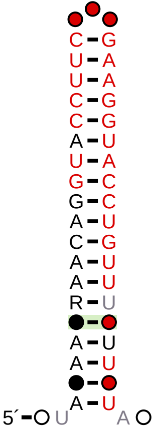

HMP2_1956_1

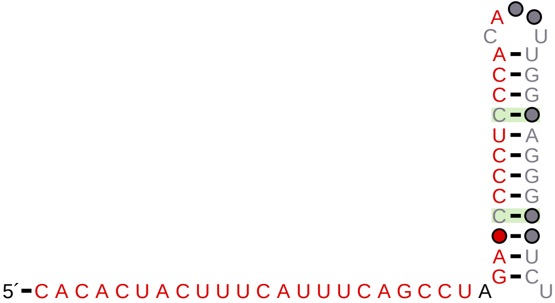

HMP2_1957_1

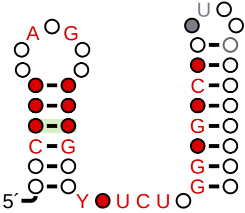

HMP2_1962_1

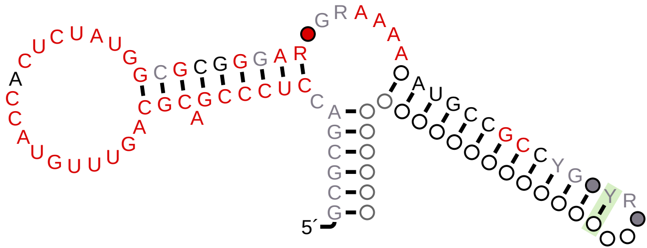

HMP2_1970_1

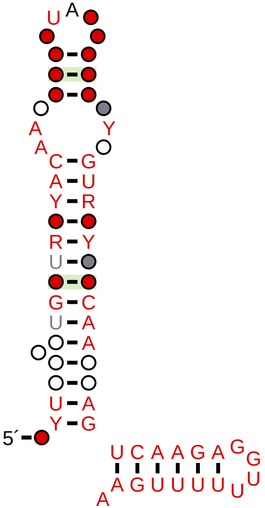

HMP2_1977_1

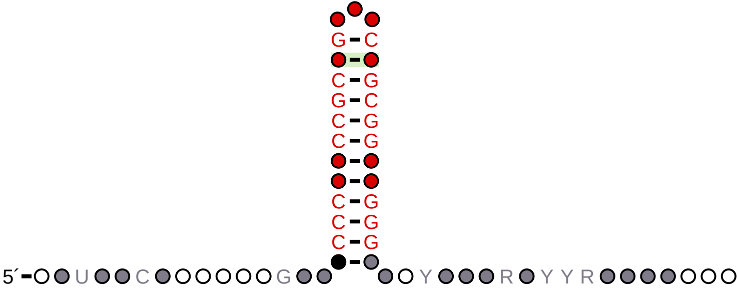

HMP2_1982_1

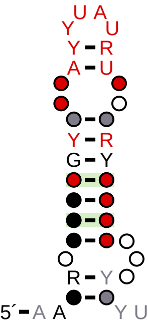

HMP2_1986_1

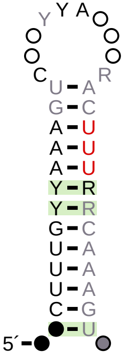

HMP2_1987_1

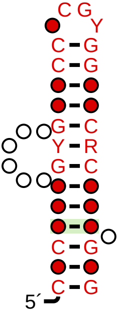

HMP2_1988_1

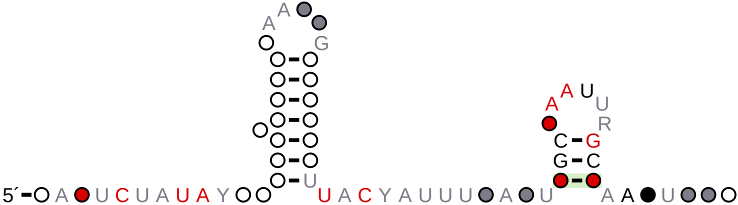

HMP2_1991_1

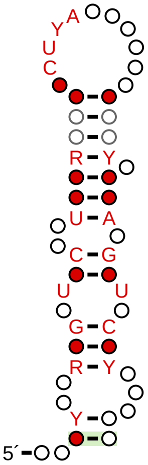

HMP2_1992_1

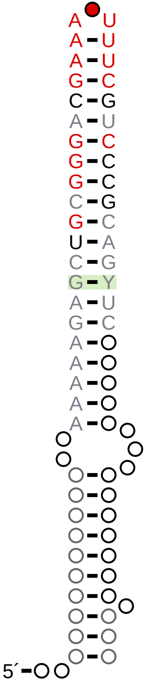

HMP2_1994_1

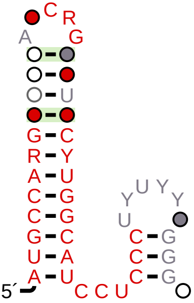

HMP2_1995_1

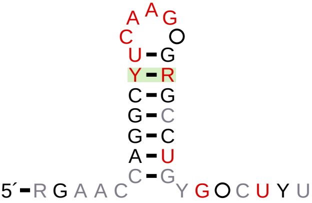

HMP2_2002_1

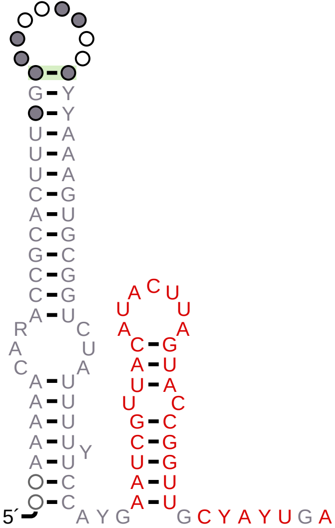
5´
A C U
U U
A A
C G
A U
U A
U C
G C
C G
U G
A U
A U
G Y
Y
U A
U A
U A
C G
A U
C G
G C
C G
C G
A U
R C
A U
C A
A U
A U
A U
A U Y
A U
C
C
A Y G
G C Y A Y U G A

HMP2_2008_1

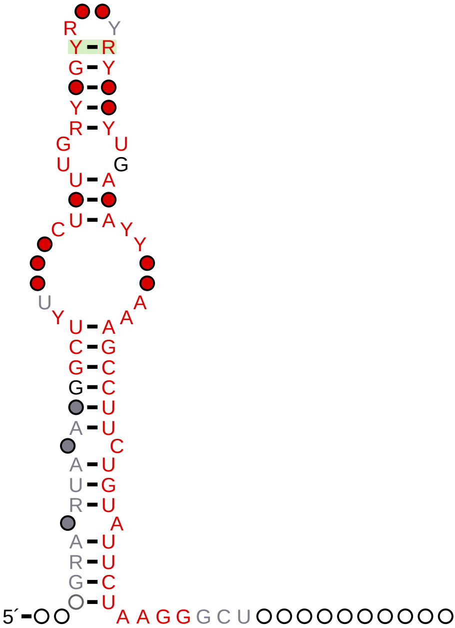

HMP2_2015_1

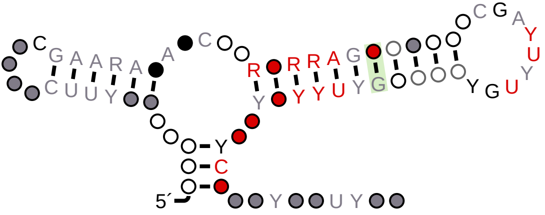

HMP2_2016_1

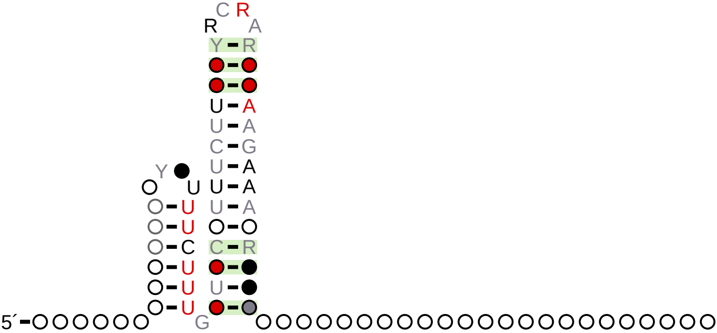

HMP2_2019_1

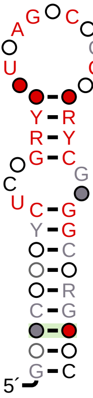

HMP2_2020_1

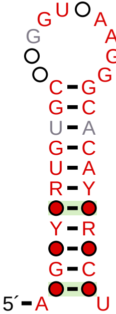

HMP2_2024_1

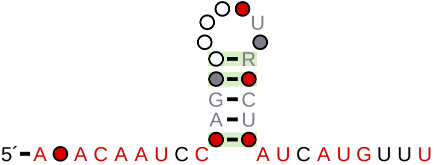

HMP2_2026_1

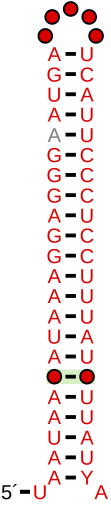

HMP2_2027_1

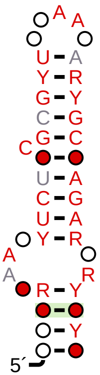

HMP2_2028_1

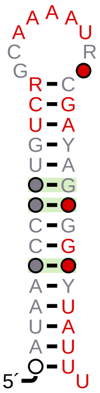

HMP2_2029_1

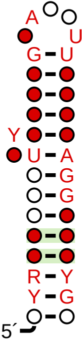

HMP2_2037_1

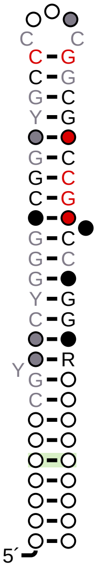

HMP2_2041_1

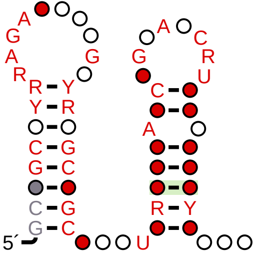

HMP2_2042_1

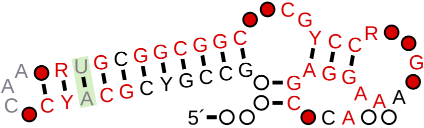

HMP2_2043_1

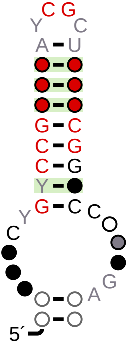

HMP2_205_1

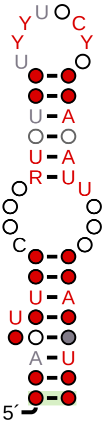

HMP2_2055_1

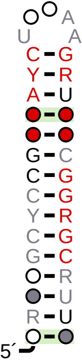

HMP2_2058_1

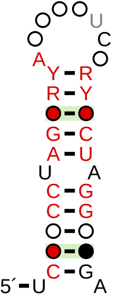

HMP2_2059_1

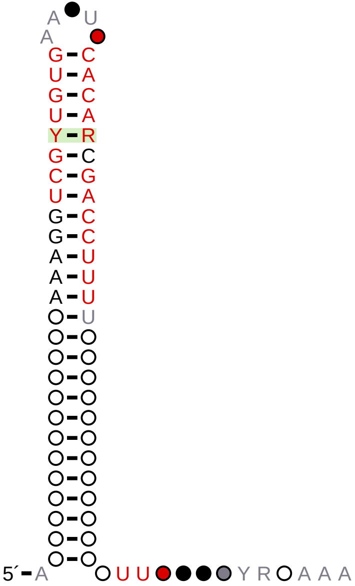

HMP2_2060_1

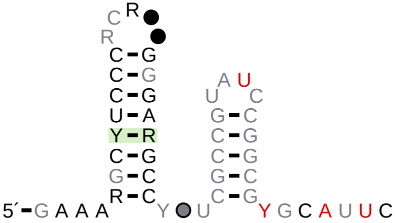

HMP2_207_1

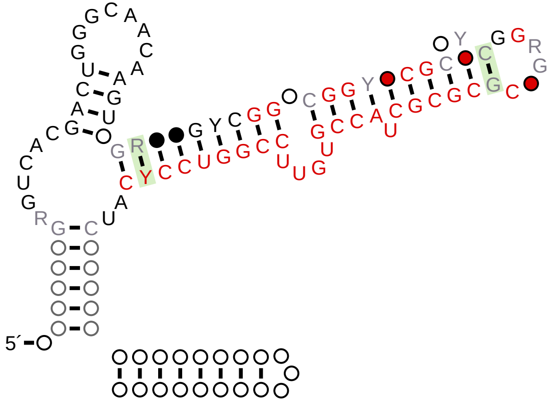

HMP2_2076_1

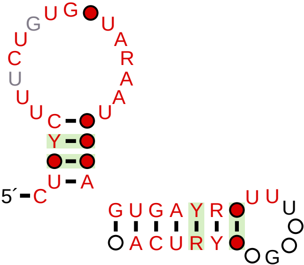

HMP2_2077_1

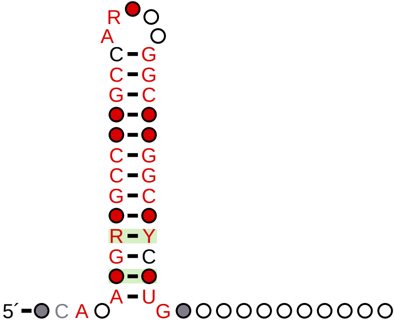

HMP2_2078_1

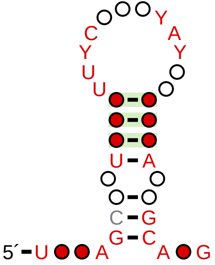

HMP2_2080_1

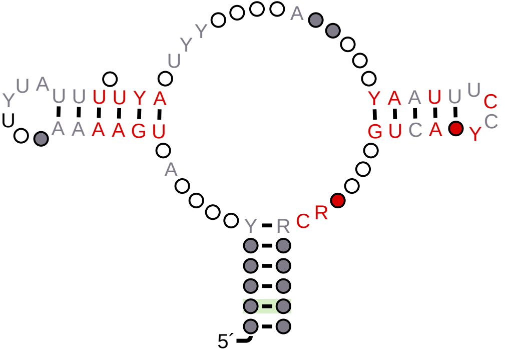

HMP2_2089_1

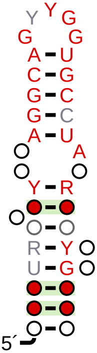

HMP2_2094_1

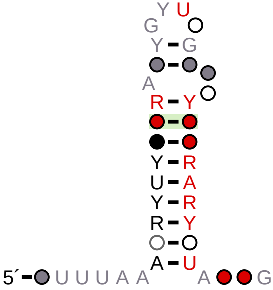

HMP2_2100_1

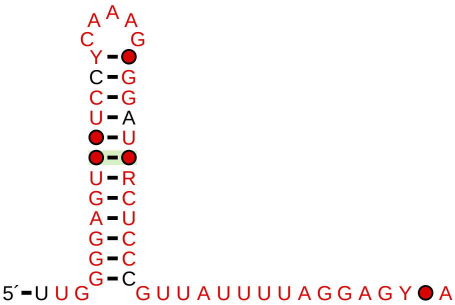

HMP2_2103_1

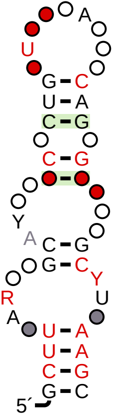

HMP2_2104_1

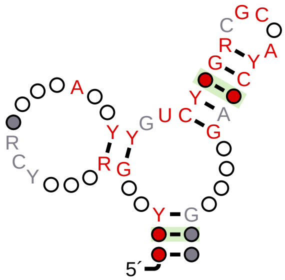

HMP2_2105_1

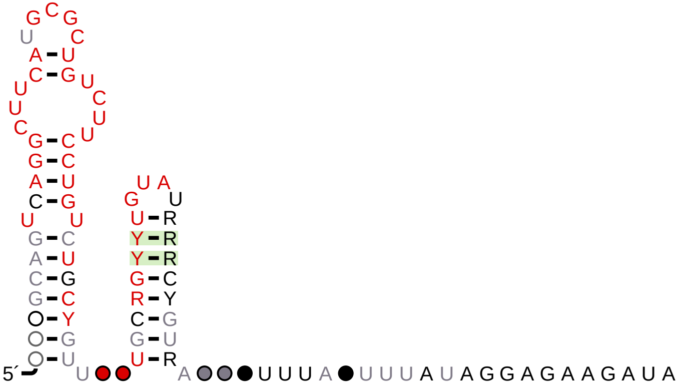

HMP2_2108_1

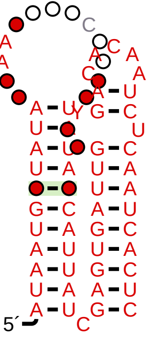

HMP2_2112_1

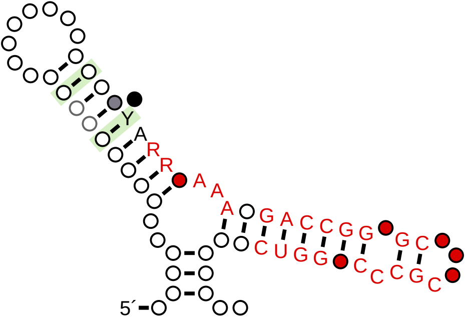

HMP2_2119_1

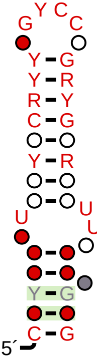

HMP2_2131_1

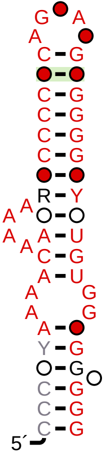

HMP2_2136_1

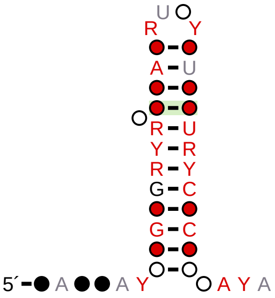

HMP2_2163_1

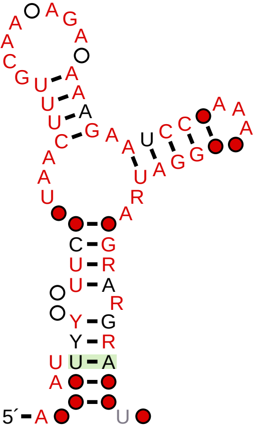

HMP2_2169_1

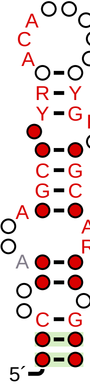

HMP2_2176_1

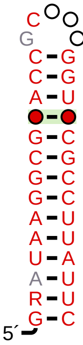

HMP2_2179_1

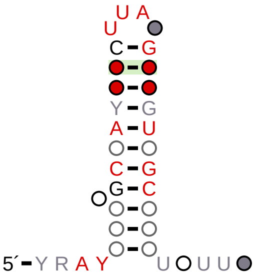

HMP2_2180_1

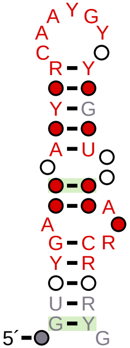

HMP2_2187_1

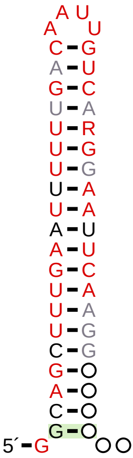

HMP2_2190_1

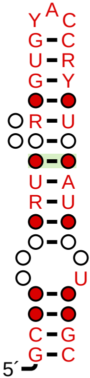

HMP2_2202_1

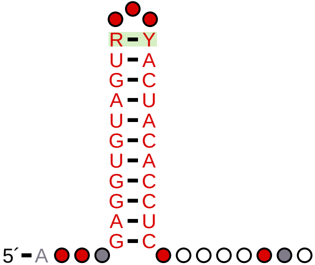

HMP2_2203_1

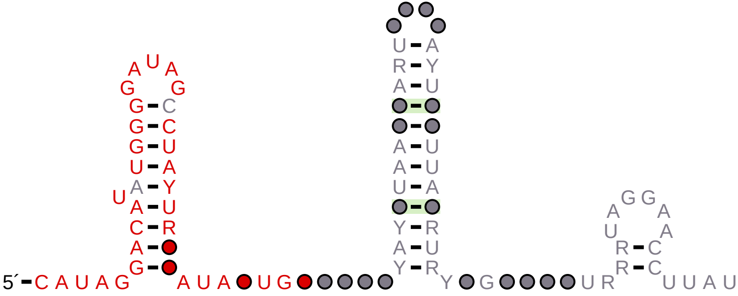

HMP2_2204_1

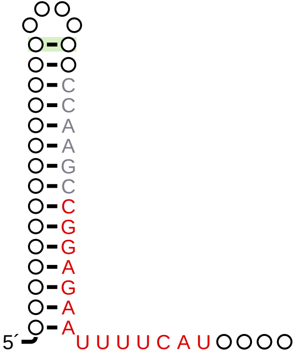

HMP2_2210_1

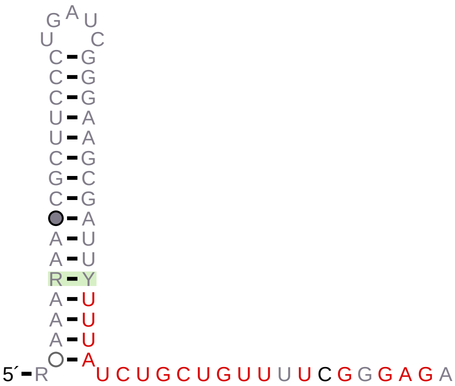

G A U
U C
C G
C G
C G
U A
U A
C G
G C
C G
A
A U
A U
R Y
A U
A U
A U
A
5´ R
UCUGCUGUUUUCGGGAGA

HMP2_2217_1

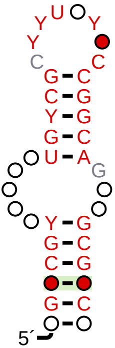

HMP2_2233_1

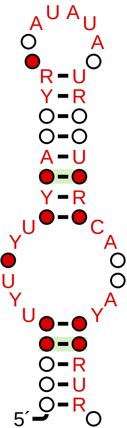

HMP2_2235_1

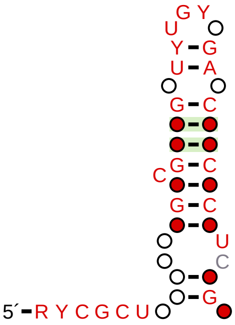

HMP2_2236_1

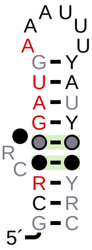

HMP2_2237_1

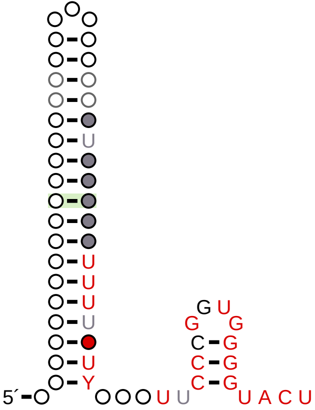

HMP2_2239_1

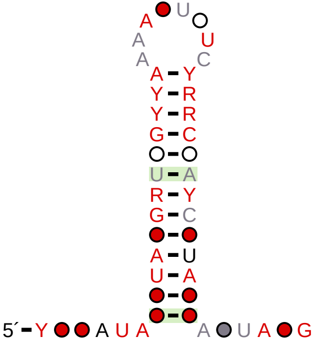

HMP2_224_1

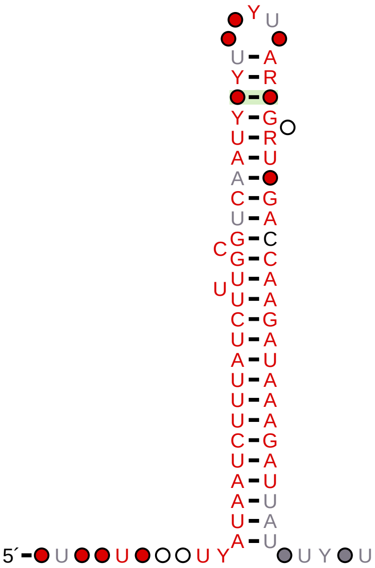

Y
U
U A
Y R
Y G
U R
A U
A
C G
U A
C
G C
G C
U
U A
U A
C G
U A
A U
U A
U A
U A
C G
U A
A U
A U
U A
A U
5´
U
U
U
U Y
U Y
U

HMP2_2240_1

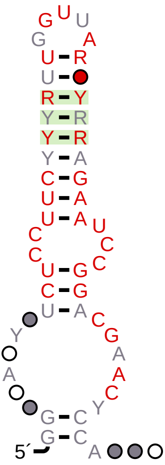

HMP2_2249_1

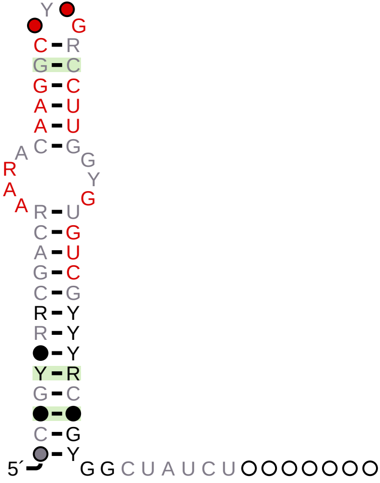

HMP2_2265_1

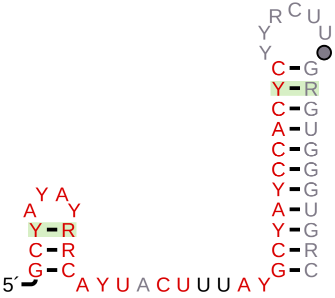

Y R C U U
Y
C G
Y R
C G
A U
C G
C G
Y G
A U
Y G
C R
G C
Y A A Y
A Y
Y R
C R
G C
5´
A Y U A C U U U A Y

HMP2_2277_1

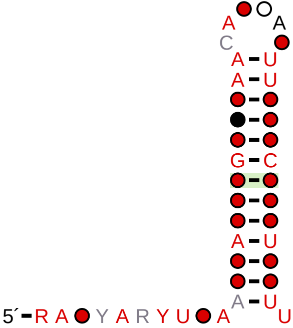

HMP2_2278_1

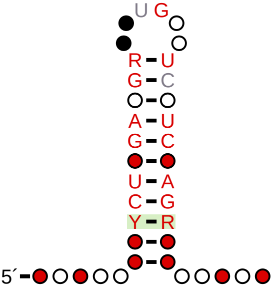

HMP2_228_1

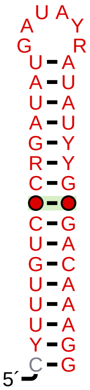

HMP2_2282_1

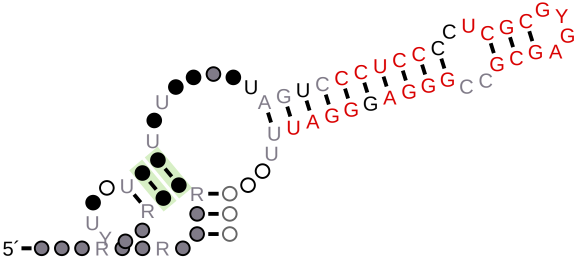

HMP2_2286_1

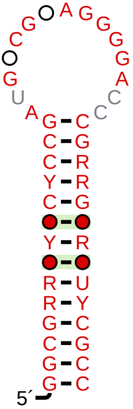

HMP2_229_1

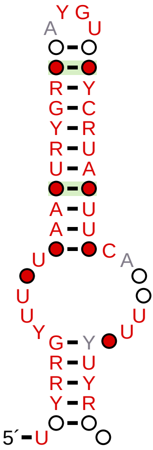

HMP2_2290_1

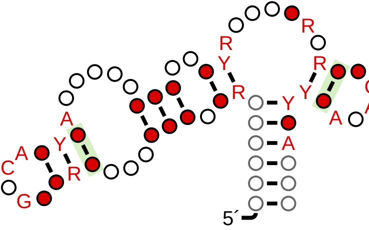

HMP2_2293_1

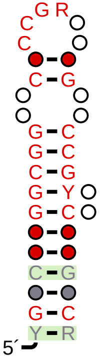

HMP2_2297_1

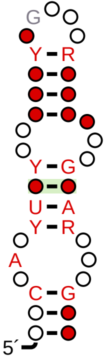

HMP2_2315_1

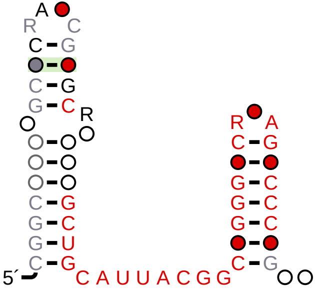

HMP2_2317_1

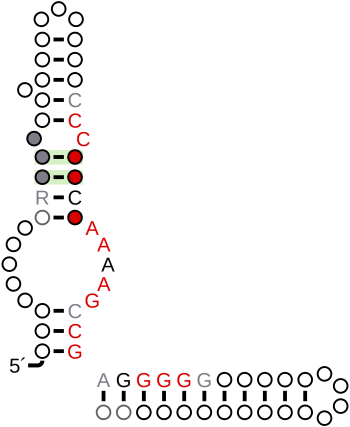

HMP2_2318_1

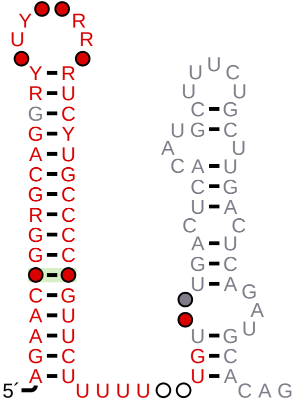

Y R
U R
Y R
R U
G C
G Y
A U
C G
G C
R C
G C
G C
C G
A U
A U
G C
A U
5´
U U U U
U U C
U U
C G
U G C
A U
C A U
C G
U A
C C
A U
G C
U A G
A
U
U G
G C
U A
C A G

HMP2_232_1

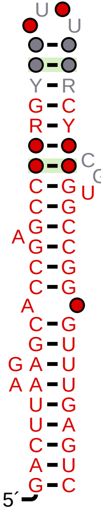

HMP2_2321_1

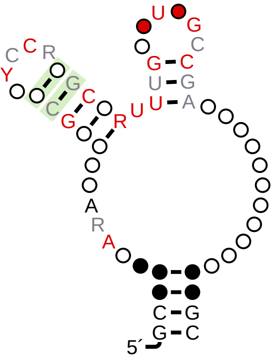

HMP2_2332_1

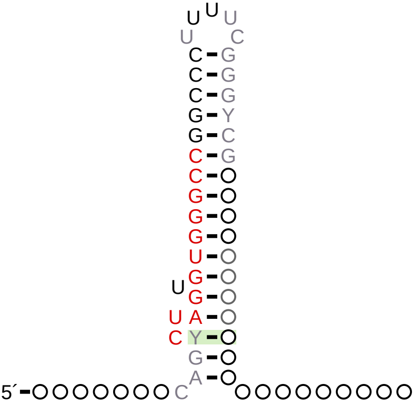

HMP2_2336_1

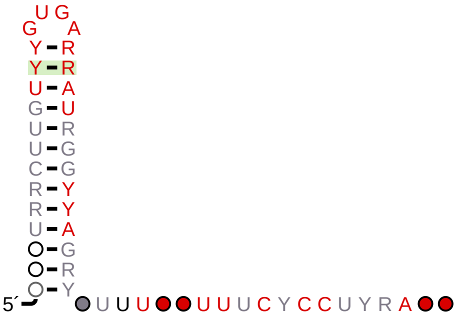

HMP2_2337_1

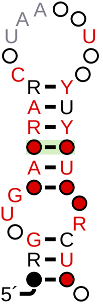

HMP2_2339_1

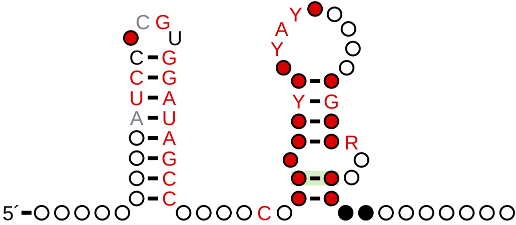

HMP2_2342_1

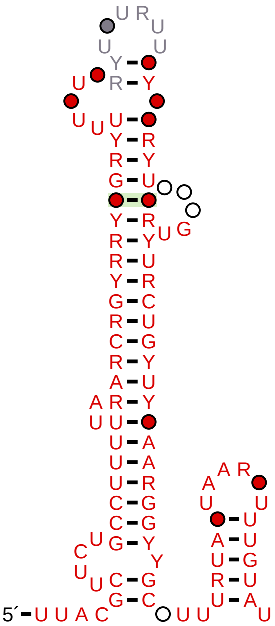

HMP2_2343_1

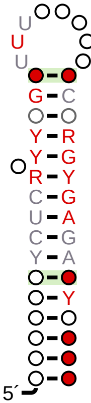

HMP2_2345_1

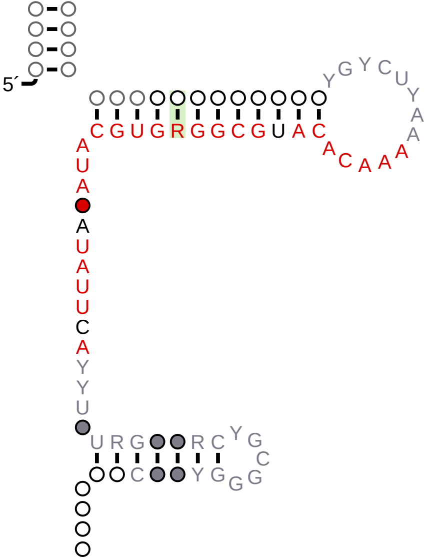
5´
C G U G R G G C G U A C
Y G Y C U Y A A
A C A A A
A U A
A U A U U C A Y Y U
U R G R C Y G C
C Y G G G

HMP2_2347_1

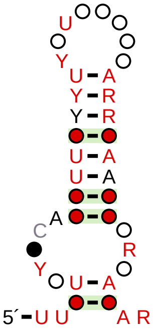

HMP2_2350_1

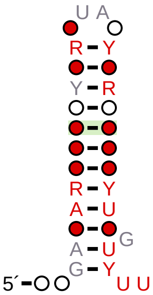

HMP2_2352_1

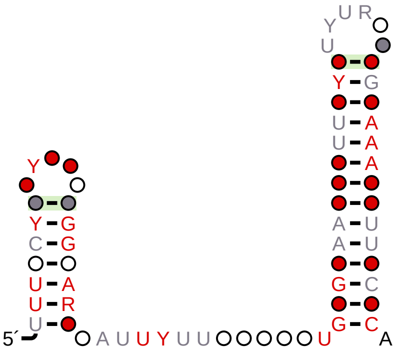

HMP2_2353_1

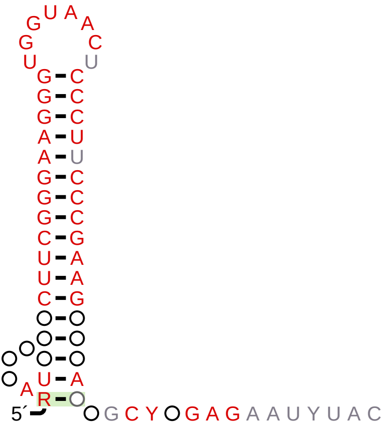
G U A A C
G U
U
G C
G C
G C
A U
A U
G C
G C
G C
C G
U A
U A
C G
A U A
R
5´ G C Y G A G A A U Y U A C

HMP2_2354_1

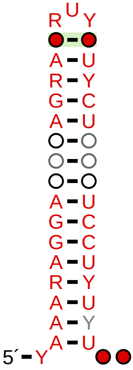

HMP2_2359_1

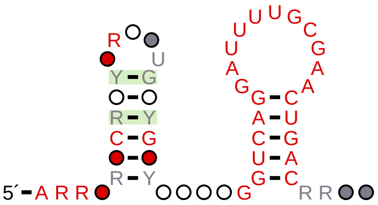

HMP2_2361_1

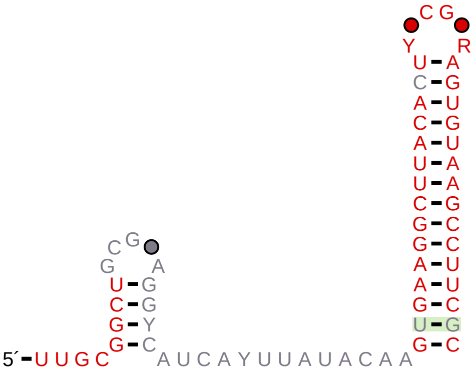

HMP2_2363_1

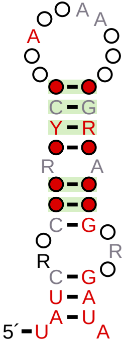

HMP2_2364_1

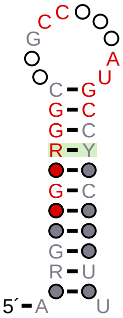

HMP2_2367_1

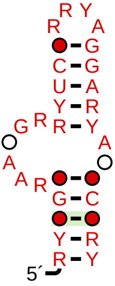

HMP2_2370_1

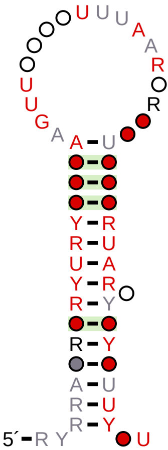

HMP2_2373_1

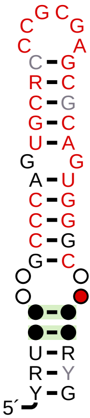

HMP2_238_1

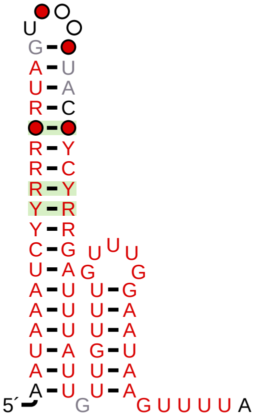

HMP2_2381_1

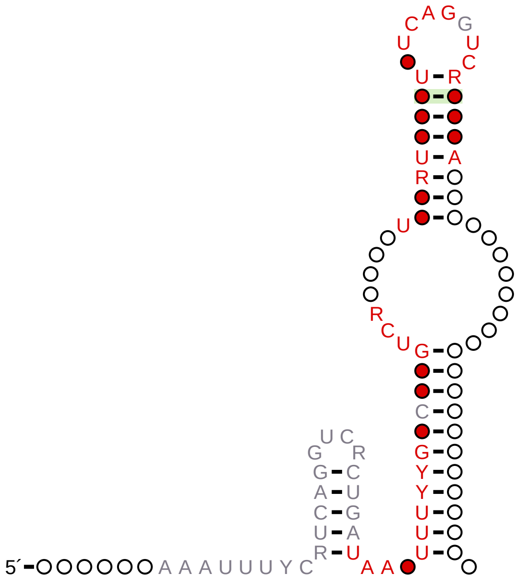

HMP2_2384_1

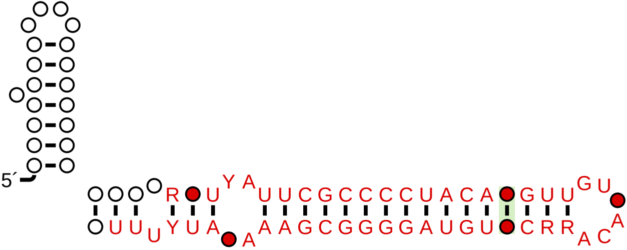

HMP2_2385_1

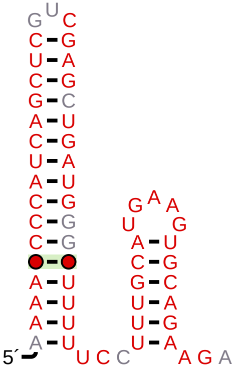

HMP2_239_1

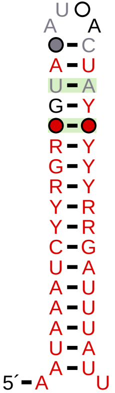

HMP2_2391_1

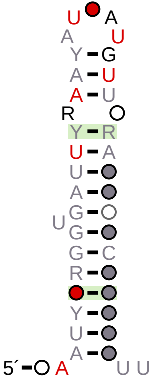

HMP2_2403_1

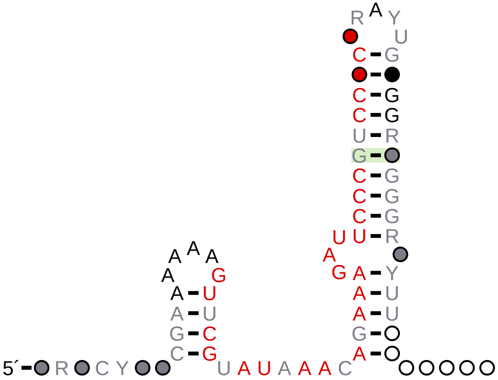

R A Y
U
C G
C G
C G
U R
G
C G
C G
C G
U U R
A A A A
A G G A Y
A U A U
A U A U
G C G
C G A
5´ R C Y U A U A A A C

HMP2_2404_1

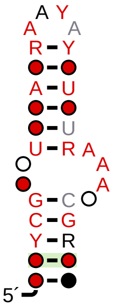

HMP2_2406_1

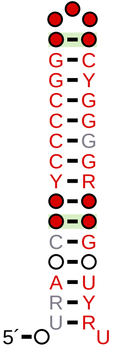

HMP2_2409_1

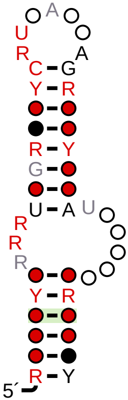

HMP2_2412_1

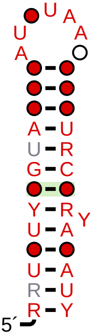

HMP2_2413_1

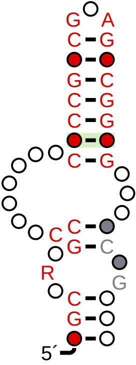

HMP2_2416_1

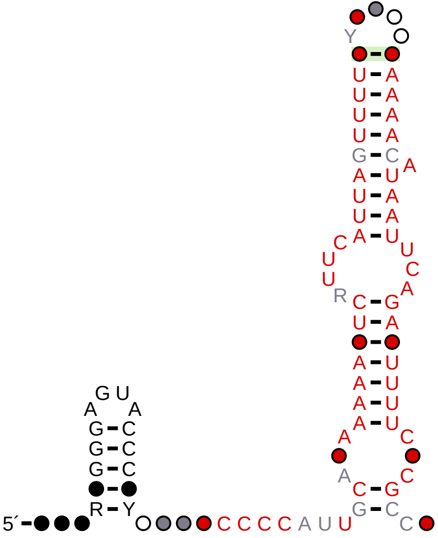

HMP2_2419_1

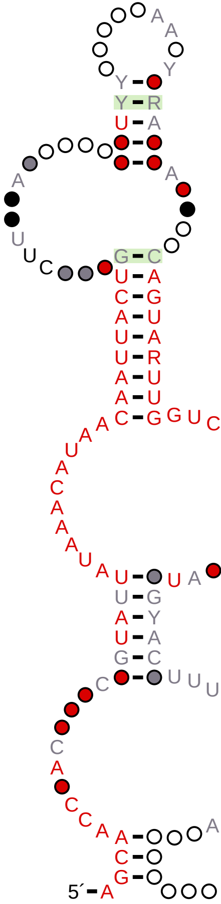

HMP2_242_1

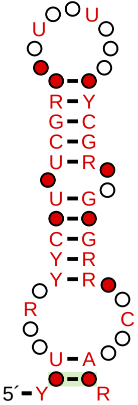

HMP2_2421_1

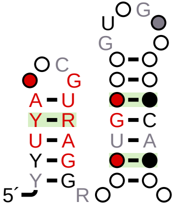

HMP2_2426_1

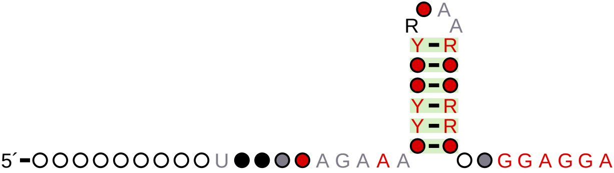

HMP2_2429_1

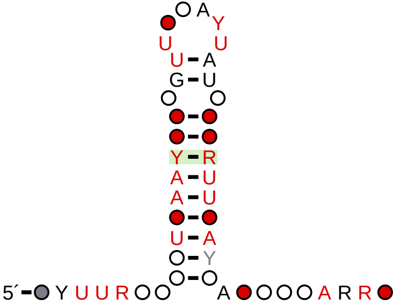

HMP2_2439_1

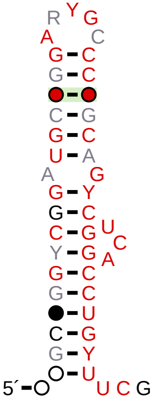

HMP2_2451_1

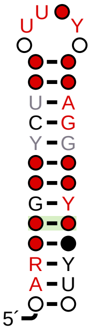

HMP2_2452_1

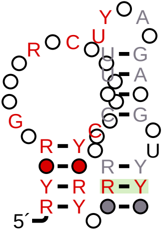

HMP2_2457_1

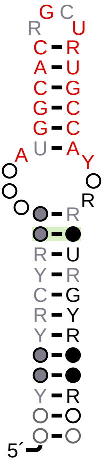

HMP2_2458_1

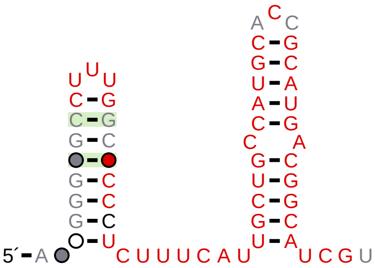
5´ A
C U U U C A U
U C G U
U U U
C G
C G
G C
G C
G C
G C
U
A C C
C G
G C
U A
A U
C G
C A
G C
U G
C G
G C
U A

HMP2_246_1

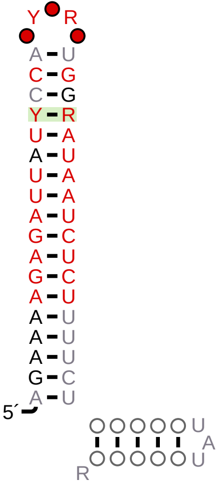

HMP2_2464_1

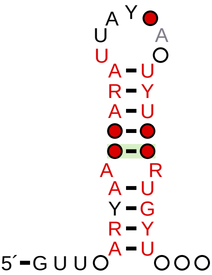

HMP2_2465_1

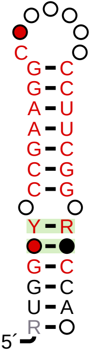

HMP2_2474_1

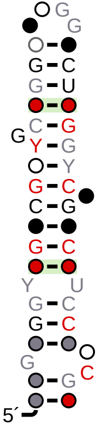

HMP2_2483_1

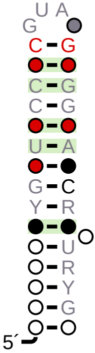

HMP2_2485_1

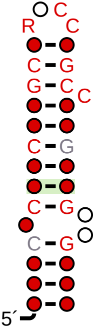

HMP2_2486_1

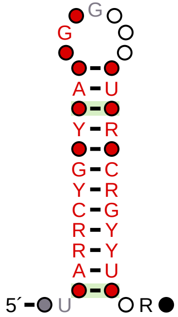

HMP2_2489_1

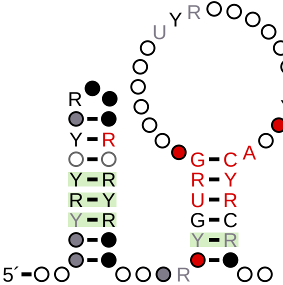

HMP2_2490_1

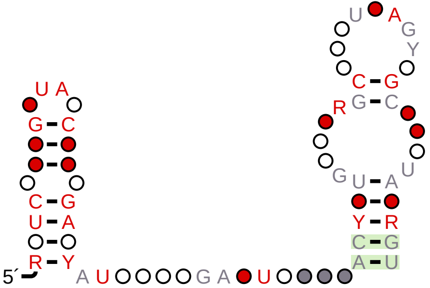

HMP2_2495_1

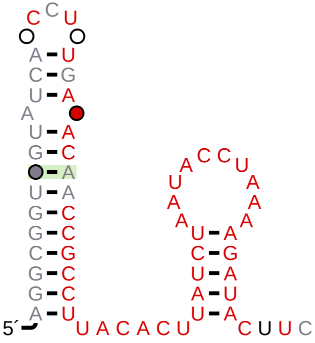

HMP2_2498_1

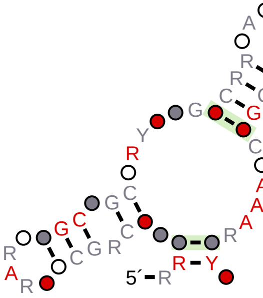

HMP2_25_1

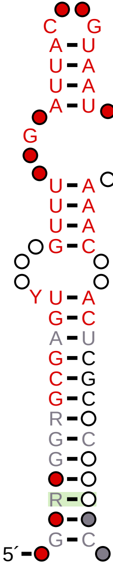

HMP2_2501_1

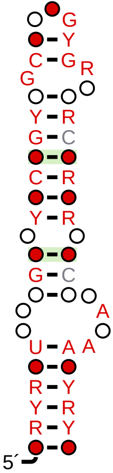

HMP2_2504_1

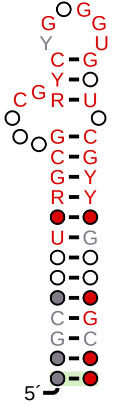

HMP2_2506_1

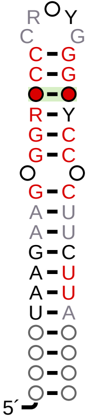

HMP2_2507_1

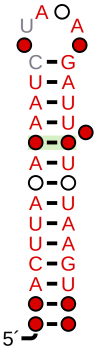

HMP2_252_1

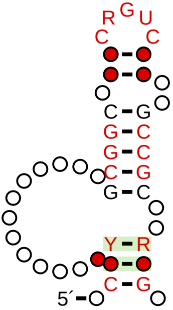

HMP2_2522_1

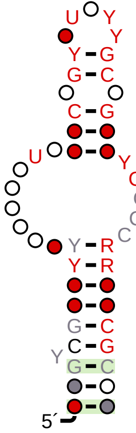

HMP2_2531_1

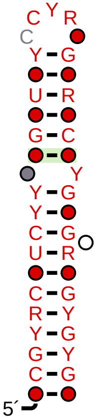

HMP2_2537_1

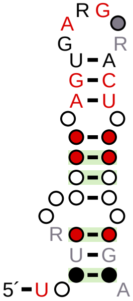

HMP2_2539_1

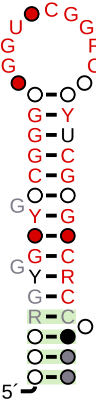

HMP2_254_1

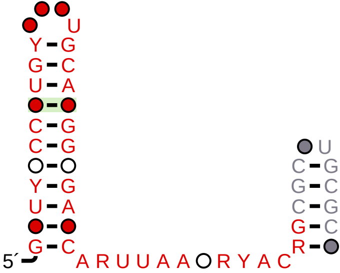

HMP2_2541_1

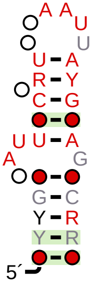

HMP2_2545_1

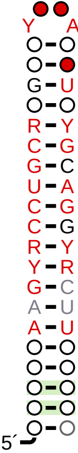

HMP2_255_1

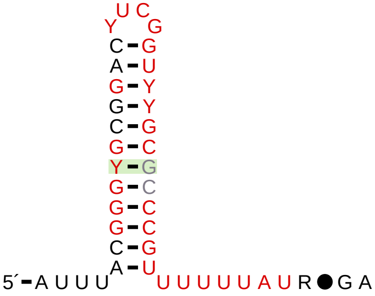

HMP2_2559_1

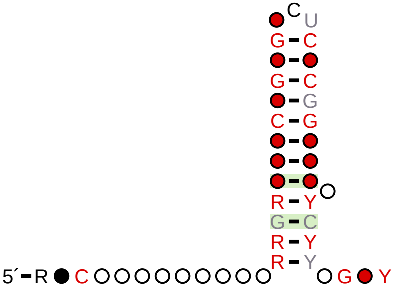

HMP2_256_1

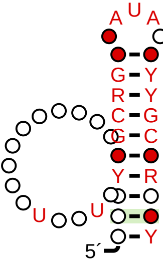

HMP2_2565_1

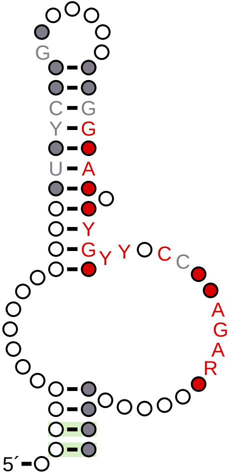

HMP2_2568_1

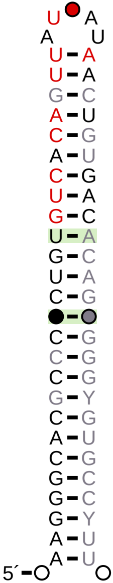

HMP2_2569_1

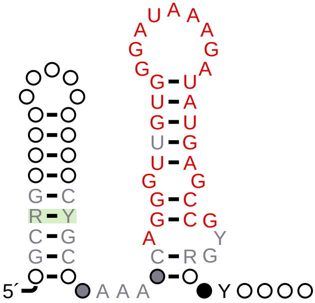

HMP2_2570_1

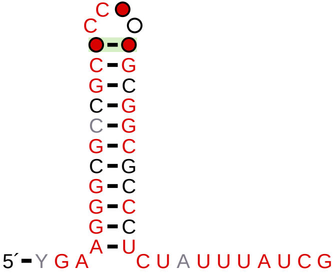

HMP2_2577_1

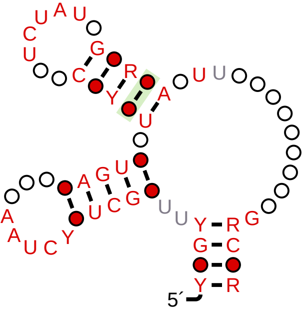

HMP2_2579_1

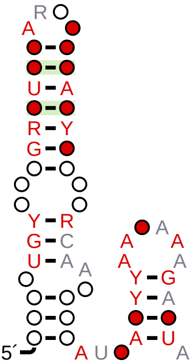

HMP2_2583_1

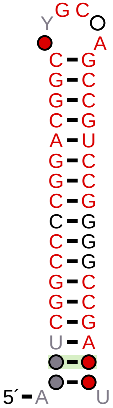

HMP2_2585_1

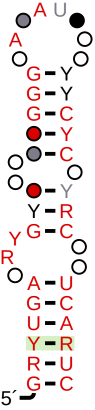

HMP2_2586_1

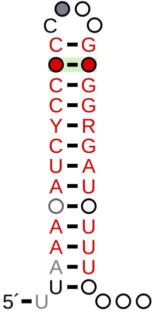

HMP2_2587_1

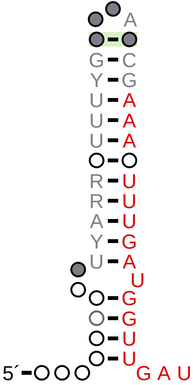

HMP2_2589_1

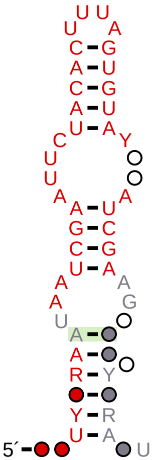

HMP2_2590_1

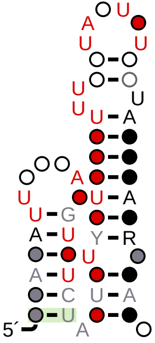

HMP2_2591_1

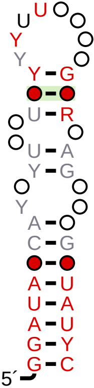

HMP2_2593_1

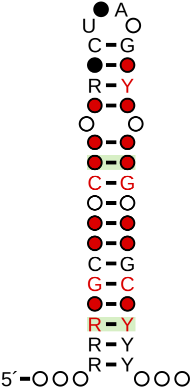

HMP2_2594_1

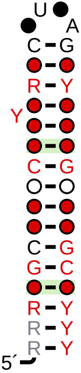

HMP2_2598_1

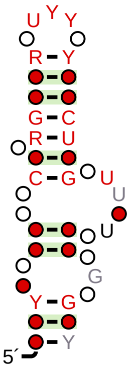

HMP2_2599_1

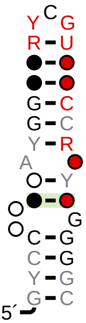

HMP2_26_1

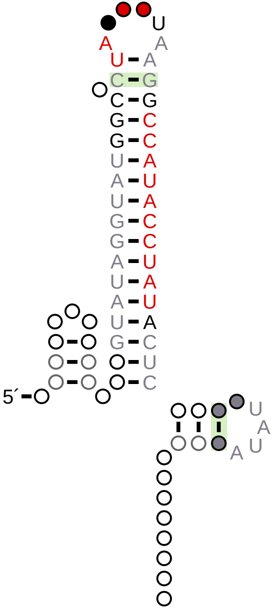

HMP2_2602_1

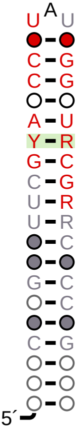

HMP2_2608_1

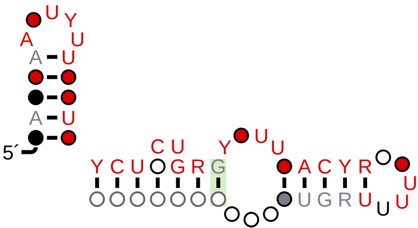

HMP2_2615_1

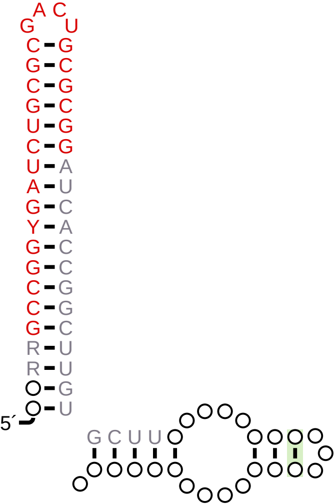

HMP2_2620_1

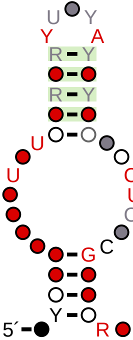

HMP2_263_1

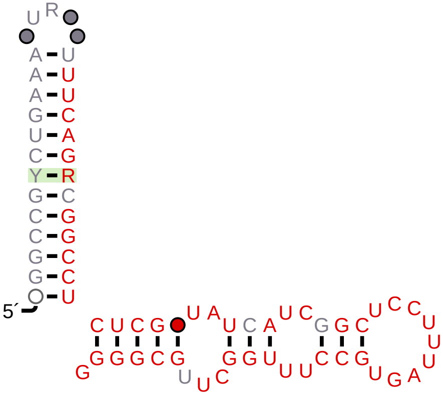

HMP2_2631_1

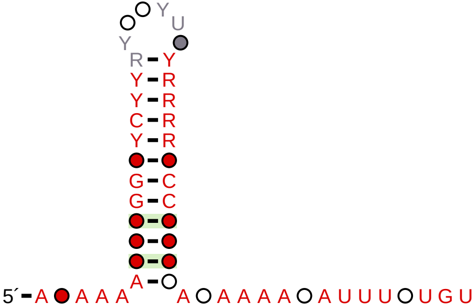

HMP2_2633_1

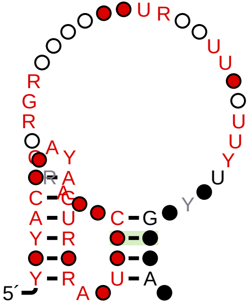

HMP2_2634_1

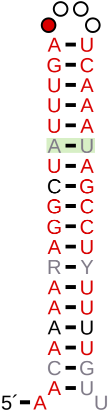

HMP2_264_1

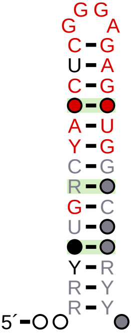

HMP2_2645_1

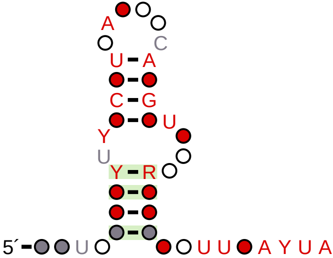

HMP2_2646_1

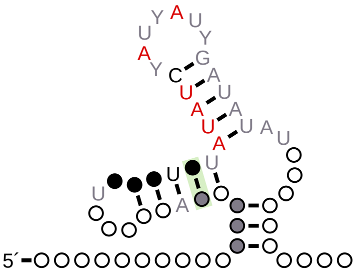

HMP2_2648_1

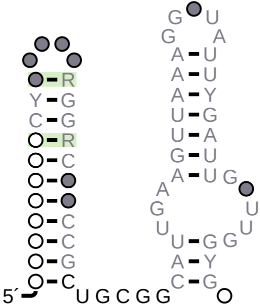

HMP2_2658_1

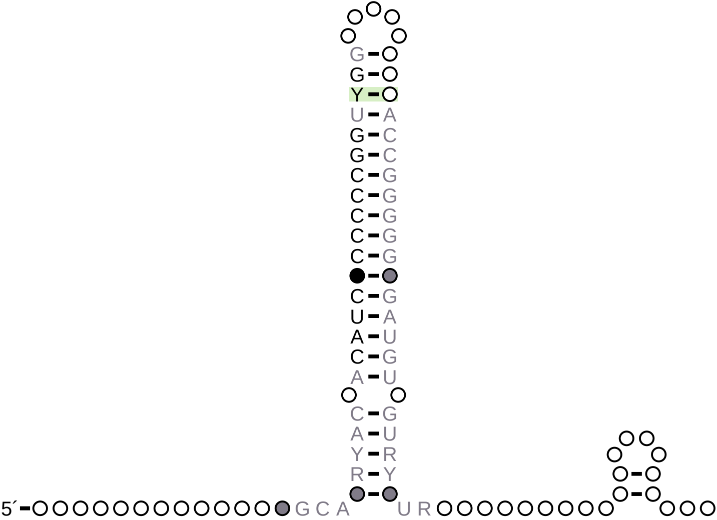

HMP2_2660_1

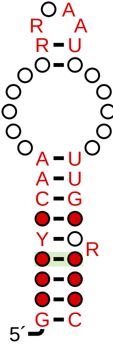

HMP2_2665_1

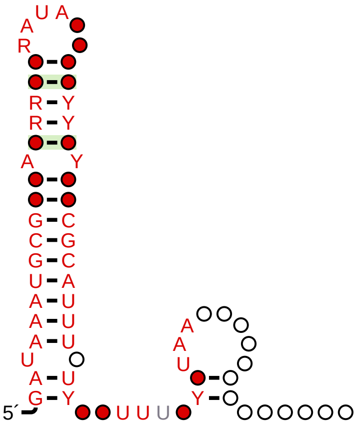

HMP2_2670_1

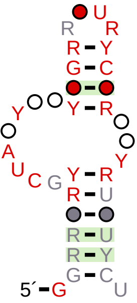

HMP2_2687_1

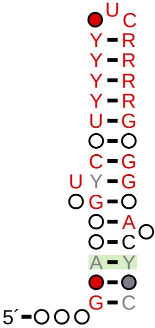

HMP2_269_1

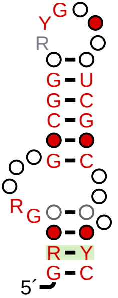

HMP2_270_1

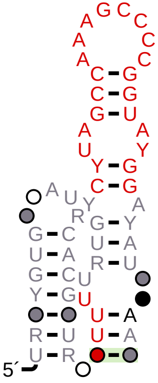

HMP2_2702_1

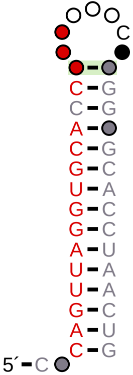

HMP2_2705_1

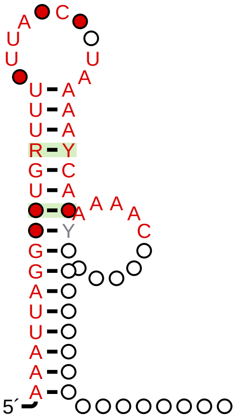

HMP2_2713_1

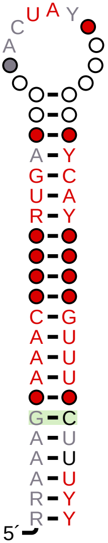

HMP2_2717_1

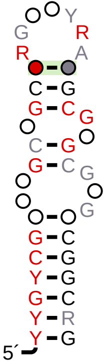

HMP2_2718_1

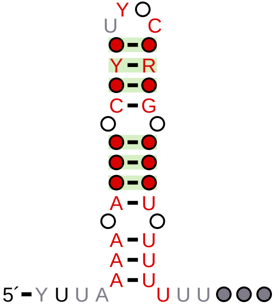

HMP2_2725_1

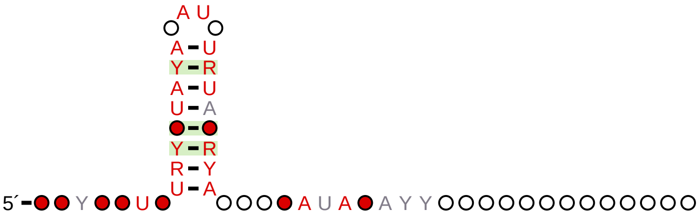

HMP2_2727_1

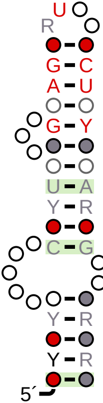

HMP2_2738_1

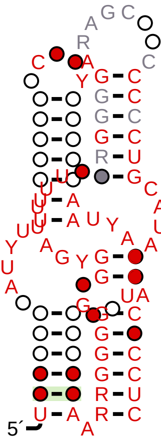

HMP2_2742_1

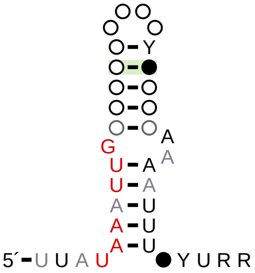

HMP2_2744_1

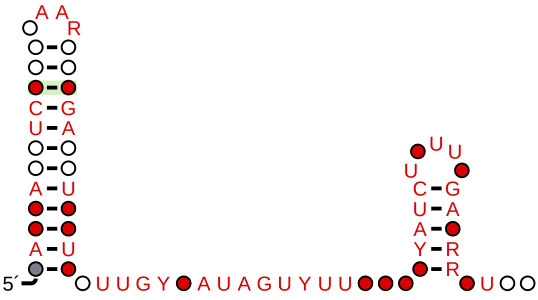

HMP2_2746_1

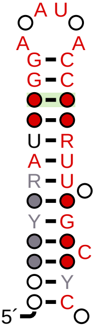

HMP2_2749_1

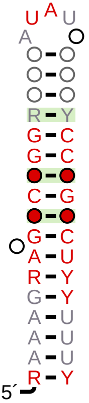

HMP2_275_1

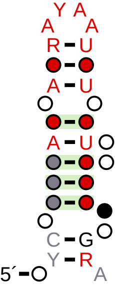

HMP2_2754_1

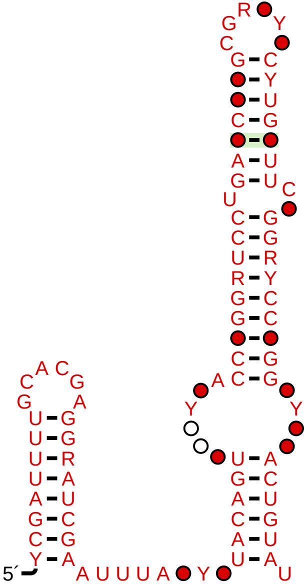

HMP2_2763_1

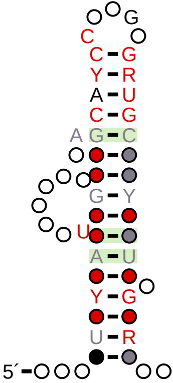

HMP2_2766_1

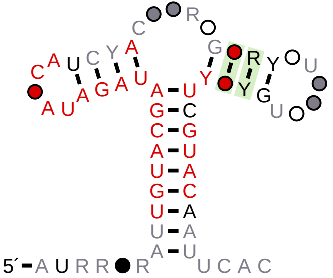

HMP2_2769_1

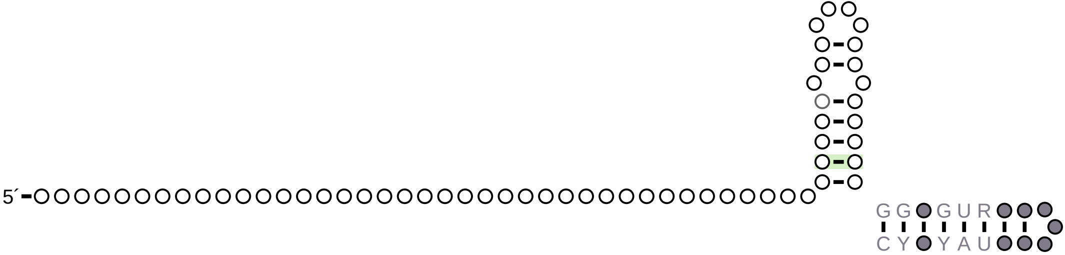

HMP2_2770_1

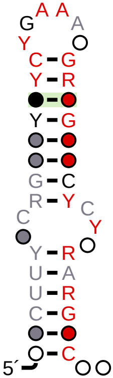

HMP2_2771_1

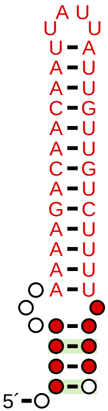

HMP2_2774_1

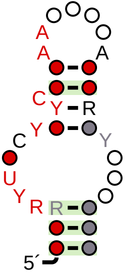

HMP2_2775_1

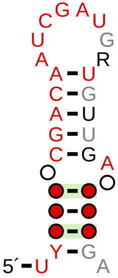

HMP2_2798_1

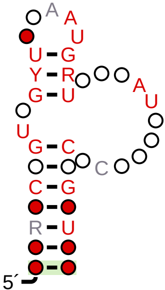

HMP2_2800_1

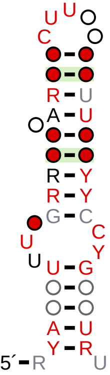

HMP2_2807_1

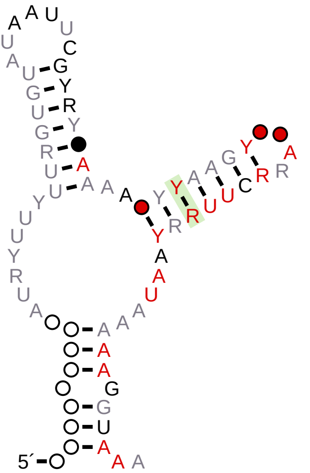

HMP2_2808_1

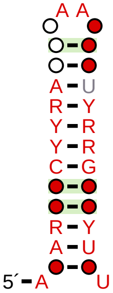

HMP2_2809_1

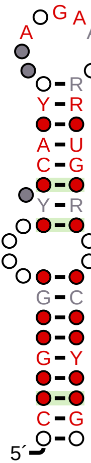

HMP2_281_1

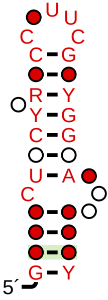

HMP2_2815_1

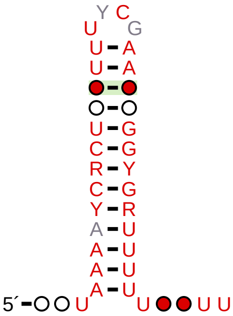

HMP2_2819_1

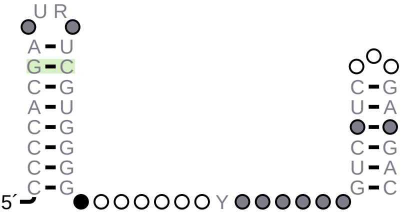

HMP2_2821_1

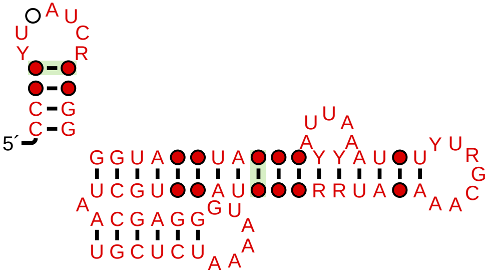

HMP2_2826_1

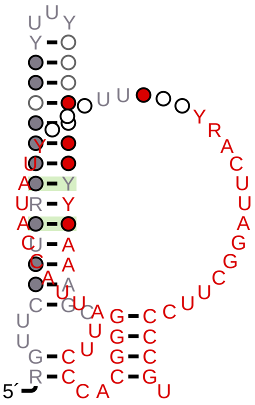

HMP2_2828_1

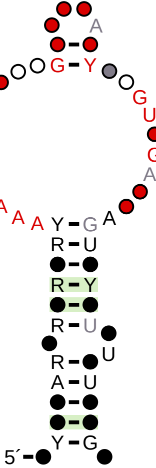

HMP2_2834_1

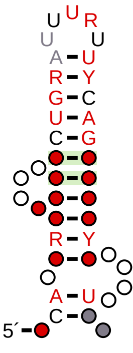

HMP2_2835_1

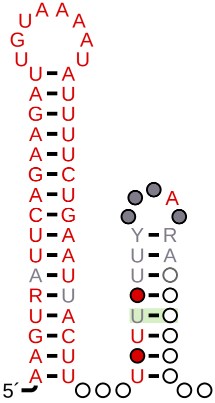

HMP2_2837_1

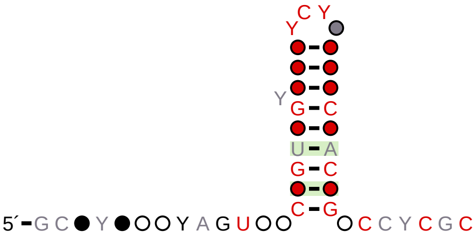

HMP2_2844_1

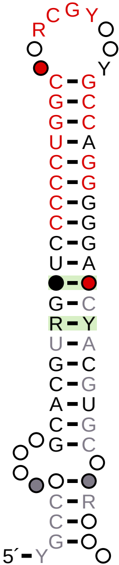

HMP2_2850_1

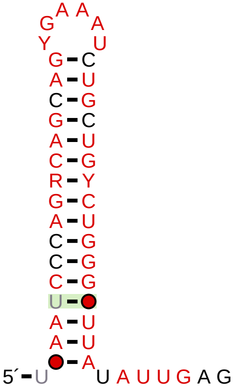

HMP2_2854_1

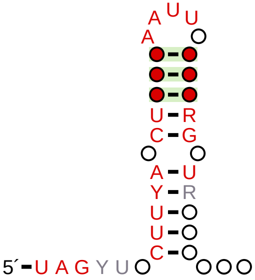

HMP2_2857_1

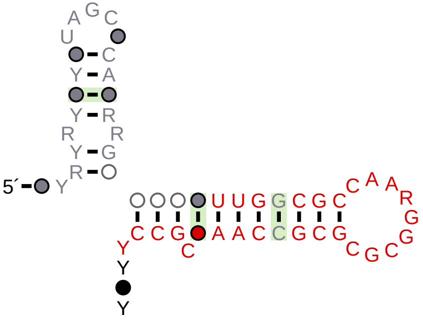

HMP2_286_1

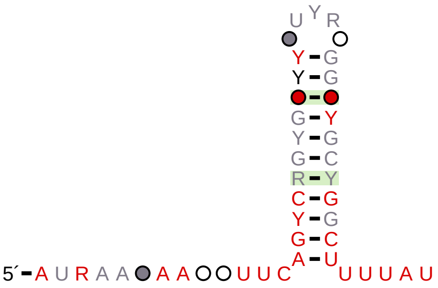

HMP2_2874_1

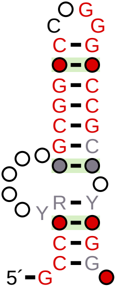

HMP2_2875_1

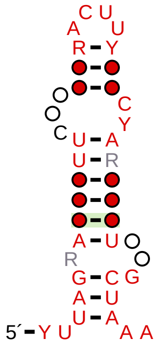

HMP2_288_1

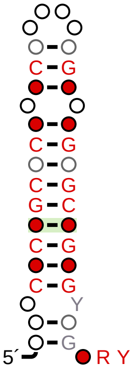

HMP2_2881_1

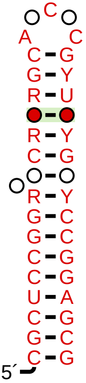

HMP2_2884_1

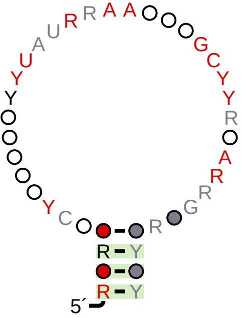

HMP2_2885_1

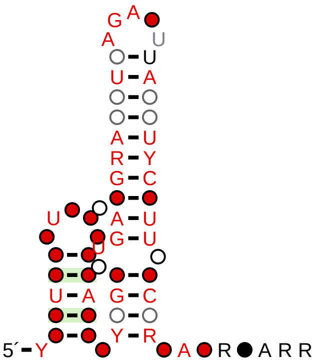

HMP2_289_1

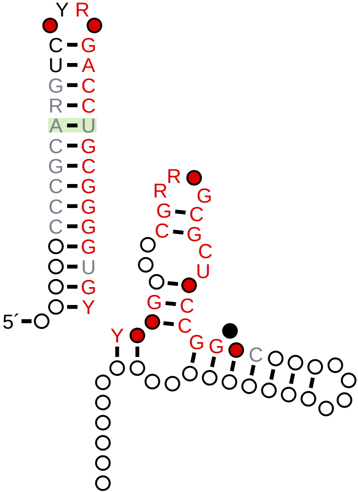

HMP2_2899_1

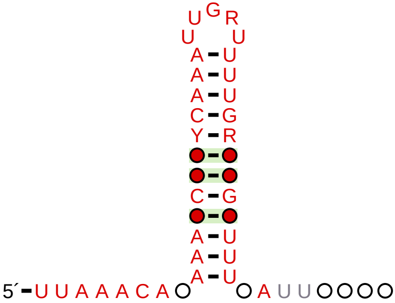

HMP2_29_1

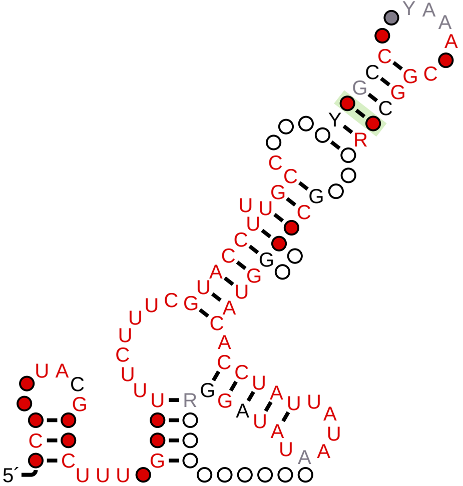

HMP2_2906_1

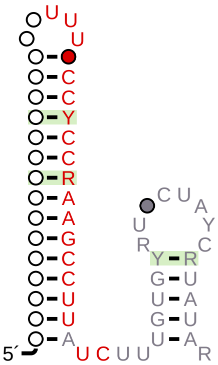

HMP2_2911_1

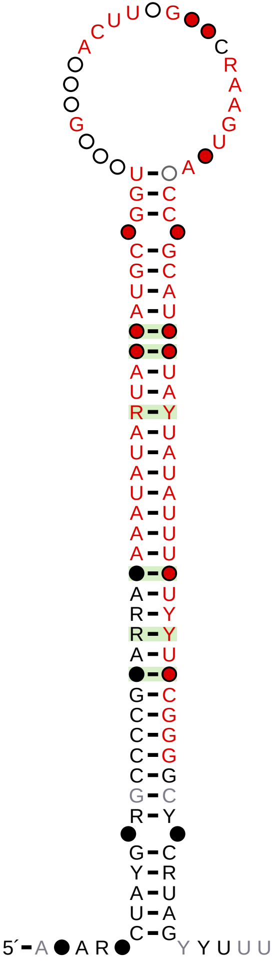

HMP2_2914_1

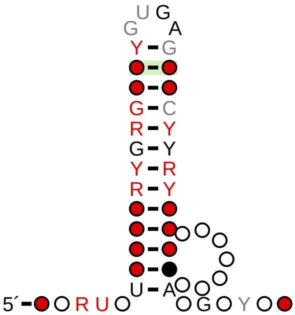

HMP2_2922_1

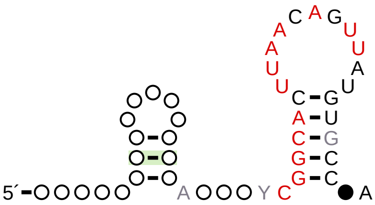

HMP2_2931_1

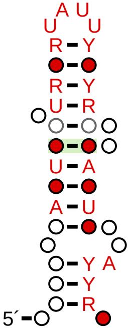

HMP2_2937_1

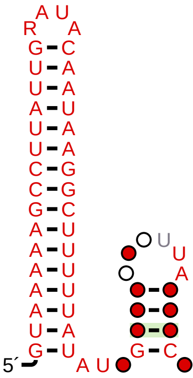

HMP2_2940_1

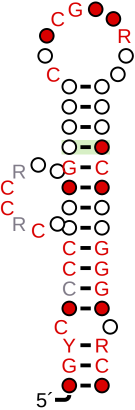

HMP2_2943_1

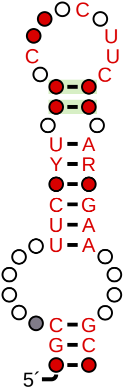

HMP2_2949_1

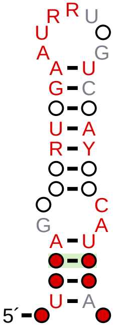

HMP2_2953_1

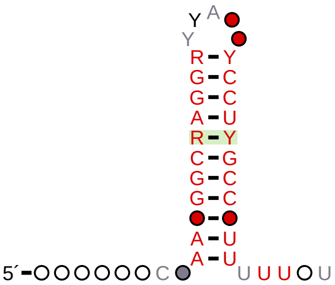

HMP2_2954_1

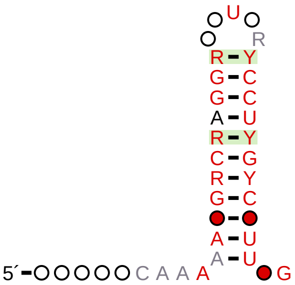

HMP2_2957_1

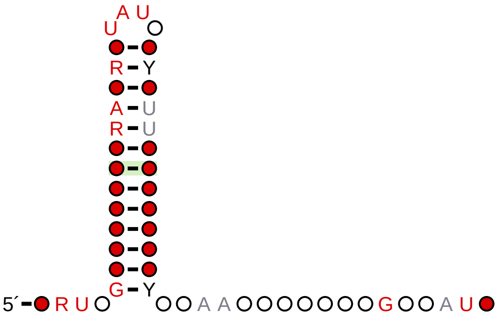

HMP2_2958_1

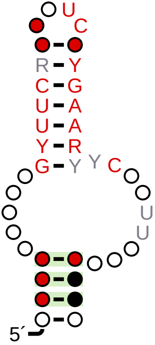

HMP2_2973_1

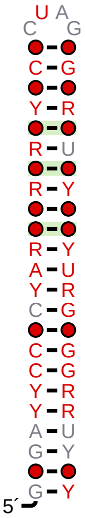

HMP2_2978_1

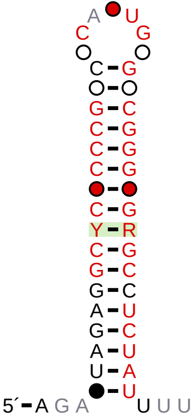

HMP2_2980_1

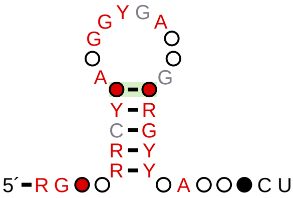

HMP2_2987_1

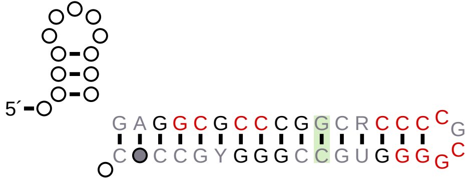

HMP2_2990_1

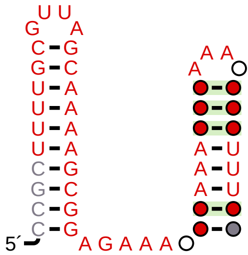

HMP2_2991_1

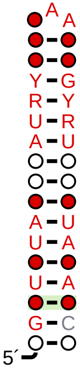

HMP2_2995_1

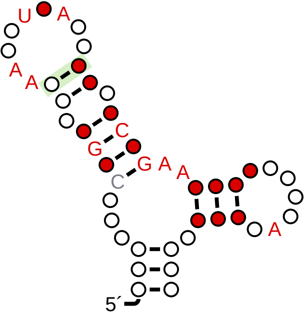

HMP2_3_1

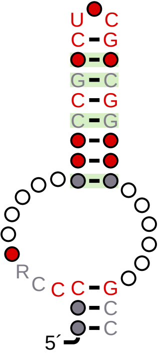

HMP2_300_1

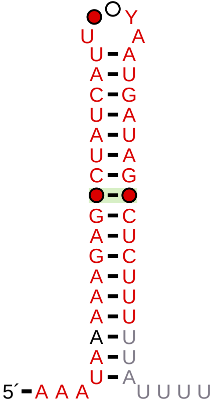

HMP2_3000_1

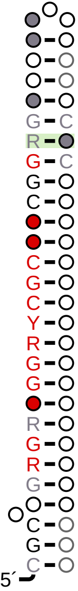

HMP2_3006_1

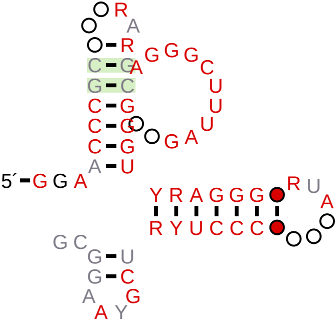

HMP2_301_1

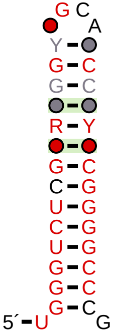

HMP2_3010_1

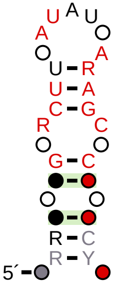

HMP2_3023_1

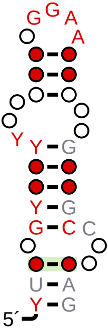

HMP2_3024_1

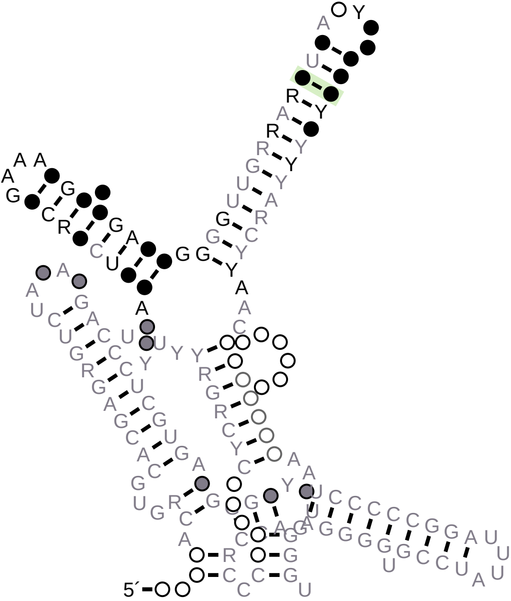

HMP2_3039_1

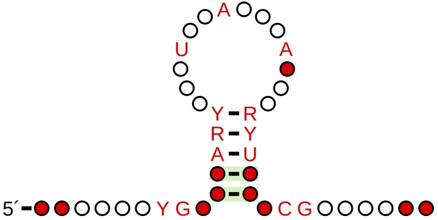

HMP2_304_1

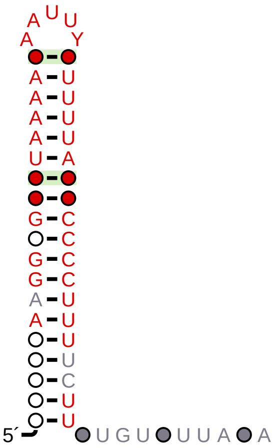

HMP2_3043_1

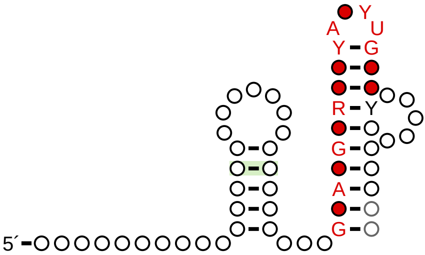

HMP2_3053_1

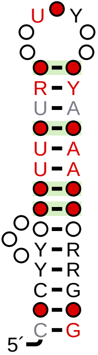

HMP2_3055_1

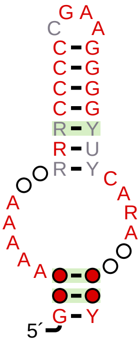

HMP2_3056_1

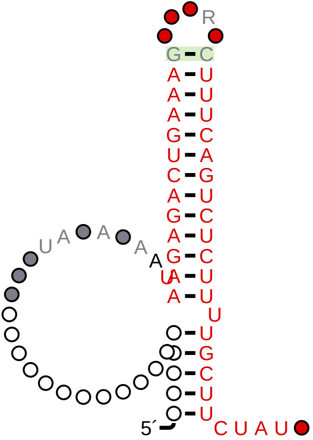

HMP2_3060_1

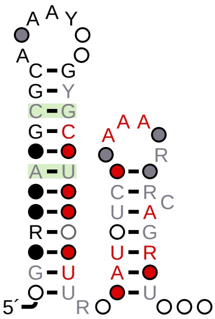

HMP2_3061_1

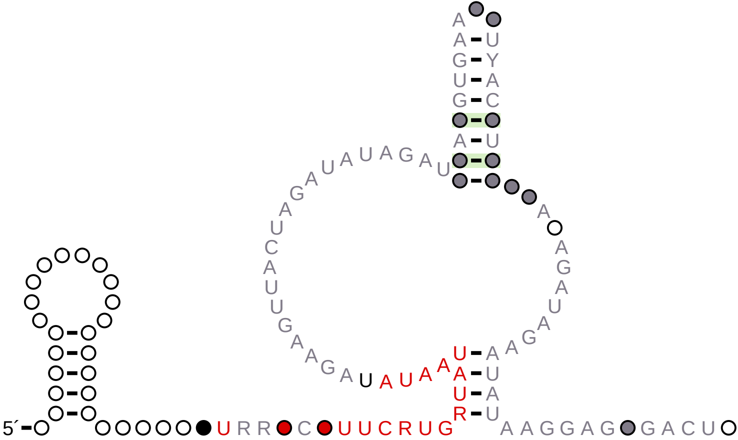

HMP2_3069_1

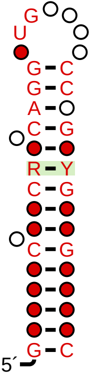

HMP2_3070_1

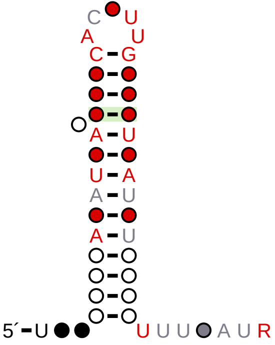

HMP2_3072_1

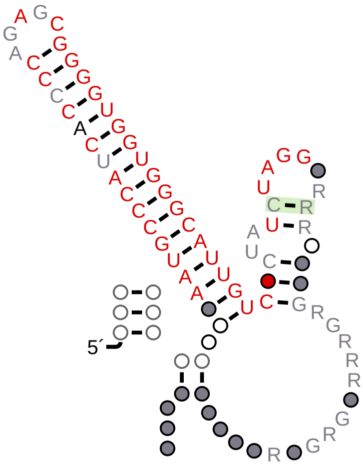

HMP2_3074_1

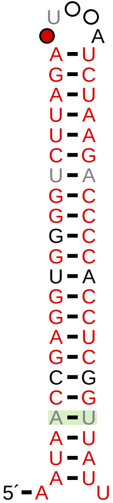

HMP2_3080_1

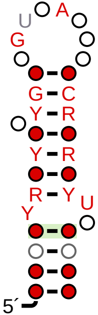

HMP2_3081_1

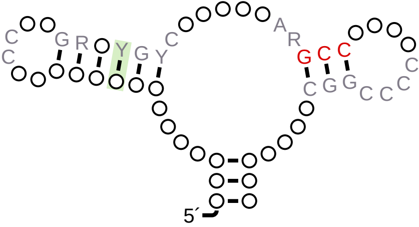

HMP2_3092_1

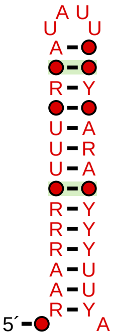

HMP2_3093_1

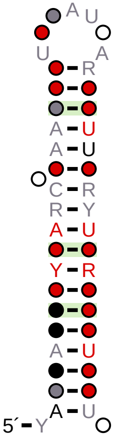

HMP2_3098_1

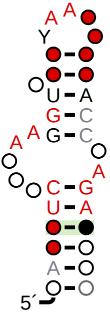

HMP2_310_1

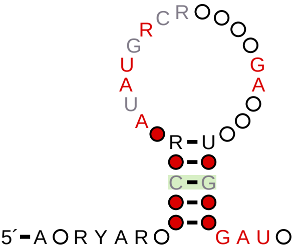

HMP2_3103_1

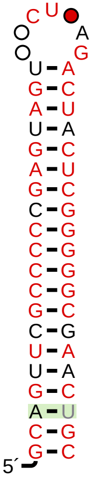

HMP2_3106_1

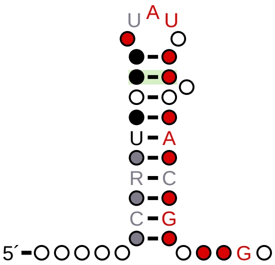

HMP2_3112_1

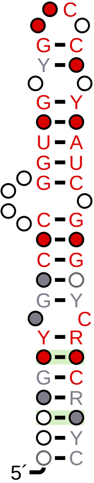

HMP2_3115_1

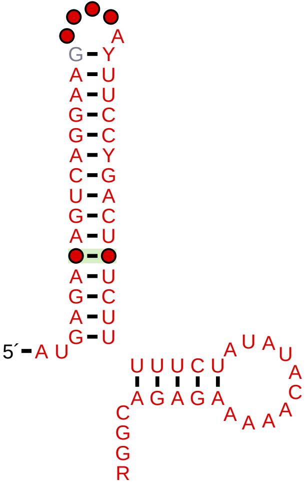

HMP2_3118_1

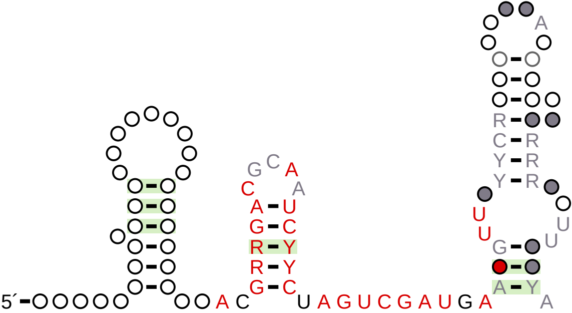

HMP2_3121_1

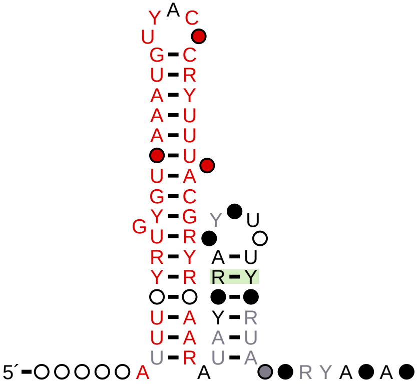

HMP2_3125_1

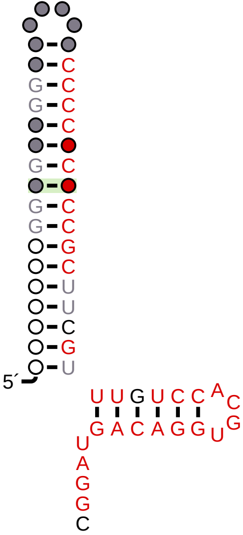

HMP2_3129_1

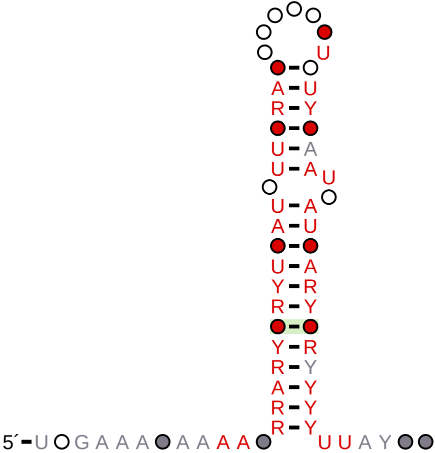

HMP2_3132_1

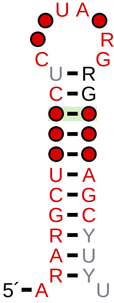

HMP2_3136_1

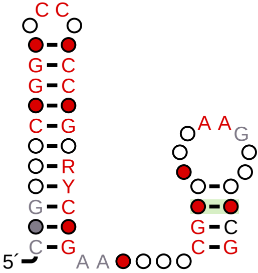

HMP2_3140_1

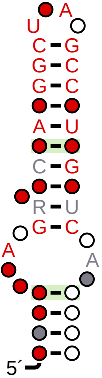

HMP2_3146_1

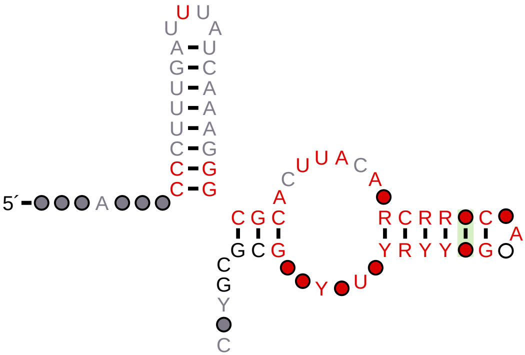

HMP2_315_1

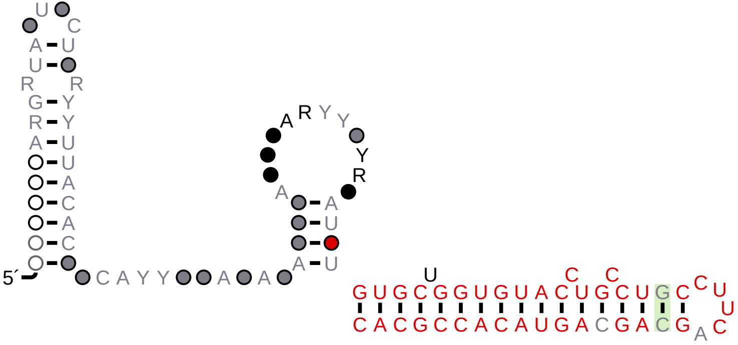

HMP2_3156_1

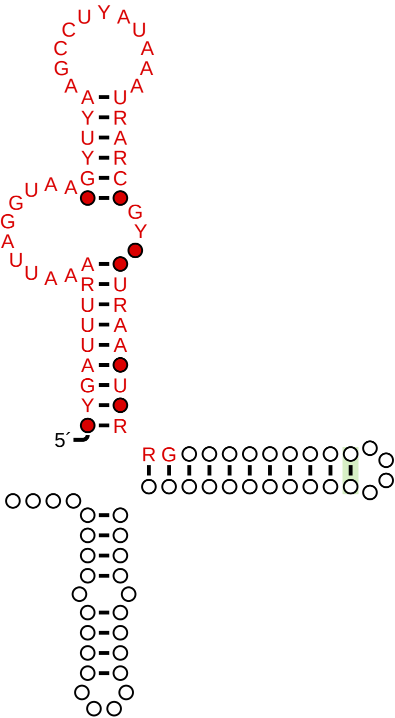

HMP2_3162_1

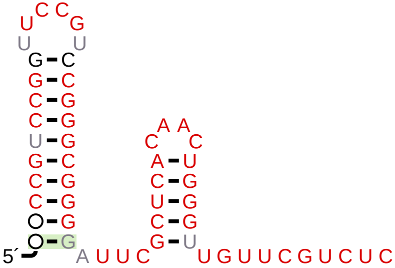

UCCG
U U
G C
G C
C G
C G
U G
G C
C G
C G
O G
O G
CAAC
A U
C G
U G
C G
G U
5´
A U U C
U G U U C G U C U C

HMP2_3175_1

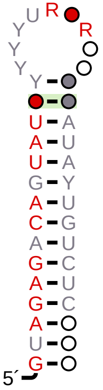

HMP2_3177_1

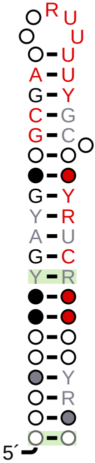

HMP2_3178_1

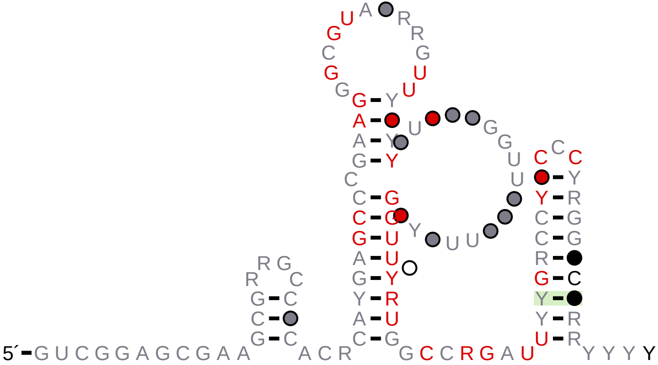

HMP2_3181_1

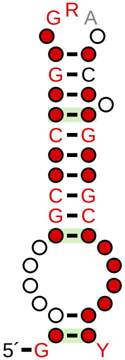

HMP2_3183_1

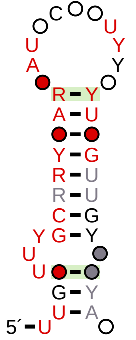

HMP2_3184_1

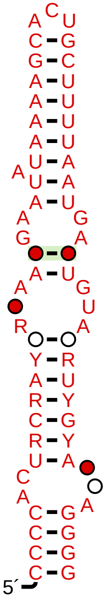

HMP2_3186_1

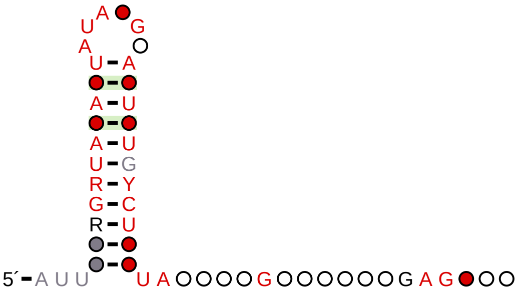

HMP2_3187_1

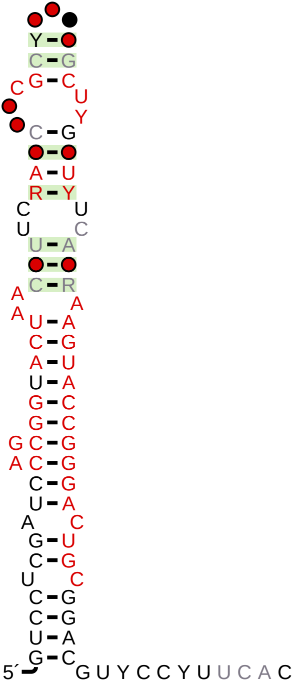

HMP2_319_1

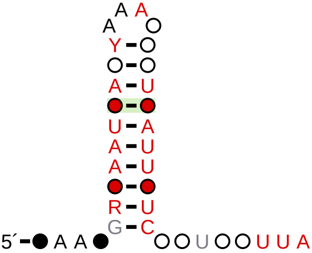

HMP2_3190_1

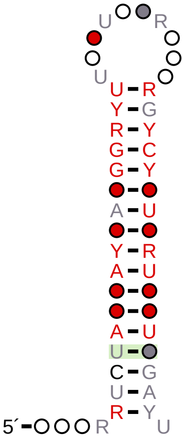

HMP2_3191_1

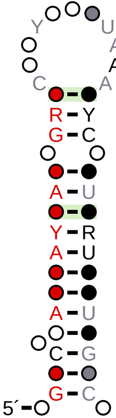

HMP2_3203_1

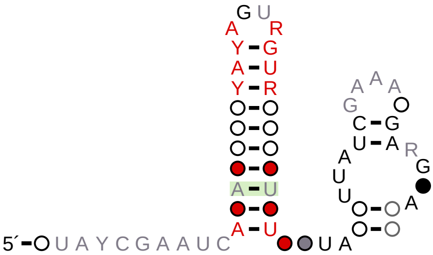

HMP2_321_1

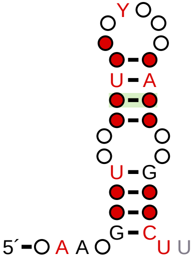

HMP2_3218_1

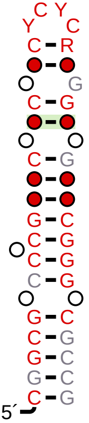

HMP2_3219_1

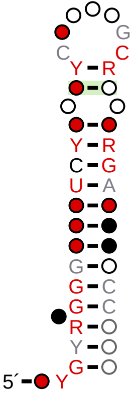

HMP2_322_1

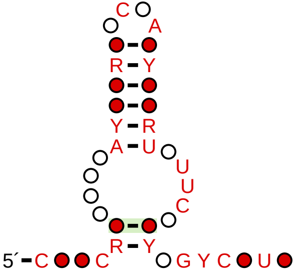

HMP2_3221_1

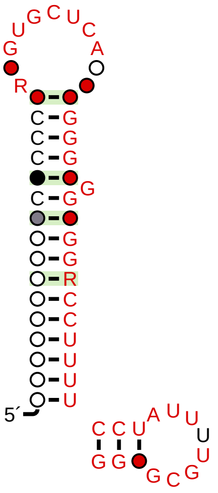

HMP2_3227_1

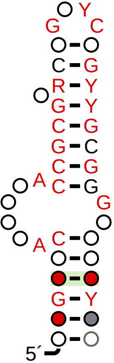

HMP2_3233_1

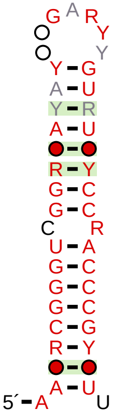

HMP2_3240_1

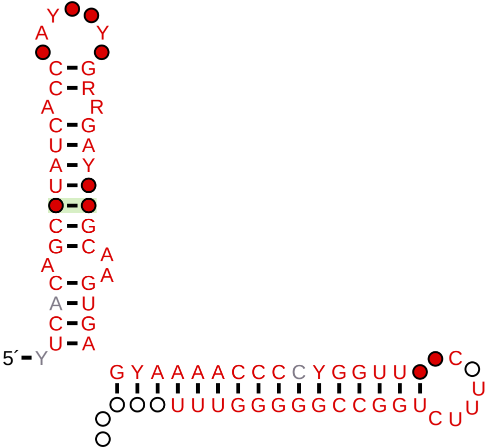
5´
Y
U A
C G
A U
C G
A
G C
C G
A
A
U
A Y
U A
C G
A
R
C R
C G
A
Y
Y
G Y A A A C C C C Y G G U U
C
U
U
U
C U
U U U G G G G G C C G G U

HMP2_3241_1

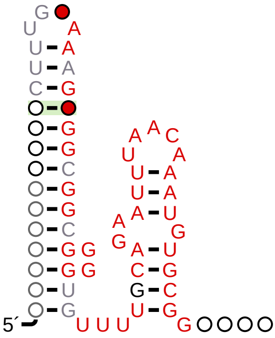

HMP2_3242_1

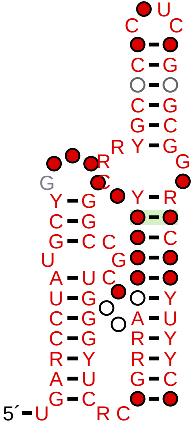

HMP2_3249_1

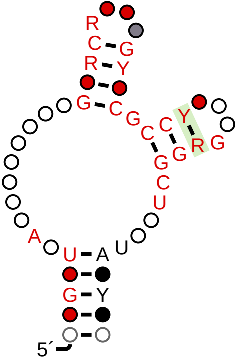

HMP2_3256_1

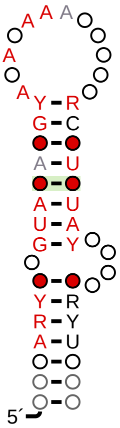

HMP2_3261_1

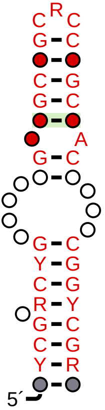

HMP2_3262_1

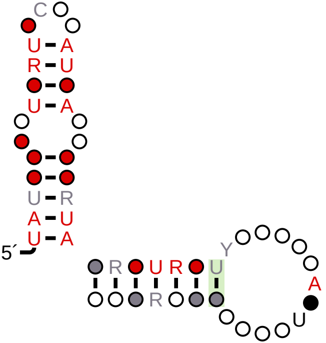

HMP2_3267_1

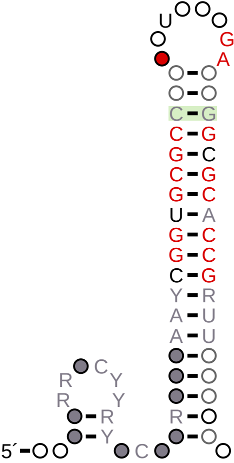

HMP2_3277_1

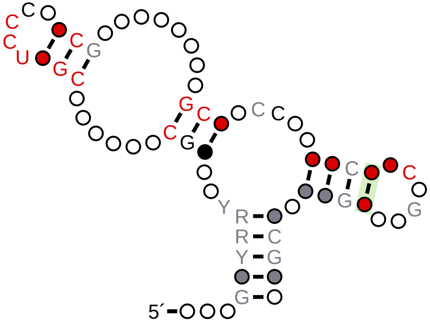

HMP2_328_1

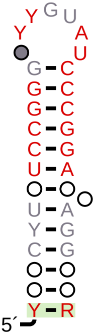

HMP2_3280_1

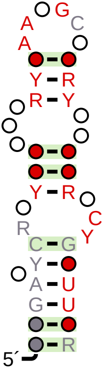

HMP2_3282_1

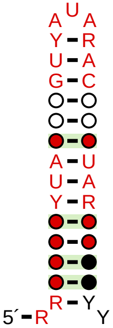

HMP2_3289_1

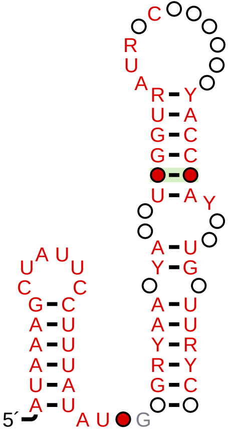

HMP2_3290_1

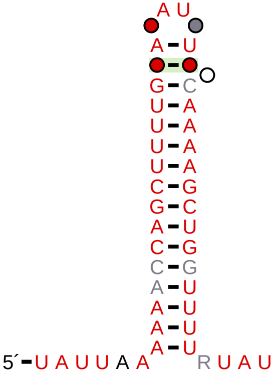

HMP2_3294_1

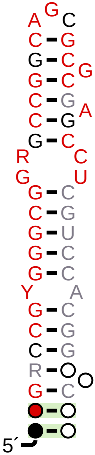

HMP2_3295_1

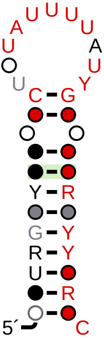

HMP2_3302_1

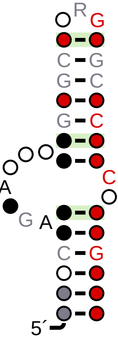

HMP2_3303_1

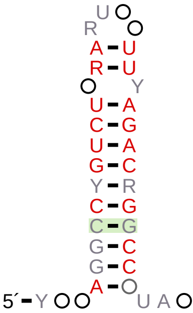

HMP2_3306_1

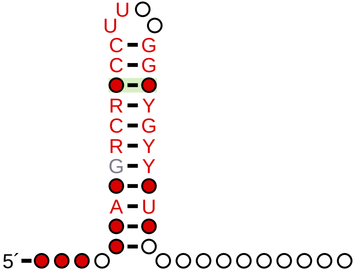

HMP2_331_1

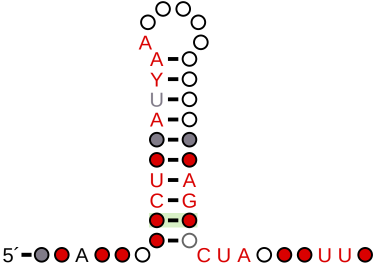

HMP2_3310_1

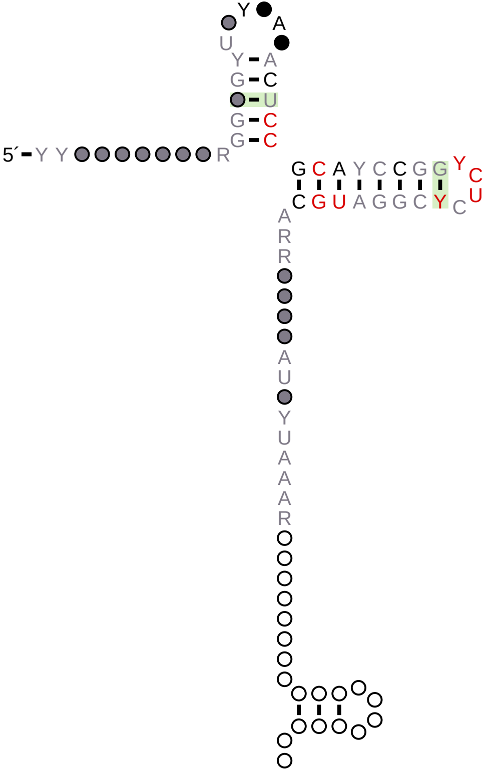

HMP2_3311_1

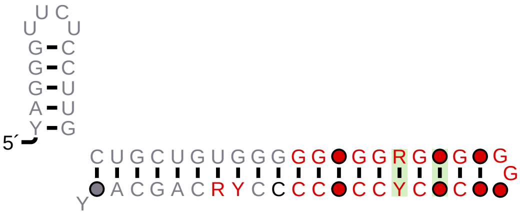

HMP2_3314_1

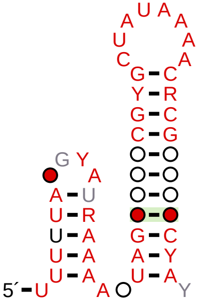

HMP2_3317_1

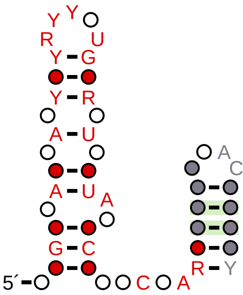

HMP2_332_1

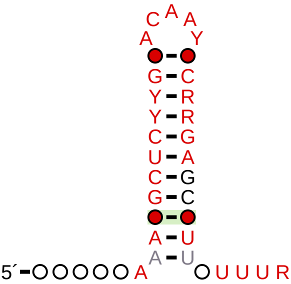

HMP2_3322_1

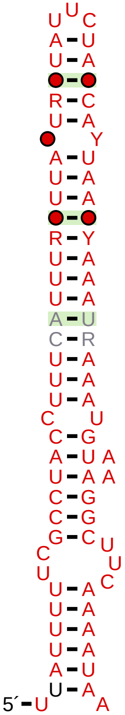

HMP2_3327_1

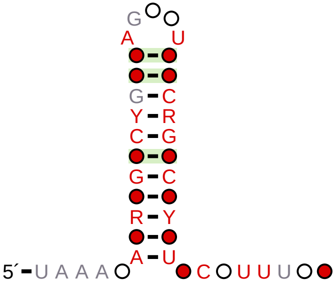

HMP2_3332_1

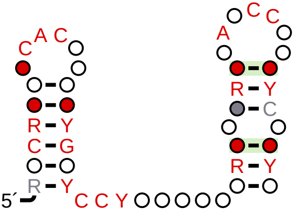

HMP2_3334_1

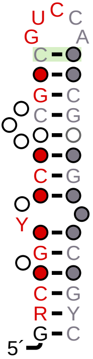

HMP2_3337_1

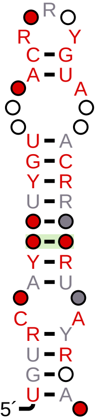

HMP2_3344_1

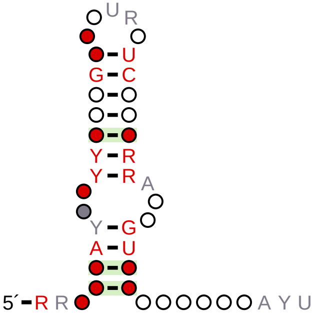

HMP2_3347_1

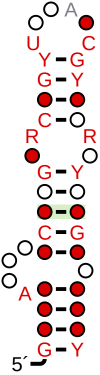

HMP2_3352_1

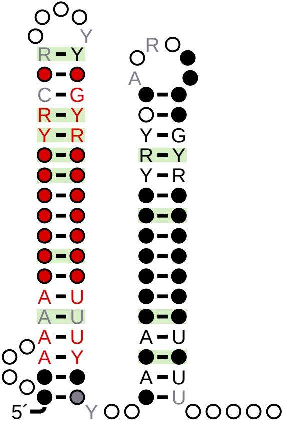

HMP2_3353_1

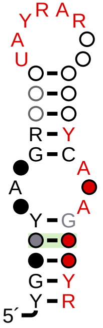

HMP2_3365_1

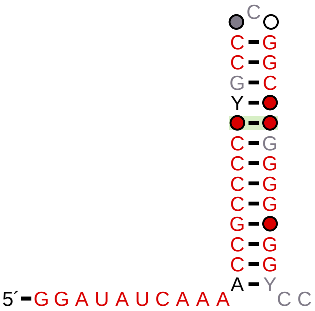

HMP2_3371_1

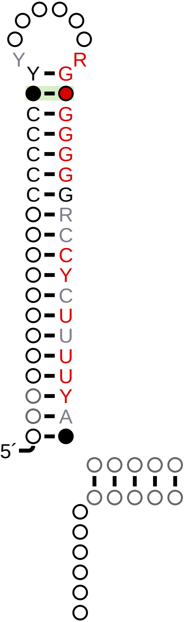

HMP2_3379_1

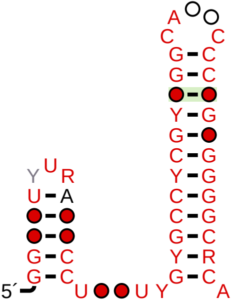

HMP2_3387_1

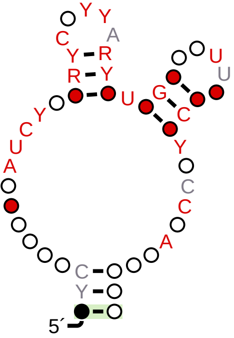

HMP2_3392_1

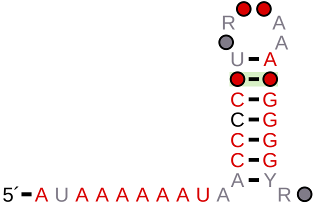

HMP2_3393_1

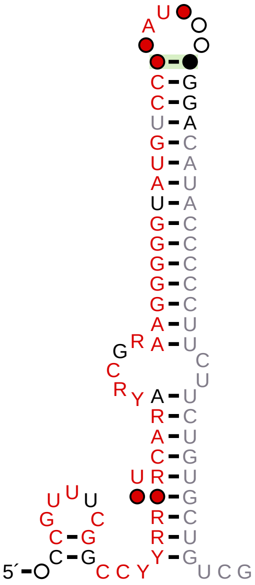

HMP2_3403_1

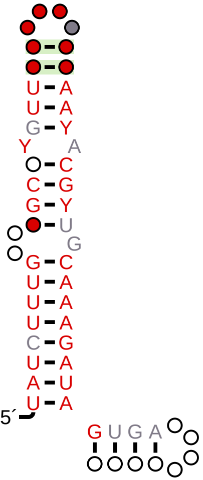

HMP2_3404_1

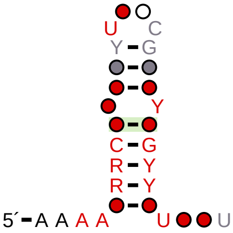

HMP2_3406_1

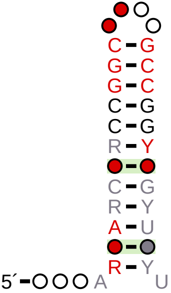

HMP2_3411_1

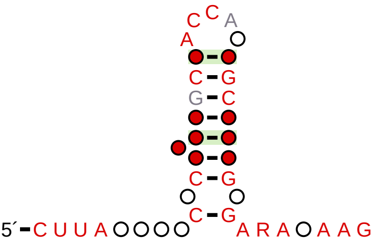

HMP2_3413_1

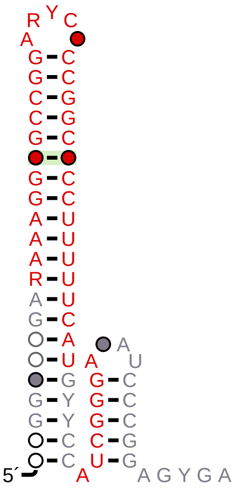

R Y C
A
G C
G C
C G
C G
G C
G C
G C
A U
A U
A U
R U
A U
G C
A
U
G
G Y
G Y
C
C
A U
G C
G C
G C
C G
U G
5´
A
A G Y G A

HMP2_3414_1

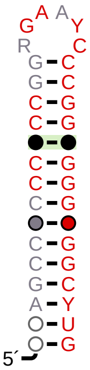

HMP2_3424_1

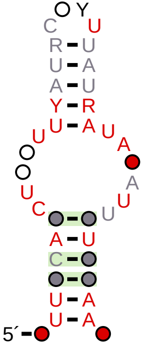

HMP2_3427_1

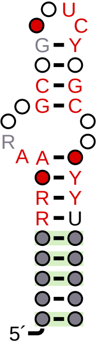

HMP2_3429_1

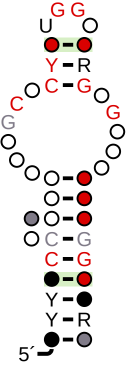

HMP2_344_1

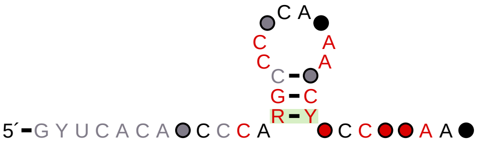

HMP2_3443_1

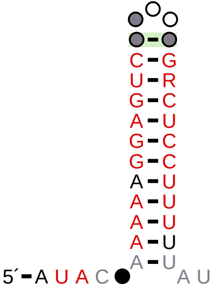

HMP2_3444_1

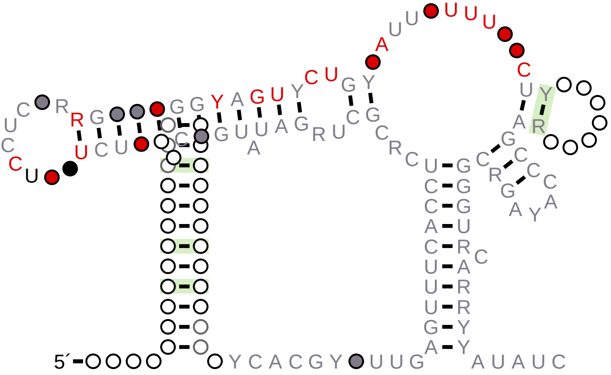

HMP2_3452_1

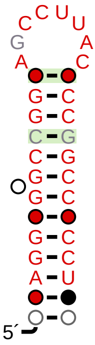

HMP2_3459_1

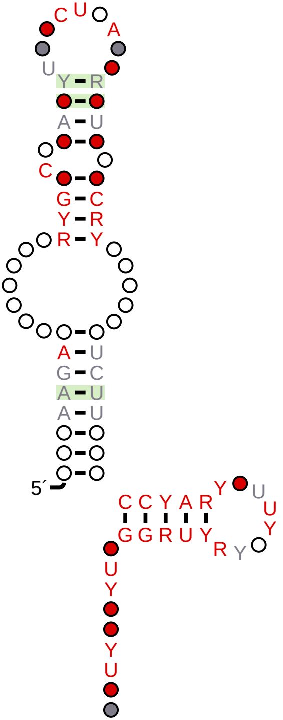

HMP2_3469_1

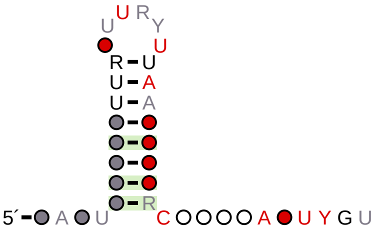

HMP2_3474_1

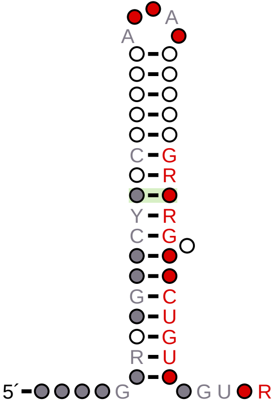

HMP2_3475_1

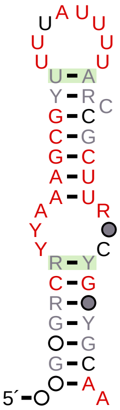

HMP2_3493_1

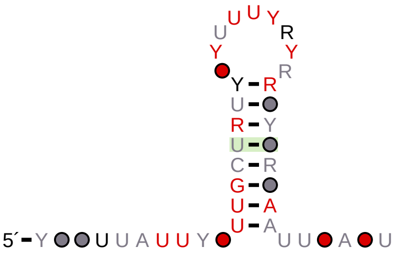

HMP2_3495_1

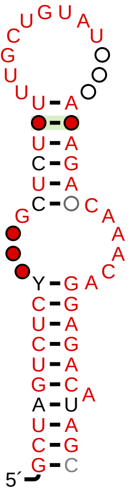

HMP2_3496_1

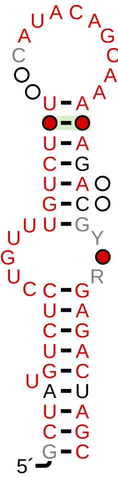

HMP2_350_1

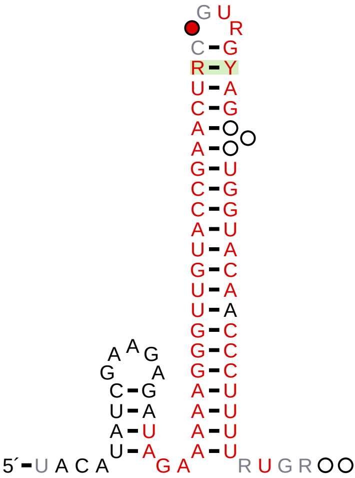

HMP2_3500_1

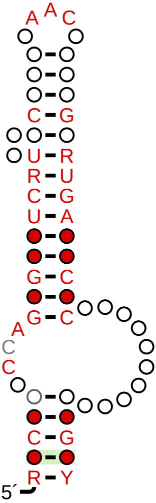

HMP2_3502_1

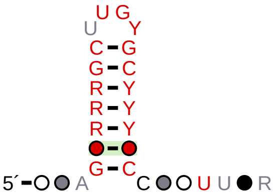

HMP2_3504_1

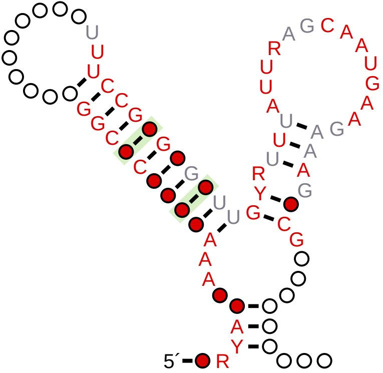

HMP2_351_1

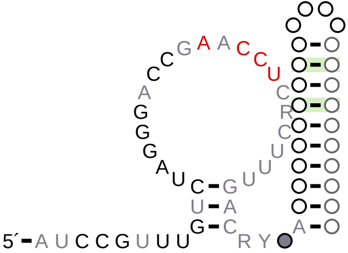

HMP2_3510_1

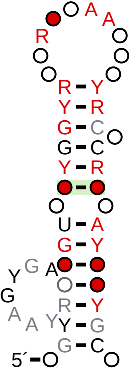

HMP2_3514_1

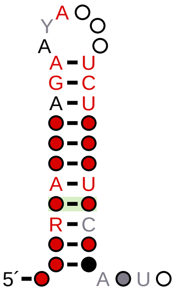

HMP2_3516_1

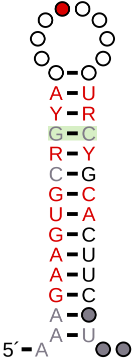

HMP2_3521_1

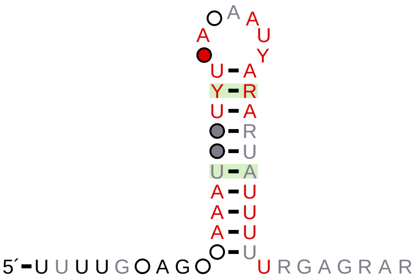

HMP2_3524_1

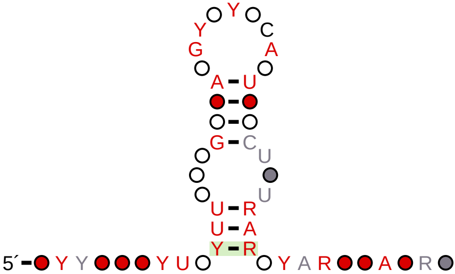

HMP2_3526_1

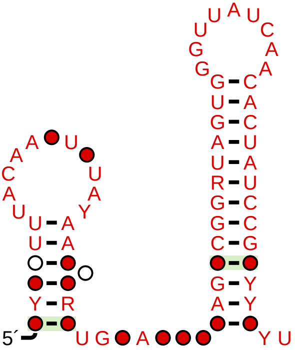

HMP2_3533_1

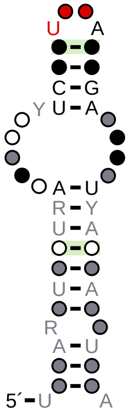

HMP2_3540_1

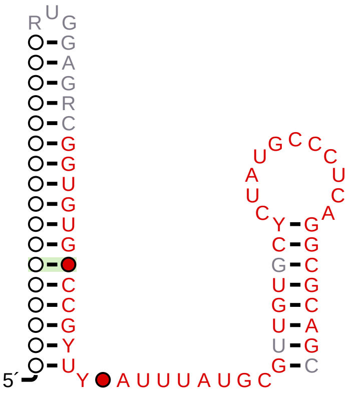

HMP2_3551_1

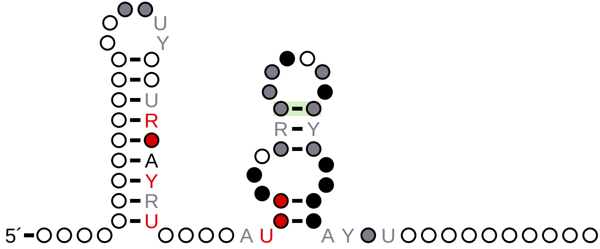

HMP2_3556_1

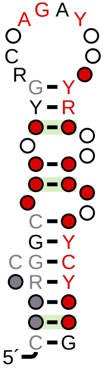

HMP2_3559_1

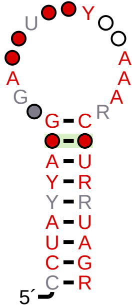

HMP2_3563_1

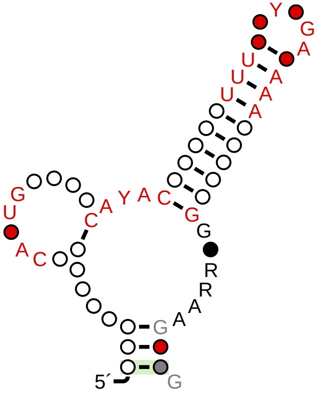

HMP2_3564_1

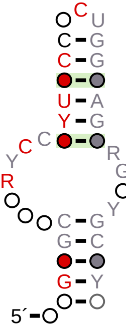

HMP2_3565_1

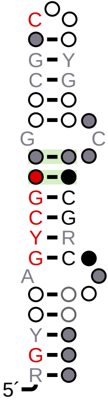

HMP2_3570_1

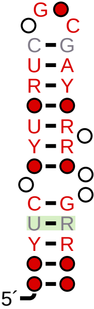

HMP2_3571_1

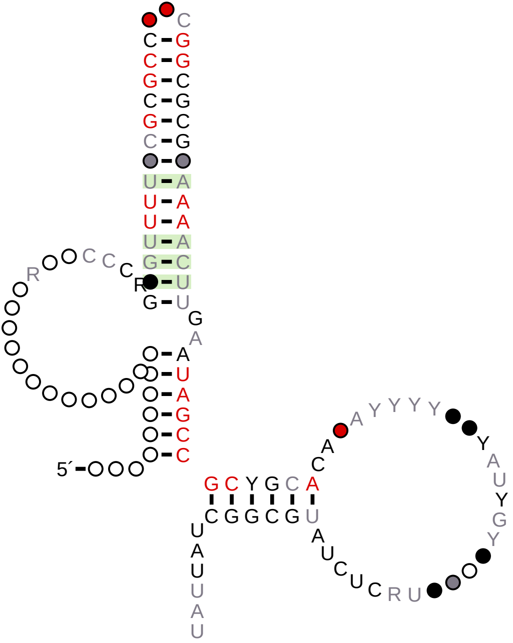

HMP2_3576_1

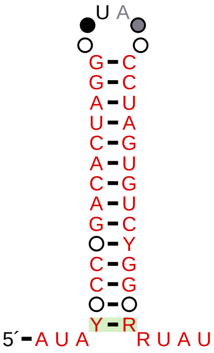

HMP2_359_1

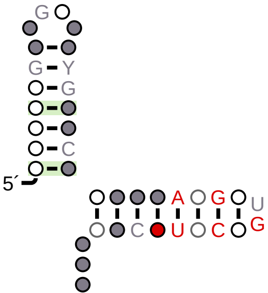

HMP2_3594_1

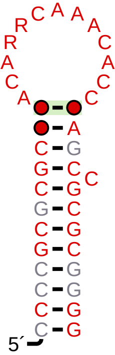

HMP2_3597_1

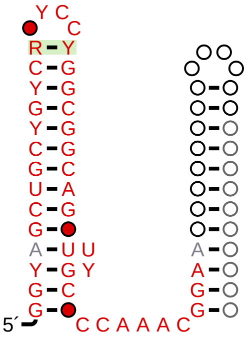

HMP2_3598_1

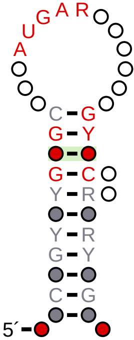

HMP2_3599_1

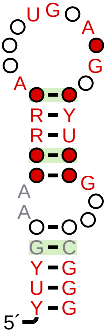

HMP2_3614_1

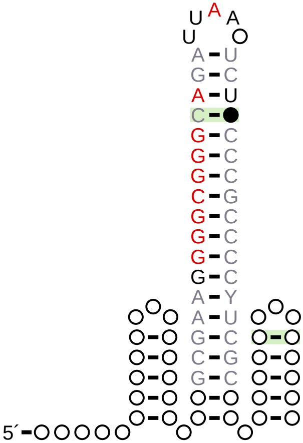

HMP2_362_1

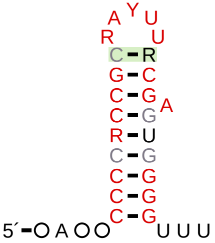

HMP2_3621_1

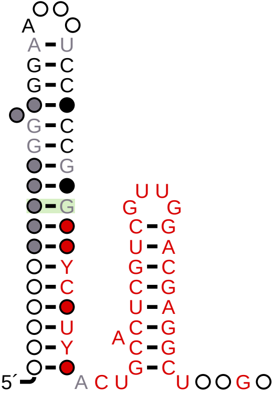

HMP2_3628_1

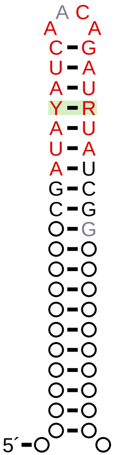

HMP2_3629_1

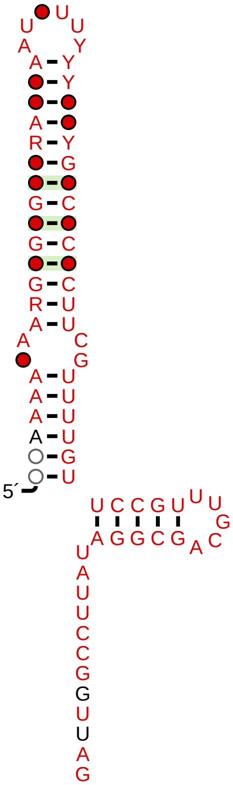

HMP2_3634_1

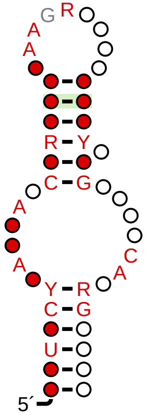

HMP2_3638_1

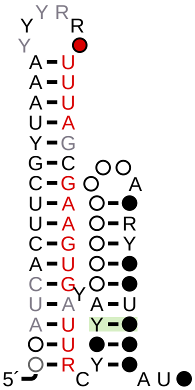

HMP2_3640_1

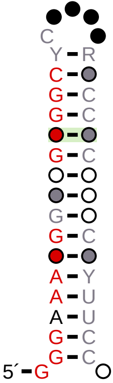

HMP2_3645_1

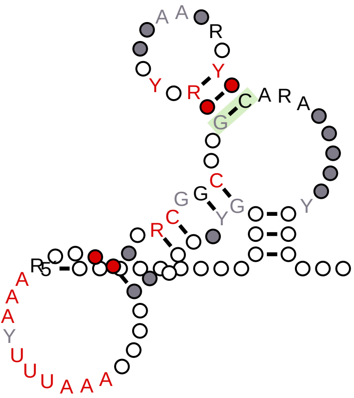

HMP2_3651_1

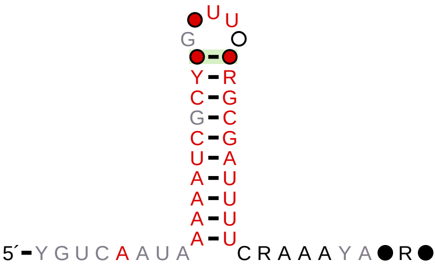

HMP2_3655_1

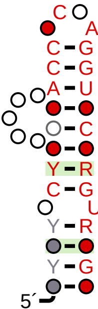

HMP2_3678_1

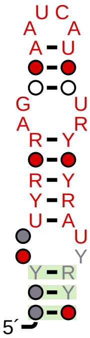

HMP2_368_1

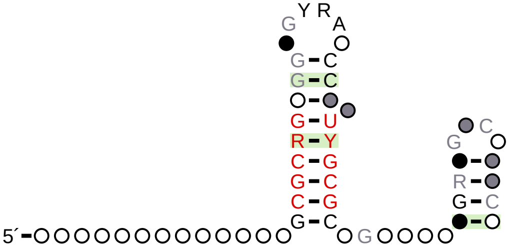

HMP2_3680_1

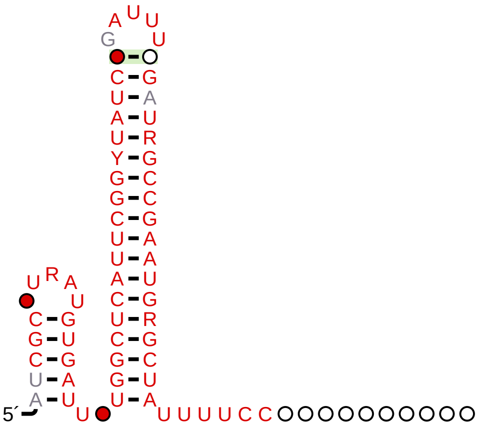

HMP2_3686_1

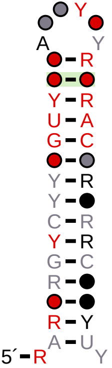

HMP2_3687_1

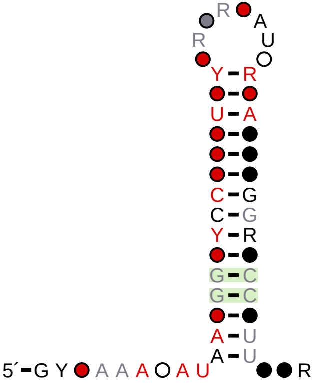

HMP2_3689_1

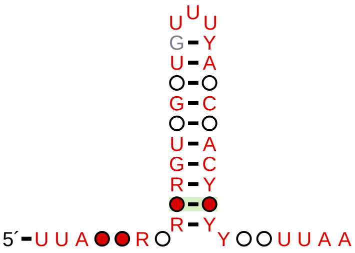

HMP2_3698_1

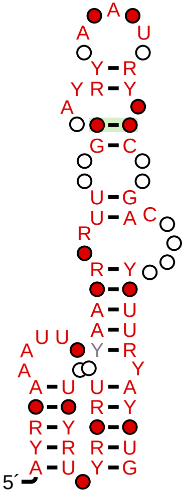

HMP2_3701_1

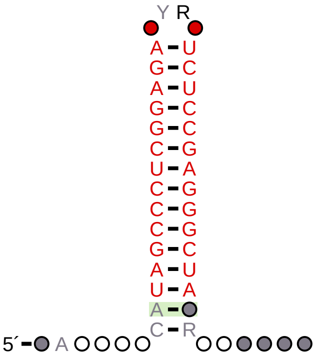

HMP2_3715_1

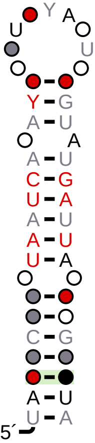

HMP2_3716_1

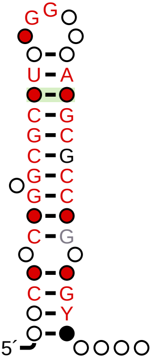

HMP2_3718_1

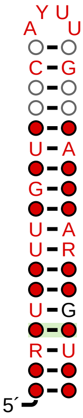

HMP2_372_1

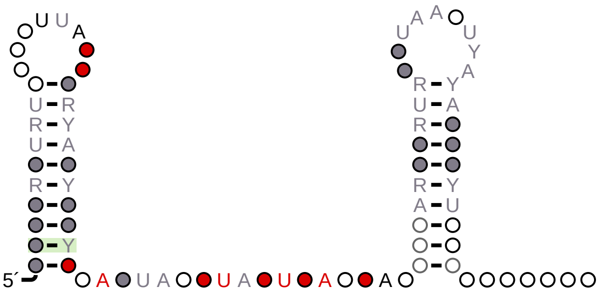

HMP2_3720_1

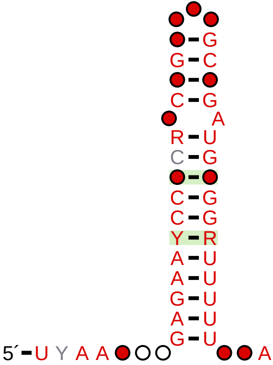

HMP2_3723_1

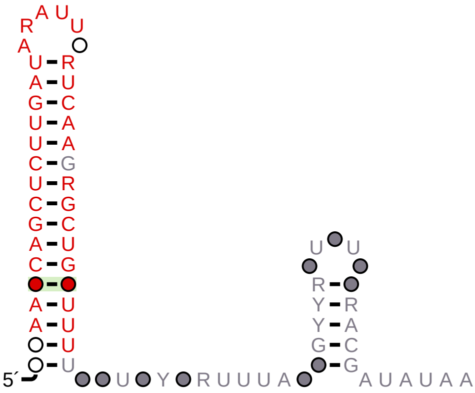

HMP2_3724_1

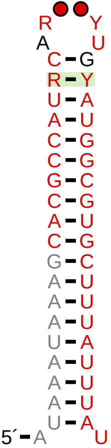

HMP2_373_1

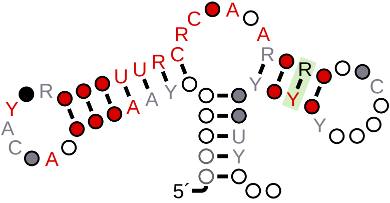

HMP2_3732_1

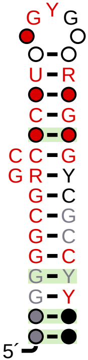

HMP2_3745_1

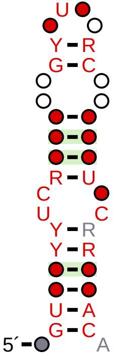

HMP2_3746_1

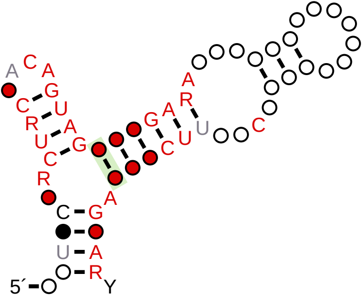

HMP2_3747_1

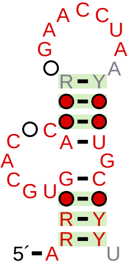

HMP2_3748_1

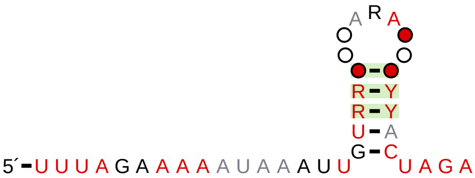

HMP2_3750_1

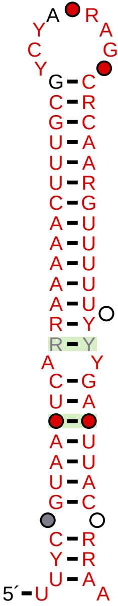

HMP2_3756_1

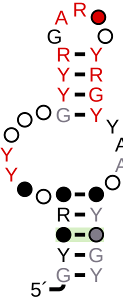

HMP2_376_1

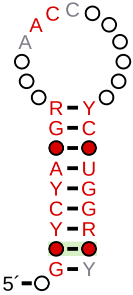

HMP2_3766_1

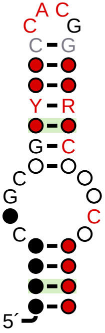

HMP2_3772_1

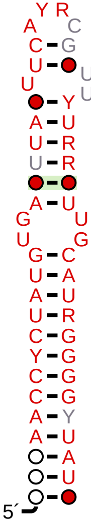

HMP2_3783_1

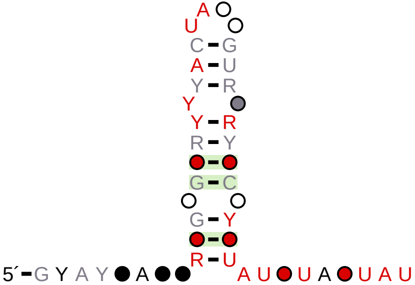

HMP2_3794_1

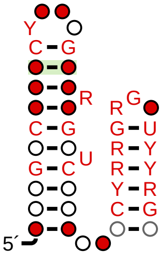

HMP2_3795_1

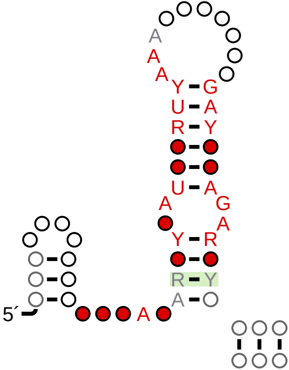

HMP2_38_1

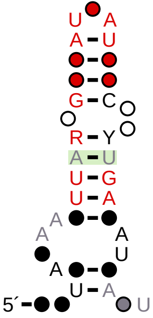

HMP2_3804_1

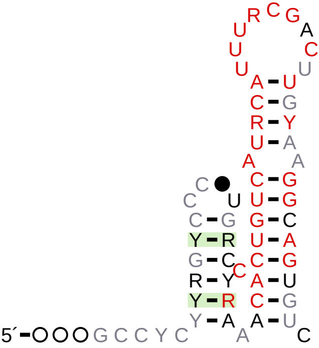

HMP2_3806_1

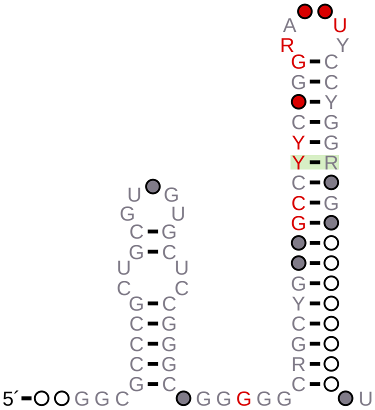

HMP2_3807_1

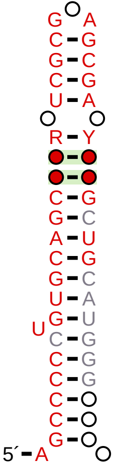

HMP2_381_1

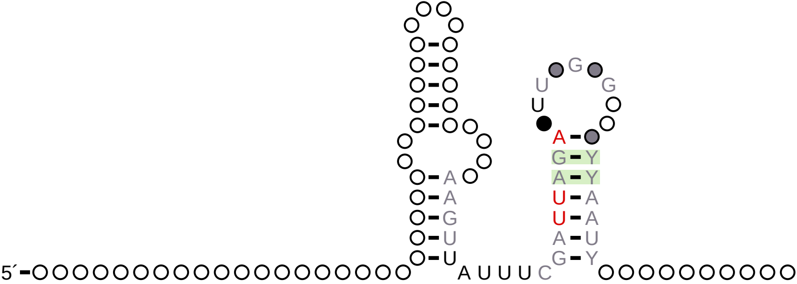

HMP2_3816_1

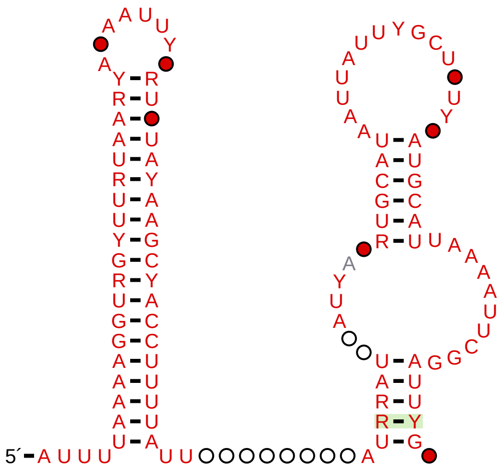

HMP2_382_1

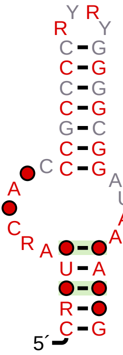

HMP2_3822_1

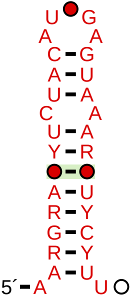

HMP2_3826_1

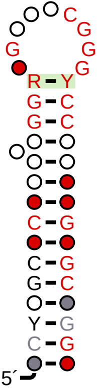

HMP2_3837_1

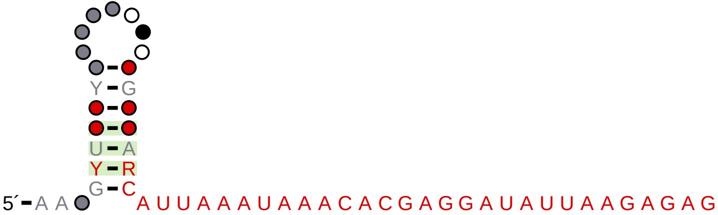

HMP2_384_1

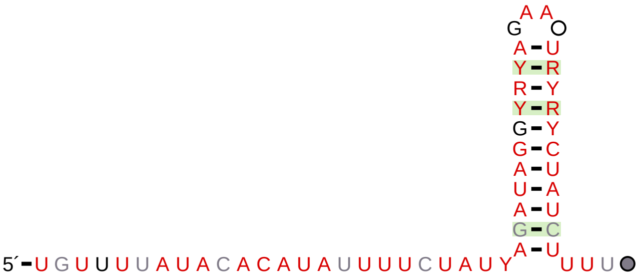

A A
G
A U
Y R
R Y
Y R
G Y
G C
A U
U A
A U
G C
A U
5´ U G U U U U A U A C A C A U A U U U U C U A U Y
U U U

HMP2_3841_1

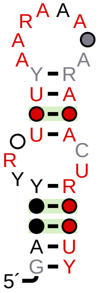

HMP2_3848_1

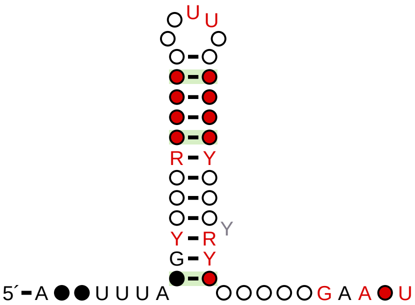

HMP2_3849_1

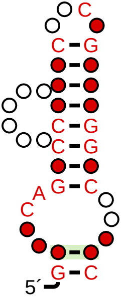

HMP2_3854_1

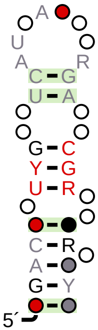

HMP2_3859_1

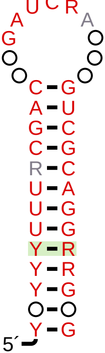

HMP2_3865_1

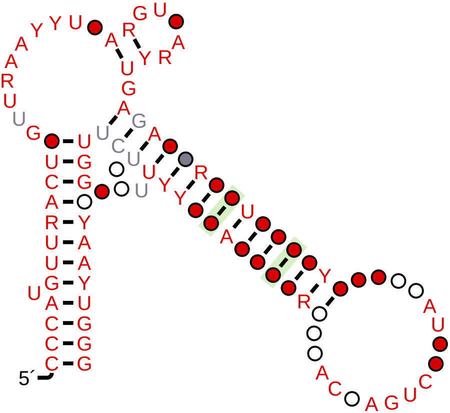

HMP2_3867_1

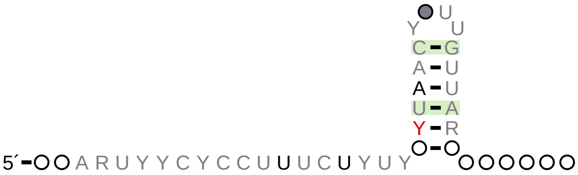

HMP2_3870_1

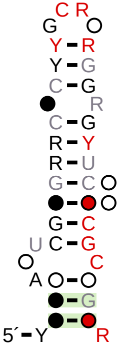

HMP2_3871_1

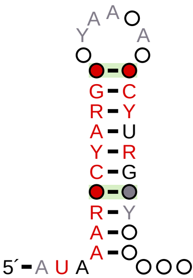

HMP2_3874_1

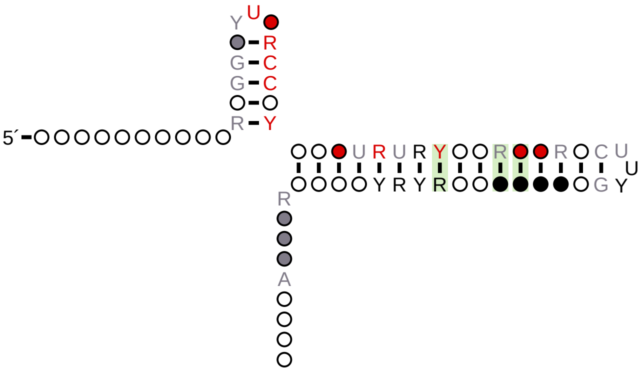

HMP2_3877_1

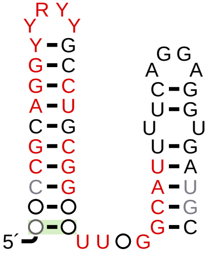

HMP2_3893_1

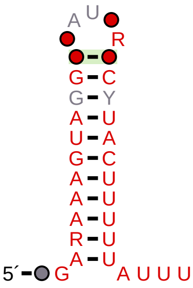

HMP2_3895_1

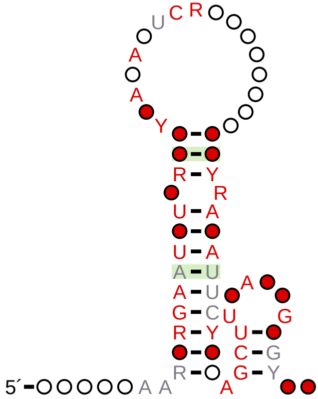

HMP2_3897_1

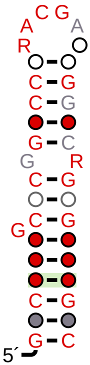

HMP2_3899_1

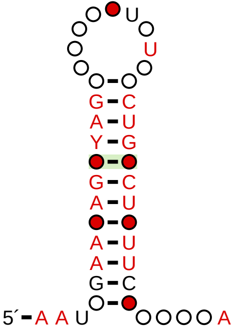

HMP2_39_1

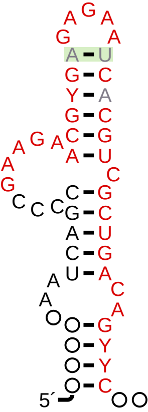

HMP2_3901_1

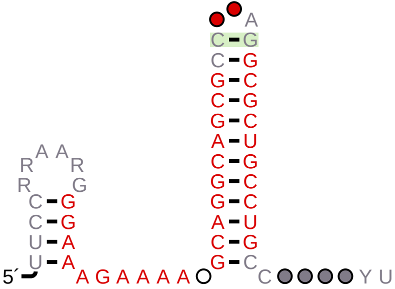

HMP2_3911_1

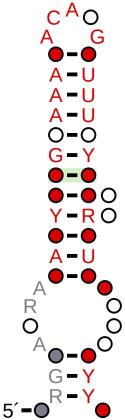

HMP2_3917_1

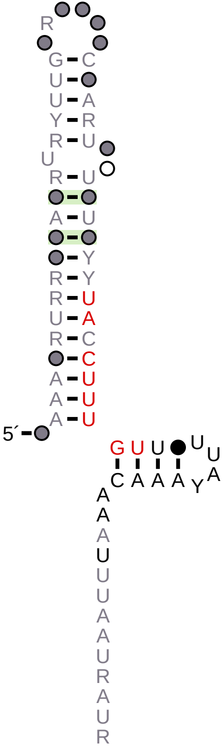

HMP2_3922_1

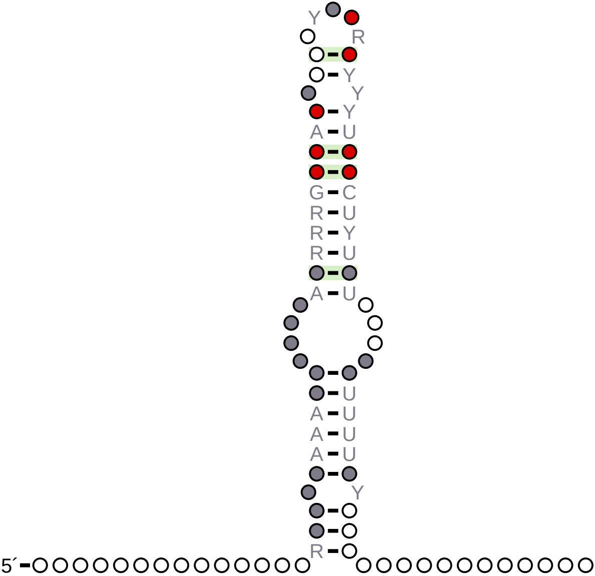

HMP2_3926_1

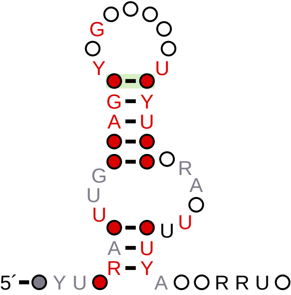

HMP2_3933_1

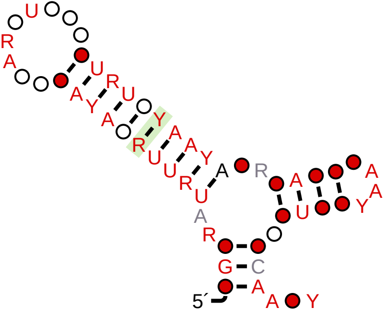

HMP2_3945_1

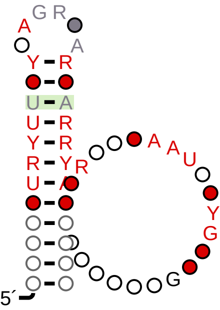

HMP2_3963_1

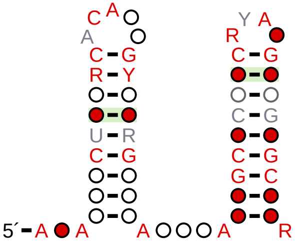

HMP2_3977_1

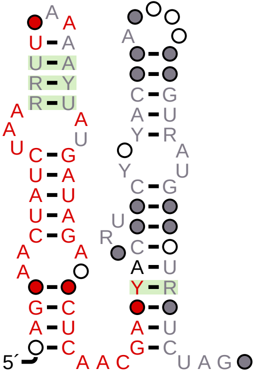

HMP2_3979_1

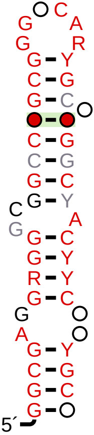

HMP2_3987_1

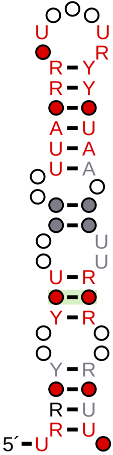

HMP2_3992_1

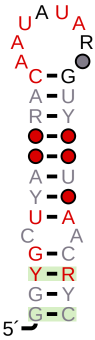

HMP2_3993_1

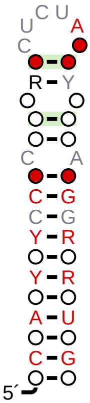

HMP2_3994_1

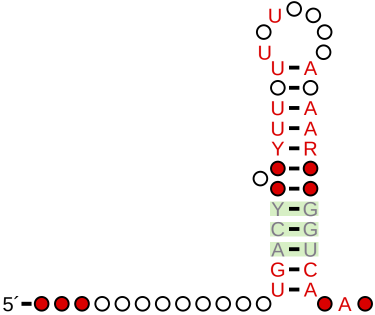

HMP2_40_1

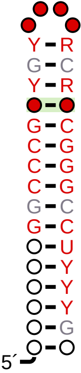

HMP2_4000_1

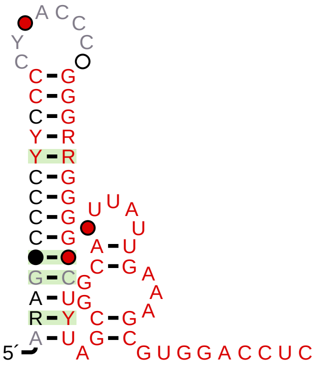

HMP2_4009_1

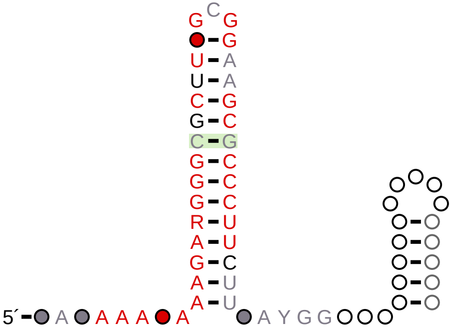

HMP2_4010_1

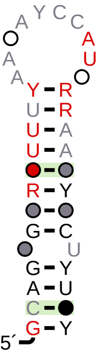

HMP2_4013_1

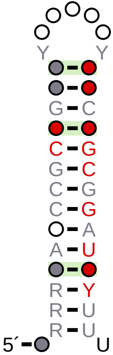

HMP2_4014_1

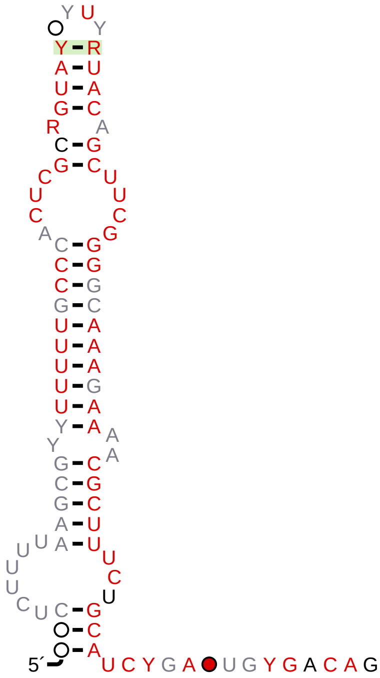

HMP2_4019_1

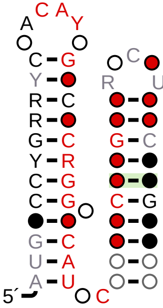

HMP2_4024_1

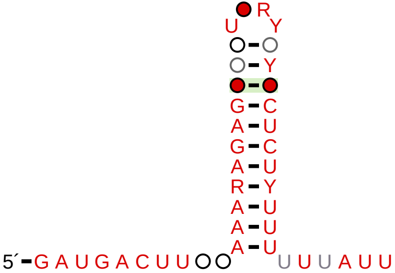

HMP2_4031_1

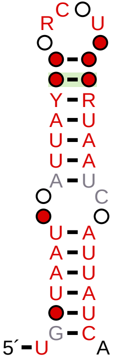

HMP2_4034_1

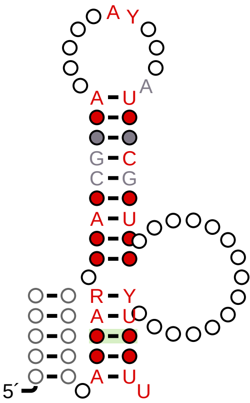

HMP2_4036_1

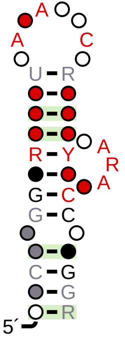

HMP2_4041_1

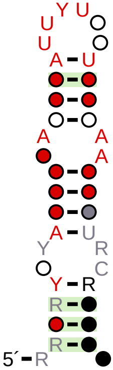

HMP2_4043_1

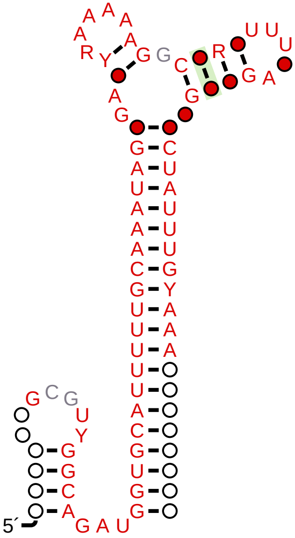

HMP2_4046_1

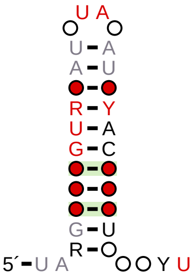

HMP2_4048_1

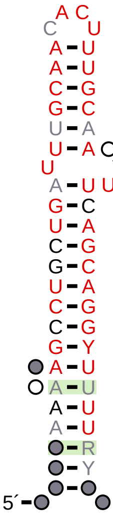

HMP2_405_1

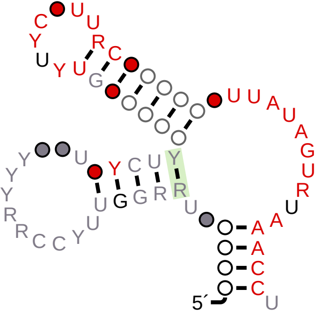

HMP2_4057_1

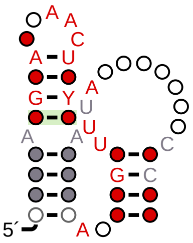

HMP2_4060_1

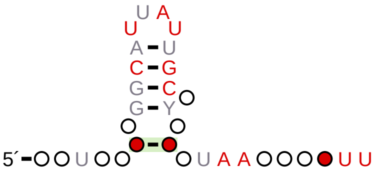

HMP2_4061_1

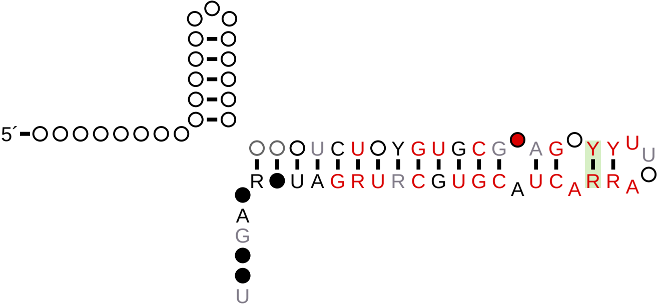

HMP2_4069_1

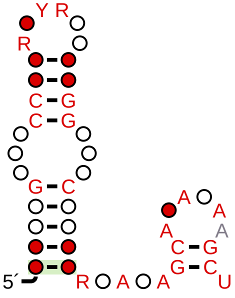

HMP2_4070_1

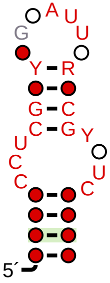

HMP2_4072_1

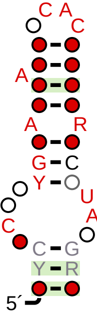

HMP2_4078_1

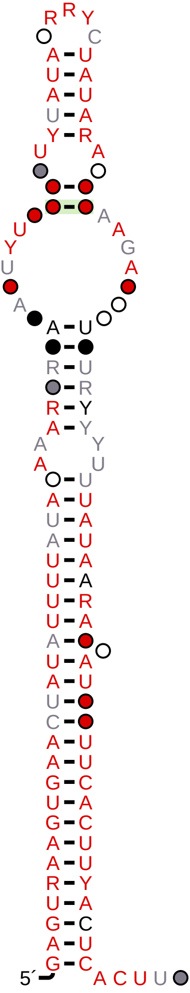

HMP2_4079_1

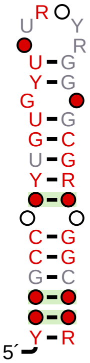

HMP2_408_1

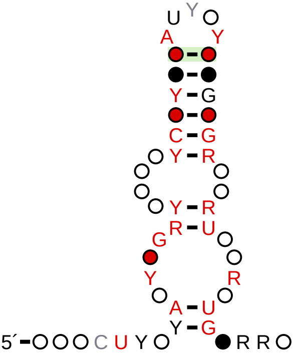

HMP2_4080_1

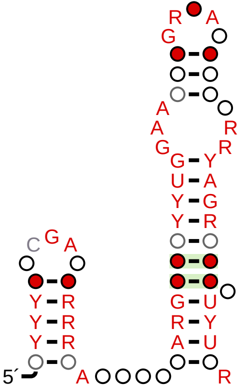

HMP2_4089_1

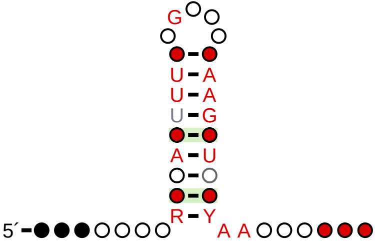

HMP2_4094_1

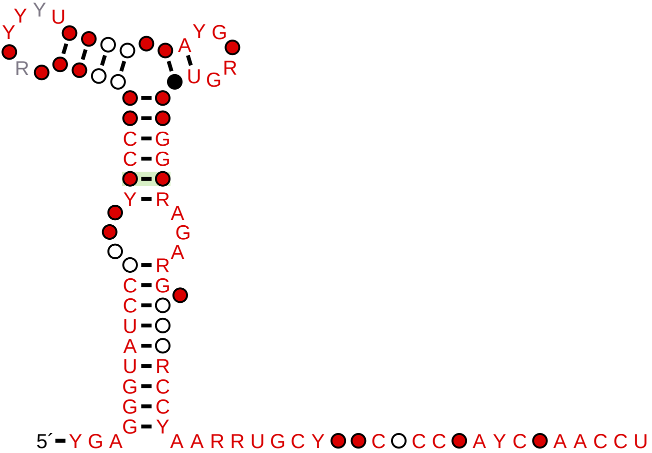

HMP2_4105_1

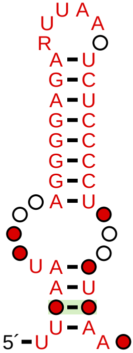

HMP2_4107_1

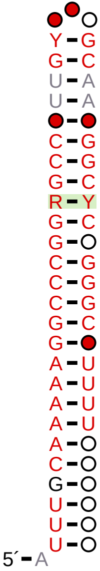

HMP2_4114_1

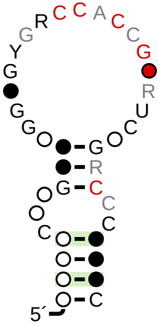

HMP2_4118_1

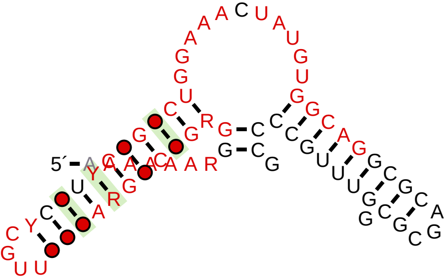

HMP2_4127_1

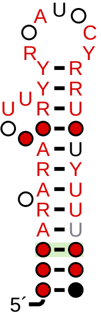

HMP2_4129_1

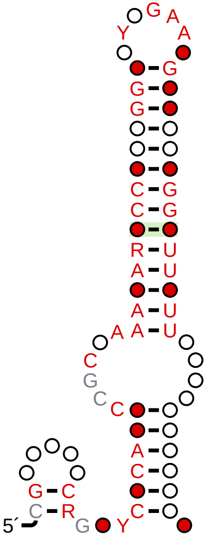

HMP2_4130_1

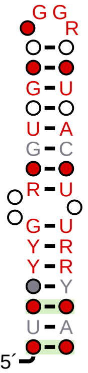

HMP2_4141_1

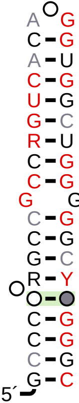

HMP2_4143_1

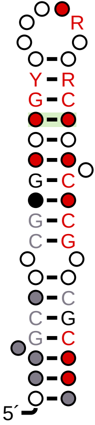

HMP2_4146_1

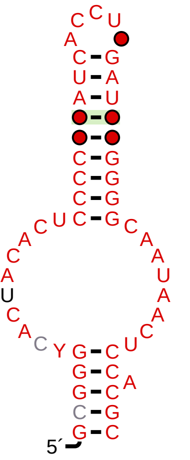

HMP2_4154_1

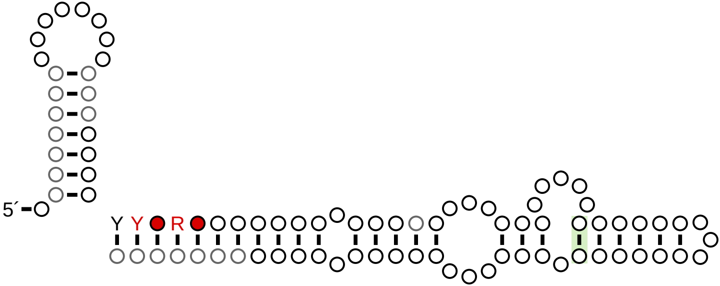

HMP2_4156_1

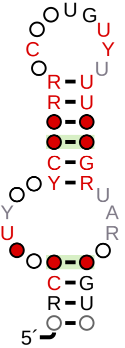

HMP2_4160_1

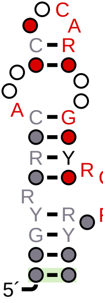

HMP2_4168_1

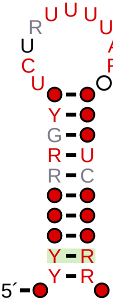

HMP2_4169_1

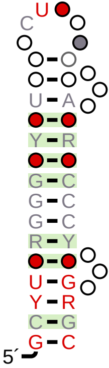

HMP2_417_1

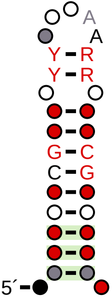

HMP2_4170_1

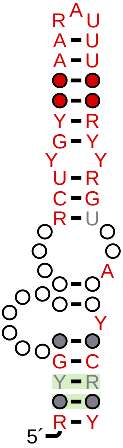

HMP2_4171_1

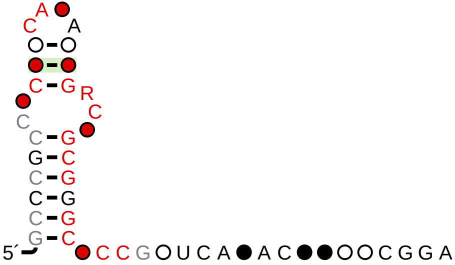

HMP2_4184_1

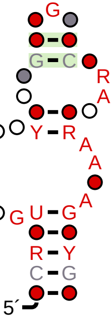

HMP2_4186_1

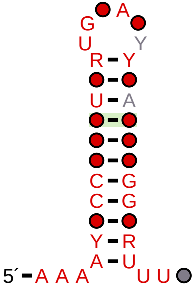

HMP2_4198_1

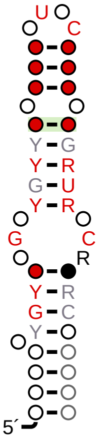

HMP2_42_1

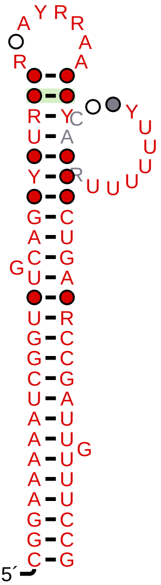

A
Y
R
R
A
R
A
R
Y
C
U
A
Y
U
U
R
U
U
U
Y
R
G
C
A
U
C
G
G
U
A
U
R
G
C
G
C
C
G
U
A
A
U
A
U
G
A
U
A
U
A
U
G
C
G
C
C
G
5´

HMP2_420_1

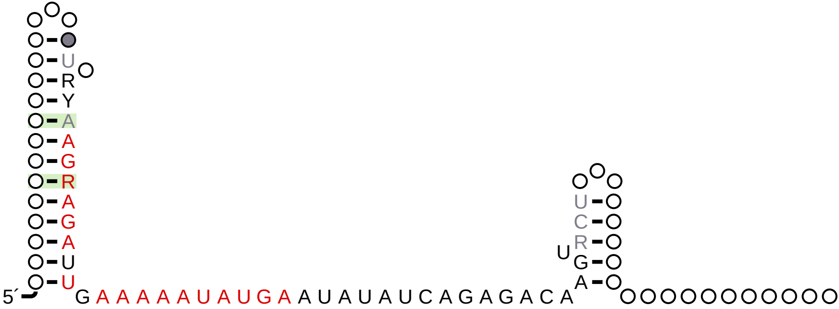

HMP2_4201_1

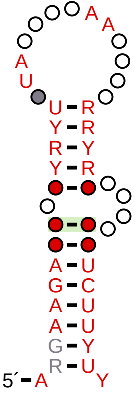

HMP2_4204_1

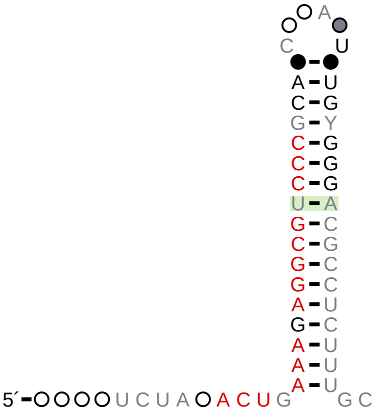

HMP2_4205_1

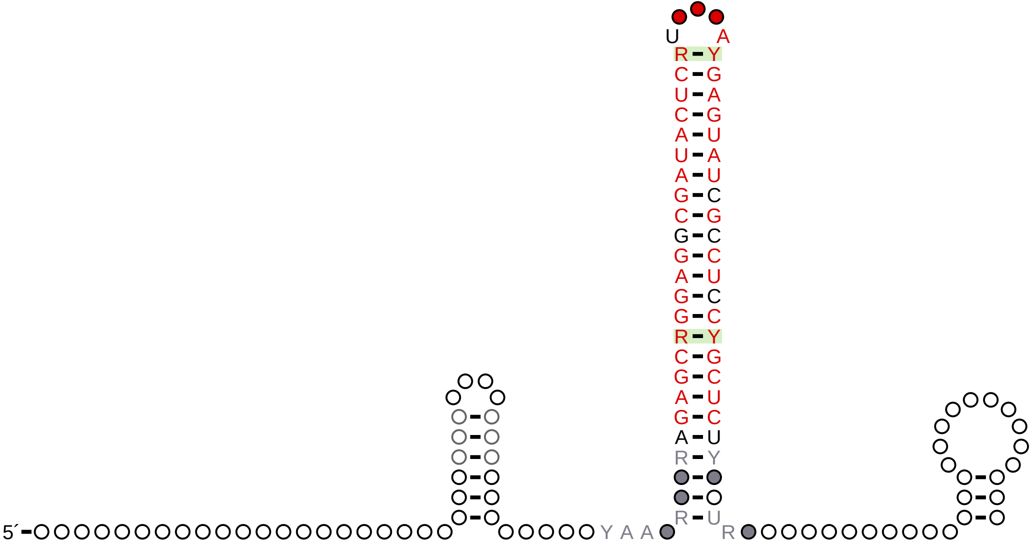
5´
U
A
R Y
C G
U A
C G
A U
U A
A U
G C
C G
G C
G C
A U
G C
G C
R Y
C G
G C
A U
G C
A U
R Y
R U
Y A A
R

HMP2_4209_1

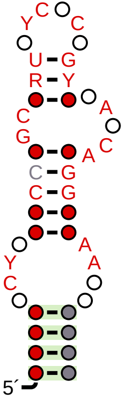

HMP2_421_1

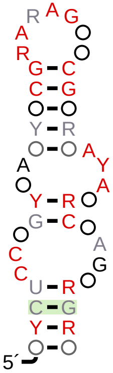

HMP2_4219_1

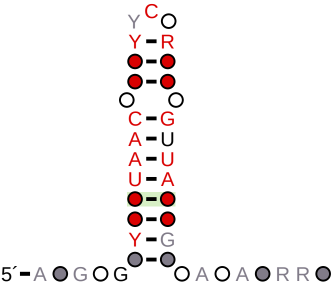

HMP2_4226_1

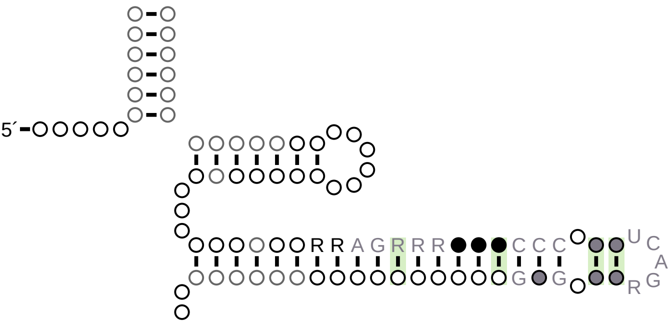

HMP2_4235_1

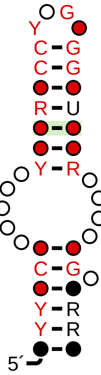

HMP2_4237_1

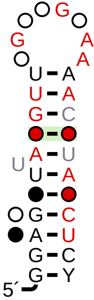

HMP2_4241_1

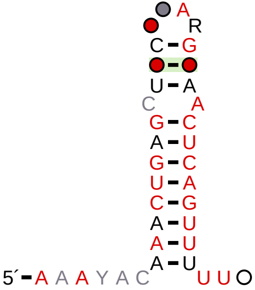

HMP2_4244_1

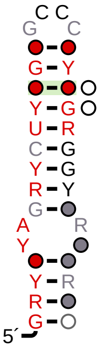

HMP2_4246_1

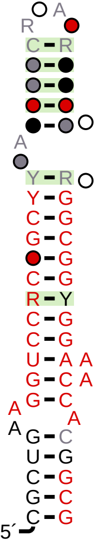

HMP2_4247_1

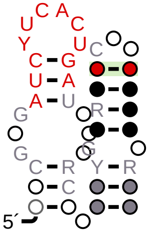

HMP2_4248_1

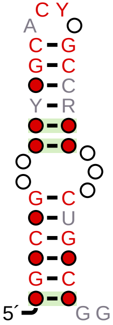

HMP2_4251_1

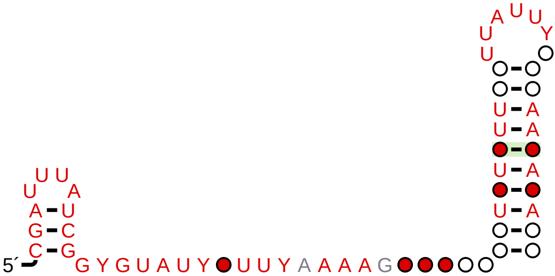

HMP2_426_1

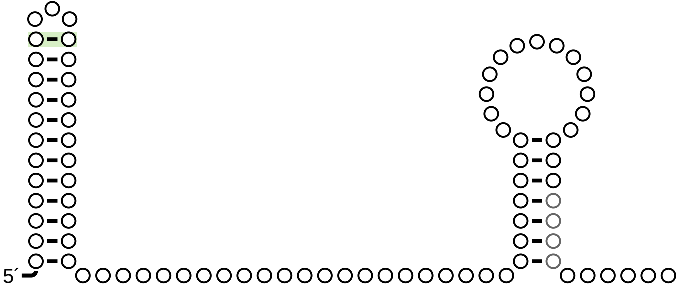

HMP2_4262_1

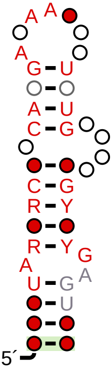

HMP2_4266_1

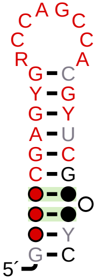

HMP2_4267_1

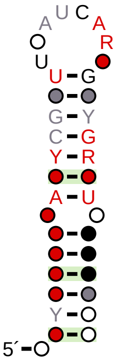

HMP2_4272_1

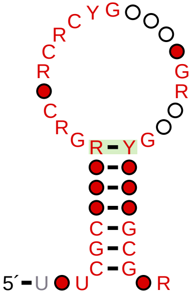

HMP2_4277_1

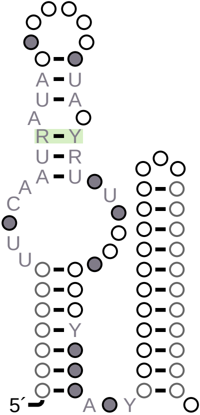

HMP2_4278_1

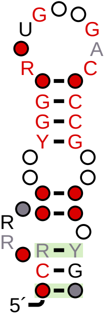

HMP2_4280_1

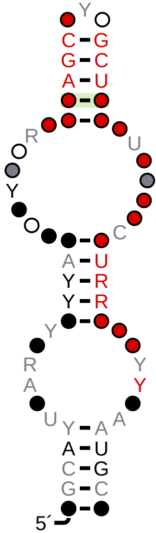

HMP2_4282_1

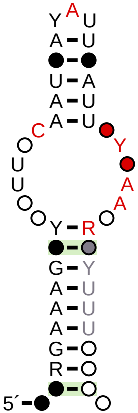

HMP2_4288_1

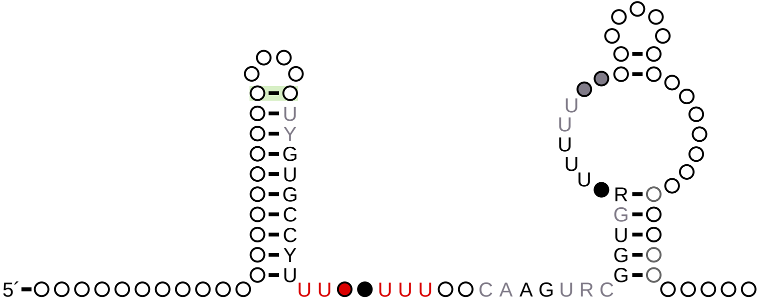

HMP2_429_1

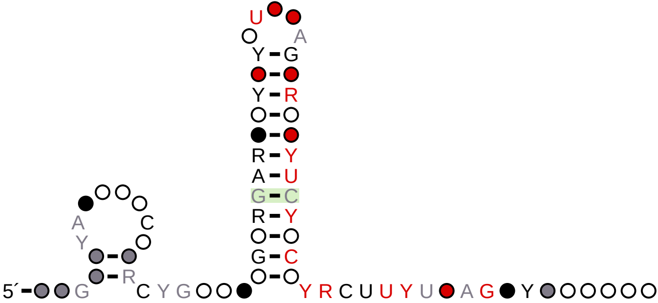

HMP2_4299_1

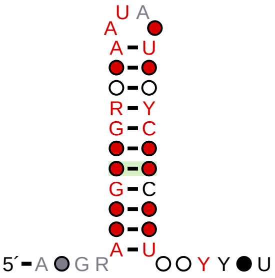

HMP2_4301_1

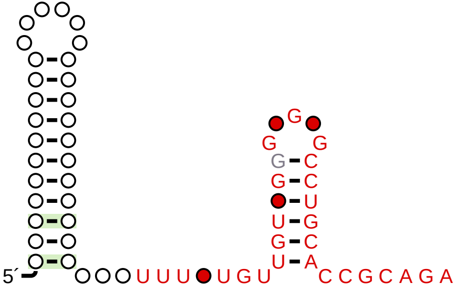

HMP2_4308_1

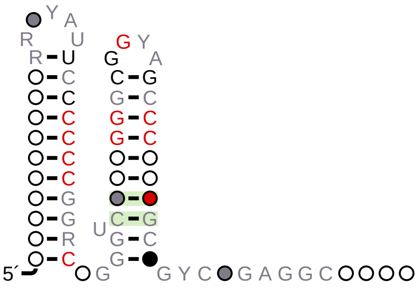

HMP2_4309_1

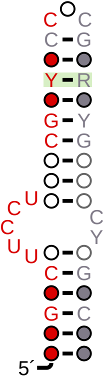

HMP2_4312_1

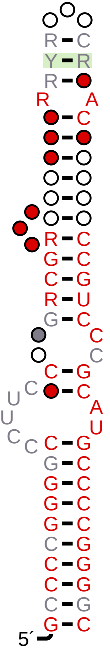

HMP2_4313_1

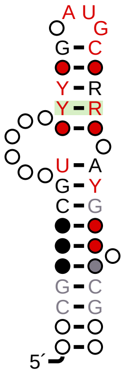

HMP2_4321_1

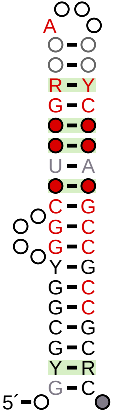

HMP2_4322_1

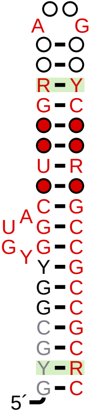

HMP2_4324_1

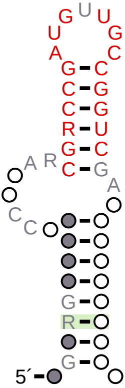

HMP2_4325_1

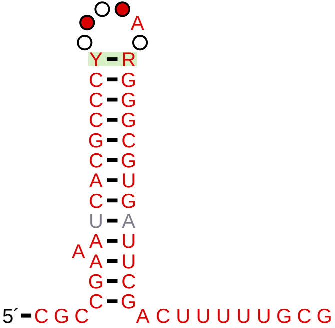

HMP2_4327_1

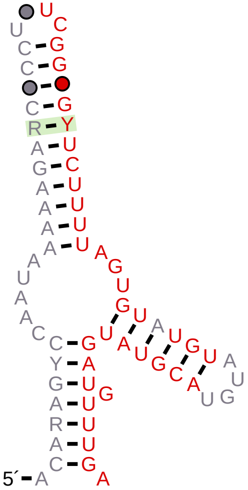

HMP2_4329_1

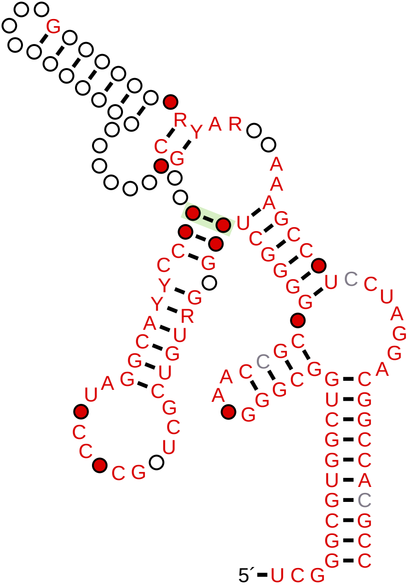

HMP2_4330_1

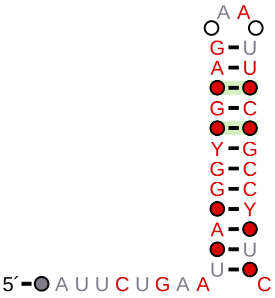

HMP2_4331_1

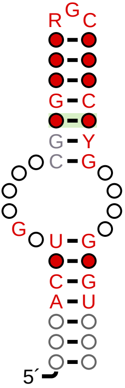

HMP2_4333_1

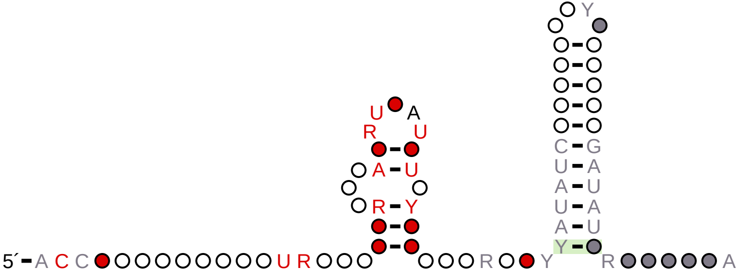

HMP2_4342_1

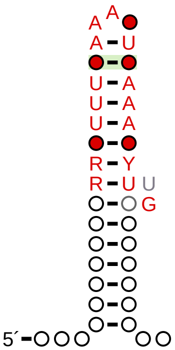

HMP2_4345_1

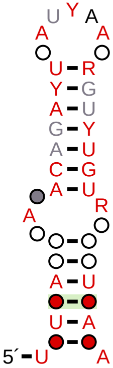

HMP2_4346_1

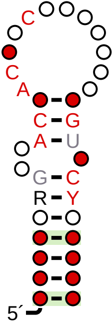

HMP2_4348_1

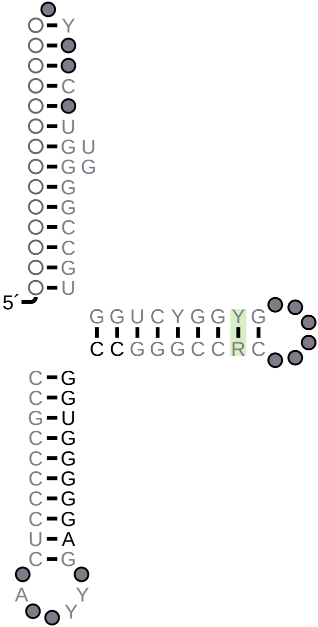

HMP2_435_1

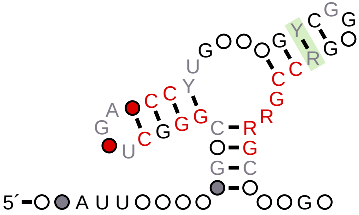

HMP2_4350_1

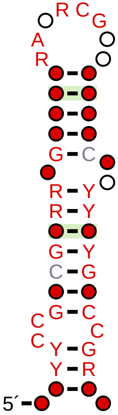

HMP2_4356_1

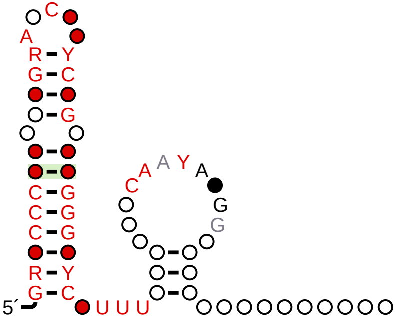

HMP2_4369_1

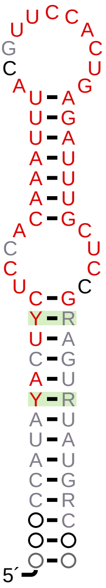

HMP2_4374_1

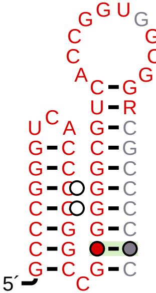

HMP2_438_1

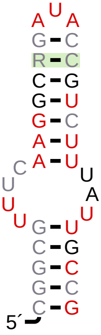

HMP2_4392_1

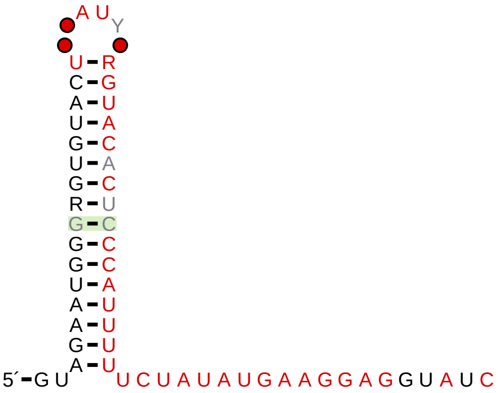

A U
Y
U R
C G
A U
U A
G C
U A
G C
R U
G C
G C
G C
U A
A U
A U
G U
A U
5´ G U
U C U A U A U G A A G G A G G U A U C

HMP2_4397_1

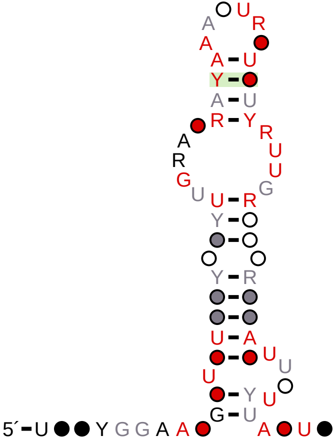

HMP2_4405_1

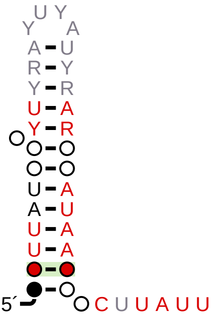

HMP2_4411_1

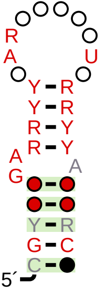

HMP2_4416_1

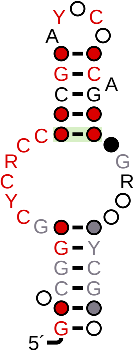

HMP2_4419_1

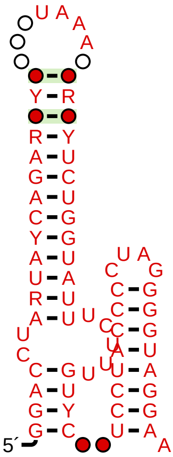

HMP2_4425_1

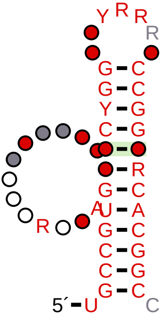

HMP2_4429_1

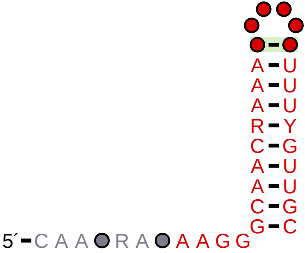

HMP2_443_1

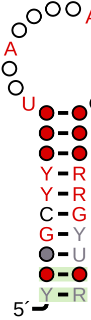

HMP2_4431_1

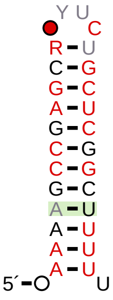

HMP2_4436_1

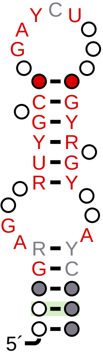

HMP2_4438_1

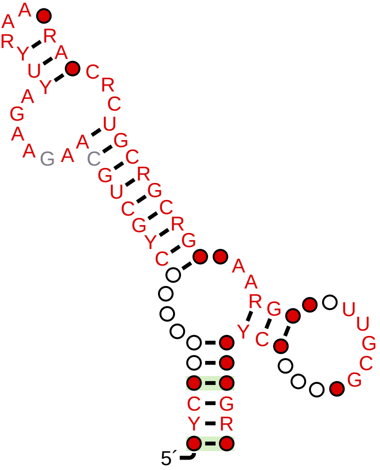

HMP2_4442_1

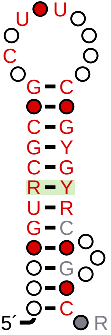

HMP2_4446_1

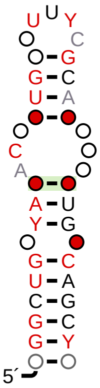

HMP2_445_1

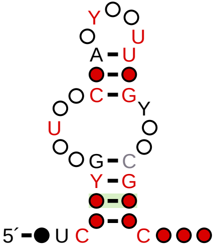

HMP2_4451_1

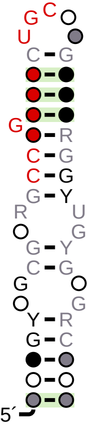

HMP2_4461_1

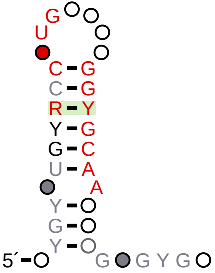

HMP2_4468_1

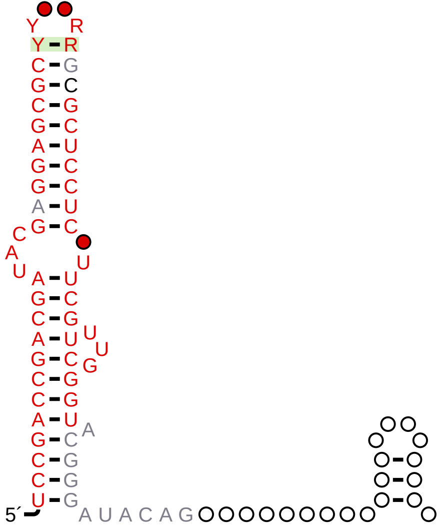
Y R
Y-R
C-G
G-C
C-G
G-C
A-U
G-C
G-C
A-U
G-C
C
A
U
U
A-U
G-C
C-G
A-U
U
U
G-C
G
C-G
C-G
A-U
A
G-C
C-G
C-G
U-G
5´
AUACAG

HMP2_4472_1

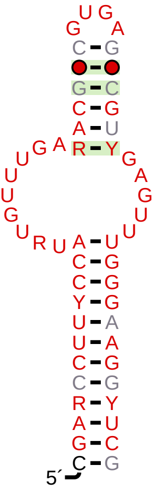

HMP2_4473_1

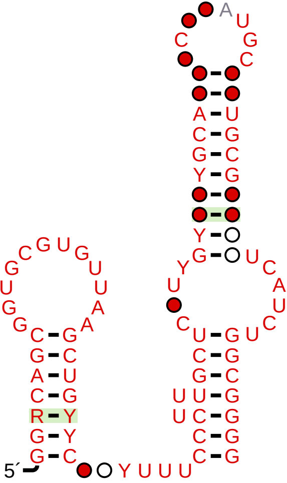

HMP2_4476_1

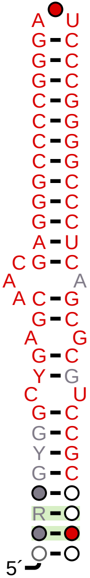

HMP2_4481_1

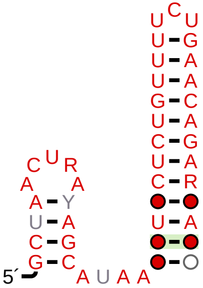

HMP2_4482_1

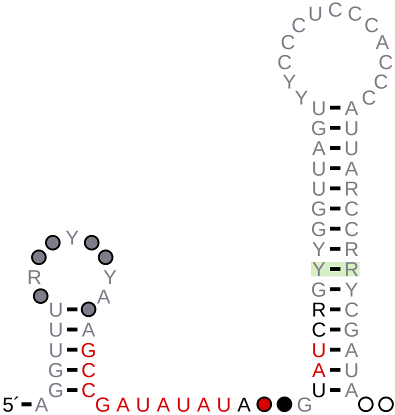

HMP2_4486_1

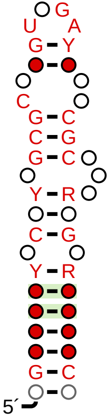

HMP2_4493_1

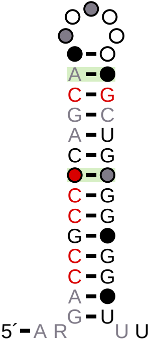

HMP2_4495_1

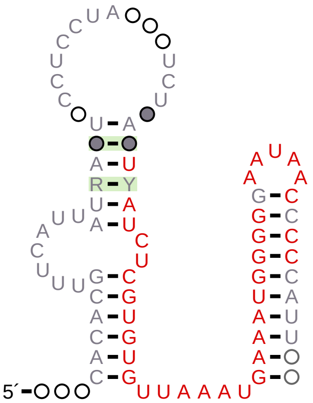

HMP2_4496_1

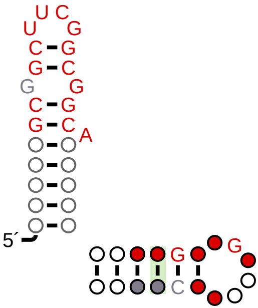

HMP2_450_1

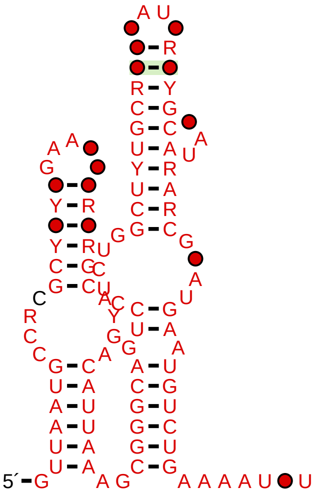

HMP2_4505_1

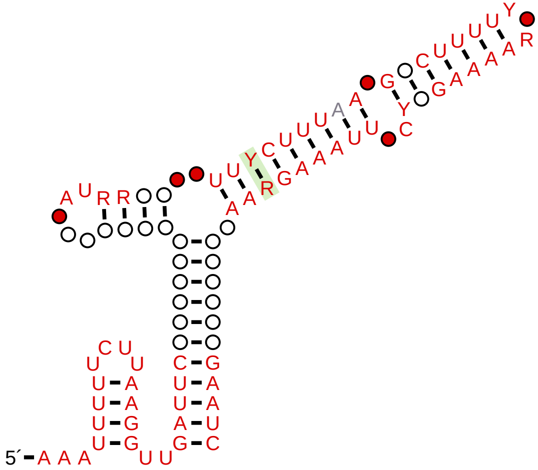

HMP2_4507_1

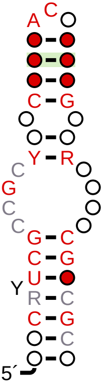

HMP2_451_1

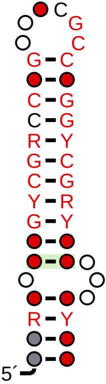

HMP2_4512_1

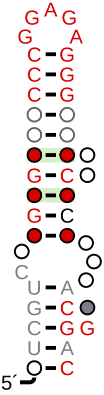

HMP2_4521_1

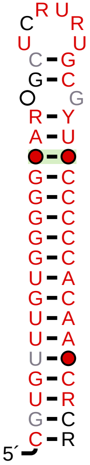

HMP2_4523_1

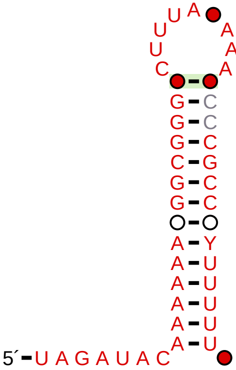

HMP2_4524_1

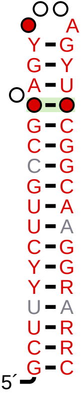

HMP2_4527_1

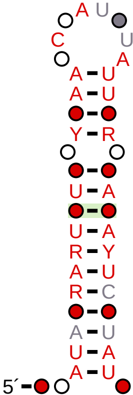

HMP2_4534_1

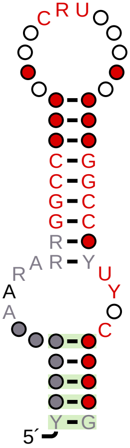

HMP2_454_1

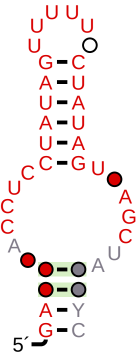

HMP2_4543_1

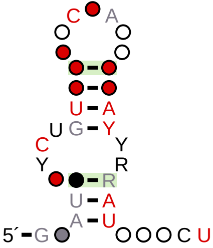

HMP2_4547_1

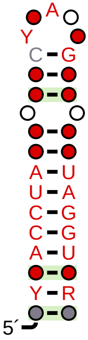

HMP2_4552_1

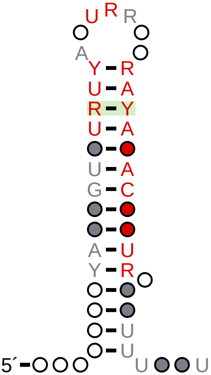

HMP2_4555_1

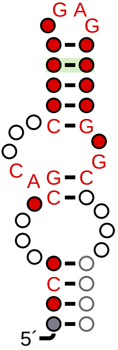

HMP2_4560_1

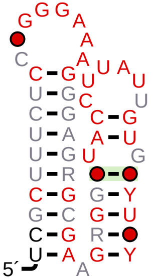

HMP2_4561_1

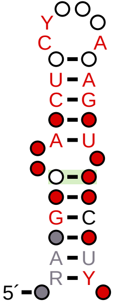

HMP2_4572_1

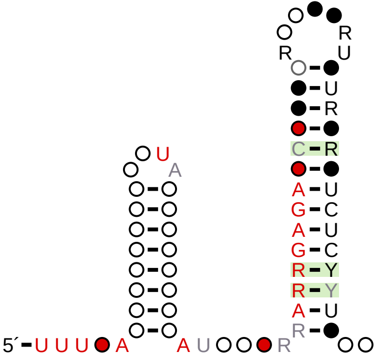

HMP2_458_1

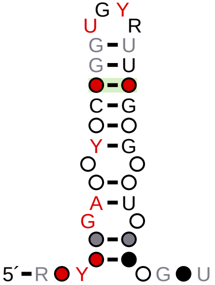

HMP2_4583_1

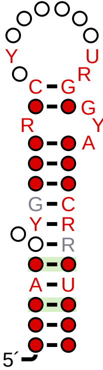

HMP2_4585_1

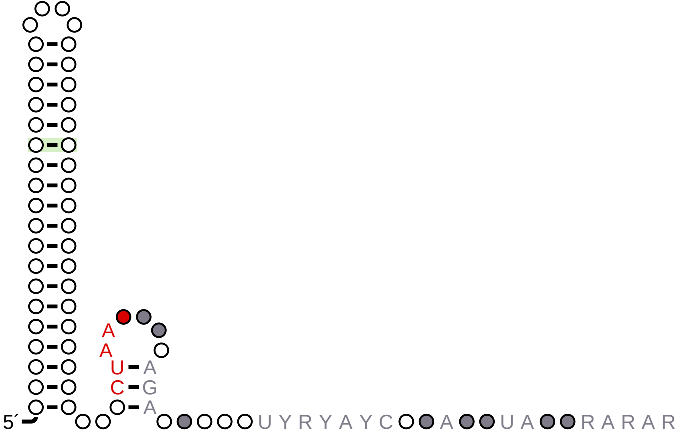

HMP2_4594_1

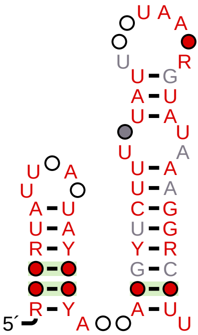

HMP2_460_1

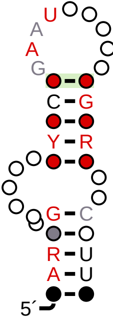

HMP2_4601_1

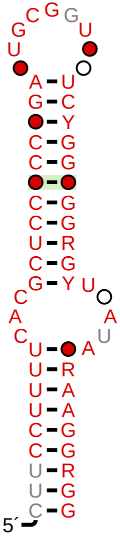

HMP2_4602_1

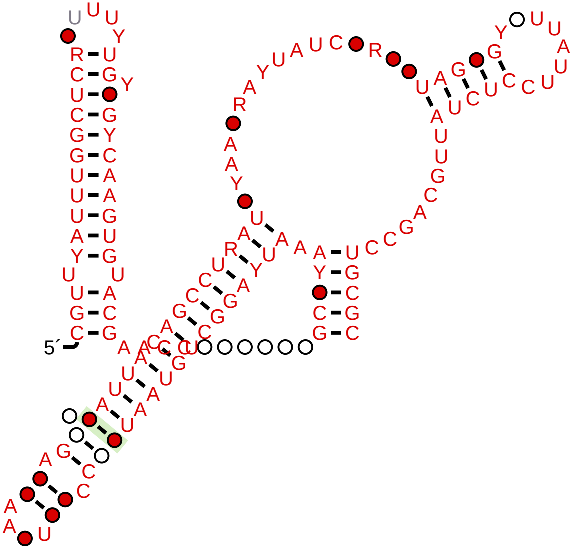

HMP2_4604_1

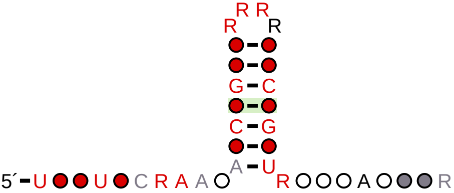

HMP2_4606_1

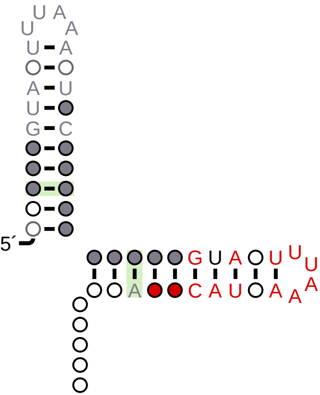

HMP2_461_1

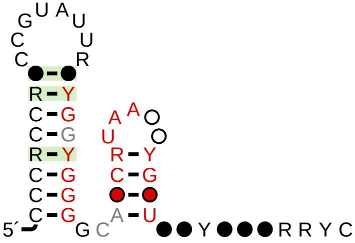

HMP2_4610_1

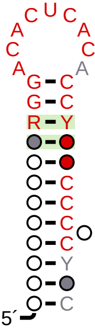

HMP2_4612_1

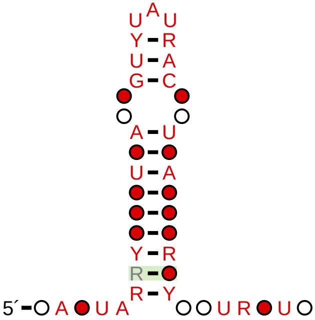

HMP2_4613_1

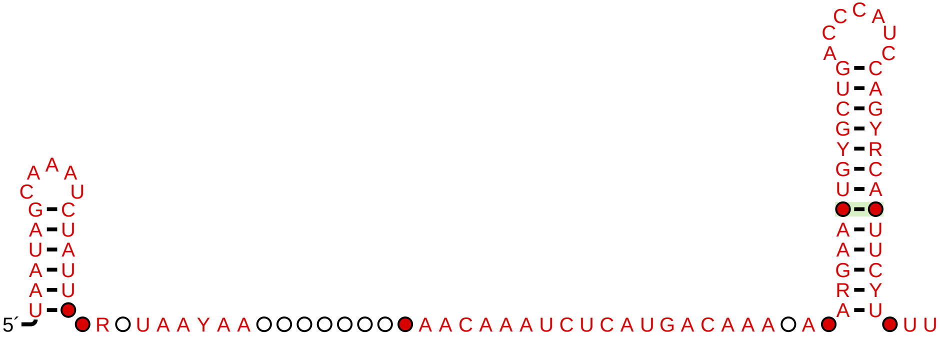

HMP2_4616_1

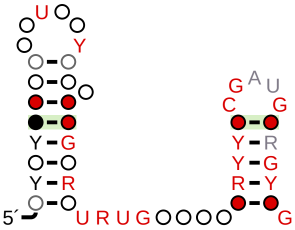

HMP2_4619_1

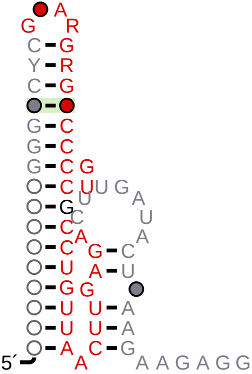

HMP2_4624_1

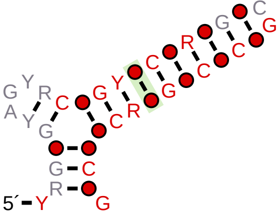

HMP2_4627_1

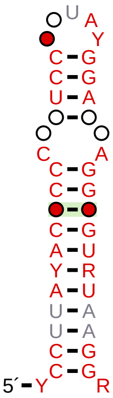

HMP2_4642_1

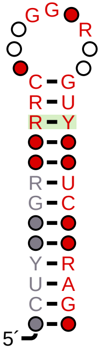

HMP2_4646_1

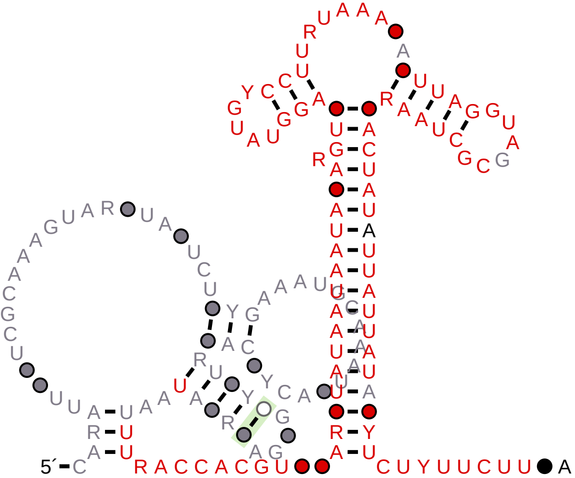

HMP2_4648_1

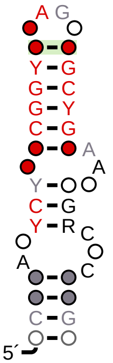

HMP2_465_1

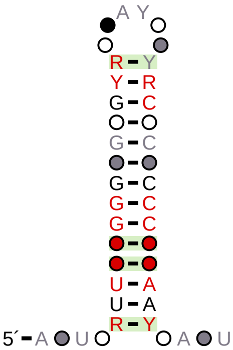

HMP2_4658_1

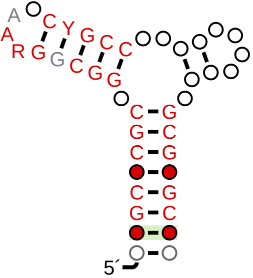

HMP2_4677_1

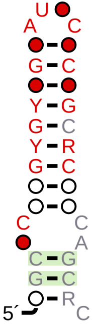

HMP2_4679_1

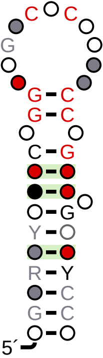

HMP2_4681_1

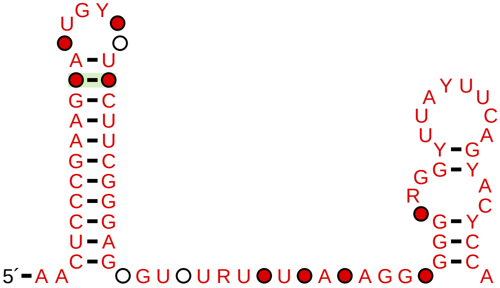

HMP2_4683_1

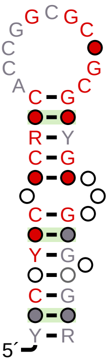

HMP2_4690_1

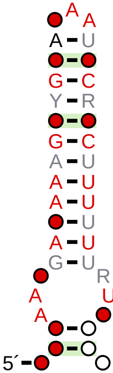

HMP2_4692_1

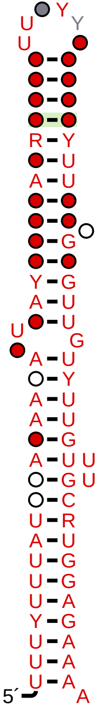

HMP2_4694_1

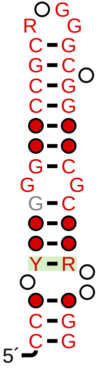

HMP2_47_1

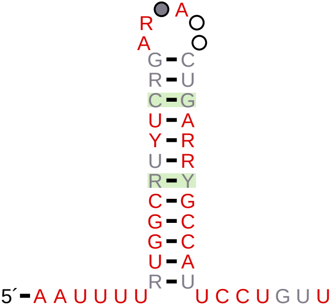

HMP2_4700_1

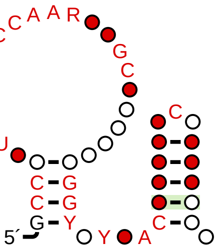

HMP2_4708_1

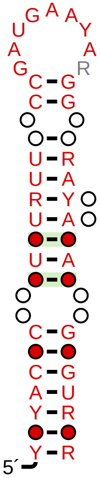

HMP2_4709_1

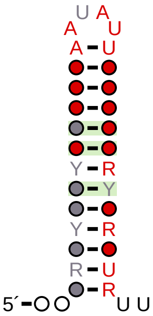

HMP2_471_1

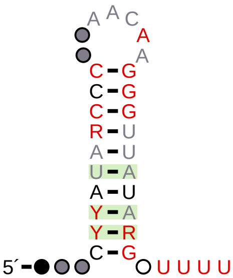

HMP2_4712_1

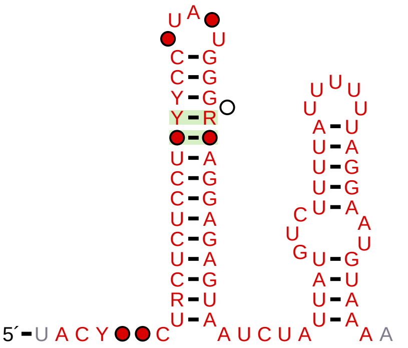

HMP2_4714_1

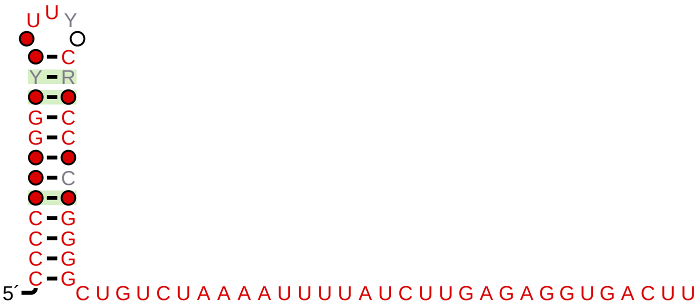
U U Y
C
Y R
G C
G C
C
C G
C G
C G
C G
5´
C U G U C U A A A A U U U U A U C U U G A G A G G U G A C U U

HMP2_4715_1

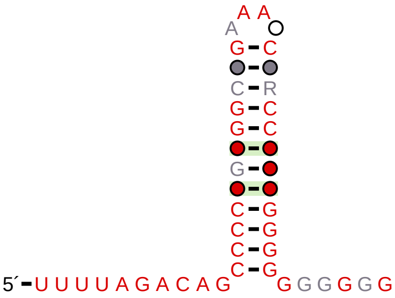

HMP2_4720_1

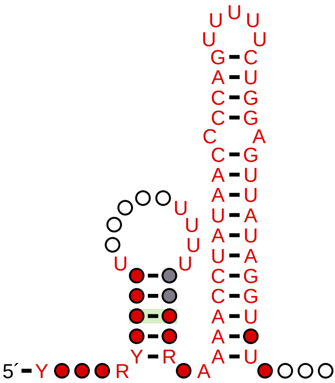

HMP2_4733_1

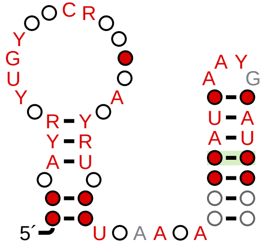

HMP2_474_1

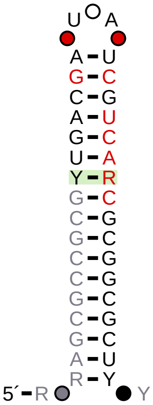

HMP2_4750_1

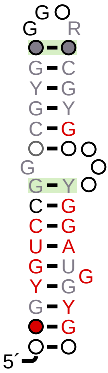

HMP2_4756_1

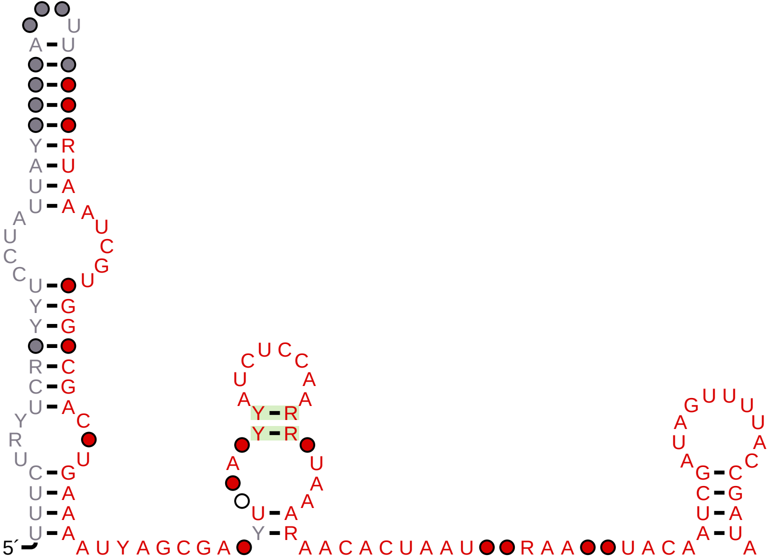

HMP2_4765_1

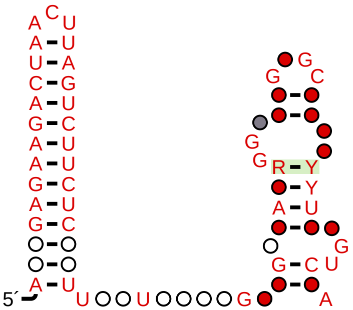

HMP2_4770_1

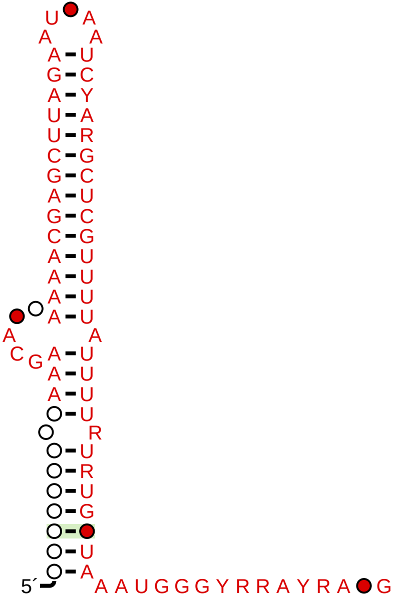

HMP2_4781_1

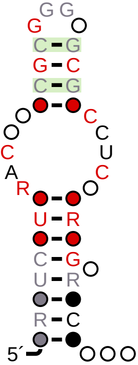

HMP2_4791_1

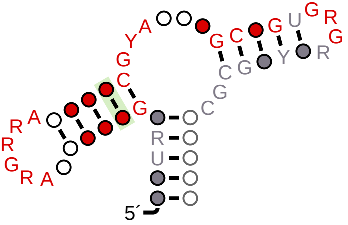

HMP2_4795_1

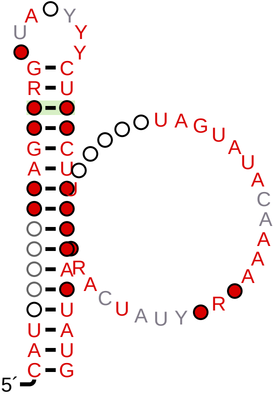

HMP2_4798_1

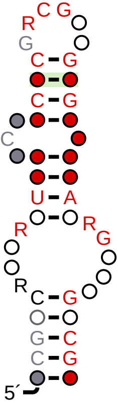

HMP2_4799_1

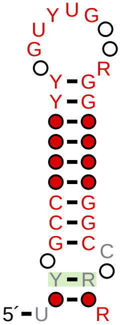

HMP2_4803_1

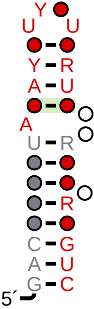

HMP2_4805_1

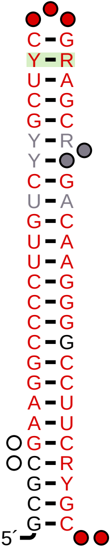
C-G
Y-R
U-A
C-G
G-C
Y R
Y-
C-G
U-A
G-C
U-A
U-A
C-G
C-G
C-G
C-G
G-C
G-C
A-U
A-U
G-C
C-R
G-Y
C-G
G-C
5´

HMP2_4809_1

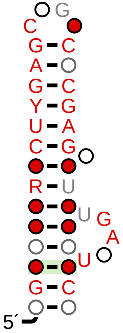

HMP2_4825_1

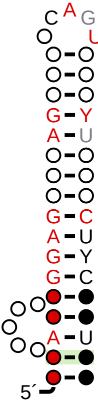

HMP2_4834_1

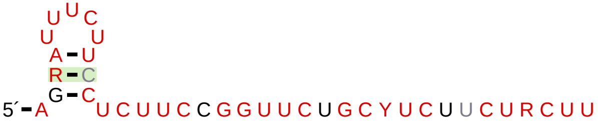

HMP2_4837_1

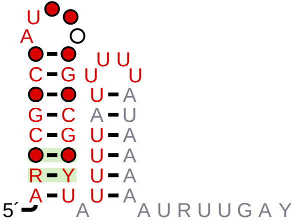

HMP2_4838_1

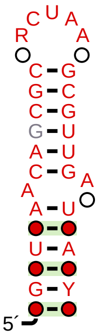

HMP2_4840_1

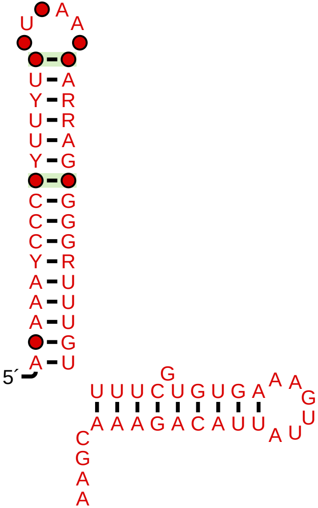

HMP2_4842_1

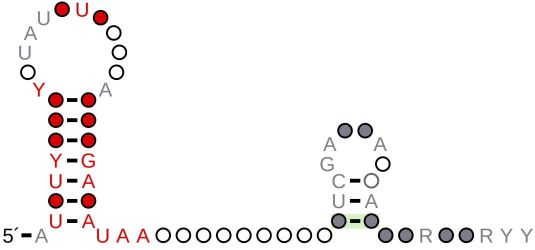

HMP2_4843_1

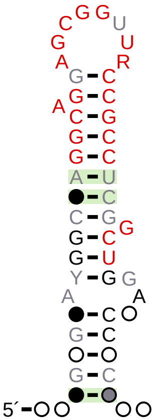

HMP2_4846_1

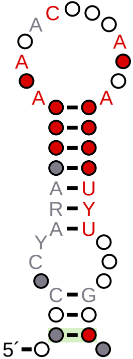

HMP2_4847_1

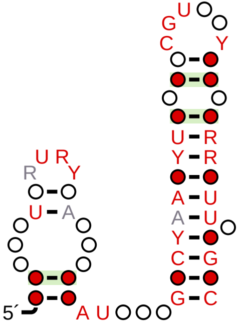

HMP2_4850_1

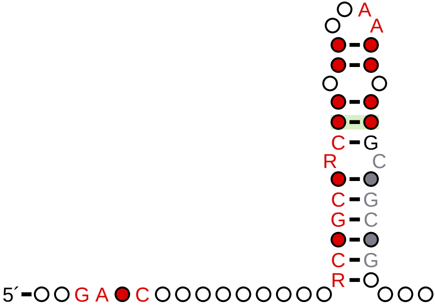

HMP2_4865_1

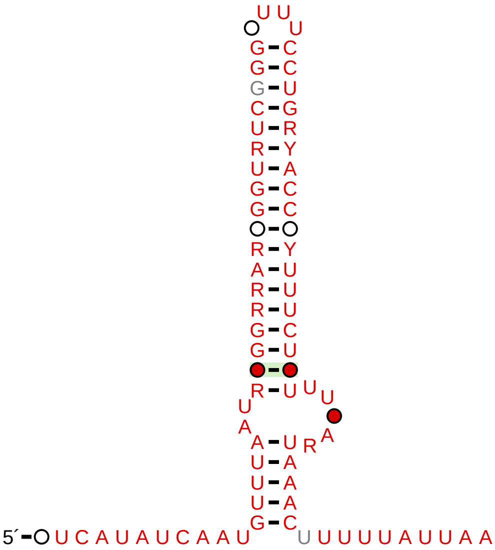

HMP2_4875_1

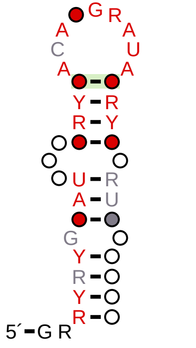

HMP2_4886_1

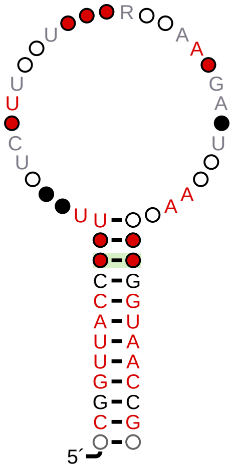

HMP2_4890_1

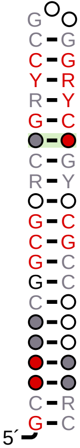

HMP2_4895_1

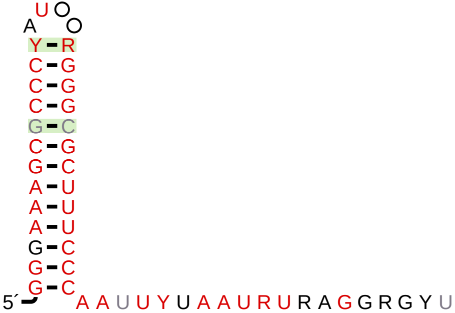

HMP2_4898_1

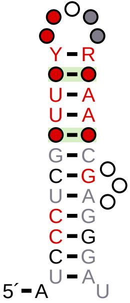

HMP2_4899_1

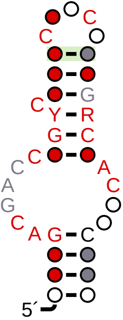

HMP2_49_1

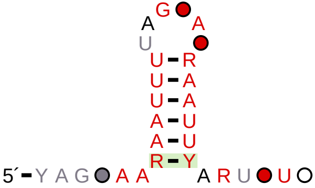

HMP2_491_1

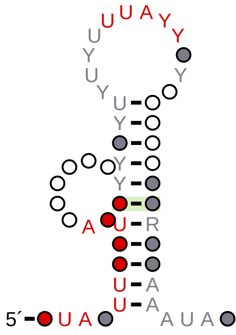

HMP2_4917_1

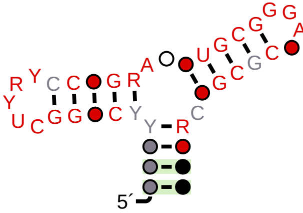

HMP2_492_1

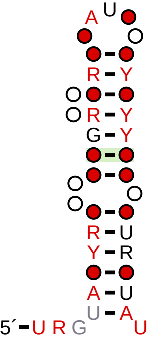

HMP2_4921_1

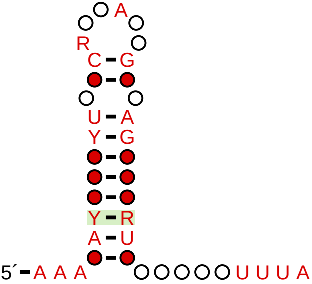

HMP2_4928_1

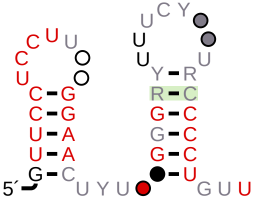

HMP2_493_1

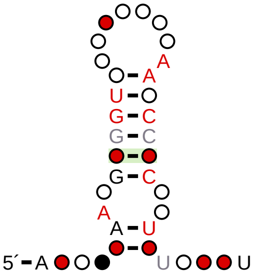

HMP2_4930_1

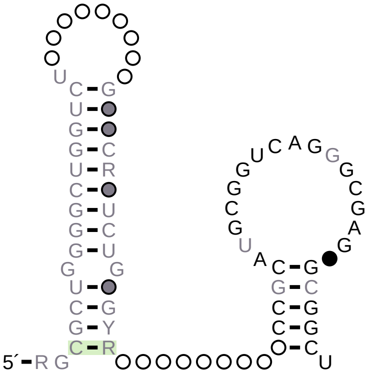

5´
R G
U
C G
U
G
G C
U R
C
G U
G C
G U
G G
U
C G
G Y
C R
G U C A G G
G G
G C
C G
G A
U G
A C G
G C
C G
C G
C
U

HMP2_4945_1

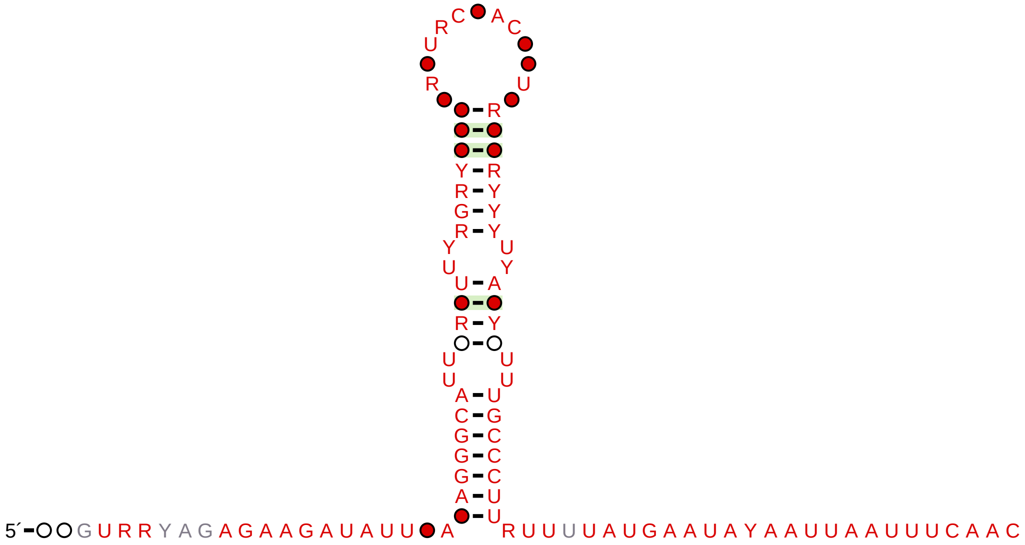

HMP2_4946_1

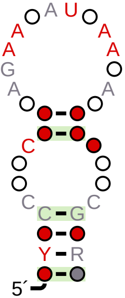

HMP2_495_1

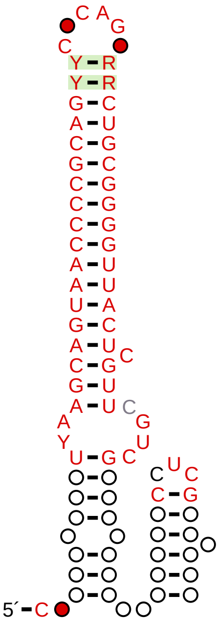

HMP2_4962_1

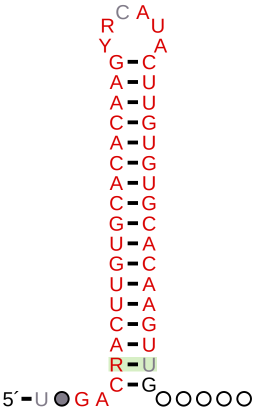

HMP2_4972_1

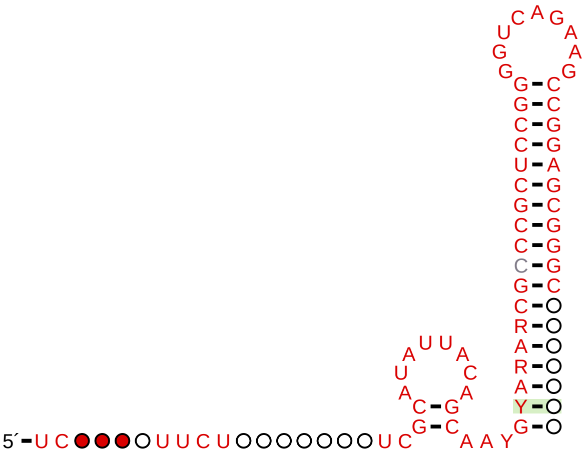
5´ U C U U C U U C G C A A Y G Y A R A R C G C C G C U C C G G G G U C A G A A G C C G G A G G C G G C

HMP2_4974_1

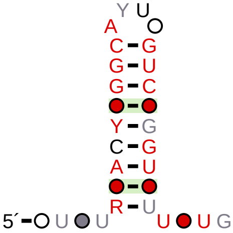

HMP2_4975_1

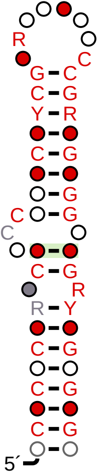

HMP2_4977_1

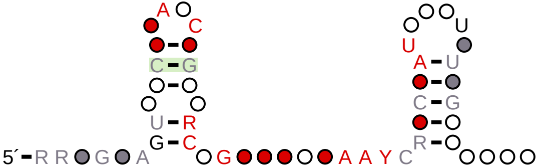

HMP2_4979_1

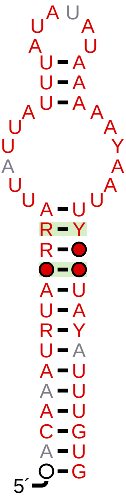

HMP2_4980_1

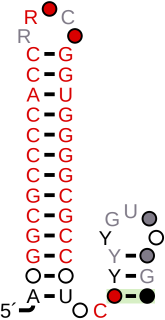

HMP2_4981_1

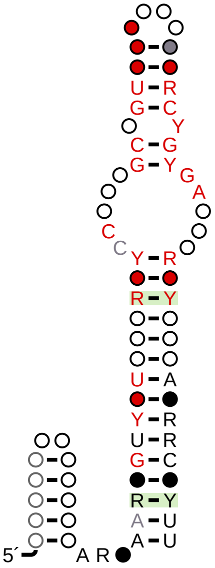

HMP2_4984_1

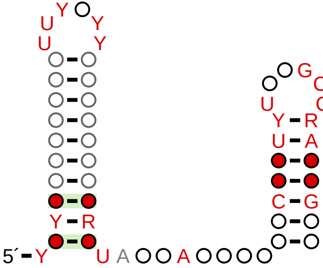

HMP2_499_1

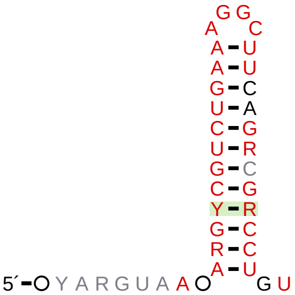

HMP2_4991_1

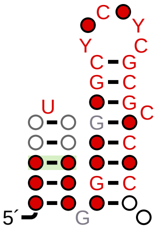

HMP2_4998_1

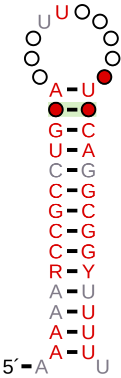

HMP2_50_1

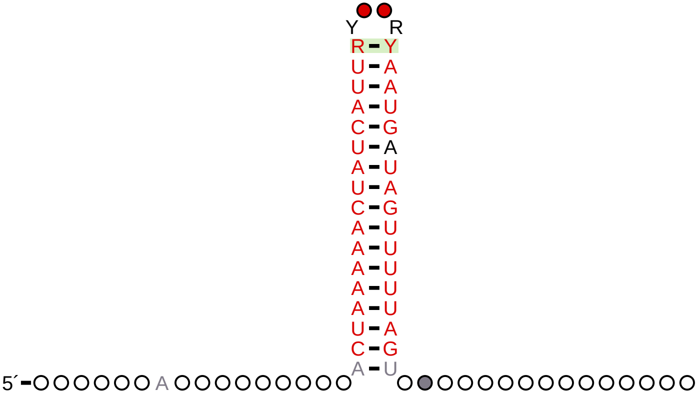

HMP2_500_1

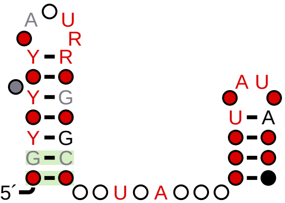

HMP2_501_1

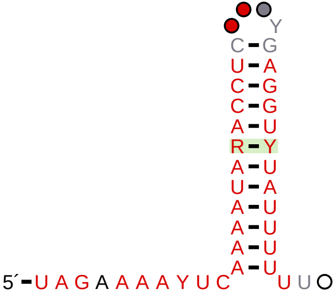

HMP2_5018_1

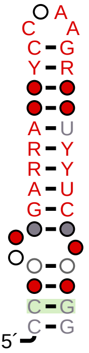

HMP2_5019_1

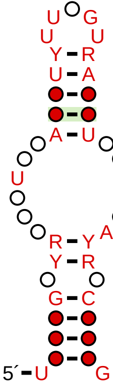

HMP2_5028_1

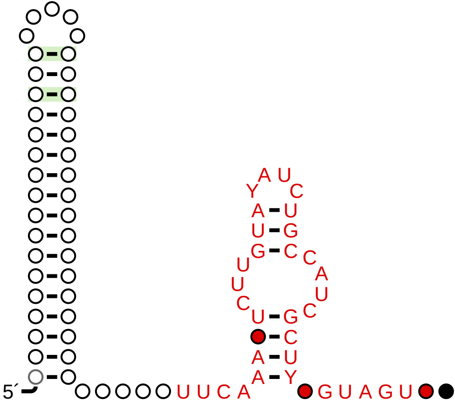

HMP2_5035_1

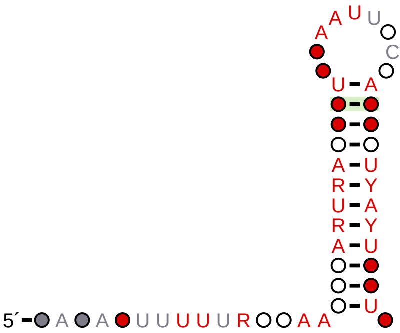

HMP2_5042_1

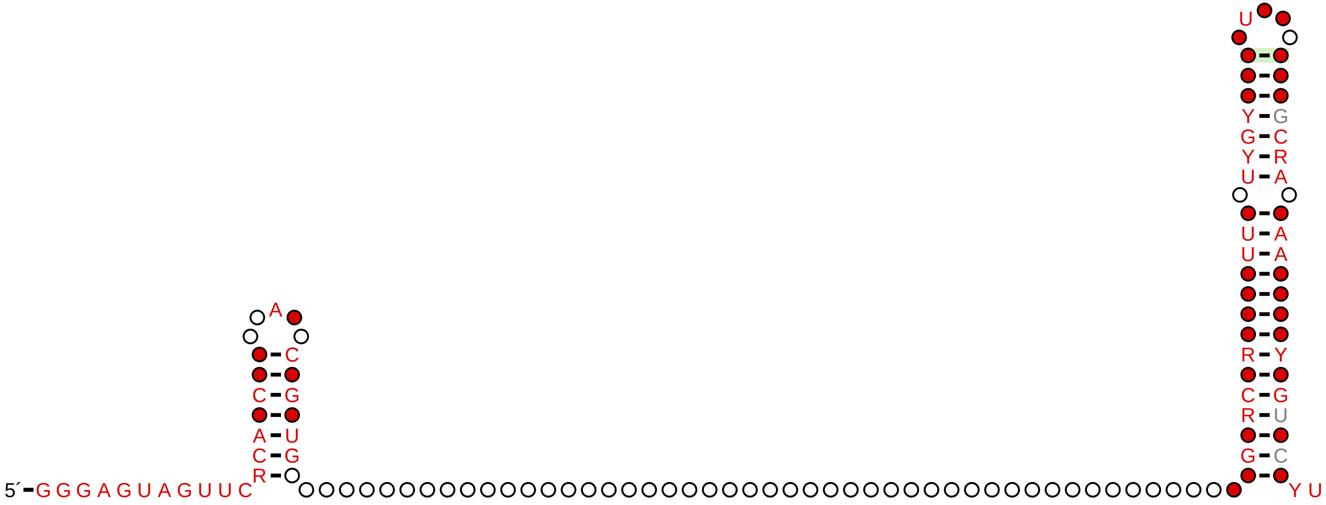

HMP2_5043_1

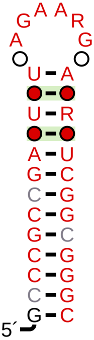

HMP2_505_1

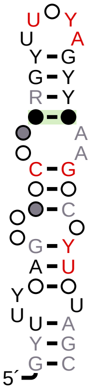

HMP2_5054_1

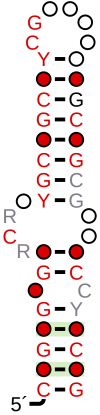

HMP2_5057_1

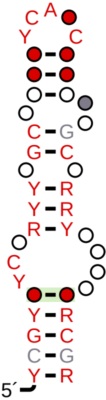

HMP2_506_1

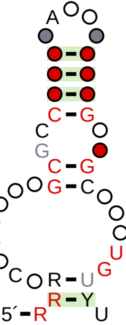

HMP2_5062_1

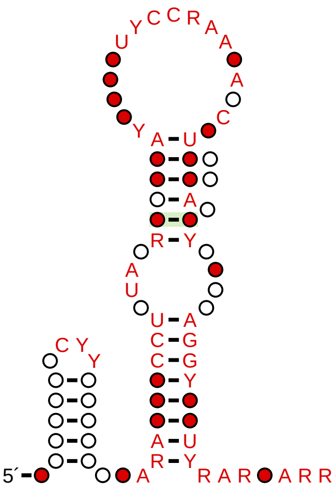

HMP2_5066_1

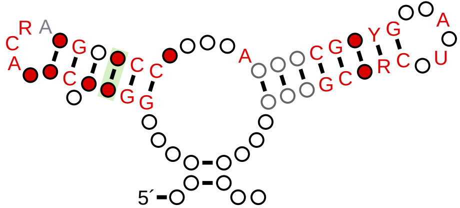

HMP2_5067_1

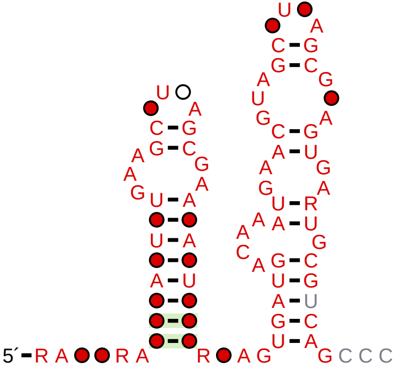

HMP2_5069_1

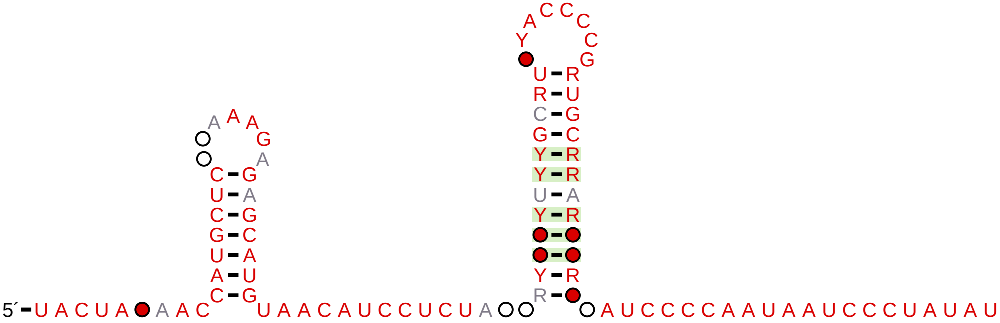

HMP2_5070_1

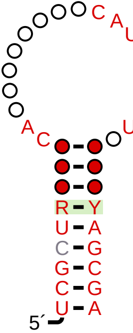

HMP2_5074_1

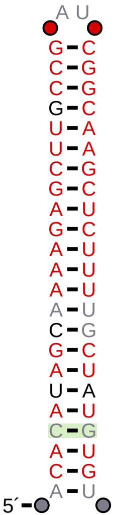

HMP2_5084_1

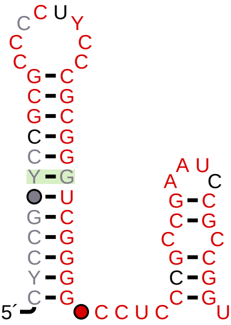

HMP2_5090_1

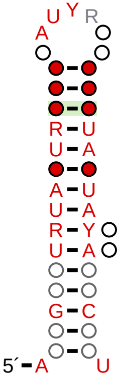

HMP2_5093_1

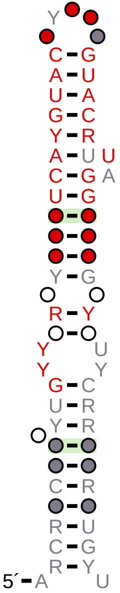

HMP2_5097_1

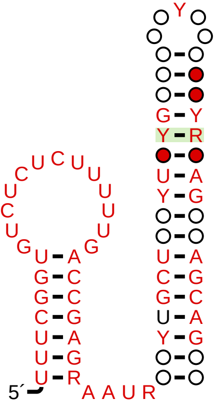

HMP2_5111_1

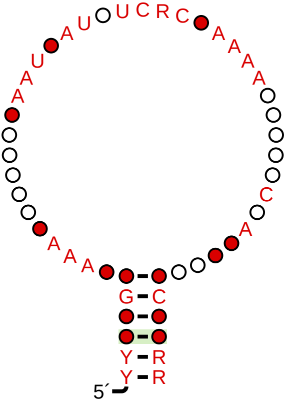

HMP2_5116_1

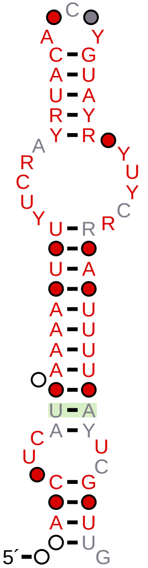

HMP2_5120_1

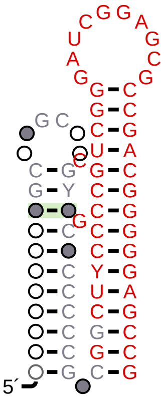

HMP2_5127_1

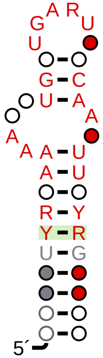

HMP2_5129_1

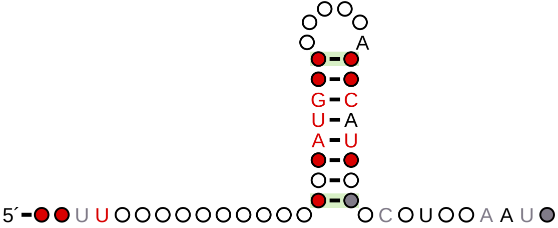

HMP2_5132_1

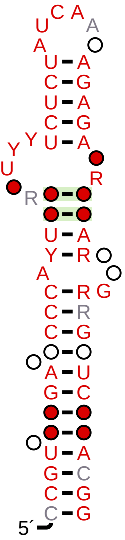

HMP2_5150_1

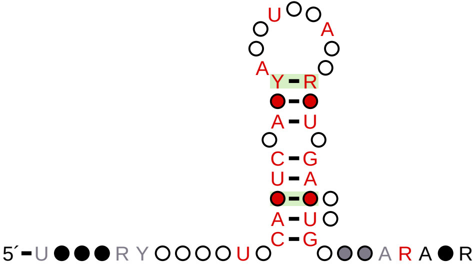

HMP2_5151_1

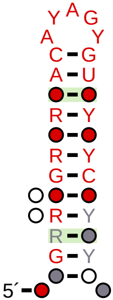

HMP2_5153_1

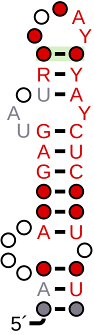

HMP2_5154_1

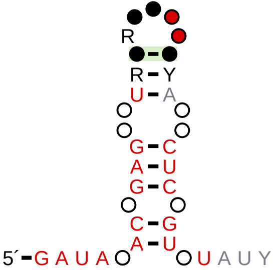

HMP2_5158_1

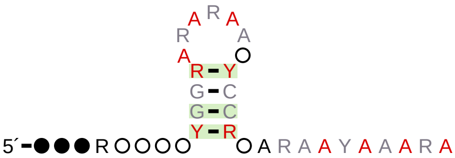

HMP2_5165_1

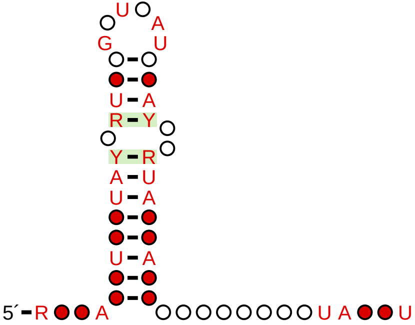

HMP2_5167_1

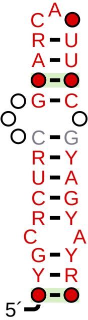

HMP2_5168_1

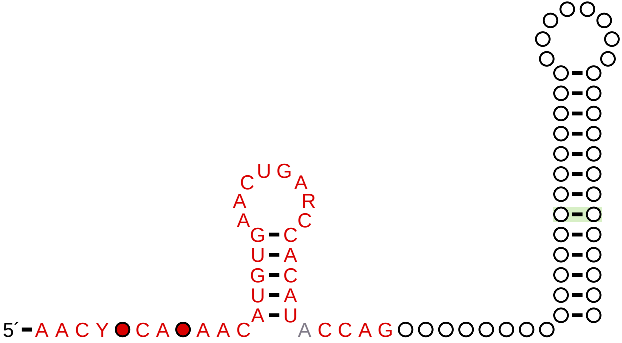

HMP2_5176_1

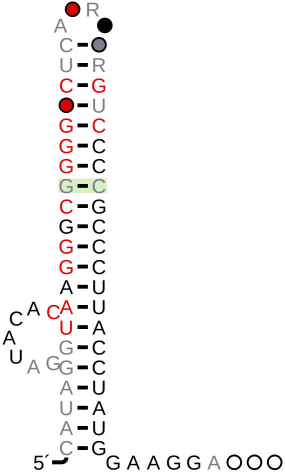

R
A
C
U R
C G
U
G C
G C
G C
G C
C G
G C
G C
G C
A U
C A C A U
A U A
U G C
A G G C
A U
U A
A U
C G
5´ G A A G G A

HMP2_5177_1

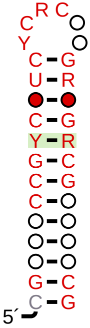

HMP2_5180_1

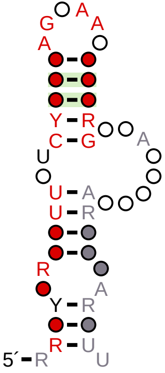

HMP2_5187_1

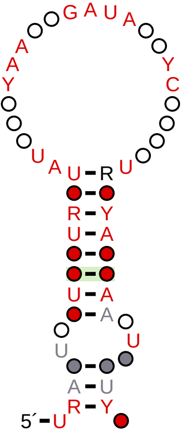

HMP2_5189_1

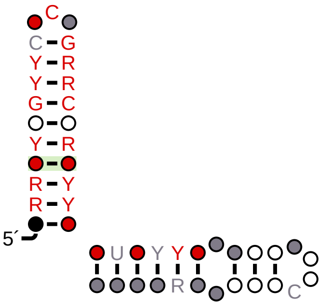

HMP2_5198_1

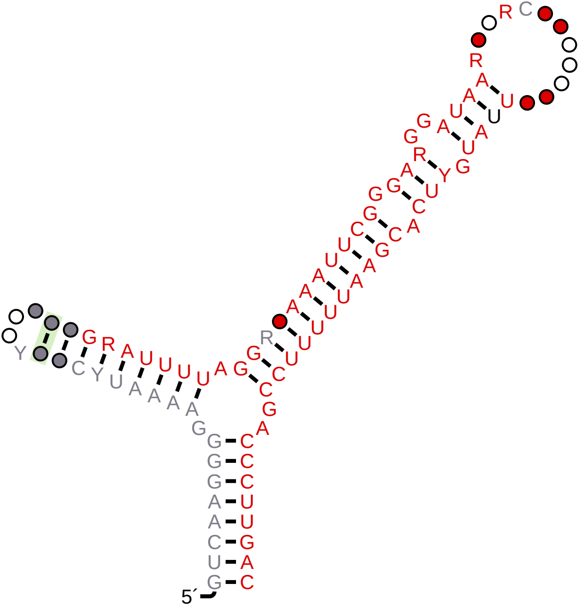

HMP2_5204_1

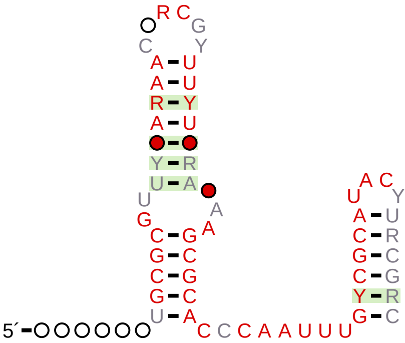

HMP2_5208_1

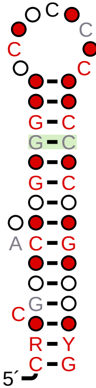

HMP2_5221_1

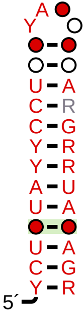

HMP2_5222_1

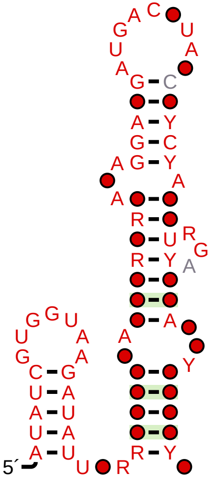

HMP2_5223_1

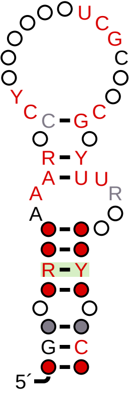

HMP2_5226_1

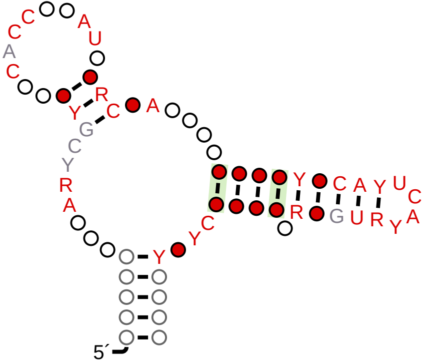

HMP2_5233_1

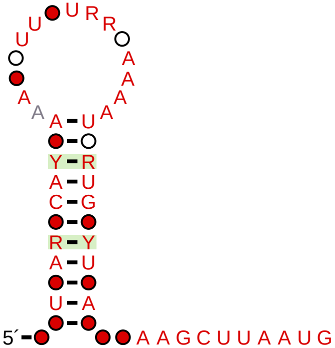

HMP2_5238_1

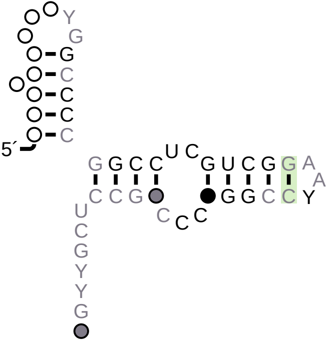

HMP2_524_1

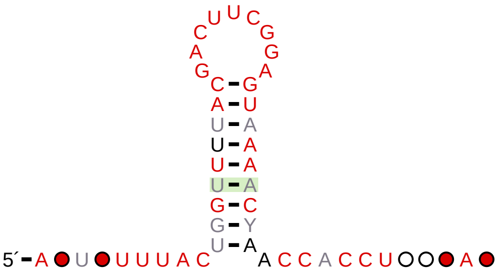

HMP2_5245_1

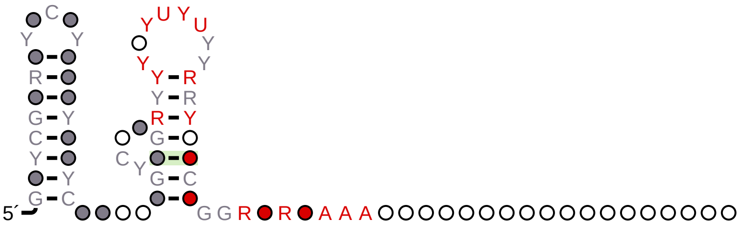

HMP2_5253_1

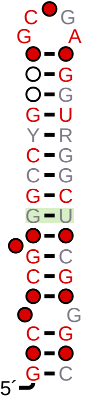

HMP2_5265_1

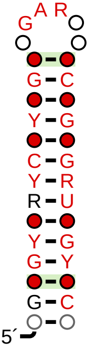

HMP2_5268_1

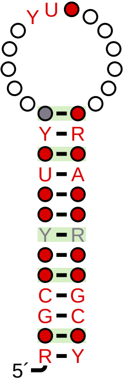

HMP2_5270_1

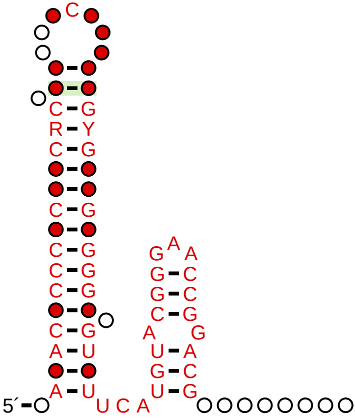

HMP2_5272_1

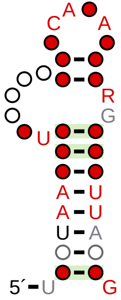

HMP2_5274_1

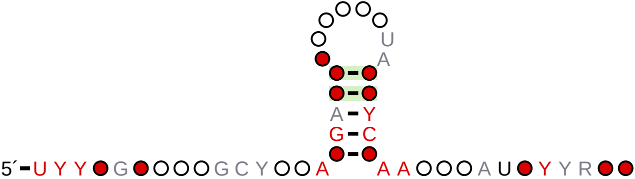

HMP2_5275_1

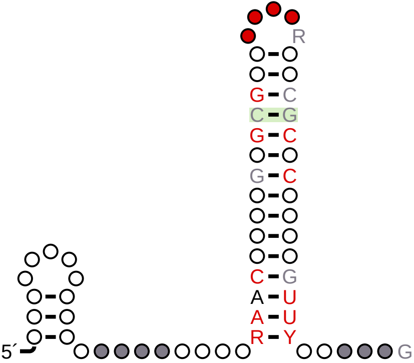

HMP2_5284_1

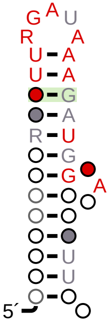

HMP2_529_1

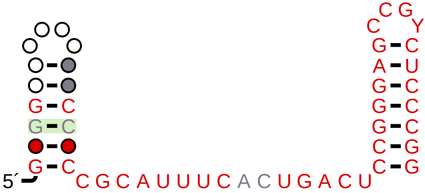

HMP2_5291_1

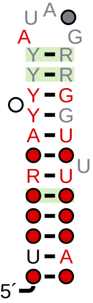

HMP2_5299_1

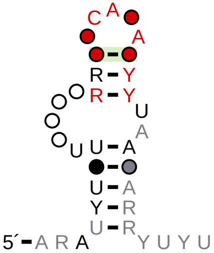

HMP2_53_1

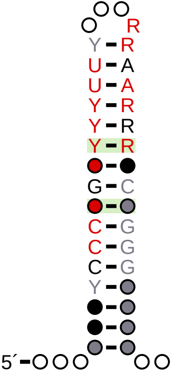

HMP2_530_1

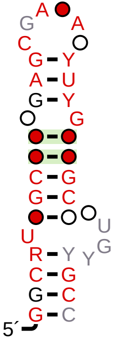

HMP2_5301_1

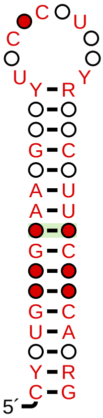

HMP2_5304_1

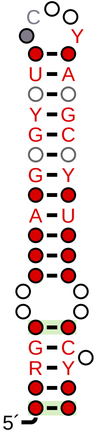

HMP2_5309_1

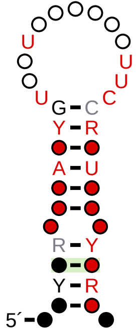

HMP2_5332_1

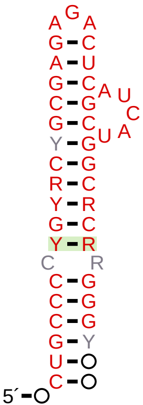

HMP2_5333_1

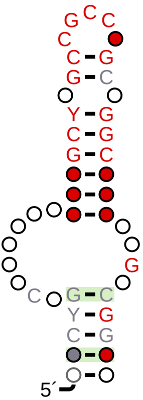

HMP2_5336_1

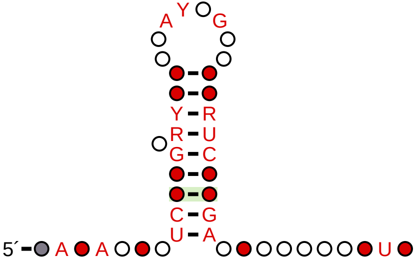

HMP2_5340_1

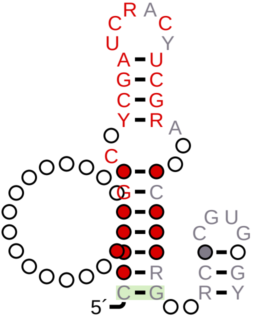

HMP2_5343_1

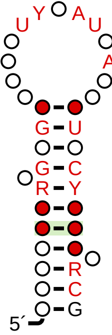

HMP2_5346_1

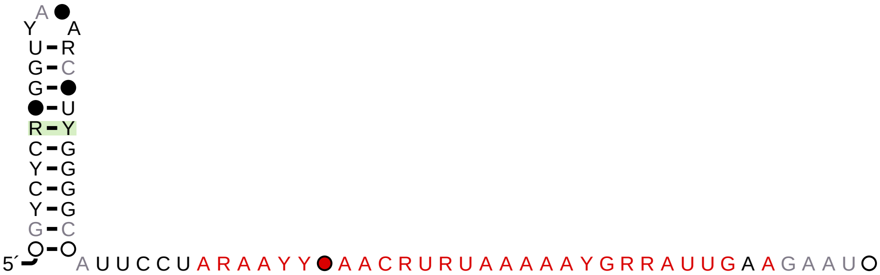

HMP2_5355_1

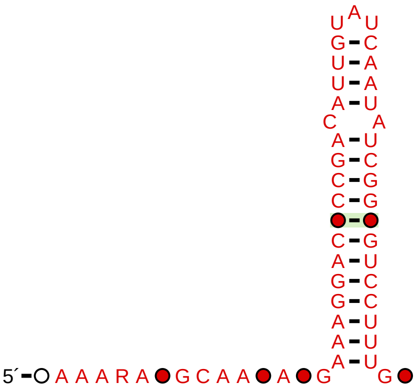

HMP2_5357_1

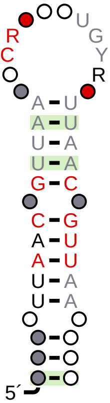

HMP2_5358_1

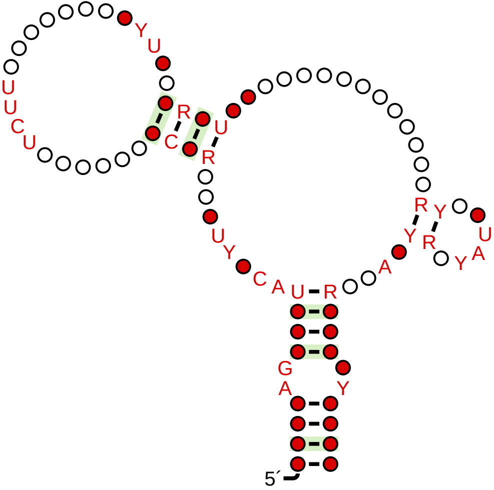

HMP2_5363_1

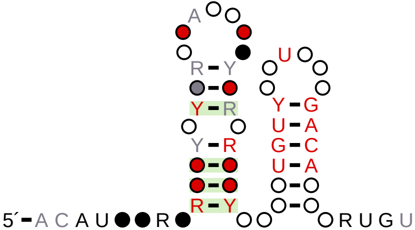

HMP2_5367_1

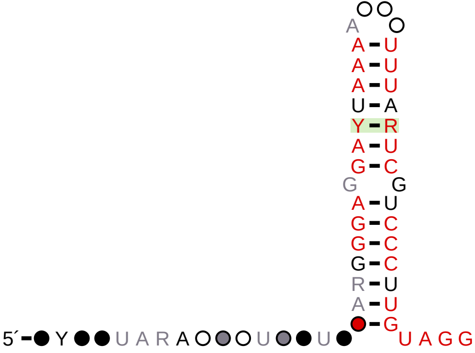

HMP2_5368_1

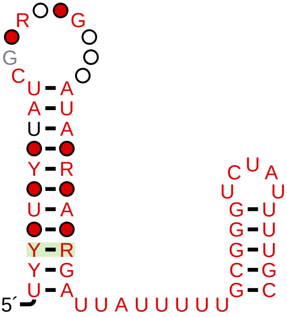

HMP2_5369_1

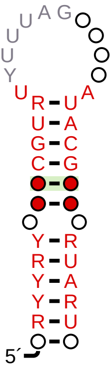

HMP2_5381_1

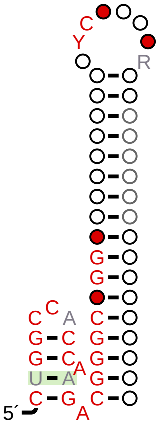

HMP2_5384_1

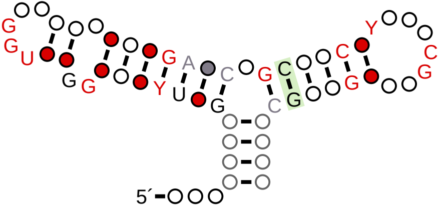

HMP2_5386_1

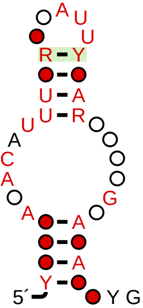

HMP2_5390_1

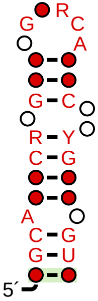

HMP2_5392_1

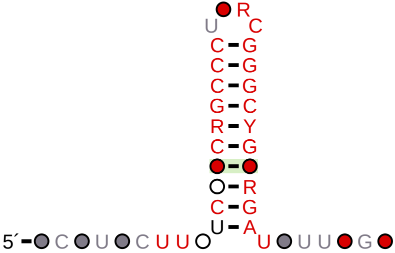

HMP2_5397_1

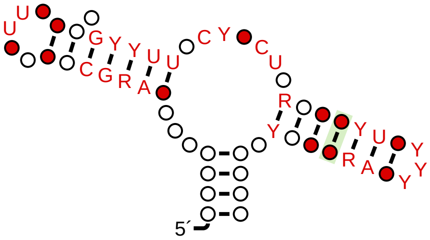

HMP2_540_1

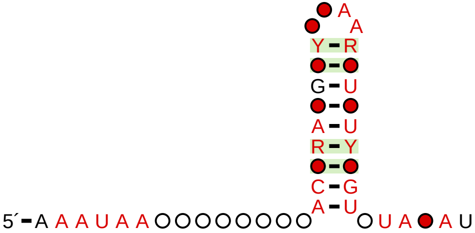

HMP2_5405_1

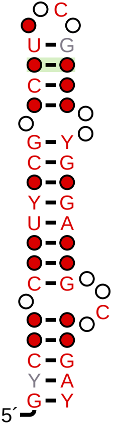

HMP2_5409_1

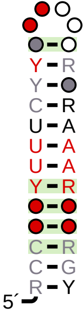

HMP2_5411_1

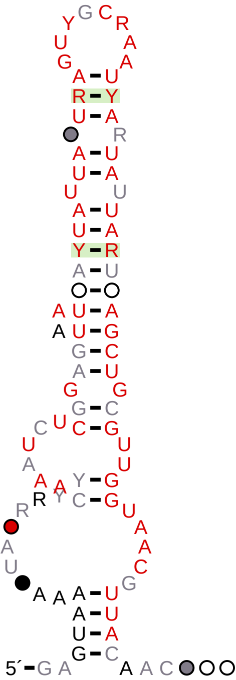

HMP2_5421_1

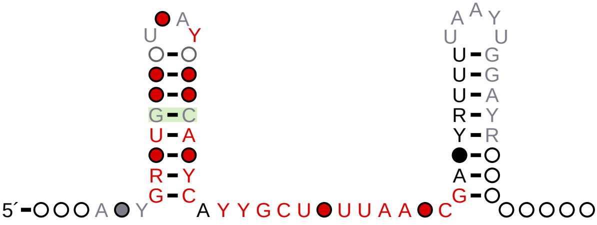

HMP2_5423_1

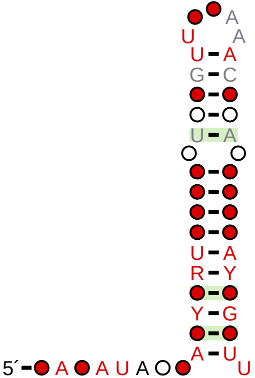

HMP2_5429_1

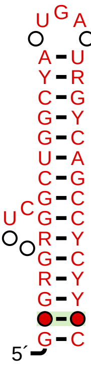

HMP2_5434_1

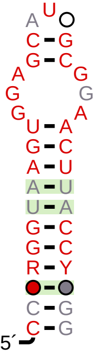

HMP2_5452_1

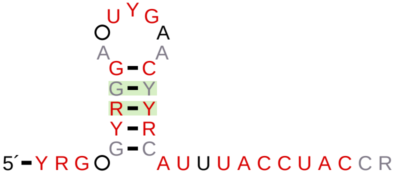

HMP2_5454_1

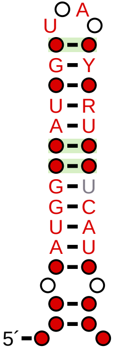

HMP2_5461_1

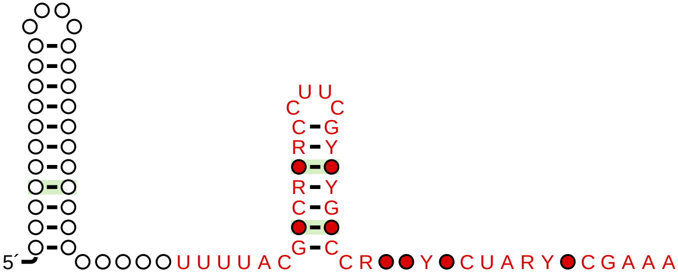

HMP2_5465_1

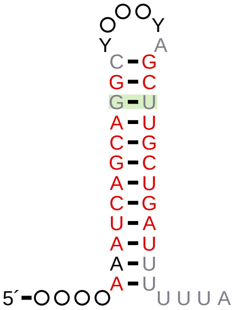

HMP2_5466_1

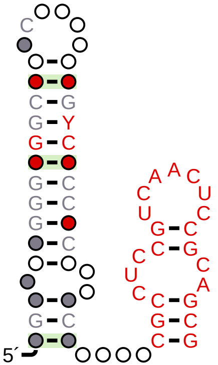

HMP2_5472_1

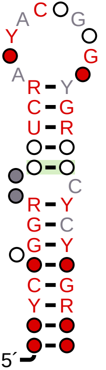

HMP2_5478_1

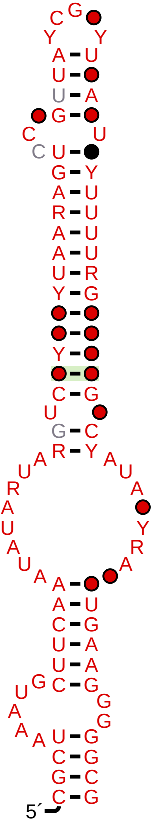

HMP2_5481_1

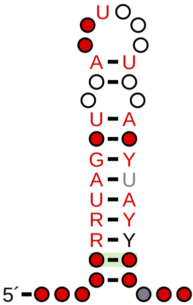

HMP2_5483_1

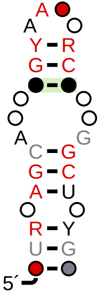

HMP2_5497_1

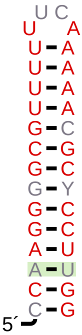

HMP2_5498_1

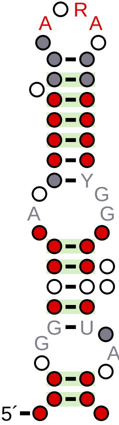

HMP2_5502_1

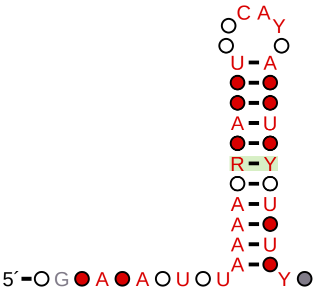

HMP2_5508_1

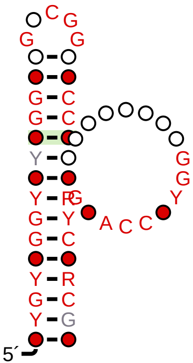

HMP2_5517_1

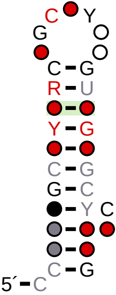

HMP2_5521_1

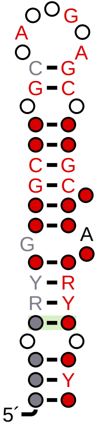

HMP2_5538_1

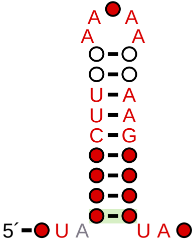

HMP2_5547_1

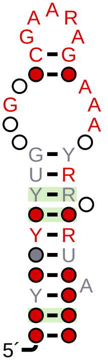

HMP2_555_1

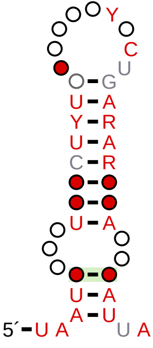

HMP2_5550_1

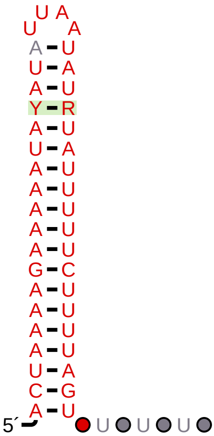

HMP2_5551_1

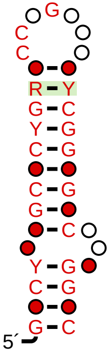

HMP2_5552_1

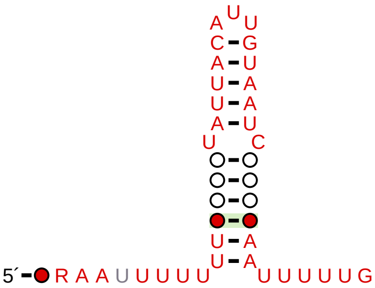

HMP2_5558_1

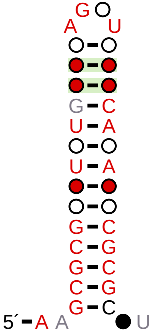

HMP2_556_1

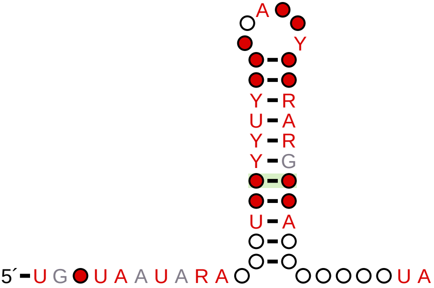

HMP2_5563_1

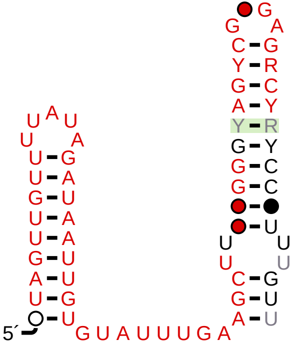

HMP2_5566_1

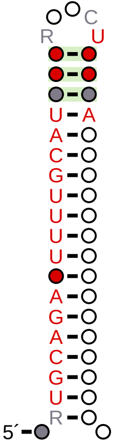

HMP2_5572_1

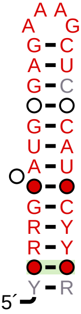

HMP2_5589_1

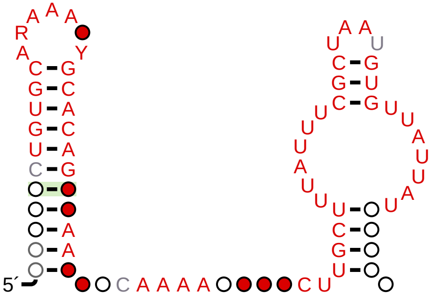

HMP2_560_1

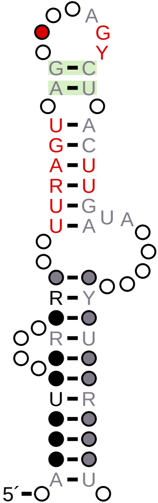

HMP2_5606_1

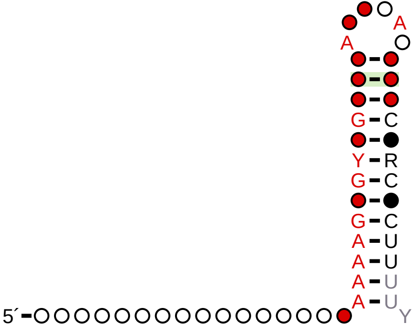

HMP2_5608_1

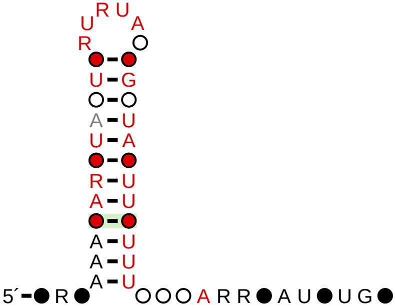

HMP2_5619_1

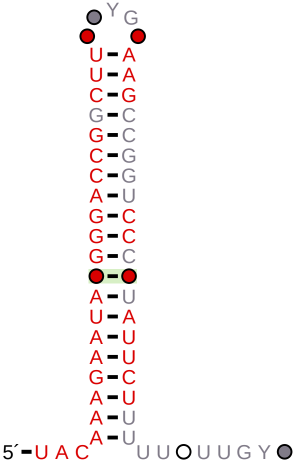

HMP2_562_1

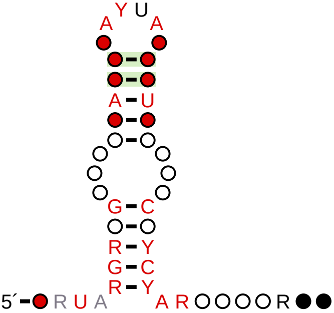

HMP2_5637_1

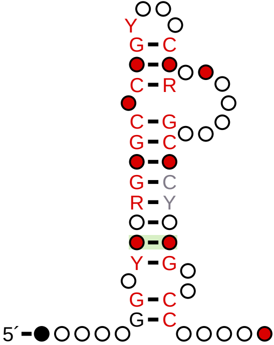

HMP2_5640_1

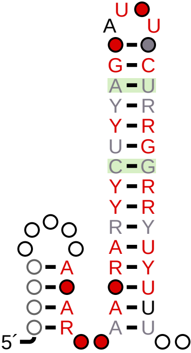

HMP2_5642_1

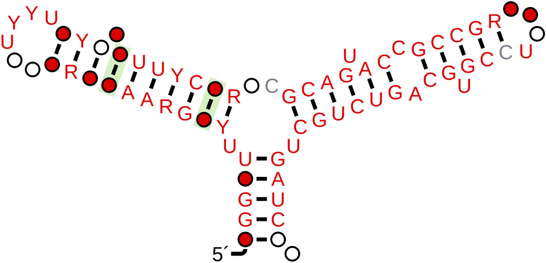

HMP2_5645_1

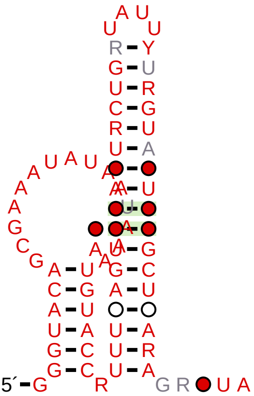

HMP2_5647_1

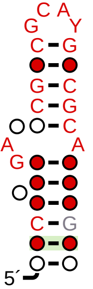

HMP2_5654_1

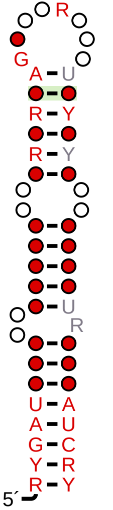

HMP2_5658_1

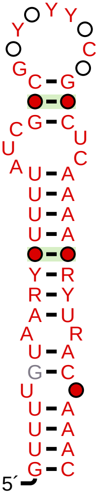

HMP2_5662_1

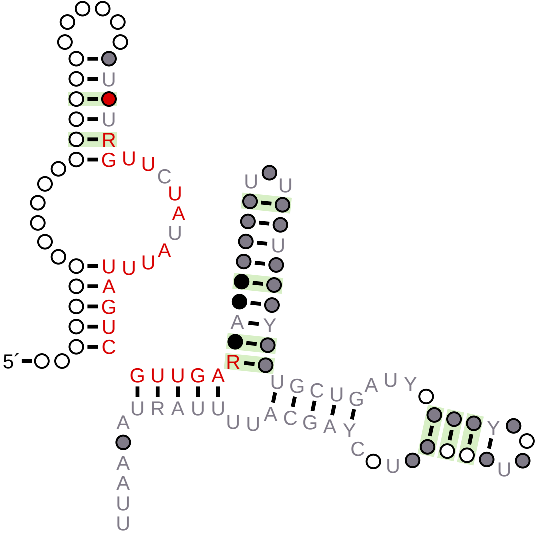

HMP2_5665_1

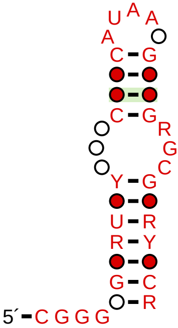

HMP2_5685_1

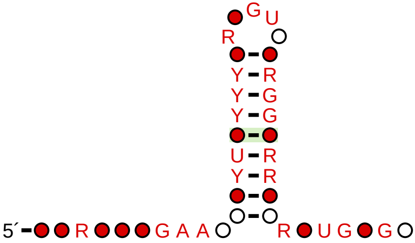

HMP2_569_1

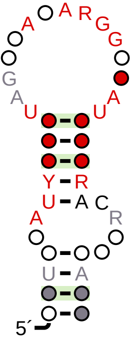

HMP2_5691_1

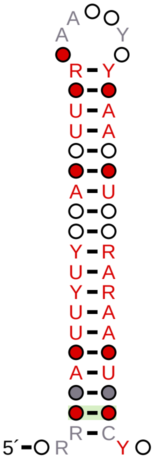

HMP2_5699_1

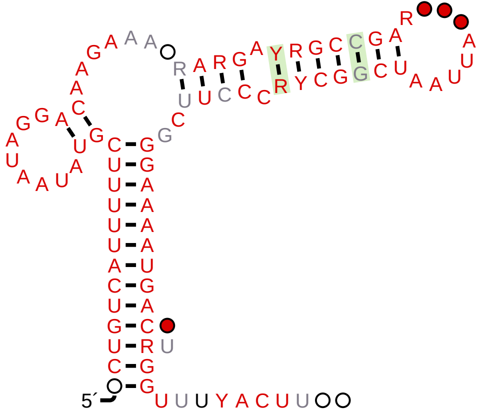

HMP2_5702_1

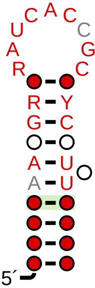

HMP2_5703_1

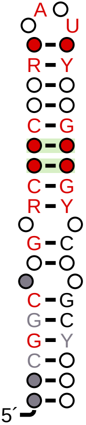

HMP2_5706_1

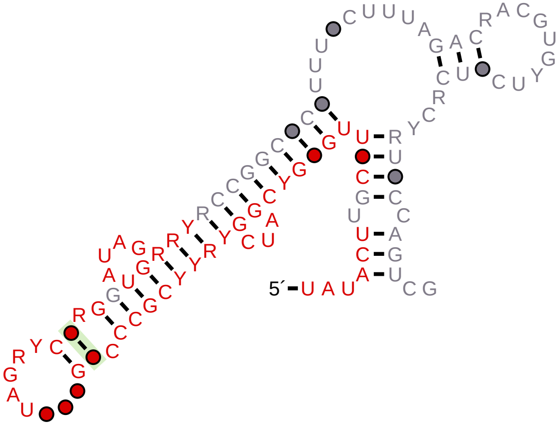

HMP2_5709_1

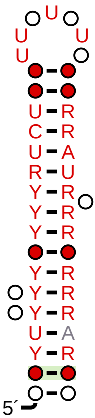

HMP2_5711_1

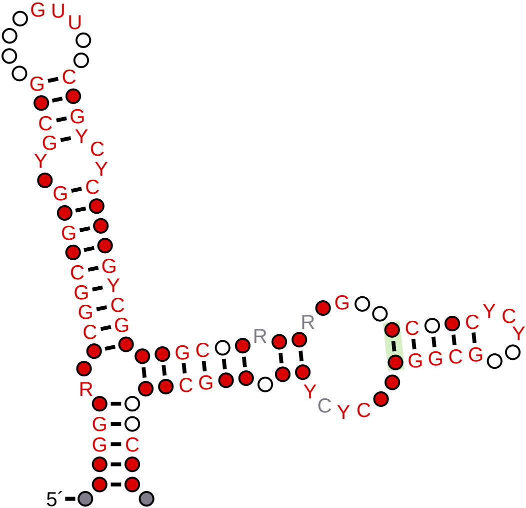

HMP2_5712_1

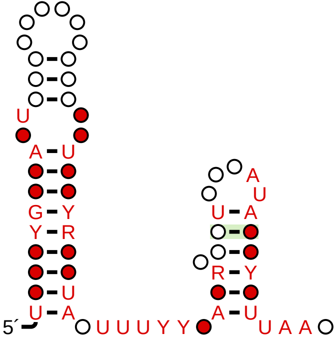

HMP2_5736_1

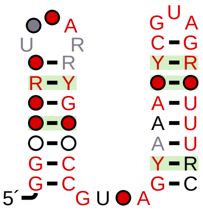

HMP2_574_1

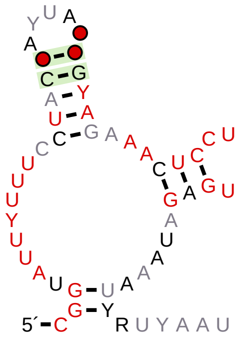

Y U A
A
C G
A Y
U A
C G A
C
U
U
U
Y
U
U
A
U
G U
G Y
5´ C
R U Y A A U
A A
C U
C
U C U
C
U
G A G U A
A
U
A
A
A

HMP2_5742_1

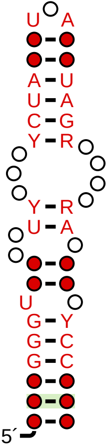

HMP2_5750_1

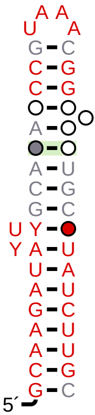

HMP2_5759_1

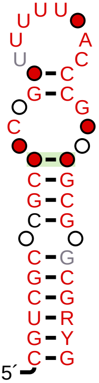

HMP2_5763_1

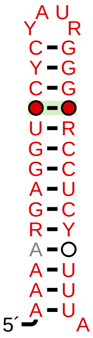

HMP2_5766_1

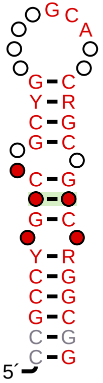

HMP2_577_1

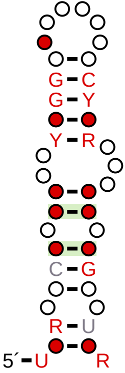

HMP2_5773_1

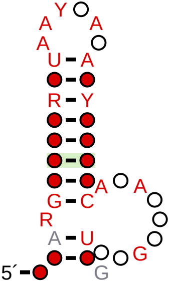

HMP2_5776_1

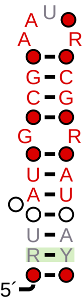

HMP2_5785_1

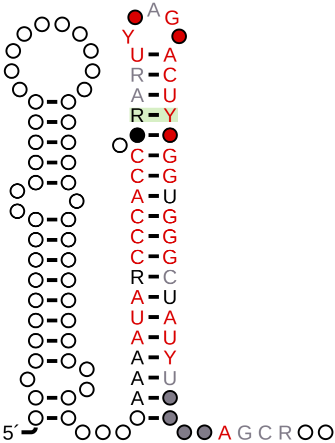

HMP2_5790_1

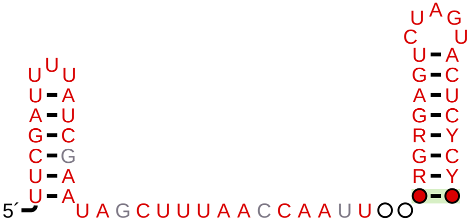

5´
U A G C U U U A A C C A A U U
U U U
U A
A U
G C
C G
U A
U A
U A G
C U
U A
G C
A U
G C
R Y
G C
R Y

HMP2_5794_1

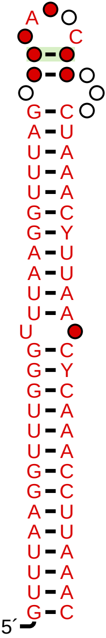
A
C
G-C
A-U
U-A
U-A
U-A
G-C
G-Y
A-U
A-U
U-A
U-A
U
G-C
G-Y
G-C
U-A
U-A
U-A
G-C
G-C
A-U
A-U
U-A
U-A
U-A
G-C
5´

HMP2_5801_1

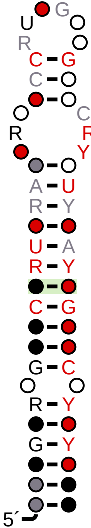

HMP2_5807_1

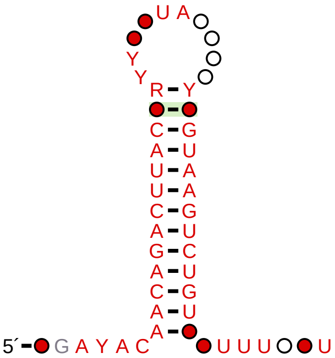

HMP2_5817_1

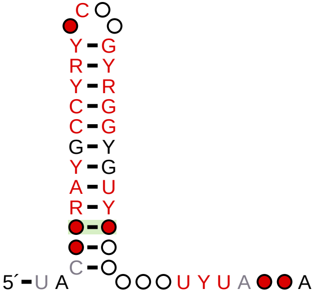

HMP2_5818_1

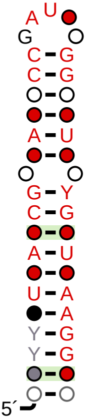

HMP2_5822_1

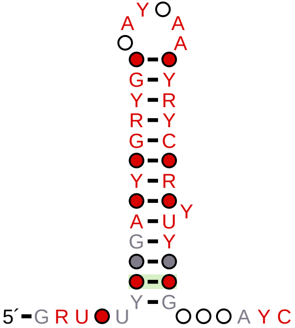

HMP2_5823_1

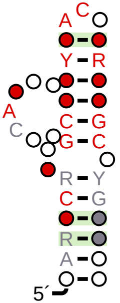

HMP2_5826_1

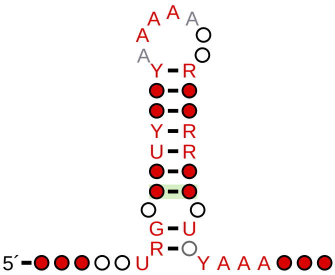

HMP2_583_1

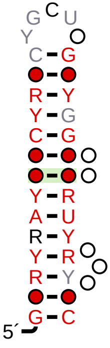

HMP2_5831_1

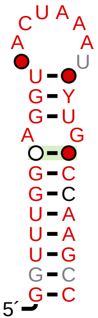

HMP2_5840_1

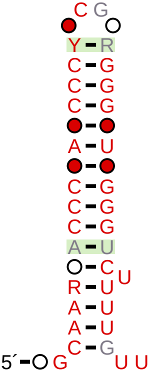

HMP2_585_1

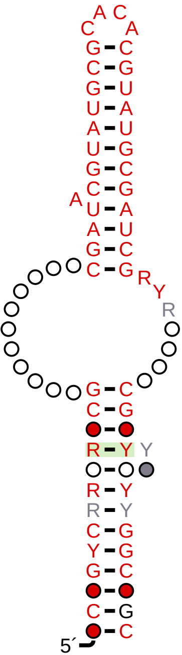

HMP2_5863_1

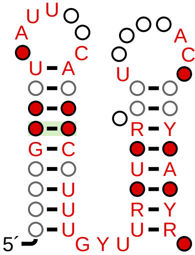

HMP2_5864_1

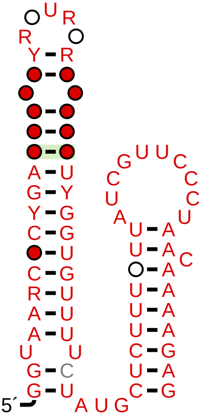

HMP2_587_1

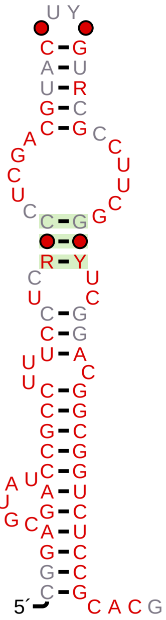

HMP2_5870_1

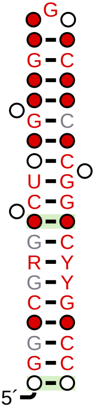

HMP2_5875_1

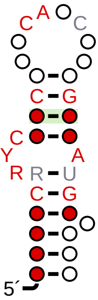

HMP2_5881_1

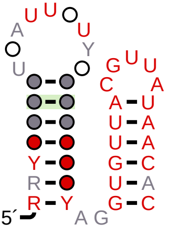

HMP2_5883_1

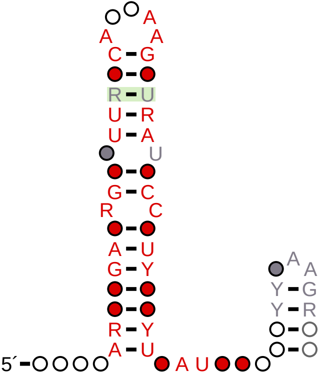

HMP2_590_1

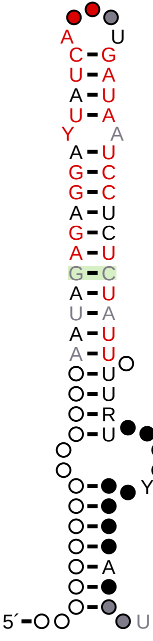

HMP2_5903_1

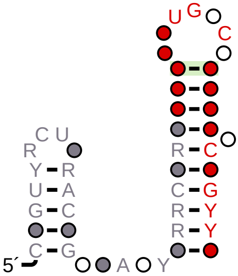

HMP2_5910_1

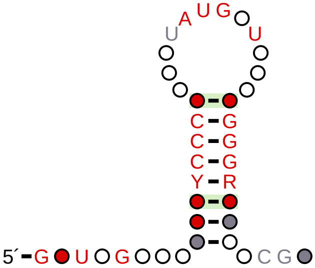

HMP2_5911_1

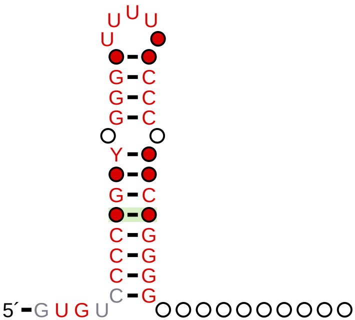

HMP2_5914_1

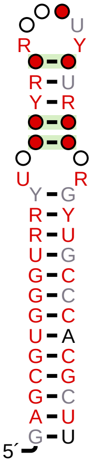

HMP2_5920_1

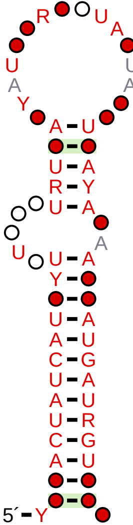

HMP2_5926_1

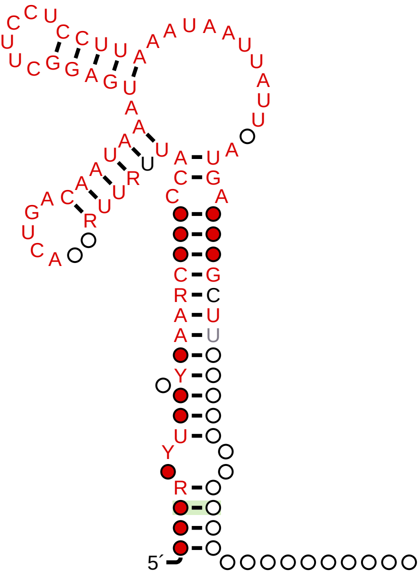

HMP2_5933_1

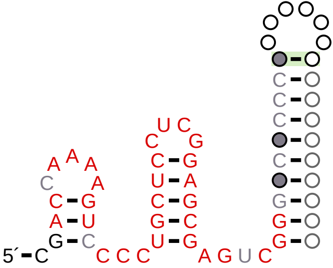

HMP2_5935_1

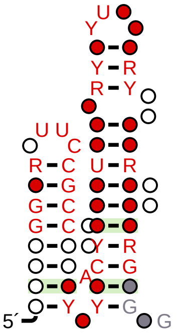

HMP2_5937_1

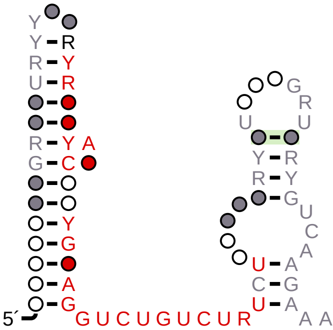

HMP2_5938_1

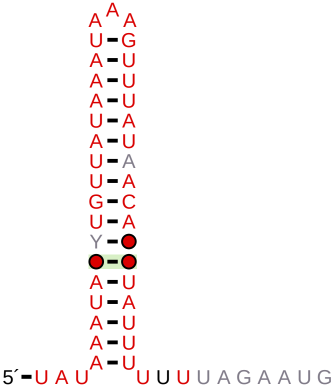

HMP2_5941_1

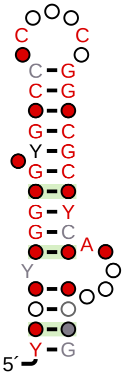

HMP2_5948_1

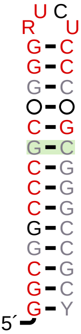

HMP2_5952_1

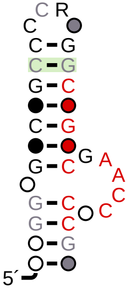

HMP2_5954_1

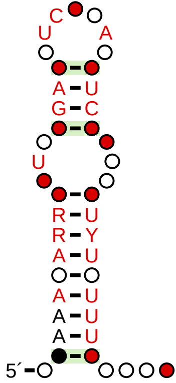

HMP2_5956_1

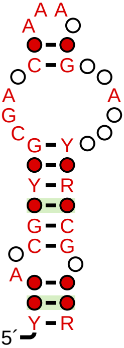

HMP2_596_1

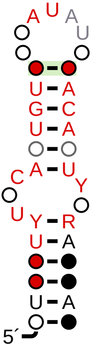

HMP2_5961_1

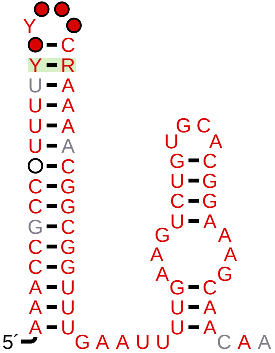

HMP2_5971_1

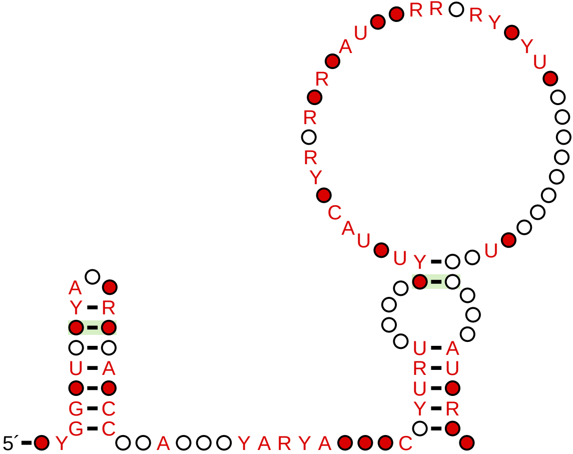

HMP2_5977_1

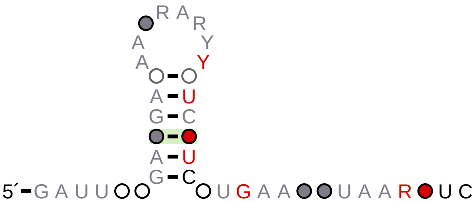

HMP2_598_1

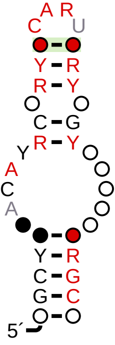

HMP2_5983_1

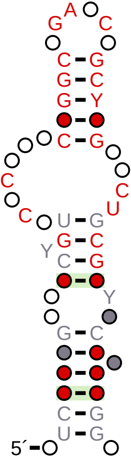

HMP2_5992_1

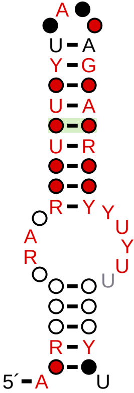

HMP2_5993_1

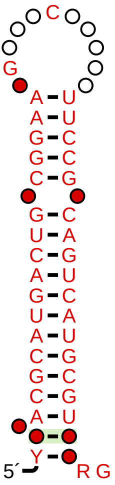

HMP2_5997_1

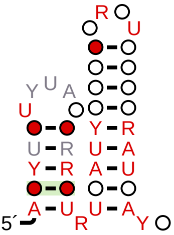

HMP2_5999_1

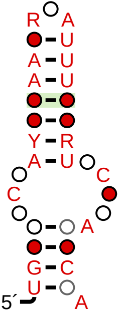

HMP2_6001_1

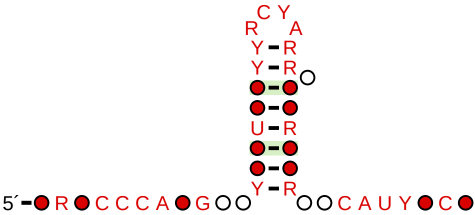

HMP2_6003_1

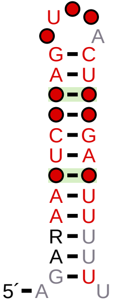

HMP2_6005_1

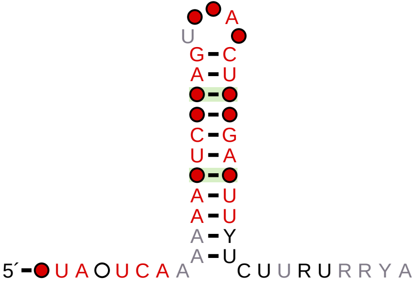

HMP2_6009_1

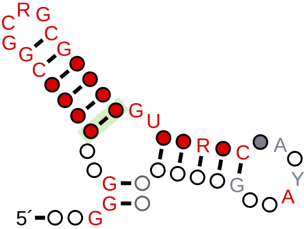

HMP2_6014_1

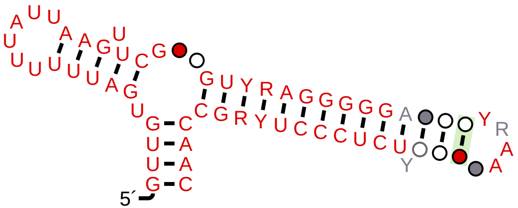

HMP2_6018_1

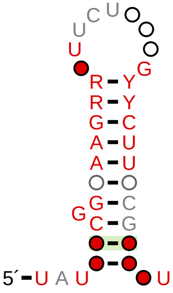

HMP2_6021_1

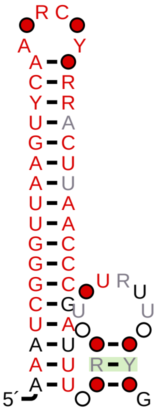

HMP2_6022_1

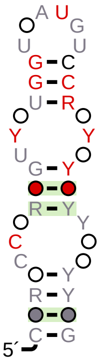

HMP2_6031_1

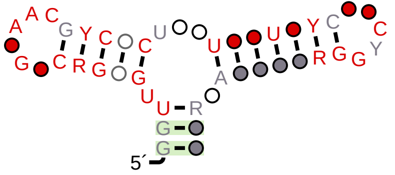

HMP2_6035_1

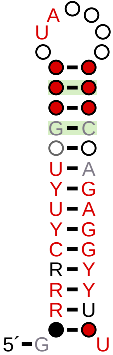

HMP2_6037_1

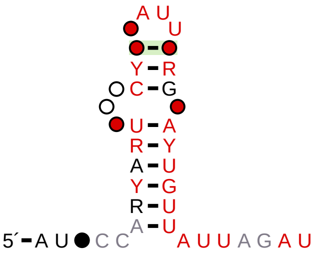

HMP2_6045_1

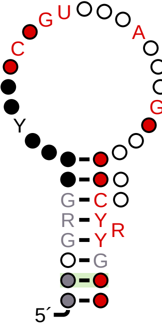

HMP2_6046_1

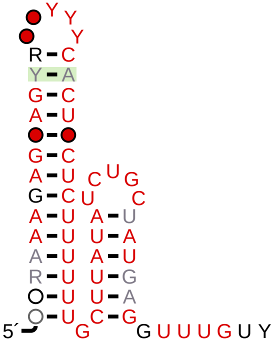
Y Y Y
R C
Y A
G C
A U
G C
A U
G C
A U
A U
A U
R U
U
U
5′
C U G
U C
A U
U A
A U
U G
U A
C G
G
G U U U G U Y

HMP2_6048_1

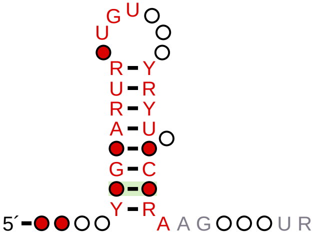

HMP2_6055_1

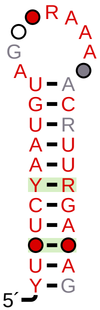

HMP2_6066_1

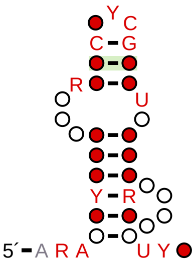

HMP2_6080_1

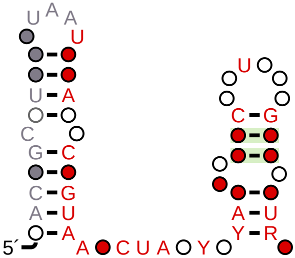

HMP2_6082_1

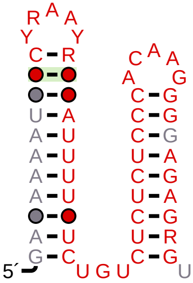

HMP2_6085_1

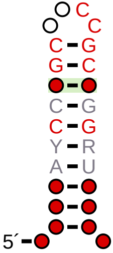

HMP2_6086_1

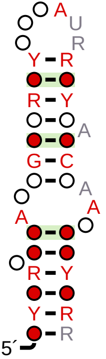

HMP2_6091_1

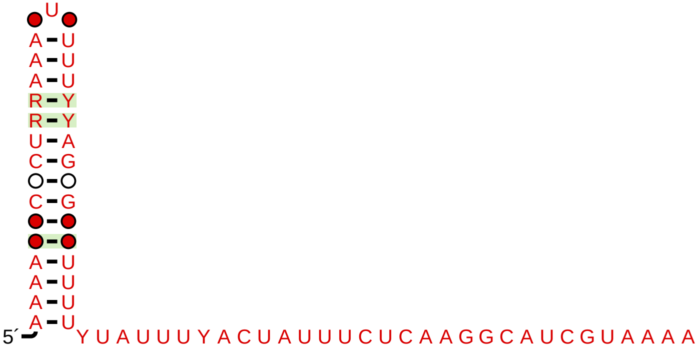
U
A U
A U
A U
R Y
R Y
U A
C G
C G
A U
A U
A U
A U
5´
Y U A U U U Y A C U A U U U C U C A A G G C A U C G U A A A A

HMP2_6097_1

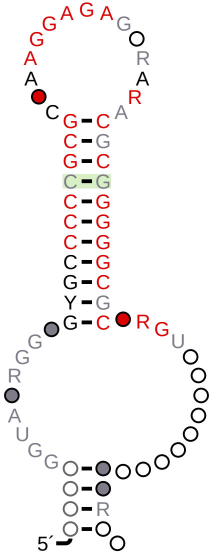

HMP2_6104_1

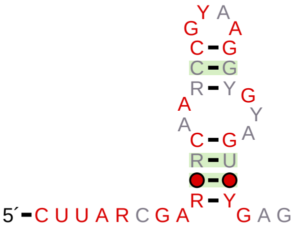

HMP2_6107_1

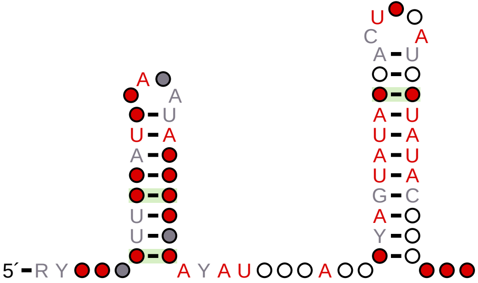

HMP2_6108_1

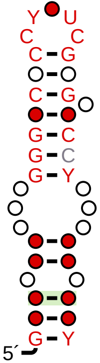

HMP2_6109_1

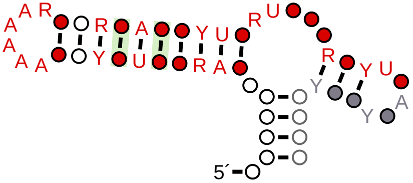

HMP2_6114_1

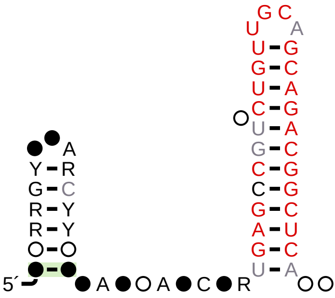

HMP2_6123_1

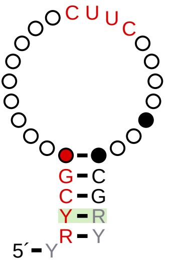

HMP2_6125_1

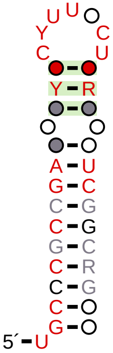

HMP2_6134_1

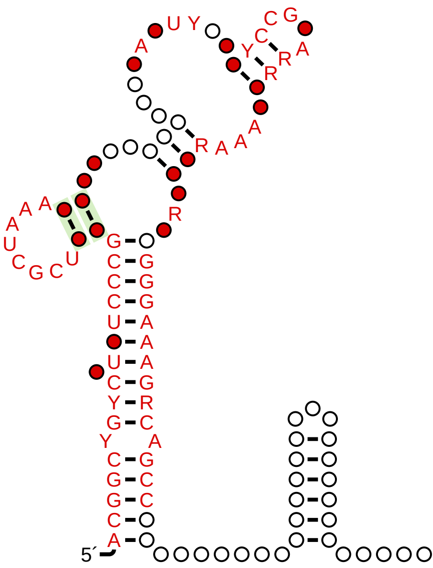

HMP2_6143_1

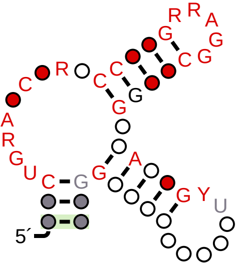

HMP2_6158_1

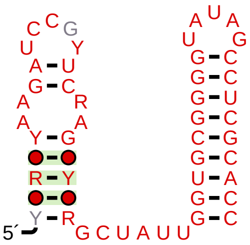

HMP2_6159_1

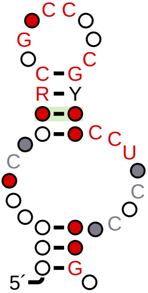

HMP2_6166_1

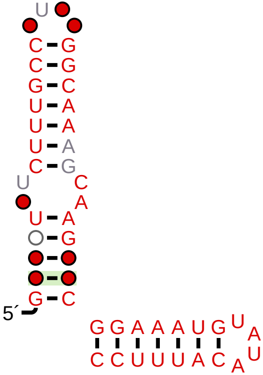

HMP2_6169_1

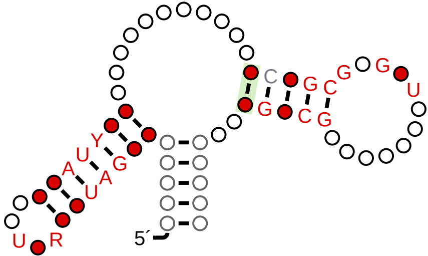

HMP2_6170_1

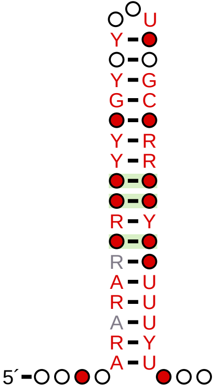

HMP2_6178_1

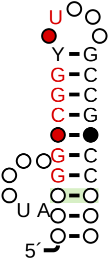

HMP2_6179_1

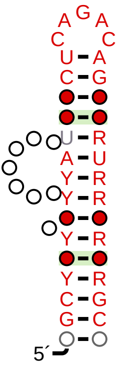

HMP2_618_1

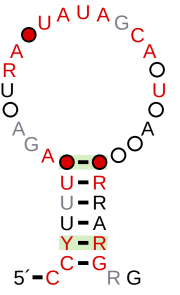

HMP2_6184_1

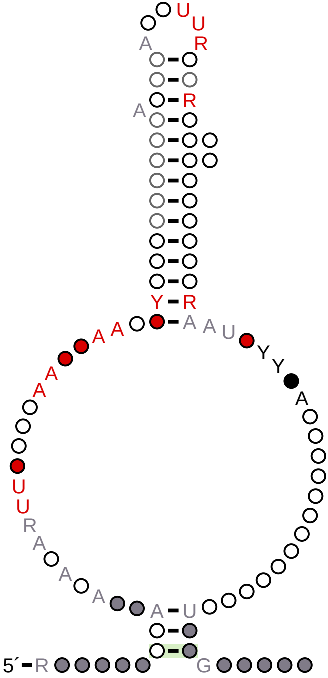

HMP2_6185_1

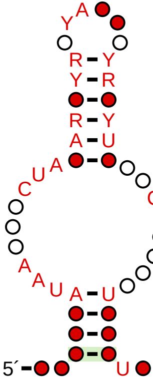

HMP2_6187_1

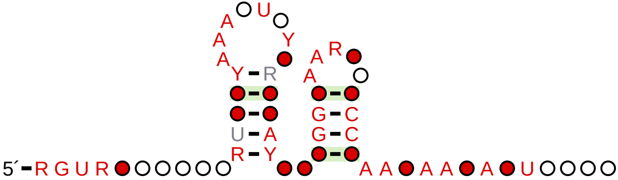

HMP2_6198_1

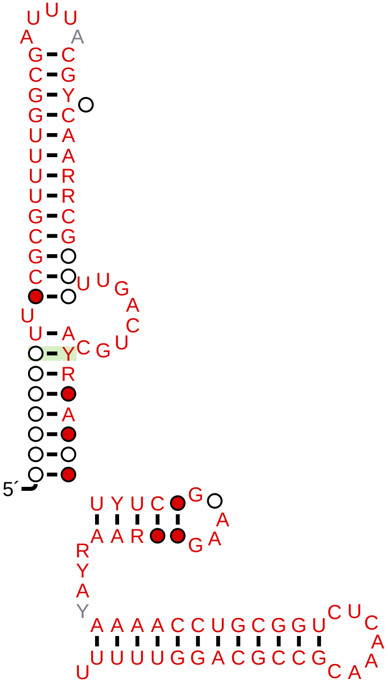

HMP2_62_1

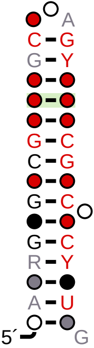

HMP2_6204_1

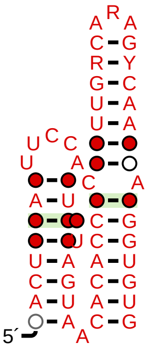

HMP2_6205_1

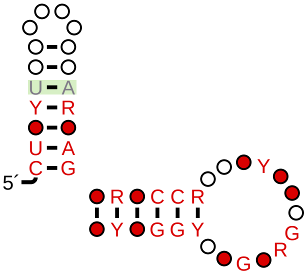

HMP2_6208_1

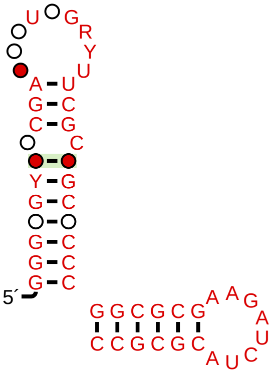

HMP2_6219_1

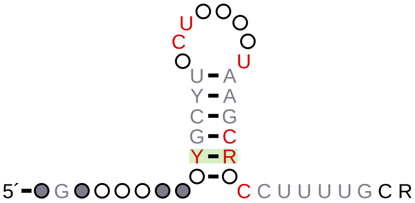

HMP2_6221_1

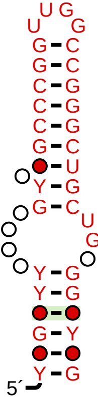

HMP2_6226_1

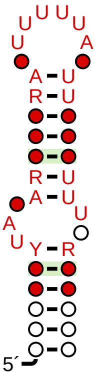

HMP2_6231_1

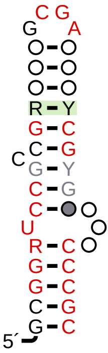

HMP2_624_1

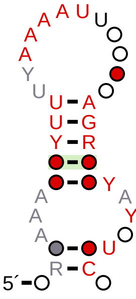

HMP2_6240_1

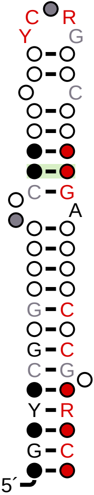

HMP2_6242_1

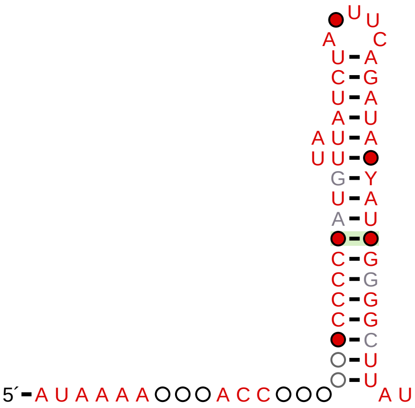

HMP2_6243_1

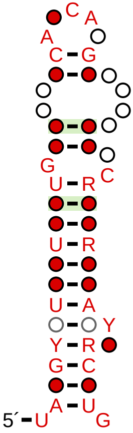

HMP2_6253_1

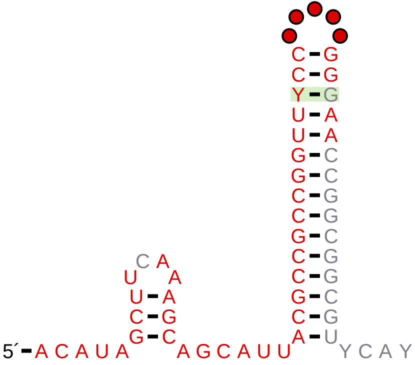
5´
ACAUA
GCUUCA
AAGC
AGCAUU
ACGGCCGCCGGUUYCC
GGAAGGCCGCCGCAU
YCAY

HMP2_6258_1

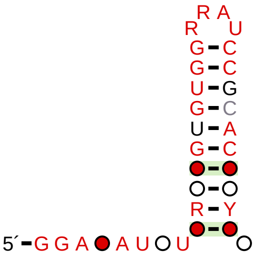

HMP2_6265_1

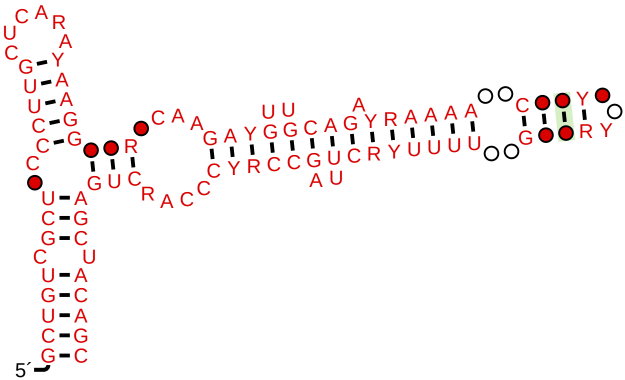

HMP2_6268_1

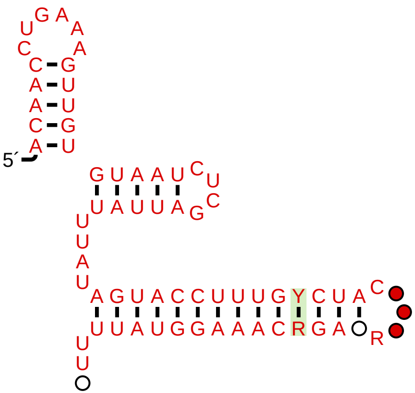

UGAA
C A
C G
A U
A U
C G
A U
5´
GUAAUCU
UAUUAGC
UUAU
AGUACCUUUGYCUAC
UUAUGGAAACRGA R
UU

HMP2_6273_1

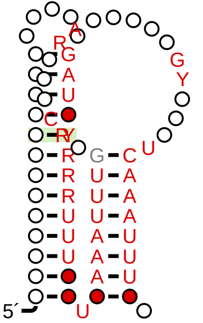

HMP2_6288_1

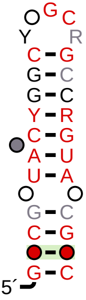

HMP2_6290_1

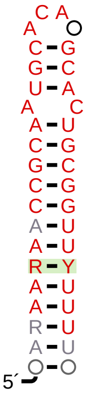

HMP2_6295_1

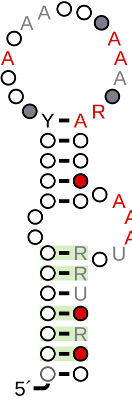

HMP2_6299_1

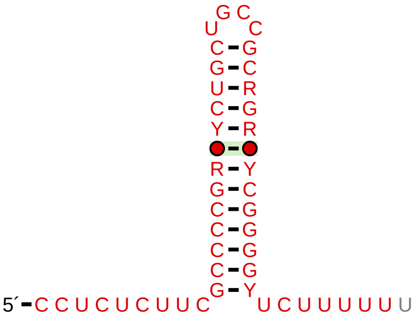

HMP2_6301_1

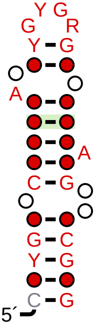

HMP2_6309_1

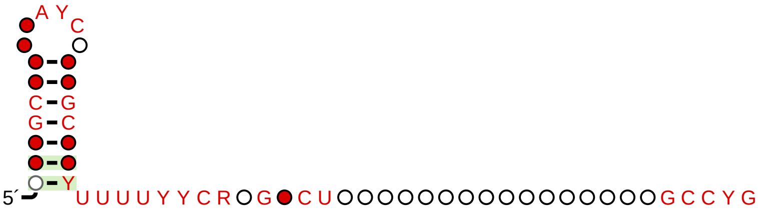

HMP2_6311_1

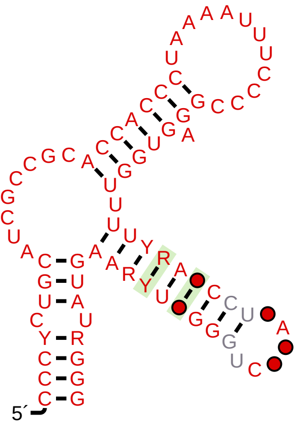

HMP2_6313_1

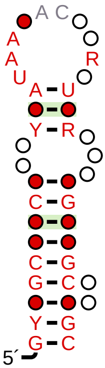

HMP2_6315_1

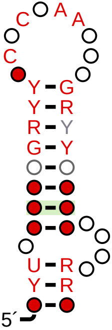

HMP2_6317_1

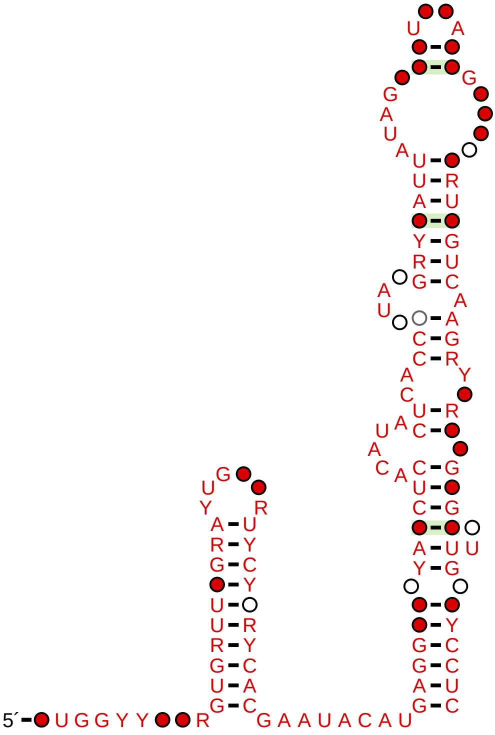

HMP2_6320_1

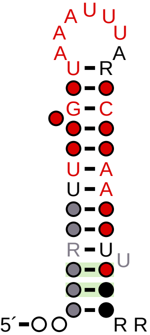

HMP2_6323_1

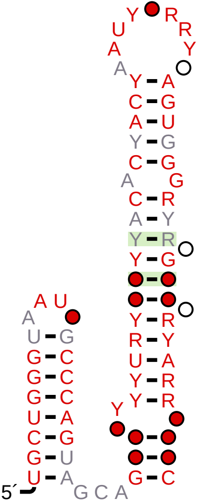

HMP2_6326_1

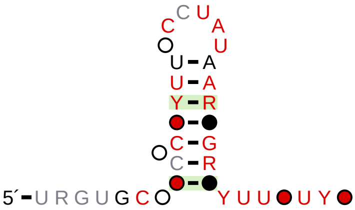

HMP2_6327_1

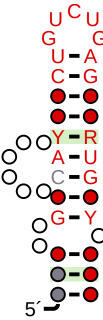

HMP2_6328_1

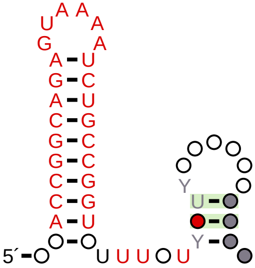

HMP2_6330_1

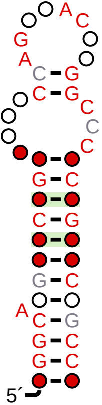

HMP2_6365_1

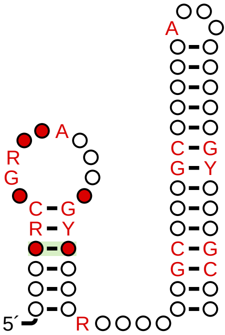

HMP2_6367_1

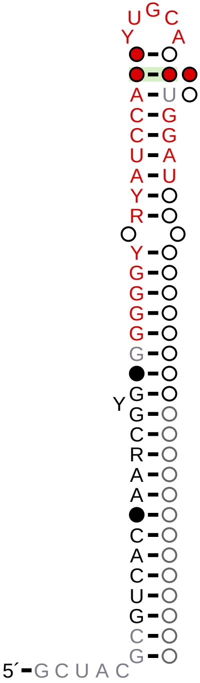

HMP2_637_1

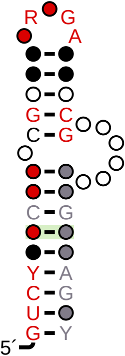

HMP2_6376_1

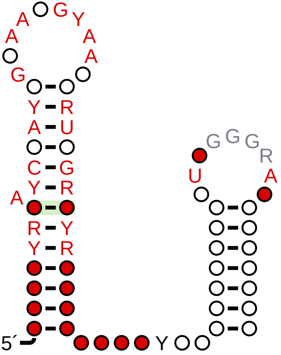

HMP2_6384_1

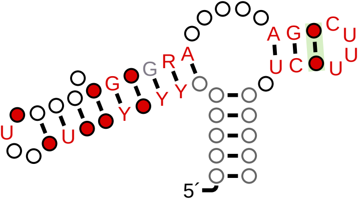

HMP2_6386_1

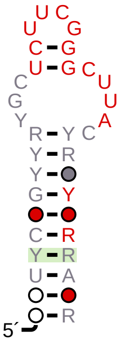

HMP2_6387_1

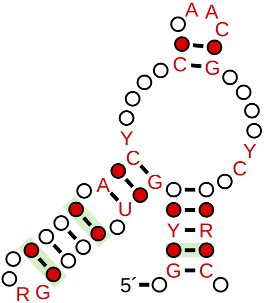

HMP2_6391_1

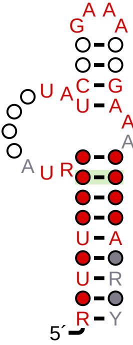

HMP2_6405_1

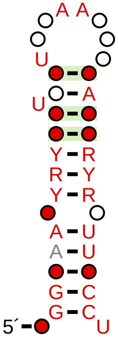

HMP2_6408_1

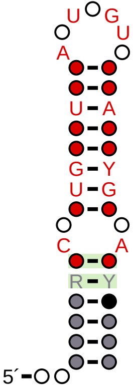

HMP2_6416_1

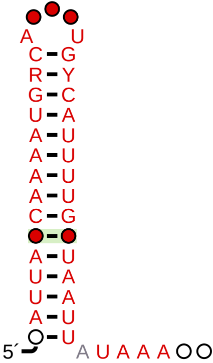

HMP2_6422_1

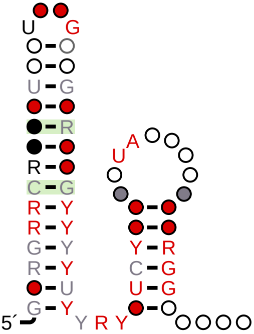

HMP2_6423_1

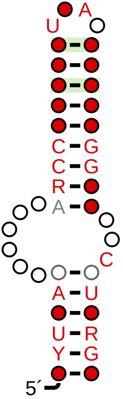

HMP2_6426_1

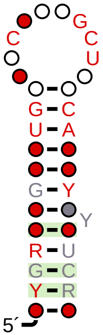

HMP2_6428_1

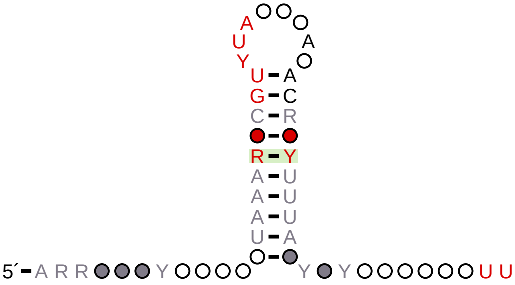

HMP2_6433_1

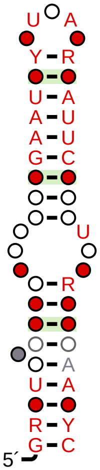

HMP2_644_1

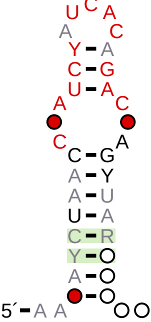

HMP2_6447_1

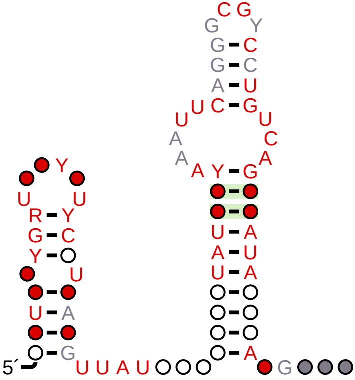

HMP2_6449_1

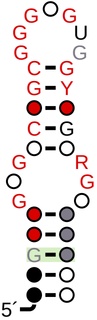

HMP2_6465_1

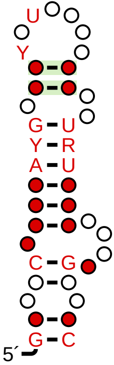

HMP2_6466_1

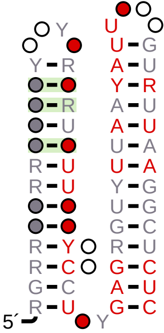

HMP2_6467_1

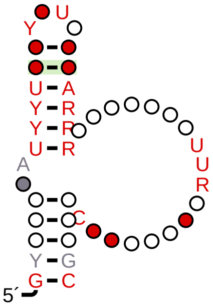

HMP2_6473_1

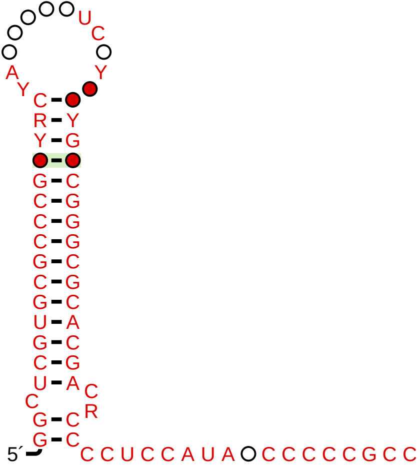
5´
U
C
A
Y
C
R
Y
G
C
C
C
G
C
G
U
G
C
U
C
G
G
C
C
Y
G
C
G
G
G
C
G
C
A
C
G
A
C
R
C
C
CCUCCAUA
CCCCCGCC

HMP2_648_1

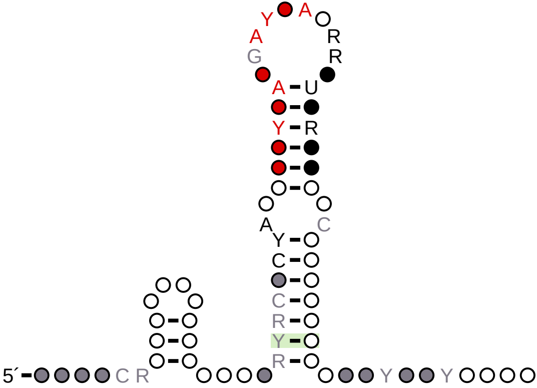

HMP2_6481_1

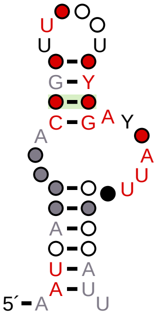

HMP2_6484_1

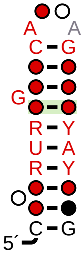

HMP2_6491_1

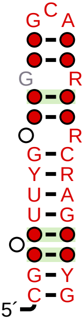

HMP2_6497_1

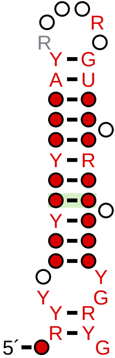

HMP2_6504_1

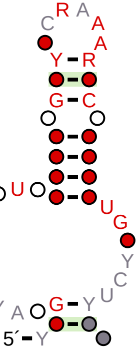

HMP2_6509_1

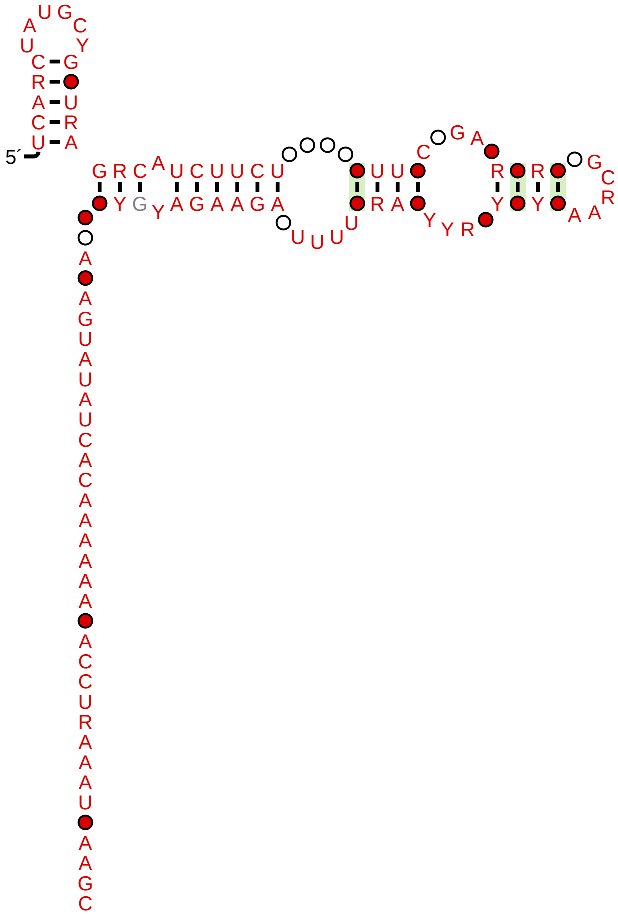

HMP2_6518_1

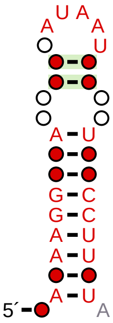

HMP2_6523_1

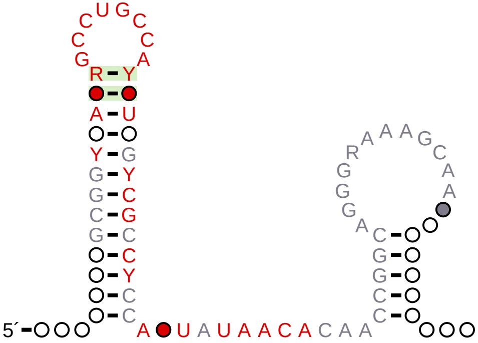

HMP2_6529_1

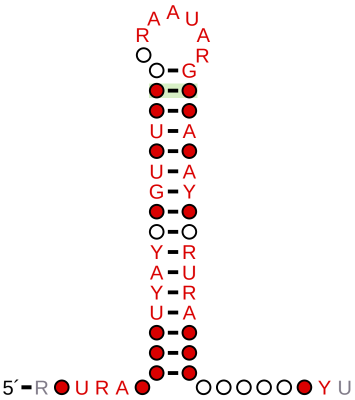

HMP2_6533_1

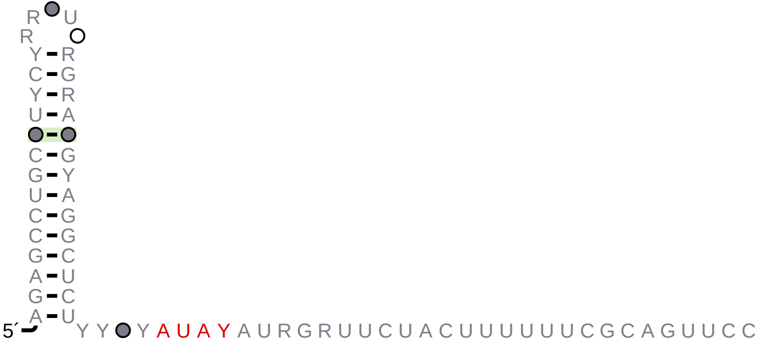

R U
R
Y R
C G
Y R
U A
C G
G Y
U A
C G
C G
G C
A U
G C
A U
5´
Y Y Y A U A Y A U R G R U U C U A C U U U U U U C G C A G U U C C

HMP2_6540_1

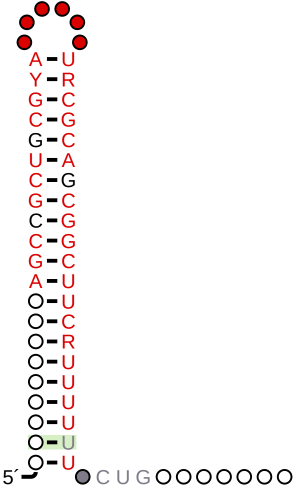

HMP2_6541_1

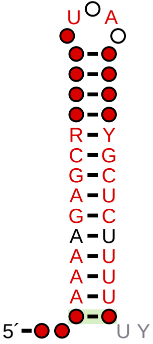

HMP2_6547_1

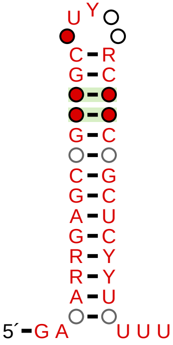

HMP2_6562_1

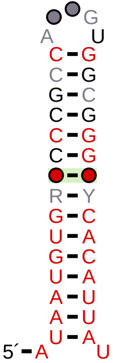

HMP2_6564_1

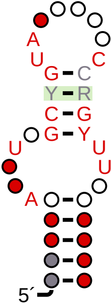

HMP2_6565_1

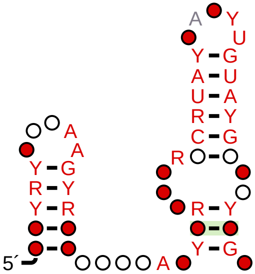

HMP2_6566_1

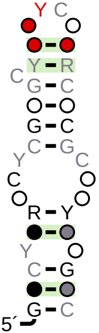

HMP2_6568_1

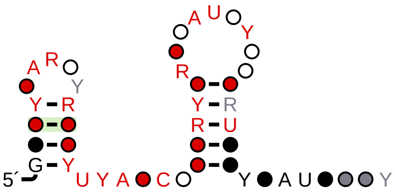

HMP2_6572_1

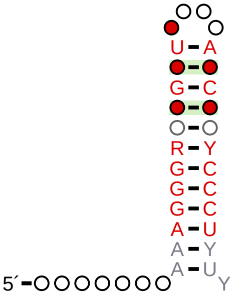

HMP2_6573_1

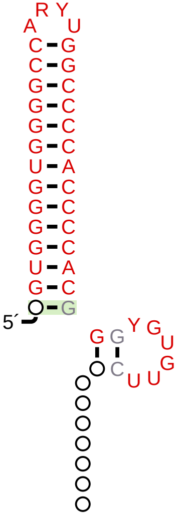

HMP2_6577_1

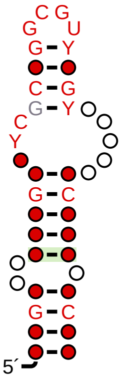

HMP2_6579_1

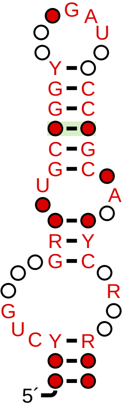

HMP2_6581_1

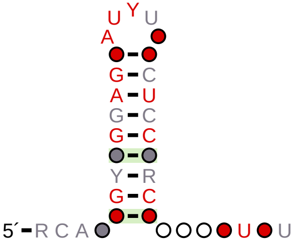

HMP2_6590_1

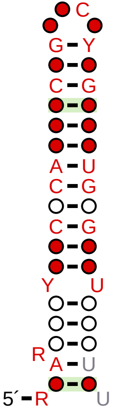

HMP2_6596_1

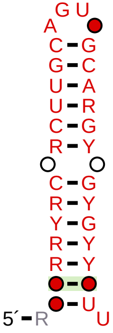

HMP2_66_1

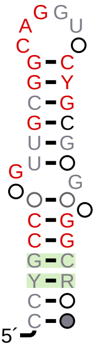

HMP2_6600_1

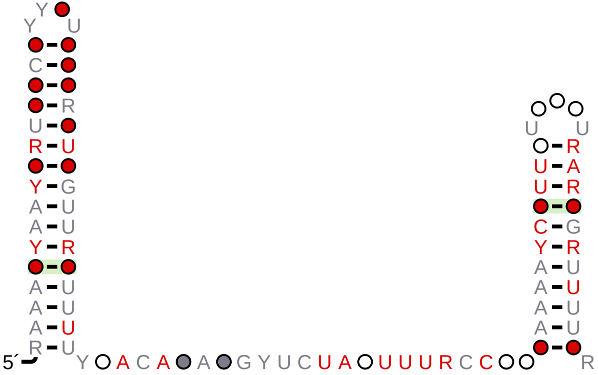
5´
Y A C A A G Y U C U A U U U R C C R
Y Y U
C
R
U
R U
Y G
A U
A U
Y R
A U
A U
A U
R U
U U
R
U A
U R
C G
Y R
A U
A U
A U
A U

HMP2_6602_1

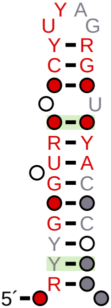

HMP2_6612_1

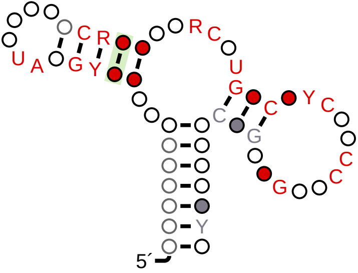

HMP2_6617_1

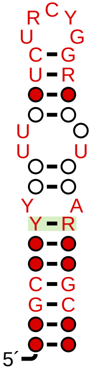

HMP2_6624_1

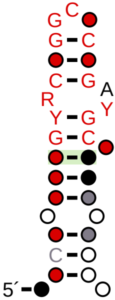

HMP2_6632_1

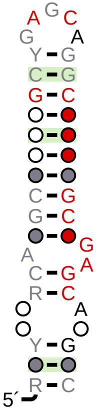

HMP2_6636_1

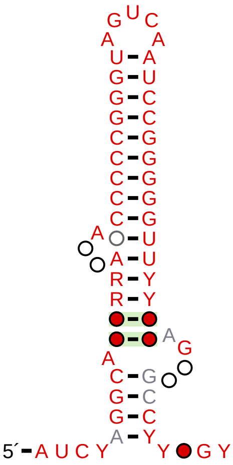

HMP2_6637_1

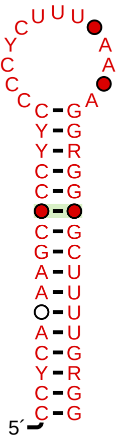

HMP2_6639_1

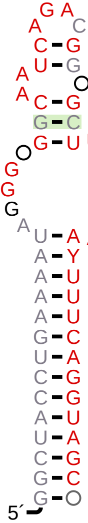

HMP2_6643_1

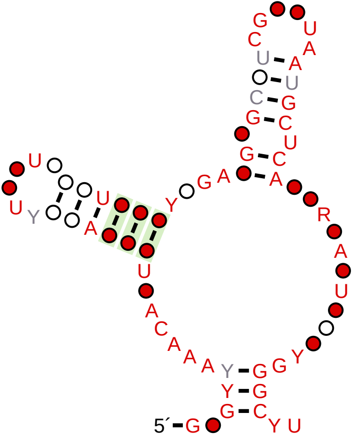

HMP2_6659_1

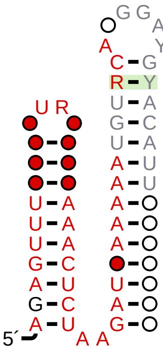

HMP2_6663_1

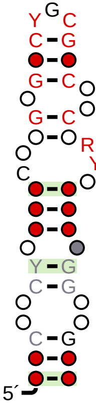

HMP2_6669_1

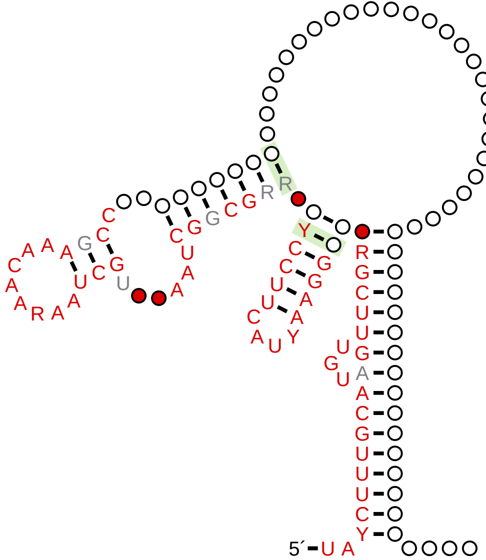

HMP2_6673_1

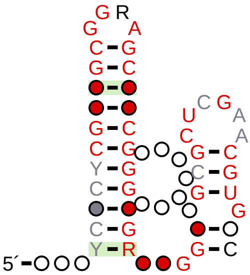

HMP2_6682_1

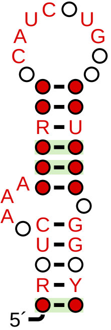

HMP2_6688_1

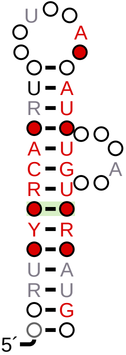

HMP2_669_1

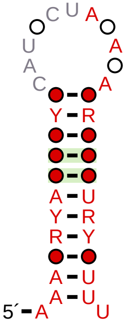

HMP2_6695_1

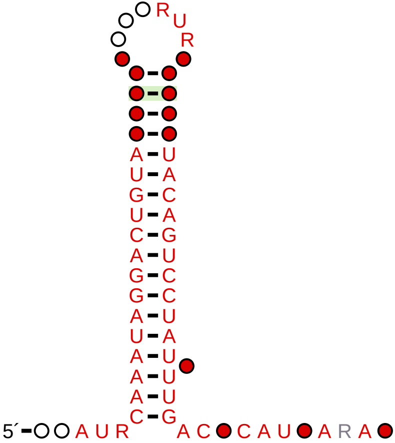

HMP2_6696_1

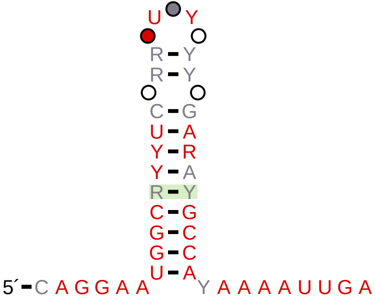

HMP2_6703_1

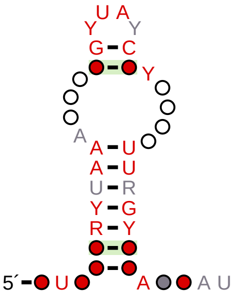

HMP2_6707_1

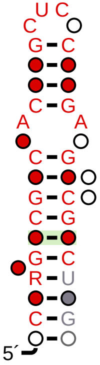

HMP2_6712_1

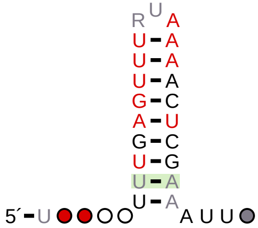

HMP2_6713_1

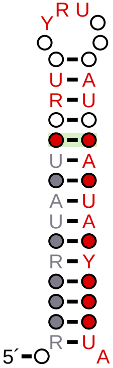

HMP2_6719_1

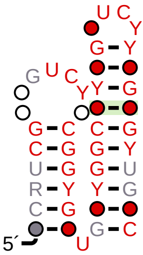

HMP2_6737_1

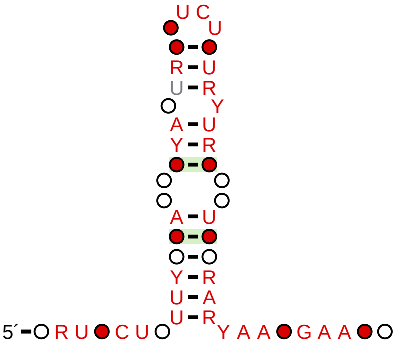

HMP2_6739_1

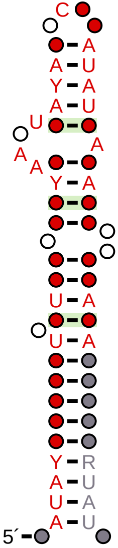

HMP2_6740_1

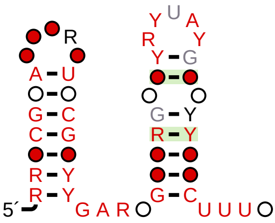

HMP2_6744_1

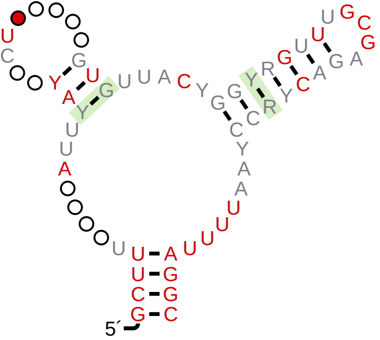

HMP2_6746_1

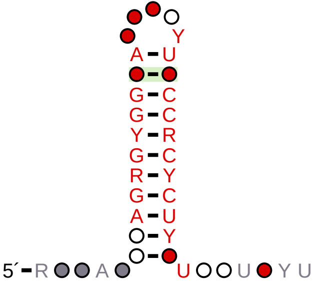

HMP2_6760_1

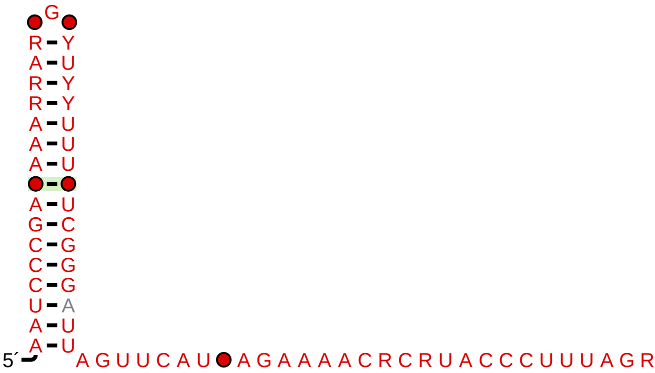
G
R Y
A U
R Y
R Y
A U
A U
A U
A U
G C
C G
C G
C G
U A
A U
A U
5´
A G U U C A U
A G A A A A C R C R U A C C C U U U A G R

HMP2_6763_1

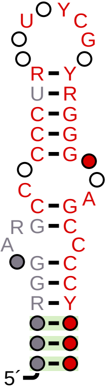

HMP2_6764_1

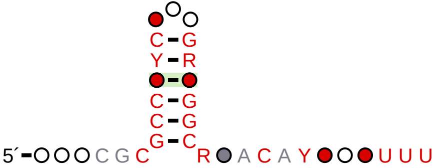

HMP2_6767_1

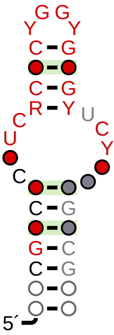

HMP2_6768_1

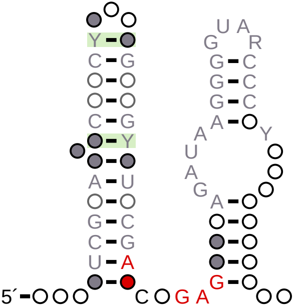

HMP2_6776_1

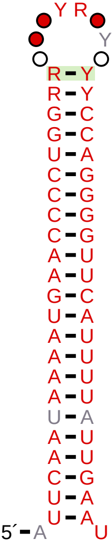

HMP2_6780_1

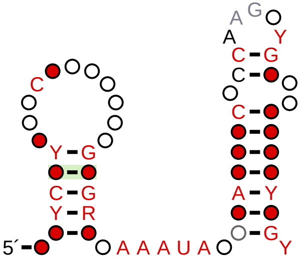

HMP2_6788_1

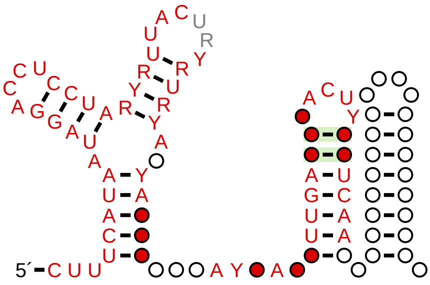

HMP2_679_1

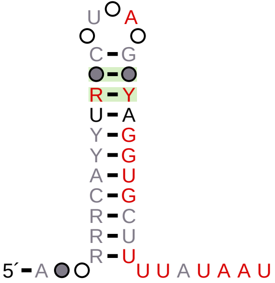

HMP2_6791_1

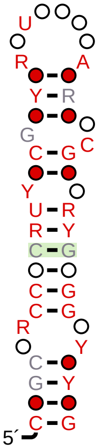

HMP2_6794_1

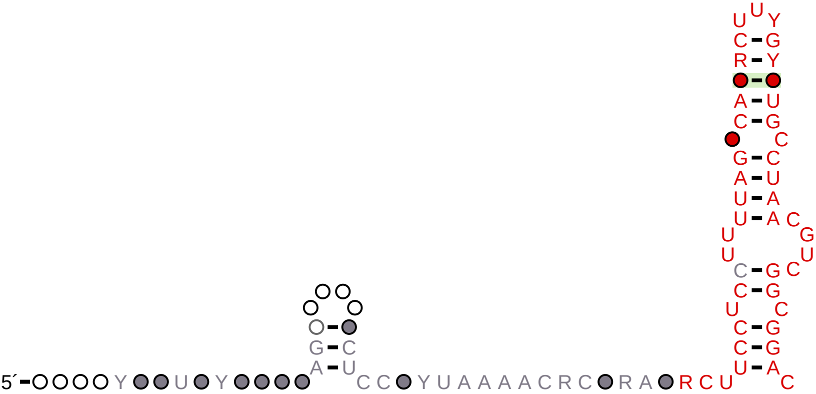

HMP2_68_1

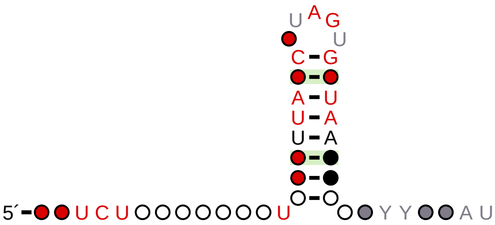

HMP2_6800_1

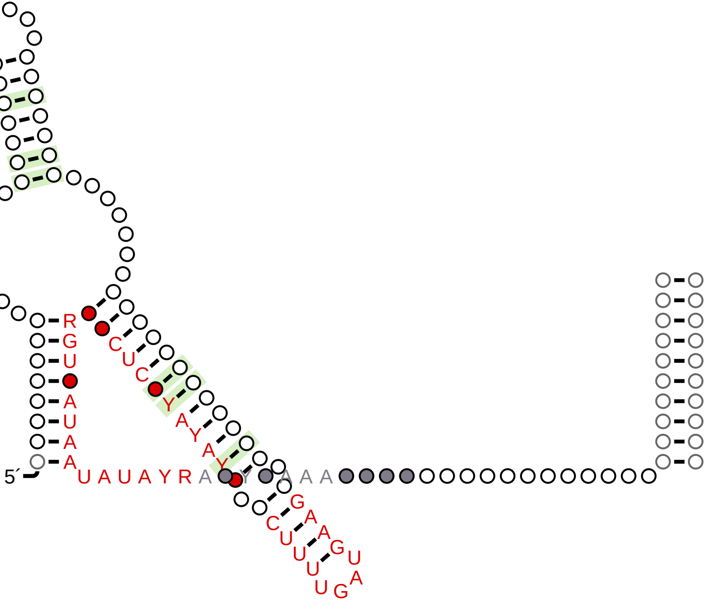

HMP2_6804_1

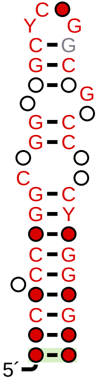

HMP2_6805_1

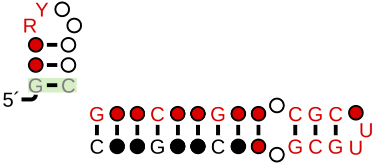

HMP2_681_1

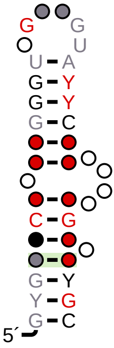

HMP2_6818_1

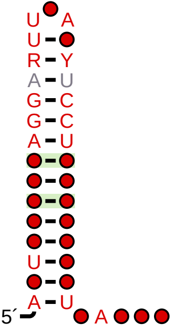

HMP2_6821_1

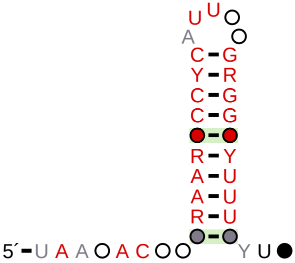

HMP2_6825_1

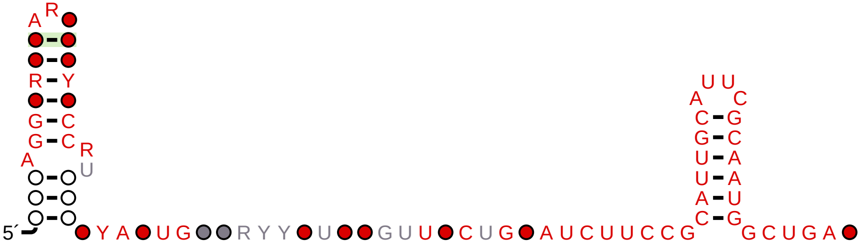

5´
Y A U G R Y Y U G U U C U G A U C U U C C G G C U G A
A R
R Y
G C
G C
A
R
U
U U
A C
C G
G C
U A
U A
A U
C G

HMP2_6826_1

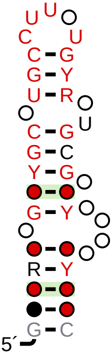

HMP2_683_1

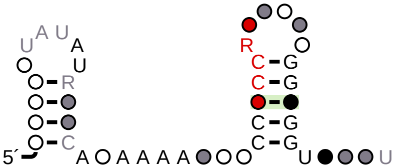

HMP2_6834_1

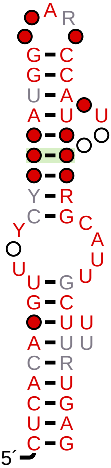

HMP2_6835_1

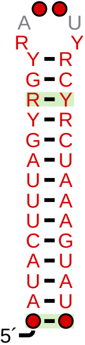

HMP2_6846_1

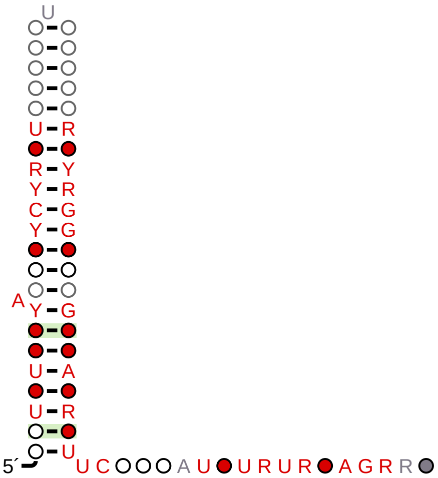

HMP2_6858_1

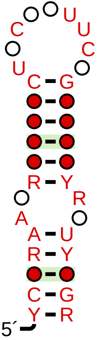

HMP2_686_1

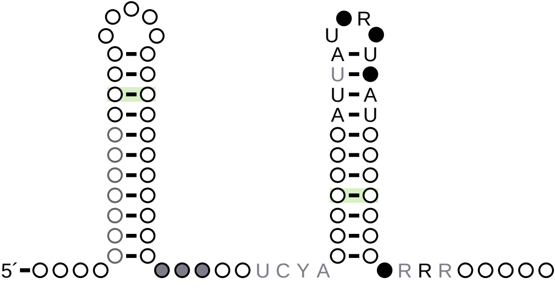

HMP2_6867_1

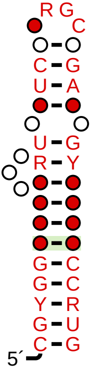

HMP2_6868_1

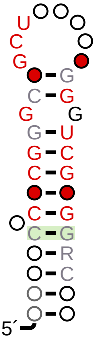

HMP2_687_1

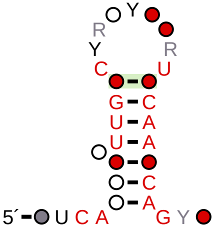

HMP2_6873_1

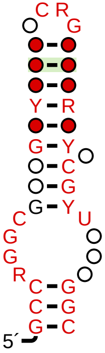

HMP2_6879_1

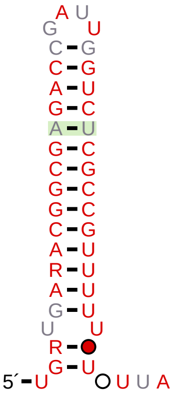

HMP2_6885_1

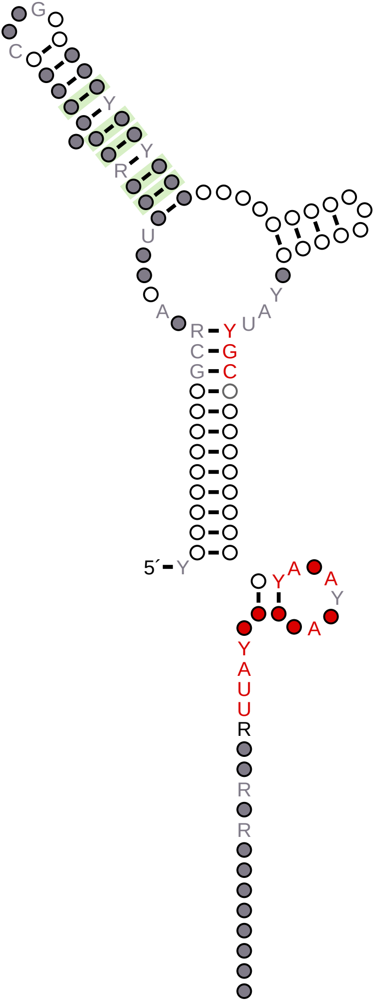

HMP2_6893_1

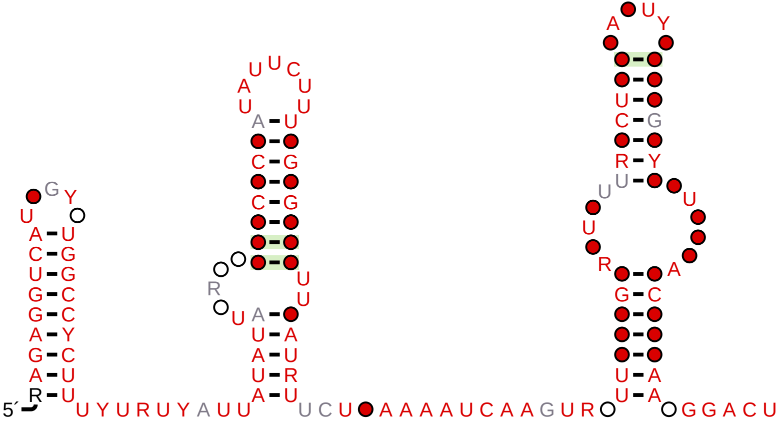

HMP2_690_1

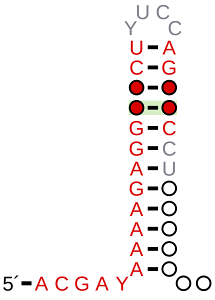

HMP2_6902_1

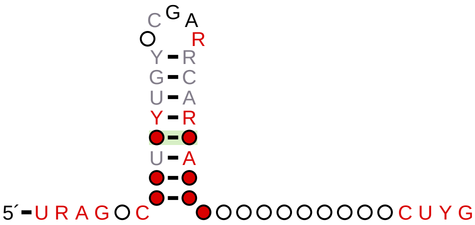

HMP2_6906_1

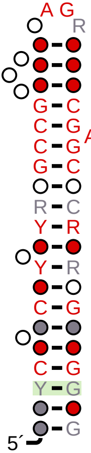

HMP2_6920_1

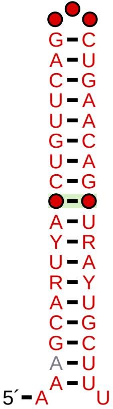

HMP2_6921_1

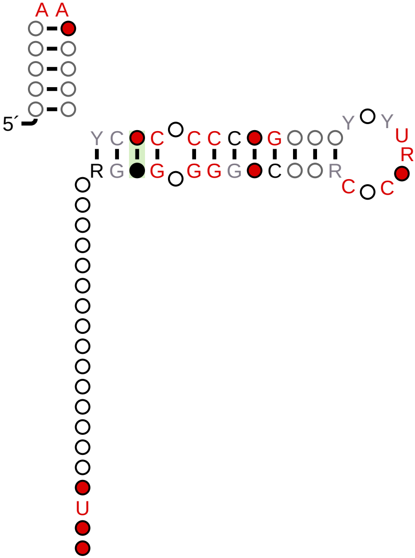

HMP2_6922_1

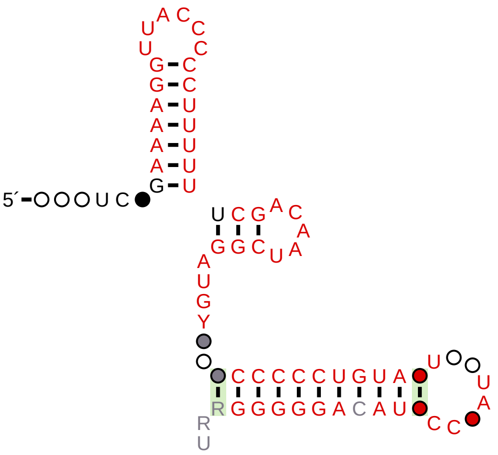

HMP2_6923_1

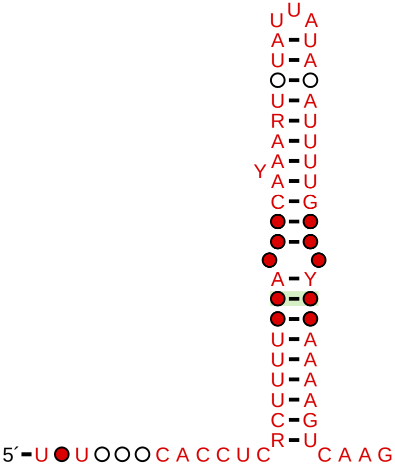

HMP2_6927_1

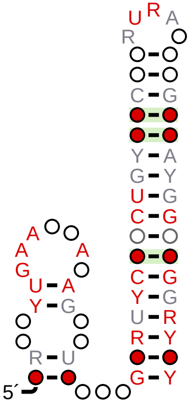

HMP2_6930_1

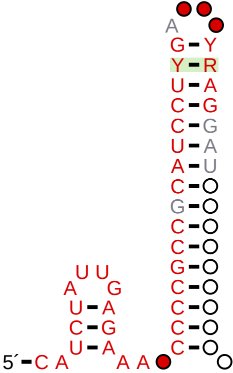

HMP2_6936_1

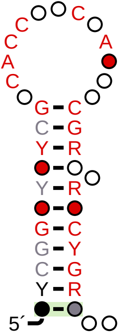

HMP2_694_1

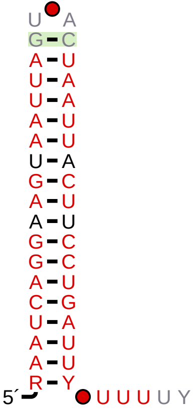

HMP2_6953_1

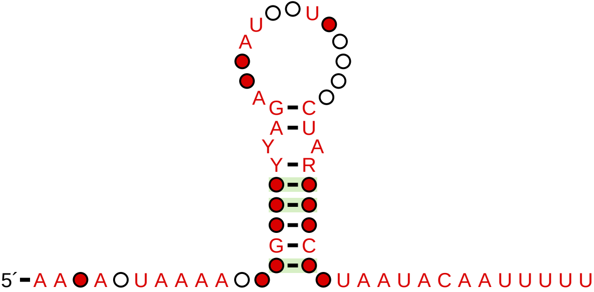

HMP2_6955_1

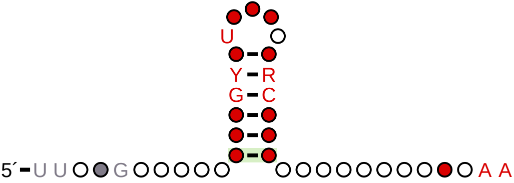

HMP2_6957_1

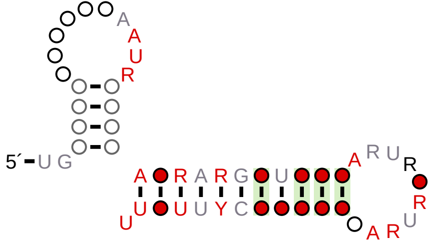

HMP2_6966_1

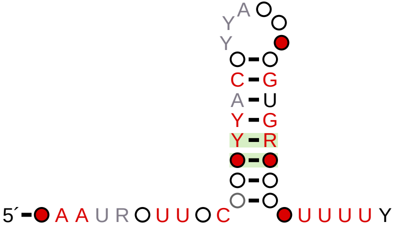

HMP2_6967_1

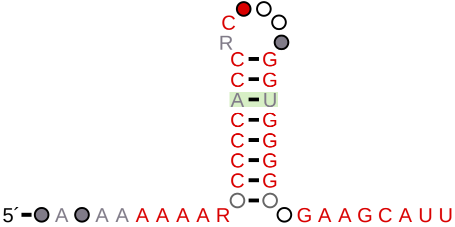

HMP2_6985_1

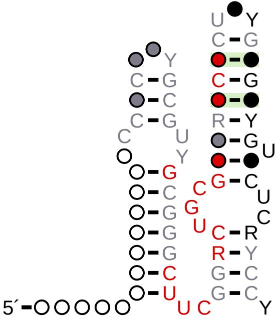

HMP2_6988_1

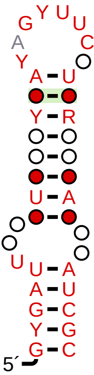

HMP2_699_1

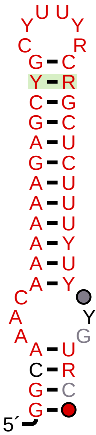

HMP2_6992_1

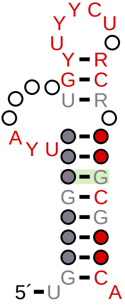

HMP2_6999_1

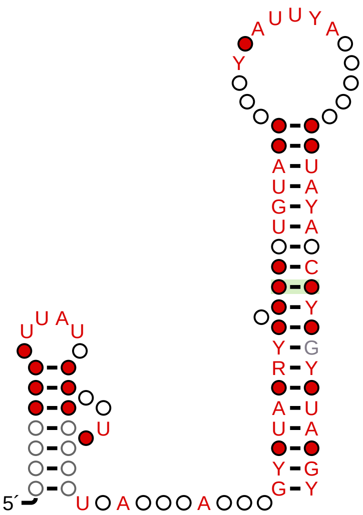

HMP2_7003_1

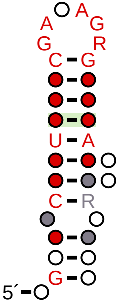

HMP2_7016_1

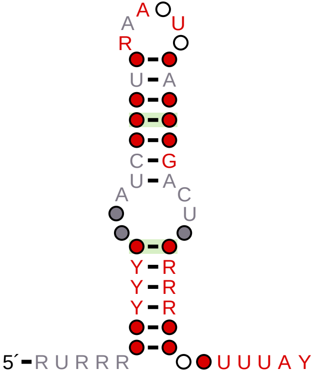

HMP2_7017_1

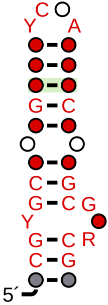

HMP2_7020_1

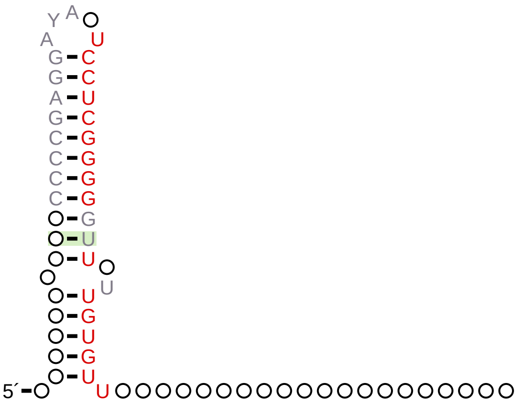

HMP2_7038_1

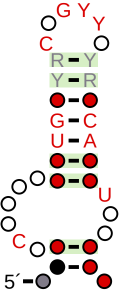

HMP2_7042_1

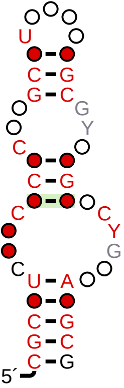

HMP2_7064_1

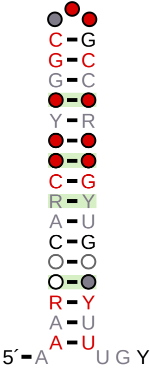

HMP2_7067_1

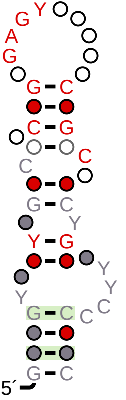

HMP2_7072_1

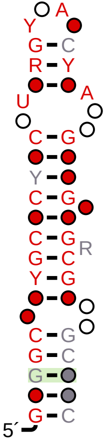

HMP2_7082_1

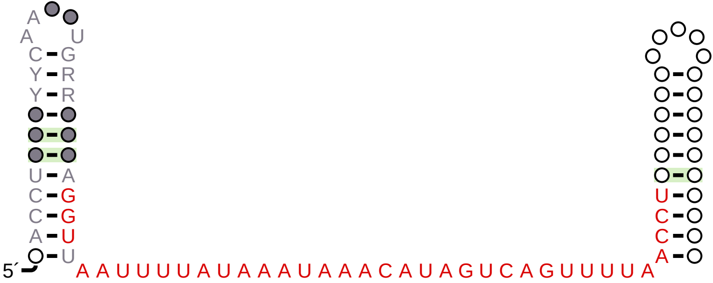

A
A U
C G
Y R
Y R
U A
C G
C G
A U
U
5´
A A U U U U A U A A A U A A A C A U A G U C A G U U U U A
U
C
C
A

HMP2_7092_1

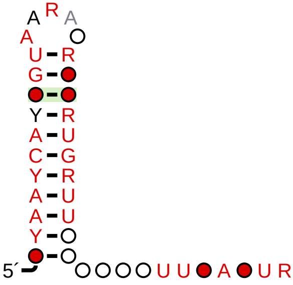

HMP2_7093_1

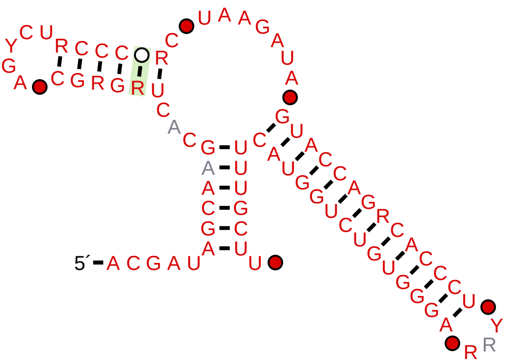

HMP2_71_1

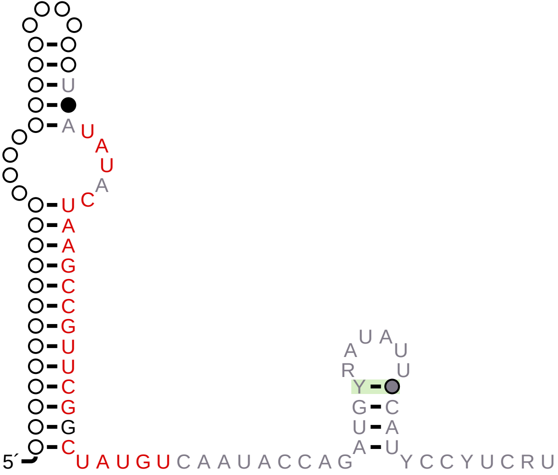

HMP2_710_1

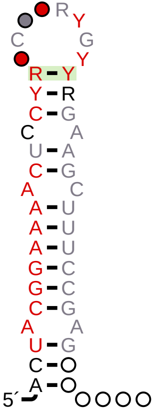

HMP2_7101_1

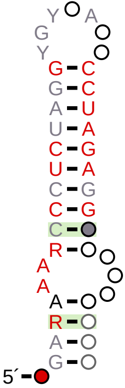

HMP2_7114_1

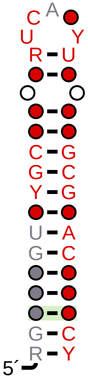

HMP2_712_1

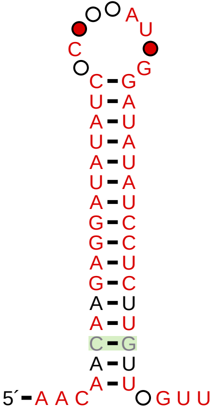

HMP2_7121_1

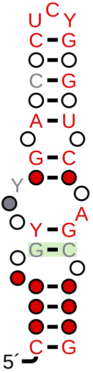

HMP2_7127_1

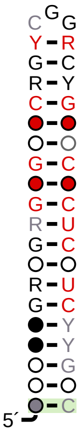

HMP2_7133_1

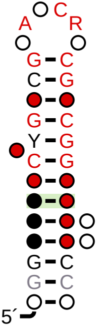

HMP2_7157_1

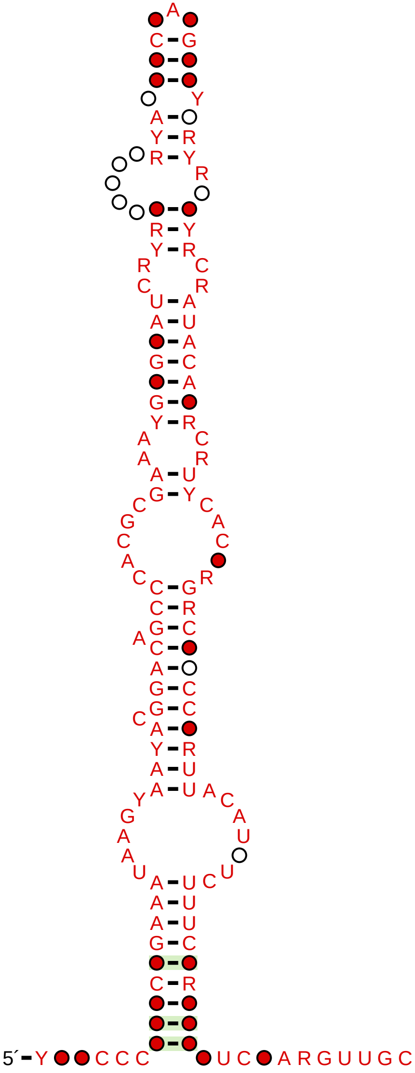

HMP2_7163_1

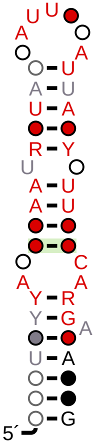

HMP2_7170_1

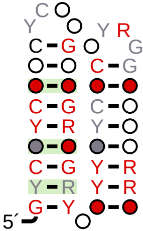

HMP2_7174_1

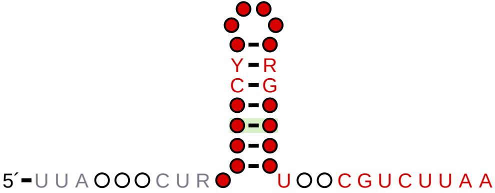

HMP2_7176_1

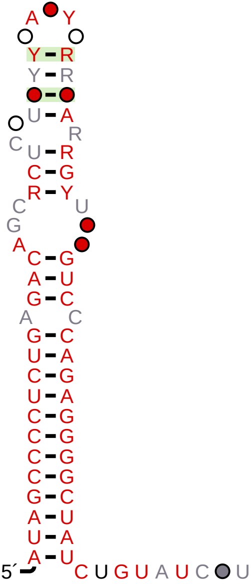

HMP2_719_1

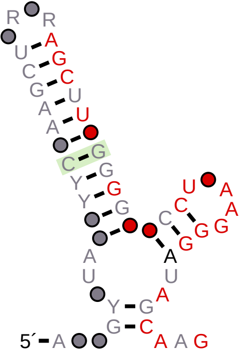

HMP2_7202_1

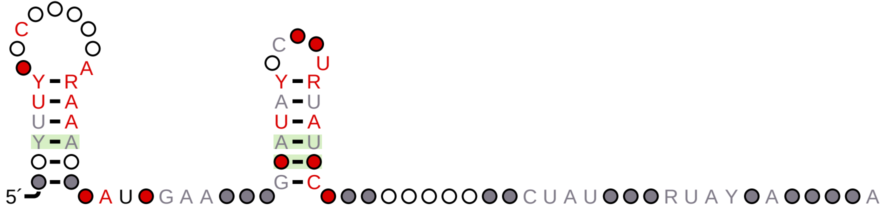

HMP2_7207_1

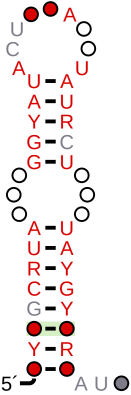

HMP2_721_1

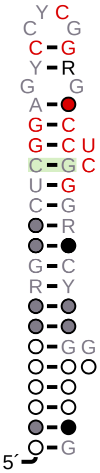

HMP2_722_1

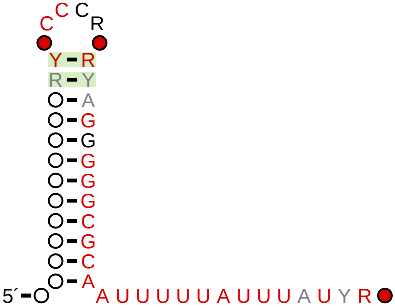

HMP2_7223_1

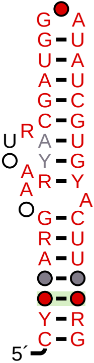

HMP2_7227_1

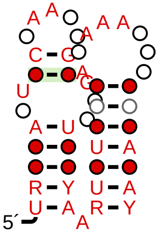

HMP2_7228_1

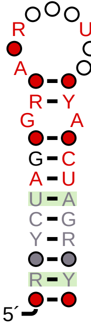

HMP2_7229_1

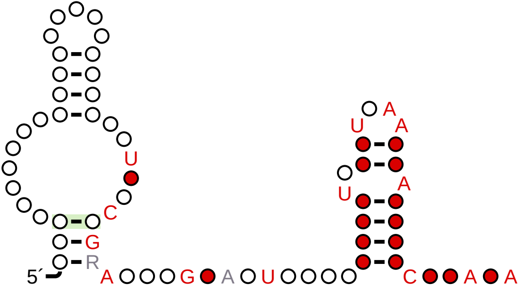

HMP2_723_1

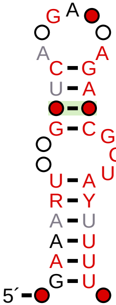

HMP2_7231_1

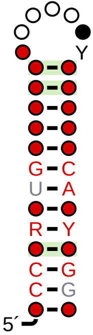

HMP2_7237_1

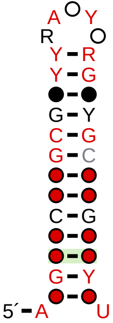

HMP2_7246_1

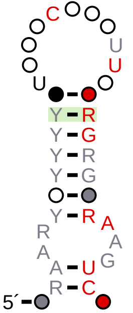

HMP2_7249_1

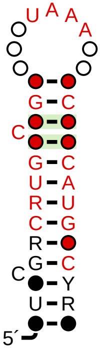

HMP2_7250_1

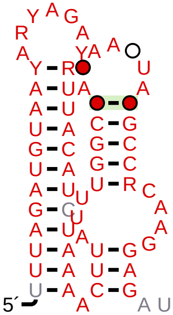

HMP2_7263_1

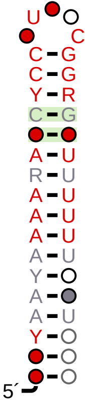

HMP2_7265_1

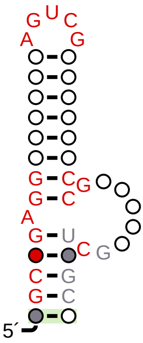

HMP2_7269_1

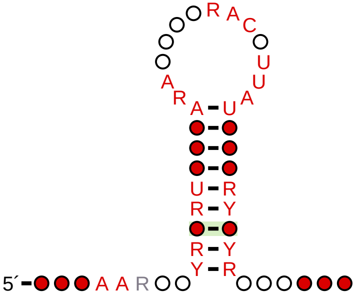

HMP2_7275_1

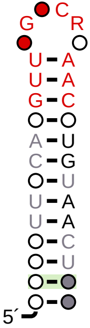

HMP2_7277_1

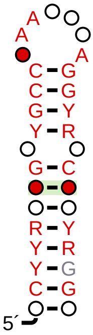

HMP2_728_1

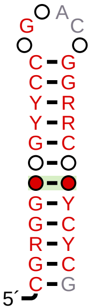

HMP2_7289_1

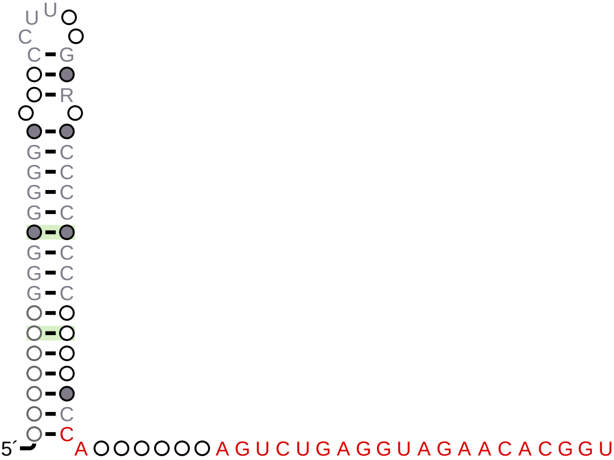

HMP2_7292_1

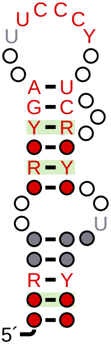

HMP2_7295_1

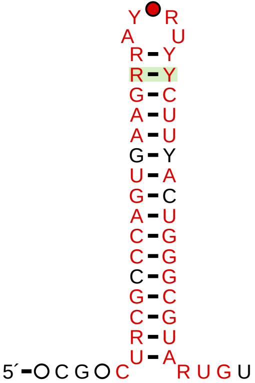

HMP2_7302_1

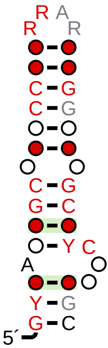

HMP2_7306_1

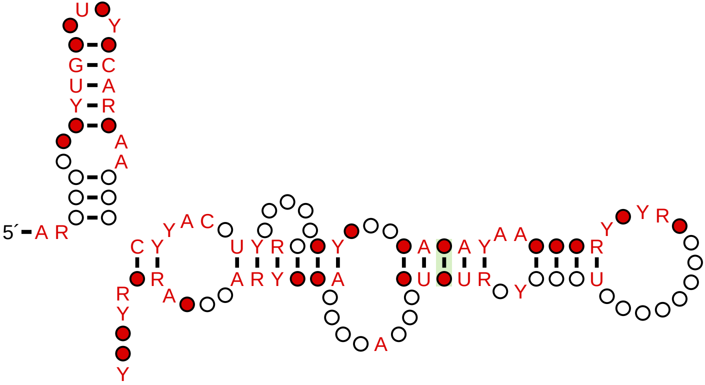

HMP2_731_1

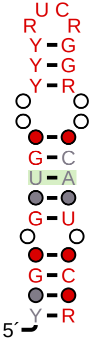

HMP2_7312_1

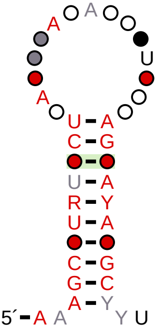

HMP2_7313_1

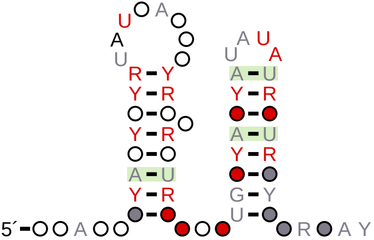

HMP2_7316_1

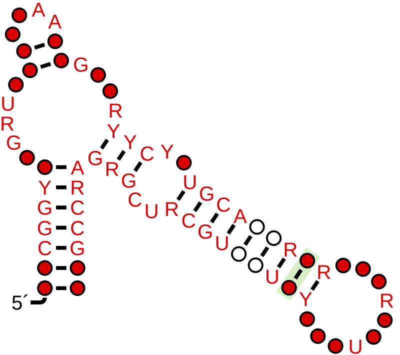

HMP2_7320_1

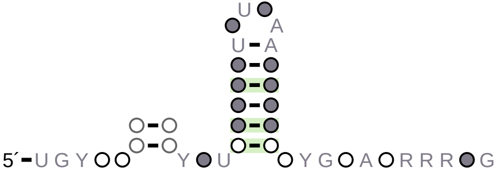

HMP2_7323_1

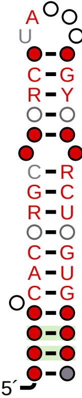

HMP2_7330_1

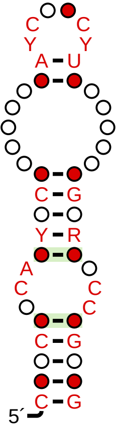

HMP2_7331_1

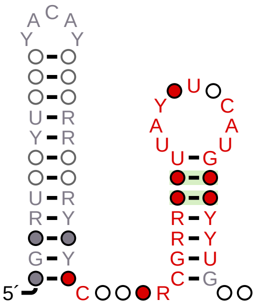

HMP2_7336_1

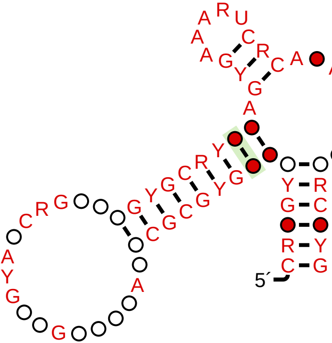

HMP2_7338_1

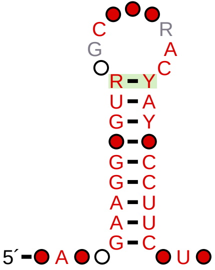

HMP2_734_1

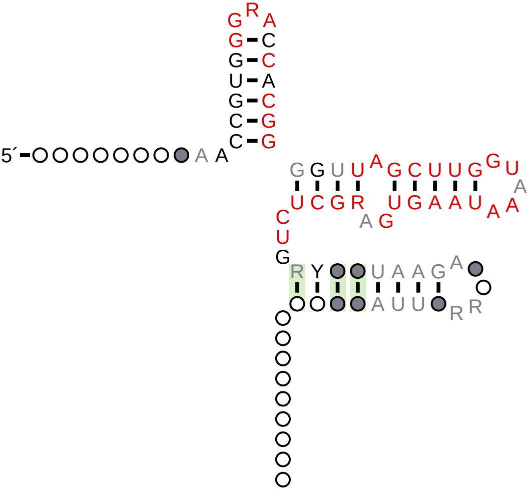

HMP2_7341_1

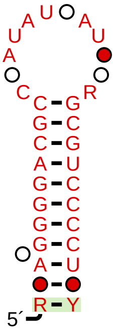

HMP2_7342_1

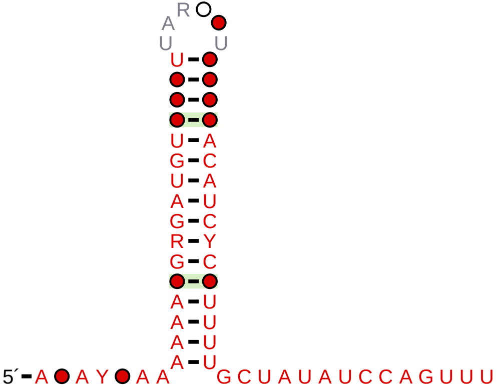

HMP2_7349_1

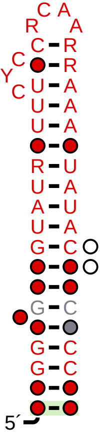

HMP2_7351_1

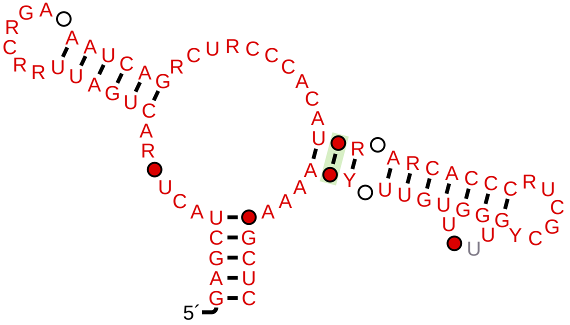

HMP2_7355_1

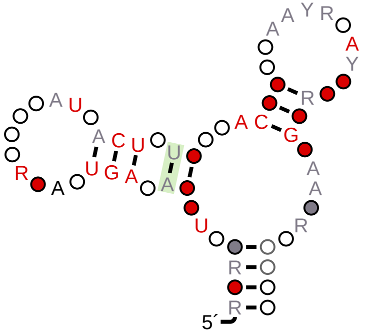

HMP2_7357_1

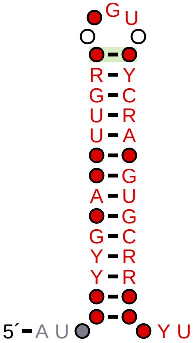

HMP2_7364_1

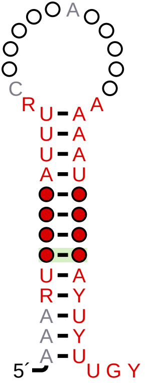

HMP2_7373_1

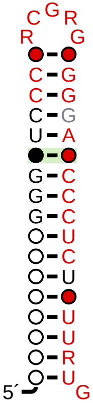

HMP2_7374_1

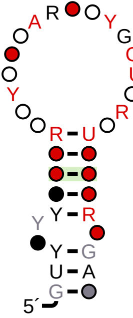

HMP2_7380_1

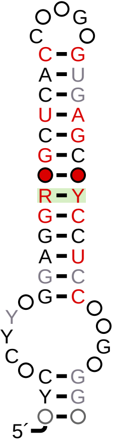

HMP2_739_1

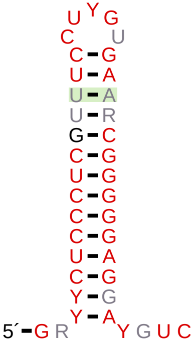

HMP2_7390_1

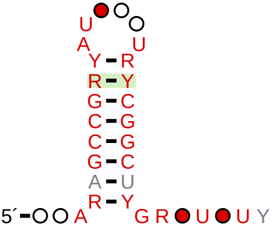

HMP2_7397_1

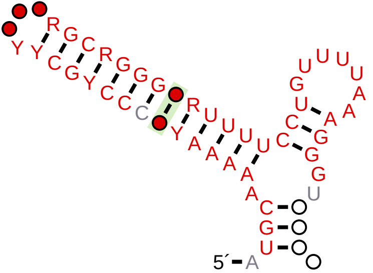

HMP2_74_1

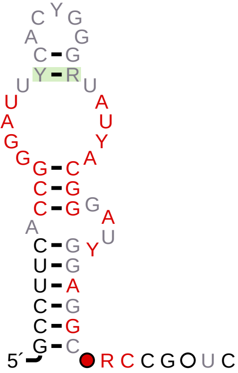

HMP2_7408_1

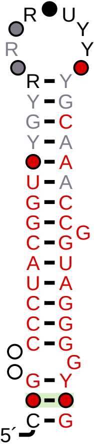

HMP2_7415_1

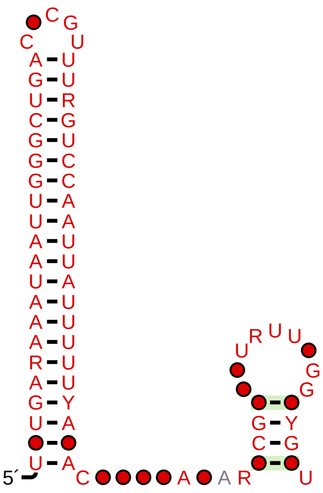

HMP2_7417_1

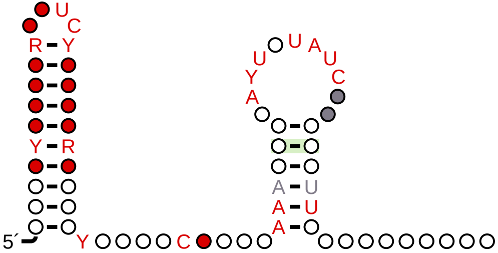

HMP2_742_1

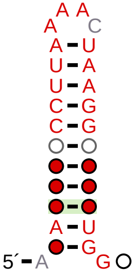

HMP2_7420_1

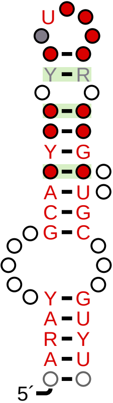

HMP2_7422_1

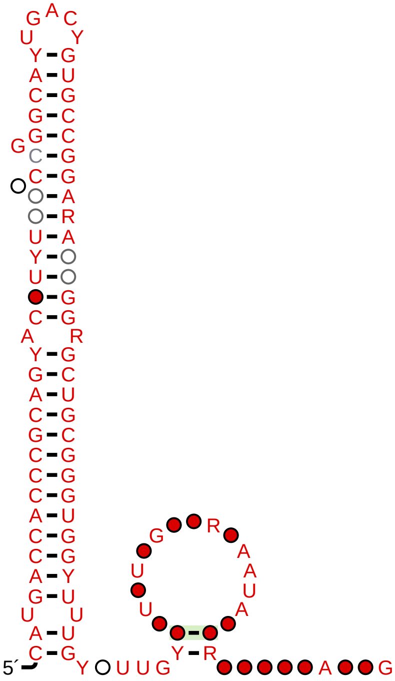

HMP2_7425_1

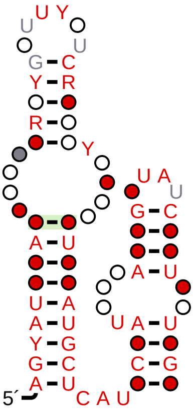

HMP2_7435_1

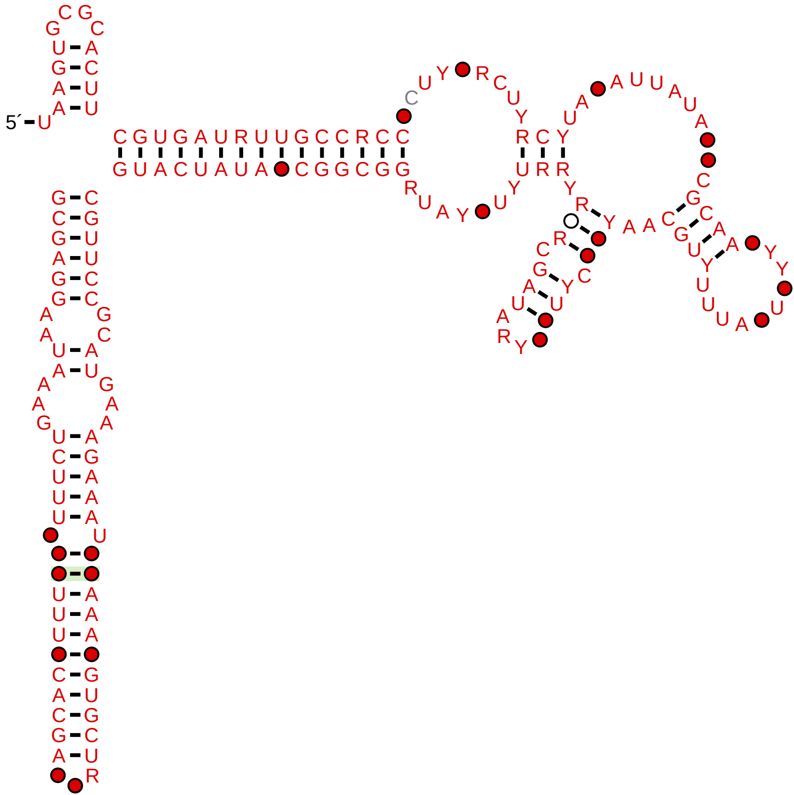

HMP2_7443_1

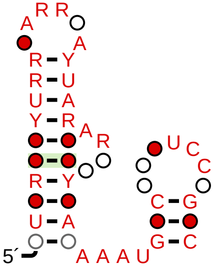

HMP2_7457_1

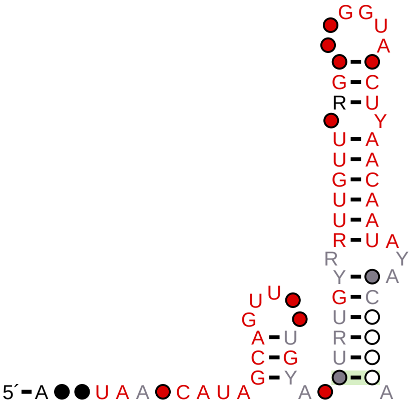

HMP2_7458_1

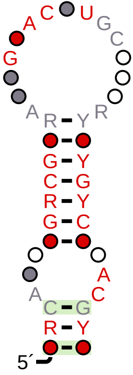

HMP2_7461_1

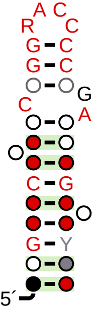

HMP2_7464_1

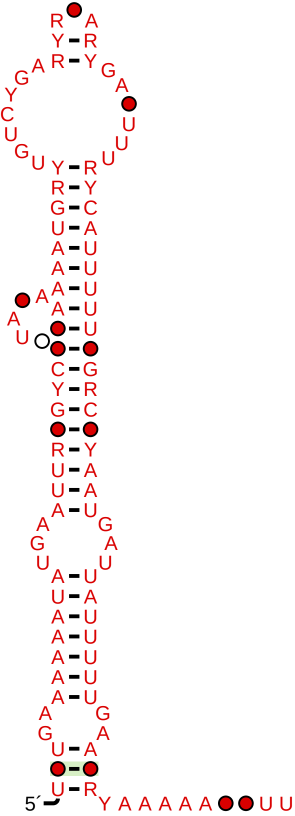

HMP2_7466_1

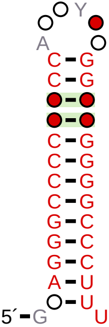

HMP2_7474_1

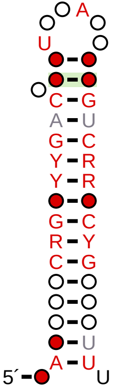

HMP2_7477_1

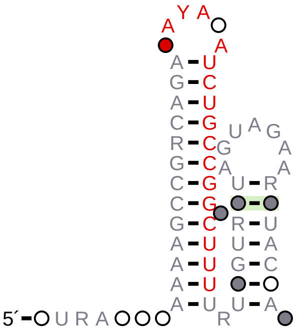

HMP2_7478_1

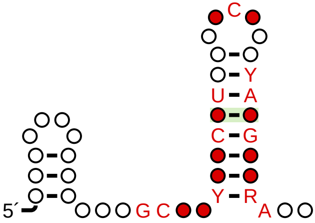

HMP2_7484_1

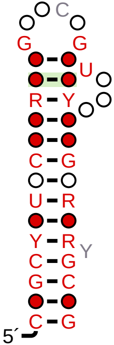

HMP2_749_1

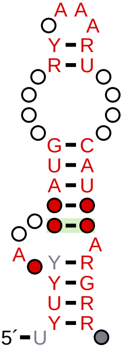

HMP2_7495_1

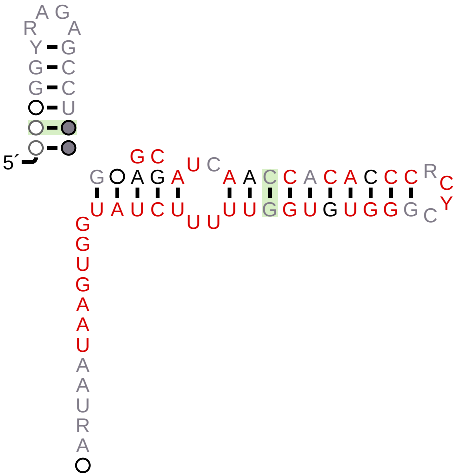

HMP2_7497_1

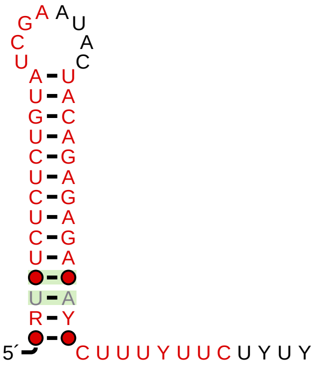

HMP2_7501_1

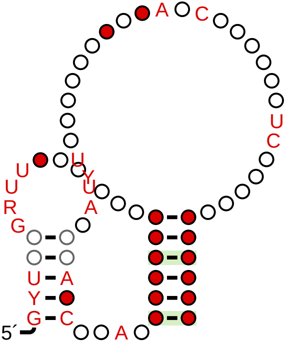

HMP2_751_1

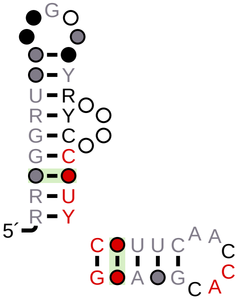

HMP2_7510_1

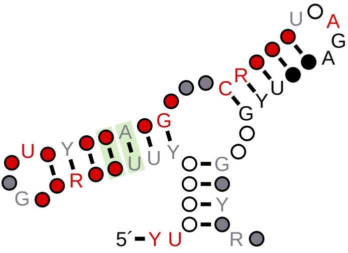

HMP2_7512_1

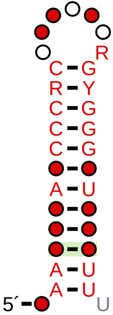

HMP2_7519_1

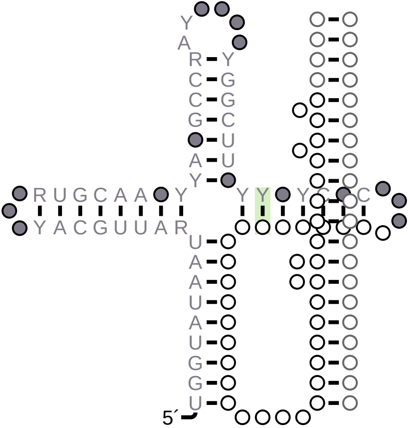

HMP2_7522_1

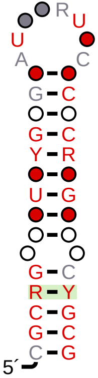

HMP2_7528_1

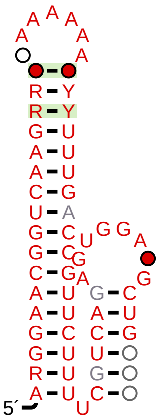

HMP2_7529_1

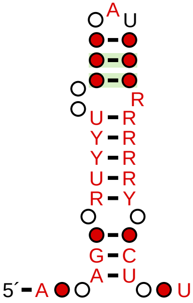

HMP2_753_1

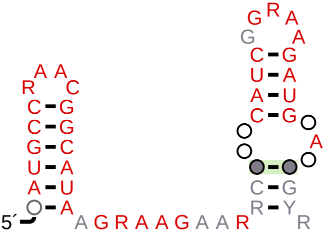

HMP2_7530_1

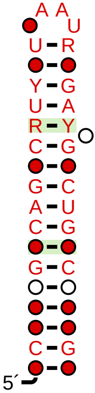

HMP2_7535_1

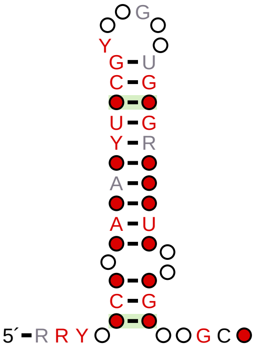

HMP2_7542_1

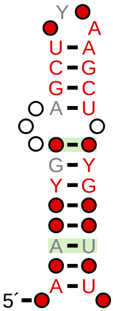

HMP2_7547_1

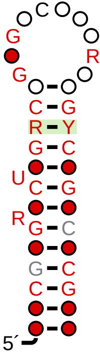

HMP2_7549_1

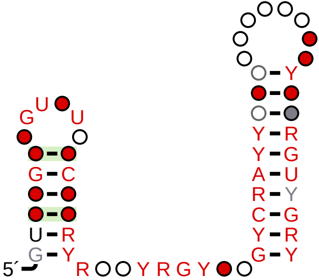

HMP2_7555_1

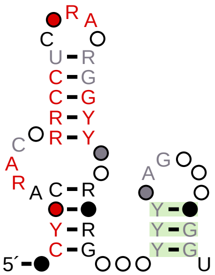

HMP2_7569_1

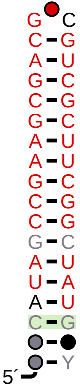

HMP2_7573_1

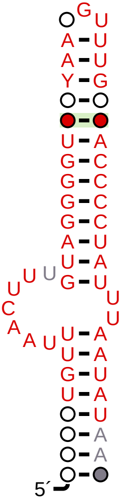

HMP2_7577_1

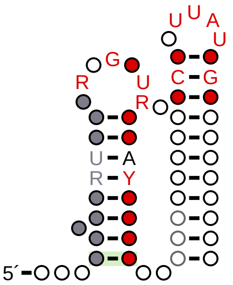

HMP2_758_1

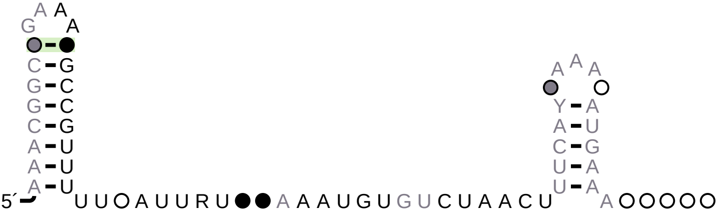

A A
G A
C G
G C
G C
C G
A U
A U
A U
5´
U U O A U U R U ● ● A A A U G U G U C U A A C U
A A A
Y A
A U
C G
U A
U A
A O O O O O

HMP2_7584_1

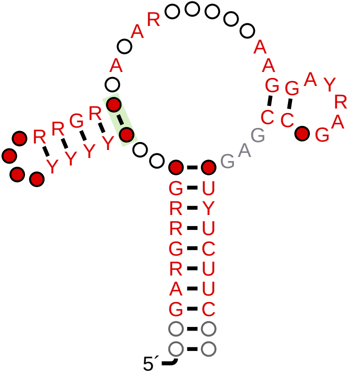

HMP2_7595_1

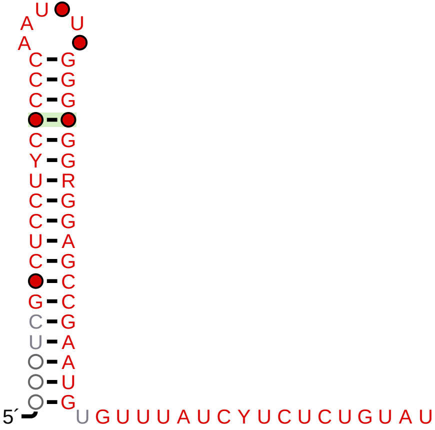

U
A U
A
C G
C G
C G
C G
Y G
U R
C G
C G
U A
C G
C
G C
C G
U A
A
U
G
5´
U G U U U A U C Y U C U C U G U A U

HMP2_7605_1

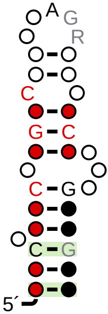

HMP2_7627_1

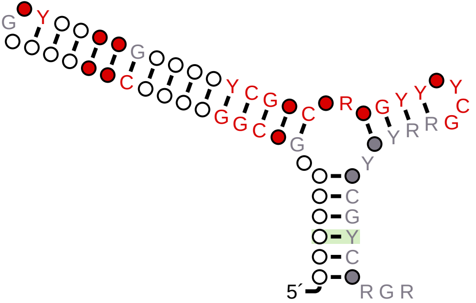

HMP2_7628_1

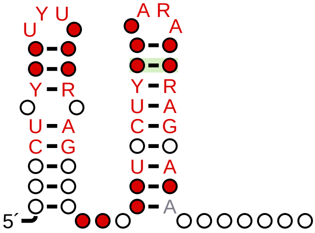

HMP2_7632_1

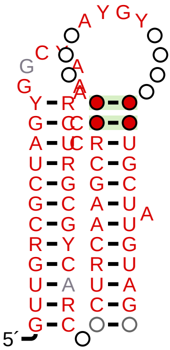

HMP2_7640_1

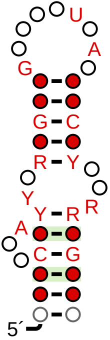

HMP2_7642_1

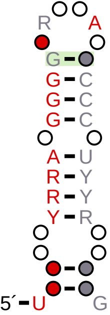

HMP2_7643_1

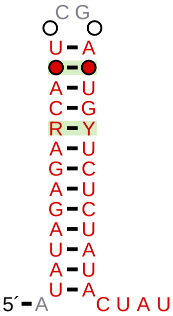

HMP2_7653_1

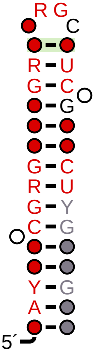

HMP2_7659_1

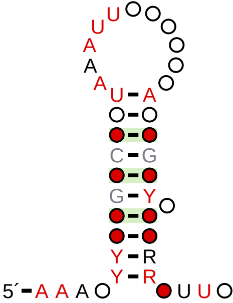

HMP2_7660_1

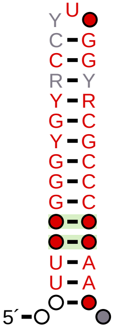

HMP2_7663_1

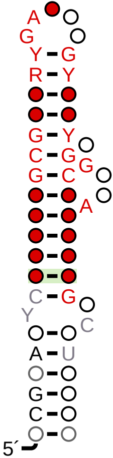

HMP2_7667_1

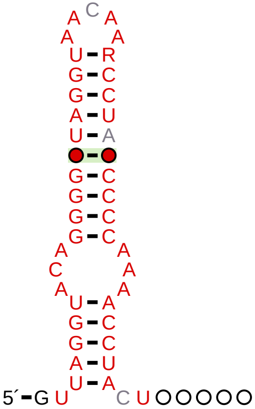

HMP2_7669_1

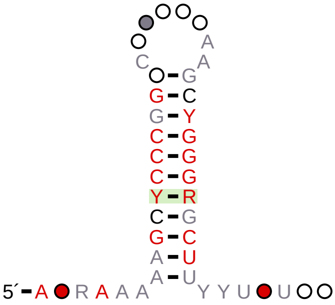

HMP2_767_1

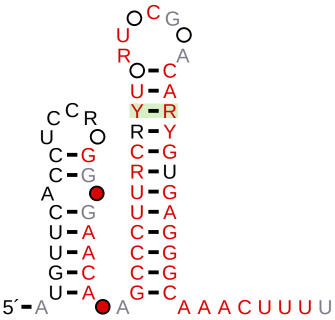

HMP2_7674_1

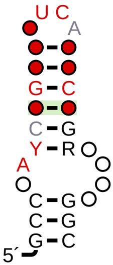

HMP2_7676_1

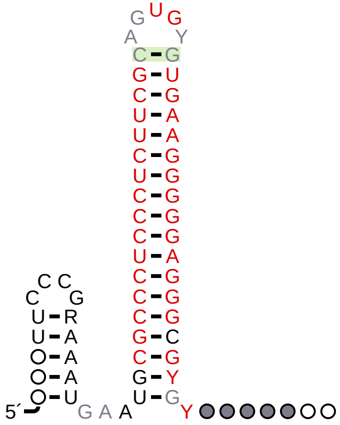

G U G
A Y
C G
G U
C G
U A
U A
C G
U G
C G
C G
C G
U A
C G
C G
C G
G C
C G
G Y
U G
C C
C G
U R
U A
A
A
U
5´
G A A
Y

HMP2_7687_1

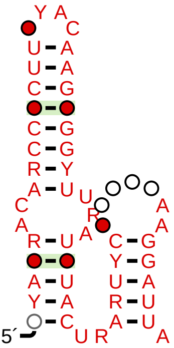

HMP2_7697_1

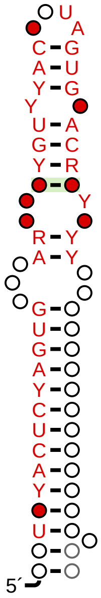

HMP2_771_1

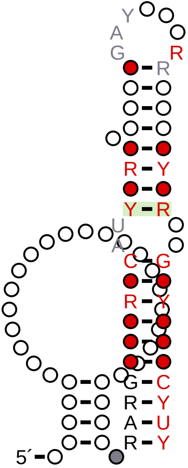

HMP2_7710_1

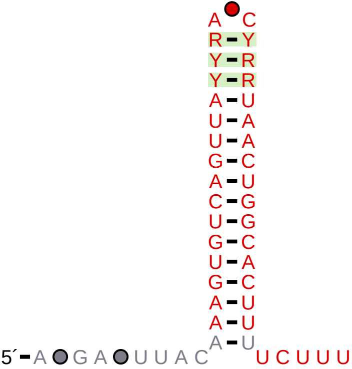

HMP2_7712_1

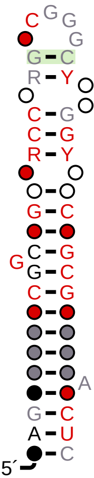

HMP2_7714_1

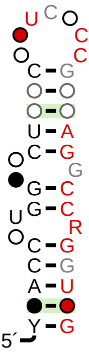

HMP2_7718_1

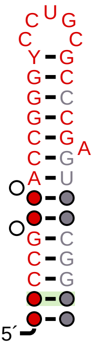

HMP2_7725_1

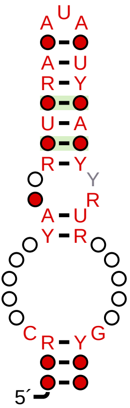

HMP2_7730_1

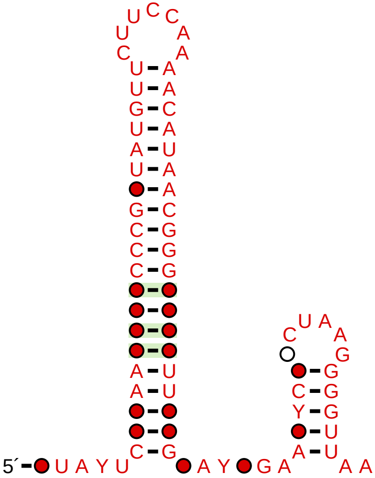
5´
U A Y U
A Y
G A
A A
CUUCCAA
U-A
U-A
G-C
U-A
A-U
U-A
G-C
C-G
C-G
C-G
A-U
A-U
C-G
CUAAG
G
C-G
Y-G
U
A-U

HMP2_7733_1

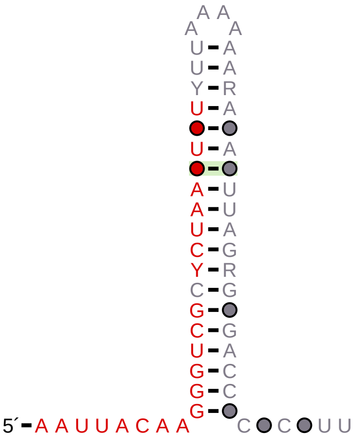

HMP2_7743_1

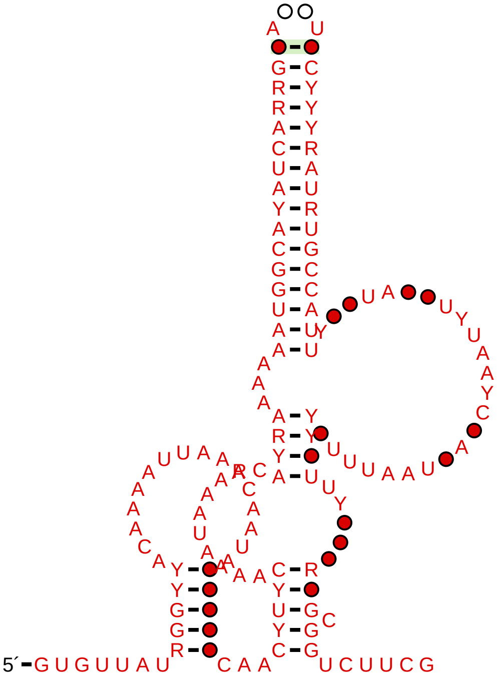

HMP2_7747_1

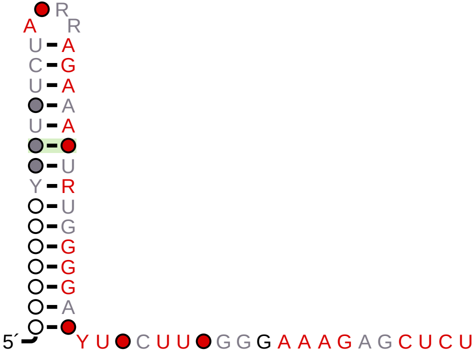

HMP2_7748_1

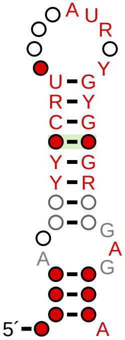

HMP2_7753_1

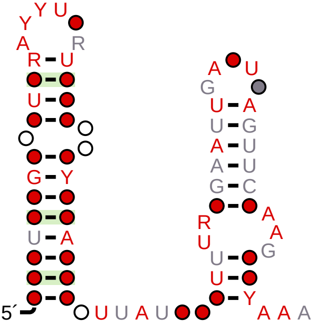

HMP2_7756_1

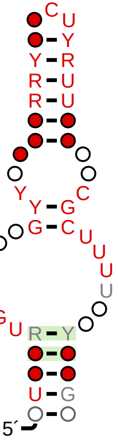

HMP2_7757_1

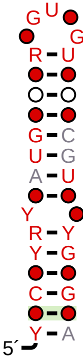

HMP2_7766_1

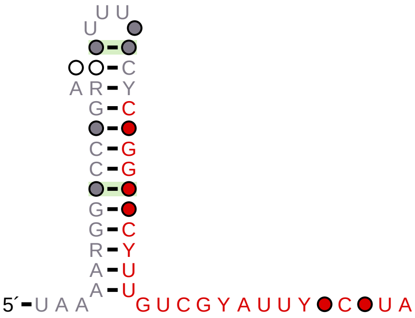

HMP2_777_1

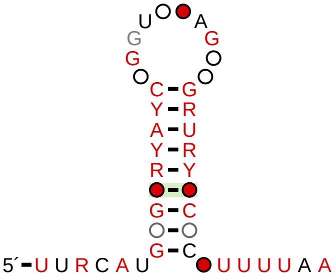

HMP2_7771_1

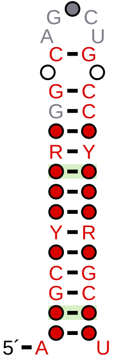

HMP2_7775_1

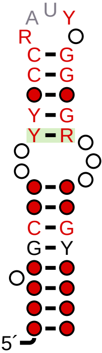

HMP2_7776_1

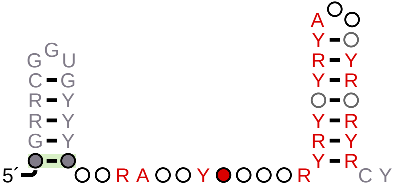

HMP2_7777_1

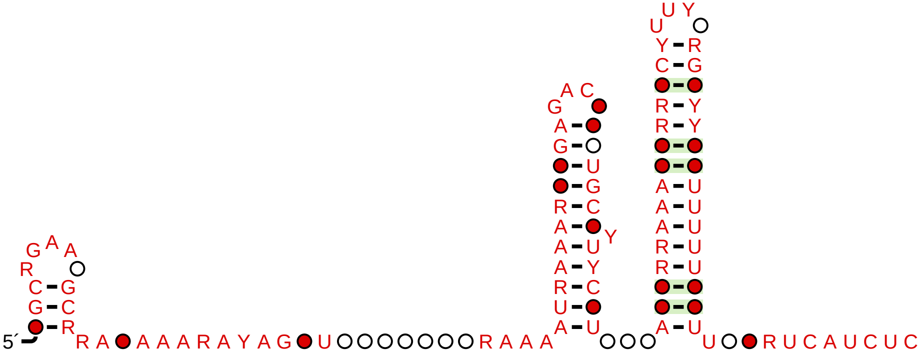

HMP2_778_1

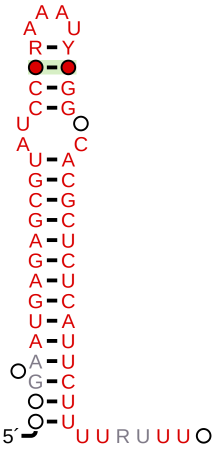

HMP2_7790_1

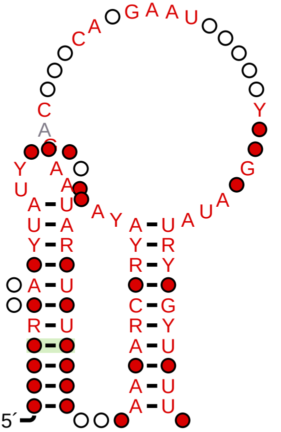

HMP2_7794_1

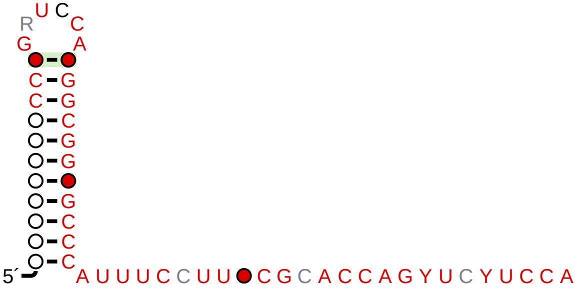

HMP2_7797_1

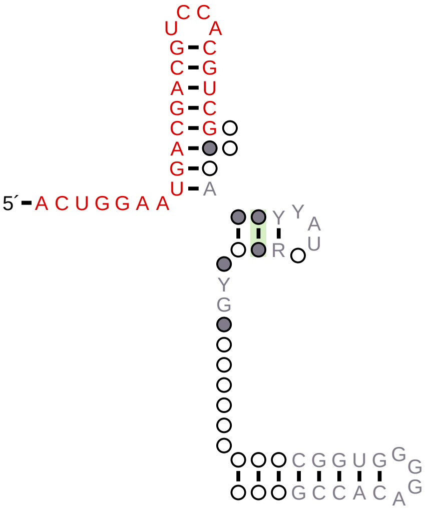

HMP2_7798_1

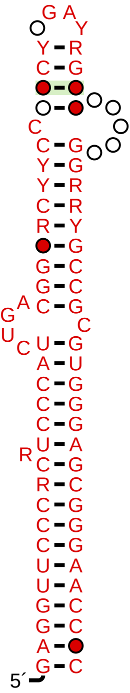

HMP2_7799_1

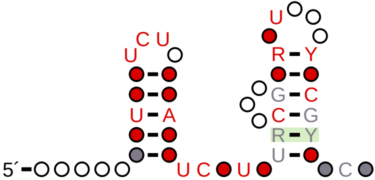

HMP2_7802_1

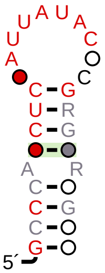

HMP2_7805_1

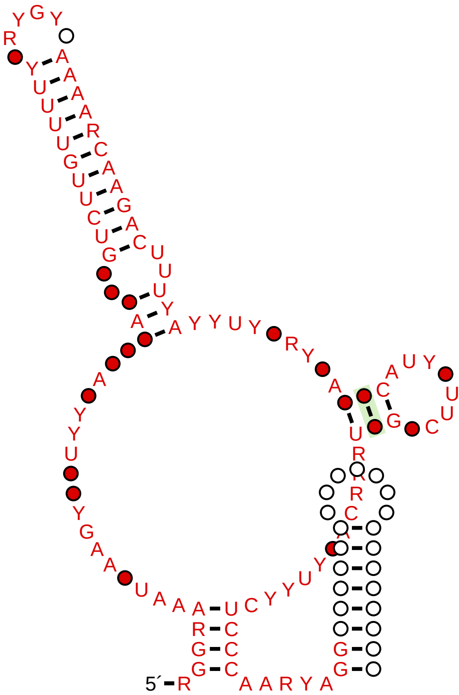

HMP2_781_1

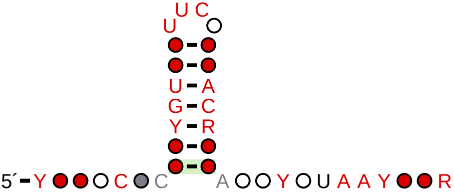

HMP2_7813_1

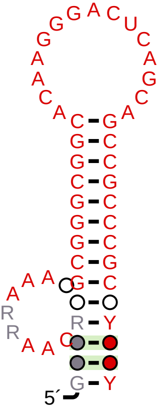

HMP2_7816_1

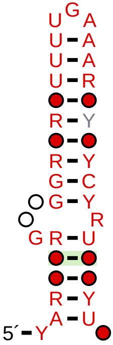

HMP2_7819_1

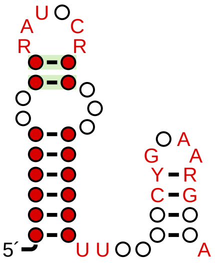

HMP2_7825_1

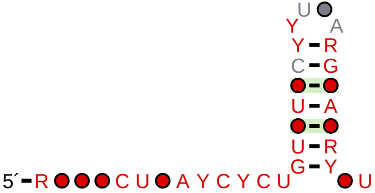

HMP2_7831_1

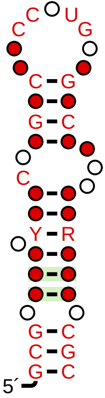

HMP2_7835_1

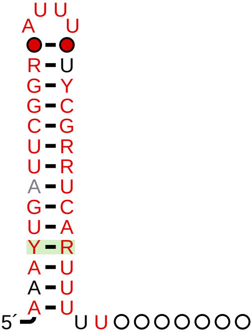

HMP2_7838_1

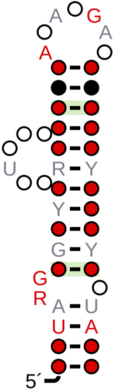

HMP2_7842_1

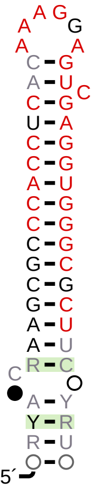

HMP2_7845_1

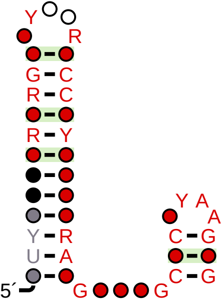

HMP2_7848_1

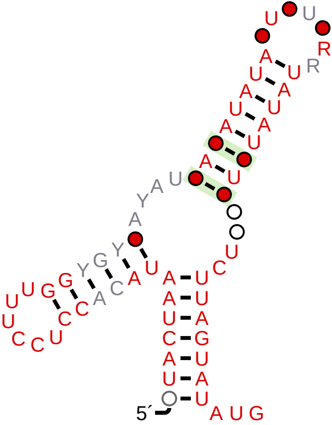

HMP2_7849_1

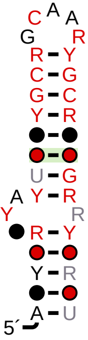

HMP2_7859_1

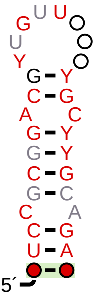

HMP2_786_1

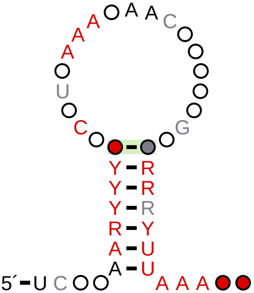

HMP2_7866_1

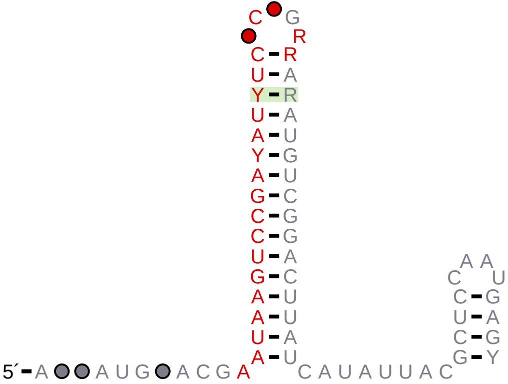

HMP2_787_1

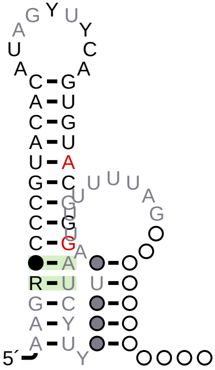

HMP2_7871_1

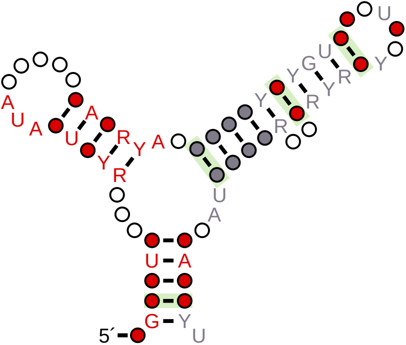

HMP2_7874_1

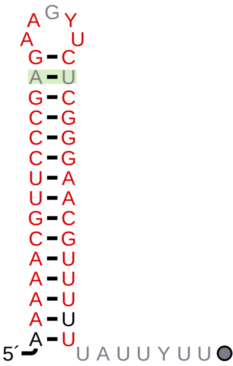

HMP2_7883_1

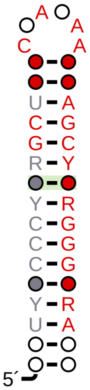

HMP2_7884_1

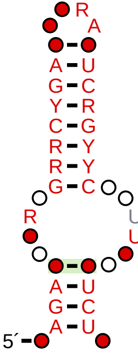

HMP2_7886_1

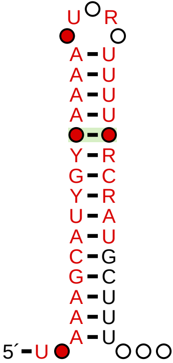

HMP2_7888_1

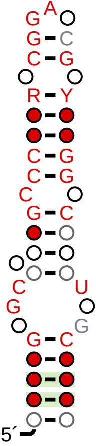

HMP2_7893_1

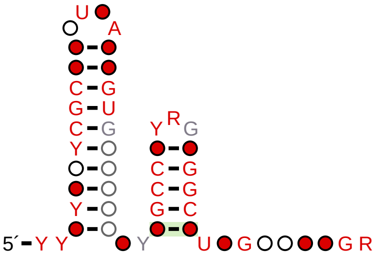

HMP2_7894_1

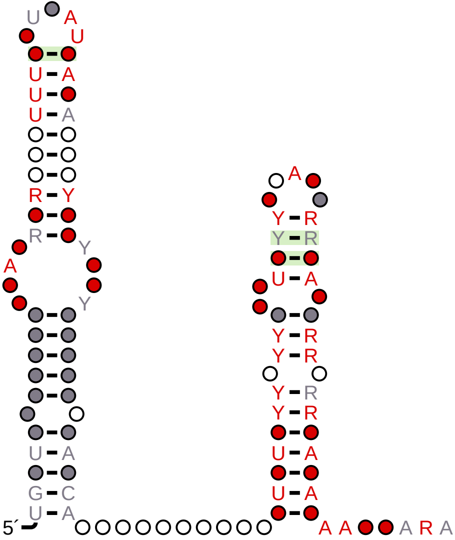

HMP2_7917_1

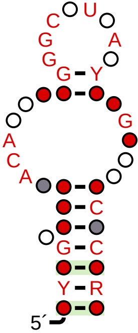

HMP2_7918_1

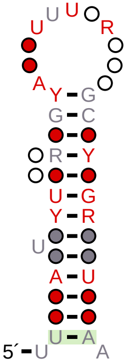

HMP2_7919_1

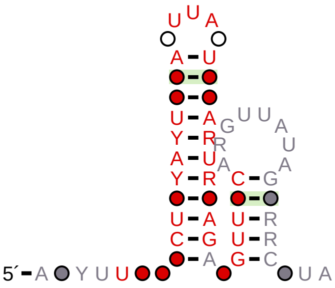

HMP2_792_1

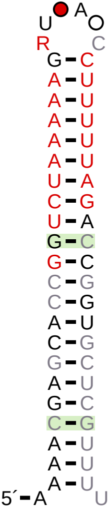

HMP2_7926_1

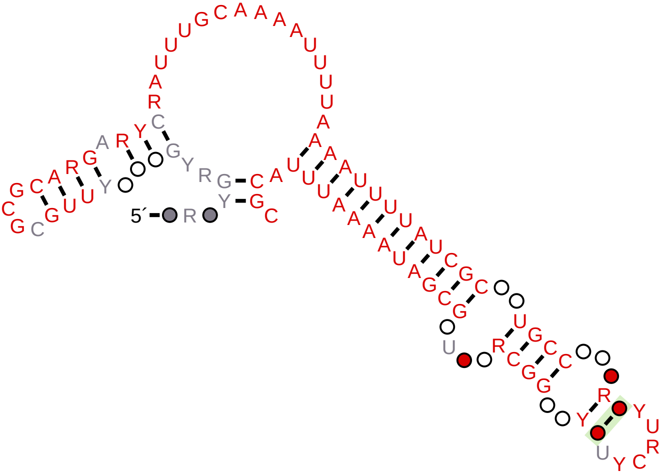

HMP2_7929_1

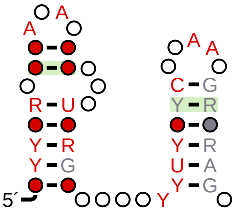

HMP2_7937_1

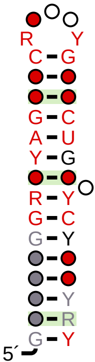

HMP2_7943_1

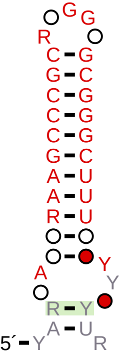

HMP2_7950_1

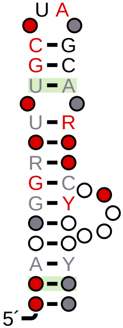

HMP2_7952_1

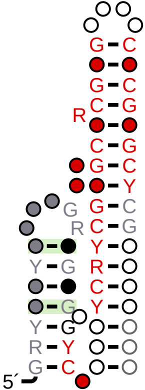

HMP2_7957_1

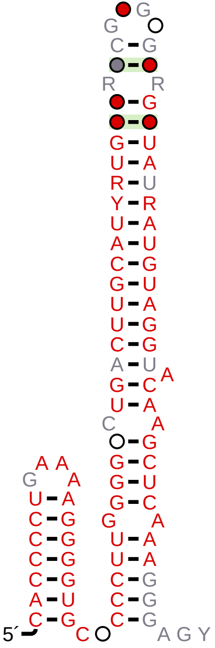

HMP2_7962_1

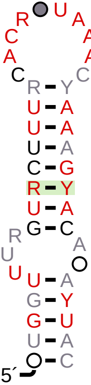

HMP2_7964_1

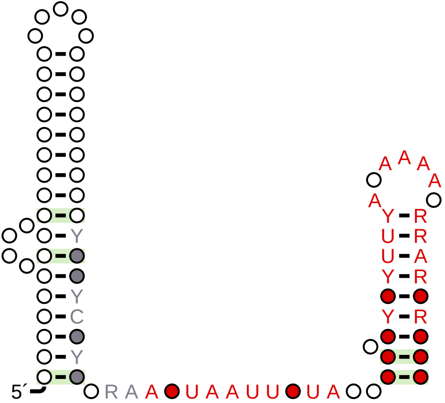

HMP2_7975_1

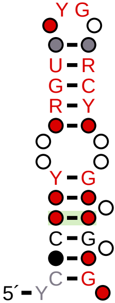

HMP2_7976_1

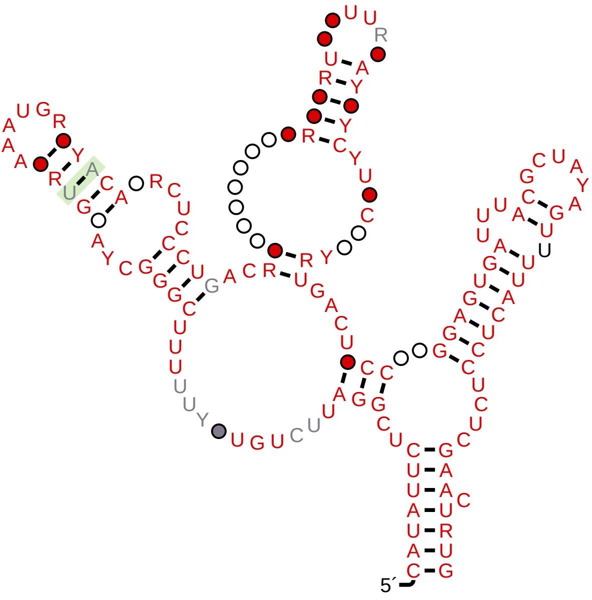

HMP2_7979_1

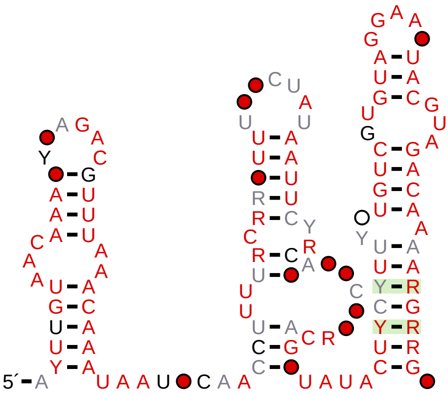

HMP2_7980_1

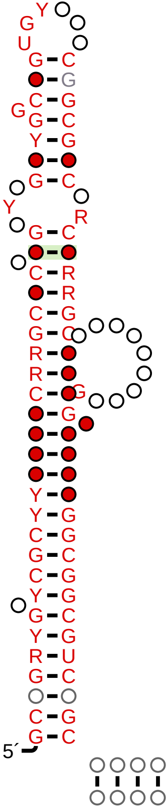

HMP2_7982_1

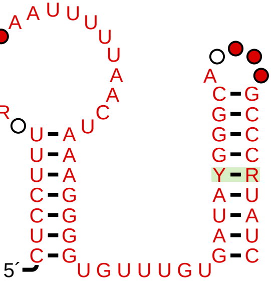

HMP2_8006_1

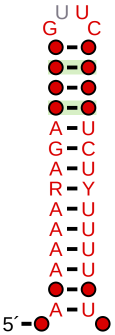

HMP2_8009_1

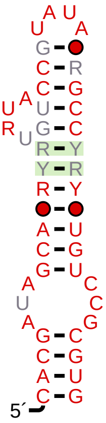

HMP2_8015_1

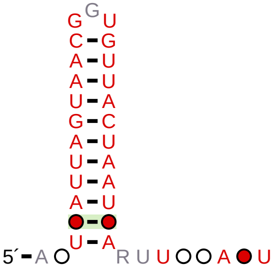

HMP2_8026_1

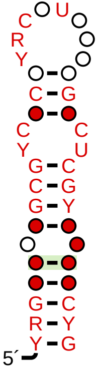

HMP2_8028_1

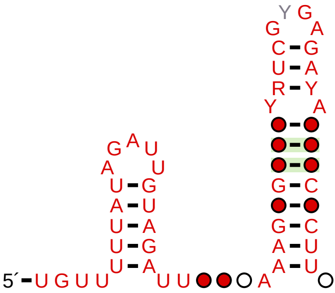

HMP2_8036_1

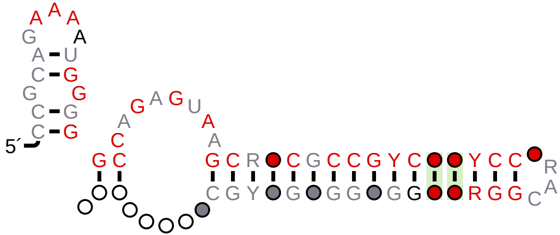

HMP2_8041_1

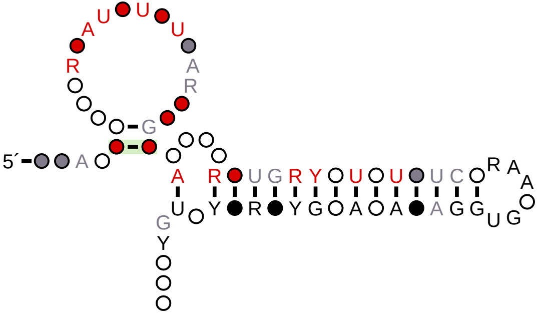

HMP2_8042_1

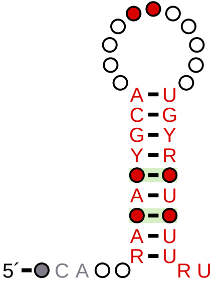

HMP2_8046_1

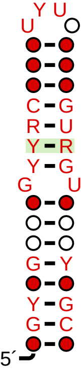

HMP2_8048_1

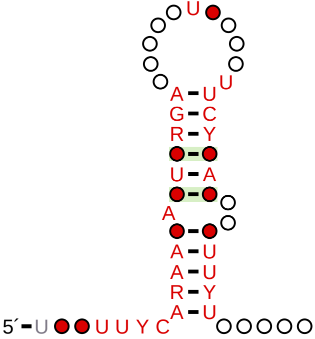

HMP2_8054_1

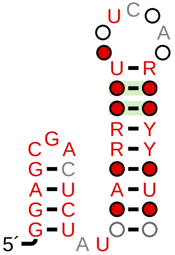

HMP2_8058_1

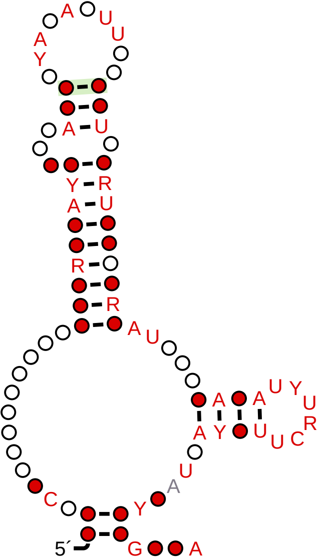

HMP2_8060_1

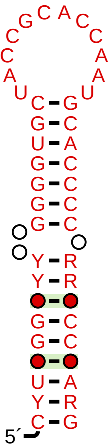

HMP2_8067_1

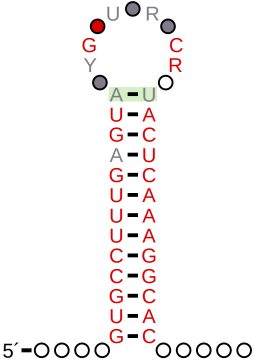

HMP2_8069_1

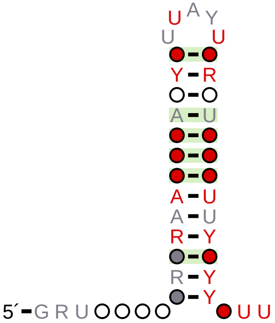

HMP2_8073_1

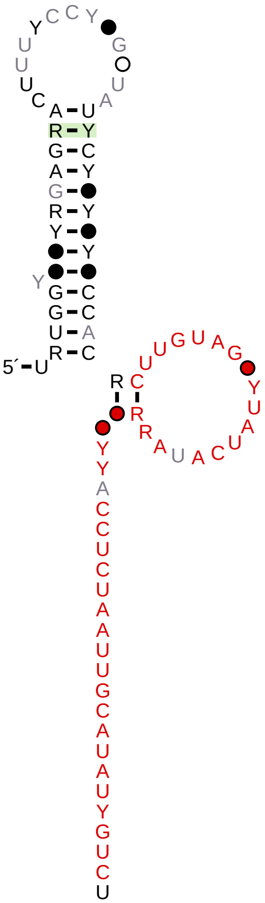

HMP2_8074_1

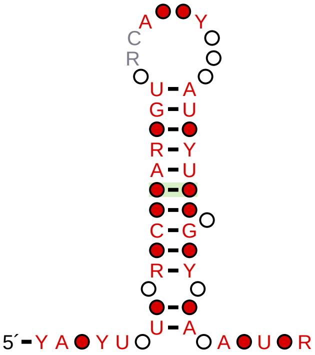

HMP2_8079_1

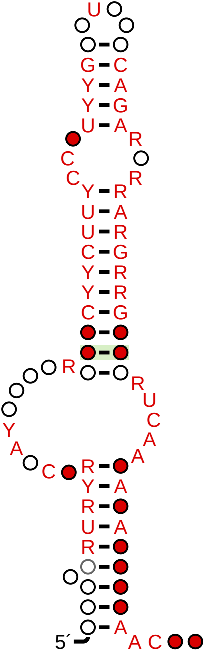

HMP2_808_1

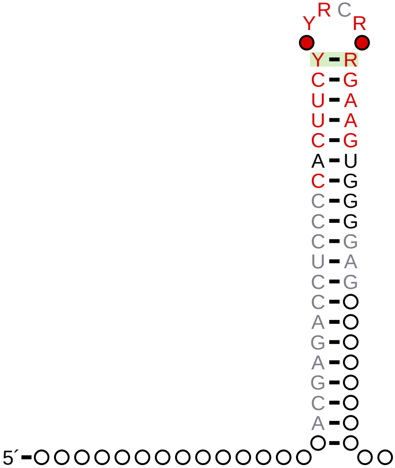

HMP2_8087_1

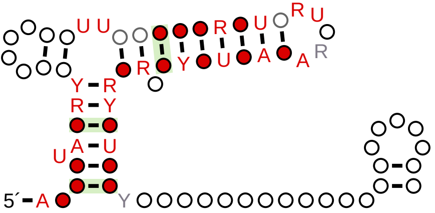

HMP2_8094_1

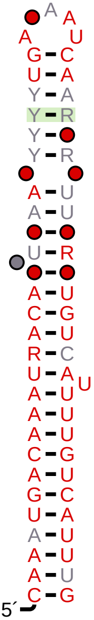

HMP2_8095_1

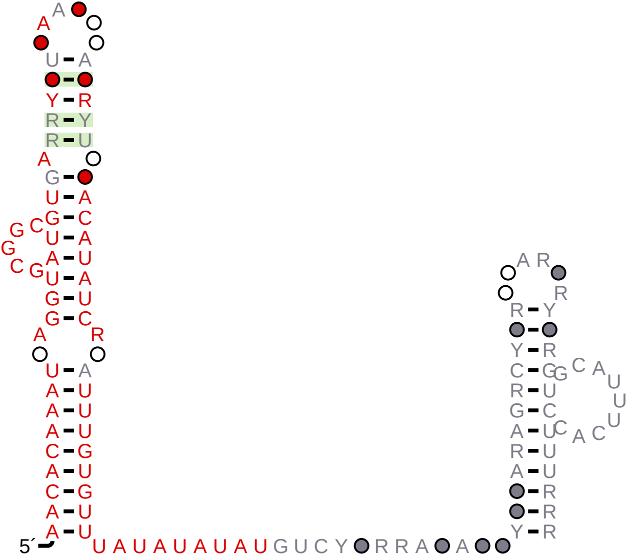

HMP2_8096_1

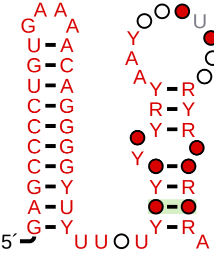

HMP2_8097_1

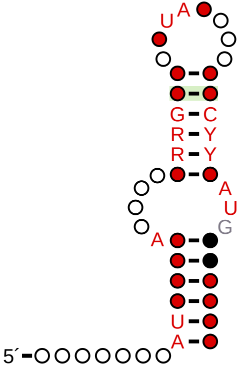

HMP2_8109_1

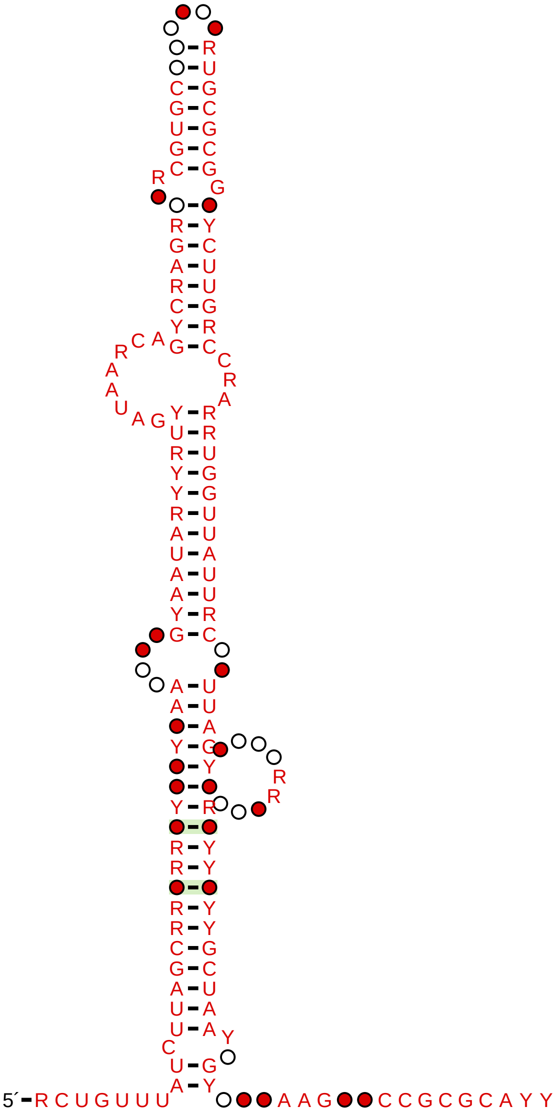

HMP2_8110_1

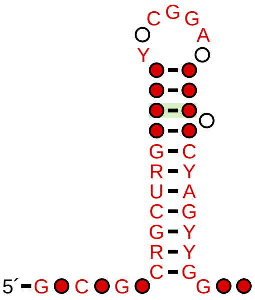

HMP2_8111_1

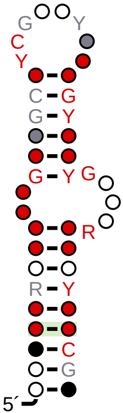

HMP2_8114_1

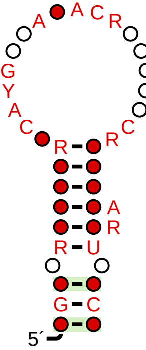

HMP2_8115_1

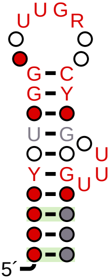

HMP2_8116_1

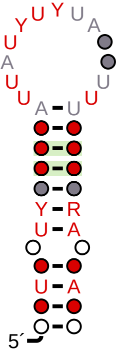

HMP2_8121_1

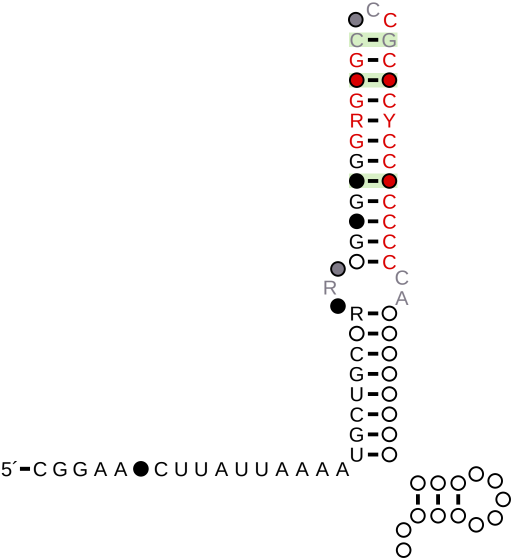

HMP2_8122_1

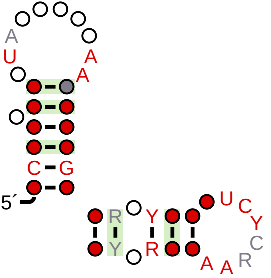

HMP2_8123_1

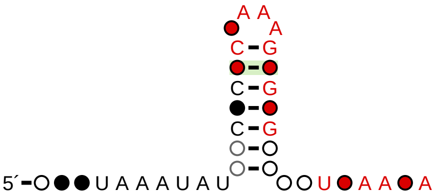

HMP2_8134_1

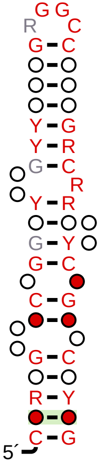

HMP2_8135_1

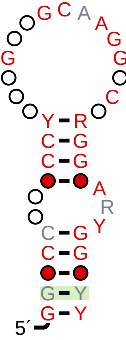

HMP2_8139_1

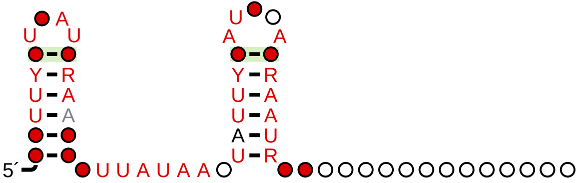

HMP2_8140_1

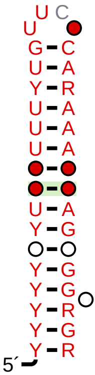

HMP2_8141_1

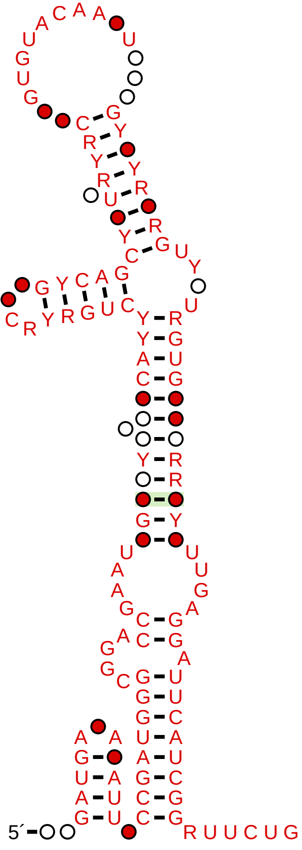

HMP2_815_1

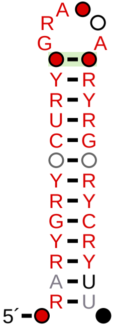

HMP2_8154_1

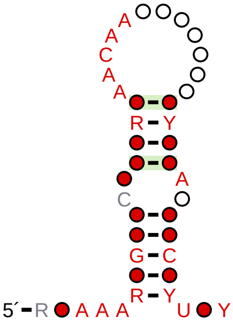

HMP2_8155_1

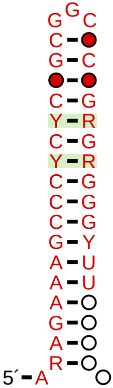

HMP2_8157_1

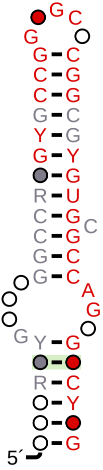

HMP2_8165_1

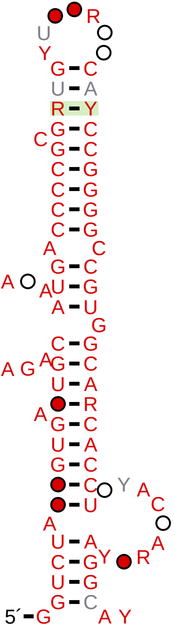

HMP2_82_1

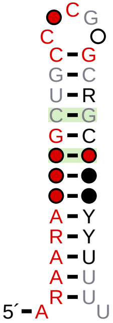

HMP2_821_1

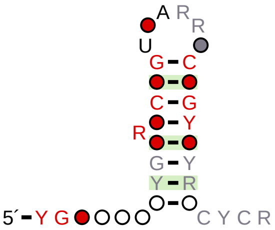

HMP2_8215_1

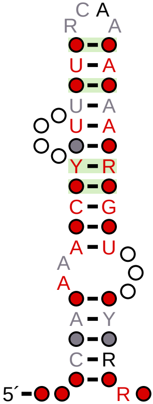

HMP2_8218_1

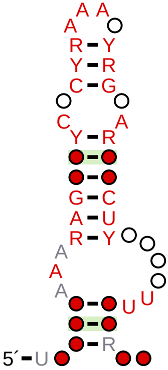

HMP2_822_1

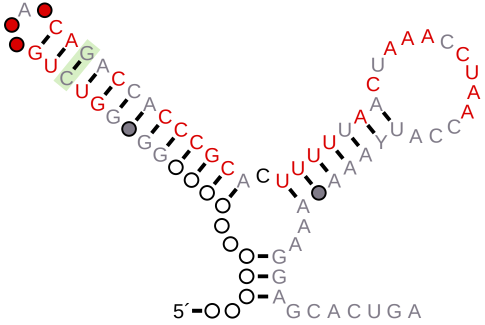

HMP2_8224_1

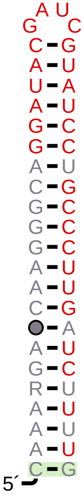

HMP2_8227_1

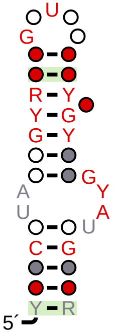

HMP2_8234_1

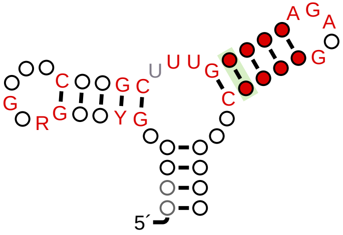

HMP2_8235_1

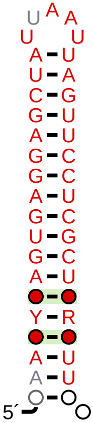

HMP2_8237_1

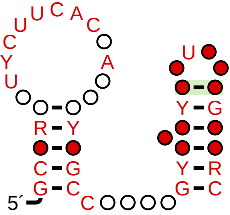

HMP2_8243_1

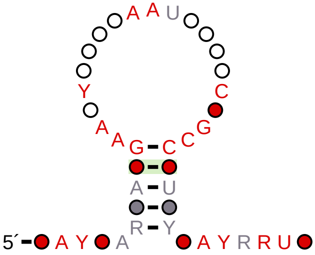

HMP2_8247_1

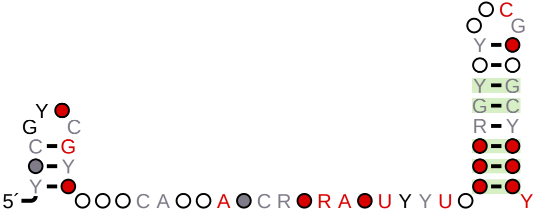

HMP2_8256_1

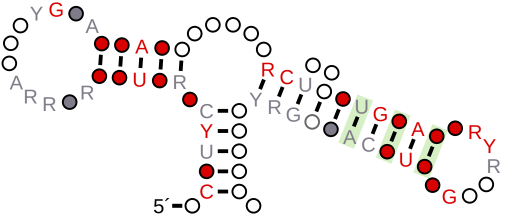

HMP2_8264_1

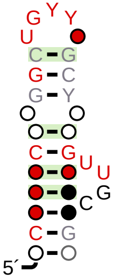

HMP2_8265_1

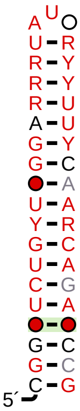

HMP2_8274_1

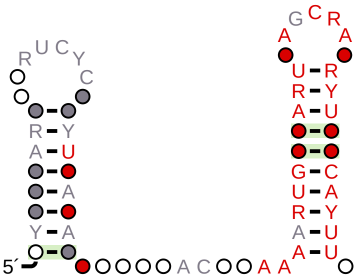

HMP2_8279_1

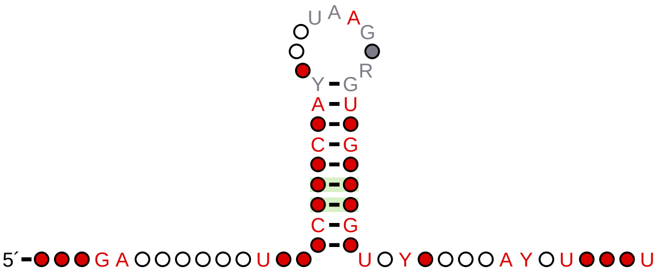

HMP2_8280_1

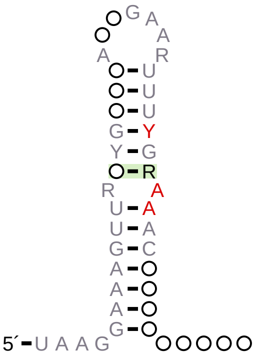

HMP2_8288_1

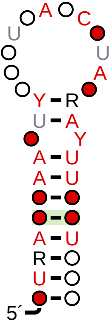

HMP2_8289_1

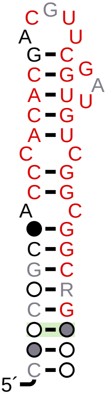

HMP2_829_1

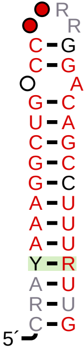

HMP2_8293_1

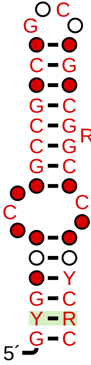

HMP2_8294_1

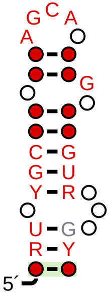

HMP2_83_1

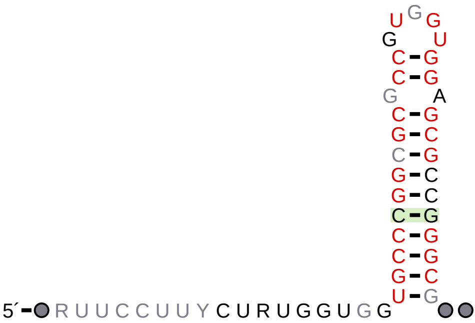

HMP2_8304_1

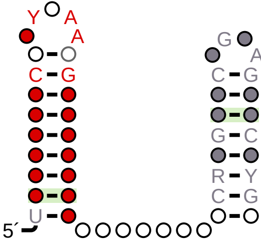

HMP2_8306_1

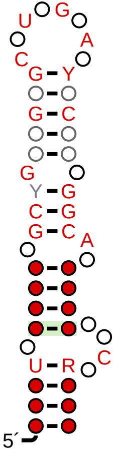

HMP2_8307_1

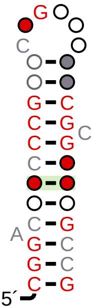

HMP2_8316_1

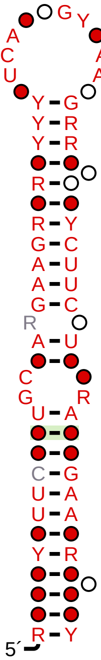

HMP2_8321_1

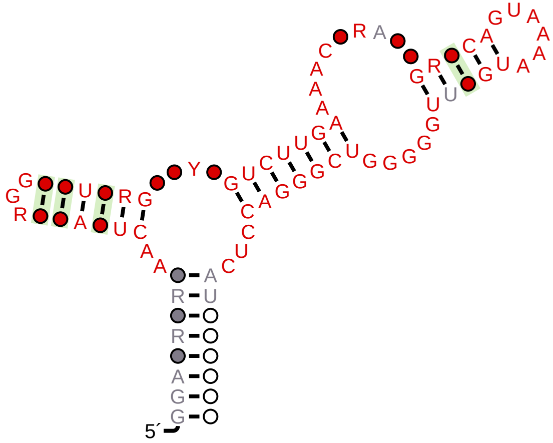

HMP2_8327_1

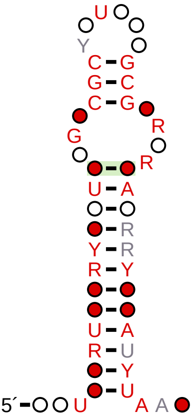

HMP2_833_1

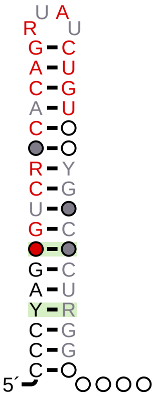

HMP2_8338_1

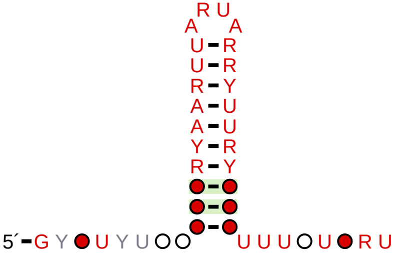

HMP2_834_1

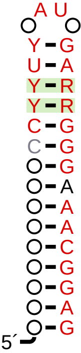

HMP2_8341_1

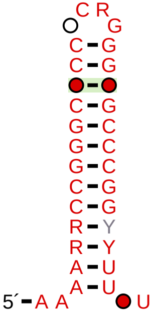

HMP2_8343_1

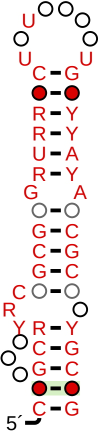

HMP2_8348_1

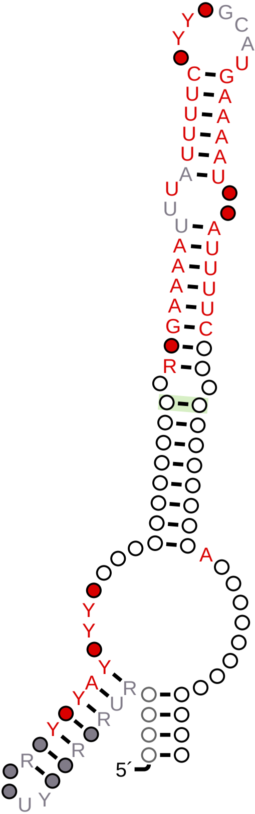

HMP2_8359_1

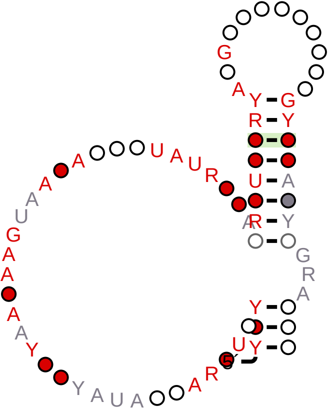

HMP2_8363_1

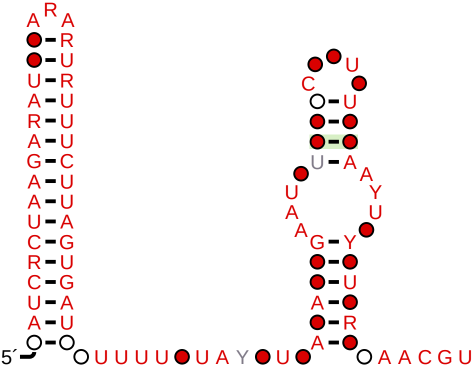

HMP2_8368_1

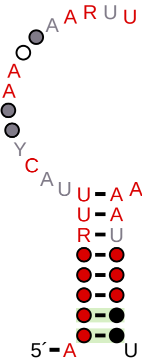

HMP2_8371_1

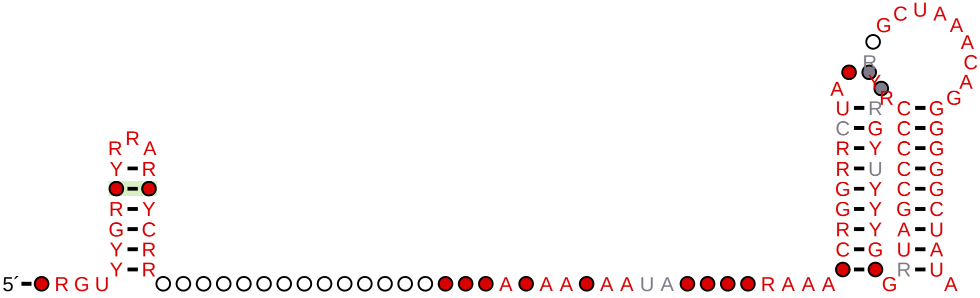

HMP2_8378_1

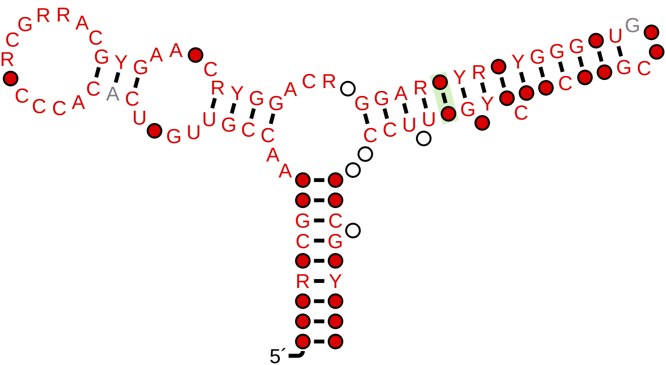

HMP2_8379_1

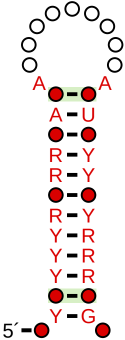

HMP2_838_1

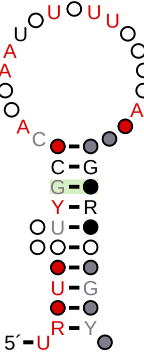

HMP2_8382_1

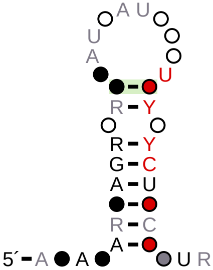

HMP2_8392_1

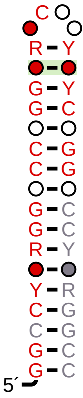

HMP2_8398_1

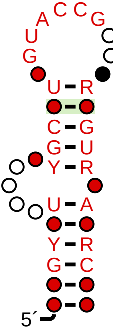

HMP2_840_1

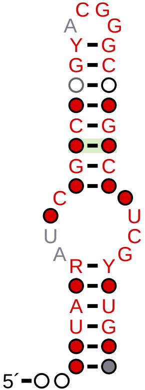

HMP2_8408_1

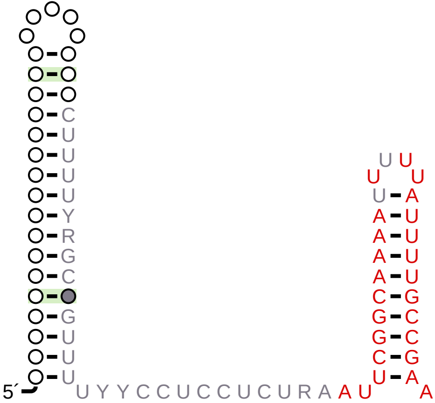

5´
C
U
U
U
U
Y
R
G
C
G
U
U
U
UYYCCUCCUCURAAU
UU
U U
U A
A U
A U
A U
A U
C G
G C
G C
C G
U A
A

HMP2_841_1

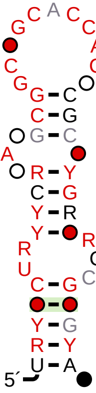

HMP2_8419_1

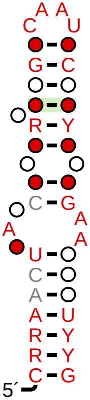

HMP2_843_1

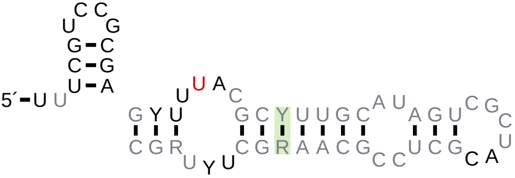

C C
U G
G C
C G
U A
U A C
5´ U U
U
A U C G
G Y U
G C Y U U G C
A G U
C
C G R
C G R A A C G
U C G
U
U Y U
C C
C A

HMP2_8438_1

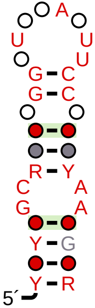

HMP2_8440_1

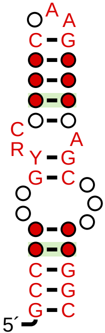

HMP2_846_1

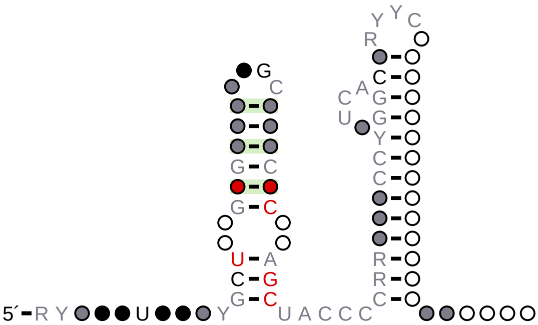

HMP2_8461_1

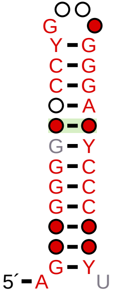

HMP2_847_1

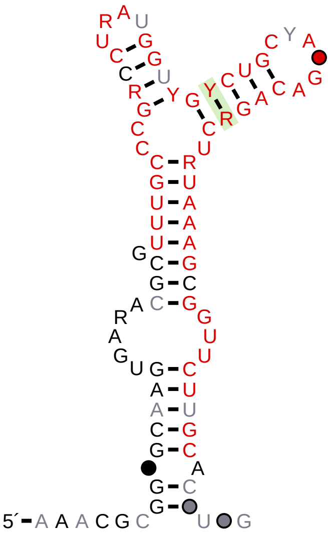

HMP2_8487_1

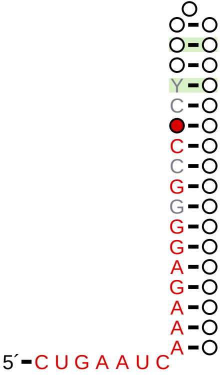

HMP2_8490_1

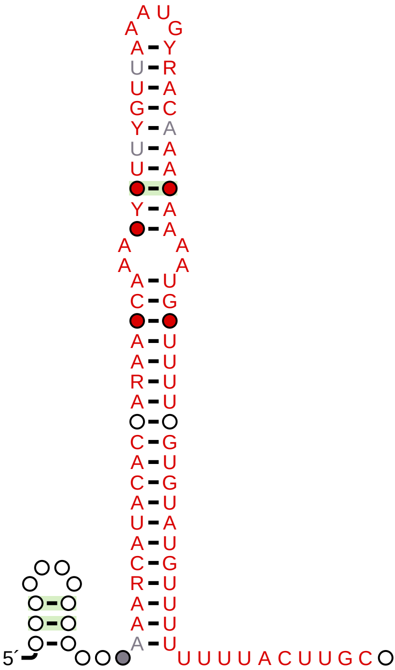

HMP2_8491_1

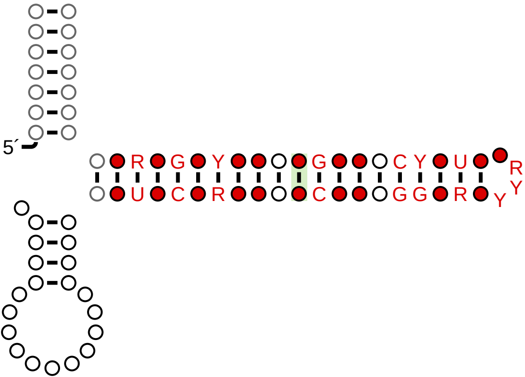

HMP2_8492_1

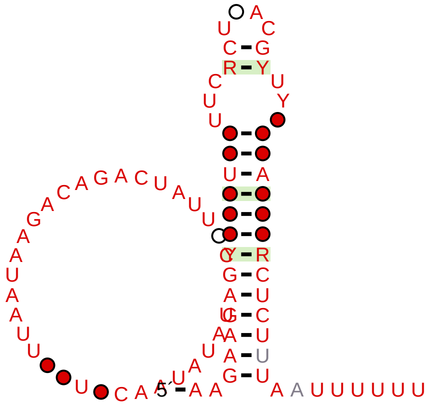

HMP2_8500_1

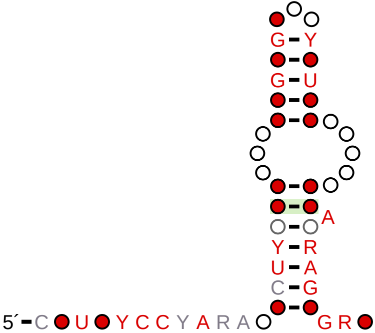

HMP2_8504_1

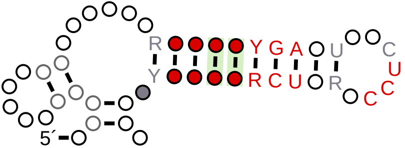

HMP2_8513_1

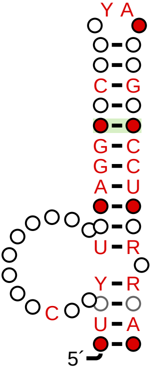

HMP2_8517_1

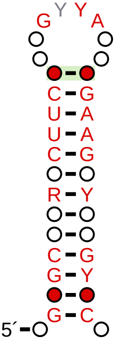

HMP2_8520_1

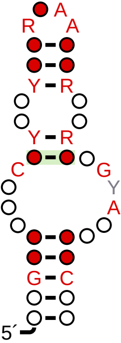

HMP2_8526_1

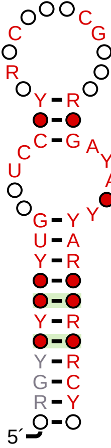

HMP2_8535_1

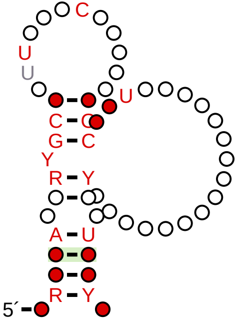

HMP2_8536_1

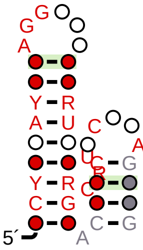

HMP2_8537_1

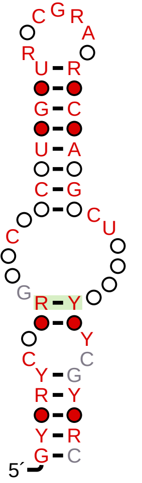

HMP2_8541_1

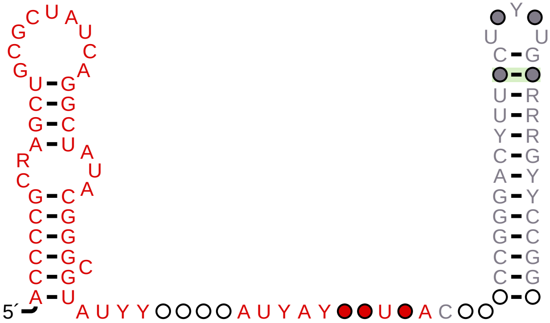

HMP2_8543_1

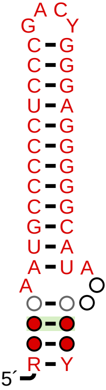

HMP2_8544_1

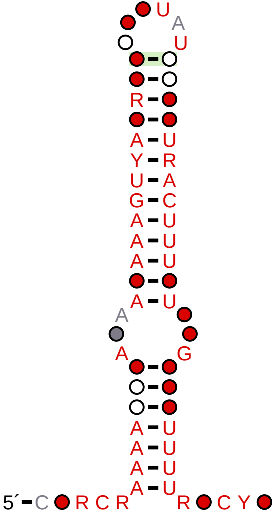

HMP2_8545_1

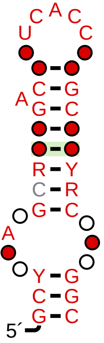

HMP2_8549_1

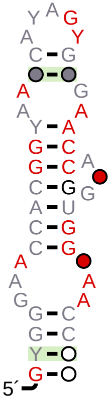

HMP2_856_1

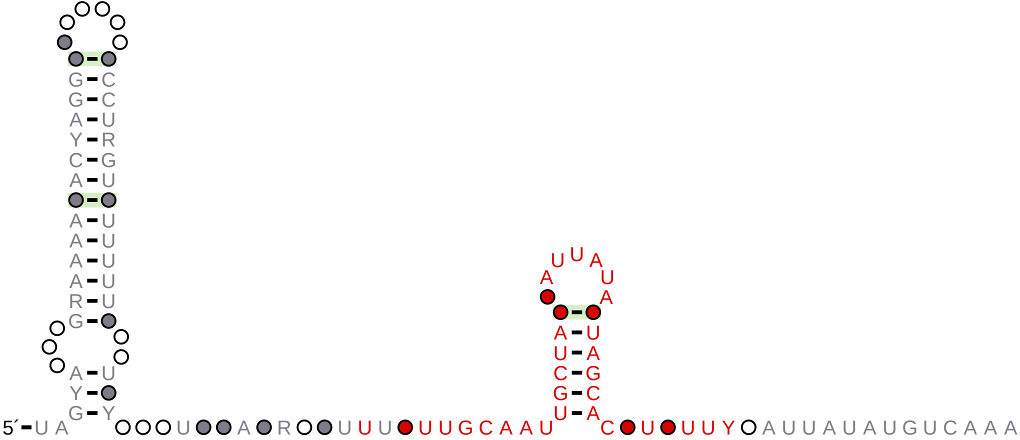

G-C
G-C
A-U
Y-R
C-G
A-U
A-U
A-U
A-U
A-U
R-U
G
A-U
Y
G-Y
U U A
A U
A
A-U
U-A
C-G
G-C
U-A
5´ U A U A R U U U U U G C A A U C U U U Y A U U A U A U G U C A A A

HMP2_8574_1

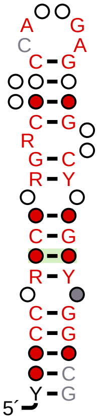

HMP2_8575_1

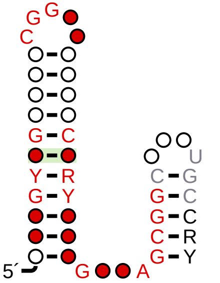

HMP2_858_1

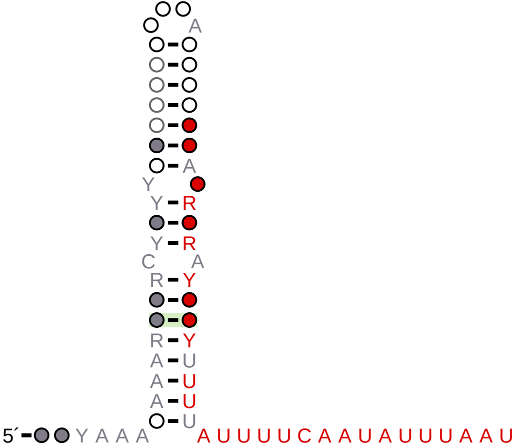

HMP2_859_1

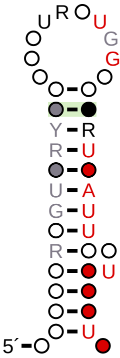

HMP2_8591_1

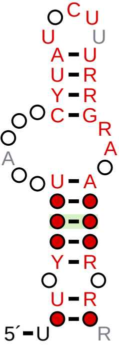

HMP2_8596_1

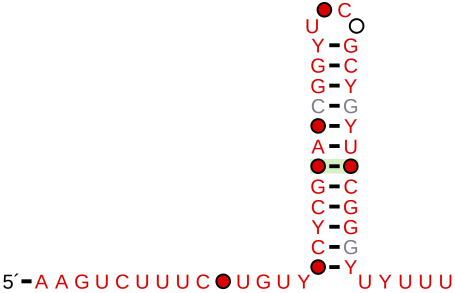

HMP2_8597_1

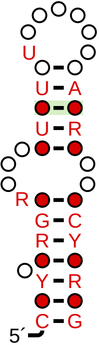

HMP2_86_1

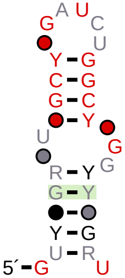

HMP2_8609_1

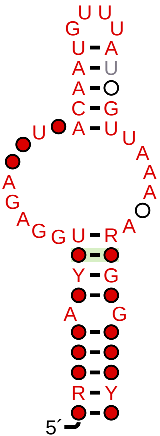

HMP2_8613_1

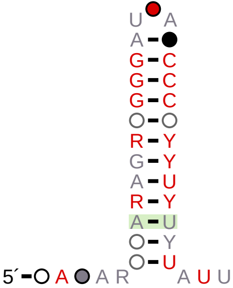

HMP2_8616_1

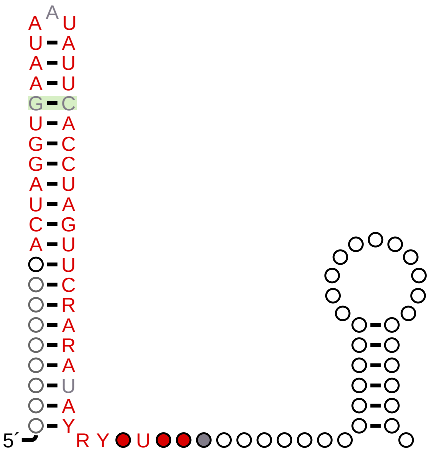

HMP2_8617_1

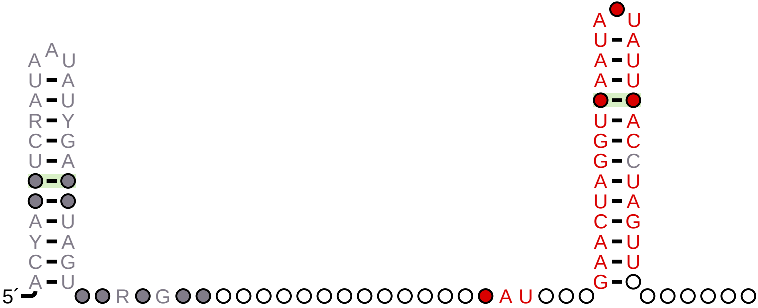

HMP2_8619_1

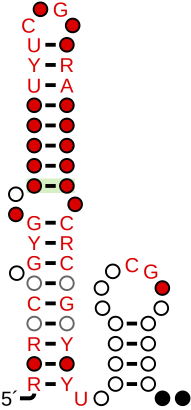

HMP2_8622_1

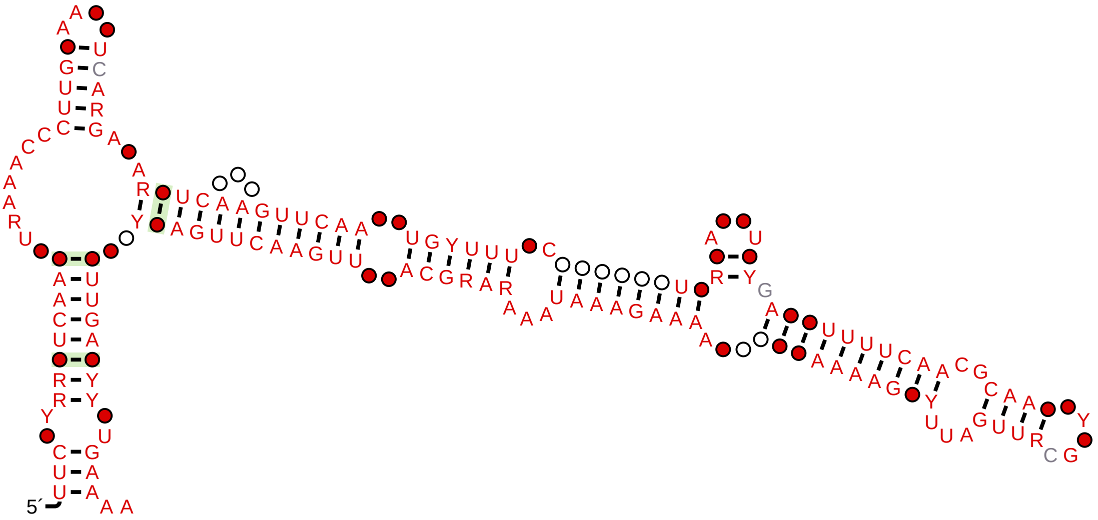

HMP2_8623_1

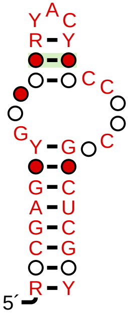

HMP2_8624_1

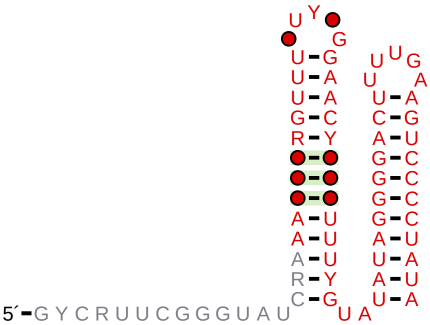

HMP2_863_1

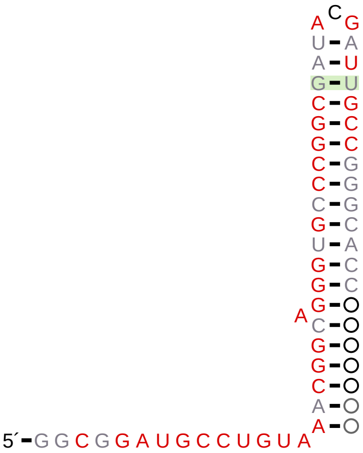

HMP2_8635_1

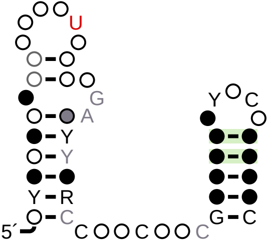

HMP2_8637_1

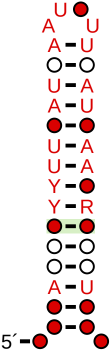

HMP2_8638_1

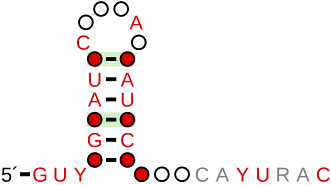

HMP2_8644_1

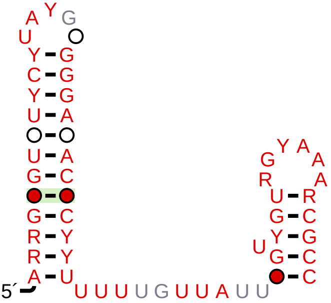

HMP2_8647_1

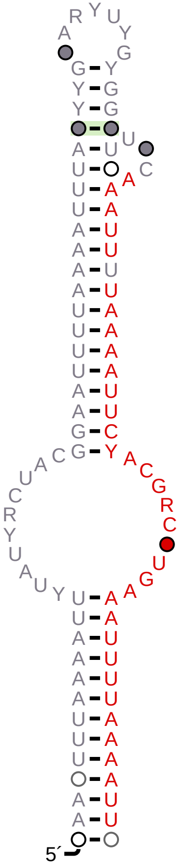

HMP2_8663_1

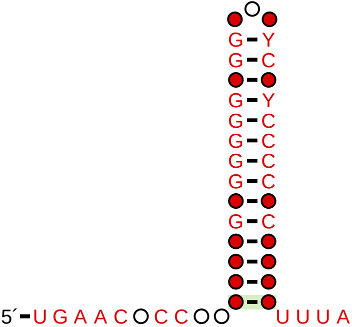

HMP2_8670_1

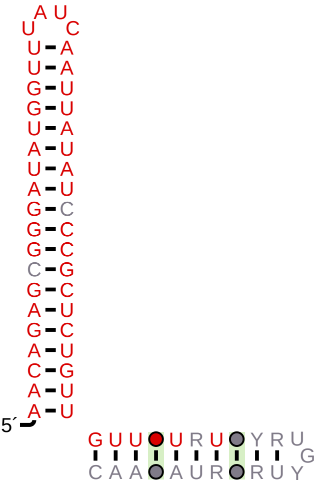

HMP2_8678_1

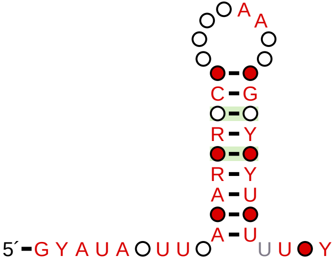

HMP2_8679_1

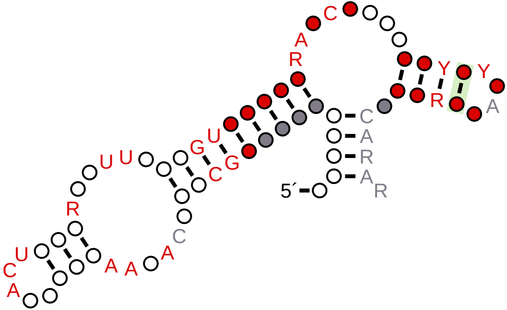

HMP2_8681_1

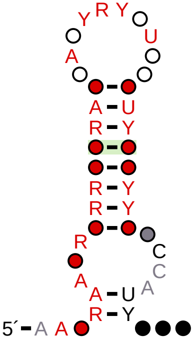

HMP2_8683_1

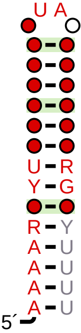

HMP2_8686_1

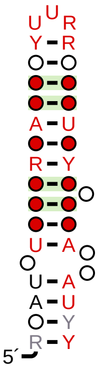

HMP2_8687_1

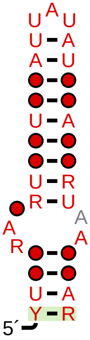

HMP2_8694_1

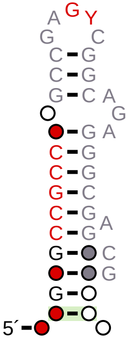

HMP2_8705_1

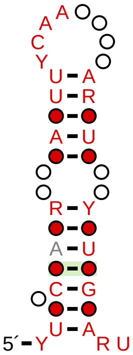

HMP2_8706_1

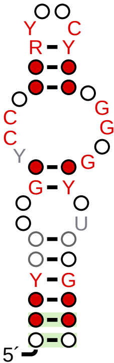

HMP2_8708_1

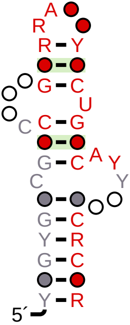

HMP2_8718_1

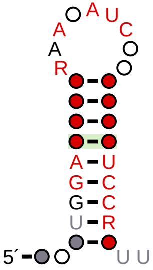

HMP2_872_1

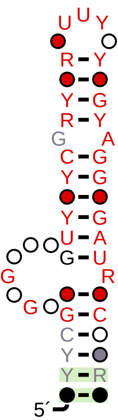

HMP2_8728_1

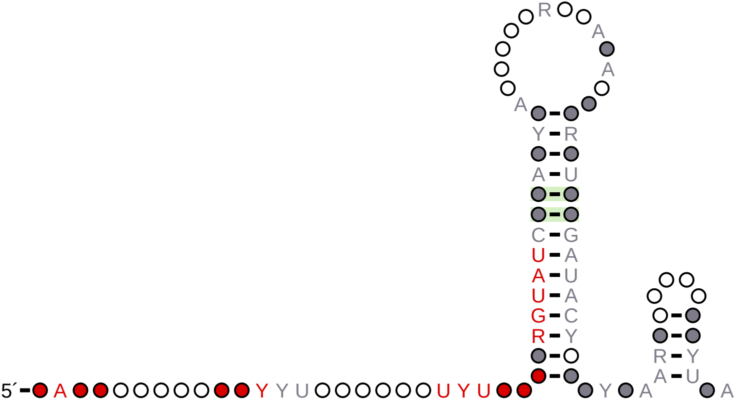

HMP2_873_1

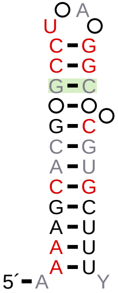

HMP2_8730_1

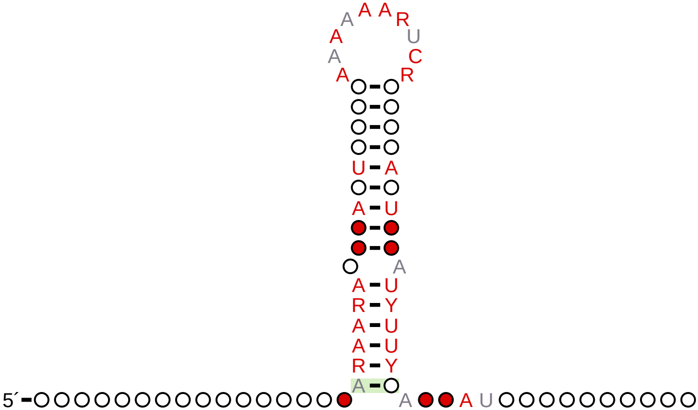

HMP2_8733_1

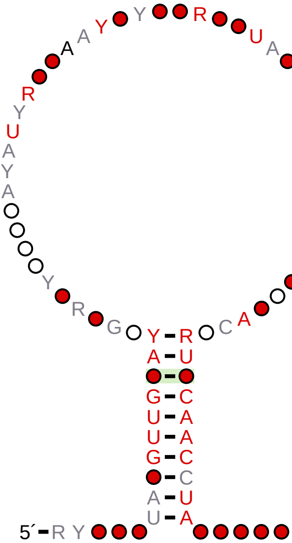

HMP2_8734_1

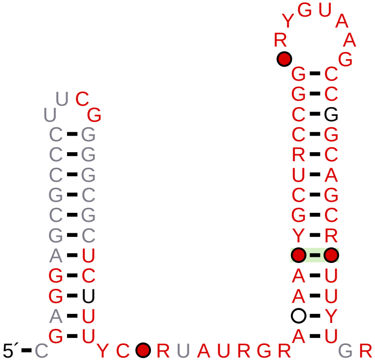

HMP2_8744_1

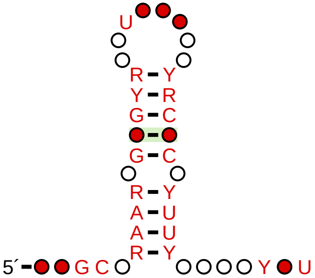

HMP2_8753_1

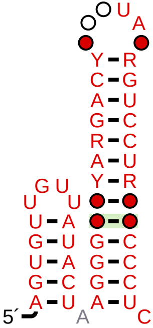

HMP2_8759_1

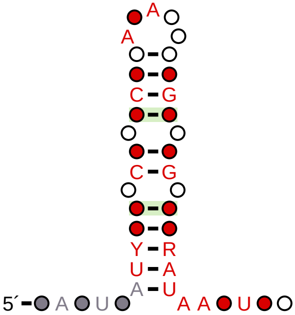

HMP2_8765_1

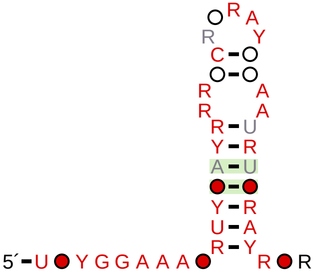

HMP2_8772_1

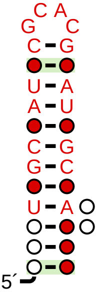

HMP2_8782_1

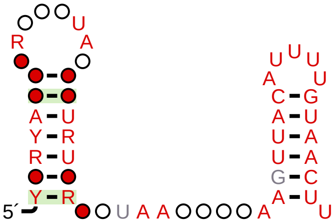

HMP2_8783_1

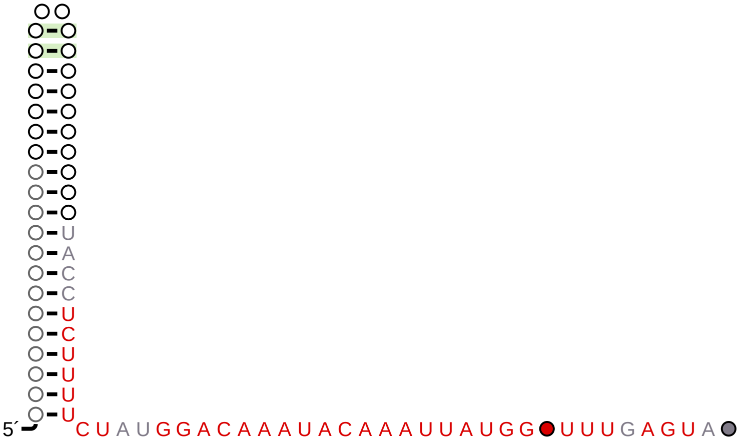

HMP2_8800_1

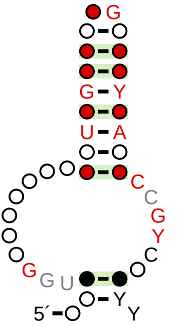

HMP2_8808_1

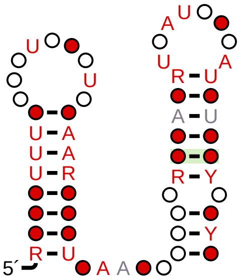

HMP2_8809_1

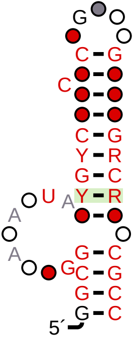

HMP2_881_1

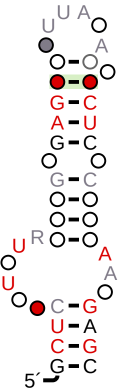

HMP2_8812_1

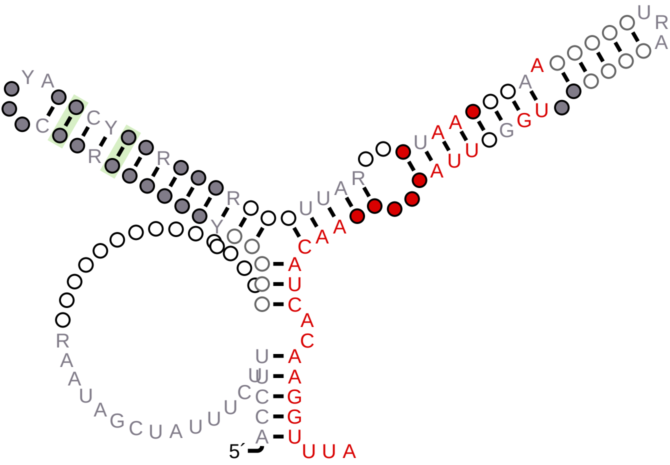

HMP2_8819_1

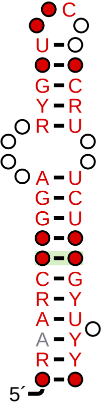

HMP2_8823_1

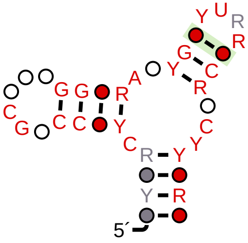

HMP2_8828_1

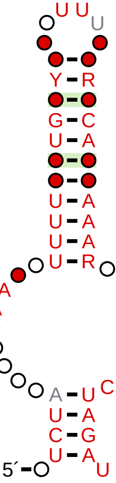

HMP2_883_1

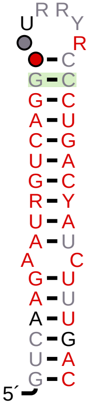

HMP2_8830_1

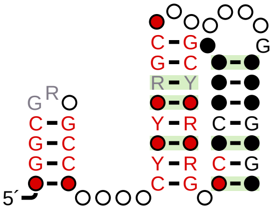

HMP2_8838_1

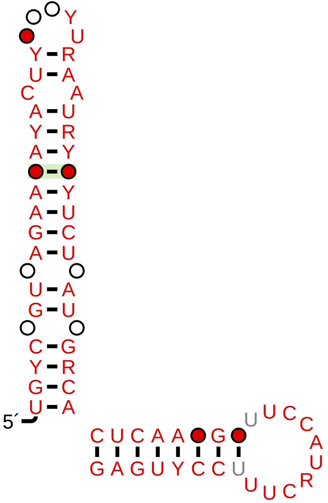

5´
Y U
Y R
U A
C A
A U
Y R
A Y
A Y
A U
G C
A U
U A
G U
C G
Y R
G C
U A
CUCAAG
GAGUYCCU
UUCCAURCUU

HMP2_884_1

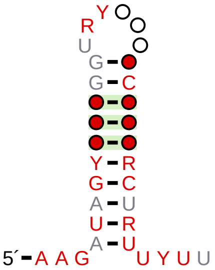

HMP2_8844_1

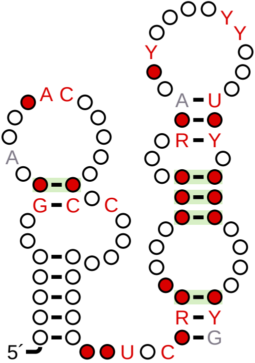

HMP2_8852_1

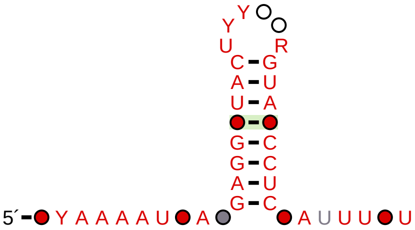

HMP2_8855_1

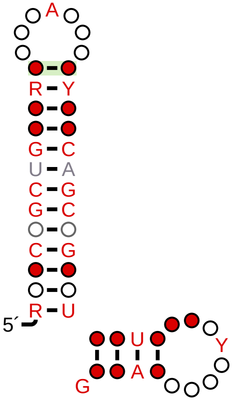

HMP2_8856_1

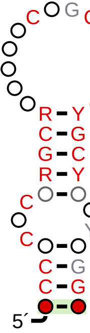

HMP2_8858_1

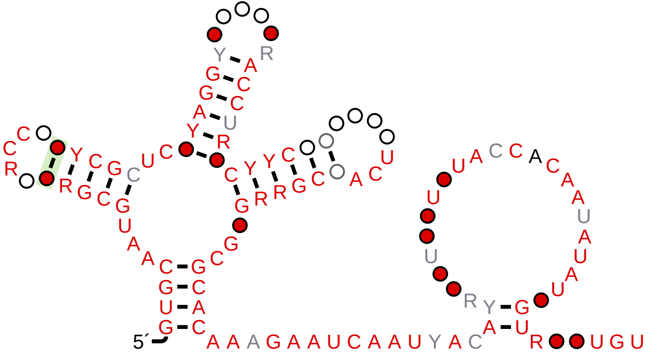

HMP2_8861_1

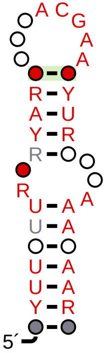

HMP2_8862_1

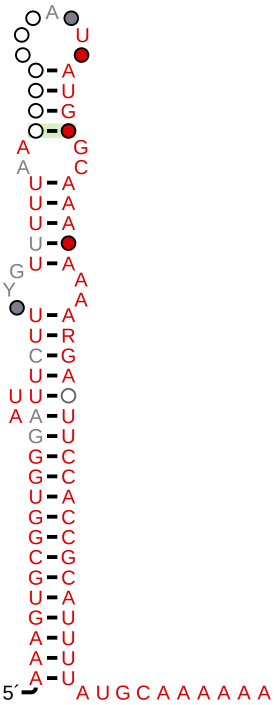

HMP2_8869_1

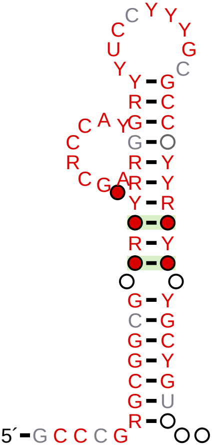

HMP2_8872_1

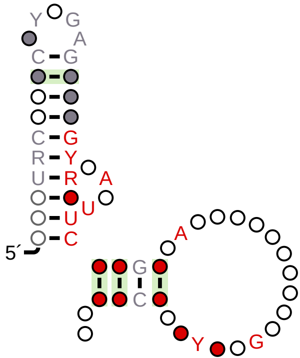

HMP2_8882_1

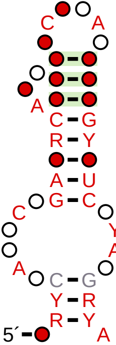

HMP2_8883_1

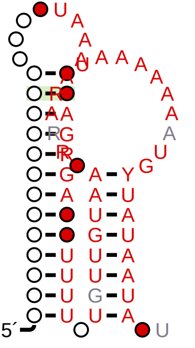

HMP2_889_1

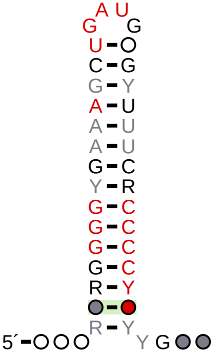

HMP2_8913_1

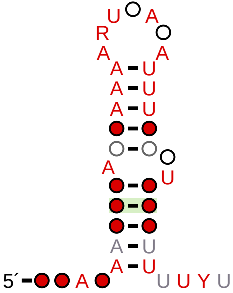

HMP2_892_1

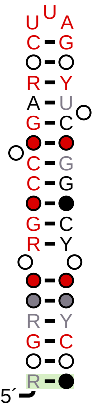

HMP2_8921_1

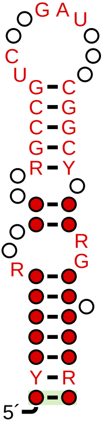

HMP2_8931_1

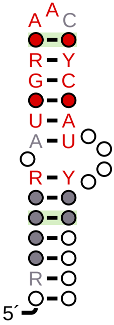

HMP2_8935_1

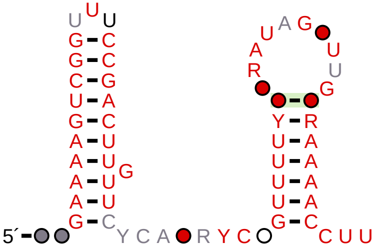

HMP2_8936_1

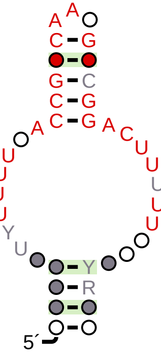

HMP2_8937_1

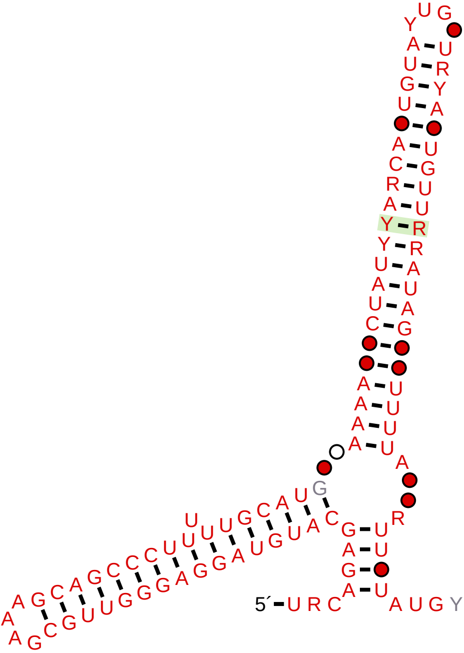

HMP2_8946_1

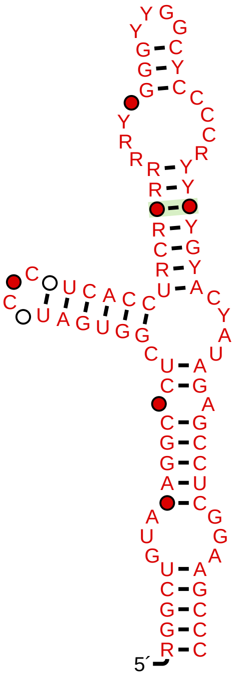

HMP2_8951_1

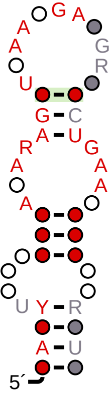

HMP2_8954_1

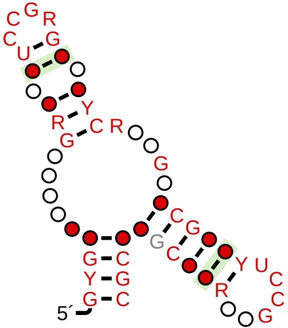

HMP2_8957_1

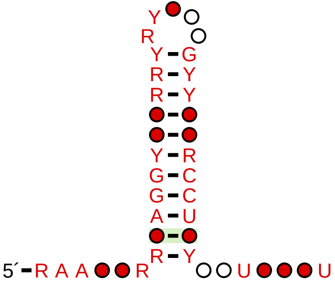

HMP2_8959_1

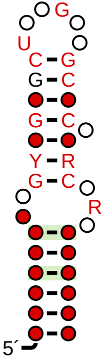

HMP2_8962_1

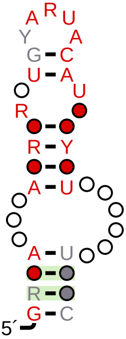

HMP2_8966_1

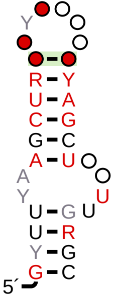

HMP2_897_1

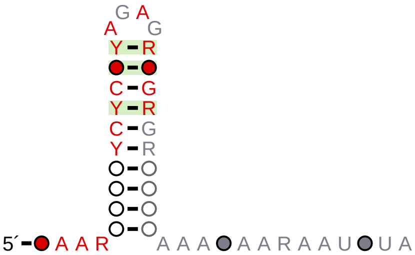

HMP2_8972_1

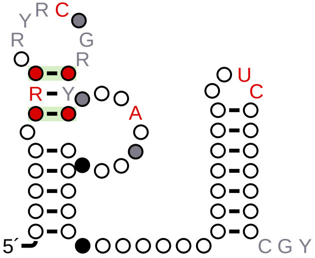

HMP2_8981_1

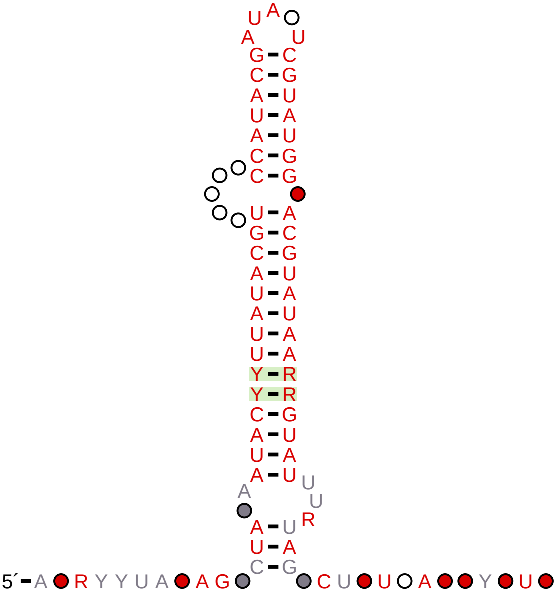

HMP2_8985_1

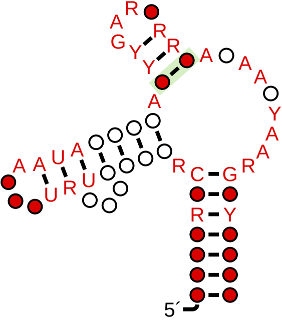

HMP2_8988_1

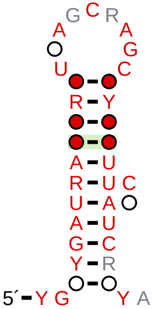

HMP2_8989_1

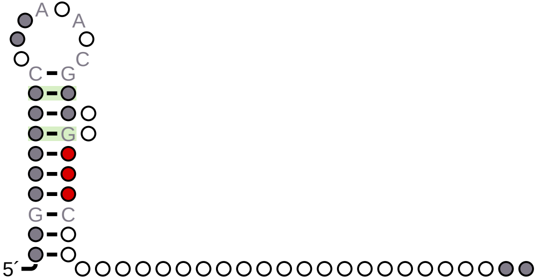

HMP2_9_1

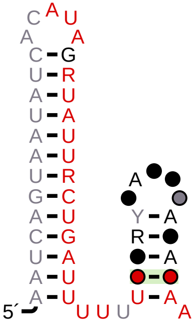

HMP2_9013_1

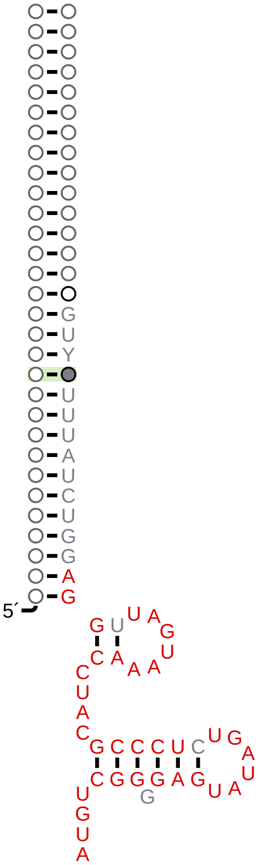

G
U
Y
U
U
U
A
U
C
U
G
G
A
G
5´
G U U A G
C A A A U
C
U
A
C
G C C C U C U G A
C G G G A G U A U
G
U
G
U
A

HMP2_9017_1

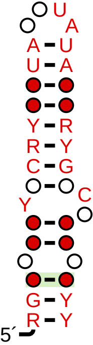

HMP2_9027_1

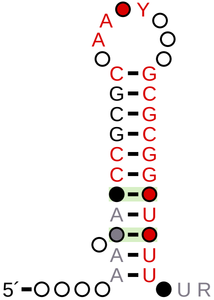

HMP2_9048_1

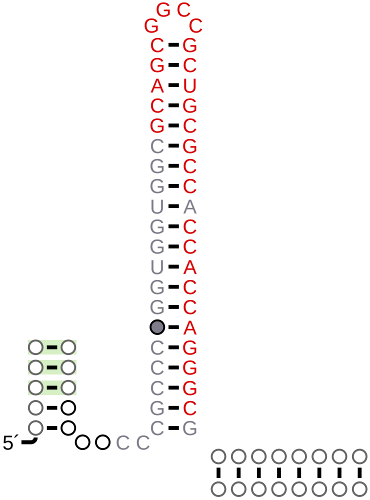

HMP2_905_1

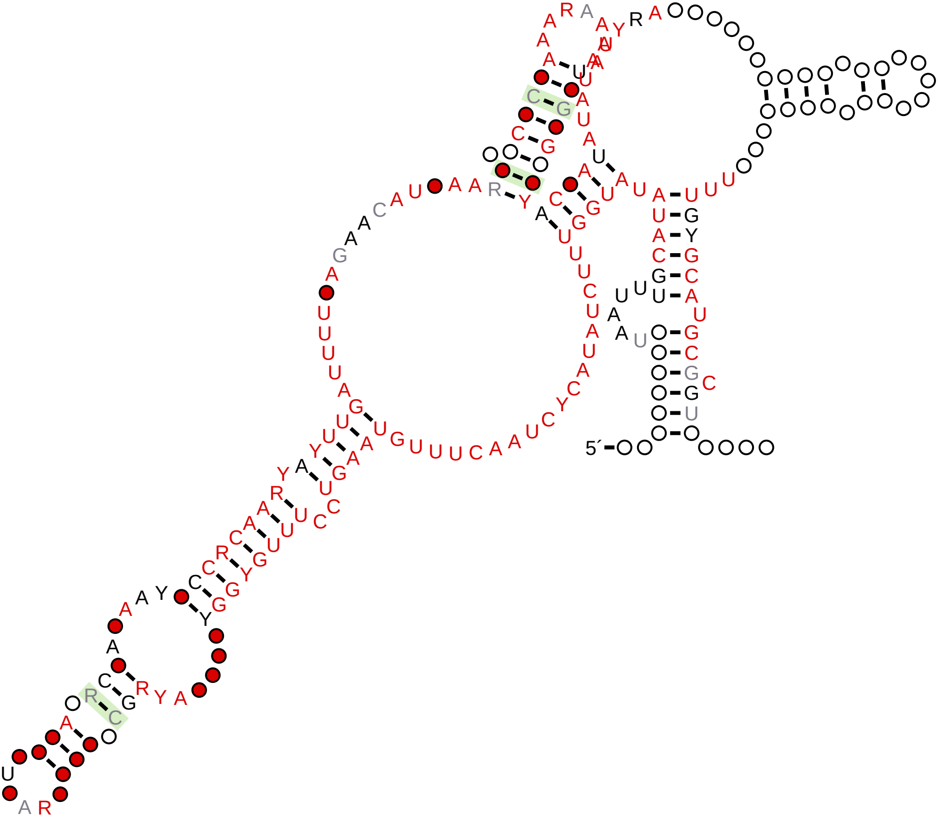

HMP2_9059_1

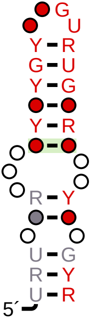

HMP2_9062_1

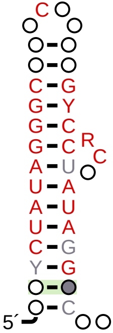

HMP2_9064_1

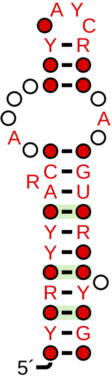

HMP2_9066_1

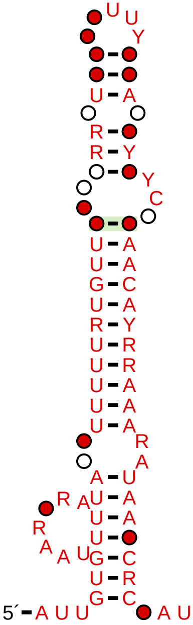

U U
Y
U A
R
R Y
Y
C
U A
U A
G C
U A
R Y
U R
U R
U A
U A
U A
R
A
A U
R A
U A
U
R
A A U
G C
U R
G C
5´ A U U
A U

HMP2_9075_1

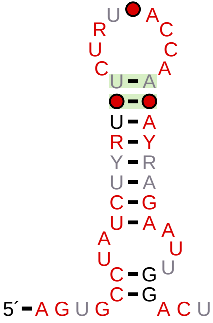

HMP2_9080_1

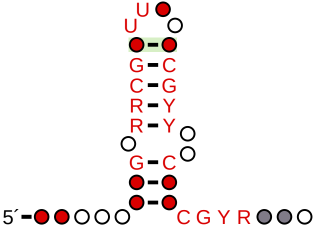

HMP2_9100_1

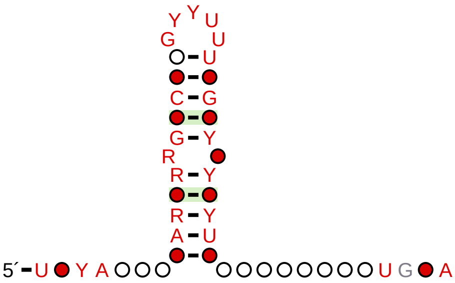

HMP2_9101_1

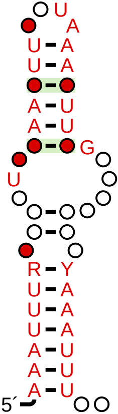

HMP2_9112_1

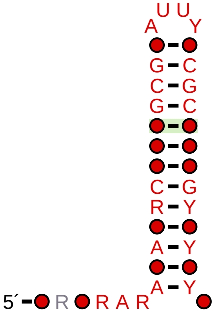

HMP2_9118_1

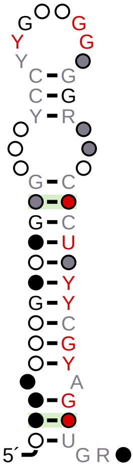

HMP2_9119_1

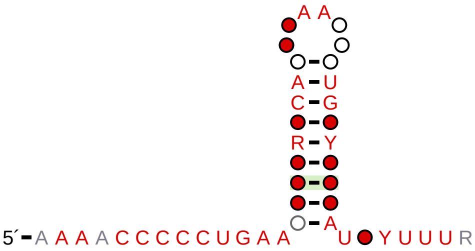

HMP2_9123_1

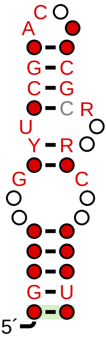

HMP2_9132_1

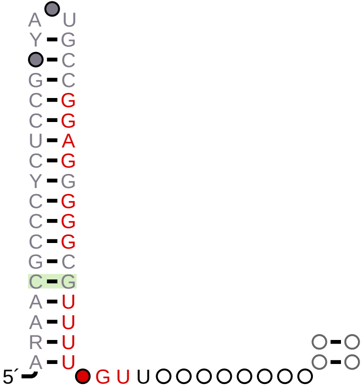

HMP2_9134_1

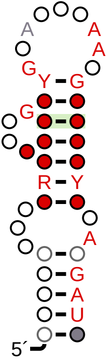

HMP2_9138_1

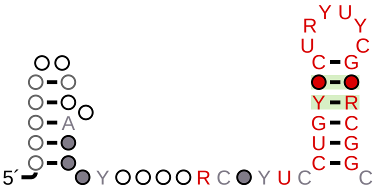

HMP2_9145_1

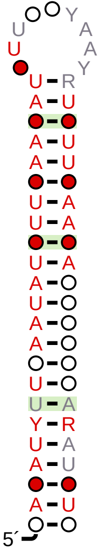

HMP2_9153_1

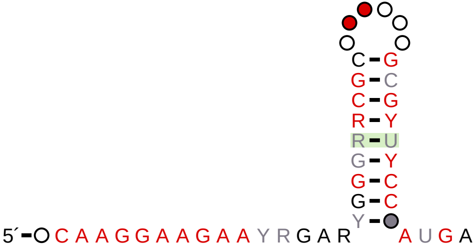

HMP2_9161_1

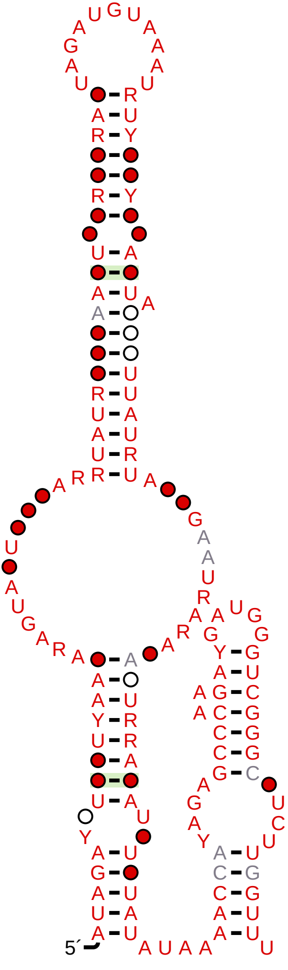

HMP2_917_1

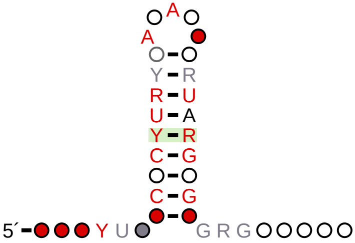

HMP2_9170_1

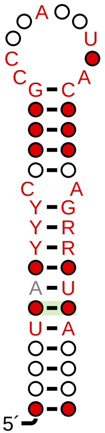

HMP2_9171_1

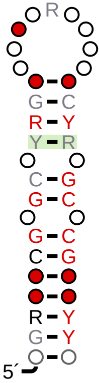

HMP2_9173_1

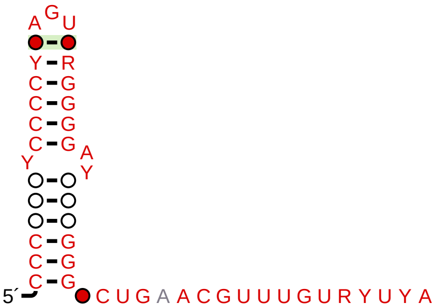

HMP2_9176_1

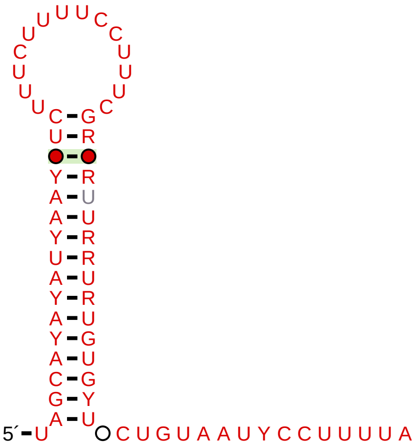

HMP2_9180_1

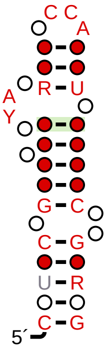

HMP2_9184_1

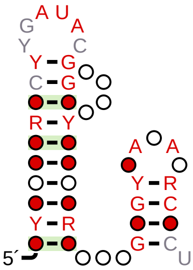

HMP2_9191_1

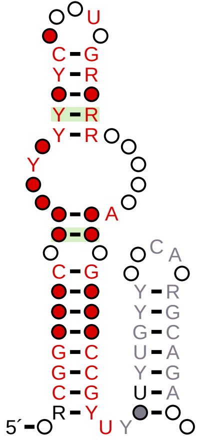

HMP2_9193_1

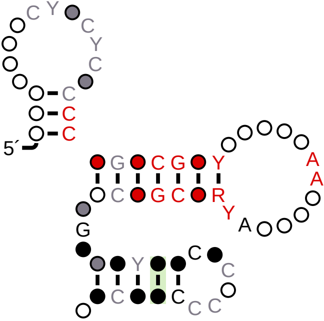

HMP2_9198_1

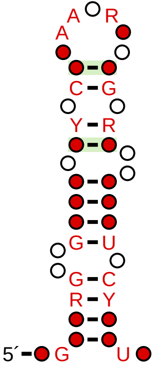

HMP2_9203_1

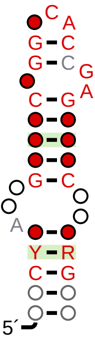

HMP2_9226_1

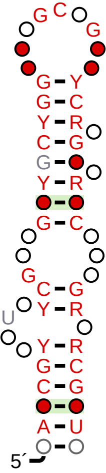

HMP2_9228_1

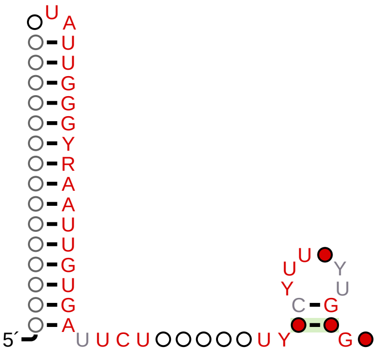

HMP2_9235_1

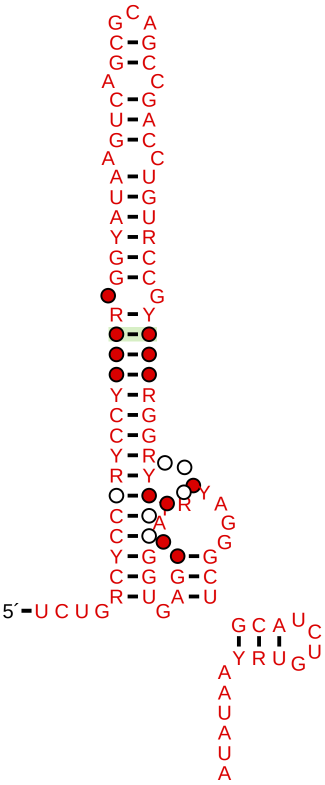

HMP2_924_1

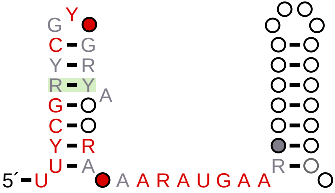

HMP2_9242_1

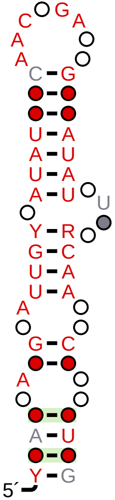

HMP2_9246_1

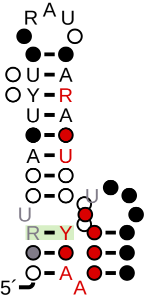

HMP2_9251_1

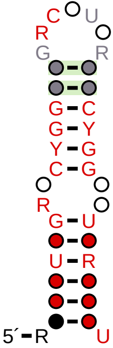

HMP2_9253_1

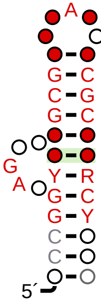

HMP2_9256_1

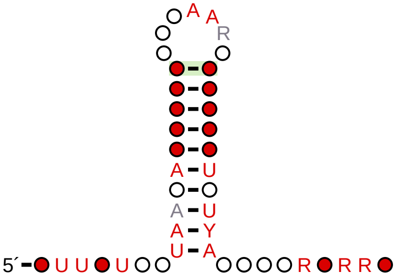

HMP2_9258_1

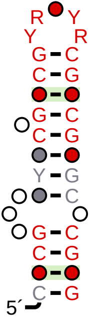

HMP2_9260_1

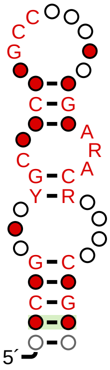

HMP2_9262_1

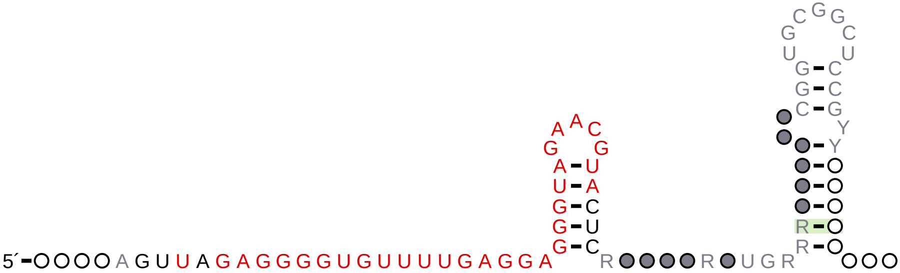

HMP2_9269_1

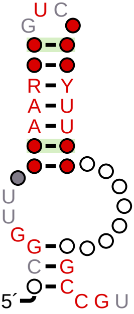

HMP2_9274_1

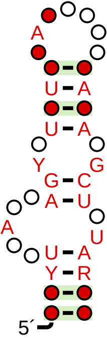

HMP2_9275_1

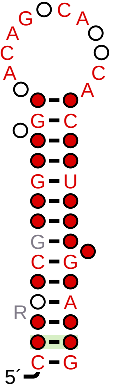

HMP2_928_1

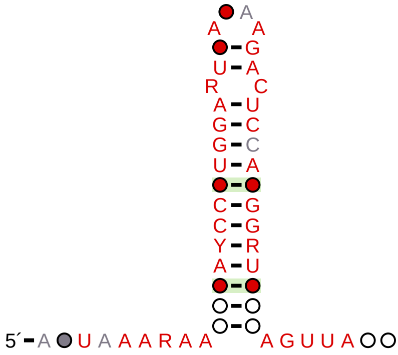

HMP2_9282_1

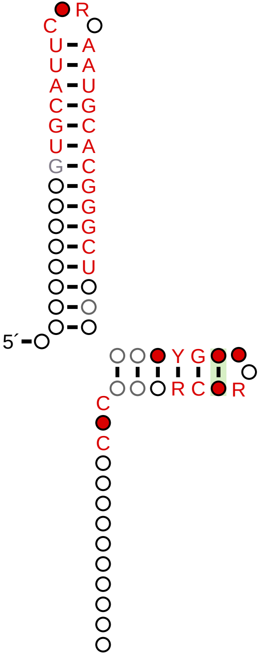

HMP2_9284_1

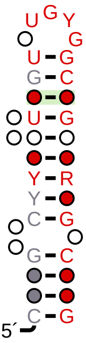

HMP2_929_1

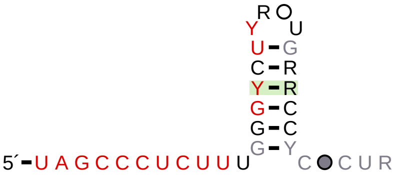

HMP2_9294_1

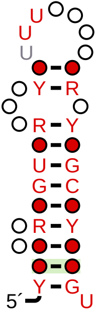

HMP2_9297_1

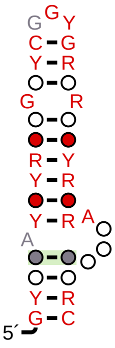

HMP2_9306_1

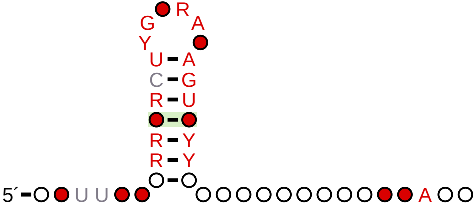

HMP2_9308_1

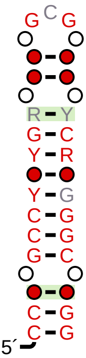

HMP2_931_1

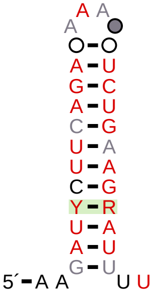

HMP2_9310_1

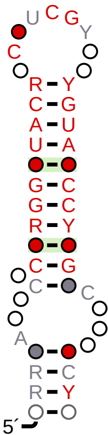

HMP2_9314_1

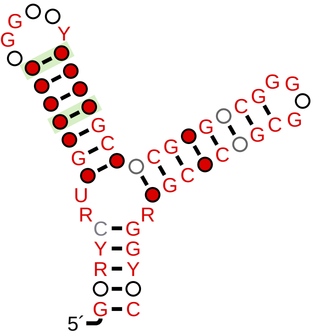

HMP2_9315_1

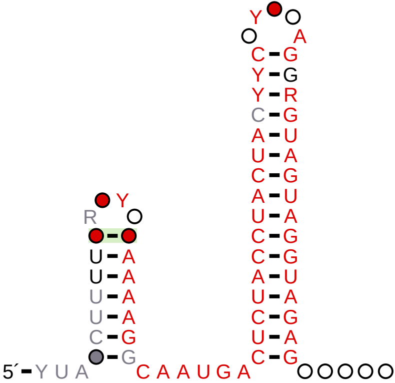

HMP2_9316_1

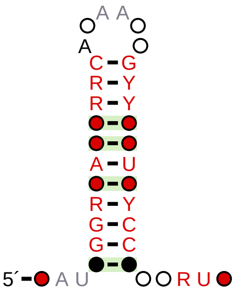

HMP2_932_1

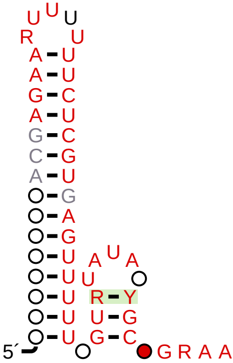

HMP2_9320_1

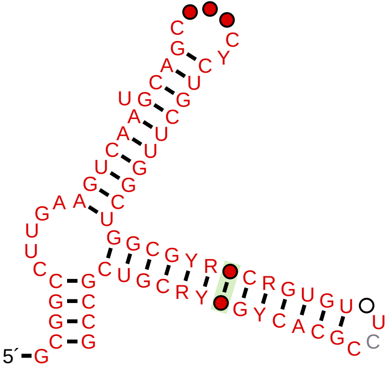

HMP2_9324_1

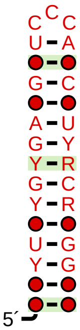

HMP2_9328_1

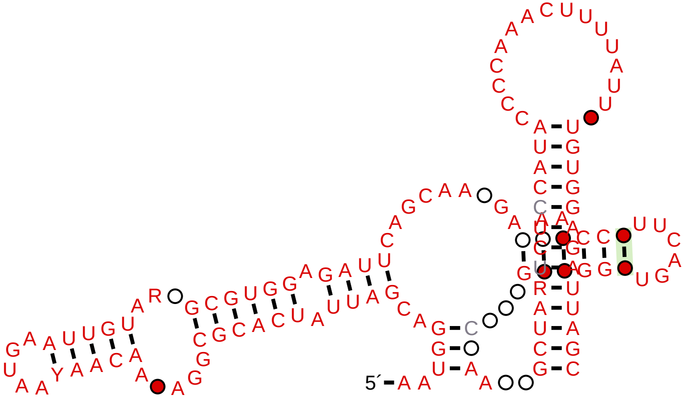

HMP2_9330_1

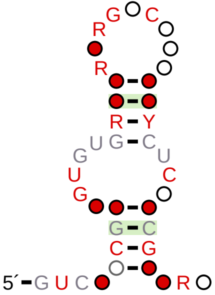

HMP2_9335_1

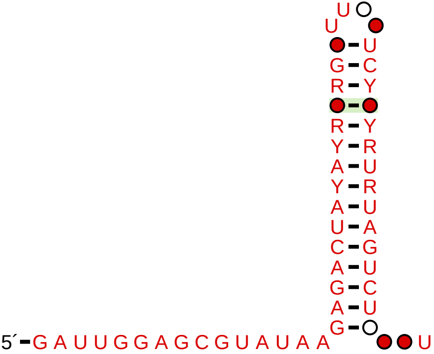

HMP2_934_1

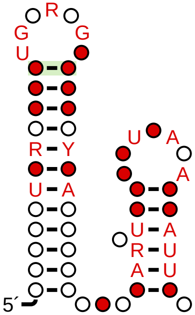

HMP2_9342_1

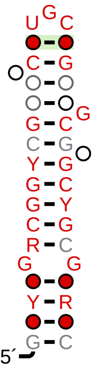

HMP2_9343_1

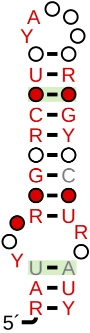

HMP2_9345_1

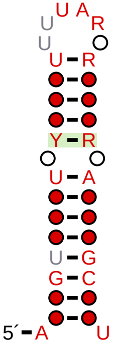

HMP2_9348_1

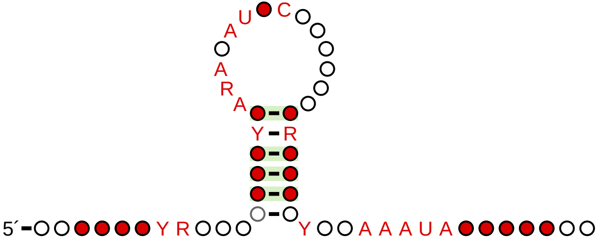

HMP2_9349_1

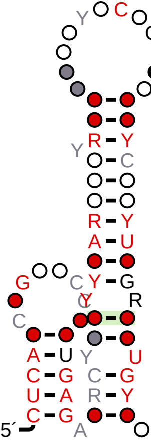

HMP2_9356_1

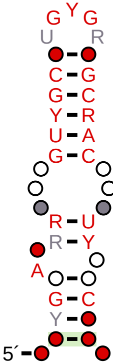

HMP2_9363_1

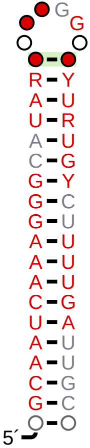

HMP2_9364_1

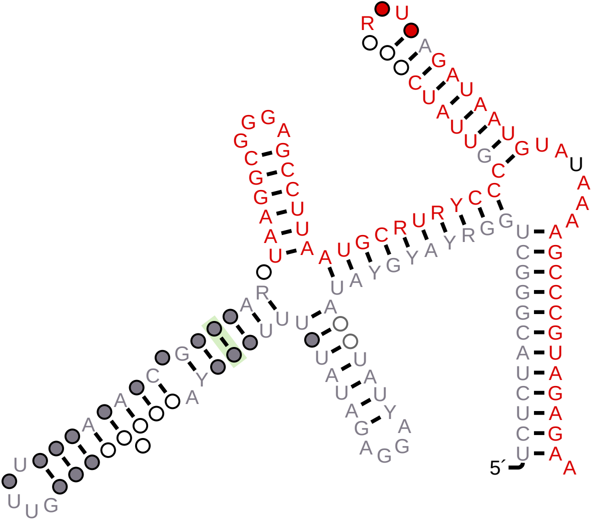

HMP2_9367_1

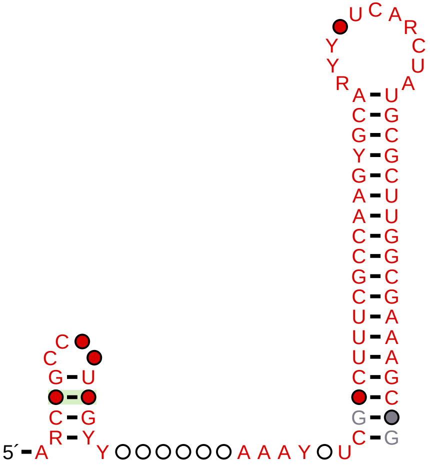

HMP2_9368_1

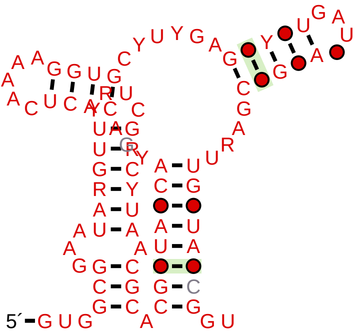

HMP2_938_1

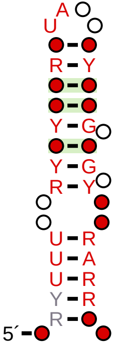

HMP2_9380_1

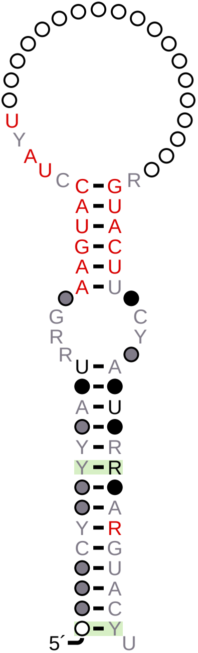

HMP2_9386_1

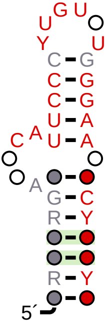

HMP2_9393_1

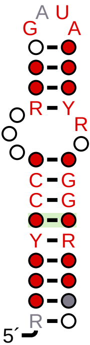

HMP2_9402_1

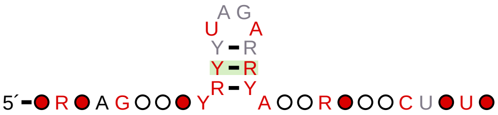

HMP2_9407_1

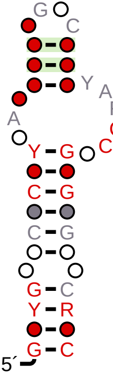

HMP2_9415_1

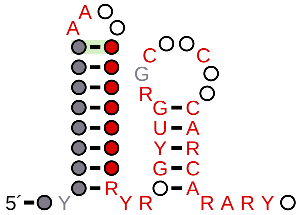

HMP2_9419_1

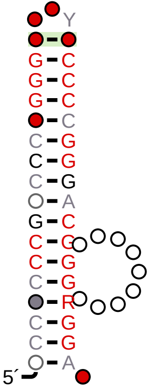

HMP2_942_1

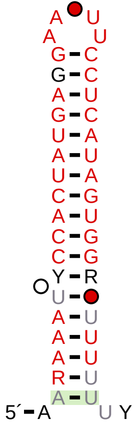

HMP2_9436_1

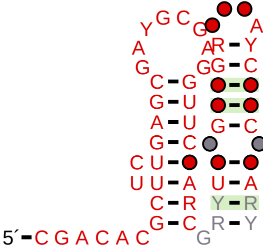

HMP2_9438_1

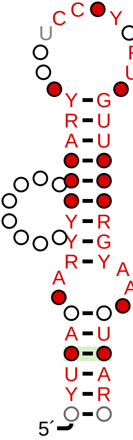

HMP2_9439_1

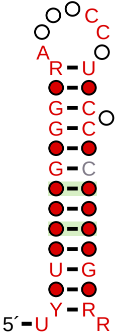

HMP2_9448_1

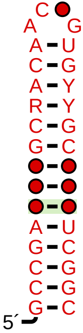

HMP2_9454_1

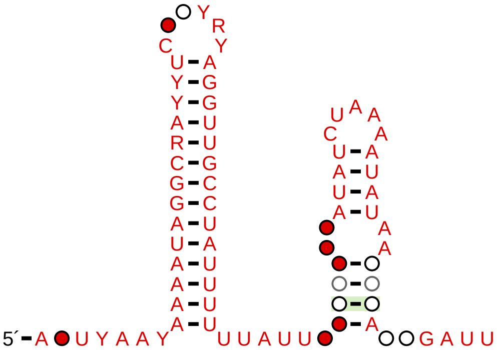

5´
A U Y A A Y
C U Y Y A R C G G A U A A A A
Y R Y A G G U U G C C U A U U U U
U U A U U
U A A C A U A U A A U A U A A A
A
G A U U

HMP2_9455_1

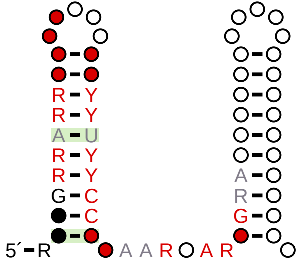

HMP2_9456_1

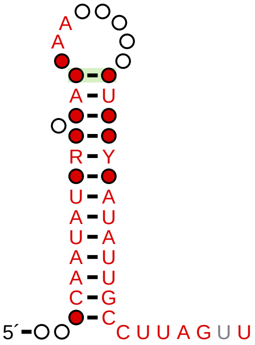

HMP2_9461_1

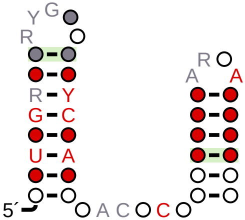

HMP2_9462_1

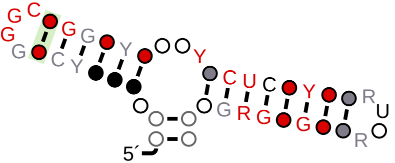

HMP2_9466_1

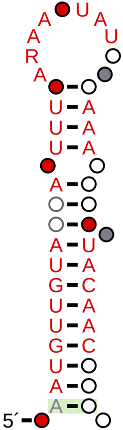

HMP2_9473_1

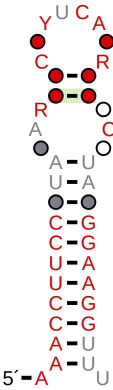

HMP2_9477_1

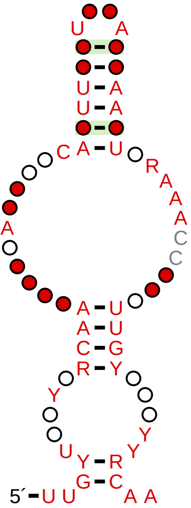

HMP2_9480_1

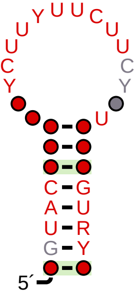

HMP2_9481_1

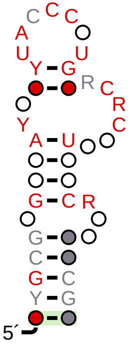

HMP2_9487_1

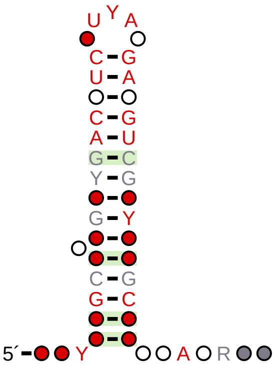

HMP2_9488_1

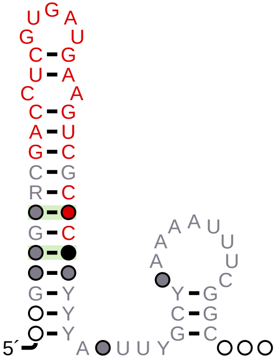

HMP2_9489_1

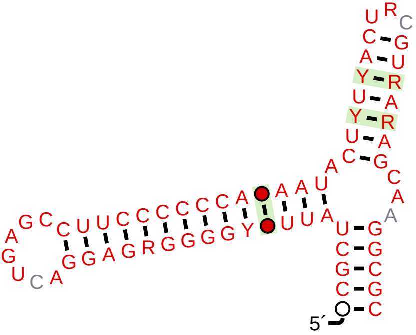

HMP2_949_1

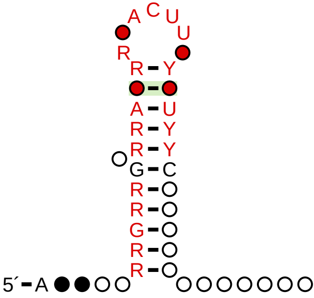

HMP2_9492_1

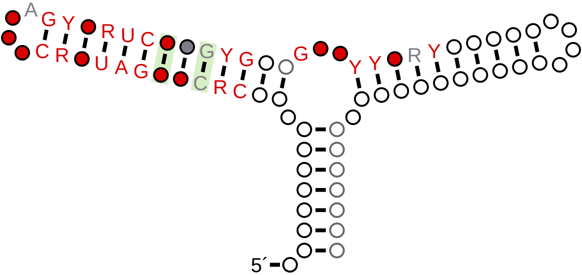

HMP2_95_1

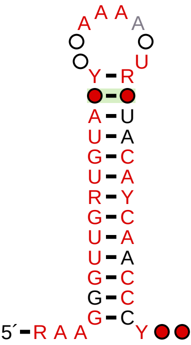

HMP2_9500_1

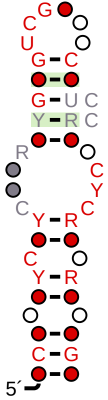

HMP2_9507_1

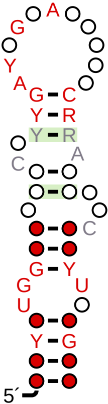

HMP2_9512_1

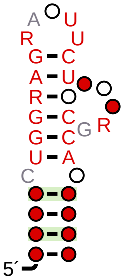

HMP2_9519_1

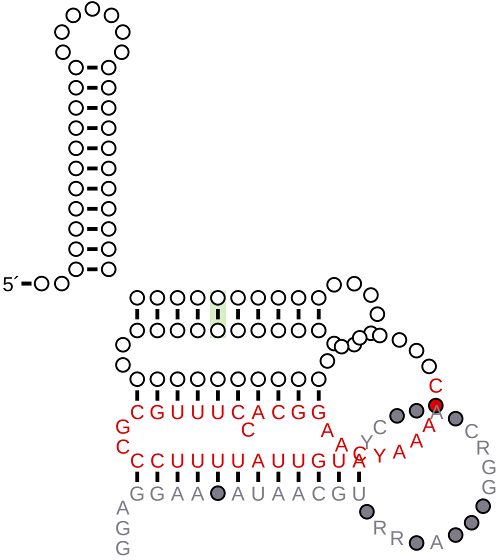
5´
C G U U U C A C G G
G
C
C
C C U U U U A U U G U A
A A C Y A A A
C
G G A A A U A A C G U
A
G
G
C
Y
C
R
G
G
R R A
A

HMP2_9520_1

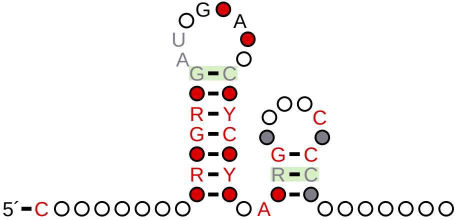

HMP2_9531_1

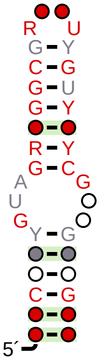

HMP2_9535_1

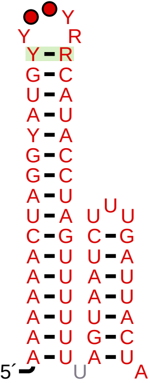

Y
Y
R
Y R
G C
U A
A U
Y A
G C
G C
A U
U A
C G
A U
A U
A U
A U
A U
A U
U U U
C G
U A
A U
A U
U A
G C
A U
5´
U
A

HMP2_9536_1

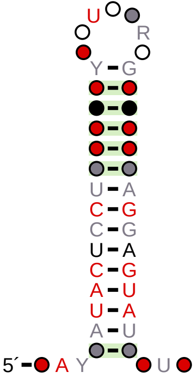

HMP2_9540_1

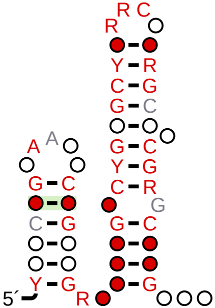

HMP2_9549_1

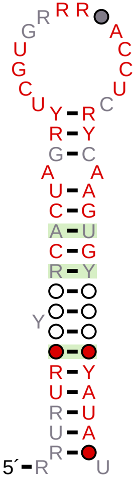

HMP2_9555_1

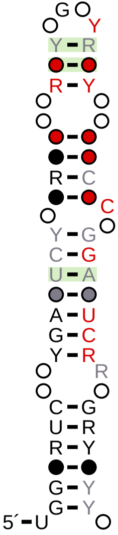

HMP2_9559_1

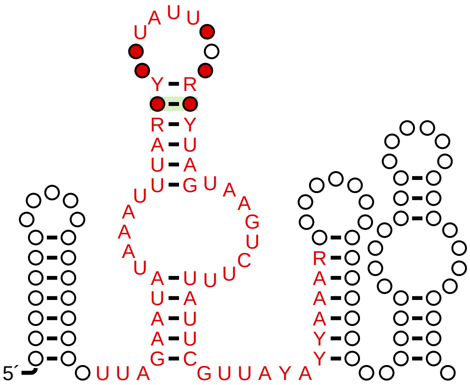

HMP2_9561_1

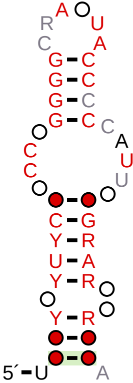

HMP2_957_1

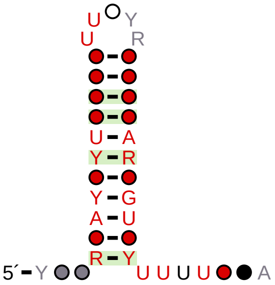

HMP2_9573_1

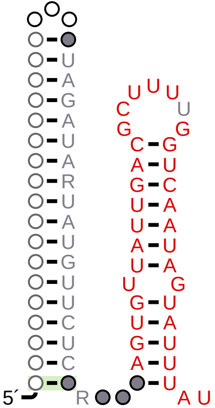

HMP2_9579_1

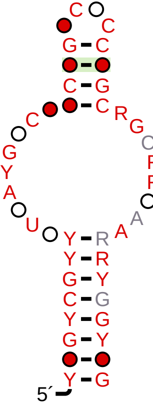

HMP2_9582_1

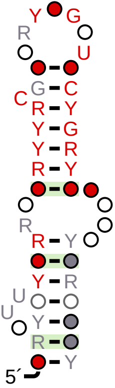

HMP2_9593_1

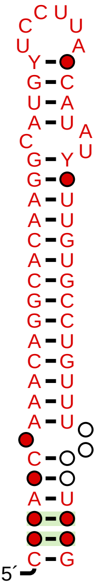

HMP2_9594_1

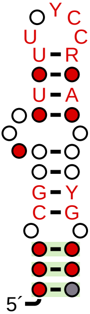

HMP2_9605_1

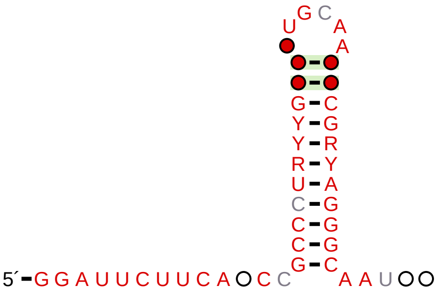

HMP2_9606_1

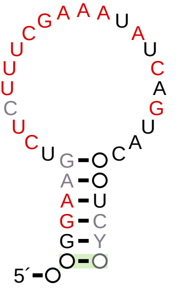

HMP2_9607_1

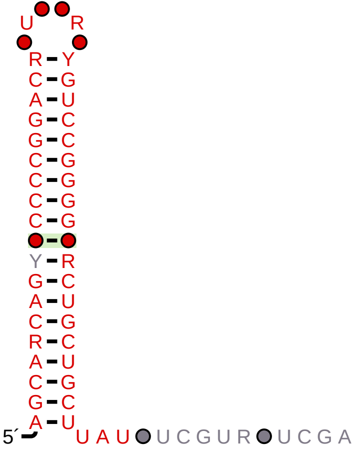

U R
R Y
C G
A U
G C
G C
C G
C G
C G
C G
Y R
G C
A U
C G
R C
A U
C G
G C
A U
5´
UAU UCGUR UCGA

HMP2_9609_1

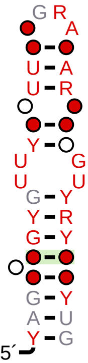

HMP2_9610_1

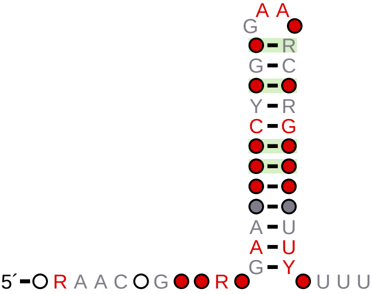

HMP2_9614_1

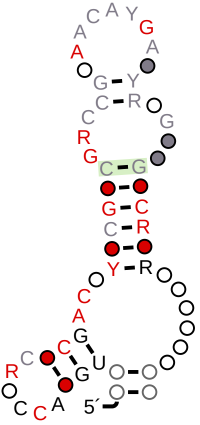

HMP2_9618_1

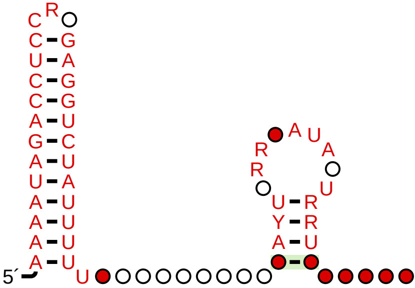

HMP2_962_1

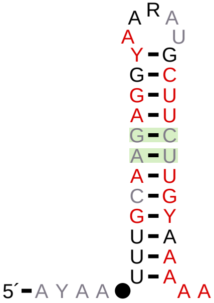

HMP2_9620_1

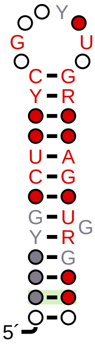

HMP2_9622_1

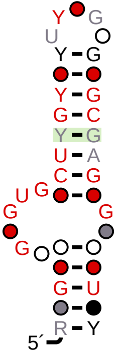

HMP2_9630_1

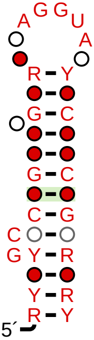

HMP2_9638_1

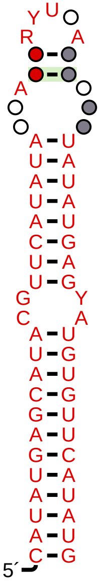

U
Y
A
R
A
A U
U A
A U
U A
A U
C G
U A
U G
G
Y
C
A
A U
U G
A U
C G
G U
A U
G C
U A
A U
U A
A U
C G
5´

HMP2_9643_1

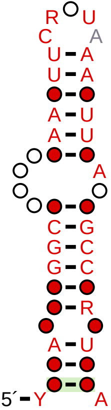

HMP2_965_1

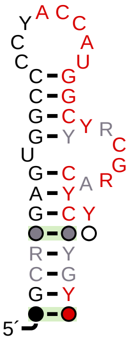

HMP2_9652_1

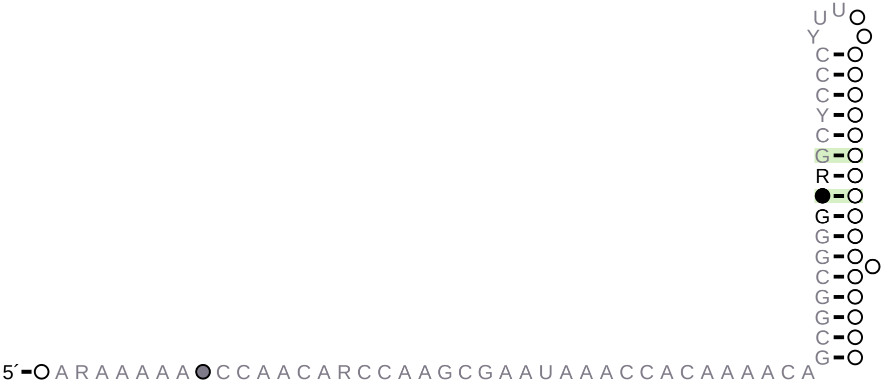
5´
A R A A A A A
C C A A C A R C C A A G C G A A U A A A C C A C A A A A C A
U U
Y
C C C Y C G R
G G G C G G C G

HMP2_9654_1

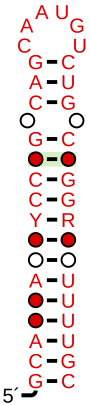

HMP2_9657_1

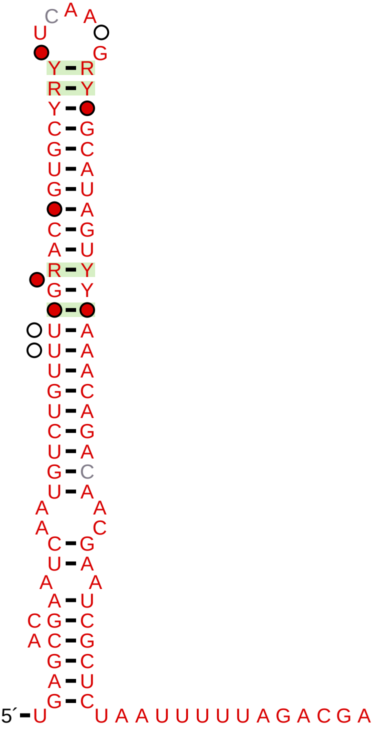

HMP2_9662_1

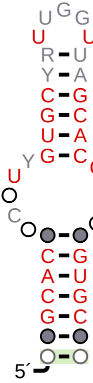

HMP2_9664_1

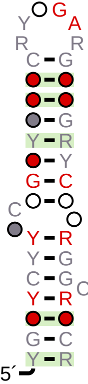

HMP2_9666_1

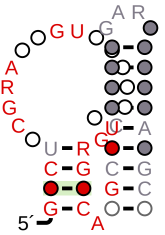

HMP2_9668_1

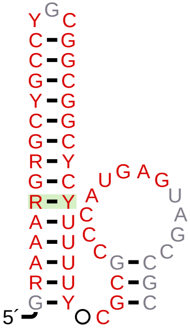

HMP2_9679_1

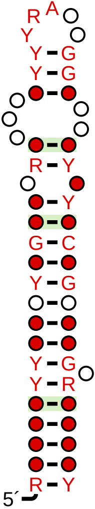

HMP2_9688_1

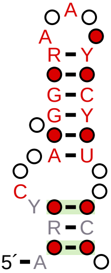

HMP2_9690_1

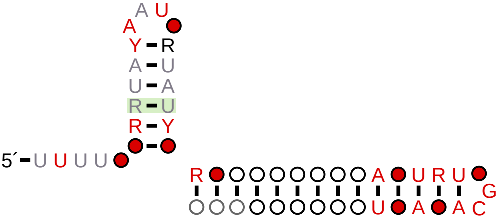

HMP2_9695_1

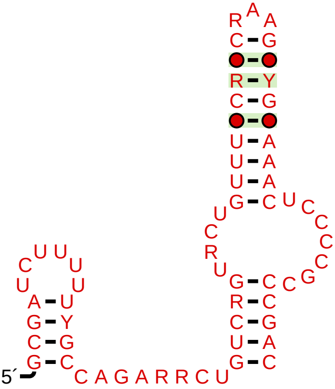

HMP2_9696_1

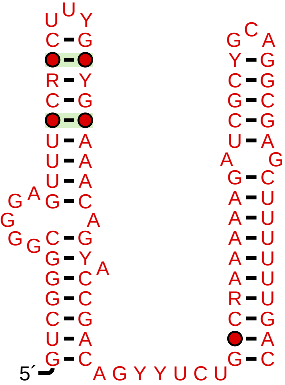

HMP2_9698_1

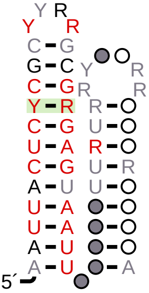

HMP2_970_1

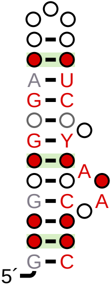

HMP2_9700_1

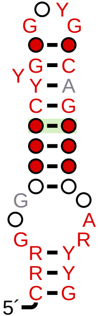

HMP2_971_1

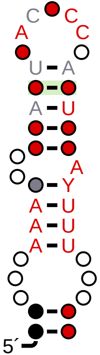

HMP2_9711_1

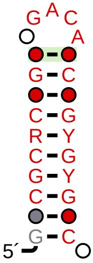

HMP2_9712_1

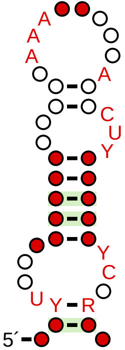

HMP2_9725_1

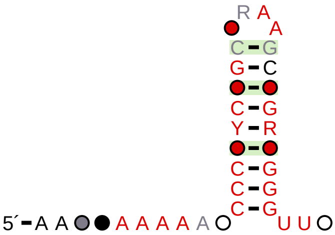

HMP2_9726_1

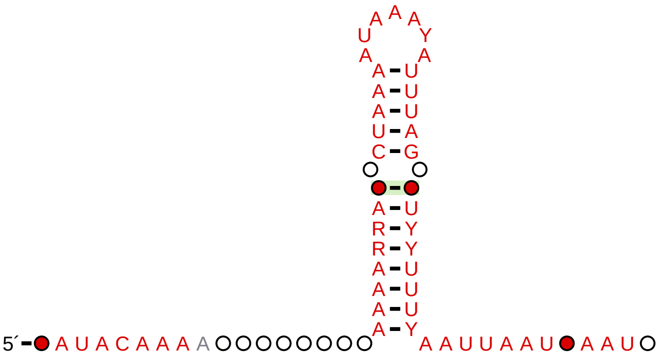
5´
A U A C A A A A
A A U U A A U A A U
A A A U Y A A
A A A U C
U U U A G
A R R A A A A
U Y Y U U U Y

HMP2_9733_1

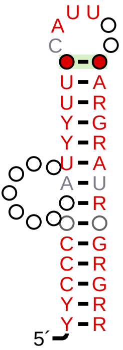

HMP2_9736_1

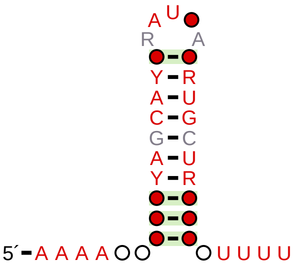

HMP2_9738_1

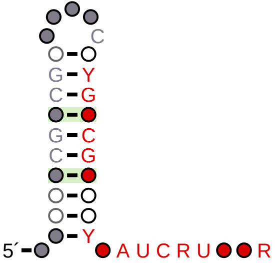

HMP2_9740_1

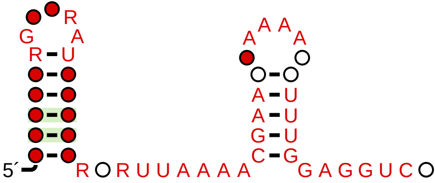

HMP2_9751_1

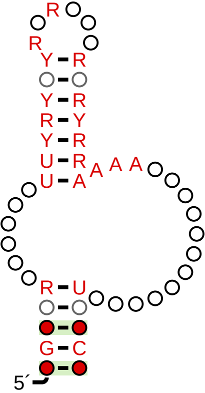

HMP2_9758_1

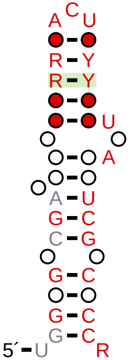

HMP2_9767_1

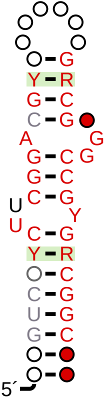

HMP2_9769_1

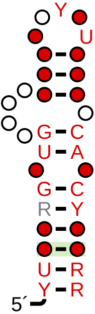

HMP2_977_1

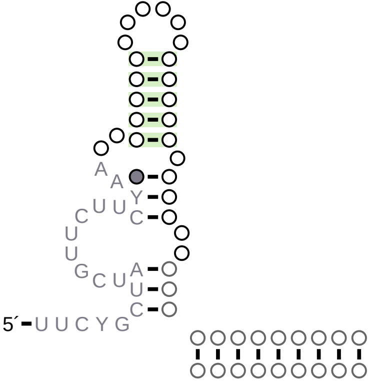

HMP2_9779_1

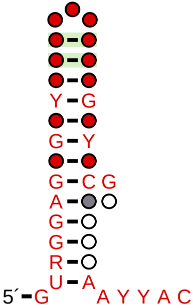

HMP2_9784_1

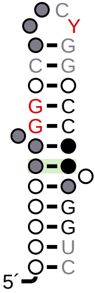

HMP2_9785_1

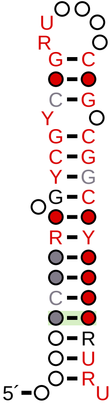

HMP2_9791_1

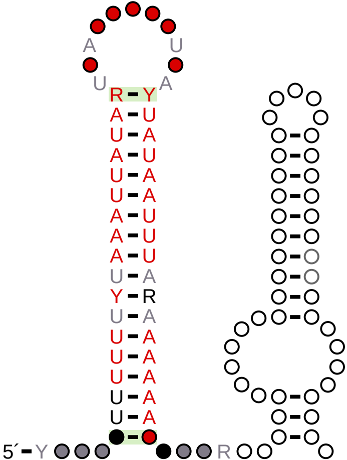

HMP2_9792_1

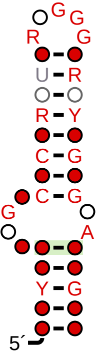

HMP2_9805_1

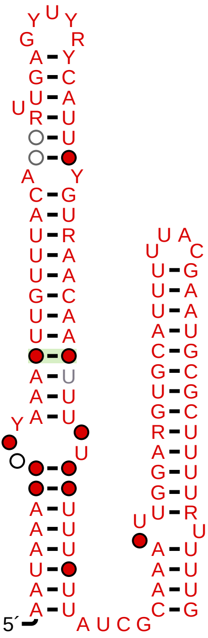

HMP2_9806_1

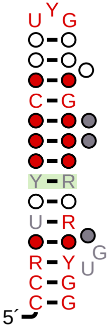

HMP2_981_1

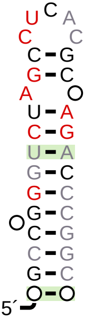

HMP2_9817_1

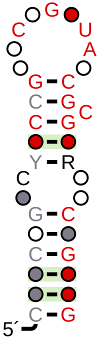

HMP2_9818_1

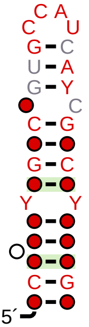

HMP2_9820_1

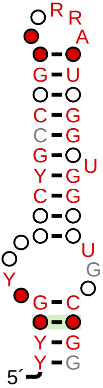

HMP2_9822_1

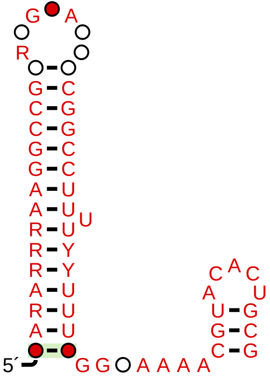

HMP2_9823_1

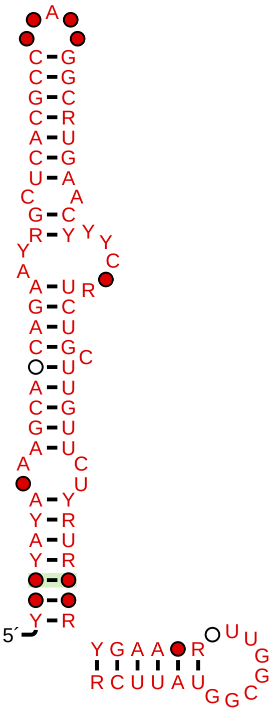

HMP2_9848_1

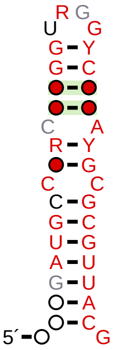

HMP2_985_1

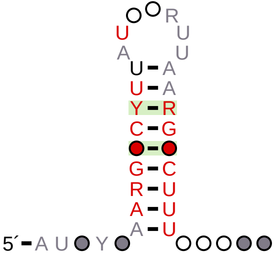

HMP2_9850_1

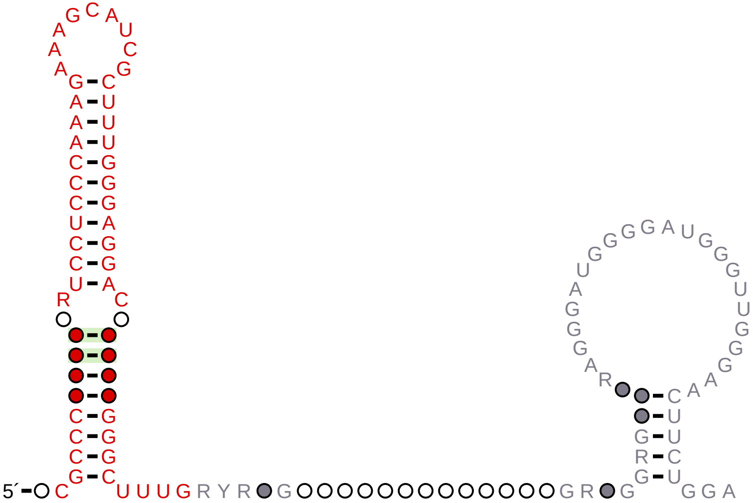

HMP2_9855_1

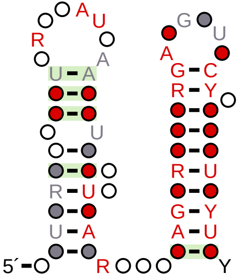

HMP2_9856_1

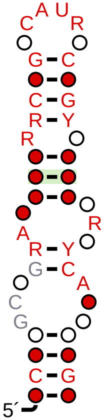

HMP2_9857_1

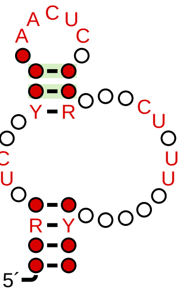

HMP2_9861_1

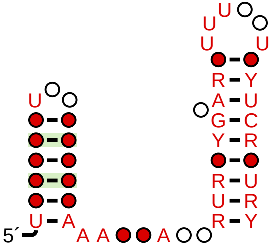

HMP2_9882_1

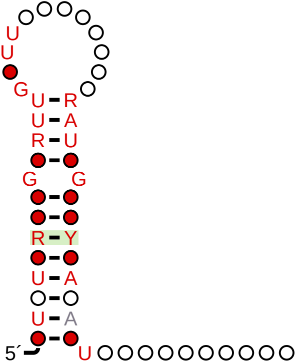

HMP2_9883_1

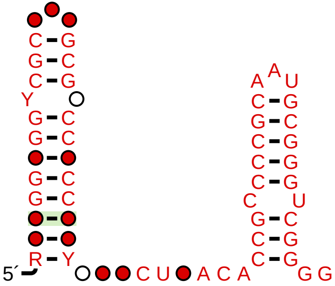

HMP2_9889_1

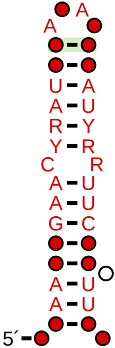

HMP2_9892_1

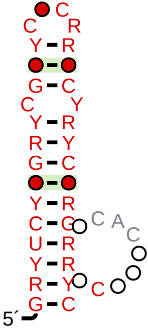

HMP2_9893_1

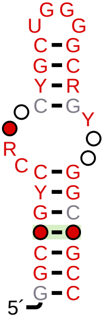

HMP2_9895_1

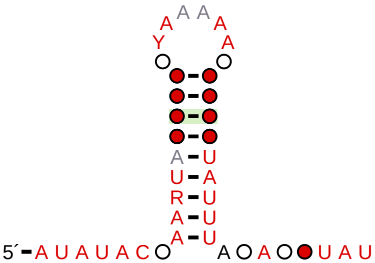

HMP2_9898_1

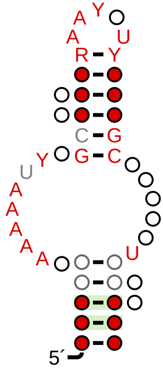

HMP2_99_1

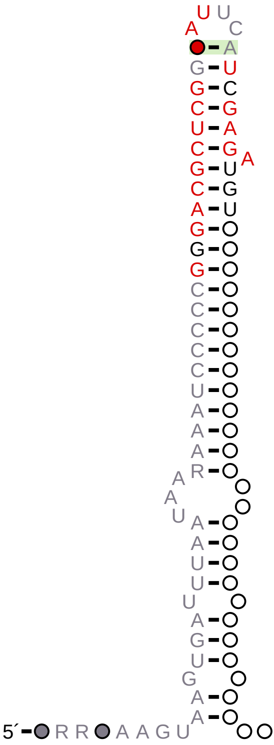

HMP2_9905_1

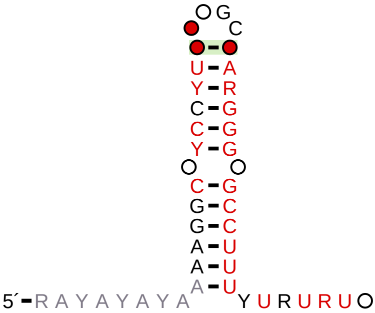

HMP2_9910_1

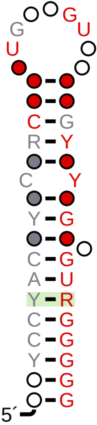

HMP2_9911_1

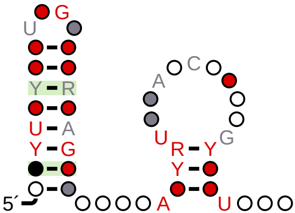

HMP2_9918_1

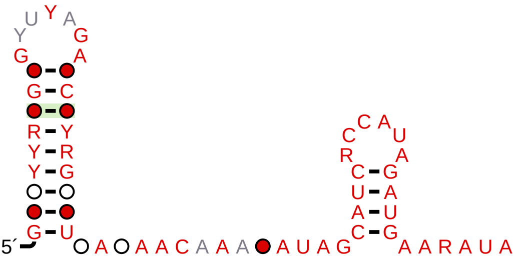

HMP2_992_1

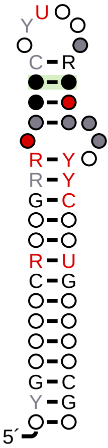

HMP2_9923_1

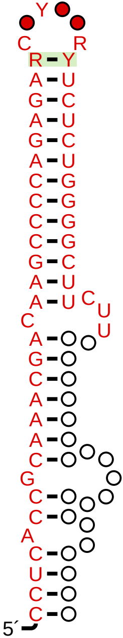

HMP2_9930_1

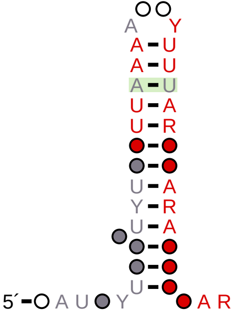

HMP2_9931_1

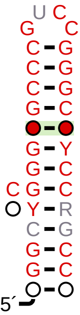

HMP2_9956_1

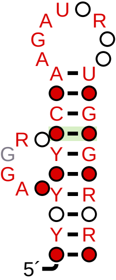

HMP2_9957_1

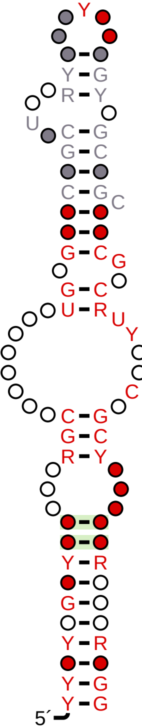

HMP2_9960_1

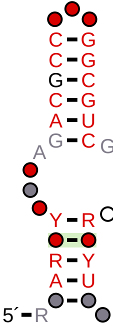

HMP2_9965_1

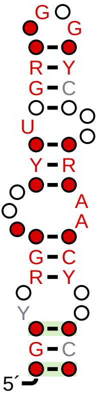

HMP2_997_1

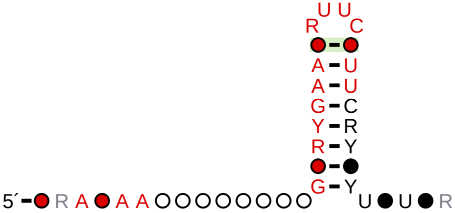

HMP2_9974_1

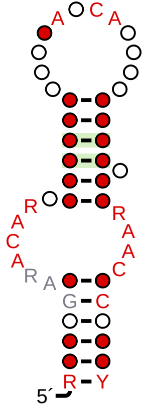

HMP2_9978_1

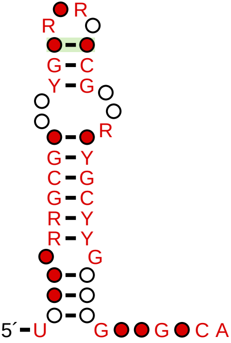

HMP2_9981_1

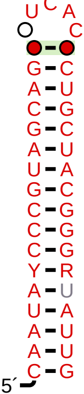

HMP2_9988_1

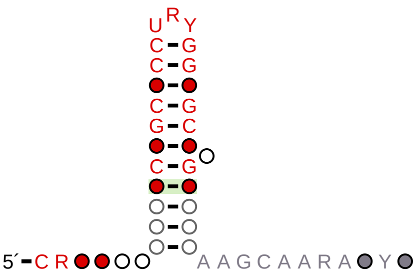

HMP2_9990_1

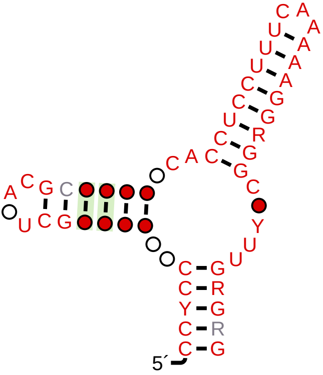

HMP2_9993_1

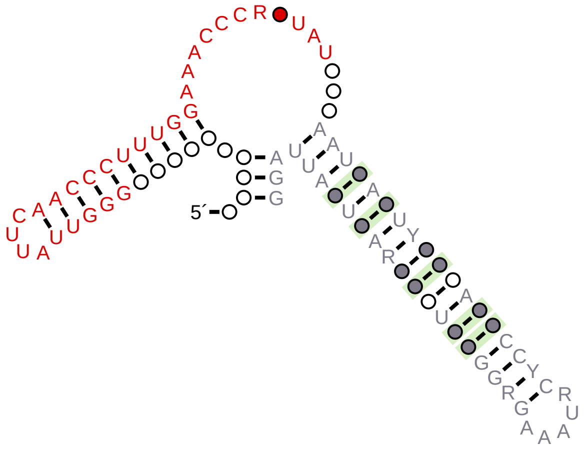

HMP2_9997_1

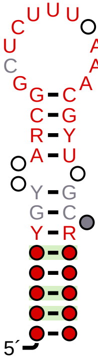

Supplement: Supplementary file 6 — Additional file 6. Visualization of the 3161 candidate structured RNAs. We show R2R [36] visualizations of the 3161 candidate structured RNAs as a pdf. [file 13059_2021_2319_MOESM6_ESM.pdf]
