# Supplementary material for: Comparative genomics identifies thousands of candidate structured RNAs in human microbiomes
Source: Genome Biol. 2021 Apr 12;22:100. doi: 10.1186/s13059-021-02319-w (PMC8040213; doi:10.1186/s13059-021-02319-w)

HMP2_1002_1

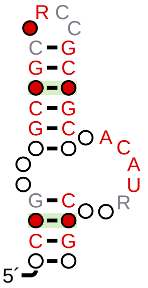

HMP2_10030_1

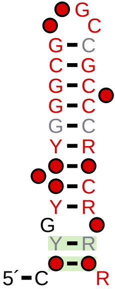

HMP2_10046_1

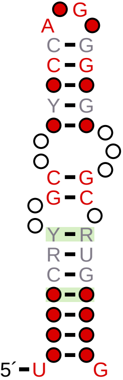

HMP2_10049_1

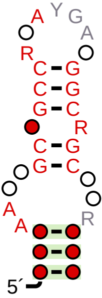

HMP2_10053_1

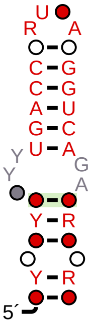

HMP2_10063_1

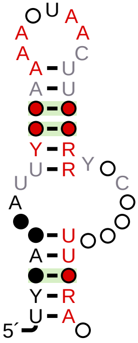

HMP2_10071_1

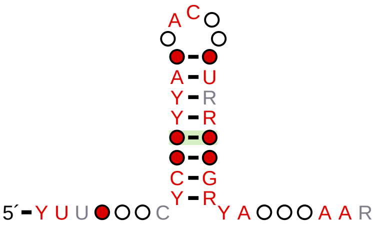

HMP2_10078_1

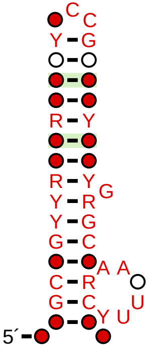

HMP2_10087_1

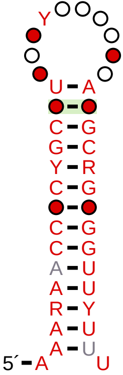

HMP2_1009_1

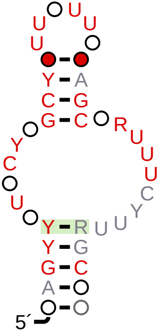

HMP2_10095_1

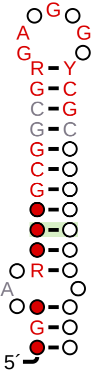

HMP2_10108_1

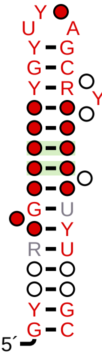

HMP2_10116_1

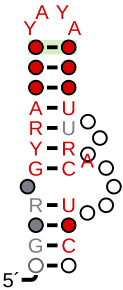

HMP2_1012_1

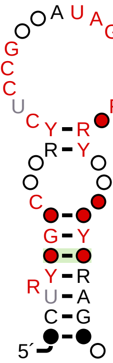

HMP2_10126_1

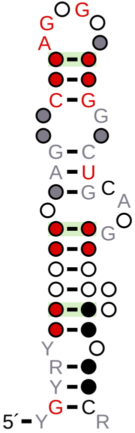

HMP2_10131_1

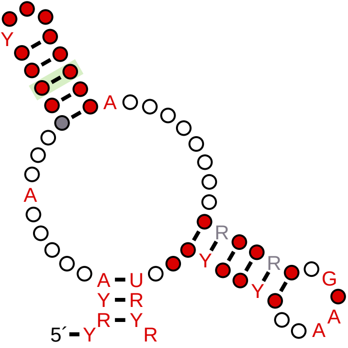

HMP2_10132_1

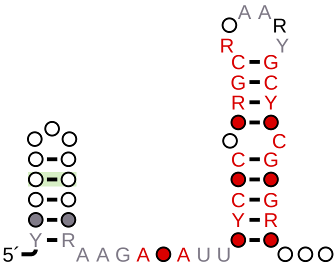

HMP2_10133_1

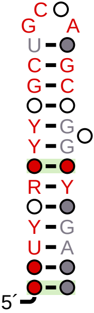

HMP2_10135_1

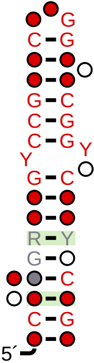

HMP2_10149_1

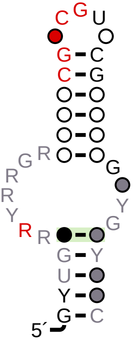

HMP2_10155_1

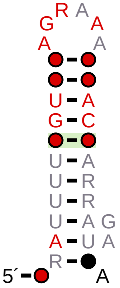

HMP2_10161_1

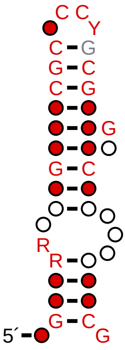

HMP2_10171_1

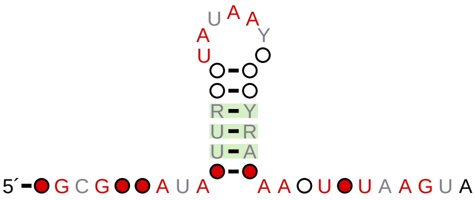

HMP2_10172_1

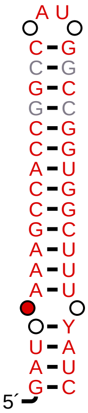

HMP2_10178_1

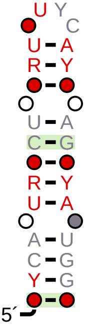

HMP2_10190_1

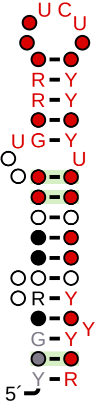

HMP2_10193_1

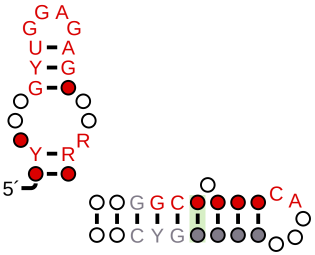

HMP2_10206_1

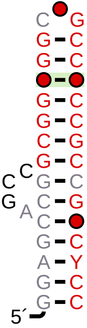

HMP2_10211_1

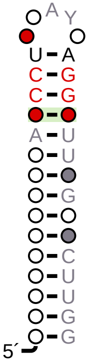

HMP2_10223_1

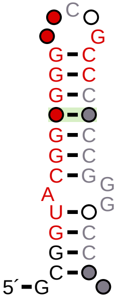

HMP2_10227_1

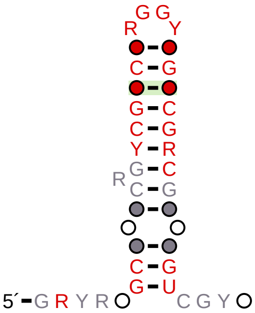

HMP2_10244_1

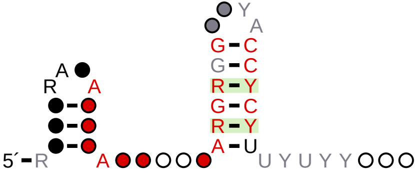

HMP2_10248_1

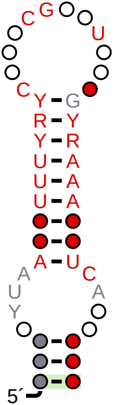

HMP2_10250_1

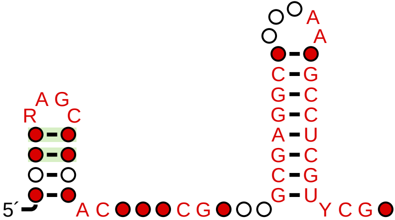

HMP2_10259_1

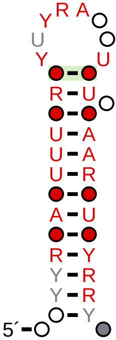

HMP2_10260_1

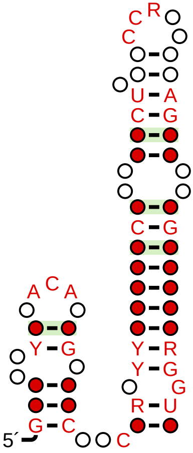

HMP2_10261_1

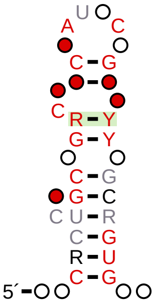

HMP2_10262_1

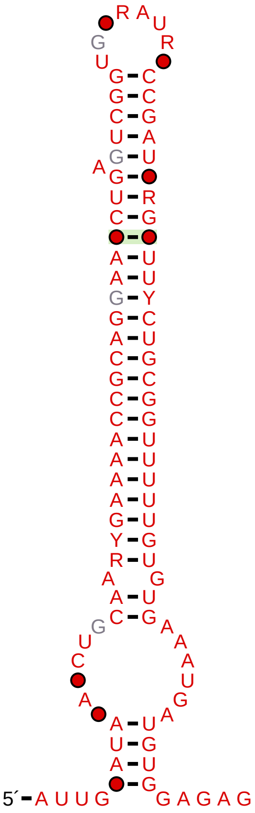

HMP2_10276_1

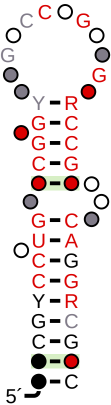

HMP2_10282_1

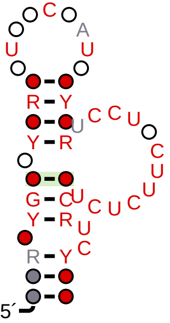

HMP2_10291_1

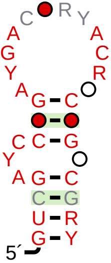

HMP2_10298_1

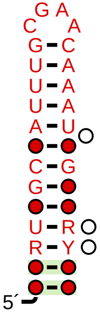

HMP2_10299_1

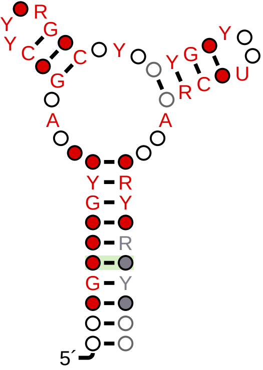

HMP2_10306_1

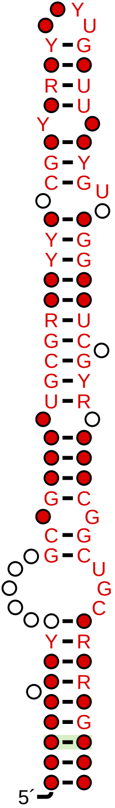

HMP2_1031_1

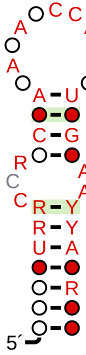

HMP2_10323_1

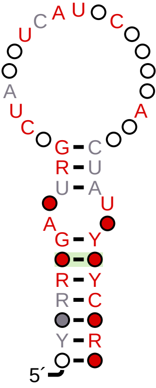

HMP2_10330_1

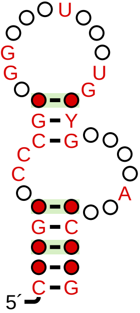

HMP2_10335_1

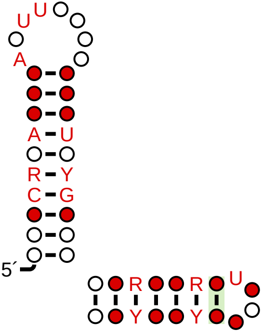

HMP2_10338_1

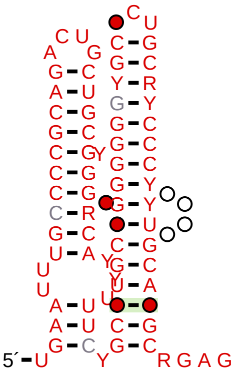

HMP2_10344_1

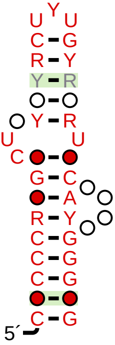

HMP2_10347_1

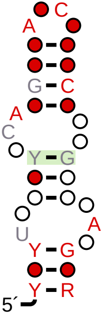

HMP2_10367_1

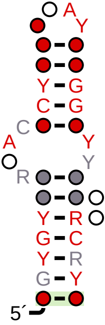

HMP2_10375_1

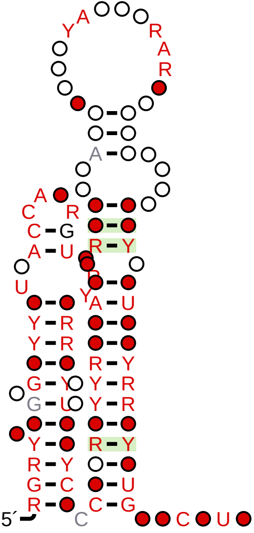

HMP2_10379_1

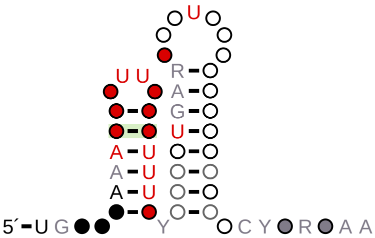

HMP2_10383_1

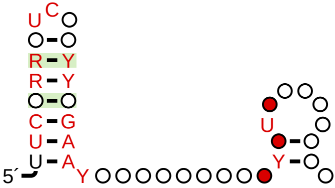

HMP2_10387_1

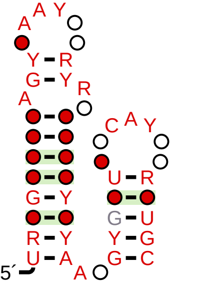

HMP2_10392_1

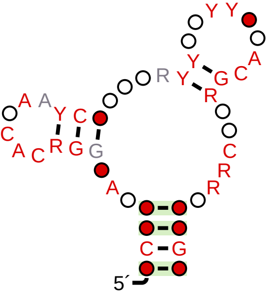

HMP2_10395_1

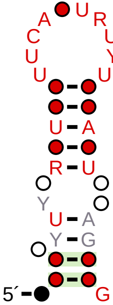

HMP2_10397_1

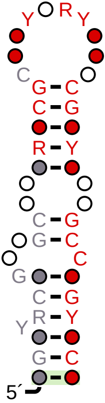

HMP2_10403_1

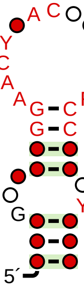

HMP2_10408_1

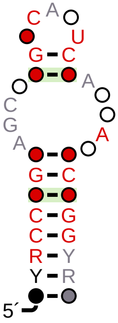

HMP2_10416_1

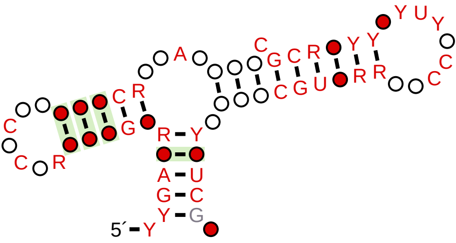

HMP2_10429_1

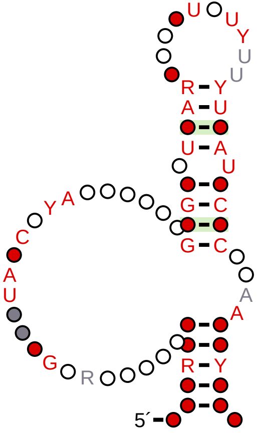

HMP2_10442_1

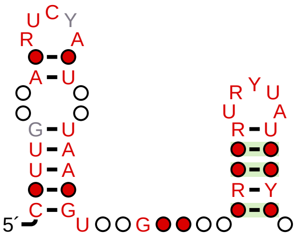

HMP2_10444_1

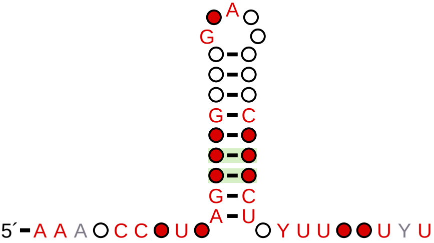

HMP2_1045_1

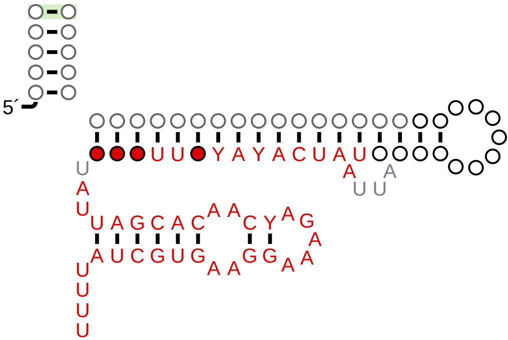

HMP2_10464_1

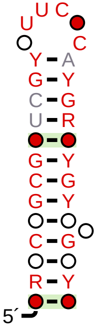

HMP2_1047_1

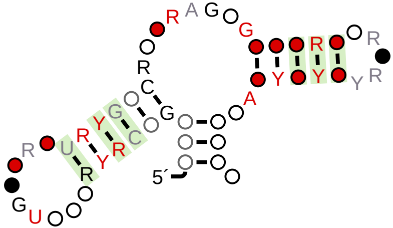

HMP2_10475_1

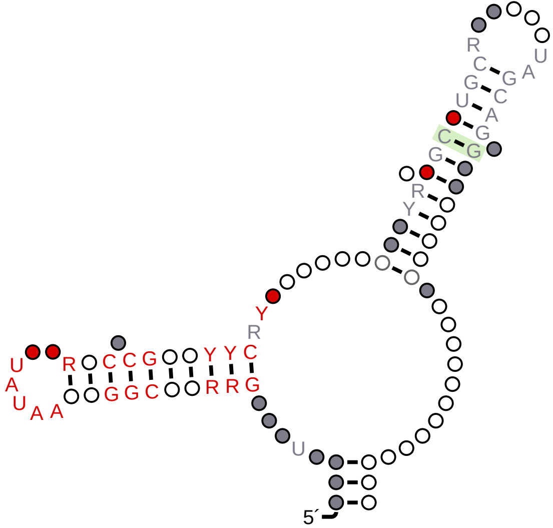

HMP2_10485_1

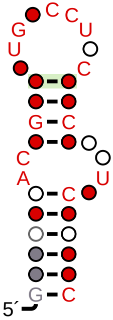

HMP2_10488_1

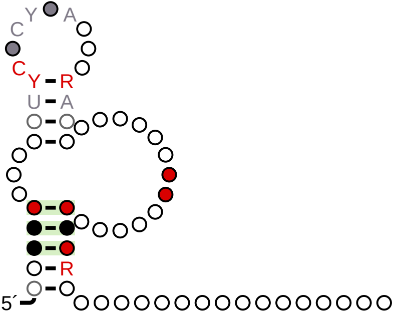

HMP2_10492_1

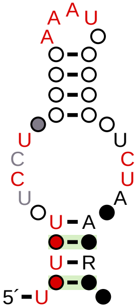

HMP2_10501_1

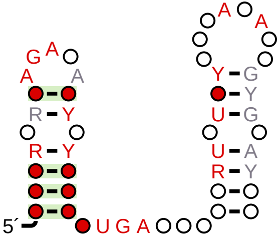

HMP2_10506_1

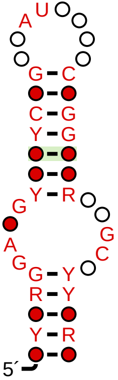

HMP2_10508_1

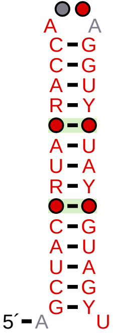

HMP2_10516_1

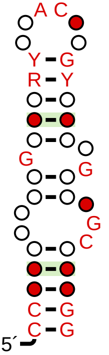

HMP2_1052_1

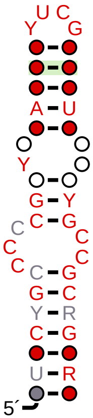

HMP2_10526_1

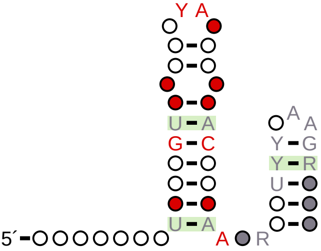

HMP2_10531_1

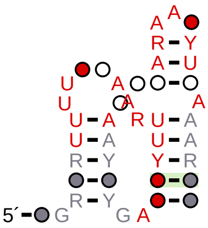

HMP2_10542_1

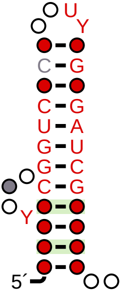

HMP2_10546_1

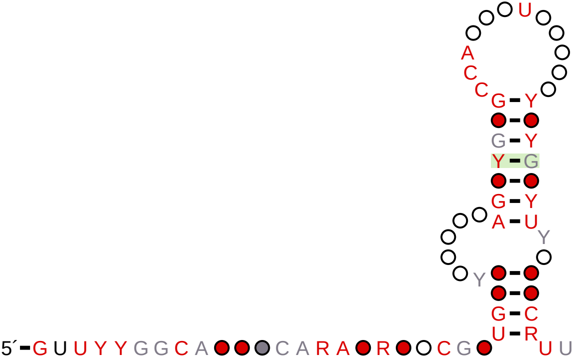

HMP2_10550_1

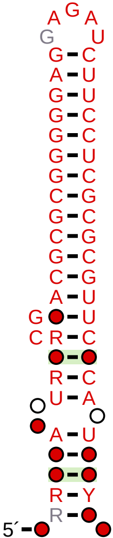

HMP2_10555_1

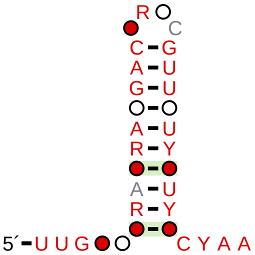

HMP2_1056_1

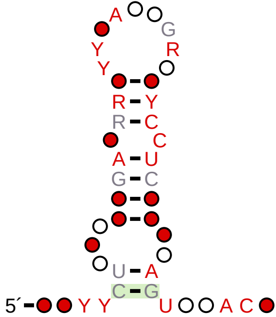

HMP2_10560_1

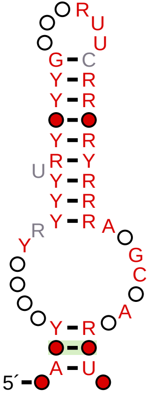

HMP2_10566_1

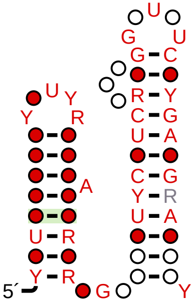

HMP2_1057_1

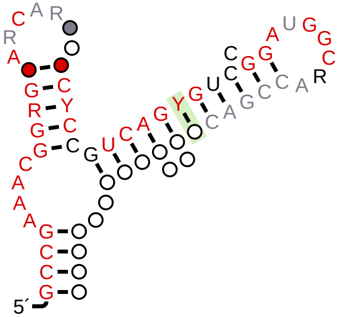

HMP2_10571_1

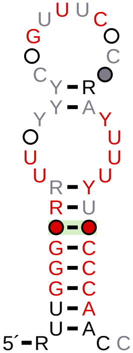

HMP2_10578_1

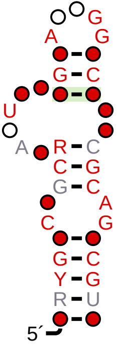

HMP2_10581_1

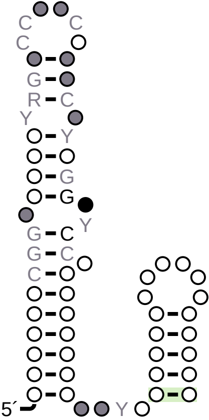

HMP2_10586_1

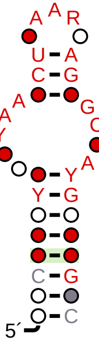

HMP2_10602_1

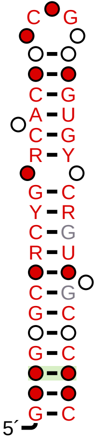

HMP2_10635_1

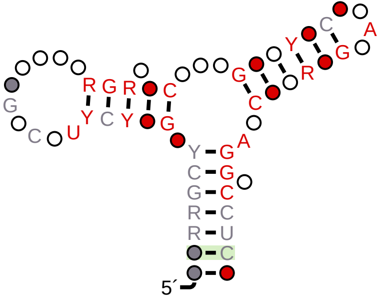

HMP2_1064_1

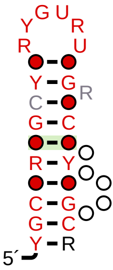

HMP2_10647_1

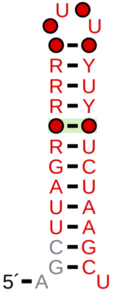

HMP2_10652_1

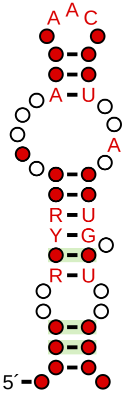

HMP2_10657_1

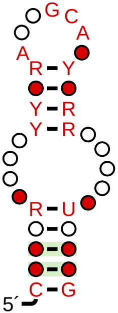

HMP2_10662_1

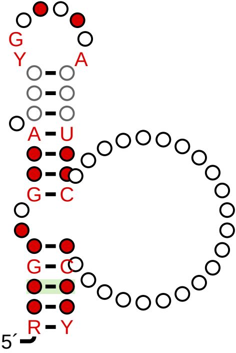

HMP2_10670_1

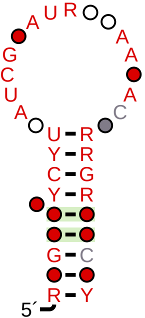

HMP2_10671_1

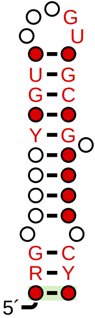

HMP2_10674_1

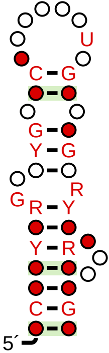

HMP2_10675_1

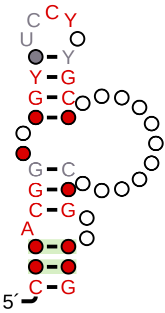

HMP2_10686_1

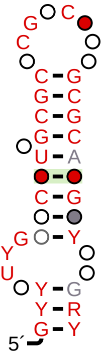

HMP2_10697_1

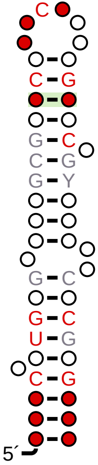

HMP2_10715_1

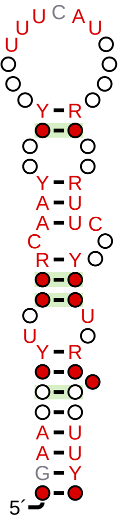

HMP2_10716_1

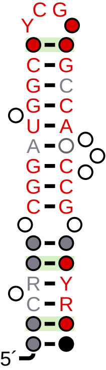

HMP2_10727_1

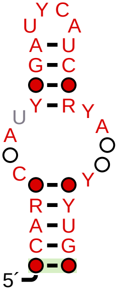

HMP2_10732_1

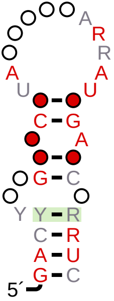

HMP2_10742_1

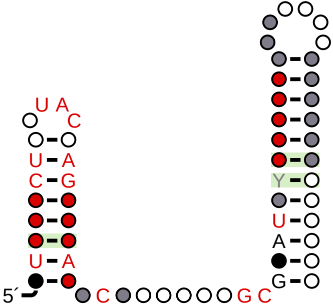

HMP2_10752_1

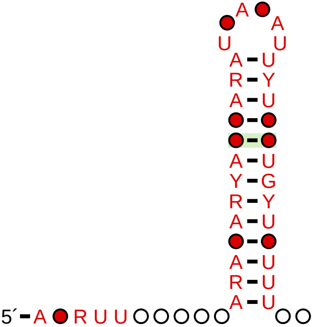

HMP2_10753_1

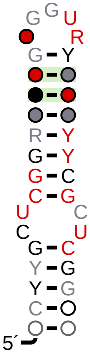

HMP2_10754_1

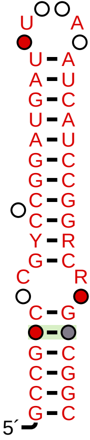

HMP2_10757_1

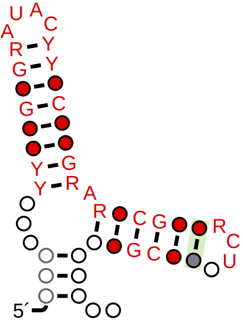

HMP2_10760_1

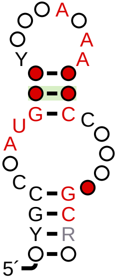

HMP2_10779_1

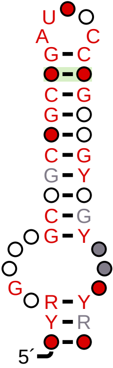

HMP2_10781_1

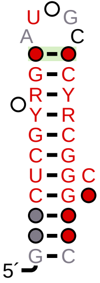

HMP2_10785_1

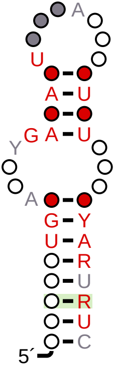

HMP2_10792_1

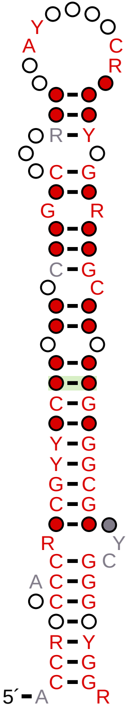

HMP2_10796_1

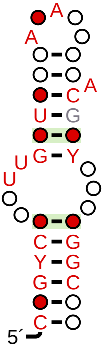

HMP2_10801_1

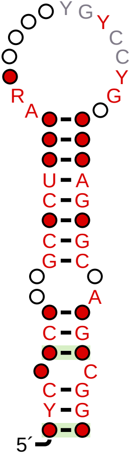

HMP2_10802_1

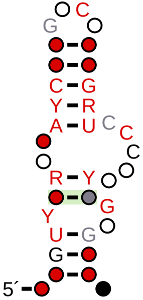

HMP2_10810_1

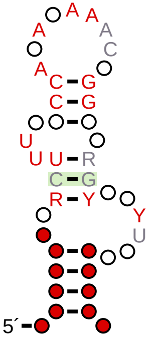

HMP2_10818_1

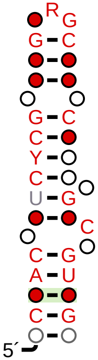

HMP2_10836_1

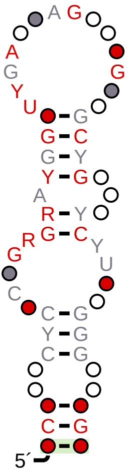

HMP2_10837_1

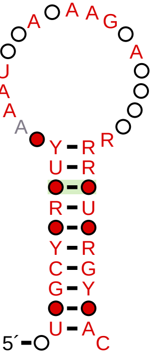

HMP2_10840_1

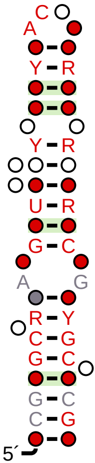

HMP2_10847_1

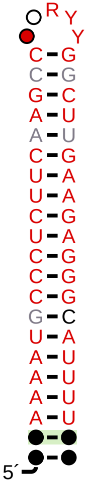

HMP2_1085_1

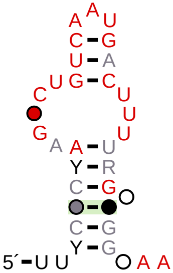

HMP2_10851_1

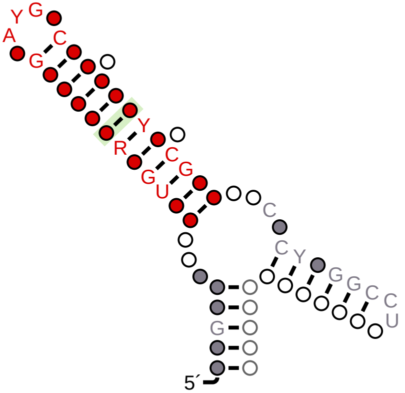

HMP2_10867_1

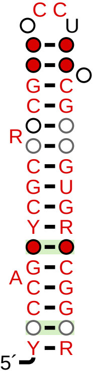

HMP2_10884_1

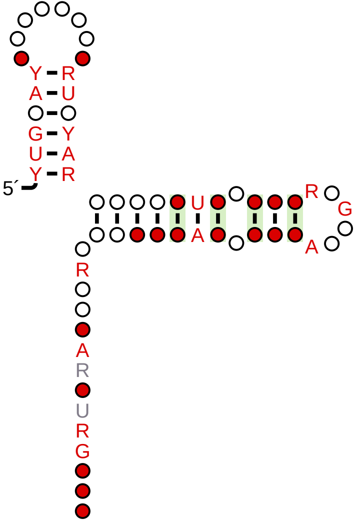

HMP2_10888_1

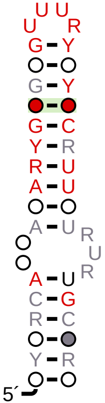

HMP2_10894_1

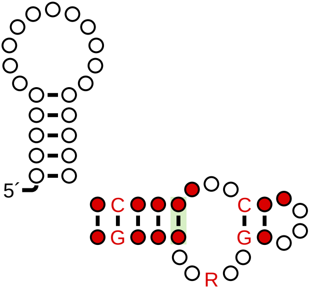

HMP2_10896_1

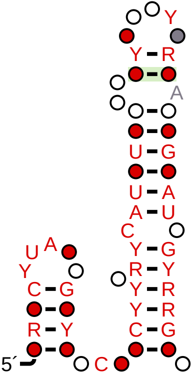

HMP2_10899_1

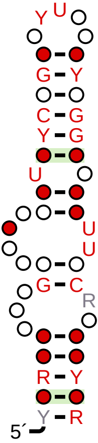

HMP2_10903_1

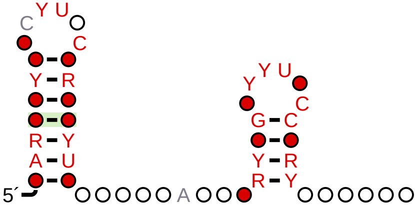

HMP2_1091_1

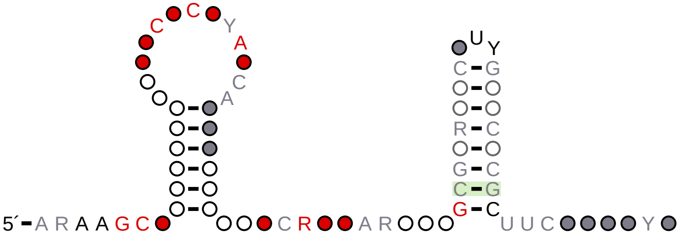

HMP2_10911_1

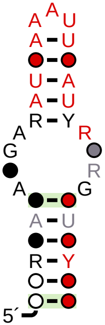

HMP2_10921_1

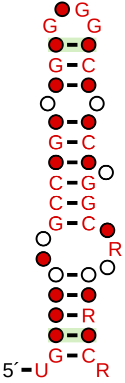

HMP2_10925_1

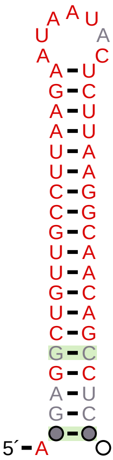

HMP2_10931_1

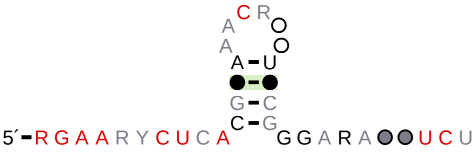

HMP2_10934_1

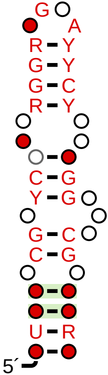

HMP2_10962_1

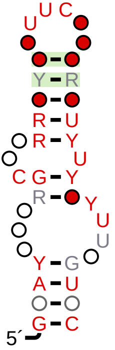

HMP2_10976_1

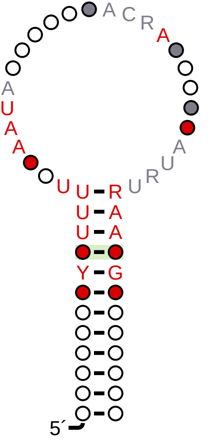

HMP2_10977_1

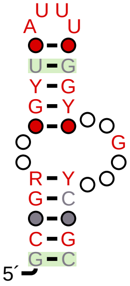

HMP2_11006_1

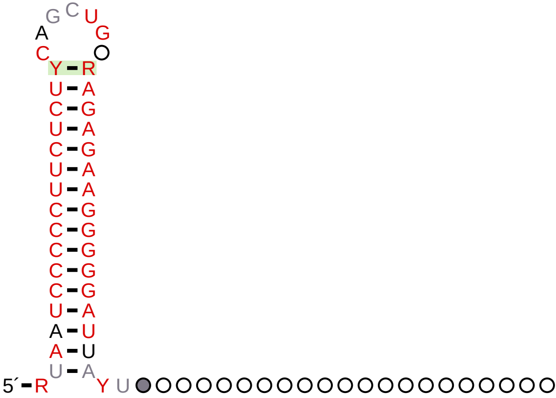

HMP2_11010_1

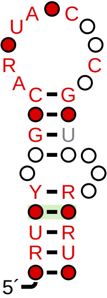

HMP2_11011_1

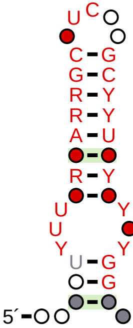

HMP2_11018_1

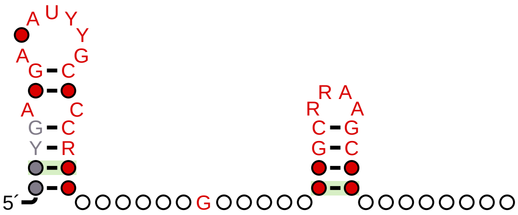

HMP2_11029_1

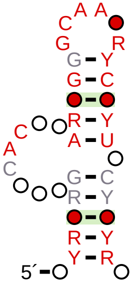

HMP2_11049_1

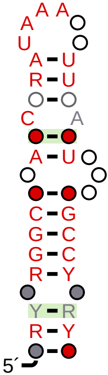

HMP2_11058_1

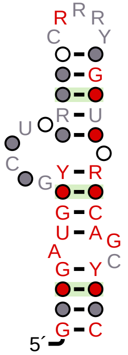

HMP2_11061_1

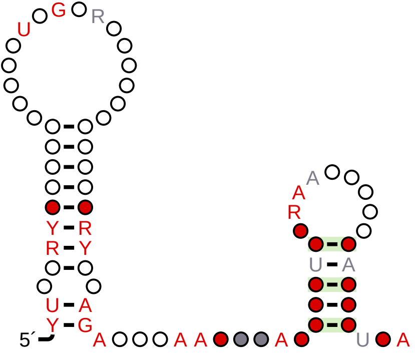

HMP2_11069_1

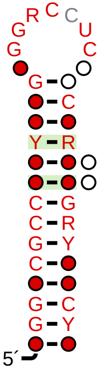

HMP2_11073_1

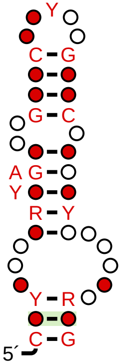

HMP2_1108_1

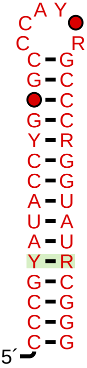

HMP2_11094_1

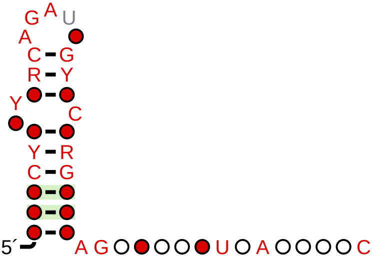

HMP2_11097_1

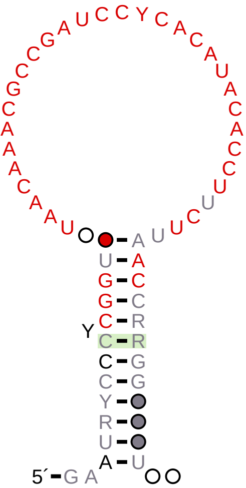

HMP2_11113_1

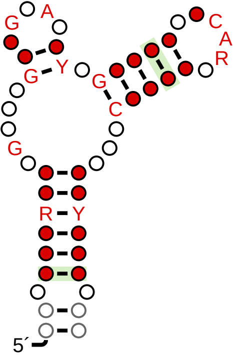

HMP2_11115_1

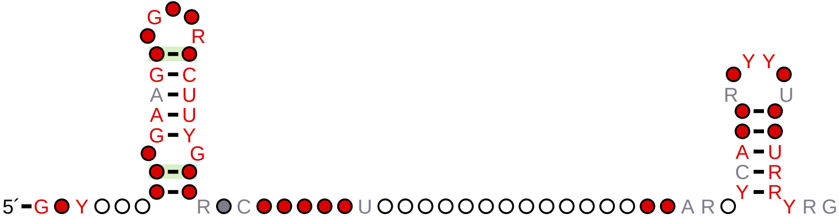

HMP2_11118_1

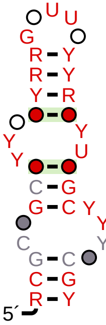

HMP2_11121_1

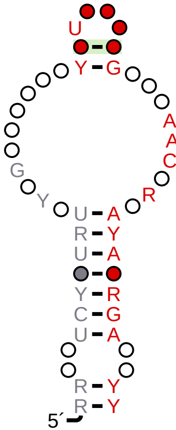

HMP2_11148_1

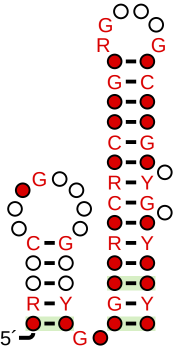

HMP2_11164_1

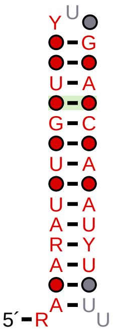

HMP2_11165_1

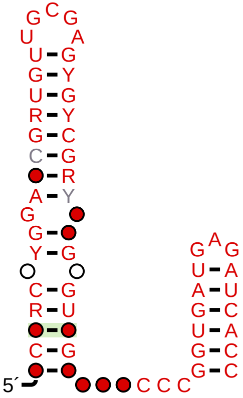

HMP2_11168_1

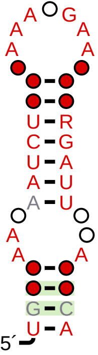

HMP2_11169_1

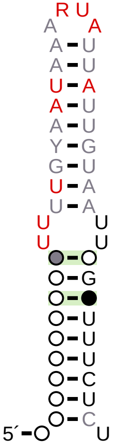

HMP2_11176_1

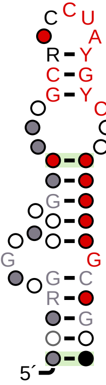

HMP2_11182_1

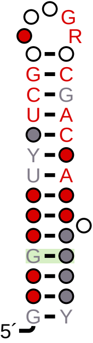

HMP2_11196_1

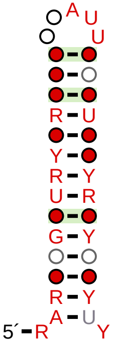

HMP2_11214_1

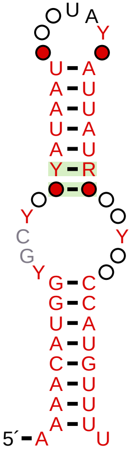

HMP2_11215_1

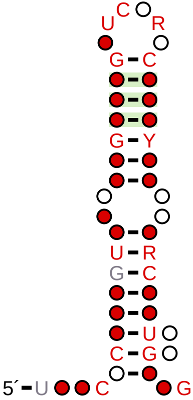

HMP2_11216_1

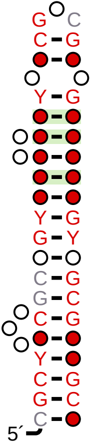

HMP2_11222_1

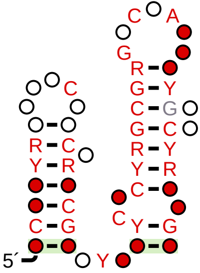

HMP2_11223_1

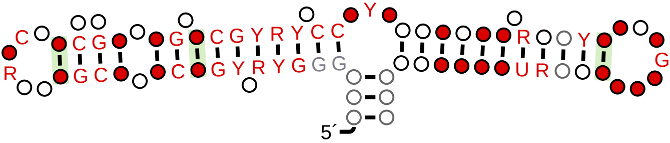

HMP2_11229_1

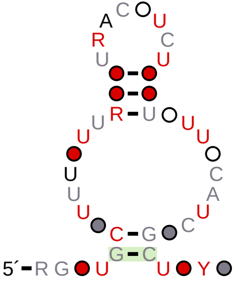

HMP2_11232_1

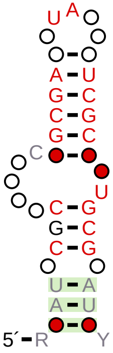

HMP2_11233_1

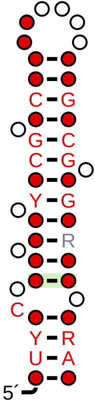

HMP2_11234_1

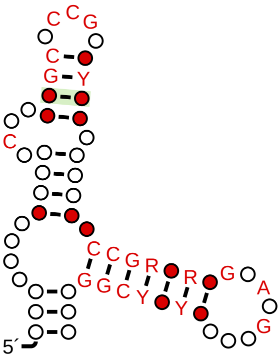

HMP2_11242_1

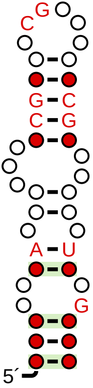

HMP2_11244_1

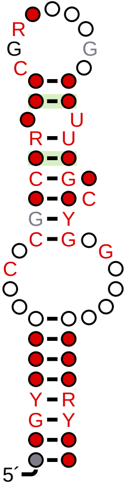

HMP2_11257_1

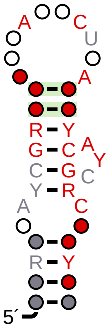

HMP2_11274_1

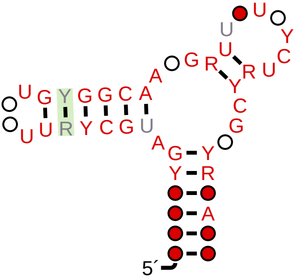

HMP2_11284_1

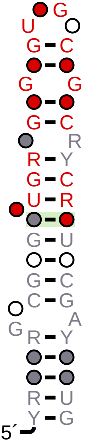

HMP2_11285_1

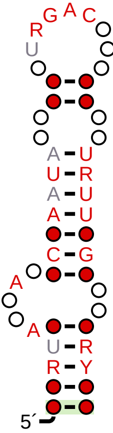

HMP2_11298_1

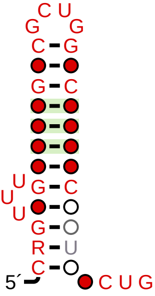

HMP2_11316_1

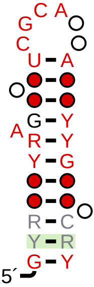

HMP2_11332_1

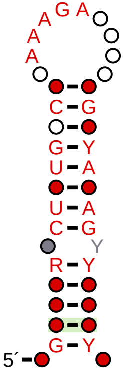

HMP2_11340_1

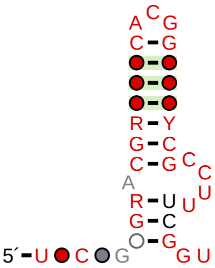

HMP2_11354_1

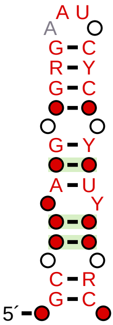

HMP2_11355_1

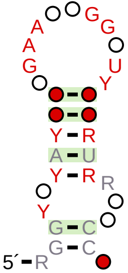

HMP2_11359_1

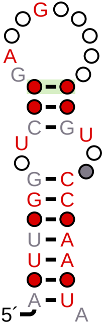

HMP2_1138_1

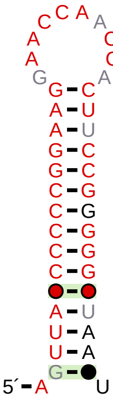

HMP2_11391_1

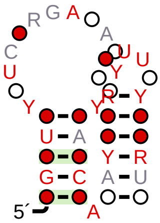

HMP2_11396_1

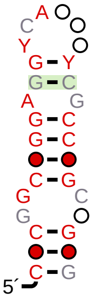

HMP2_114_1

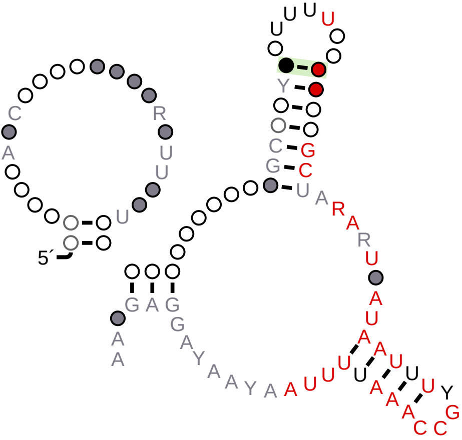

HMP2_11410_1

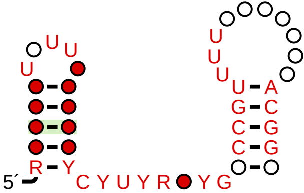

HMP2_11423_1

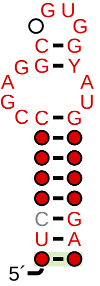

HMP2_11429_1

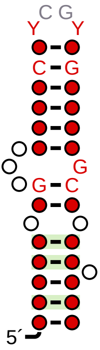

HMP2_11448_1

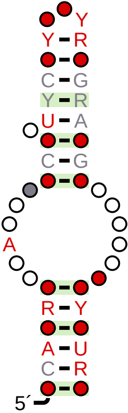

HMP2_11449_1

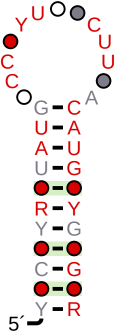

HMP2_11456_1

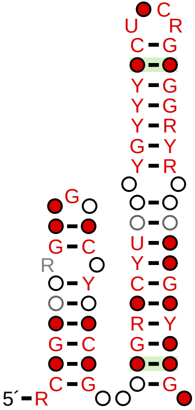

HMP2_11471_1

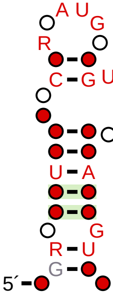

HMP2_11476_1

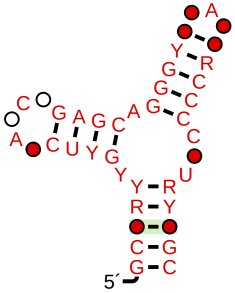

HMP2_11479_1

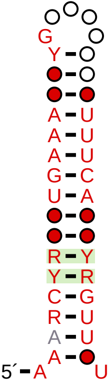

HMP2_11502_1

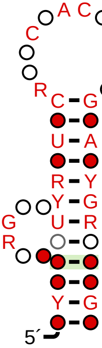

HMP2_11519_1

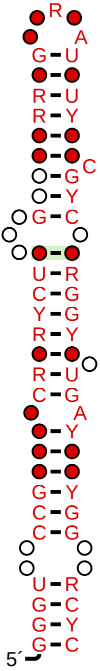

HMP2_11521_1

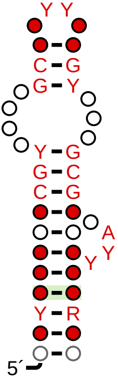

HMP2_11525_1

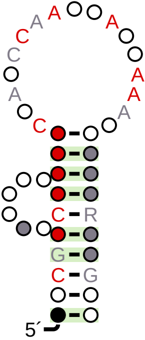

HMP2_11535_1

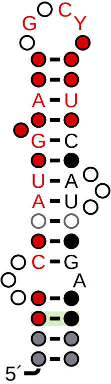

HMP2_11537_1

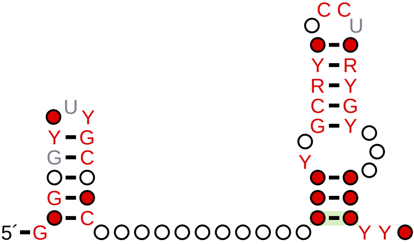

HMP2_11552_1

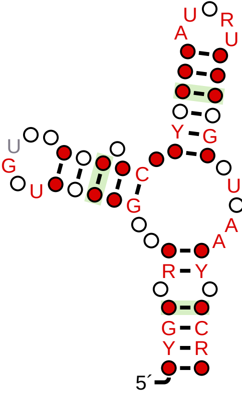

HMP2_11557_1

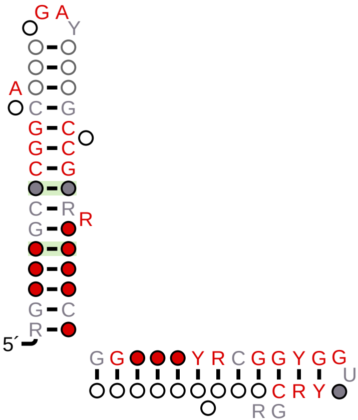

HMP2_11559_1

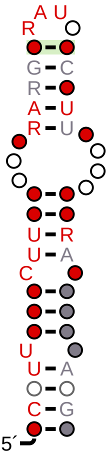

HMP2_11562_1

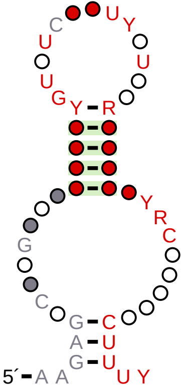

HMP2_11572_1

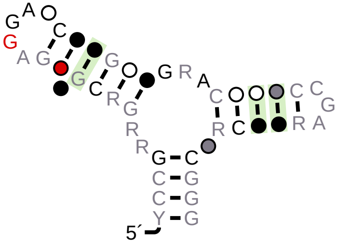

HMP2_11580_1

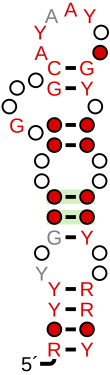

HMP2_11584_1

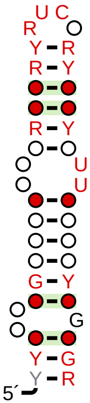

HMP2_11594_1

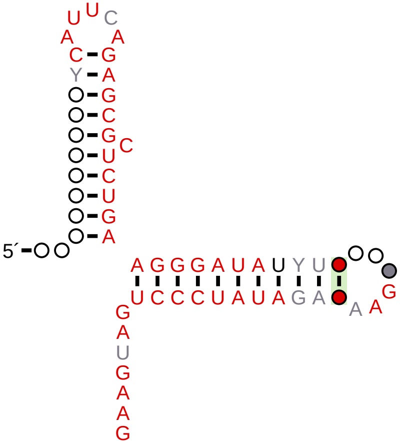

HMP2_1160_1

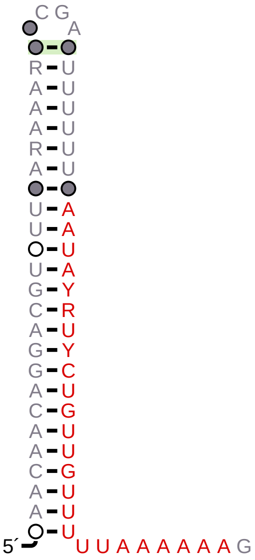

C G
A
R U
A U
A U
A U
R U
A U
U A
U A
U
U A
G Y
C R
A U
G Y
G C
A U
C G
A U
A U
C G
A U
A U
U
5´
U U A A A A A A G

HMP2_11601_1

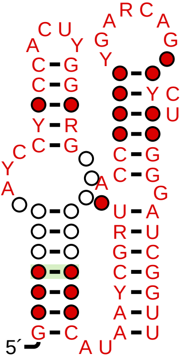

HMP2_11618_1

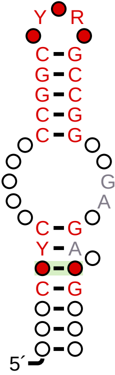

HMP2_11620_1

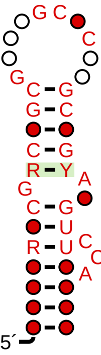

HMP2_11628_1

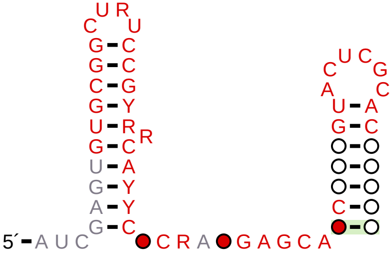

HMP2_11632_1

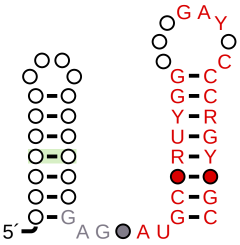

HMP2_11636_1

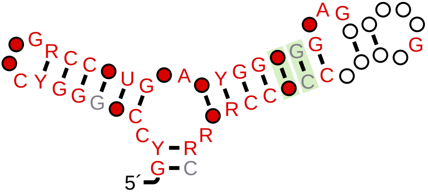

HMP2_11659_1

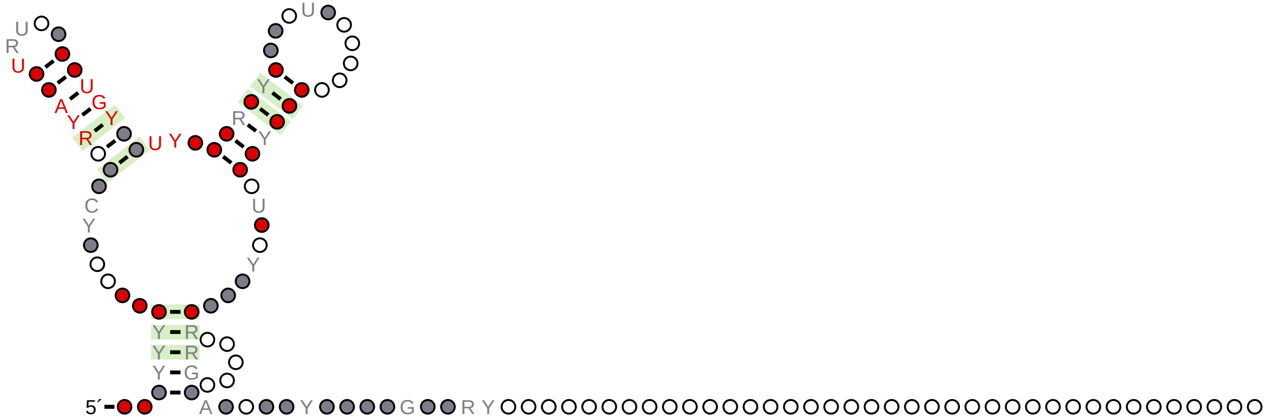

HMP2_11670_1

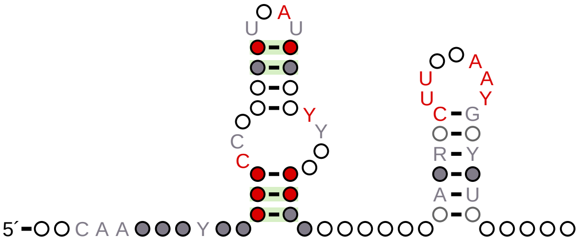

HMP2_11682_1

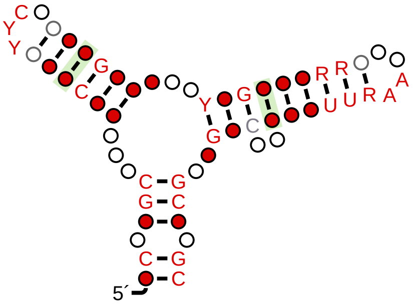

HMP2_1169_1

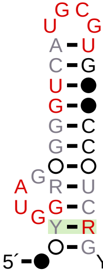

HMP2_11693_1

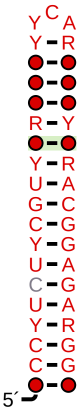

HMP2_11717_1

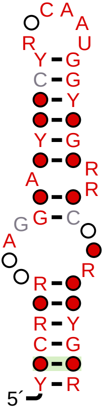

HMP2_11721_1

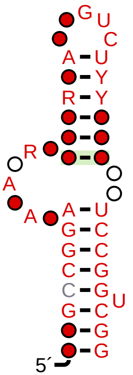

HMP2_11728_1

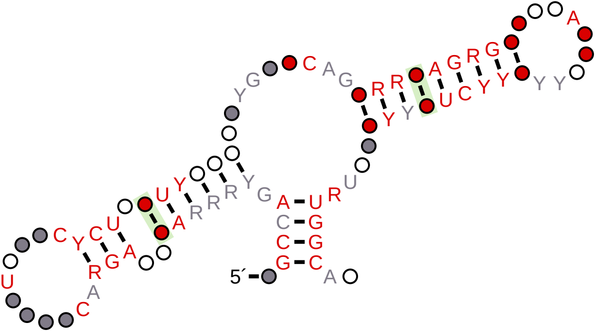

HMP2_11734_1

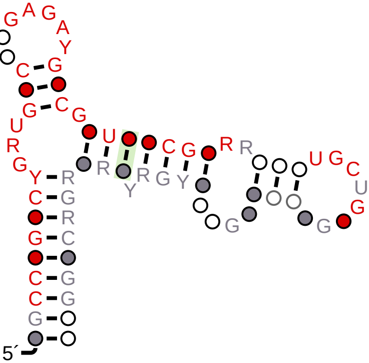

HMP2_1174_1

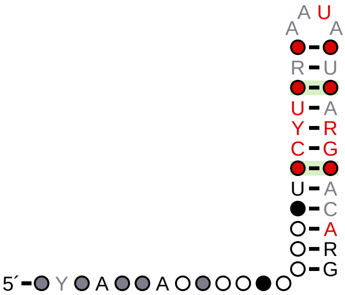

HMP2_11746_1

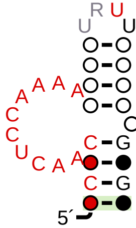

HMP2_11749_1

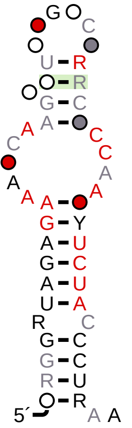

HMP2_11752_1

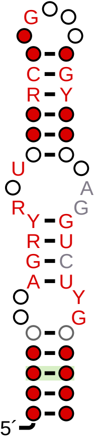

HMP2_11766_1

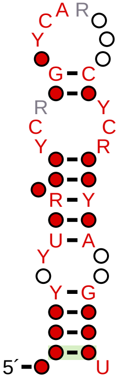

HMP2_11772_1

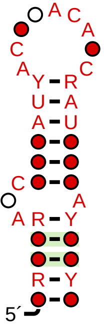

HMP2_11775_1

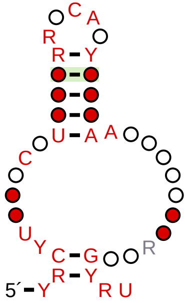

HMP2_11783_1

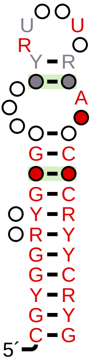

HMP2_1179_1

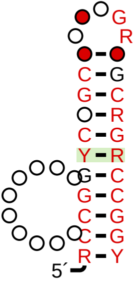

HMP2_11793_1

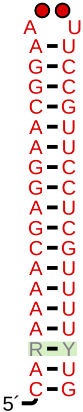

HMP2_11795_1

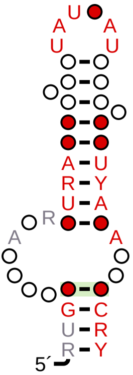

HMP2_11806_1

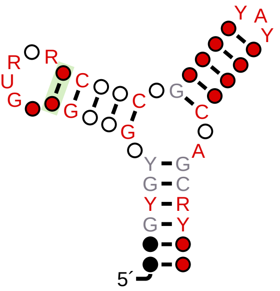

HMP2_11807_1

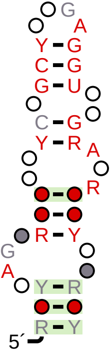

HMP2_11818_1

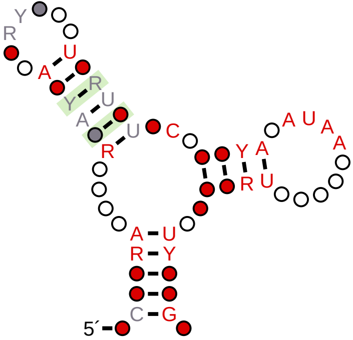

HMP2_11821_1

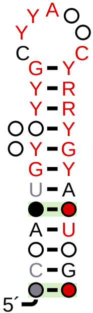

HMP2_11823_1

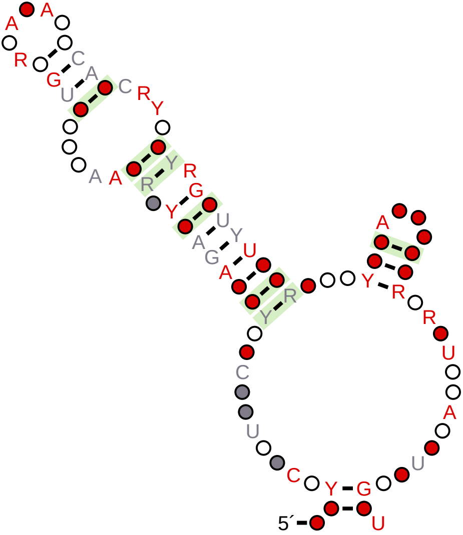

HMP2_11841_1

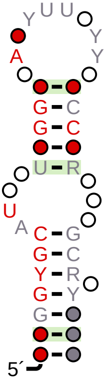

HMP2_11851_1

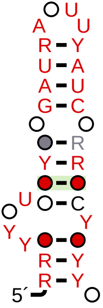

HMP2_11854_1

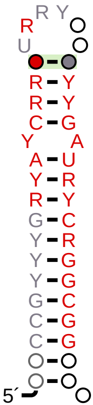

HMP2_11870_1

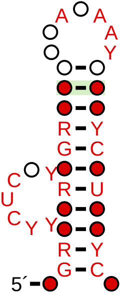

HMP2_11887_1

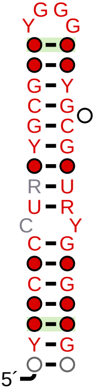

HMP2_1189_1

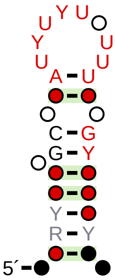

HMP2_11893_1

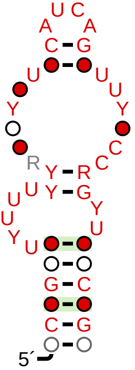

HMP2_11896_1

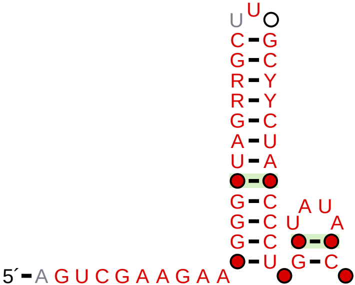

HMP2_11911_1

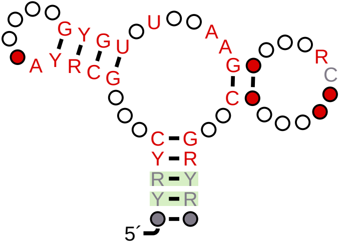

HMP2_11924_1

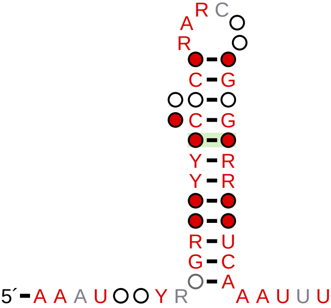

HMP2_11929_1

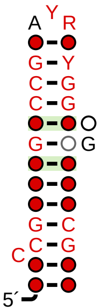

HMP2_1193_1

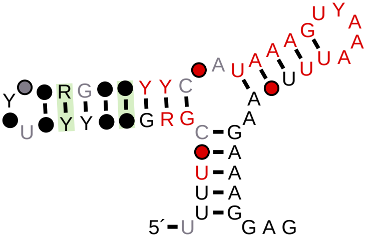

HMP2_11930_1

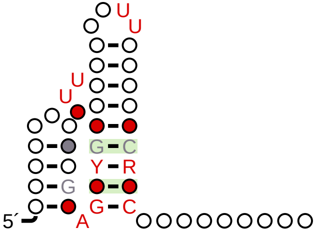

HMP2_11932_1

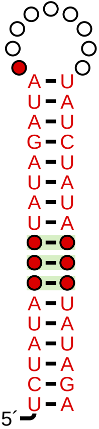

HMP2_11937_1

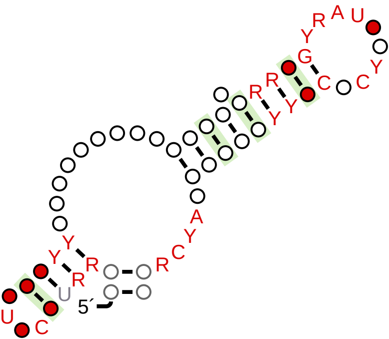

HMP2_11949_1

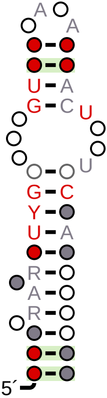

HMP2_11952_1

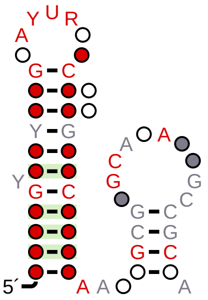

HMP2_11955_1

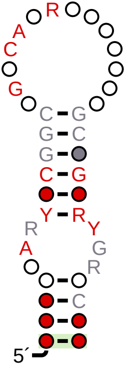

HMP2_11956_1

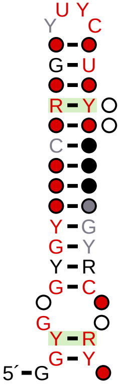

HMP2_11966_1

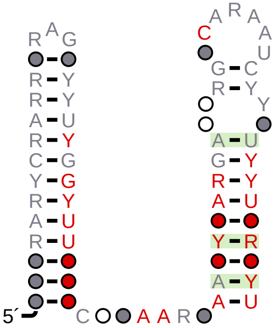

HMP2_11967_1

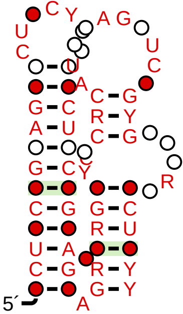

HMP2_1197_1

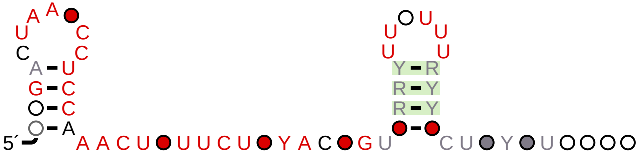

HMP2_11976_1

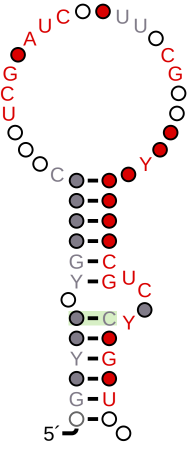

HMP2_11985_1

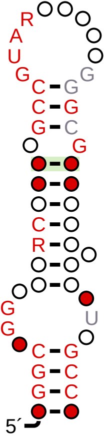

HMP2_11987_1

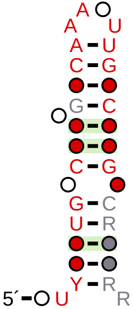

HMP2_11991_1

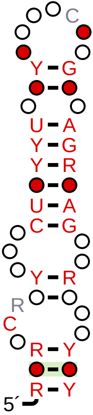

HMP2_11994_1

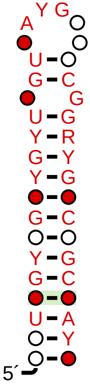

HMP2_11996_1

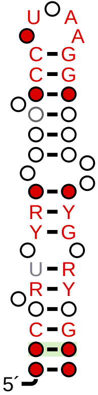

HMP2_12001_1

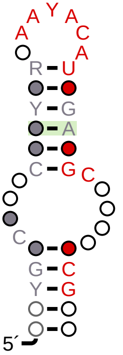

HMP2_12041_1

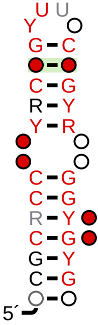

HMP2_12050_1

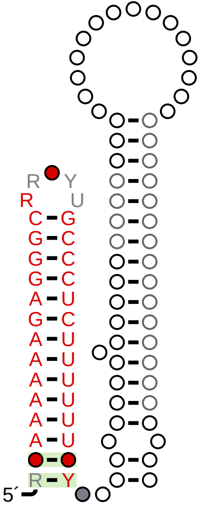

HMP2_12053_1

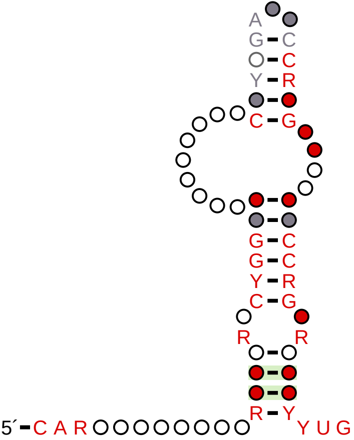

HMP2_12069_1

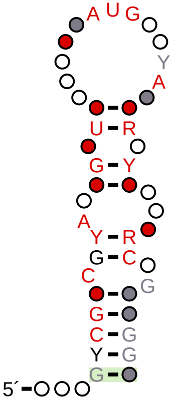

HMP2_12079_1

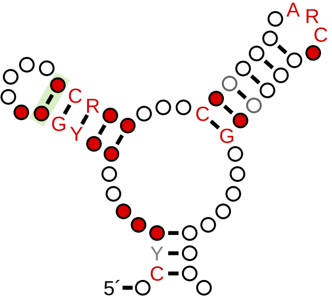

HMP2_12084_1

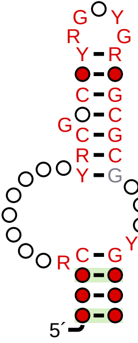

HMP2_12088_1

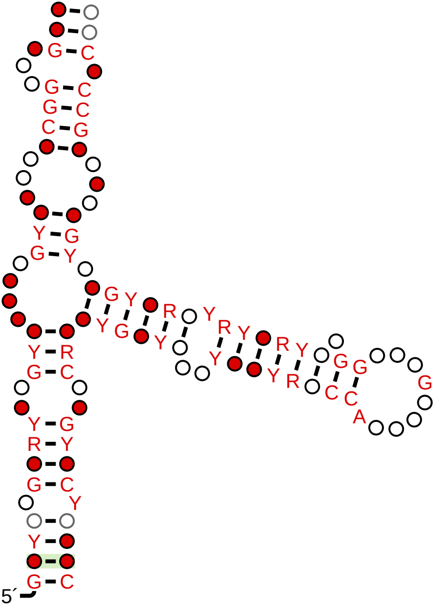

HMP2_12092_1

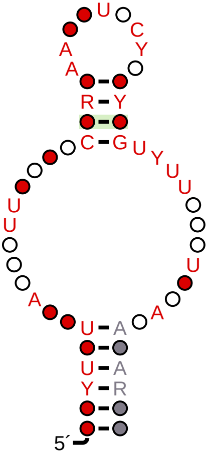

HMP2_12093_1

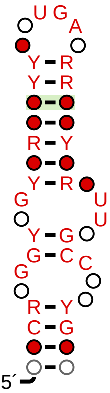

HMP2_12107_1

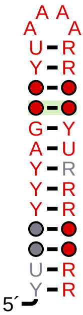

HMP2_12112_1

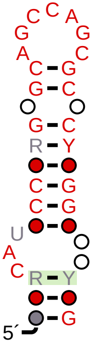

HMP2_1212_1

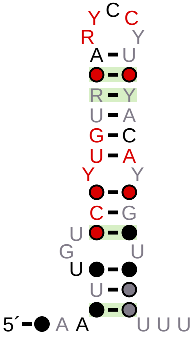

HMP2_12121_1

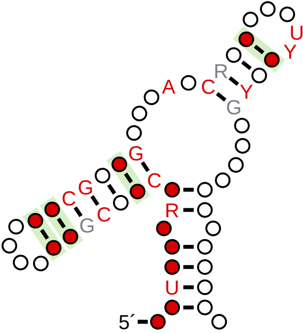

HMP2_12128_1

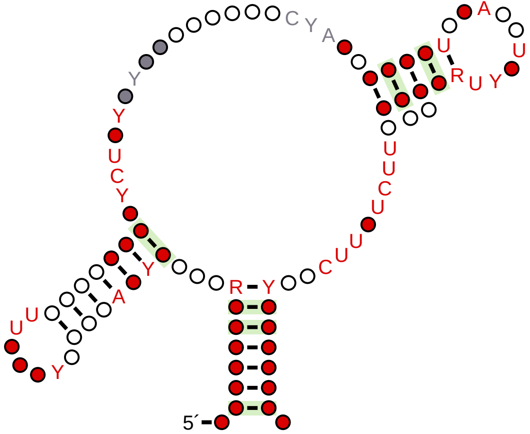

HMP2_12133_1

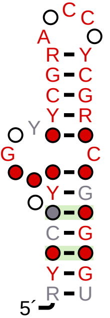

HMP2_12138_1

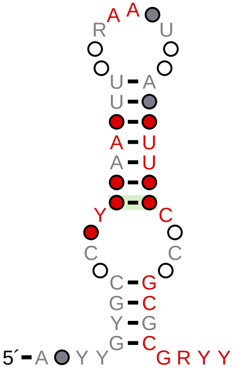

HMP2_12157_1

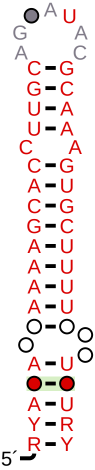

HMP2_12170_1

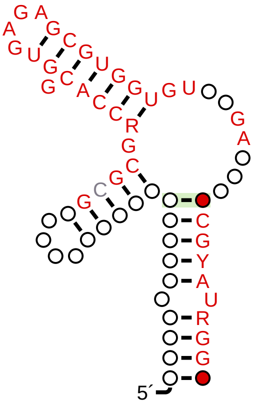

HMP2_12181_1

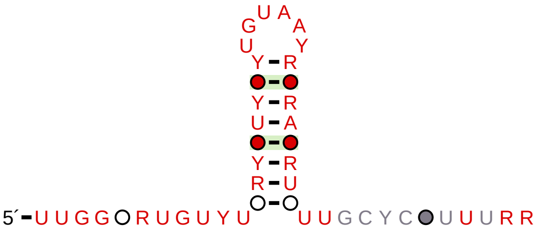

HMP2_12188_1

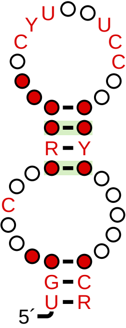

HMP2_12196_1

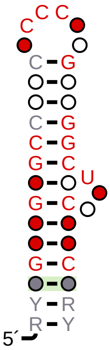

HMP2_12226_1

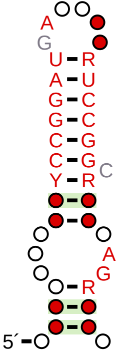

HMP2_12239_1

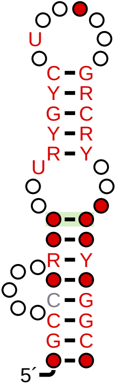

HMP2_12242_1

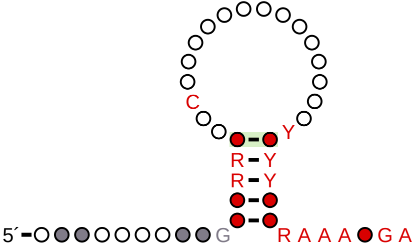

HMP2_12248_1

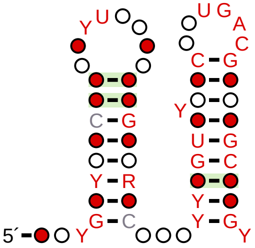

HMP2_12257_1

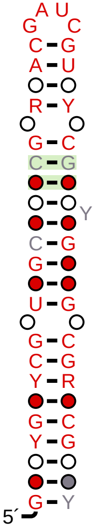

HMP2_12260_1

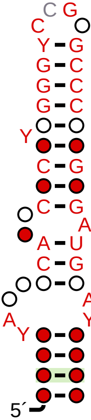

HMP2_12271_1

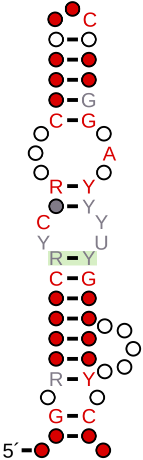

HMP2_12272_1

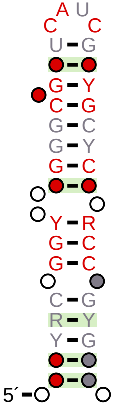

HMP2_12275_1

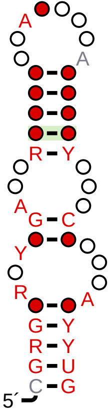

HMP2_12280_1

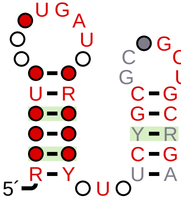

HMP2_12281_1

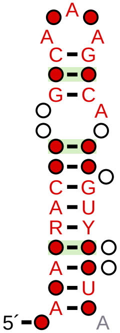

HMP2_12282_1

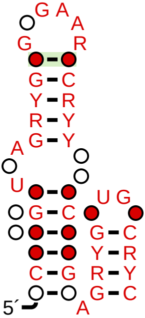

HMP2_12289_1

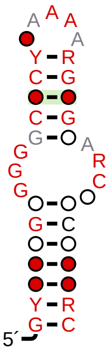

HMP2_123_1

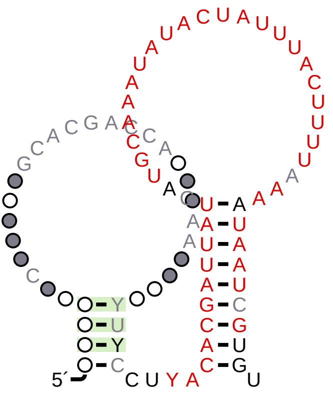

HMP2_12303_1

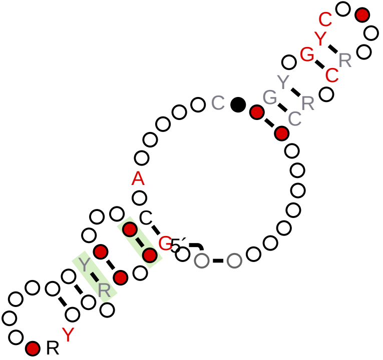

HMP2_12305_1

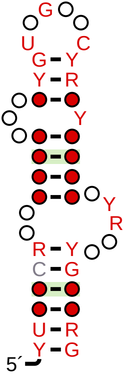

HMP2_12308_1

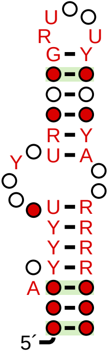

HMP2_12310_1

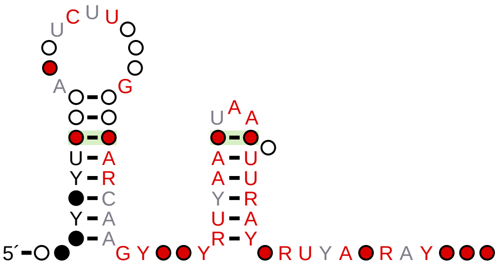

HMP2_12313_1

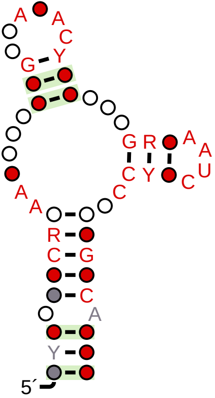

HMP2_12324_1

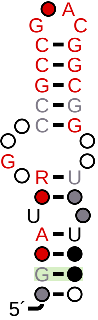

HMP2_12364_1

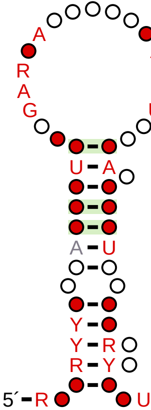

HMP2_1237_1

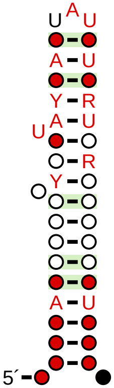

HMP2_12413_1

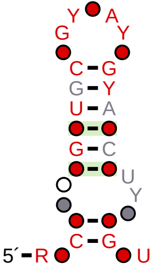

HMP2_12423_1

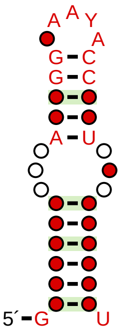

HMP2_1243_1

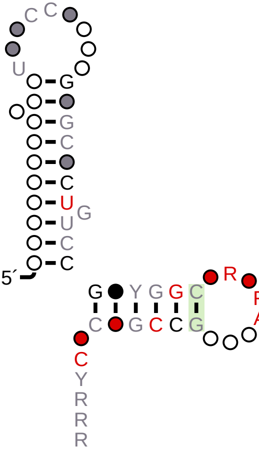

HMP2_12433_1

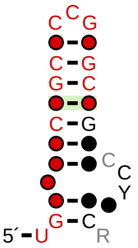

HMP2_12435_1

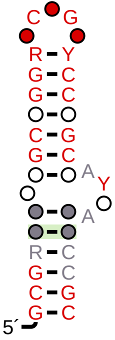

HMP2_12466_1

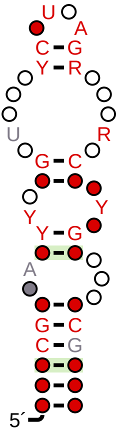

HMP2_12478_1

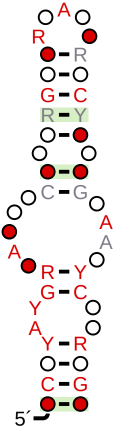

HMP2_1248_1

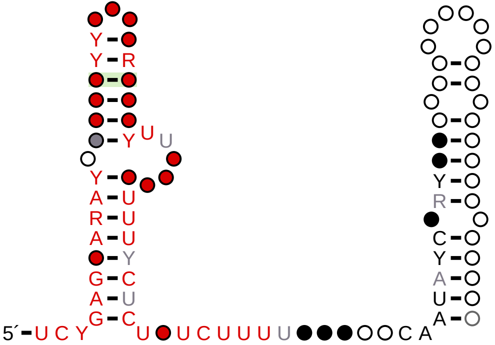

HMP2_12482_1

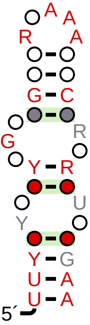

HMP2_1250_1

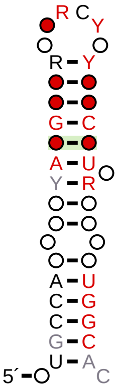

HMP2_12518_1

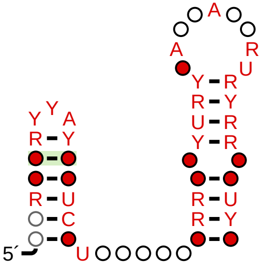

HMP2_1253_1

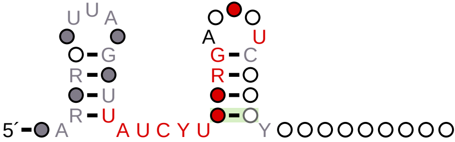

HMP2_12532_1

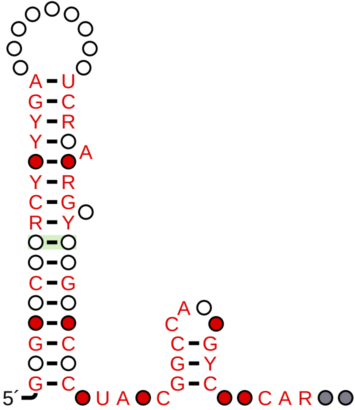

HMP2_12534_1

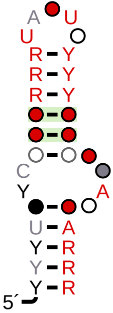

HMP2_12604_1

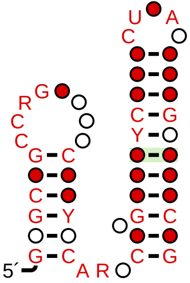

HMP2_12614_1

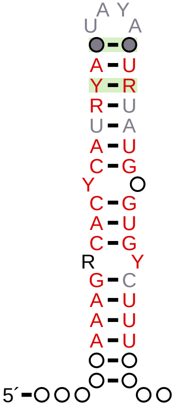

HMP2_12618_1

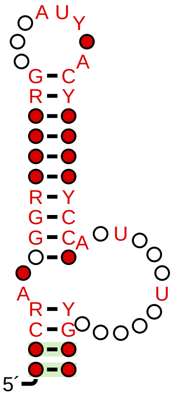

HMP2_1265_1

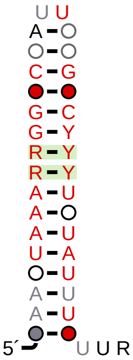

HMP2_1267_1

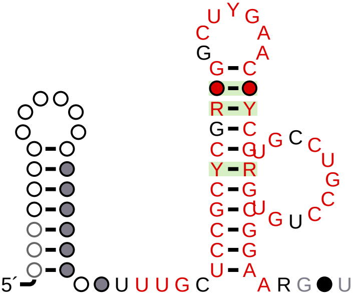

HMP2_12682_1

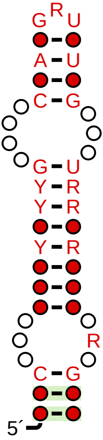

HMP2_12684_1

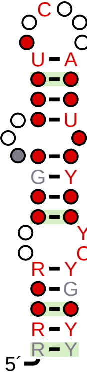

HMP2_12705_1

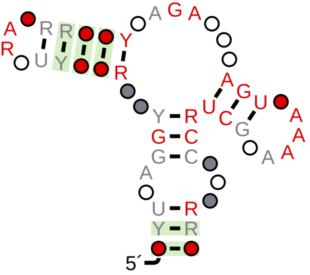

HMP2_12725_1

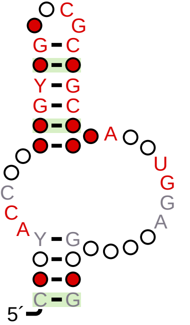

HMP2_12740_1

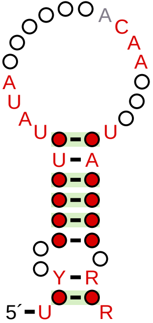

HMP2_12753_1

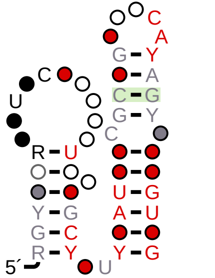

HMP2_12775_1

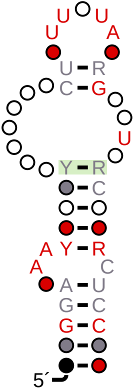

HMP2_12794_1

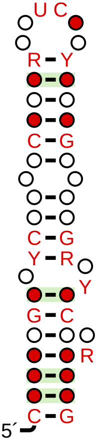

HMP2_1280_1

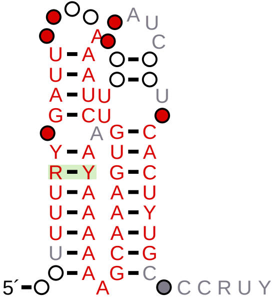

A
U
C
A
U-A
U-A
A-UU
U
G-CU
A
G-C
Y-A
U-A
R-Y
G-C
U-A
A-U
U-A
A-Y
U-A
A-U
U-A
C-G
A
G-C
5′
A
CCRUY

HMP2_1282_1

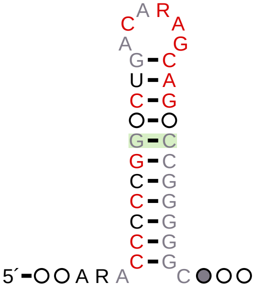

HMP2_12826_1

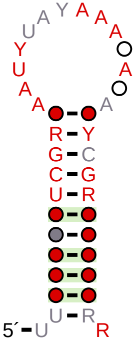

HMP2_12828_1

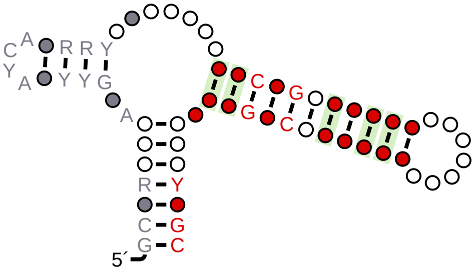

HMP2_12840_1

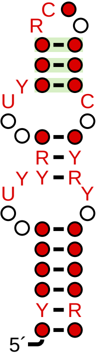

HMP2_12879_1

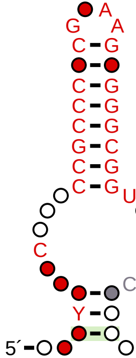

HMP2_12887_1

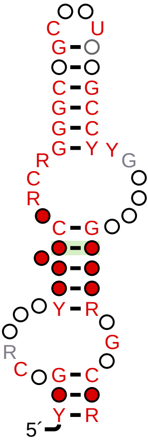

HMP2_12889_1

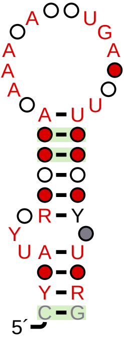

HMP2_12893_1

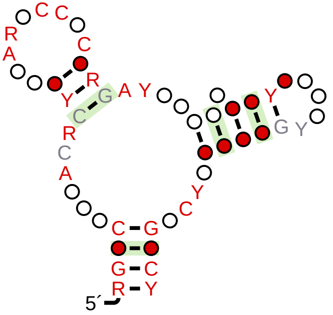

HMP2_12897_1

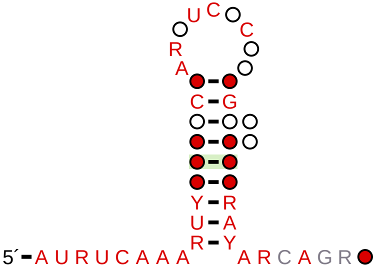

HMP2_12909_1

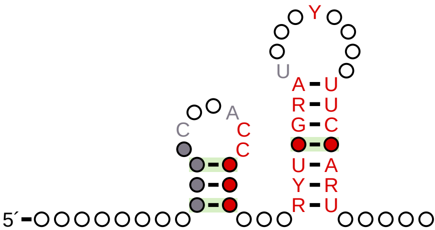

HMP2_12913_1

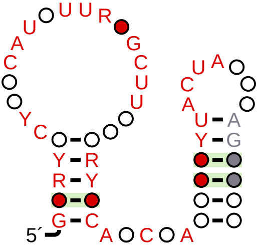

HMP2_1292_1

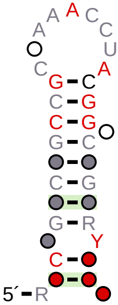

HMP2_12923_1

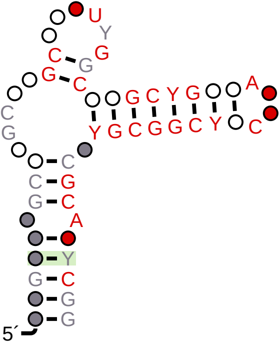

HMP2_1294_1

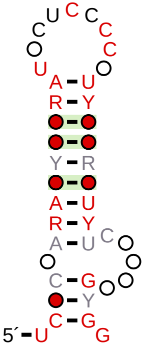

HMP2_12941_1

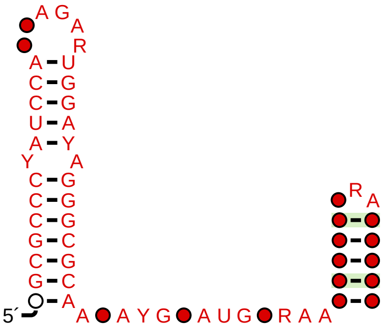

HMP2_12944_1

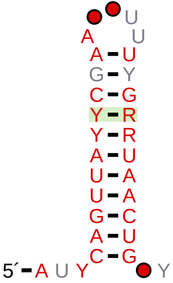

HMP2_12946_1

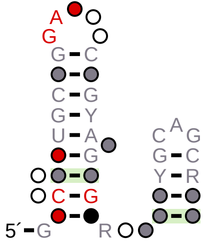

HMP2_12956_1

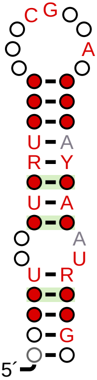

HMP2_12959_1

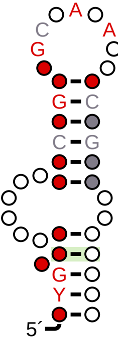

HMP2_12996_1

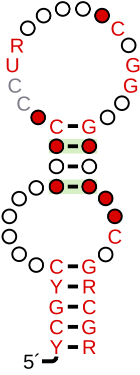

HMP2_13005_1

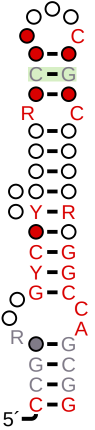

HMP2_13006_1

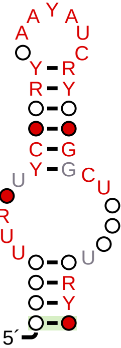

HMP2_13011_1

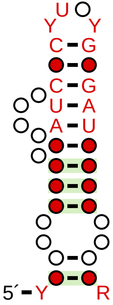

HMP2_13014_1

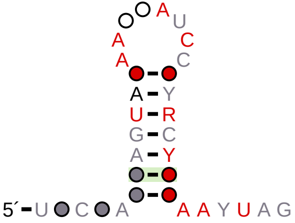

HMP2_13018_1

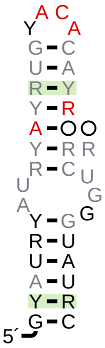

HMP2_13020_1

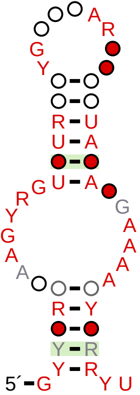

HMP2_13025_1

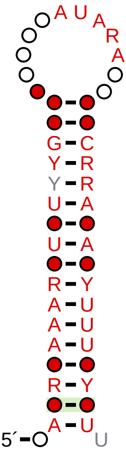

HMP2_13029_1

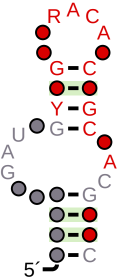

HMP2_13031_1

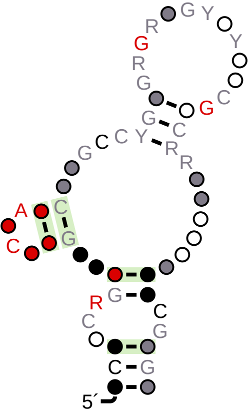

HMP2_13037_1

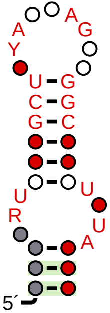

HMP2_13051_1

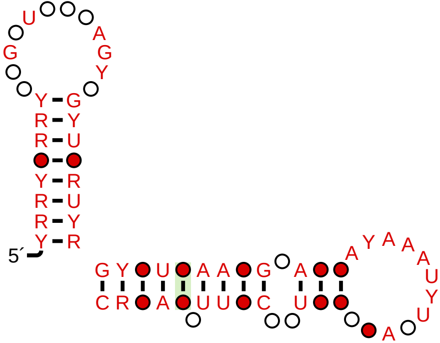

HMP2_13054_1

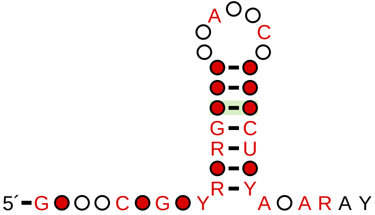

HMP2_13055_1

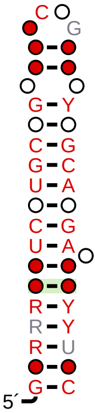

HMP2_13057_1

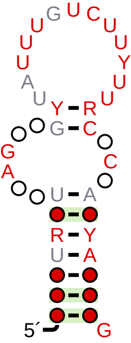

HMP2_1306_1

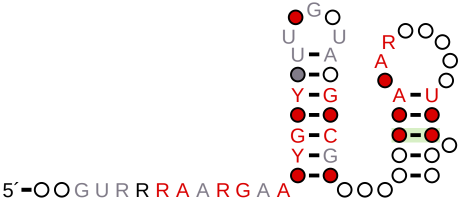

HMP2_13063_1

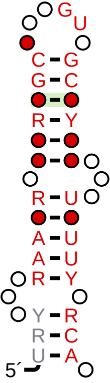

HMP2_13071_1

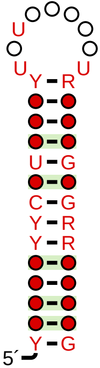

HMP2_13075_1

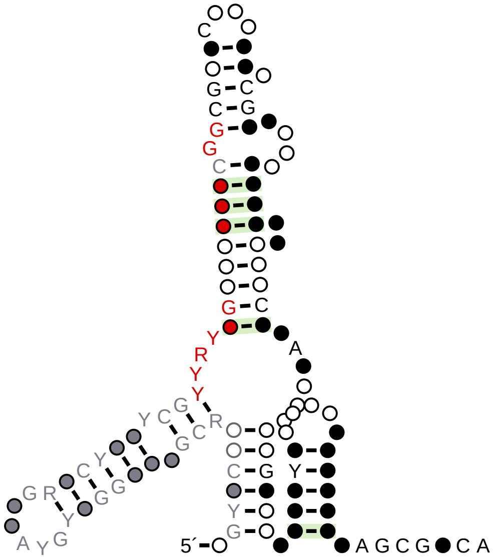

HMP2_131_1

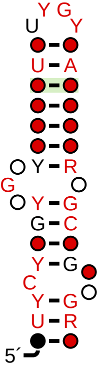

HMP2_13102_1

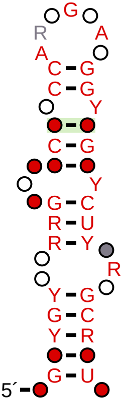

HMP2_13103_1

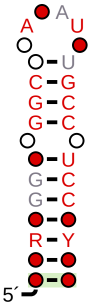

HMP2_13109_1

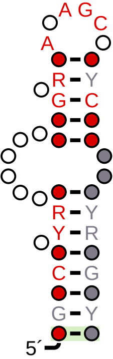

HMP2_13127_1

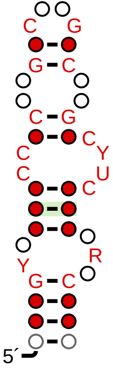

HMP2_13132_1

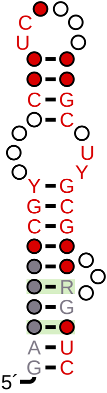

HMP2_13137_1

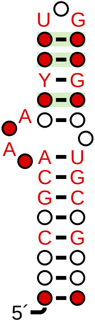

HMP2_13146_1

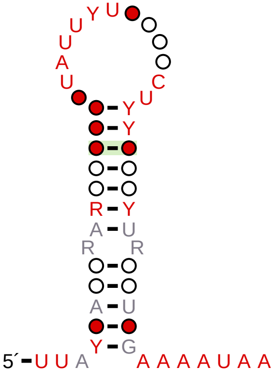

HMP2_1315_1

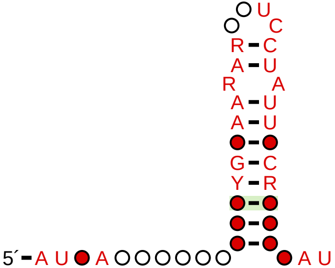

HMP2_13156_1

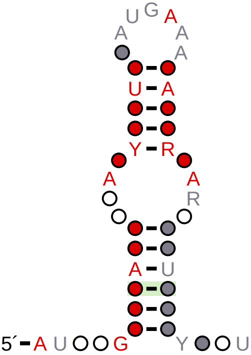

HMP2_13161_1

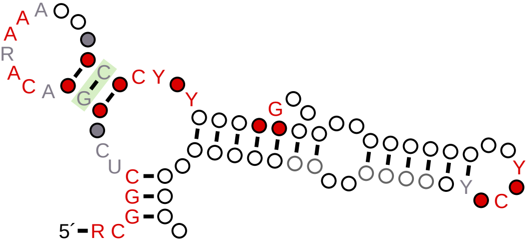

HMP2_13163_1

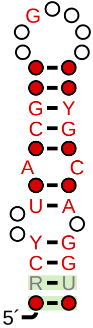

HMP2_13166_1

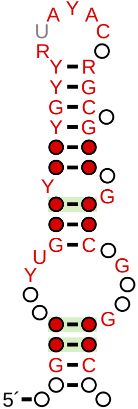

HMP2_13175_1

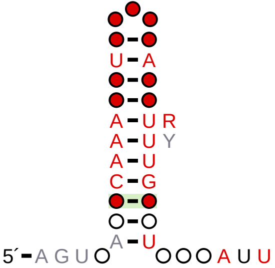

HMP2_1318_1

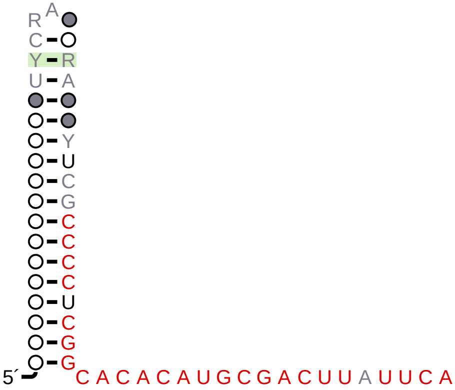

HMP2_13187_1

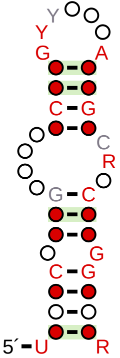

HMP2_13190_1

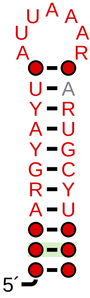

HMP2_13201_1

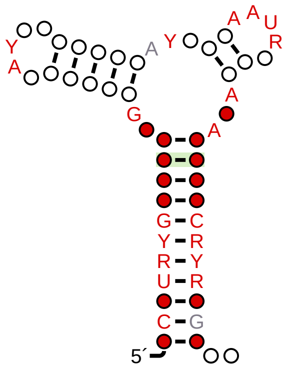

HMP2_1321_1

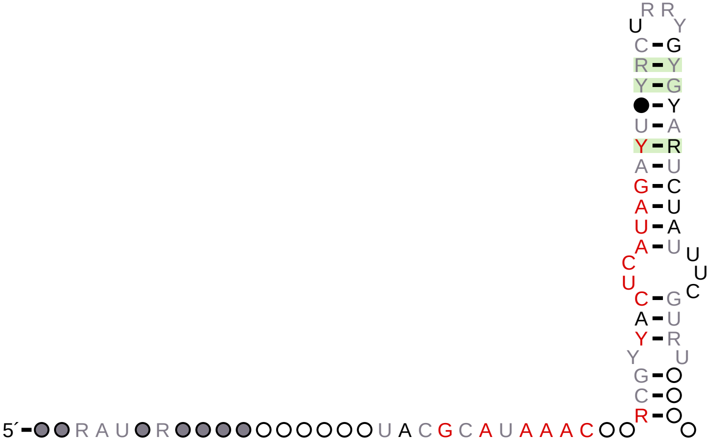
R R
U Y
C G
R Y
Y G
Y
U A
Y R
A U
G C
A U
U A
A U
C U
U U
C G C
A U
Y R
Y U
G
C
R
5´ R A U R U A C G C A U A A A C

HMP2_13212_1

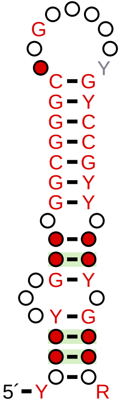

HMP2_13216_1

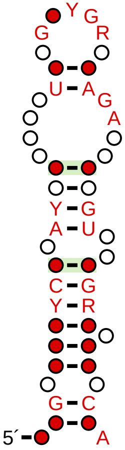

HMP2_13219_1

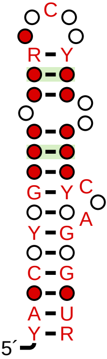

HMP2_13227_1

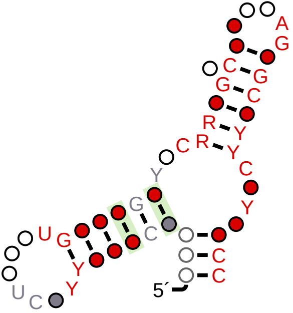

HMP2_13239_1

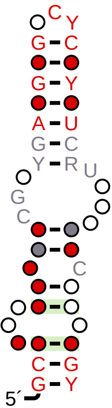

HMP2_1324_1

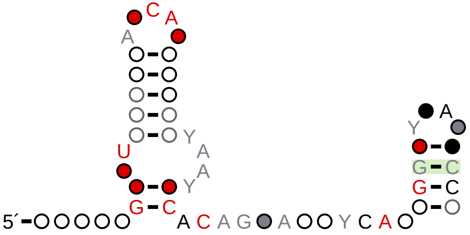

HMP2_13241_1

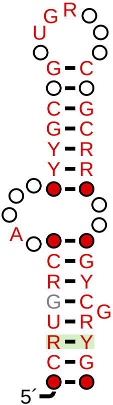

HMP2_13256_1

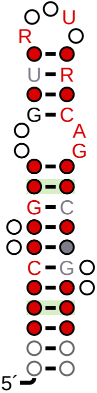

HMP2_1326_1

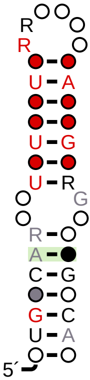

HMP2_13263_1

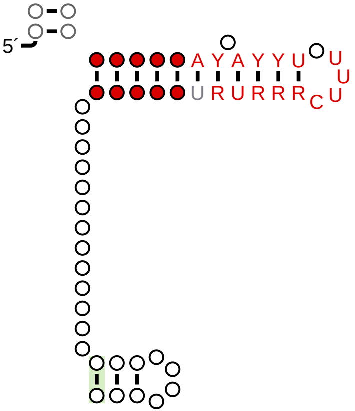

HMP2_13265_1

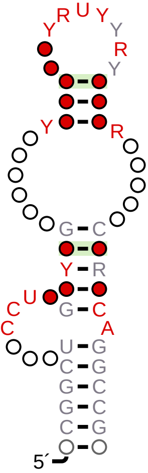

HMP2_13266_1

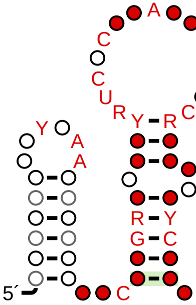

HMP2_13267_1

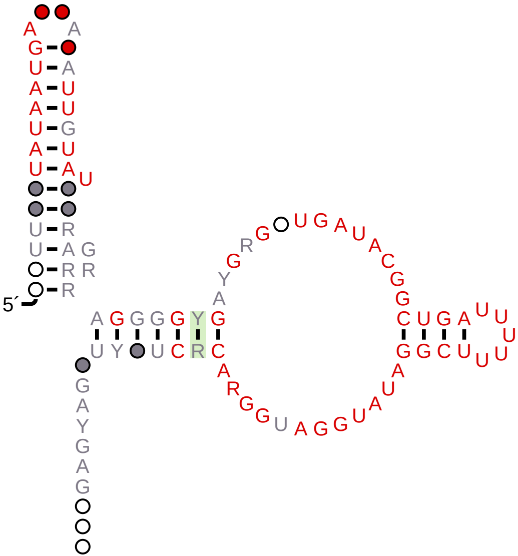

HMP2_13268_1

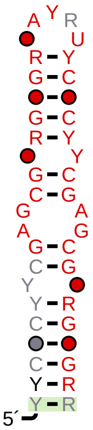

HMP2_13274_1

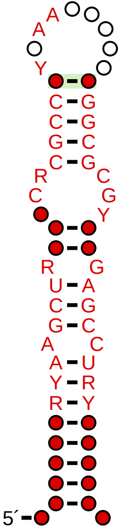

HMP2_13278_1

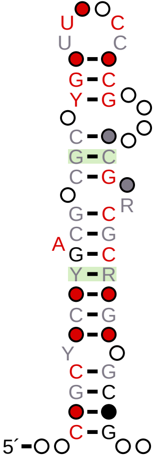

HMP2_13284_1

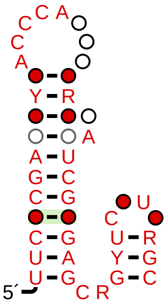

HMP2_13296_1

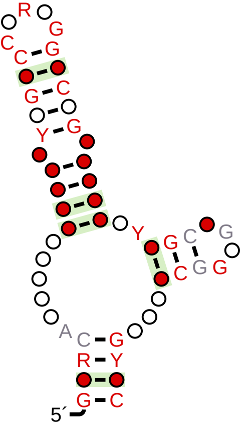

HMP2_13301_1

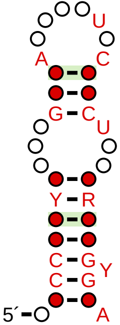

HMP2_13323_1

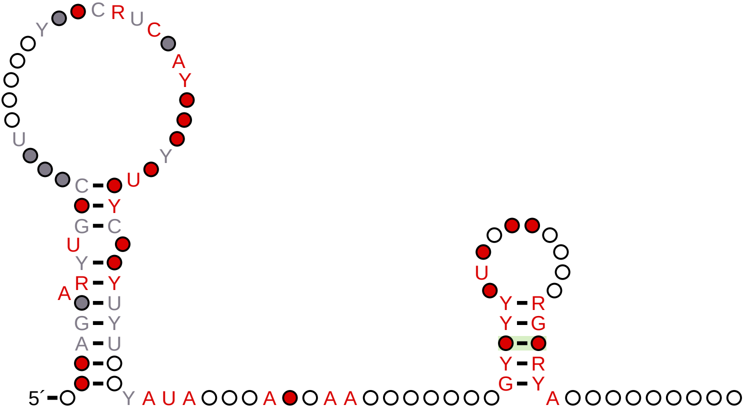

HMP2_13325_1

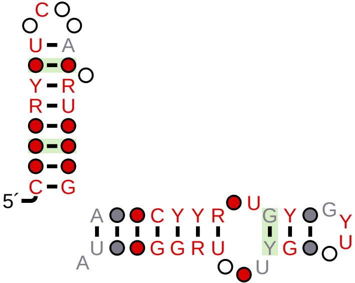

HMP2_13326_1

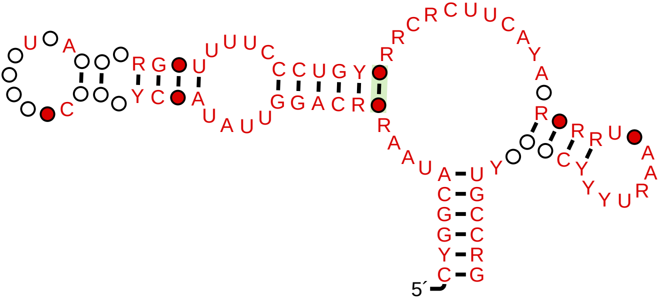

HMP2_1333_1

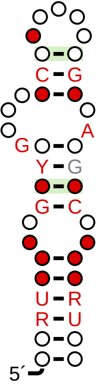

HMP2_1334_1

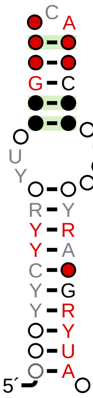

HMP2_13348_1

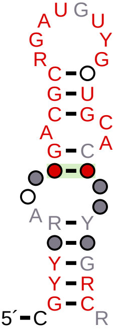

HMP2_13356_1

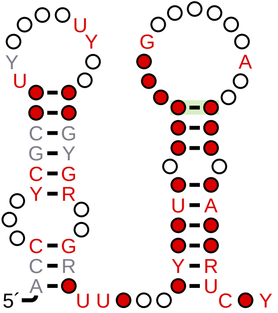

HMP2_13390_1

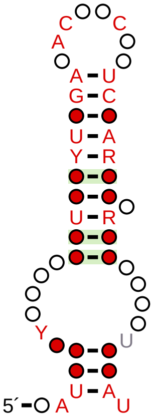

HMP2_13400_1

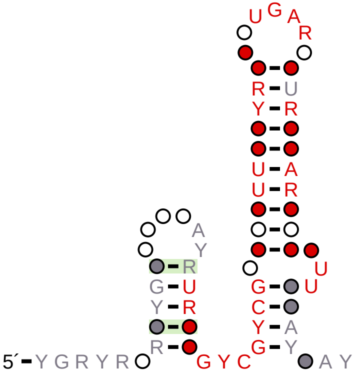

HMP2_13402_1

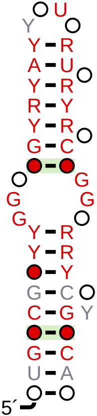

HMP2_13412_1

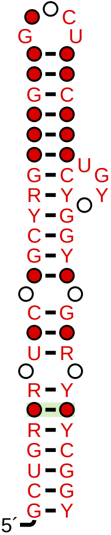

HMP2_13426_1

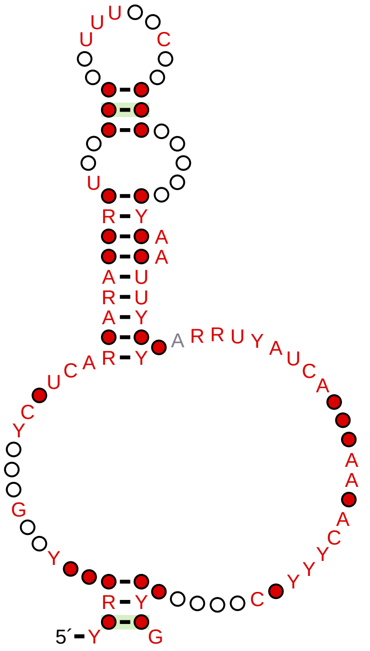

HMP2_13428_1

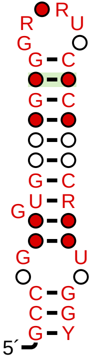

HMP2_13436_1

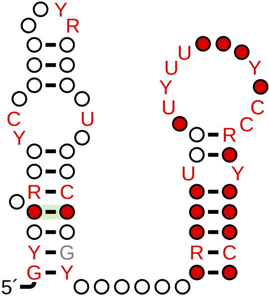

HMP2_13452_1

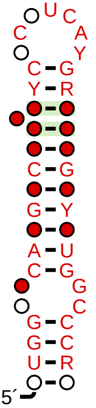

HMP2_13454_1

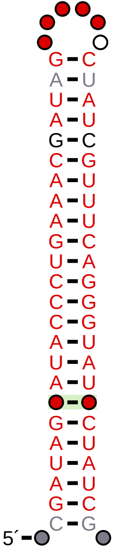

HMP2_13463_1

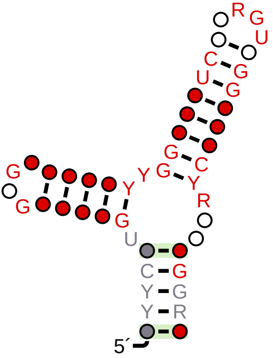

HMP2_13475_1

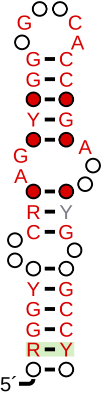

HMP2_13497_1

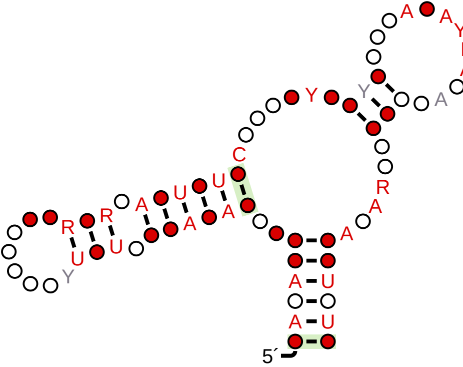

HMP2_13501_1

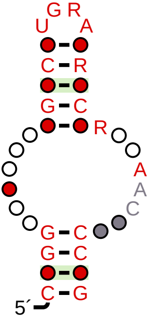

HMP2_13505_1

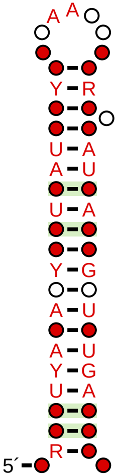

HMP2_13507_1

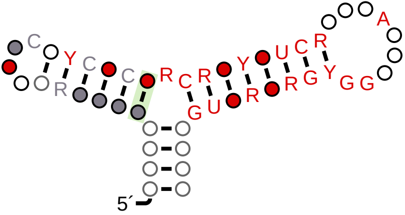

HMP2_13524_1

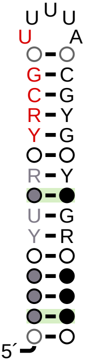

HMP2_13534_1

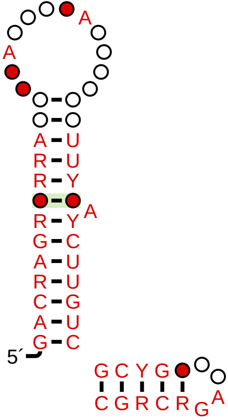

HMP2_13538_1

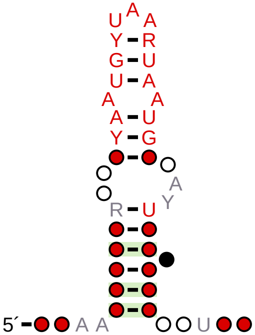

HMP2_13582_1

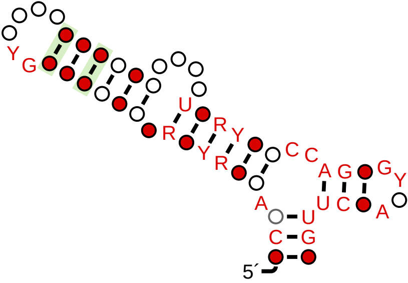

HMP2_13583_1

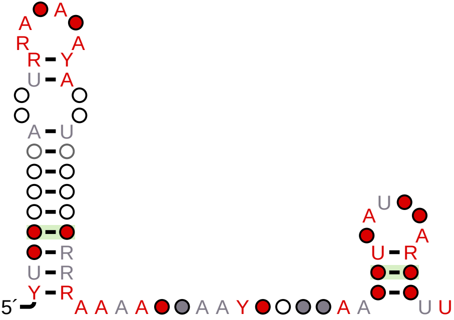

HMP2_13589_1

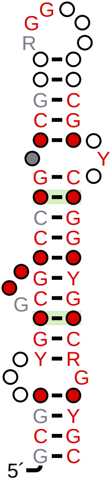

HMP2_13602_1

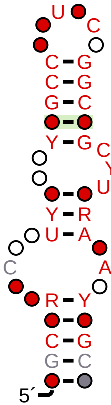

HMP2_13609_1

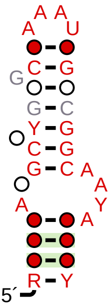

HMP2_13613_1

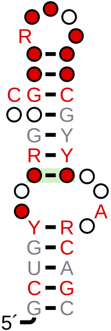

HMP2_13618_1

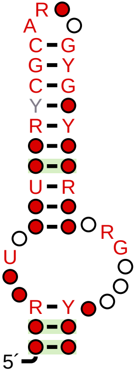

HMP2_1364_1

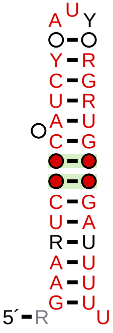

HMP2_13640_1

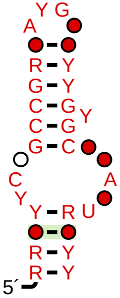

HMP2_13665_1

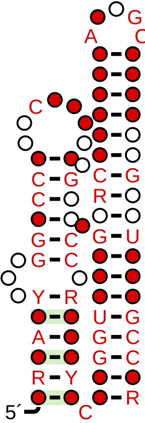

HMP2_13679_1

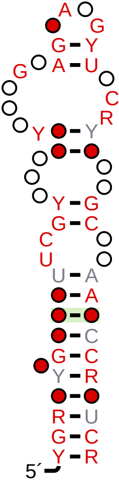

HMP2_13690_1

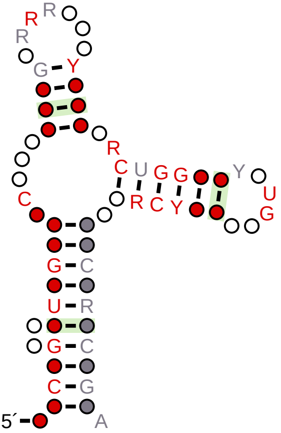

HMP2_13696_1

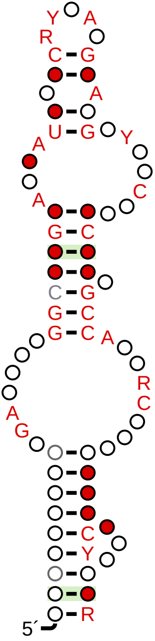

HMP2_13699_1

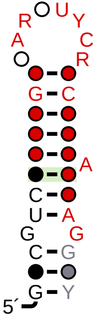

HMP2_137_1

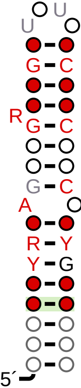

HMP2_1370_1

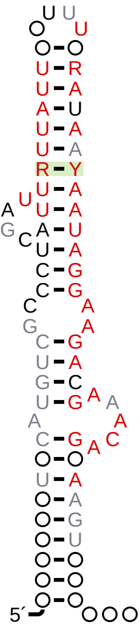

HMP2_13702_1

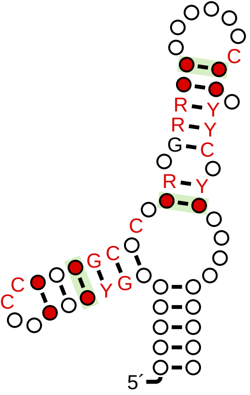

HMP2_13723_1

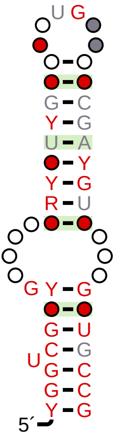

HMP2_13725_1

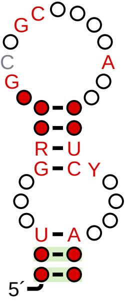

HMP2_13735_1

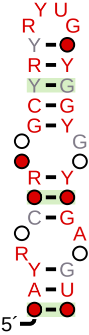

HMP2_13736_1

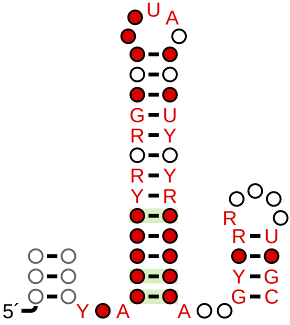

HMP2_13738_1

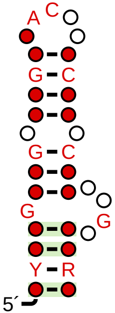

HMP2_13749_1

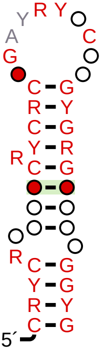

HMP2_13750_1

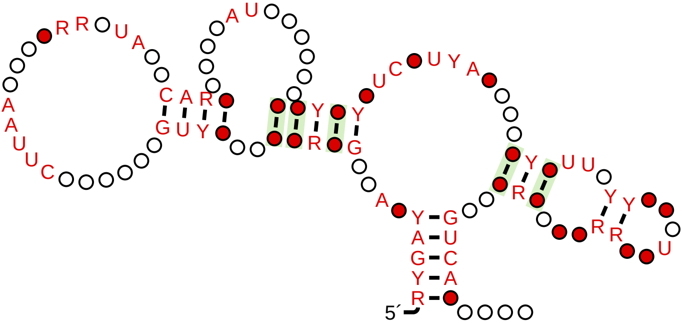

HMP2_13752_1

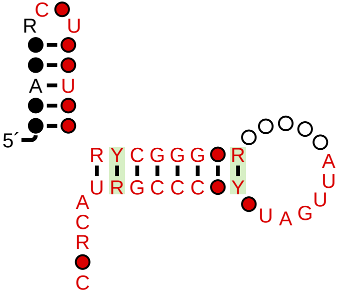

HMP2_13756_1

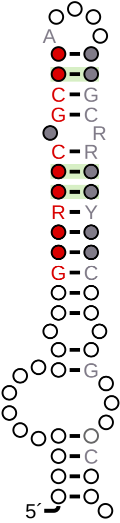

HMP2_13765_1

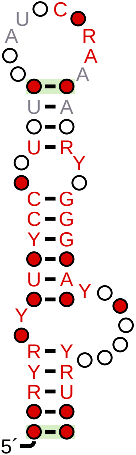

HMP2_13769_1

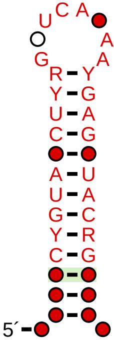

HMP2_13787_1

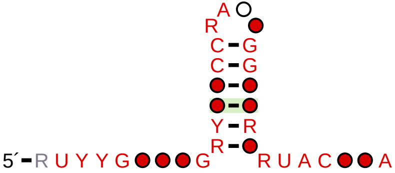

HMP2_13791_1

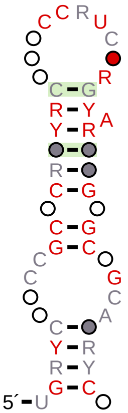

HMP2_13794_1

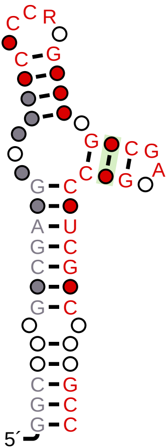

HMP2_13797_1

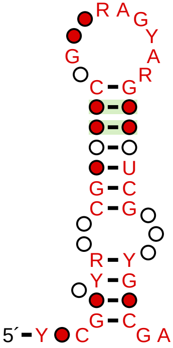

HMP2_1380_1

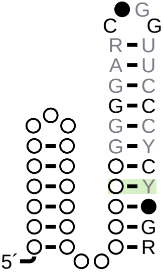

HMP2_13809_1

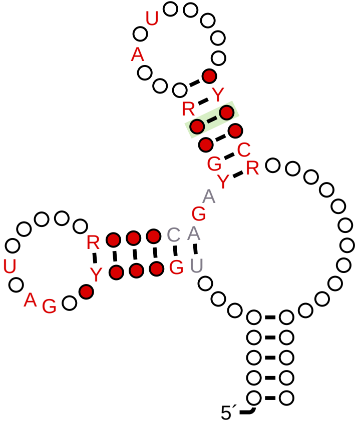

HMP2_13812_1

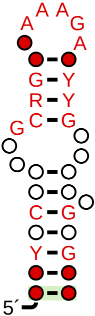

HMP2_13816_1

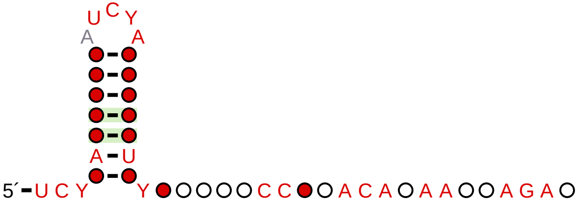

HMP2_13819_1

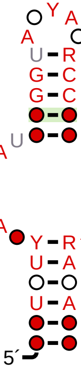

HMP2_13826_1

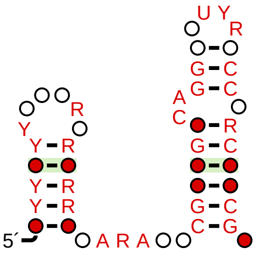

HMP2_13830_1

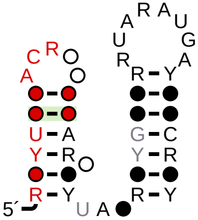

HMP2_13836_1

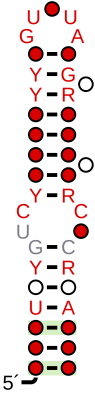

HMP2_13840_1

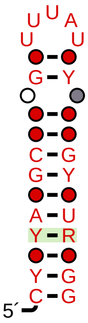

HMP2_13855_1

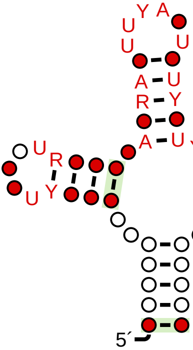

HMP2_1386_1

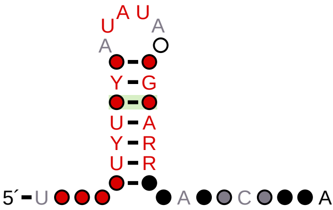

HMP2_13869_1

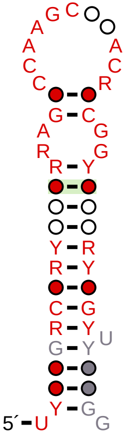

HMP2_13872_1

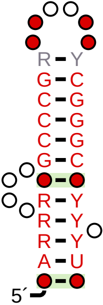

HMP2_1390_1

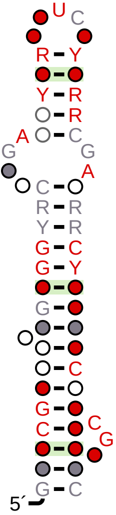

HMP2_13905_1

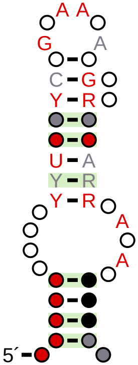

HMP2_13912_1

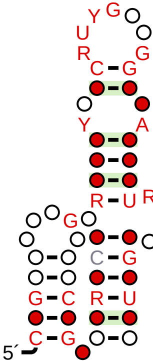

HMP2_13921_1

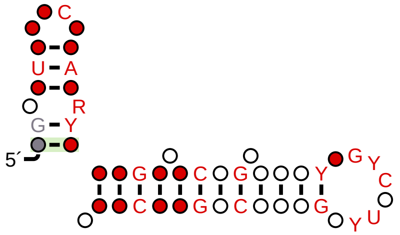

HMP2_13925_1

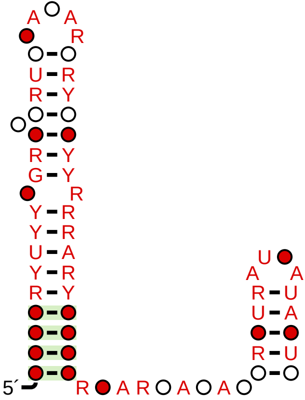

HMP2_13934_1

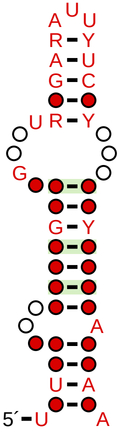

HMP2_13947_1

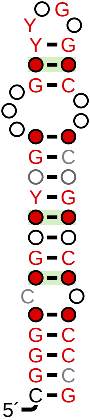

HMP2_13957_1

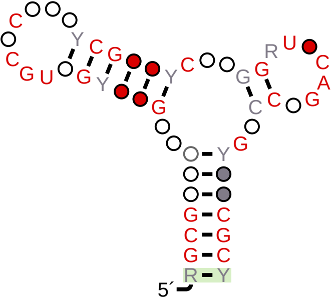

HMP2_13962_1

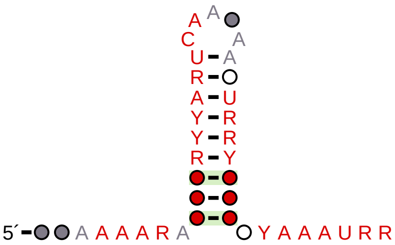

HMP2_13963_1

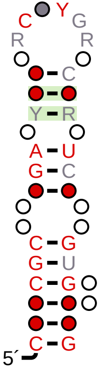

HMP2_13974_1

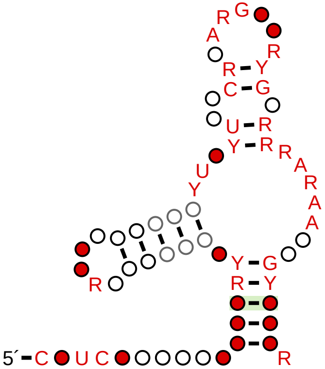

HMP2_13978_1

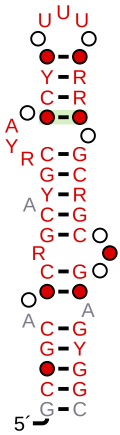

HMP2_13985_1

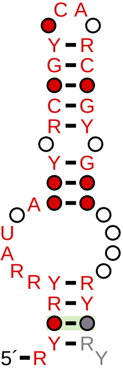

HMP2_13986_1

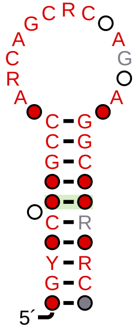

HMP2_13989_1

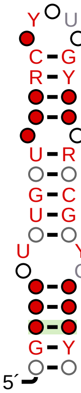

HMP2_13997_1

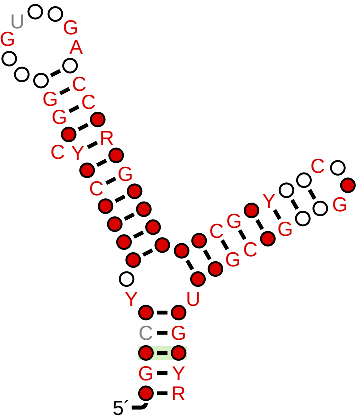

HMP2_14002_1

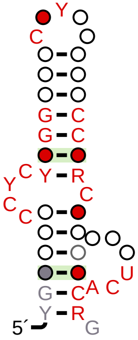

HMP2_14005_1

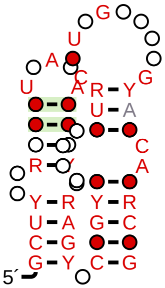

HMP2_14018_1

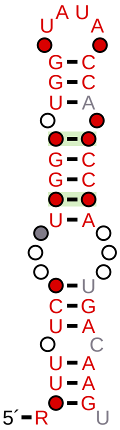

HMP2_14019_1

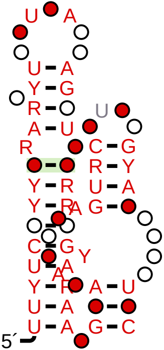

HMP2_14063_1

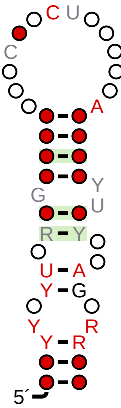

HMP2_14071_1

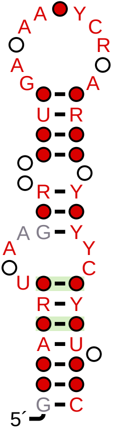

HMP2_14082_1

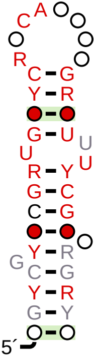

HMP2_14084_1

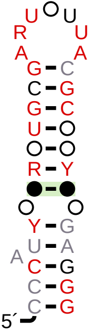

HMP2_14088_1

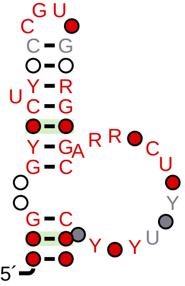

HMP2_14098_1

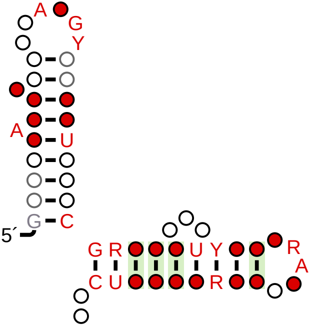

HMP2_14104_1

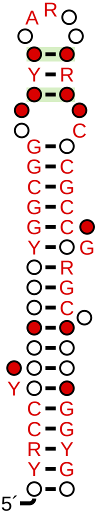

HMP2_14106_1

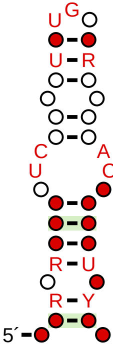

HMP2_14120_1

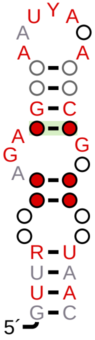

HMP2_14126_1

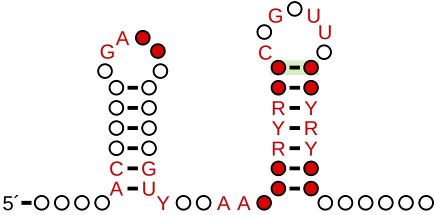

HMP2_14137_1

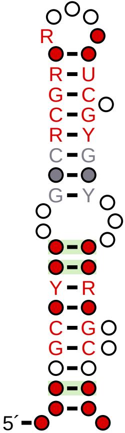

HMP2_14142_1

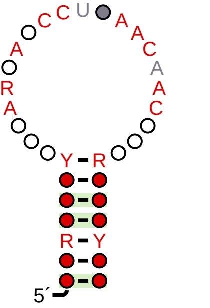

HMP2_14148_1

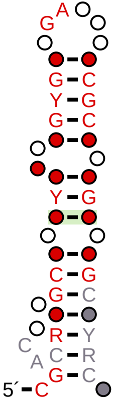

HMP2_14163_1

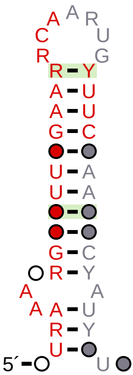

HMP2_14167_1

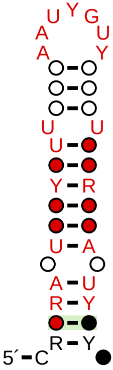

HMP2_14175_1

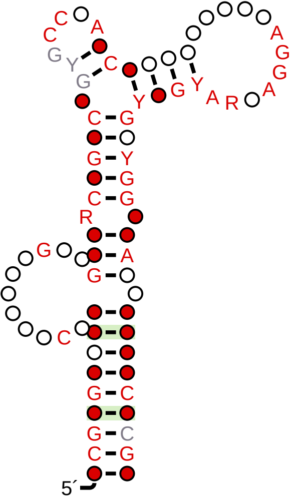

HMP2_14182_1

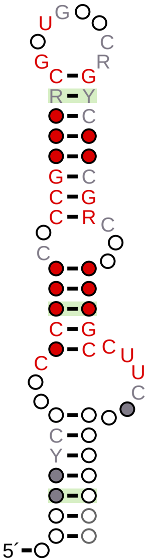

HMP2_14188_1

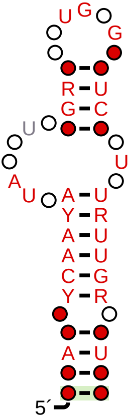

HMP2_14200_1

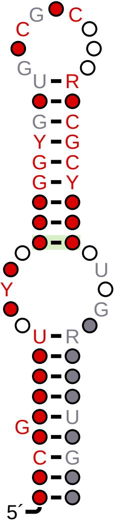

HMP2_14206_1

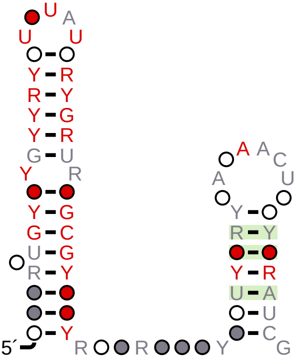

HMP2_14216_1

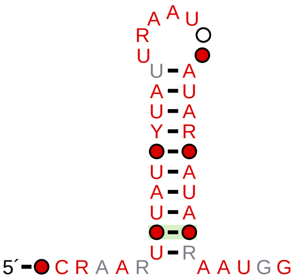

HMP2_14220_1

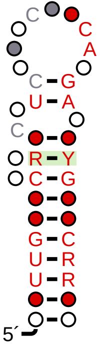

HMP2_14228_1

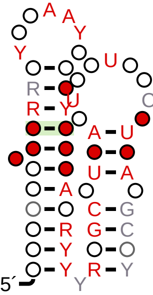

HMP2_14236_1

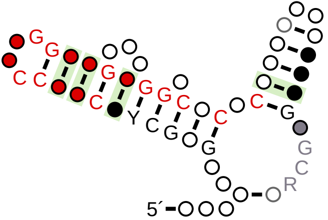

HMP2_14237_1

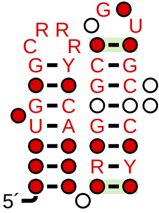

HMP2_14244_1

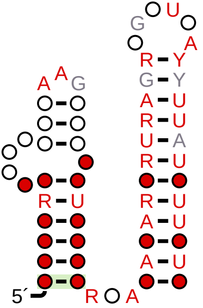

HMP2_14254_1

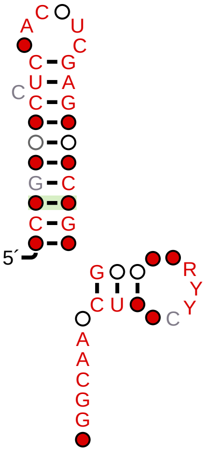

HMP2_14269_1

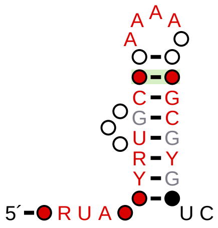

HMP2_14270_1

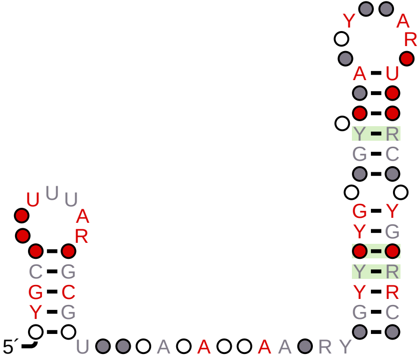

HMP2_14271_1

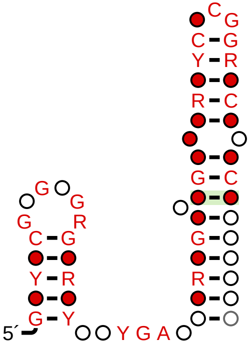

HMP2_14294_1

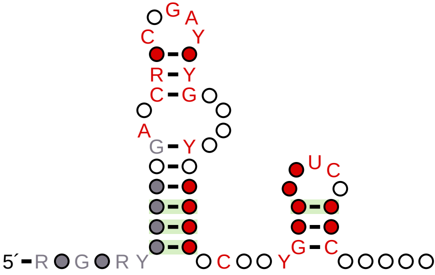

HMP2_14298_1

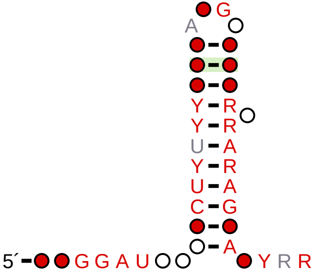

HMP2_143_1

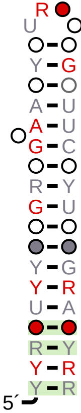

HMP2_1430_1

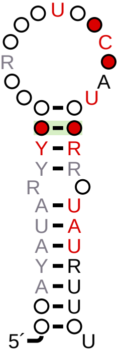

HMP2_14302_1

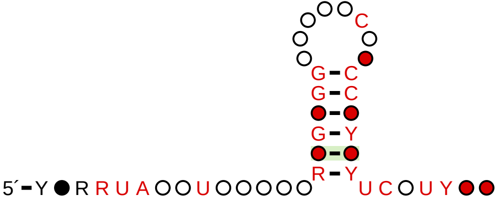

HMP2_14308_1

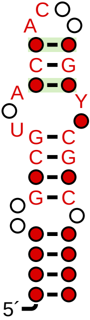

HMP2_14314_1

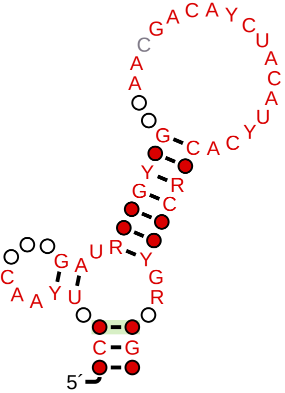

HMP2_14329_1

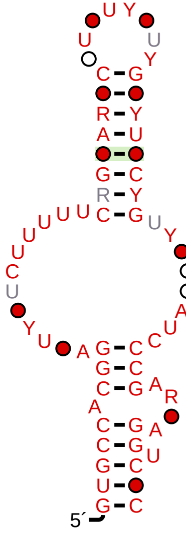

HMP2_1433_1

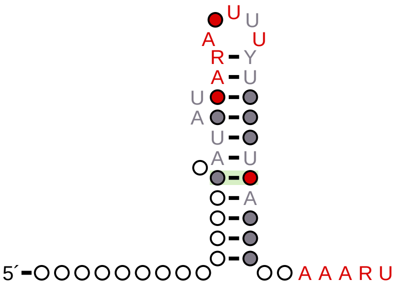

HMP2_14338_1

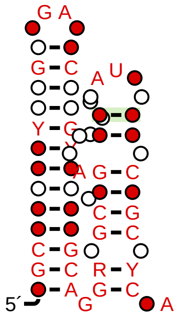

HMP2_14341_1

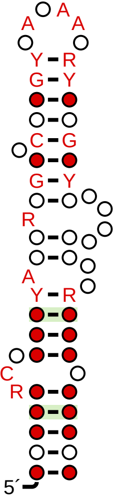

HMP2_14345_1

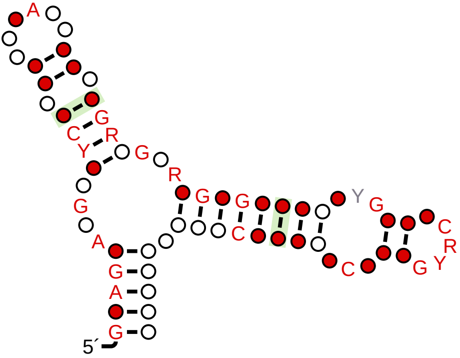

HMP2_14346_1

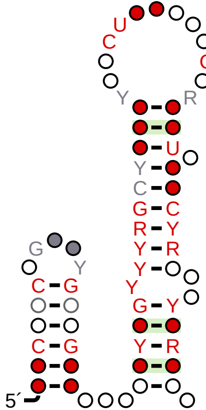

HMP2_14358_1

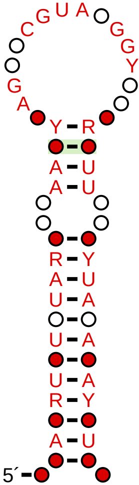

HMP2_14359_1

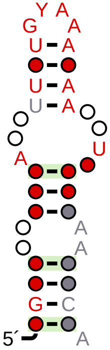

HMP2_14368_1

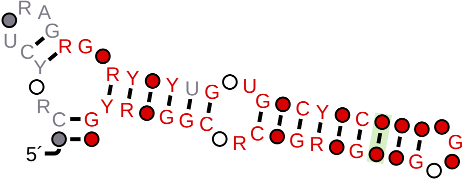

HMP2_14371_1

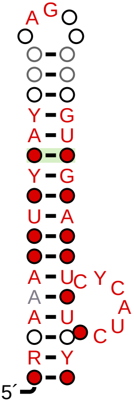

HMP2_14384_1

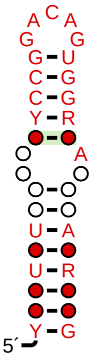

HMP2_14385_1

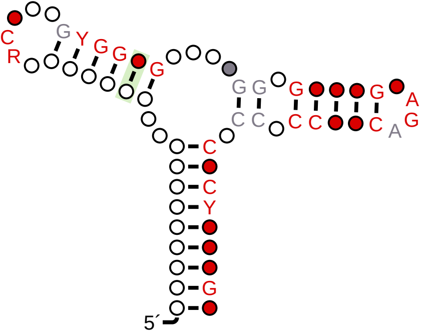

HMP2_14390_1

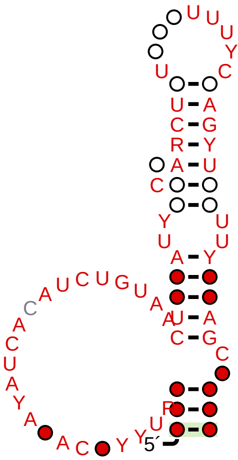

HMP2_14391_1

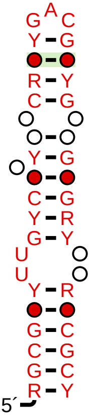

HMP2_14392_1

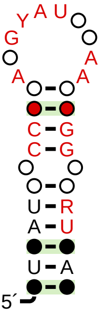

HMP2_14399_1

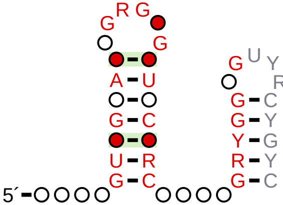

HMP2_14401_1

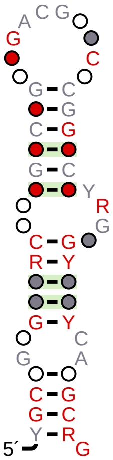

HMP2_14404_1

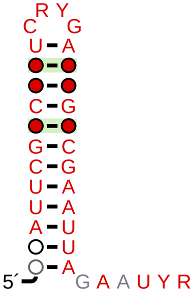

HMP2_14432_1

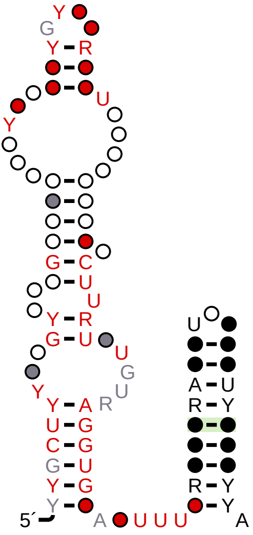

HMP2_14435_1

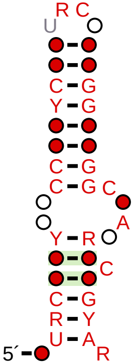

HMP2_14443_1

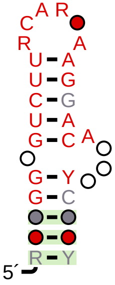

HMP2_14448_1

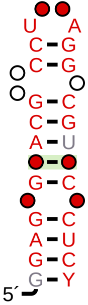

HMP2_14451_1

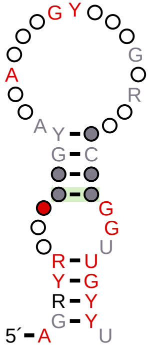

HMP2_14456_1

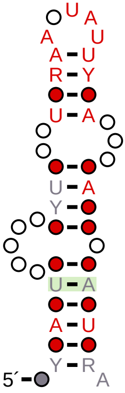

HMP2_14457_1

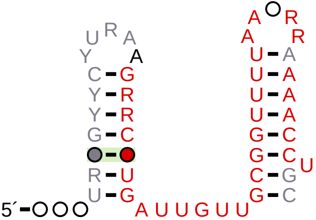

HMP2_14462_1

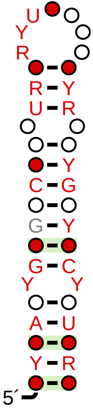

HMP2_14465_1

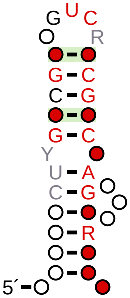

HMP2_14481_1

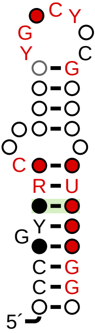

HMP2_14491_1

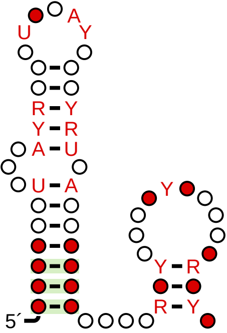

HMP2_14501_1

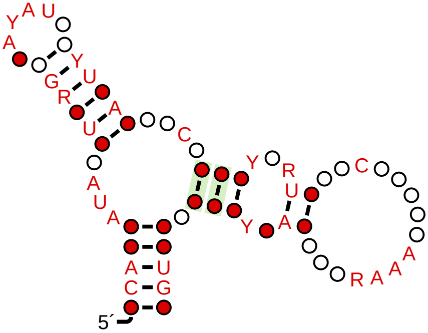

HMP2_1453_1

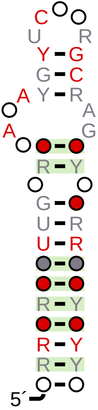

HMP2_14544_1

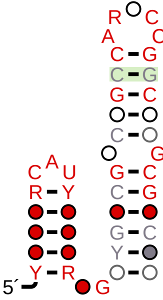

HMP2_14550_1

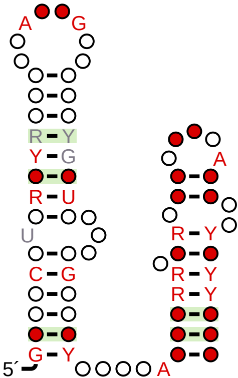

HMP2_14557_1

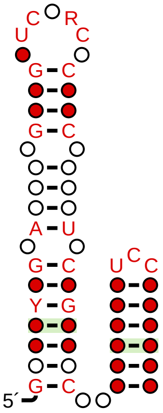

HMP2_14564_1

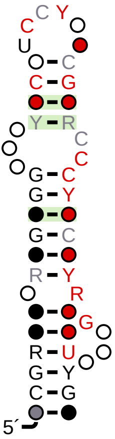

HMP2_14565_1

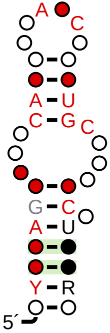

HMP2_14589_1

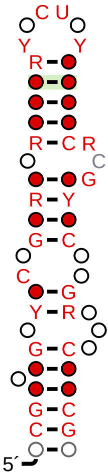

HMP2_14602_1

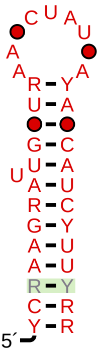

HMP2_14610_1

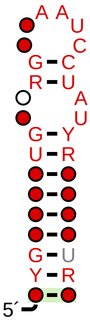

HMP2_14623_1

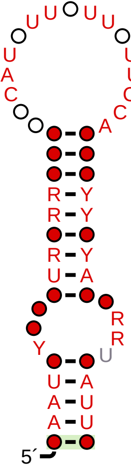

HMP2_14626_1

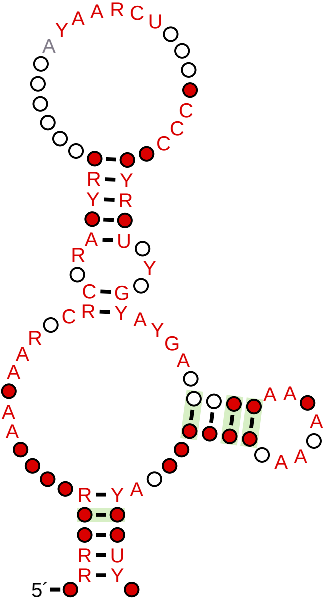

HMP2_14634_1

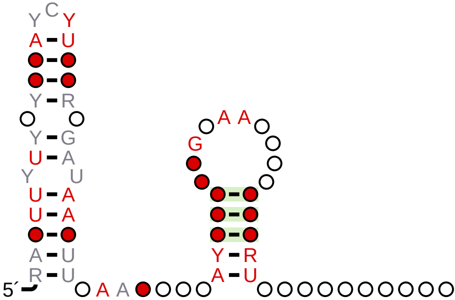

HMP2_14641_1

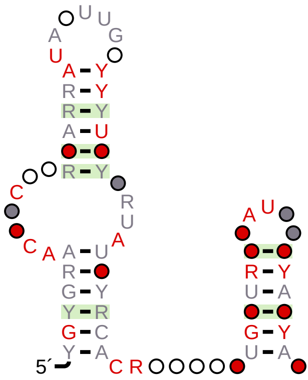

HMP2_14650_1

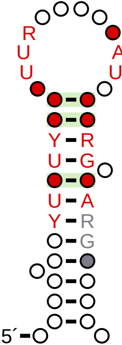

HMP2_14663_1

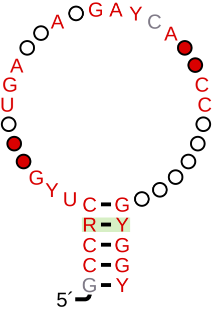

HMP2_14669_1

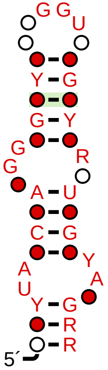

HMP2_14671_1

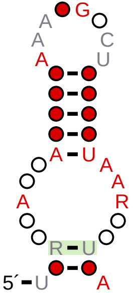

HMP2_14679_1

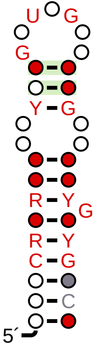

HMP2_14690_1

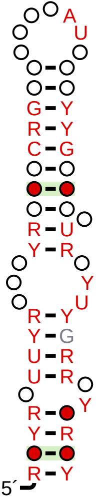

HMP2_14703_1

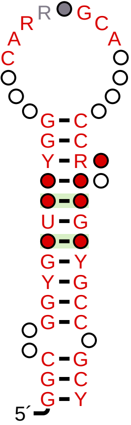

HMP2_14706_1

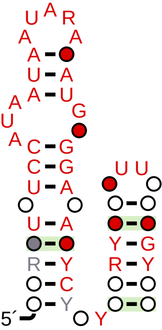

HMP2_14710_1

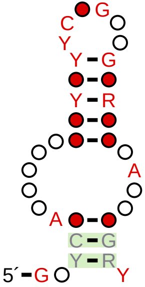

HMP2_14715_1

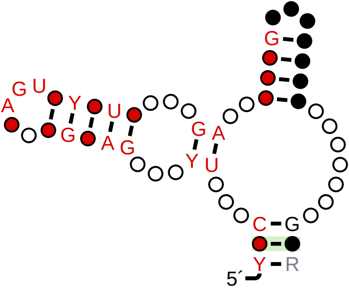

HMP2_14716_1

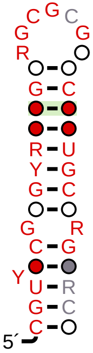

HMP2_14729_1

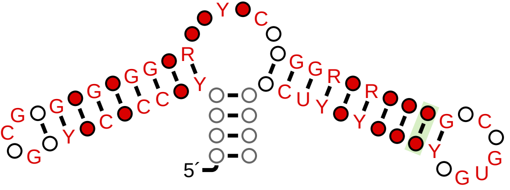

HMP2_14732_1

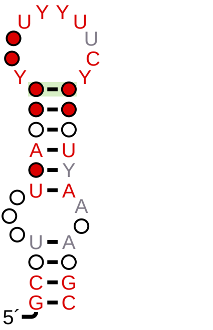

HMP2_14735_1

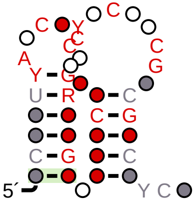

HMP2_14738_1

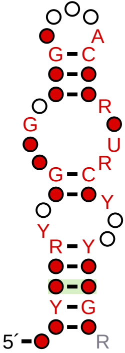

HMP2_14739_1

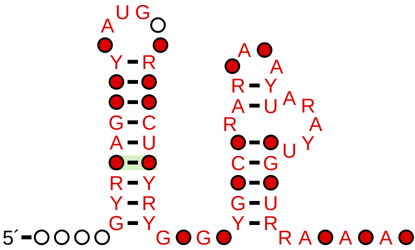

HMP2_14749_1

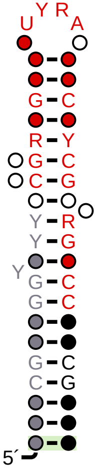

HMP2_14761_1

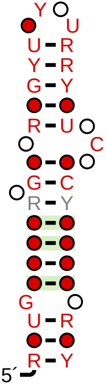

HMP2_14764_1

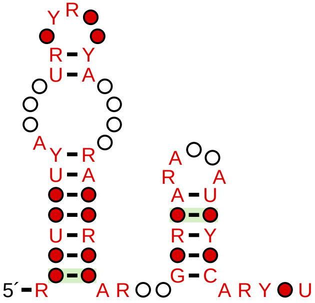

HMP2_14766_1

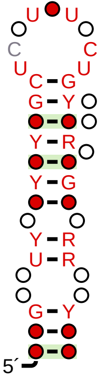

HMP2_14767_1

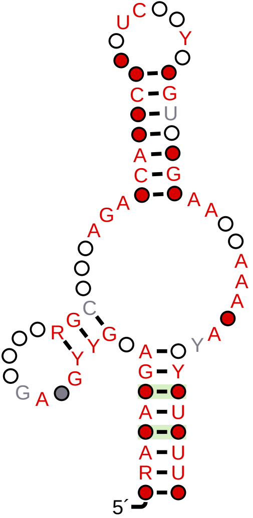

HMP2_14768_1

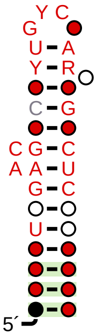

HMP2_1478_1

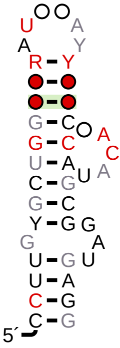

HMP2_14781_1

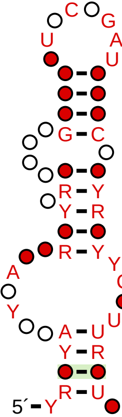

HMP2_14789_1

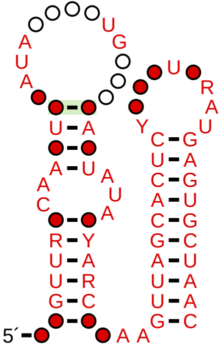

HMP2_1480_1

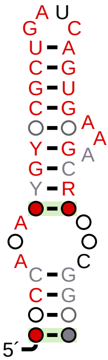

HMP2_14807_1

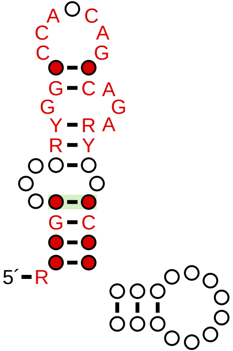

HMP2_14810_1

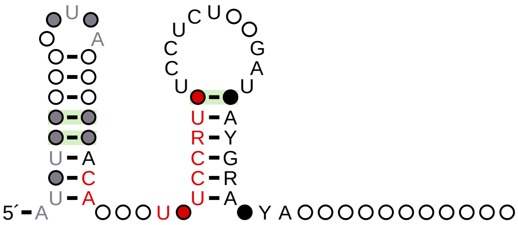

HMP2_14813_1

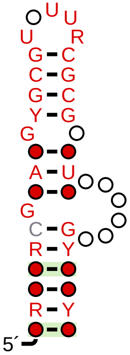

HMP2_14815_1

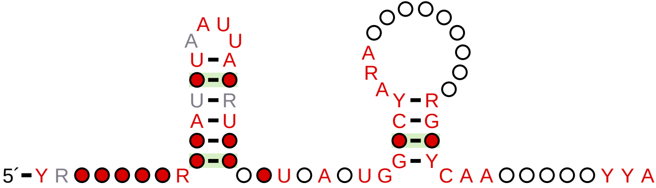

HMP2_14837_1

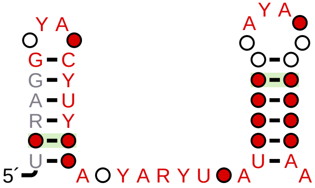

HMP2_14846_1

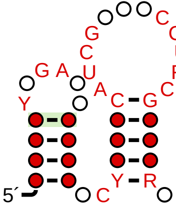

HMP2_1485_1

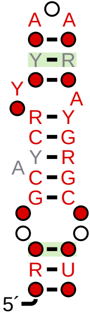

HMP2_14861_1

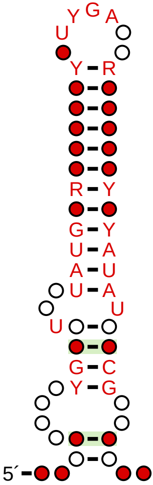

HMP2_14862_1

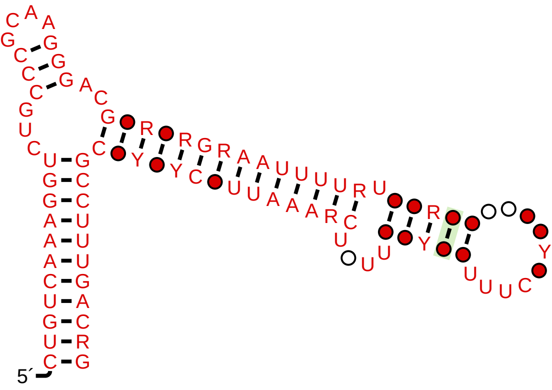

HMP2_14866_1

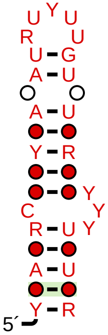

HMP2_14874_1

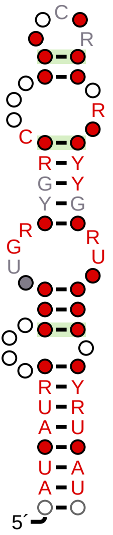

HMP2_14875_1

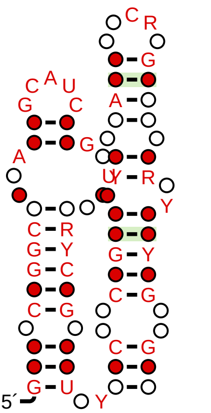

HMP2_14877_1

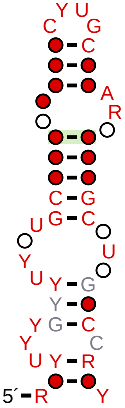

HMP2_14893_1

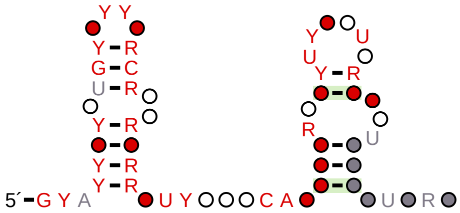

HMP2_14896_1

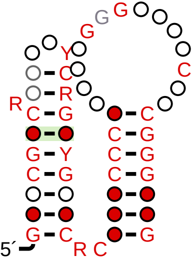

HMP2_14900_1

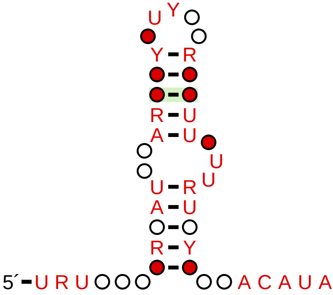

HMP2_14901_1

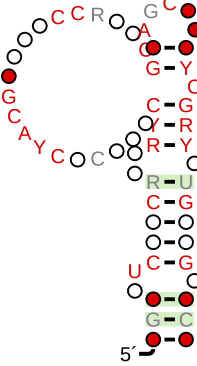

HMP2_14904_1

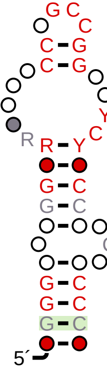

HMP2_14922_1

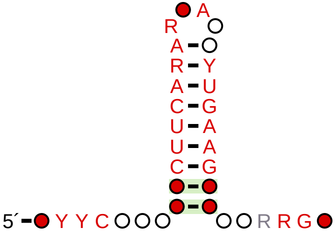

HMP2_14925_1

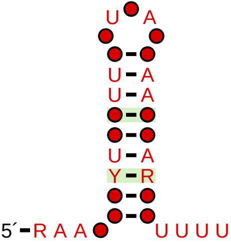

HMP2_14937_1

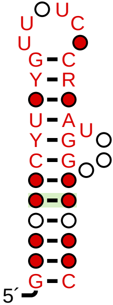

HMP2_14949_1

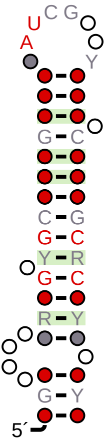

HMP2_14950_1

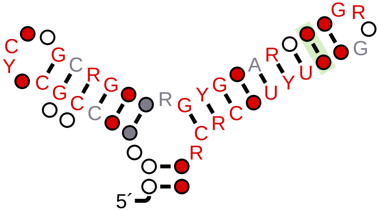

HMP2_14952_1

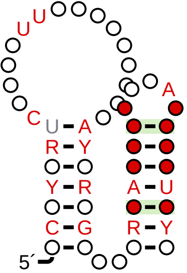

HMP2_14963_1

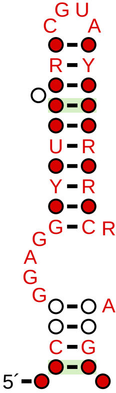

HMP2_14964_1

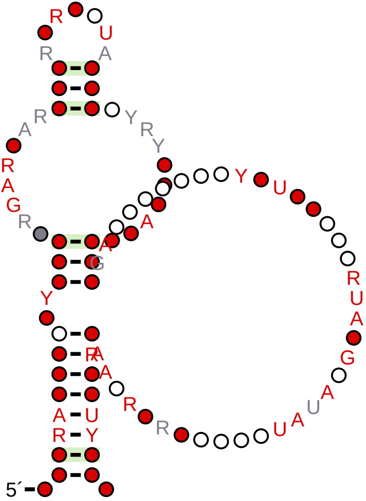

HMP2_14978_1

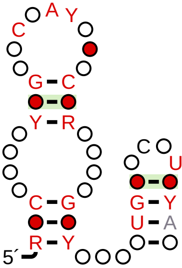

HMP2_14983_1

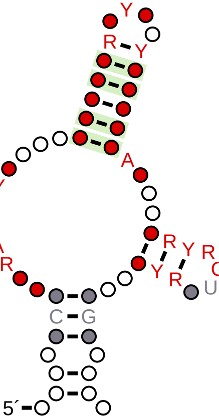

HMP2_14989_1

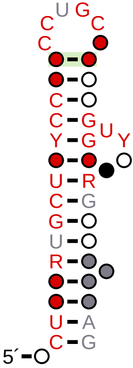

HMP2_15010_1

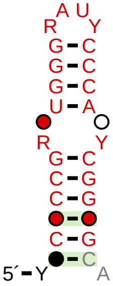

HMP2_15012_1

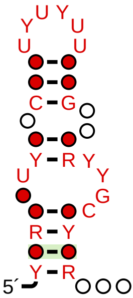

HMP2_15014_1

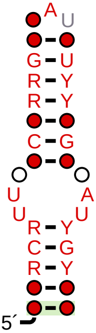

HMP2_15020_1

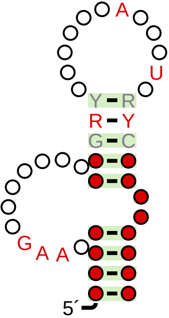

HMP2_1503_1

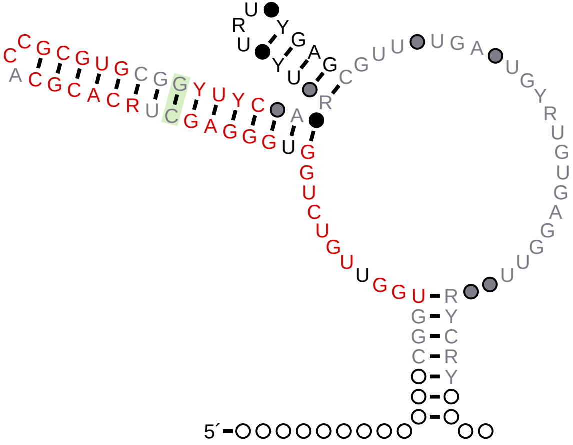

HMP2_15031_1

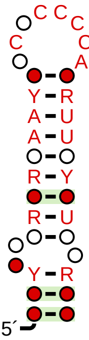

HMP2_15038_1

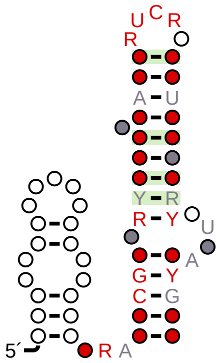

HMP2_15041_1

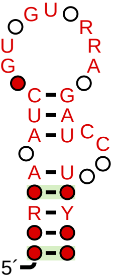

HMP2_15049_1

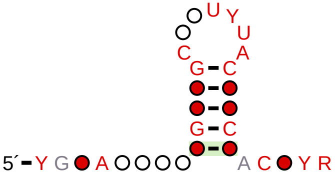

HMP2_15050_1

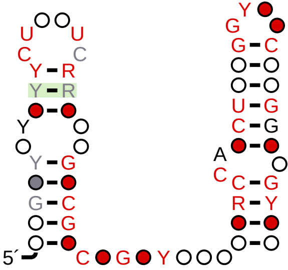

HMP2_15056_1

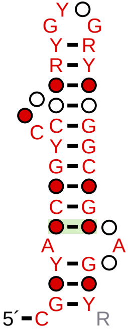

HMP2_15063_1

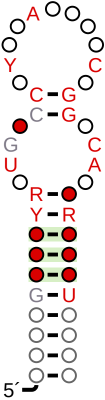

HMP2_15072_1

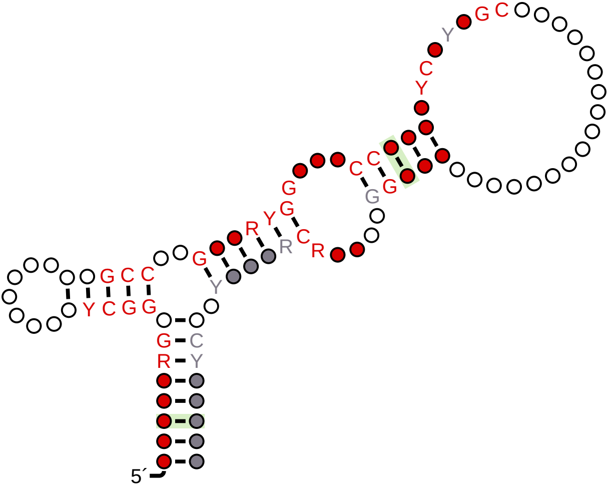

HMP2_15085_1

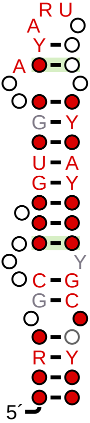

HMP2_15086_1

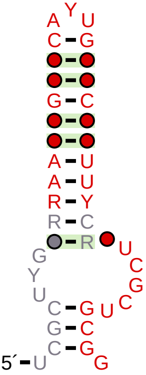

HMP2_15097_1

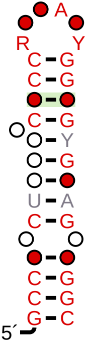

HMP2_15105_1

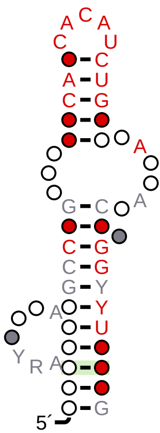

HMP2_15115_1

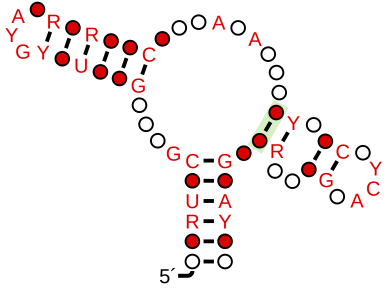

HMP2_15120_1

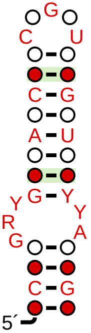

HMP2_15131_1

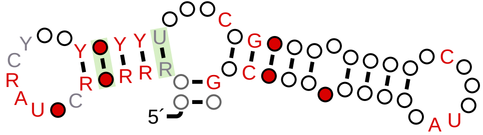

HMP2_15132_1

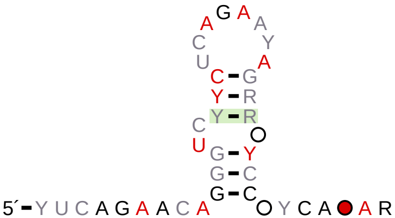

HMP2_15140_1

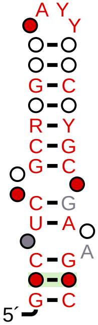

HMP2_15152_1

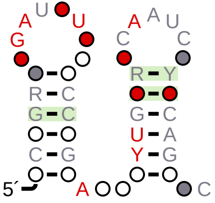

HMP2_15154_1

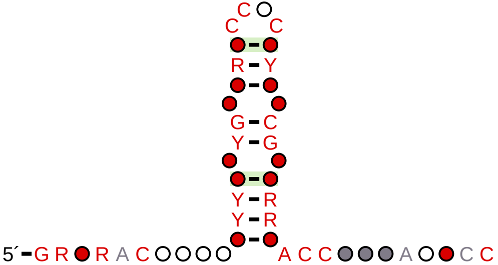

HMP2_15155_1

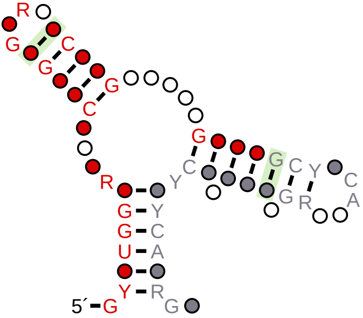

HMP2_15166_1

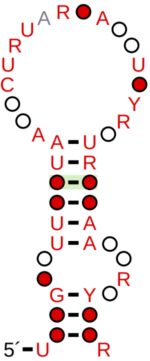

HMP2_15175_1

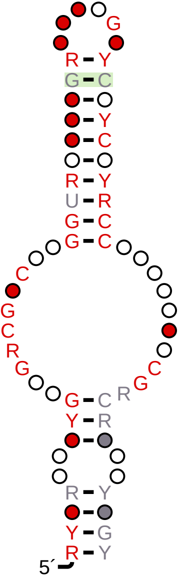

HMP2_15180_1

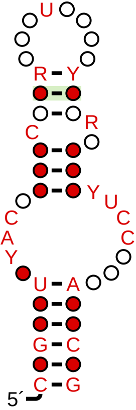

HMP2_15198_1

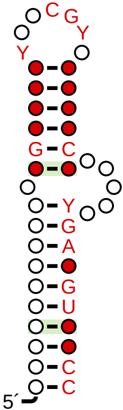

HMP2_1520_1

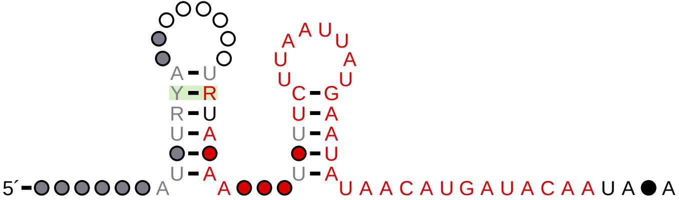

HMP2_15207_1

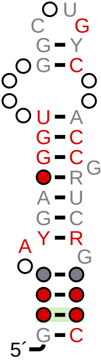

HMP2_15208_1

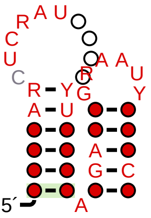

HMP2_15217_1

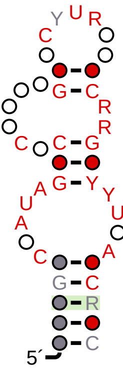

HMP2_15224_1

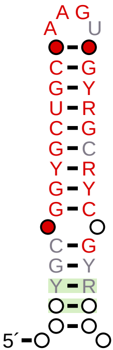

HMP2_1523_1

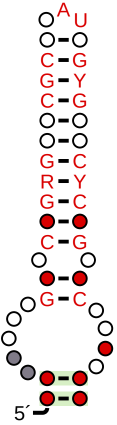

HMP2_15232_1

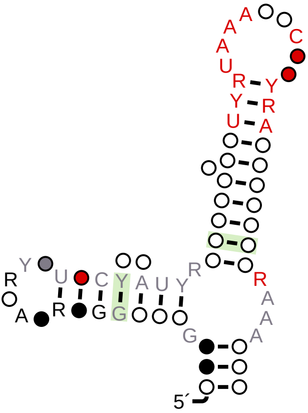

HMP2_15233_1

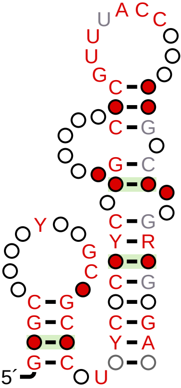

HMP2_15234_1

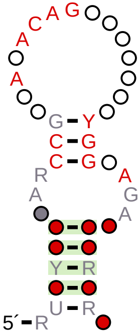

HMP2_15244_1

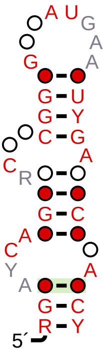

HMP2_15248_1

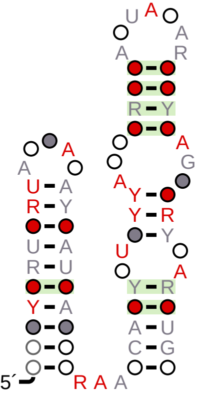

HMP2_15254_1

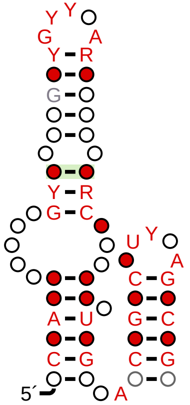

HMP2_15257_1

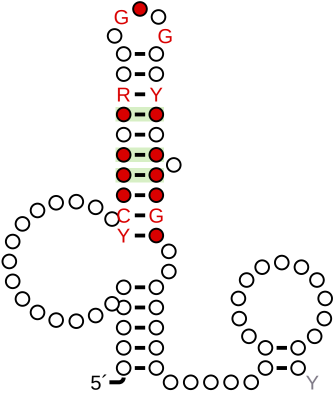

HMP2_1526_1

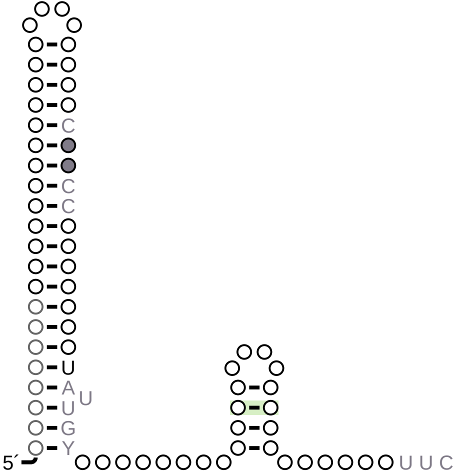

HMP2_15262_1

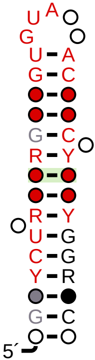

HMP2_15263_1

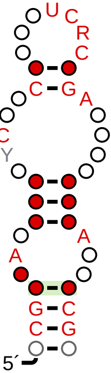

HMP2_15286_1

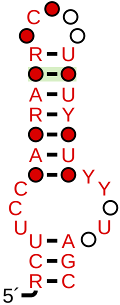

HMP2_15300_1

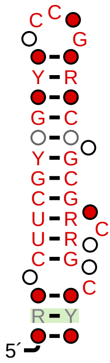

HMP2_15305_1

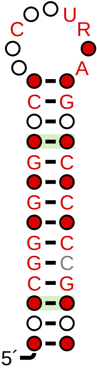

HMP2_15308_1

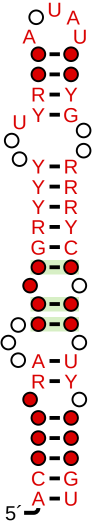

HMP2_15330_1

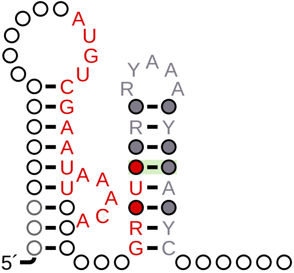

HMP2_15331_1

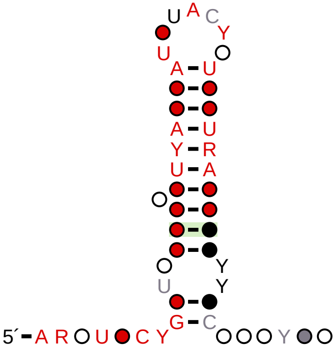

HMP2_15333_1

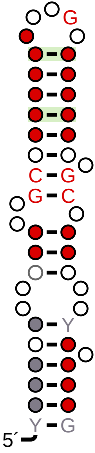

HMP2_15335_1

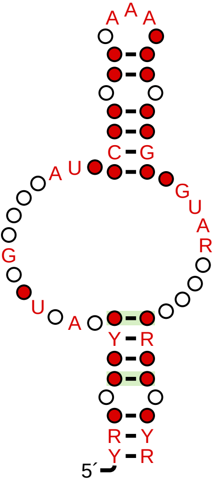

HMP2_15347_1

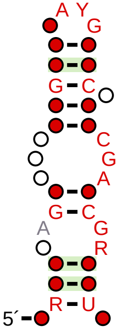

HMP2_15349_1

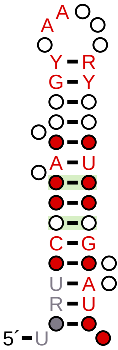

HMP2_15358_1

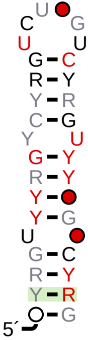

HMP2_15366_1

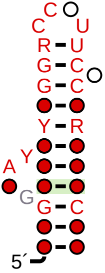

HMP2_15374_1

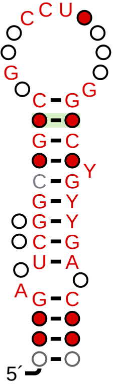

HMP2_15375_1

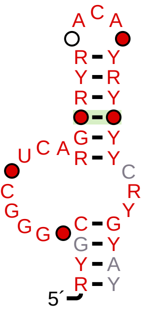

HMP2_15378_1

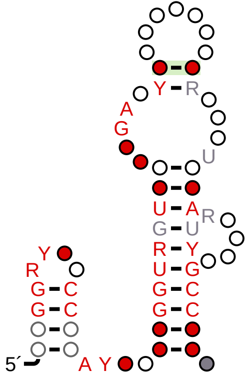

HMP2_15380_1

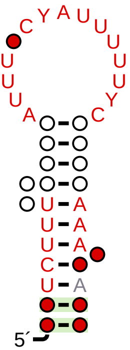

HMP2_154_1

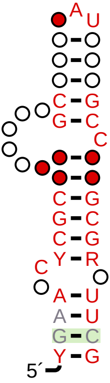

HMP2_15401_1

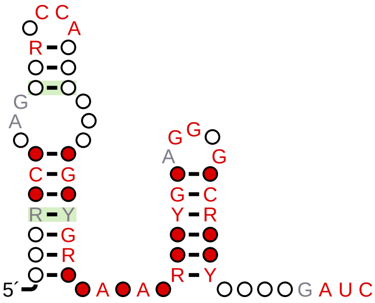

HMP2_15410_1

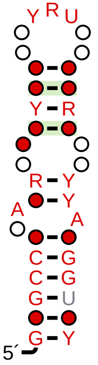

HMP2_15411_1

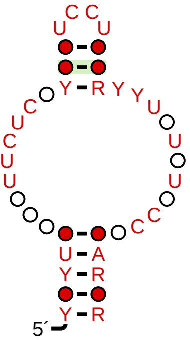

HMP2_15412_1

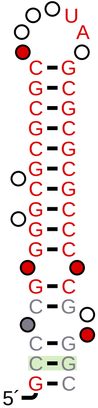

HMP2_15415_1

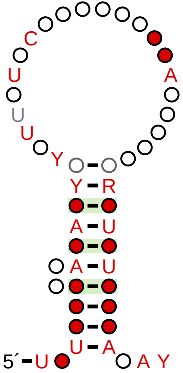

HMP2_15436_1

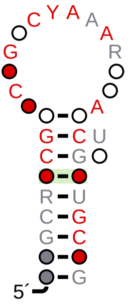

HMP2_15447_1

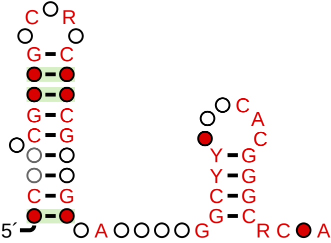

HMP2_15451_1

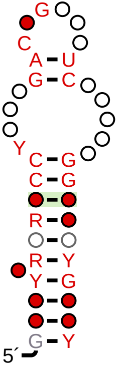

HMP2_15456_1

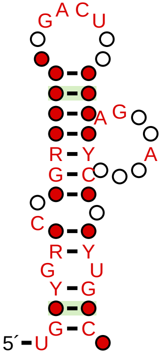

HMP2_1547_1

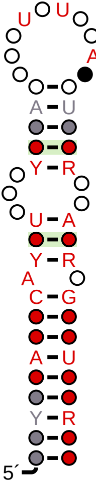

HMP2_15474_1

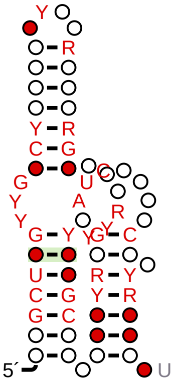

HMP2_15476_1

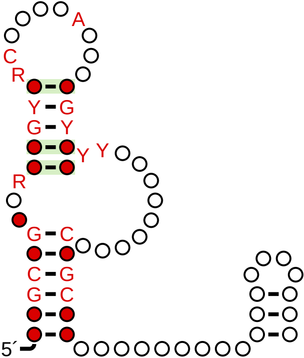

HMP2_15486_1

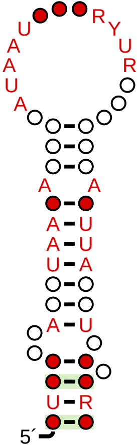

HMP2_15490_1

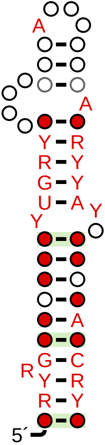

HMP2_15494_1

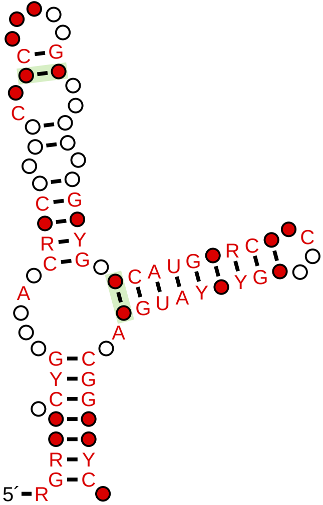

HMP2_15503_1

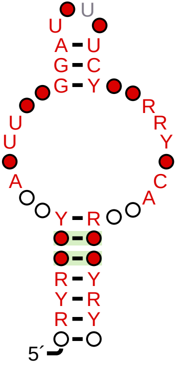

HMP2_15508_1

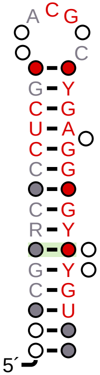

HMP2_15511_1

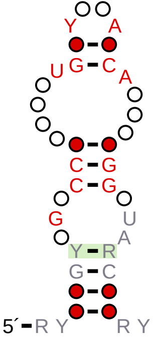

HMP2_15516_1

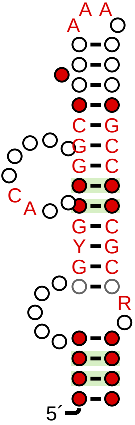

HMP2_15529_1

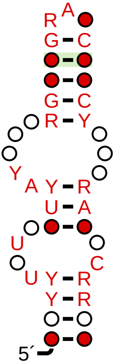

HMP2_15535_1

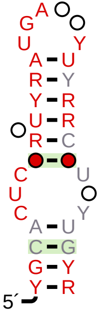

HMP2_15536_1

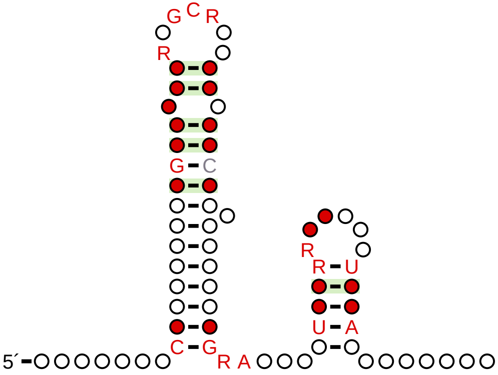

HMP2_15569_1

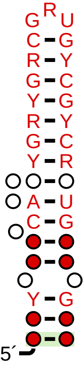

HMP2_15570_1

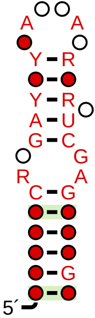

HMP2_15573_1

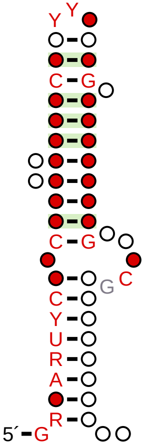

HMP2_15578_1

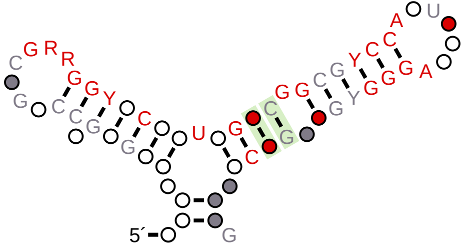

HMP2_15579_1

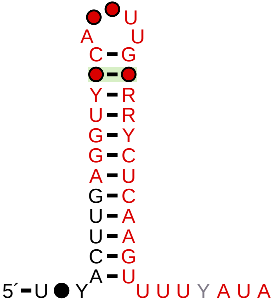

HMP2_1559_1

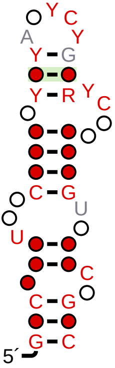

HMP2_15596_1

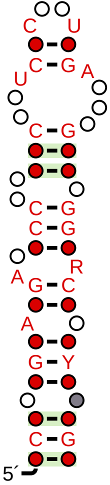

HMP2_15609_1

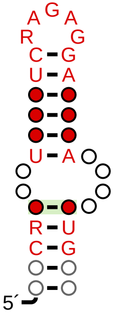

HMP2_15633_1

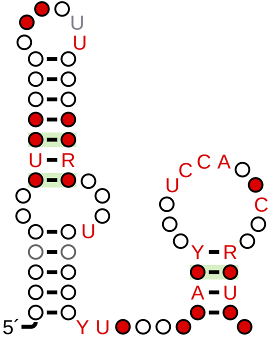

HMP2_15639_1

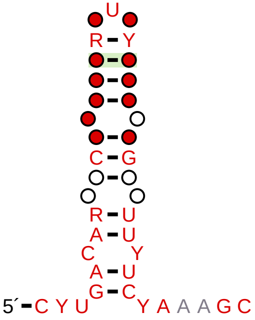

HMP2_15640_1

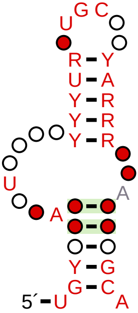

HMP2_15643_1

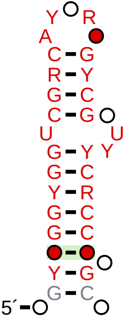

HMP2_15663_1

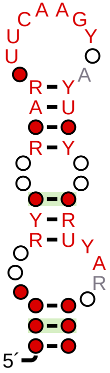

HMP2_15668_1

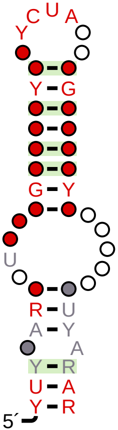

HMP2_15679_1

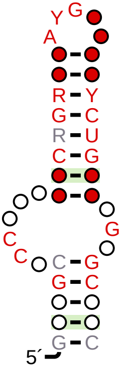

HMP2_15693_1

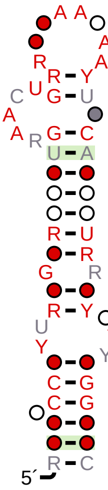

HMP2_15694_1

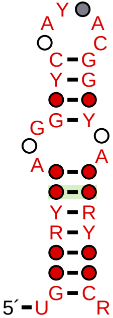

HMP2_15708_1

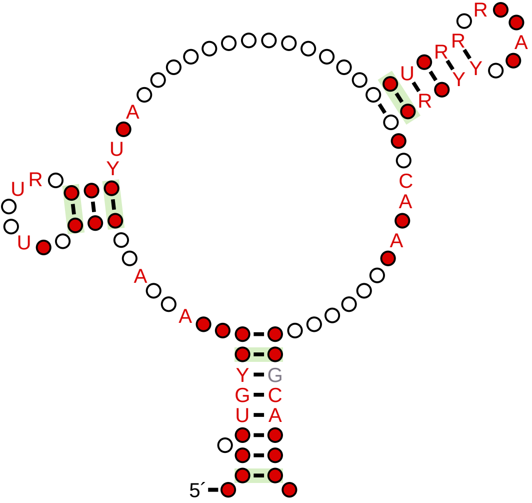

HMP2_1571_1

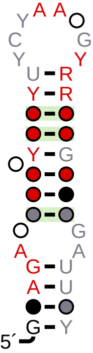

HMP2_15711_1

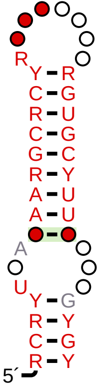

HMP2_15717_1

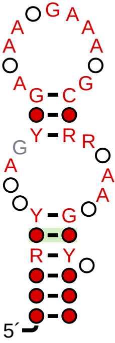

HMP2_15718_1

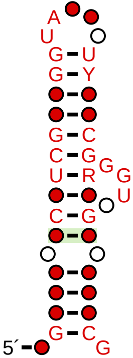

HMP2_15742_1

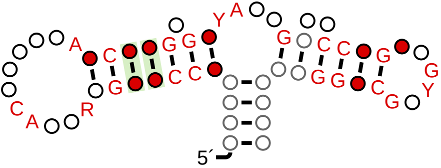

HMP2_15753_1

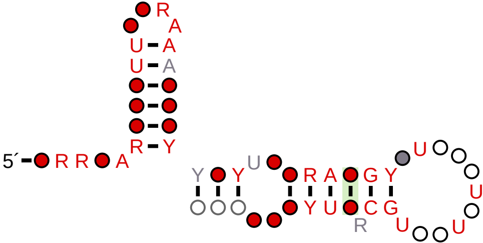

HMP2_15754_1

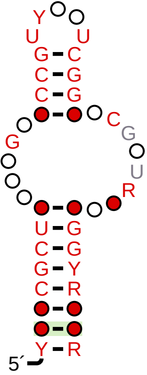

HMP2_15766_1

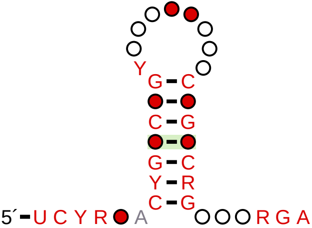

HMP2_15773_1

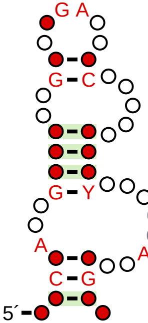

HMP2_15775_1

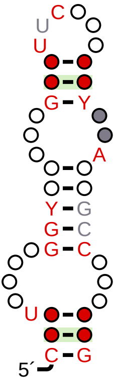

HMP2_15778_1

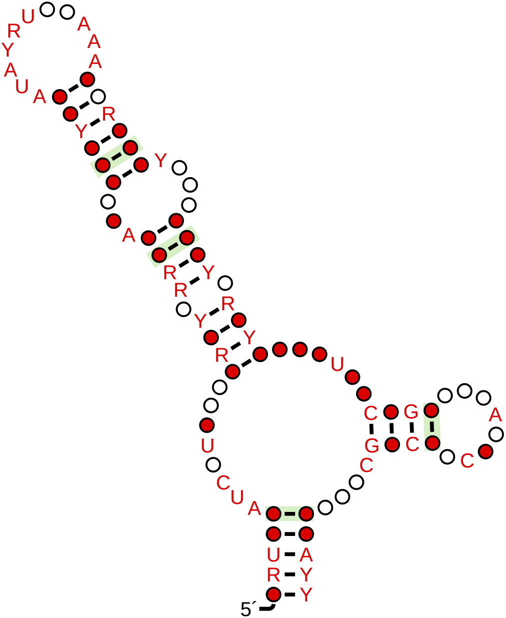

HMP2_15785_1

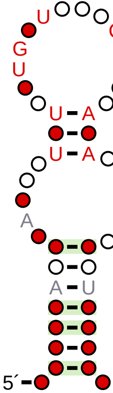

HMP2_15802_1

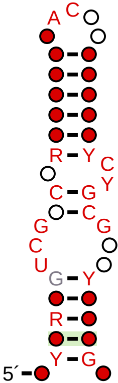

HMP2_15808_1

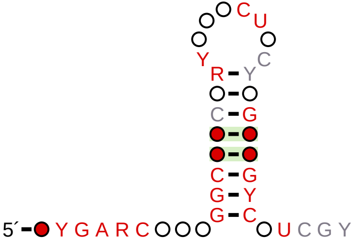

HMP2_15813_1

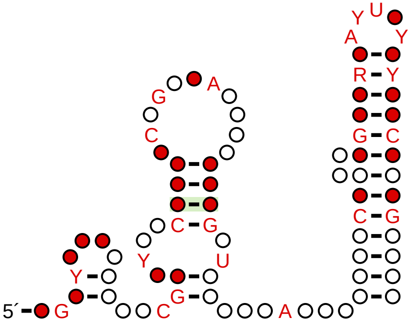

HMP2_15815_1

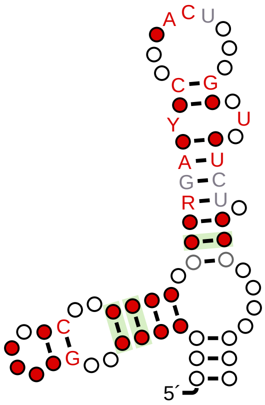

HMP2_15822_1

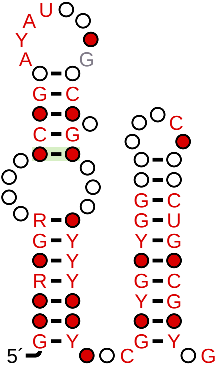

HMP2_15825_1

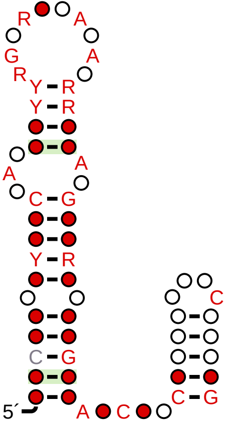

HMP2_15828_1

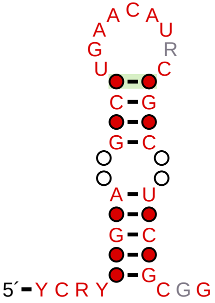

HMP2_15843_1

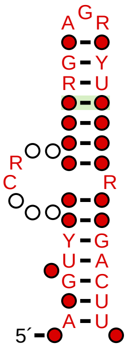

HMP2_15873_1

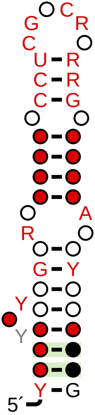

HMP2_15877_1

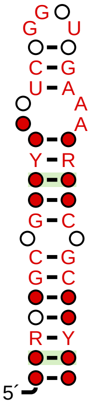

HMP2_15897_1

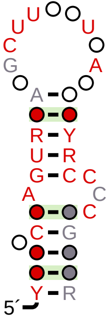

HMP2_15906_1

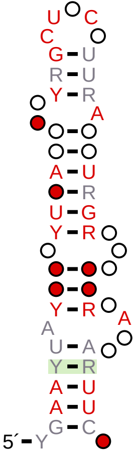

HMP2_15915_1

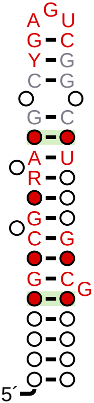

HMP2_15922_1

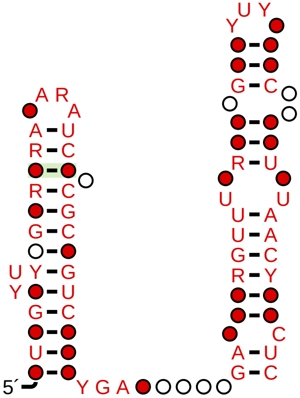

HMP2_15936_1

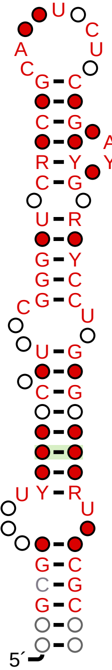

HMP2_15942_1

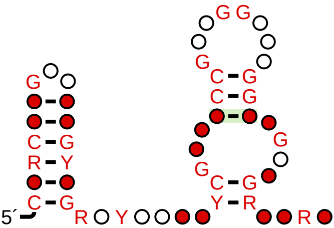

HMP2_15946_1

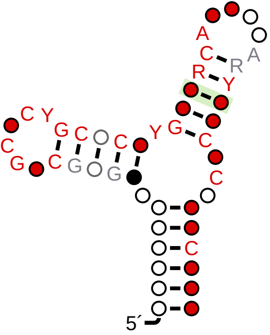

HMP2_1596_1

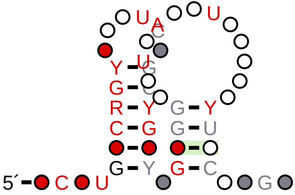

HMP2_15962_1

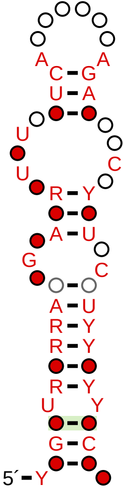

HMP2_15963_1

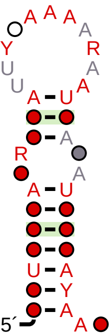

HMP2_15967_1

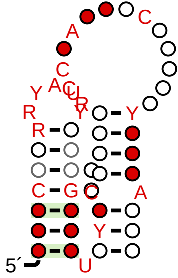

HMP2_15968_1

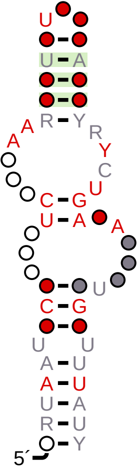

HMP2_15970_1

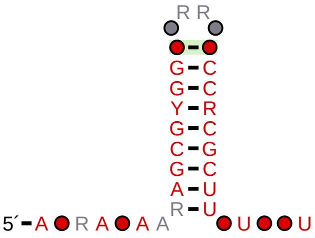

HMP2_15973_1

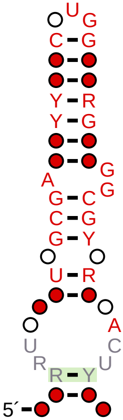

HMP2_15981_1

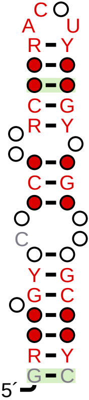

HMP2_15984_1

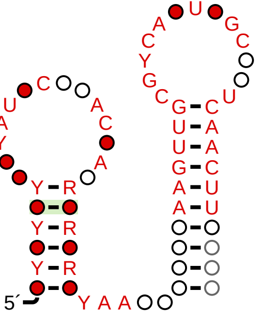

HMP2_1599_1

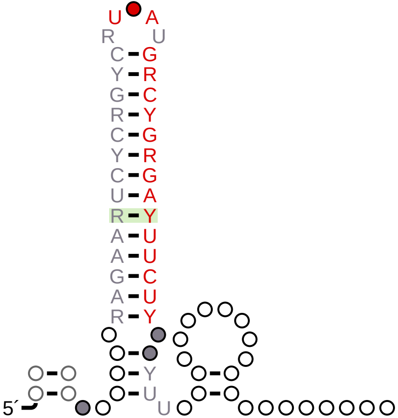

HMP2_15994_1

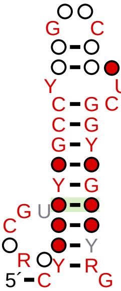

HMP2_16003_1

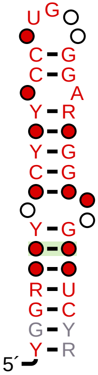

HMP2_16022_1

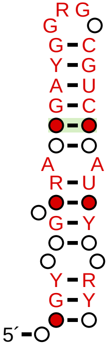

HMP2_16024_1

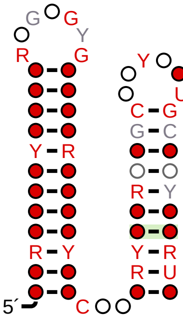

HMP2_16030_1

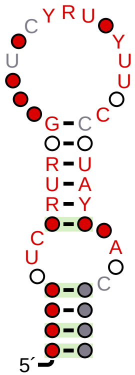

HMP2_16037_1

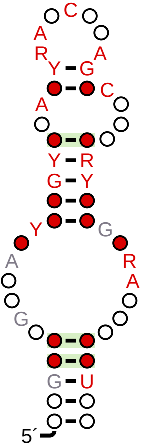

HMP2_16046_1

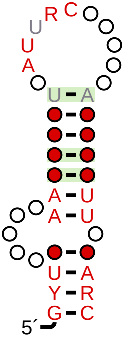

HMP2_16062_1

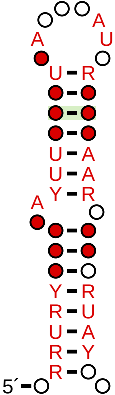

HMP2_16063_1

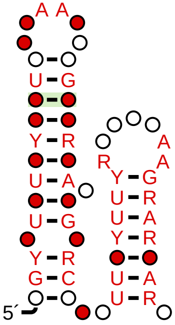

HMP2_16066_1

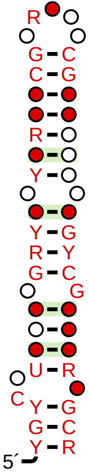

HMP2_16067_1

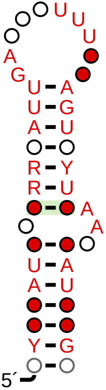

HMP2_16077_1

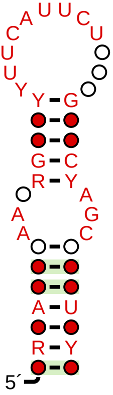

HMP2_16081_1

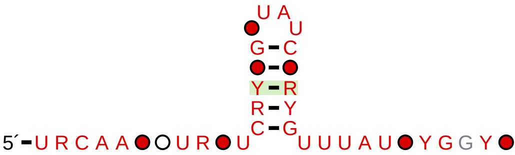

HMP2_16085_1

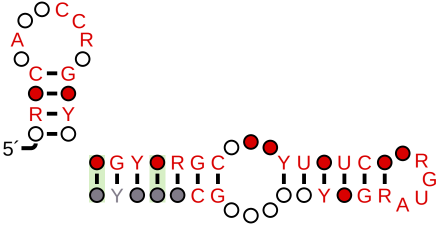

HMP2_16089_1

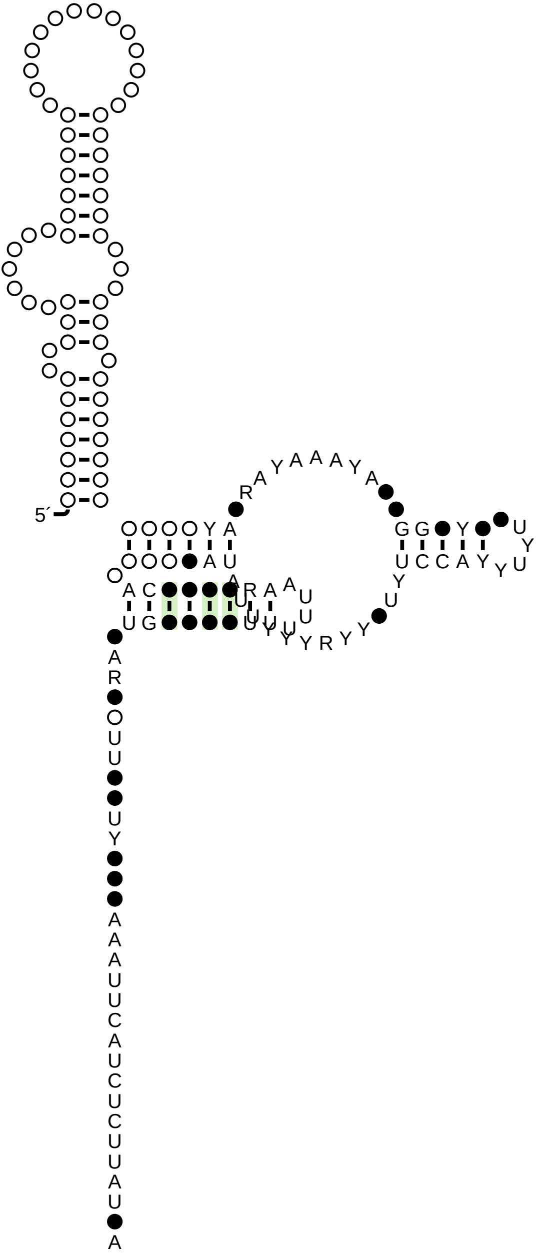

HMP2_16095_1

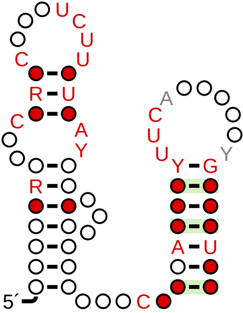

HMP2_1611_1

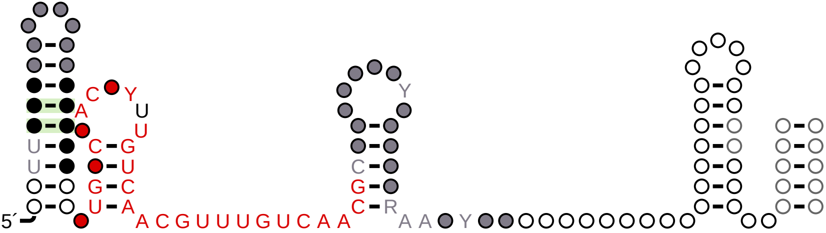

HMP2_16114_1

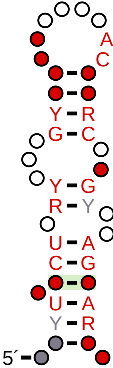

HMP2_16117_1

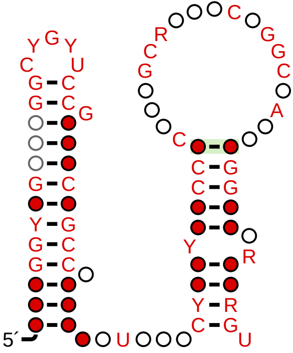

HMP2_1612_1

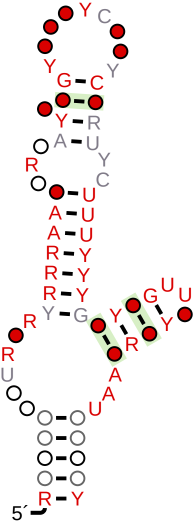

HMP2_16123_1

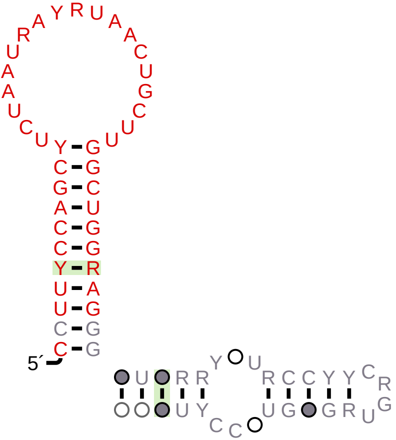

HMP2_16124_1

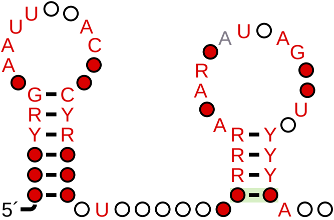

HMP2_16126_1

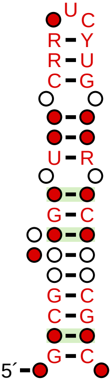

HMP2_16133_1

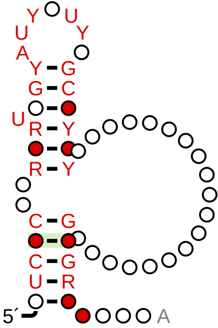

HMP2_16134_1

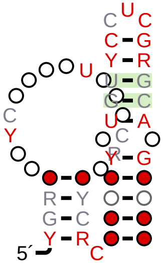

HMP2_1614_1

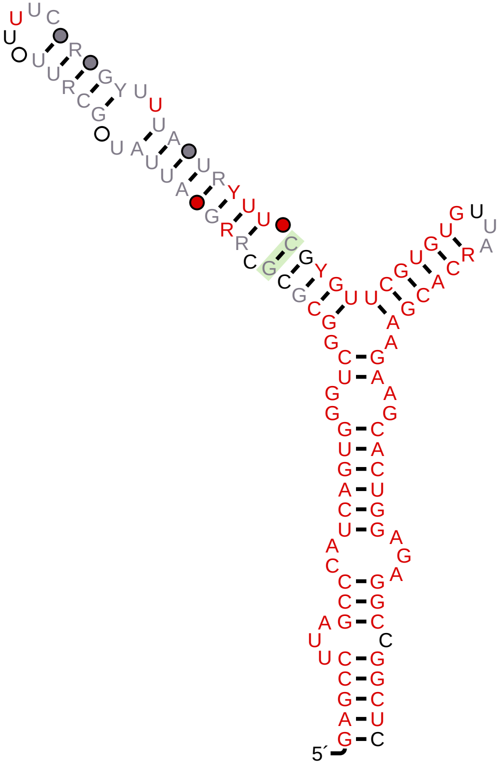

HMP2_16149_1

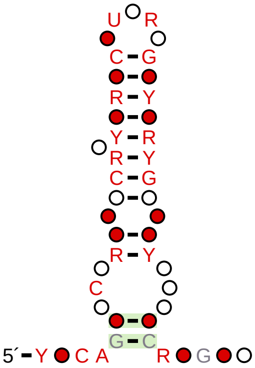

HMP2_16153_1

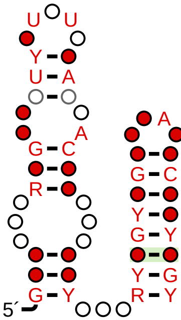

HMP2_16167_1

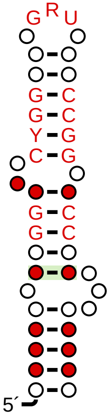

HMP2_16173_1

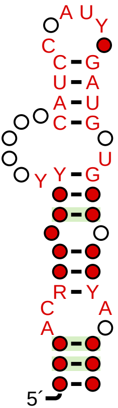

HMP2_16179_1

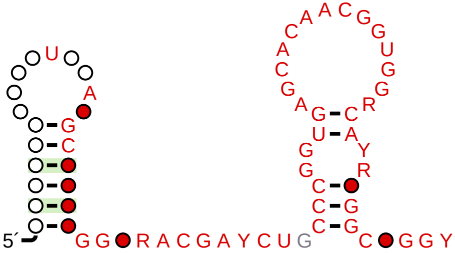

HMP2_16181_1

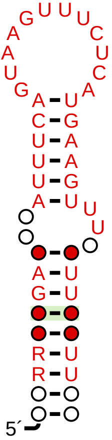

HMP2_16187_1

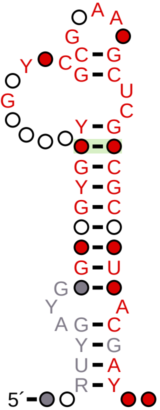

HMP2_16193_1

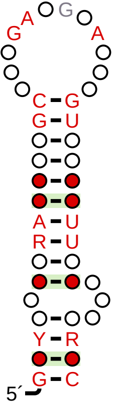

HMP2_16197_1

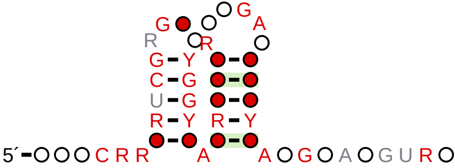

HMP2_16201_1

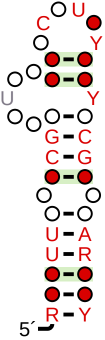

HMP2_16211_1

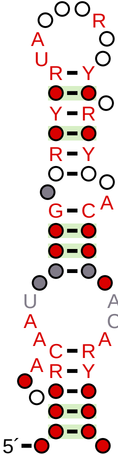

HMP2_1622_1

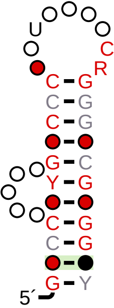

HMP2_16223_1

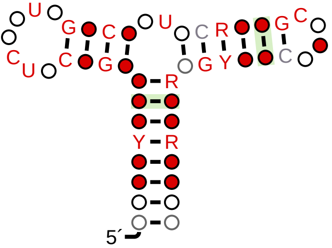

HMP2_16224_1

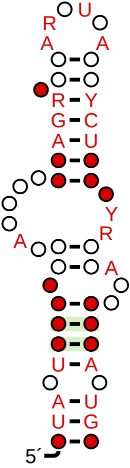

HMP2_16234_1

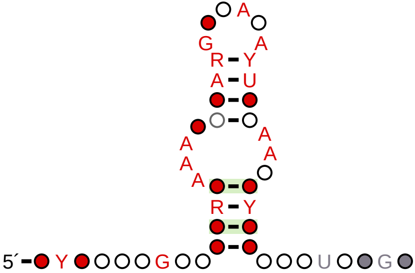

HMP2_16239_1

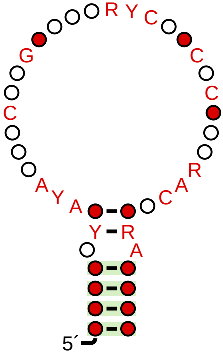

HMP2_16242_1

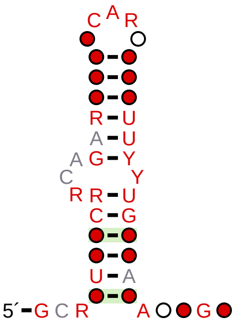

HMP2_16247_1

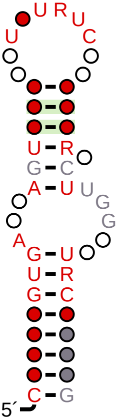

HMP2_16248_1

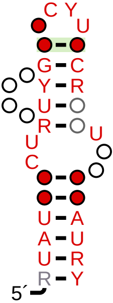

HMP2_16252_1

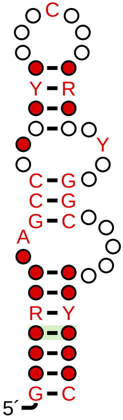

HMP2_16263_1

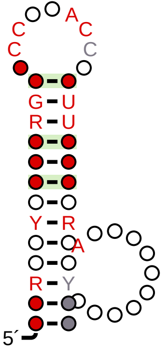

HMP2_16271_1

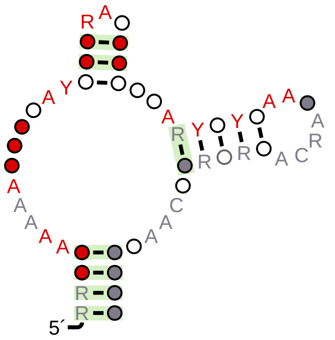

HMP2_16273_1

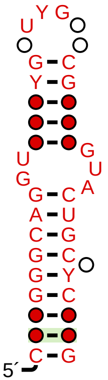

HMP2_16275_1

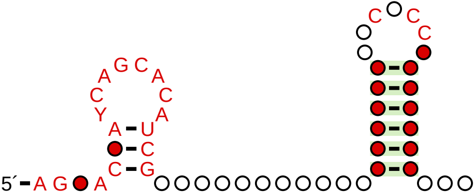

HMP2_16308_1

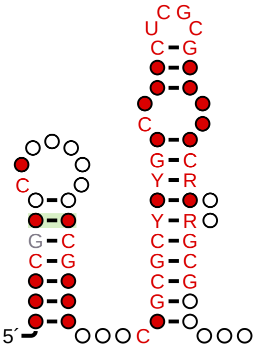

HMP2_16311_1

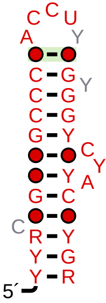

HMP2_16324_1

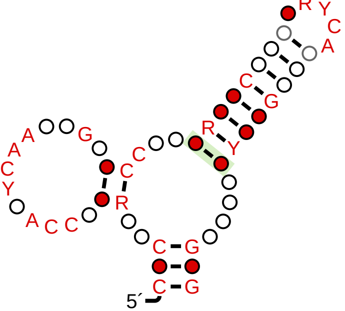

HMP2_16339_1

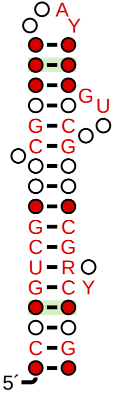

HMP2_16341_1

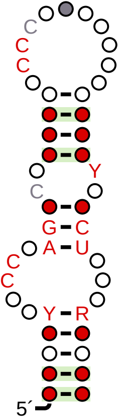

HMP2_16355_1

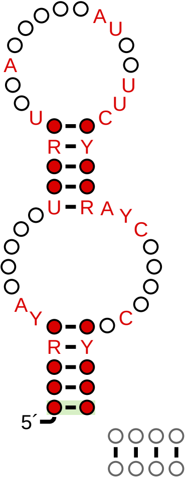

HMP2_16372_1

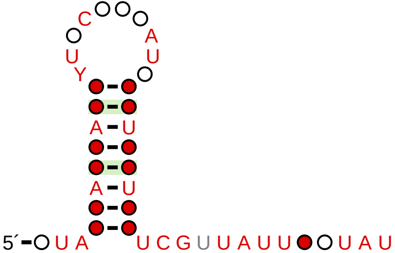

HMP2_16375_1

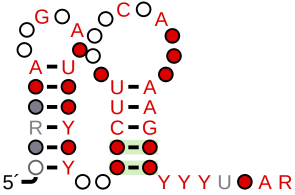

HMP2_16377_1

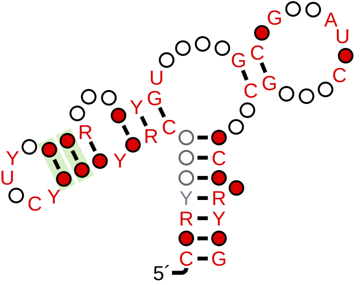

HMP2_16389_1

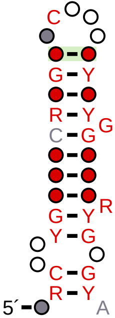

HMP2_16390_1

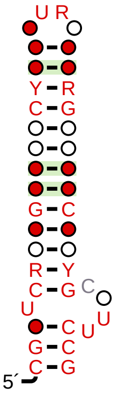

HMP2_16397_1

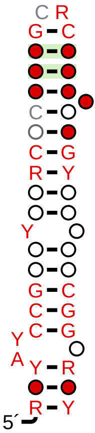

HMP2_164_1

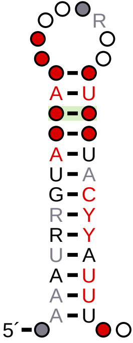

HMP2_16407_1

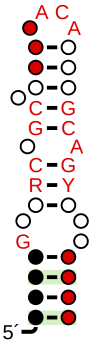

HMP2_16416_1

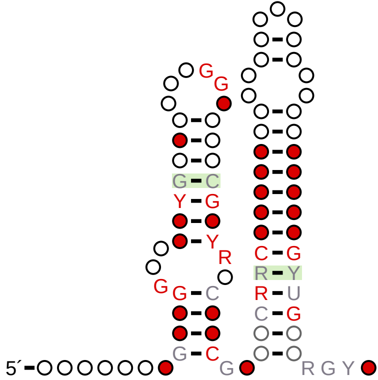

HMP2_16418_1

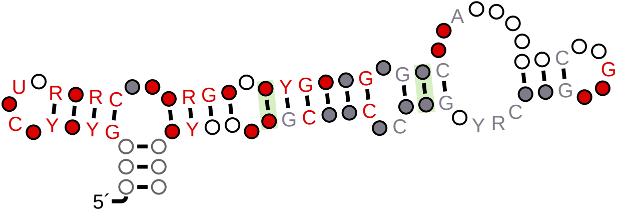

HMP2_16432_1

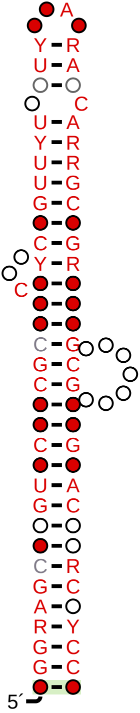

HMP2_16435_1

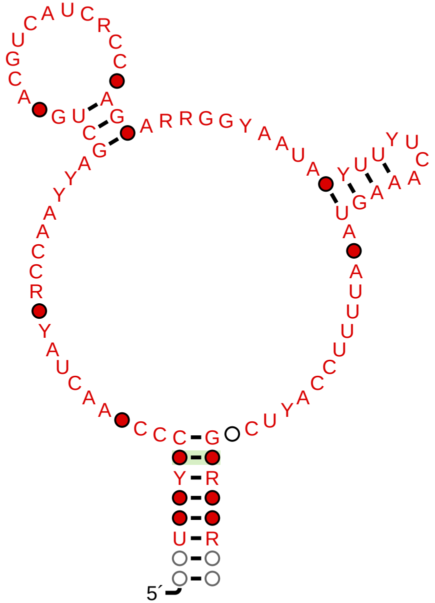

HMP2_16437_1

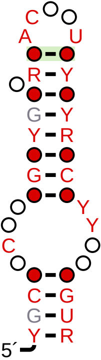

HMP2_16457_1

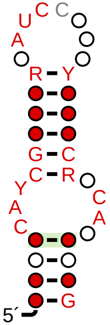

HMP2_1647_1

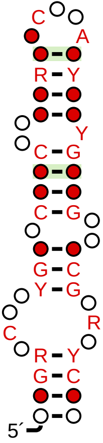

HMP2_16474_1

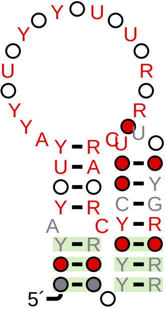

HMP2_16477_1

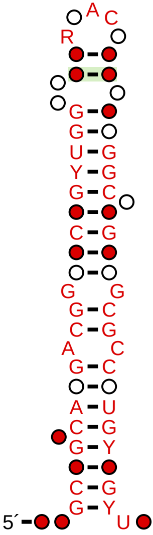

HMP2_16479_1

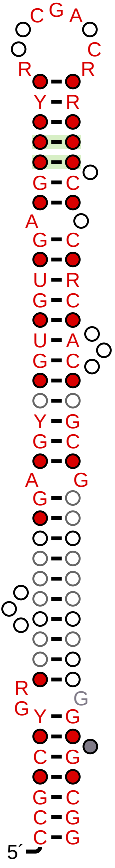

HMP2_16480_1

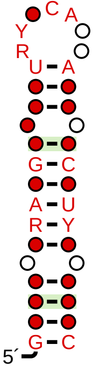

HMP2_16491_1

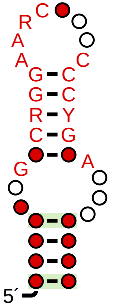

HMP2_16496_1

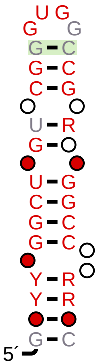

HMP2_16498_1

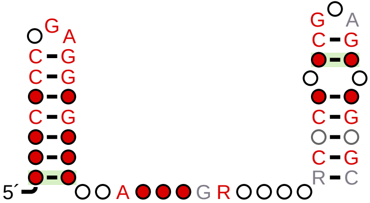

HMP2_16502_1

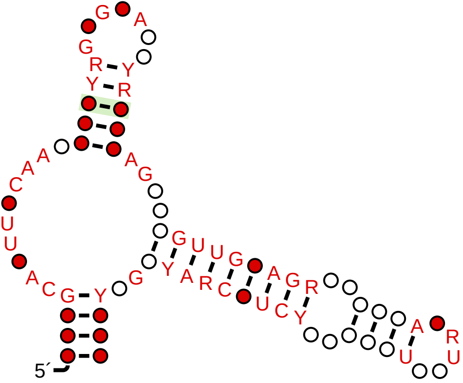

HMP2_16508_1

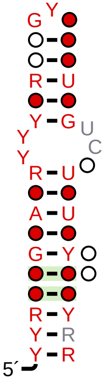

HMP2_1679_1

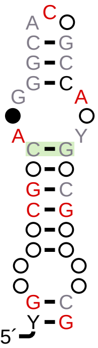

HMP2_1680_1

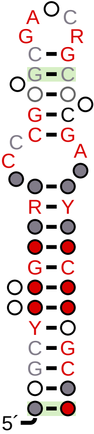

HMP2_1681_1

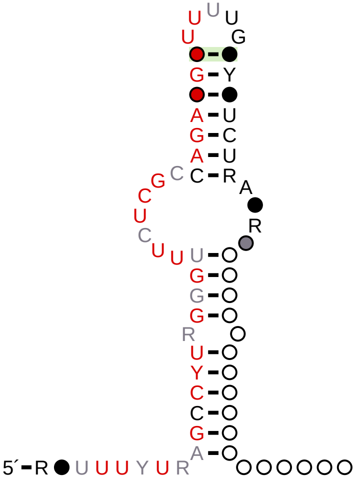

HMP2_1688_1

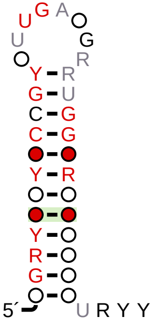

HMP2_169_1

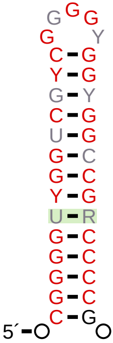

HMP2_1704_1

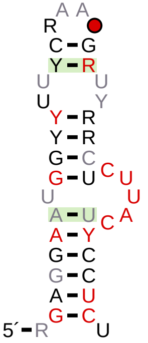

HMP2_1706_1

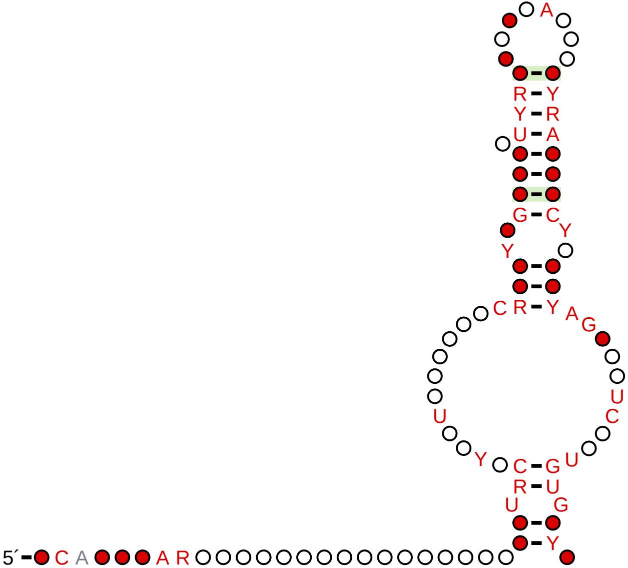

HMP2_1710_1

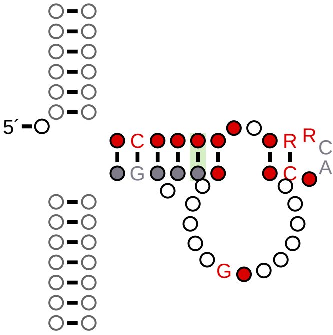

HMP2_1711_1

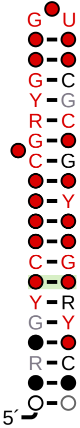

HMP2_173_1

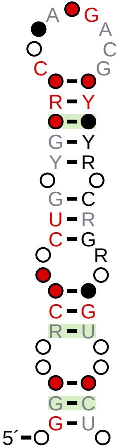

HMP2_1754_1

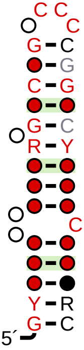

HMP2_1755_1

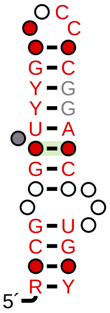

HMP2_1761_1

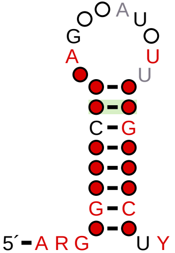

HMP2_1768_1

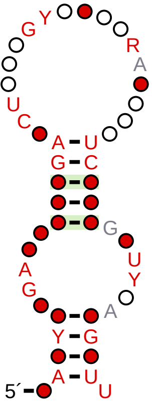

HMP2_177_1

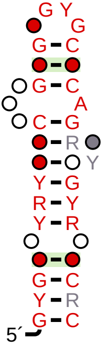

HMP2_1770_1

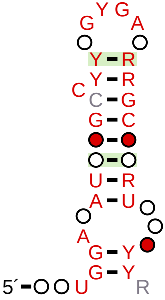

HMP2_1797_1

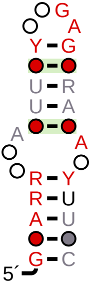

HMP2_1798_1

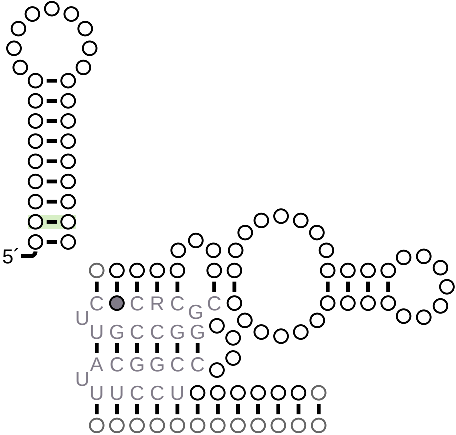

HMP2_1800_1

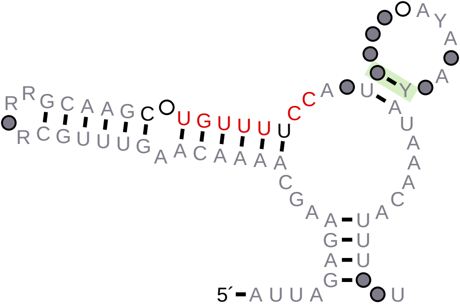
A Y
A
A
Y
C A U A
R R G C A A G C U G U U U U C C U
R C G U U U G A A C A A A A U
A
C A
A A
C
G A
A U C
G U
A U
G
5´ A U U A U

HMP2_1814_1

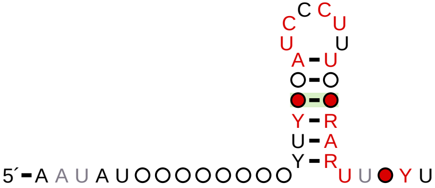

HMP2_1827_1

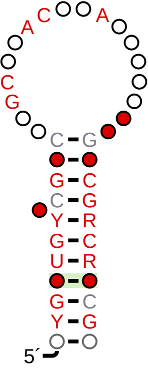

HMP2_184_1

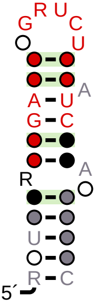

HMP2_185_1

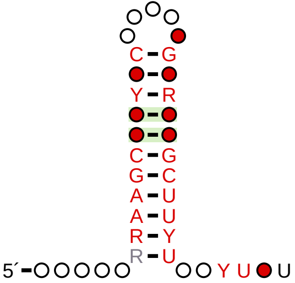

HMP2_1861_1

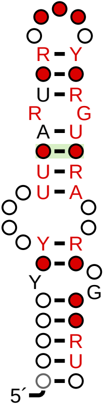

HMP2_1869_1

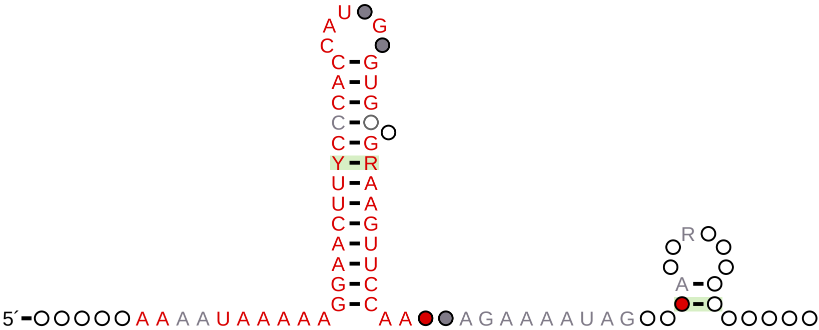

U
A
G
C
C G
A U
C G
C
C G
Y R
U A
U A
C G
A U
A U
G C
G C
5´
A A A A U A A A A A
A A
A G A A A A U A G
R
A

HMP2_1888_1

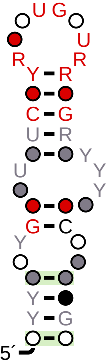

HMP2_1900_1

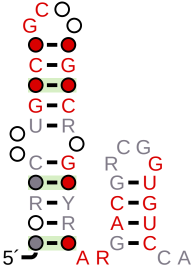

HMP2_193_1

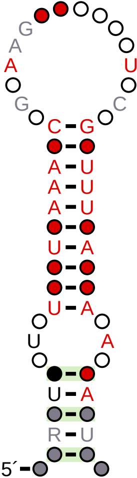

HMP2_1930_1

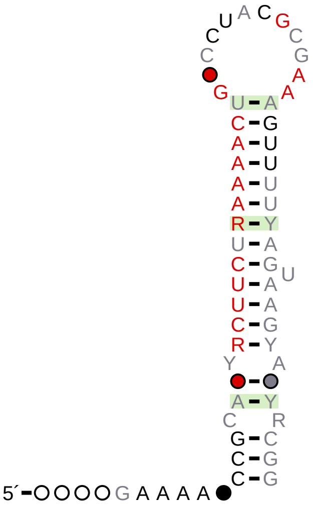

HMP2_1931_1

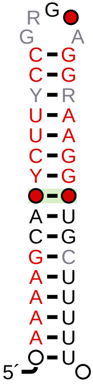

HMP2_1947_1

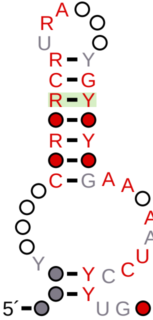

HMP2_1966_1

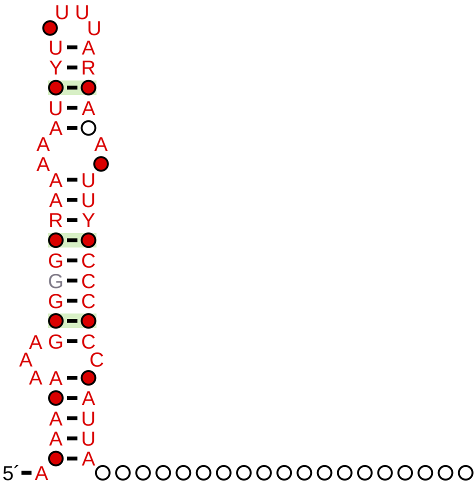

HMP2_1983_1

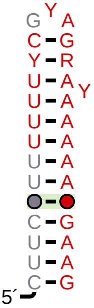

HMP2_1997_1

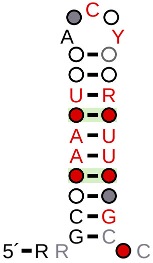

HMP2_20_1

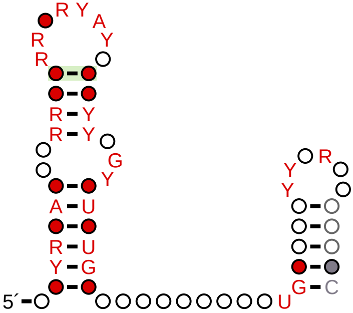

HMP2_2000_1

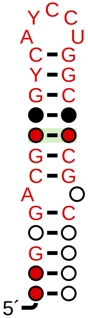

HMP2_2012_1

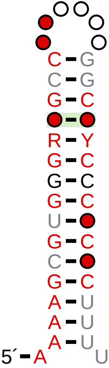

HMP2_204_1

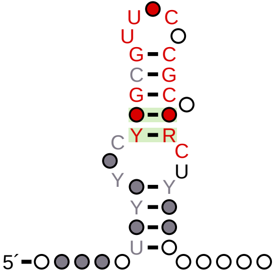

HMP2_2049_1

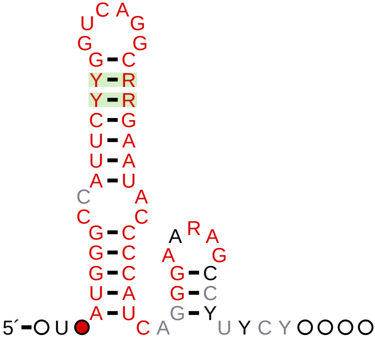

U C A G
G G
G C
Y R
Y R
C G
U A
U A
A U
C A
C C
G C
G C
G C
U A
A U
A R A
A G
G C
G C
G Y
5´ O U C A U Y C Y O O O O

HMP2_2050_1

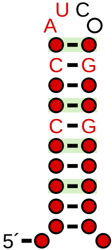

HMP2_2062_1

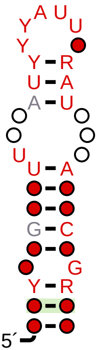

HMP2_2063_1

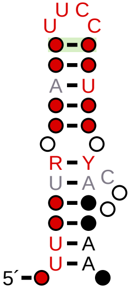

HMP2_2081_1

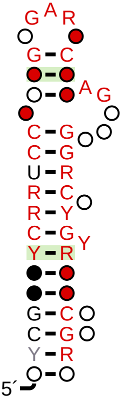

HMP2_2096_1

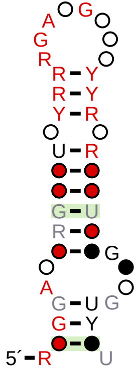

HMP2_2098_1

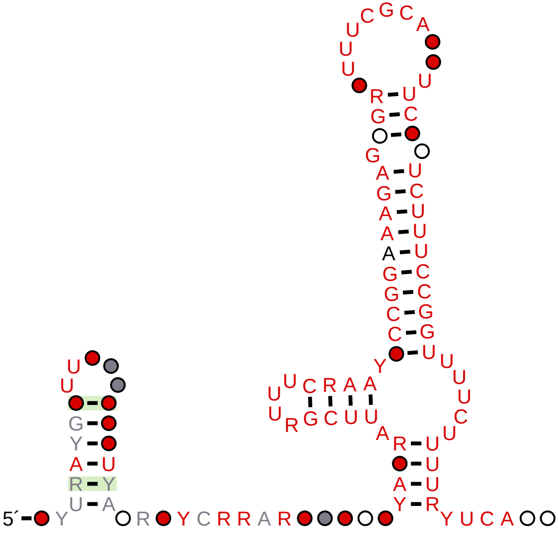

HMP2_211_1

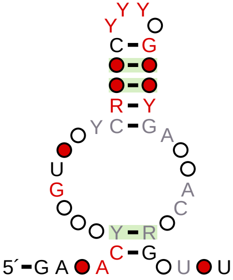

HMP2_2111_1

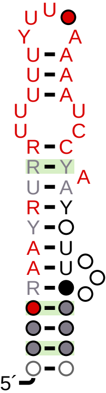

HMP2_2115_1

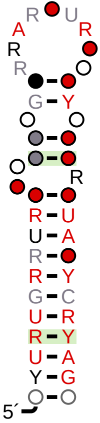

HMP2_212_1

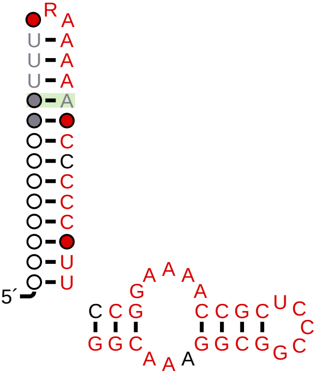

HMP2_2129_1

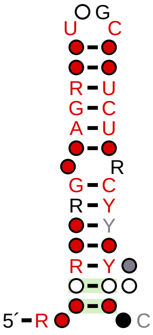

HMP2_2137_1

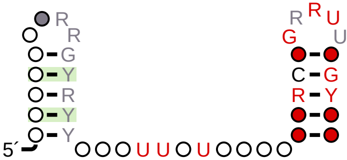

HMP2_2141_1

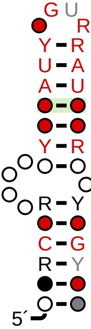

HMP2_2142_1

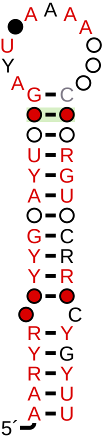

HMP2_2147_1

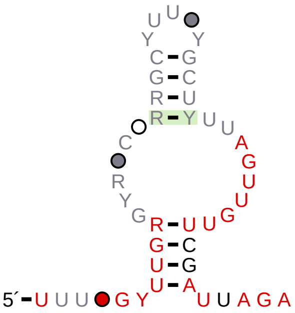

HMP2_2153_1

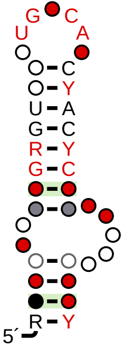

HMP2_2166_1

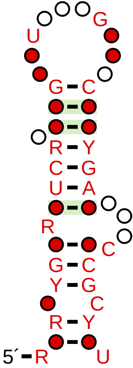

HMP2_2168_1

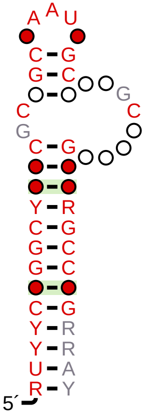

HMP2_2175_1

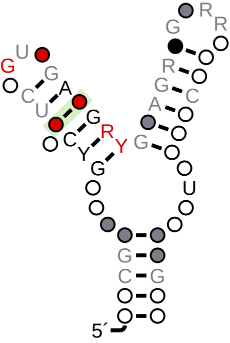

HMP2_2192_1

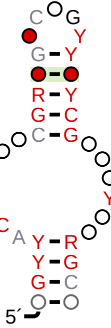

HMP2_2193_1

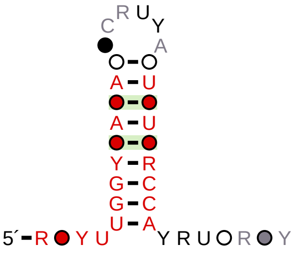

HMP2_2199_1

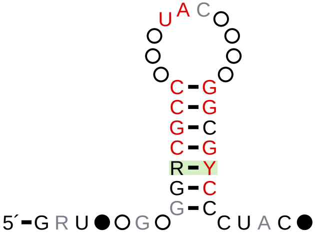

HMP2_22_1

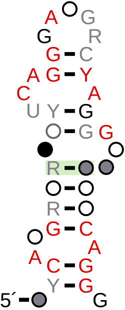

HMP2_2205_1

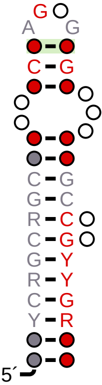

HMP2_2207_1

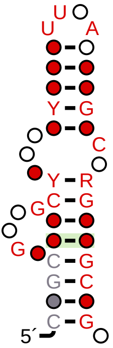

HMP2_2216_1

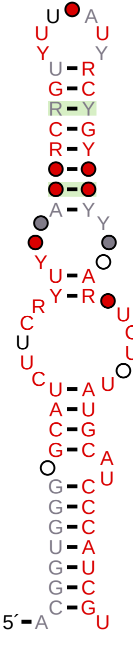

HMP2_2219_1

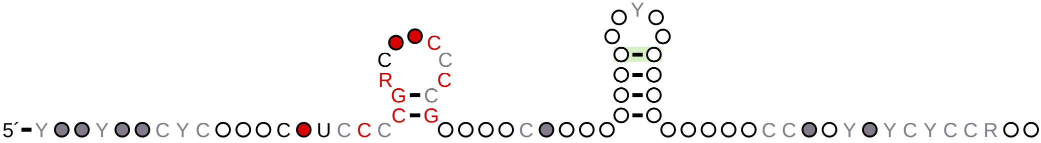

HMP2_2221_1

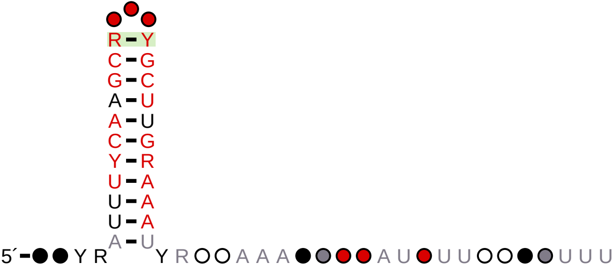

HMP2_2226_1

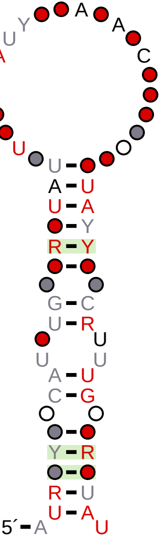

HMP2_2229_1

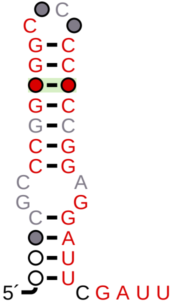

HMP2_2242_1

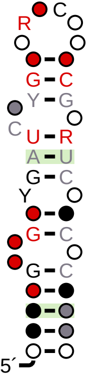

HMP2_2247_1

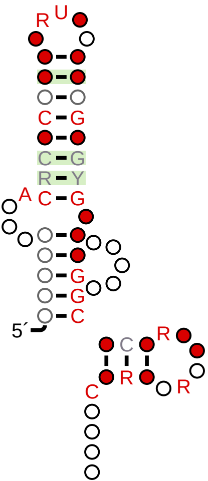

HMP2_2254_1

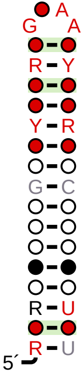

HMP2_2259_1

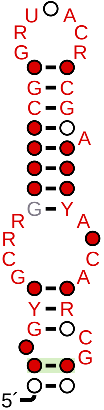

HMP2_226_1

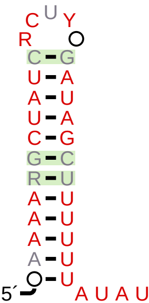

HMP2_2261_1

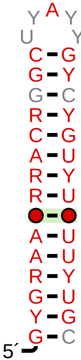

HMP2_2274_1

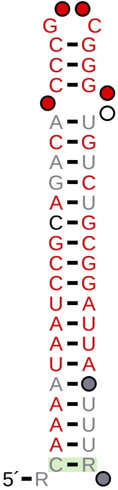

HMP2_2275_1

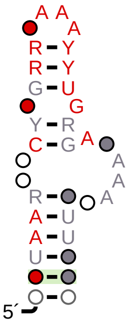

HMP2_2281_1

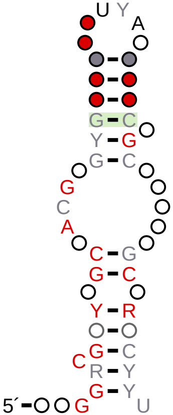

HMP2_2283_1

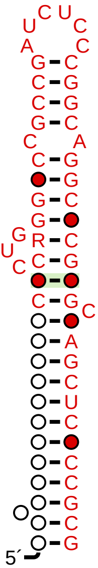

HMP2_2294_1

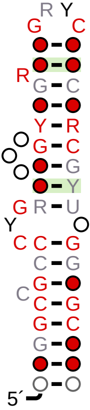

HMP2_2304_1

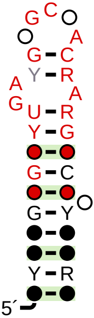

HMP2_2308_1

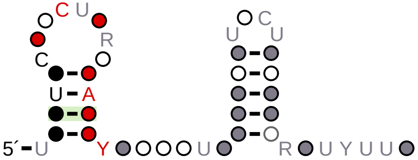

HMP2_2319_1

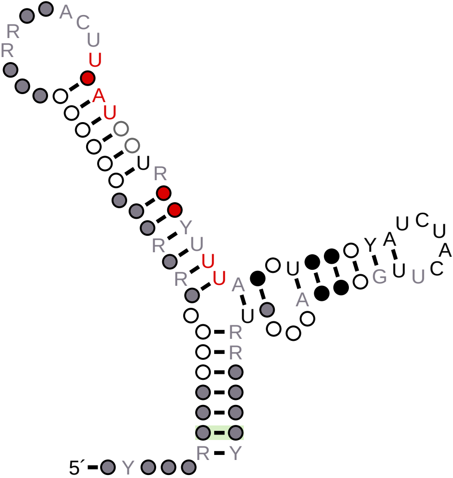

HMP2_2323_1

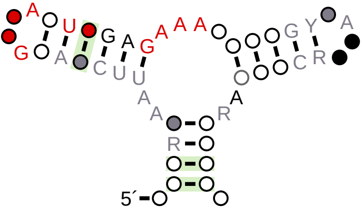

HMP2_2325_1

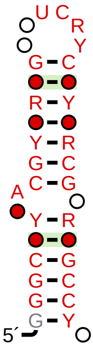

HMP2_2327_1

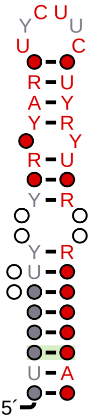

HMP2_234_1

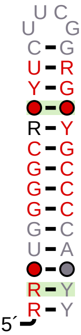

HMP2_2344_1

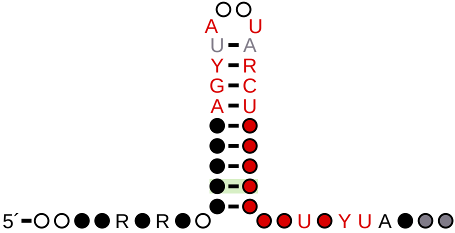

HMP2_2349_1

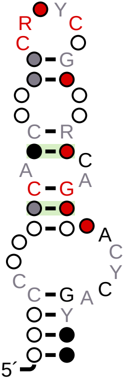

HMP2_2366_1

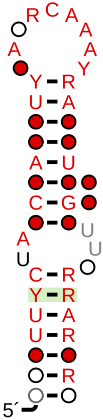

HMP2_2389_1

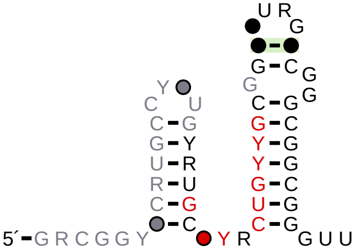

HMP2_2390_1

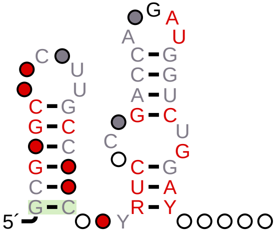

HMP2_2392_1

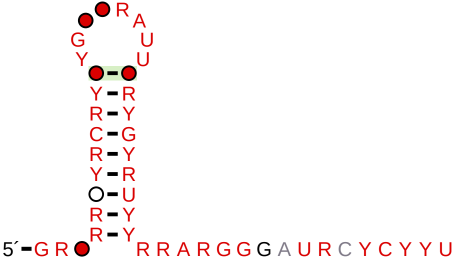

HMP2_2393_1

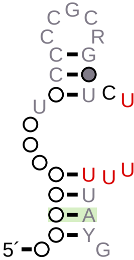

HMP2_2401_1

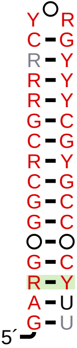

HMP2_241_1

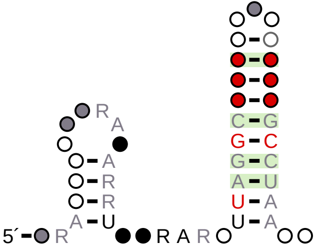

HMP2_2424_1

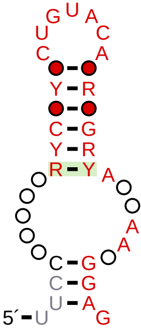

HMP2_2431_1

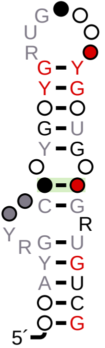

HMP2_2432_1

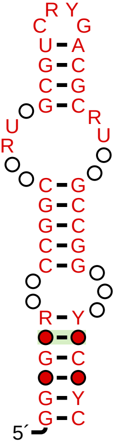

HMP2_2440_1

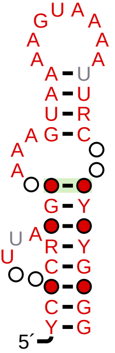

HMP2_2445_1

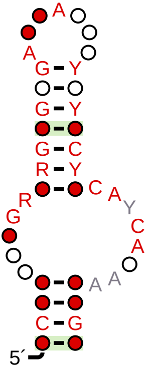

HMP2_2447_1

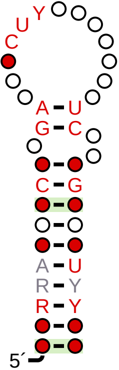

HMP2_2463_1

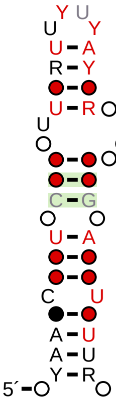

HMP2_249_1

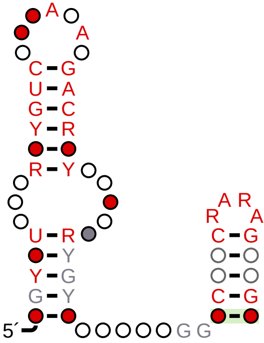

HMP2_2493_1

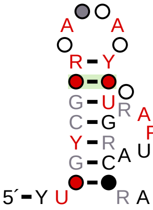

HMP2_2502_1

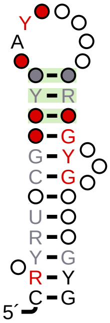

HMP2_251_1

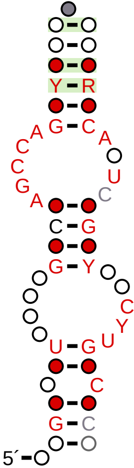

HMP2_2514_1

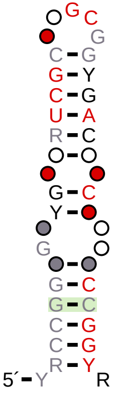

HMP2_2519_1

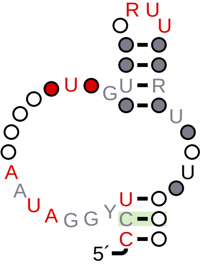

HMP2_2520_1

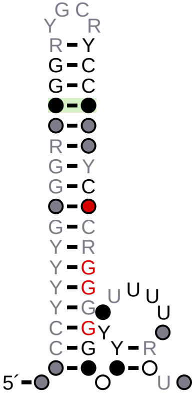

HMP2_2527_1

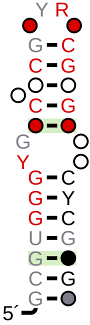

HMP2_2533_1

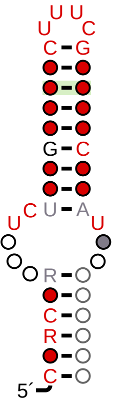

HMP2_2543_1

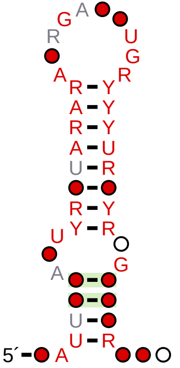

HMP2_2544_1

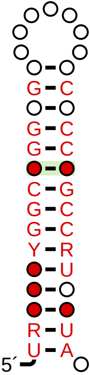

HMP2_2553_1

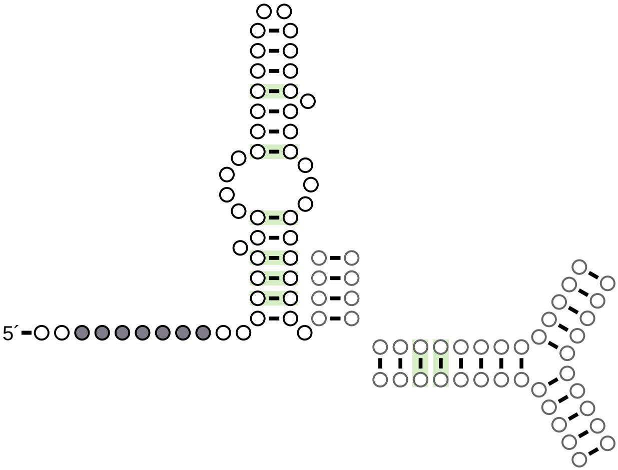

HMP2_2560_1

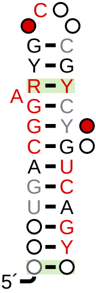

HMP2_2563_1

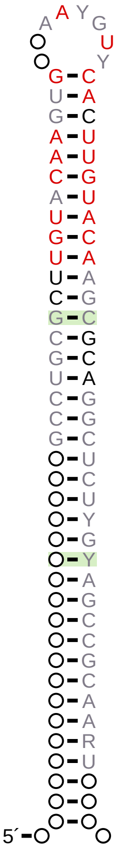

HMP2_2567_1

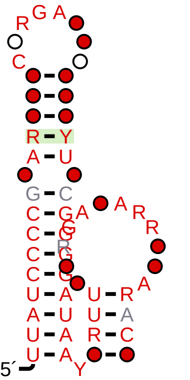

HMP2_2581_1

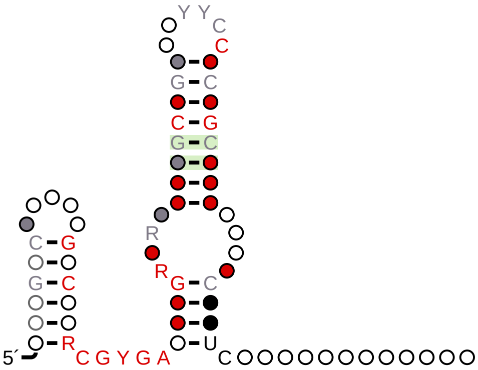

HMP2_260_1

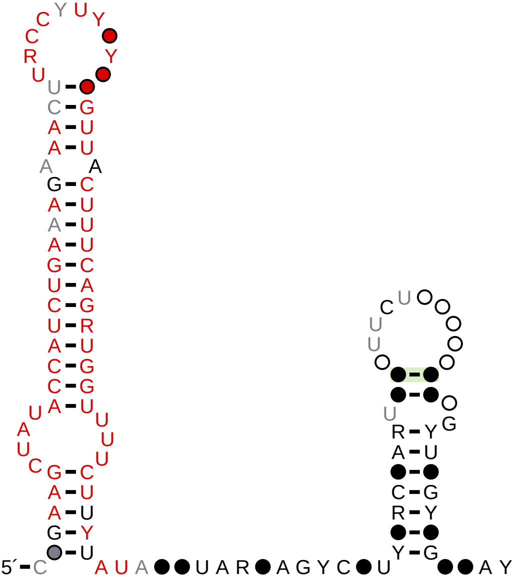

HMP2_2612_1

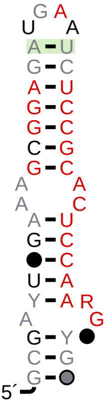

HMP2_2619_1

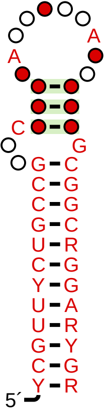

HMP2_2627_1

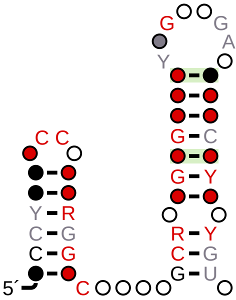

HMP2_2635_1

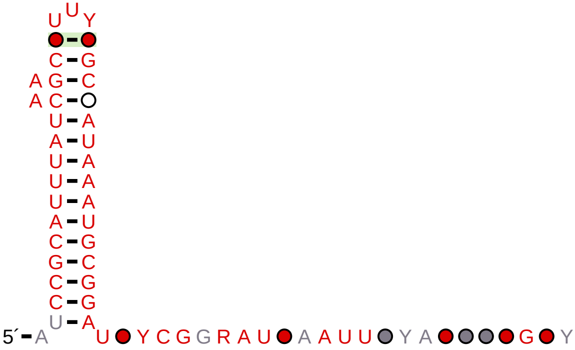
U U Y
C G
A G C
A C
U A
A U
U A
U A
U A
A U
C G
G C
C G
C G
U A
5′ A
U Y C G G R A U A A U U Y A G Y

HMP2_2636_1

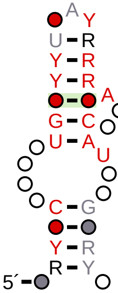

HMP2_2638_1

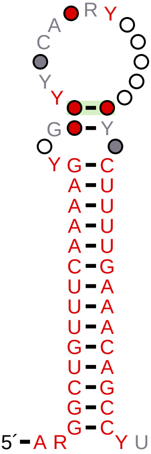

HMP2_2639_1

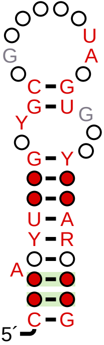

HMP2_2643_1

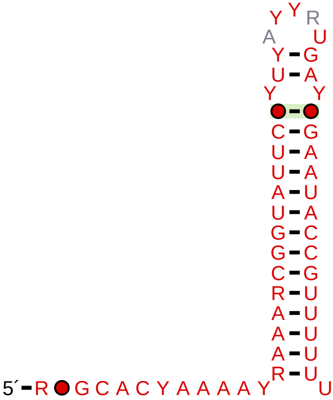

HMP2_266_1

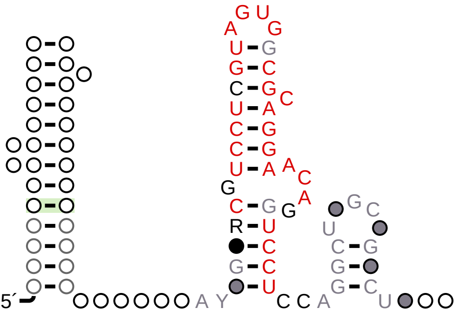

HMP2_2663_1

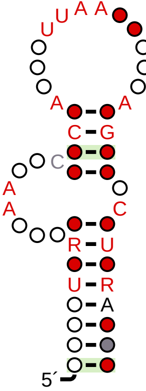

HMP2_2666_1

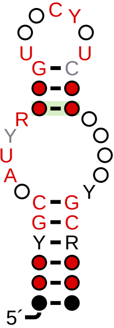

HMP2_2667_1

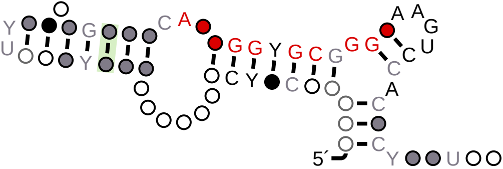

HMP2_2668_1

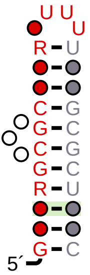

HMP2_2678_1

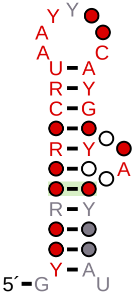

HMP2_2679_1

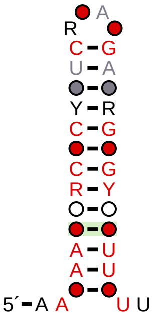

HMP2_2682_1

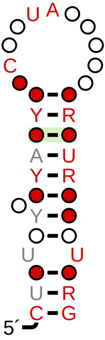

HMP2_2683_1

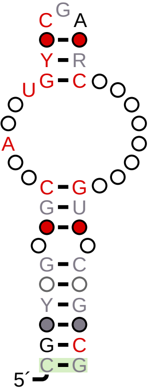

HMP2_2686_1

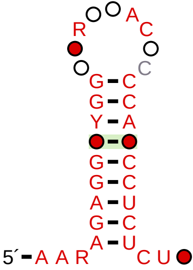

HMP2_2692_1

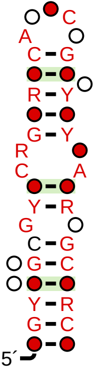

HMP2_2697_1

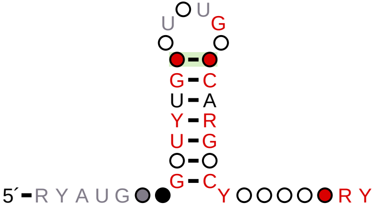

HMP2_2715_1

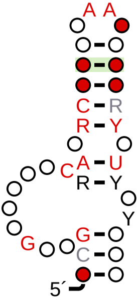

HMP2_2716_1

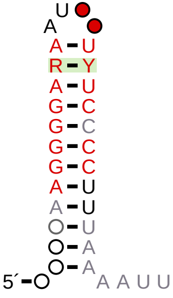

HMP2_2722_1

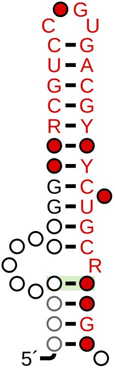

HMP2_2734_1

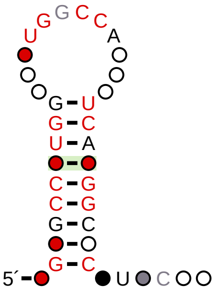

HMP2_2743_1

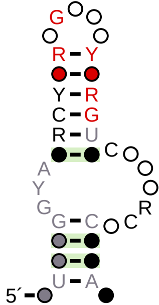

HMP2_2752_1

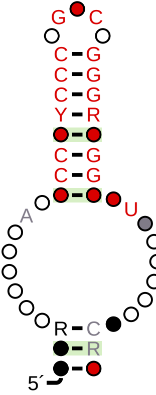

HMP2_2765_1

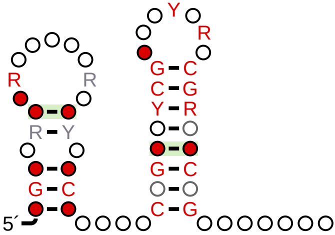

HMP2_2779_1

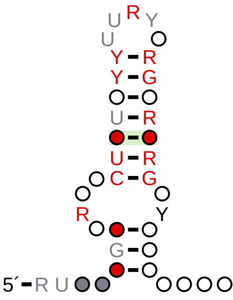

HMP2_2786_1

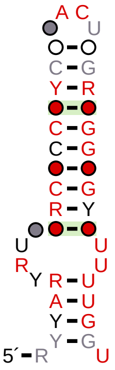

HMP2_2787_1

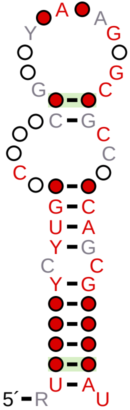

HMP2_2788_1

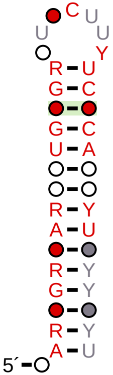

HMP2_2791_1

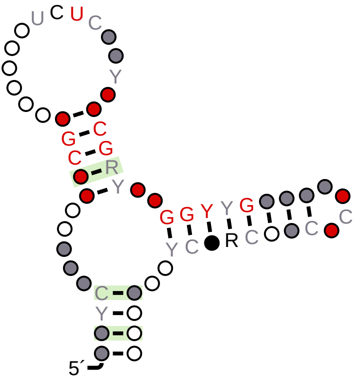

HMP2_280_1

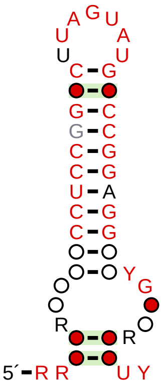

HMP2_2829_1

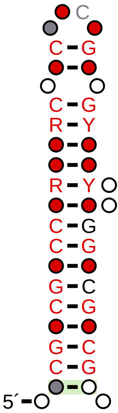

HMP2_2832_1

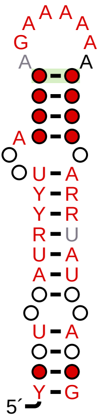

HMP2_2840_1

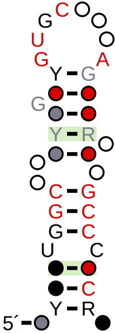

HMP2_2843_1

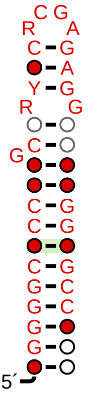

HMP2_2849_1

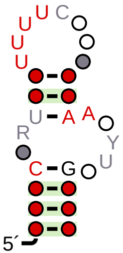

HMP2_2860_1

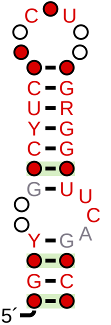

HMP2_2861_1

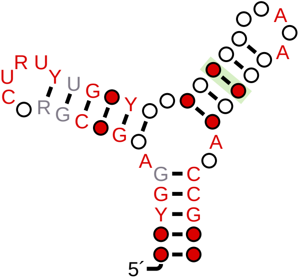

HMP2_2869_1

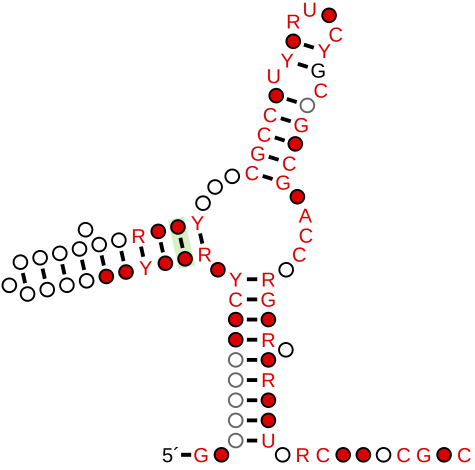

HMP2_2872_1

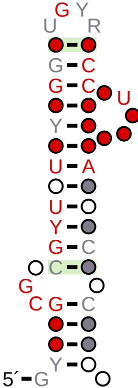

HMP2_2893_1

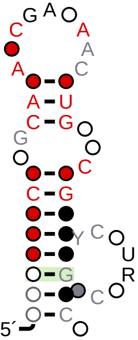

HMP2_2898_1

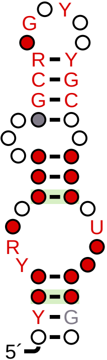

HMP2_2909_1

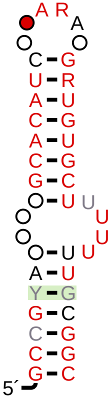

HMP2_2917_1

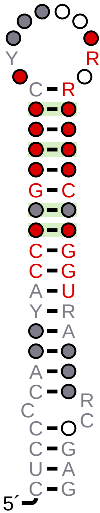

HMP2_2924_1

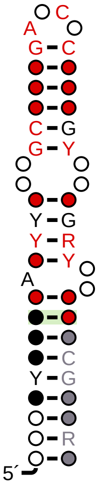

HMP2_2926_1

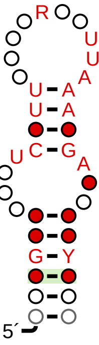

HMP2_2929_1

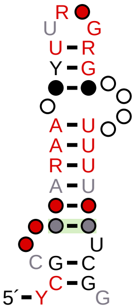

HMP2_2938_1

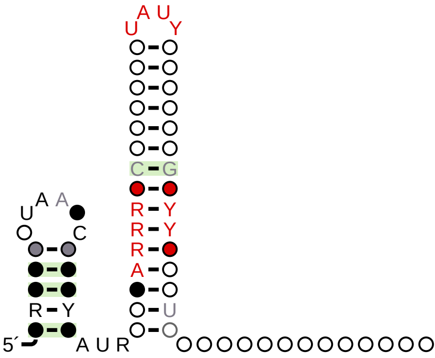

HMP2_2947_1

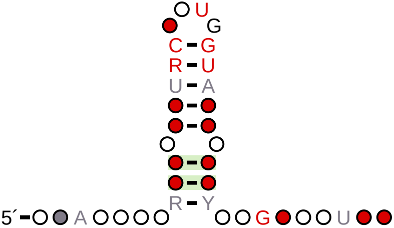

HMP2_2948_1

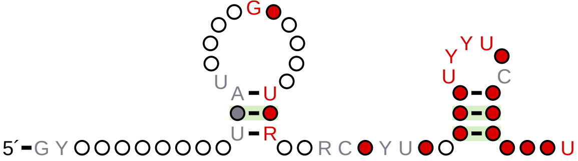

HMP2_2952_1

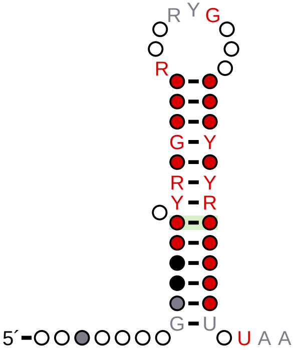

HMP2_2956_1

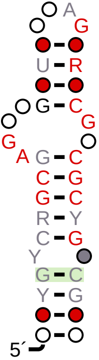

HMP2_2961_1

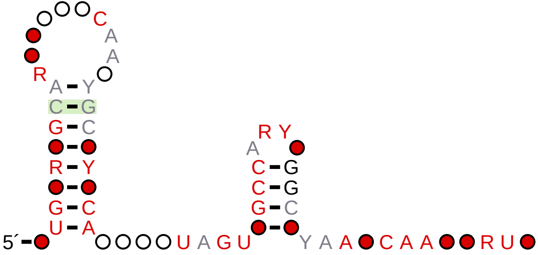

HMP2_2962_1

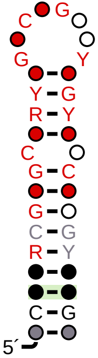

HMP2_2964_1

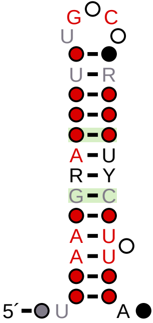

HMP2_2986_1

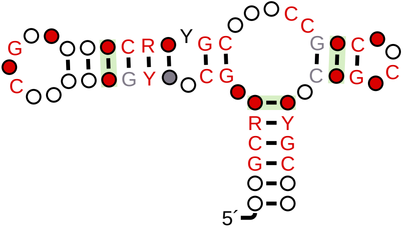

HMP2_2993_1

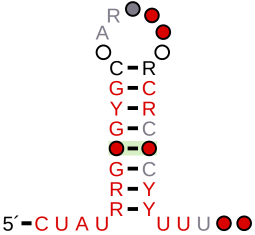

HMP2_30_1

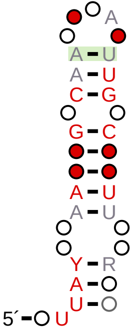

HMP2_3017_1

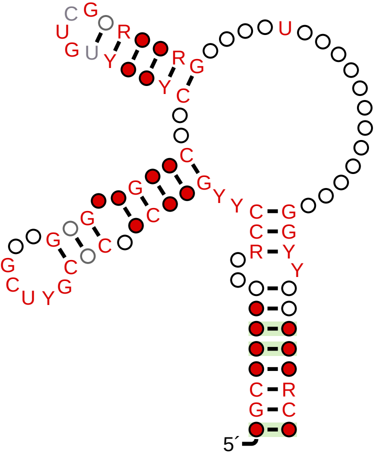

HMP2_302_1

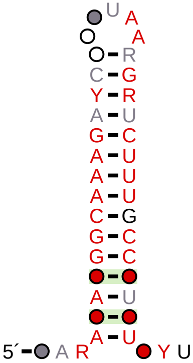

HMP2_3028_1

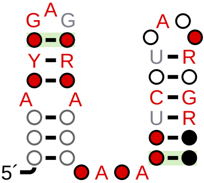

HMP2_3030_1

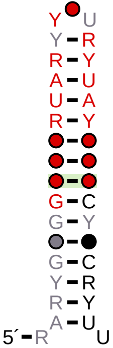

HMP2_3032_1

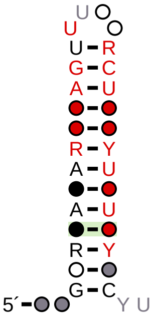

HMP2_3033_1

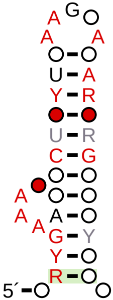

HMP2_3034_1

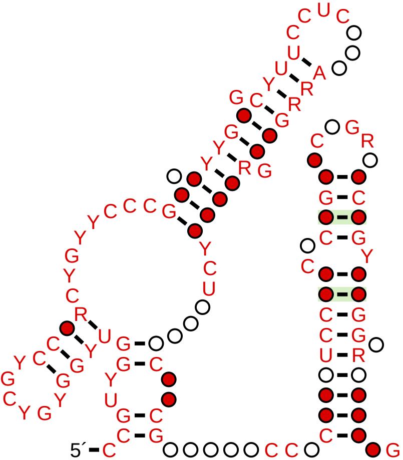

HMP2_3040_1

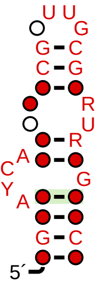

HMP2_3048_1

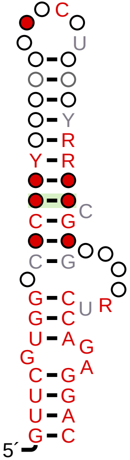

HMP2_3078_1

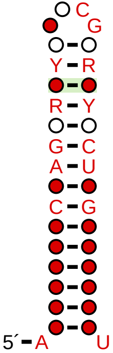

HMP2_3086_1

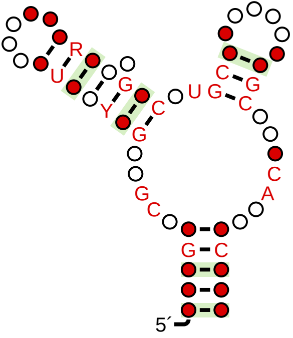

HMP2_3089_1

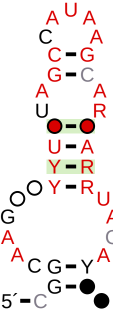

HMP2_309_1

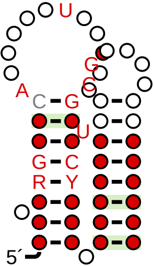

HMP2_3091_1

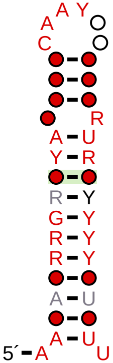

HMP2_3094_1

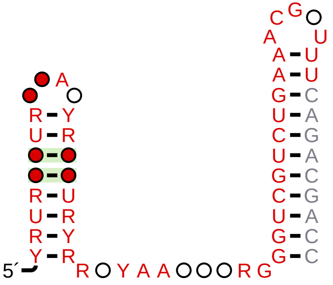

HMP2_3102_1

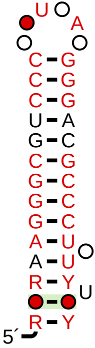

HMP2_3109_1

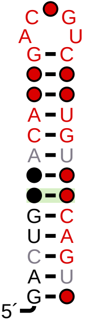

HMP2_3123_1

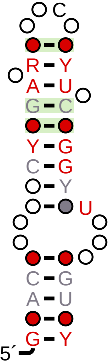

HMP2_3126_1

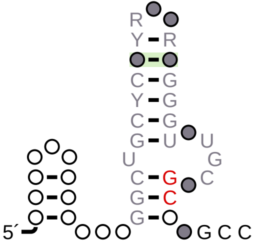

HMP2_3135_1

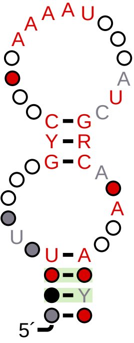

HMP2_3138_1

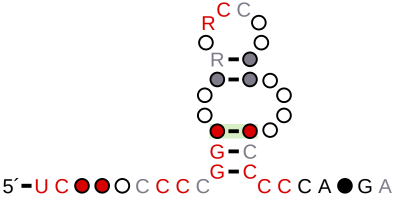

HMP2_3142_1

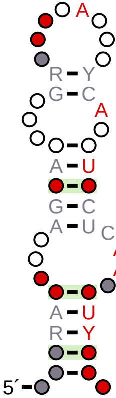

HMP2_3148_1

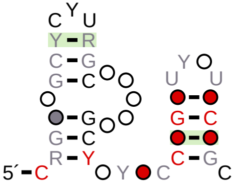

HMP2_3149_1

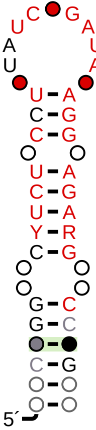

HMP2_3158_1

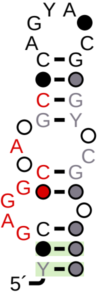

HMP2_3164_1

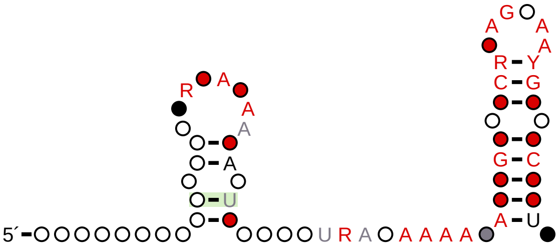

HMP2_3176_1

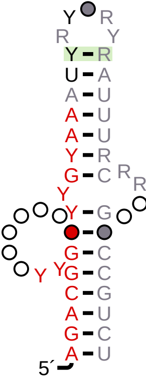

HMP2_3182_1

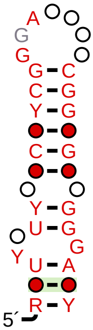

HMP2_3185_1

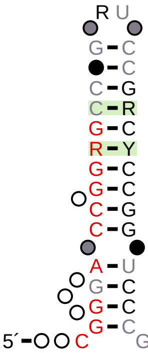

HMP2_3199_1

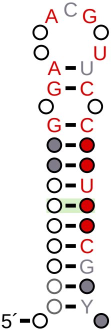

HMP2_3211_1

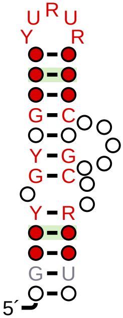

HMP2_3225_1

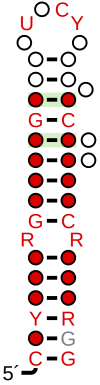

HMP2_3230_1

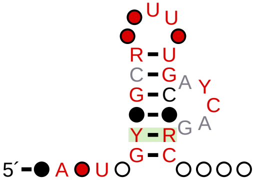

HMP2_3234_1

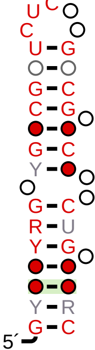

HMP2_3238_1

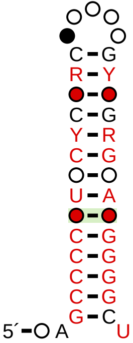

HMP2_3251_1

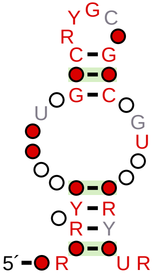

HMP2_3264_1

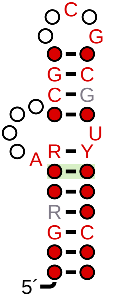

HMP2_3268_1

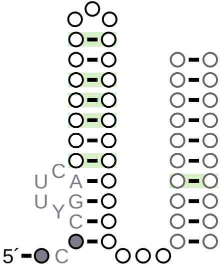

HMP2_3287_1

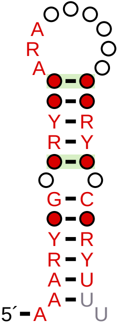

HMP2_3297_1

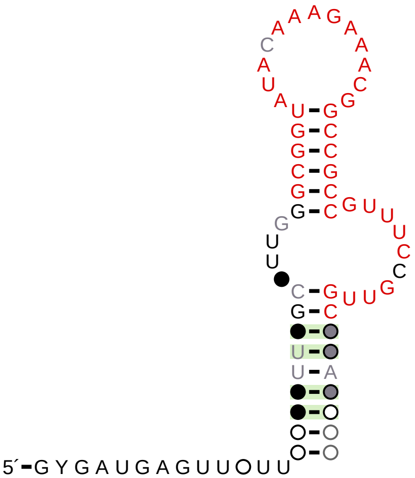

HMP2_3315_1

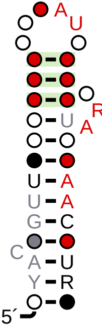

HMP2_3316_1

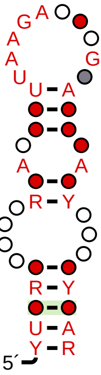

HMP2_3323_1

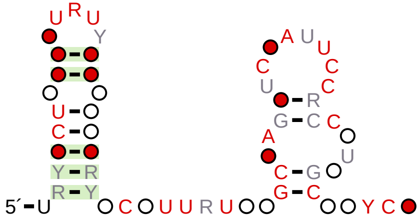

HMP2_3339_1

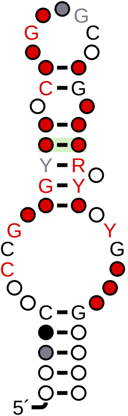

HMP2_3341_1

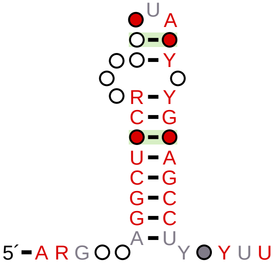

HMP2_3343_1

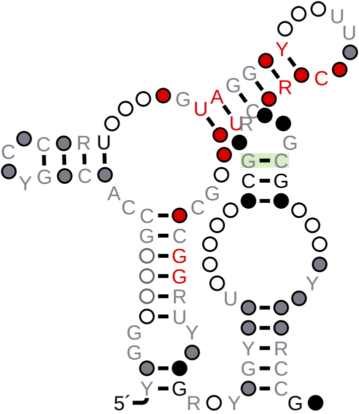

HMP2_3348_1

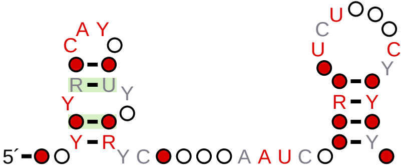

HMP2_3350_1

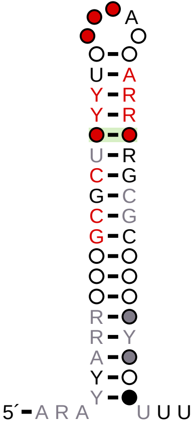

HMP2_3356_1

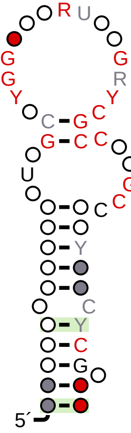

HMP2_336_1

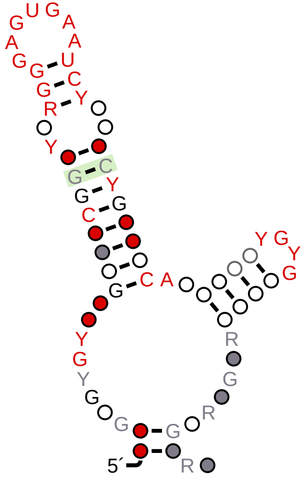

HMP2_3360_1

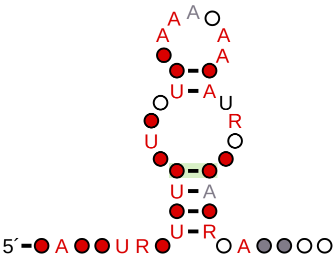

HMP2_3367_1

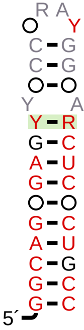

HMP2_3369_1

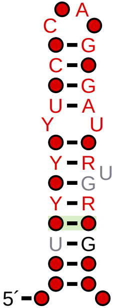

HMP2_3376_1

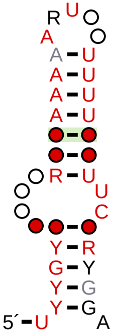

HMP2_3378_1

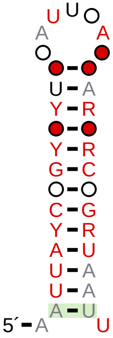

HMP2_3391_1

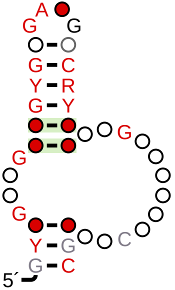

HMP2_3398_1

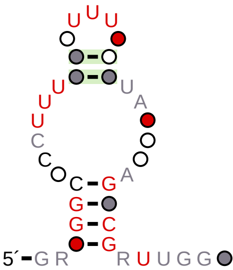

HMP2_3405_1

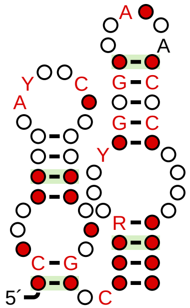

HMP2_3410_1

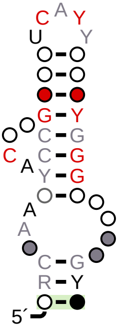

HMP2_3423_1

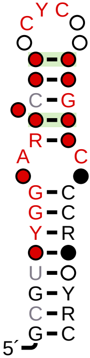

HMP2_3433_1

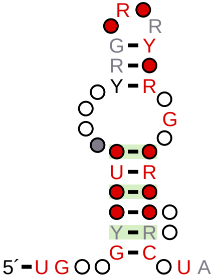

HMP2_346_1

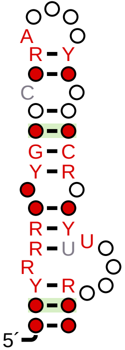

HMP2_3463_1

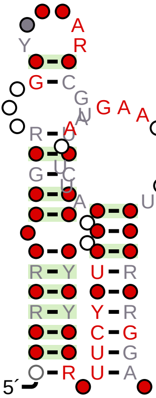

HMP2_3464_1

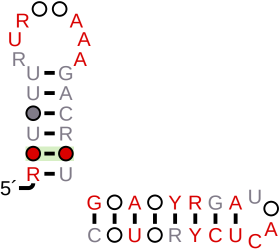

HMP2_3465_1

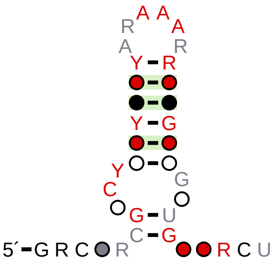

HMP2_3476_1

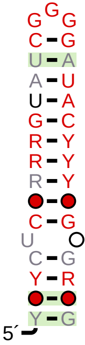

HMP2_3489_1

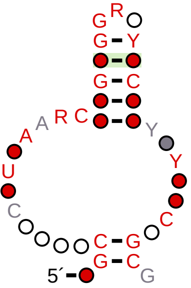

HMP2_3490_1

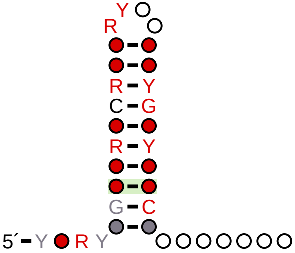

HMP2_3501_1

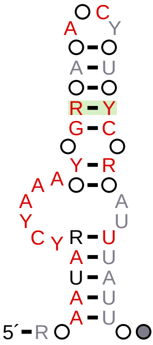

HMP2_3538_1

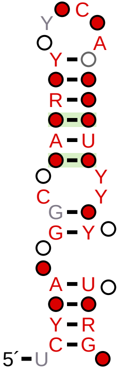

HMP2_3544_1

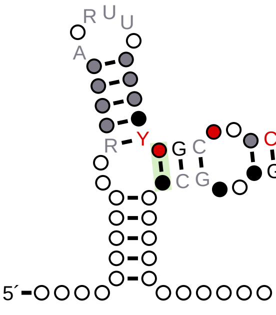

HMP2_3547_1

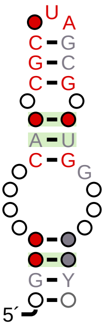

HMP2_3585_1

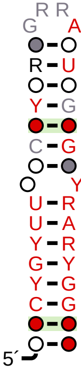

HMP2_3593_1

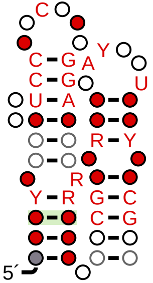

HMP2_3596_1

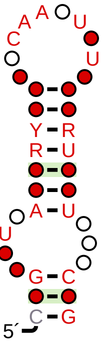

HMP2_3602_1

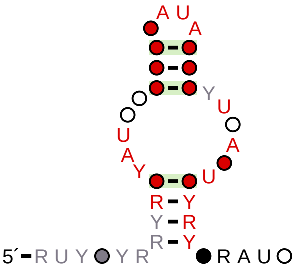

HMP2_3604_1

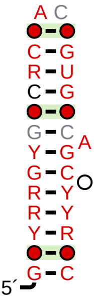

HMP2_3606_1

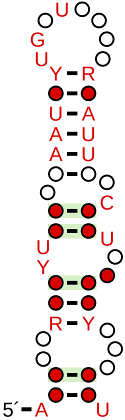

HMP2_3613_1

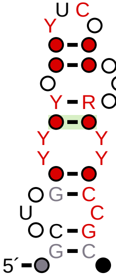

HMP2_3631_1

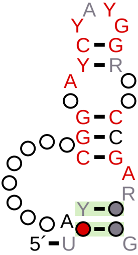

HMP2_3650_1

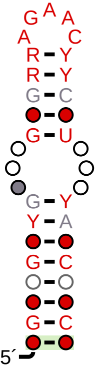

HMP2_3656_1

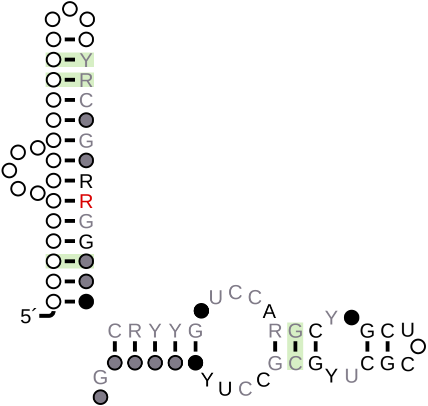

HMP2_3661_1

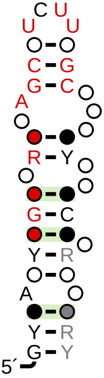

HMP2_3670_1

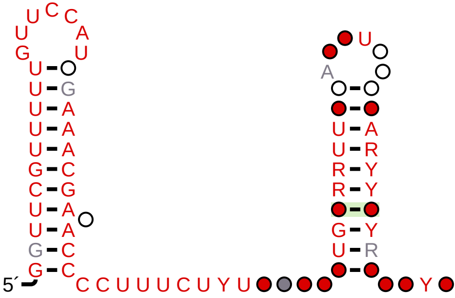

HMP2_3671_1

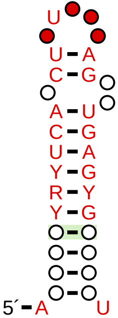

HMP2_3676_1

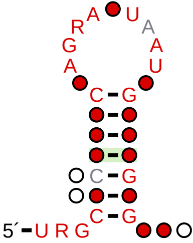

HMP2_3677_1

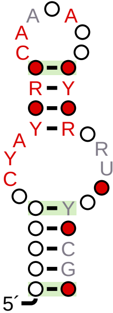

HMP2_3683_1

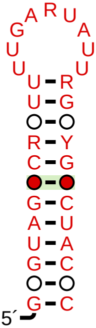

HMP2_3685_1

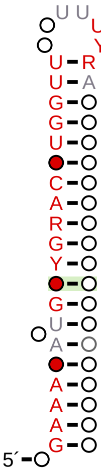

HMP2_3696_1

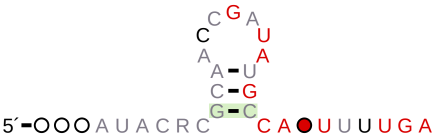

HMP2_3702_1

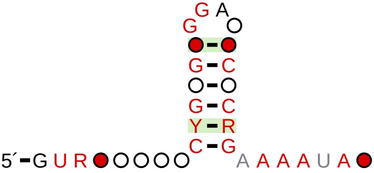

HMP2_3739_1

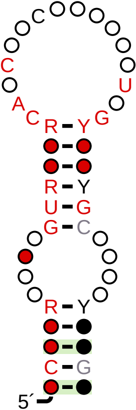

HMP2_374_1

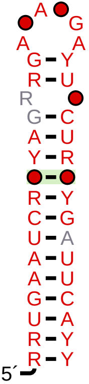

HMP2_3741_1

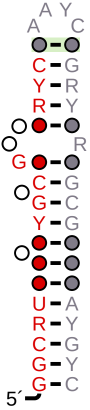

HMP2_3744_1

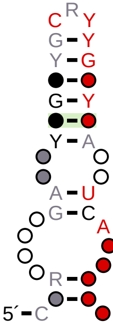

HMP2_3758_1

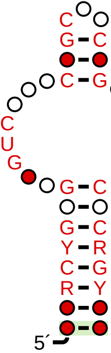

HMP2_3759_1

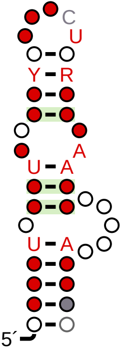

HMP2_3771_1

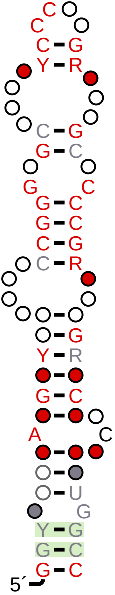

HMP2_3790_1

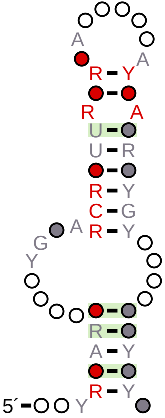

HMP2_3797_1

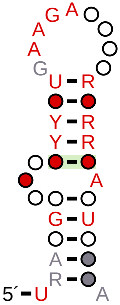

HMP2_3799_1

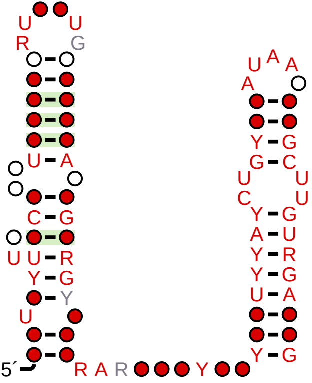

HMP2_3802_1

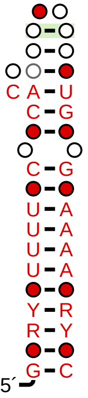

HMP2_3803_1

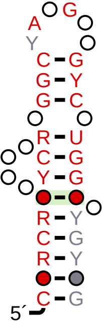

HMP2_3813_1

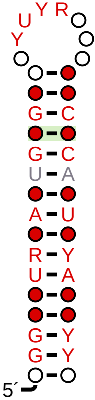

HMP2_3817_1

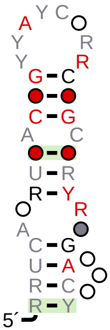

HMP2_3818_1

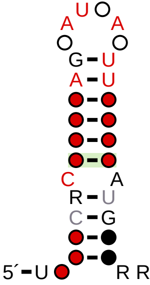

HMP2_3824_1

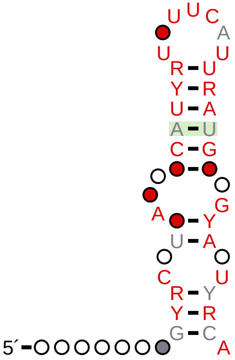

HMP2_3827_1

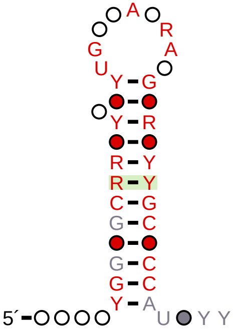

HMP2_3828_1

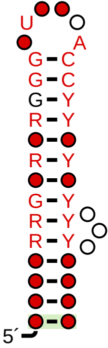

HMP2_3833_1

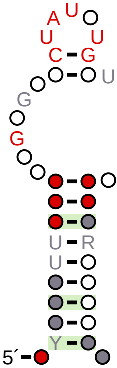

HMP2_3835_1

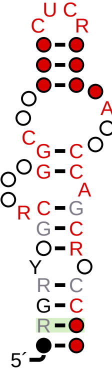

HMP2_3855_1

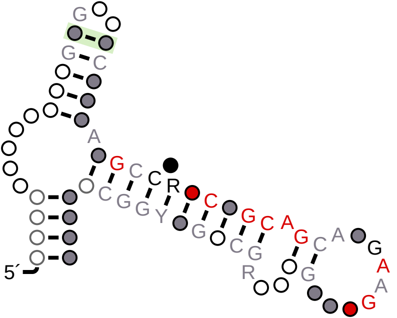

HMP2_3860_1

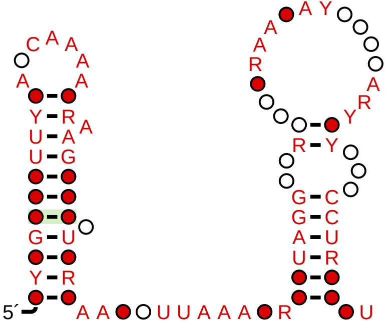

HMP2_3861_1

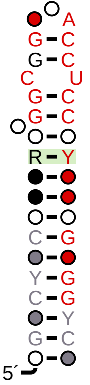

HMP2_3864_1

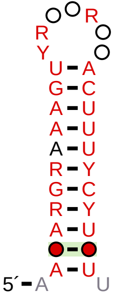

HMP2_3873_1

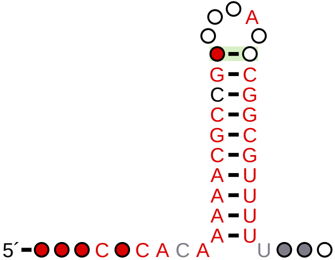

HMP2_3875_1

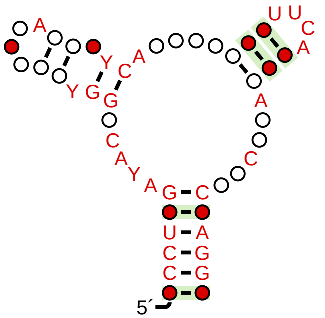

HMP2_3884_1

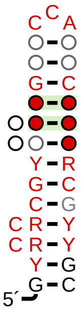

HMP2_389_1

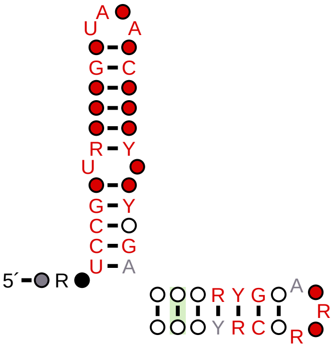

HMP2_3891_1

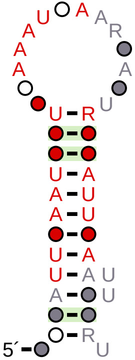

HMP2_3905_1

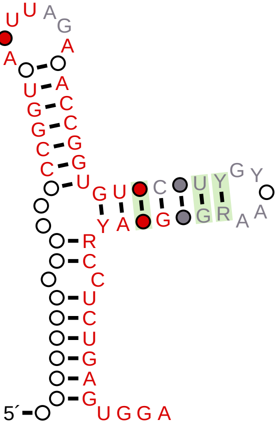

HMP2_3912_1

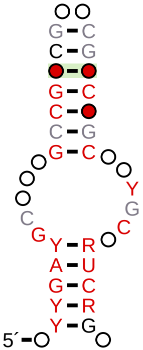

HMP2_3923_1

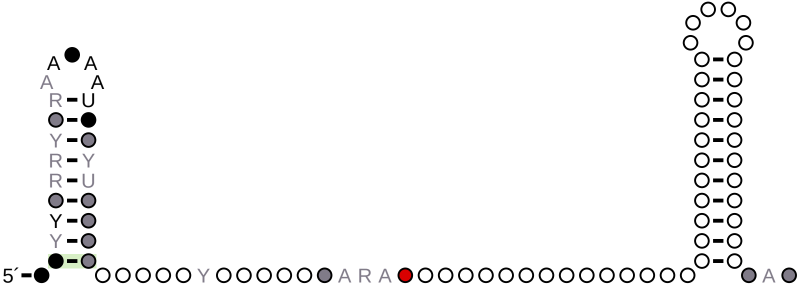

HMP2_3942_1

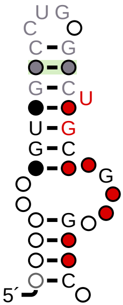

HMP2_3965_1

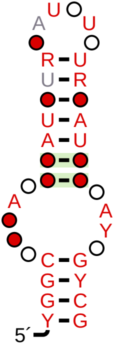

HMP2_3968_1

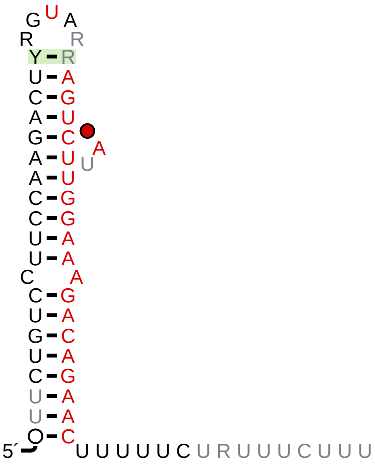

HMP2_3975_1

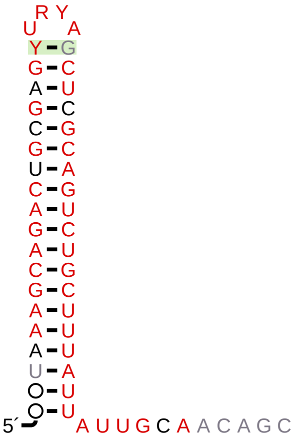

HMP2_3980_1

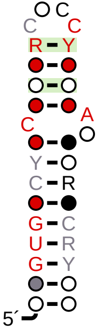

HMP2_3982_1

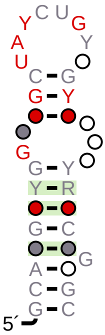

HMP2_3988_1

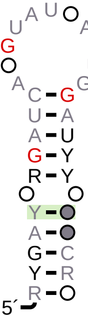

HMP2_3989_1

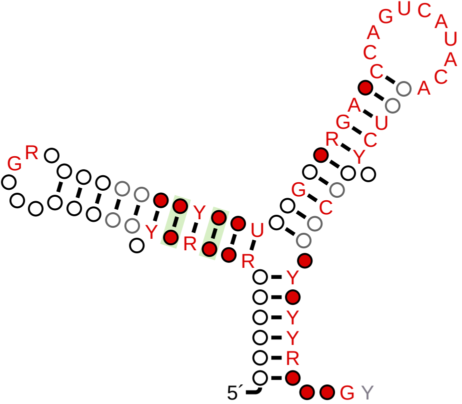

HMP2_399_1

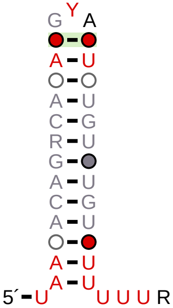

HMP2_3997_1

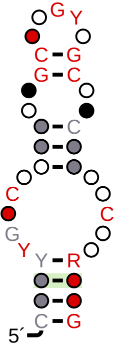

HMP2_4004_1

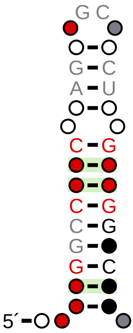

HMP2_4017_1

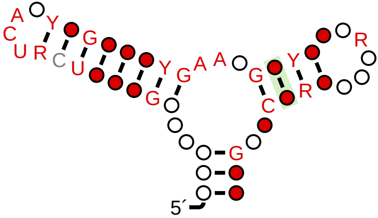

HMP2_4018_1

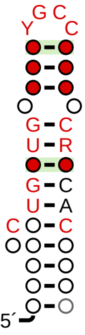

HMP2_402_1

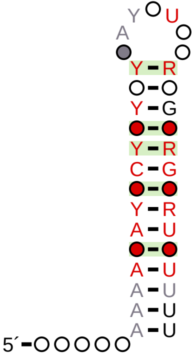

HMP2_4025_1

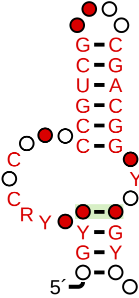

HMP2_4049_1

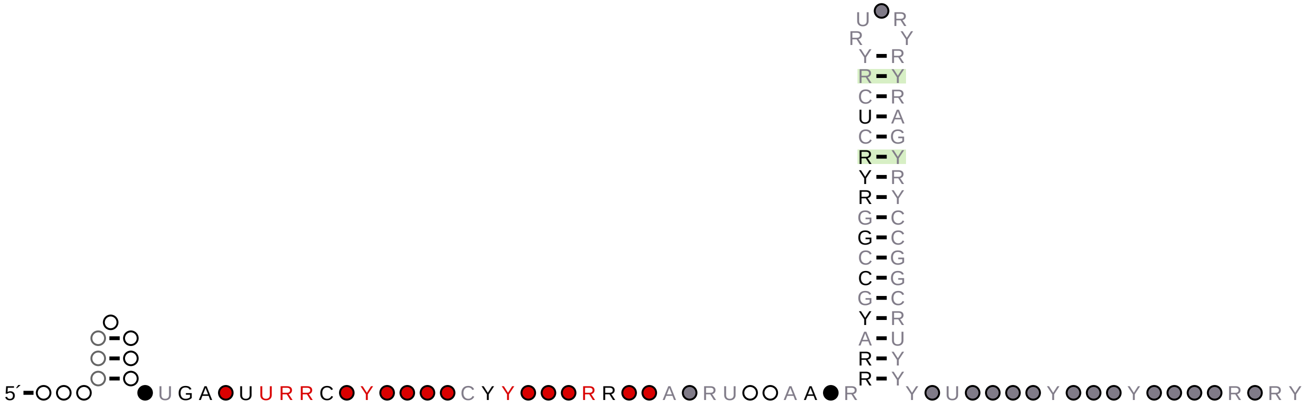

HMP2_4059_1

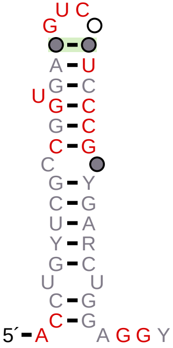

HMP2_406_1

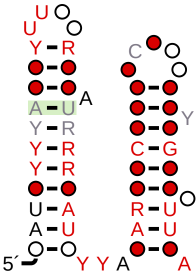

HMP2_4075_1

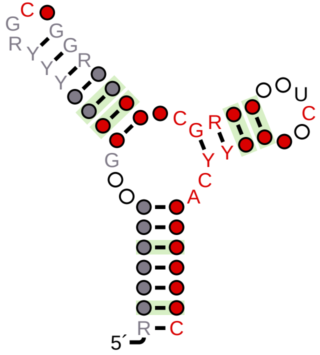

HMP2_409_1

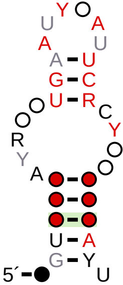

HMP2_4096_1

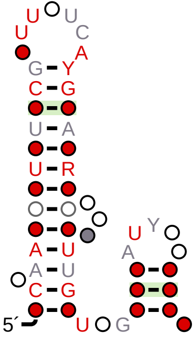

HMP2_4102_1

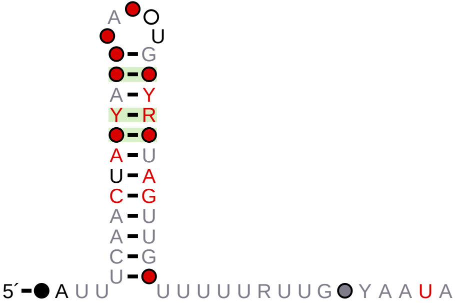

HMP2_4103_1

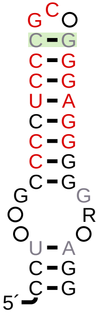

HMP2_4111_1

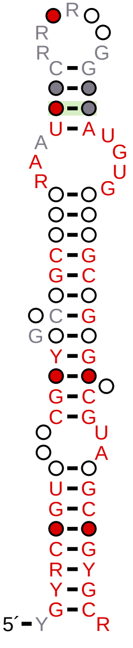

HMP2_4117_1

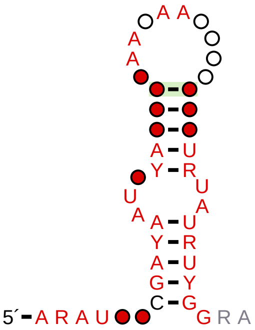

HMP2_4119_1

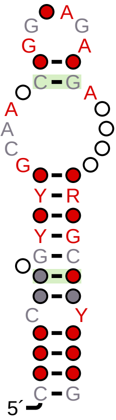

HMP2_4131_1

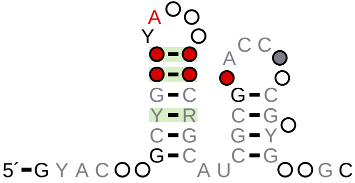

HMP2_414_1

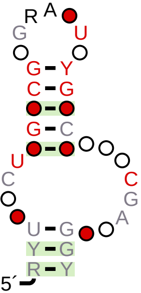

HMP2_4140_1

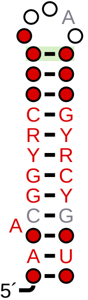

HMP2_4142_1

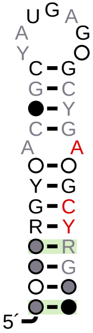

HMP2_4147_1

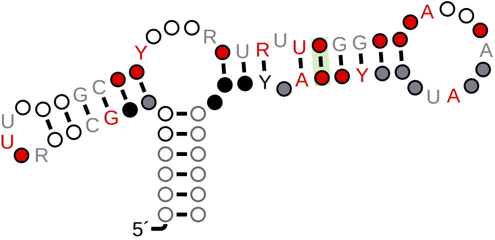

HMP2_415_1

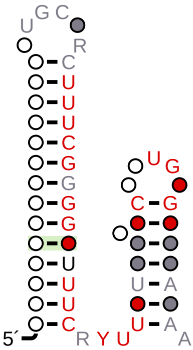

HMP2_4153_1

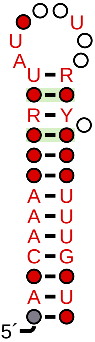

HMP2_4157_1

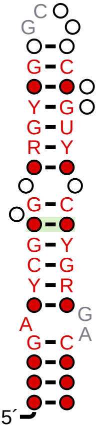

HMP2_4162_1

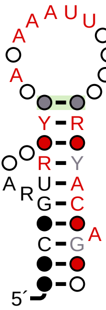

HMP2_4164_1

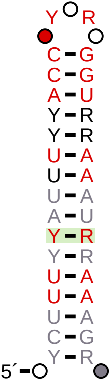

HMP2_4166_1

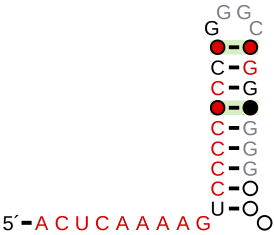

HMP2_4174_1

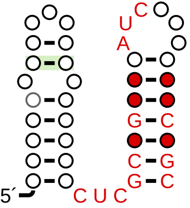

HMP2_4187_1

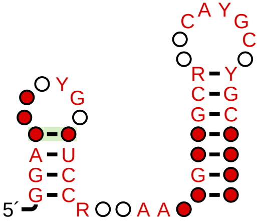

HMP2_4208_1

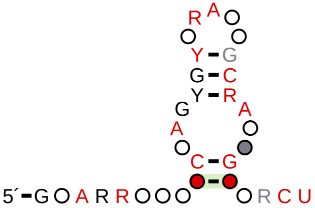

HMP2_4215_1

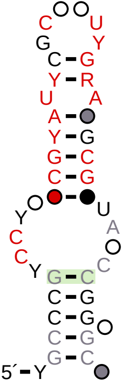

HMP2_4224_1

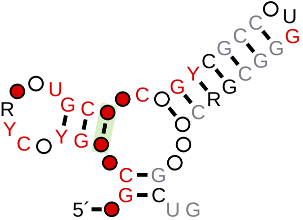

HMP2_4238_1

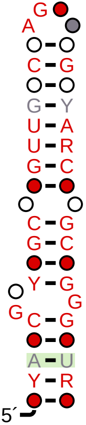

HMP2_4260_1

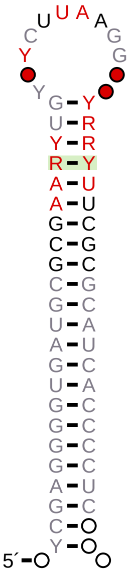

HMP2_4263_1

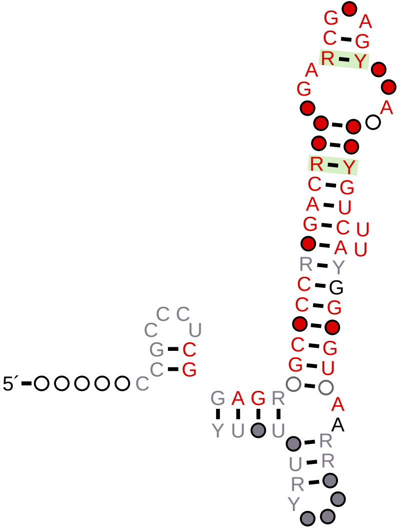

HMP2_4285_1

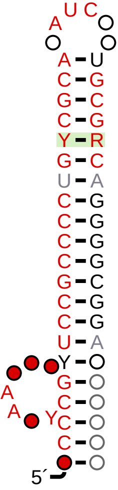

HMP2_430_1

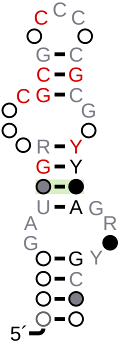

HMP2_4300_1

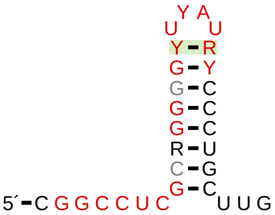

HMP2_4326_1

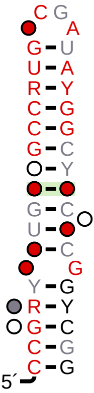

HMP2_4328_1

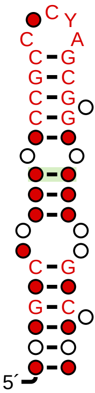

HMP2_4332_1

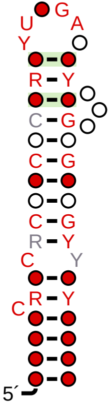

HMP2_4336_1

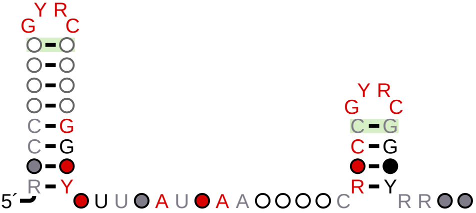

HMP2_4337_1

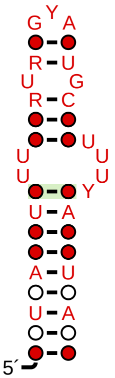

HMP2_4341_1

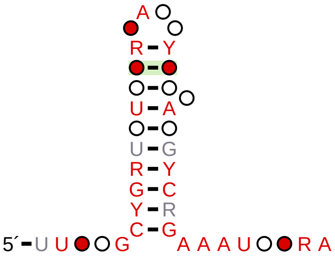

HMP2_4347_1

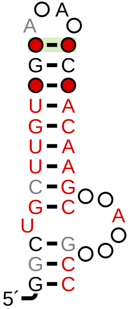

HMP2_4351_1

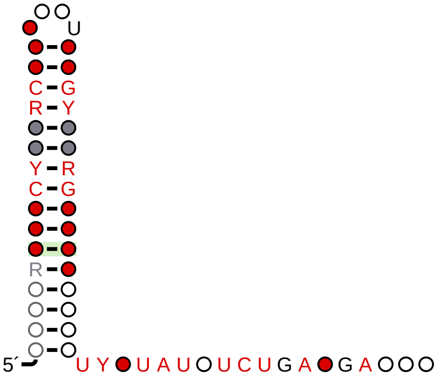

HMP2_4354_1

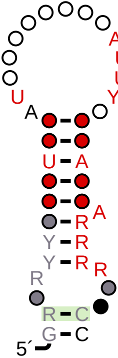

HMP2_4357_1

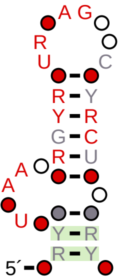

HMP2_4361_1

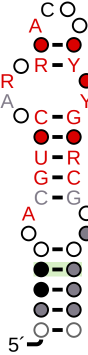

HMP2_4363_1

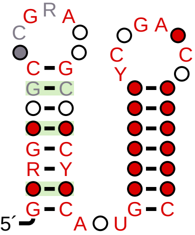

HMP2_4372_1

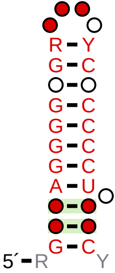

HMP2_4383_1

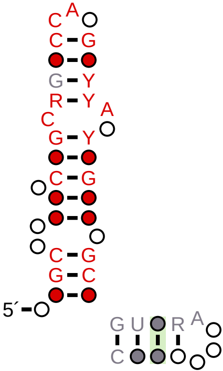

HMP2_4384_1

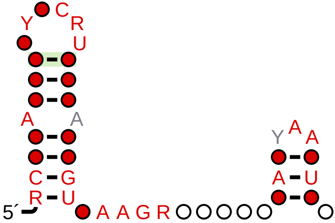

HMP2_4387_1

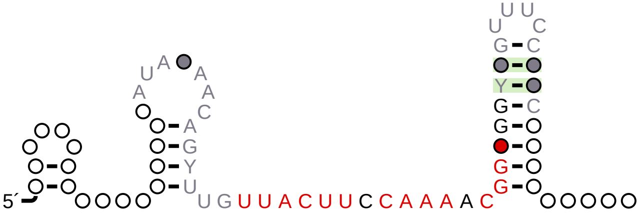

HMP2_4396_1

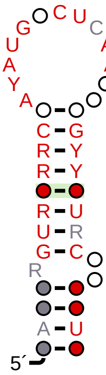

HMP2_4401_1

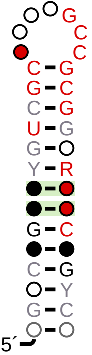

HMP2_4404_1

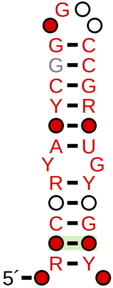

HMP2_4408_1

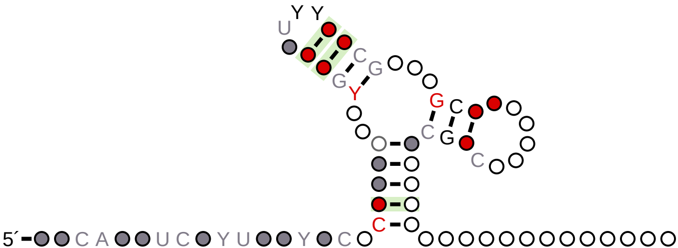

HMP2_4421_1

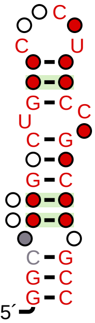

HMP2_4423_1

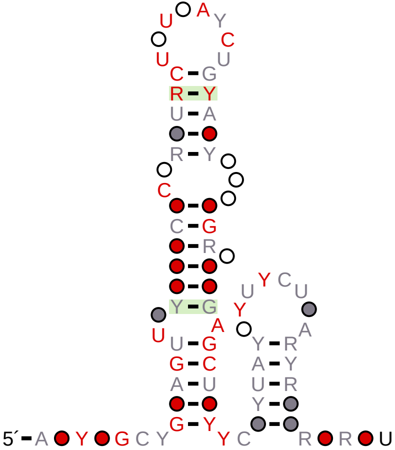

HMP2_4424_1

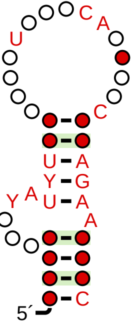

HMP2_4426_1

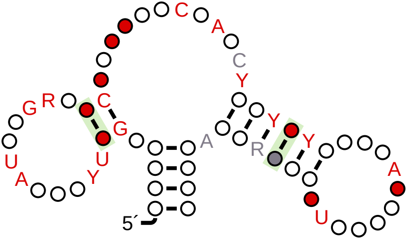

HMP2_4444_1

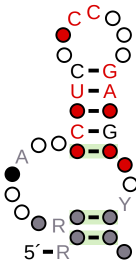

HMP2_4449_1

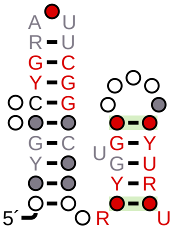

HMP2_4452_1

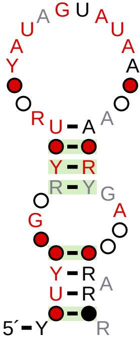

HMP2_4462_1

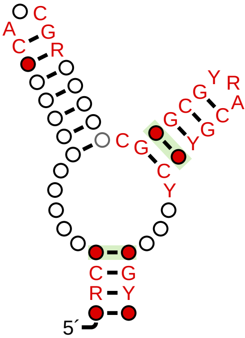

HMP2_4463_1

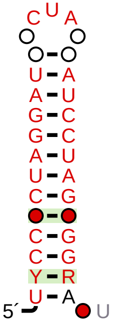

HMP2_4464_1

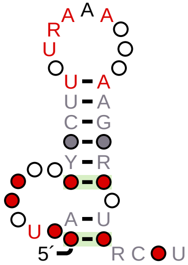

HMP2_447_1

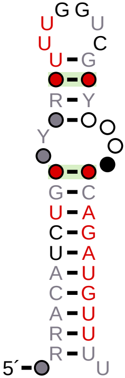

HMP2_4474_1

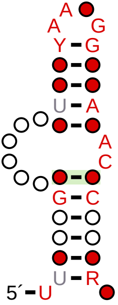

HMP2_4475_1

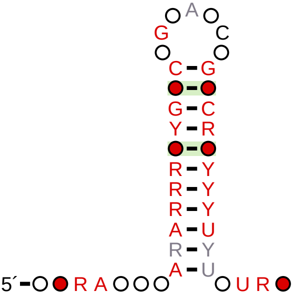

HMP2_4484_1

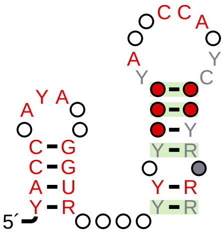

HMP2_4503_1

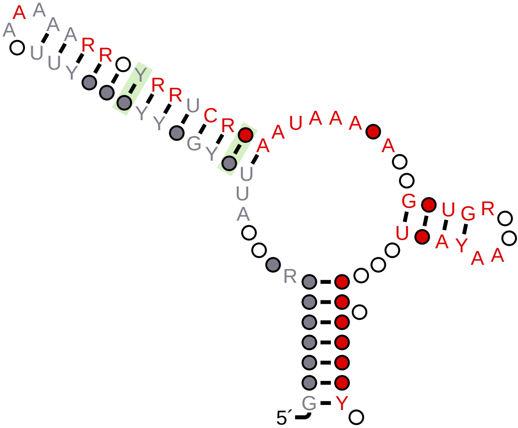

HMP2_4508_1

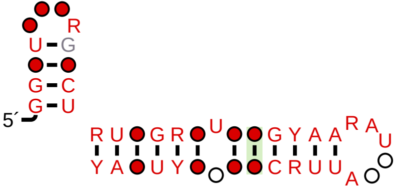

HMP2_4515_1

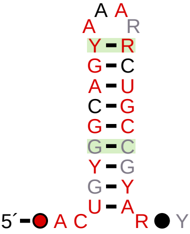

HMP2_4516_1

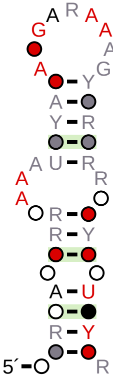

HMP2_4525_1

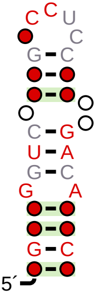

HMP2_4537_1

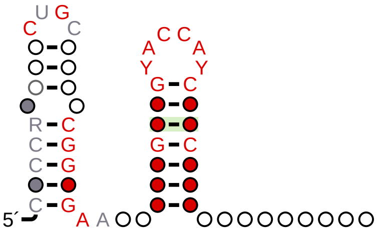

HMP2_4539_1

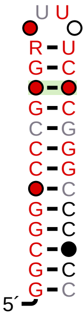

HMP2_4553_1

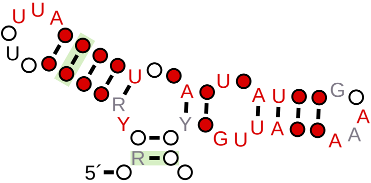

HMP2_4554_1

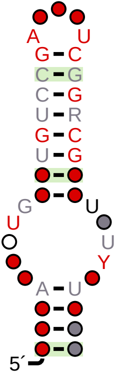

HMP2_4558_1

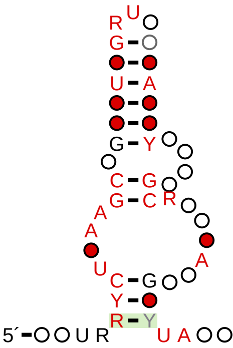

HMP2_4571_1

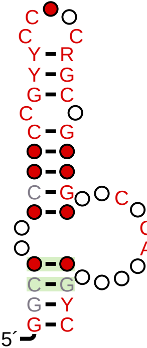

HMP2_4574_1

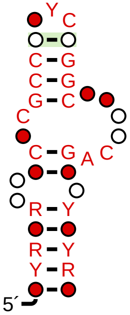

HMP2_4580_1

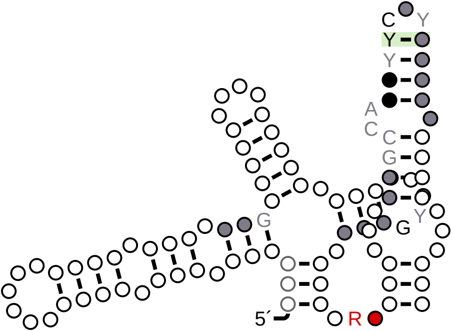

HMP2_4590_1

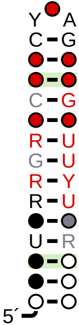

HMP2_4592_1

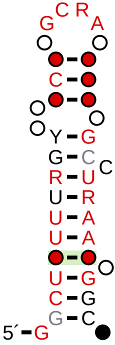

HMP2_4595_1

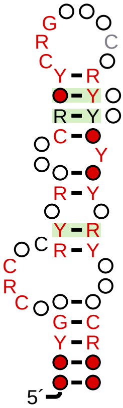

HMP2_4598_1

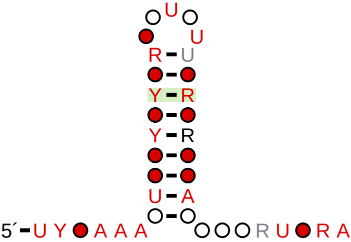

HMP2_4603_1

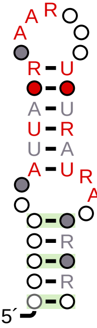

HMP2_4605_1

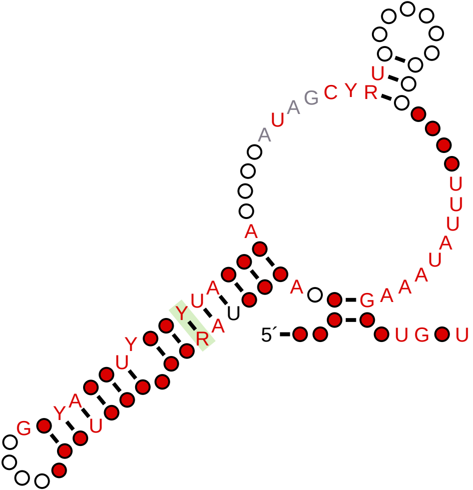

HMP2_4609_1

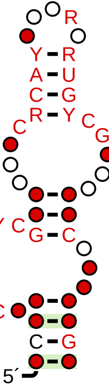

HMP2_4623_1

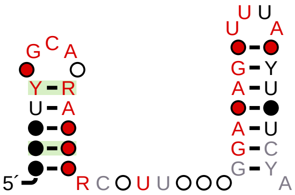

HMP2_4628_1

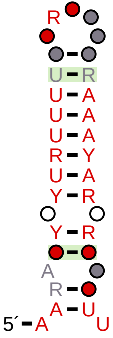

HMP2_4633_1

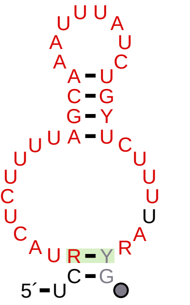

HMP2_4634_1

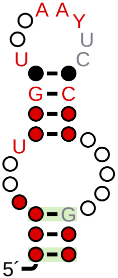

HMP2_4637_1

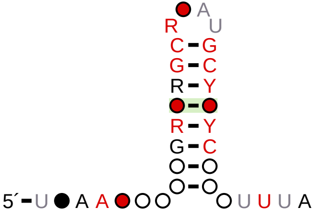

HMP2_4639_1

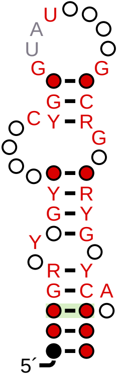

HMP2_466_1

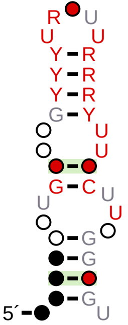

HMP2_4667_1

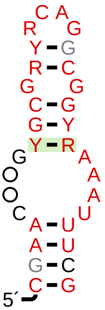

HMP2_4684_1

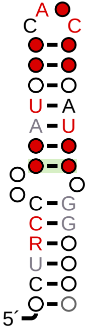

HMP2_4688_1

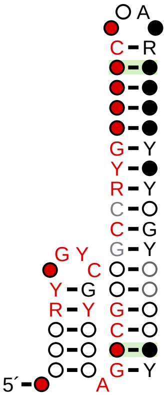

HMP2_4689_1

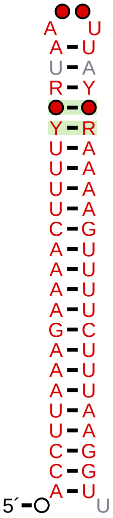

HMP2_4699_1

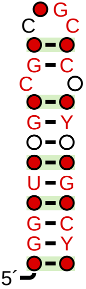

HMP2_4702_1

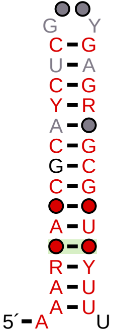

HMP2_4705_1

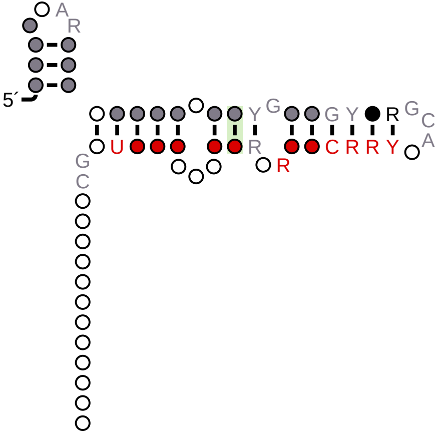

HMP2_4706_1

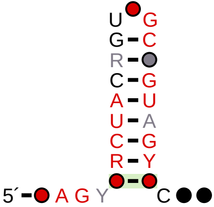

HMP2_4713_1

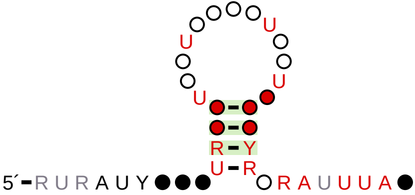

HMP2_4719_1

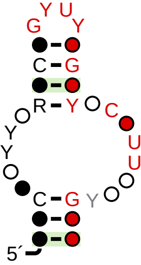

HMP2_4722_1

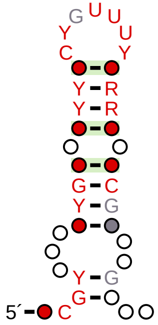

HMP2_4734_1

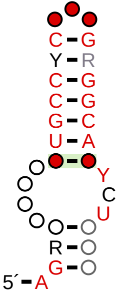

HMP2_4735_1

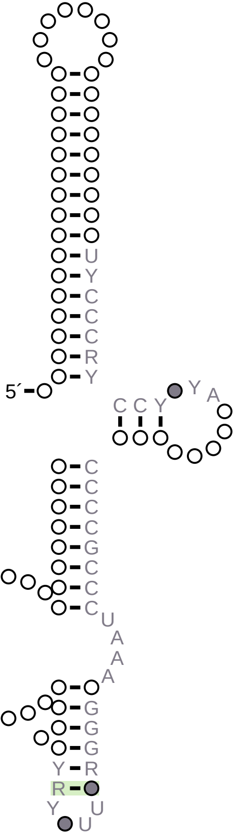

HMP2_4741_1

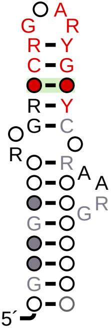

HMP2_4757_1

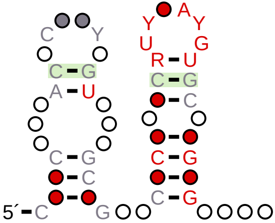

HMP2_4764_1

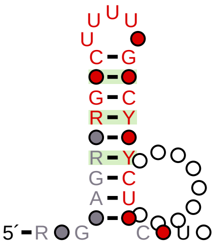

HMP2_4766_1

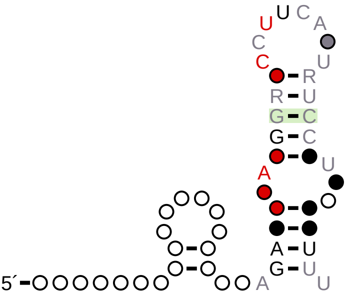

HMP2_4772_1

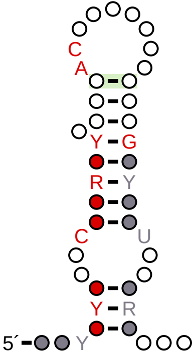

HMP2_4783_1

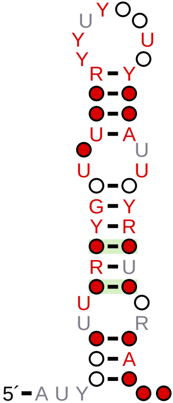

HMP2_4801_1

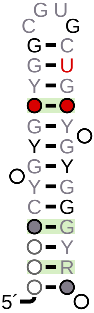

HMP2_4808_1

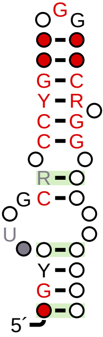

HMP2_481_1

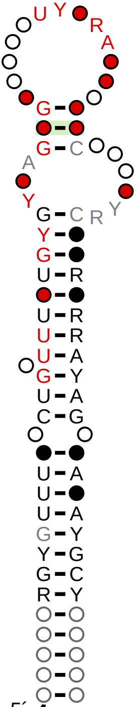

HMP2_4811_1

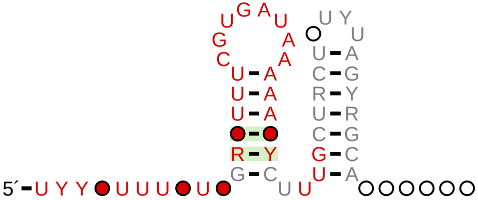

HMP2_4812_1

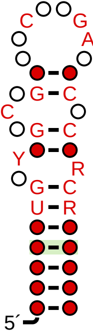

HMP2_4818_1

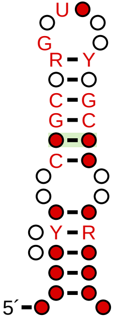

HMP2_4819_1

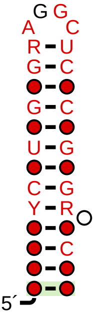

HMP2_4828_1

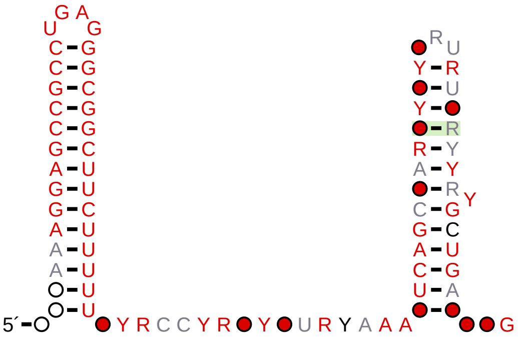

HMP2_4829_1

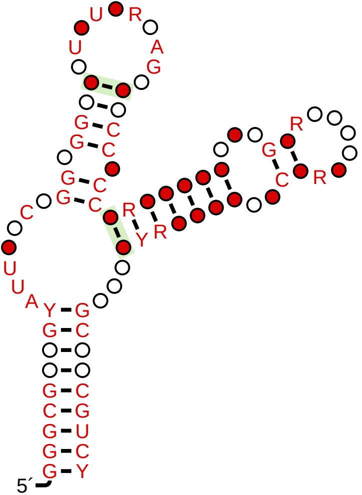

HMP2_4836_1

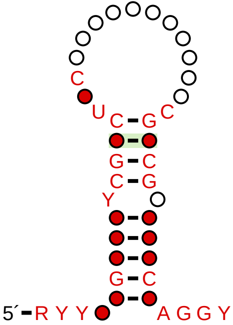

HMP2_4839_1

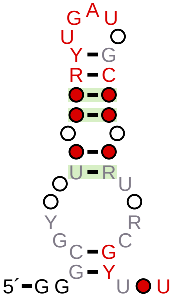

HMP2_4851_1

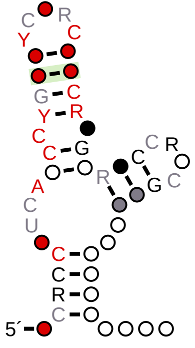

HMP2_4860_1

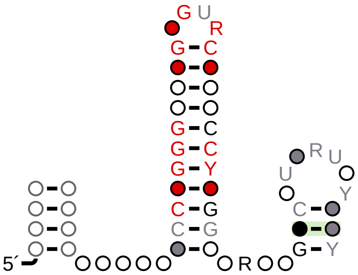

HMP2_4861_1

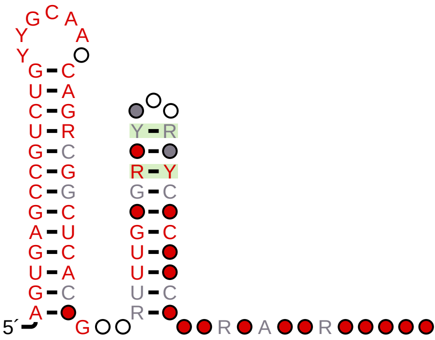

HMP2_4871_1

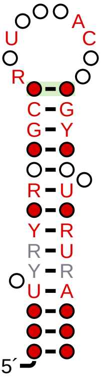

HMP2_4884_1

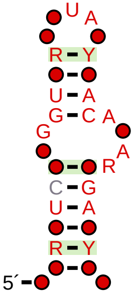

HMP2_4892_1

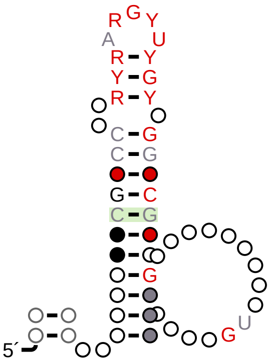

HMP2_4893_1

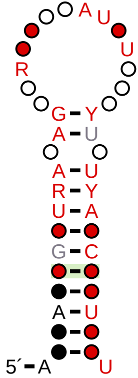

HMP2_4904_1

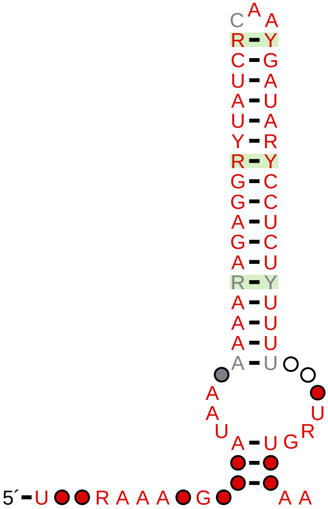

HMP2_4924_1

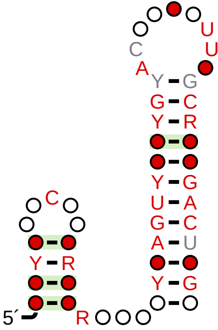

HMP2_4933_1

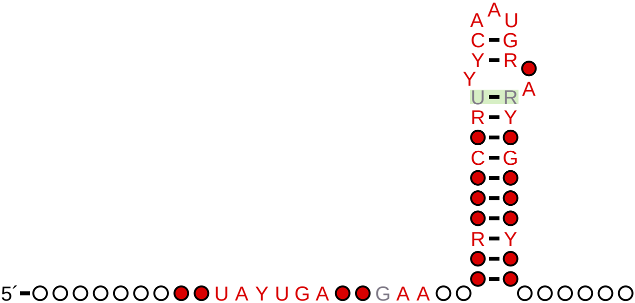

HMP2_494_1

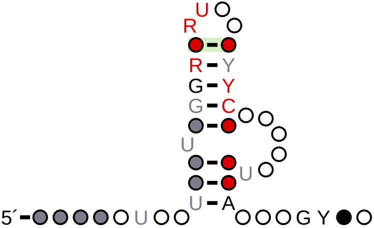

HMP2_4958_1

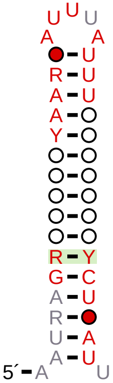

HMP2_4960_1

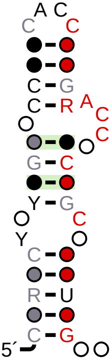

HMP2_4965_1

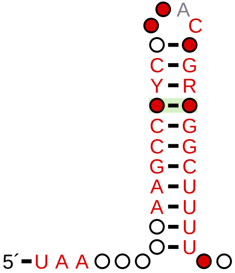

HMP2_4971_1

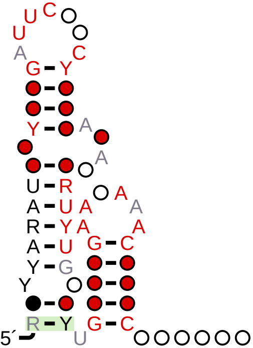

HMP2_4996_1

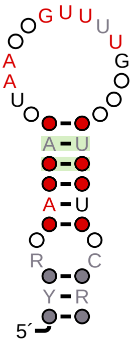

HMP2_5_1

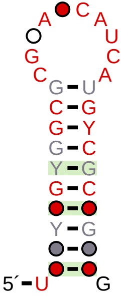

HMP2_5007_1

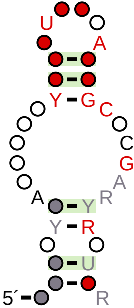

HMP2_5011_1

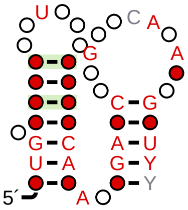

HMP2_5012_1

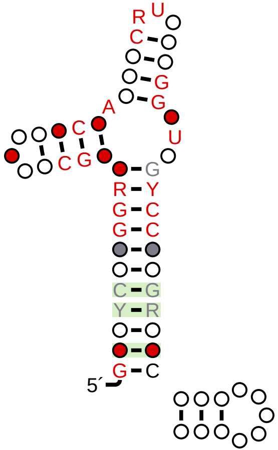

HMP2_5021_1

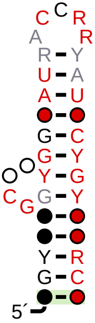

HMP2_5055_1

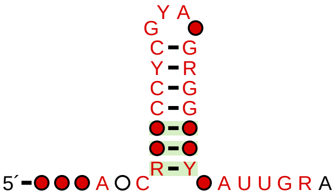

HMP2_5059_1

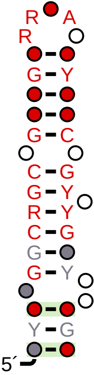

HMP2_5063_1

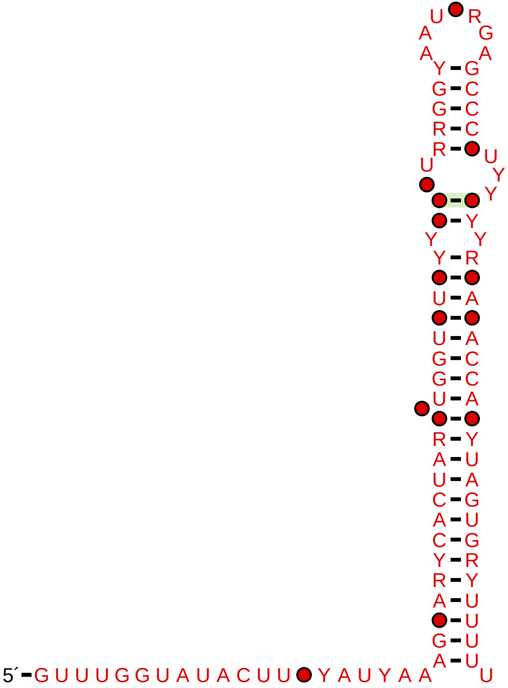

HMP2_5101_1

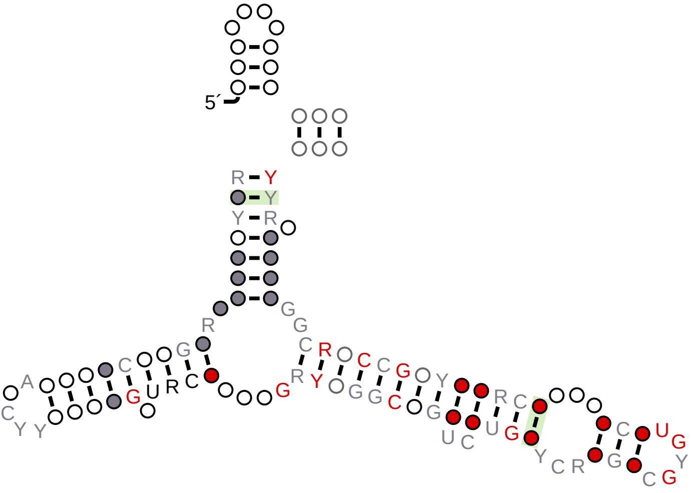

HMP2_5108_1

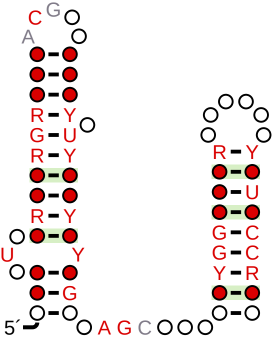

HMP2_512_1

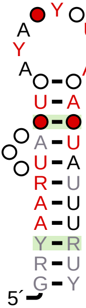

HMP2_5122_1

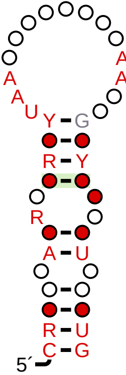

HMP2_5123_1

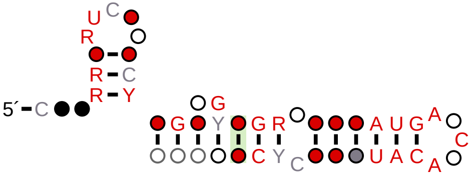

HMP2_5134_1

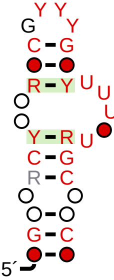

HMP2_5136_1

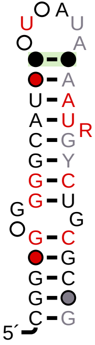

HMP2_5142_1

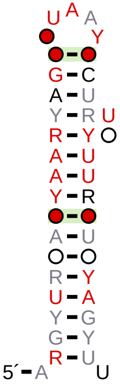

HMP2_5144_1

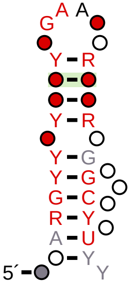

HMP2_5148_1

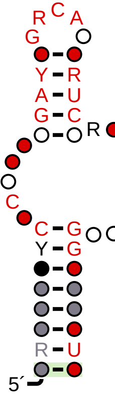

HMP2_5162_1

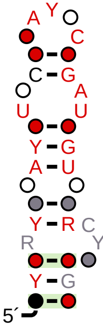

HMP2_5166_1

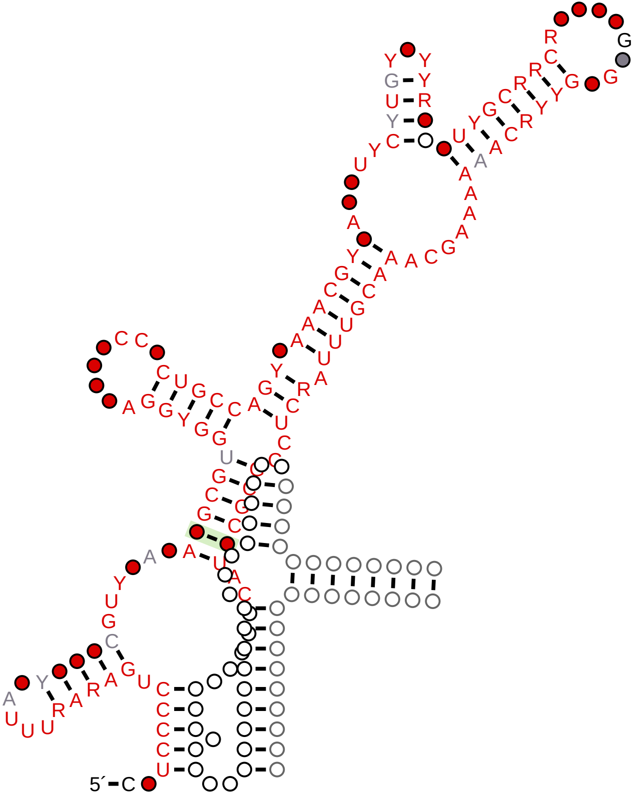

HMP2_5173_1

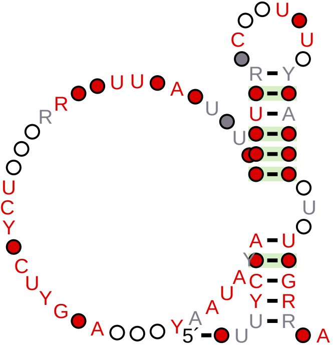

HMP2_5181_1

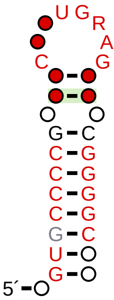

HMP2_5184_1

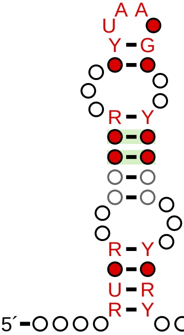

HMP2_5185_1

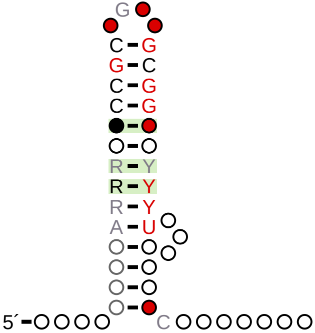

HMP2_5191_1

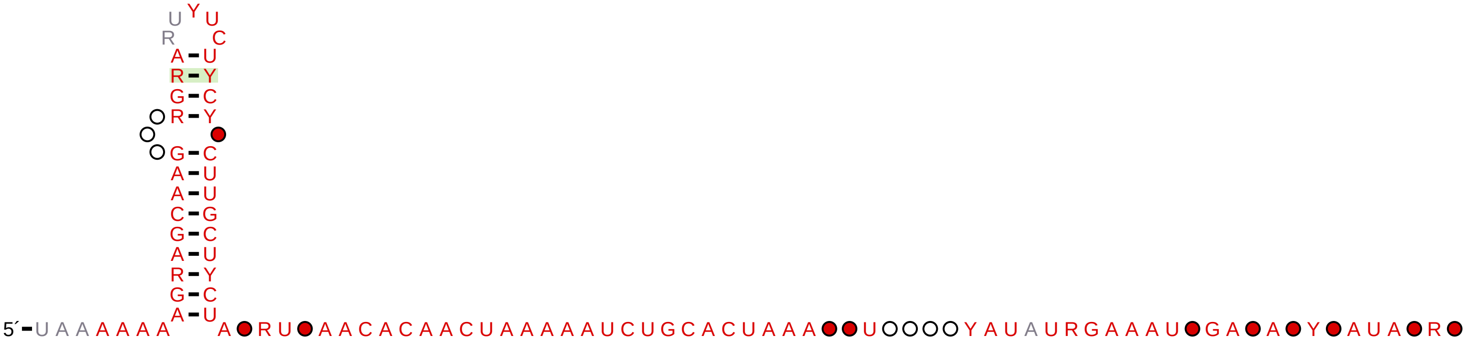

HMP2_5217_1

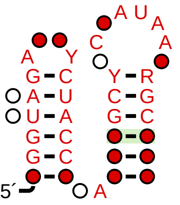

HMP2_5240_1

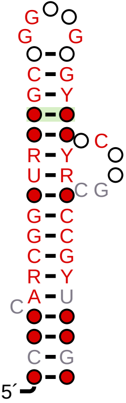

HMP2_526_1

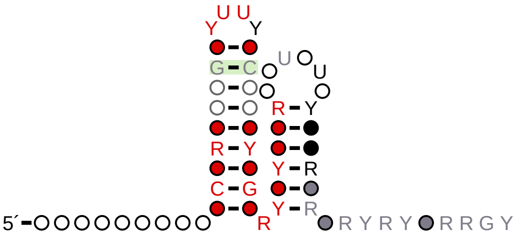

HMP2_5267_1

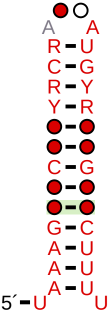

HMP2_5289_1

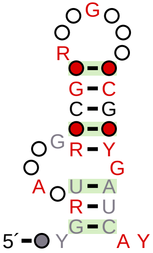

HMP2_5302_1

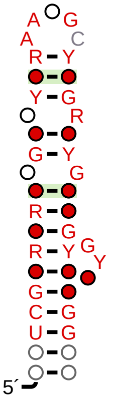

HMP2_5319_1

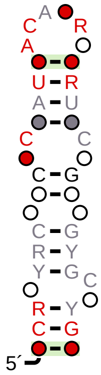

HMP2_5321_1

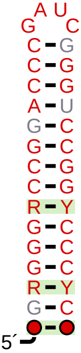

HMP2_5322_1

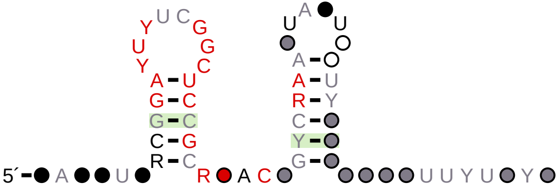

HMP2_5325_1

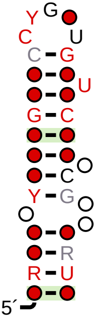

HMP2_5345_1

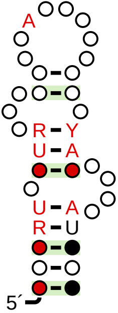

HMP2_5350_1

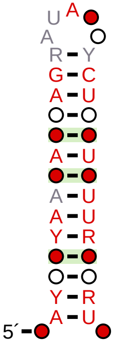

HMP2_5387_1

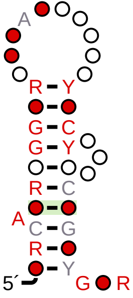

HMP2_5395_1

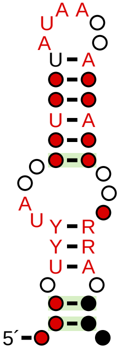

HMP2_5401_1

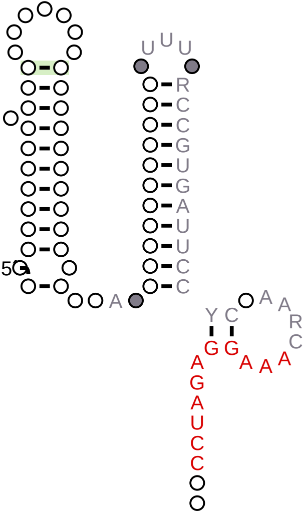

HMP2_5407_1

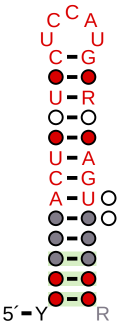

HMP2_542_1

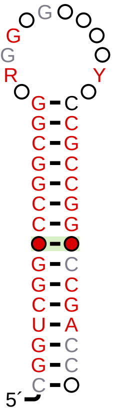

HMP2_5420_1

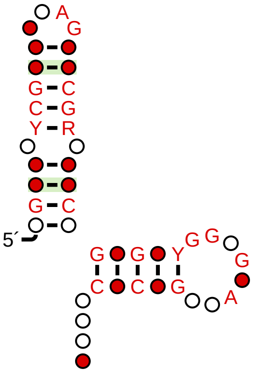

HMP2_5425_1

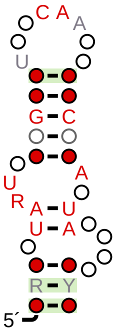

HMP2_5442_1

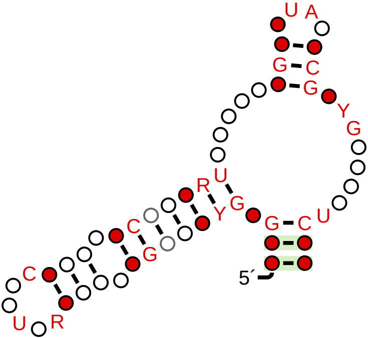

HMP2_5467_1

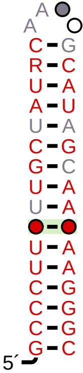

HMP2_5469_1

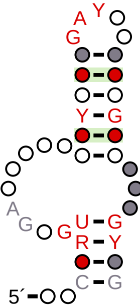

HMP2_5474_1

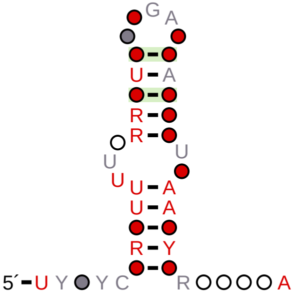

HMP2_5485_1

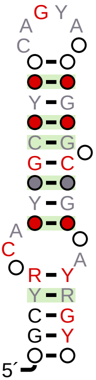

HMP2_5488_1

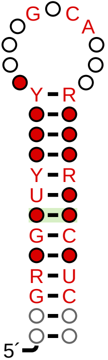

HMP2_5499_1

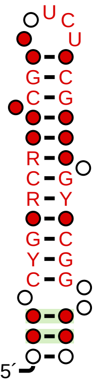

HMP2_5520_1

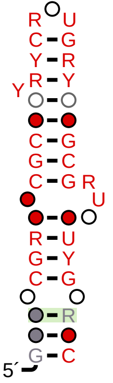

HMP2_553_1

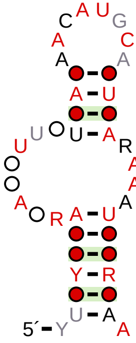

HMP2_5533_1

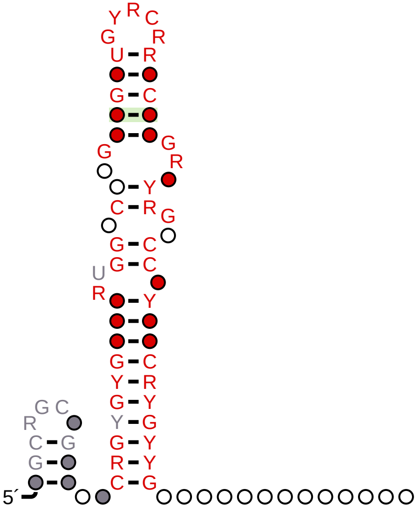

HMP2_5571_1

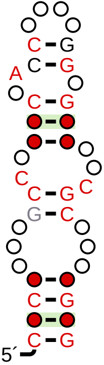

HMP2_5573_1

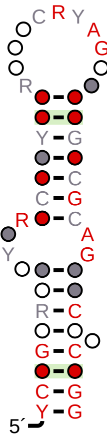

HMP2_5583_1

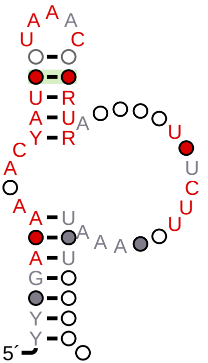

HMP2_5587_1

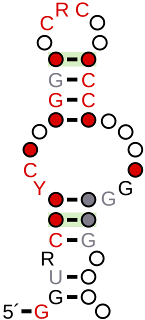

HMP2_5591_1

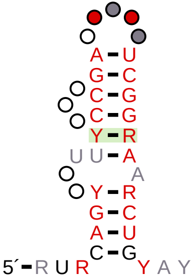

HMP2_5595_1

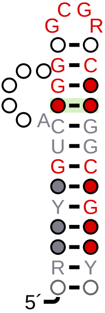

HMP2_5600_1

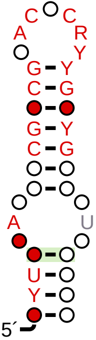

HMP2_5601_1

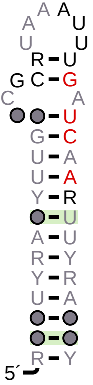

HMP2_5603_1

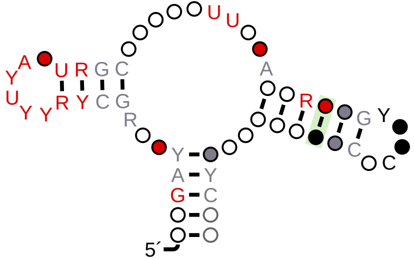

HMP2_5613_1

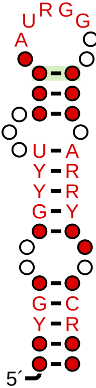

HMP2_5626_1

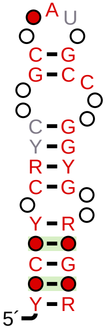

HMP2_5627_1

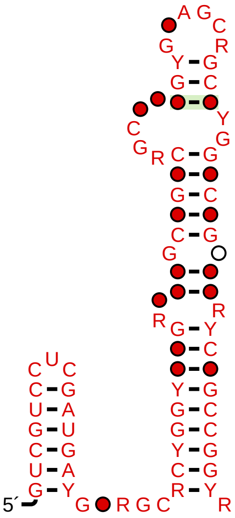

HMP2_5632_1

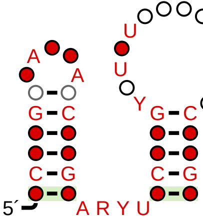

HMP2_5633_1

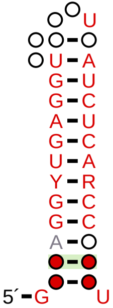

HMP2_5648_1

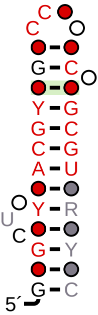

HMP2_565_1

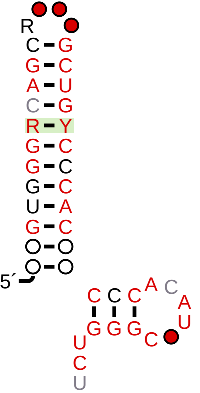

HMP2_5652_1

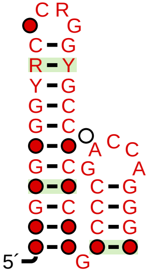

HMP2_5655_1

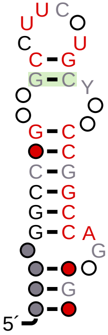

HMP2_5676_1

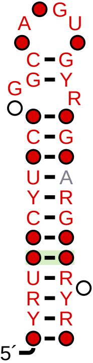

HMP2_5680_1

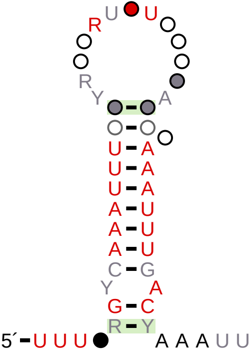

HMP2_5684_1

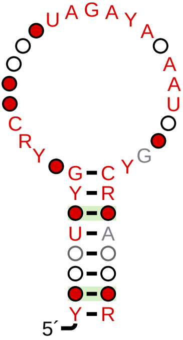

HMP2_5688_1

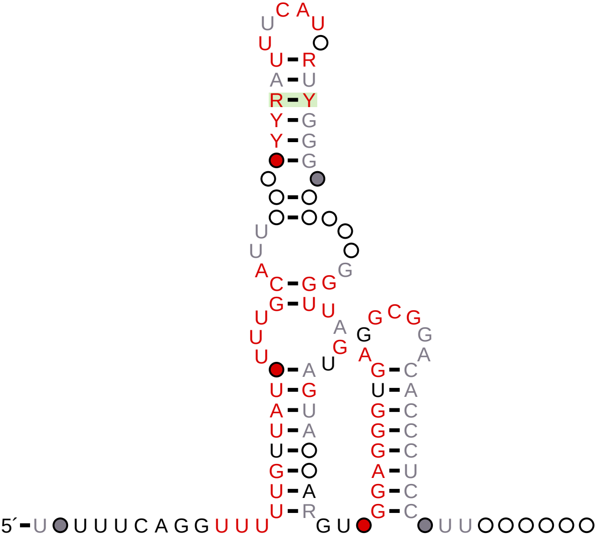

HMP2_5714_1

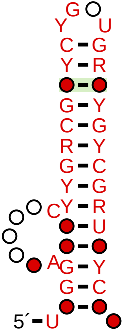

HMP2_5715_1

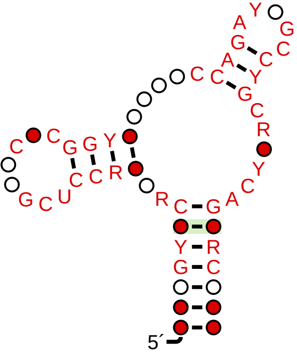

HMP2_572_1

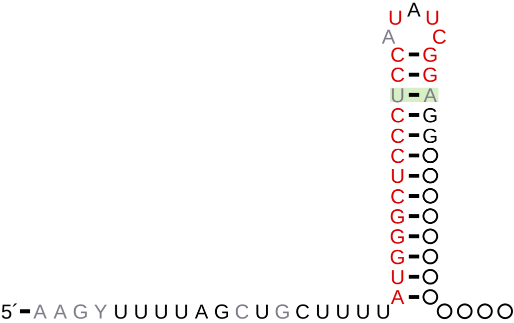

HMP2_5722_1

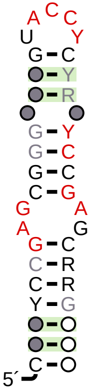

HMP2_5723_1

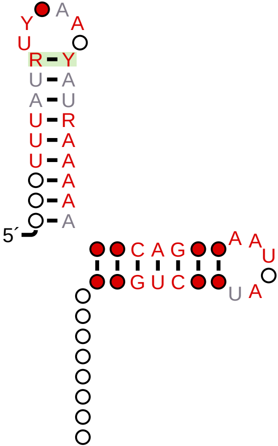

HMP2_5724_1

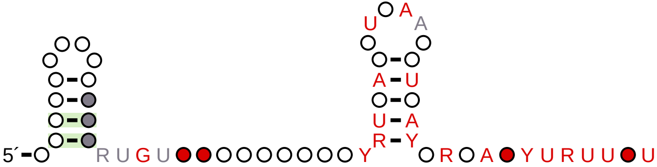

HMP2_5728_1

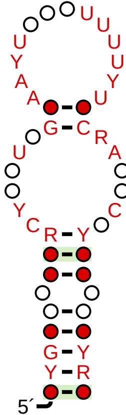

HMP2_5737_1

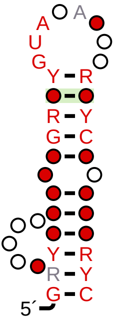

HMP2_5739_1

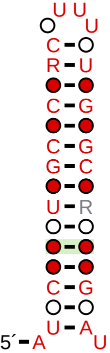

HMP2_575_1

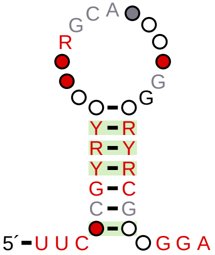

HMP2_5755_1

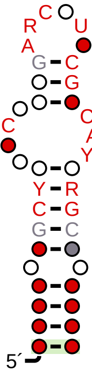

HMP2_5762_1

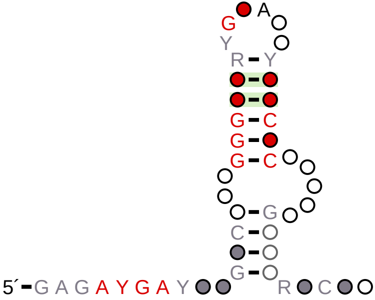

HMP2_5765_1

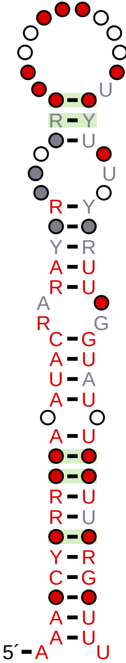

HMP2_5775_1

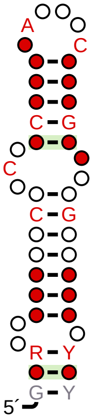

HMP2_5783_1

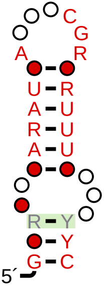

HMP2_5793_1

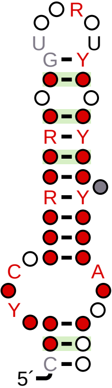

HMP2_5800_1

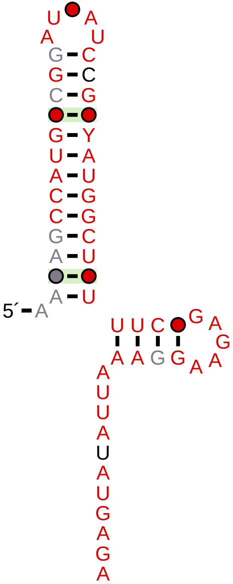

HMP2_5802_1

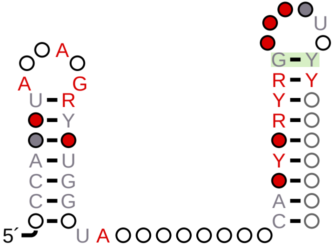

HMP2_5811_1

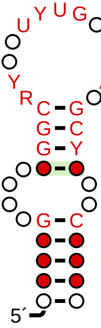

HMP2_5819_1

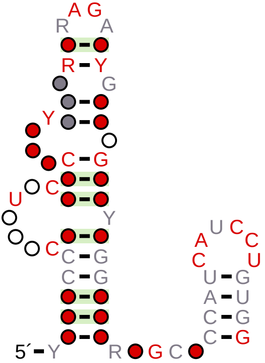

HMP2_5825_1

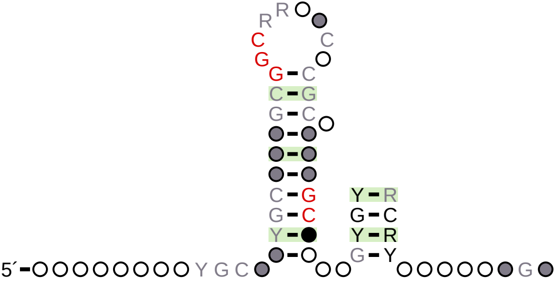

HMP2_5839_1

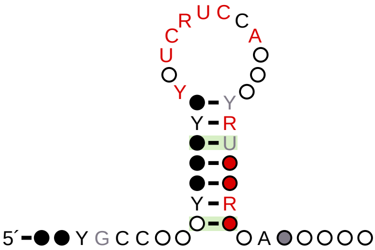

HMP2_584_1

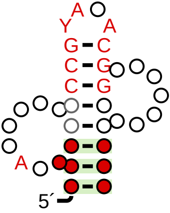

HMP2_5843_1

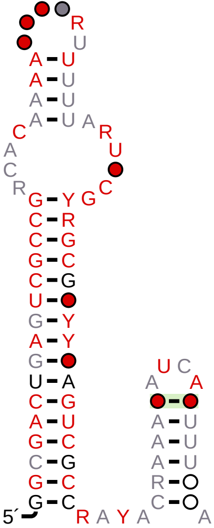

HMP2_5849_1

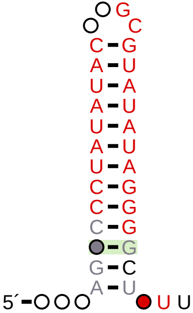

HMP2_5851_1

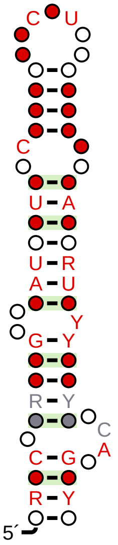

HMP2_5853_1

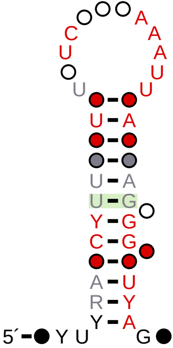

HMP2_5857_1

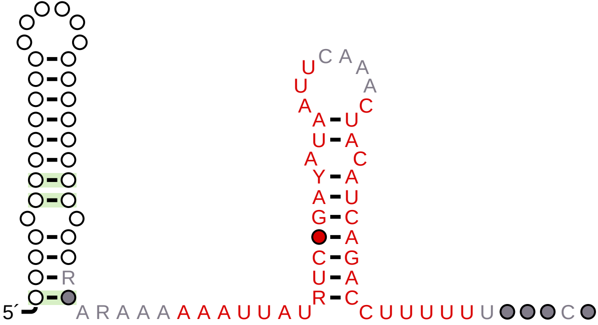

HMP2_5878_1

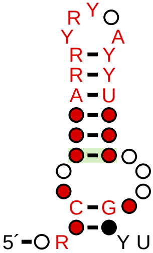

HMP2_5879_1

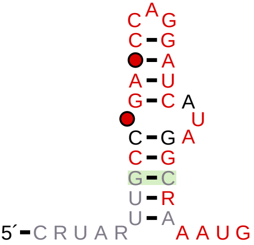

HMP2_588_1

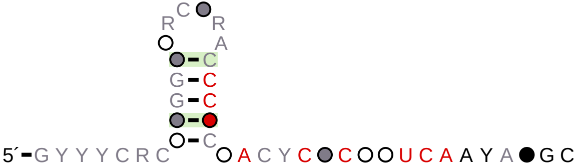

HMP2_5880_1

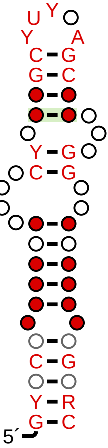

HMP2_5893_1

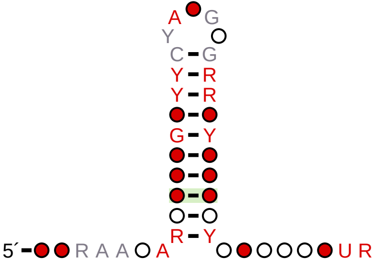

HMP2_5899_1

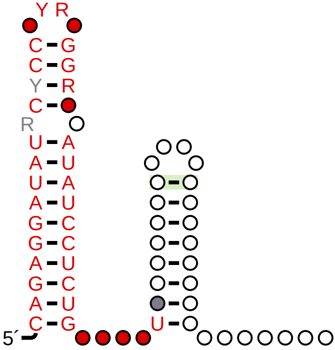

HMP2_5919_1

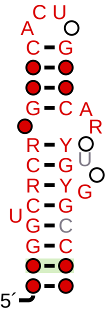

HMP2_5921_1

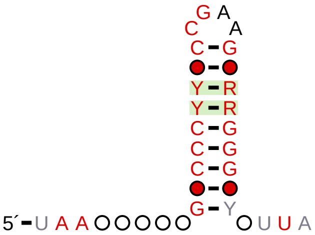

HMP2_5922_1

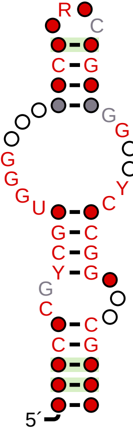

HMP2_5936_1

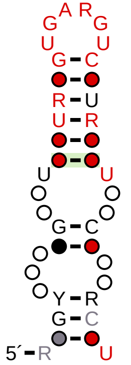

HMP2_5951_1

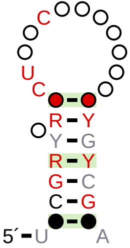

HMP2_5955_1

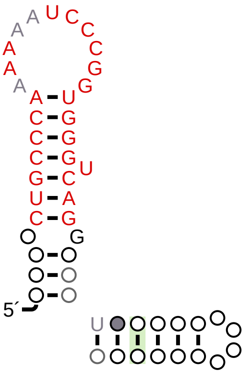

HMP2_5960_1

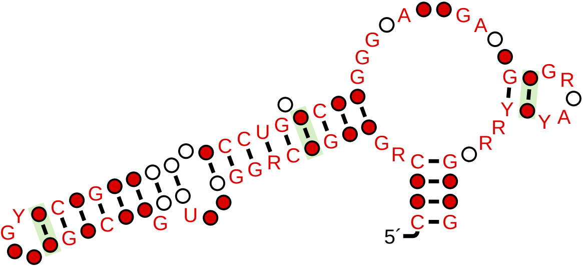

HMP2_5982_1

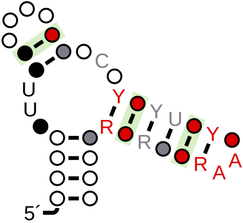

HMP2_5986_1

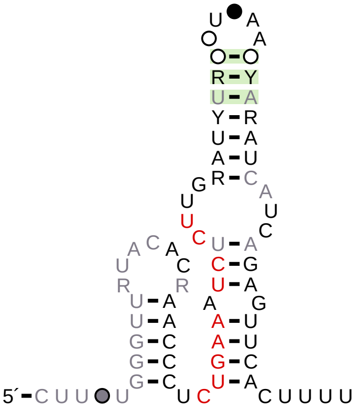

HMP2_5989_1

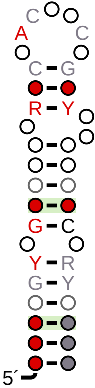

HMP2_600_1

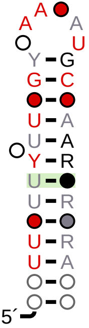

HMP2_602_1

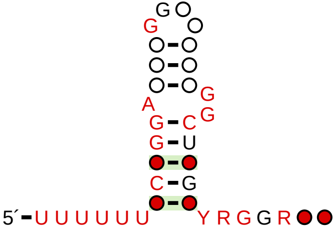

HMP2_6023_1

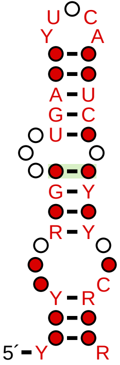

HMP2_603_1

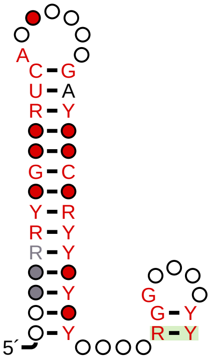

HMP2_6036_1

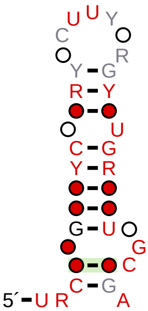

HMP2_6062_1

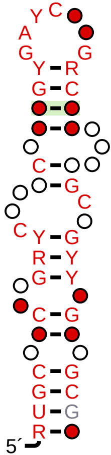

HMP2_608_1

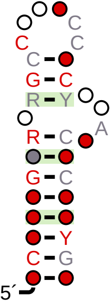

HMP2_610_1

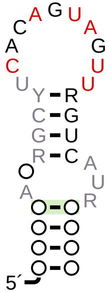

HMP2_6117_1

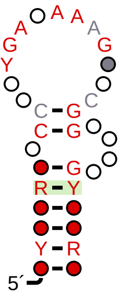

HMP2_6121_1

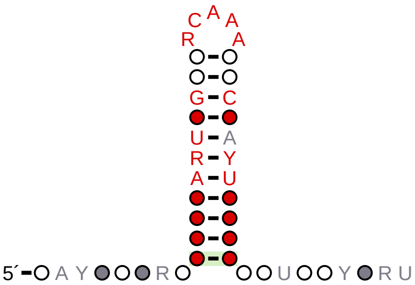

HMP2_6122_1

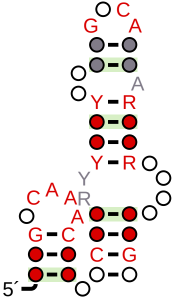

HMP2_6126_1

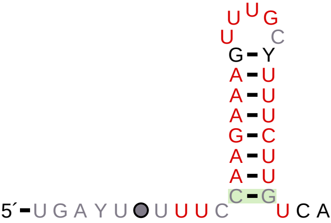

HMP2_6137_1

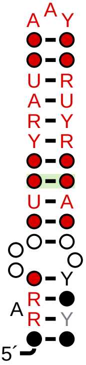

HMP2_6152_1

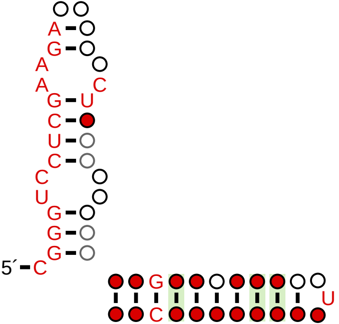

HMP2_6156_1

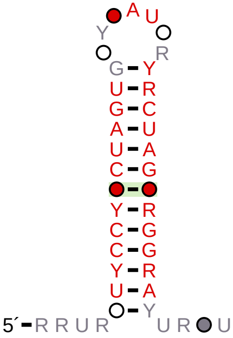

HMP2_6157_1

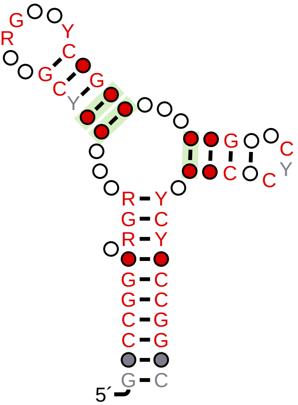

HMP2_6162_1

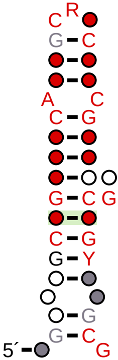

HMP2_6172_1

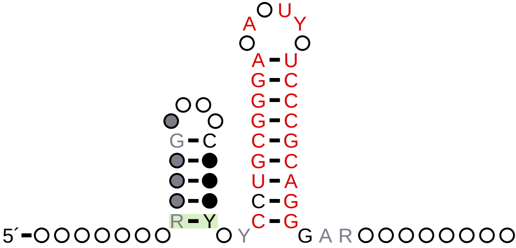

HMP2_6176_1

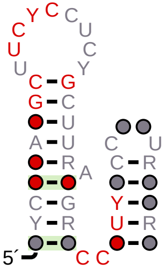

HMP2_6183_1

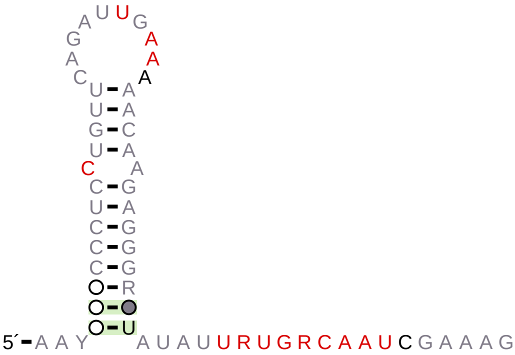

HMP2_6189_1

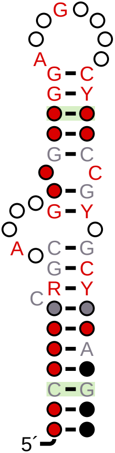

HMP2_6193_1

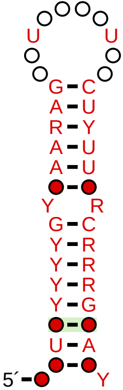

HMP2_6201_1

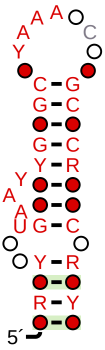

HMP2_6203_1

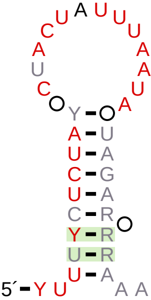

HMP2_6206_1

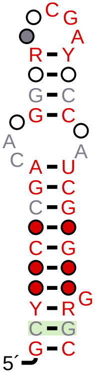

HMP2_6216_1

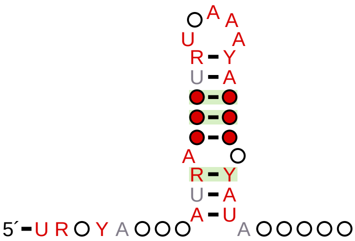

HMP2_6217_1

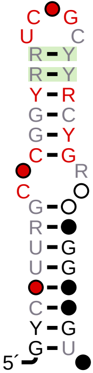

HMP2_6218_1

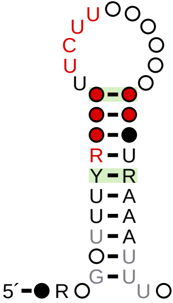

HMP2_6220_1

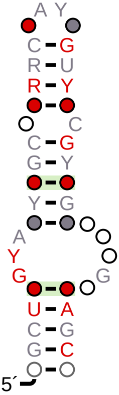

HMP2_6227_1

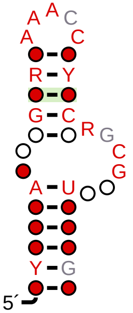

HMP2_6233_1

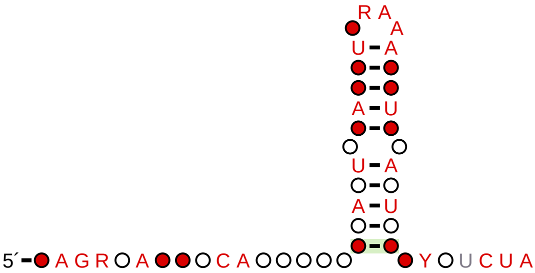

HMP2_6237_1

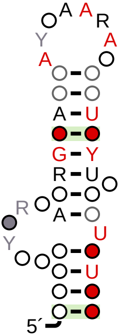

HMP2_6241_1

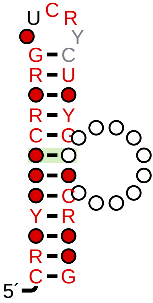

HMP2_6245_1

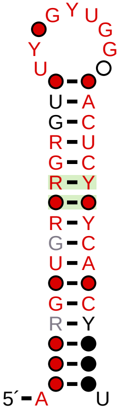

HMP2_6261_1

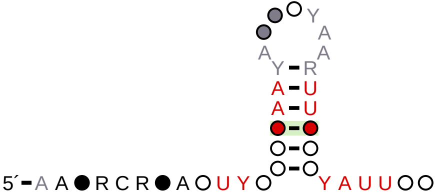

HMP2_6263_1

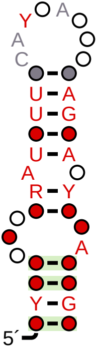

HMP2_6275_1

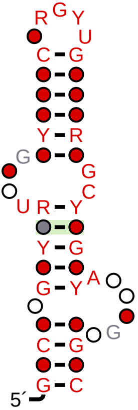

HMP2_6286_1

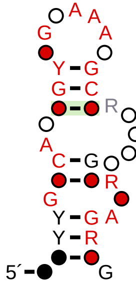

HMP2_6289_1

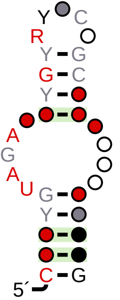

HMP2_6314_1

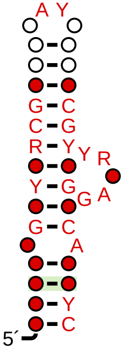

HMP2_632_1

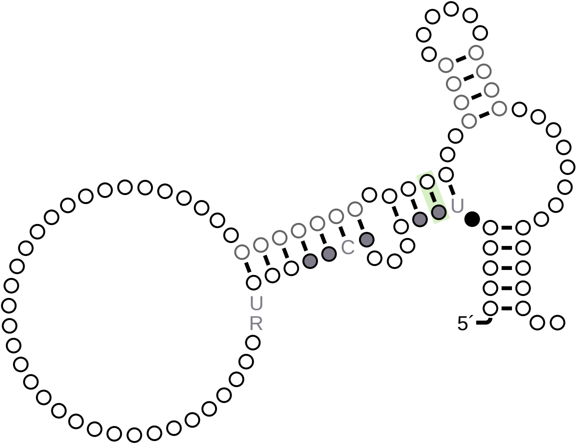

HMP2_6331_1

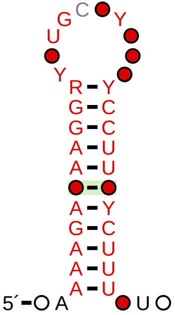

HMP2_6339_1

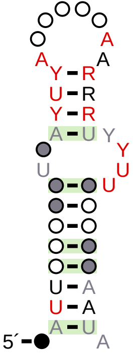

HMP2_634_1

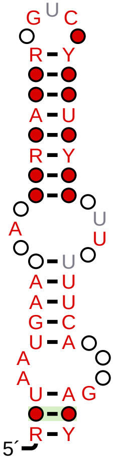

HMP2_6348_1

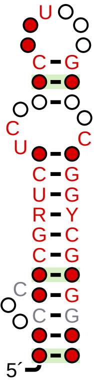

HMP2_6349_1

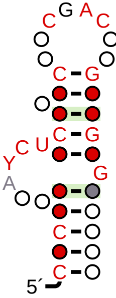

HMP2_6351_1

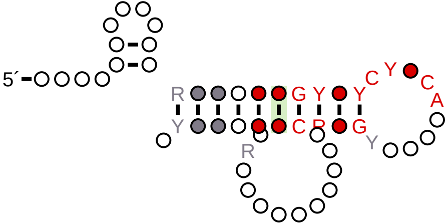

HMP2_6359_1

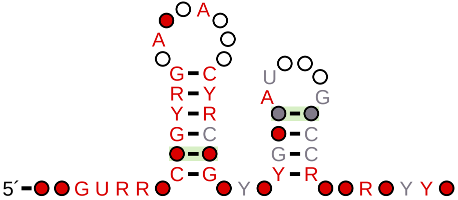

HMP2_6364_1

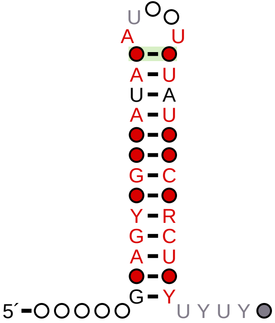

HMP2_638_1

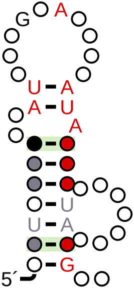

HMP2_6385_1

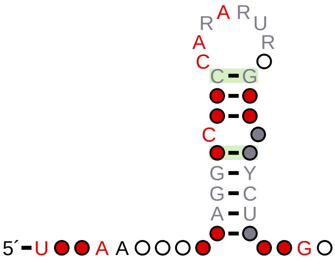

HMP2_6393_1

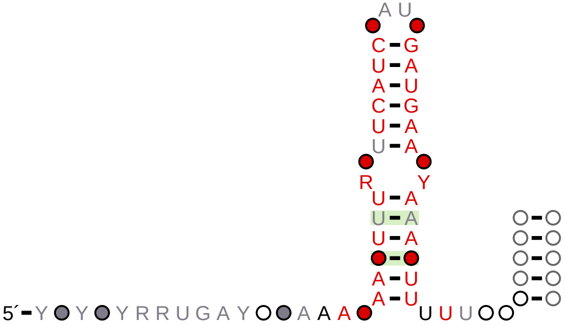

HMP2_6397_1

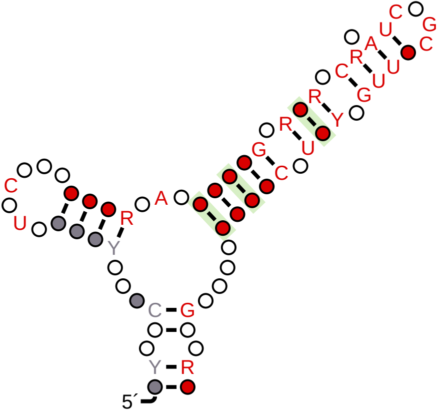

HMP2_6399_1

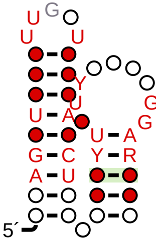

HMP2_6409_1

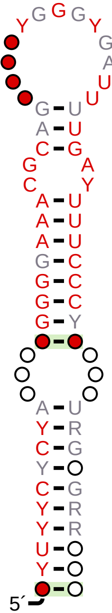

HMP2_6410_1

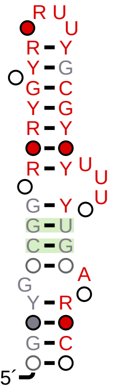

HMP2_6424_1

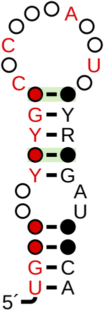

HMP2_6430_1

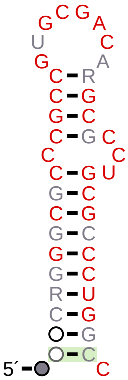

HMP2_6439_1

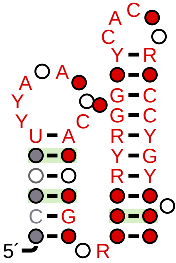

HMP2_6441_1

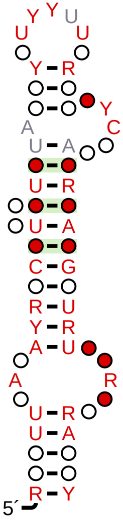

HMP2_6442_1

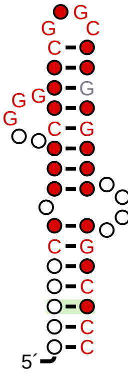

HMP2_6443_1

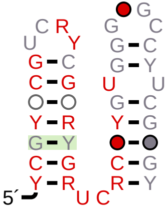

HMP2_6448_1

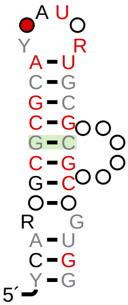

HMP2_6450_1

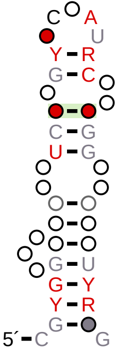

HMP2_6452_1

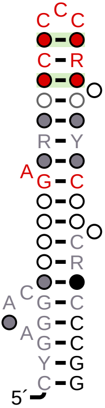

HMP2_6457_1

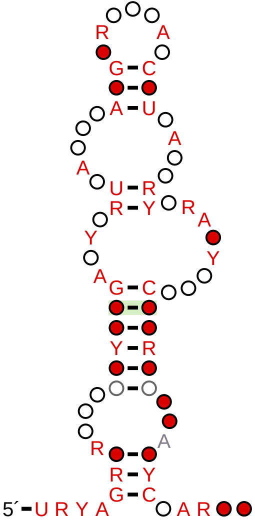

HMP2_6458_1

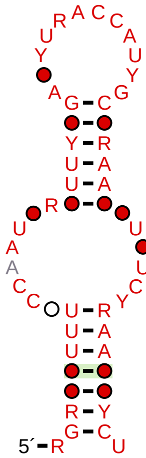

HMP2_6460_1

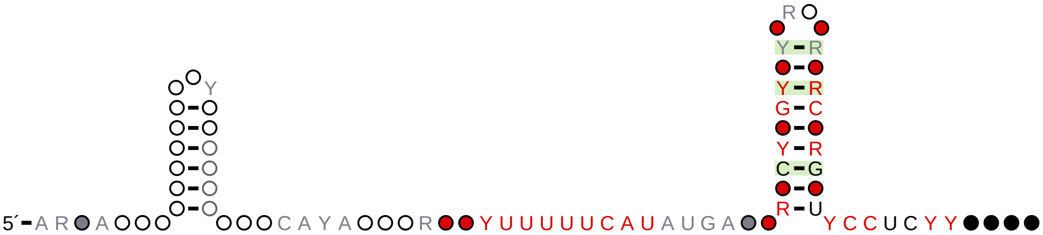

HMP2_6472_1

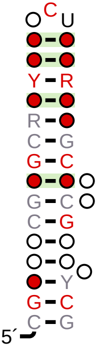

HMP2_6477_1

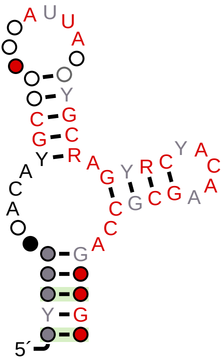

HMP2_6479_1

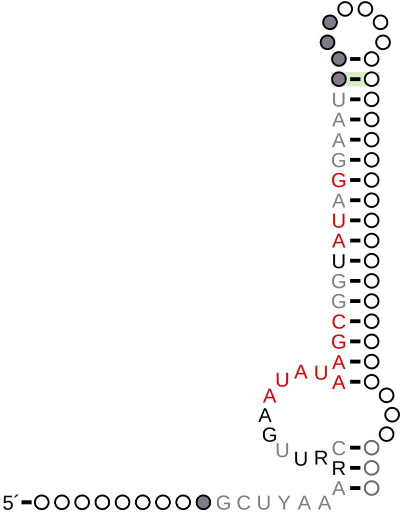

HMP2_6482_1

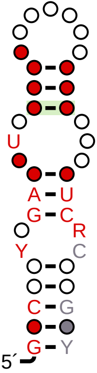

HMP2_6495_1

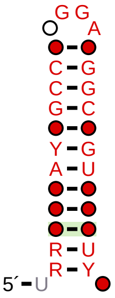

HMP2_6502_1

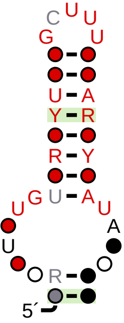

HMP2_6507_1

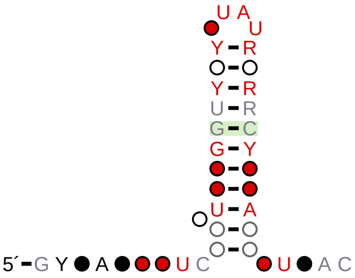

HMP2_6522_1

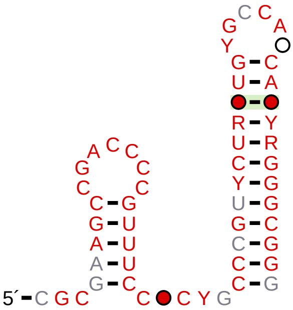

HMP2_6524_1

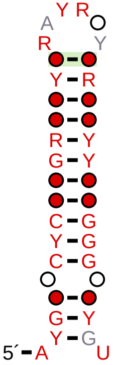

HMP2_6534_1

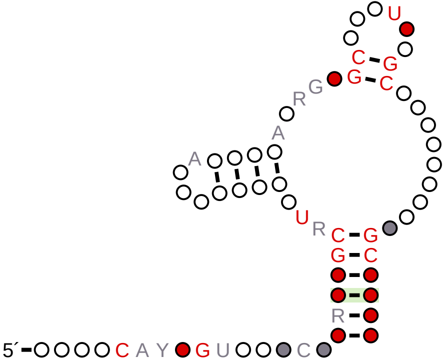

HMP2_654_1

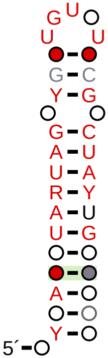

HMP2_6552_1

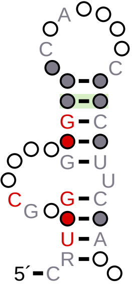

HMP2_6557_1

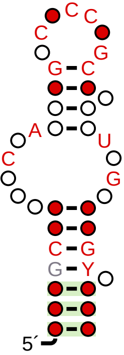

HMP2_6559_1

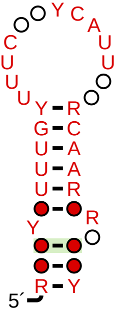

HMP2_6561_1

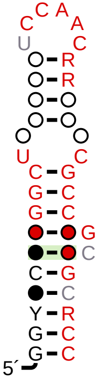

HMP2_6567_1

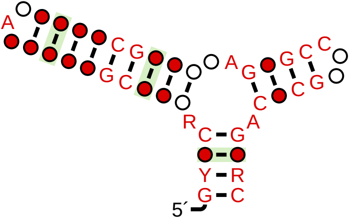

HMP2_6570_1

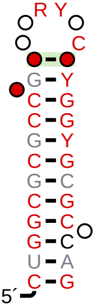

HMP2_6583_1

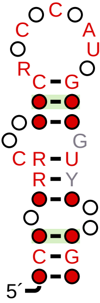

HMP2_6585_1

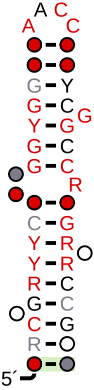

HMP2_6601_1

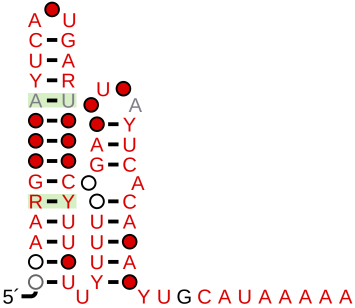

HMP2_6621_1

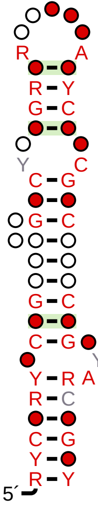

HMP2_6628_1

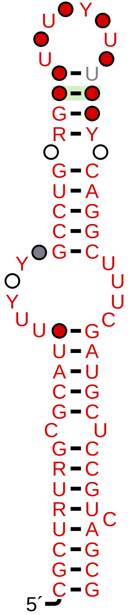

HMP2_6631_1

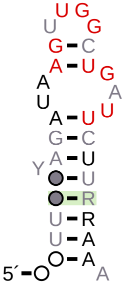

HMP2_6633_1

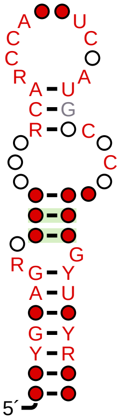

HMP2_6646_1

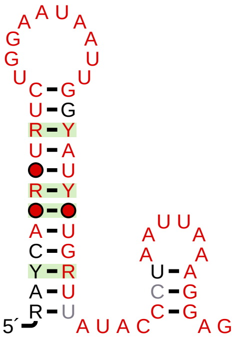

HMP2_666_1

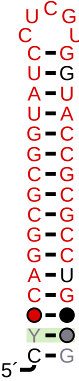

HMP2_6661_1

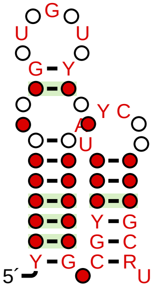

HMP2_6676_1

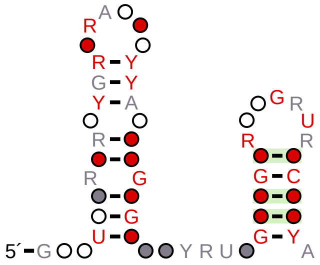

HMP2_668_1

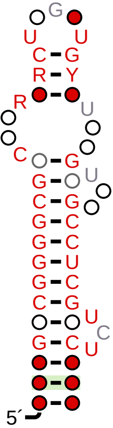

HMP2_6681_1

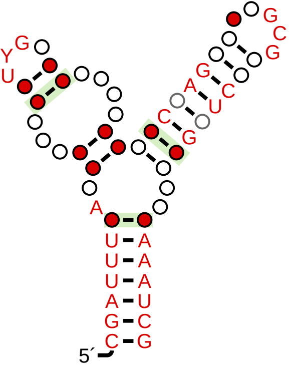

HMP2_6683_1

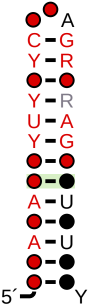

HMP2_6686_1

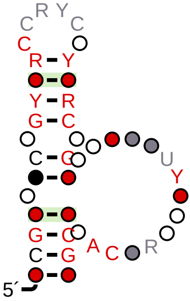

HMP2_6697_1

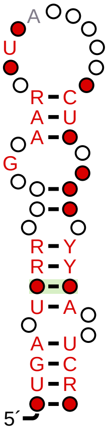

HMP2_67_1

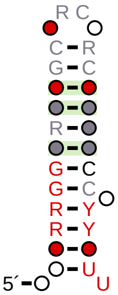

HMP2_6705_1

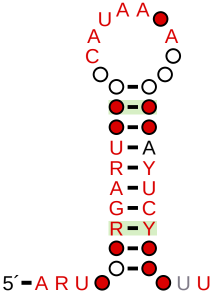

HMP2_6706_1

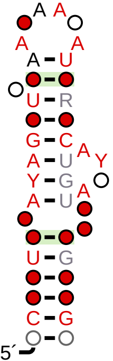

HMP2_6723_1

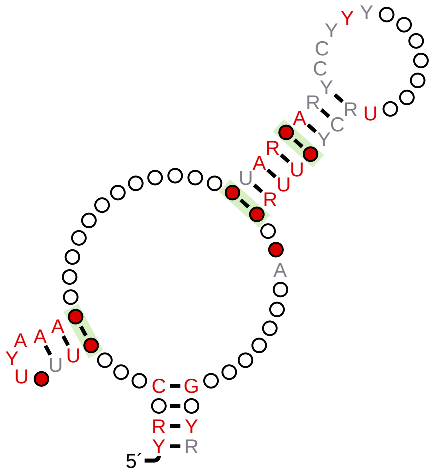

HMP2_6725_1

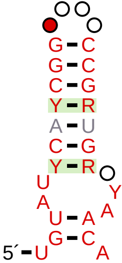

HMP2_6731_1

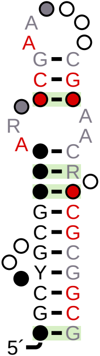

HMP2_6732_1

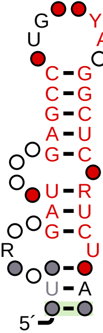

HMP2_6738_1

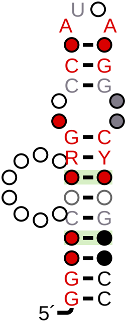

HMP2_674_1

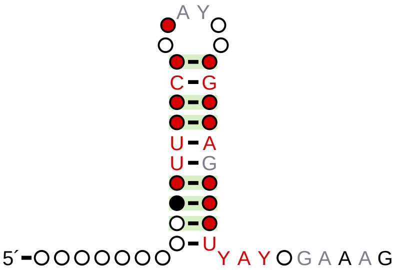

HMP2_6743_1

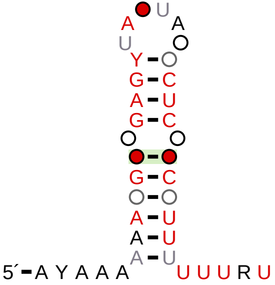

HMP2_675_1

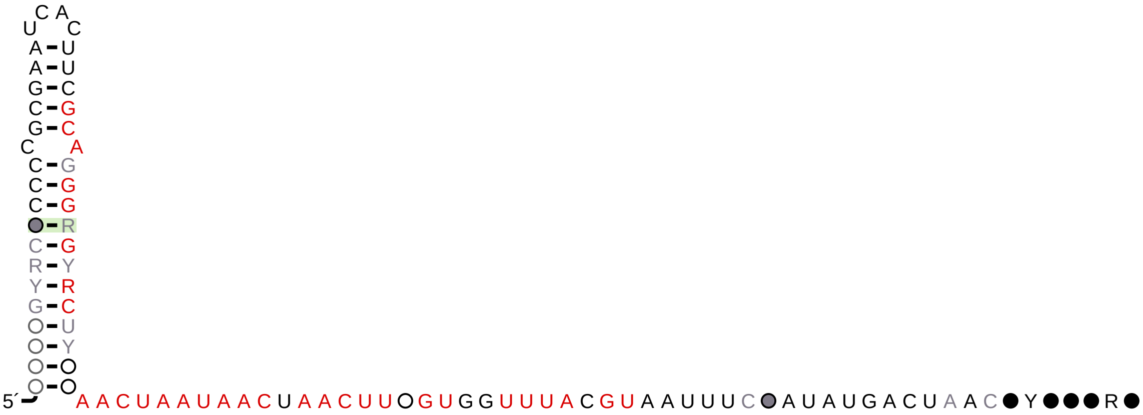

HMP2_6752_1

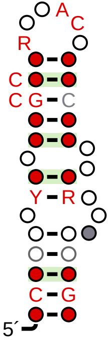

HMP2_6754_1

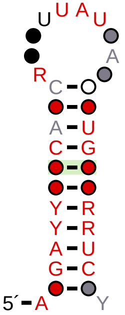

HMP2_6761_1

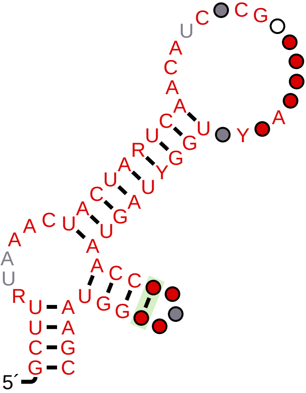

HMP2_6762_1

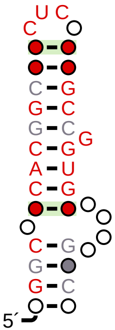

HMP2_6782_1

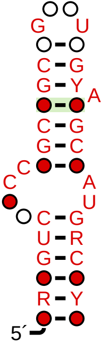

HMP2_6785_1

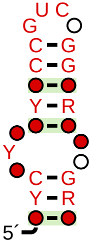

HMP2_6787_1

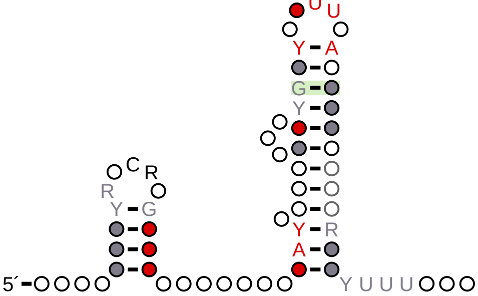

HMP2_6801_1

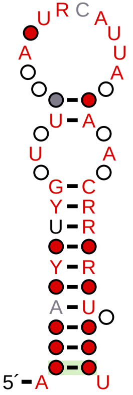

HMP2_6806_1

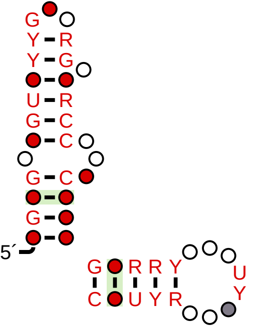

HMP2_6807_1

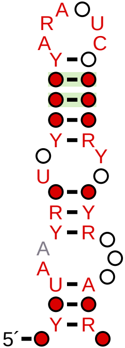

HMP2_6813_1

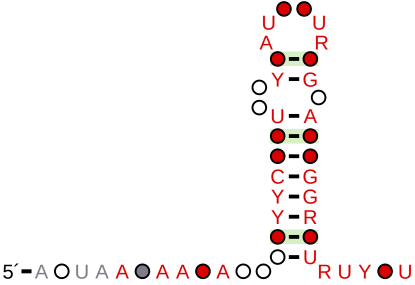

HMP2_6838_1

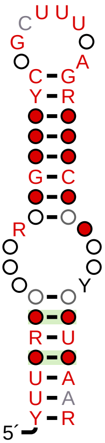

HMP2_684_1

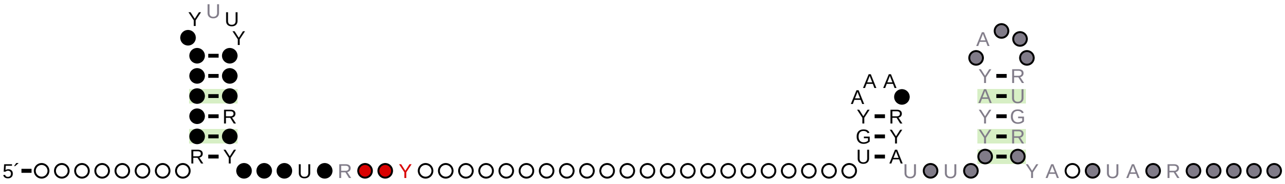

HMP2_6840_1

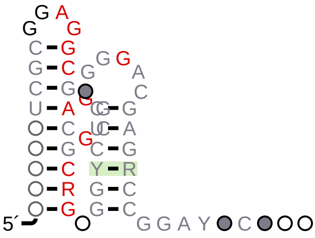

HMP2_6849_1

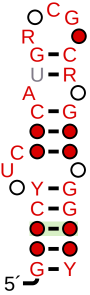

HMP2_6854_1

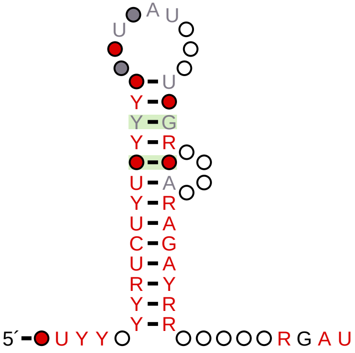

HMP2_6869_1

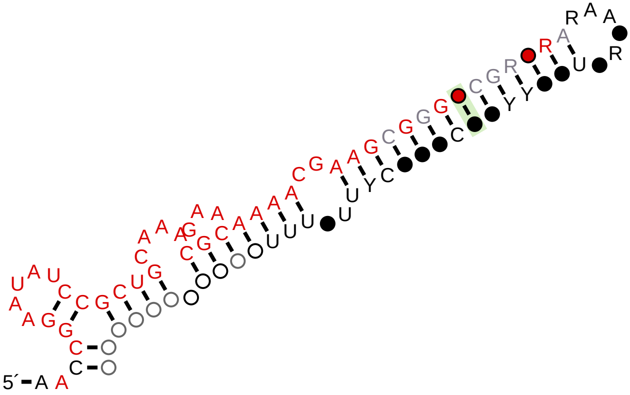

HMP2_6872_1

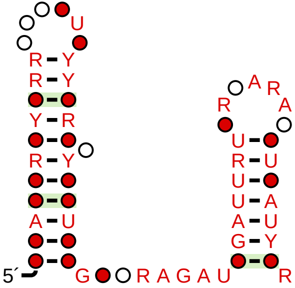

HMP2_6878_1

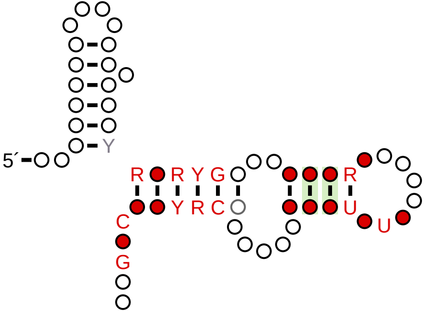

HMP2_688_1

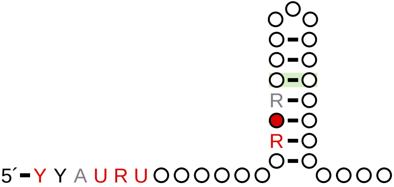

HMP2_6880_1

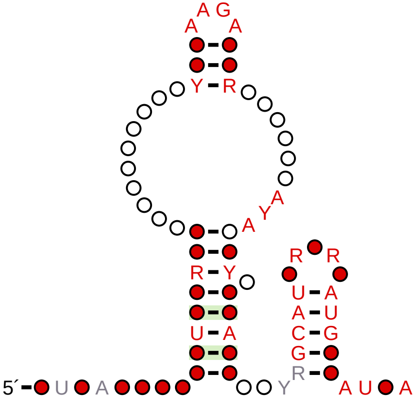

HMP2_6883_1

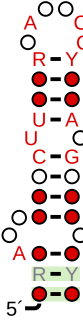

HMP2_6884_1

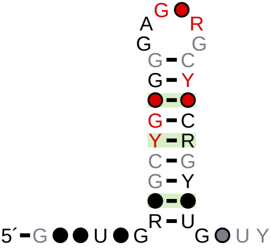

HMP2_6888_1

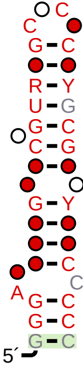

HMP2_689_1

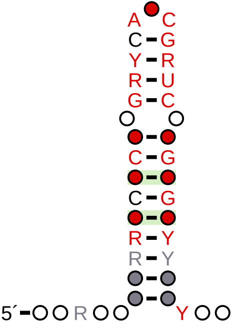

HMP2_6894_1

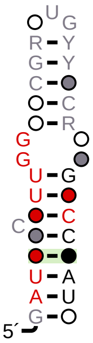

HMP2_6897_1

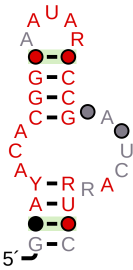

HMP2_6907_1

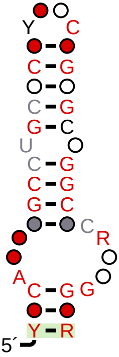

HMP2_691_1

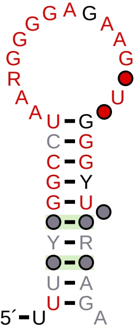

HMP2_6931_1

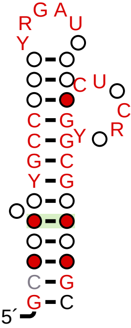

HMP2_6946_1

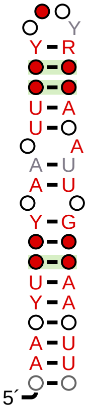

HMP2_6950_1

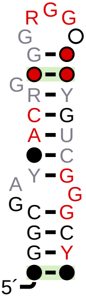

HMP2_6958_1

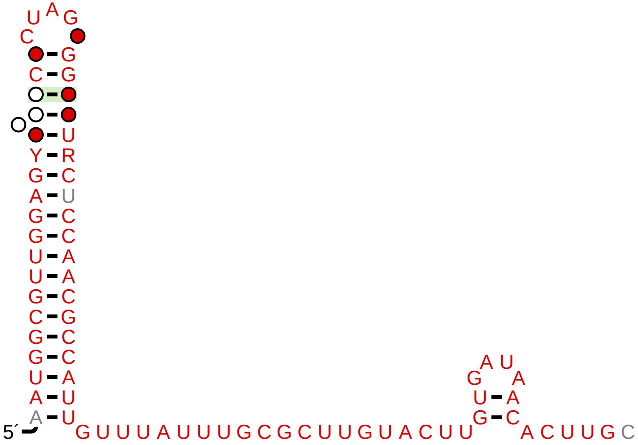

HMP2_6962_1

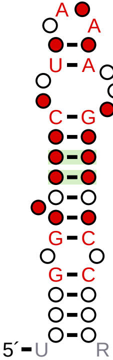

HMP2_6963_1

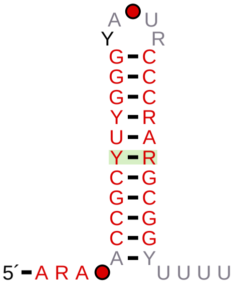

HMP2_6971_1

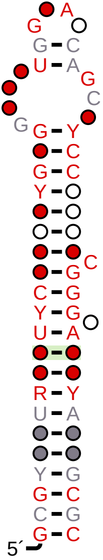

HMP2_6975_1

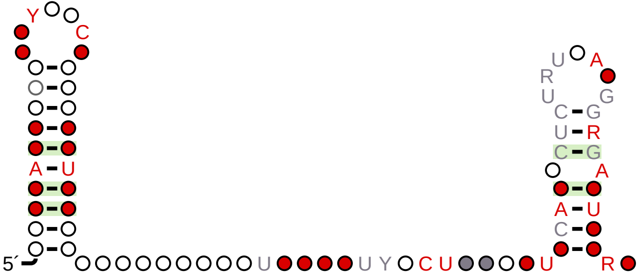

HMP2_6978_1

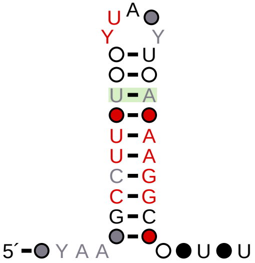

HMP2_6989_1

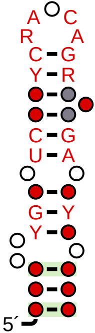

HMP2_7006_1

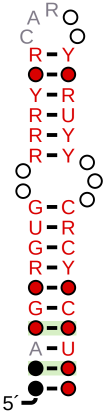

HMP2_7011_1

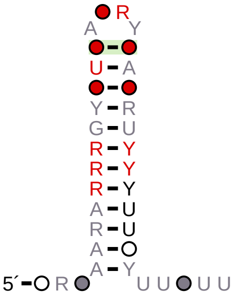

HMP2_7023_1

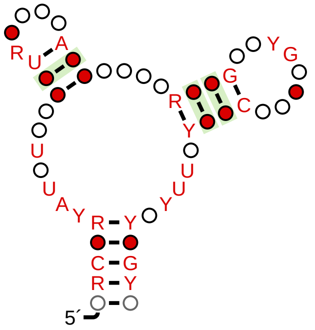

HMP2_7027_1

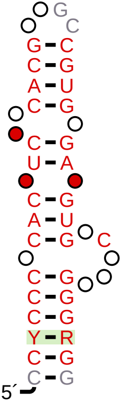

HMP2_7029_1

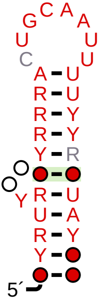

HMP2_7031_1

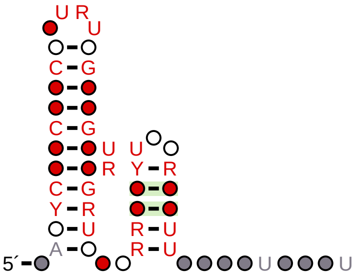

HMP2_7035_1

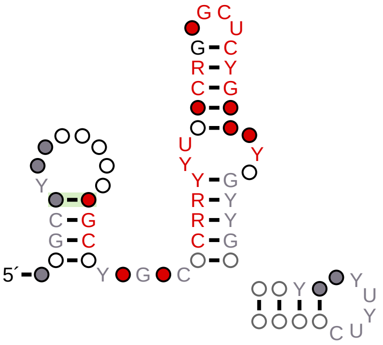

HMP2_7047_1

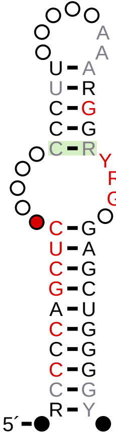

HMP2_7050_1

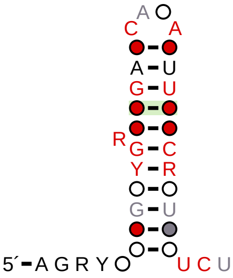

HMP2_7054_1

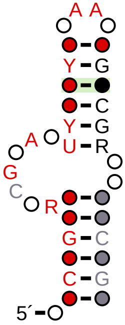

HMP2_706_1

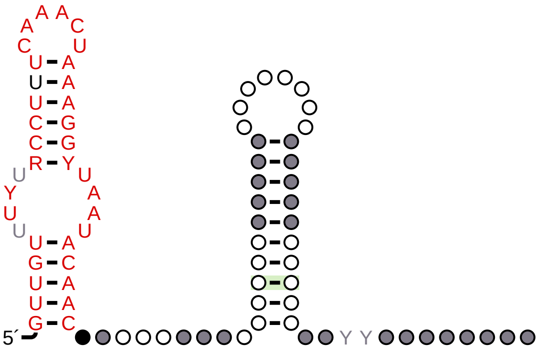

HMP2_7069_1

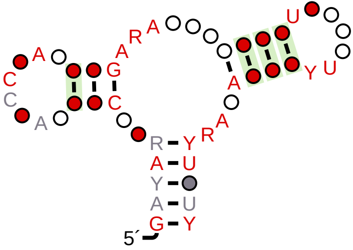

HMP2_7070_1

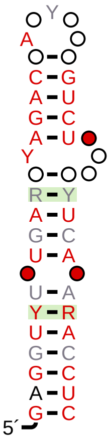

HMP2_7075_1

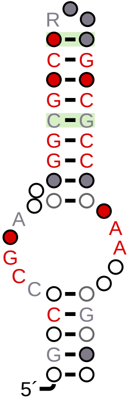

HMP2_7083_1

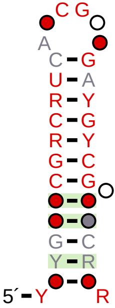

HMP2_7084_1

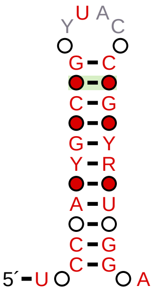

HMP2_7095_1

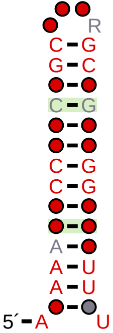

HMP2_7110_1

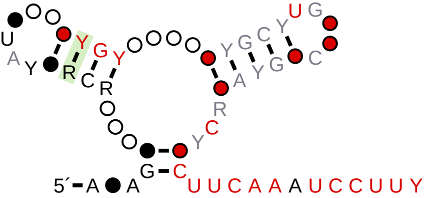

HMP2_7116_1

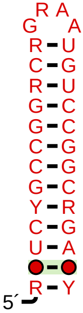

HMP2_7119_1

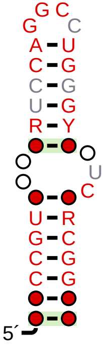

HMP2_7147_1

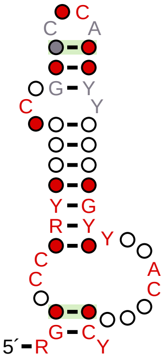

HMP2_715_1

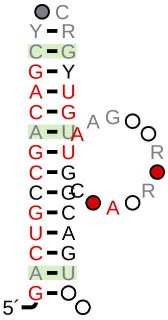

HMP2_7156_1

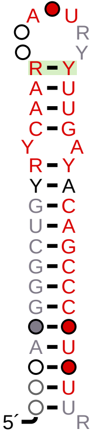

HMP2_7158_1

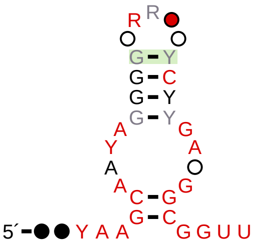

HMP2_7160_1

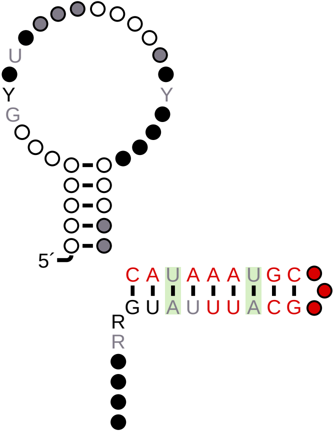

HMP2_7166_1

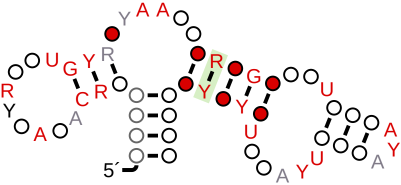

HMP2_7173_1

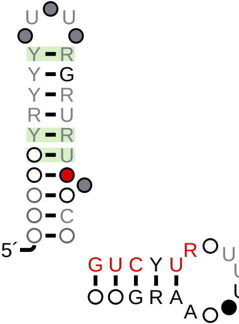

HMP2_7175_1

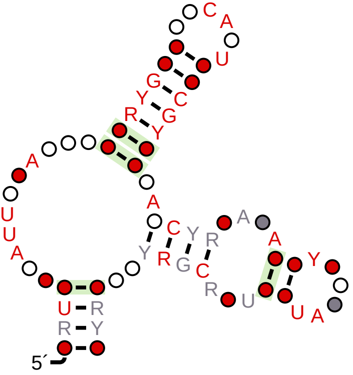

HMP2_7179_1

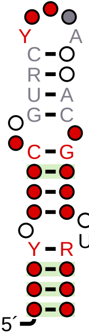

HMP2_7203_1

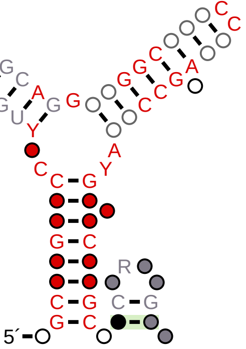

HMP2_7211_1

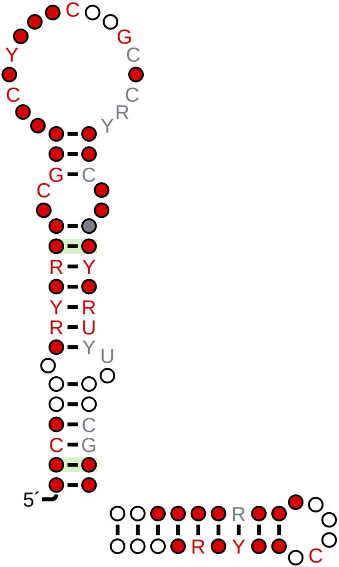

HMP2_7224_1

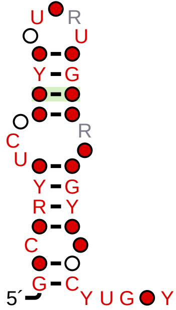

HMP2_7225_1

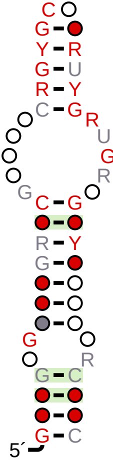

HMP2_7226_1

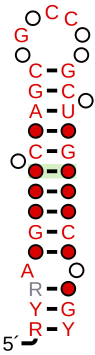

HMP2_724_1

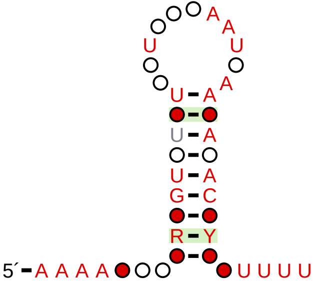

HMP2_7240_1

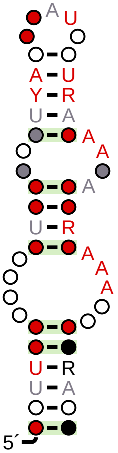

HMP2_7243_1

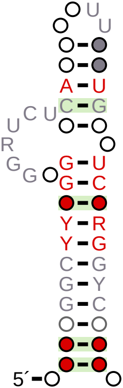

HMP2_7245_1

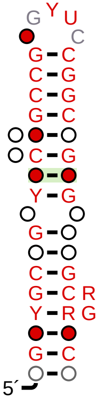

HMP2_7264_1

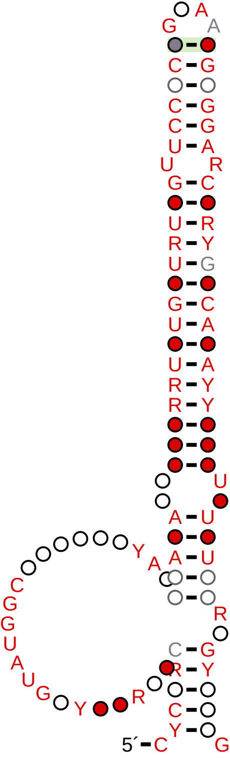

HMP2_7278_1

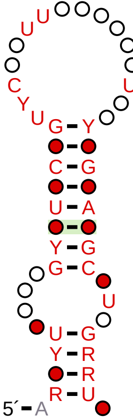

HMP2_7282_1

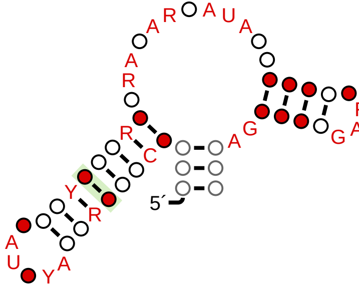

HMP2_7297_1

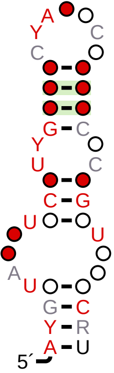

HMP2_7301_1

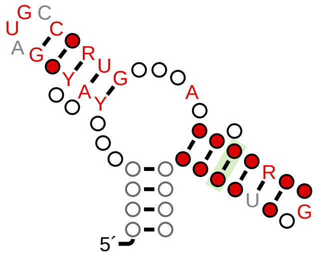

HMP2_7317_1

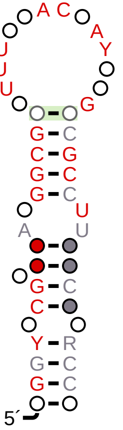

HMP2_7319_1

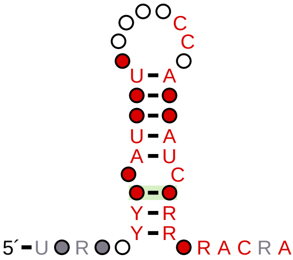

HMP2_7329_1

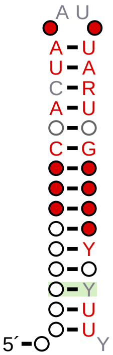

HMP2_7332_1

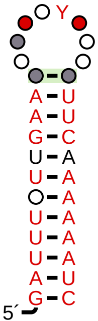

HMP2_7334_1

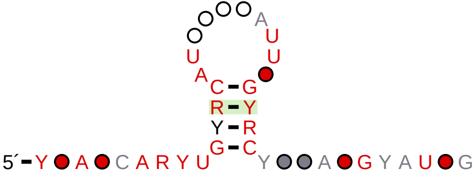

HMP2_7343_1

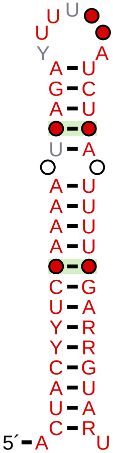

HMP2_7346_1

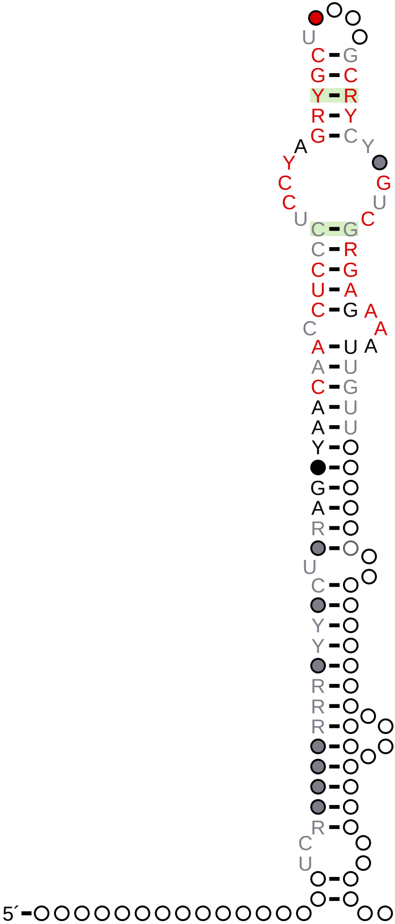

HMP2_735_1

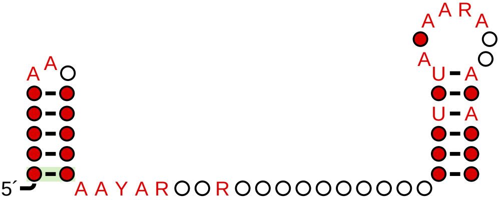

HMP2_7360_1

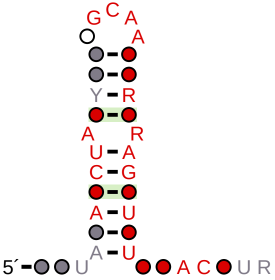

HMP2_7371_1

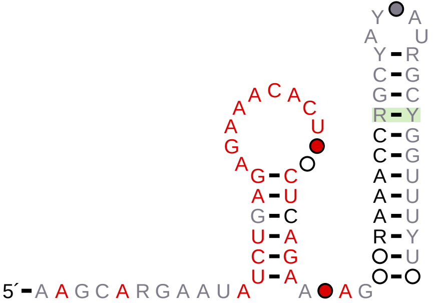

HMP2_7376_1

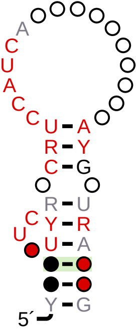

HMP2_7384_1

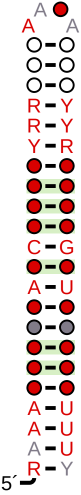

HMP2_7386_1

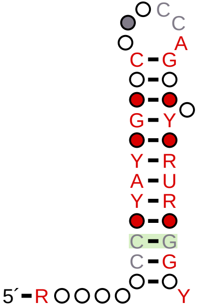

HMP2_7388_1

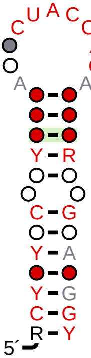

HMP2_7394_1

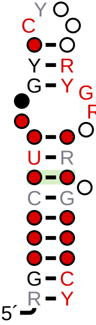

HMP2_741_1

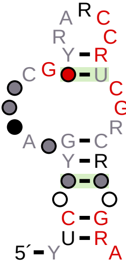

HMP2_7412_1

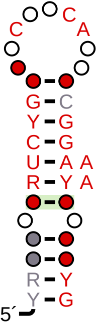

HMP2_7419_1

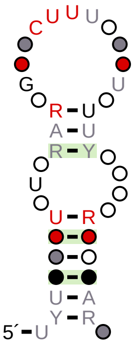

HMP2_7427_1

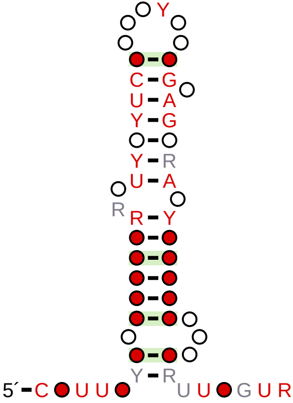

HMP2_7434_1

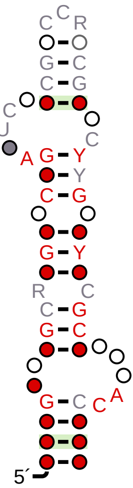

HMP2_7439_1

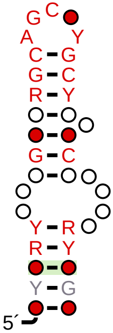

HMP2_7453_1

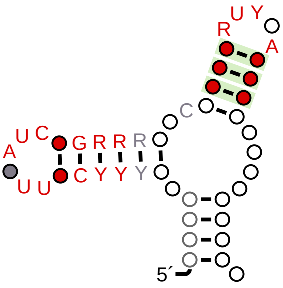

HMP2_7459_1

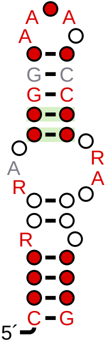

HMP2_7476_1

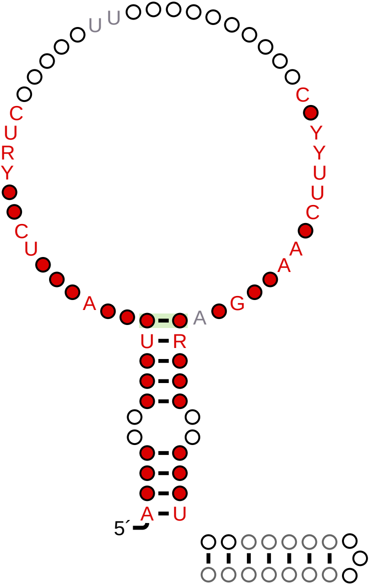

HMP2_7481_1

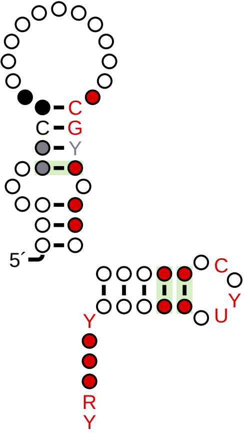

HMP2_7486_1

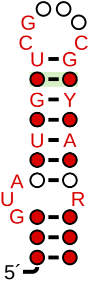

HMP2_7496_1

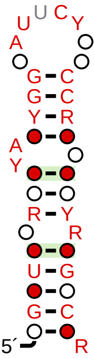

HMP2_7504_1

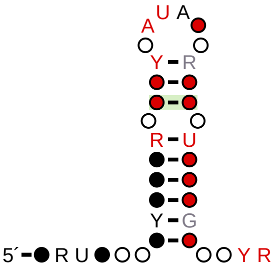

HMP2_7505_1

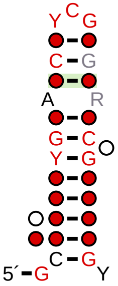

HMP2_7513_1

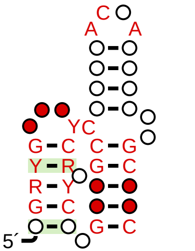

HMP2_752_1

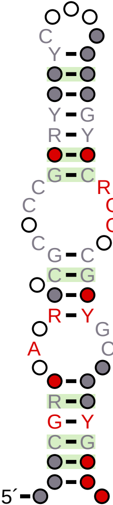

HMP2_7521_1

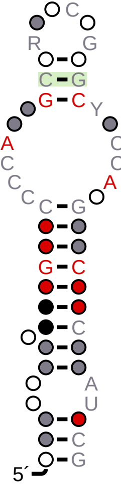

HMP2_7527_1

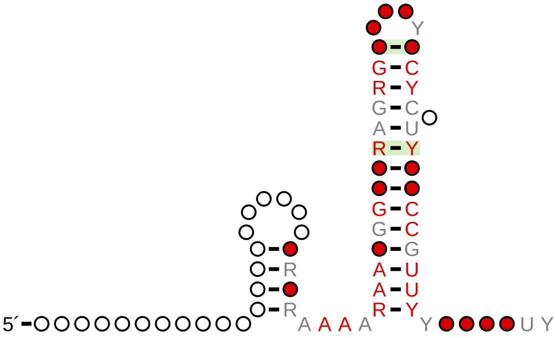

HMP2_7546_1

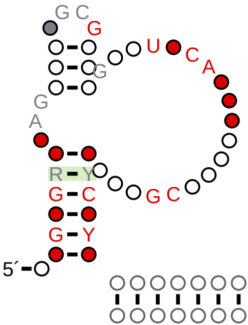

HMP2_7552_1

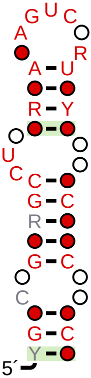

HMP2_7576_1

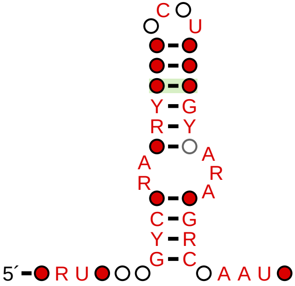

HMP2_7580_1

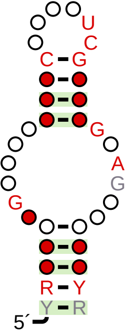

HMP2_7585_1

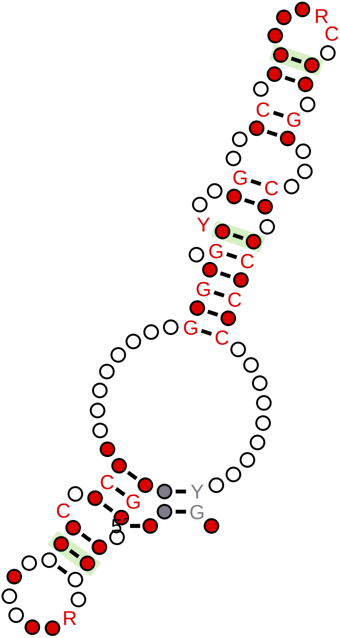

HMP2_7588_1

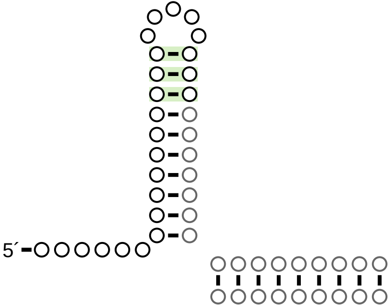

HMP2_7591_1

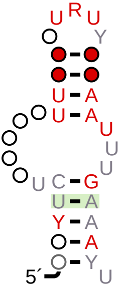

HMP2_7593_1

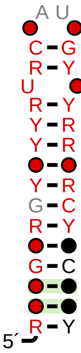

HMP2_7596_1

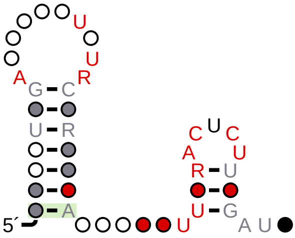

HMP2_7597_1

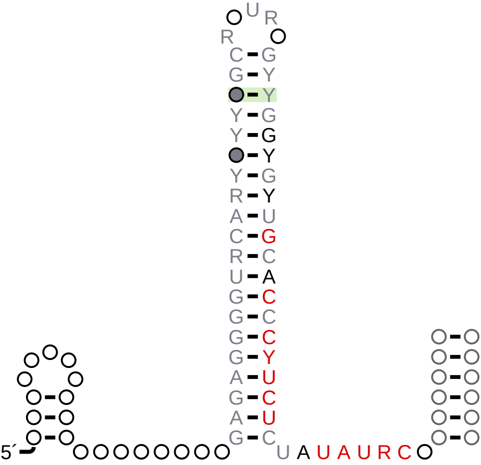

HMP2_7598_1

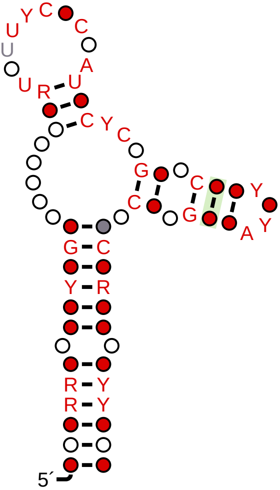

HMP2_7601_1

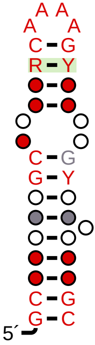

HMP2_7615_1

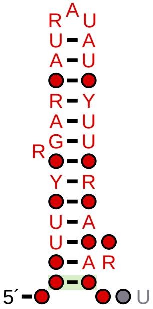

HMP2_762_1

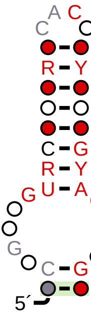

HMP2_7620_1

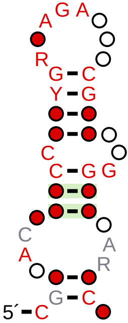

HMP2_7626_1

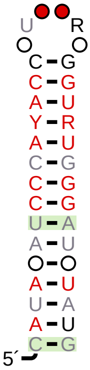

HMP2_763_1

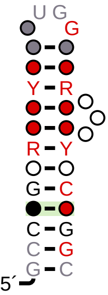

HMP2_7657_1

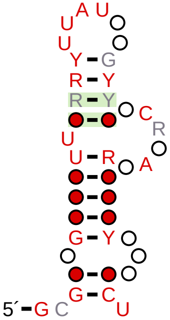

HMP2_7670_1

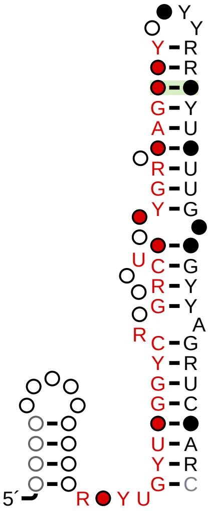

HMP2_7678_1

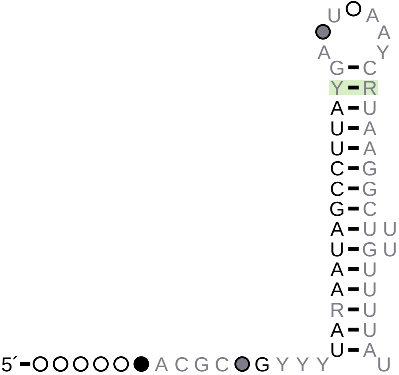

HMP2_7683_1

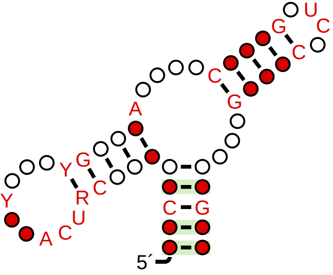

HMP2_7690_1

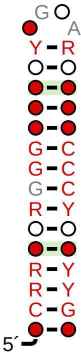

HMP2_7702_1

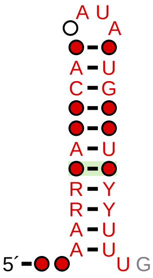

HMP2_7717_1

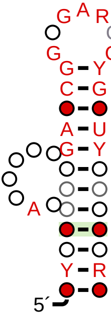

HMP2_7722_1

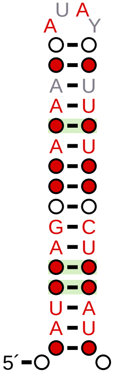

HMP2_7726_1

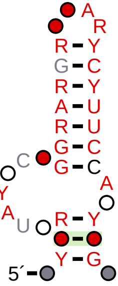

HMP2_7739_1

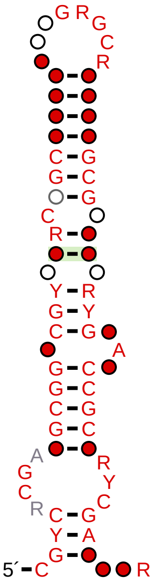

HMP2_7745_1

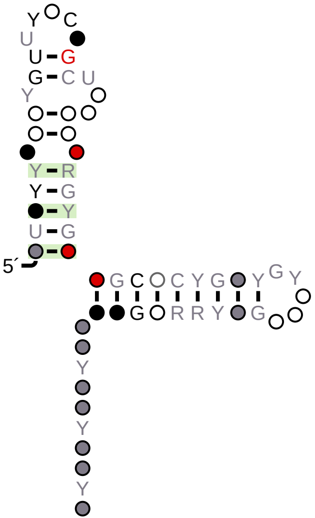

HMP2_7763_1

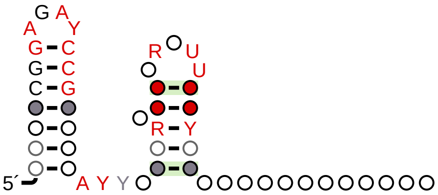

HMP2_7788_1

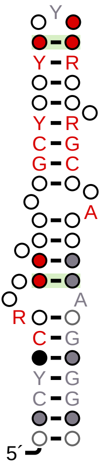

HMP2_7791_1

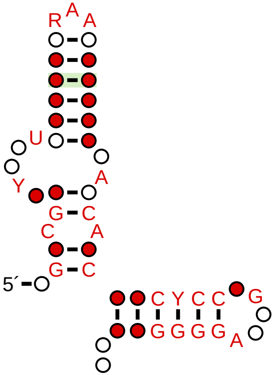

HMP2_7808_1

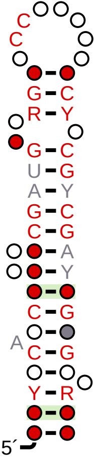

HMP2_7815_1

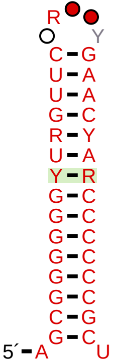

HMP2_7818_1

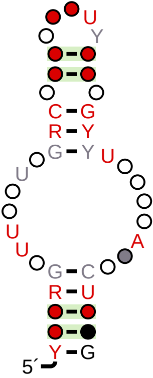

HMP2_782_1

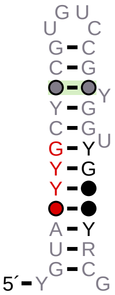

HMP2_7820_1

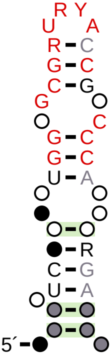

HMP2_7821_1

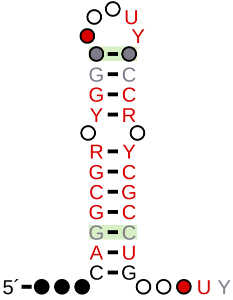

HMP2_783_1

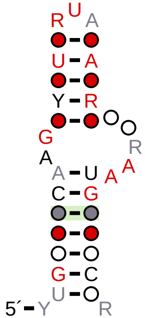

HMP2_7840_1

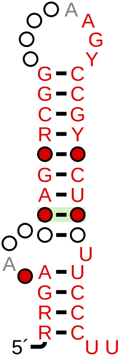

HMP2_7843_1

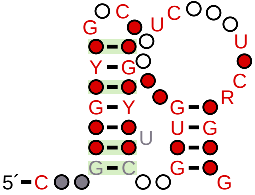

HMP2_7847_1

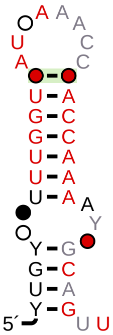

HMP2_7855_1

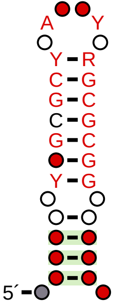

HMP2_7858_1

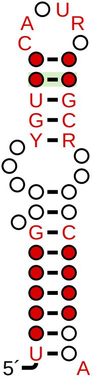

HMP2_7860_1

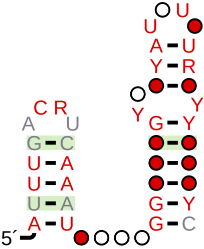

HMP2_7861_1

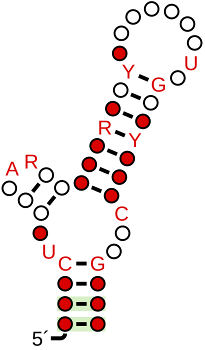

HMP2_7865_1

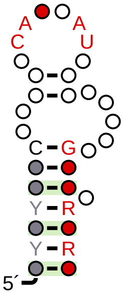

HMP2_7868_1

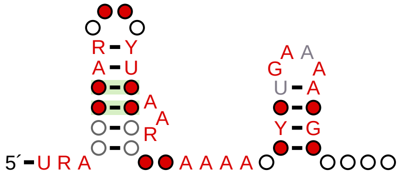

HMP2_7876_1

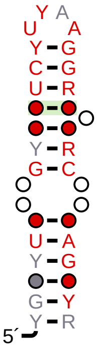

HMP2_7878_1

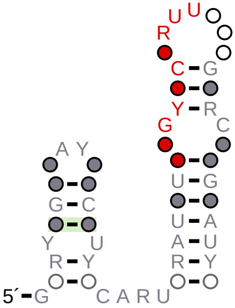

HMP2_7897_1

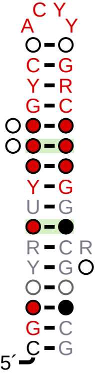

HMP2_7899_1

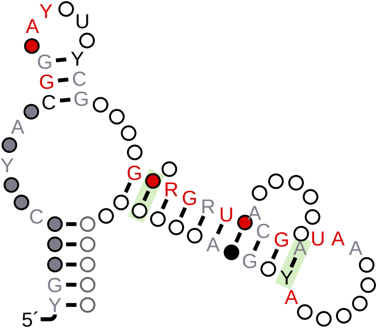

HMP2_7900_1

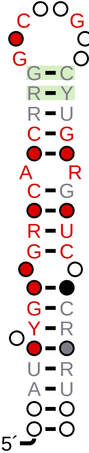

HMP2_7906_1

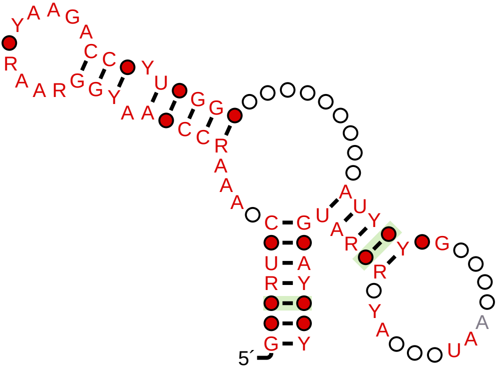

HMP2_7908_1

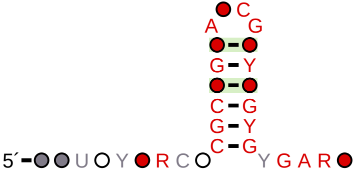

HMP2_7909_1

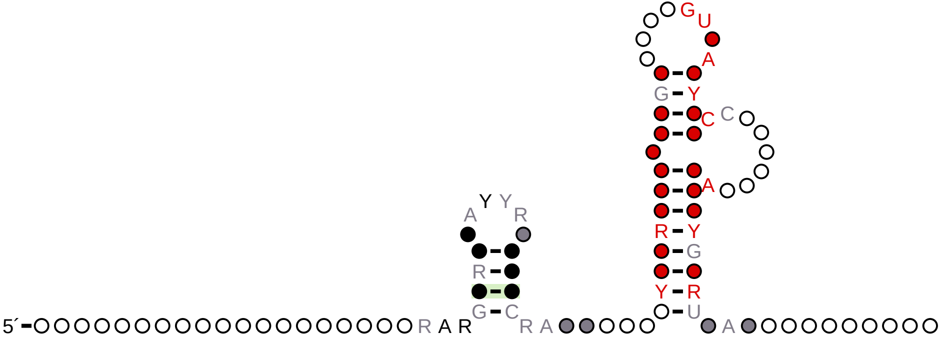

HMP2_7911_1

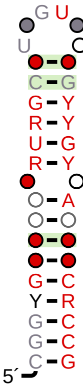

HMP2_7940_1

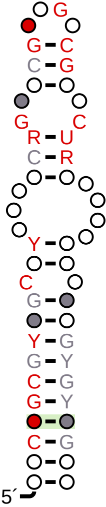

HMP2_7953_1

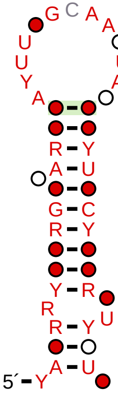

HMP2_7956_1

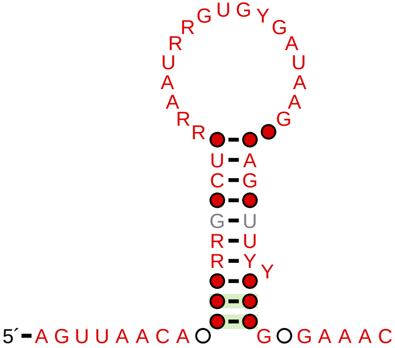

HMP2_7959_1

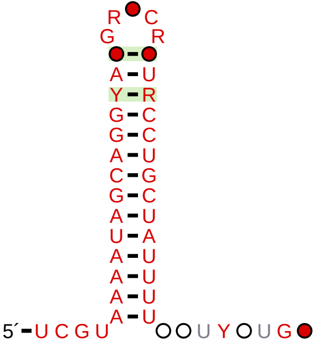

HMP2_7963_1

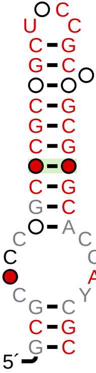

HMP2_7966_1

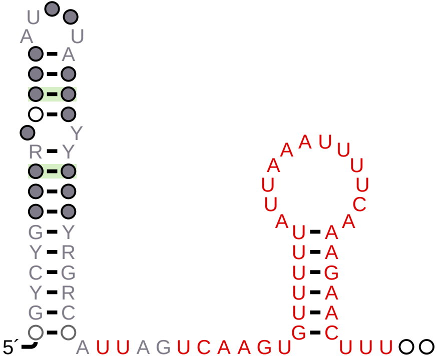

HMP2_7981_1

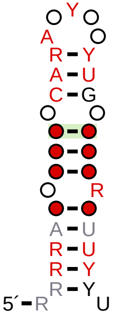

HMP2_7985_1

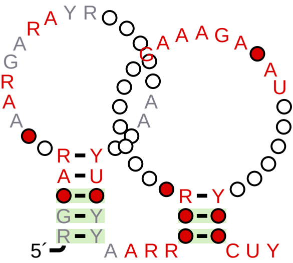

HMP2_7992_1

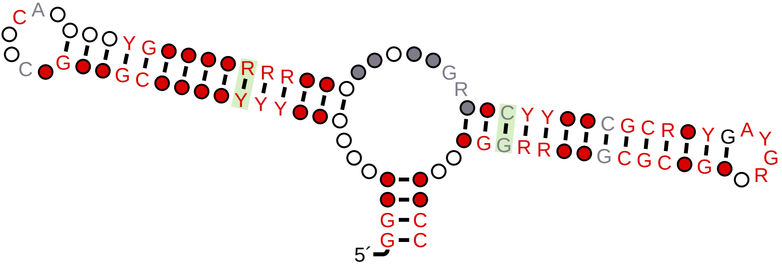

HMP2_7993_1

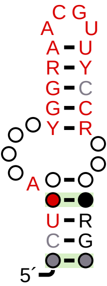

HMP2_800_1

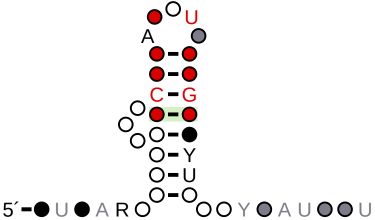

HMP2_8010_1

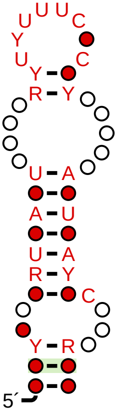

HMP2_8017_1

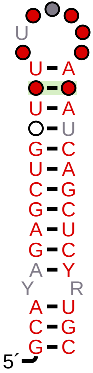

HMP2_8029_1

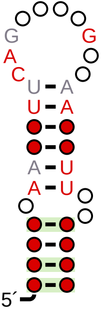

HMP2_8038_1

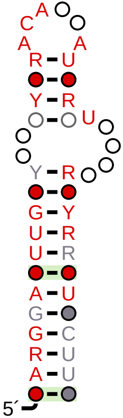

HMP2_8044_1

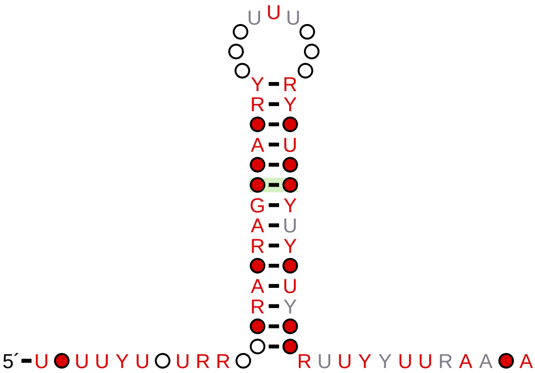

HMP2_805_1

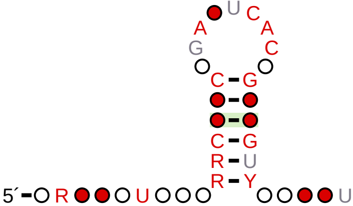

HMP2_8051_1

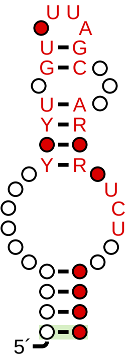

HMP2_8078_1

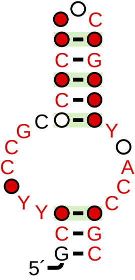

HMP2_8092_1

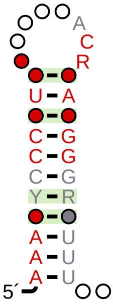

HMP2_8108_1

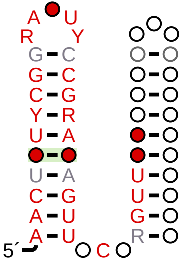

HMP2_8120_1

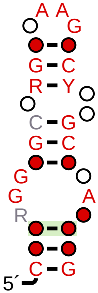

HMP2_8127_1

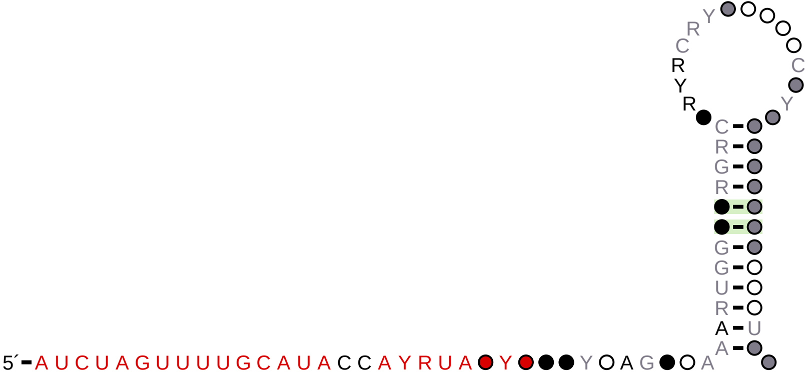

HMP2_813_1

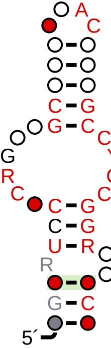

HMP2_8136_1

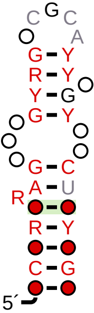

HMP2_8137_1

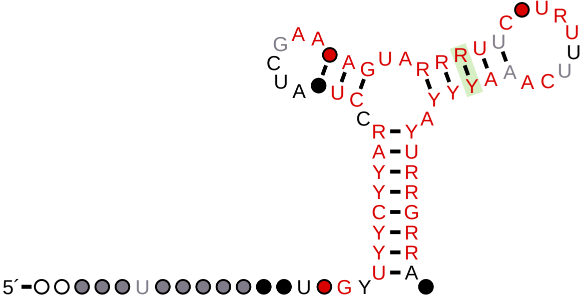

HMP2_8152_1

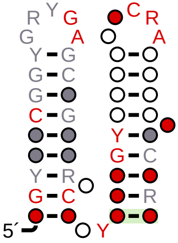

HMP2_8159_1

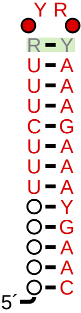

HMP2_8161_1

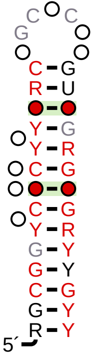

HMP2_8166_1

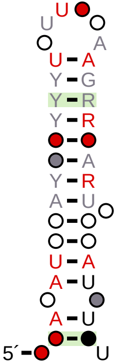

HMP2_8167_1

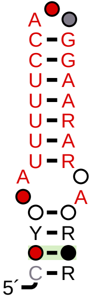

HMP2_817_1

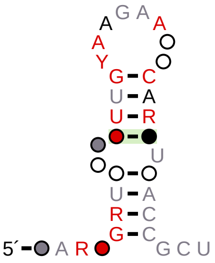

HMP2_8173_1

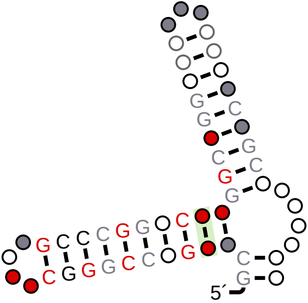

HMP2_8174_1

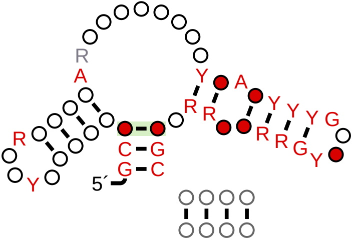

HMP2_8192_1

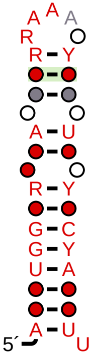

HMP2_8193_1

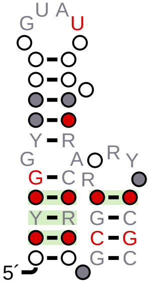

HMP2_8198_1

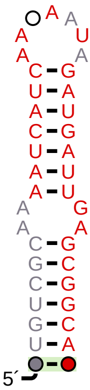

HMP2_8204_1

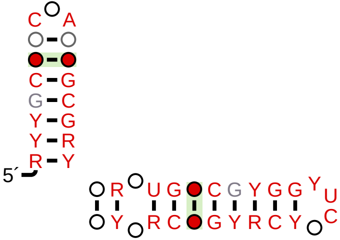

HMP2_8213_1

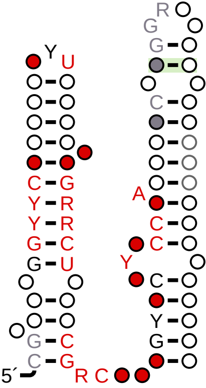

HMP2_8216_1

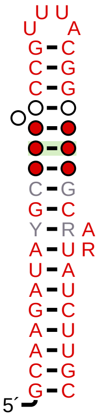

HMP2_8220_1

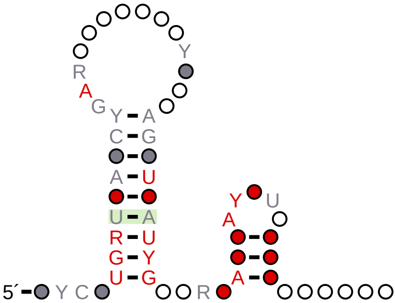

HMP2_8232_1

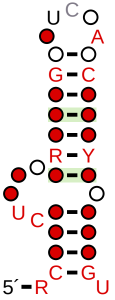

HMP2_8241_1

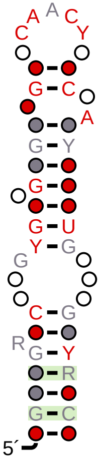

HMP2_8242_1

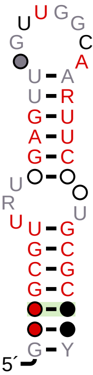

HMP2_8245_1

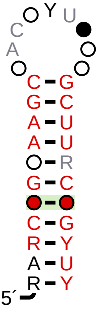

HMP2_8249_1

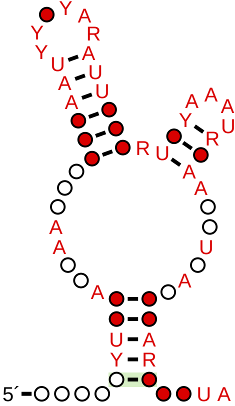

HMP2_8254_1

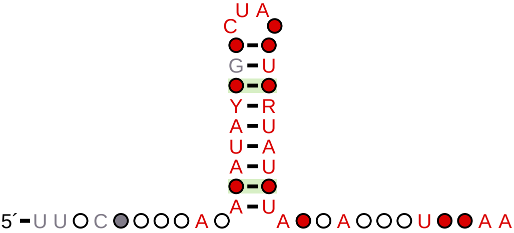

HMP2_8257_1

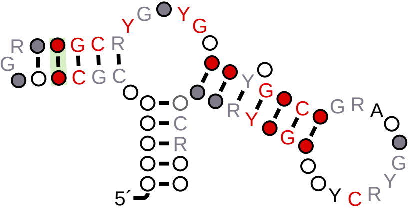

HMP2_8259_1

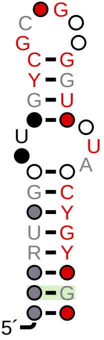

HMP2_8262_1

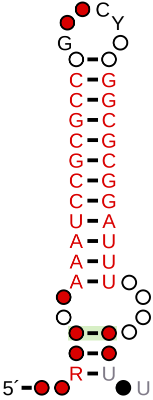

HMP2_8269_1

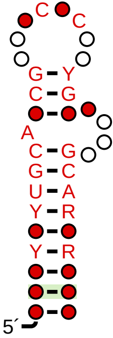

HMP2_8272_1

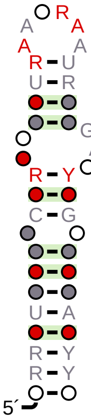

HMP2_8295_1

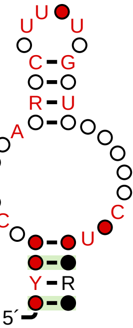

HMP2_8296_1

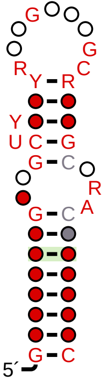

HMP2_830_1

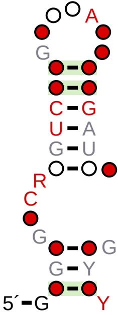

HMP2_8326_1

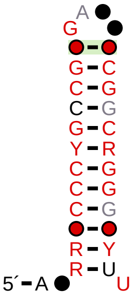

HMP2_8328_1

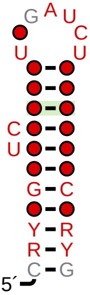

HMP2_8331_1

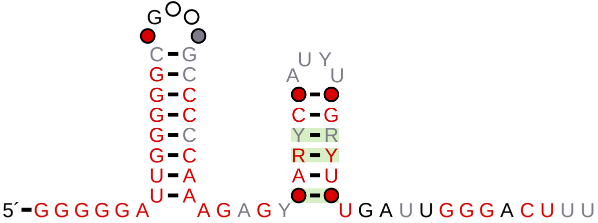

G
C G
G C
G C
G C
G C
G C
U A
U A
U Y
A U
C G
Y R
R Y
A U
5´ G G G G G A A G A G Y U G A U U G G G A C U U U

HMP2_8345_1

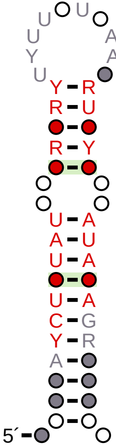

HMP2_8351_1

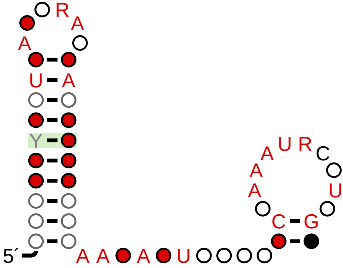

HMP2_8352_1

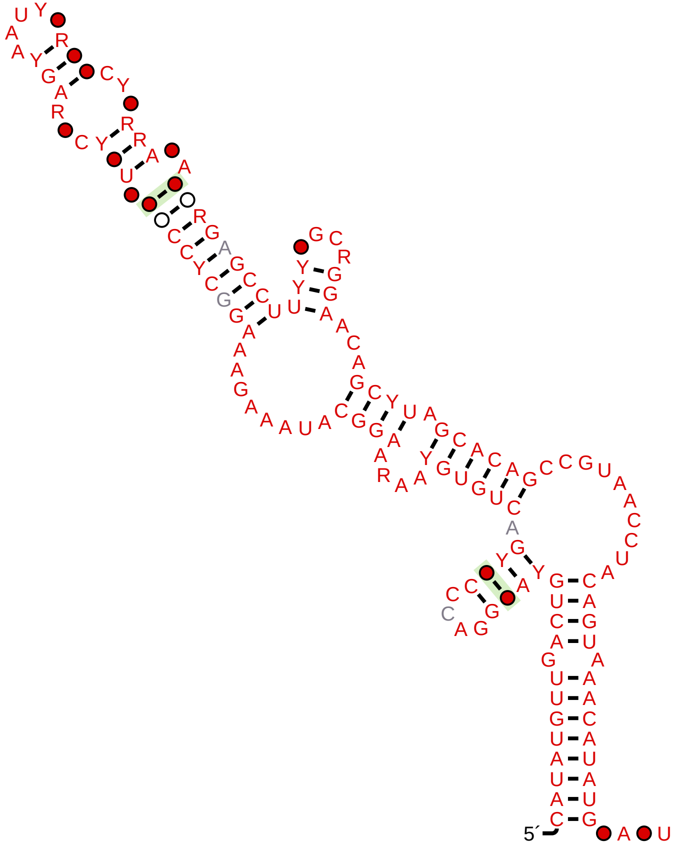

HMP2_8362_1

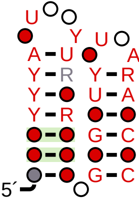

HMP2_8365_1

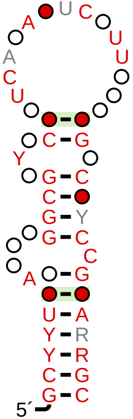

HMP2_8366_1

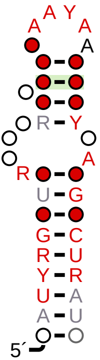

HMP2_8375_1

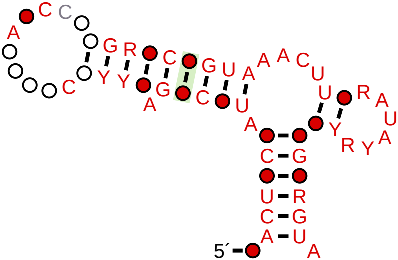

HMP2_8386_1

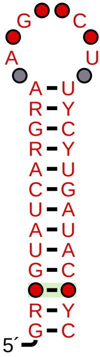

HMP2_8390_1

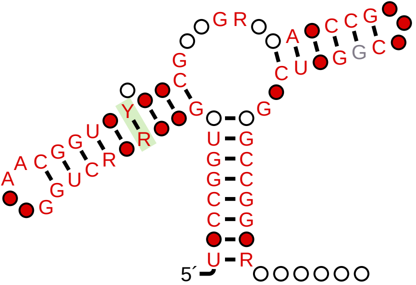

HMP2_8397_1

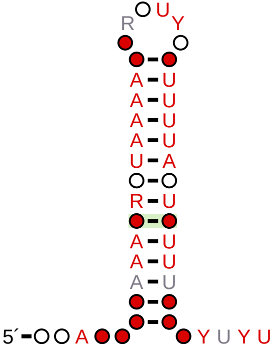

HMP2_84_1

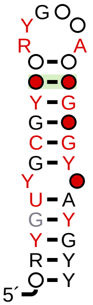

HMP2_8401_1

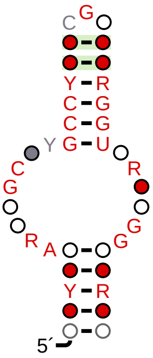

HMP2_8403_1

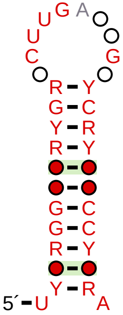

HMP2_8405_1

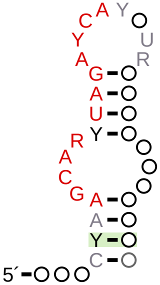

HMP2_8409_1

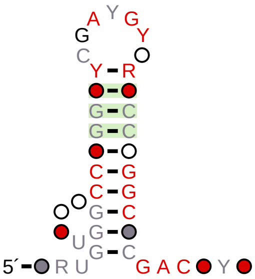

HMP2_8412_1

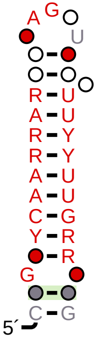

HMP2_8417_1

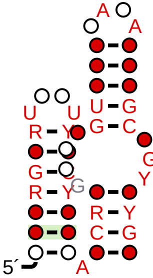

HMP2_8435_1

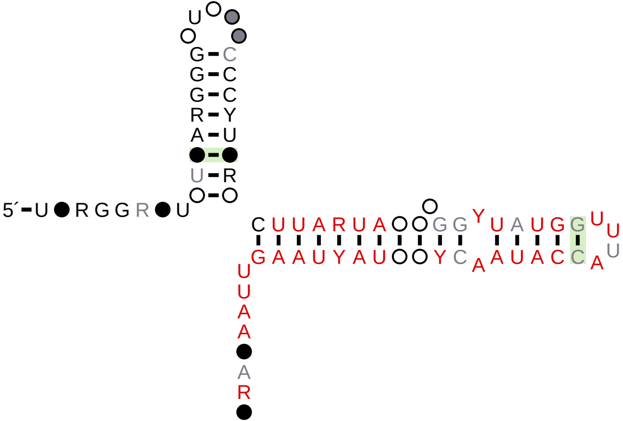

5´
U
R G G R
U
G C
G C
G C
R Y
A U
U R
C U U A R U A
G G
Y
U A U G G
U
U
U
G A A U Y A U
Y C
A
A U A C C
A
U
U
A
A
A
R

HMP2_8437_1

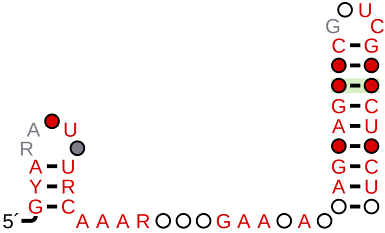

HMP2_844_1

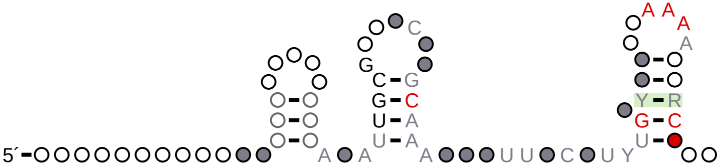

HMP2_8470_1

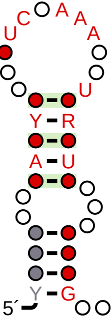

HMP2_8472_1

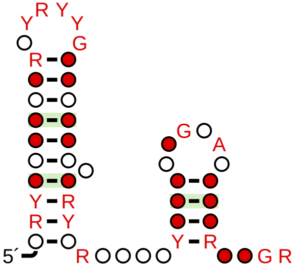

HMP2_8473_1

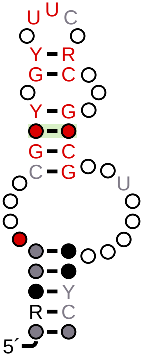

HMP2_8495_1

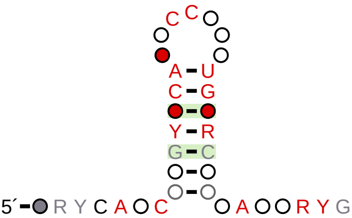

HMP2_8502_1

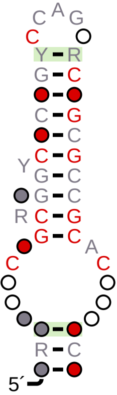

HMP2_8508_1

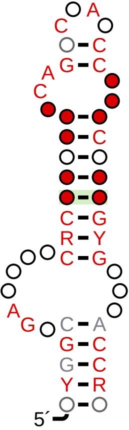

HMP2_8509_1

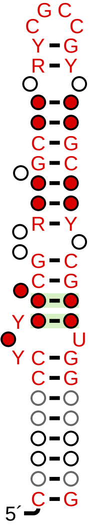

HMP2_8510_1

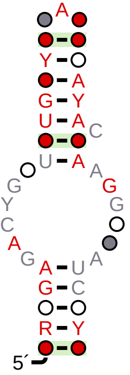

HMP2_8516_1

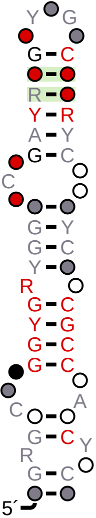

HMP2_8519_1

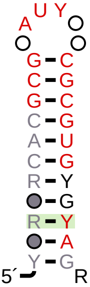

HMP2_852_1

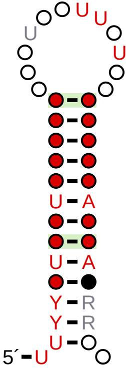

HMP2_8528_1

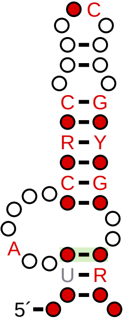

HMP2_8530_1

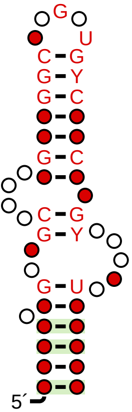

HMP2_8532_1

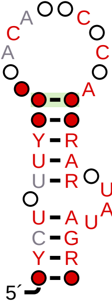

HMP2_8534_1

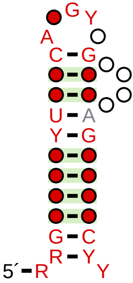

HMP2_8542_1

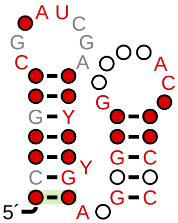

HMP2_8546_1

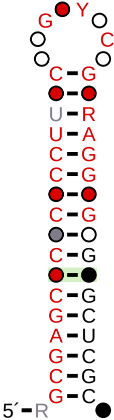

HMP2_8553_1

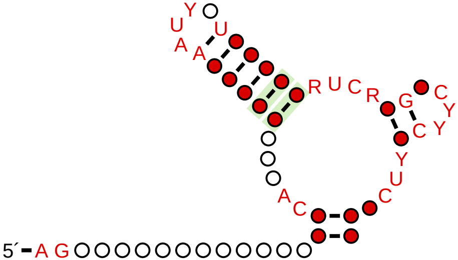

HMP2_8554_1

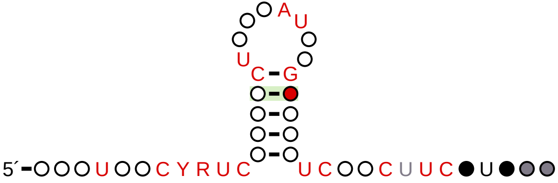

HMP2_8567_1

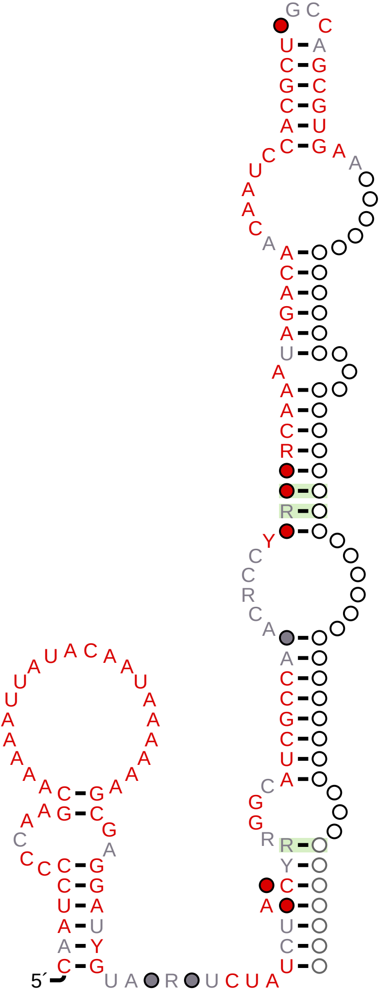

HMP2_8568_1

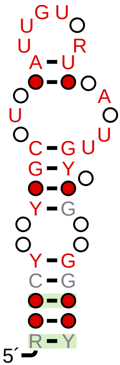

HMP2_8570_1

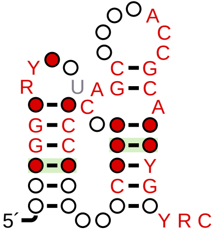

HMP2_8572_1

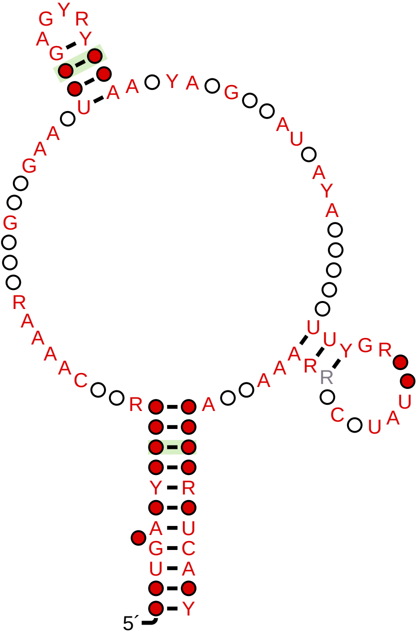

HMP2_8580_1

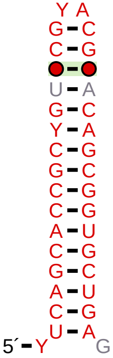

HMP2_8582_1

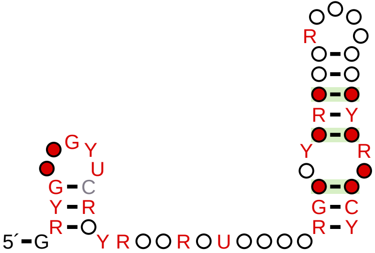

HMP2_8589_1

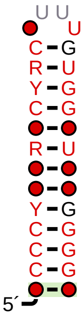

HMP2_8626_1

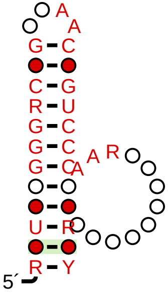

HMP2_8630_1

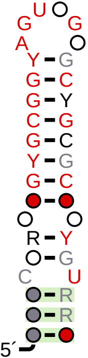

HMP2_8639_1

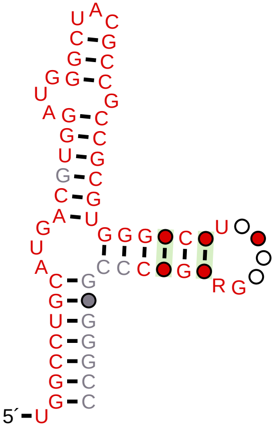

HMP2_864_1

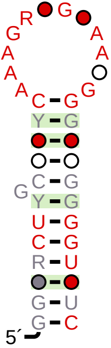

HMP2_8643_1

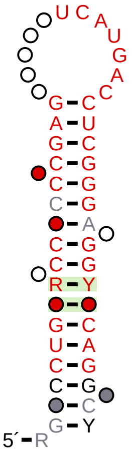

HMP2_8659_1

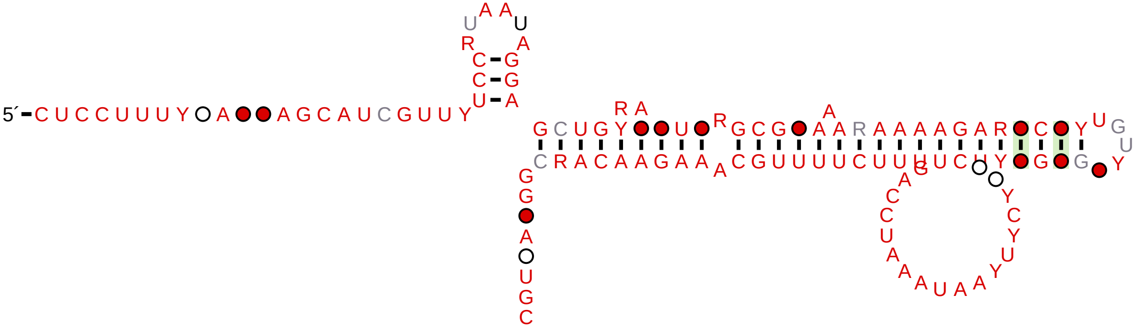

HMP2_8662_1

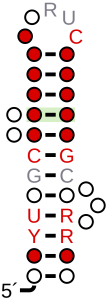

HMP2_8665_1

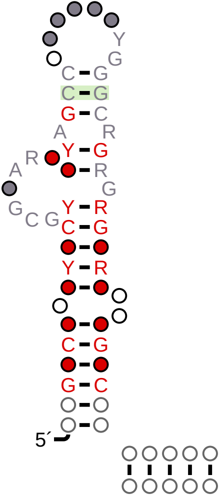

HMP2_8682_1

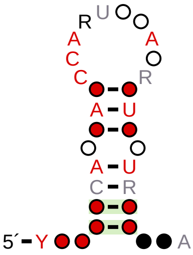

HMP2_8685_1

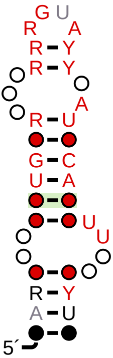

HMP2_8688_1

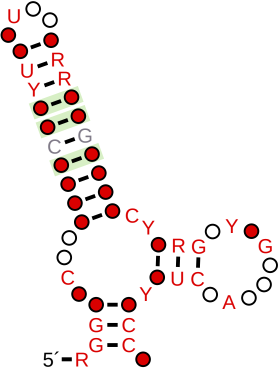

HMP2_8689_1

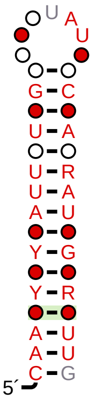

HMP2_8702_1

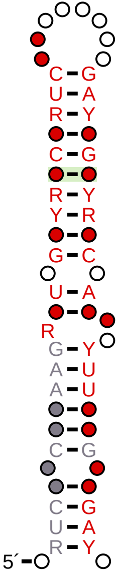

HMP2_8712_1

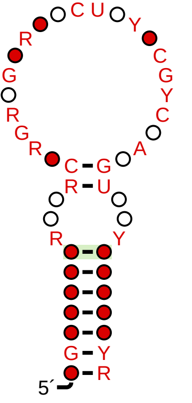

HMP2_8714_1

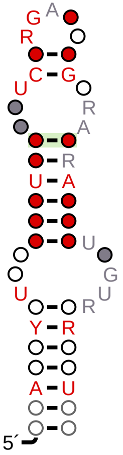

HMP2_8715_1

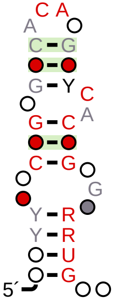

HMP2_8717_1

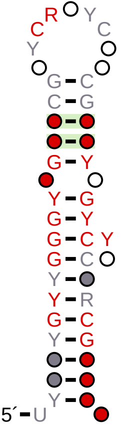

HMP2_8723_1

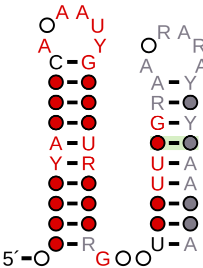

HMP2_8735_1

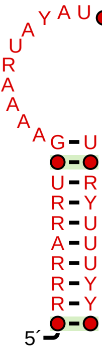

HMP2_8738_1

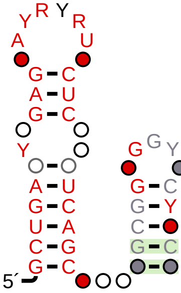

HMP2_874_1

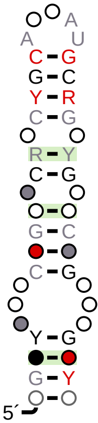

HMP2_8742_1

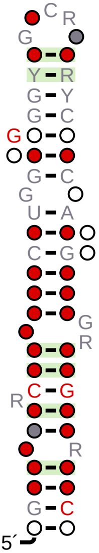

HMP2_8746_1

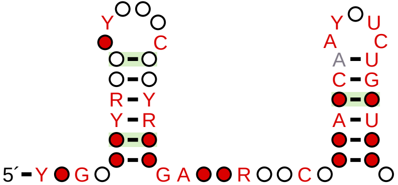

HMP2_8763_1

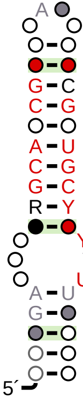

HMP2_8766_1

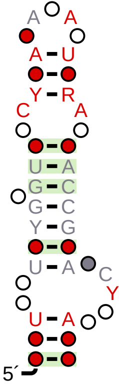

HMP2_8769_1

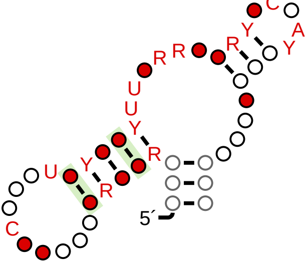

HMP2_8773_1

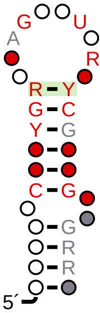

HMP2_8780_1

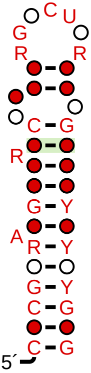

HMP2_8788_1

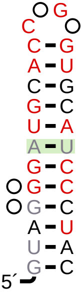

HMP2_8790_1

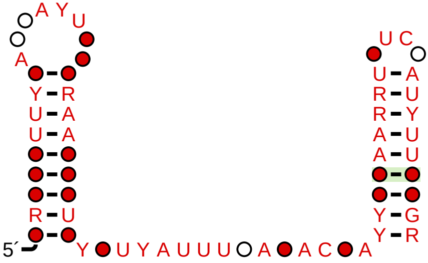

HMP2_8791_1

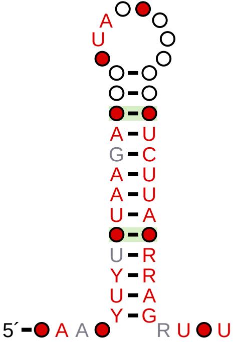

HMP2_8794_1

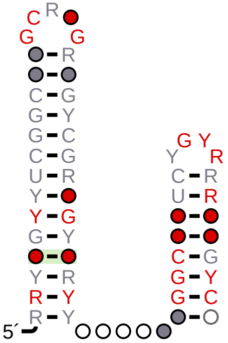

HMP2_8798_1

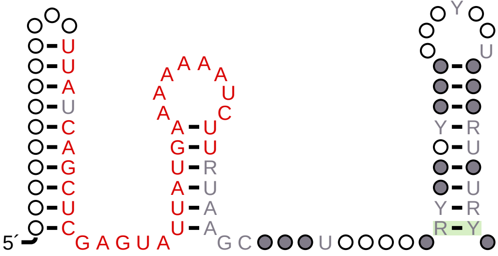

HMP2_8799_1

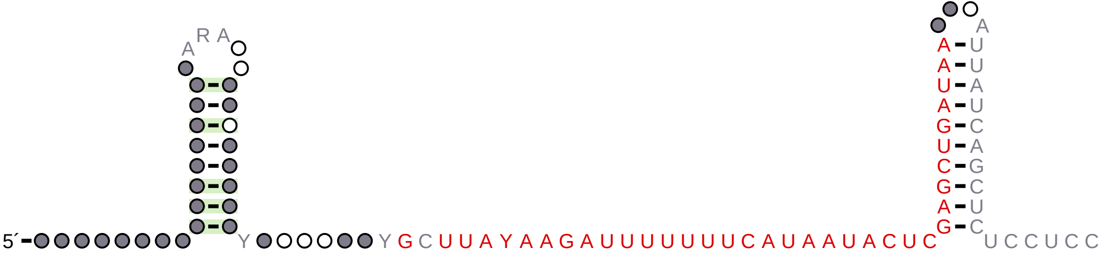
5´
A
R A
Y
Y G C U U A Y A A G A U U U U U U U C A U A A U A C U C
A A U A G U C G A G
A
U U A U C A G C U C
U C C U C C

HMP2_8821_1

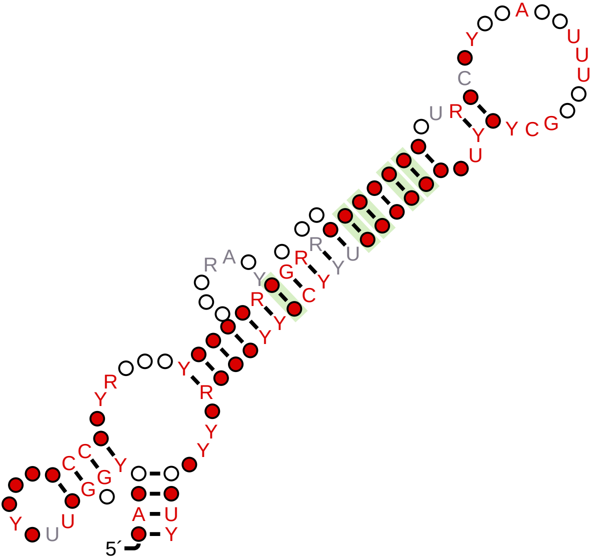

HMP2_8831_1

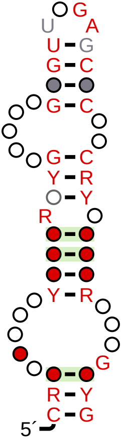

HMP2_8835_1

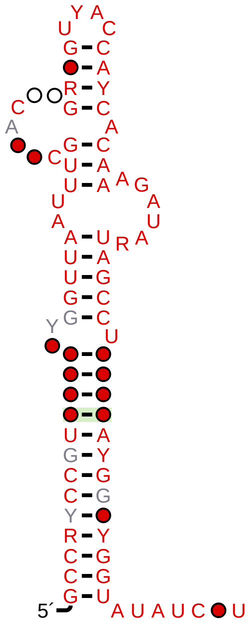

HMP2_8842_1

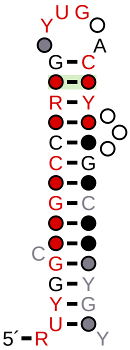

HMP2_8846_1

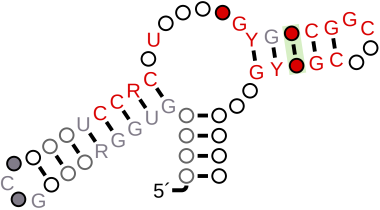

HMP2_8854_1

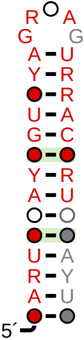

HMP2_886_1

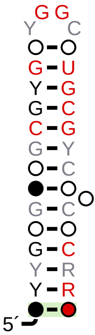

HMP2_8888_1

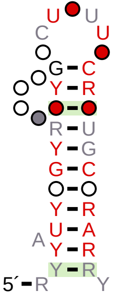

HMP2_8889_1

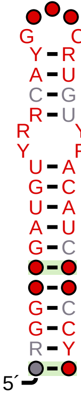

HMP2_8899_1

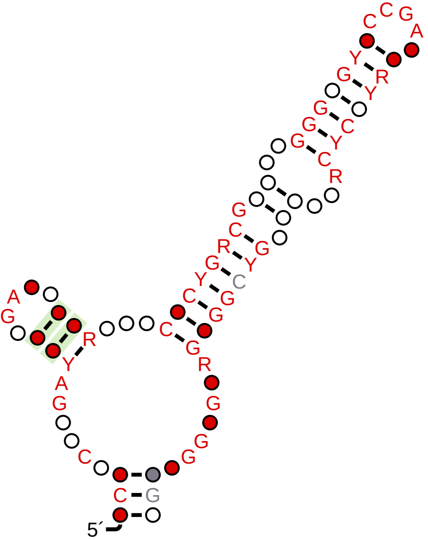

HMP2_8901_1

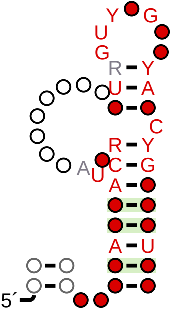

HMP2_8910_1

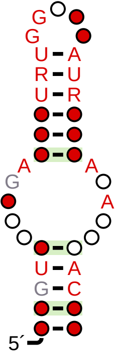

HMP2_8915_1

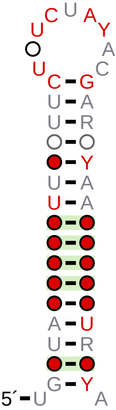

HMP2_8924_1

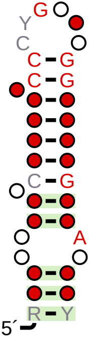

HMP2_894_1

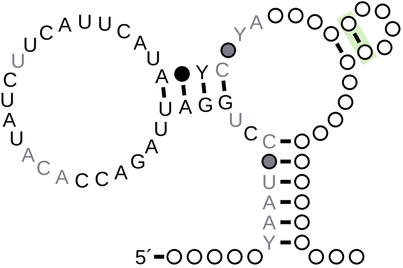

HMP2_8955_1

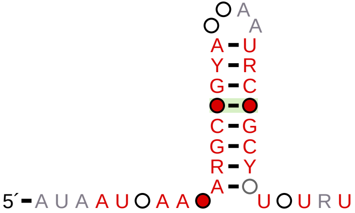

HMP2_8956_1

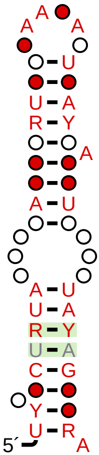

HMP2_8960_1

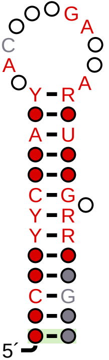

HMP2_8968_1

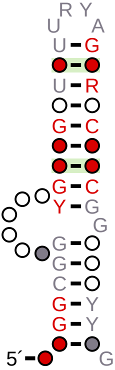

HMP2_8973_1

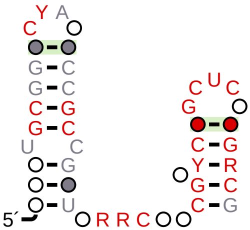

HMP2_8976_1

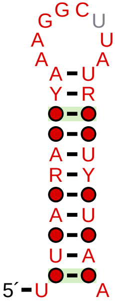

HMP2_8978_1

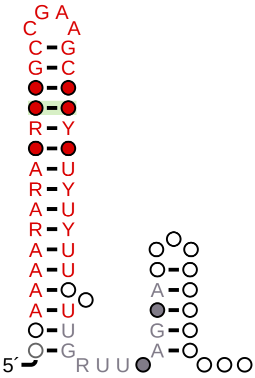

HMP2_8980_1

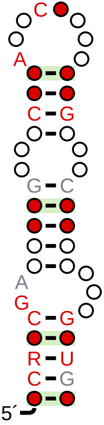

HMP2_8982_1

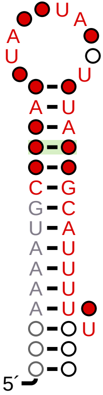

HMP2_8983_1

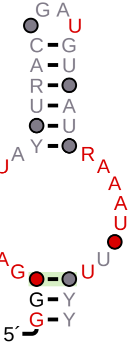

HMP2_8990_1

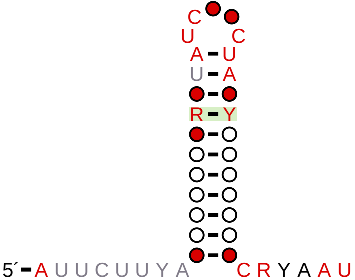

HMP2_8994_1

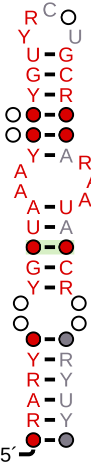

HMP2_9000_1

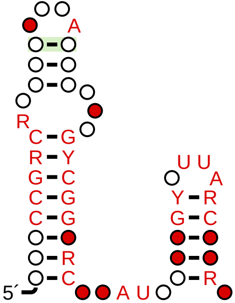

HMP2_9005_1

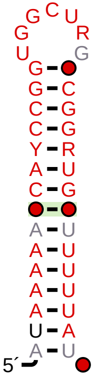

HMP2_9006_1

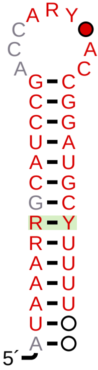

HMP2_9008_1

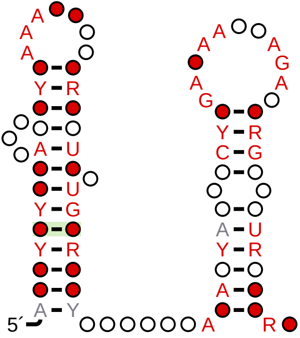

HMP2_9015_1

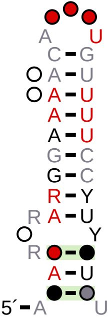

HMP2_9022_1

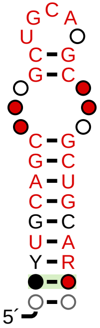

HMP2_9036_1

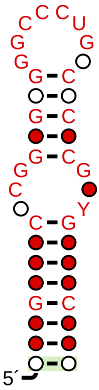

HMP2_9037_1

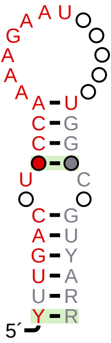

HMP2_9038_1

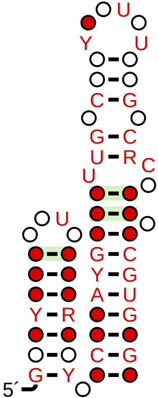

HMP2_9039_1

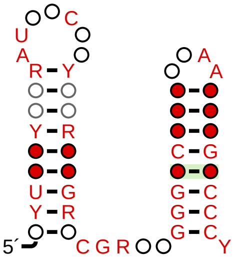

HMP2_9043_1

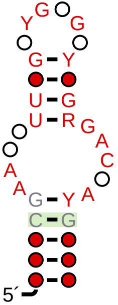

HMP2_9045_1

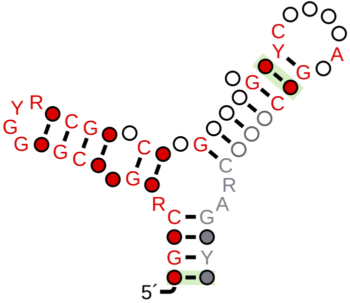

HMP2_9052_1

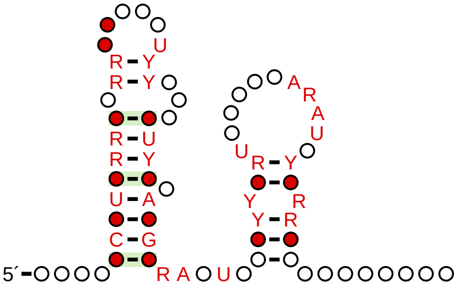

HMP2_9071_1

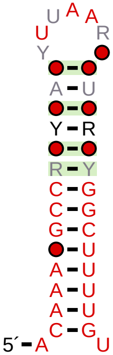

HMP2_9073_1

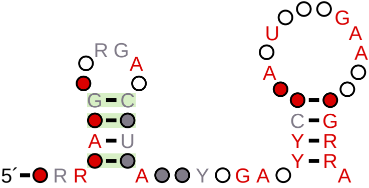

HMP2_9083_1

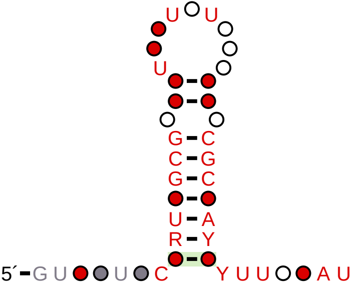

HMP2_9097_1

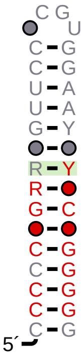

HMP2_9103_1

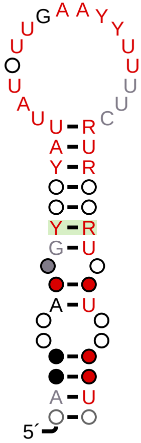

HMP2_9105_1

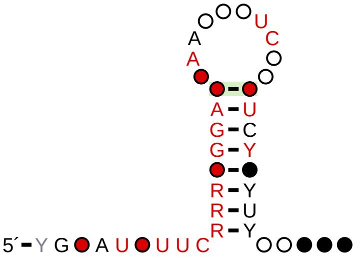

HMP2_911_1

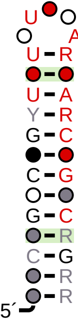

HMP2_9110_1

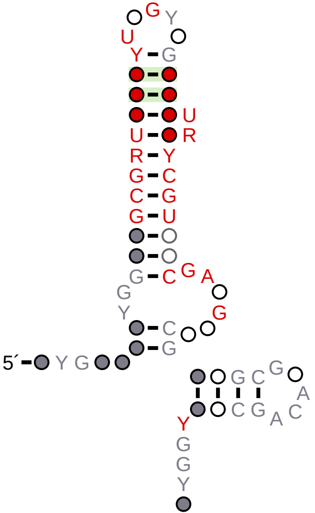

HMP2_9111_1

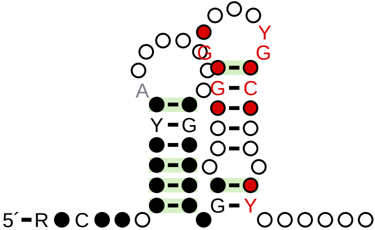

HMP2_9117_1

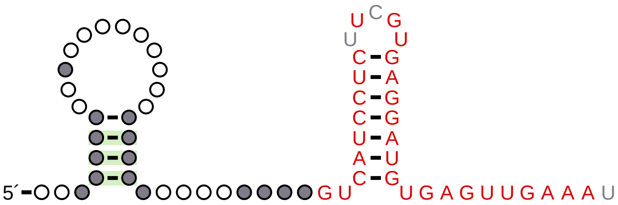

HMP2_9146_1

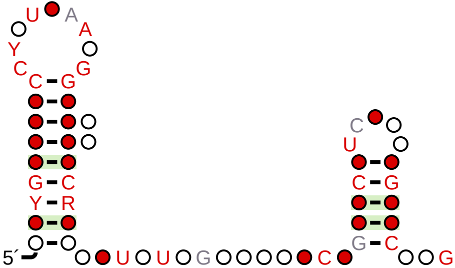

HMP2_9175_1

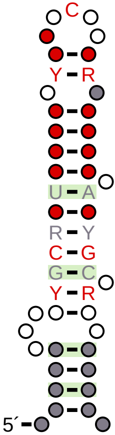

HMP2_9179_1

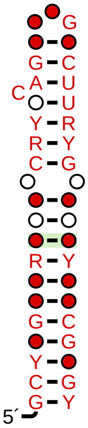

HMP2_918_1

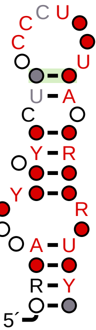

HMP2_9195_1

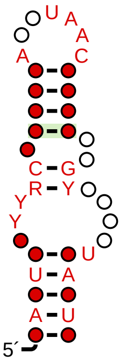

HMP2_9218_1

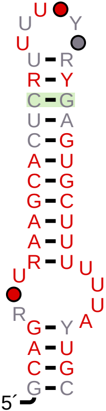

HMP2_9219_1

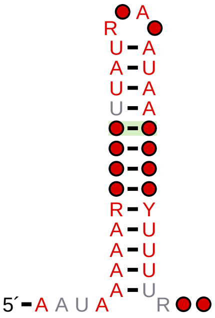

HMP2_9233_1

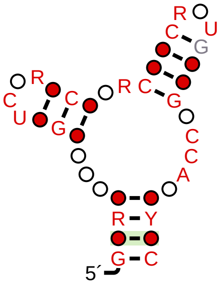

HMP2_9237_1

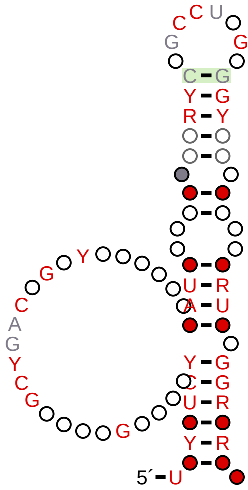

HMP2_926_1

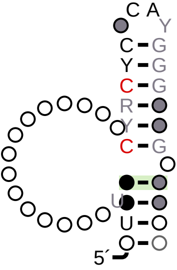

HMP2_9270_1

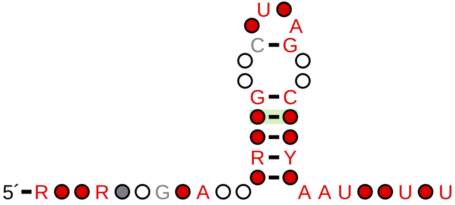

HMP2_9277_1

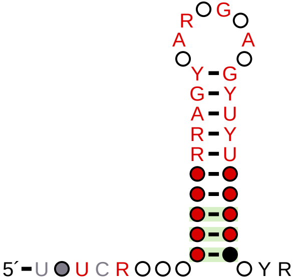

HMP2_9291_1

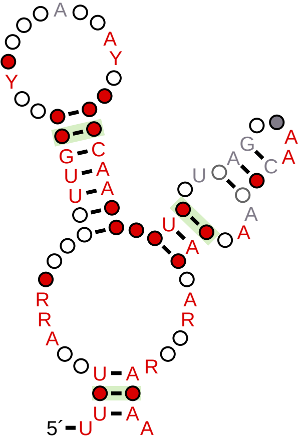

HMP2_9300_1

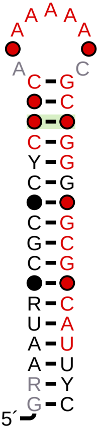

HMP2_9302_1

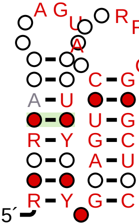

HMP2_9317_1

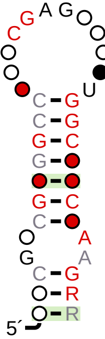

HMP2_9325_1

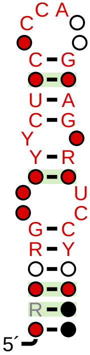

HMP2_9375_1

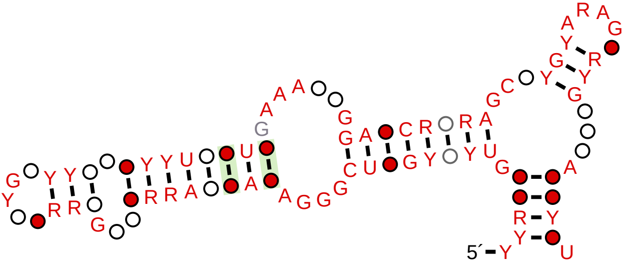

HMP2_9381_1

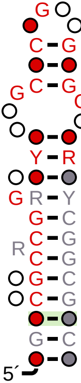

HMP2_9406_1

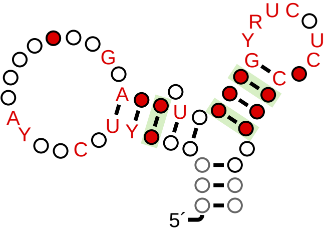

HMP2_9423_1

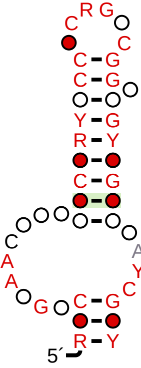

HMP2_9427_1

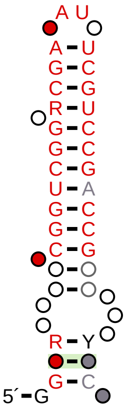

HMP2_9430_1

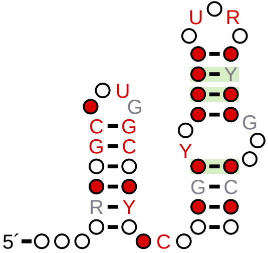

HMP2_9435_1

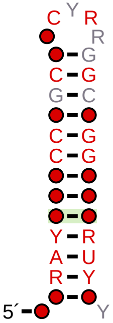

HMP2_9437_1

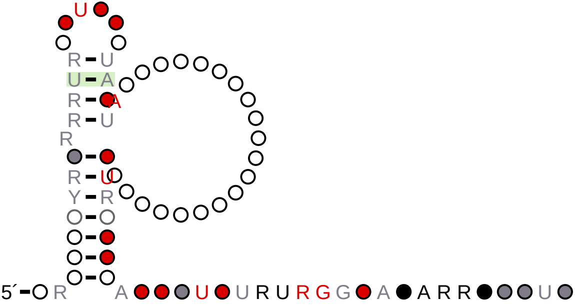

HMP2_944_1

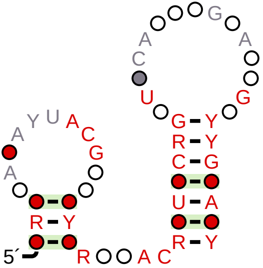

HMP2_9445_1

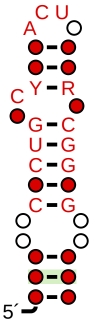

HMP2_945_1

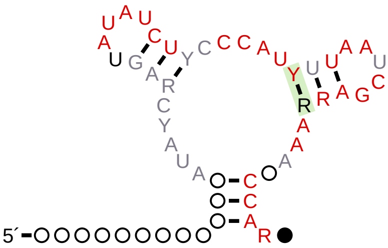

HMP2_9460_1

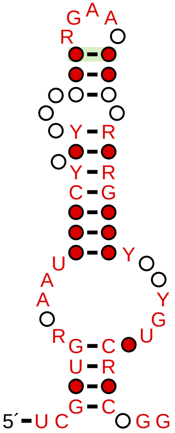

HMP2_9470_1

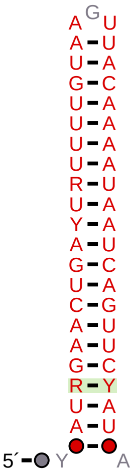

HMP2_9471_1

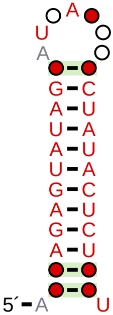

HMP2_948_1

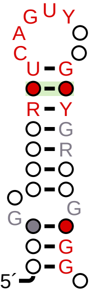

HMP2_9482_1

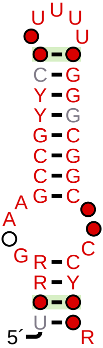

HMP2_9497_1

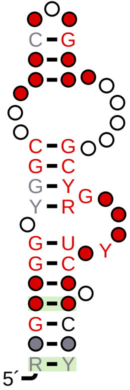

HMP2_950_1

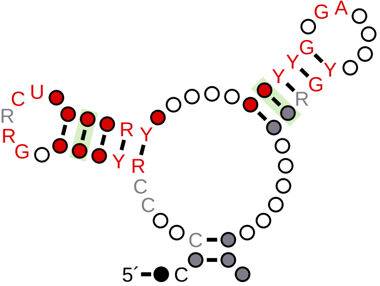

HMP2_9504_1

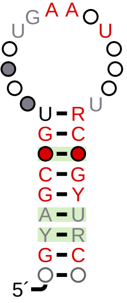

HMP2_9513_1

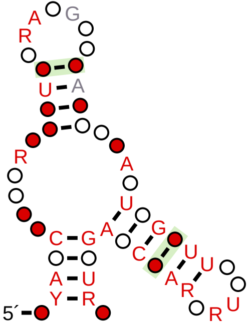

HMP2_9527_1

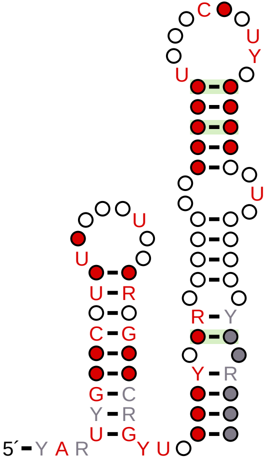

HMP2_9542_1

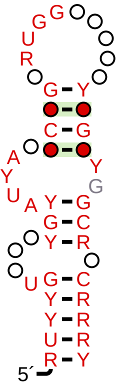

HMP2_9545_1

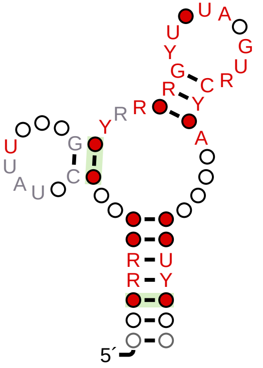

HMP2_9557_1

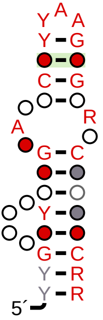

HMP2_9564_1

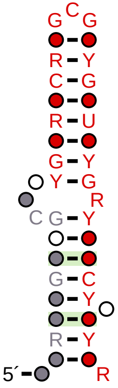

HMP2_9569_1

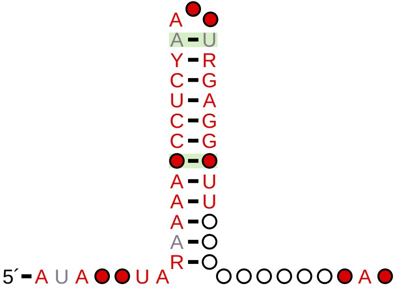

HMP2_9577_1

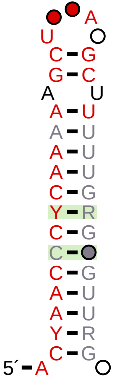

HMP2_9580_1

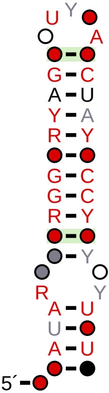

HMP2_9581_1

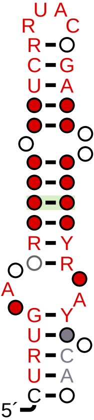

HMP2_9584_1

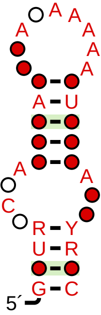

HMP2_9586_1

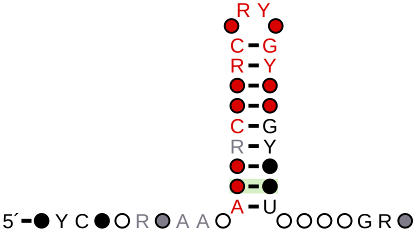

HMP2_9595_1

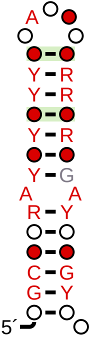

HMP2_9601_1

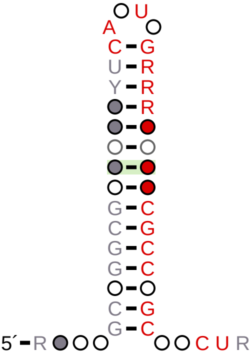

HMP2_9611_1

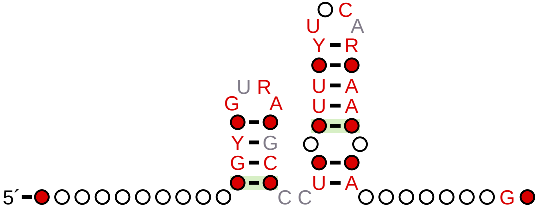

HMP2_9623_1

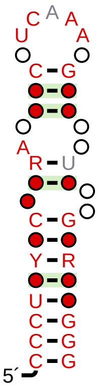

HMP2_9628_1

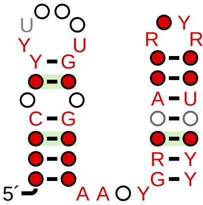

HMP2_9631_1

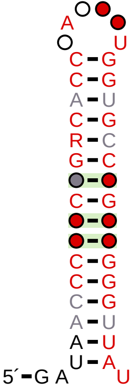

HMP2_9649_1

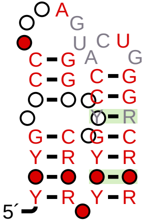

HMP2_9663_1

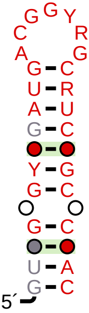

HMP2_9667_1

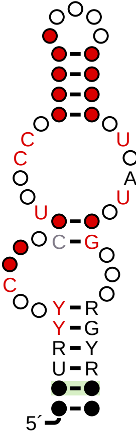

HMP2_9676_1

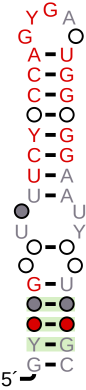

HMP2_9678_1

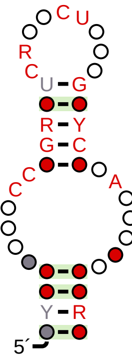

HMP2_9684_1

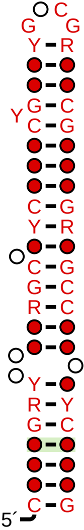

HMP2_9714_1

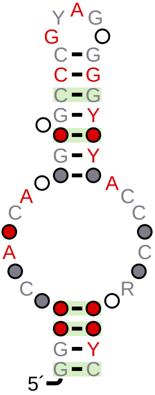

HMP2_9724_1

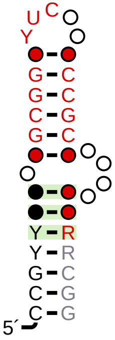

HMP2_9730_1

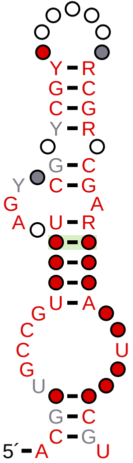

HMP2_9732_1

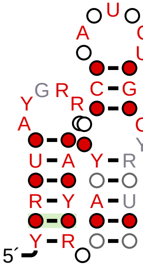

HMP2_974_1

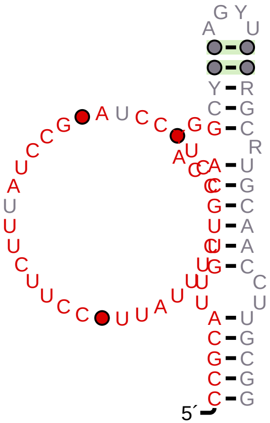

HMP2_9743_1

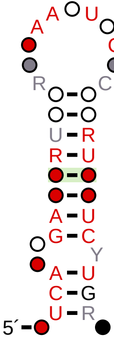

HMP2_9745_1

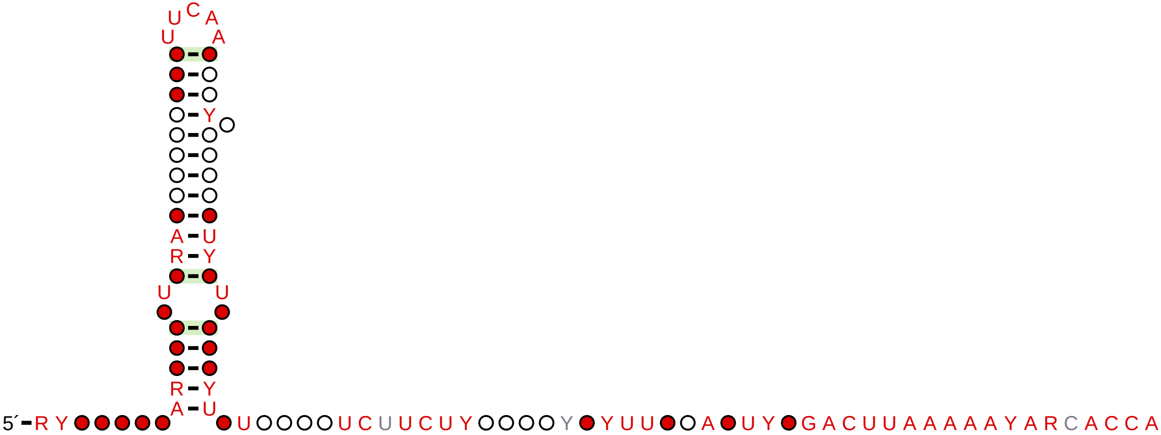
U C A
U A
Y
A U
R Y
U U
R Y
A U
5´ R Y U U C U U C U Y Y Y U U A U Y G A C U U A A A A A Y A R C A C C A

HMP2_9752_1

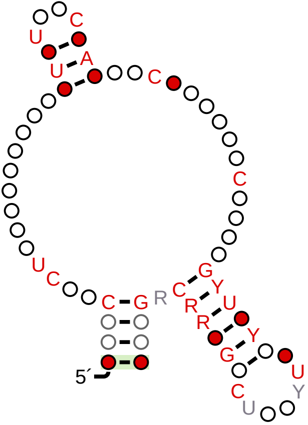

HMP2_9756_1

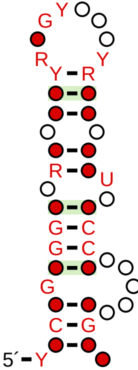

HMP2_9764_1

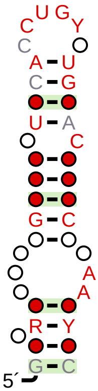

HMP2_9775_1

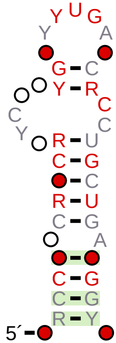

HMP2_9780_1

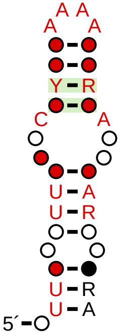

HMP2_9795_1

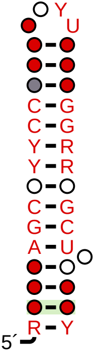

HMP2_9796_1

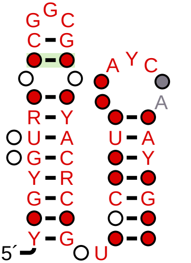

HMP2_98_1

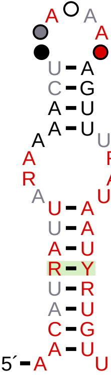

HMP2_9800_1

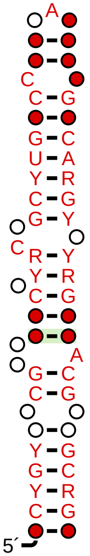

HMP2_9801_1

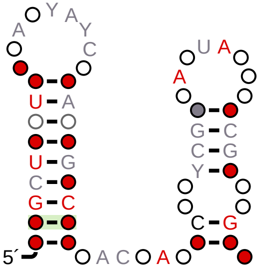

HMP2_9807_1

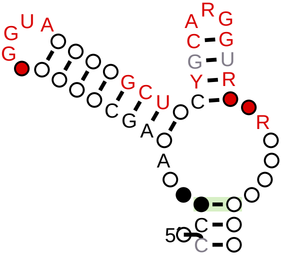

HMP2_9810_1

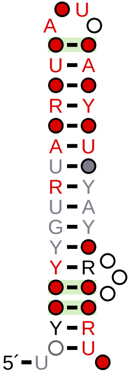

HMP2_9811_1

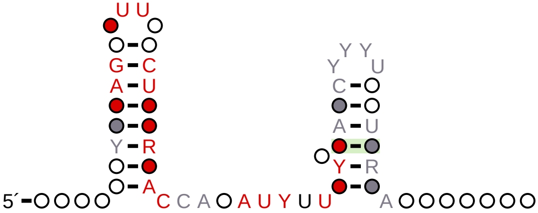

HMP2_9821_1

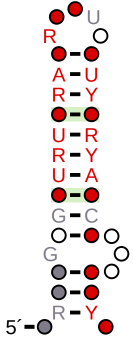

HMP2_984_1

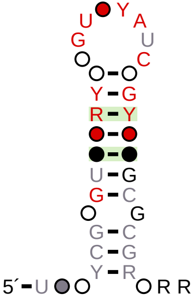

HMP2_9847_1

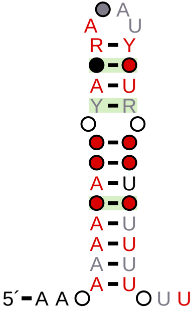

HMP2_9851_1

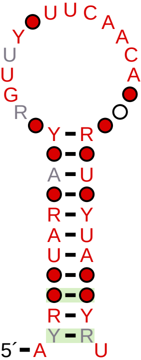

HMP2_9852_1

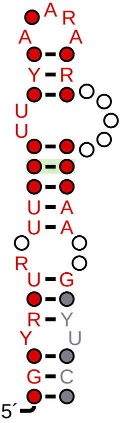

HMP2_9853_1

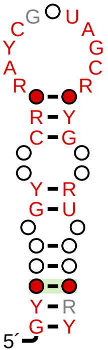

HMP2_9872_1

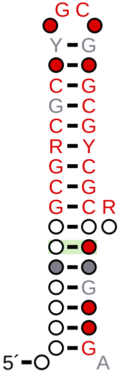

HMP2_9875_1

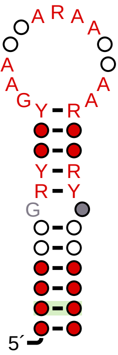

HMP2_9877_1

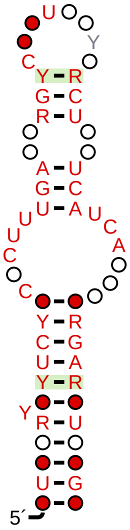

HMP2_9878_1

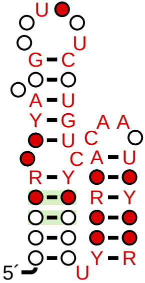

HMP2_9897_1

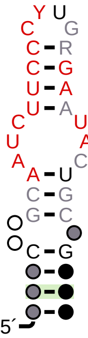

HMP2_9902_1

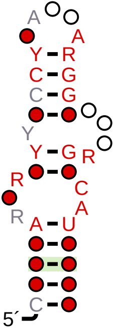

HMP2_9912_1

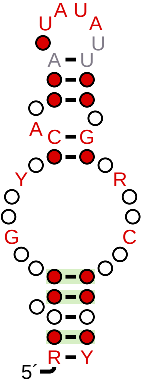

HMP2_9917_1

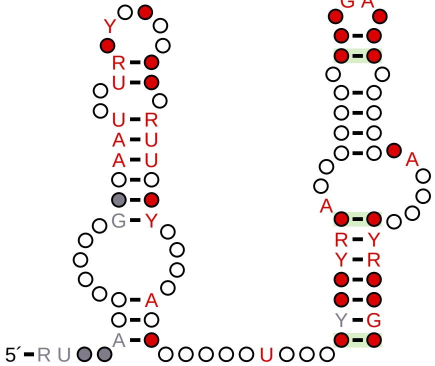

HMP2_9933_1

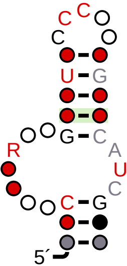

HMP2_9940_1

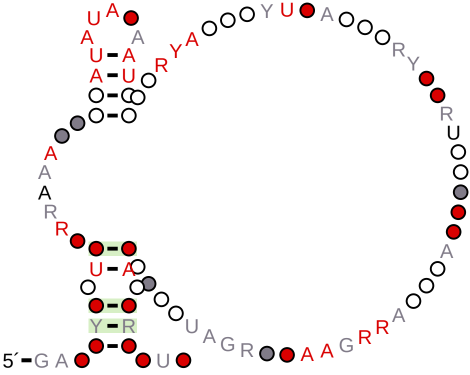

HMP2_9952_1

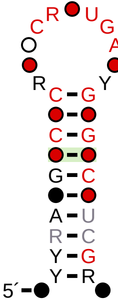

HMP2_9972_1

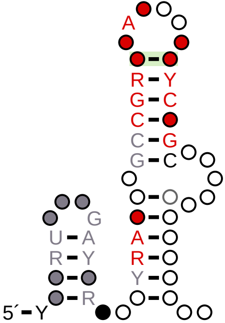

HMP2_9976_1

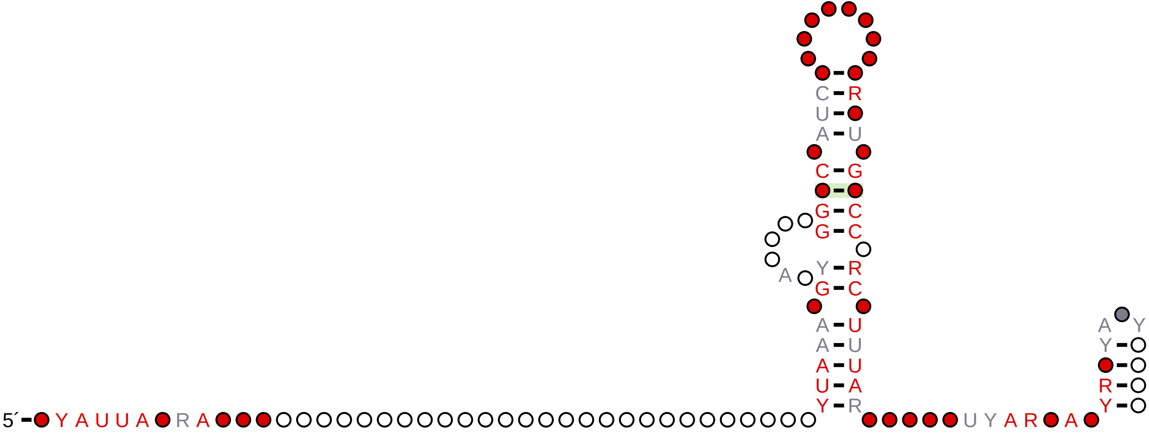

HMP2_1002_1

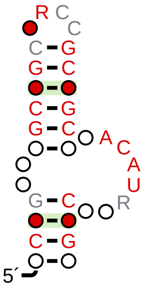

HMP2_10030_1

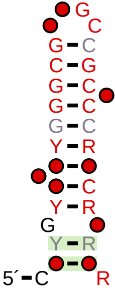

HMP2_10046_1

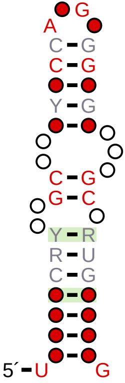

HMP2_10049_1

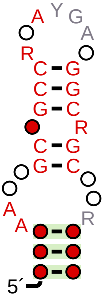

HMP2_10053_1

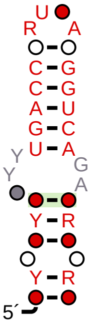

HMP2_10063_1

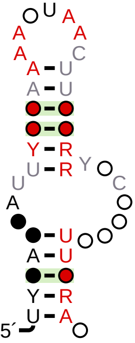

HMP2_10071_1

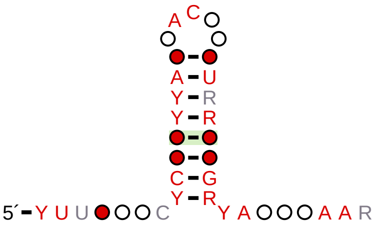

HMP2_10078_1

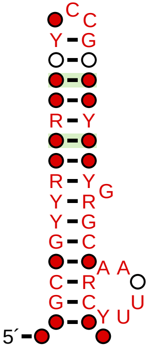

HMP2_10087_1

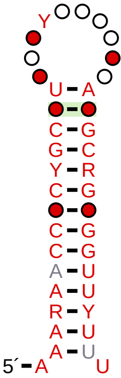

HMP2_1009_1

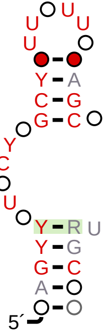

HMP2_10095_1

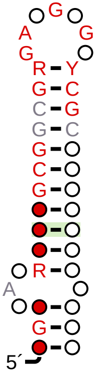

HMP2_10108_1

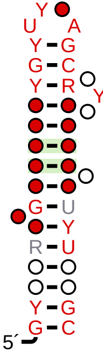

HMP2_10116_1

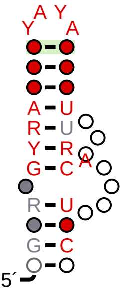

HMP2_1012_1

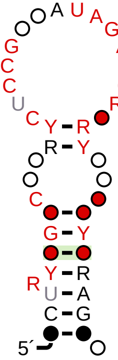

HMP2_10126_1

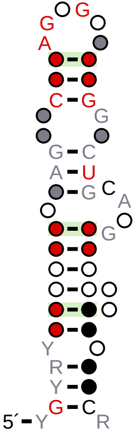

HMP2_10131_1

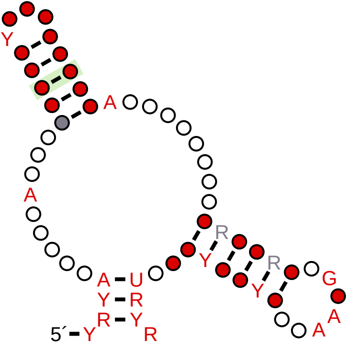

HMP2_10132_1

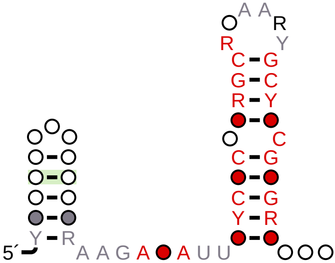

HMP2_10133_1

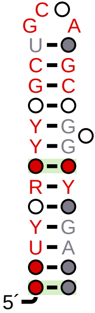

HMP2_10135_1

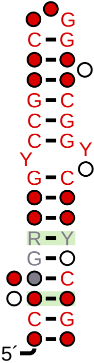

HMP2_10149_1

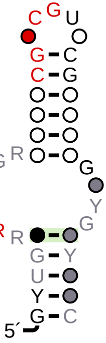

HMP2_10155_1

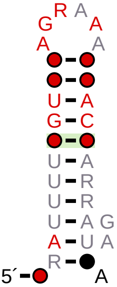

HMP2_10161_1

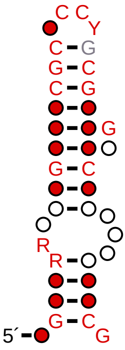

HMP2_10171_1

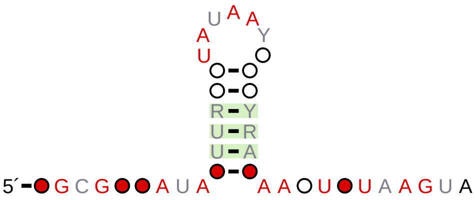

HMP2_10172_1

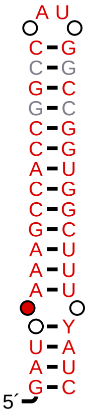

HMP2_10178_1

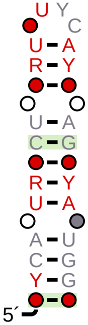

HMP2_10190_1

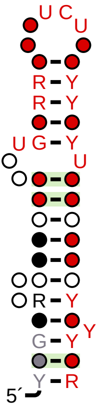

HMP2_10193_1

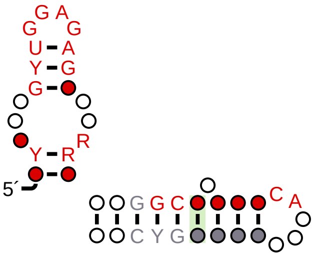

HMP2_10206_1

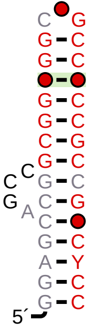

HMP2_10211_1

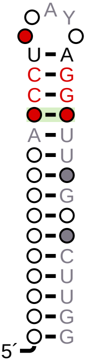

HMP2_10223_1

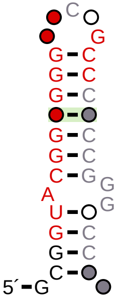

HMP2_10227_1

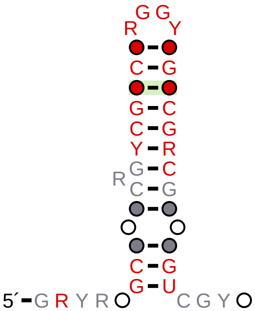

HMP2_10244_1

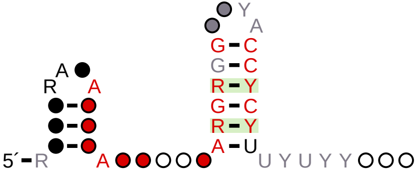

HMP2_10248_1

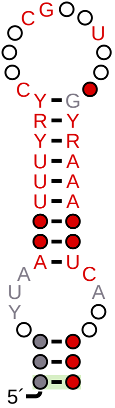

HMP2_10250_1

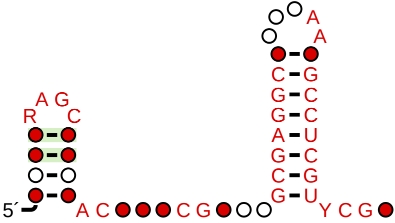

HMP2_10259_1

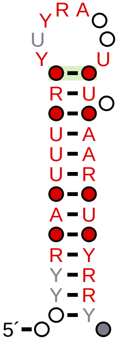

HMP2_10260_1

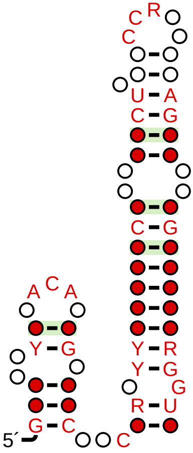

HMP2_10261_1

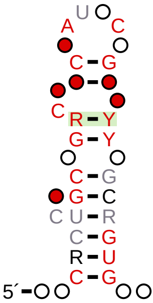

HMP2_10262_1

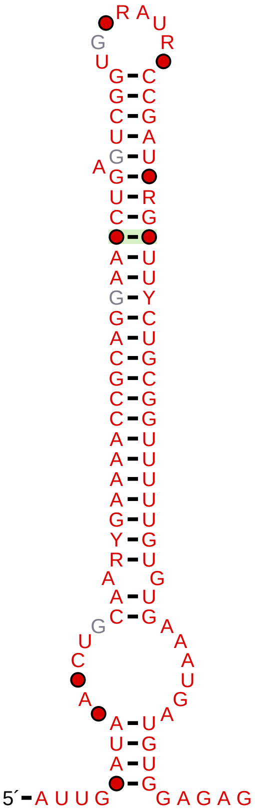

HMP2_10276_1

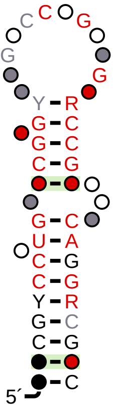

HMP2_10282_1

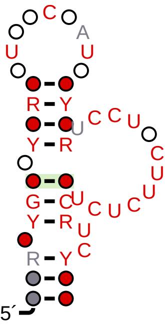

HMP2_10291_1

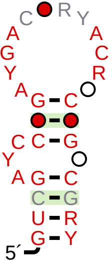

HMP2_10298_1

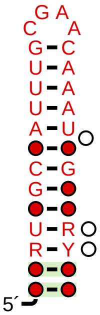

HMP2_10299_1

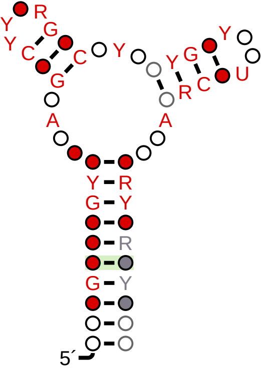

HMP2_10306_1

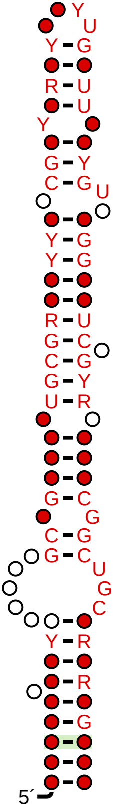

HMP2_1031_1

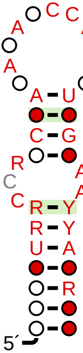

HMP2_10323_1

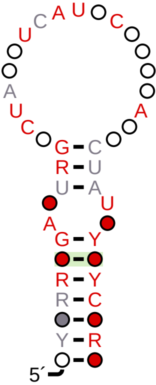

HMP2_10330_1

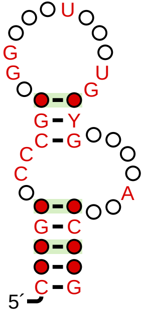

HMP2_10335_1

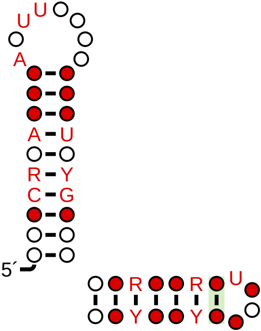

HMP2_10338_1

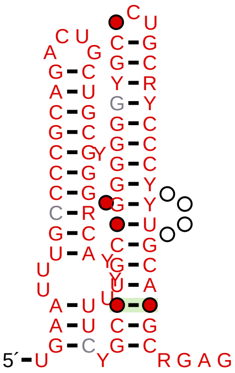

HMP2_10344_1

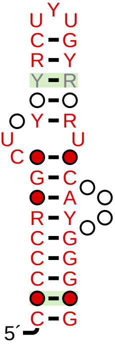

HMP2_10347_1

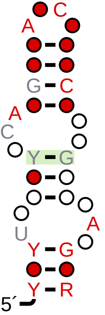

HMP2_10367_1

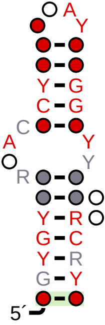

HMP2_10375_1

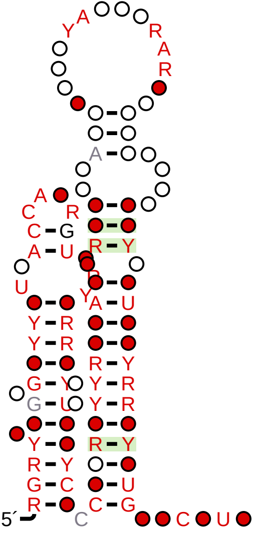

HMP2_10379_1

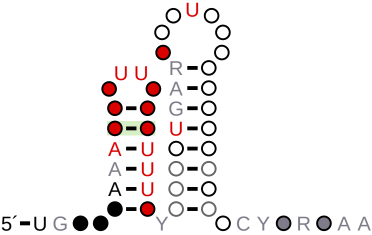

HMP2_10383_1

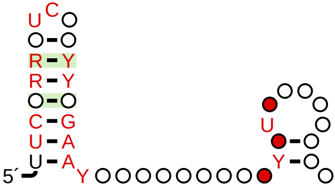

HMP2_10387_1

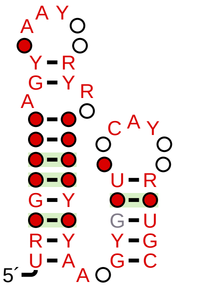

HMP2_10392_1

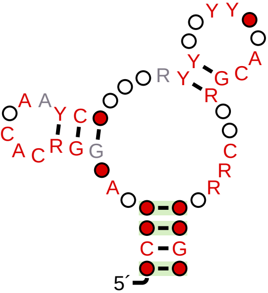

HMP2_10395_1

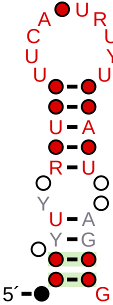

HMP2_10397_1

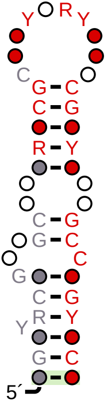

HMP2_10403_1

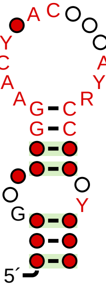

HMP2_10408_1

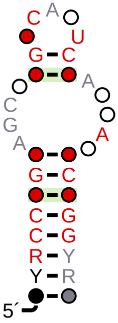

HMP2_10416_1

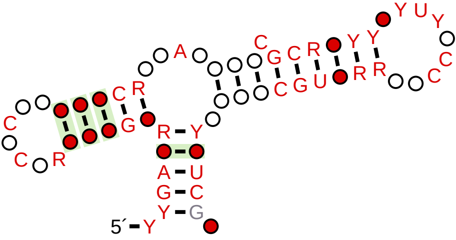

HMP2_10429_1

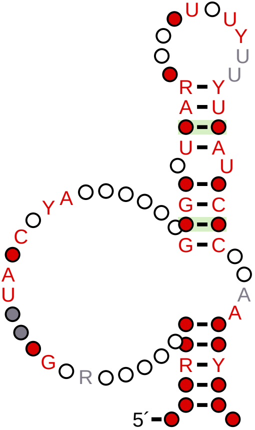

HMP2_10442_1

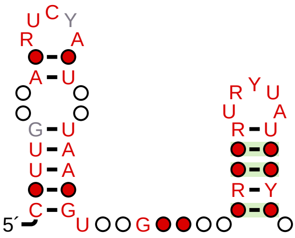

HMP2_10444_1

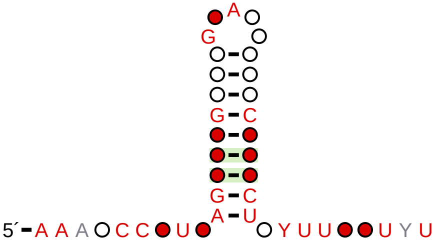

HMP2_1045_1

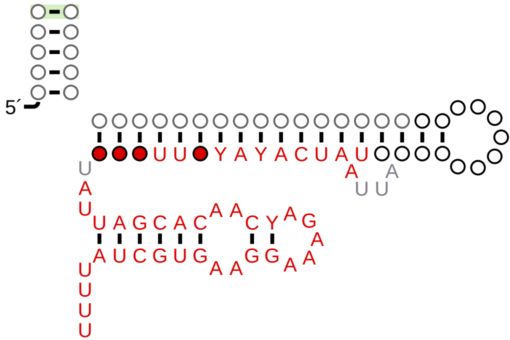

HMP2_10464_1

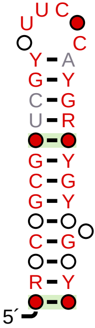

HMP2_1047_1

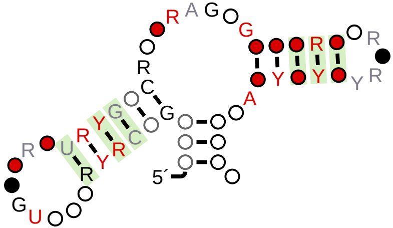

HMP2_10475_1

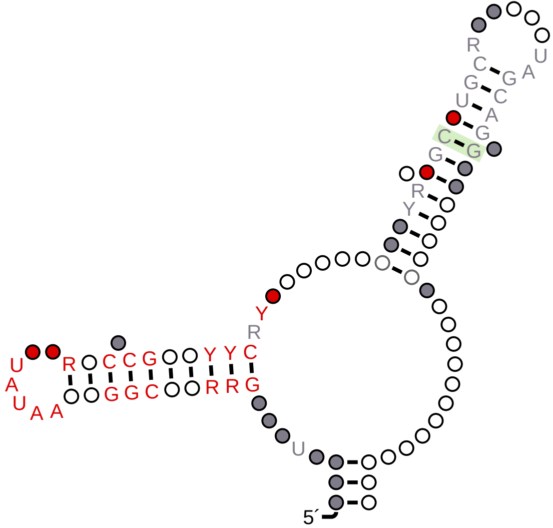

HMP2_10485_1

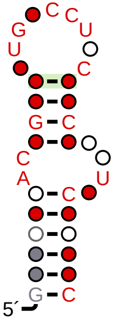

HMP2_10488_1

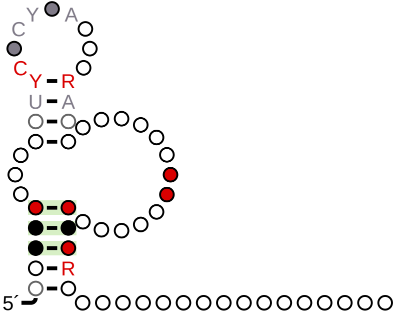

HMP2_10492_1

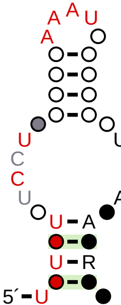

HMP2_10501_1

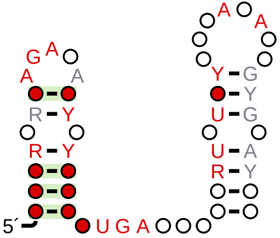

HMP2_10506_1

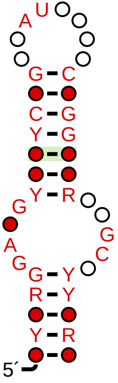

HMP2_10508_1

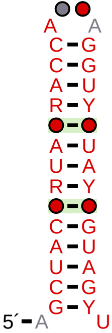

HMP2_10516_1

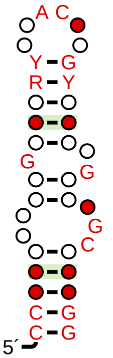

HMP2_1052_1

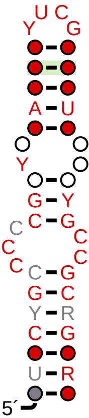

HMP2_10526_1

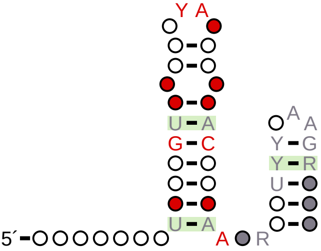

HMP2_10531_1

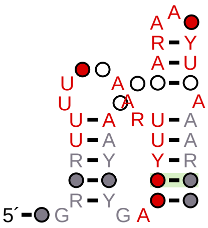

HMP2_10542_1

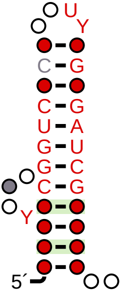

HMP2_10546_1

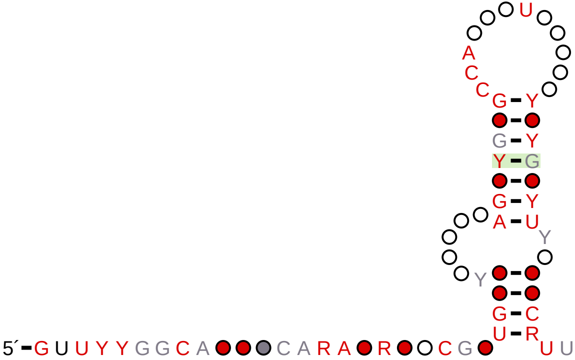

HMP2_10550_1

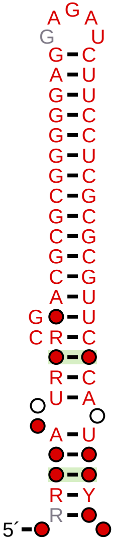

HMP2_10555_1

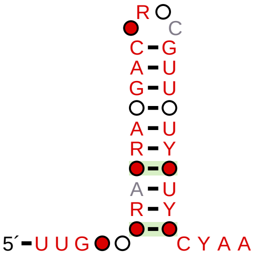

HMP2_1056_1

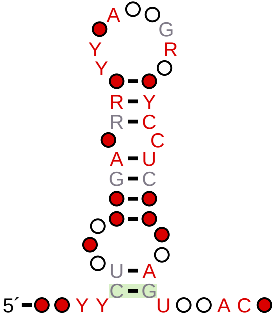

HMP2_10560_1

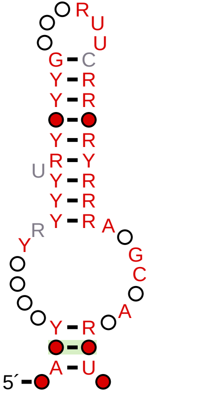

HMP2_10566_1

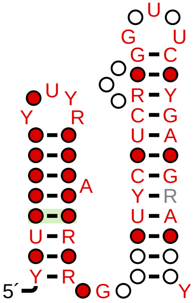

HMP2_1057_1

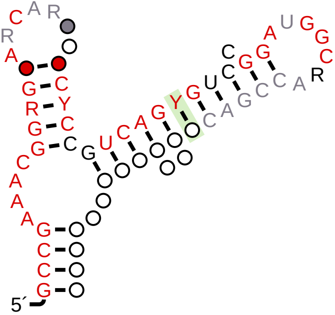

HMP2_10571_1

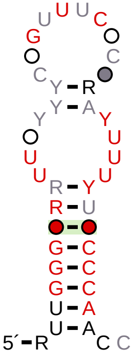

HMP2_10578_1

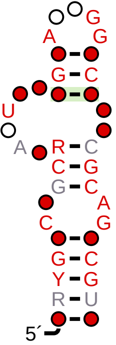

HMP2_10581_1

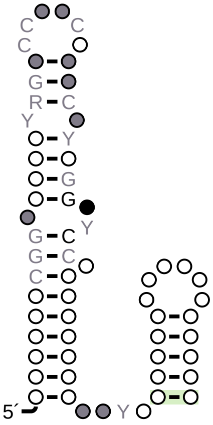

HMP2_10586_1

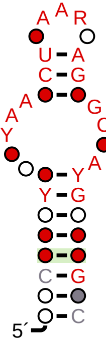

HMP2_10602_1

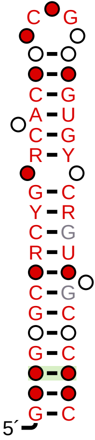

HMP2_10635_1

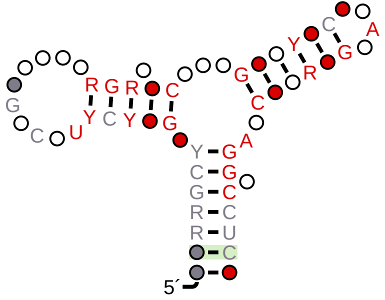

HMP2_1064_1

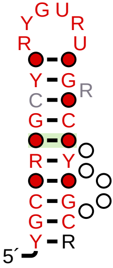

HMP2_10647_1

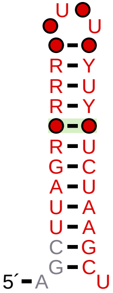

HMP2_10652_1

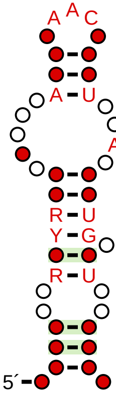

HMP2_10657_1

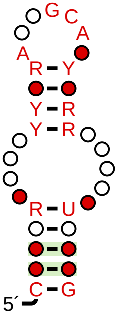

HMP2_10662_1

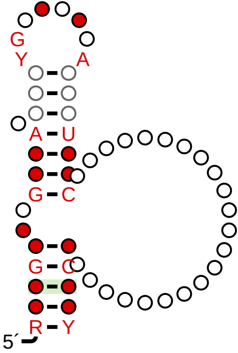

HMP2_10670_1

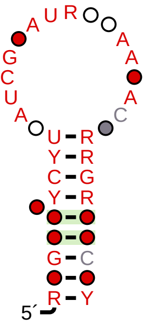

HMP2_10671_1

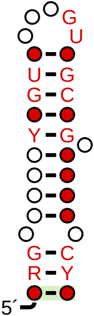

HMP2_10674_1

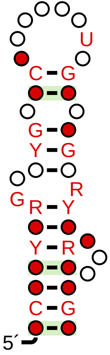

HMP2_10675_1

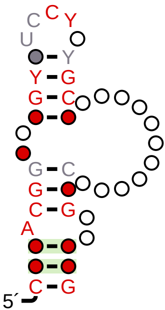

HMP2_10686_1

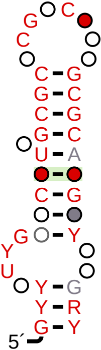

HMP2_10697_1

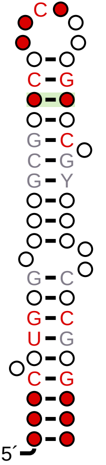

HMP2_10715_1

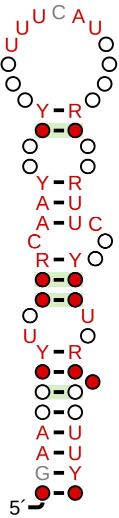

HMP2_10716_1

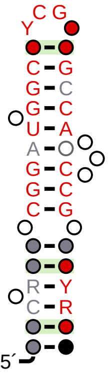

HMP2_10727_1

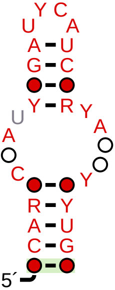

HMP2_10732_1

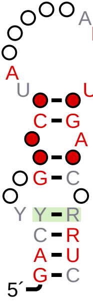

HMP2_10742_1

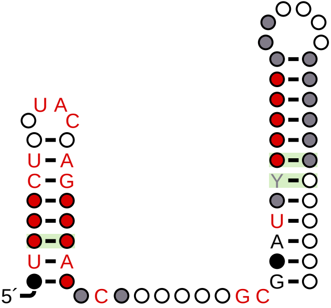

HMP2_10752_1

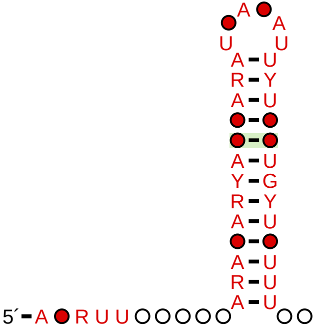

HMP2_10753_1

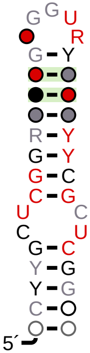

HMP2_10754_1

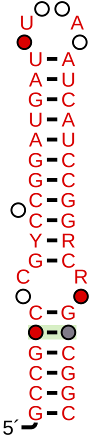

HMP2_10757_1

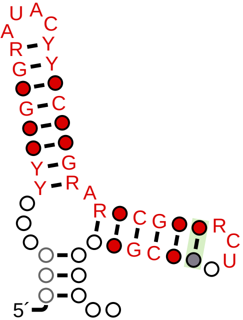

HMP2_10760_1

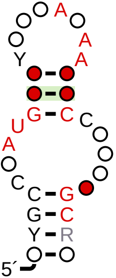

HMP2_10779_1

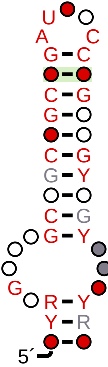

HMP2_10781_1

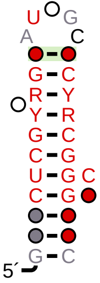

HMP2_10785_1

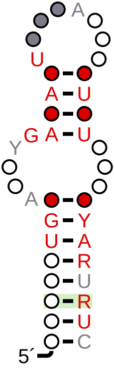

HMP2_10792_1

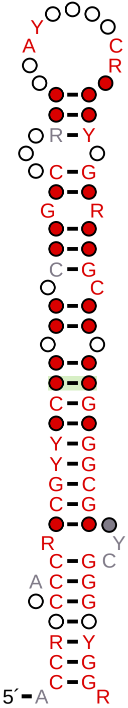

HMP2_10796_1

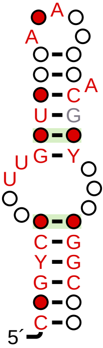

HMP2_10801_1

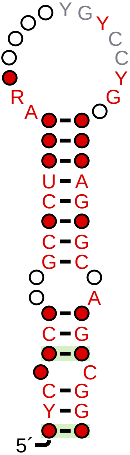

HMP2_10802_1

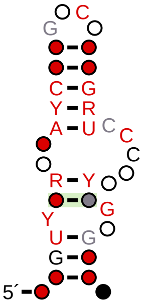

HMP2_10810_1

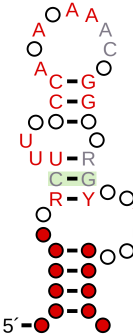

HMP2_10818_1

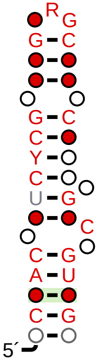

HMP2_10836_1

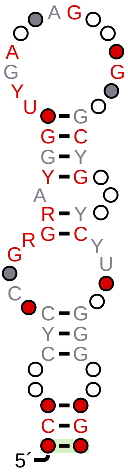

HMP2_10837_1

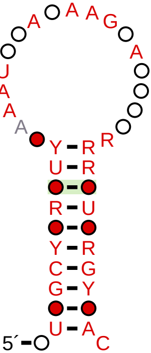

HMP2_10840_1

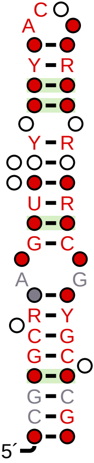

HMP2_10847_1

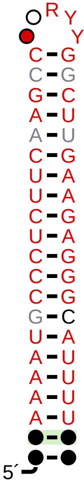

HMP2_1085_1

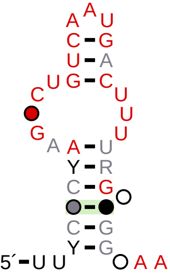

HMP2_10851_1

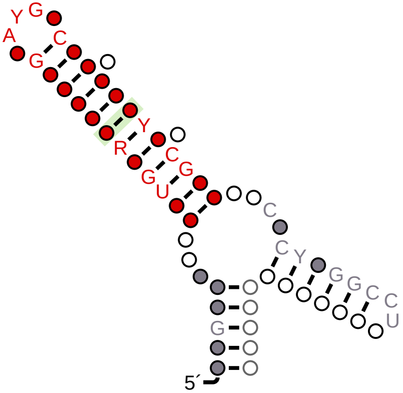

HMP2_10867_1

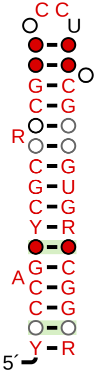

HMP2_10884_1

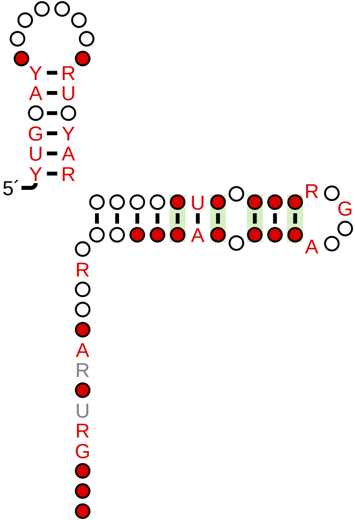

HMP2_10888_1

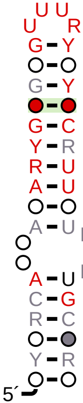

HMP2_10894_1

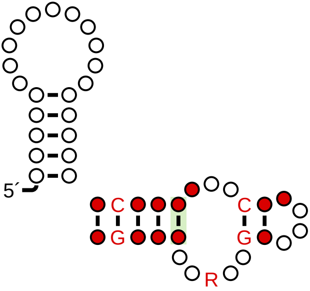

HMP2_10896_1

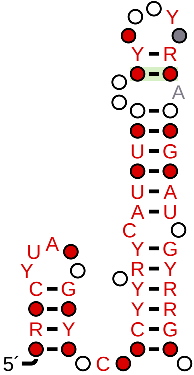

HMP2_10899_1

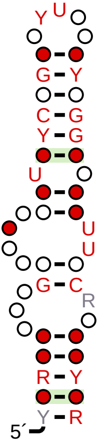

HMP2_10903_1

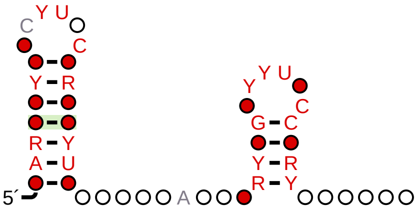

HMP2_1091_1

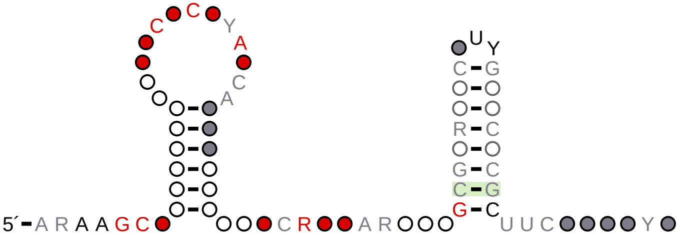

HMP2_10911_1

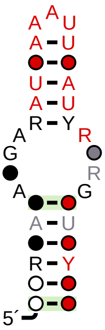

HMP2_10921_1

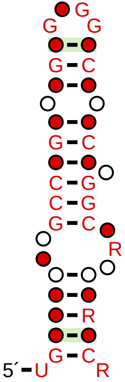

HMP2_10925_1

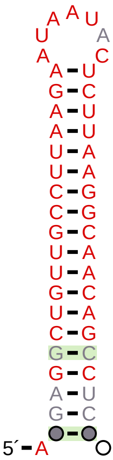

HMP2_10931_1

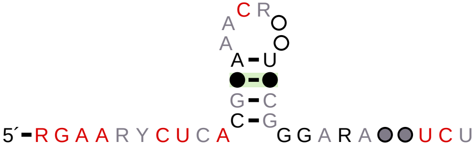

HMP2_10934_1

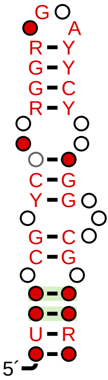

HMP2_10962_1

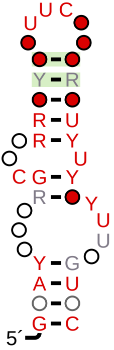

HMP2_10976_1

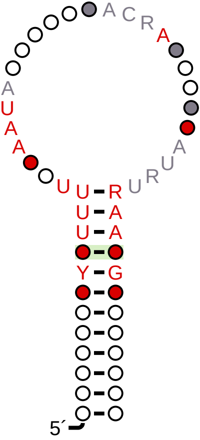

HMP2_10977_1

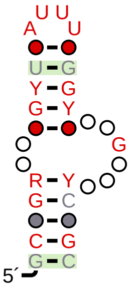

HMP2_11006_1

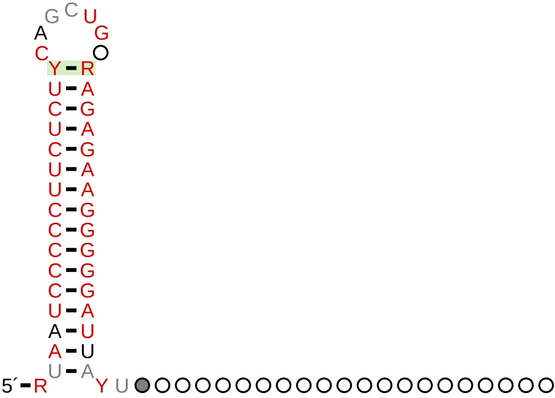

HMP2_11010_1

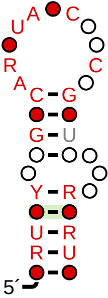

HMP2_11011_1

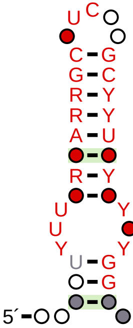

HMP2_11018_1

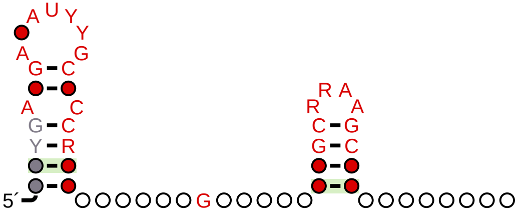

HMP2_11029_1

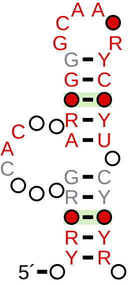

HMP2_11049_1

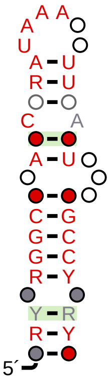

HMP2_11058_1

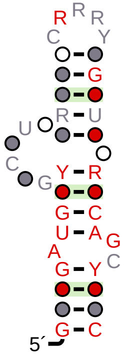

HMP2_11061_1

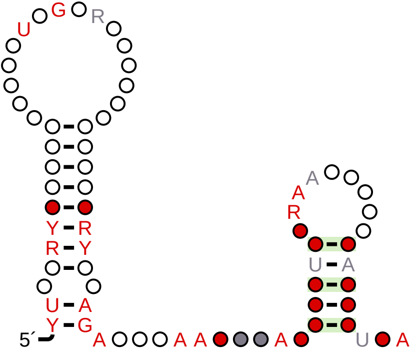

HMP2_11069_1

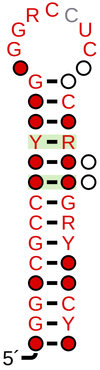

HMP2_11073_1

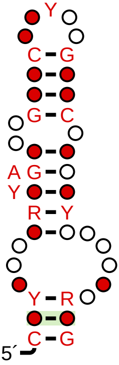

HMP2_1108_1

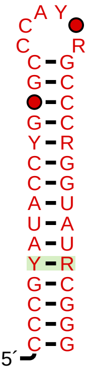

HMP2_11094_1

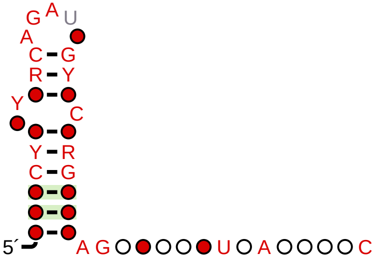

HMP2_11097_1

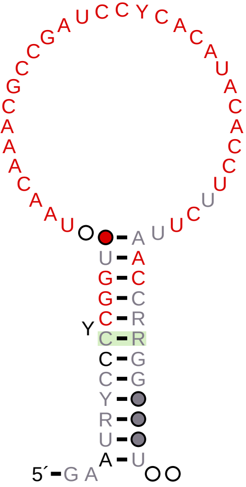

HMP2_11113_1

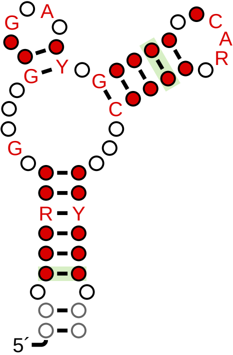

HMP2_11115_1

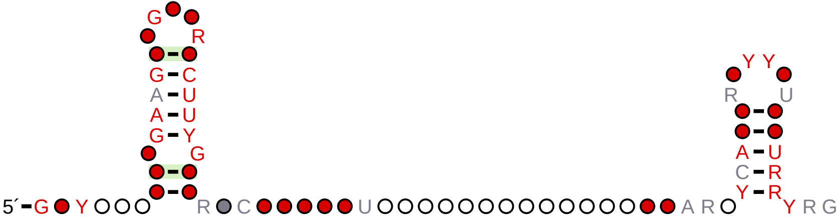

HMP2_11118_1

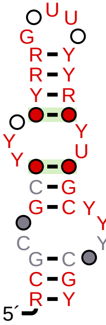

HMP2_11121_1

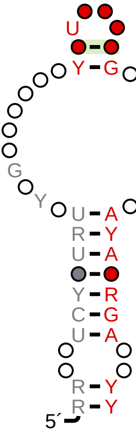

HMP2_11148_1

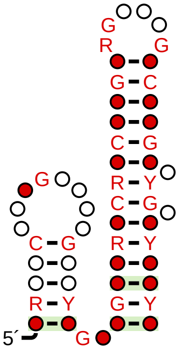

HMP2_11164_1

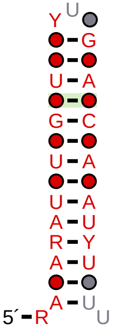

HMP2_11165_1

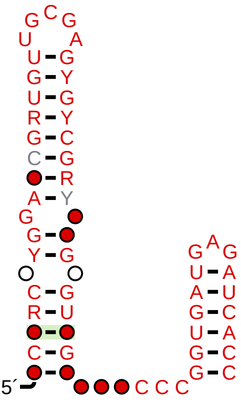

HMP2_11168_1

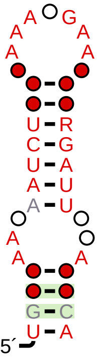

HMP2_11169_1

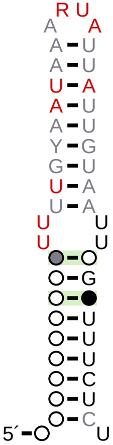

HMP2_11176_1

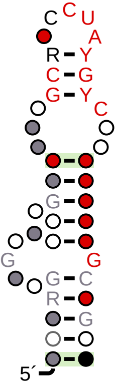

HMP2_11182_1

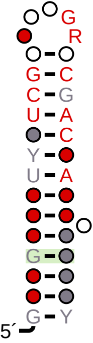

HMP2_11196_1

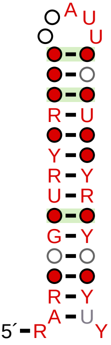

HMP2_11214_1

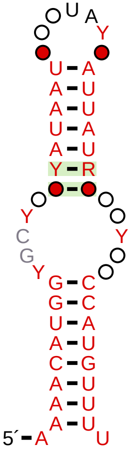

HMP2_11215_1

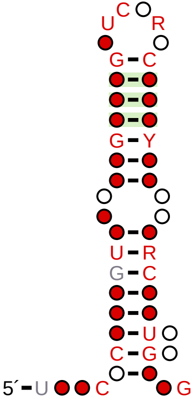

HMP2_11216_1

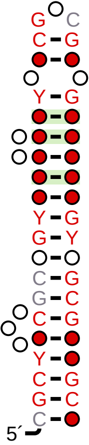

HMP2_11222_1

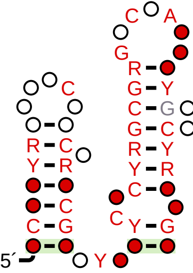

HMP2_11223_1

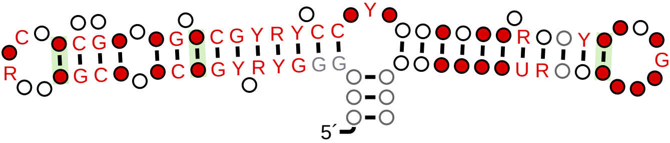

HMP2_11229_1

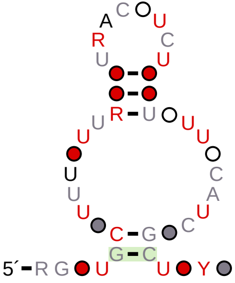

HMP2_11232_1

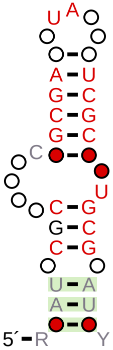

HMP2_11233_1

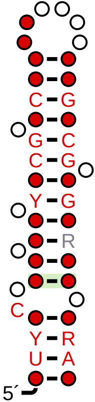

HMP2_11234_1

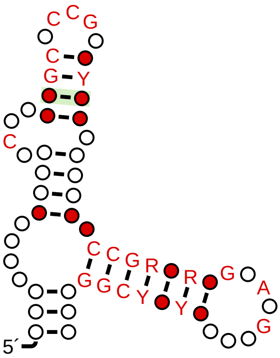

HMP2_11242_1

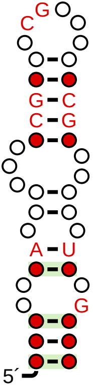

HMP2_11244_1

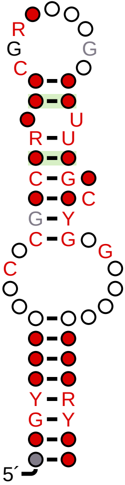

HMP2_11257_1

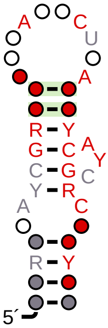

HMP2_11274_1

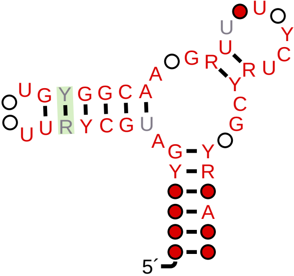

HMP2_11284_1

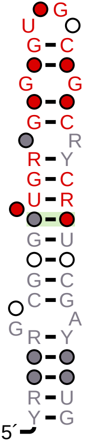

HMP2_11285_1

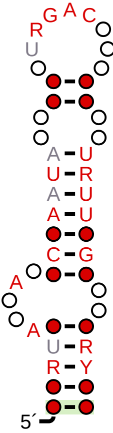

HMP2_11298_1

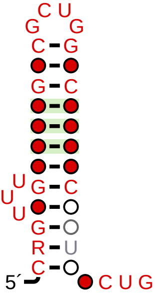

HMP2_11316_1

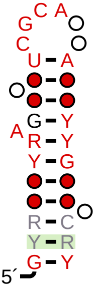

HMP2_11332_1

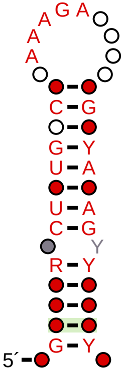

HMP2_11340_1

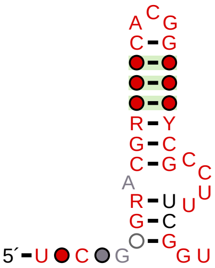

HMP2_11354_1

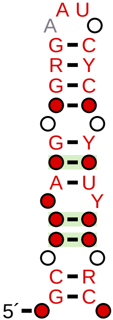

HMP2_11355_1

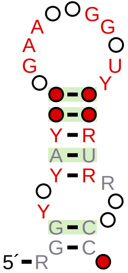

HMP2_11359_1

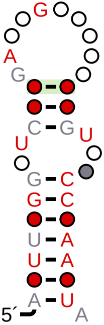

HMP2_1138_1

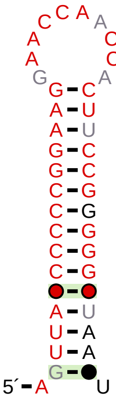

HMP2_11391_1

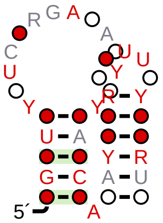

HMP2_11396_1

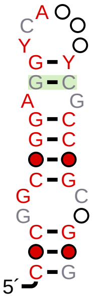

HMP2_114_1

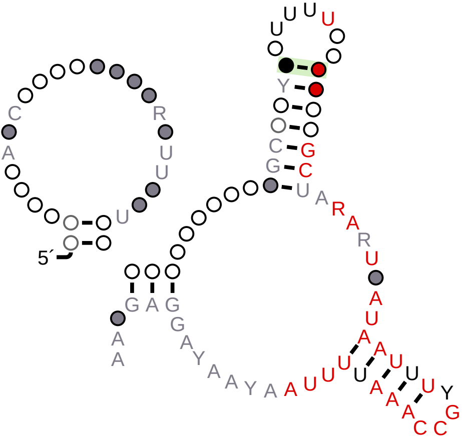

HMP2_11410_1

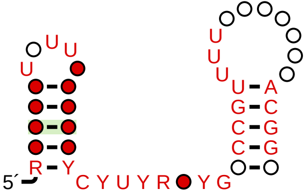

HMP2_11423_1

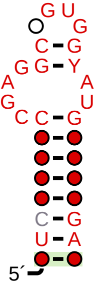

HMP2_11429_1

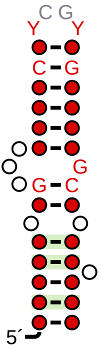

HMP2_11448_1

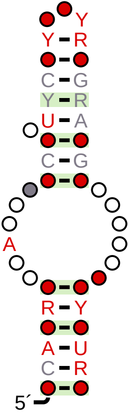

HMP2_11449_1

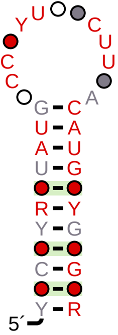

HMP2_11456_1

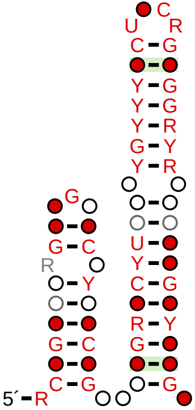

HMP2_11471_1

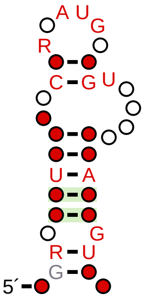

HMP2_11476_1

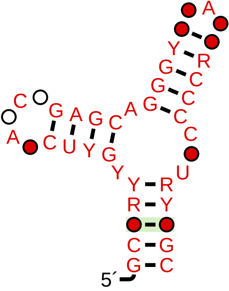

HMP2_11479_1

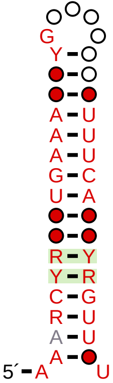

HMP2_11502_1

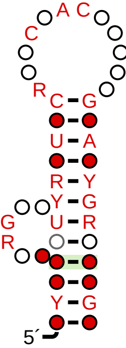

HMP2_11519_1

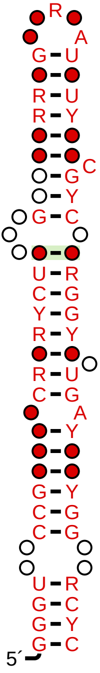

HMP2_11521_1

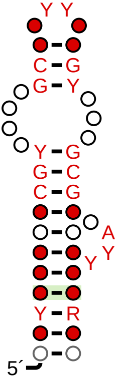

HMP2_11525_1

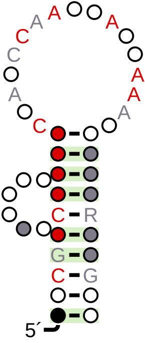

HMP2_11535_1

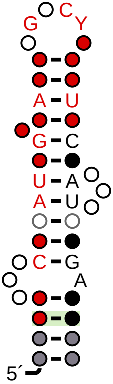

HMP2_11537_1

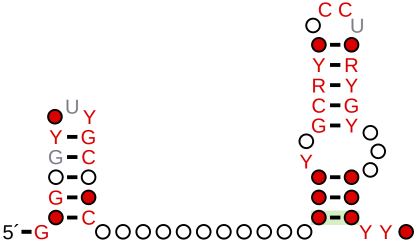

HMP2_11552_1

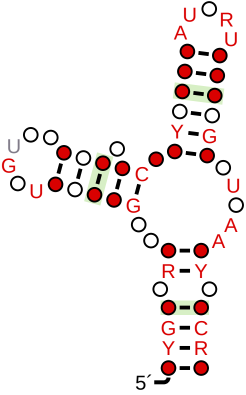

HMP2_11557_1

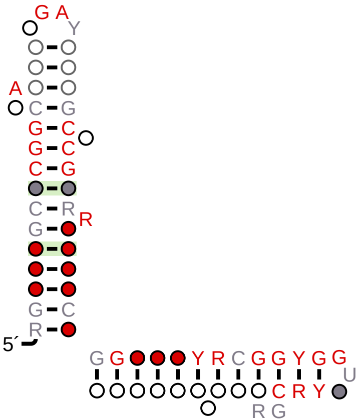

HMP2_11559_1

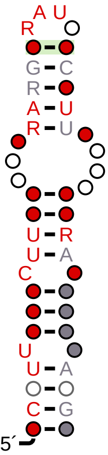

HMP2_11562_1

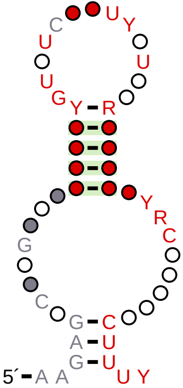

HMP2_11572_1

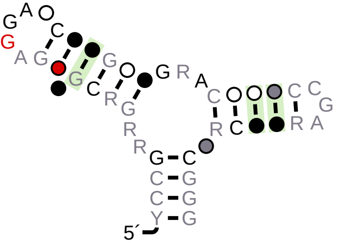

HMP2_11580_1

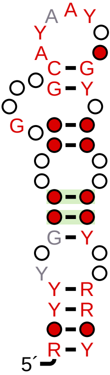

HMP2_11584_1

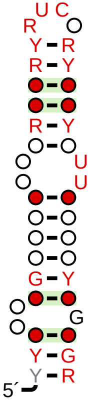

HMP2_11594_1

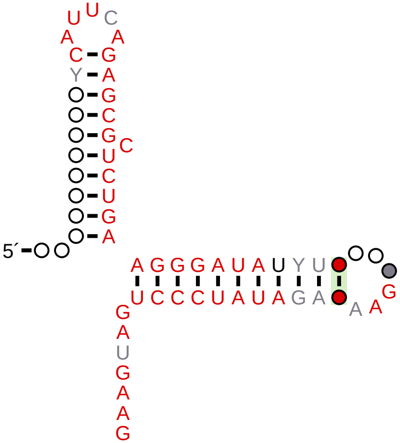

HMP2_1160_1

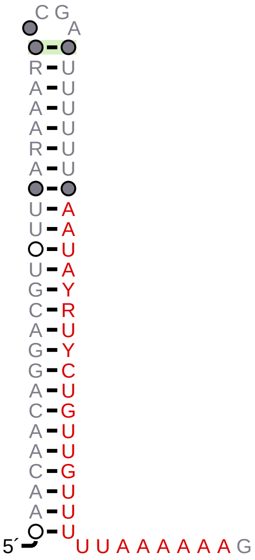

C G
A
R U
A U
A U
A U
R U
A U
U A
U A
U
U A
G Y
C R
A U
G Y
G C
A U
C G
A U
A U
C G
A U
A U
U
5´
U U A A A A A A G

HMP2_11601_1

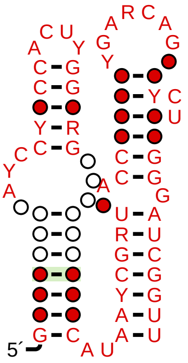

HMP2_11618_1

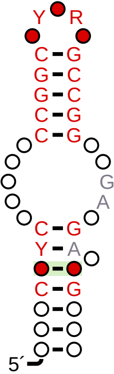

HMP2_11620_1

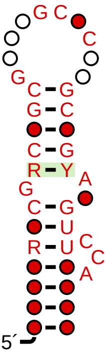

HMP2_11628_1

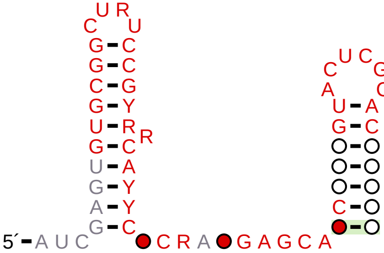

HMP2_11632_1

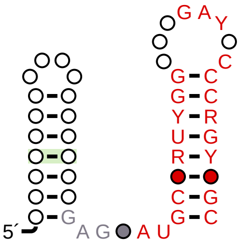

HMP2_11636_1

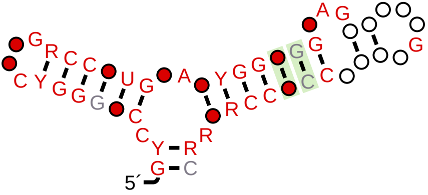

HMP2_11659_1

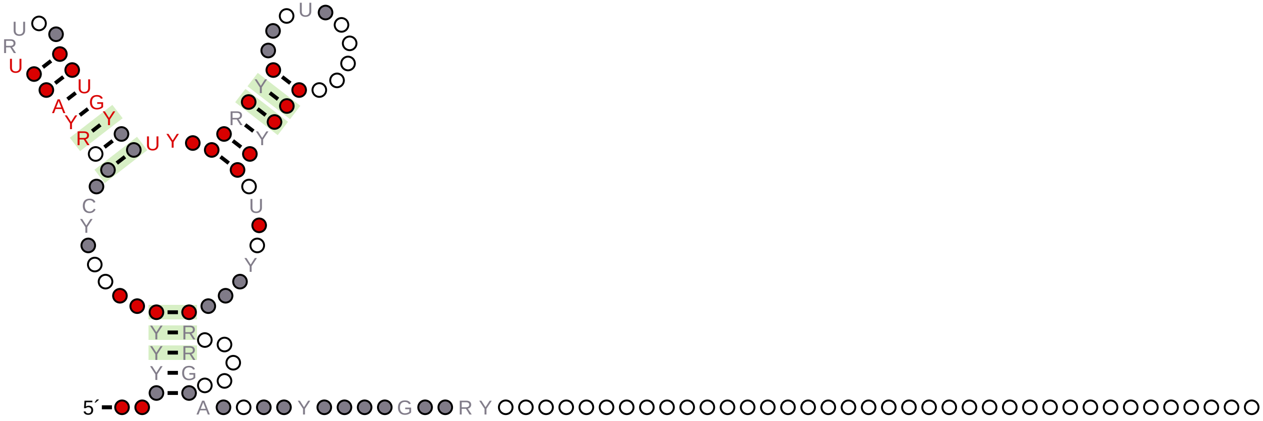

HMP2_11670_1

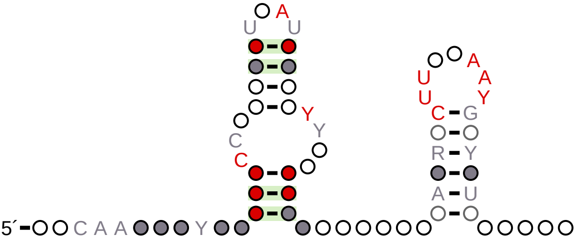

HMP2_11682_1

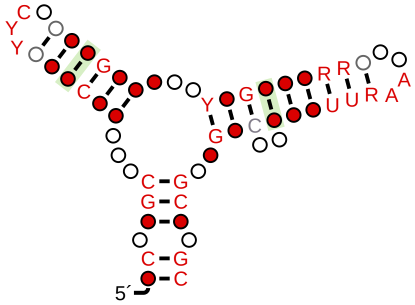

HMP2_1169_1

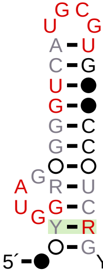

HMP2_11693_1

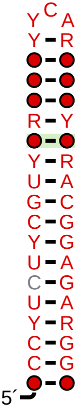

HMP2_11717_1

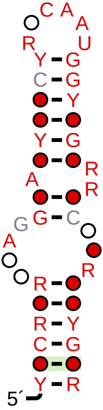

HMP2_11721_1

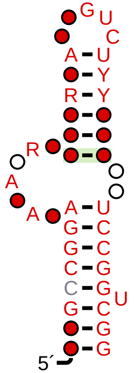

HMP2_11728_1

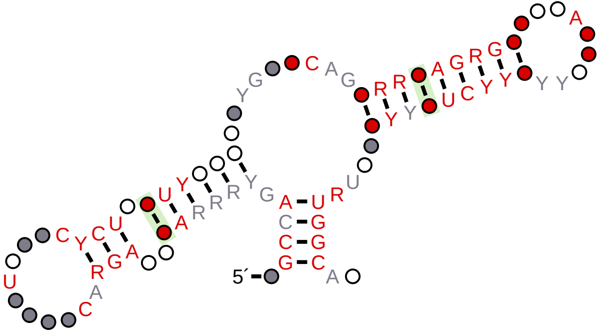

HMP2_11734_1

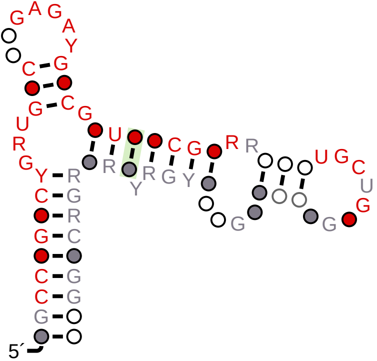

HMP2_1174_1

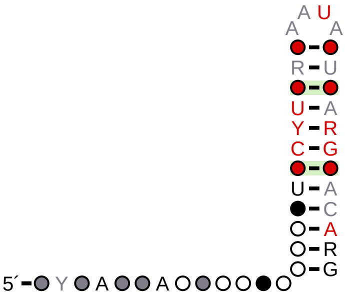

HMP2_11746_1

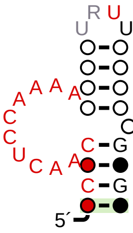

HMP2_11749_1

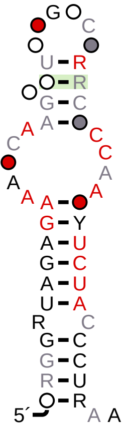

HMP2_11752_1

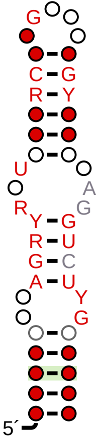

HMP2_11766_1

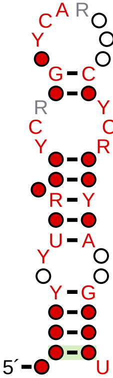

HMP2_11772_1

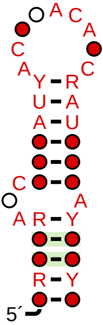

HMP2_11775_1

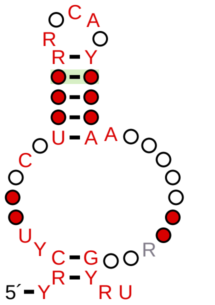

HMP2_11783_1

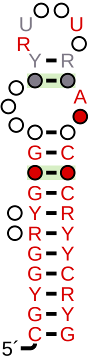

HMP2_1179_1

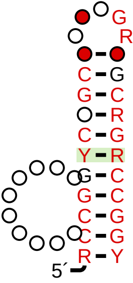

HMP2_11793_1

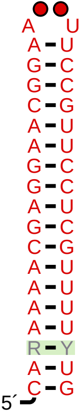

HMP2_11795_1

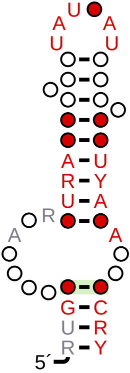

HMP2_11806_1

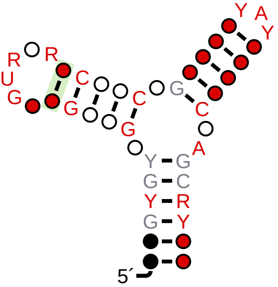

HMP2_11807_1

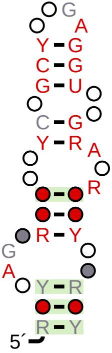

HMP2_11818_1

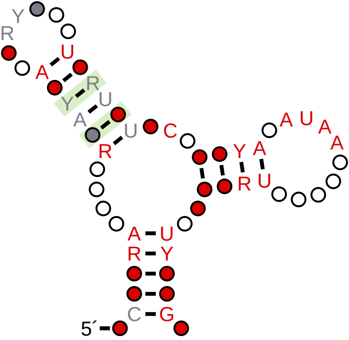

HMP2_11821_1

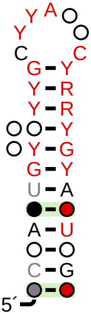

HMP2_11823_1

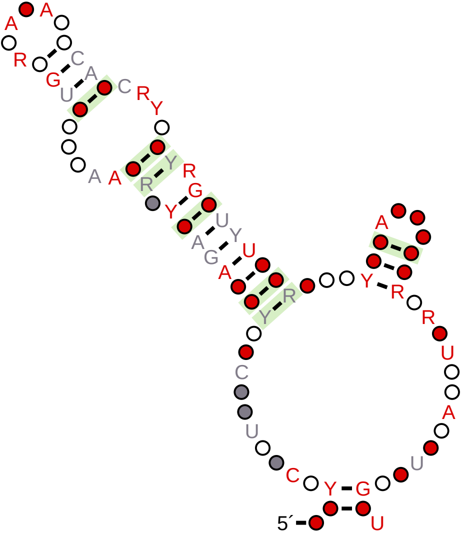

HMP2_11841_1

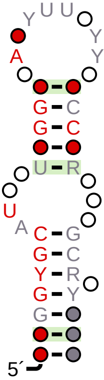

HMP2_11851_1

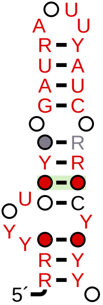

HMP2_11854_1

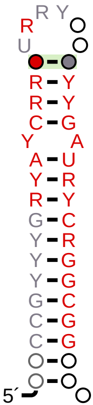

HMP2_11870_1

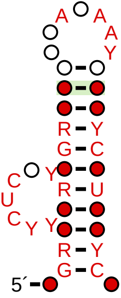

HMP2_11887_1

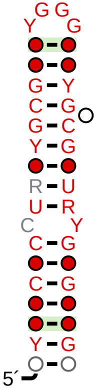

HMP2_1189_1

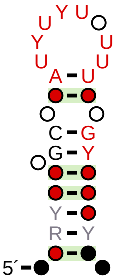

HMP2_11893_1

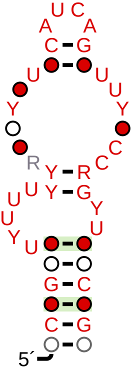

HMP2_11896_1

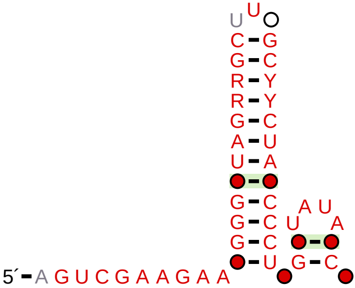

HMP2_11911_1

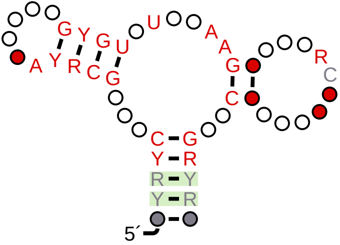

HMP2_11924_1

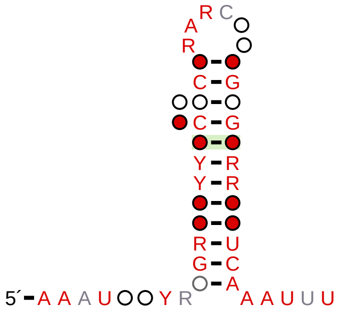

HMP2_11929_1

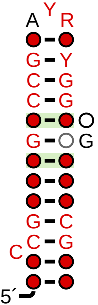

HMP2_1193_1

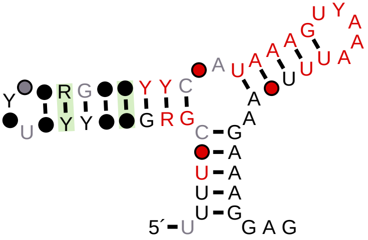

HMP2_11930_1

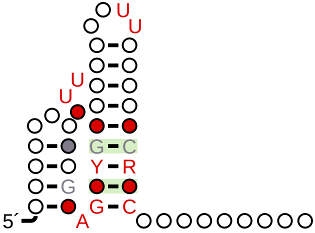

HMP2_11932_1

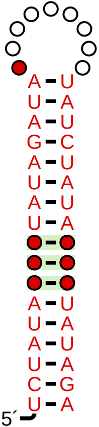

HMP2_11937_1

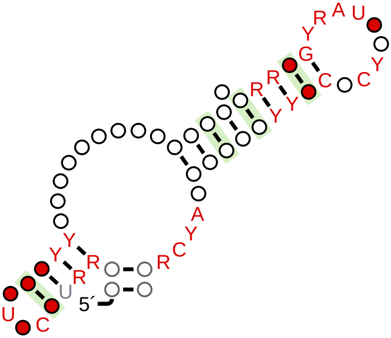

HMP2_11949_1

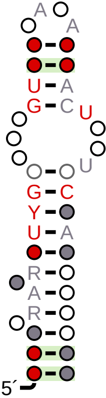

HMP2_11952_1

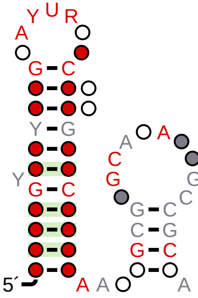

HMP2_11955_1

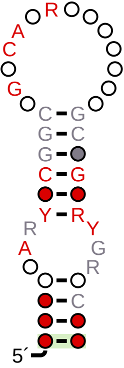

HMP2_11956_1

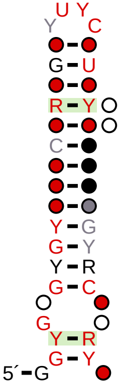

HMP2_11966_1

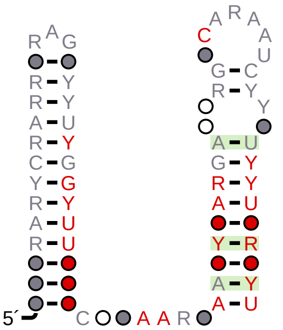

HMP2_11967_1

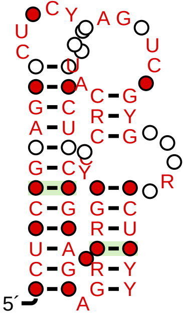

HMP2_1197_1

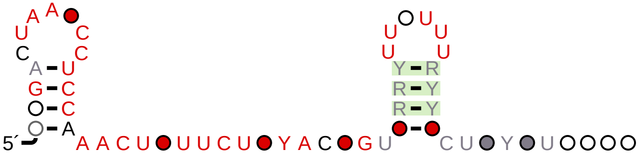

HMP2_11976_1

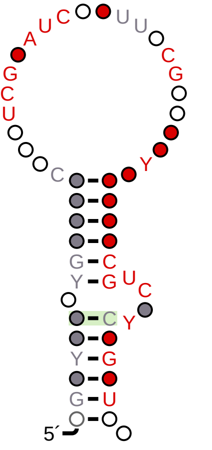

HMP2_11985_1

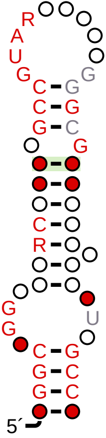

HMP2_11987_1

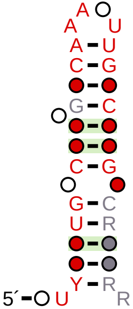

HMP2_11991_1

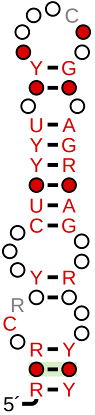

HMP2_11994_1

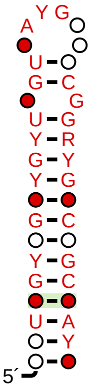

HMP2_11996_1

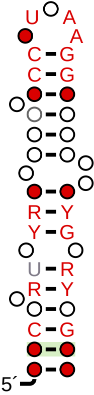

HMP2_12001_1

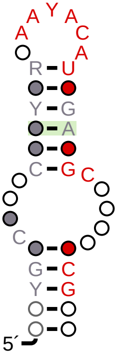

HMP2_12041_1

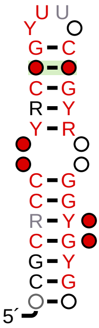

HMP2_12050_1

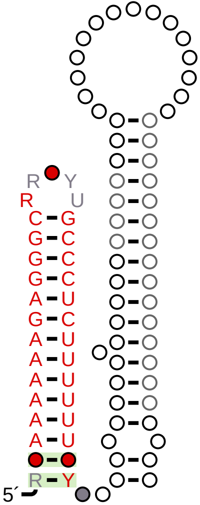

HMP2_12053_1

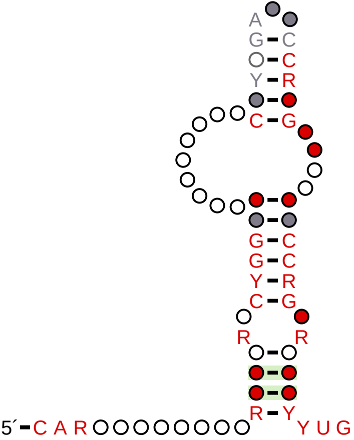

HMP2_12069_1

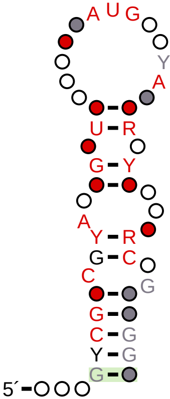

HMP2_12079_1

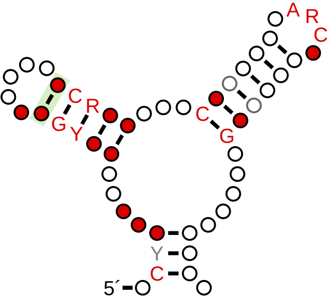

HMP2_12084_1

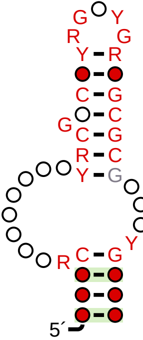

HMP2_12088_1

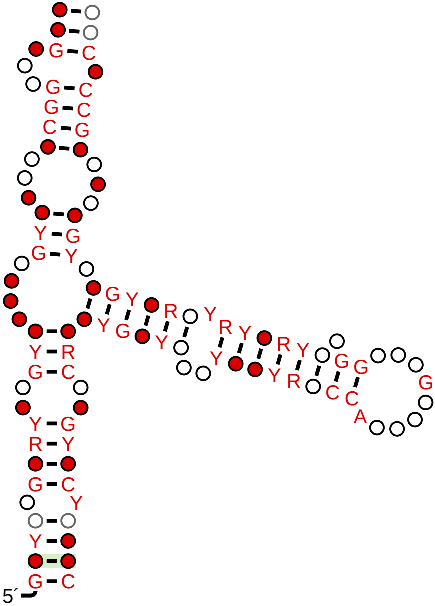

HMP2_12092_1

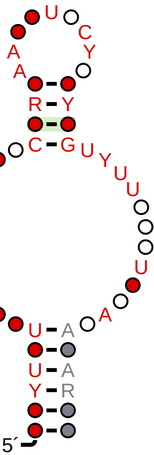

HMP2_12093_1

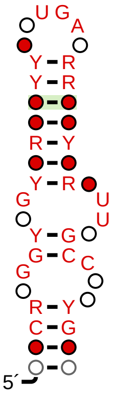

HMP2_12107_1

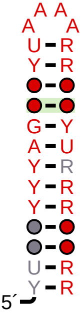

HMP2_12112_1

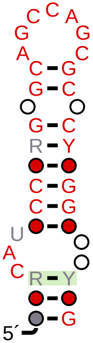

HMP2_1212_1

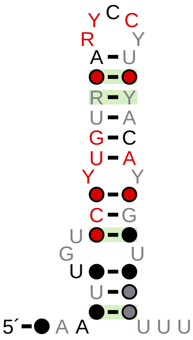

HMP2_12121_1

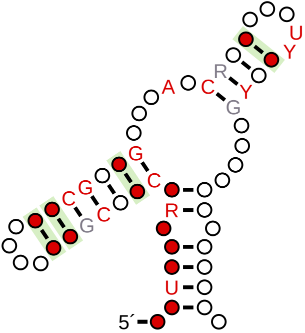

HMP2_12128_1

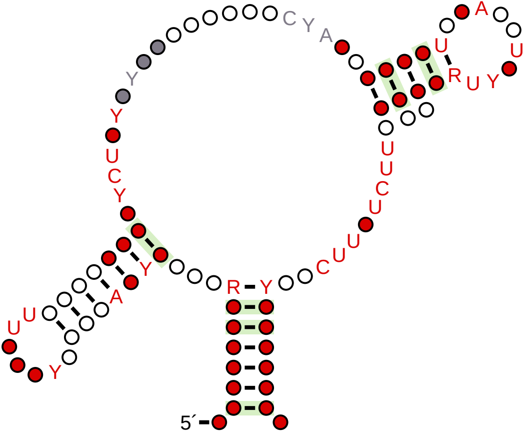

HMP2_12133_1

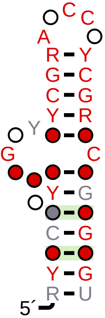

HMP2_12138_1

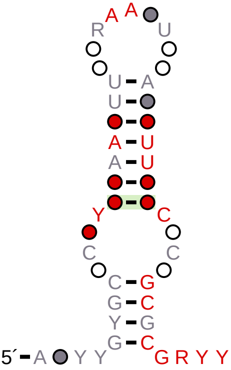

HMP2_12157_1

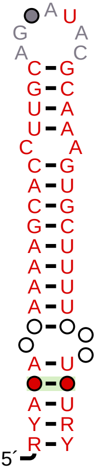

HMP2_12170_1

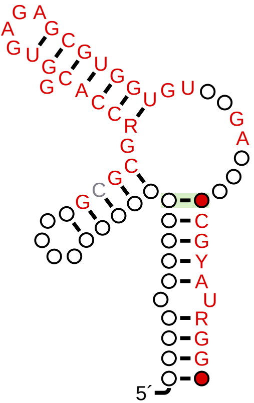

HMP2_12181_1

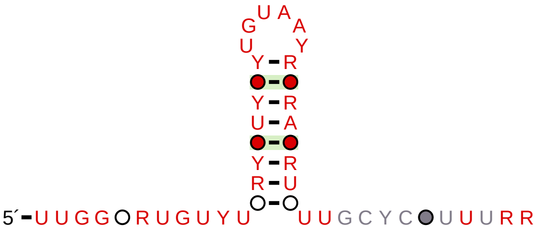

HMP2_12188_1

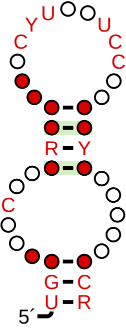

HMP2_12196_1

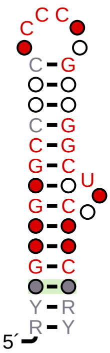

HMP2_12226_1

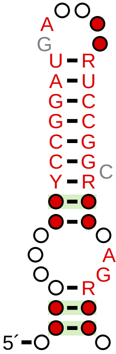

HMP2_12239_1

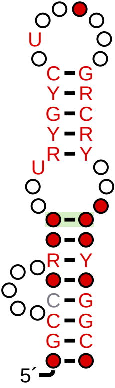

HMP2_12242_1

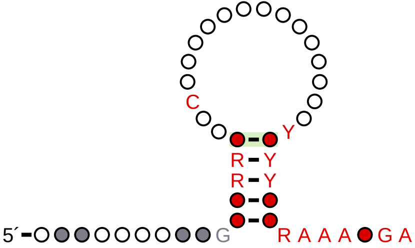

HMP2_12248_1

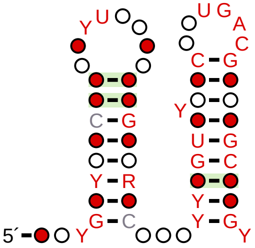

HMP2_12257_1

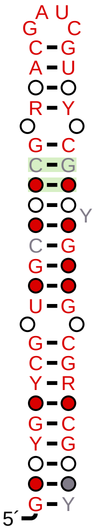

HMP2_12260_1

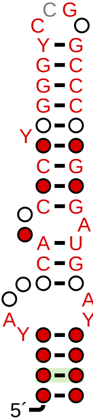

HMP2_12271_1

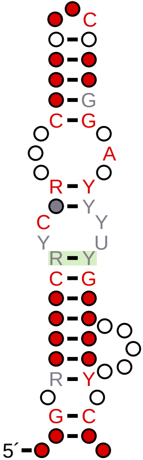

HMP2_12272_1

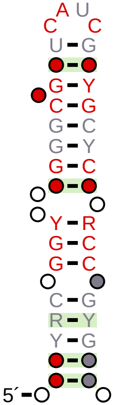

HMP2_12275_1

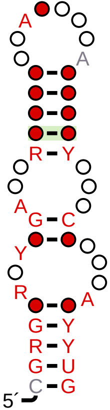

HMP2_12280_1

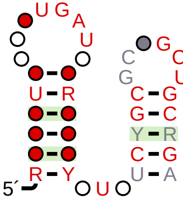

HMP2_12281_1

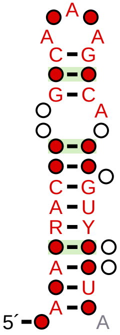

HMP2_12282_1

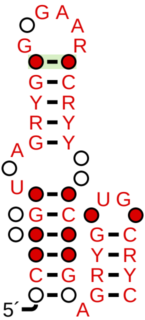

HMP2_12289_1

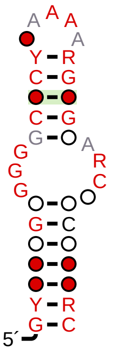

HMP2_123_1

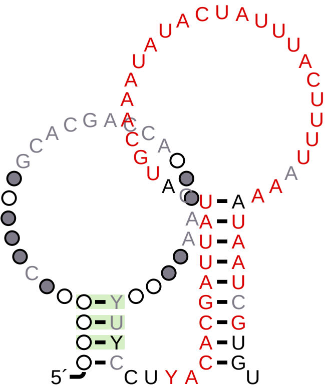

HMP2_12303_1

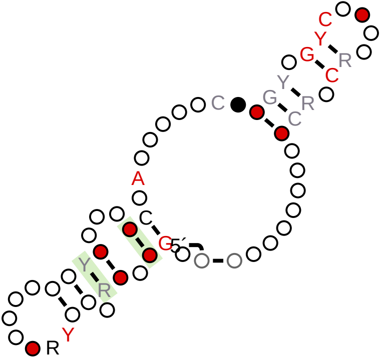

HMP2_12305_1

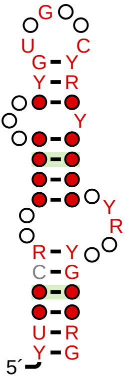

HMP2_12308_1

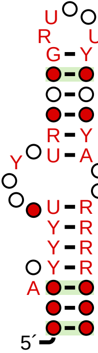

HMP2_12310_1

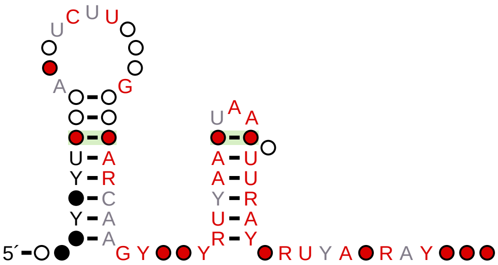

HMP2_12313_1

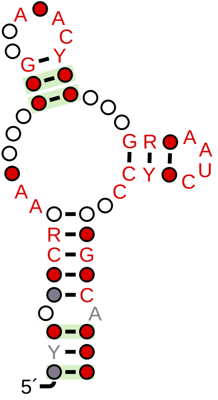

HMP2_12324_1

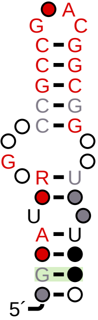

HMP2_12364_1

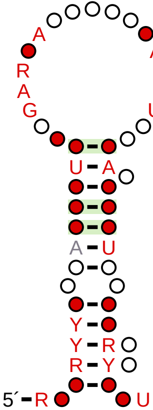

HMP2_1237_1

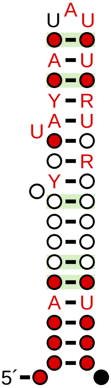

HMP2_12413_1

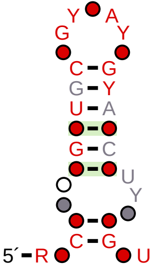

HMP2_12423_1

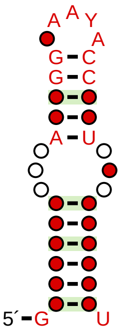

HMP2_1243_1

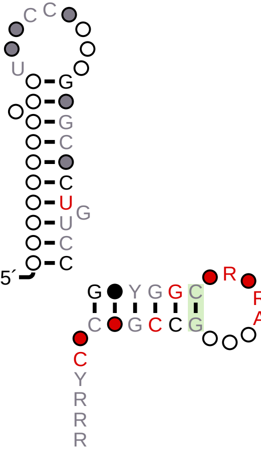

HMP2_12433_1

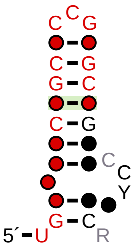

HMP2_12435_1

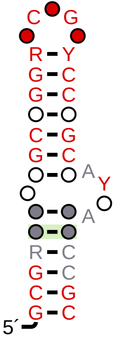

HMP2_12466_1

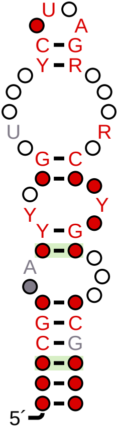

HMP2_12478_1

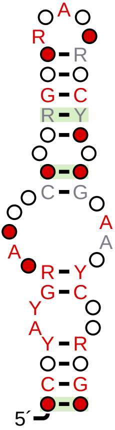

HMP2_1248_1

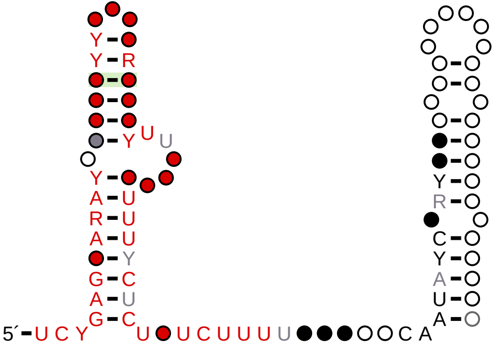

HMP2_12482_1

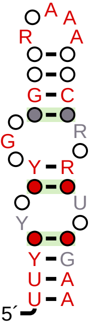

HMP2_1250_1

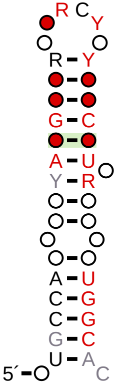

HMP2_12518_1

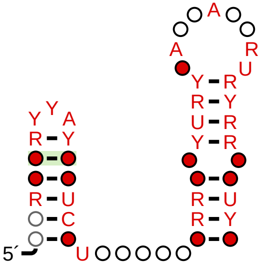

HMP2_1253_1

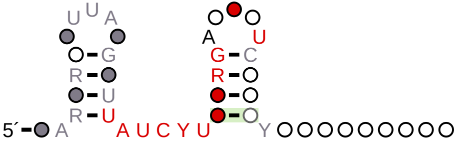

HMP2_12532_1

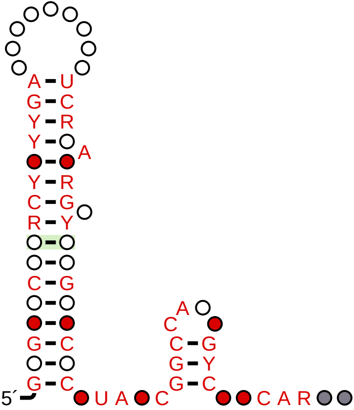

HMP2_12534_1

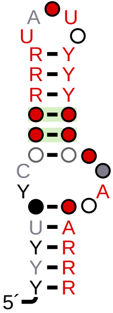

HMP2_12604_1

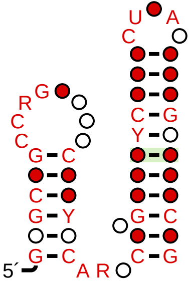

HMP2_12614_1

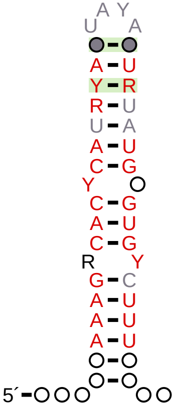

HMP2_12618_1

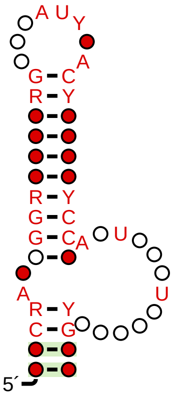

HMP2_1265_1

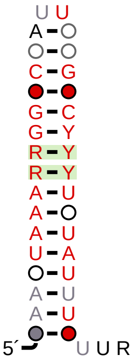

HMP2_1267_1

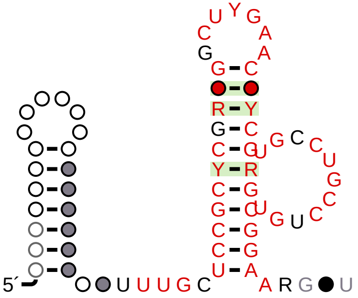

HMP2_12682_1

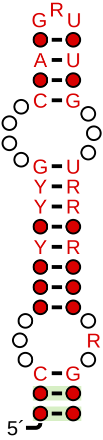

HMP2_12684_1

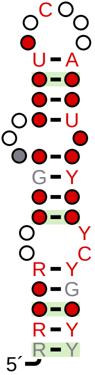

HMP2_12705_1

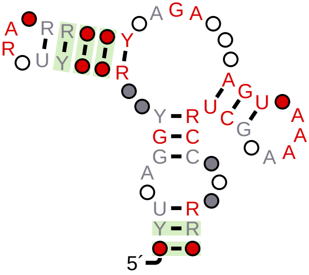

HMP2_12725_1

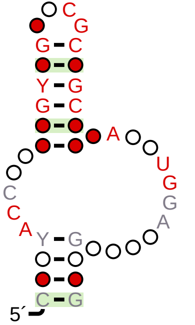

HMP2_12740_1

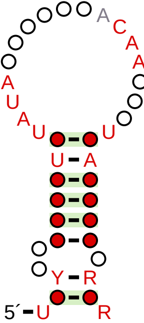

HMP2_12753_1

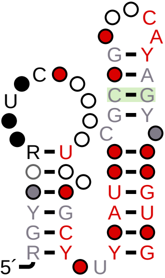

HMP2_12775_1

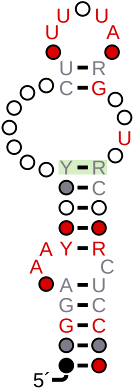

HMP2_12794_1

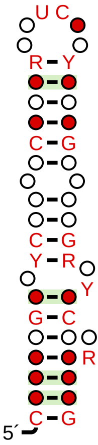

HMP2_1280_1

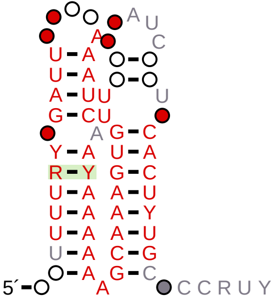

A U
A
C
U - A
U - A
A - UU
U
G - CU
A G - C
Y - A U - A
R - Y G - C
U - A A - U
U - A A - Y
U - A A - U
U - A C - G
A G - C
5´
A
C C R U Y

HMP2_1282_1

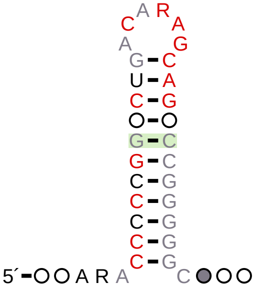

HMP2_12826_1

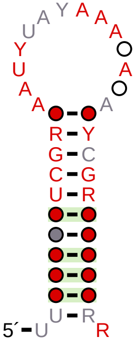

HMP2_12828_1

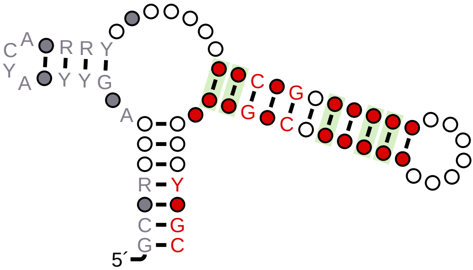

HMP2_12840_1

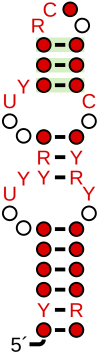

HMP2_12879_1

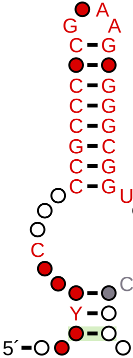

HMP2_12887_1

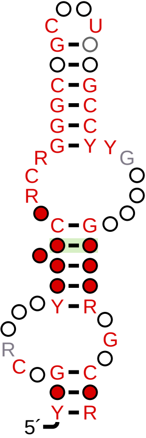

HMP2_12889_1

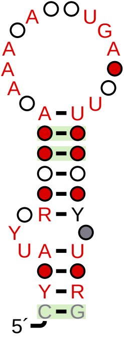

HMP2_12893_1

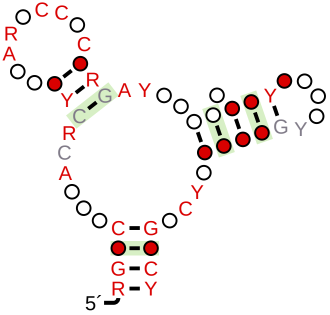

HMP2_12897_1

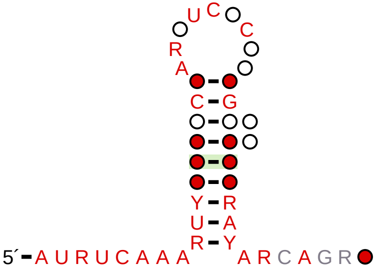

HMP2_12909_1

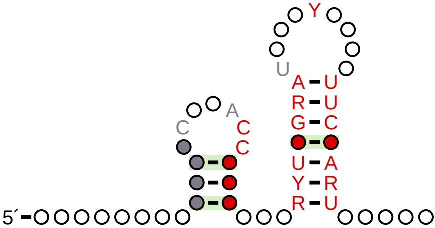

HMP2_12913_1

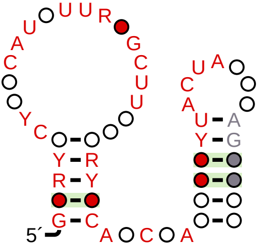

HMP2_1292_1

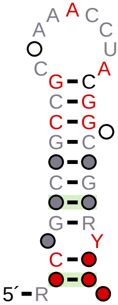

HMP2_12923_1

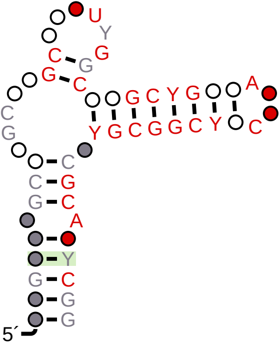

HMP2_1294_1

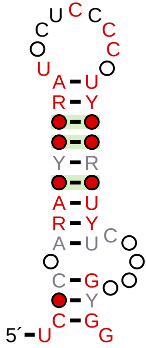

HMP2_12941_1

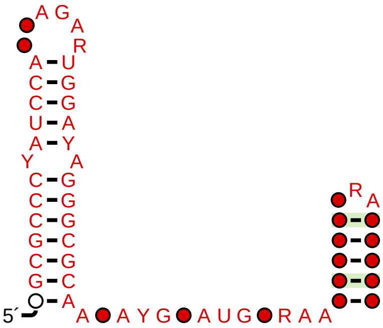

HMP2_12944_1

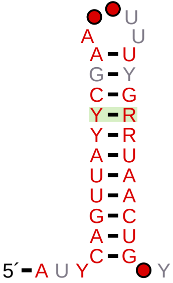

HMP2_12946_1

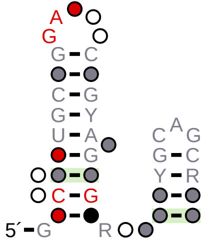

HMP2_12956_1

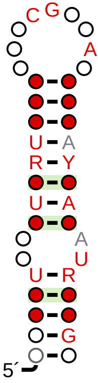

HMP2_12959_1

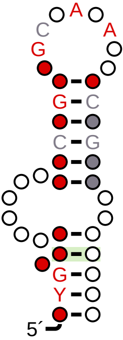

HMP2_12996_1

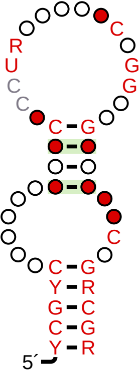

HMP2_13005_1

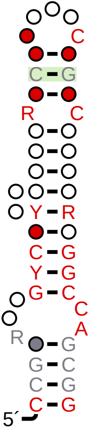

HMP2_13006_1

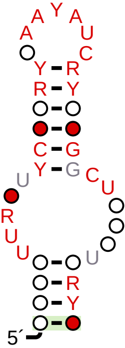

HMP2_13011_1

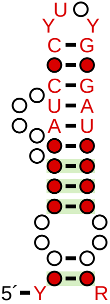

HMP2_13014_1

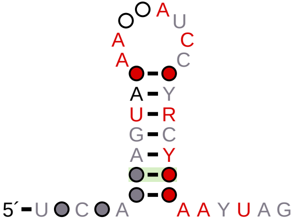

HMP2_13018_1

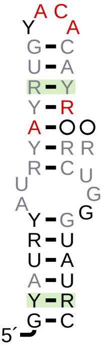

HMP2_13020_1

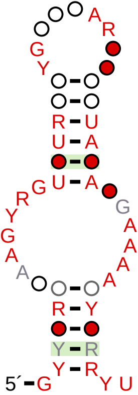

HMP2_13025_1

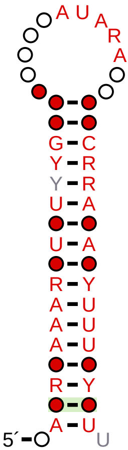

HMP2_13029_1

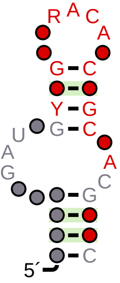

HMP2_13031_1

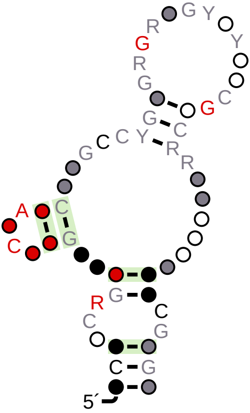

HMP2_13037_1

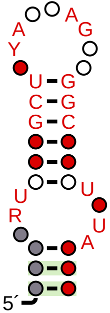

HMP2_13051_1

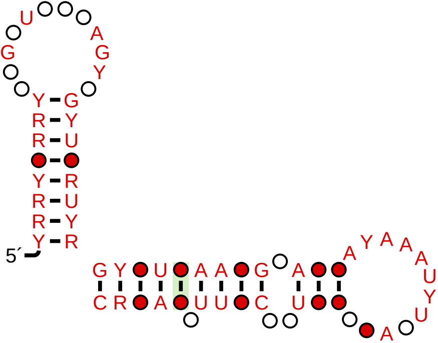

HMP2_13054_1

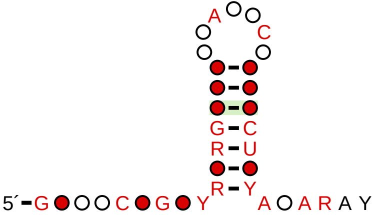

HMP2_13055_1

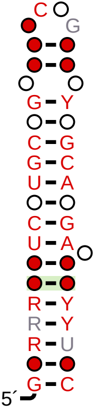

HMP2_13057_1

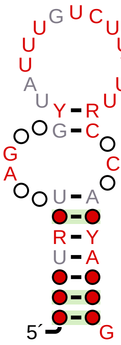

HMP2_1306_1

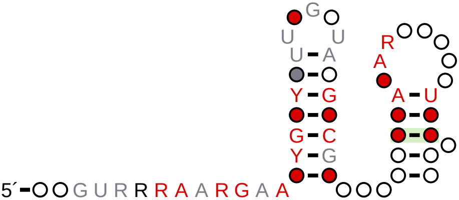

HMP2_13063_1

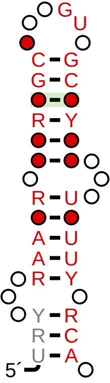

HMP2_13071_1

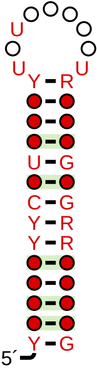

HMP2_13075_1

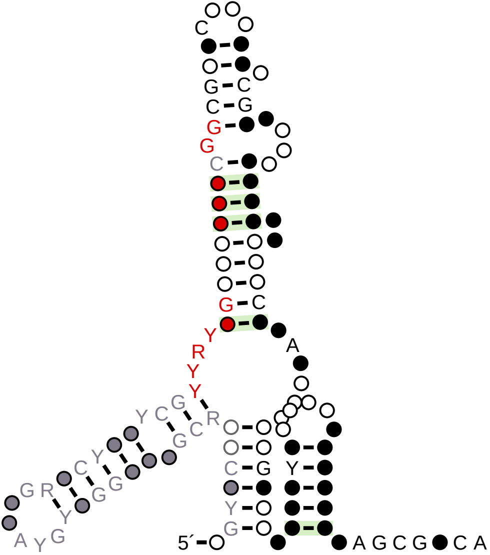

HMP2_131_1

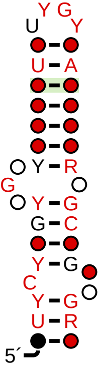

HMP2_13102_1

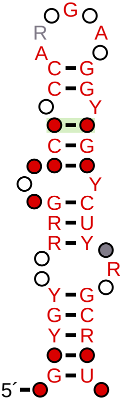

HMP2_13103_1

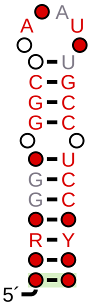

HMP2_13109_1

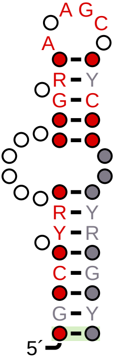

HMP2_13127_1

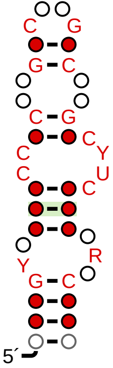

HMP2_13132_1

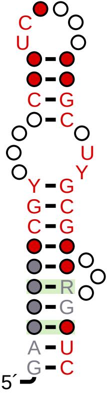

HMP2_13137_1

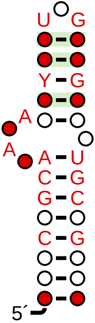

HMP2_13146_1

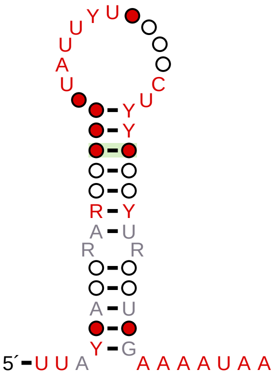

HMP2_1315_1

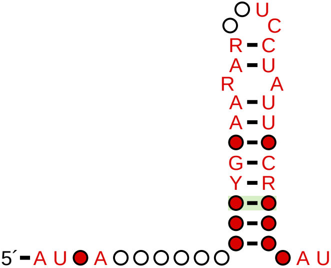

HMP2_13156_1

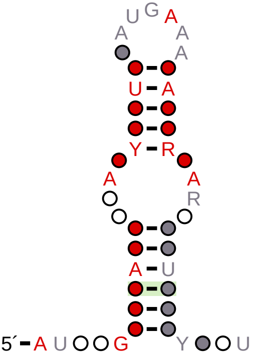

HMP2_13161_1

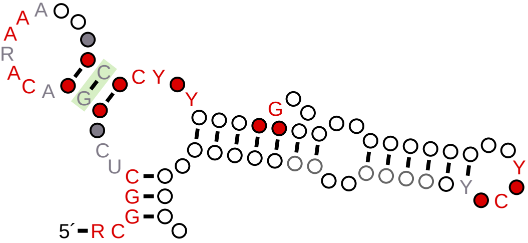

HMP2_13163_1

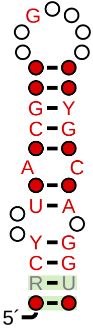

HMP2_13166_1

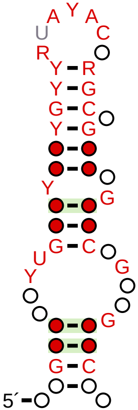

HMP2_13175_1

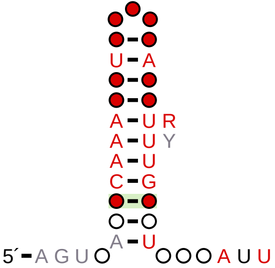

HMP2_1318_1

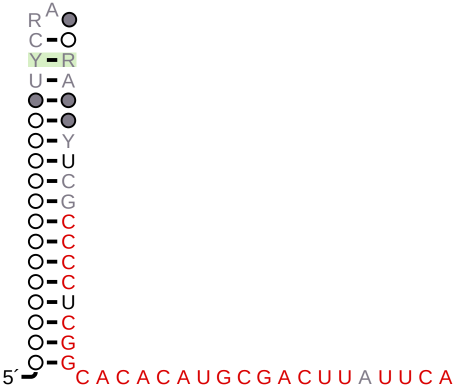

HMP2_13187_1

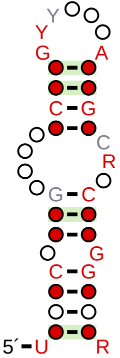

HMP2_13190_1

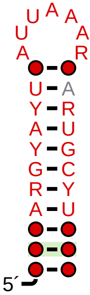

HMP2_13201_1

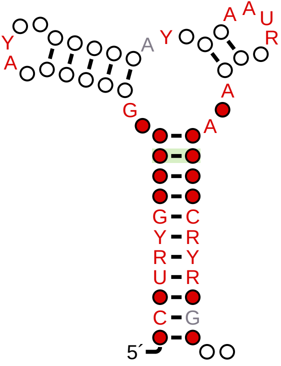

HMP2_1321_1

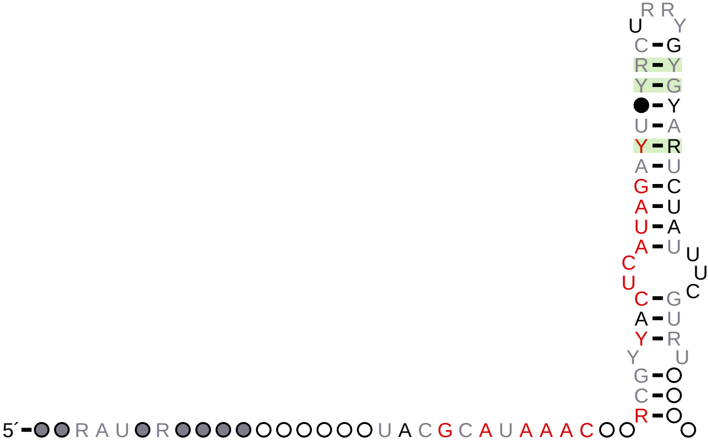

HMP2_13212_1

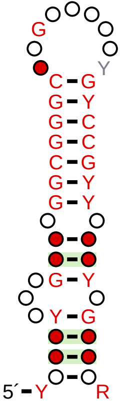

HMP2_13216_1

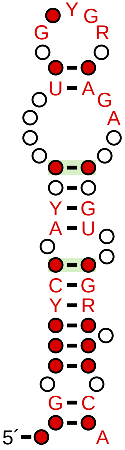

HMP2_13219_1

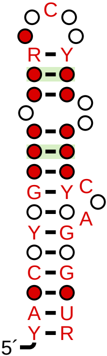

HMP2_13227_1

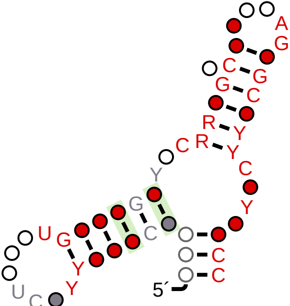

HMP2_13239_1

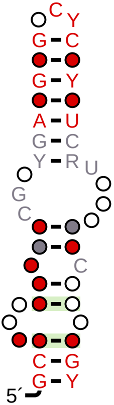

HMP2_1324_1

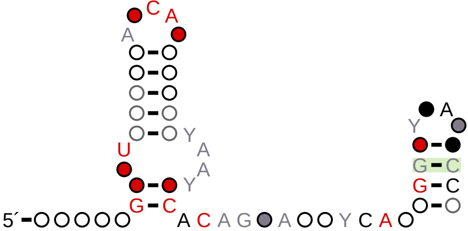

HMP2_13241_1

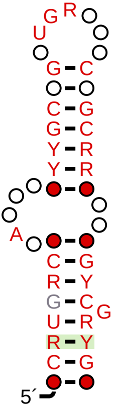

HMP2_13256_1

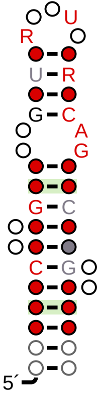

HMP2_1326_1

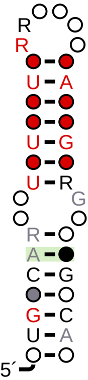

HMP2_13263_1

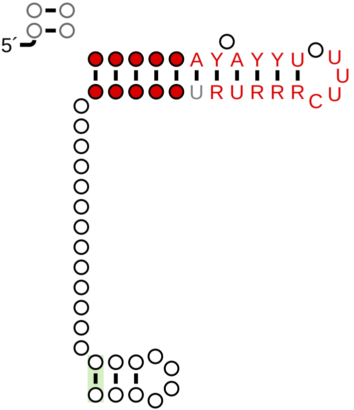

HMP2_13265_1

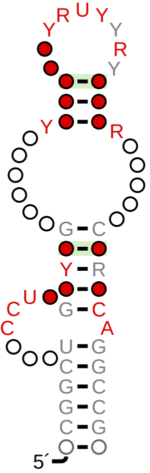

HMP2_13266_1

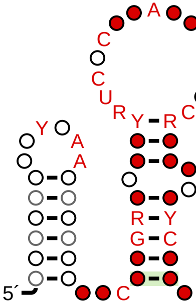

HMP2_13267_1

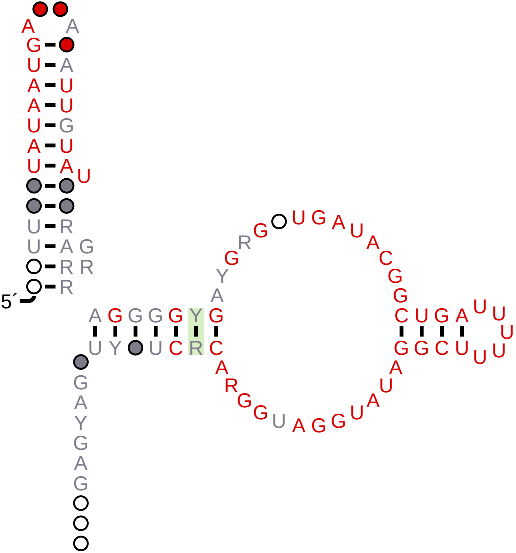

HMP2_13268_1

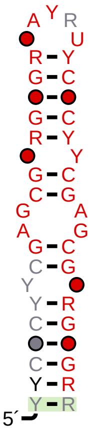

HMP2_13274_1

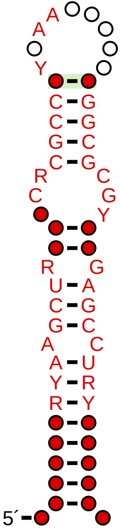

HMP2_13278_1

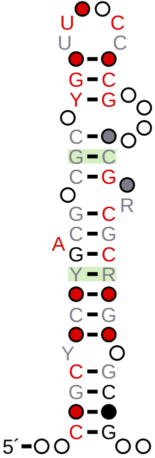

HMP2_13284_1

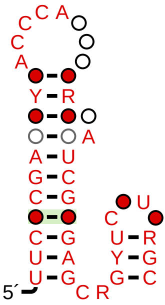

HMP2_13296_1

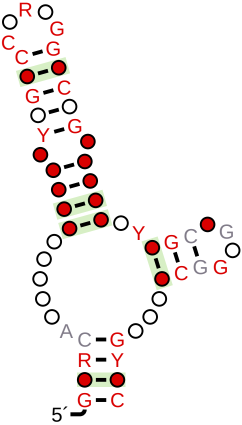

HMP2_13301_1

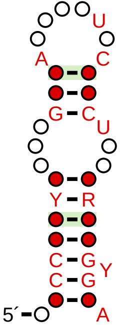

HMP2_13323_1

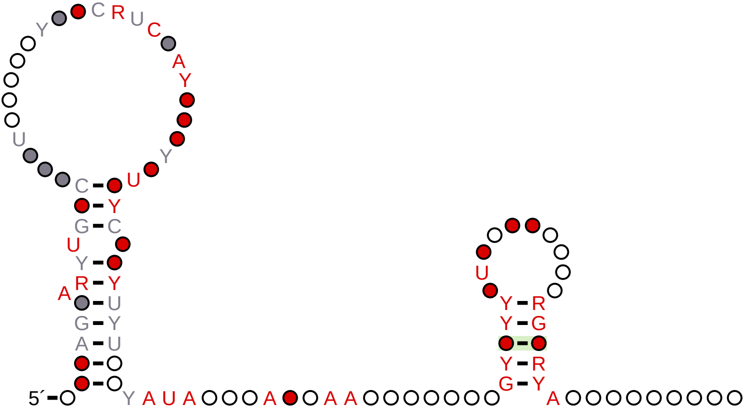

HMP2_13325_1

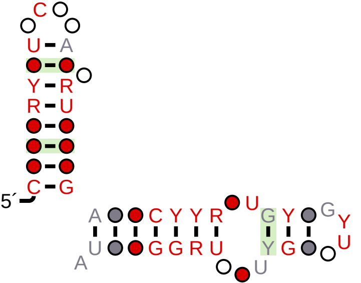

HMP2_13326_1

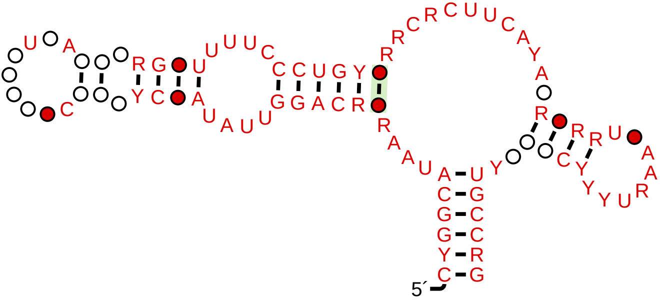

HMP2_1333_1

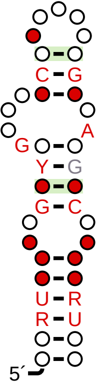

HMP2_1334_1

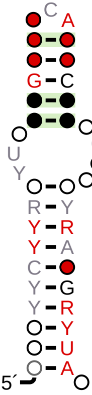

HMP2_13348_1

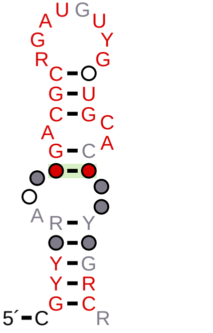

HMP2_13356_1

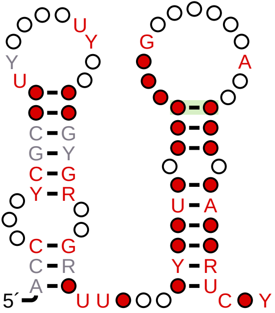

HMP2_13390_1

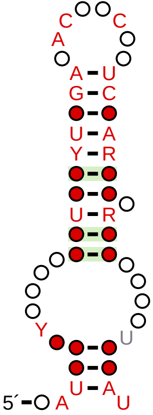

HMP2_13400_1

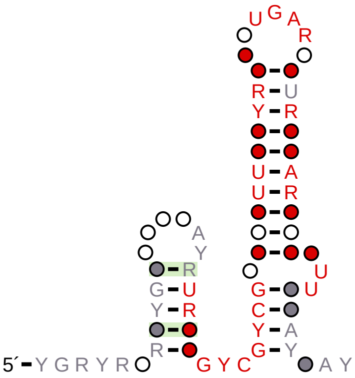

HMP2_13402_1

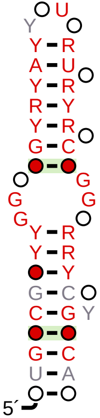

HMP2_13412_1

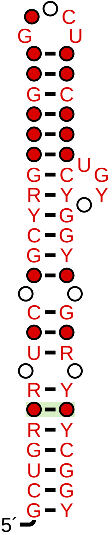

HMP2_13426_1

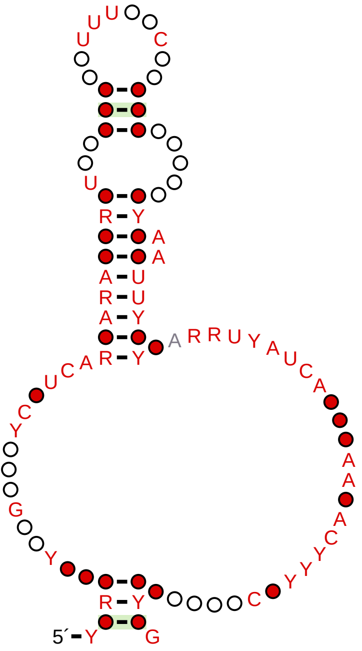

HMP2_13428_1

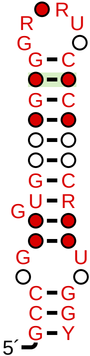

HMP2_13436_1

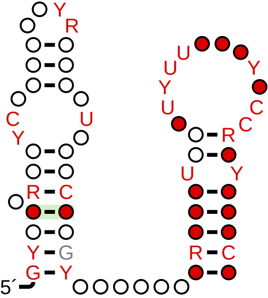

HMP2_13452_1

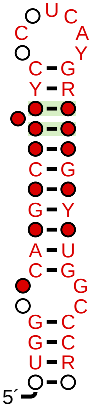

HMP2_13454_1

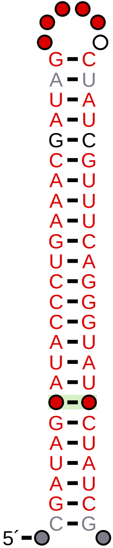

HMP2_13463_1

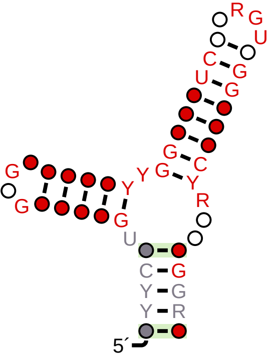

HMP2_13475_1

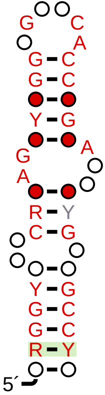

HMP2_13497_1

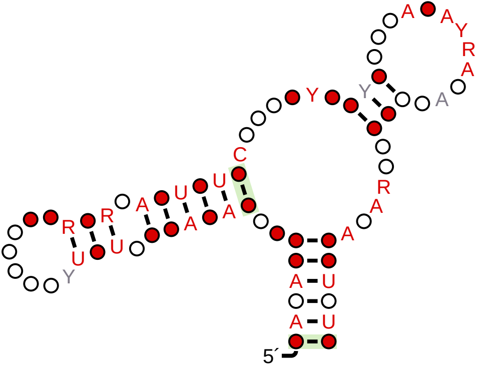

HMP2_13501_1

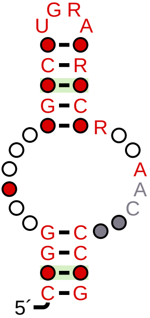

HMP2_13505_1

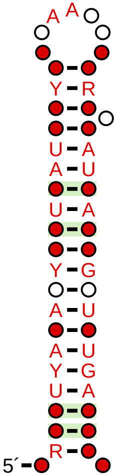

HMP2_13507_1

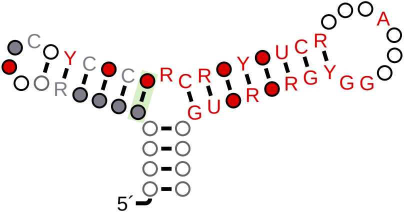

HMP2_13524_1

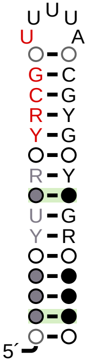

HMP2_13534_1

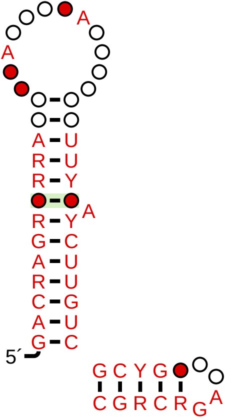

HMP2_13538_1

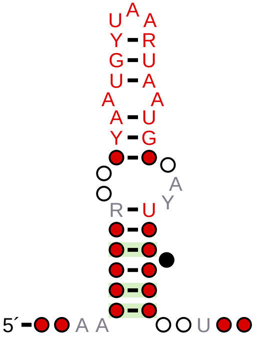

HMP2_13582_1

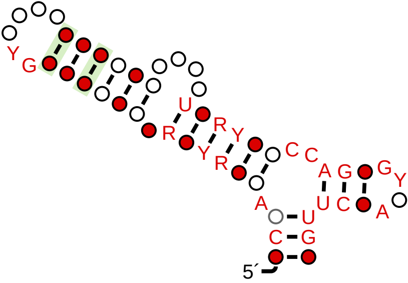

HMP2_13583_1

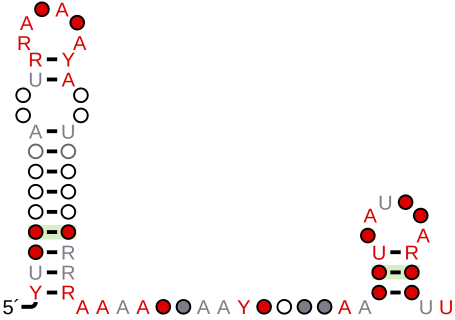

HMP2_13589_1

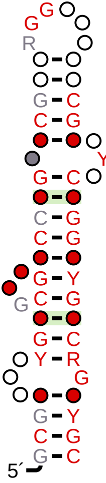

HMP2_13602_1

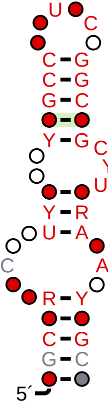

HMP2_13609_1

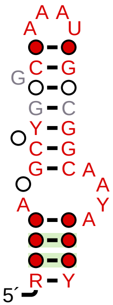

HMP2_13613_1

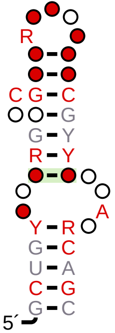

HMP2_13618_1

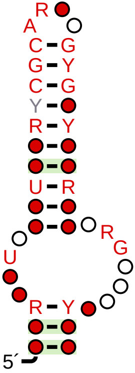

HMP2_1364_1

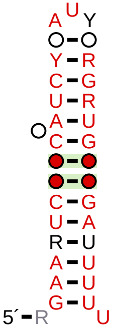

HMP2_13640_1

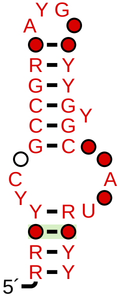

HMP2_13665_1

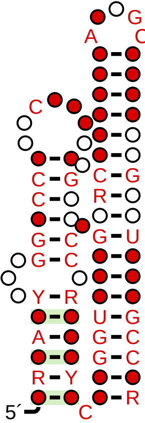

HMP2_13679_1

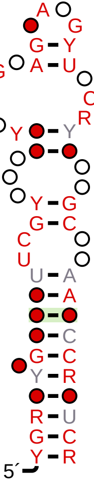

HMP2_13690_1

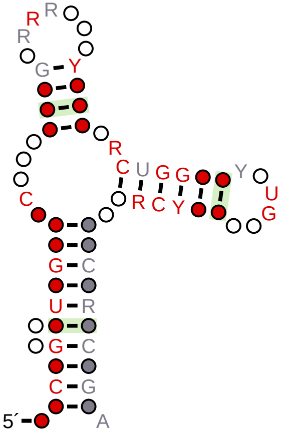

HMP2_13696_1

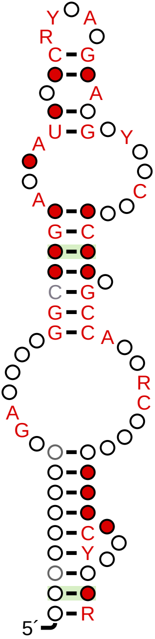

HMP2_13699_1

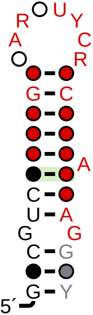

HMP2_137_1

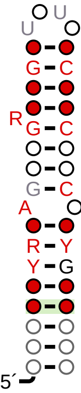

HMP2_1370_1

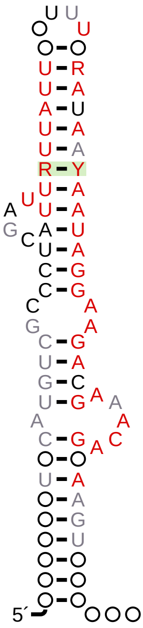

HMP2_13702_1

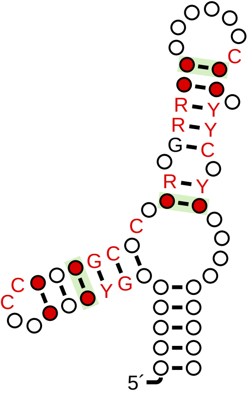

HMP2_13723_1

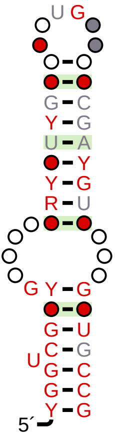

HMP2_13725_1

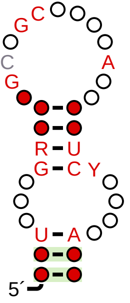

HMP2_13735_1

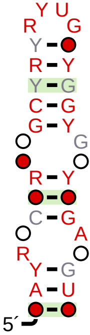

HMP2_13736_1

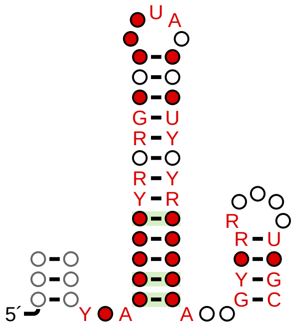

HMP2_13738_1

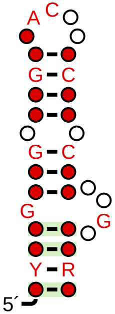

HMP2_13749_1

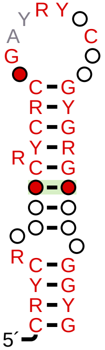

HMP2_13750_1

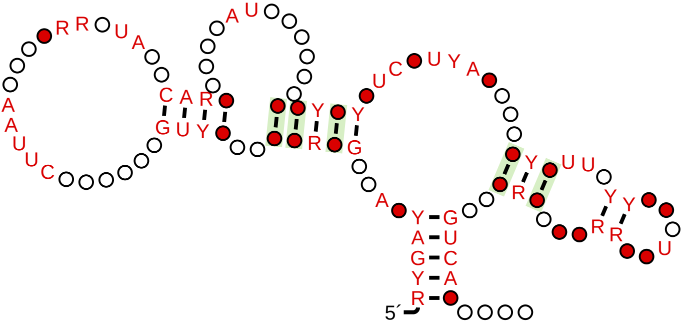

HMP2_13752_1

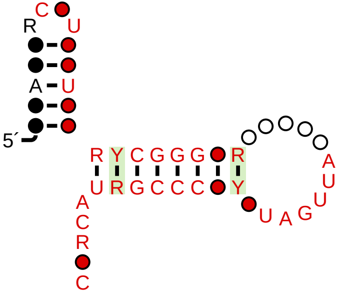

HMP2_13756_1

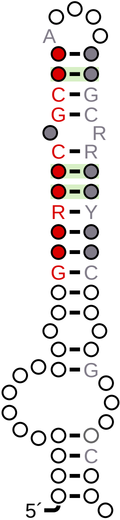

HMP2_13765_1

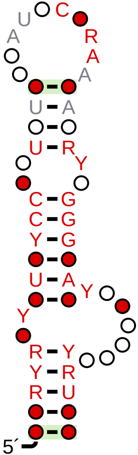

HMP2_13769_1

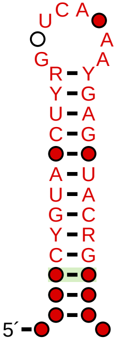

HMP2_13787_1

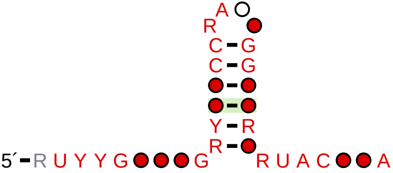

HMP2_13791_1

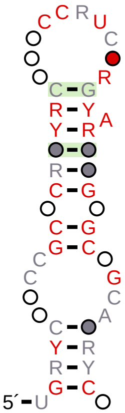

HMP2_13794_1

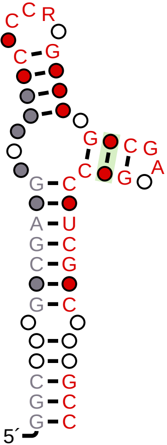

HMP2_13797_1

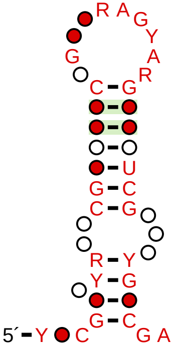

HMP2_1380_1

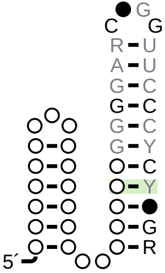

HMP2_13809_1

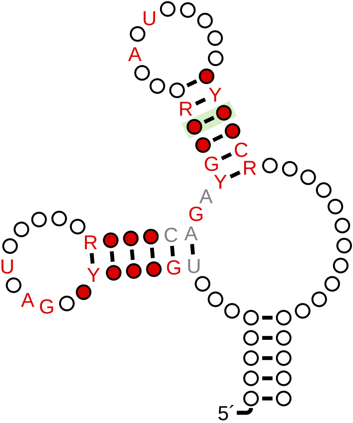

HMP2_13812_1

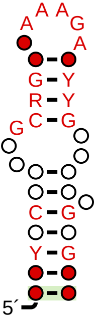

HMP2_13816_1

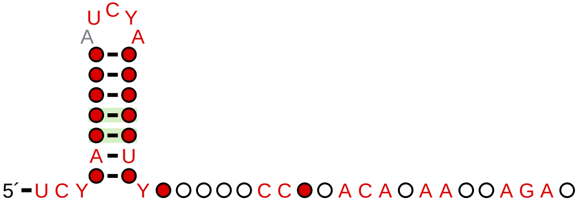

HMP2_13819_1

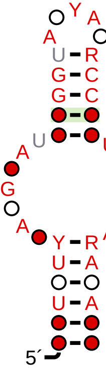

HMP2_13826_1

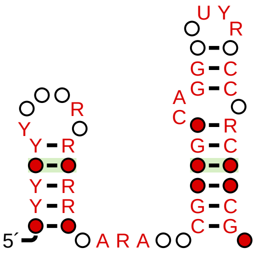

HMP2_13830_1

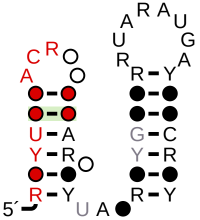

HMP2_13836_1

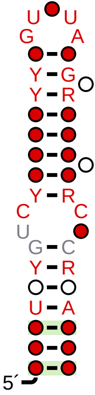

HMP2_13840_1

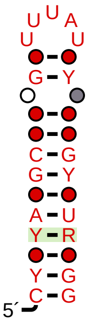

HMP2_13855_1

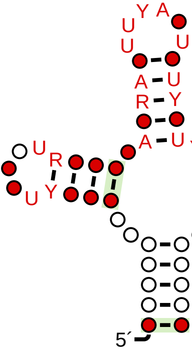

HMP2_1386_1

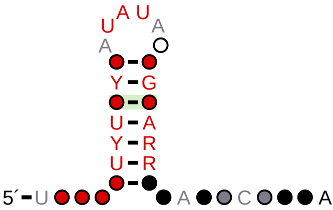

HMP2_13869_1

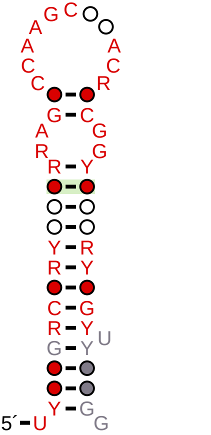

HMP2_13872_1

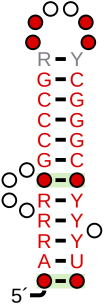

HMP2_1390_1

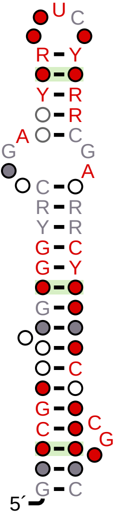

HMP2_13905_1

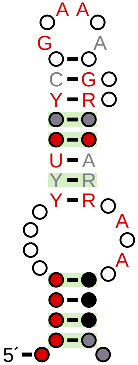

HMP2_13912_1

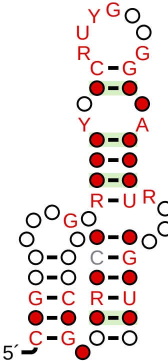

HMP2_13921_1

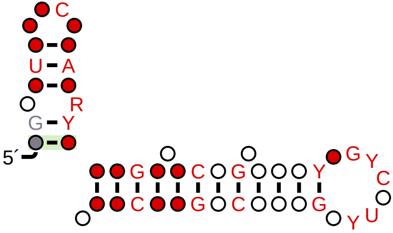

HMP2_13925_1

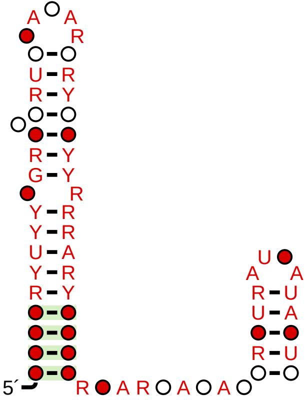

HMP2_13934_1

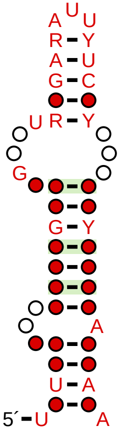

HMP2_13947_1

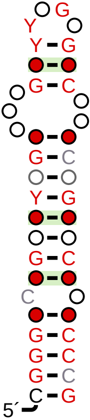

HMP2_13957_1

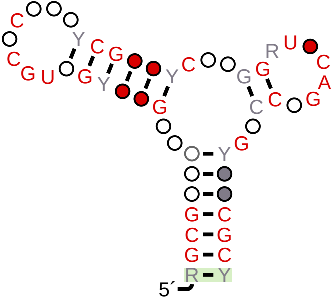

HMP2_13962_1

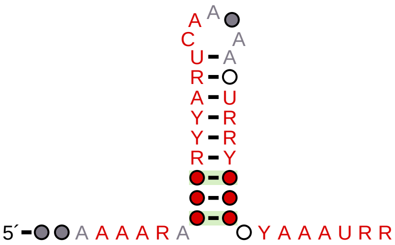

HMP2_13963_1

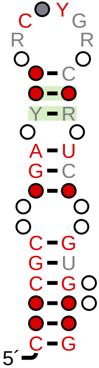

HMP2_13974_1

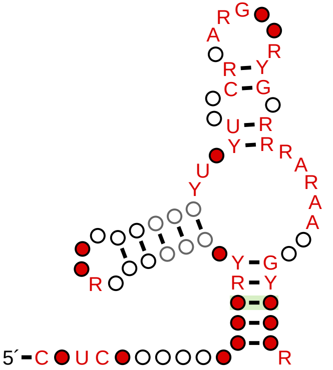

HMP2_13978_1

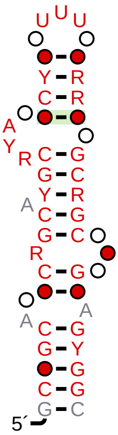

HMP2_13985_1

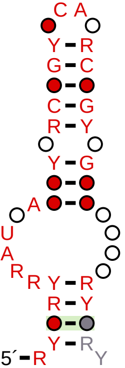

HMP2_13986_1

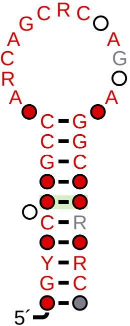

HMP2_13989_1

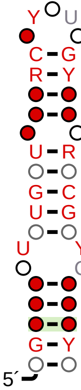

HMP2_13997_1

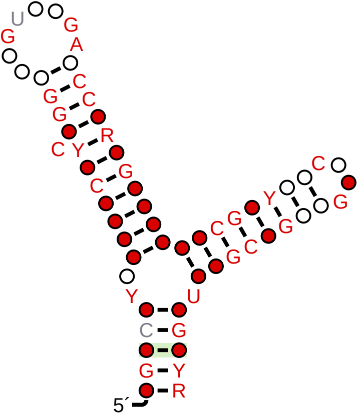

HMP2_14002_1

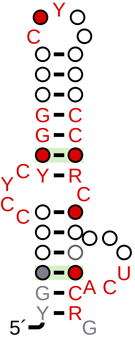

HMP2_14005_1

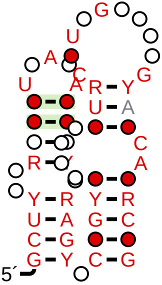

HMP2_14018_1

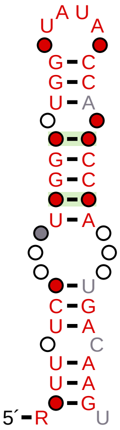

HMP2_14019_1

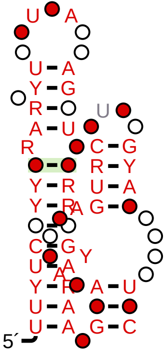

HMP2_14063_1

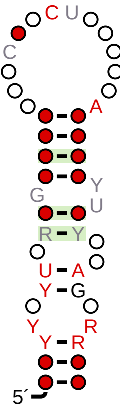

HMP2_14071_1

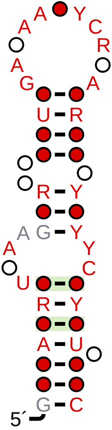

HMP2_14082_1

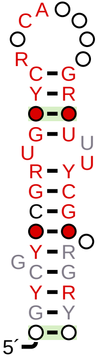

HMP2_14084_1

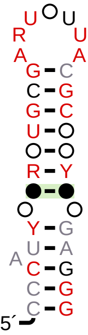

HMP2_14088_1

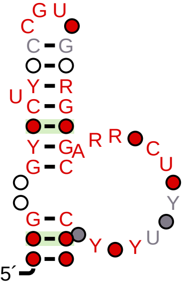

HMP2_14098_1

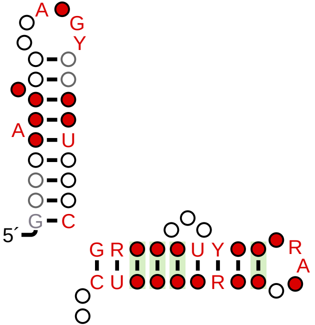

HMP2_14104_1

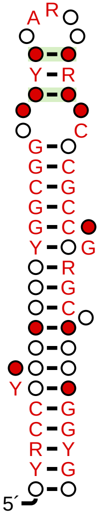

HMP2_14106_1

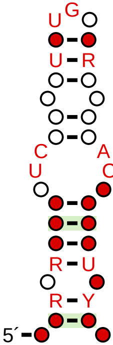

HMP2_14120_1

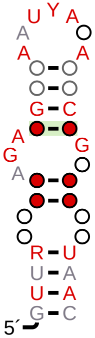

HMP2_14126_1

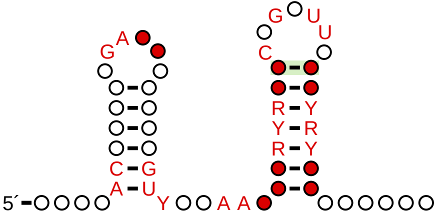

HMP2_14137_1

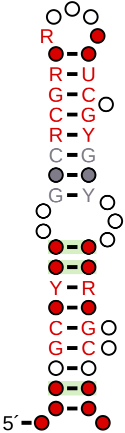

HMP2_14142_1

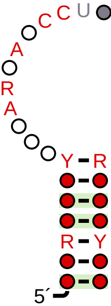

HMP2_14148_1

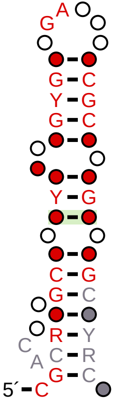

HMP2_14163_1

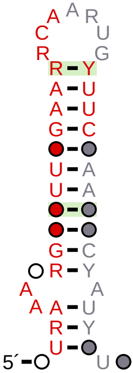

HMP2_14167_1

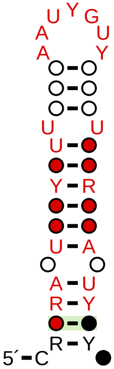

HMP2_14175_1

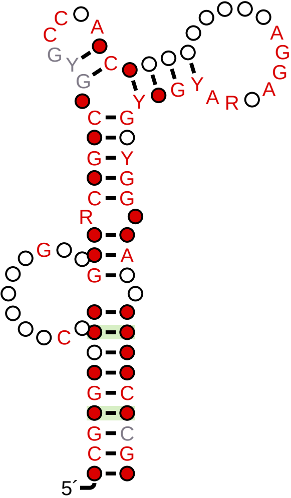

HMP2_14182_1

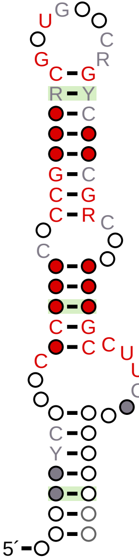

HMP2_14188_1

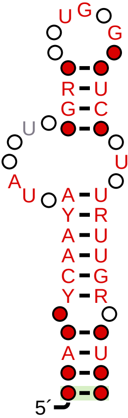

HMP2_14200_1

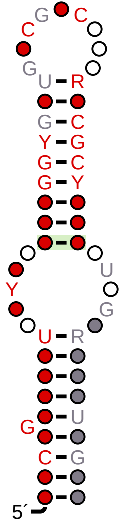

HMP2_14206_1

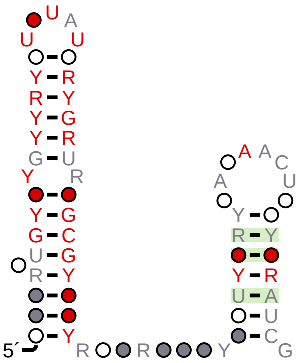

HMP2_14216_1

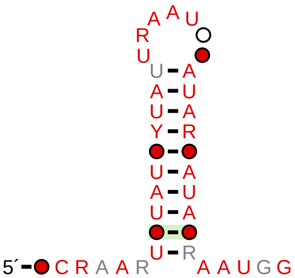

HMP2_14220_1

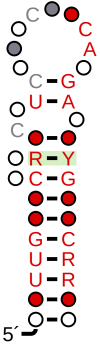

HMP2_14228_1

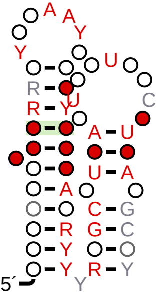

HMP2_14236_1

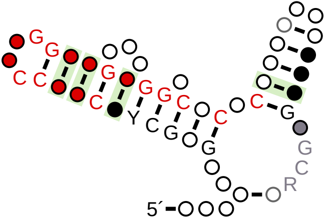

HMP2_14237_1

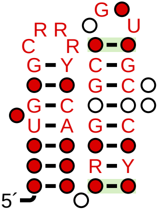

HMP2_14244_1

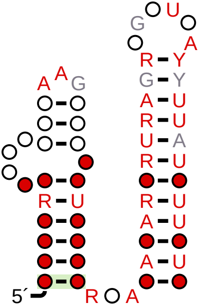

HMP2_14254_1

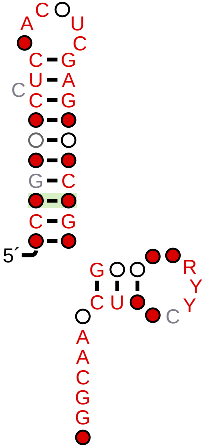

HMP2_14269_1

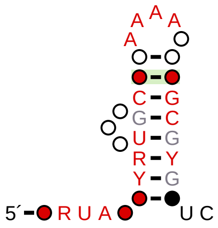

HMP2_14270_1

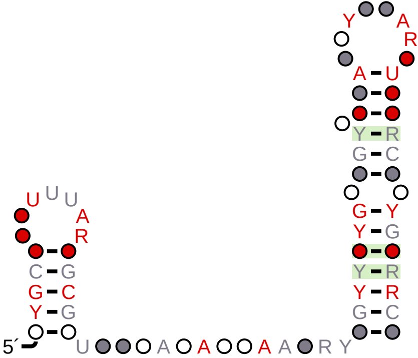

HMP2_14271_1

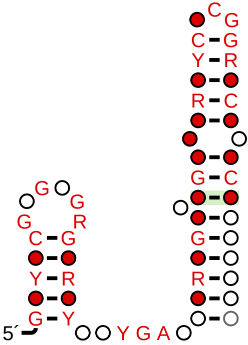

HMP2_14294_1

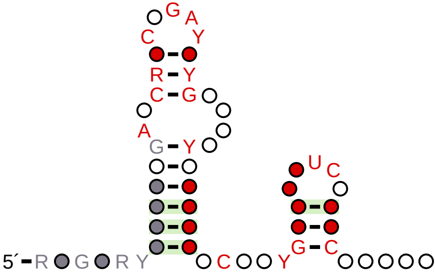

HMP2_14298_1

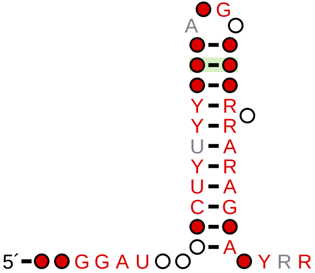

HMP2_143_1

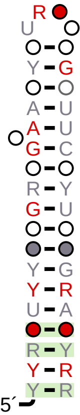

HMP2_1430_1

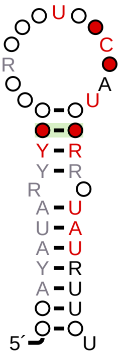

HMP2_14302_1

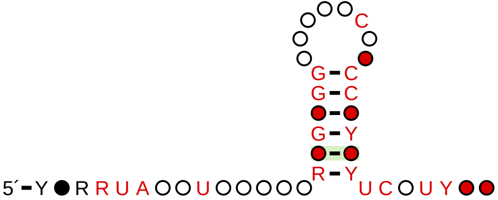

HMP2_14308_1

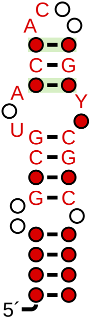

HMP2_14314_1

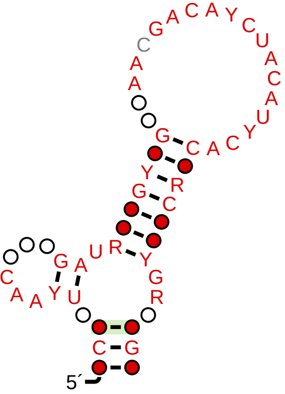

HMP2_14329_1

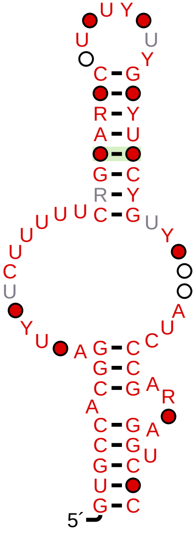

HMP2_1433_1

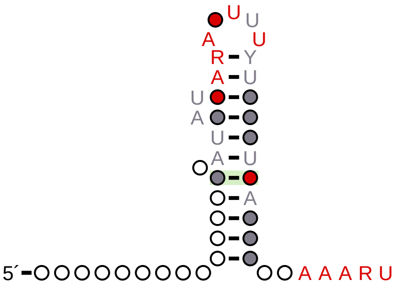

HMP2_14338_1

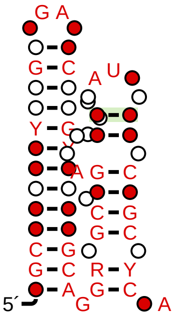

HMP2_14341_1

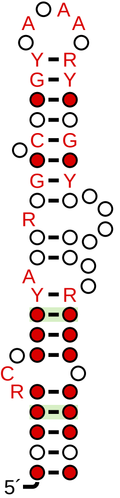

HMP2_14345_1

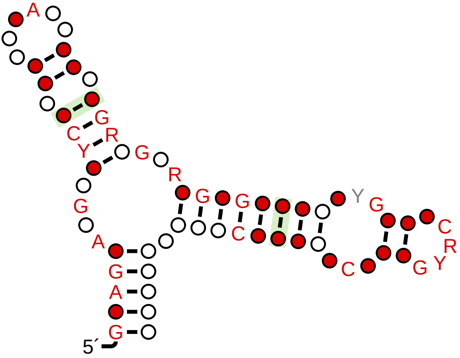

HMP2_14346_1

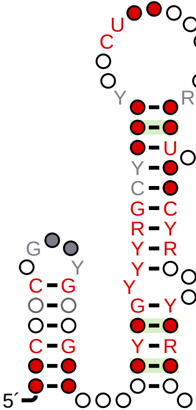

HMP2_14358_1

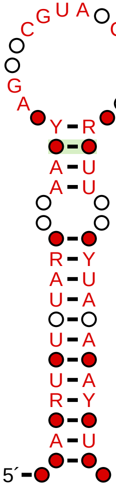

HMP2_14359_1

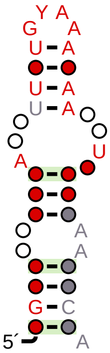

HMP2_14368_1

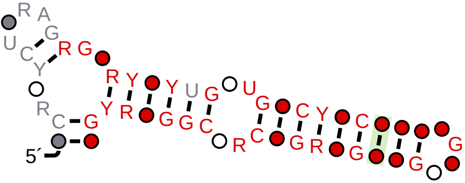

HMP2_14371_1

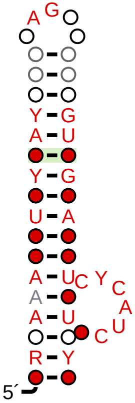

HMP2_14384_1

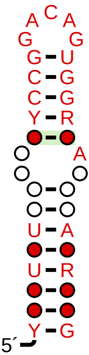

HMP2_14385_1

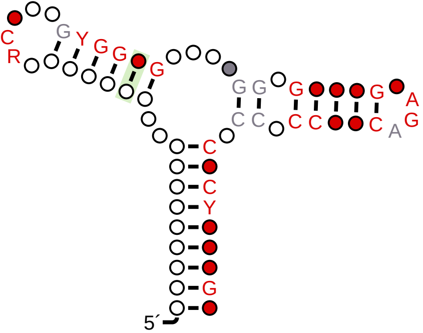

HMP2_14390_1

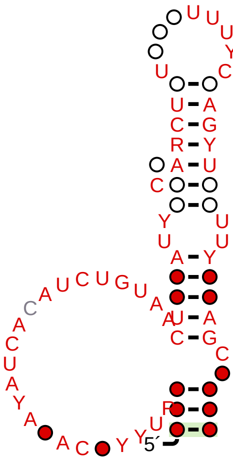

HMP2_14391_1

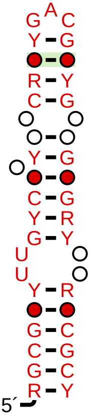

HMP2_14392_1

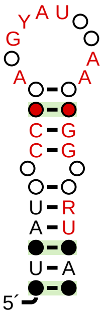

HMP2_14399_1

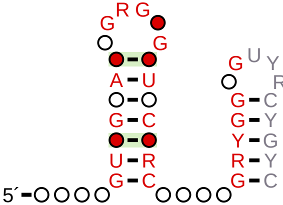

HMP2_14401_1

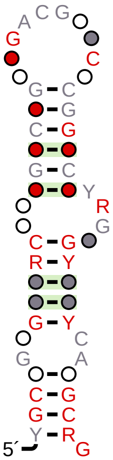

HMP2_14404_1

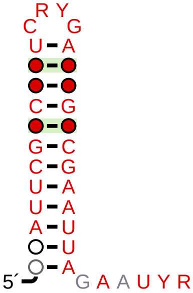

HMP2_14432_1

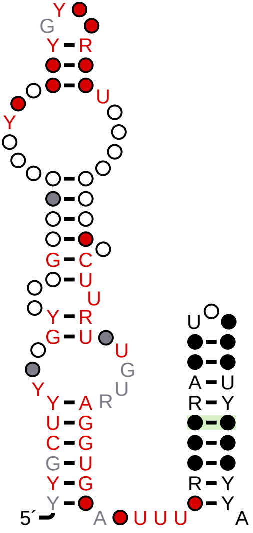

HMP2_14435_1

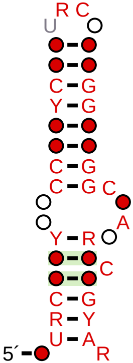

HMP2_14443_1

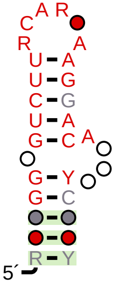

HMP2_14448_1

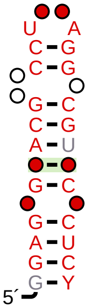

HMP2_14451_1

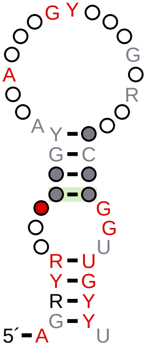

HMP2_14456_1

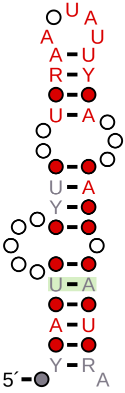

HMP2_14457_1

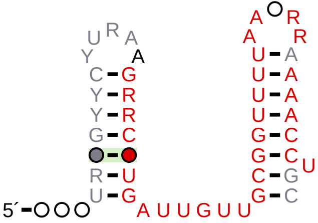

HMP2_14462_1

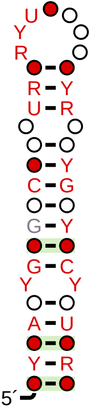

HMP2_14465_1

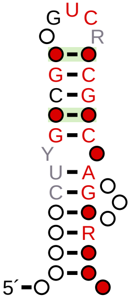

HMP2_14481_1

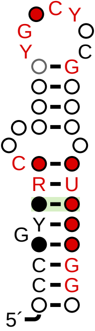

HMP2_14491_1

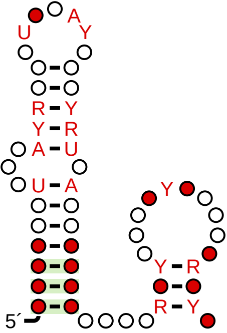

HMP2_14501_1

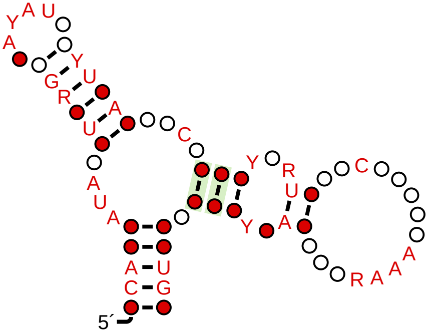

HMP2_1453_1

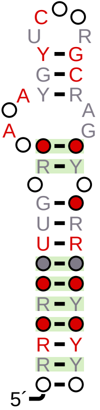

HMP2_14544_1

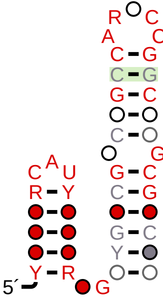

HMP2_14550_1

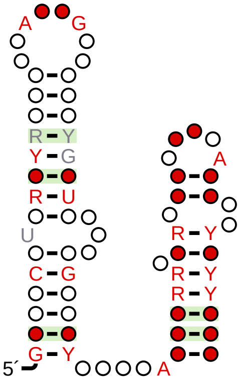

HMP2_14557_1

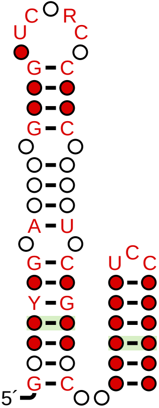

HMP2_14564_1

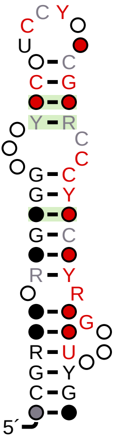

HMP2_14565_1

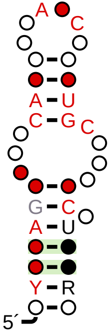

HMP2_14589_1

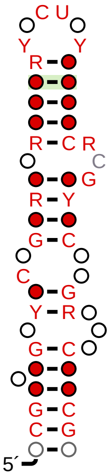

HMP2_14602_1

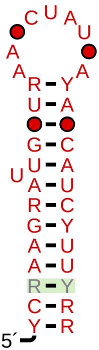

HMP2_14610_1

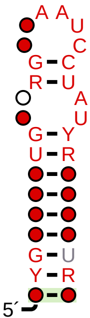

HMP2_14623_1

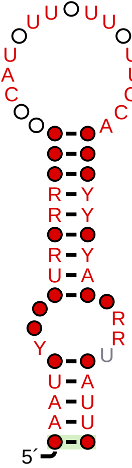

HMP2_14626_1

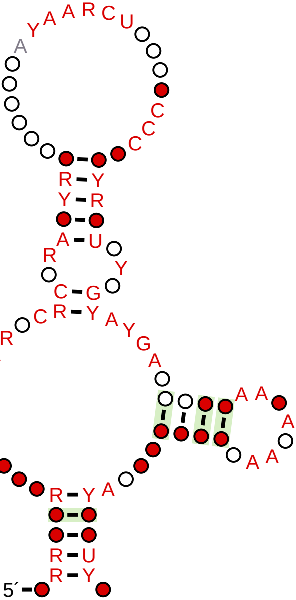

HMP2_14634_1

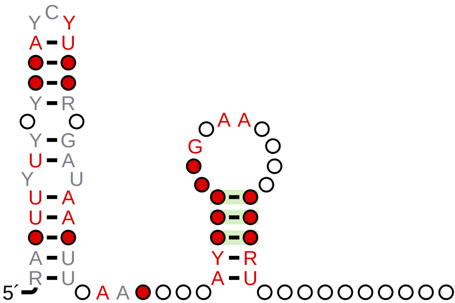

HMP2_14641_1

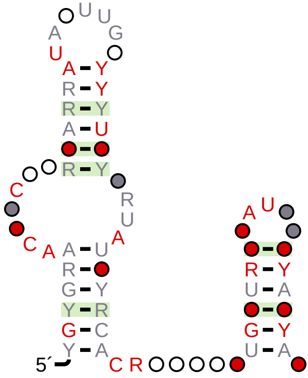

HMP2_14650_1

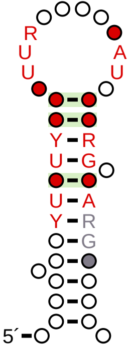

HMP2_14663_1

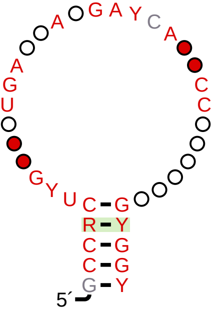

HMP2_14669_1

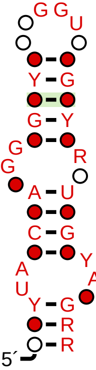

HMP2_14671_1

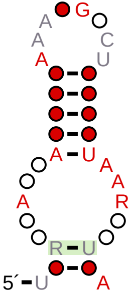

HMP2_14679_1

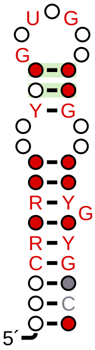

HMP2_14690_1

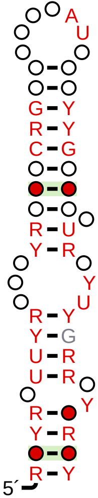

HMP2_14703_1

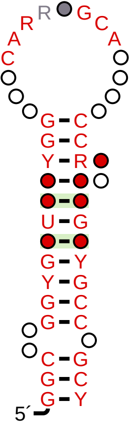

HMP2_14706_1

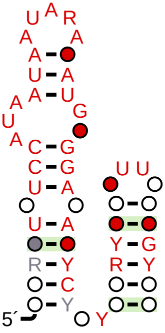

HMP2_14710_1

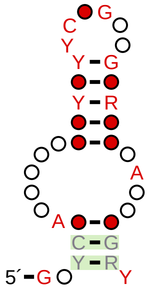

HMP2_14715_1

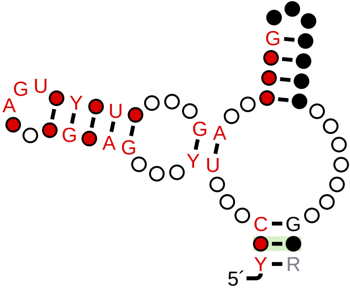

HMP2_14716_1

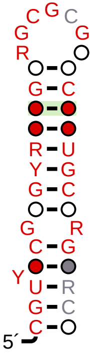

HMP2_14729_1

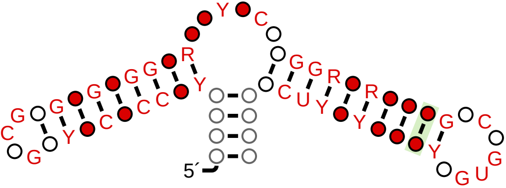

HMP2_14732_1

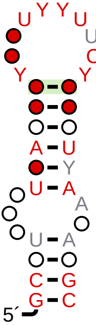

HMP2_14735_1

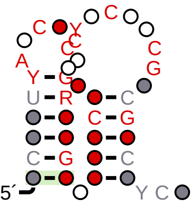

HMP2_14738_1

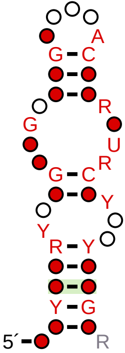

HMP2_14739_1

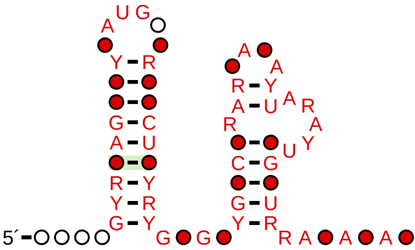

HMP2_14749_1

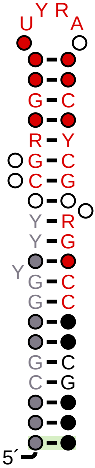

HMP2_14761_1

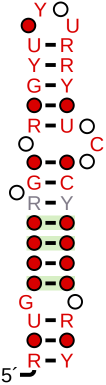

HMP2_14764_1

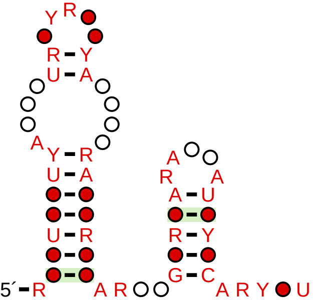

HMP2_14766_1

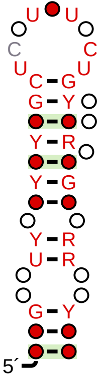

HMP2_14767_1

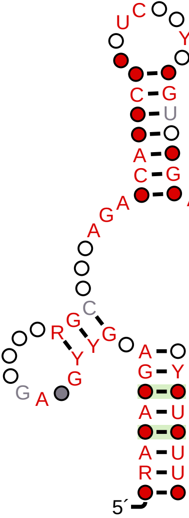

HMP2_14768_1

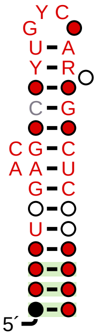

HMP2_1478_1

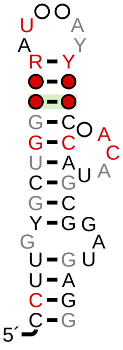

HMP2_14781_1

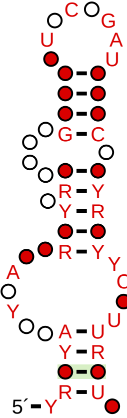

HMP2_14789_1

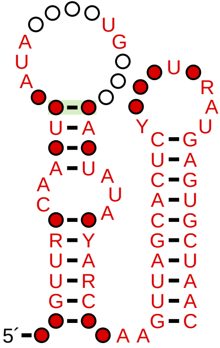

HMP2_1480_1

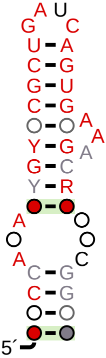

HMP2_14807_1

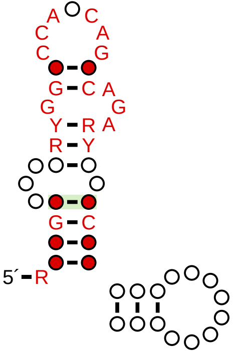

HMP2_14810_1

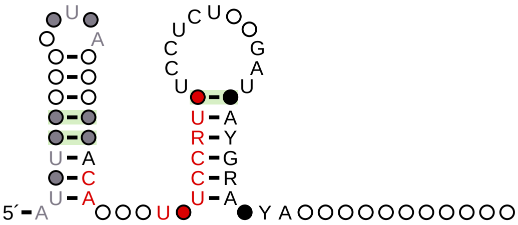

HMP2_14813_1

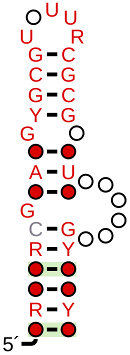

HMP2_14815_1

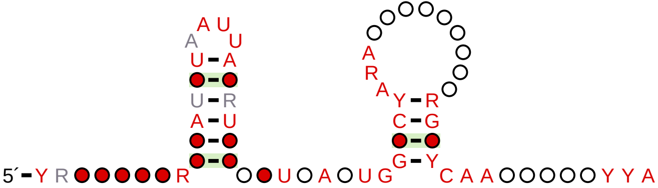

HMP2_14837_1

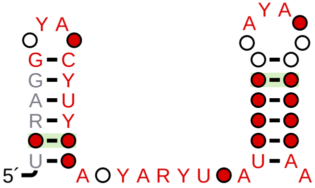

HMP2_14846_1

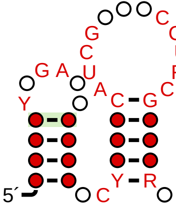

HMP2_1485_1

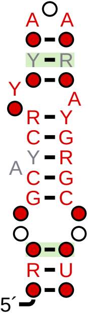

HMP2_14861_1

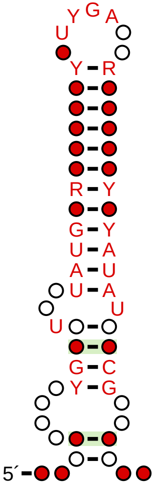

HMP2_14862_1

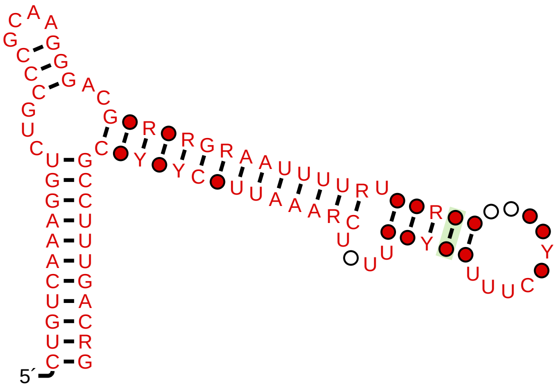

HMP2_14866_1

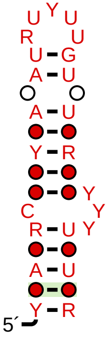

HMP2_14874_1

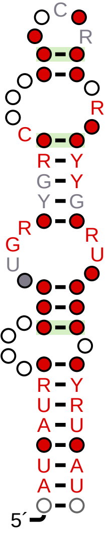

HMP2_14875_1

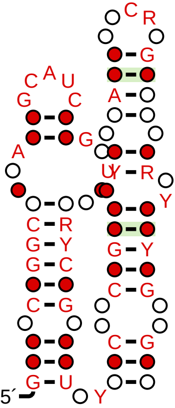

HMP2_14877_1

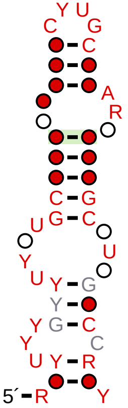

HMP2_14893_1

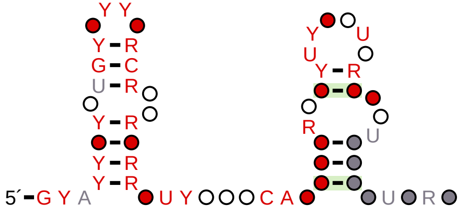

HMP2_14896_1

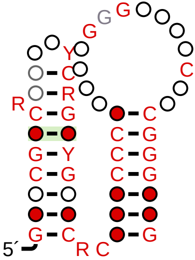

HMP2_14900_1

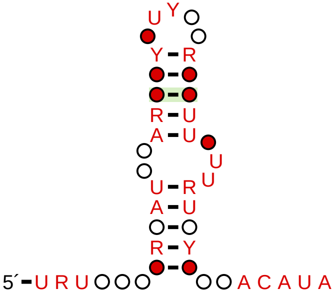

HMP2_14901_1

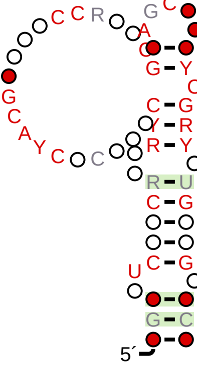

HMP2_14904_1

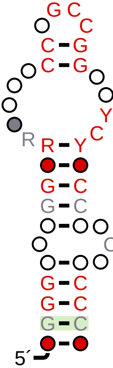

HMP2_14922_1

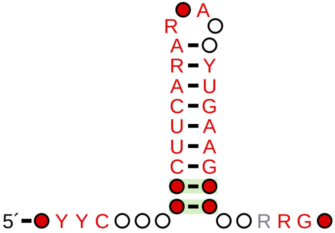

HMP2_14925_1

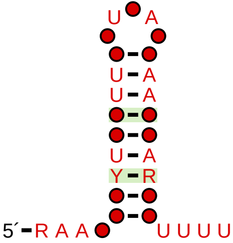

HMP2_14937_1

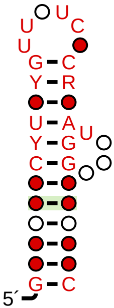

HMP2_14949_1

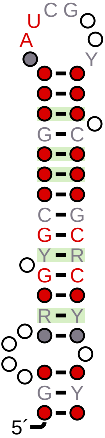

HMP2_14950_1

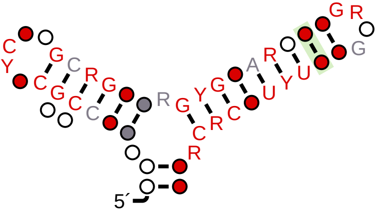

HMP2_14952_1

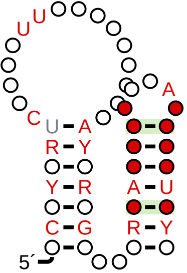

HMP2_14963_1

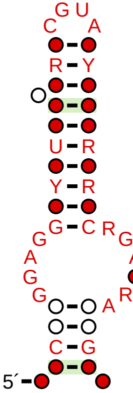

HMP2_14964_1

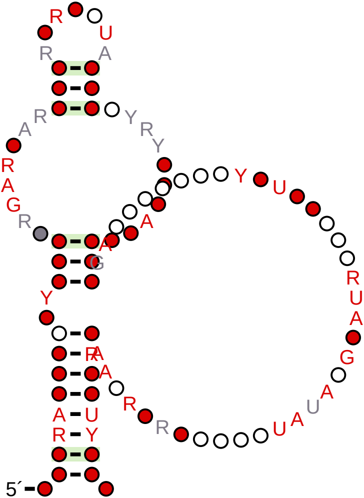

HMP2_14978_1

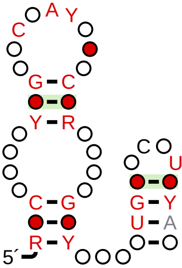

HMP2_14983_1

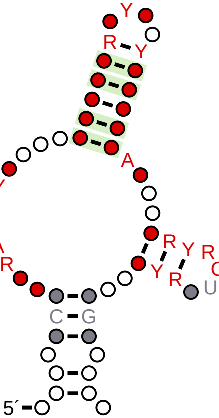

HMP2_14989_1

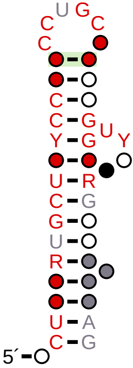

HMP2_15010_1

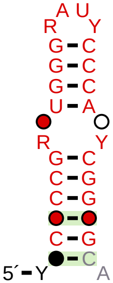

HMP2_15012_1

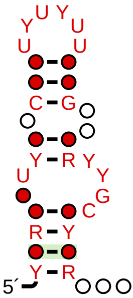

HMP2_15014_1

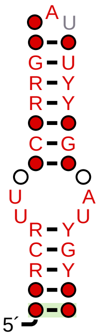

HMP2_15020_1

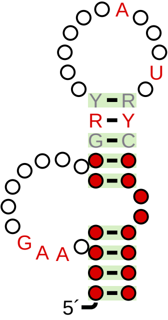

HMP2_1503_1

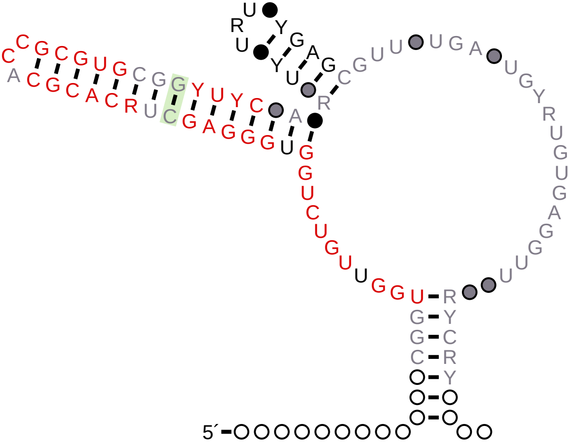

HMP2_15031_1

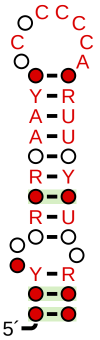

HMP2_15038_1

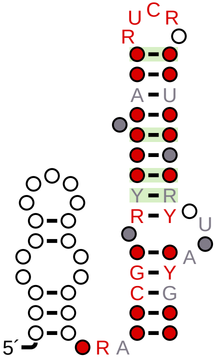

HMP2_15041_1

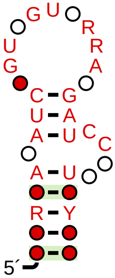

HMP2_15049_1

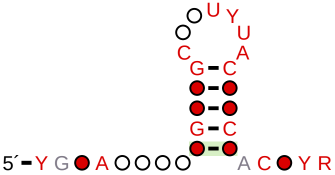

HMP2_15050_1

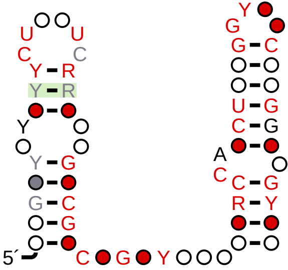

HMP2_15056_1

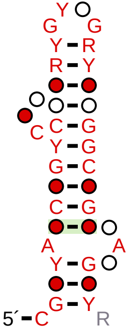

HMP2_15063_1

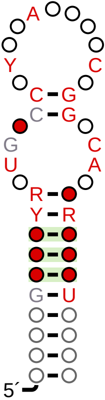

HMP2_15072_1

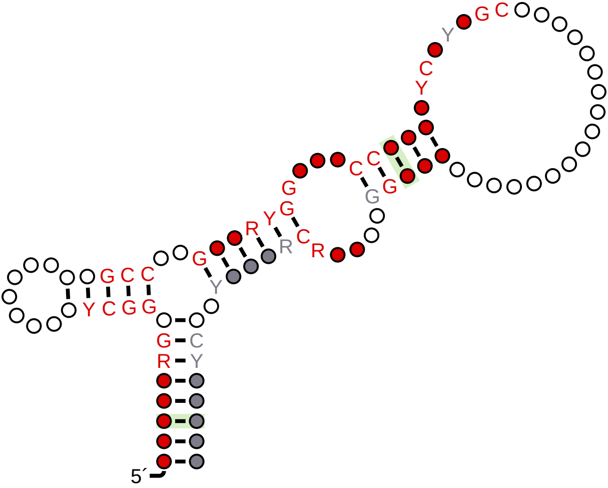

HMP2_15085_1

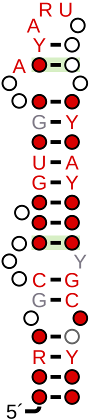

HMP2_15086_1

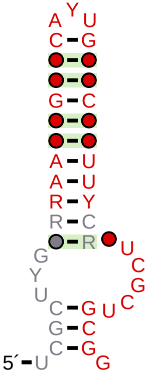

HMP2_15097_1

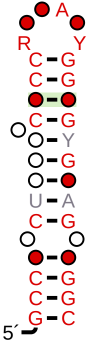

HMP2_15105_1

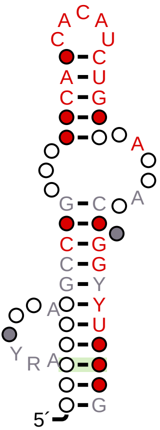

HMP2_15115_1

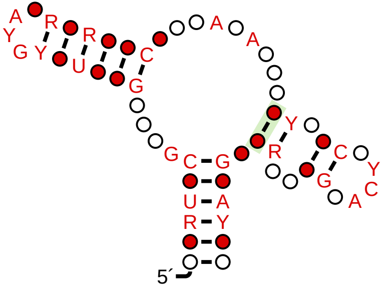

HMP2_15120_1

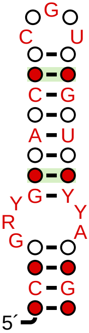

HMP2_15131_1

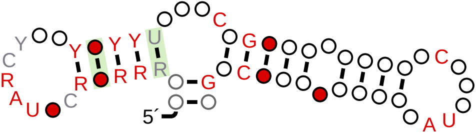

HMP2_15132_1

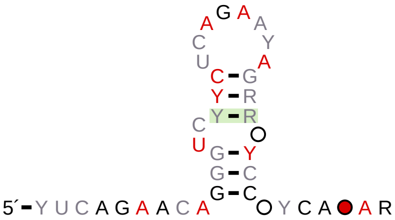

HMP2_15140_1

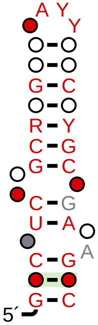

HMP2_15152_1

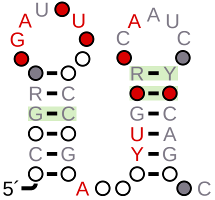

HMP2_15154_1

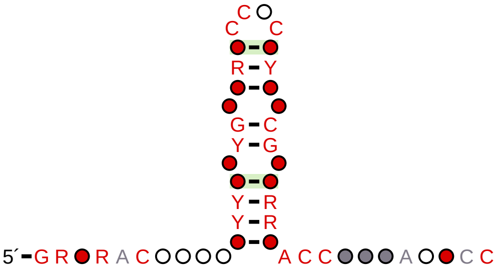

HMP2_15155_1

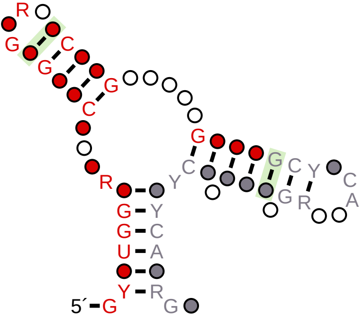

HMP2_15166_1

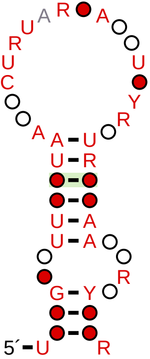

HMP2_15175_1

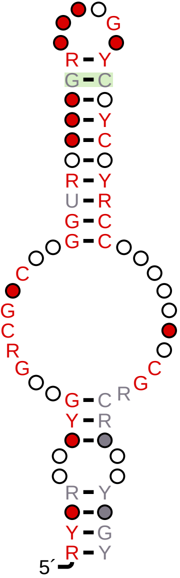

HMP2_15180_1

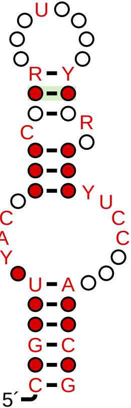

HMP2_15198_1

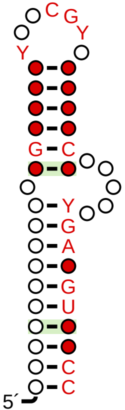

HMP2_1520_1

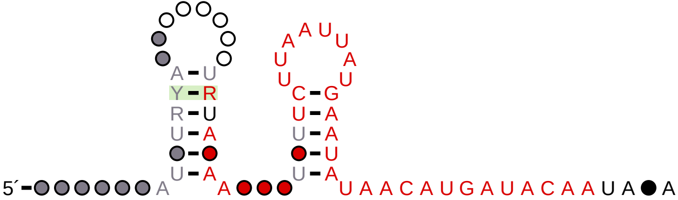

HMP2_15207_1

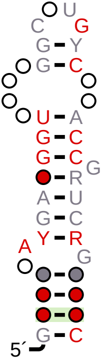

HMP2_15208_1

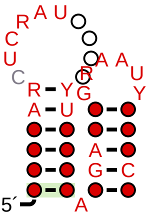

HMP2_15217_1

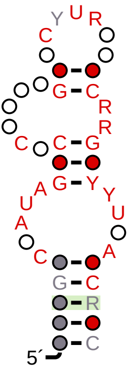

HMP2_15224_1

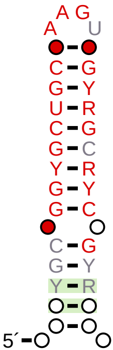

HMP2_1523_1

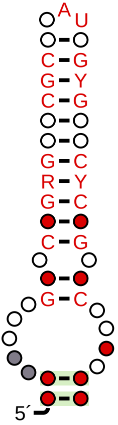

HMP2_15232_1

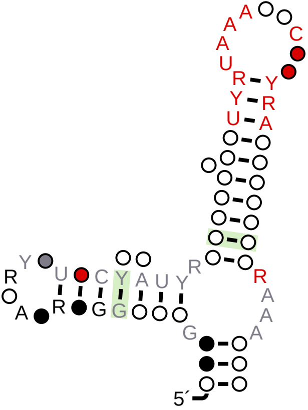

HMP2_15233_1

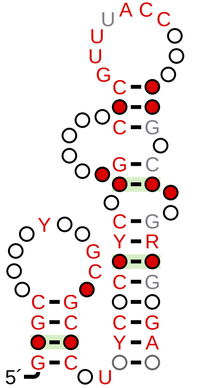

HMP2_15234_1

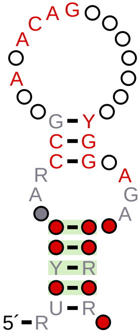

HMP2_15244_1

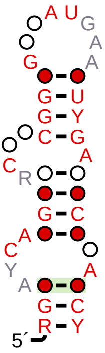

HMP2_15248_1

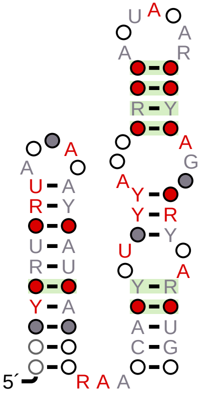

HMP2_15254_1

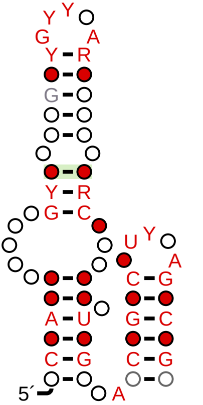

HMP2_15257_1

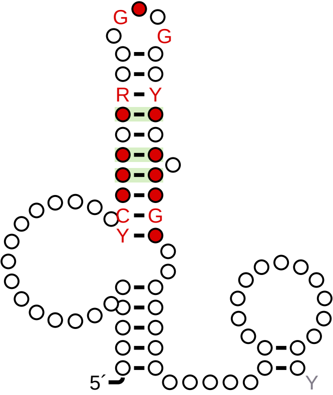

HMP2_1526_1

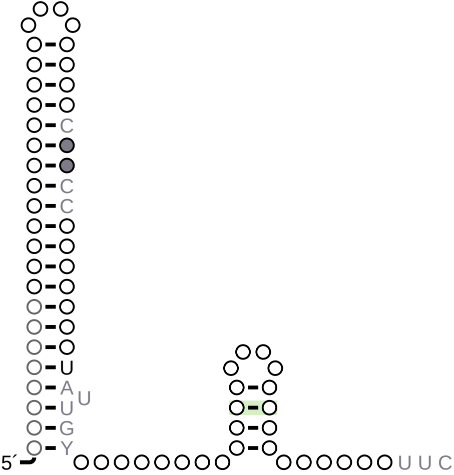

HMP2_15262_1

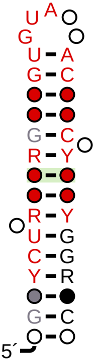

HMP2_15263_1

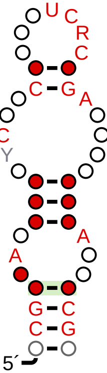

HMP2_15286_1

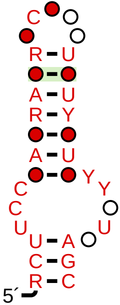

HMP2_15300_1

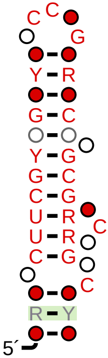

HMP2_15305_1

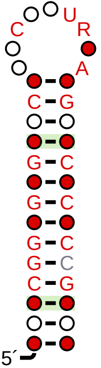

HMP2_15308_1

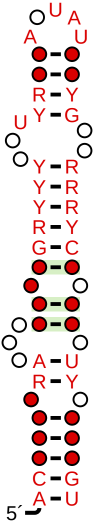

HMP2_15330_1

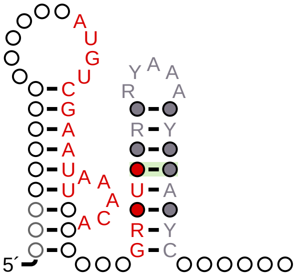

HMP2_15331_1

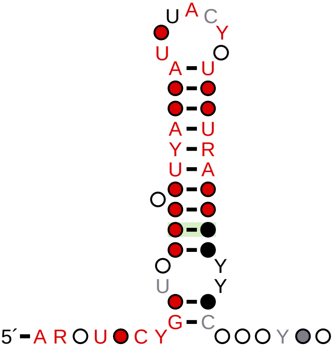

HMP2_15333_1

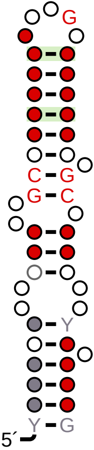

HMP2_15335_1

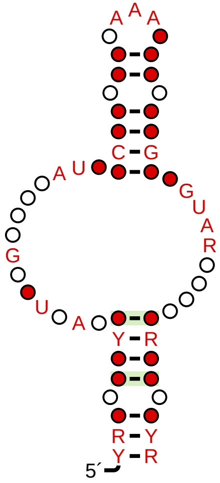

HMP2_15347_1

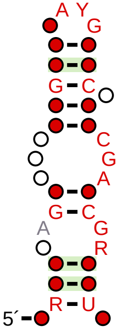

HMP2_15349_1

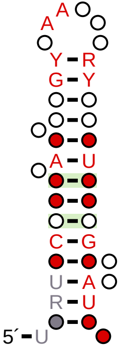

HMP2_15358_1

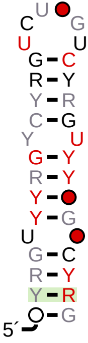

HMP2_15366_1

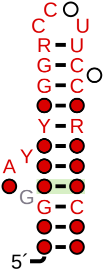

HMP2_15374_1

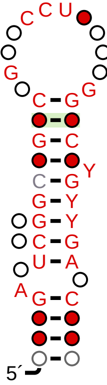

HMP2_15375_1

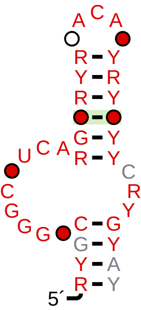

HMP2_15378_1

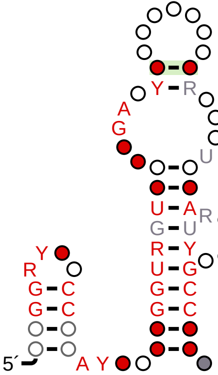

HMP2_15380_1

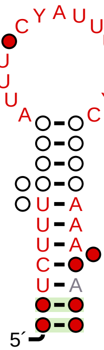

HMP2_154_1

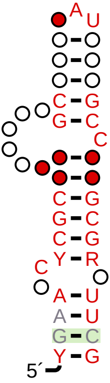

HMP2_15401_1

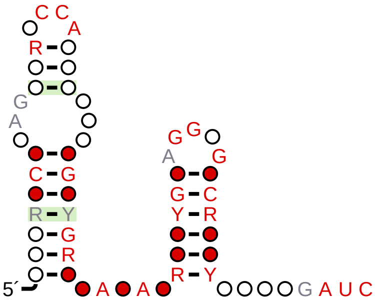

HMP2_15410_1

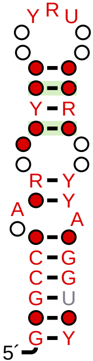

HMP2_15411_1

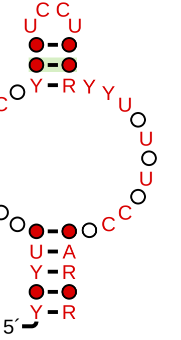

HMP2_15412_1

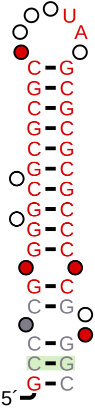

HMP2_15415_1

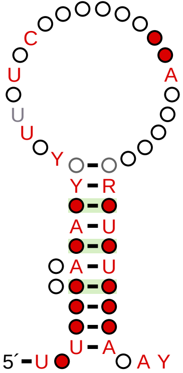

HMP2_15436_1

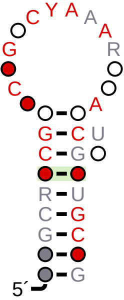

HMP2_15447_1

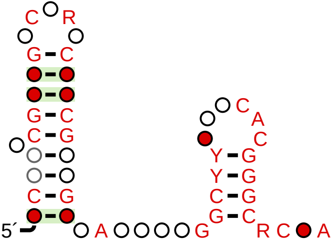

HMP2_15451_1

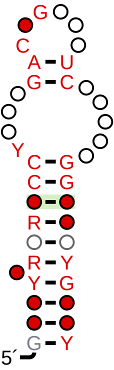

HMP2_15456_1

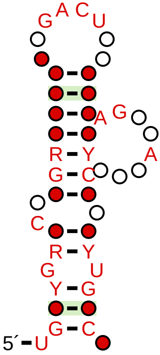

HMP2_1547_1

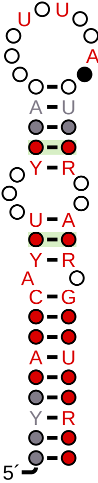

HMP2_15474_1

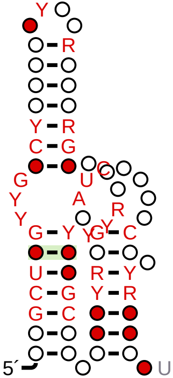

HMP2_15476_1

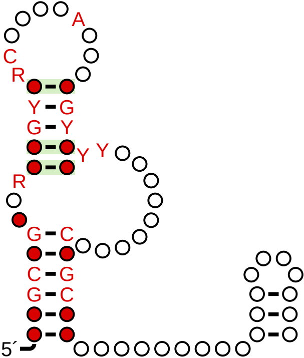

HMP2_15486_1

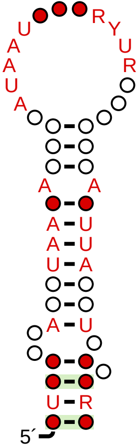

HMP2_15490_1

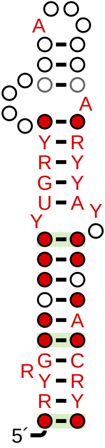

HMP2_15494_1

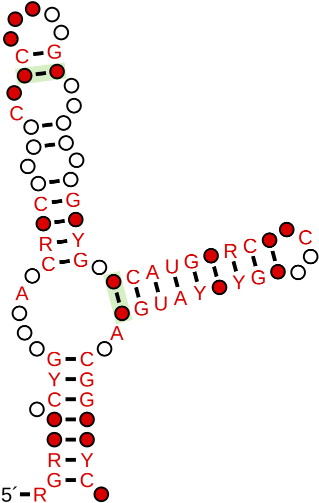

HMP2_15503_1

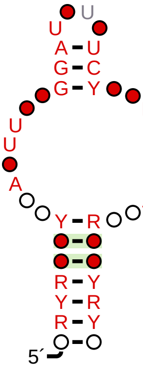

HMP2_15508_1

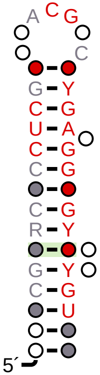

HMP2_15511_1

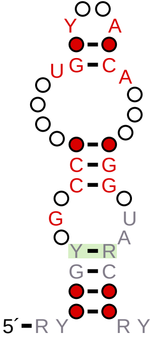

HMP2_15516_1

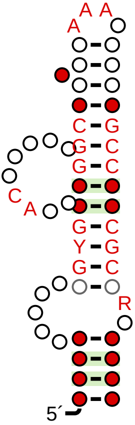

HMP2_15529_1

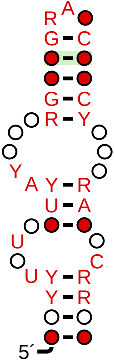

HMP2_15535_1

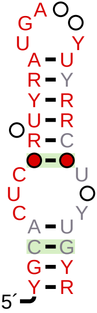

HMP2_15536_1

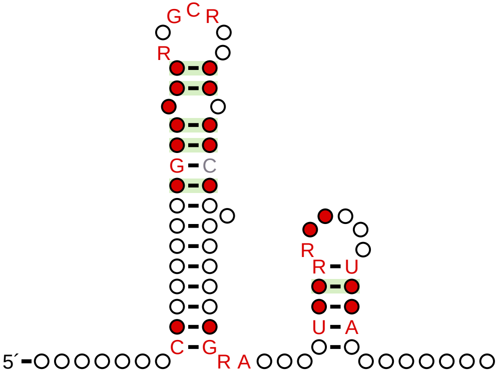

HMP2_15569_1

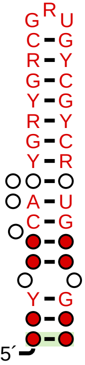

HMP2_15570_1

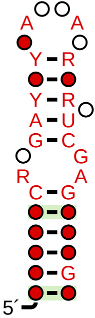

HMP2_15573_1

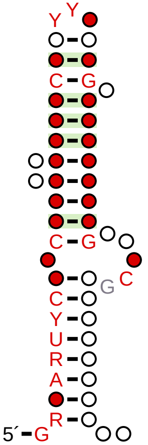

HMP2_15578_1

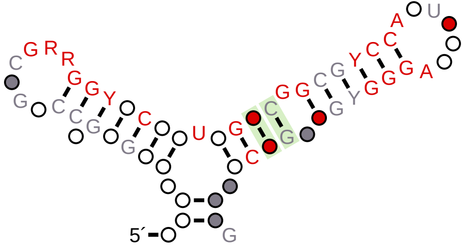

HMP2_15579_1

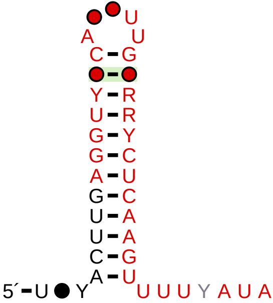

HMP2_1559_1

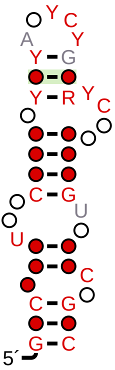

HMP2_15596_1

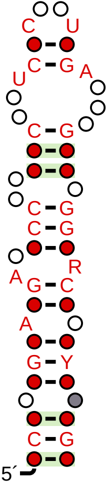

HMP2_15609_1

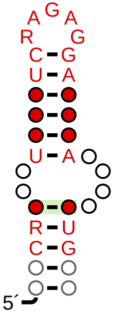

HMP2_15633_1

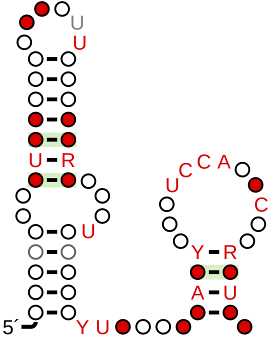

HMP2_15639_1

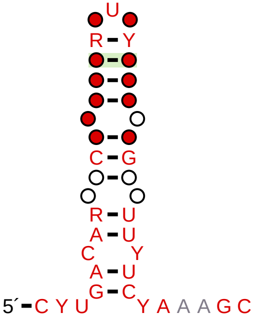

HMP2_15640_1

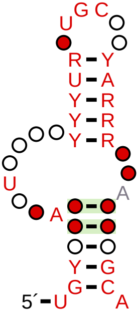

HMP2_15643_1

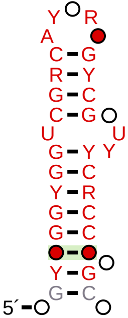

HMP2_15663_1

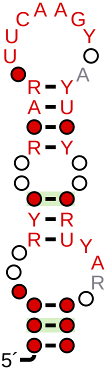

HMP2_15668_1

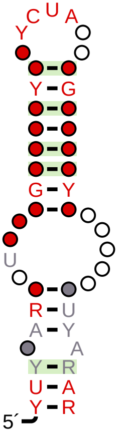

HMP2_15679_1

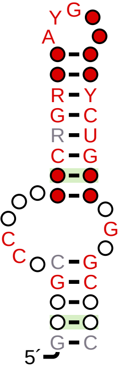

HMP2_15693_1

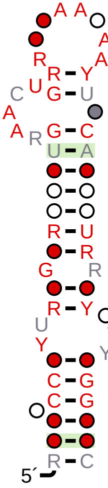

HMP2_15694_1

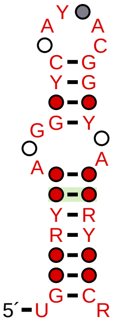

HMP2_15708_1

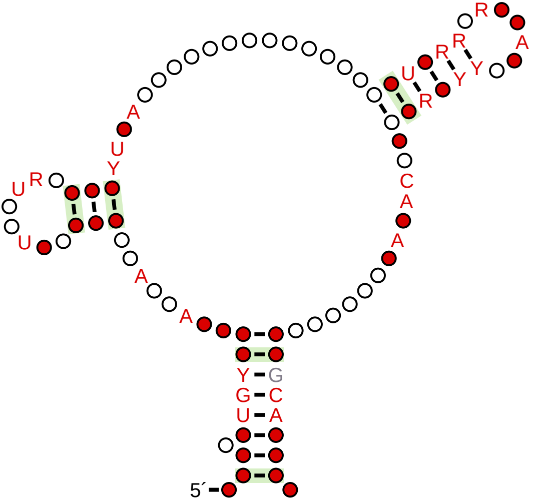

HMP2_1571_1

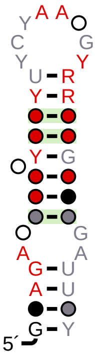

HMP2_15711_1

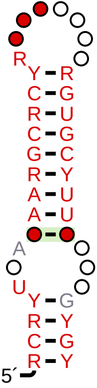

HMP2_15717_1

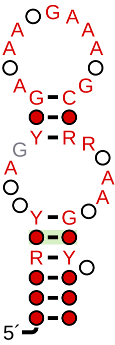

HMP2_15718_1

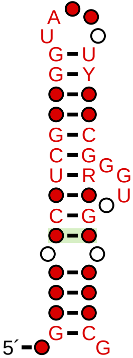

HMP2_15742_1

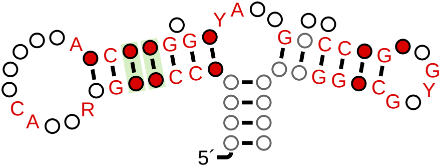

HMP2_15753_1

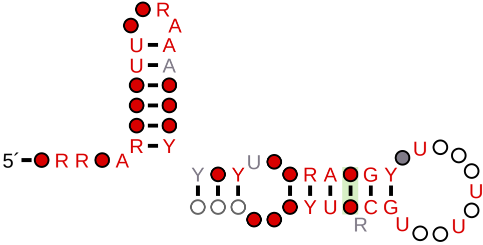

HMP2_15754_1

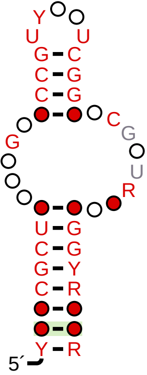

HMP2_15766_1

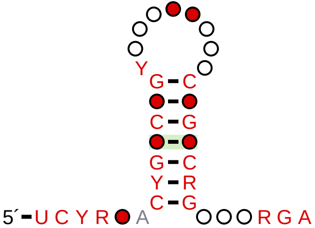

HMP2_15773_1

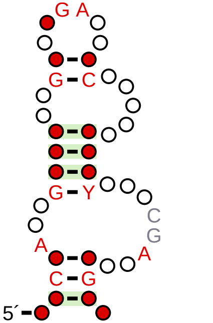

HMP2_15775_1

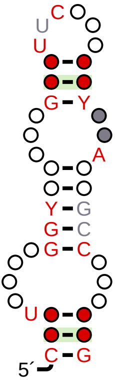

HMP2_15778_1

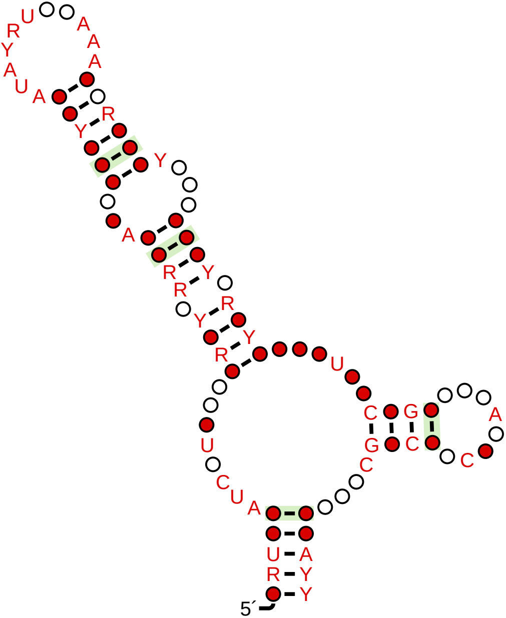

HMP2_15785_1

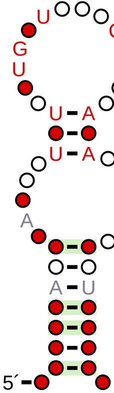

HMP2_15802_1

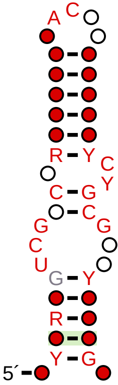

HMP2_15808_1

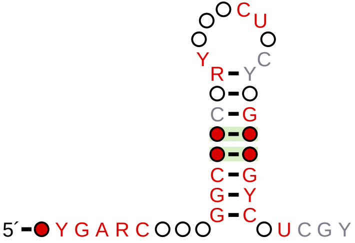

HMP2_15813_1

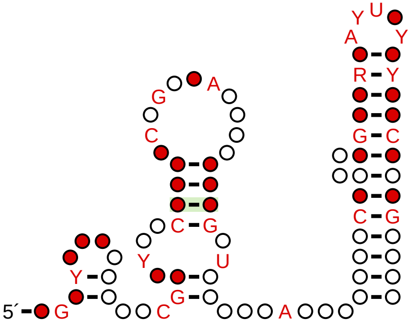

HMP2_15815_1

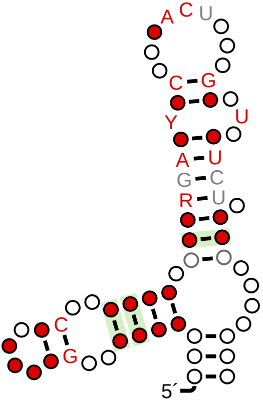

HMP2_15822_1

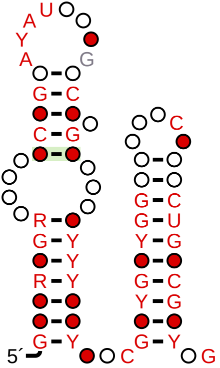

HMP2_15825_1

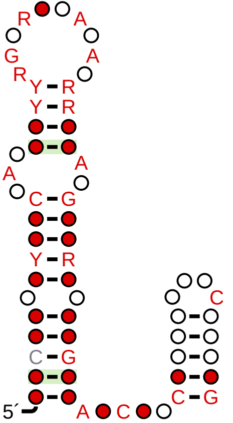

HMP2_15828_1

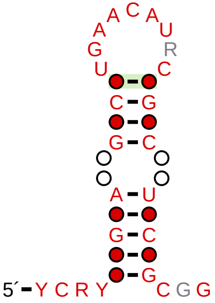

HMP2_15843_1

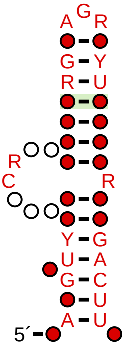

HMP2_15873_1

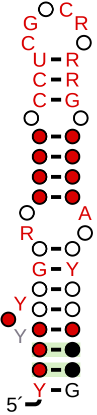

HMP2_15877_1

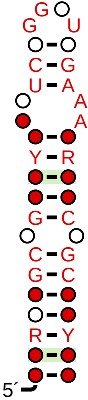

HMP2_15897_1

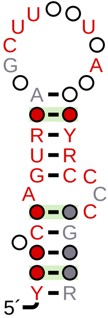

HMP2_15906_1

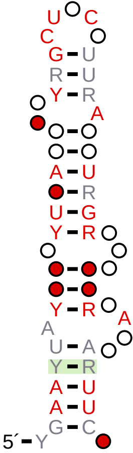

HMP2_15915_1

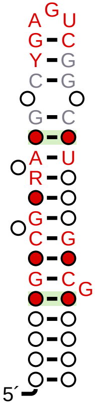

HMP2_15922_1

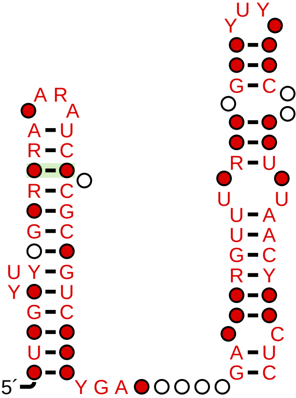

HMP2_15936_1

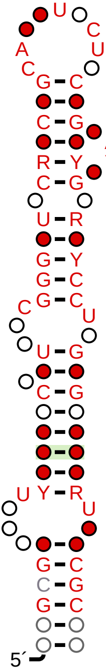

HMP2_15942_1

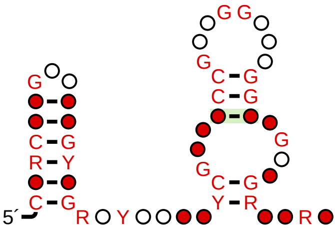

HMP2_15946_1

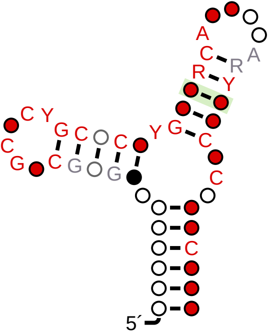

HMP2_1596_1

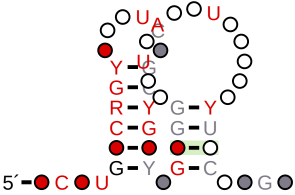

HMP2_15962_1

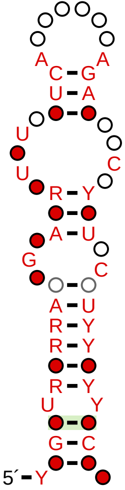

HMP2_15963_1

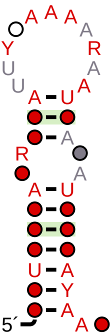

HMP2_15967_1

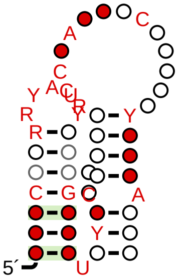

HMP2_15968_1

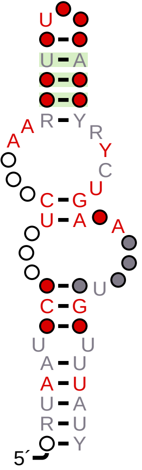

HMP2_15970_1

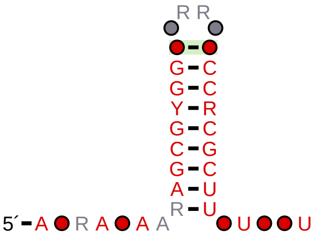

HMP2_15973_1

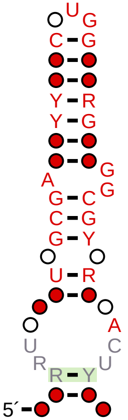

HMP2_15981_1

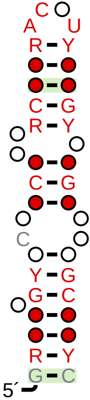

HMP2_15984_1

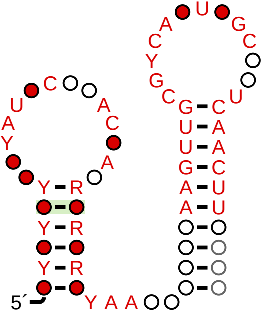

HMP2_1599_1

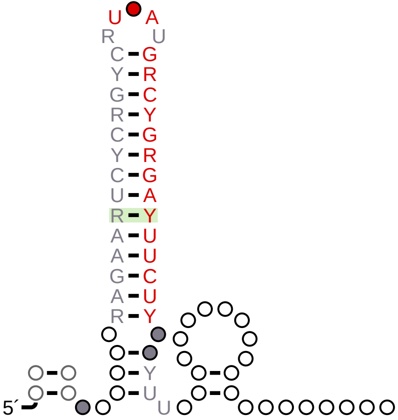

HMP2_15994_1

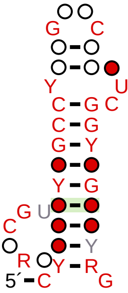

HMP2_16003_1

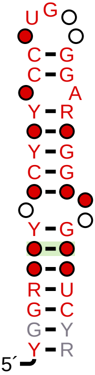

HMP2_16022_1

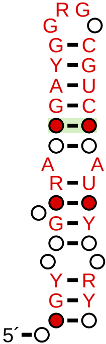

HMP2_16024_1

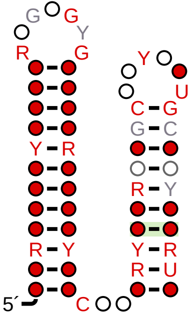

HMP2_16030_1

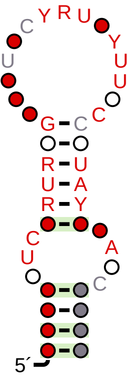

HMP2_16037_1

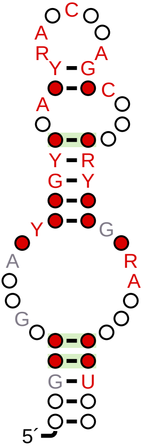

HMP2_16046_1

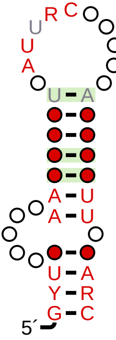

HMP2_16062_1

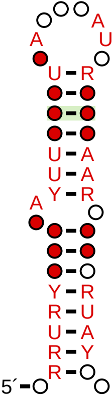

HMP2_16063_1

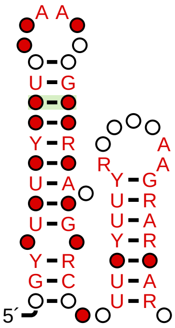

HMP2_16066_1

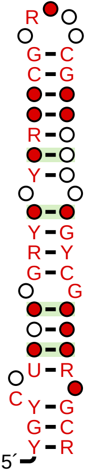

HMP2_16067_1

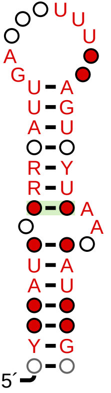

HMP2_16077_1

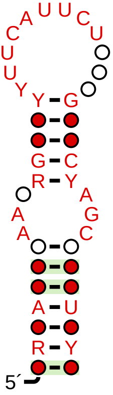

HMP2_16081_1

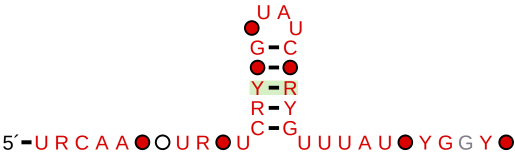

HMP2_16085_1

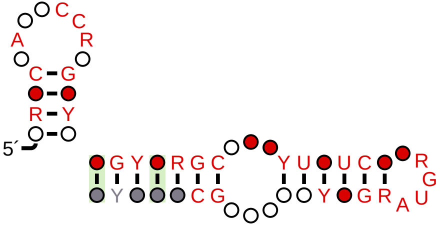

HMP2_16089_1

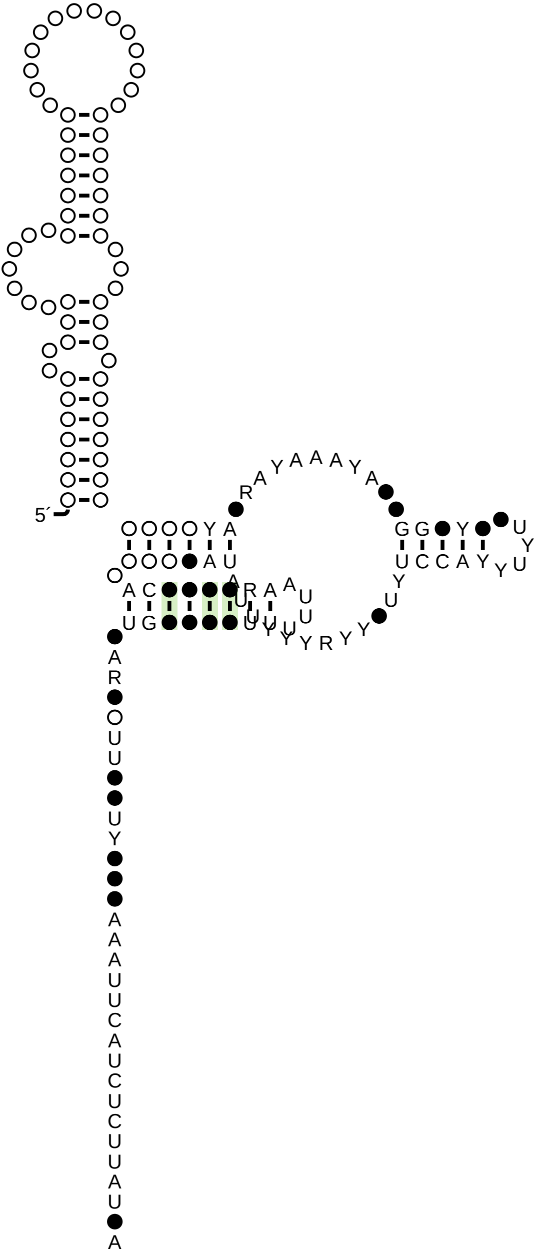

HMP2_16095_1

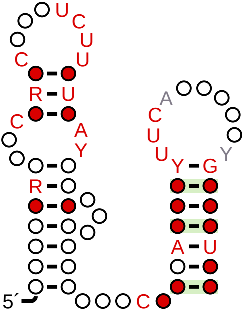

HMP2_1611_1

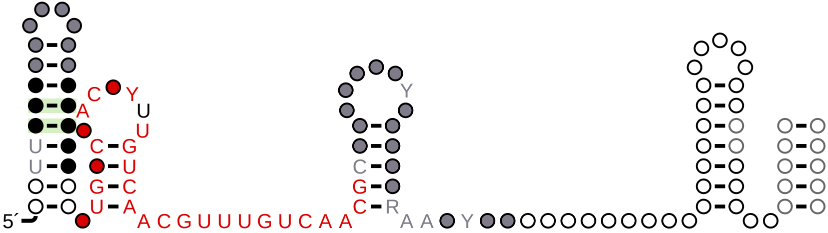

HMP2_16114_1

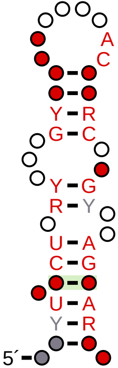

HMP2_16117_1

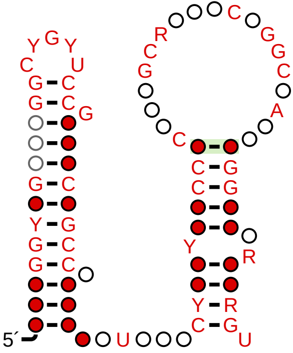

HMP2_1612_1

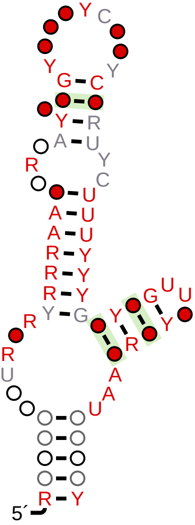

HMP2_16123_1

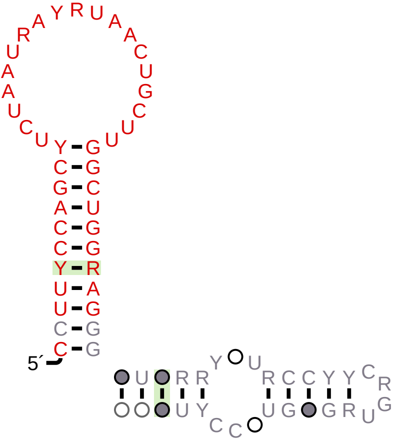

HMP2_16124_1

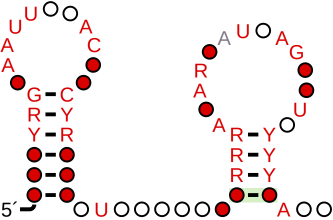

HMP2_16126_1

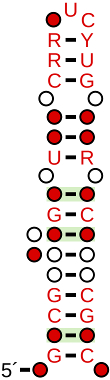

HMP2_16133_1

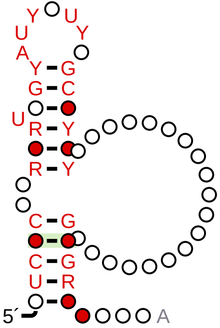

HMP2_16134_1

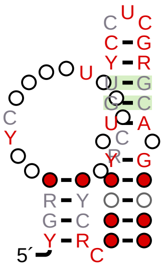

HMP2_1614_1

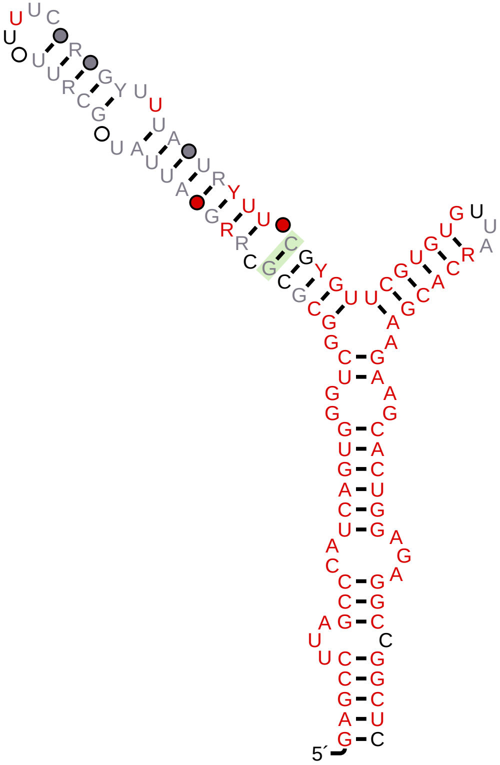

HMP2_16149_1

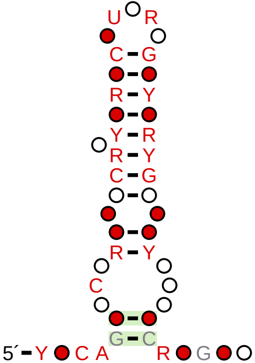

HMP2_16153_1

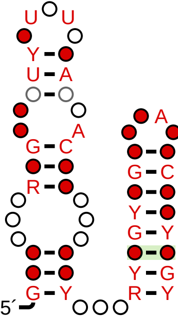

HMP2_16167_1

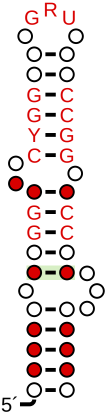

HMP2_16173_1

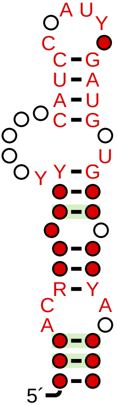

HMP2_16179_1

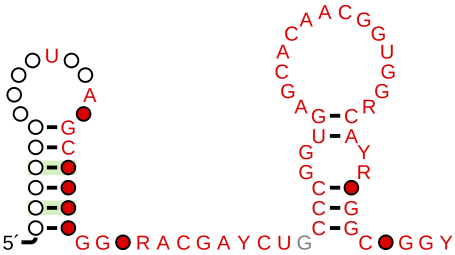

HMP2_16181_1

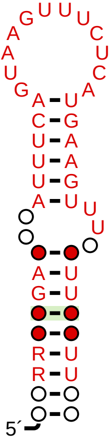

HMP2_16187_1

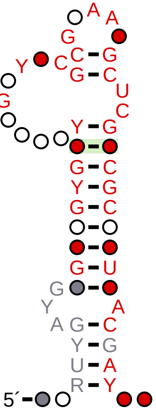

HMP2_16193_1

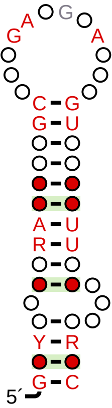

HMP2_16197_1

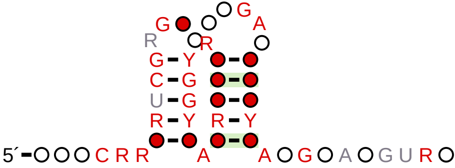

HMP2_16201_1

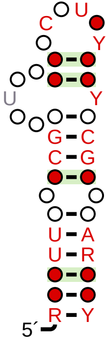

HMP2_16211_1

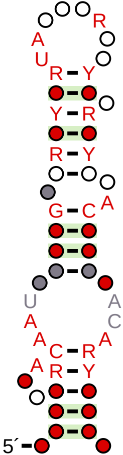

HMP2_1622_1

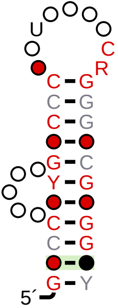

HMP2_16223_1

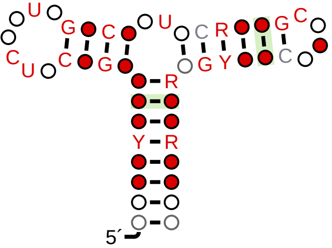

HMP2_16224_1

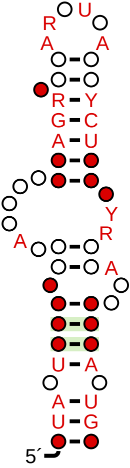

HMP2_16234_1

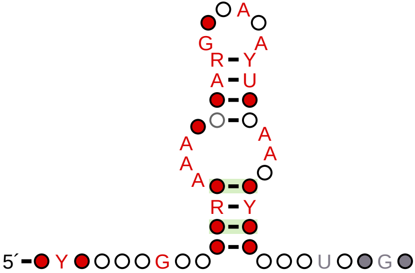

HMP2_16239_1

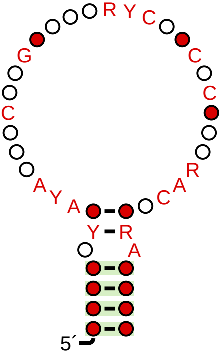

HMP2_16242_1

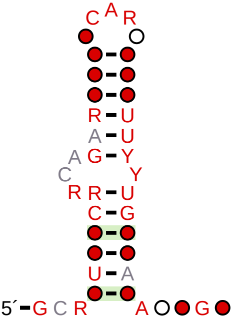

HMP2_16247_1

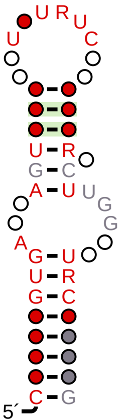

HMP2_16248_1

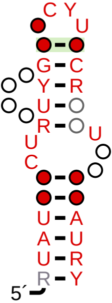

HMP2_16252_1

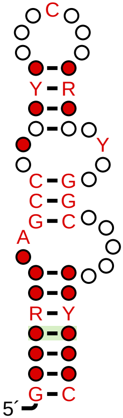

HMP2_16263_1

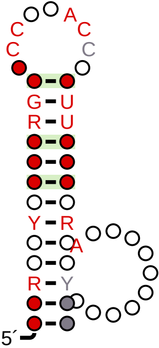

HMP2_16271_1

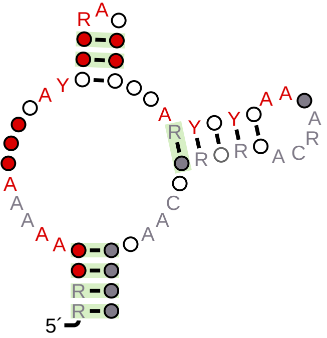

HMP2_16273_1

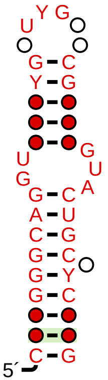

HMP2_16275_1

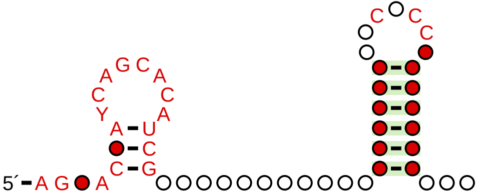

HMP2_16308_1

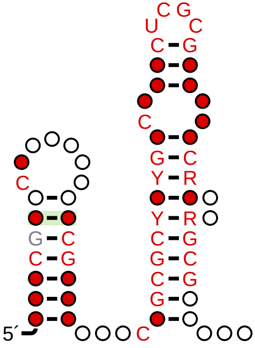

HMP2_16311_1

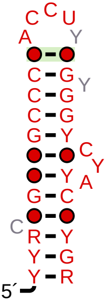

HMP2_16324_1

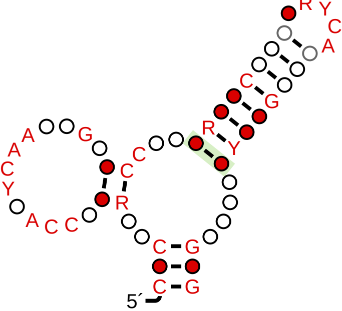

HMP2_16339_1

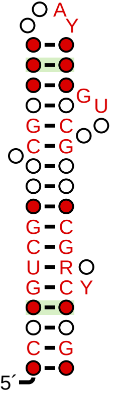

HMP2_16341_1

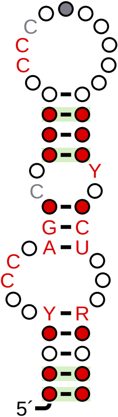

HMP2_16355_1

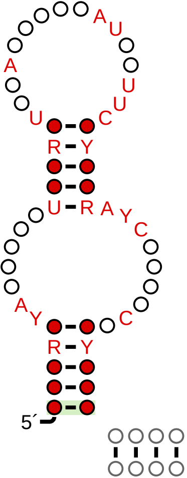

HMP2_16372_1

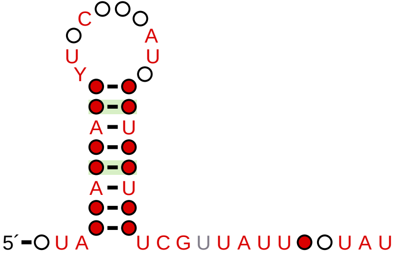

HMP2_16375_1

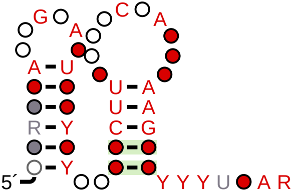

HMP2_16377_1

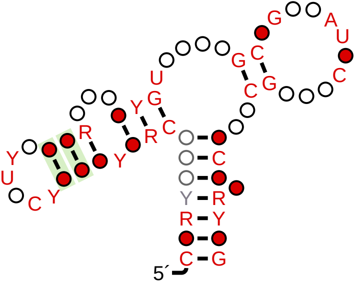

HMP2_16389_1

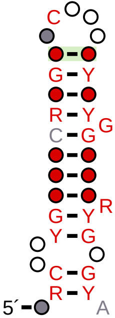

HMP2_16390_1

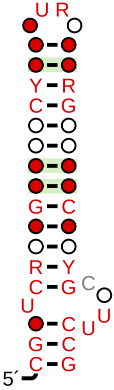

HMP2_16397_1

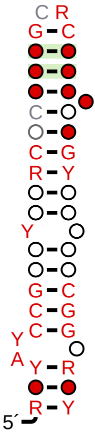

HMP2_164_1

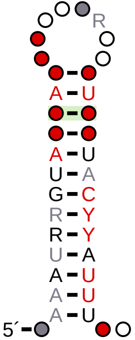

HMP2_16407_1

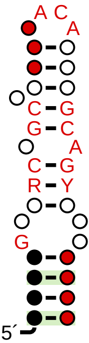

HMP2_16416_1

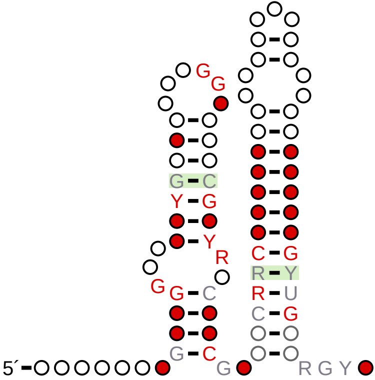

HMP2_16418_1

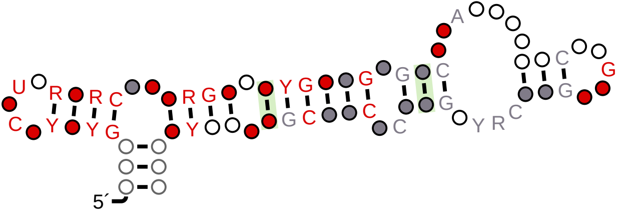

HMP2_16432_1

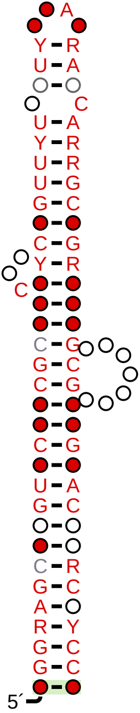

HMP2_16435_1

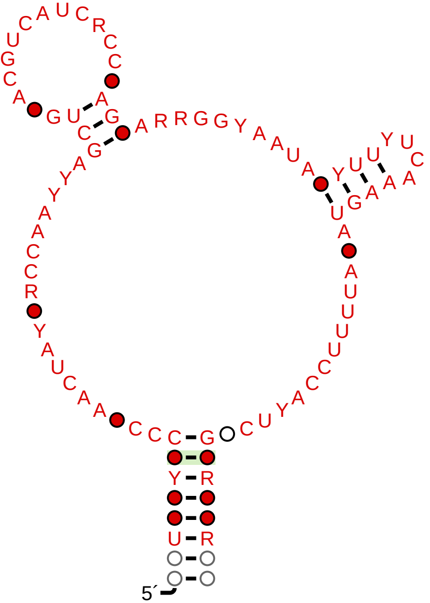

HMP2_16437_1

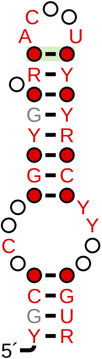

HMP2_16457_1

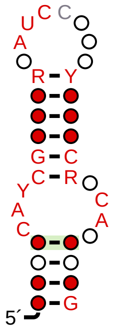

HMP2_1647_1

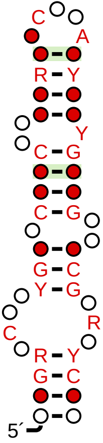

HMP2_16474_1

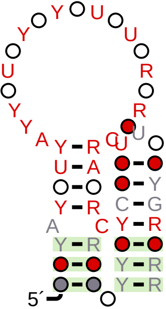

HMP2_16477_1

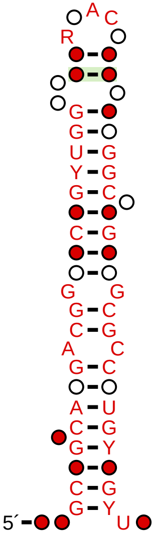

HMP2_16479_1

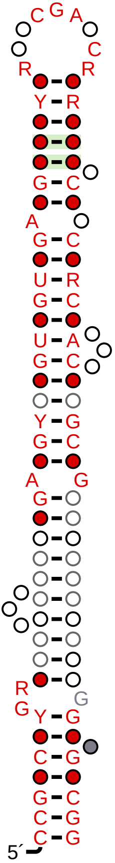

HMP2_16480_1

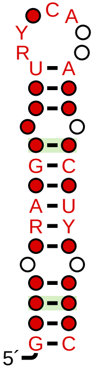

HMP2_16491_1

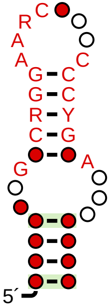

HMP2_16496_1

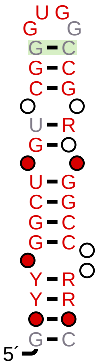

HMP2_16498_1

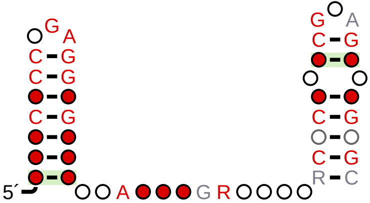

HMP2_16502_1

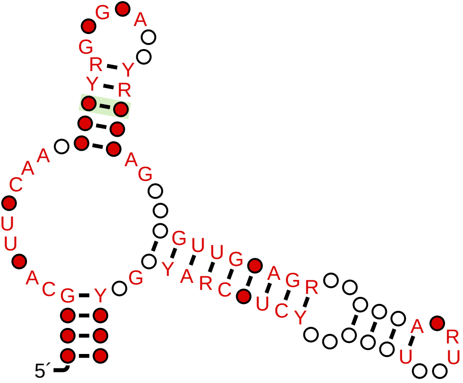

HMP2_16508_1

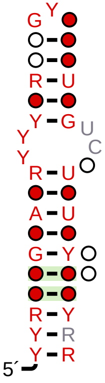

HMP2_1679_1

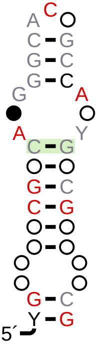

HMP2_1680_1

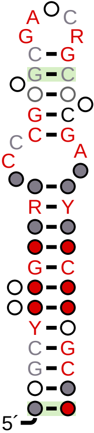

HMP2_1681_1

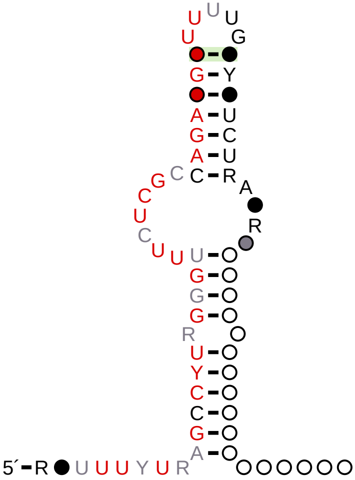

HMP2_1688_1

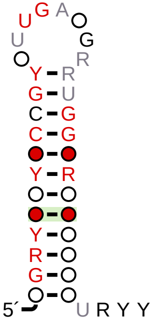

HMP2_169_1

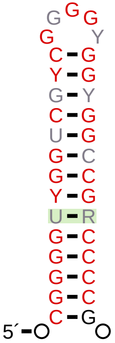

HMP2_1704_1

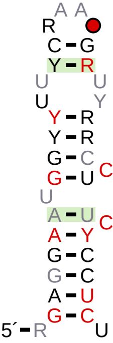

HMP2_1706_1

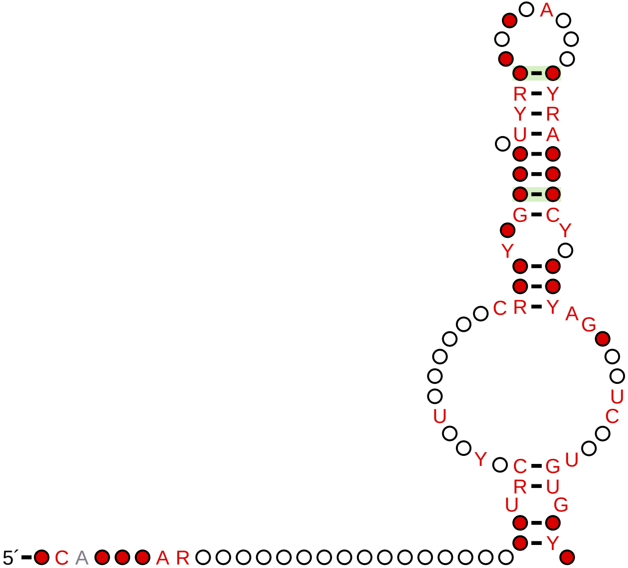

HMP2_1710_1

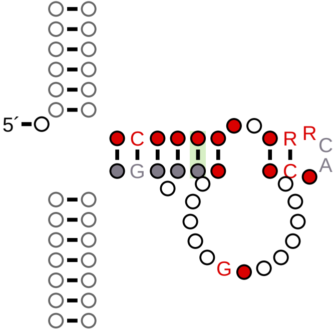

HMP2_1711_1

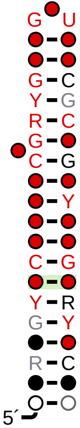

HMP2_173_1

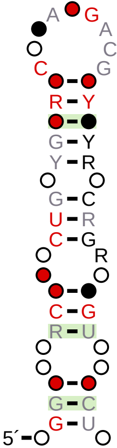

HMP2_1754_1

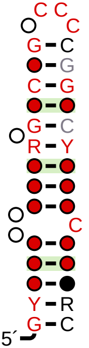

HMP2_1755_1

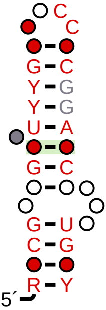

HMP2_1761_1

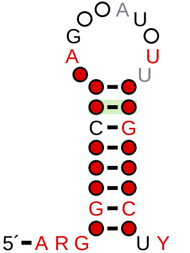

HMP2_1768_1

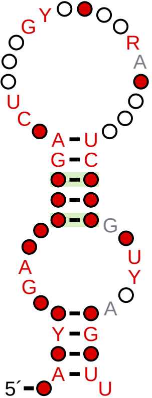

HMP2_177_1

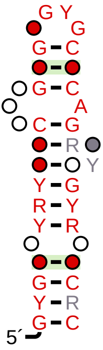

HMP2_1770_1

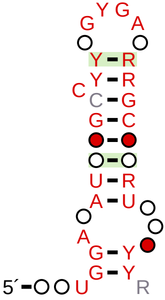

HMP2_1797_1

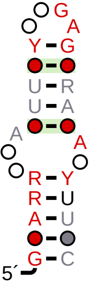

HMP2_1798_1

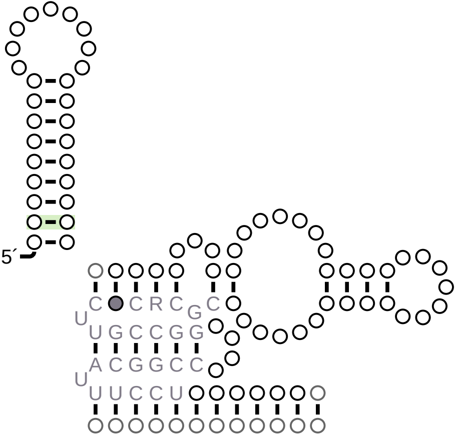

HMP2_1800_1

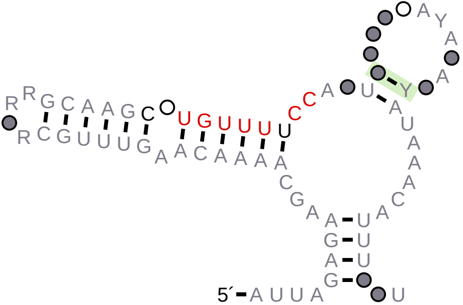

HMP2_1814_1

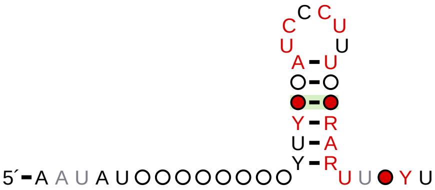

HMP2_1827_1

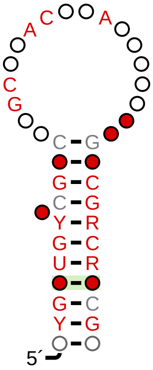

HMP2_184_1

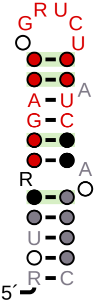

HMP2_185_1

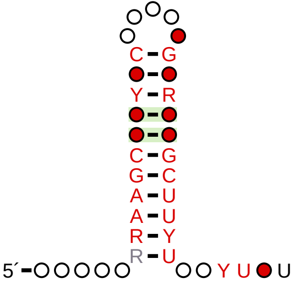

HMP2_1861_1

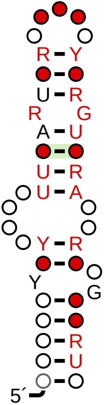

HMP2_1869_1

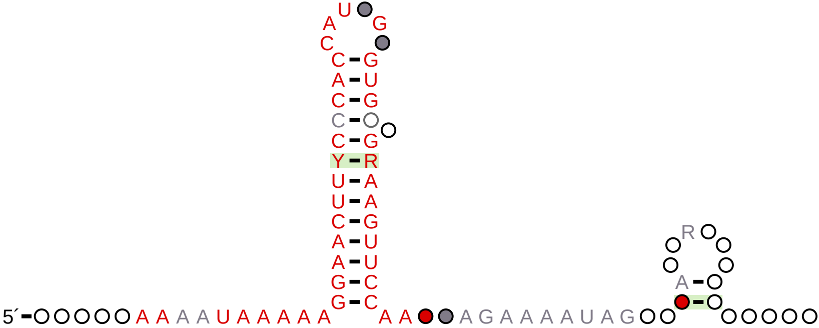

HMP2_1888_1

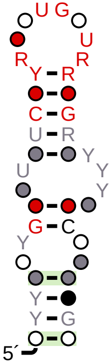

HMP2_1900_1

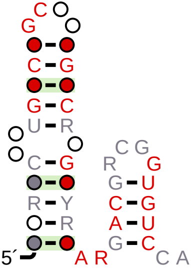

HMP2_193_1

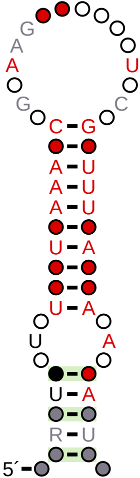

HMP2_1930_1

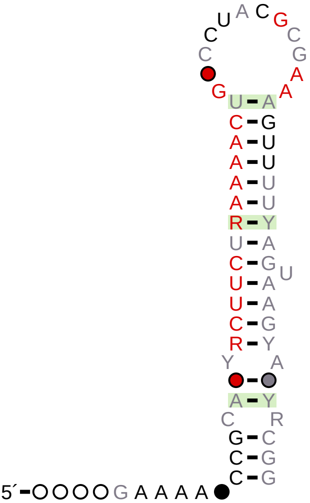

HMP2_1931_1

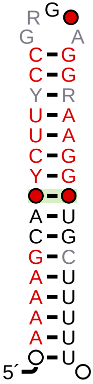

HMP2_1947_1

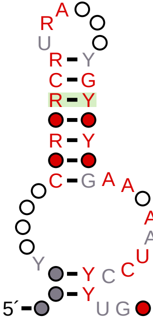

HMP2_1966_1

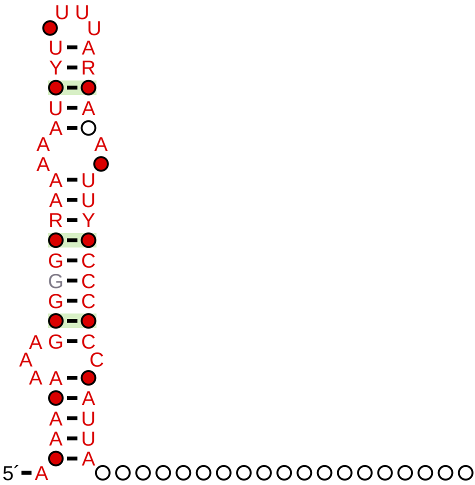

HMP2_1983_1

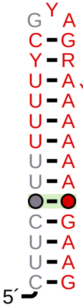

HMP2_1997_1

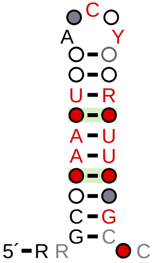

HMP2_20_1

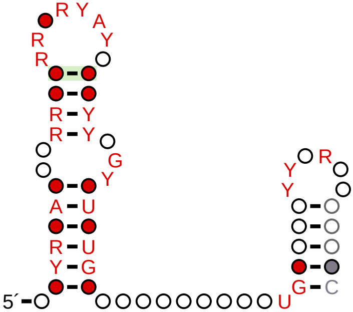

HMP2_2000_1

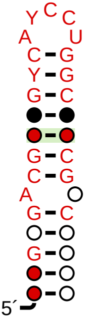

HMP2_2012_1

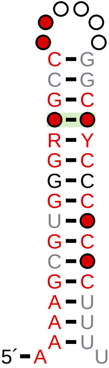

HMP2_204_1

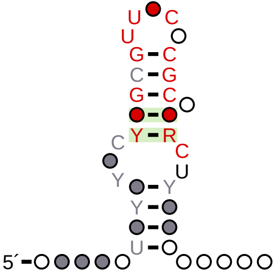

HMP2_2049_1

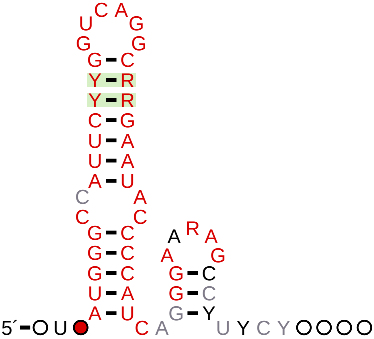

HMP2_2050_1

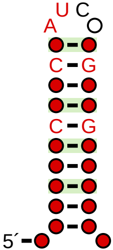

HMP2_2062_1

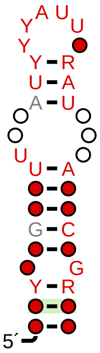

HMP2_2063_1

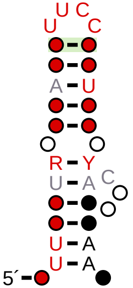

HMP2_2081_1

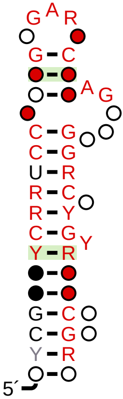

HMP2_2096_1

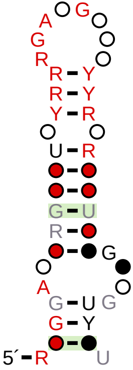

HMP2_2098_1

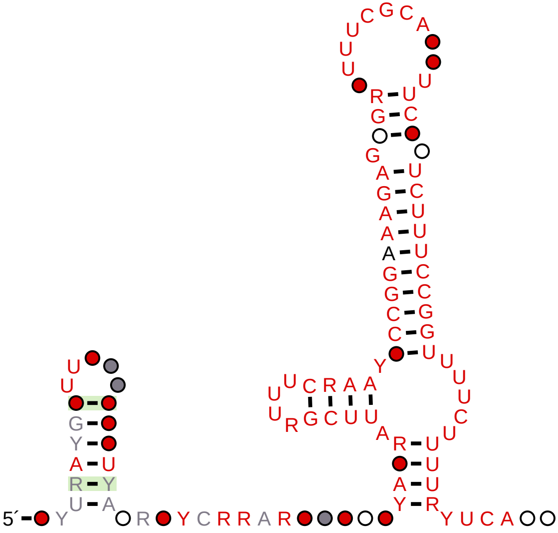

HMP2_211_1

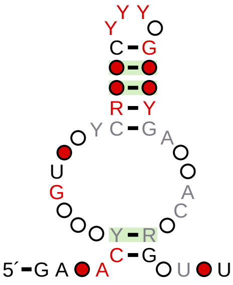

HMP2_2111_1

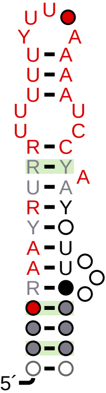

HMP2_2115_1

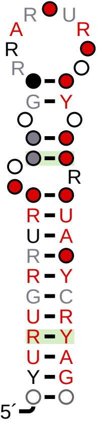

HMP2_212_1

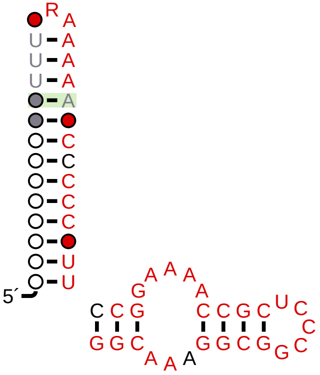

HMP2_2129_1

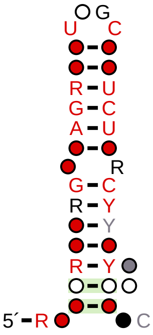

HMP2_2137_1

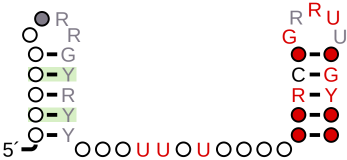

HMP2_2141_1

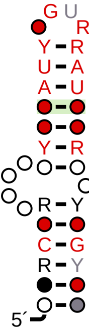

HMP2_2142_1

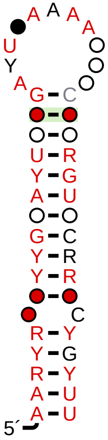

HMP2_2147_1

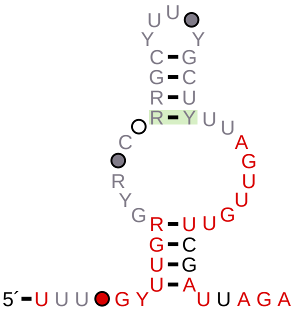

HMP2_2153_1

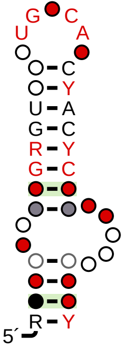

HMP2_2166_1

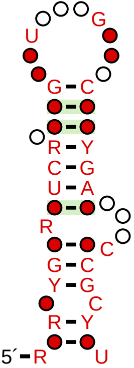

HMP2_2168_1

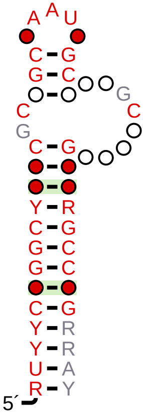

HMP2_2175_1

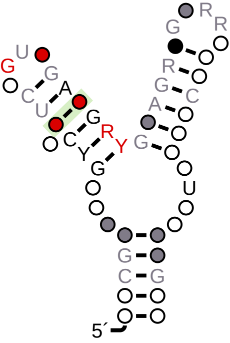

HMP2_2192_1

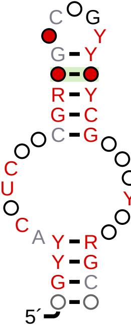

HMP2_2193_1

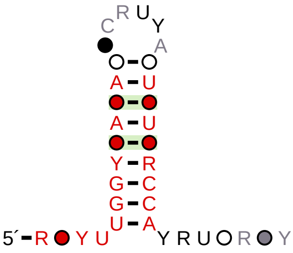

HMP2_2199_1

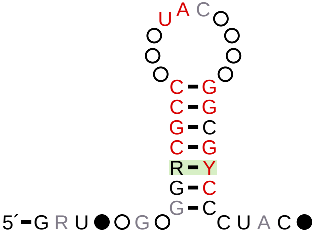

HMP2_22_1

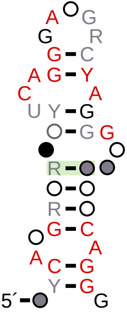

HMP2_2205_1

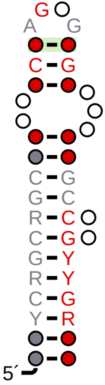

HMP2_2207_1

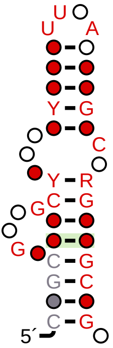

HMP2_2216_1

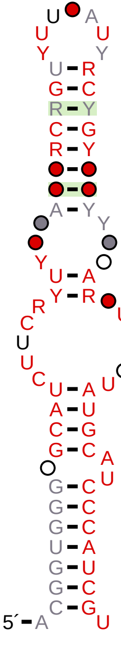

HMP2_2219_1

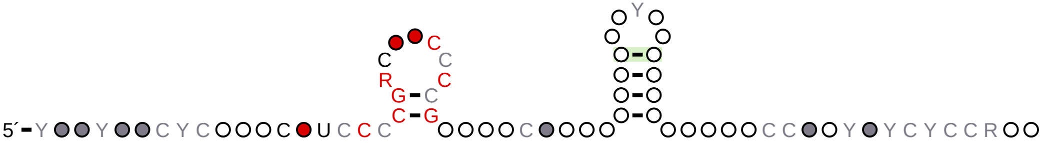

HMP2_2221_1

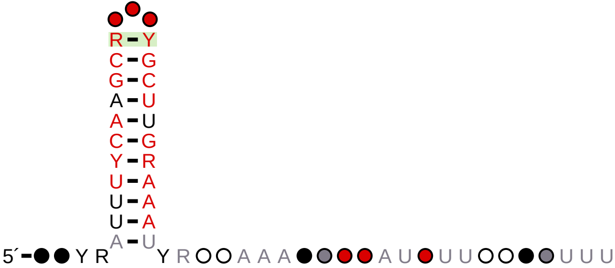

HMP2_2226_1

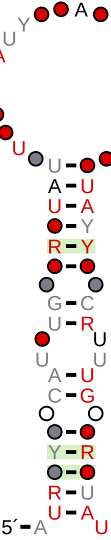

HMP2_2229_1

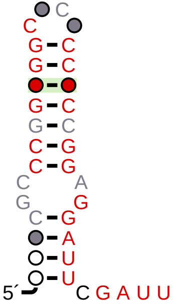

HMP2_2242_1

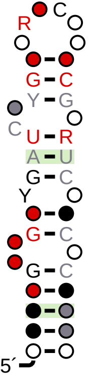

HMP2_2247_1

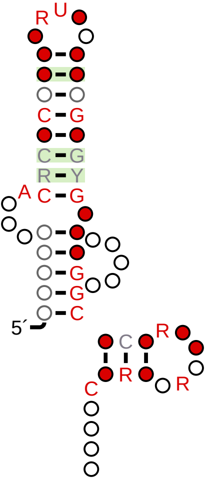

HMP2_2254_1

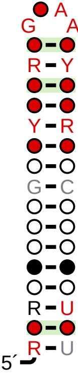

HMP2_2259_1

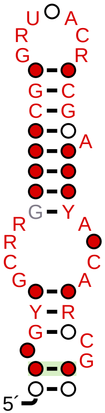

HMP2_226_1

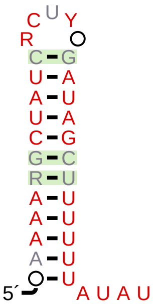

HMP2_2261_1

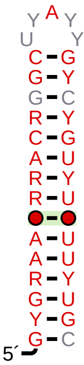

HMP2_2274_1

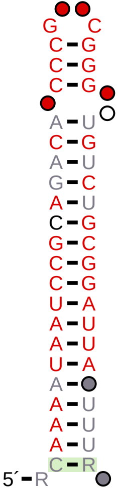

HMP2_2275_1

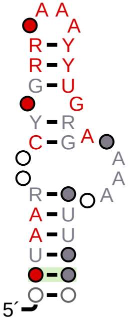

HMP2_2281_1

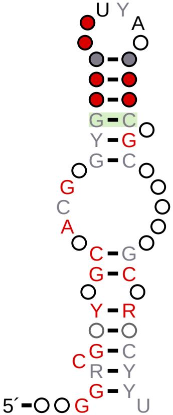

HMP2_2283_1

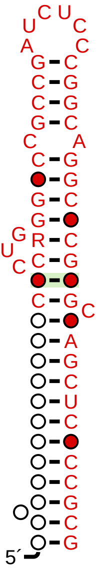

HMP2_2294_1

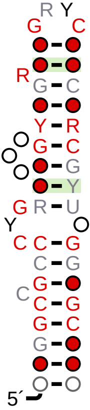

HMP2_2304_1

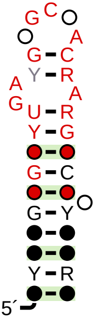

HMP2_2308_1

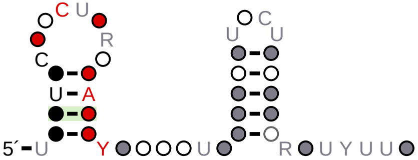

HMP2_2319_1

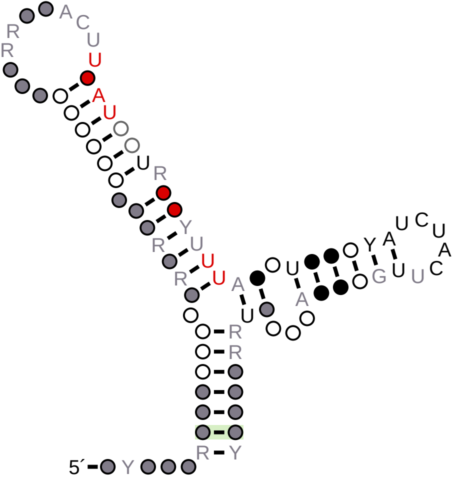

HMP2_2323_1

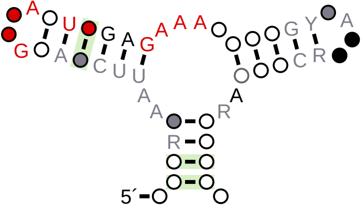

HMP2_2325_1

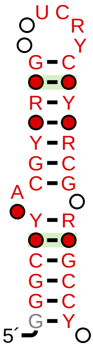

HMP2_2327_1

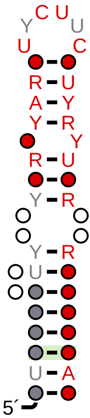

HMP2_234_1

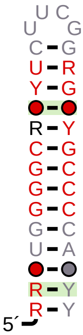

HMP2_2344_1

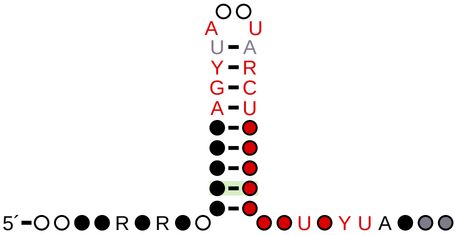

HMP2_2349_1

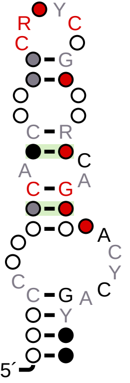

HMP2_2366_1

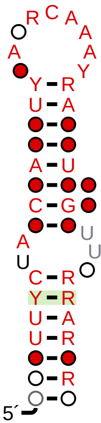

HMP2_2389_1

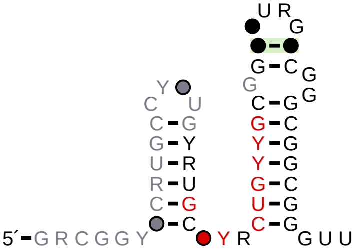

HMP2_2390_1

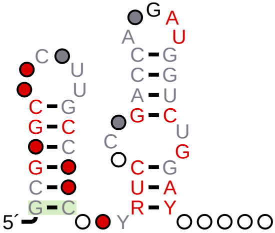

HMP2_2392_1

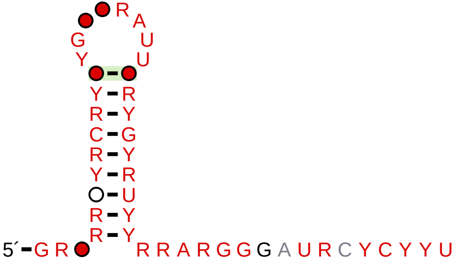

HMP2_2393_1

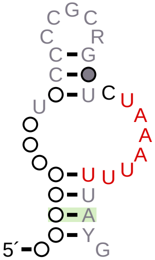

HMP2_2401_1

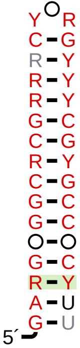

HMP2_241_1

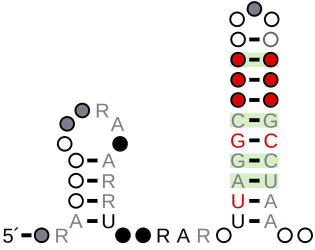

HMP2_2424_1

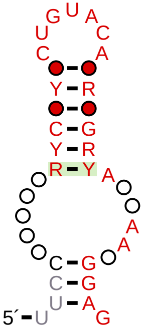

HMP2_2431_1

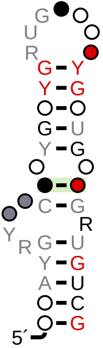

HMP2_2432_1

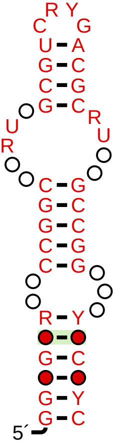

HMP2_2440_1

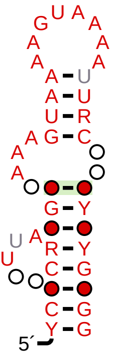

HMP2_2445_1

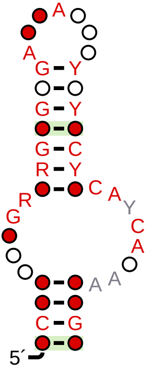

HMP2_2447_1

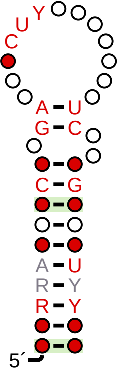

HMP2_2463_1

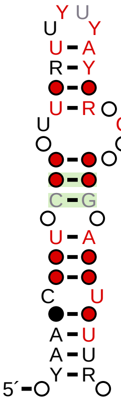

HMP2_249_1

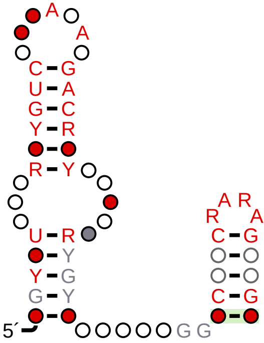

HMP2_2493_1

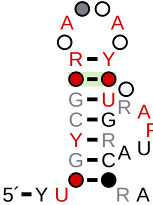

HMP2_2502_1

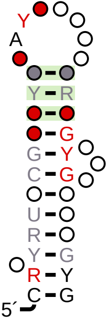

HMP2_251_1

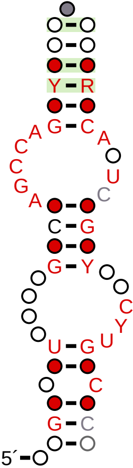

HMP2_2514_1

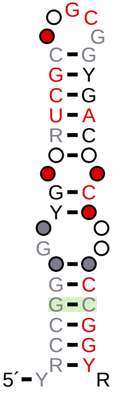

HMP2_2519_1

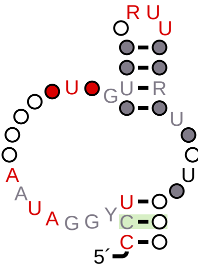

HMP2_2520_1

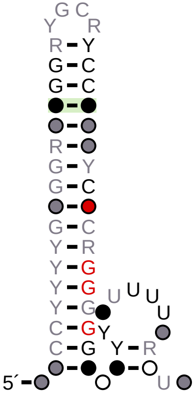

HMP2_2527_1

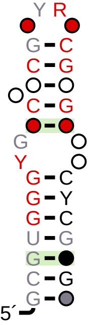

HMP2_2533_1

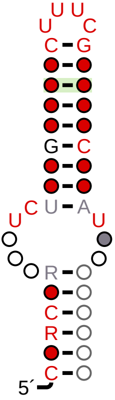

HMP2_2543_1

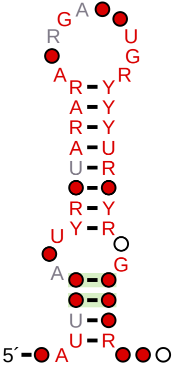

HMP2_2544_1

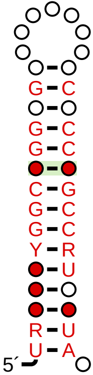

HMP2_2553_1

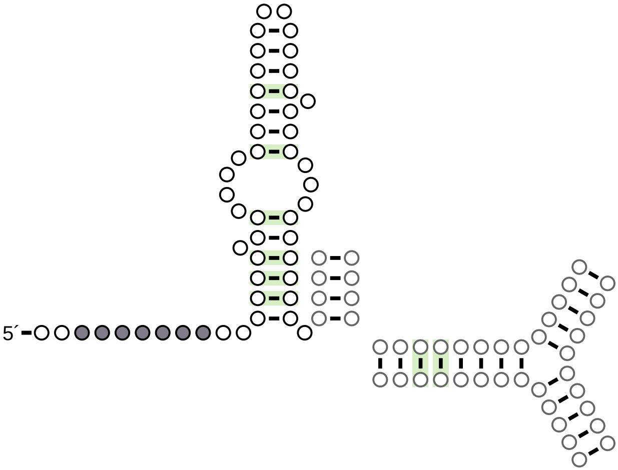

HMP2_2560_1

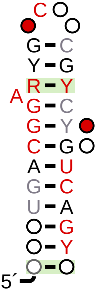

HMP2_2563_1

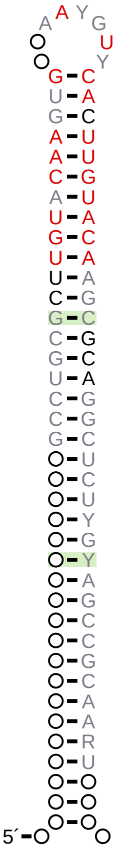

HMP2_2567_1

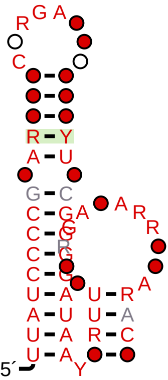

HMP2_2581_1

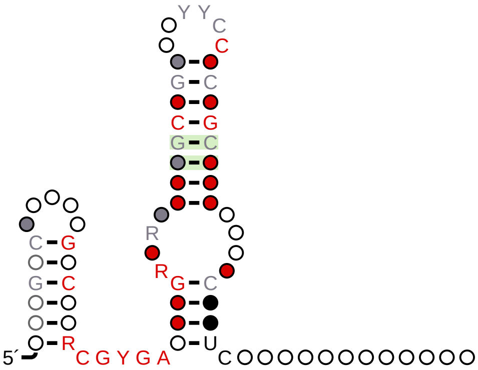

HMP2_260_1

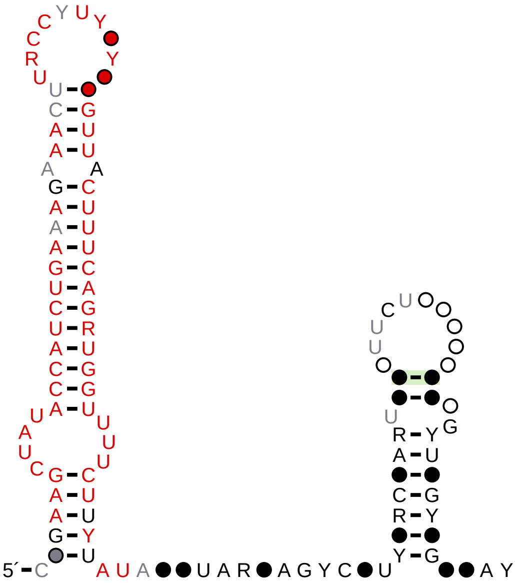

HMP2_2612_1

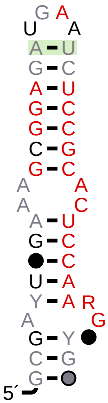

HMP2_2619_1

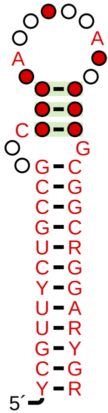

HMP2_2627_1

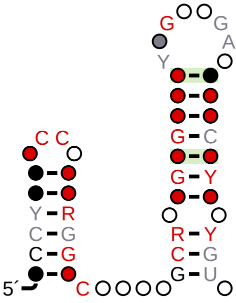

HMP2_2635_1

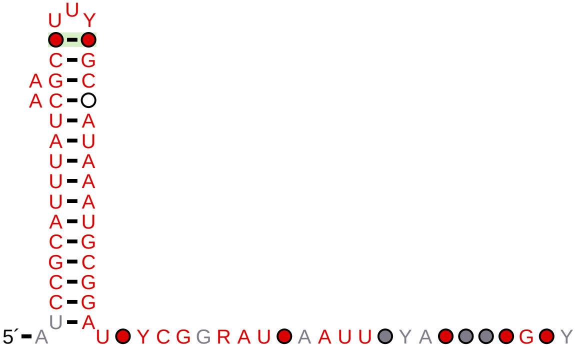

U U Y
C G
A G C
A C
U A
A U
U A
U A
U A
A U
C G
G C
C G
C G
U A
5´ A
U Y C G G R A U A A U U Y A G Y

HMP2_2636_1

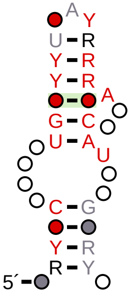

HMP2_2638_1

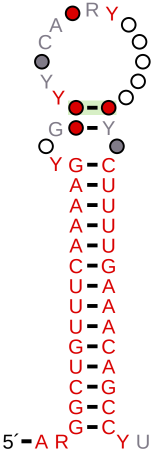

HMP2_2639_1

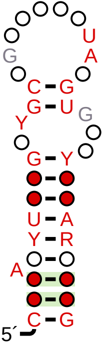

HMP2_2643_1

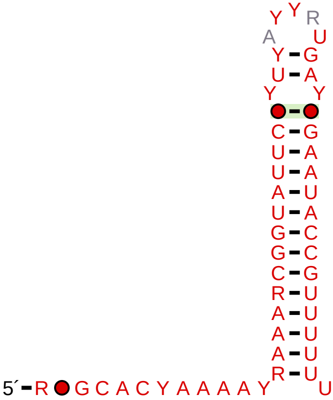

HMP2_266_1

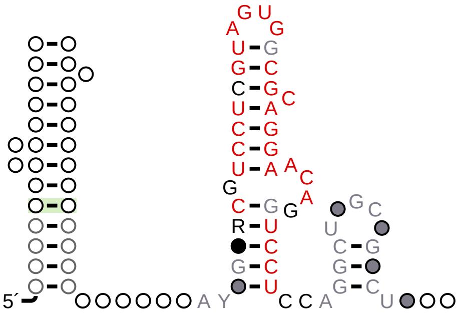

HMP2_2663_1

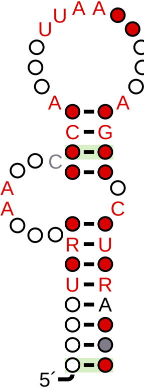

HMP2_2666_1

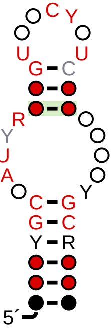

HMP2_2667_1

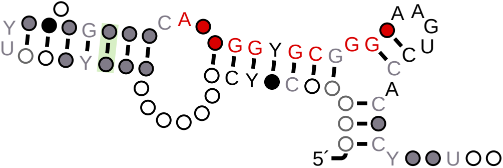

HMP2_2668_1

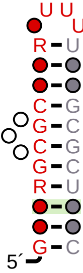

HMP2_2678_1

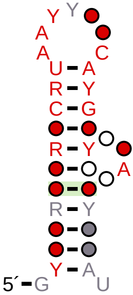

HMP2_2679_1

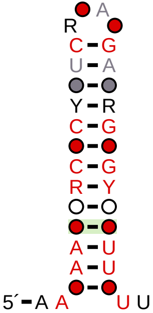

HMP2_2682_1

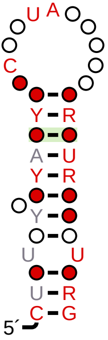

HMP2_2683_1

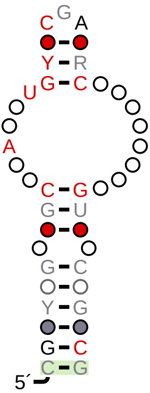

HMP2_2686_1

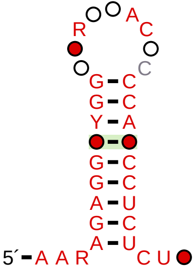

HMP2_2692_1

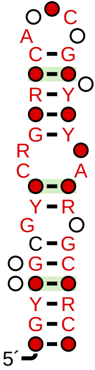

HMP2_2697_1

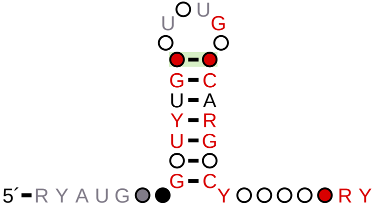

HMP2_2715_1

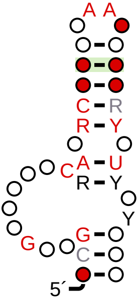

HMP2_2716_1

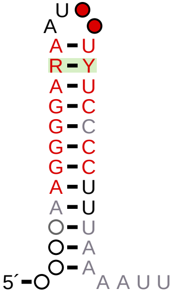

HMP2_2722_1

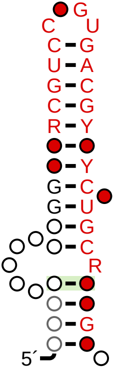

HMP2_2734_1

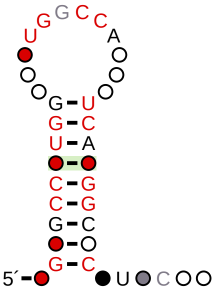

HMP2_2743_1

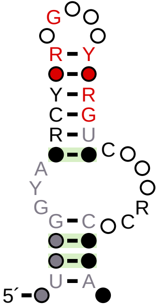

HMP2_2752_1

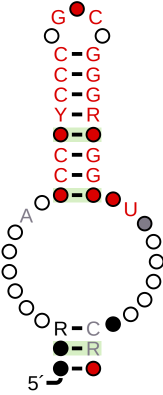

HMP2_2765_1

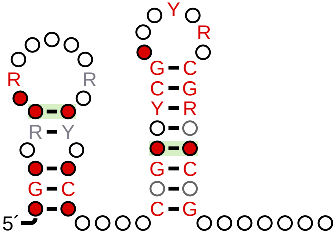

HMP2_2779_1

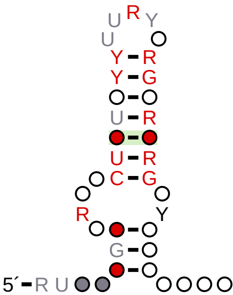

HMP2_2786_1

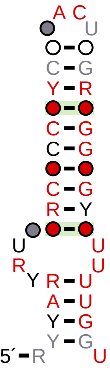

HMP2_2787_1

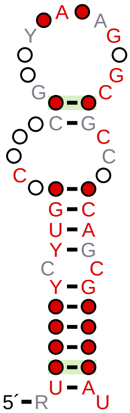

HMP2_2788_1

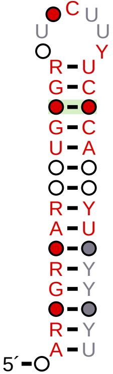

HMP2_2791_1

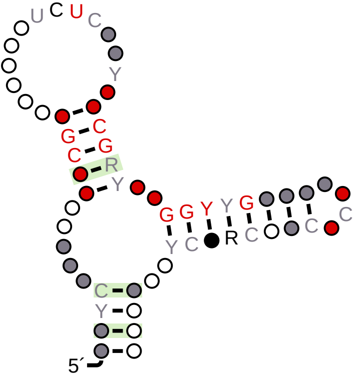

HMP2_280_1

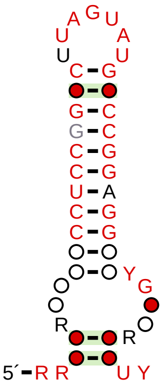

HMP2_2829_1

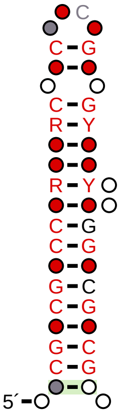

HMP2_2832_1

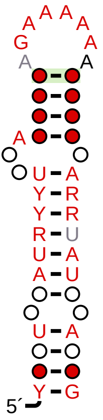

HMP2_2840_1

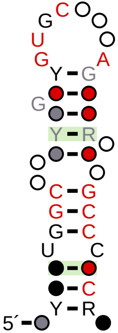

HMP2_2843_1

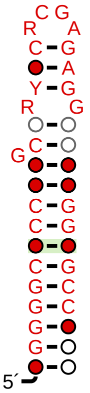

HMP2_2849_1

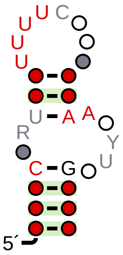

HMP2_2860_1

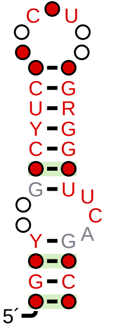

HMP2_2861_1

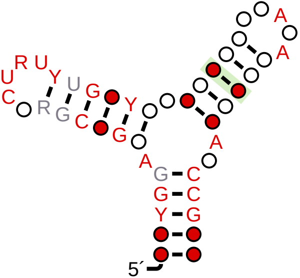

HMP2_2869_1

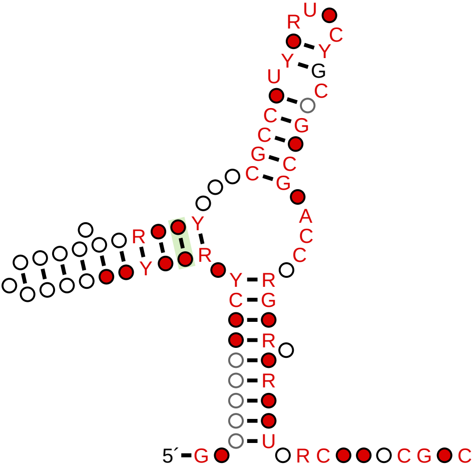

HMP2_2872_1

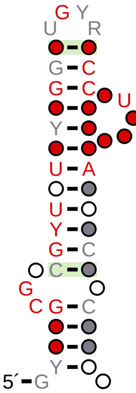

HMP2_2893_1

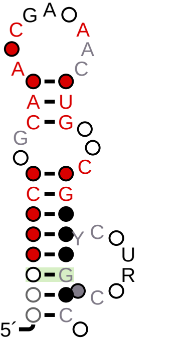

HMP2_2898_1

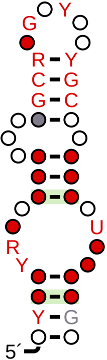

HMP2_2909_1

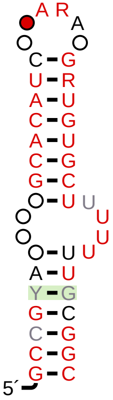

HMP2_2917_1

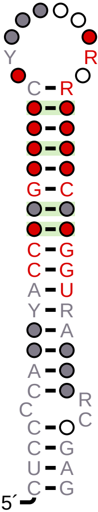

HMP2_2924_1

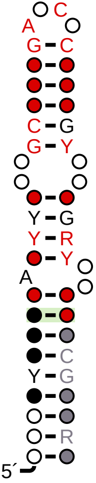

HMP2_2926_1

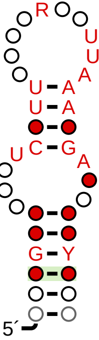

HMP2_2929_1

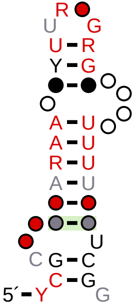

HMP2_2938_1

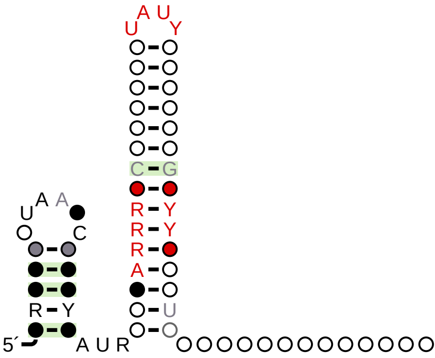

HMP2_2947_1

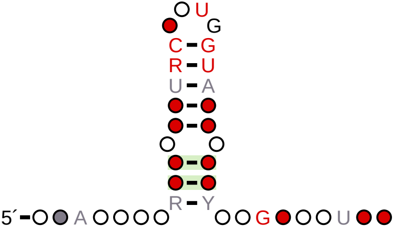

HMP2_2948_1

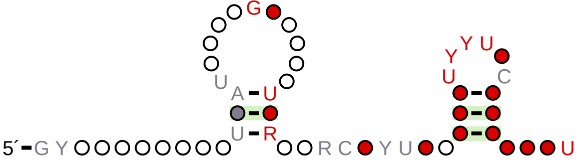

HMP2_2952_1

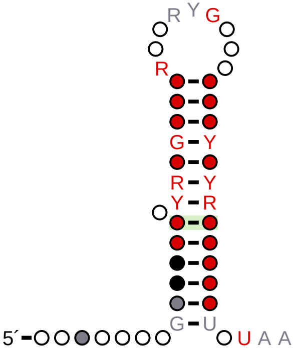

HMP2_2956_1

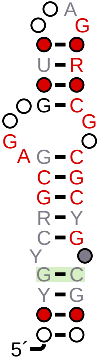

HMP2_2961_1

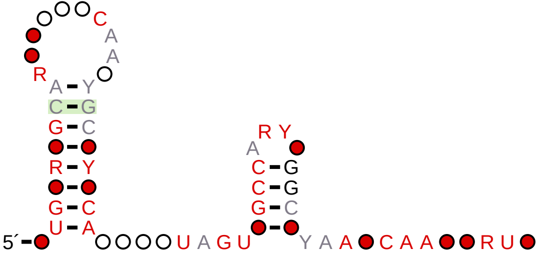

HMP2_2962_1

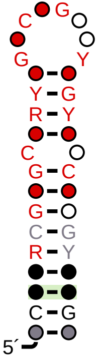

HMP2_2964_1

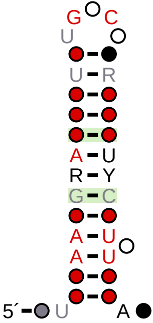

HMP2_2986_1

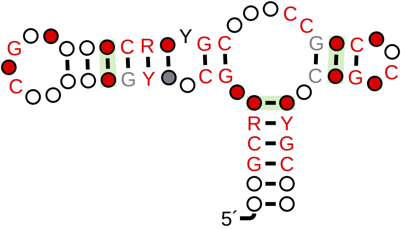

HMP2_2993_1

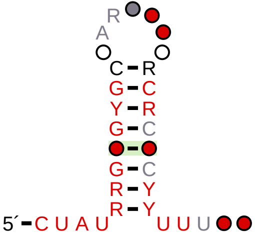

HMP2_30_1

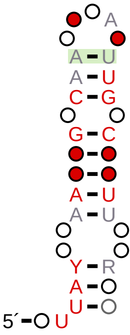

HMP2_3017_1

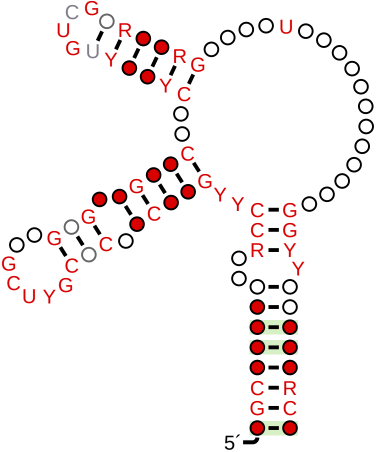

HMP2_302_1

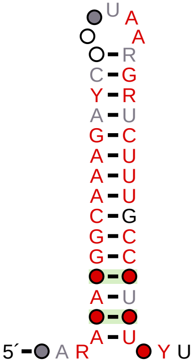

HMP2_3028_1

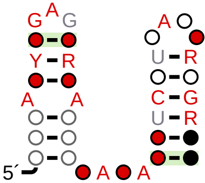

HMP2_3030_1

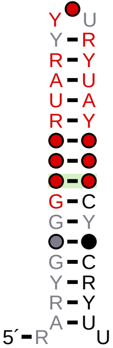

HMP2_3032_1

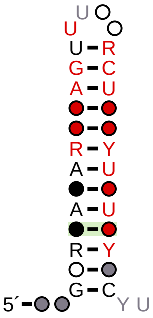

HMP2_3033_1

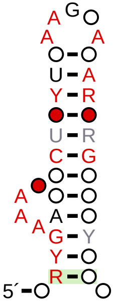

HMP2_3034_1

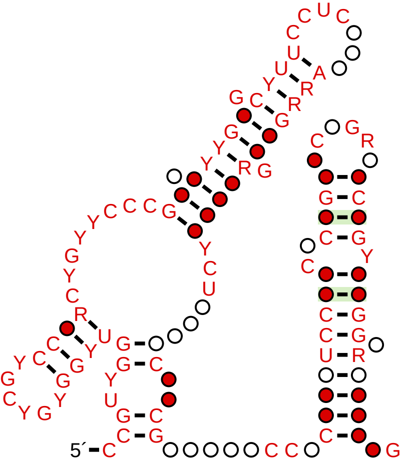

HMP2_3040_1

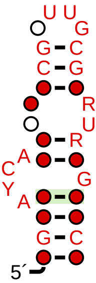

HMP2_3048_1

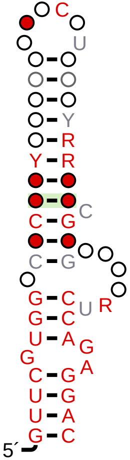

HMP2_3078_1

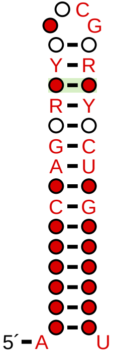

HMP2_3086_1

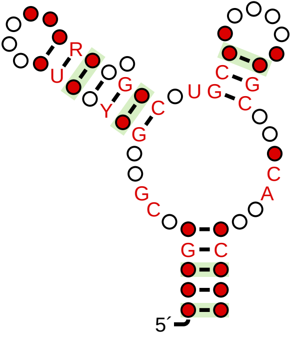

HMP2_3089_1

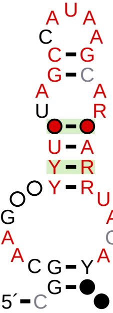

HMP2_309_1

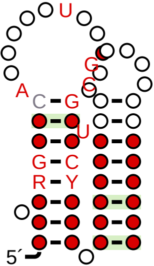

HMP2_3091_1

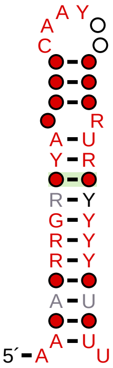

HMP2_3094_1

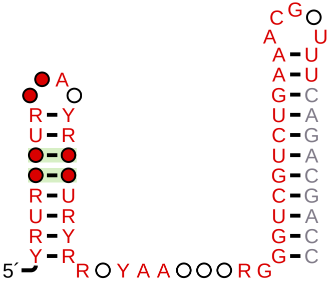

HMP2_3102_1

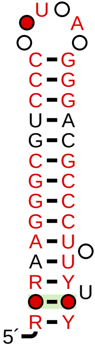

HMP2_3109_1

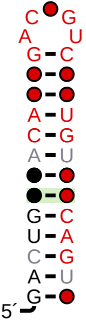

HMP2_3123_1

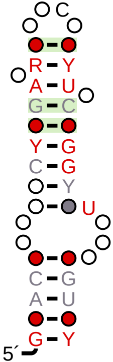

HMP2_3126_1

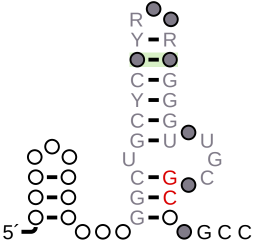

HMP2_3135_1

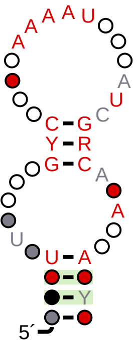

HMP2_3138_1

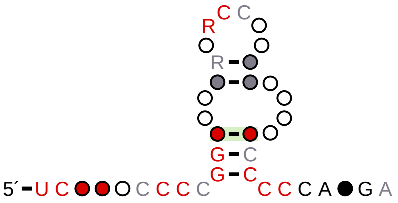

HMP2_3142_1

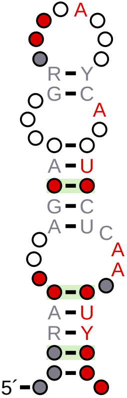

HMP2_3148_1

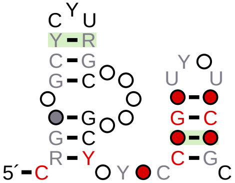

HMP2_3149_1

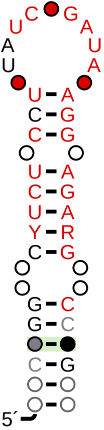

HMP2_3158_1

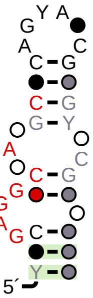

HMP2_3164_1

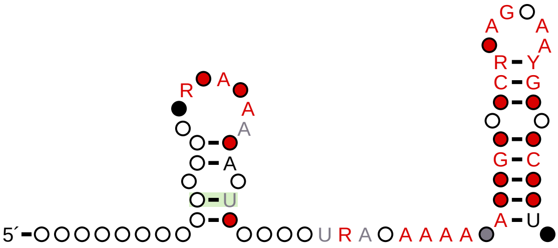

HMP2_3176_1

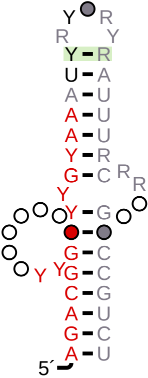

HMP2_3182_1

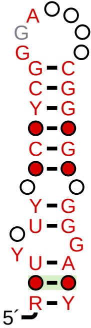

HMP2_3185_1

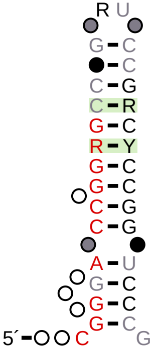

HMP2_3199_1

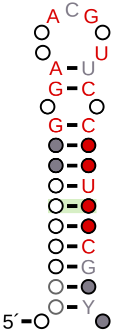

HMP2_3211_1

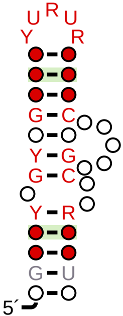

HMP2_3225_1

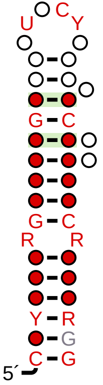

HMP2_3230_1

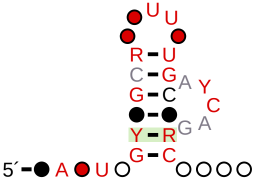

HMP2_3234_1

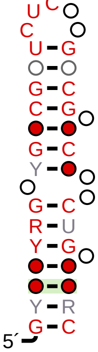

HMP2_3238_1

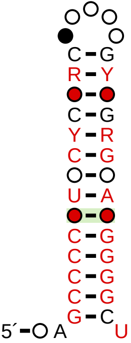

HMP2_3251_1

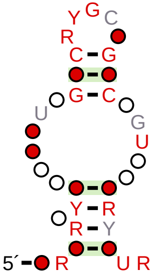

HMP2_3264_1

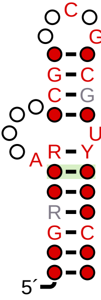

HMP2_3268_1

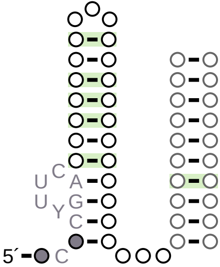

HMP2_3287_1

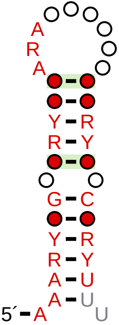

HMP2_3297_1

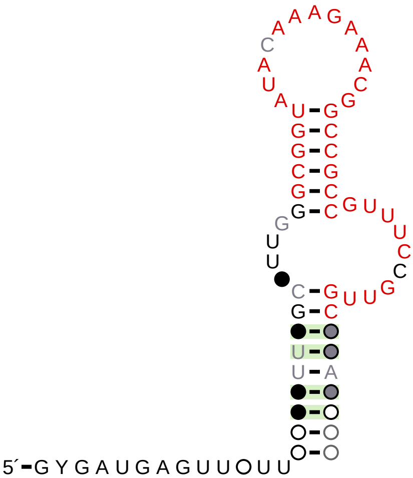

HMP2_3315_1

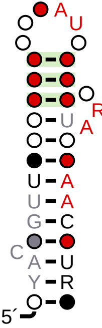

HMP2_3316_1

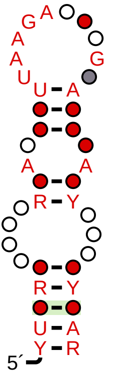

HMP2_3323_1

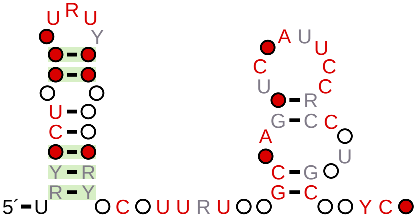

HMP2_3339_1

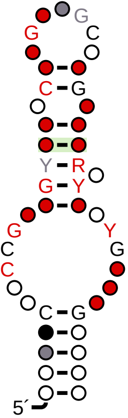

HMP2_3341_1

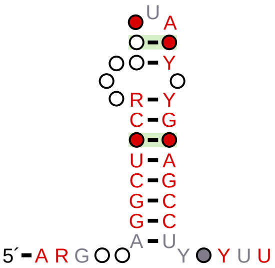

HMP2_3343_1

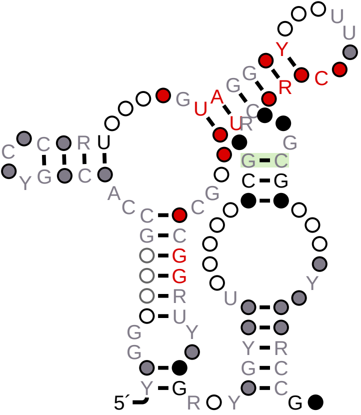

HMP2_3348_1

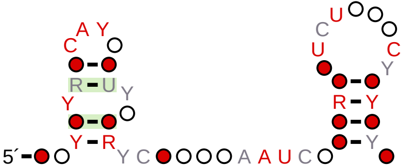

HMP2_3350_1

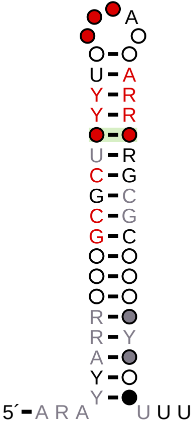

HMP2_3356_1

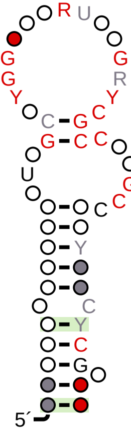

HMP2_336_1

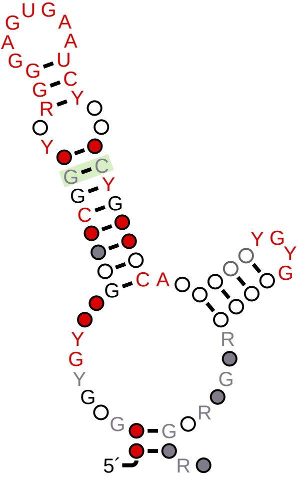

HMP2_3360_1

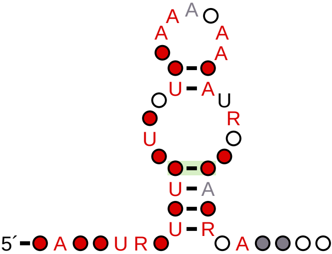

HMP2_3367_1

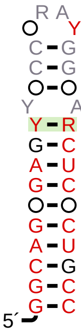

HMP2_3369_1

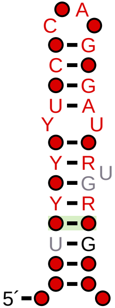

HMP2_3376_1

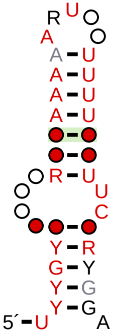

HMP2_3378_1

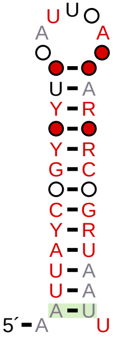

HMP2_3391_1

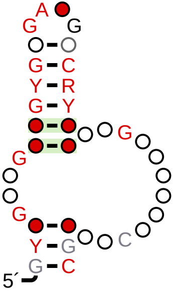

HMP2_3398_1

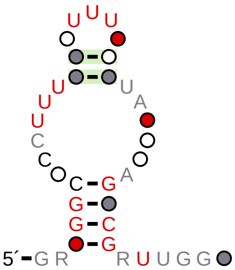

HMP2_3405_1

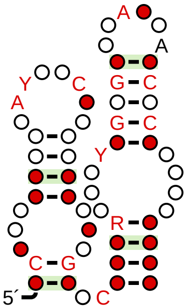

HMP2_3410_1

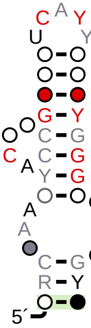

HMP2_3423_1

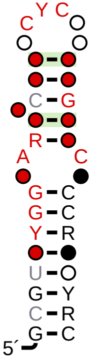

HMP2_3433_1

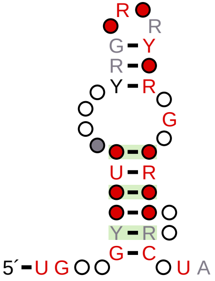

HMP2_346_1

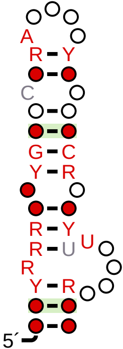

HMP2_3463_1

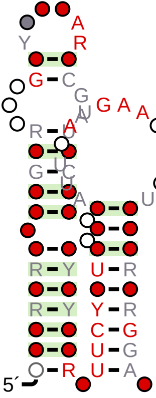

HMP2_3464_1

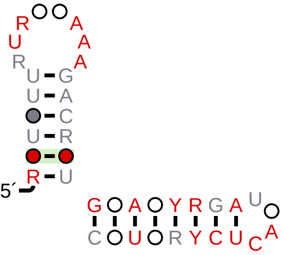

HMP2_3465_1

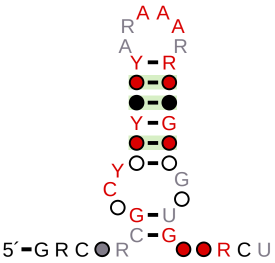

HMP2_3476_1

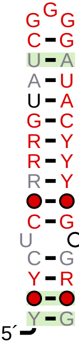

HMP2_3489_1

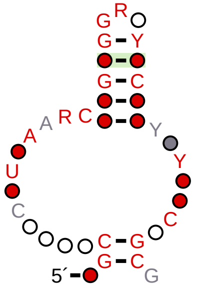

HMP2_3490_1

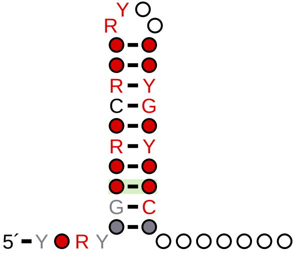

HMP2_3501_1

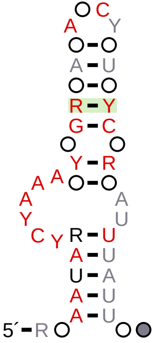

HMP2_3538_1

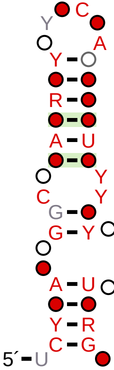

HMP2_3544_1

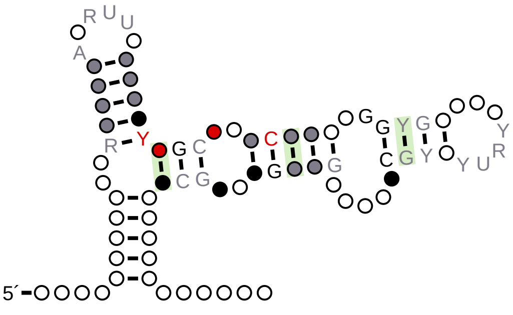

HMP2_3547_1

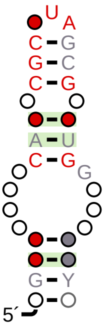

HMP2_3585_1

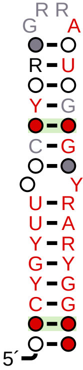

HMP2_3593_1

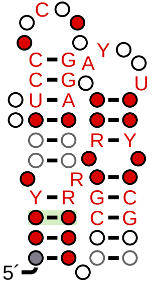

HMP2_3596_1

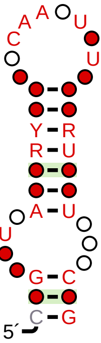

HMP2_3602_1

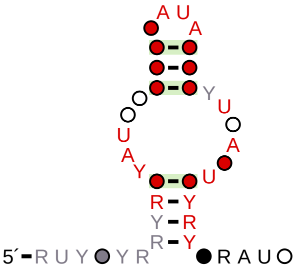

HMP2_3604_1

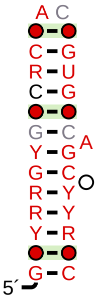

HMP2_3606_1

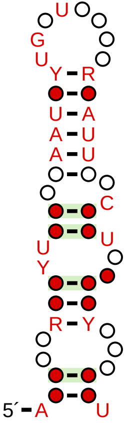

HMP2_3613_1

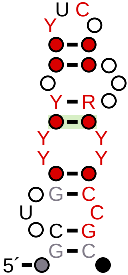

HMP2_3631_1

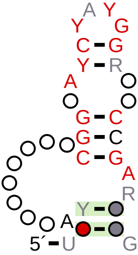

HMP2_3650_1

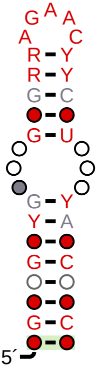

HMP2_3656_1

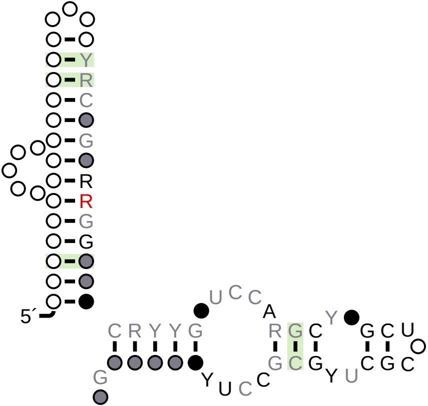

HMP2_3661_1

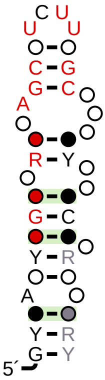

HMP2_3670_1

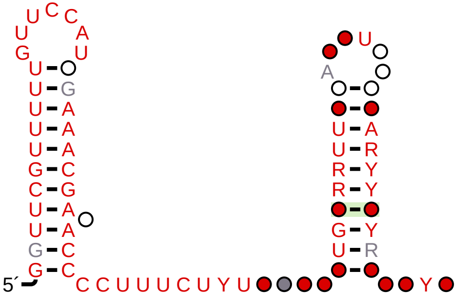

U C C
U A
G U
U
U G
U A
U A
U A
G C
C G
U A
U A
G C
G C
5´
C C U U U C U Y U
U
A
U A
U R
R Y
R Y
G Y
U R
Y

HMP2_3671_1

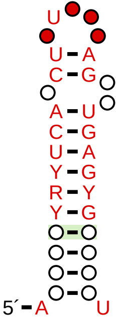

HMP2_3676_1

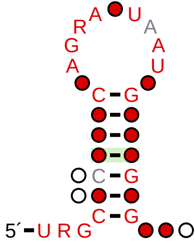

HMP2_3677_1

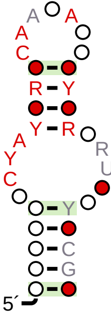

HMP2_3683_1

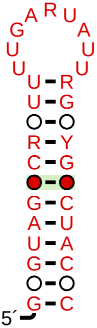

HMP2_3685_1

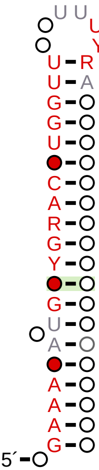

HMP2_3696_1

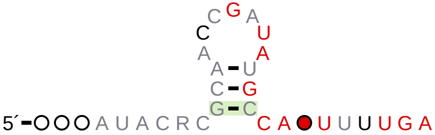

HMP2_3702_1

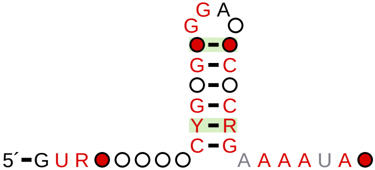

HMP2_3739_1

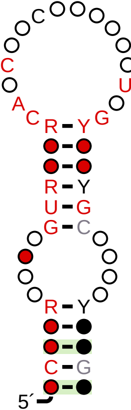

HMP2_374_1

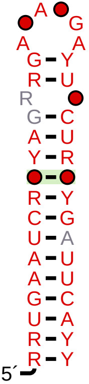

HMP2_3741_1

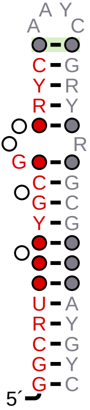

HMP2_3744_1

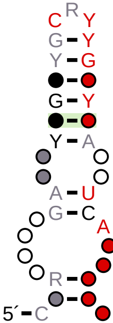

HMP2_3758_1

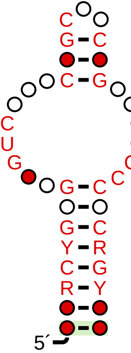

HMP2_3759_1

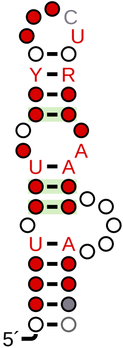

HMP2_3771_1

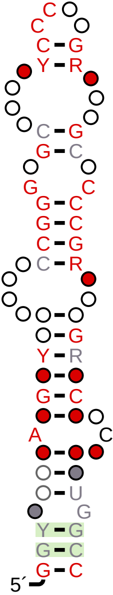

HMP2_3790_1

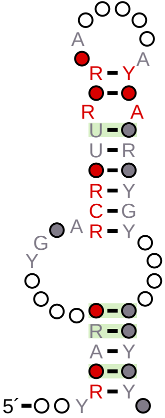

HMP2_3797_1

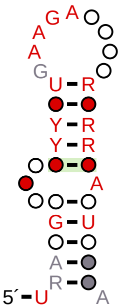

HMP2_3799_1

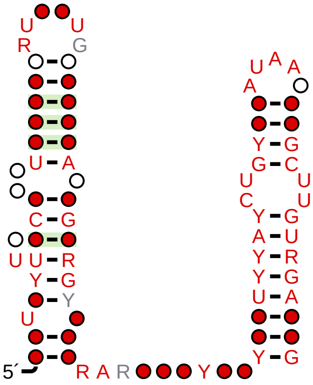

HMP2_3802_1

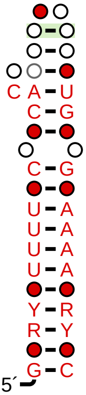

HMP2_3803_1

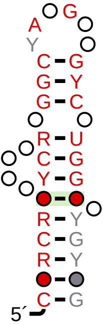

HMP2_3813_1

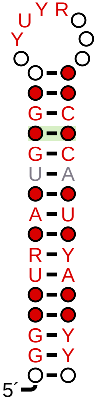

HMP2_3817_1

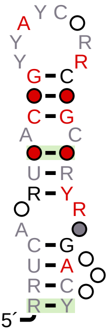

HMP2_3818_1

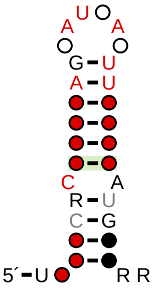

HMP2_3824_1

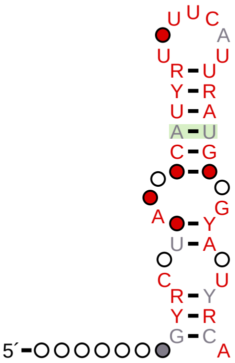

HMP2_3827_1

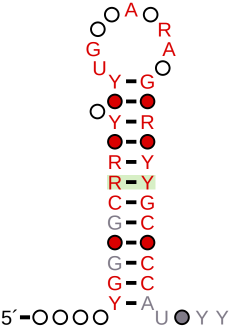

HMP2_3828_1

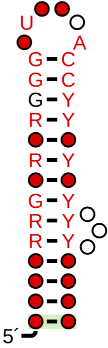

HMP2_3833_1

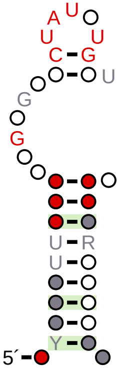

HMP2_3835_1

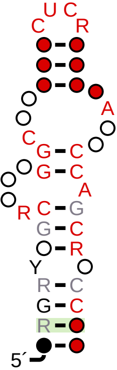

HMP2_3855_1

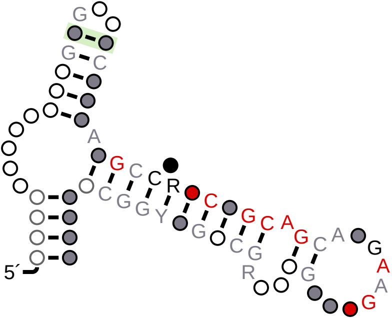

HMP2_3860_1

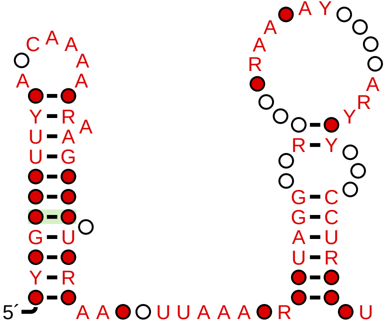

HMP2_3861_1

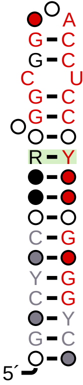

HMP2_3864_1

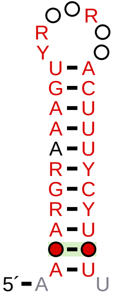

HMP2_3873_1

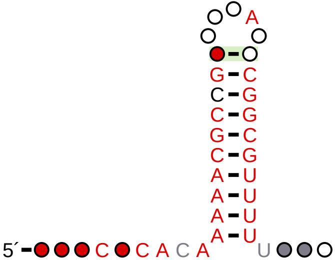

HMP2_3875_1

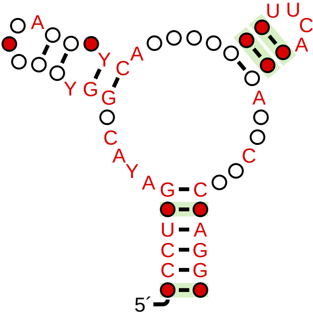

HMP2_3884_1

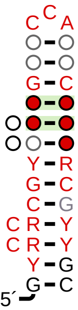

HMP2_389_1

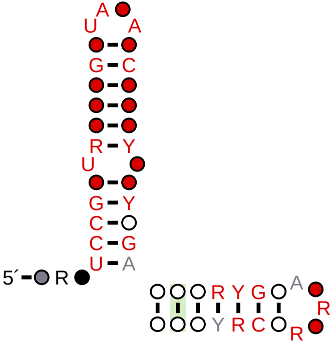

HMP2_3891_1

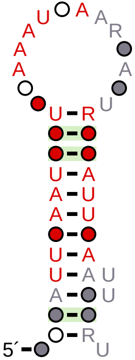

HMP2_3905_1

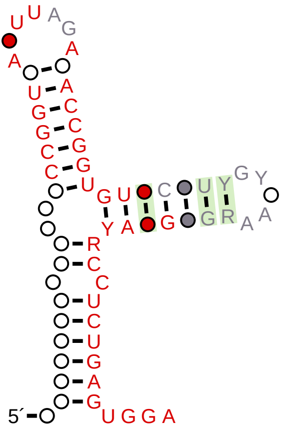

HMP2_3912_1

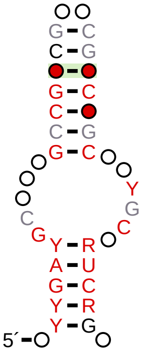

HMP2_3923_1

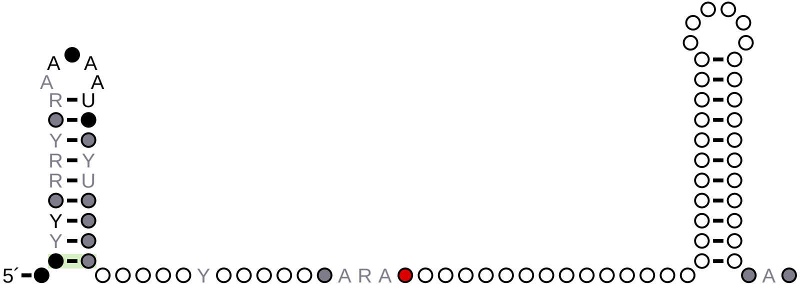

HMP2_3942_1

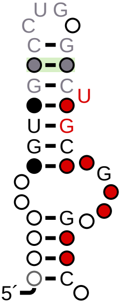

HMP2_3965_1

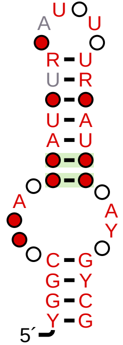

HMP2_3968_1

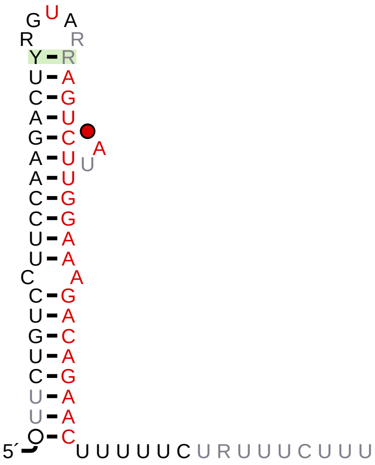

HMP2_3975_1

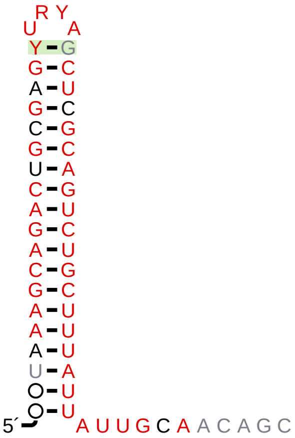

HMP2_3980_1

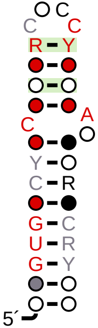

HMP2_3982_1

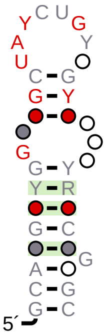

HMP2_3988_1

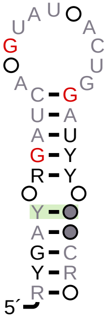

HMP2_3989_1

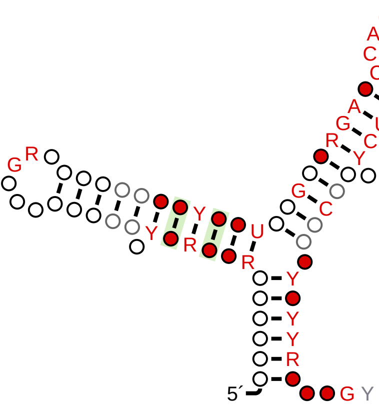

HMP2_399_1

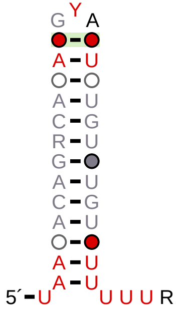

HMP2_3997_1

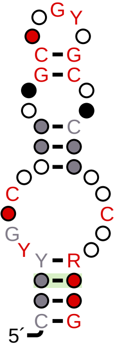

HMP2_4004_1

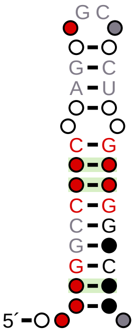

HMP2_4017_1

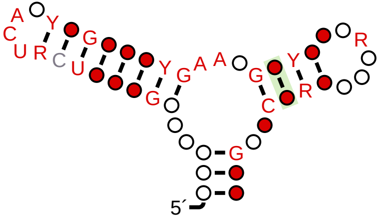

HMP2_4018_1

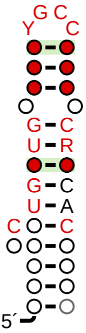

HMP2_402_1

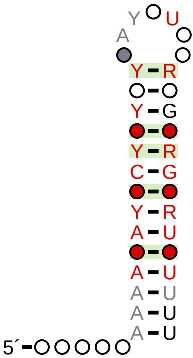

HMP2_4025_1

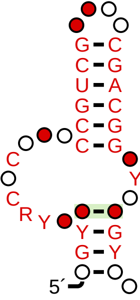

HMP2_4049_1

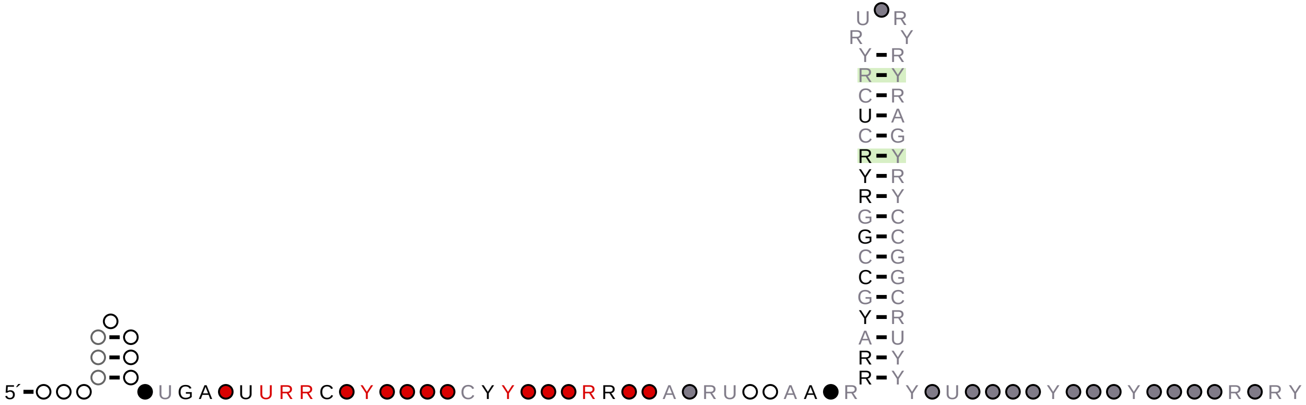

HMP2_4059_1

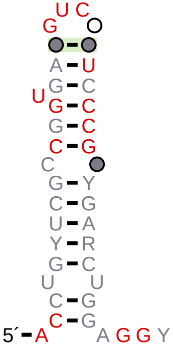

HMP2_406_1

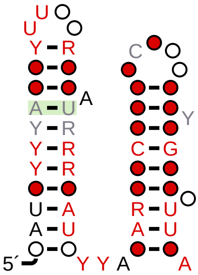

HMP2_4075_1

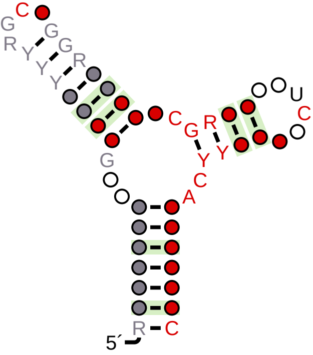

HMP2_409_1

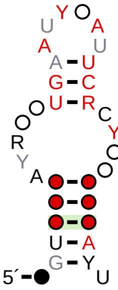

HMP2_4096_1

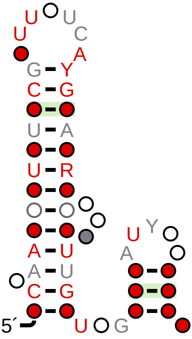

HMP2_4102_1

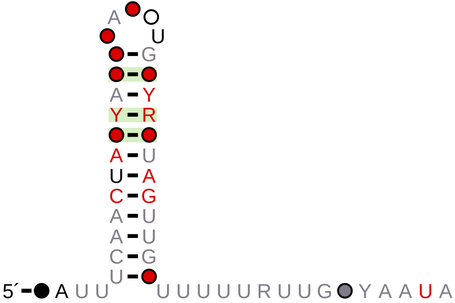

HMP2_4103_1

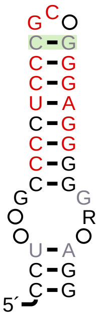

HMP2_4111_1

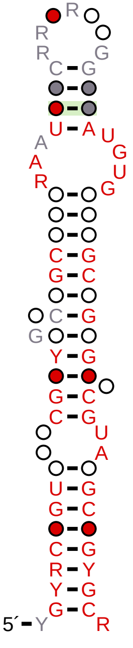

HMP2_4117_1

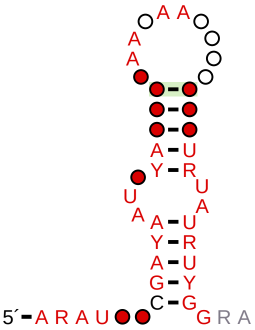

HMP2_4119_1

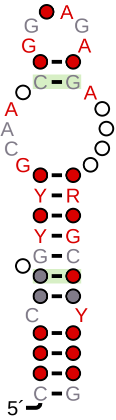

HMP2_4131_1

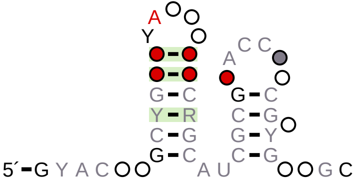

HMP2_414_1

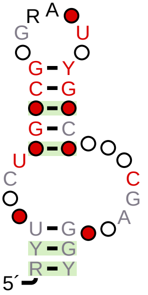

HMP2_4140_1

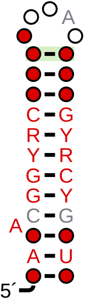

HMP2_4142_1

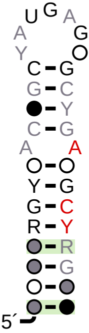

HMP2_4147_1

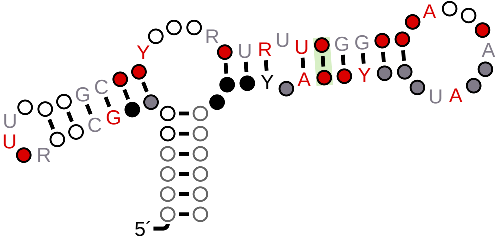

HMP2_415_1

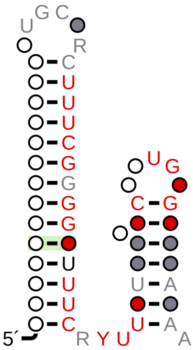

HMP2_4153_1

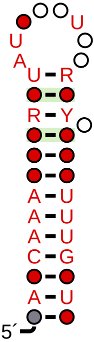

HMP2_4157_1

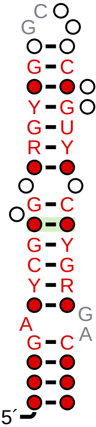

HMP2_4162_1

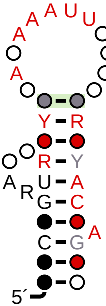

HMP2_4164_1

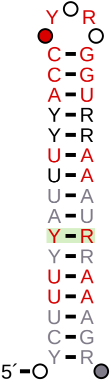

HMP2_4166_1

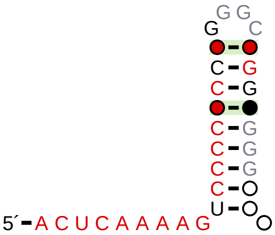

HMP2_4174_1

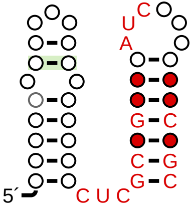

HMP2_4187_1

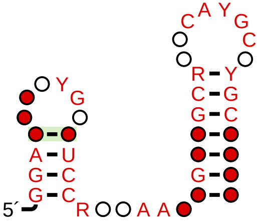

HMP2_4208_1

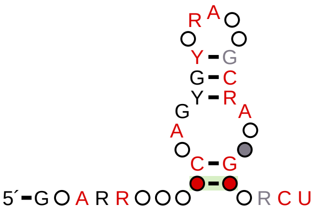

HMP2_4215_1

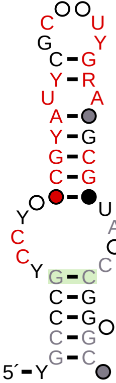

HMP2_4224_1

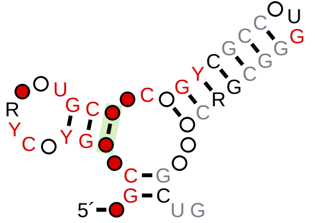

HMP2_4238_1

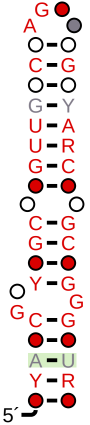

HMP2_4260_1

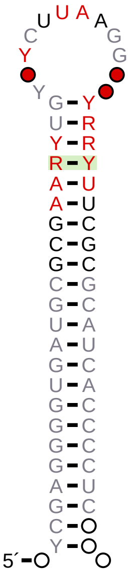

HMP2_4263_1

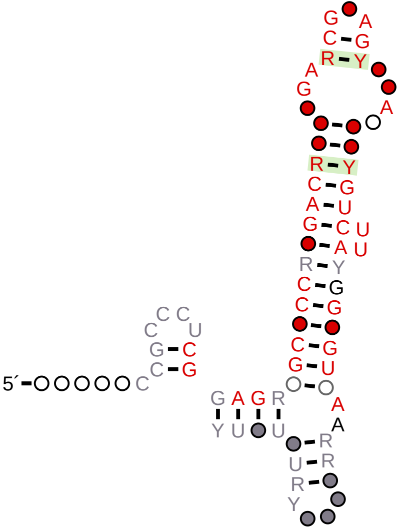

HMP2_4285_1

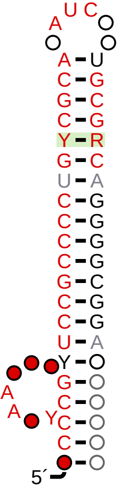

HMP2_430_1

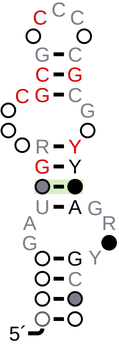

HMP2_4300_1

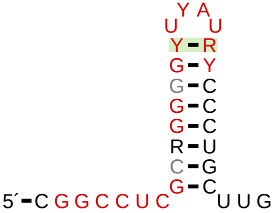

HMP2_4326_1

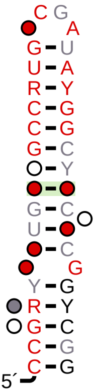

HMP2_4328_1

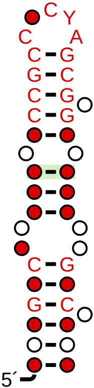

HMP2_4332_1

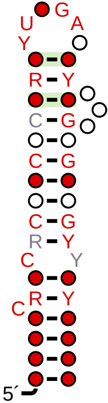

HMP2_4336_1

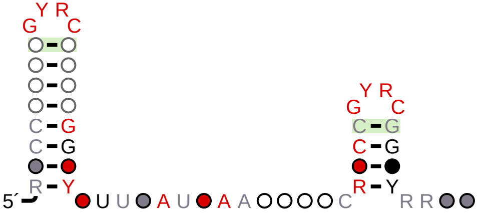

HMP2_4337_1

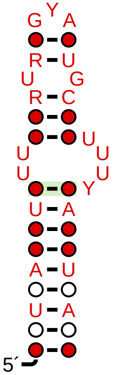

HMP2_4341_1

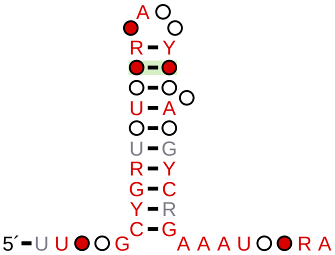

HMP2_4347_1

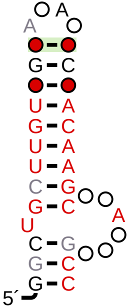

HMP2_4351_1

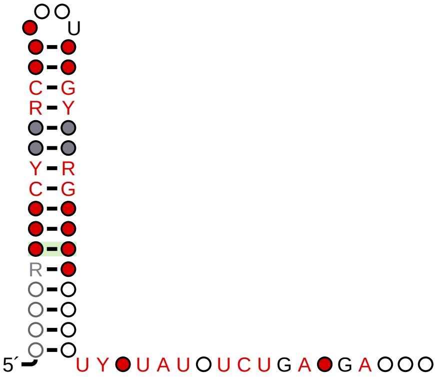

HMP2_4354_1

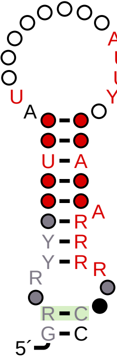

HMP2_4357_1

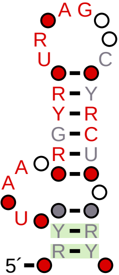

HMP2_4361_1

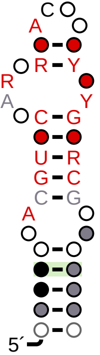

HMP2_4363_1

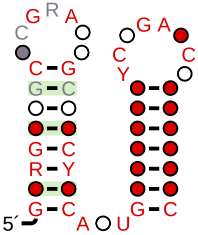

HMP2_4372_1

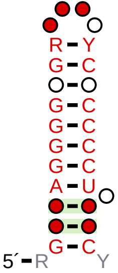

HMP2_4383_1

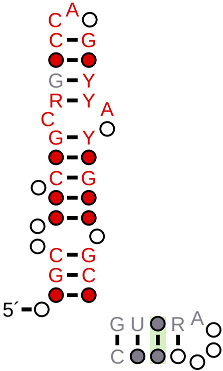

HMP2_4384_1

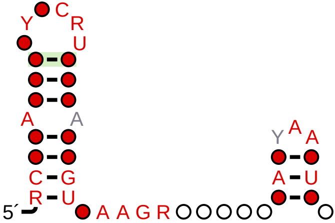

HMP2_4387_1

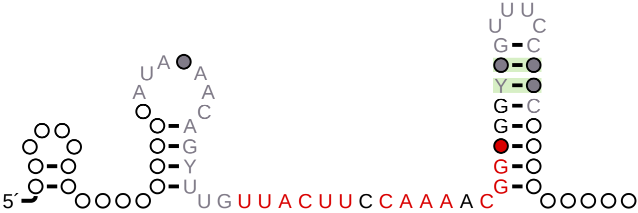

HMP2_4396_1

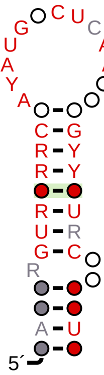

HMP2_4401_1

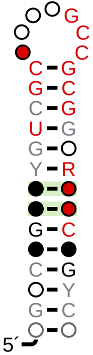

HMP2_4404_1

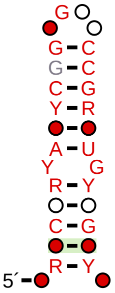

HMP2_4408_1

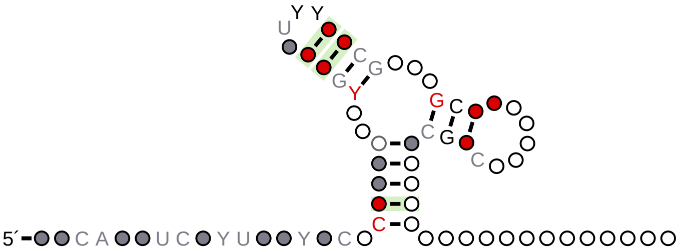

HMP2_4421_1

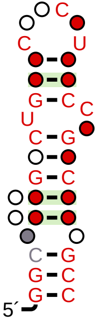

HMP2_4423_1

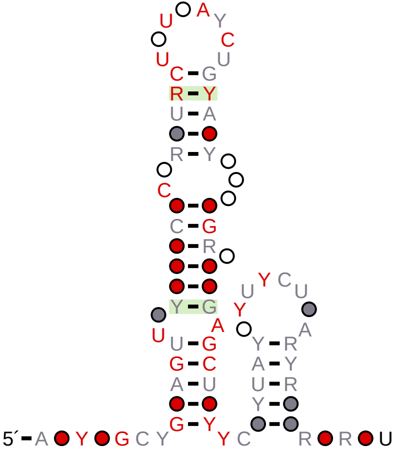

HMP2_4424_1

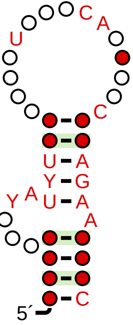

HMP2_4426_1

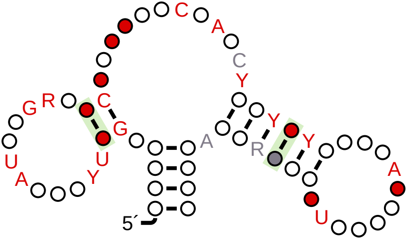

HMP2_4444_1

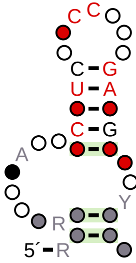

HMP2_4449_1

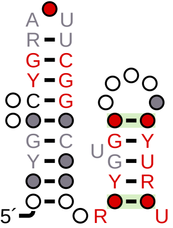

HMP2_4452_1

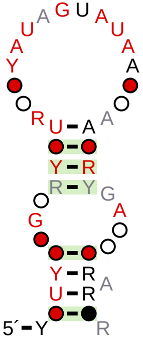

HMP2_4462_1

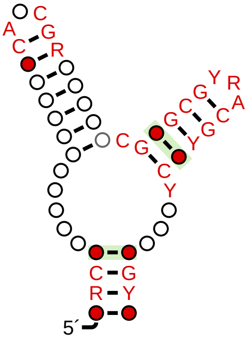

HMP2_4463_1

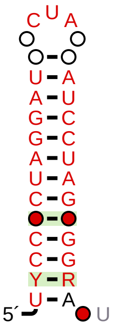

HMP2_4464_1

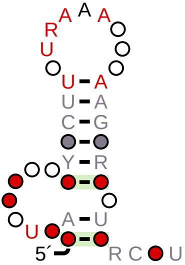

HMP2_447_1

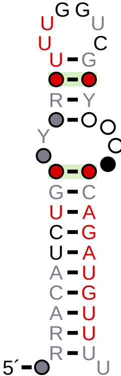

HMP2_4474_1

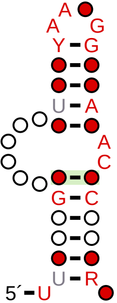

HMP2_4475_1

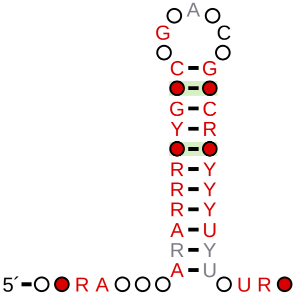

HMP2_4484_1

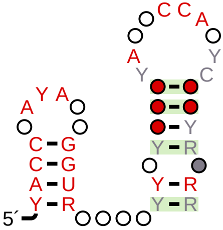

HMP2_4503_1

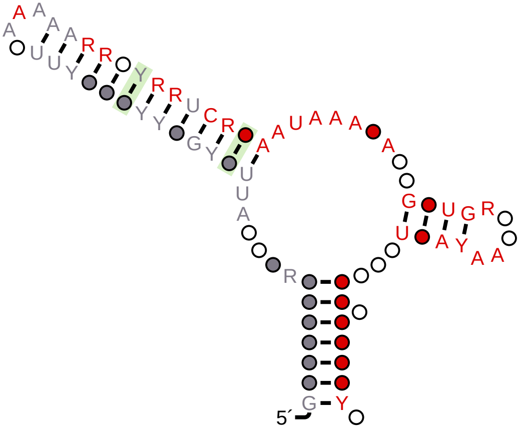

HMP2_4508_1

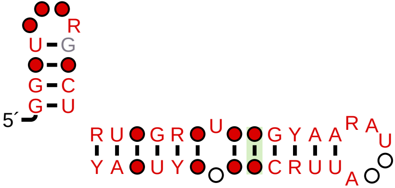

HMP2_4515_1

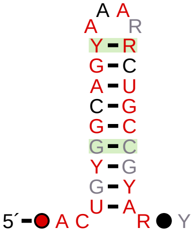

HMP2_4516_1

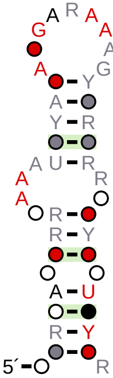

HMP2_4525_1

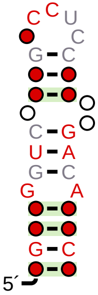

HMP2_4537_1

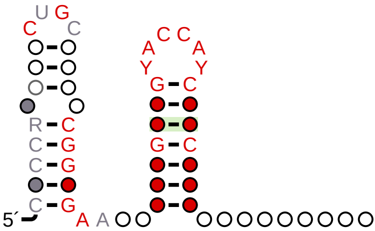

HMP2_4539_1

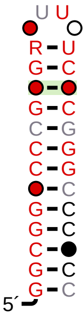

HMP2_4553_1

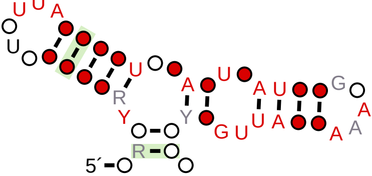

HMP2_4554_1

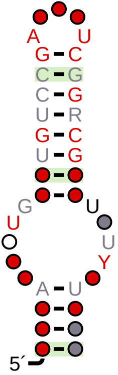

HMP2_4558_1

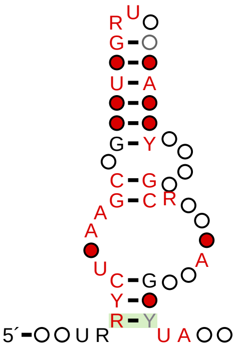

HMP2_4571_1

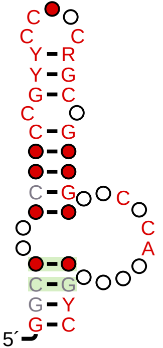

HMP2_4574_1

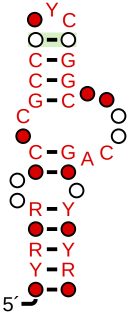

HMP2_4580_1

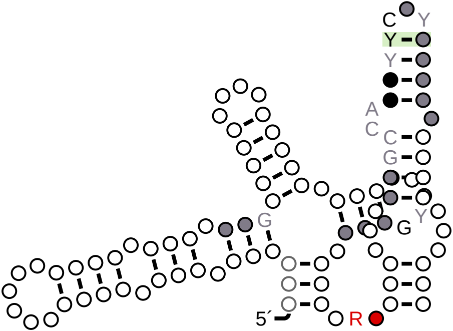

HMP2_4590_1

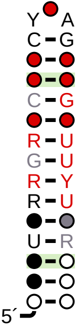

HMP2_4592_1

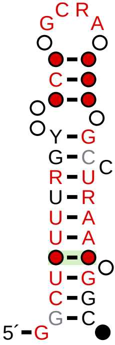

HMP2_4595_1

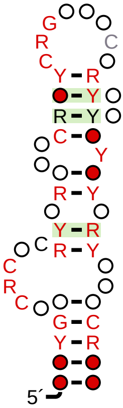

HMP2_4598_1

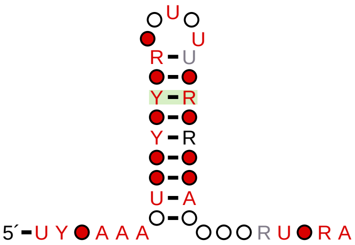

HMP2_4603_1

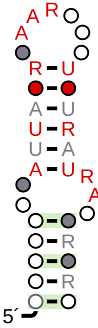

HMP2_4605_1

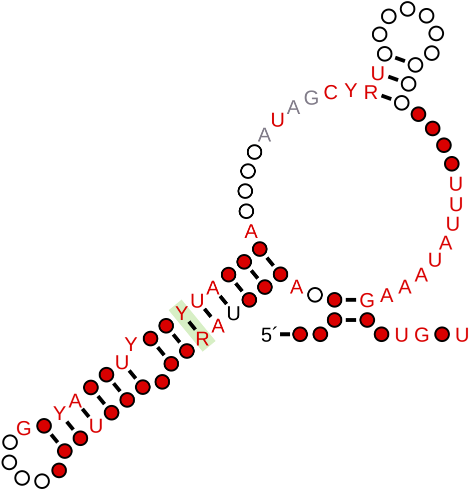

HMP2_4609_1

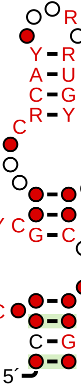

HMP2_4623_1

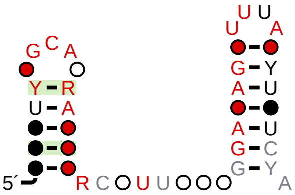

HMP2_4628_1

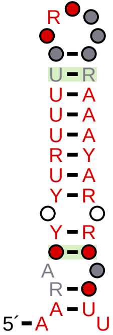

HMP2_4633_1

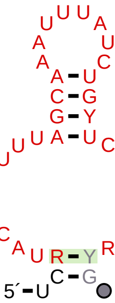

HMP2_4634_1

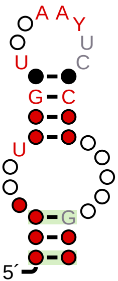

HMP2_4637_1

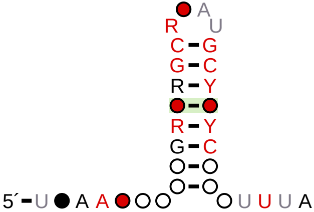

HMP2_4639_1

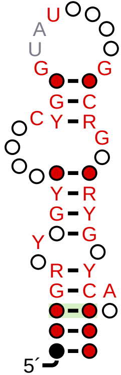

HMP2_466_1

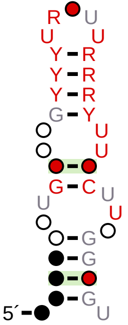

HMP2_4667_1

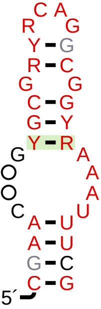

HMP2_4684_1

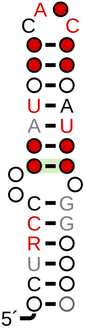

HMP2_4688_1

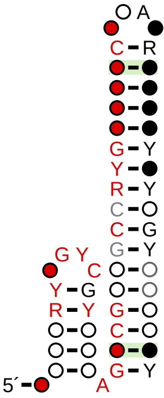

HMP2_4689_1

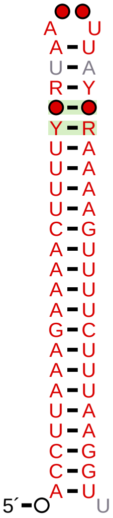

HMP2_4699_1

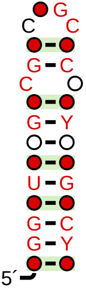

HMP2_4702_1

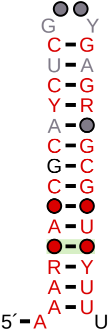

HMP2_4705_1

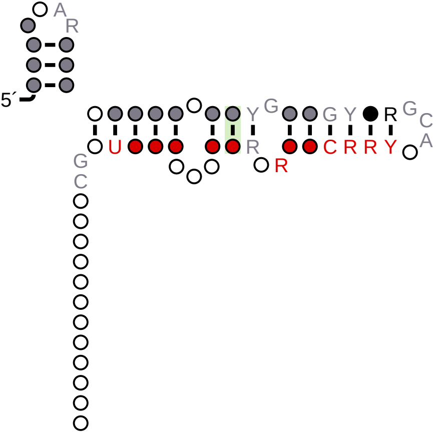

HMP2_4706_1

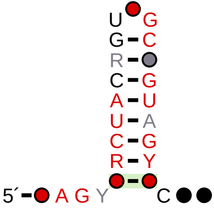

HMP2_4713_1

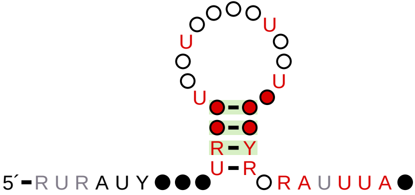

HMP2_4719_1

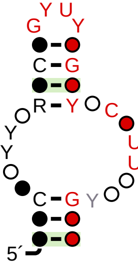

HMP2_4722_1

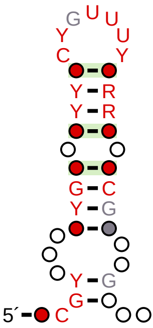

HMP2_4734_1

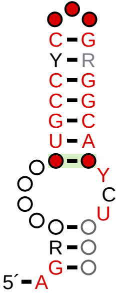

HMP2_4735_1

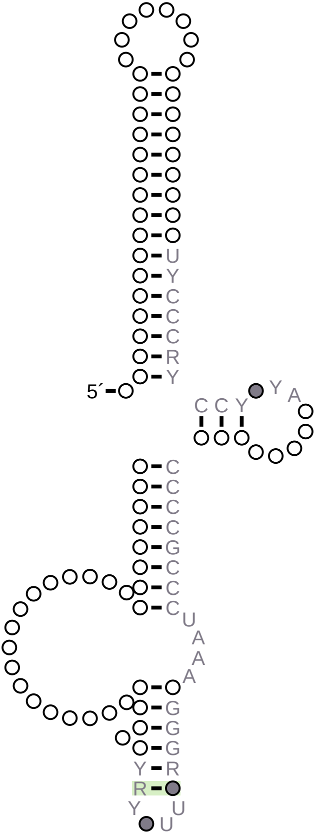

HMP2_4741_1

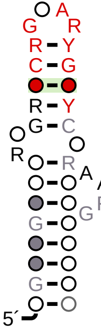

HMP2_4757_1

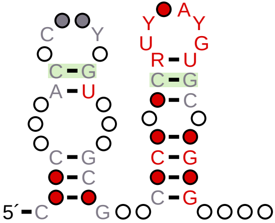

HMP2_4764_1

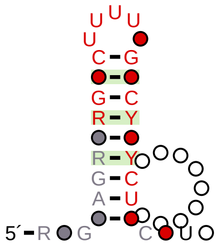

HMP2_4766_1

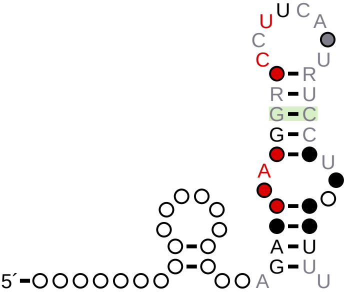

HMP2_4772_1

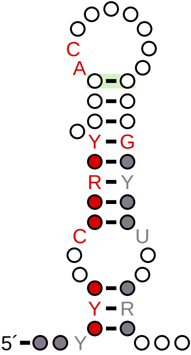

HMP2_4783_1

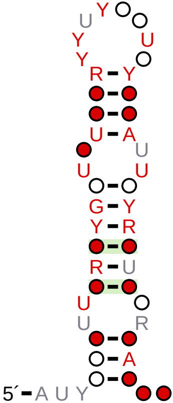

HMP2_4801_1

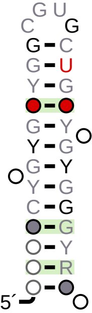

HMP2_4808_1

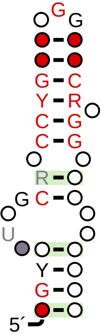

HMP2_481_1

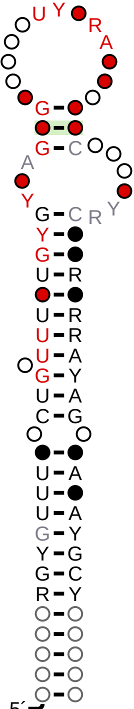

HMP2_4811_1

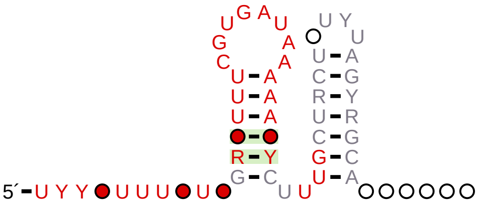

HMP2_4812_1

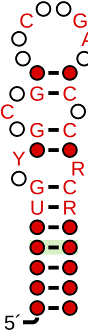

HMP2_4818_1

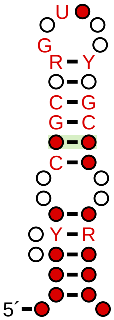

HMP2_4819_1

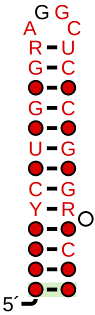

HMP2_4828_1

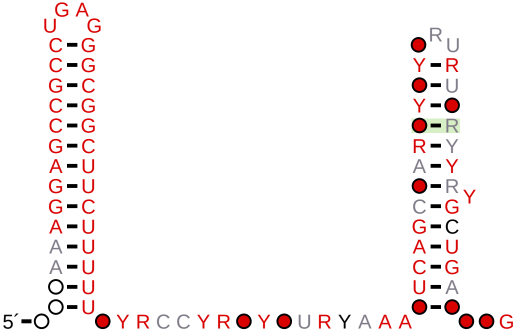
5´
GA
U G
C-G
C-G
G-C
C-G
C-G
G-C
A-U
G-U
G-C
A-U
A-U
A-U
-U
-U
YRCCYRYURYAAA
G
RU
Y-R
-U
Y-
-R
R-Y
A-Y
-R Y
C-G
G-C
A-U
C-G
U-A

HMP2_4829_1

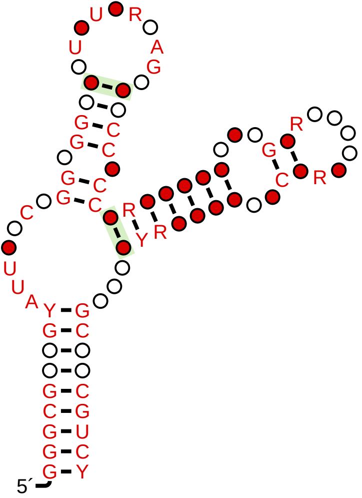

HMP2_4836_1

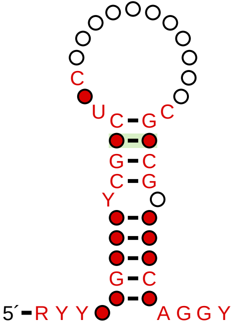

HMP2_4839_1

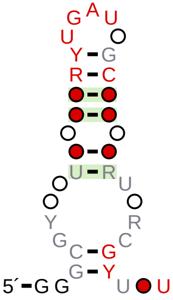

HMP2_4851_1

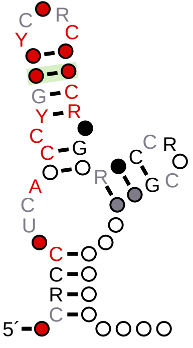

HMP2_4860_1

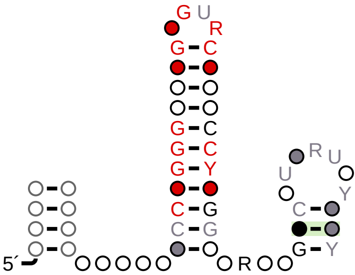

HMP2_4861_1

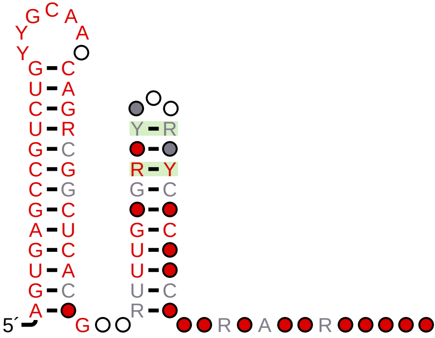

HMP2_4871_1

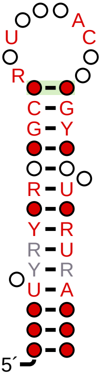

HMP2_4884_1

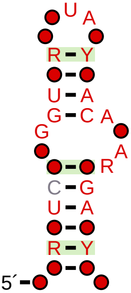

HMP2_4892_1

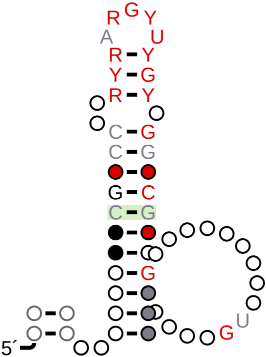

HMP2_4893_1

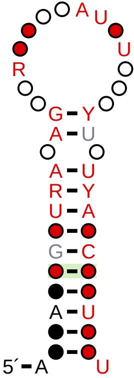

HMP2_4904_1

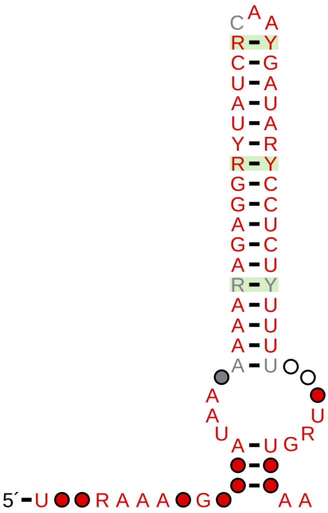

HMP2_4924_1

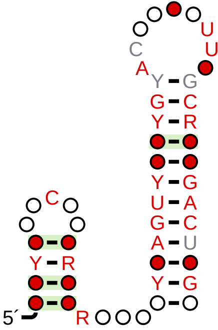

HMP2_4933_1

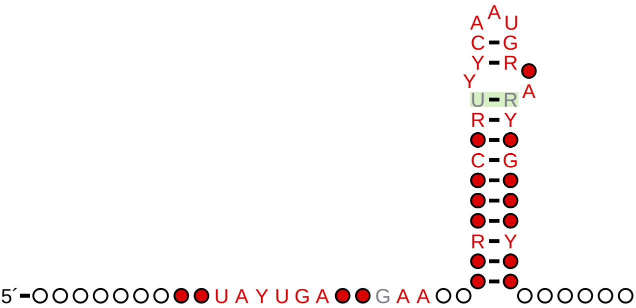

HMP2_494_1

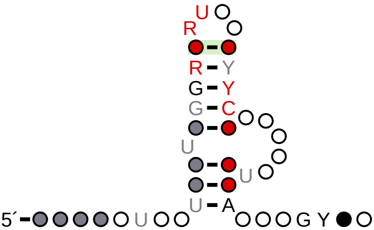

HMP2_4958_1

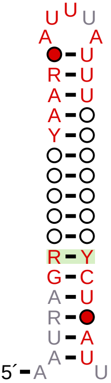

HMP2_4960_1

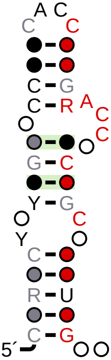

HMP2_4965_1

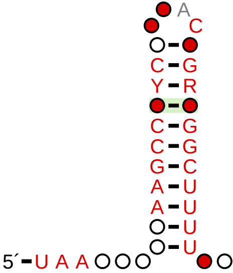

HMP2_4971_1

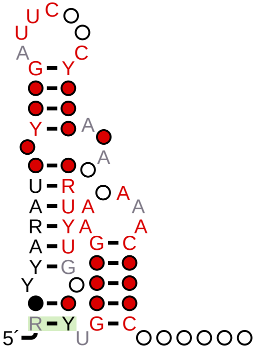

HMP2_4996_1

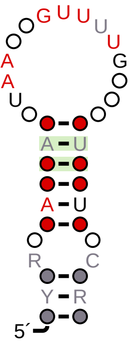

HMP2_5_1

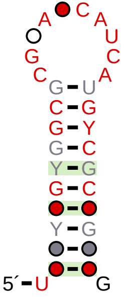

HMP2_5007_1

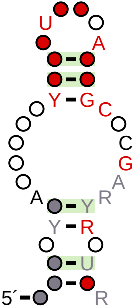

HMP2_5011_1

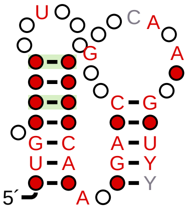

HMP2_5012_1

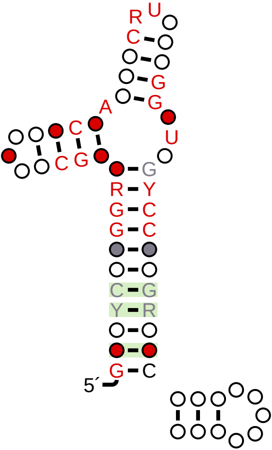

HMP2_5021_1

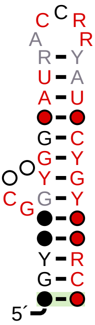

HMP2_5055_1

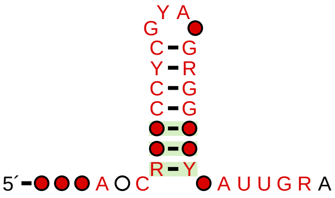

HMP2_5059_1

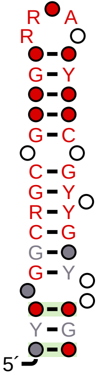

HMP2_5063_1

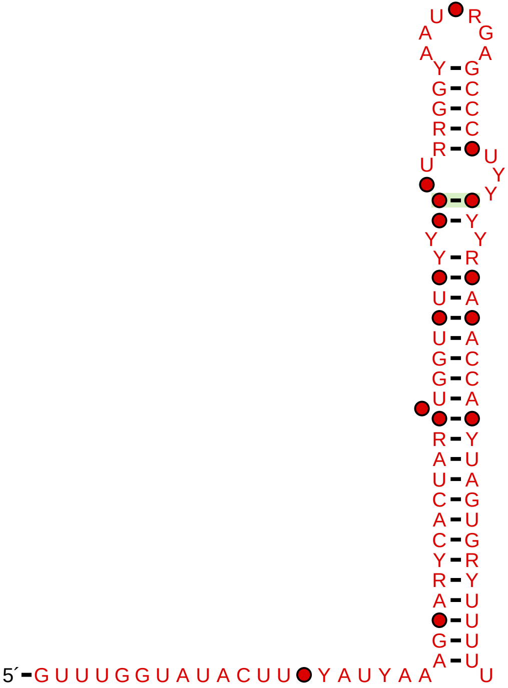

HMP2_5101_1

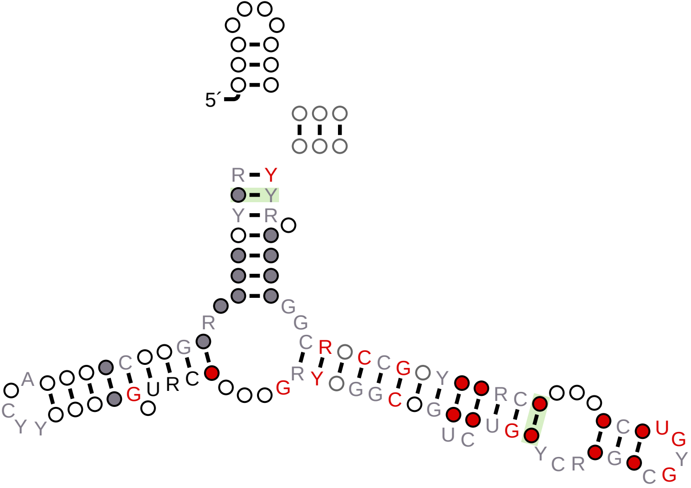

HMP2_5108_1

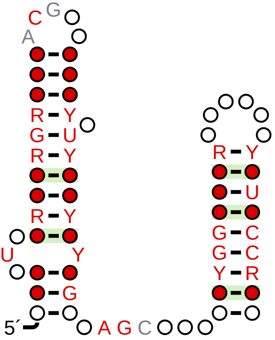

HMP2_512_1

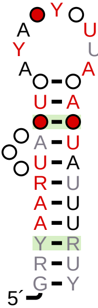

HMP2_5122_1

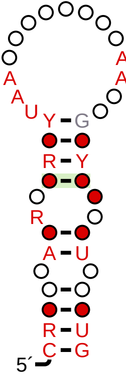

HMP2_5123_1

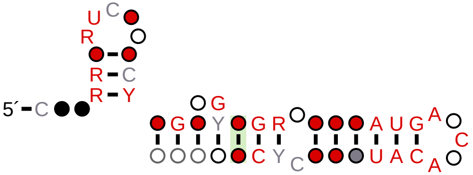

HMP2_5134_1

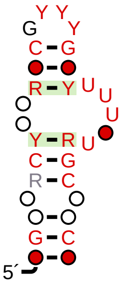

HMP2_5136_1

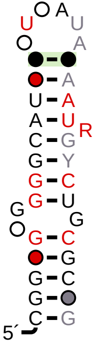

HMP2_5142_1

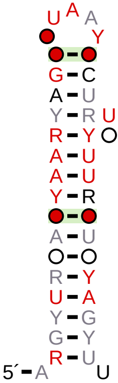

HMP2_5144_1

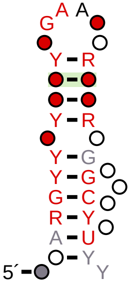

HMP2_5148_1

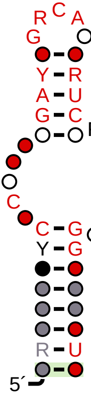

HMP2_5162_1

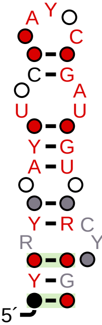

HMP2_5166_1

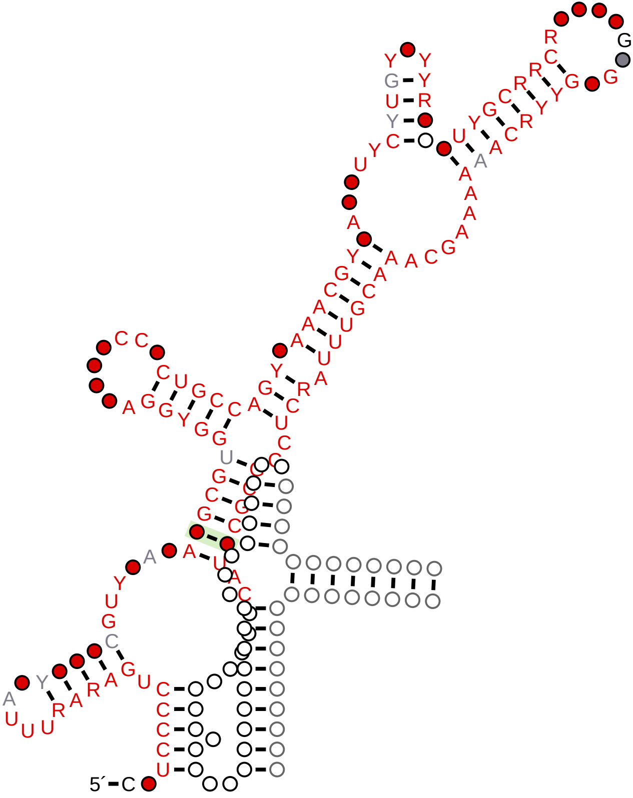

HMP2_5173_1

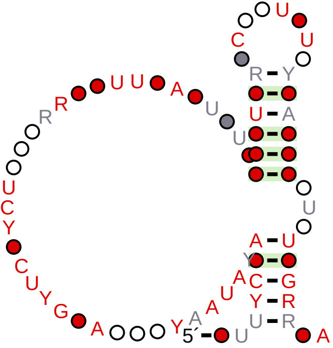

HMP2_5181_1

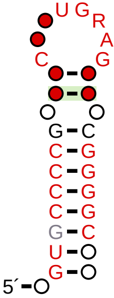

HMP2_5184_1

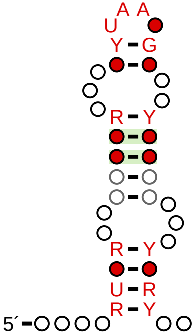

HMP2_5185_1

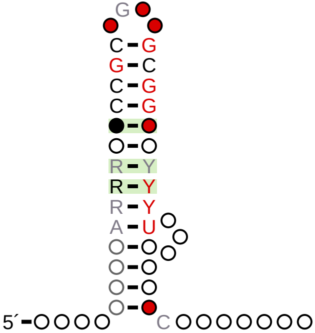

HMP2_5191_1

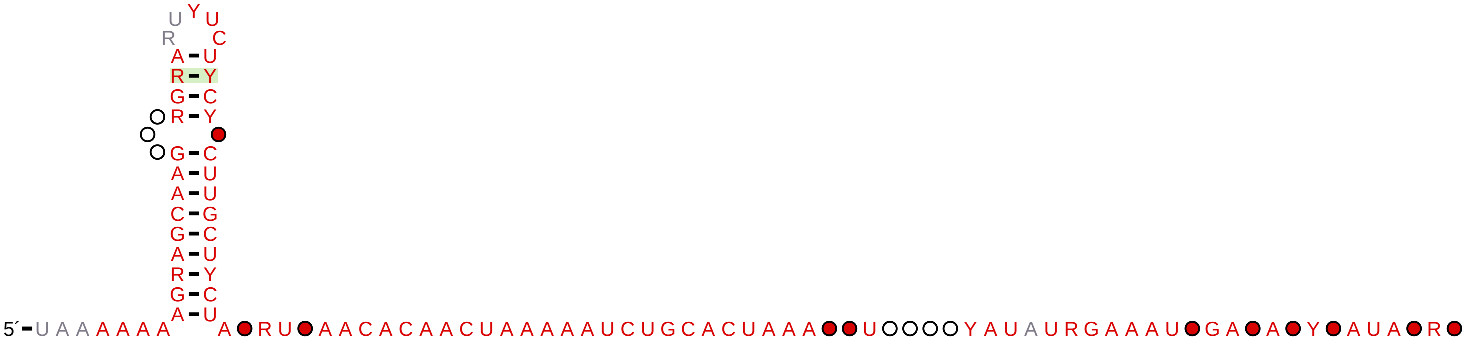

HMP2_5217_1

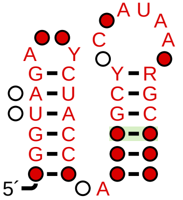

HMP2_5240_1

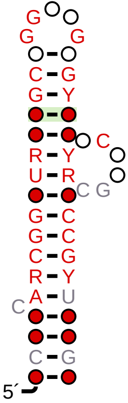

HMP2_526_1

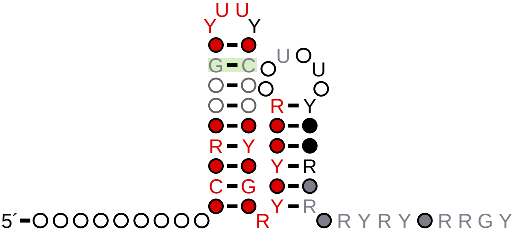

HMP2_5267_1

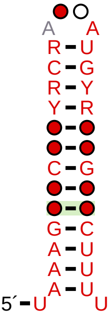

HMP2_5289_1

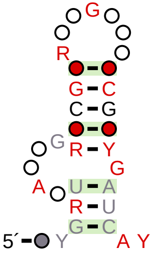

HMP2_5302_1

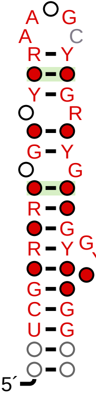

HMP2_5319_1

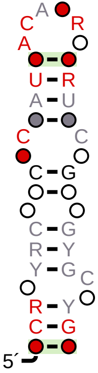

HMP2_5321_1

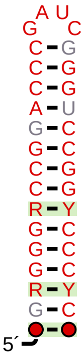

HMP2_5322_1

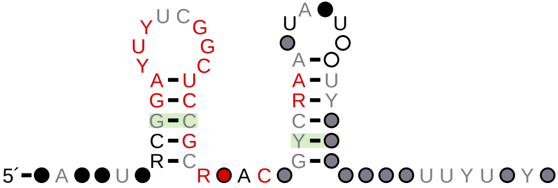

HMP2_5325_1

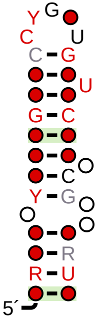

HMP2_5345_1

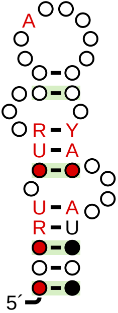

HMP2_5350_1

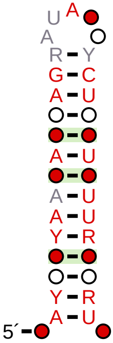

HMP2_5387_1

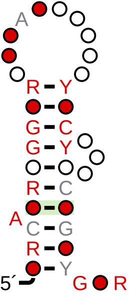

HMP2_5395_1

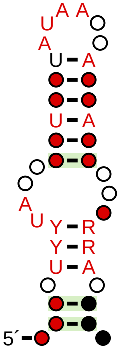

HMP2_5401_1

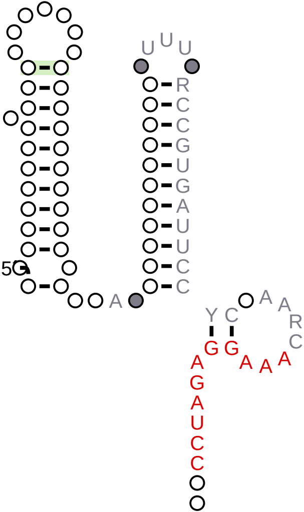

HMP2_5407_1

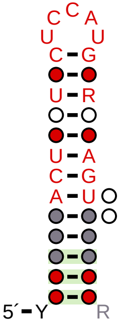

HMP2_542_1

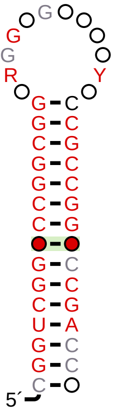

HMP2_5420_1

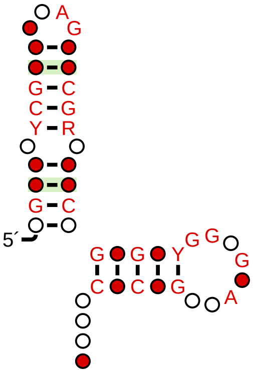

HMP2_5425_1

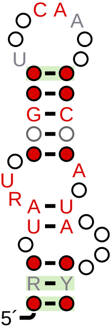

HMP2_5442_1

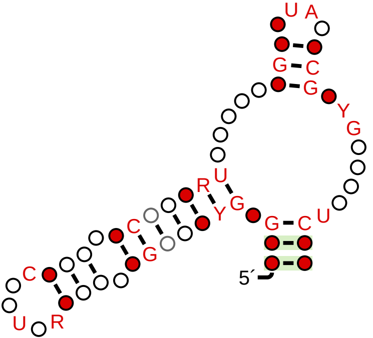

HMP2_5467_1

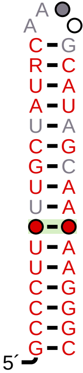

HMP2_5469_1

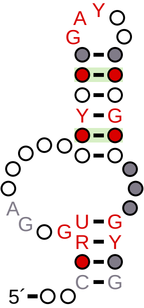

HMP2_5474_1

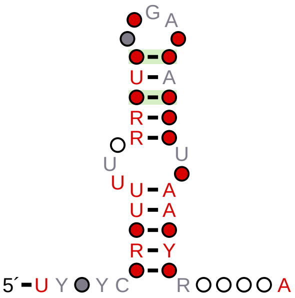

HMP2_5485_1

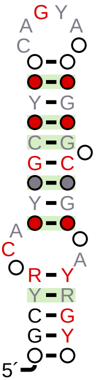

HMP2_5488_1

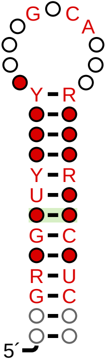

HMP2_5499_1

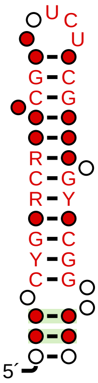

HMP2_5520_1

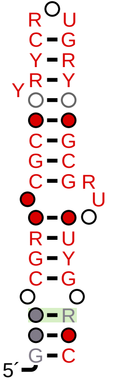

HMP2_553_1

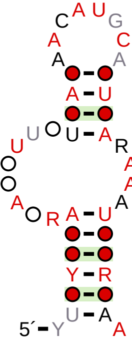

HMP2_5533_1

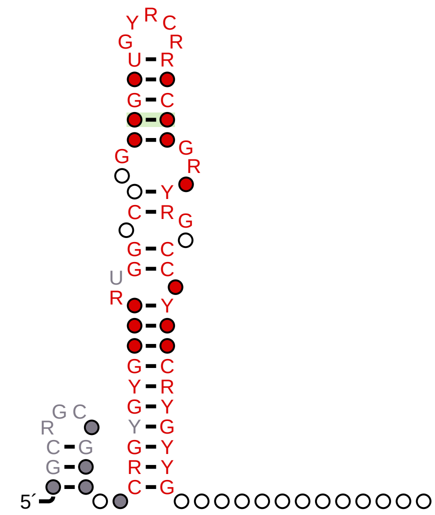

HMP2_5571_1

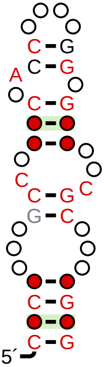

HMP2_5573_1

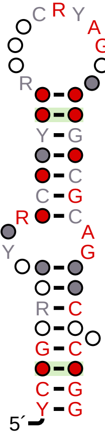

HMP2_5583_1

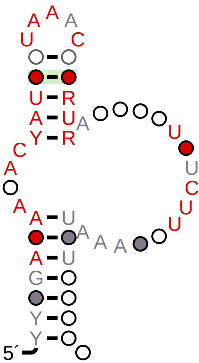

HMP2_5587_1

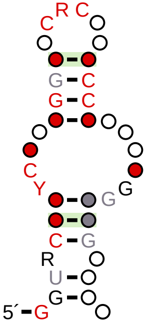

HMP2_5591_1

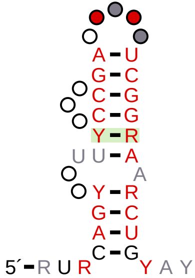

HMP2_5595_1

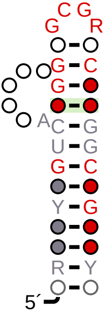

HMP2_5600_1

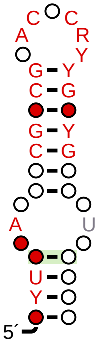

HMP2_5601_1

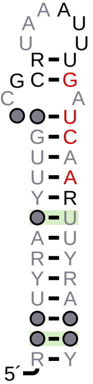

HMP2_5603_1

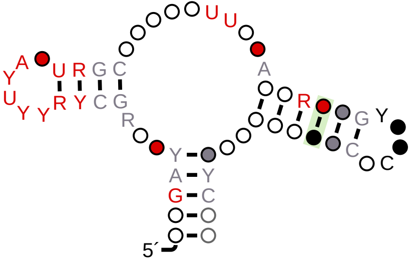

HMP2_5613_1

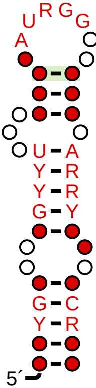

HMP2_5626_1

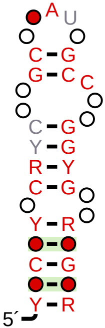

HMP2_5627_1

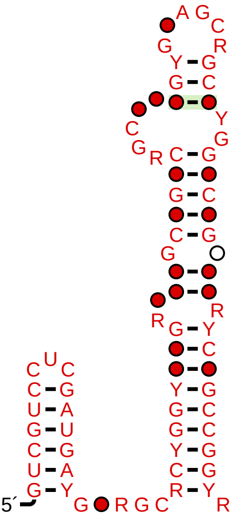

HMP2_5632_1

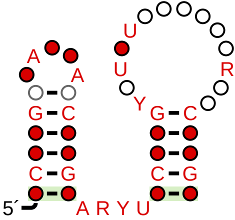

HMP2_5633_1

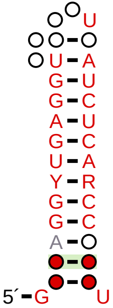

HMP2_5648_1

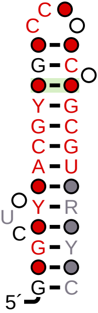

HMP2_565_1

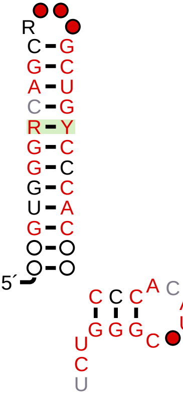

HMP2_5652_1

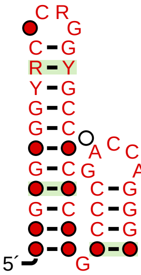

HMP2_5655_1

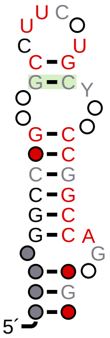

HMP2_5676_1

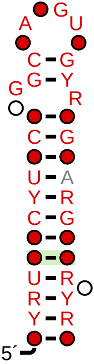

HMP2_5680_1

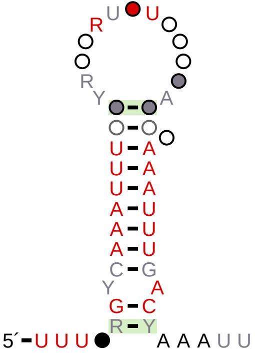

HMP2_5684_1

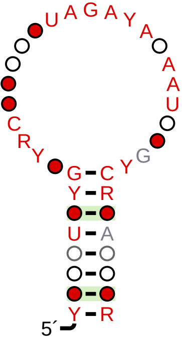

HMP2_5688_1

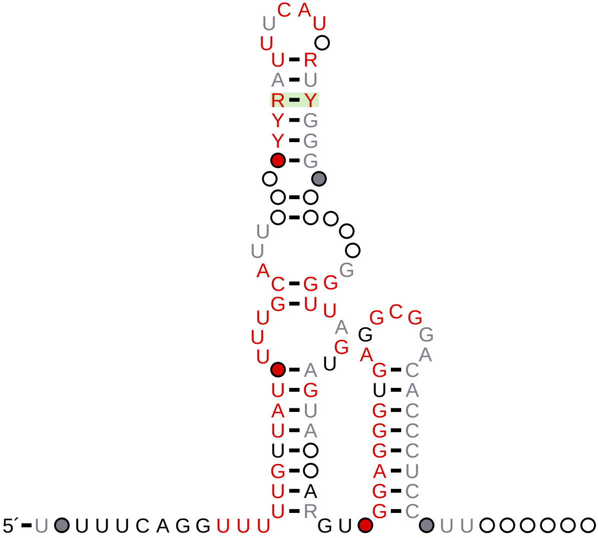

HMP2_5714_1

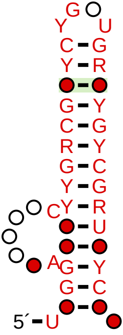

HMP2_5715_1

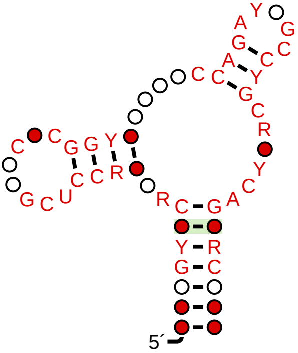

HMP2_572_1

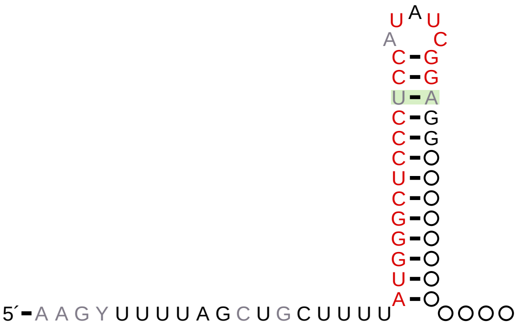

HMP2_5722_1

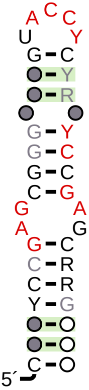

HMP2_5723_1

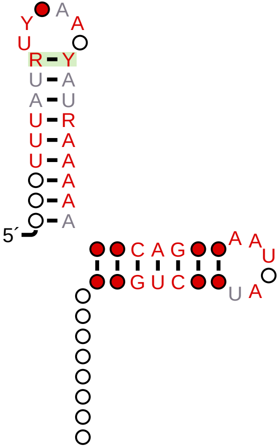

HMP2_5724_1

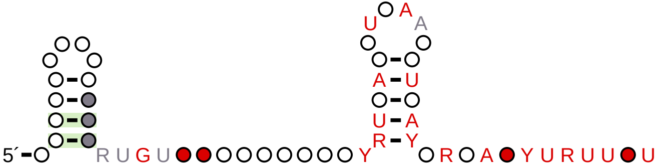

HMP2_5728_1

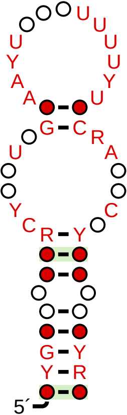

HMP2_5737_1

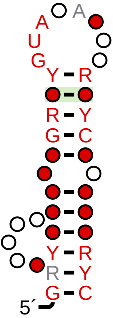

HMP2_5739_1

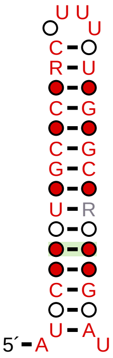

HMP2_575_1

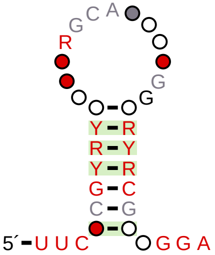

HMP2_5755_1

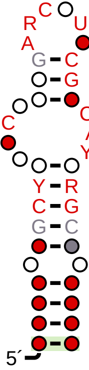

HMP2_5762_1

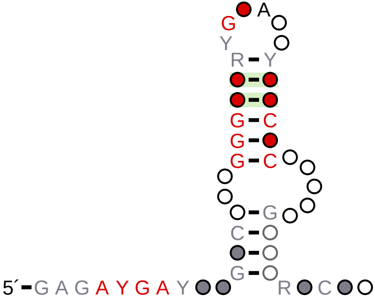

HMP2_5765_1

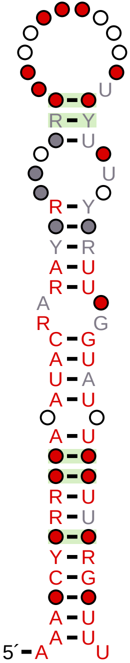

HMP2_5775_1

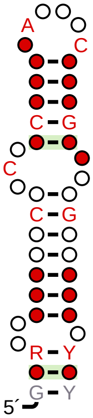

HMP2_5783_1

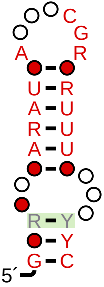

HMP2_5793_1

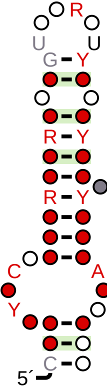

HMP2_5800_1

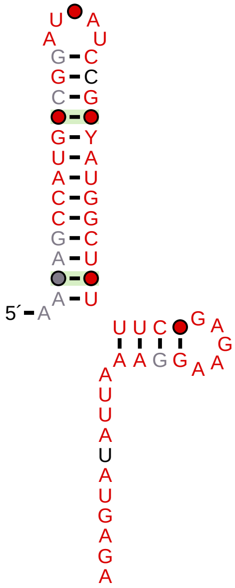

HMP2_5802_1

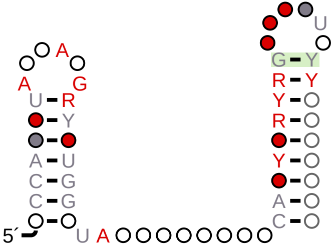

HMP2_5811_1

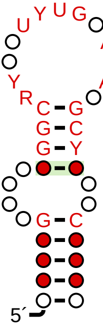

HMP2_5819_1

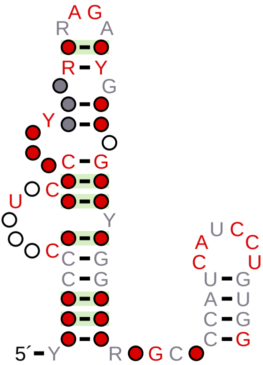

HMP2_5825_1

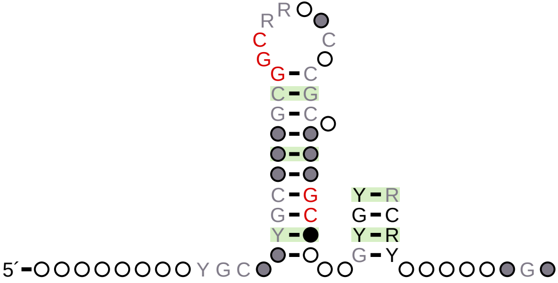

HMP2_5839_1

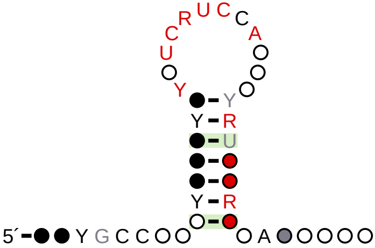

HMP2_584_1

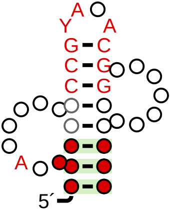

HMP2_5843_1

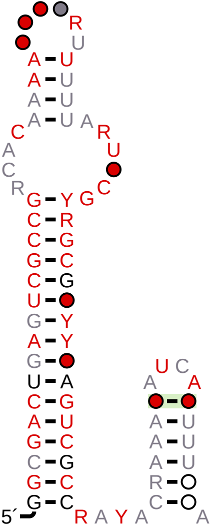

HMP2_5849_1

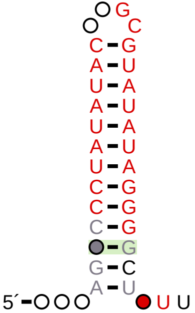

HMP2_5851_1

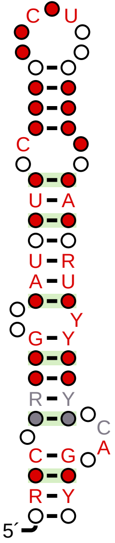

HMP2_5853_1

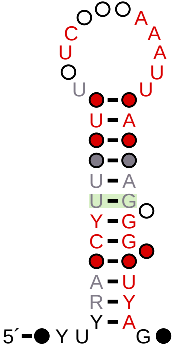

HMP2_5857_1

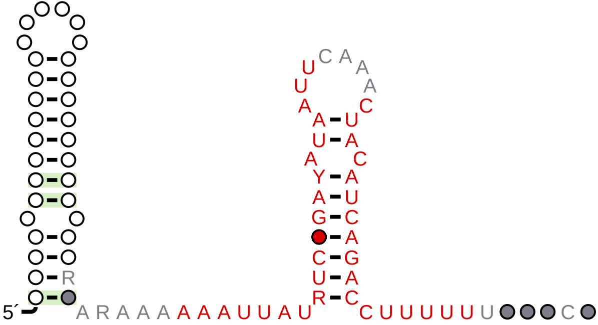

C A A A C U A A U A U C A Y A A U G C A C G U A R C
U U A
5´
A R A A A A A A U U A U
C U U U U U U C
R

HMP2_5878_1

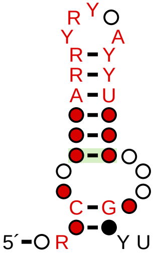

HMP2_5879_1

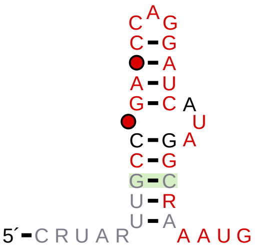

HMP2_588_1

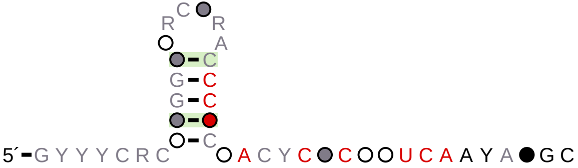

HMP2_5880_1

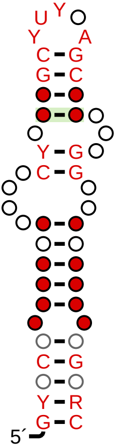

HMP2_5893_1

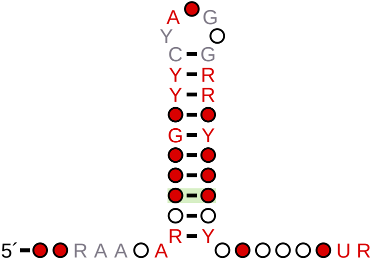

HMP2_5899_1

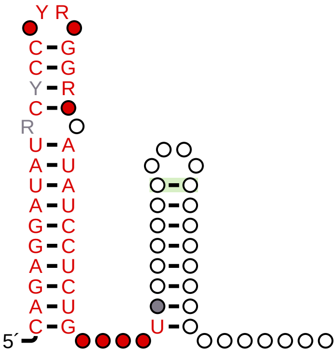

HMP2_5919_1

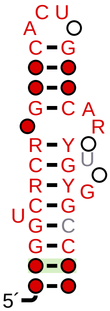

HMP2_5921_1

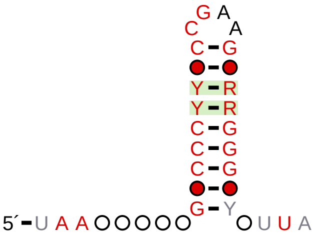

HMP2_5922_1

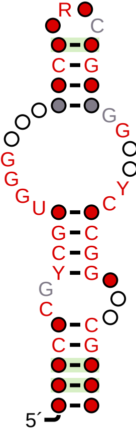

HMP2_5936_1

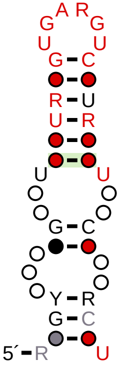

HMP2_5951_1

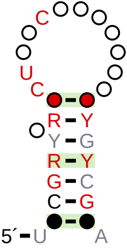

HMP2_5955_1

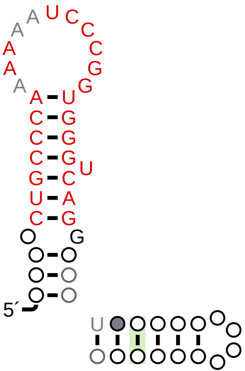

HMP2_5960_1

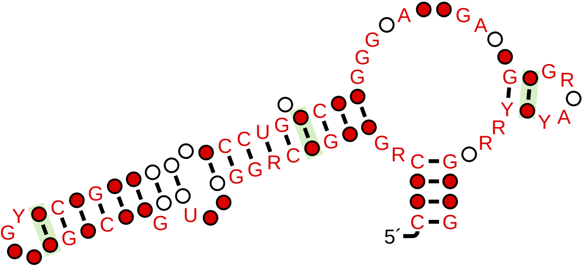

HMP2_5982_1

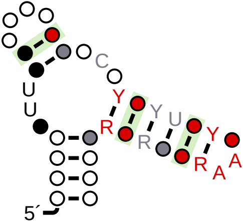

HMP2_5986_1

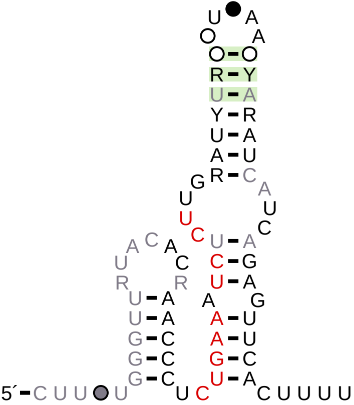

HMP2_5989_1

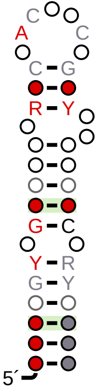

HMP2_600_1

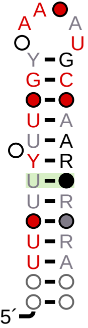

HMP2_602_1

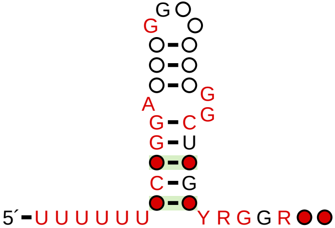

HMP2_6023_1

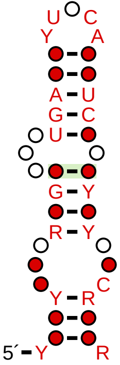

HMP2_603_1

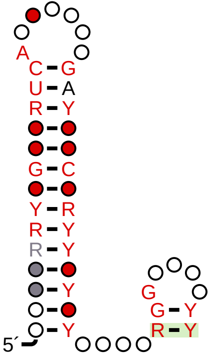

HMP2_6036_1

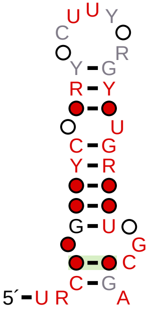

HMP2_6062_1

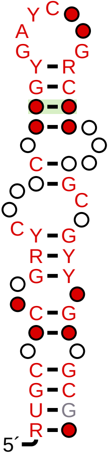

HMP2_608_1

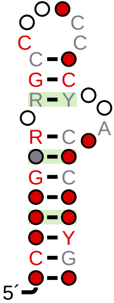

HMP2_610_1

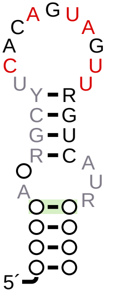

HMP2_6117_1

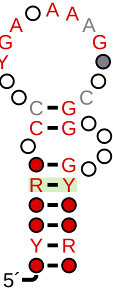

HMP2_6121_1

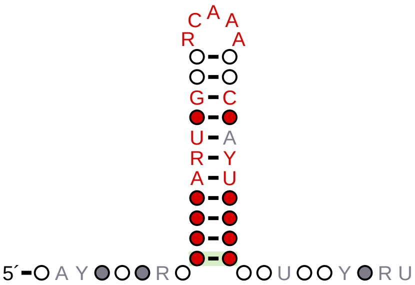

HMP2_6122_1

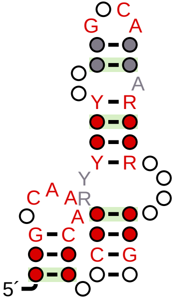

HMP2_6126_1

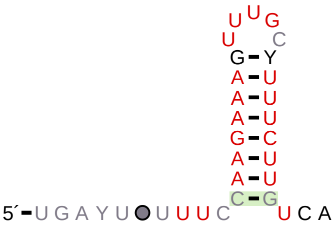

HMP2_6137_1

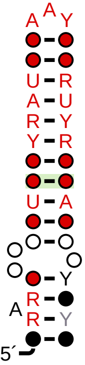

HMP2_6152_1

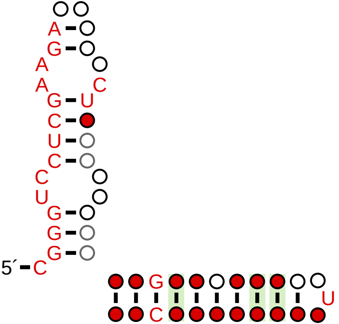

HMP2_6156_1

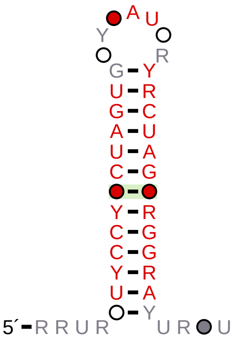

HMP2_6157_1

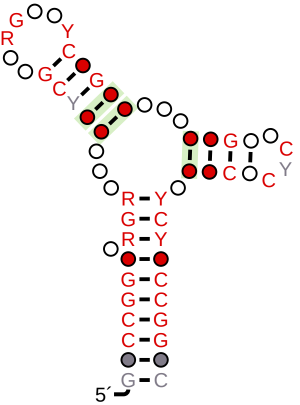

HMP2_6162_1

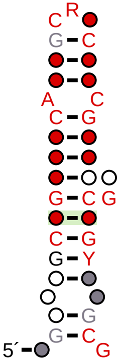

HMP2_6172_1

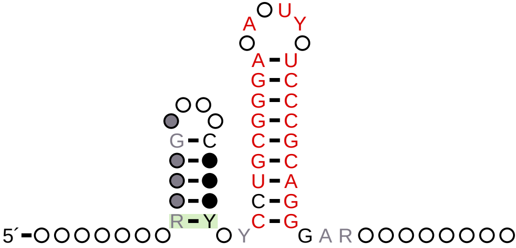

HMP2_6176_1

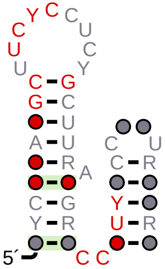

HMP2_6183_1

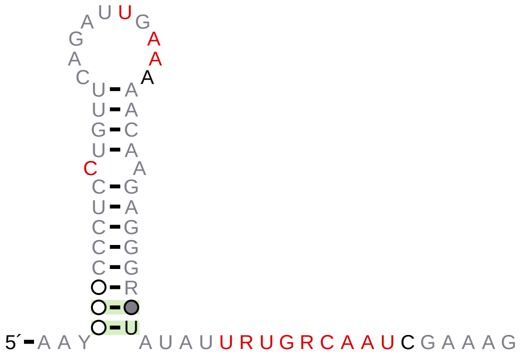

A U U G
G A
A A
C A
U A
U A
G C
U A
C A
C G
U A
C G
C G
C G
R
U
5′ A A Y A U A U U R U G R C A A U C G A A A G

HMP2_6189_1

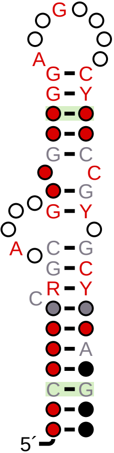

HMP2_6193_1

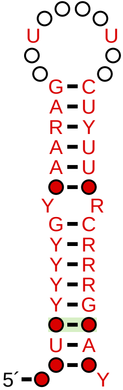

HMP2_6201_1

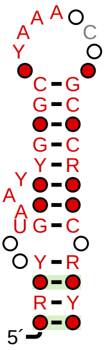

HMP2_6203_1

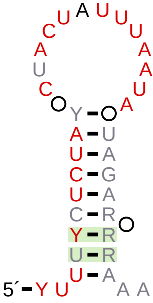

HMP2_6206_1

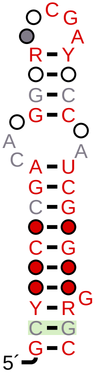

HMP2_6216_1

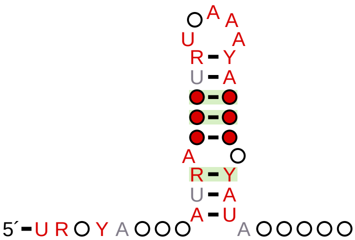

HMP2_6217_1

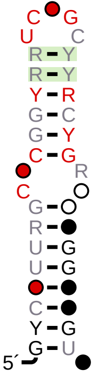

HMP2_6218_1

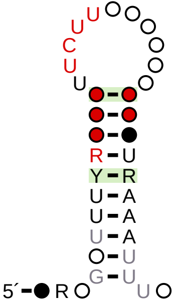

HMP2_6220_1

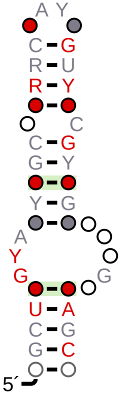

HMP2_6227_1

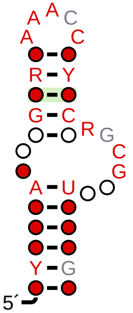

HMP2_6233_1

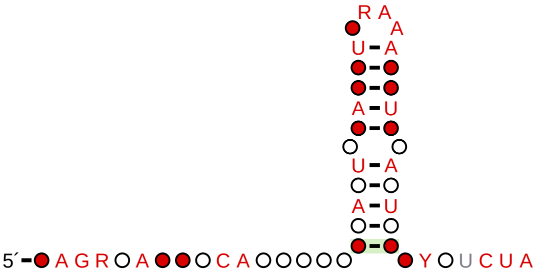

HMP2_6237_1

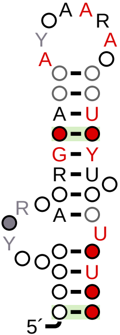

HMP2_6241_1

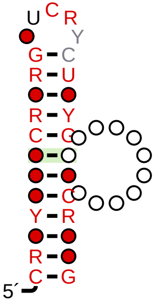

HMP2_6245_1

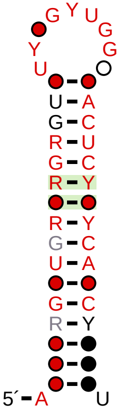

HMP2_6261_1

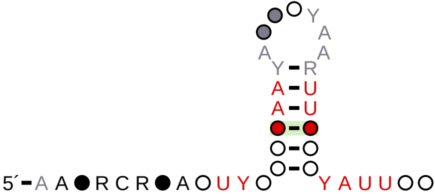

HMP2_6263_1

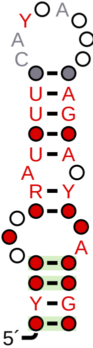

HMP2_6275_1

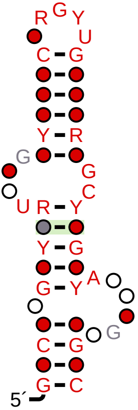

HMP2_6286_1

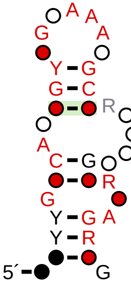

HMP2_6289_1

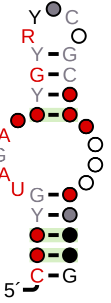

HMP2_6314_1

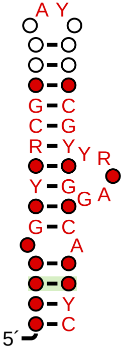

HMP2_632_1

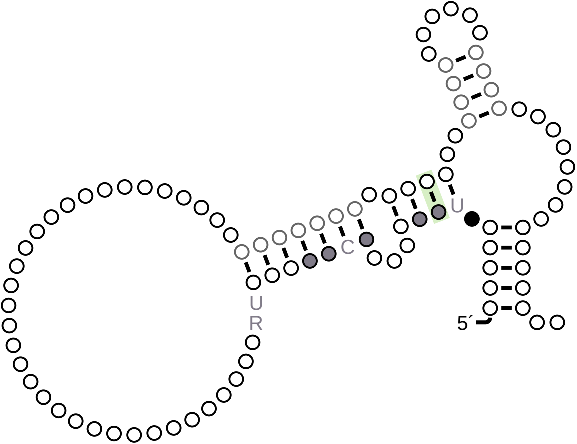

HMP2_6331_1

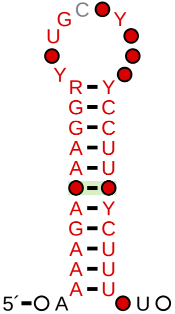

HMP2_6339_1

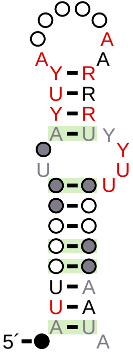

HMP2_634_1

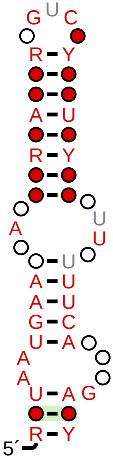

HMP2_6348_1

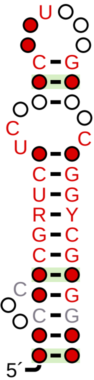

HMP2_6349_1

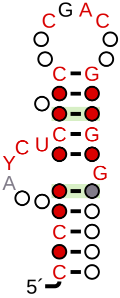

HMP2_6351_1

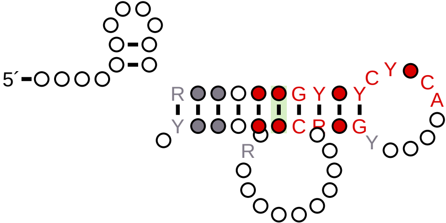

HMP2_6359_1

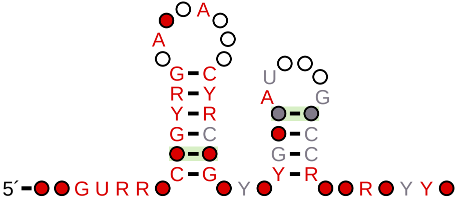

HMP2_6364_1

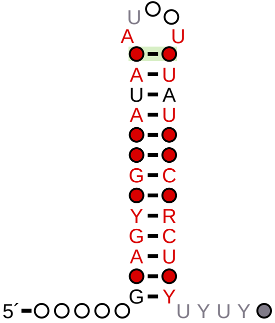

HMP2_638_1

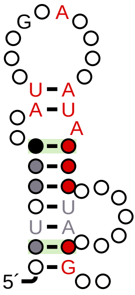

HMP2_6385_1

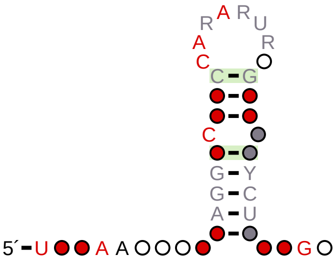

HMP2_6393_1

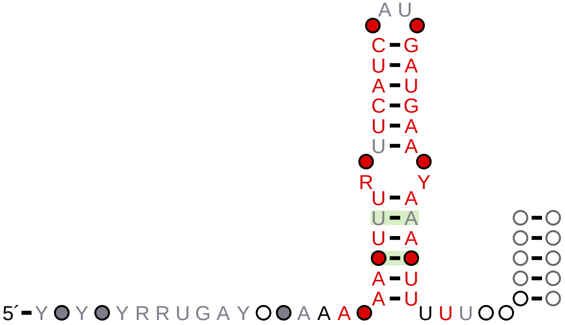

HMP2_6397_1

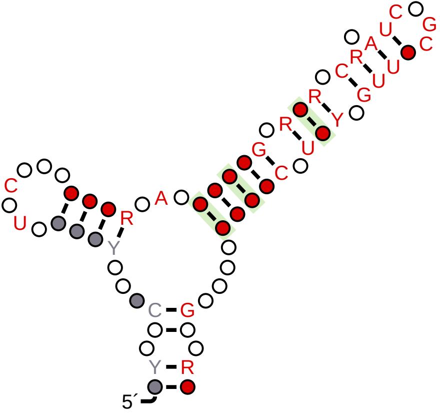

HMP2_6399_1

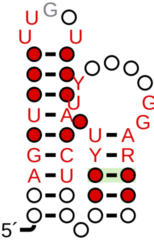

HMP2_6409_1

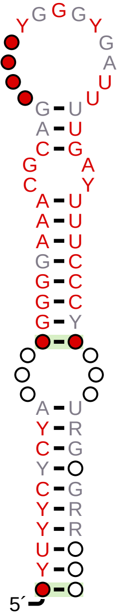

HMP2_6410_1

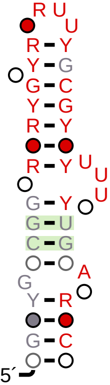

HMP2_6424_1

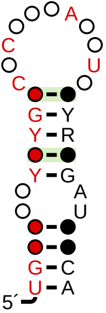

HMP2_6430_1

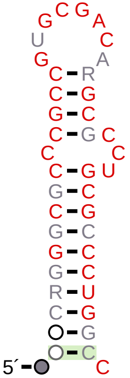

HMP2_6439_1

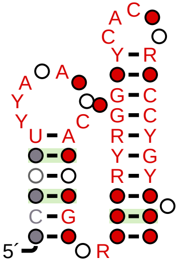

HMP2_6441_1

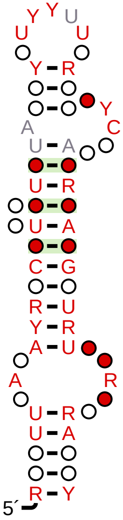

HMP2_6442_1

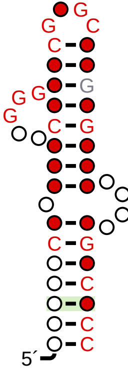

HMP2_6443_1

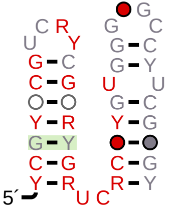

HMP2_6448_1

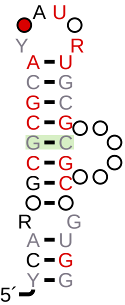

HMP2_6450_1

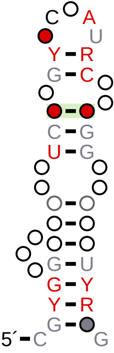

HMP2_6452_1

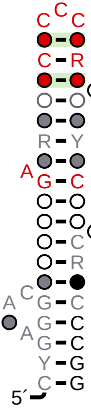

HMP2_6457_1

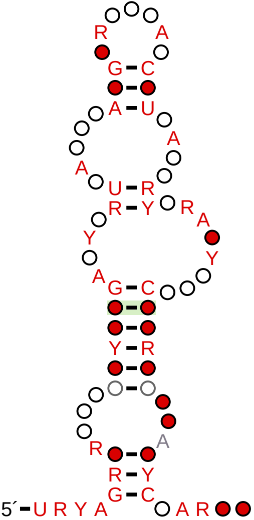

HMP2_6458_1

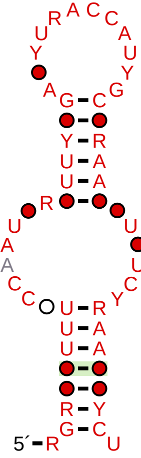

HMP2_6460_1

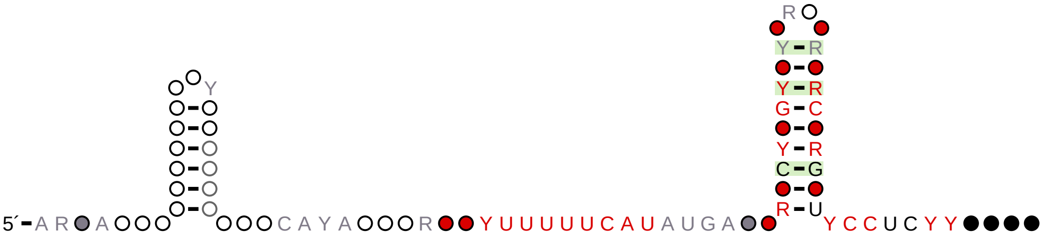

HMP2_6472_1

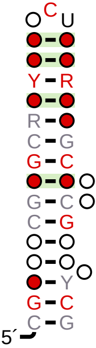

HMP2_6477_1

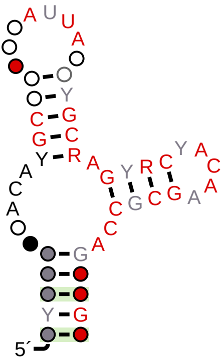

HMP2_6479_1

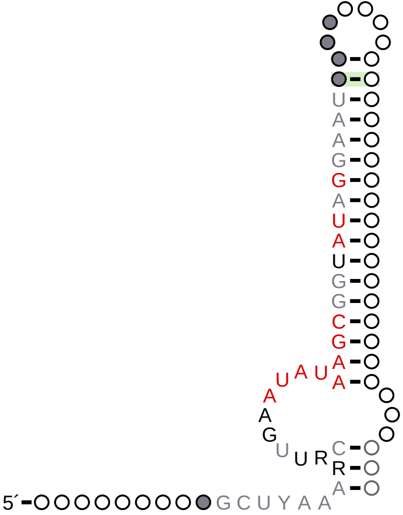

HMP2_6482_1

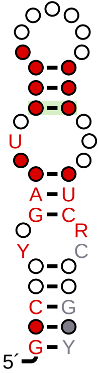

HMP2_6495_1

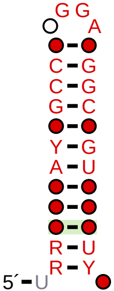

HMP2_6502_1

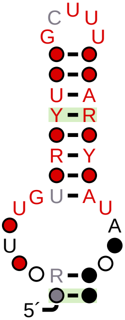

HMP2_6507_1

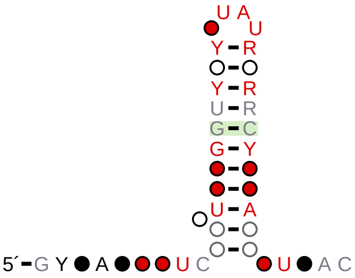

HMP2_6522_1

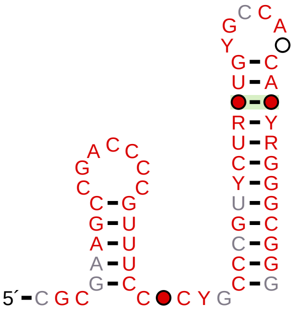

HMP2_6524_1

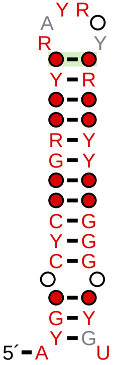

HMP2_6534_1

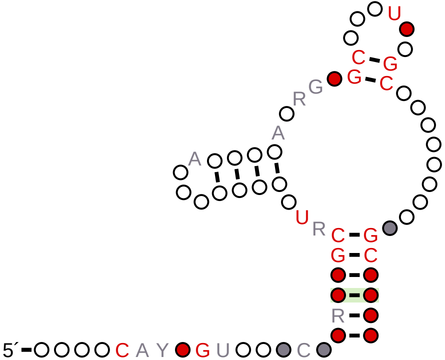

HMP2_654_1

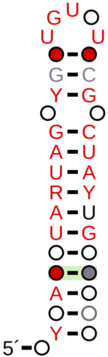

HMP2_6552_1

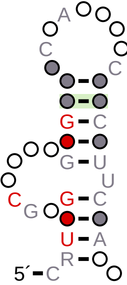

HMP2_6557_1

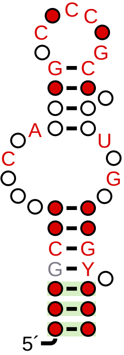

HMP2_6559_1

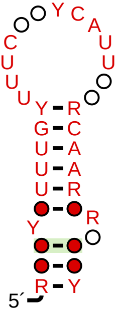

HMP2_6561_1

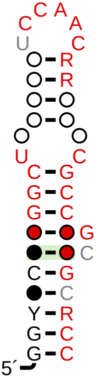

HMP2_6567_1

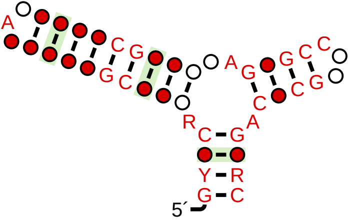

HMP2_6570_1

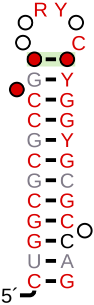

HMP2_6583_1

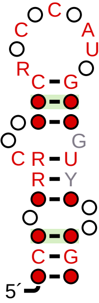

HMP2_6585_1

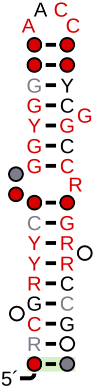

HMP2_6601_1

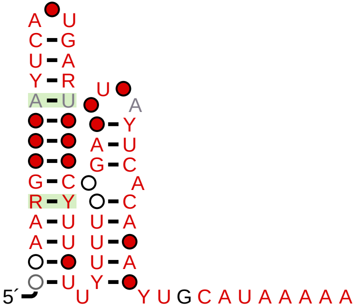

HMP2_6621_1

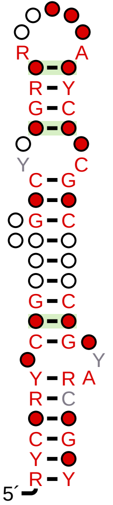

HMP2_6628_1

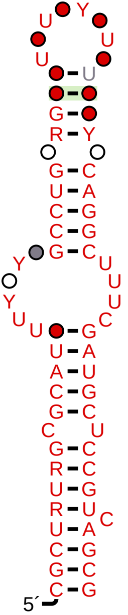

HMP2_6631_1

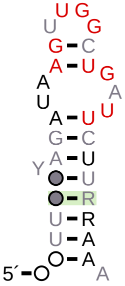

HMP2_6633_1

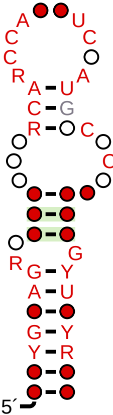

HMP2_6646_1

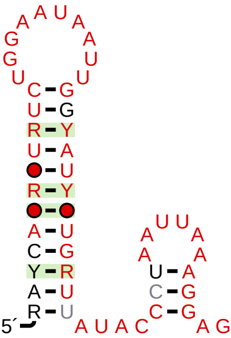

HMP2_666_1

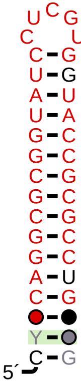

HMP2_6661_1

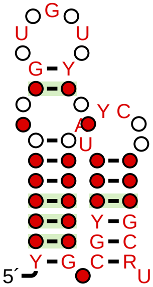

HMP2_6676_1

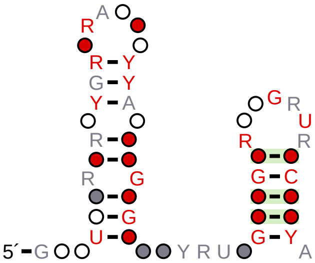

HMP2_668_1

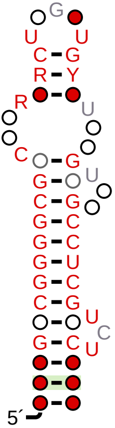

HMP2_6681_1

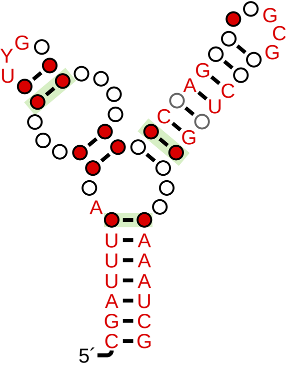

HMP2_6683_1

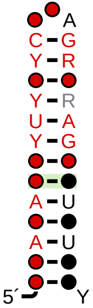

HMP2_6686_1

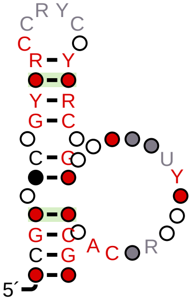

HMP2_6697_1

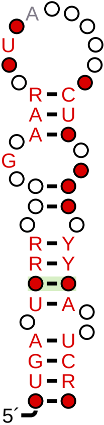

HMP2_67_1

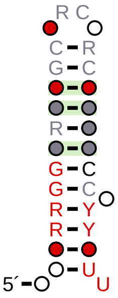

HMP2_6705_1

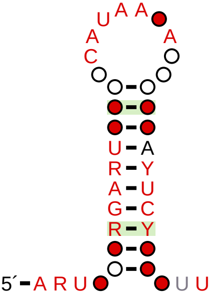

HMP2_6706_1

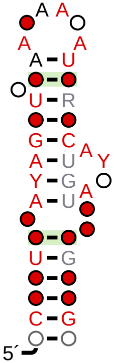

HMP2_6723_1

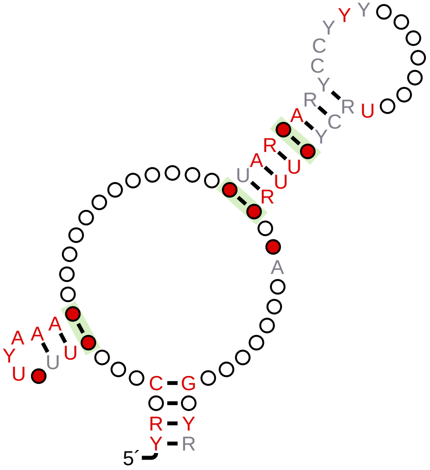

HMP2_6725_1

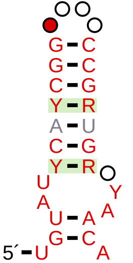

HMP2_6731_1

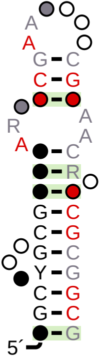

HMP2_6732_1

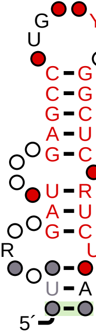

HMP2_6738_1

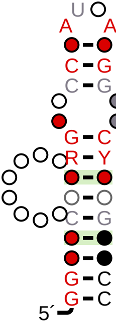

HMP2_674_1

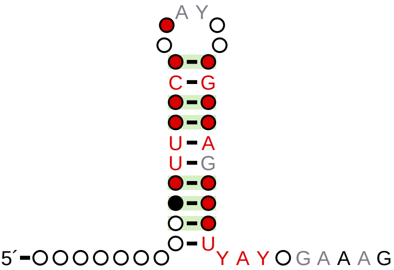

HMP2_6743_1

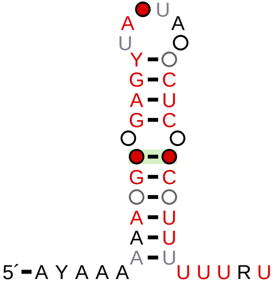

HMP2_675_1

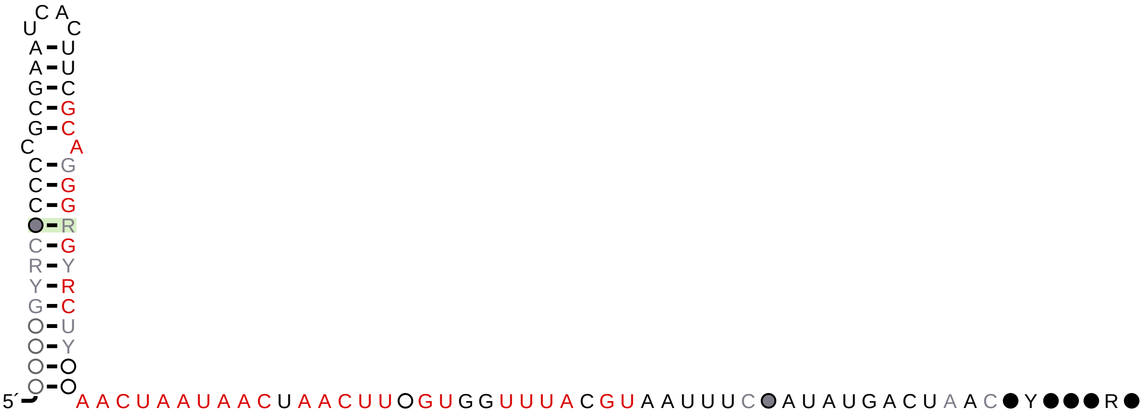

HMP2_6752_1

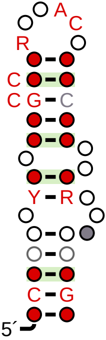

HMP2_6754_1

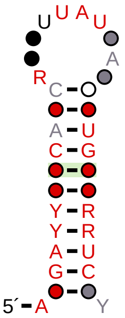

HMP2_6761_1

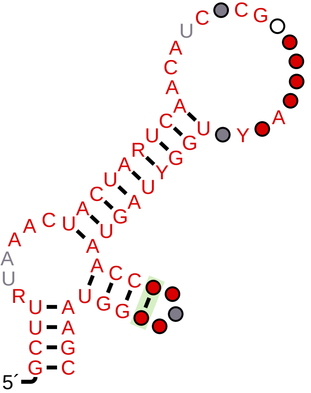

HMP2_6762_1

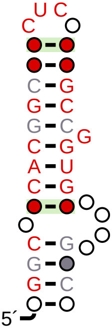

HMP2_6782_1

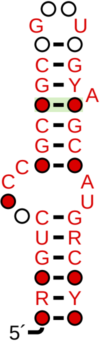

HMP2_6785_1

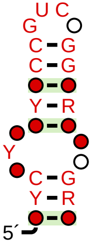

HMP2_6787_1

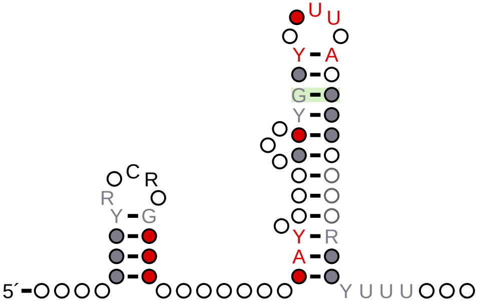

HMP2_6801_1

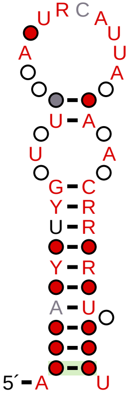

HMP2_6806_1

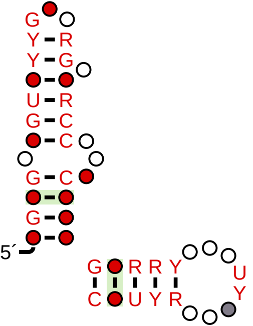

HMP2_6807_1

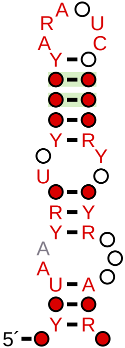

HMP2_6813_1

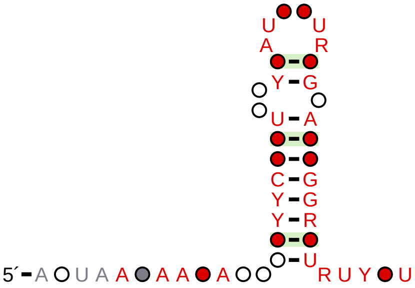

HMP2_6838_1

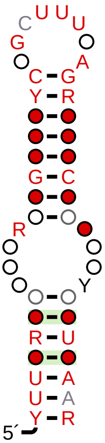

HMP2_684_1

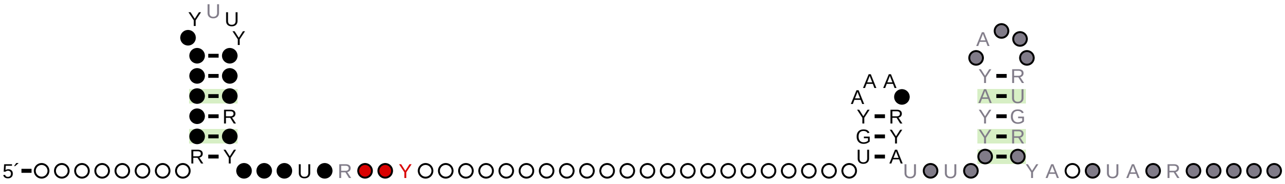

HMP2_6840_1

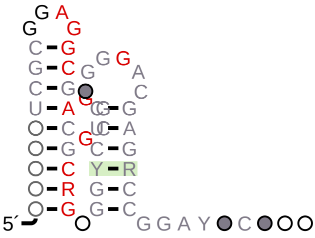

HMP2_6849_1

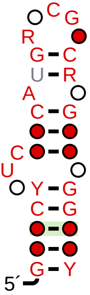

HMP2_6854_1

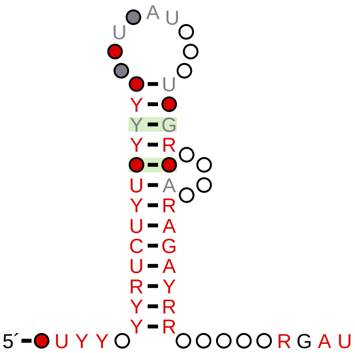

HMP2_6869_1

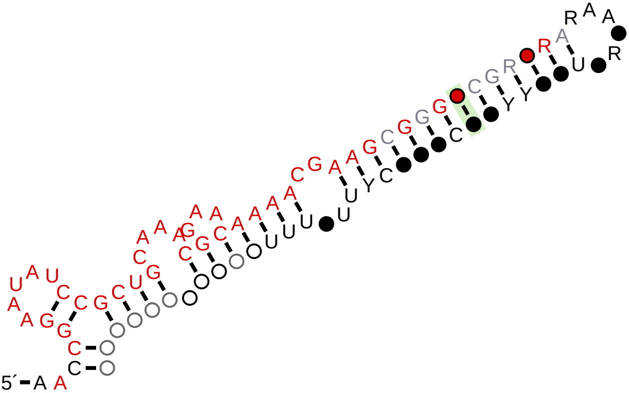

HMP2_6872_1

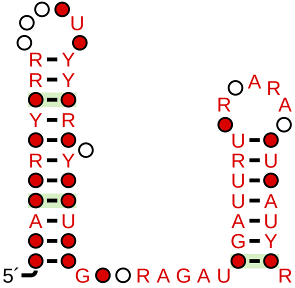

HMP2_6878_1

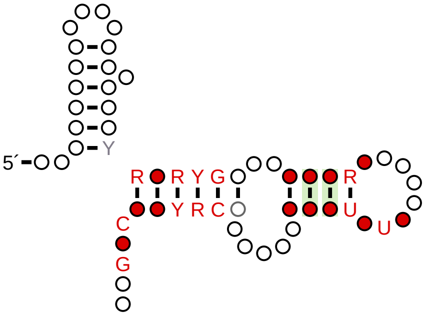

HMP2_688_1

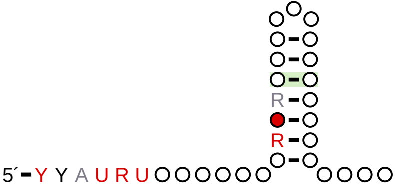

HMP2_6880_1

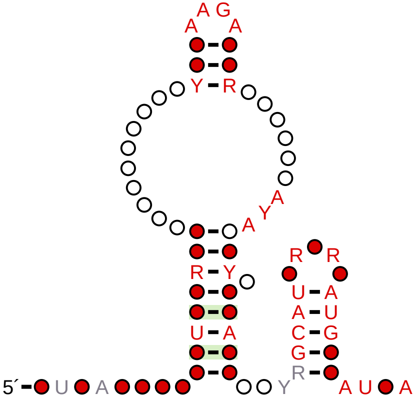

HMP2_6883_1

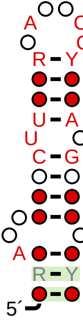

HMP2_6884_1

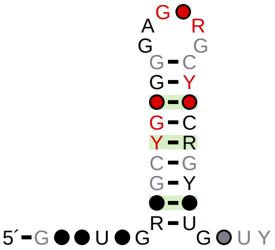

HMP2_6888_1

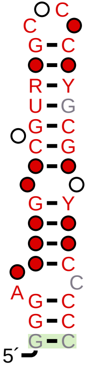

HMP2_689_1

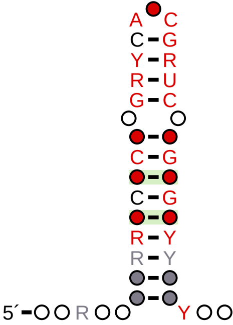

HMP2_6894_1

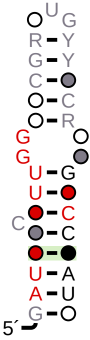

HMP2_6897_1

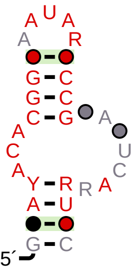

HMP2_6907_1

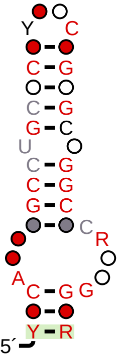

HMP2_691_1

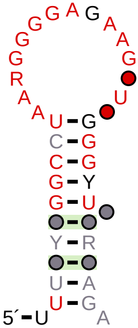

HMP2_6931_1

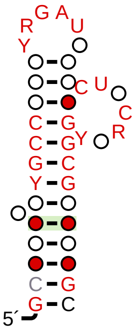

HMP2_6946_1

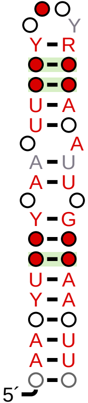

HMP2_6950_1

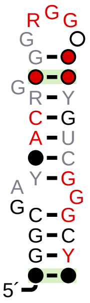

HMP2_6958_1

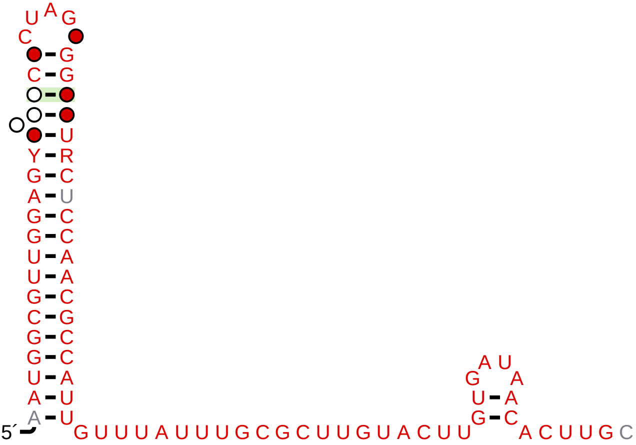

HMP2_6962_1

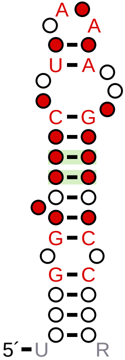

HMP2_6963_1

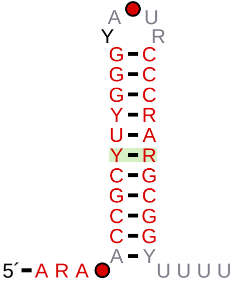

HMP2_6971_1

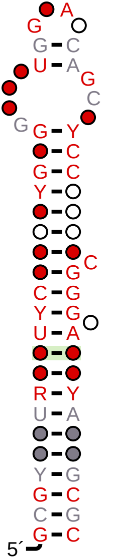

HMP2_6975_1

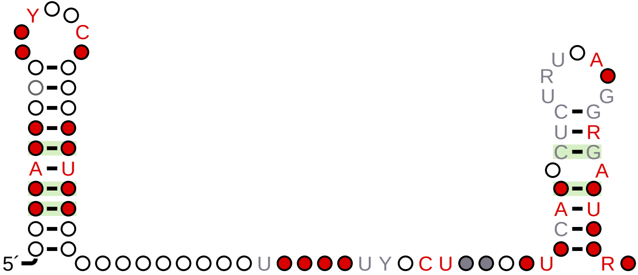

HMP2_6978_1

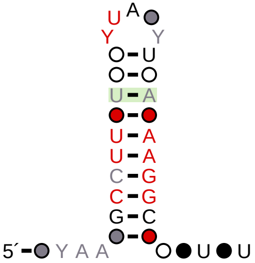

HMP2_6989_1

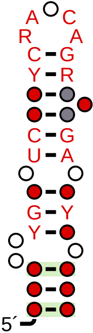

HMP2_7006_1

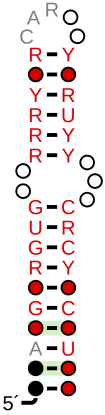

HMP2_7011_1

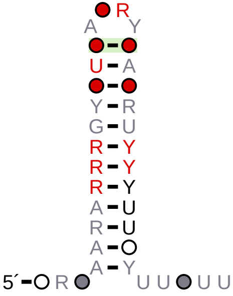

HMP2_7023_1

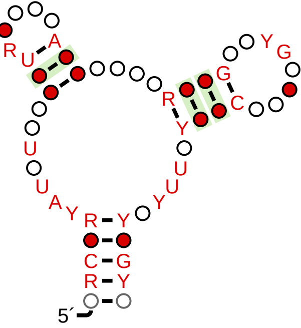

HMP2_7027_1

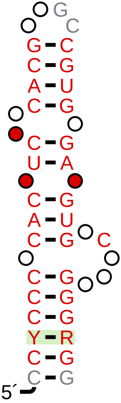

HMP2_7029_1

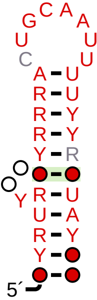

HMP2_7031_1

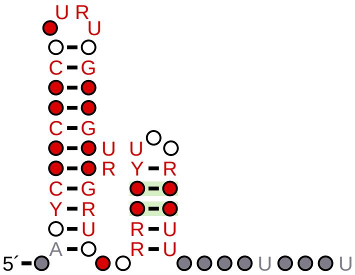

HMP2_7035_1

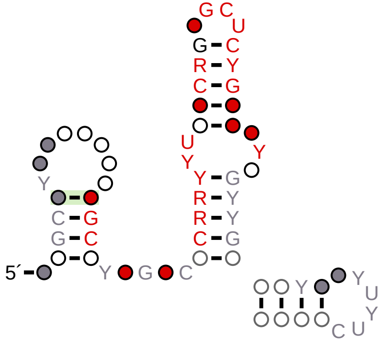

HMP2_7047_1

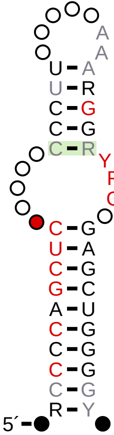

HMP2_7050_1

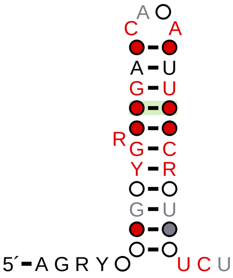

HMP2_7054_1

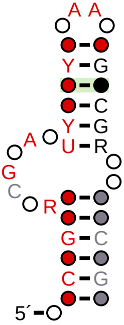

HMP2_706_1

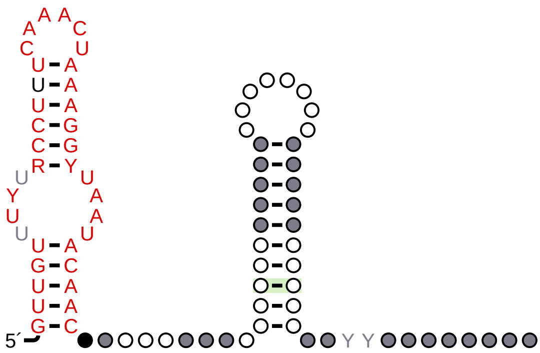

HMP2_7069_1

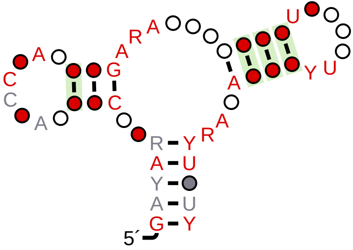

HMP2_7070_1

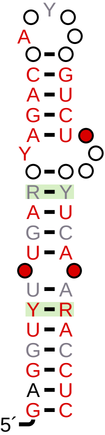

HMP2_7075_1

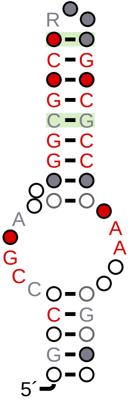

HMP2_7083_1

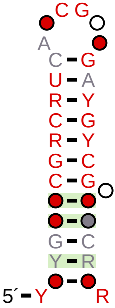

HMP2_7084_1

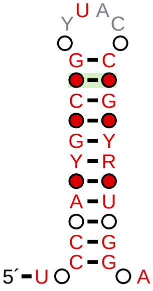

HMP2_7095_1

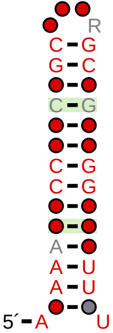

HMP2_7110_1

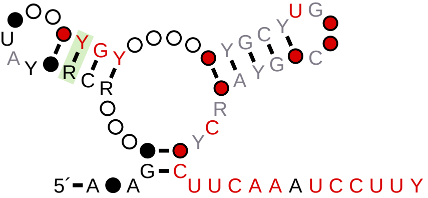

HMP2_7116_1

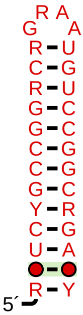

HMP2_7119_1

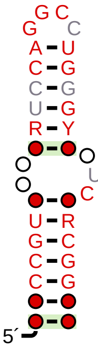

HMP2_7147_1

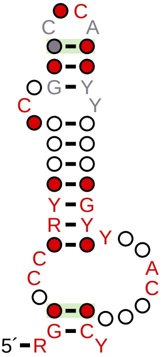

HMP2_715_1

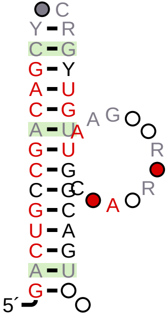

HMP2_7156_1

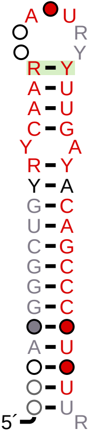

HMP2_7158_1

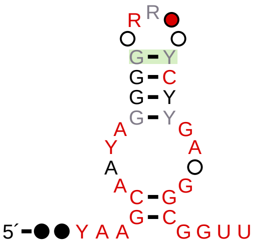

HMP2_7160_1

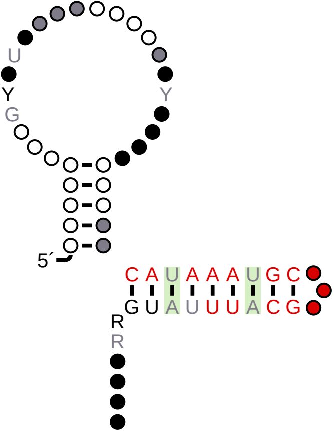

HMP2_7166_1

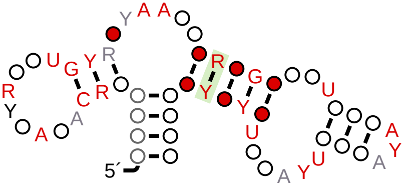

HMP2_7173_1

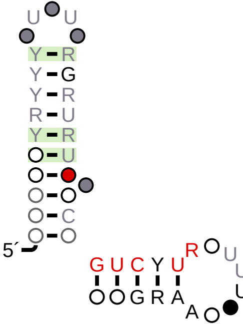

HMP2_7175_1

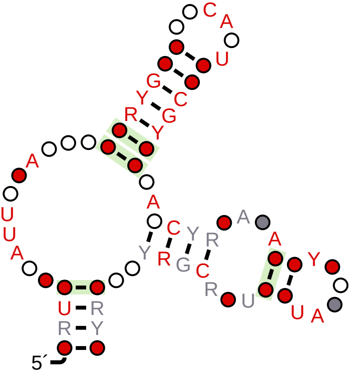

HMP2_7179_1

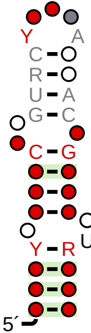

HMP2_7203_1

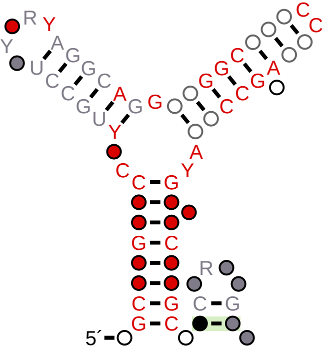

HMP2_7211_1

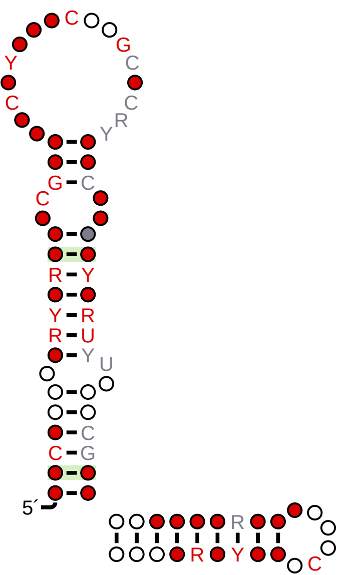

HMP2_7224_1

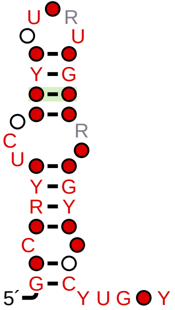

HMP2_7225_1

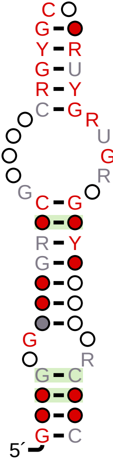

HMP2_7226_1

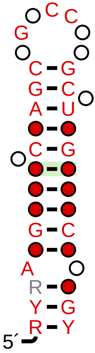

HMP2_724_1

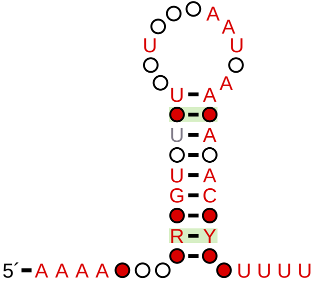

HMP2_7240_1

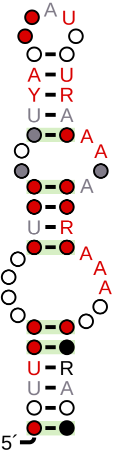

HMP2_7243_1

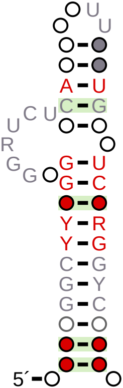

HMP2_7245_1

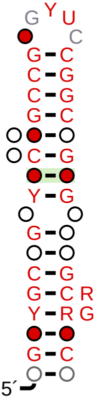

HMP2_7264_1

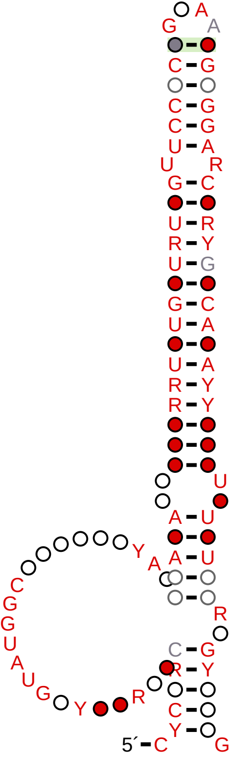

HMP2_7278_1

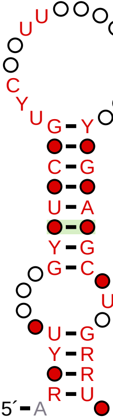

HMP2_7282_1

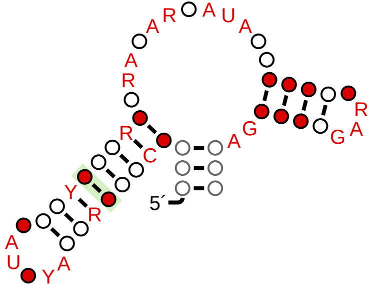

HMP2_7297_1

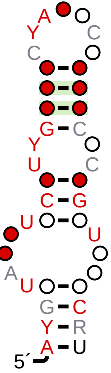

HMP2_7301_1

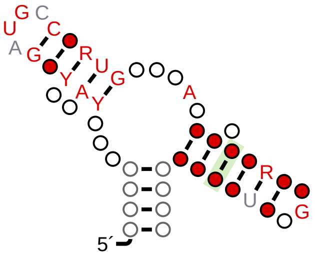

HMP2_7317_1

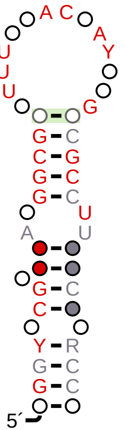

HMP2_7319_1

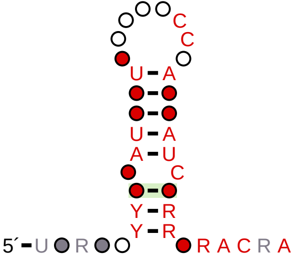

HMP2_7329_1

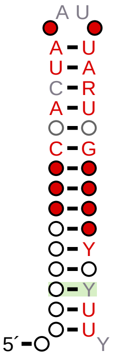

HMP2_7332_1

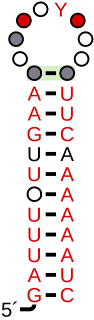

HMP2_7334_1

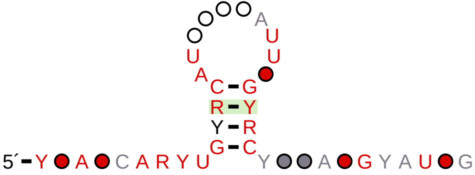

HMP2_7343_1

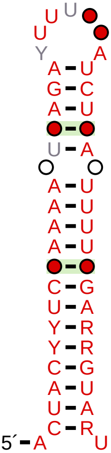

HMP2_7346_1

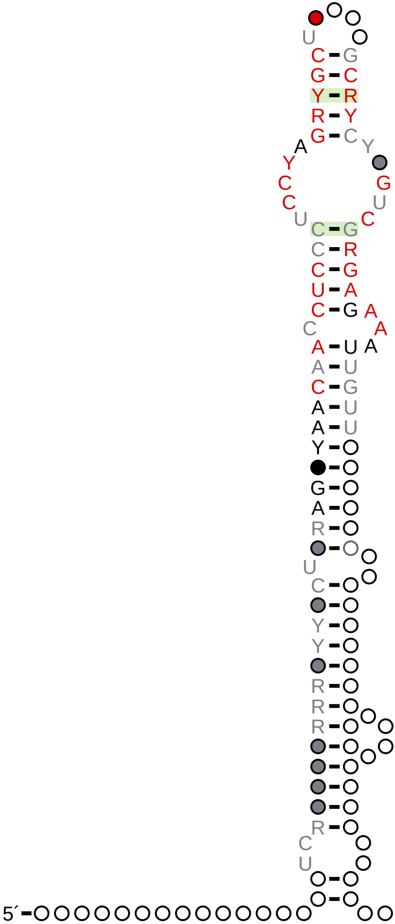

HMP2_735_1

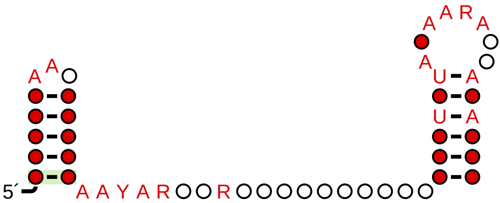

HMP2_7360_1

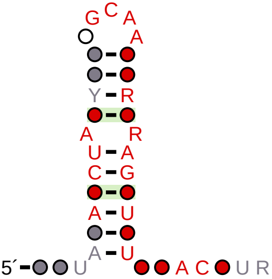

HMP2_7371_1

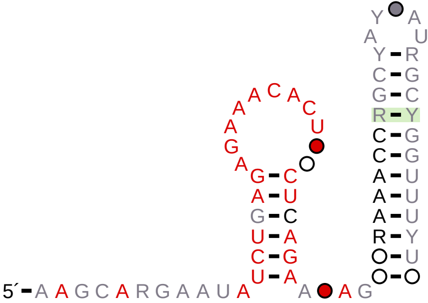

HMP2_7376_1

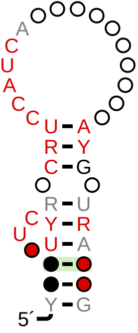

HMP2_7384_1

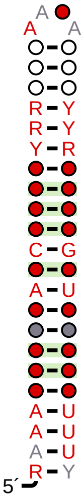

HMP2_7386_1

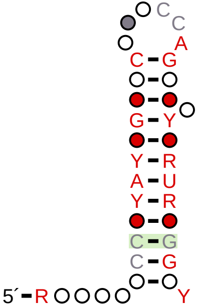

HMP2_7388_1

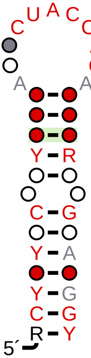

HMP2_7394_1

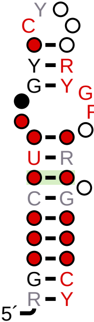

HMP2_741_1

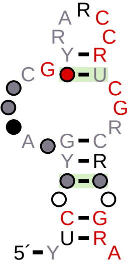

HMP2_7412_1

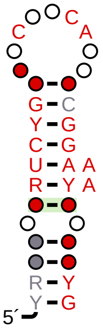

HMP2_7419_1

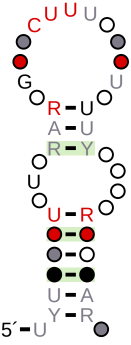

HMP2_7427_1

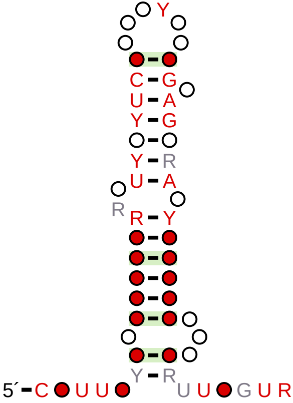

HMP2_7434_1

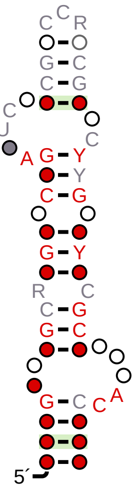

HMP2_7439_1

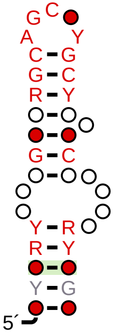

HMP2_7453_1

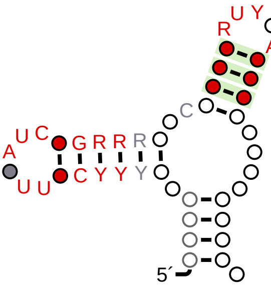

HMP2_7459_1

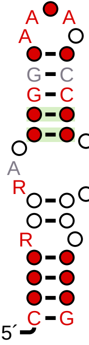

HMP2_7476_1

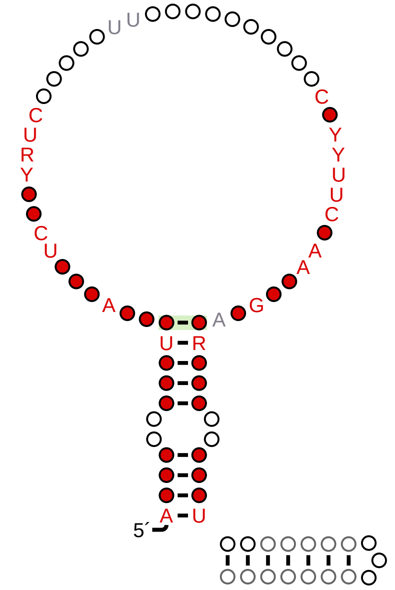

HMP2_7481_1

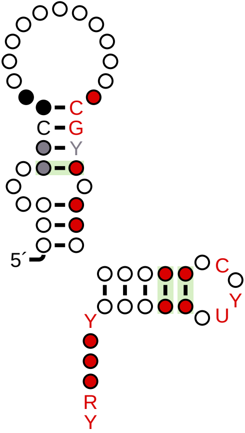

HMP2_7486_1

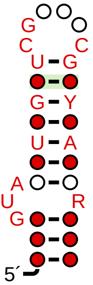

HMP2_7496_1

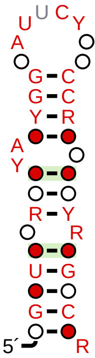

HMP2_7504_1

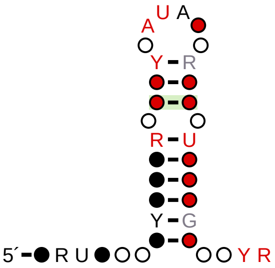

HMP2_7505_1

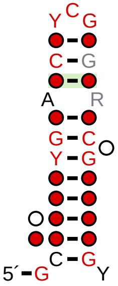

HMP2_7513_1

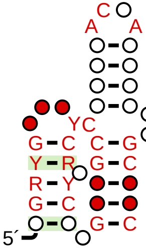

HMP2_752_1

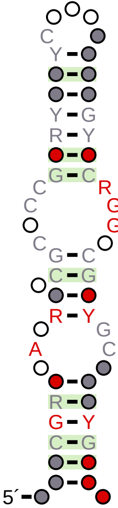

HMP2_7521_1

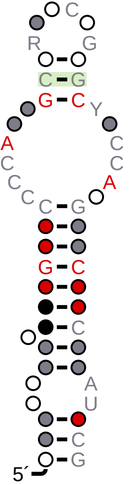

HMP2_7527_1

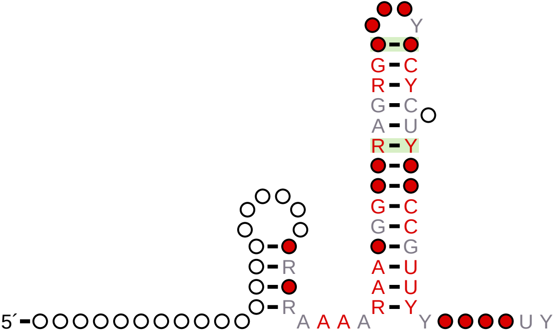

HMP2_7546_1

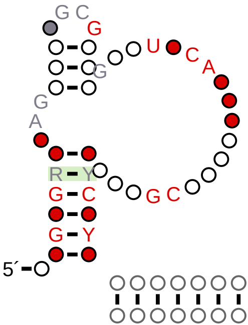

HMP2_7552_1

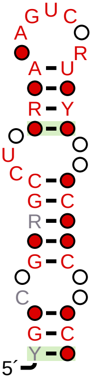

HMP2_7576_1

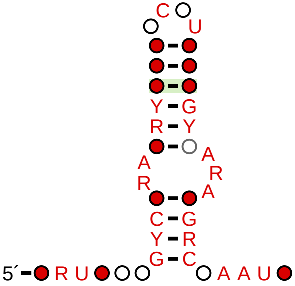

HMP2_7580_1

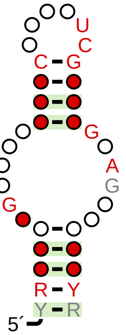

HMP2_7585_1

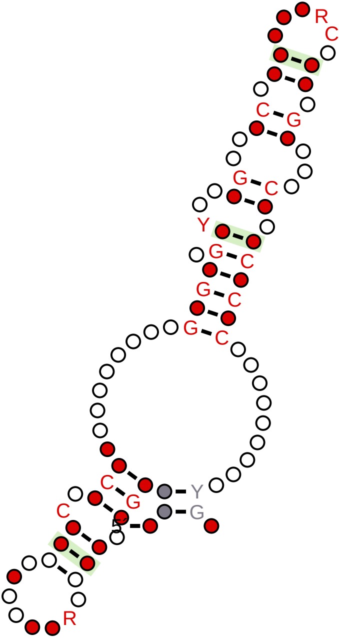

HMP2_7588_1

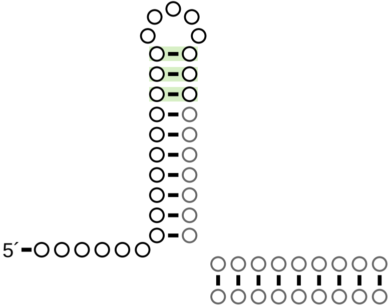

HMP2_7591_1

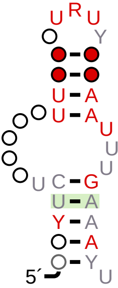

HMP2_7593_1

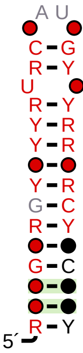

HMP2_7596_1

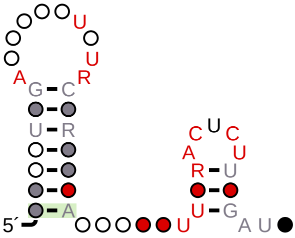

HMP2_7597_1

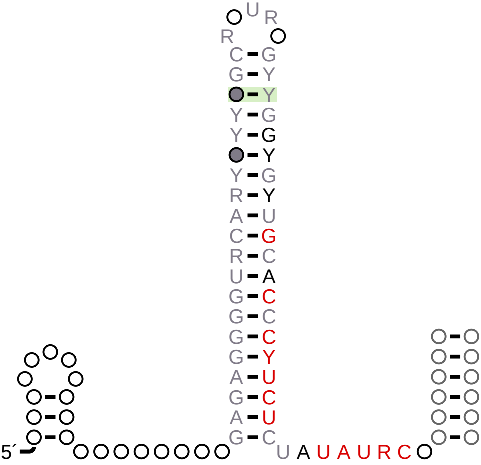

HMP2_7598_1

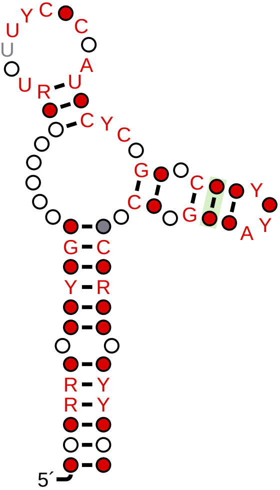

HMP2_7601_1

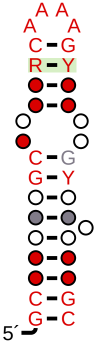

HMP2_7615_1

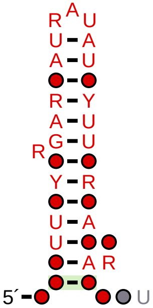

HMP2_762_1

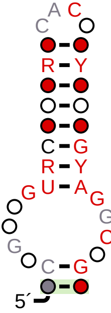

HMP2_7620_1

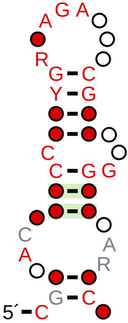

HMP2_7626_1

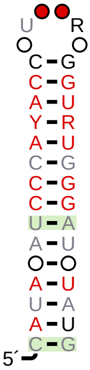

HMP2_763_1

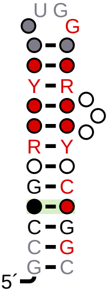

HMP2_7657_1

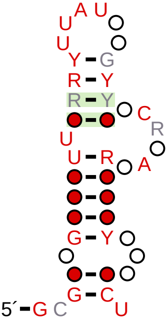

HMP2_7670_1

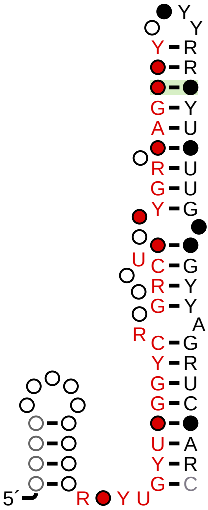

HMP2_7678_1

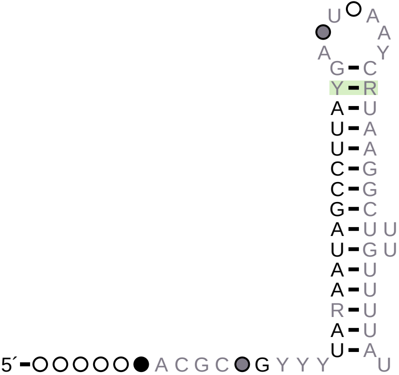

HMP2_7683_1

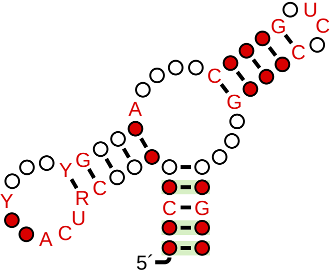

HMP2_7690_1

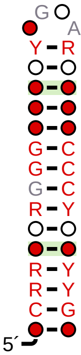

HMP2_7702_1

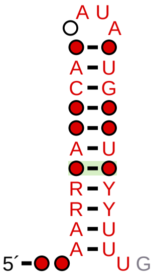

HMP2_7717_1

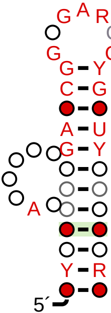

HMP2_7722_1

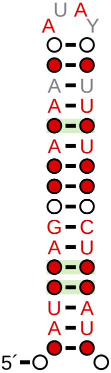

HMP2_7726_1

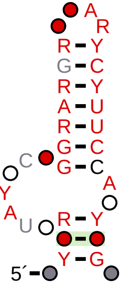

HMP2_7739_1

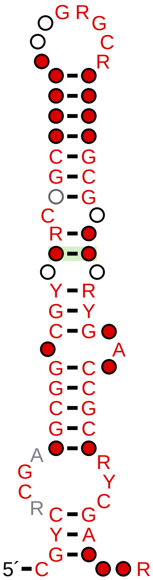

HMP2_7745_1

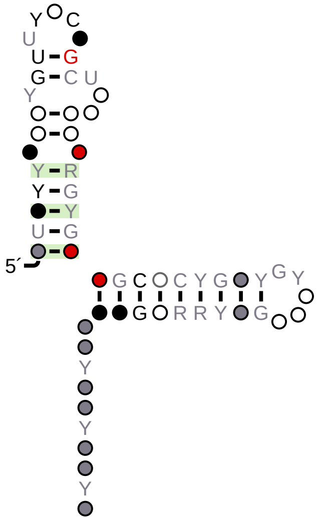

HMP2_7763_1

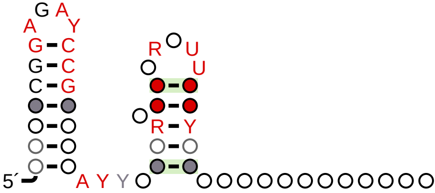

HMP2_7788_1

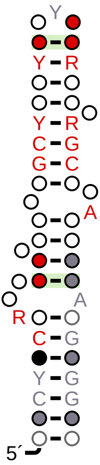

HMP2_7791_1

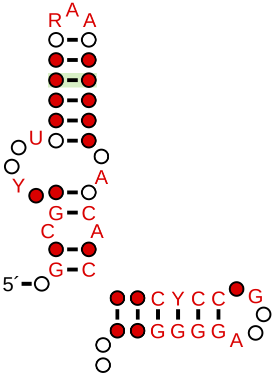

HMP2_7808_1

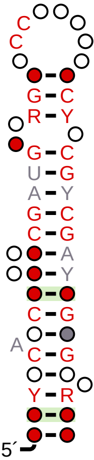

HMP2_7815_1

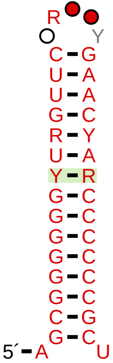

HMP2_7818_1

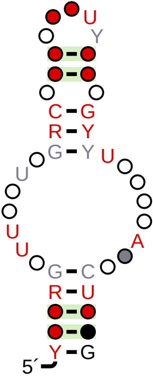

HMP2_782_1

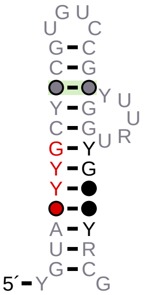

HMP2_7820_1

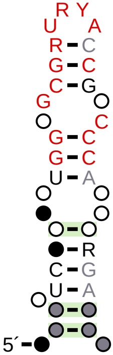

HMP2_7821_1

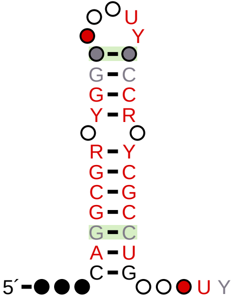

HMP2_783_1

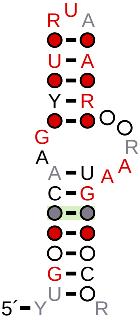

HMP2_7840_1

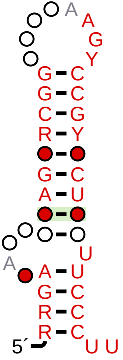

HMP2_7843_1

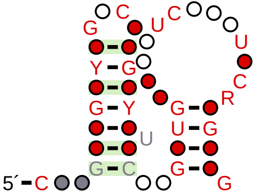

HMP2_7847_1

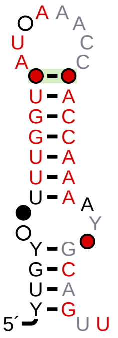

HMP2_7855_1

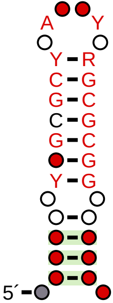

HMP2_7858_1

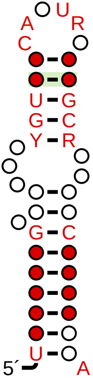

HMP2_7860_1

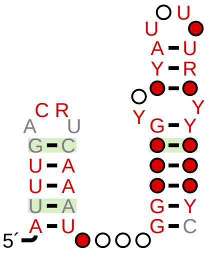

HMP2_7861_1

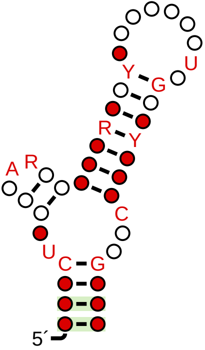

HMP2_7865_1

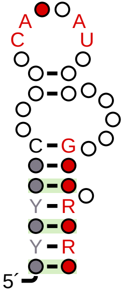

HMP2_7868_1

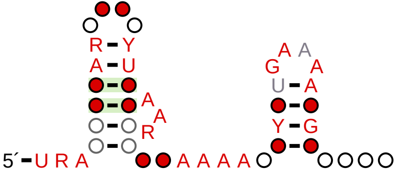

HMP2_7876_1

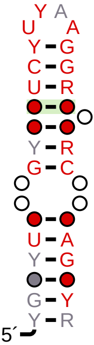

HMP2_7878_1

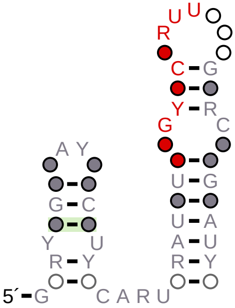

HMP2_7897_1

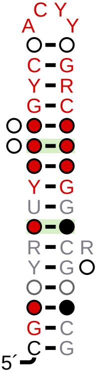

HMP2_7899_1

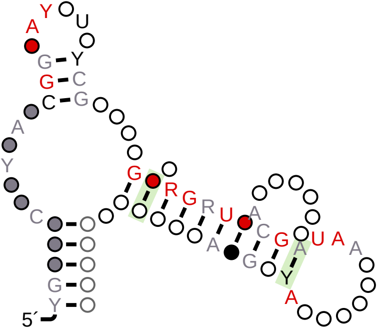

HMP2_7900_1

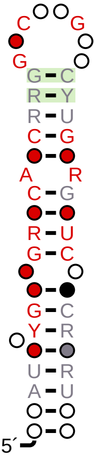

HMP2_7906_1

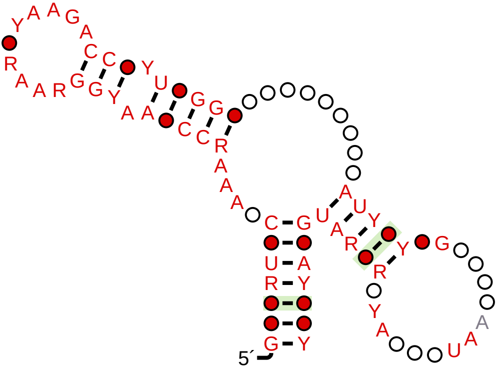

HMP2_7908_1

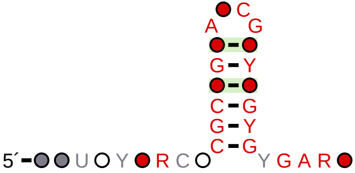

HMP2_7909_1

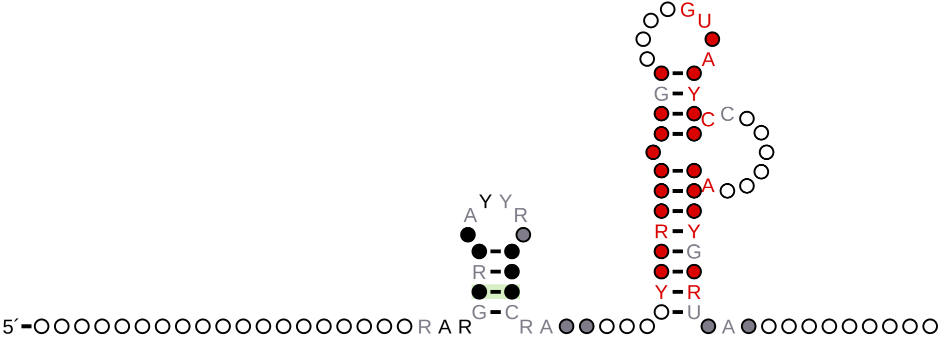

HMP2_7911_1

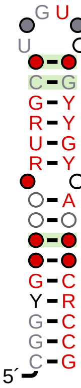

HMP2_7940_1

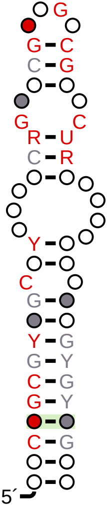

HMP2_7953_1

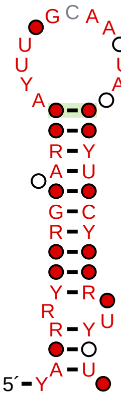

HMP2_7956_1

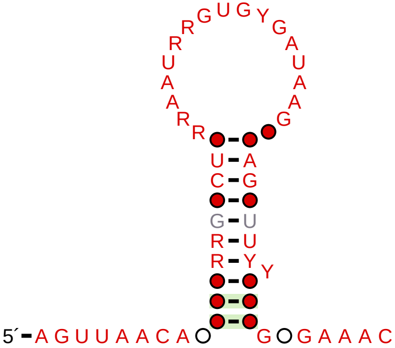

HMP2_7959_1

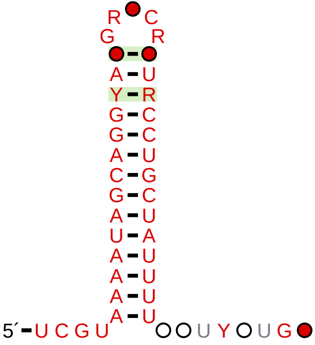

HMP2_7963_1

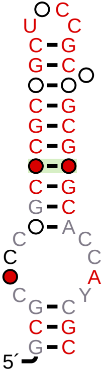

HMP2_7966_1

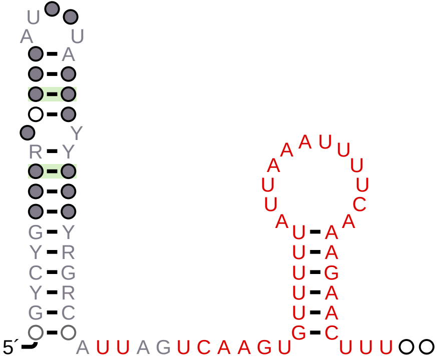

HMP2_7981_1

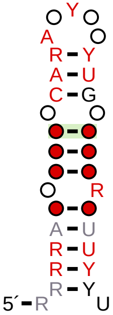

HMP2_7985_1

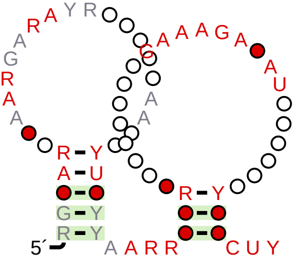

HMP2_7992_1

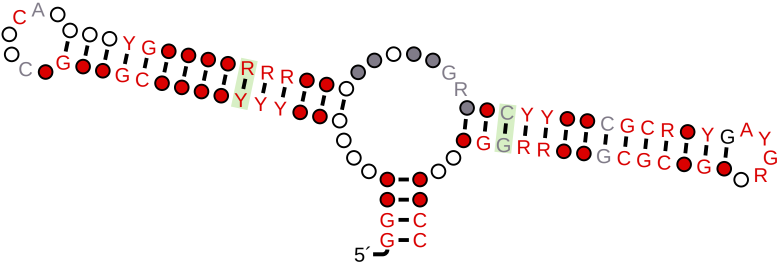

HMP2_7993_1

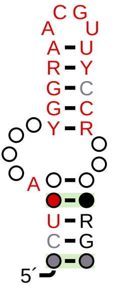

HMP2_800_1

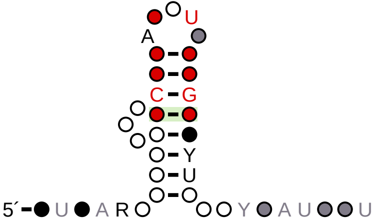

HMP2_8010_1

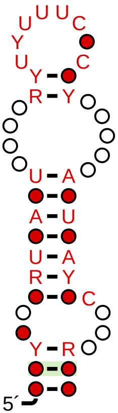

HMP2_8017_1

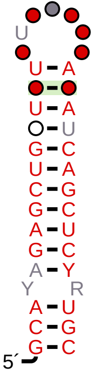

HMP2_8029_1

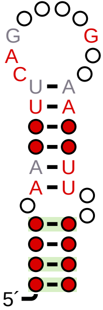

HMP2_8038_1

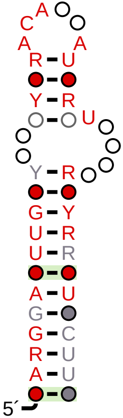

HMP2_8044_1

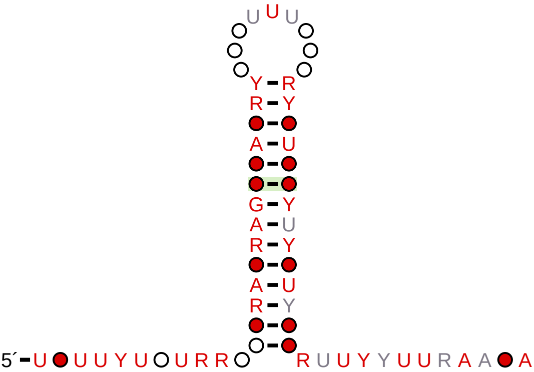

HMP2_805_1

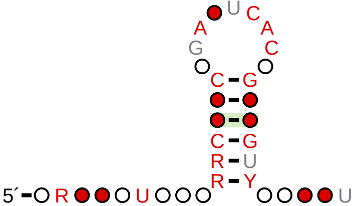

HMP2_8051_1

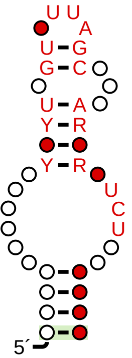

HMP2_8078_1

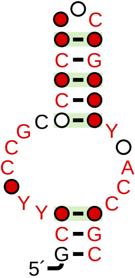

HMP2_8092_1

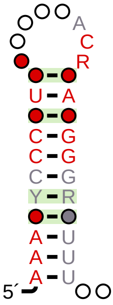

HMP2_8108_1

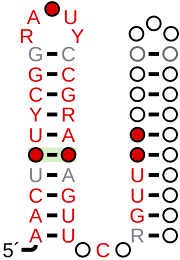

HMP2_8120_1

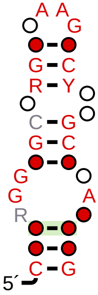

HMP2_8127_1

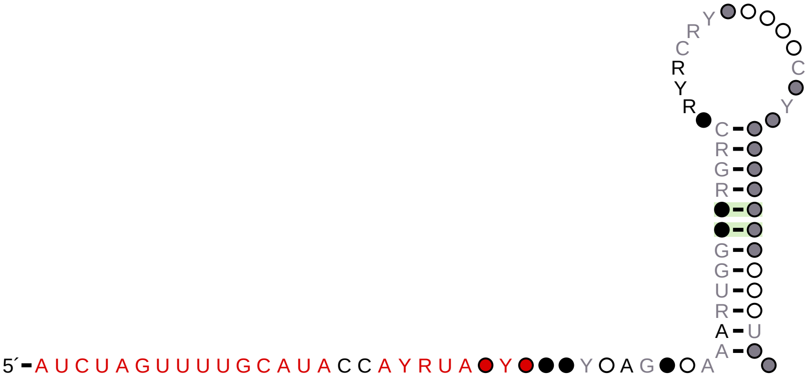

HMP2_813_1

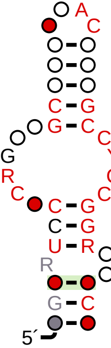

HMP2_8136_1

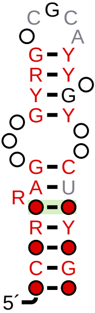

HMP2_8137_1

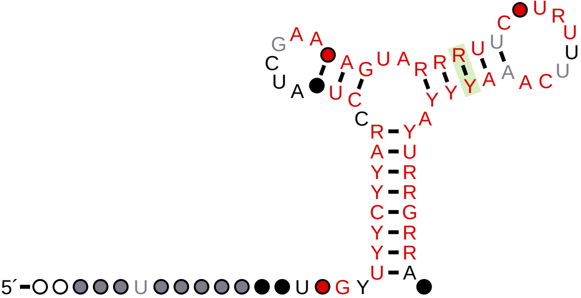

HMP2_8152_1

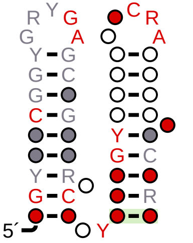

HMP2_8159_1

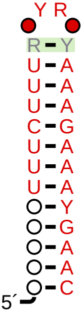

HMP2_8161_1

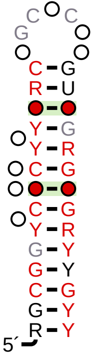

HMP2_8166_1

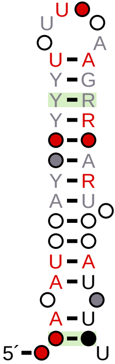

HMP2_8167_1

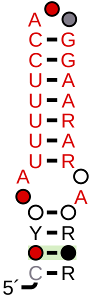

HMP2_817_1

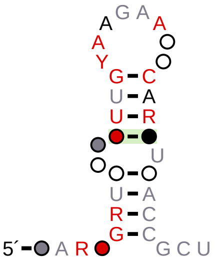

HMP2_8173_1

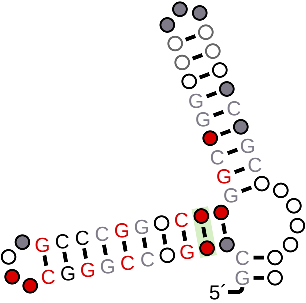

HMP2_8174_1

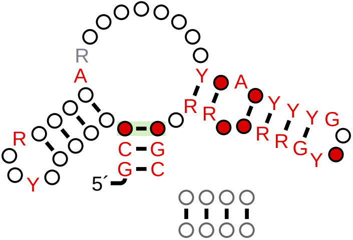

HMP2_8192_1

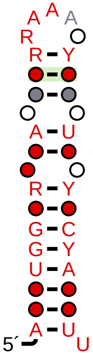

HMP2_8193_1

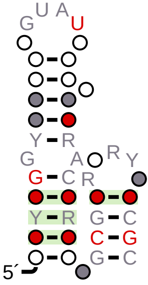

HMP2_8198_1

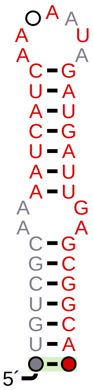

HMP2_8204_1

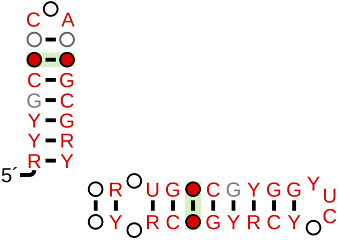

HMP2_8213_1

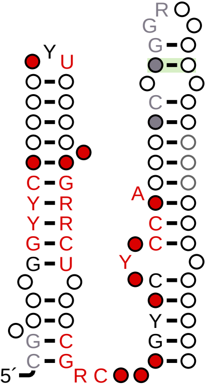

HMP2_8216_1

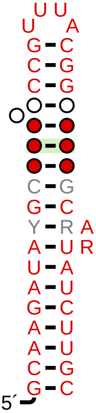

HMP2_8220_1

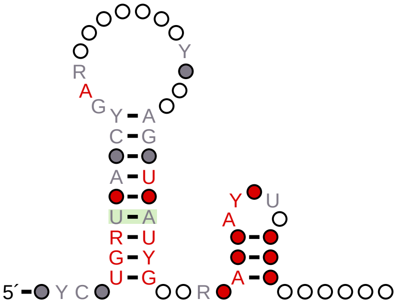

HMP2_8232_1

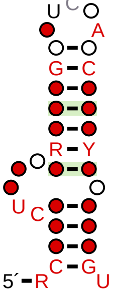

HMP2_8241_1

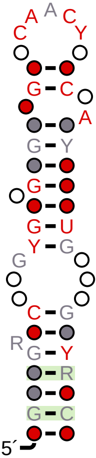

HMP2_8242_1

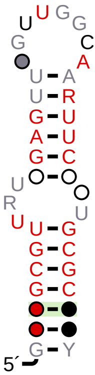

HMP2_8245_1

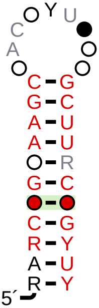

HMP2_8249_1

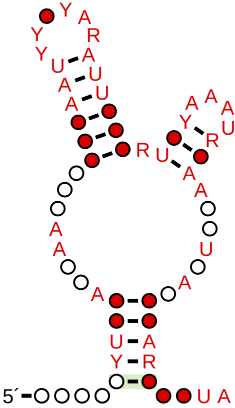

HMP2_8254_1

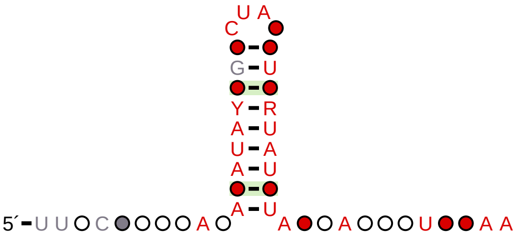

HMP2_8257_1

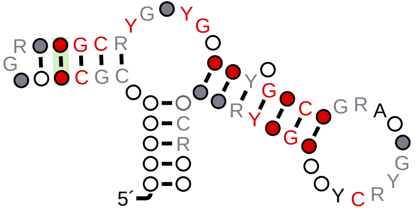

HMP2_8259_1

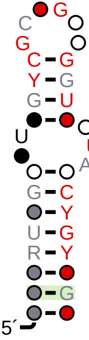

HMP2_8262_1

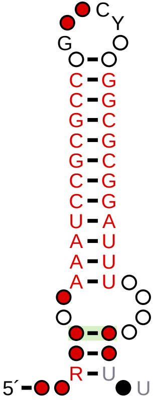

HMP2_8269_1

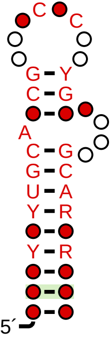

HMP2_8272_1

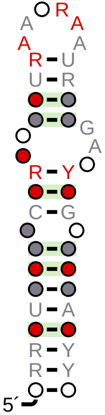

HMP2_8295_1

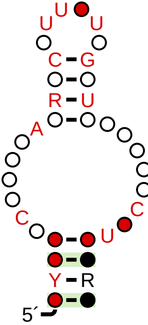

HMP2_8296_1

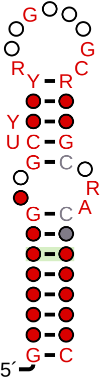

HMP2_830_1

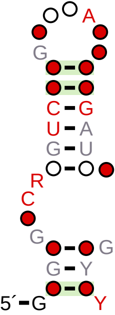

HMP2_8326_1

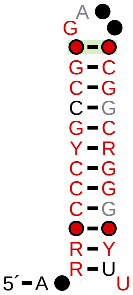

HMP2_8328_1

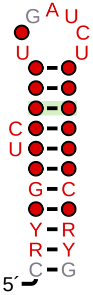

HMP2_8331_1

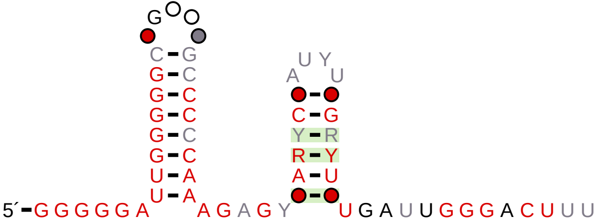

G
C G
G C
G C
G C
G C
G C
U A
U A
U Y
A U
C G
Y R
R Y
A U
5´ G G G G G A A G A G Y U G A U U G G G A C U U U

HMP2_8345_1

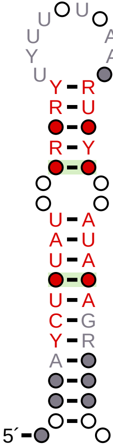

HMP2_8351_1

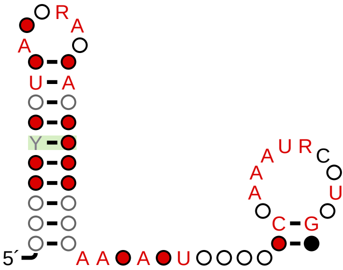

HMP2_8352_1

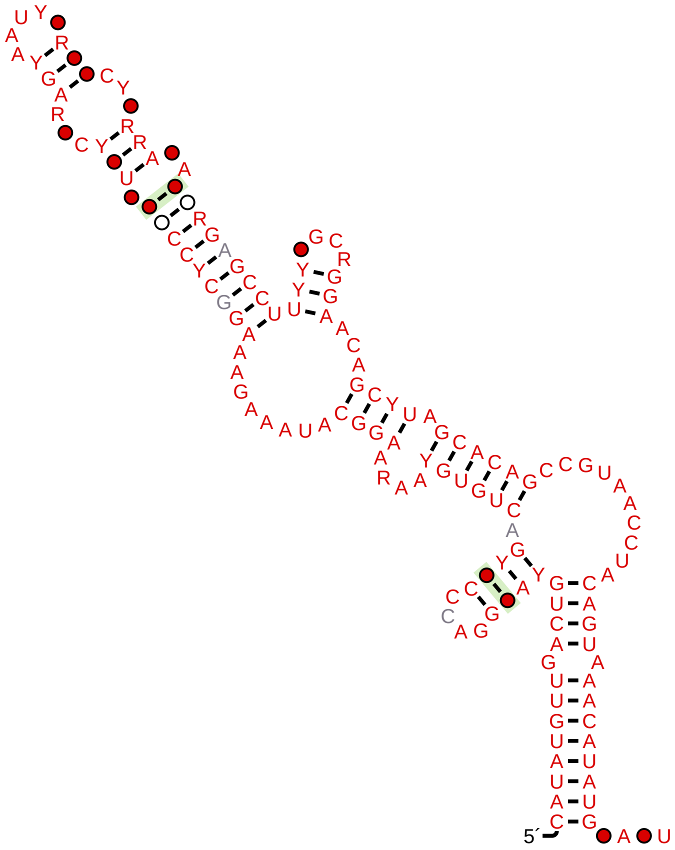

HMP2_8362_1

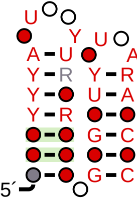

HMP2_8365_1

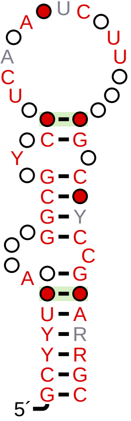

HMP2_8366_1

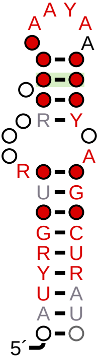

HMP2_8375_1

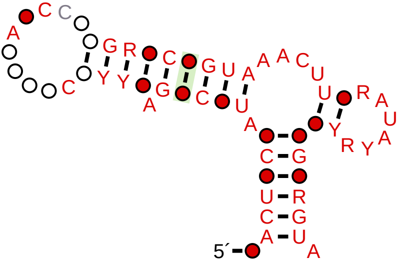

HMP2_8386_1

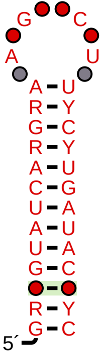

HMP2_8390_1

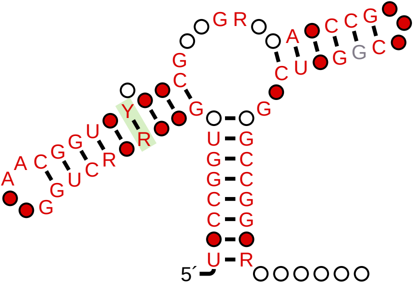

HMP2_8397_1

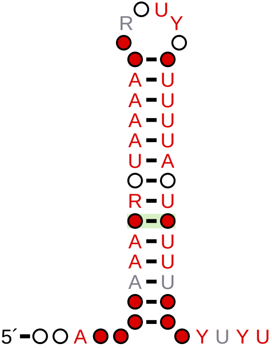

HMP2_84_1

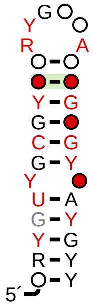

HMP2_8401_1

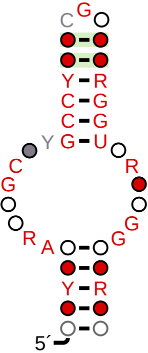

HMP2_8403_1

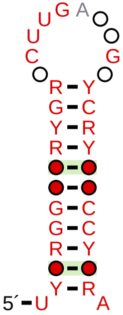

HMP2_8405_1

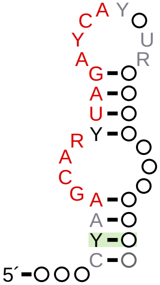

HMP2_8409_1

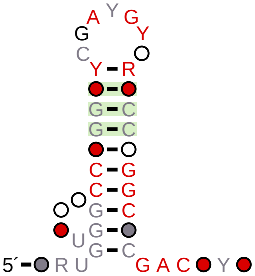

HMP2_8412_1

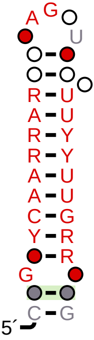

HMP2_8417_1

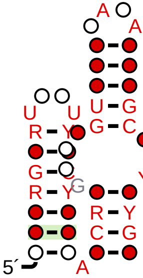

HMP2_8435_1

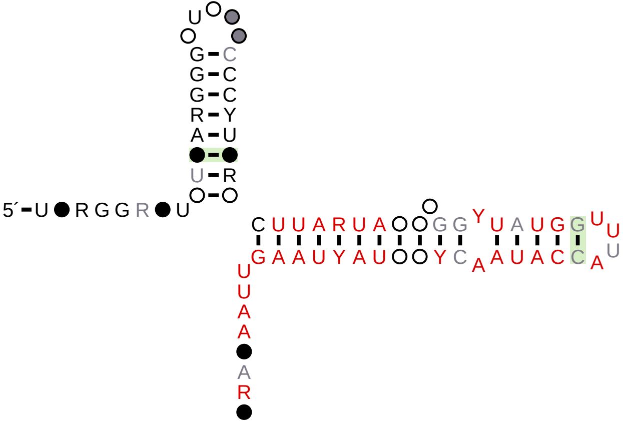

5´
U R G G R U
U
G C
G C
G C
R Y
A U
U R
C U U A R U A G G Y U A U G G U U
G A A U Y A U Y C A A U A C C A U
U
U
A
A
A
R

HMP2_8437_1

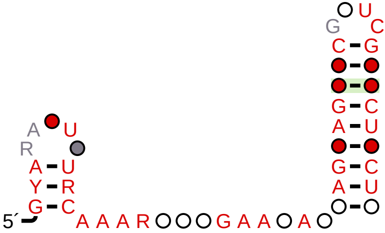

HMP2_844_1

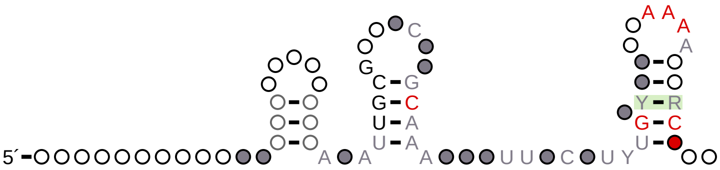

HMP2_8470_1

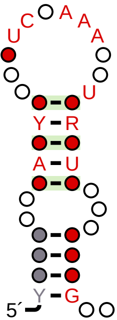

HMP2_8472_1

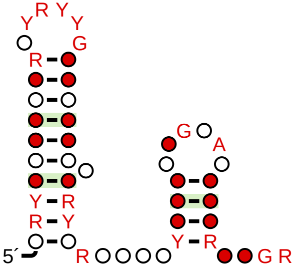

HMP2_8473_1

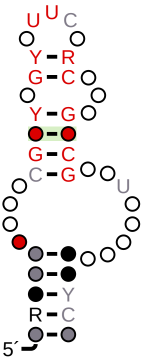

HMP2_8495_1

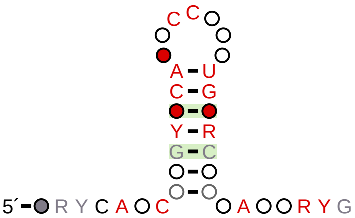

HMP2_8502_1

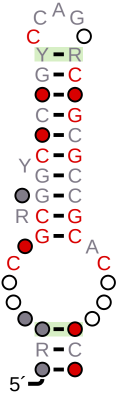

HMP2_8508_1

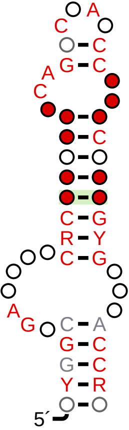

HMP2_8509_1

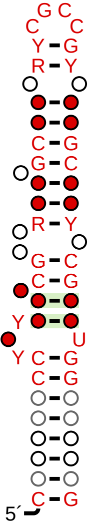

HMP2_8510_1

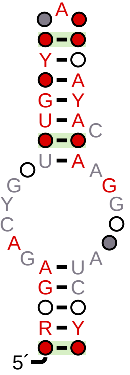

HMP2_8516_1

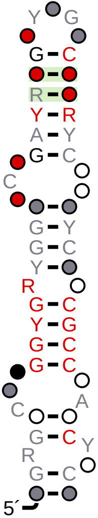

HMP2_8519_1

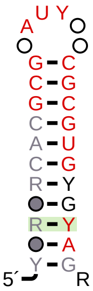

HMP2_852_1

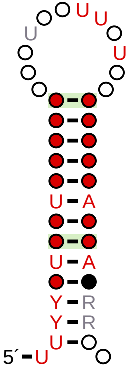

HMP2_8528_1

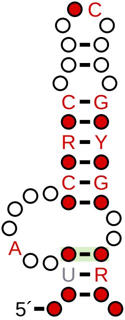

HMP2_8530_1

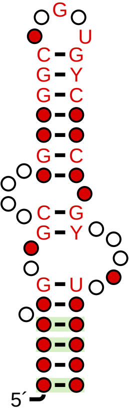

HMP2_8532_1

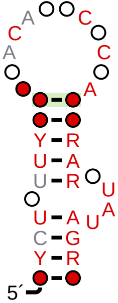

HMP2_8534_1

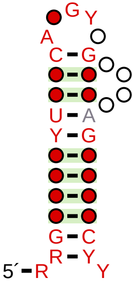

HMP2_8542_1

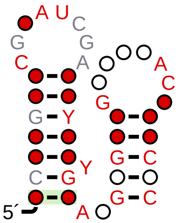

HMP2_8546_1

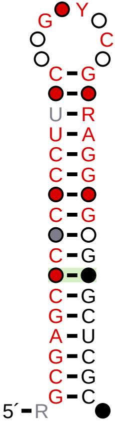

HMP2_8553_1

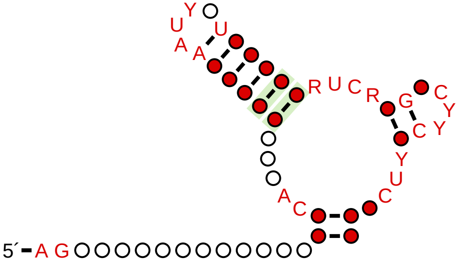

HMP2_8554_1

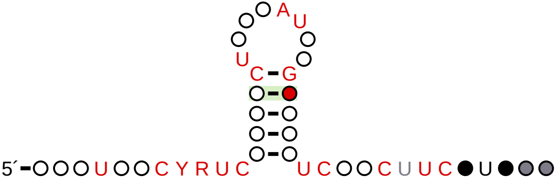

HMP2_8567_1

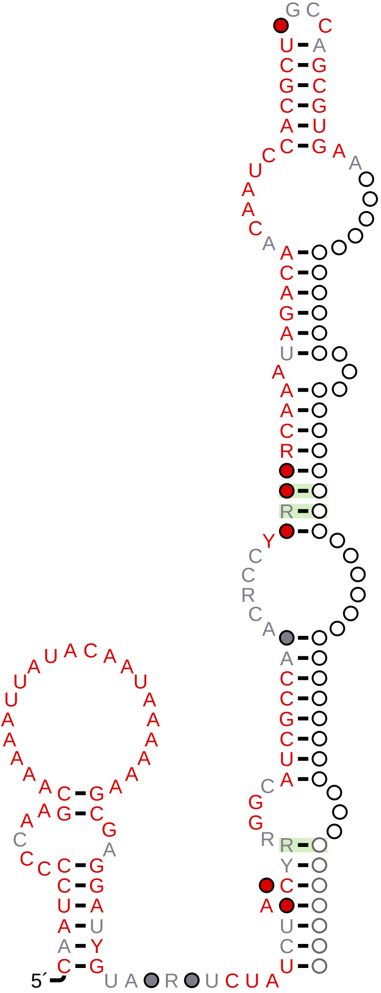

HMP2_8568_1

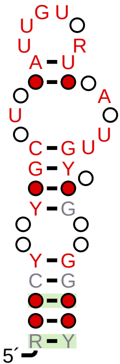

HMP2_8570_1

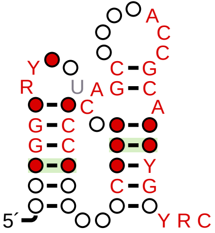

HMP2_8572_1

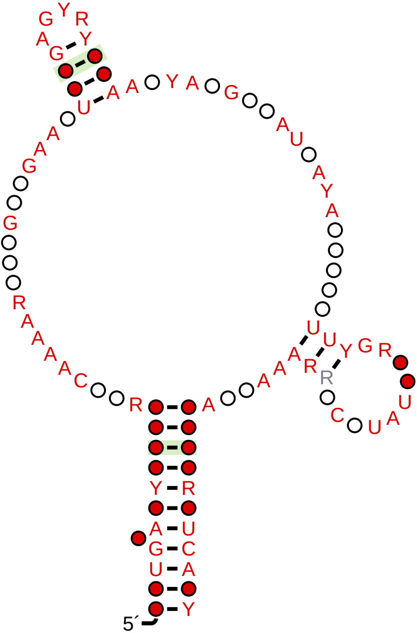

HMP2_8580_1

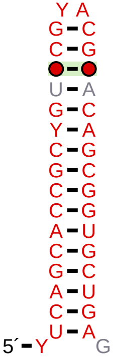

HMP2_8582_1

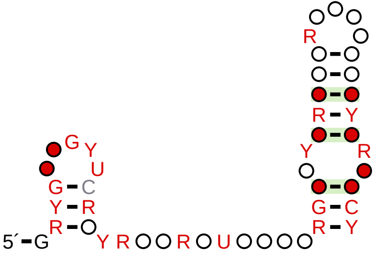

HMP2_8589_1

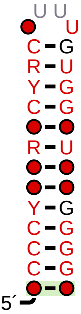

HMP2_8626_1

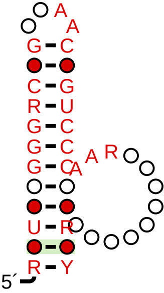

HMP2_8630_1

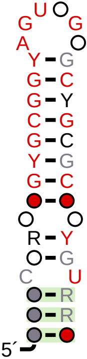

HMP2_8639_1

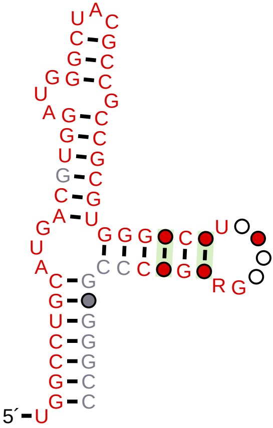

HMP2_864_1

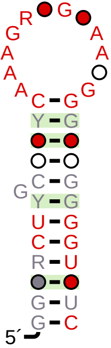

HMP2_8643_1

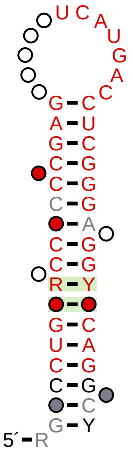

HMP2_8659_1

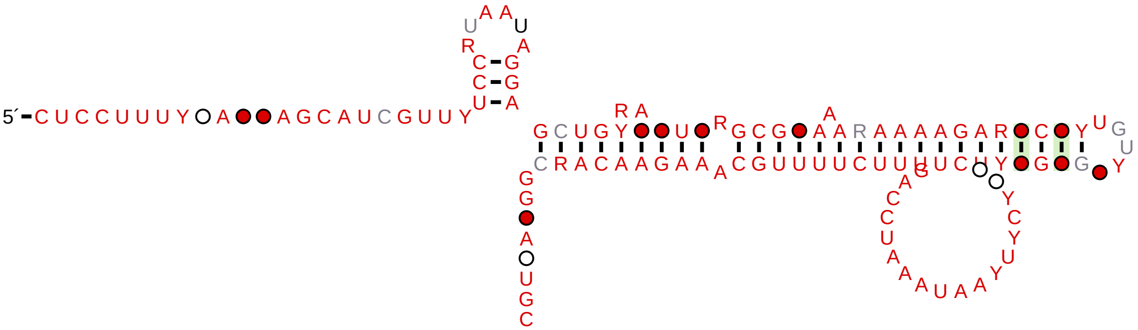

HMP2_8662_1

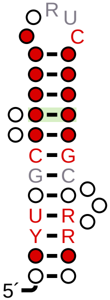

HMP2_8665_1

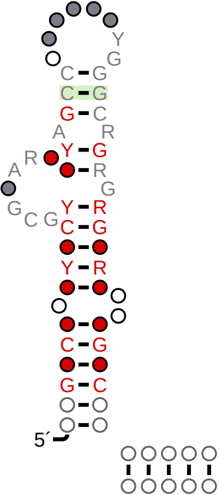

HMP2_8682_1

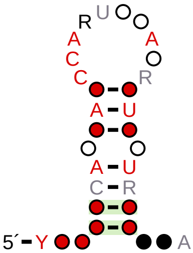

HMP2_8685_1

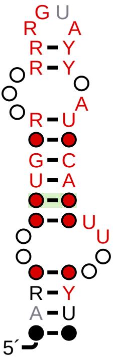

HMP2_8688_1

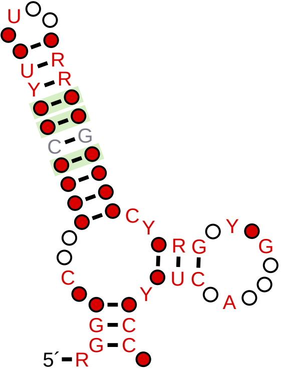

HMP2_8689_1

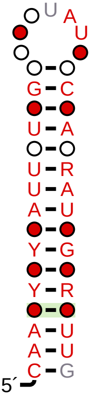

HMP2_8702_1

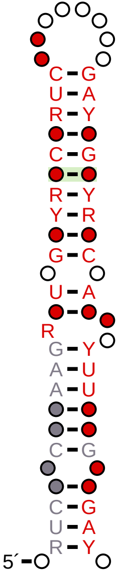

HMP2_8712_1

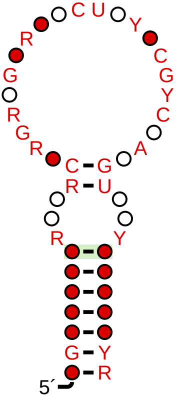

HMP2_8714_1

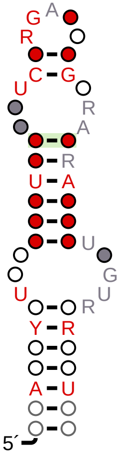

HMP2_8715_1

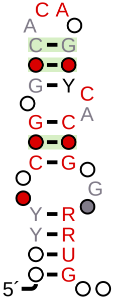

HMP2_8717_1

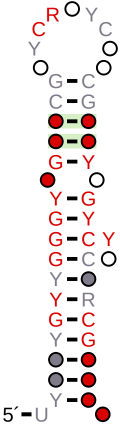

HMP2_8723_1

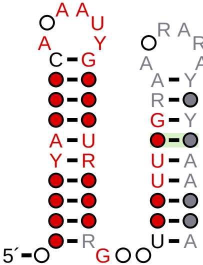

HMP2_8735_1

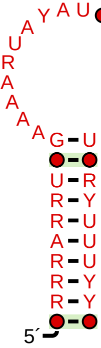

HMP2_8738_1

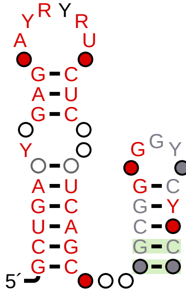

HMP2_874_1

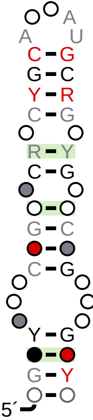

HMP2_8742_1

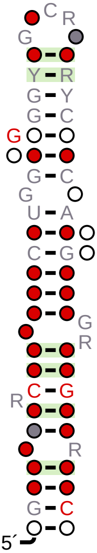

HMP2_8746_1

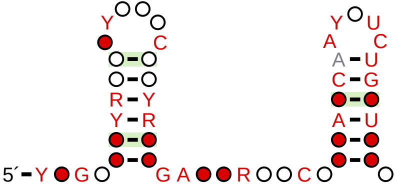

HMP2_8763_1

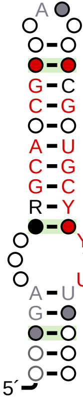

HMP2_8766_1

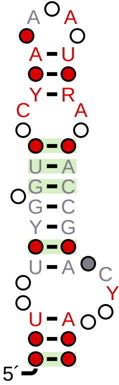

HMP2_8769_1

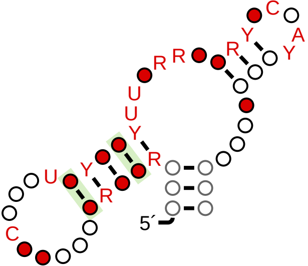

HMP2_8773_1

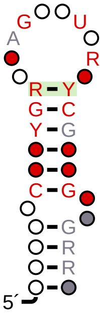

HMP2_8780_1

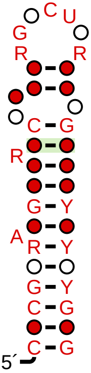

HMP2_8788_1

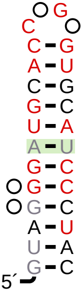

HMP2_8790_1

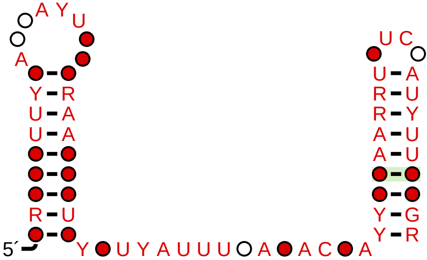

HMP2_8791_1

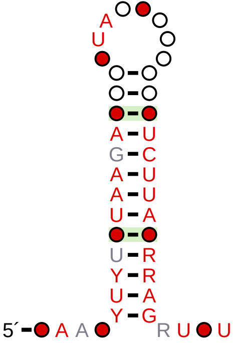

HMP2_8794_1

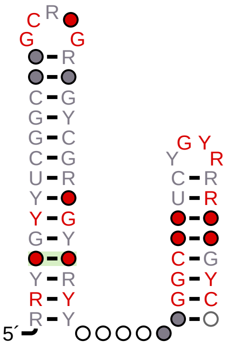

HMP2_8798_1

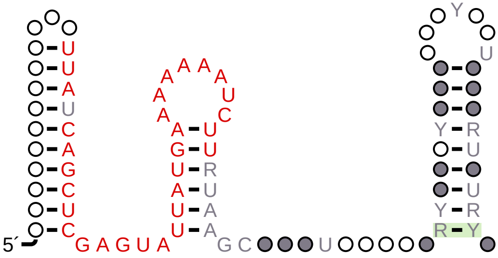

HMP2_8799_1

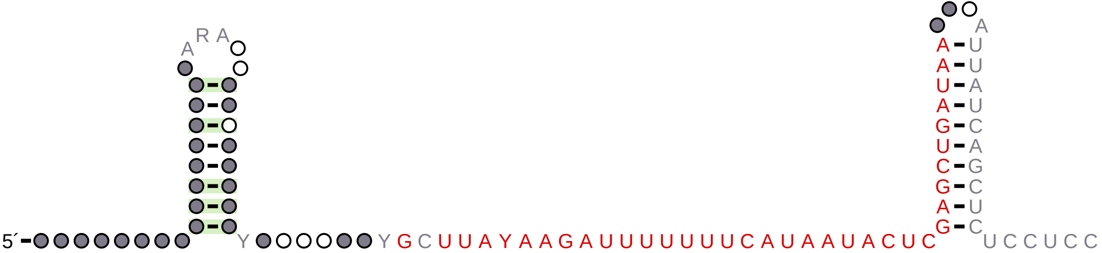

HMP2_8821_1

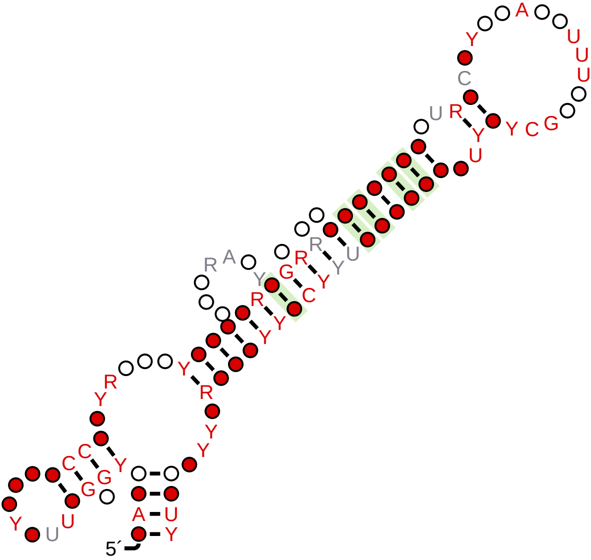

HMP2_8831_1

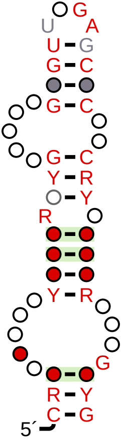

HMP2_8835_1

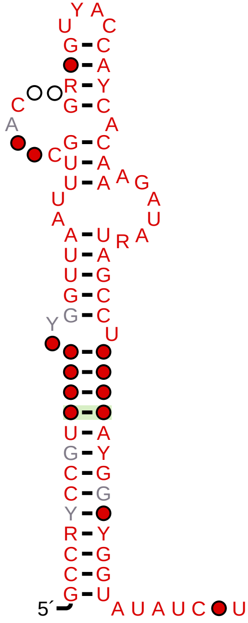

HMP2_8842_1

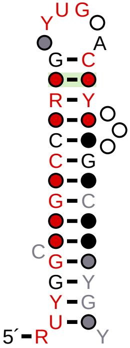

HMP2_8846_1

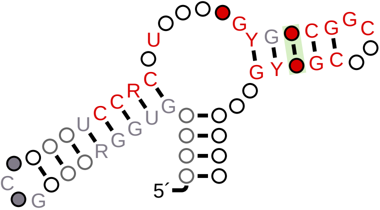

HMP2_8854_1

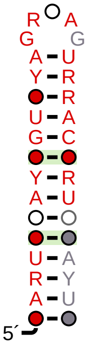

HMP2_886_1

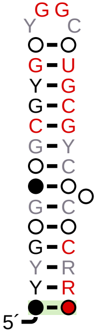

HMP2_8888_1

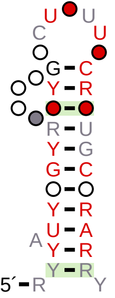

HMP2_8889_1

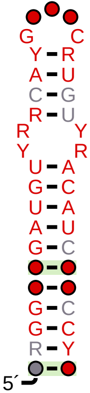

HMP2_8899_1

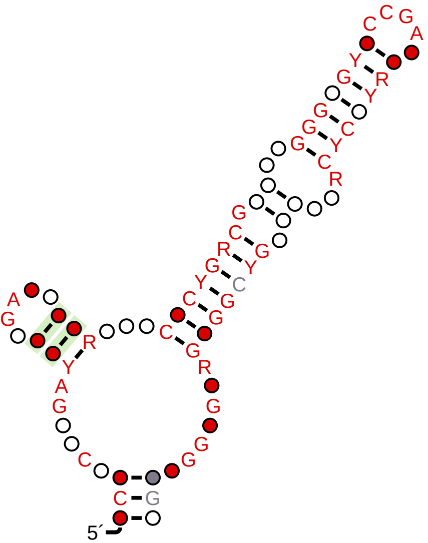

HMP2_8901_1

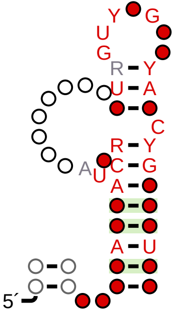

HMP2_8910_1

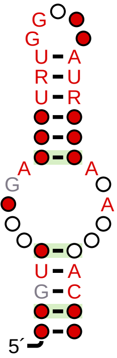

HMP2_8915_1

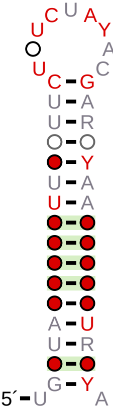

HMP2_8924_1

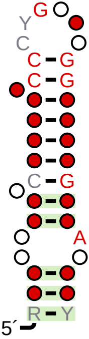

HMP2_894_1

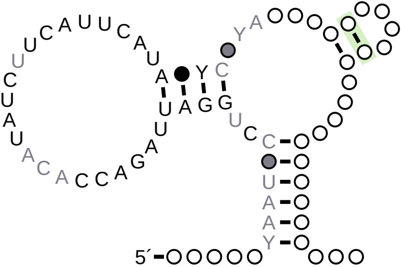

HMP2_8955_1

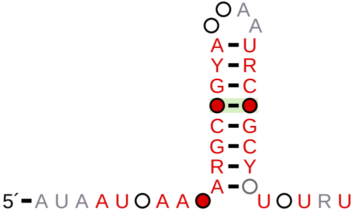

HMP2_8956_1

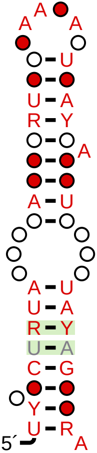

HMP2_8960_1

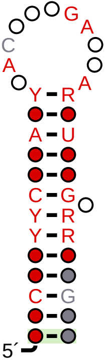

HMP2_8968_1

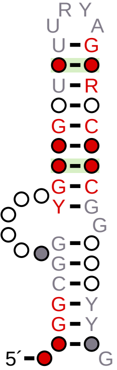

HMP2_8973_1

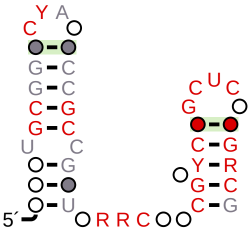

HMP2_8976_1

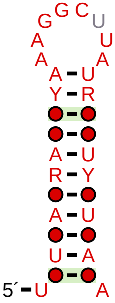

HMP2_8978_1

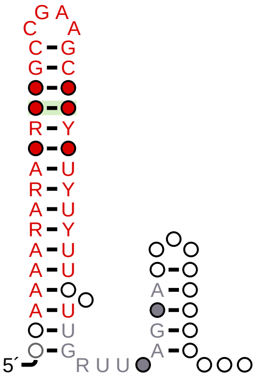

HMP2_8980_1

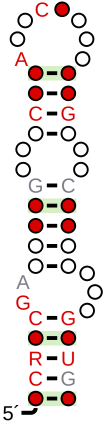

HMP2_8982_1

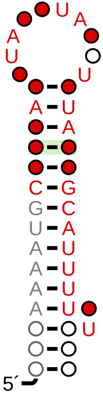

HMP2_8983_1

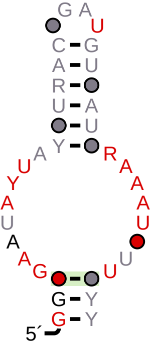

HMP2_8990_1

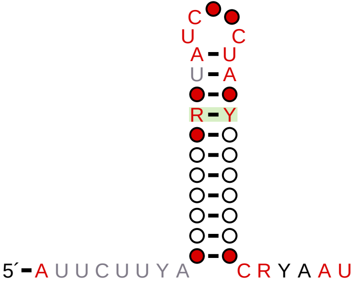

HMP2_8994_1

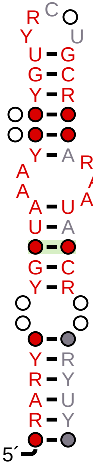

HMP2_9000_1

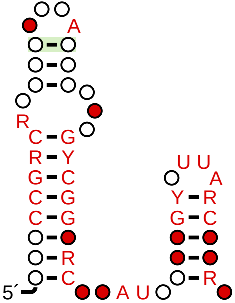

HMP2_9005_1

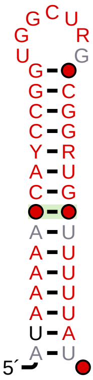

HMP2_9006_1

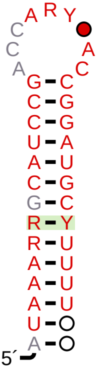

HMP2_9008_1

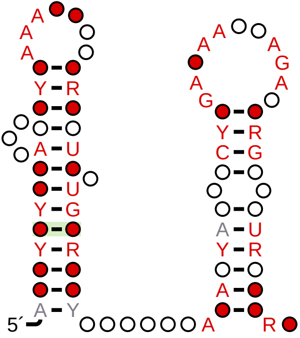

HMP2_9015_1

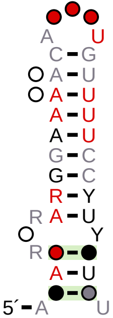

HMP2_9022_1

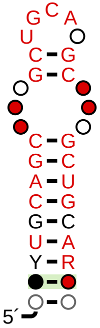

HMP2_9036_1

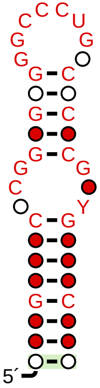

HMP2_9037_1

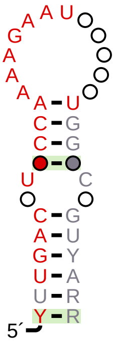

HMP2_9038_1

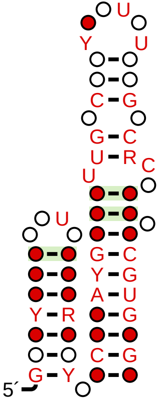

HMP2_9039_1

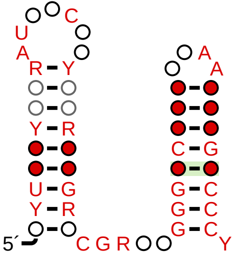

HMP2_9043_1

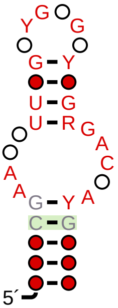

HMP2_9045_1

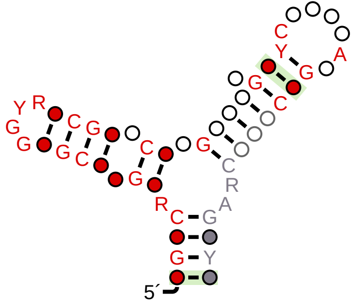

HMP2_9052_1

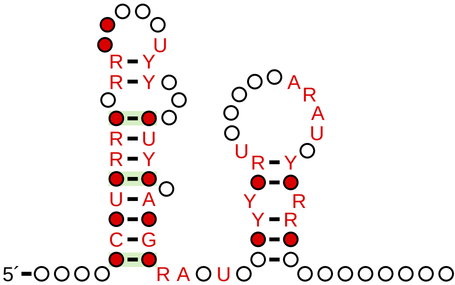

HMP2_9071_1

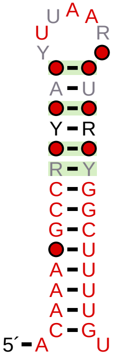

HMP2_9073_1

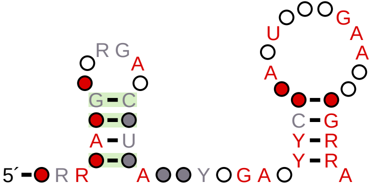

HMP2_9083_1

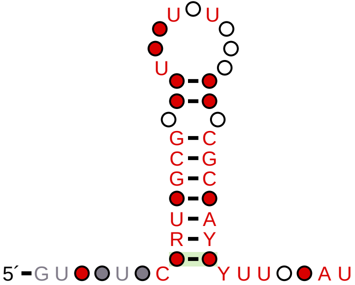

HMP2_9097_1

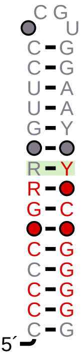

HMP2_9103_1

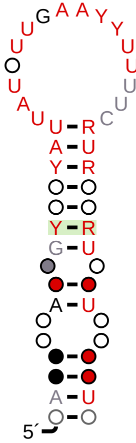

HMP2_9105_1

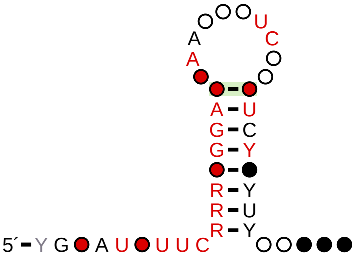

HMP2_911_1

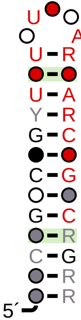

HMP2_9110_1

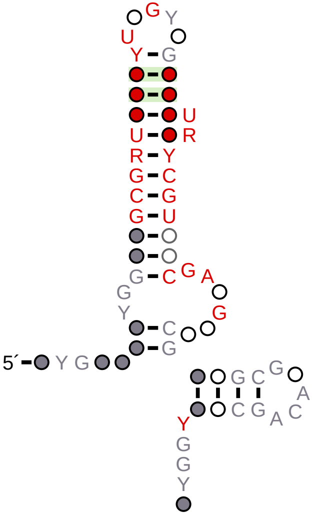

HMP2_9111_1

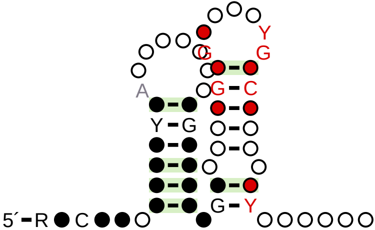

HMP2_9117_1

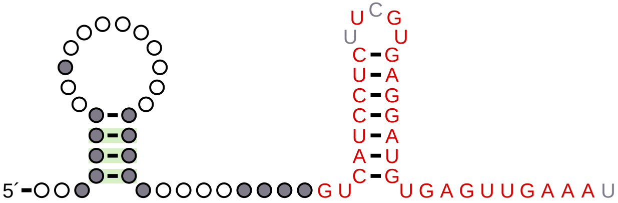

HMP2_9146_1

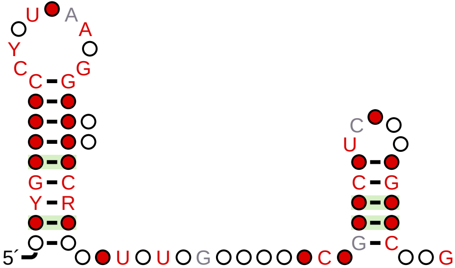

HMP2_9175_1

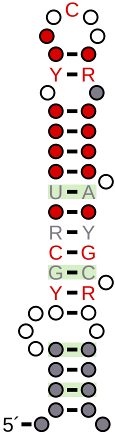

HMP2_9179_1

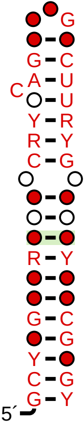

HMP2_918_1

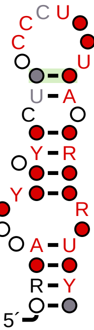

HMP2_9195_1

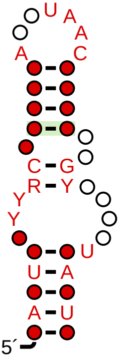

HMP2_9218_1

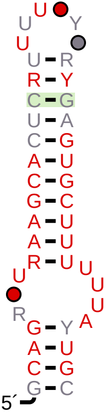

HMP2_9219_1

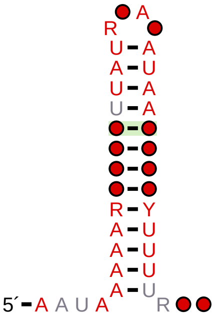

HMP2_9233_1

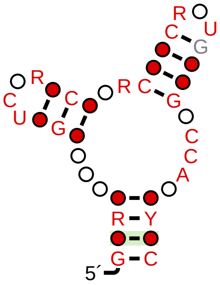

HMP2_9237_1

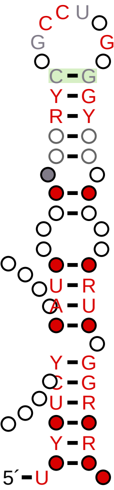

HMP2_926_1

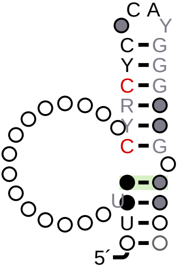

HMP2_9270_1

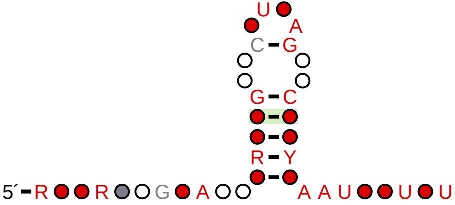

HMP2_9277_1

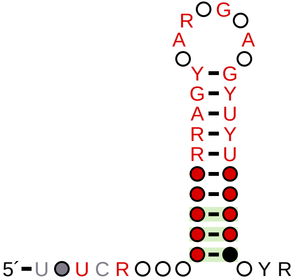

HMP2_9291_1

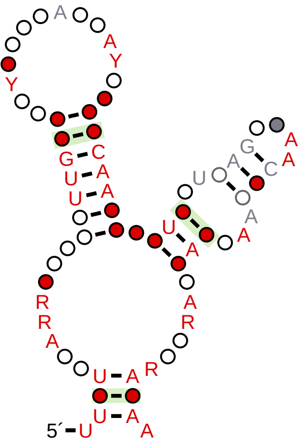

HMP2_9300_1

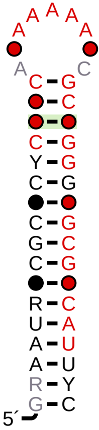

HMP2_9302_1

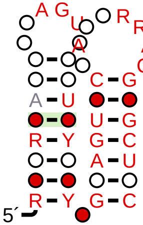

HMP2_9317_1

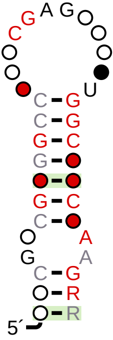

HMP2_9325_1

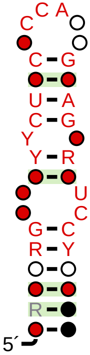

HMP2_9375_1

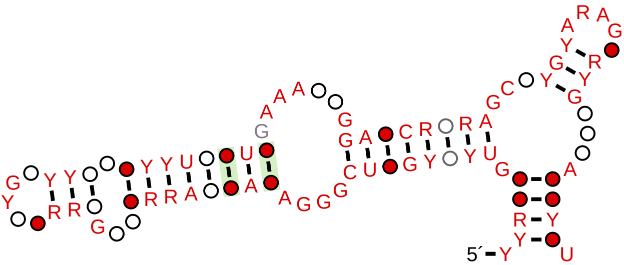

HMP2_9381_1

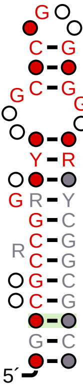

HMP2_9406_1

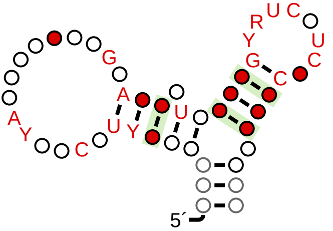

HMP2_9423_1

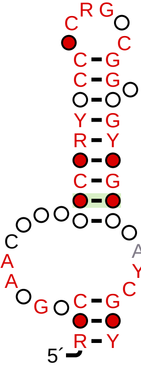

HMP2_9427_1

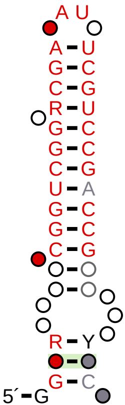

HMP2_9430_1

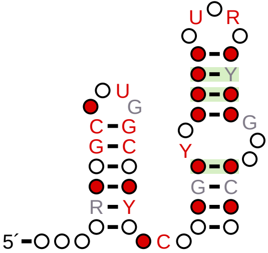

HMP2_9435_1

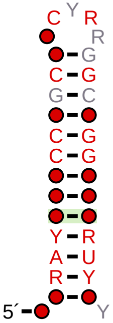

HMP2_9437_1

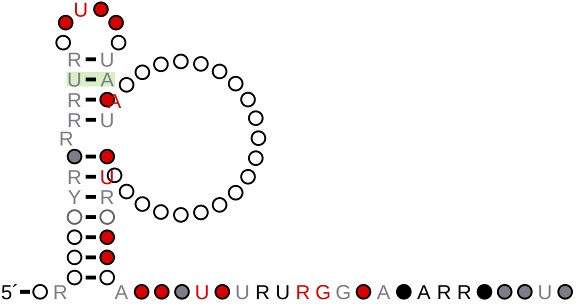

HMP2_944_1

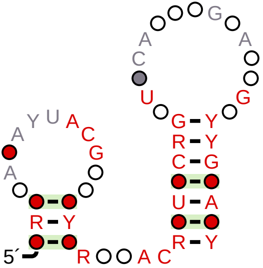

HMP2_9445_1

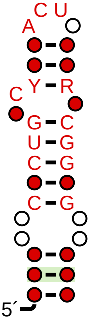

HMP2_945_1

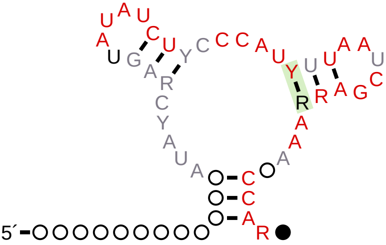

HMP2_9460_1

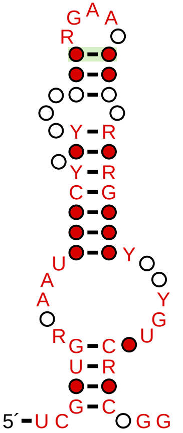

HMP2_9470_1

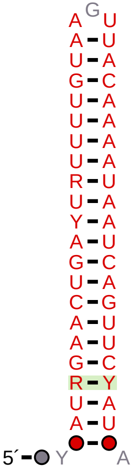

HMP2_9471_1

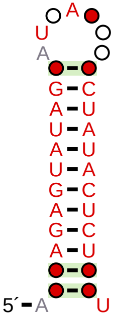

HMP2_948_1

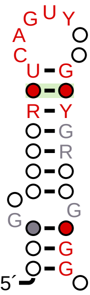

HMP2_9482_1

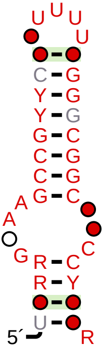

HMP2_9497_1

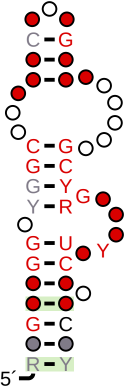

HMP2_950_1

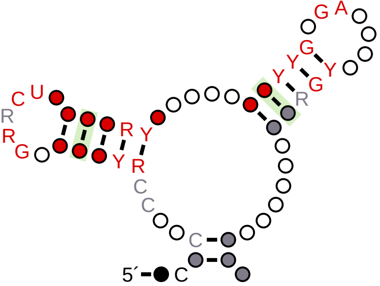

HMP2_9504_1

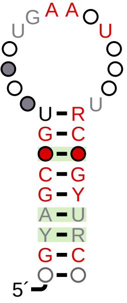

HMP2_9513_1

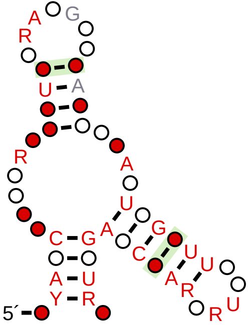

HMP2_9527_1

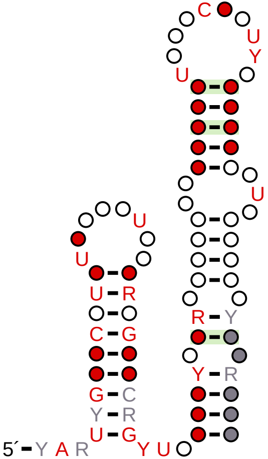

HMP2_9542_1

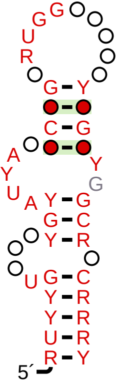

HMP2_9545_1

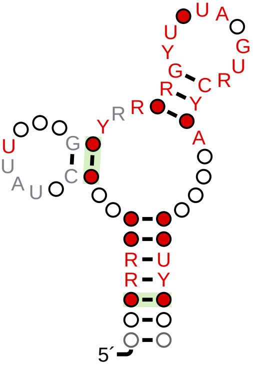

HMP2_9557_1

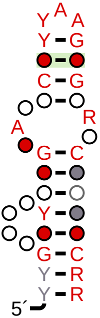

HMP2_9564_1

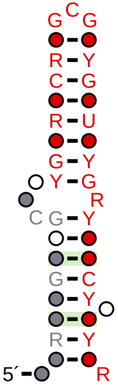

HMP2_9569_1

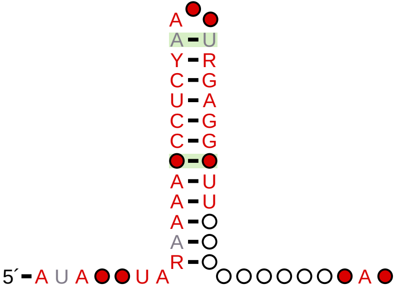

HMP2_9577_1

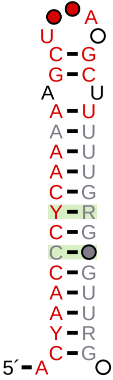

HMP2_9580_1

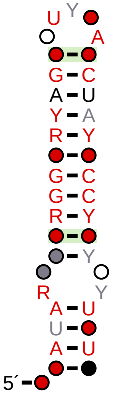

HMP2_9581_1

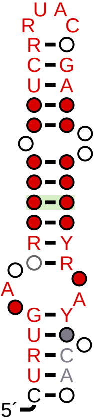

HMP2_9584_1

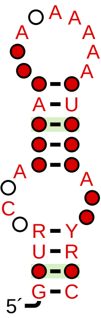

HMP2_9586_1

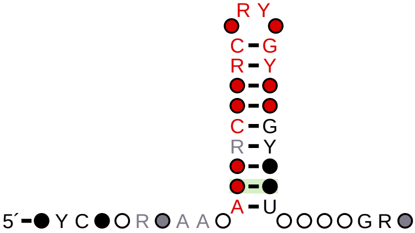

HMP2_9595_1

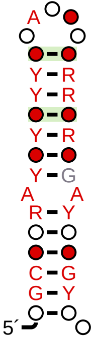

HMP2_9601_1

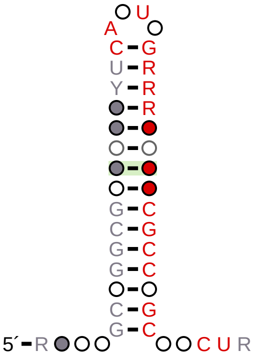

HMP2_9611_1

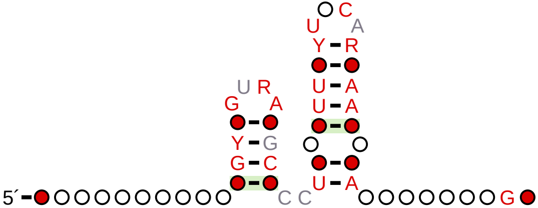

HMP2_9623_1

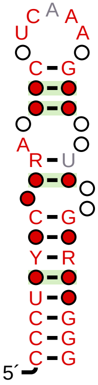

HMP2_9628_1

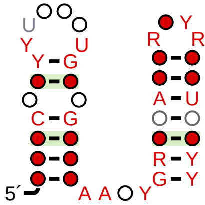

HMP2_9631_1

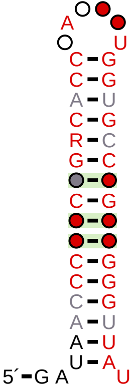

HMP2_9649_1

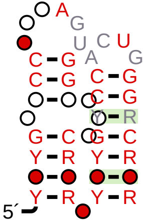

HMP2_9663_1

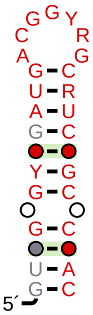

HMP2_9667_1

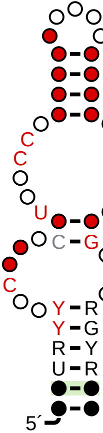

HMP2_9676_1

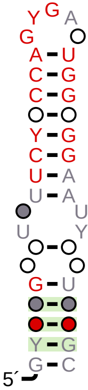

HMP2_9678_1

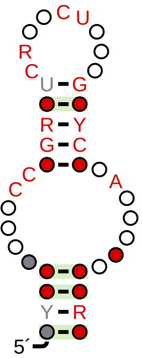

HMP2_9684_1

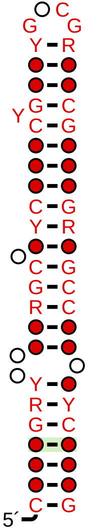

HMP2_9714_1

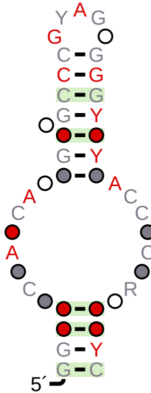

HMP2_9724_1

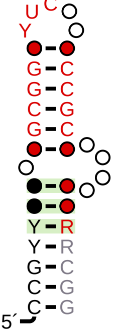

HMP2_9730_1

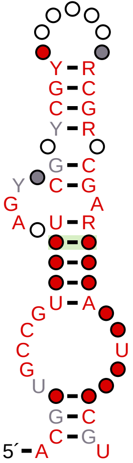

HMP2_9732_1

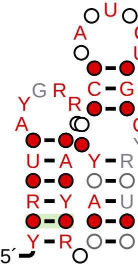

HMP2_974_1

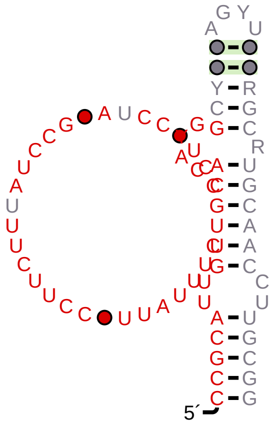

HMP2_9743_1

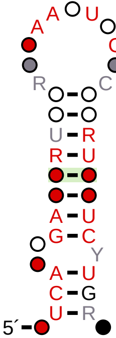

HMP2_9745_1

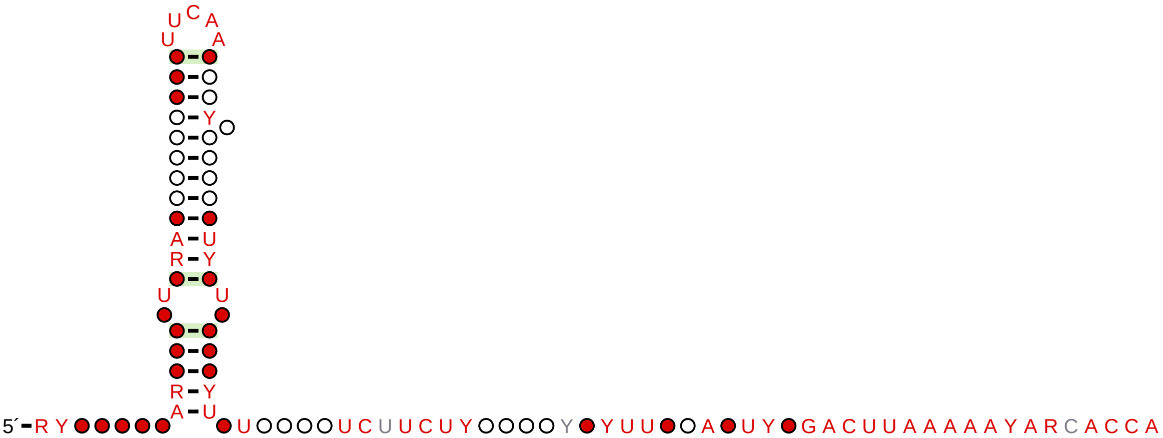

HMP2_9752_1

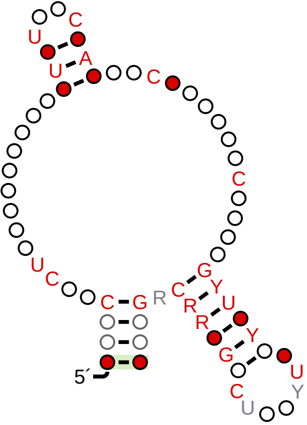

HMP2_9756_1

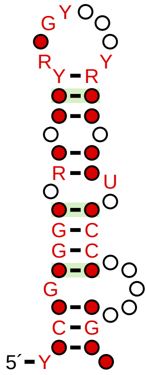

HMP2_9764_1

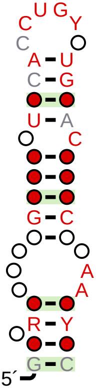

HMP2_9775_1

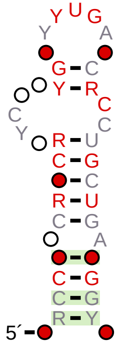

HMP2_9780_1

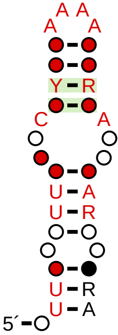

HMP2_9795_1

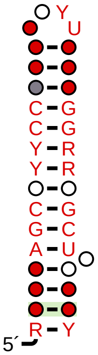

HMP2_9796_1

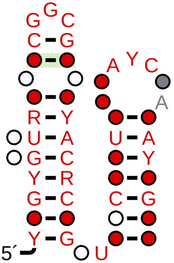

HMP2_98_1

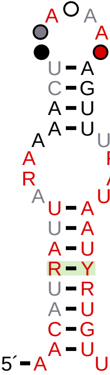

HMP2_9800_1

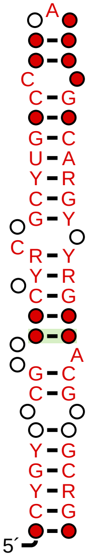

HMP2_9801_1

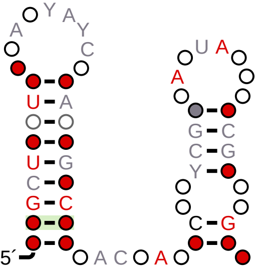

HMP2_9807_1

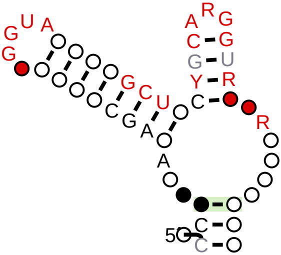

HMP2_9810_1

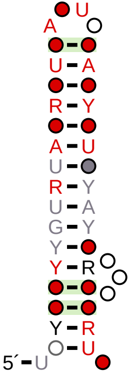

HMP2_9811_1

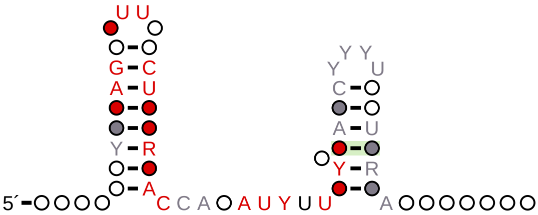

HMP2_9821_1

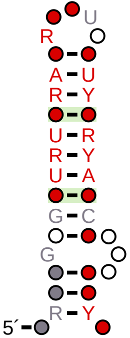

HMP2_984_1

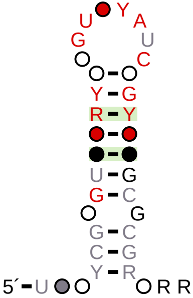

HMP2_9847_1

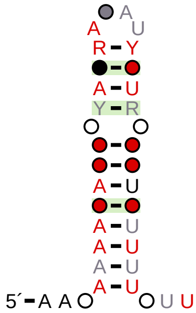

HMP2_9851_1

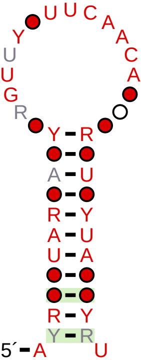

HMP2_9852_1

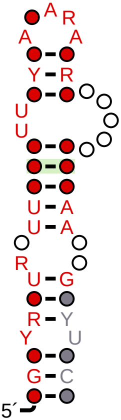

HMP2_9853_1

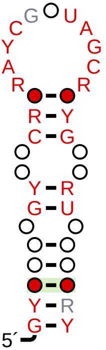

HMP2_9872_1

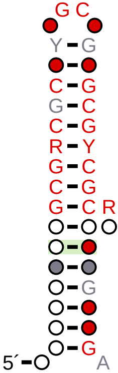

HMP2_9875_1

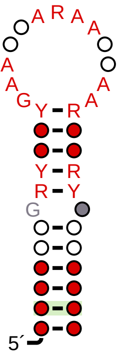

HMP2_9877_1

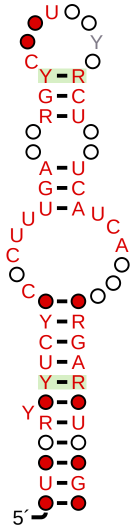

HMP2_9878_1

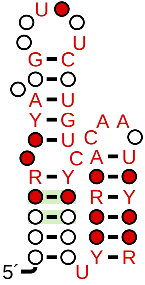

HMP2_9897_1

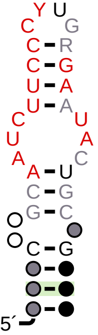

HMP2_9902_1

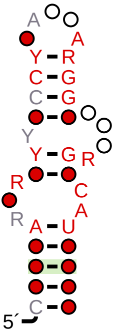

HMP2_9912_1

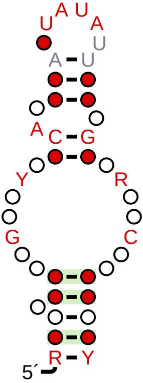

HMP2_9917_1

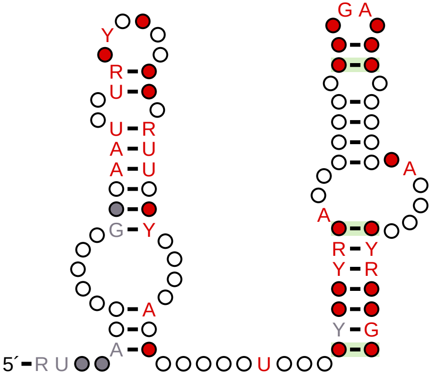

HMP2_9933_1

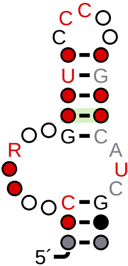

HMP2_9940_1

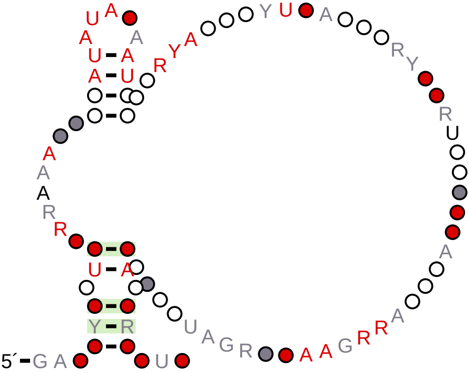

HMP2_9952_1

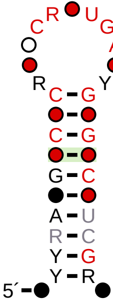

HMP2_9972_1

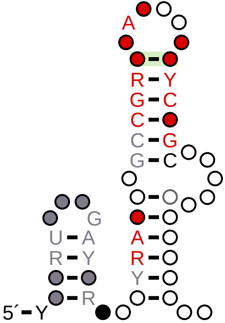

HMP2_9976_1

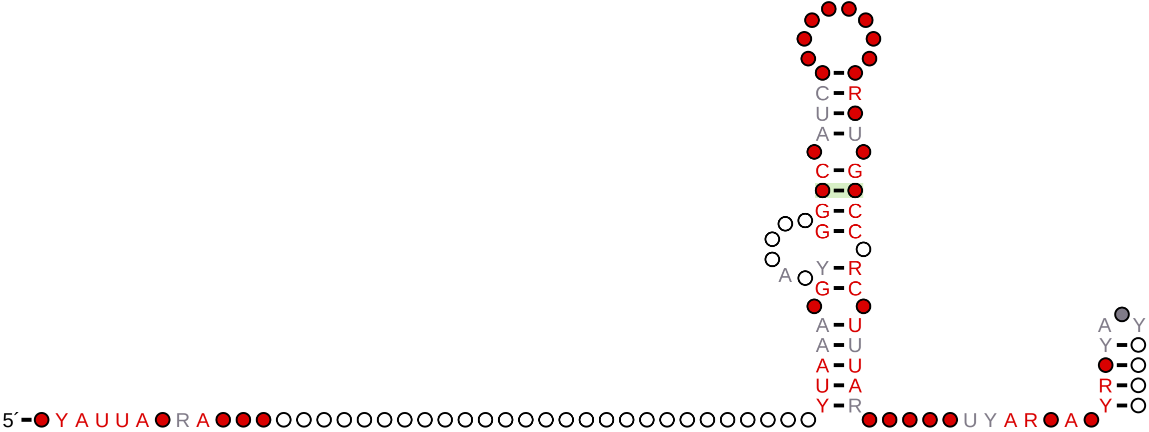

Supplement: Supplementary file 7 — Additional file 7. Visualization of the set of 2022 candidate structured RNAs that overlap the nr database. We show R2R [36] visualizations of the 2022 candidate structured RNAs with significant hits to the nr database as a pdf. [file 13059_2021_2319_MOESM7_ESM.pdf]
